# Adjacency and Area Explain Species Bioregional Shifts in Neotropical Palms

**DOI:** 10.3389/fpls.2019.00055

**Published:** 2019-02-05

**Authors:** Cintia G. Freitas, Christine D. Bacon, Advaldo C. Souza-Neto, Rosane G. Collevatti

**Affiliations:** ^1^Laboratório de Genética & Biodiversidade, Instituto de Ciências Biológicas, Universidade Federal de Goiás, Goiânia, Brazil; ^2^Department of Biological and Environmental Sciences, University of Gothenburg, Gothenburg, Sweden; ^3^Gothenburg Global Biodiversity Centre, Gothenburg, Sweden

**Keywords:** arecaceae, community phylogenetics, geographical variables, phylogenetic nestedness, phylogenetic turnover

## Abstract

Environmental and geographical variables are known drivers of community assembly, however their influence on phylogenetic structure and phylogenetic beta diversity of lineages within different bioregions is not well-understood. Using Neotropical palms as a model, we investigate how environmental and geographical variables affect the assembly of lineages into bioregions across an evolutionary time scale. We also determine lineage shifts between tropical (TRF) and non-tropical (non-TRF) forests. Our results identify that distance and area explain phylogenetic dissimilarity among bioregions. Lineages in smaller bioregions are a subset of larger bioregions and contribute significantly to the nestedness component of phylogenetic dissimilarity, here interpreted as evidence for a bioregional shift. We found a significant tendency of habitat shifts occurring preferentially between TRF and non-TRF bioregions (31 shifts) than from non-TRF to TRF (24) or from TRF to TRF (11) and non-TRF to non-TRF (9). Our results also present cases where low dissimilarity is found between TRF and non-TRF bioregions. Most bioregions showed phylogenetic clustering and larger bioregions tended to be more clustered than smaller ones, with a higher species turnover component of phylogenetic dissimilarity. However, phylogenetic structure did not differ between TRF and non-TRF bioregions and diversification rates were higher in only two lineages, Attaleinae and Bactridinae, which are widespread and overabundant in both TRF and non-TRF bioregions. Area and distance significantly affected Neotropical palm community assembly and contributed more than environmental variables. Despite palms being emblematic humid forest elements, we found multiple shifts from humid to dry bioregions, showing that palms are also important components of these environments.

## Introduction

Short- and long-term ecological and evolutionary processes affect patterns of species composition and co-occurrence (Ricklefs, 2004). Diversification may explain species distribution and richness, species-area relationships (the increase in species richness with geographical area), and the distance-decay relationship in community similarity (see Morlon, 2014 for a review). However, the assembly of species into communities also depends on environmental conditions and geographical variables (e.g., Araújo et al., 2013). Testing for the role of these different sets of variables can help explain the process of community assembly, especially for plants as they are the base of the food web in terrestrial environments and have an important role in shaping community structure and species diversity.

In community assembly, the restriction to particular ecological conditions may prevent lineages from habitat shifts and successful colonization of new areas (e.g., Wiens and Graham, 2005). Thus, habitat shifts are not common because species tend to retain their ancestral ecological niche (phylogenetic niche conservatism, PNC hereafter; Losos et al., 2003; Ackerley et al., 2006; Silvertown et al., 2006; Crisp et al., 2009). Further, shifts are thought to be rare due to the amount of morphological or physiological changes required for colonization and survival in a distinct environment (Donoghue, 2008). Despite its rarity in plants, there is accumulating evidence that habitat shifts contribute to community assembly and thus species richness and diversity within and between biomes and ecoregions (Pennington et al., 2004; Alcantara et al., 2014; Donoghue and Edwards, 2014; Souza-Neto et al., 2016; Bacon et al., 2017, 2018; Antonelli et al., 2018), with some transitions easier than others (e.g., Wiens and Donoghue, 2004; Donoghue and Edwards, 2014). For example, several plant lineages have shifted between the Amazonia tropical rain forest and savanna biomes of South America (e.g., Simon et al., 2009; Simon and Pennington, 2012; Terra-Araujo et al., 2015; Souza-Neto et al., 2016; Melo et al., 2018). Shifts indicate that lineages were able to colonize and persist in certain environments over time, leading to a nestedness pattern in phylogenetic beta diversity, i.e., the “sink” area is a subset of lineages from the “source” (Baselga, 2010; Leprieur et al., 2012). On the other hand, *in situ* diversification points to lineage diversification due to ecological opportunity to diversify and adapt, leading to a turnover pattern in phylogenetic beta diversity, i.e., phylogenetic dissimilarity will be due to exclusive lineages in each area (Baselga, 2010; Leprieur et al., 2012).

Geographical variables are also likely contributors to plant species assembly. A bioregion, defined by evolutionary history and taxonomic composition (e.g., Vilhena and Antonelli, 2015), may be colonized by lineages from adjacent ones, particularly in the case where a larger bioregion would serve as “source” of lineages. Thus, area and adjacency may increase the probability of species shifting bioregions (Donoghue and Edwards, 2014), and in doing so, bioregions may function according to source and sink dynamics (e.g., Pulliam, 1988). In source and sink dynamics, the movement of individuals is primarily from highly suitable “core” areas to lesser ones. Moreover, over deep geological time, regions with large areas of suitable habitat have higher speciation rates and low extinction rates and therefore show higher phylogenetic clustering than when the suitable area is limited (Kissling et al., 2012).

The Neotropics harbor the most diverse ecosystems on earth and is a mosaic of environments, including rainforests, wetlands, alpine areas, and dry forests (e.g., Antonelli and Sanmartín, 2011), and palms are a conspicous element of many bioregions (Gentry, 1988). Palms have been recognized as a model group for understanding the evolution of biomes (Bacon, 2013) and tropical forests (Couvreur and Baker, 2013) because they are ecologically representative, have a long and rich fossil history, as well as a robust geographical, phylogenetic, and taxonomic established framework (Baker and Dransfield, 2016). Both environmental (Bjorholm et al., 2006; Kristiansen et al., 2011) and geographical variables (Bjorholm et al., 2005) affect palm species richness, diversity, and composition. Climatic variables may cause palms to be spatially restricted along temperature gradients (Eiserhardt et al., 2013). A higher turnover component in phylogenetic beta diversity is expected under limited dispersal, limited niche evolution, preventing lineage shifts (i.e., strong PNC, see Eiserhardt et al., 2013), and higher rates of *in situ* diversification. If the environment is a major force controlling the assembly of palm lineages in the Neotropics then *in situ* diversification and turnover will be more important in explaining the assembly of palms in the Neotropics. However, if geographical variables are more important than the environment, then lineages would shift among bioregions despite differences in the environment, leading to a nestedness pattern in phylogenetic beta diversity (Leprieur et al., 2012).

Here, we use palms to address the roles of environmental and geographical variables in bioregions assembly in the Neotropics (see Table 1 for hypotheses and predictions). Specifically, we test the hypothesis that lineage shifts to new bioregions are independent of environmental differences between source and sink bioregions (H1). From H1 we predict that phylogenetic turnover will not correlate with environmental dissimilarity (accounting for geographical distance) and bioregions will present no phylogenetic clustering. Further, we predict that ancestral reconstruction will show lineage shifts between bioregions regardless of the differences in environmental variables and no difference in diversification rates among clades from different bioregions. We also test the hypothesis that lineage shift into different bioregions is a function of the area and adjacency to the source bioregion (H2). From H2 we predict that phylogenetic turnover and phylogenetic clustering positively correlate with the area of the bioregion and the sink bioregion will be a subset of the source one (Baselga, 2010; Leprieur et al., 2012). Finally, we hypothesize that TRF bioregions are the source of lineages to the non-TRF bioregions as palms are suggested to have originated in TRFs (H3, Couvreur et al., 2011). For this, we predict that phylogenetic turnover is lower among TRF/non-TRF bioregions due to shifts between TRF (source) and non-TRF (sink) bioregions, than among all other category pairs, and TRF is phylogenetic clustered while non-TRF is over dispersed due to these shifts. Through hypothesis testing, we examine the role of the environment vs. geographical variables in the assembly of palms in the Neotropics.

**Table 1:** Hypotheses and predictions for testing the roles of environmental and geographical variables in bioregions assembly in Neotropical palms.

**Hypotheses**	**Predictions**	**Support**
H_1_: Lineage shifts to new bioregions are independent of environmental differences between source and sink bioregions.	1.1. Phylogenetic dissimilarity (Phylosor) and turnover, and phylogenetic clustering (NRI) will not correlate with environmental dissimilarity (accounting for geographical distance).	Supported (Figures 3, 5)
	1.2. Bioregions will present no phylogenetic clustering (NRI and NTI) and will show overabundance of the same clades. 1.3. Ancestral reconstruction will show lineage shifts between bioregions regardless of the differences in environmental variables. 1.4. No differences in diversification rates among lineages or lineages with higher diversification rates are not restricted to one or few bioregions.	Partially supported (Table 2, Figure 6) Supported (Figure 9) Supported (Figure 10)
H_2_: Lineage shifting into different bioregions is a function of the area and adjacency to the source bioregion.	2.1. Controlled for species richness, larger biomes (sources) will be phylogenetically clustered (NRI) compared to smaller ones (sinks) that will be over dispersed due to lineage shifts. 2.2. Phylogenetic dissimilarity (Phylosor) among adjacent bioregions will be due to nestedness component evincing shifts between bioregions. 2.3. Positive correlation between the turnover component of phylogenetic dissimilarity (Phylosor) and the bioregion area and distance. 2.4. Smaller bioregions (sinks) will be a subsample (nested) of larger ones (sources) and phylogenetic dissimilarity (Phylosor) in smaller bioregions will be due to nestedness evincing bioregion shifts. 2.5. Positive correlation between number of shared borders and number of shifts.	Partially supported (Figure 5, Table 2) Partially supported (Figure 3) Supported (Figures 3, 4) Supported (Figures 3, 4) Supported (Figures 2, 9)
H_3_: Lineages shifts occurred more from TRF bioregions to non-TRF bioregions than from non-TRF to TRF or all possible category pairs.	3.1. TRF are phylogenetic clustered (NRI) while non-TRF bioregions are over dispersed due to lineages shifts.	Not supported (Figure 8)
	3.2. The ancestral reconstruction will show higher number of shifts from TRF to non-TRF bioregions than the opposite.	Supported (Figure 9)
	3.3. Non-TRF will be a sub-sample of TRF biomes, thus the nestedness component of phylogenetic dissimilarity (Phylosor) between those groups is higher than turnover, evincing bioregion shifts. 3.4. Diversification will be higher in TRF bioregions than in non-TRF.	Not supported (Figure 7) Not supported (Figure 10)

**Table 2:** Phylogenetic structure of Neotropical bioregions based on the net relatedness (NRI) and nearest taxon (NTI) indices across phylogenetic communities of Neotropical palms.

		**NRI**				**NTI**			
**Bioregions**	***N***	**MPD**	**MPDnull**	**NRI**	***p***	**MNTD**	**MNTDnull**	**NTI**	***p***
Atlantic Coastal forest (ACF)	82	120.958	152.966	**8.874**	**0.001**	32.029	35.414	1.272	0.103
Amazonia (AMA)	115	134.184	153.331	**6.771**	**0.001**	29.863	31.858	1.040	0.147
Central American moist forest (CMF)	172	152.956	156.623	**1.996**	**0.027**	31.044	28.945	−1.569	0.941
Caatinga (CAA)	44	116.618	−151.478	**6.563**	**0.001**	28.276	42.534	**3.087**	**0.001**
Caribbean (CAR)	59	155.609	157.248	0.399	0.326	31.801	39.272	**2.217**	**0.010**
Caribbean dry forest (CDF)	22	152.790	151.889	−0.124	0.534	57.713	56.573	−0.135	0.559
Central Andes (CAN)	85	152.195	154.847	0.862	0.188	29.236	34.528	**2.214**	**0.020**
Cerrado (CER)	111	130.380	153.118	**7.448**	**0.001**	27.901	32.138	**2.124**	**0.013**
Chaco and Espinal (CHE)	22	135.953	152.136	**2.105**	**0.031**	50.163	56.634	0.744	0.232
Choco (CHO)	126	152.951	155.310	0.916	0.181	30.983	30.944	−0.022	0.503
Grassland and Pampa (GRP)	12	145.400	152.594	0.655	0.253	55.620	74.297	1.253	0.093
Guiana shield (GSH)	79	134.562	152.783	**4.969**	**0.001**	34.745	36.093	0.514	0.303
Inter-Andean forest (IAF)	22	150.645	151.757	0.159	0.420	56.568	56.413	−0.018	0.514
Llanos (LLA)	56	147.252	152.907	1.325	0.092	36.462	39.598	0.860	0.204
Northern Andes (NAN)	169	152.062	156.190	**2.049**	**0.032**	26.685	28.233	1.162	0.134
Pantanal (PAN)	12	113.440	146.696	**2.723**	**0.008**	46.426	72.308	**1.715**	**0.036**
Southeastern United States (SEU)	25	152.810	156.978	0.609	0.241	49.956	55.954	0.744	0.232
Dry Tropical America (TAM)	86	155.696	156127	0.147	0.431	34.385	35.275	0.357	0.367
Western Amazonia (WAM)	143	149.638	155245	**2.279**	**0.015**	26.709	29.909	**1.926**	**0.033**
Xeric Mesoamerica (XMA)	19	140.258	158.043	**2.393**	**0.02**	31.215	61.015	**2.946**	**0.001**

*N, number of species per bioregion; MPD, mean observed phylogenetic distance; MPD_null_, mean phylogenetic distance for the null model; MNTD, mean observed nearest neighbor distance; MNTD_null_, mean nearest neighbor distance for the null model. Both MPD and MPTD are reported in millions of years before present (Ma). In bold, NRI and NTI significant values (p < 0.05)*.

## Methods

### Dated Phylogeny of the Neotropical Palms

We built a phylogenetic tree for Neotropical palms following the taxonomy of Henderson et al. (1995) and the 541 species included in Göldel et al. (2015). Following this taxonomy, a broader species concept is used and results take on a more conservative assessment of palm diversity. We obtained and sequences for 337 species from the GenBank for the chloroplast *matK* and *RPB2* nuclear gene (GenBank numbers and information in Appendices S1, S2). Where possible, the same voucher specimen was used for both genes. Alignments for each gene were built using the MUSCLE (Edgar, 2004) tool in Geneious v7.0.5 (Biomatters Ltd.) and manual adjustments were performed following Simmons (2004). A dated molecular phylogeny was inferred in BEAST v. 1.8.3 (Drummond et al., 2006) using the sequence data partitioned by locus. The analysis was run using an uncorrelated lognormal molecular clock, a Yule pure birth speciation model with random starting tree, the GTR + Γ model of nucleotide substitution with four rate categories for both partition, and the default operators. The Markov chains were run for 500 million generations and repeated three times to verify convergence and to ensure effective sample sizes exceeded 200. The divergence time analysis was constrained to incorporate robust secondary calibrations points on the root of the Arecaceae tree, as well as on three major clades of Neotropical palms: Ceroxyloideae (mean age of 52 Ma with a standard deviation of 11 Ma; Sanín et al., 2016), Geonomateae (40 ± 6 Ma; Roncal et al., 2012), and the New World Thatch Palms (41 ± 9 Ma; Cano et al., 2018). Age constraint on the crown node of Arecaceae was derived from Couvreur et al. (2011) at 100 ± 4 Ma.

After obtaining the dated phylogenetic tree (Figure S1 in Appendix S3), we added the remaining species with no sequence data available (Table S1 in Appendix S4), and excluded 37 species that did not occur in the study area to perform further analysis, which resulted in a phylogenetic tree with 504 species. Species were grafted to their respective genus following Swenson et al. (2006) using Mesquite (Maddison and Maddison, 2018) as unresolved. Further, the *multi2di* function from the *Ape* package (Paradis et al., 2004) of the R 2.15 software (R Core Team, 2014) was used to collapse and resolve polytomies by assigning branch lengths equal to 1.0. Allocating unsampled species to its respective genus unlikely significantly impact our results, since analytical sensitivity is greater at the genus and deeper branches, rather than at the species level (Swenson et al., 2006).

### Occurrence and Assembly

**Figure 1:**
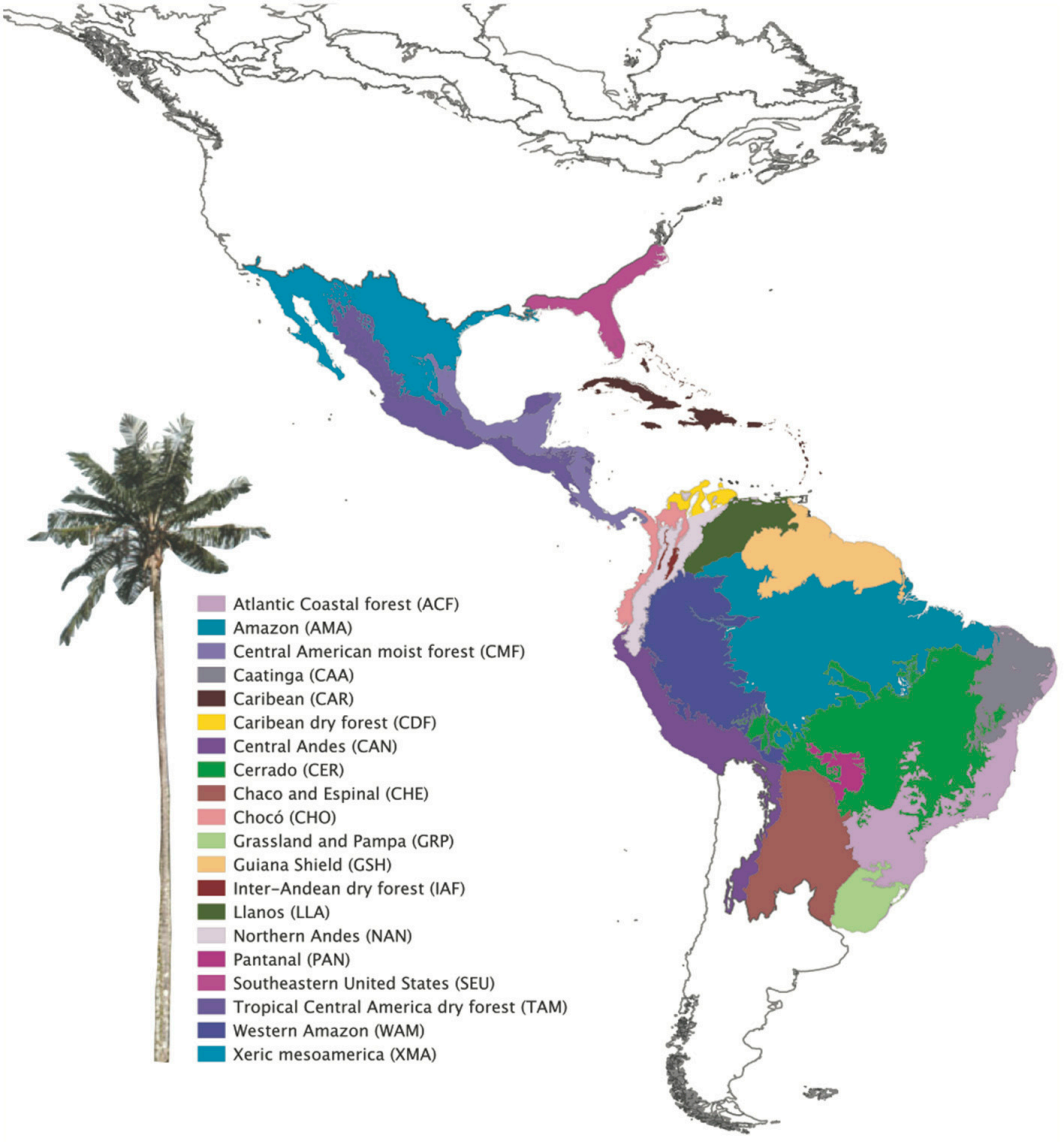
Neotropical bioregions used to study Neotropical palms evolution based on WWF classification. Modifications in bioregion classification were performed based on their biogeographical and environmental similarity (see Table S1 in Appendix S2).

A shape file of the Terrestrial Ecoregions of the World was downloaded from the World Wildlife Foundation (WWF; Olson et al., 2001) and trimmed to the Neotropics in QGIS (QGIS Team, 2012). Changes to the classified ecoregions were made by merging certain areas (Table S2 in Appendix S4) to more accurately reflect the shared geological history of our study area (*sensu* Holt et al., 2013), resulting in 20 bioregions (Figure 1). The reclassification increased the number of species per region, improving the statistical power of the analyses. For example, the Cerrado also comprises enclaves of seasonally dry forests (SDTFs). The Atlantic Coastal forest comprises both the tropical rain forest on the coast (Serra do Mar Coastal forest) and the SDTFs on the continental side, and the Araucaria forest (subtropical seasonally dry forest). We split the Amazonia biome into bioregions based on geography and soil differences (*sensu* Quesada et al., 2011), considering western, central, eastern Amazonia and Guiana shield, different from Amazonia WWF ecoregions, which were defined by interfluves. The Llanos comprises La Costa xeric shrub lands and the Apure-Villavicencio dry forest (Table S2 in Appendix S4).

We obtained geographical information for the 504 Neotropical palm species used in this study and generated an occurrence-per-bioregion matrix (hereafter occurrence matrix; Table S3 in Appendix S4). We used the *maptools* R package (Bivand, 2006) to extract georeferenced species occurrences from the Global Biodiversity Information Facility (GBIF, 2018). Ambiguous bioregion classification was checked in the newly updated List of Brazilian Flora 2020 (Flora do Brasil, 2020). Georeferenced points that were out of the study area were removed from the data and species occurrences were checked in the resulting table for misclassification. The species list and trimmed bioregions shape file were combined using the over function from the *sp* R package (Pebesma and Bivand, 2005).

### The Environment vs. Geographical Variables

To test whether the colonization of new bioregions is independent of environmental differences between source and sink bioregions (Table 1, H1) we analyzed the relationship of phylogenetic turnover and phylogenetic clustering with environmental variables. For this, we obtained the phylogenetic dissimilarity between pairs of bioregions using the phylogenetic index of beta diversity (PhyloSor index; Bryant et al., 2008) implemented in the *picante* R package (Kembel et al., 2010). Significance of PhyloSor indices were tested by maintaining species richness in each bioregion and randomizing species occurrences (presence/absences) shared between different bioregions. Each species was sampled from the species pool (all 504 species) with equal probability. Phylosor ranges from 0 (two communities share few to no lineages) to 1 (both communities are composed of the same lineages). We then calculated the PhyloSor dissimilarity index (1–PhyloSor similarity index) and partitioned it into turnover and nestedness components using the phylo.beta.pair function in *betapart* R package (Baselga and Orme, 2012) following Leprieur et al. (2012). This method uses an additive partitioning of the phylogenetic beta diversity providing two separate components without the influence of species richness gradients (Baselga, 2010). Phylogenetic turnover accounts for pure lineage replacement (turnover) and nestedness is the difference between phylogenetic beta diversity and phylogenetic turnover and reflects the increasing dissimilarity between nested assemblages due to the increasing differences in lineages.

For environmental variables, we obtained annual mean temperature and mean annual precipitation for each bioregion. Data was downloaded from WorldClim (http://worldclim.org/version2) at 5 km resolution and processed in ArcGIS 10.3.1. The environmental distance matrix between pairs of bioregions was calculated using the average distance per bioregion.

The phylogenetic structure of bioregions was assessed using the Net Relatedness Index (NRI) and the Nearest Taxon Index (NTI; Webb et al., 2002) using *picante* R package (Kembel et al., 2010). Positive NRI and NTI values indicate phylogenetic clustering, i.e., close relatives co-occur more than expected by chance, and negative values indicate phylogenetic overdispersion, i.e., close relatives co-occur less than expected by chance (Webb et al., 2002). To test for significance, we used a null model based on the independent swap algorithm (Gotelli and Entsminger, 2003), which randomizes the data matrix maintaining the sample species occurrence frequency and richness and considered all 504 species as our species pool. We then used a linear regression model to fit the relationship between the log-transformed area of each bioregion and NRI and NTI. The area of each bioregion was calculated in km^2^ using QGIS (see Table S5 in Appendix S4).

We analyzed whether turnover is more prevalent than the nestedness component of phylogenetic dissimilarity among bioregions, which would indicate that *in situ* lineage diversification is more frequent than shifts. We then tested whether the turnover and nestedness patterns and phylogenetic clustering (e.g., species are more closely related than expected by chance) were correlated to environmental dissimilarity (e.g., precipitation and temperature, Table 1, H1). Also, we tested whether species shifting into different bioregions is correlated to geographical variables, such as area and adjacency to the source bioregion (Table 1, H2). For this we performed a Multiple Matrix Regression with Randomization approach (MMRR, Wang, 2013) between the phylogenetic beta diversity (Phylosor), turnover and nestedness components of the Phylosor with the environmental and geodesic distances (logarithm) between pairs of bioregions matrices. To account for spatial autocorrelation in environmental variables we performed a Moran's I test. The analyses were performed using the *vegan* R package. The geodesic distance was calculated between the nearest-most edges between bioregions using QGIS (Table S4 in Appendix S4). We also performed a regression between the area of each bioregion and the turnover component of the Phylosor and phylogenetic clustering, also using *vegan* R package

We calculated taxonomic beta diversity (Sørensen's Index; Sørensen, 1948) to verify whether phylogenetic and taxonomic indices indicate dissimilarity among bioregions (Baselga, 2010), and a Mantel test examined the congruence between the indices. We used the unweighted pair group method (UPGMA; Sokal and Michener, 1958) to build a bioregion-based dendrogram using the PhyloSor index to test whether bioregion clustering matched the expected by their adjacency.

### Clade Overabundance in Bioregions

We analyzed if bioregions with similar environments had similar overabundant clades (Table 1, H1) using a Nodesig analysis in Phylocom 4.2 (Webb et al., 2008) to test for overabundance of terminal taxa. The observed pattern for each bioregion was compared to those for random samples using a null model comprising random draws of “x” terminal taxa from the phylogeny, where “x” is the number of taxa in each bioregion. The Nodesig analysis uses a null model similar to the swap algorithm.

### Shifts Between TRF and Non-TRF Bioregions

To test whether TRF bioregions are the source of lineages to non-TRF bioregions (Table 1, H3) we performed a Mantel test between a matrix of all possible category pairs (TRF and TRF, non-TRF and non-TRF, and TRF and non-TRF) and the phylogenetic dissimilarity, the nestedness, and the turnover components. We also tested whether phylogenetic clustering is higher in TRF compared to non-TRF bioregions using a *t*-test (Table 1, H3). We classified areas into TRF (ACF, AMA, CAR, CHO, CMF, GSH, IAF, PAN WAM,) and non-TRF (CAA, CAN, CDF, CER, CHE, GRP, LLA, NAN, SEU, TAM, XMA) following WWF (Olson et al., 2001). Non-TRF areas include tropical grasslands, savannas and shrublands, flooded grasslands and savannas, deserts and xeric shrublands, and tropical and subtropical dry broadleaf forests.

### Ancestral Area Reconstruction and Lineage Shifts

To test whether lineage shifts are independent of environmental similarities and are higher from TRF to non-TRF bioregions (Table 1, H1 and H3), we inferred the ancestral bioregion across the phylogeny topology using maximum likelihood estimates of ancestral bioregion reconstruction implemented in BioGeoBears R package (Matzke, 2013). We used the dispersal-extinction-cladogenesis model (DEC; Ree and Smith, 2008). We merged bioregions according to geographical proximity and category (TRF or non-TRF), resulting in 10 bioregions, due to the software running constrains: A, ACF; B, CAA and CER; C, CHE and GRP; D, NAN and CAN; E, WAM, GSH and AMA; F, LLH and CDF; G, IAF, CHO, CMF; H, CAR, I, PAN; J, XMA, SEU, TAM. The main limitation of ancestral area inference is the possible number of areas for analysis, where issues are due to the lack of current hardware and efficient matrix-handling algorithms (Pyron, 2014). To account for phylogenetic uncertainty, we used both the Maximum Clade Credibility tree (MCC) and a random tree drawn from the 1,000 trees.

### Shifts in Diversification Rates

To test for shifts in diversification rates (Table 1, H1 and H3) and to assess if shifts are temporally concomitant with bioregions shifts we used the software BAMM 2.0 (Rabosky, 2012). We ran BAMM for 1,500,000 generations sampling every 15 steps for a total data set of 100,000 generations sampled. The BAMM output was analyzed in R using the BAMMtools package (Rabosky, 2014). We discarded the first 25,000 steps (25%) as burn-in and verified the convergence of our data as effective sample sizes higher than 200. Convergence and effective sample sizes (ESS) were analyzed using *coda* R package (Plummer et al., 2006). To account for phylogenetic uncertainty, we used both the Maximum Clade Credibility tree (MCC) and a random tree drawn from the 1,000 trees.

## Results

### Dated Phylogeny and Species Occurrence

The results from divergence time estimation were consistent with the generic level phylogeny of Couvreur et al. (2011) with respect to both topology and ages of lineages (Figure S1 in Appendix S3). The Central American moist forest (CMF) has the highest number of palm species (*n* = 172; Table S3 in Appendix S4), followed by the Northern Andes (NAN; *n* = 169), the Western Amazonia (WAM; *n* = 143), Choco (CHO; *n* = 126), and the Amazonia (AMA; *n* = 115), all considered here as TRF. The Cerrado (CER) was the most species-rich non-TRF bioregion (*n* = 111), for example, with more species than the TRF bioregion Atlantic coastal forest (ACF; *n* = 82).

### Environmental vs. Geographical Variables

**Figure 2:**
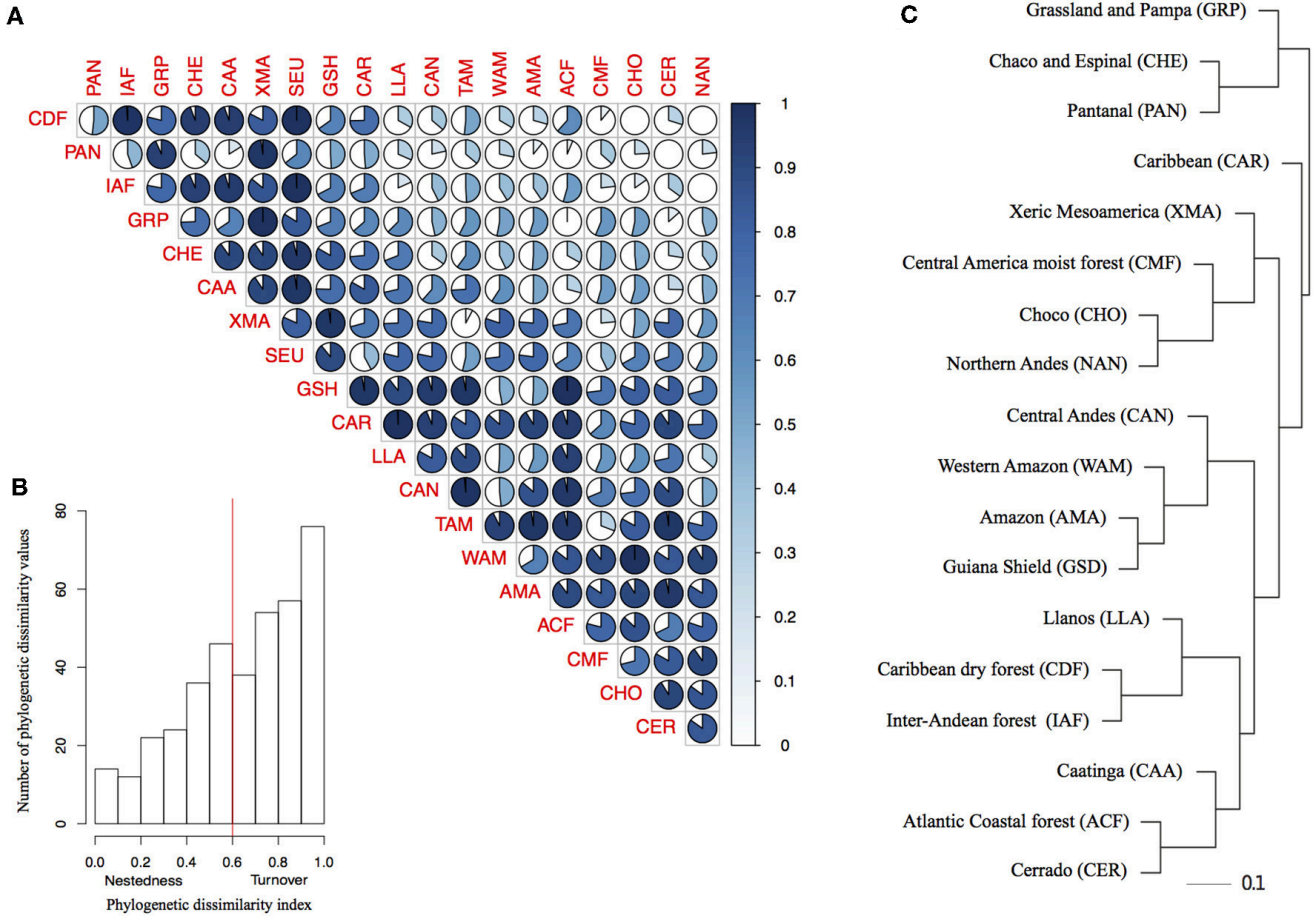
Phylogenetic dissimilarity among bioregions and the importance of nestedness and turnover components of phylogenetic dissimilarity. **(A)** Pair-wise plot of the proportion of nestedness and turnover components of phylogenetic dissimilarity for each bioregion pair. The lateral blue bar represents the increasing of turnover component represented by the gradient of blue. **(B)** The frequency distribution of the nestedness and turnover components of the phylogenetic dissimilarity index for all bioregions. The red line indicates the arbitrary threshold of 0.6 we choose to consider the turnover component as more important (>0.6) than nestedness (< 0.6), **(C)** Bioregion clusters based on the phylogenetic dissimilarity (Phylsor Index). Scale bar represents branch length (0.1 = 10% dissimilarity).

**Figure 3:**
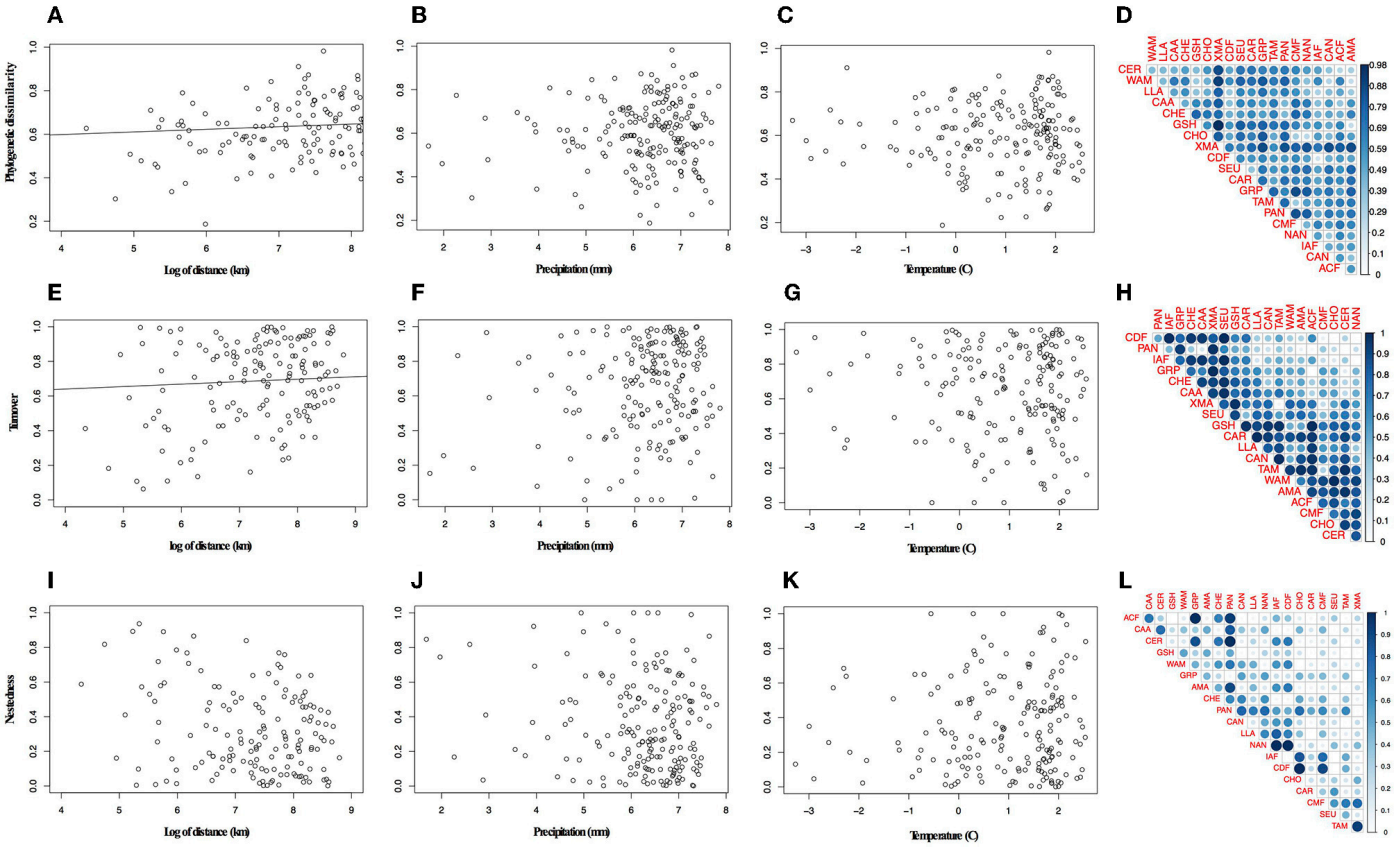
Relationship between phylogenetic dissimilarity and turnover component and distance. **(A)** Variation in phylogenetic dissimilarity with distance, **(B)** precipitation, **(C)** temperature, **(D)** pair-wise plot of the phylogenetic dissimilarity for bioregions. **(E)** Variation in turnover component with distance, **(F)** precipitation, **(G)** temperature, **(H)** pair-wise plot of the turnover component for bioregions. **(I-L)** In pair-wise plots the lateral blue bar represents the increasing of values represented in the graph.

Overall the species turnover component of phylogenetic dissimilarity was more frequent than the nestedness component (113 values above 0.6 threshold of turnover compared to 56 nestedness; Figures 2A,B and Table S6 in Appendix S4). Geographical variables better explained patterns in palm assembly. Phylogenetic dissimilarity (*r*^2^ = 0.27; *p* = 0.001; Figures 3A–D) and its turnover component (*r*^2^ = 0.12, *p* = 0.001; Figures 3E–H) and nestedness component (*r*^2^ = 0.12, *p* = 1.000; Figures 3I–L) increased with geographical distance. However, environmental variables were not correlated with phylogenetic dissimilarity. Precipitation did not affect phylogenetic dissimilarity (*r*^2^ = 0.003, *p* = 0.53; Figure 3B) or turnover (*r*^2^ = 0.002, *p* = 0.07; Figure 3F) and nestedness (*r*^2^ = 0.002, *p* = 0.07; Figure 3J). Similarly, temperature did not affect phylogenetic dissimilarity (*r*^2^ = 0.001, *p* = 0.99; Figure 3C) or turnover (*r* = 0.002, *p* = 0.49; Figure 3G) and nestedness (*r* = 0.002, *p* = 0.49; Figure 3K). Spatial autocorrelation was not significant for precipitation (Moran' I = −0.053; *p* = 0.08) or temperature (Moran' I = −0.053; *p* = 0.86).

Geographically closer bioregions tended to be phylogenetically more similar (Figure 2C). For instance, Amazonia (AMA) and the Guiana Shield (GSH) had low phylogenetic dissimilarity (0.226) with similar components of nestedness and turnover (50.9%), whereas the Atlantic Coastal forest (ACF) showed low phylogenetic dissimilarity with Caatinga (CAA; 0.371), with a higher component of nestedness (c. 71%; Table S6 in Appendix S4). We found a strong correlation between taxonomic and phylogenetic dissimilarity between areas (*r* = 0.87, *p* = 0.001), showing that indices are consistent with each other and we therefore present and interpret the results for taxonomic dissimilarity (Table S7 in Appendix S4).

**Figure 4:**
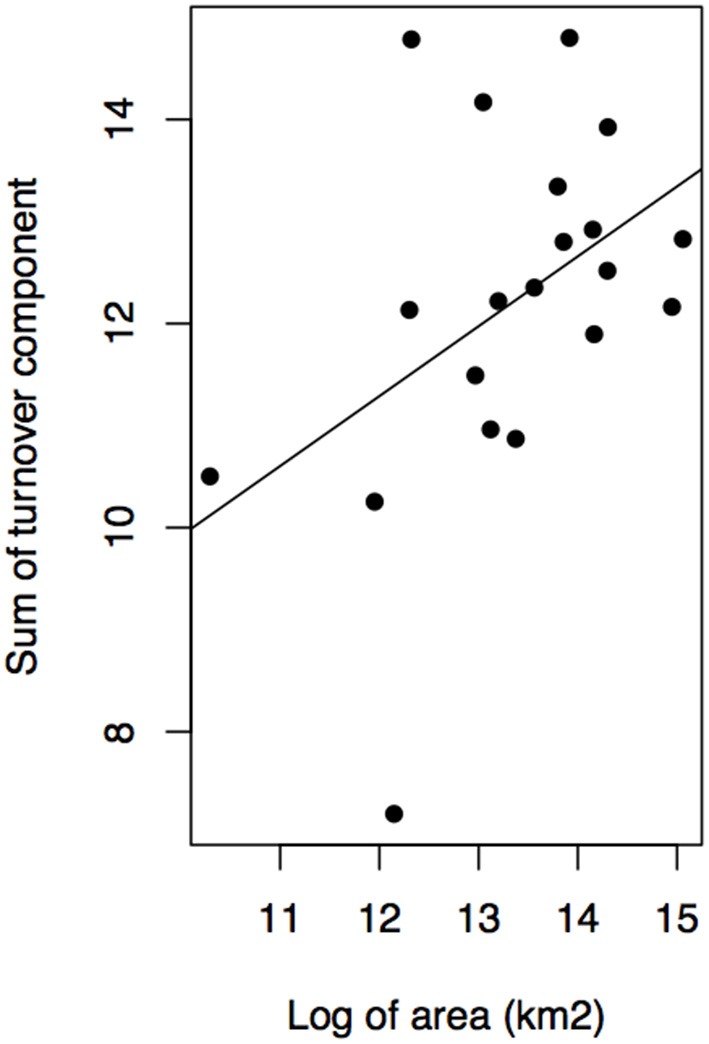
Contribution of turnover component of phylogenetic dissimilarity and bioregion area in kilometer square.

**Figure 5:**
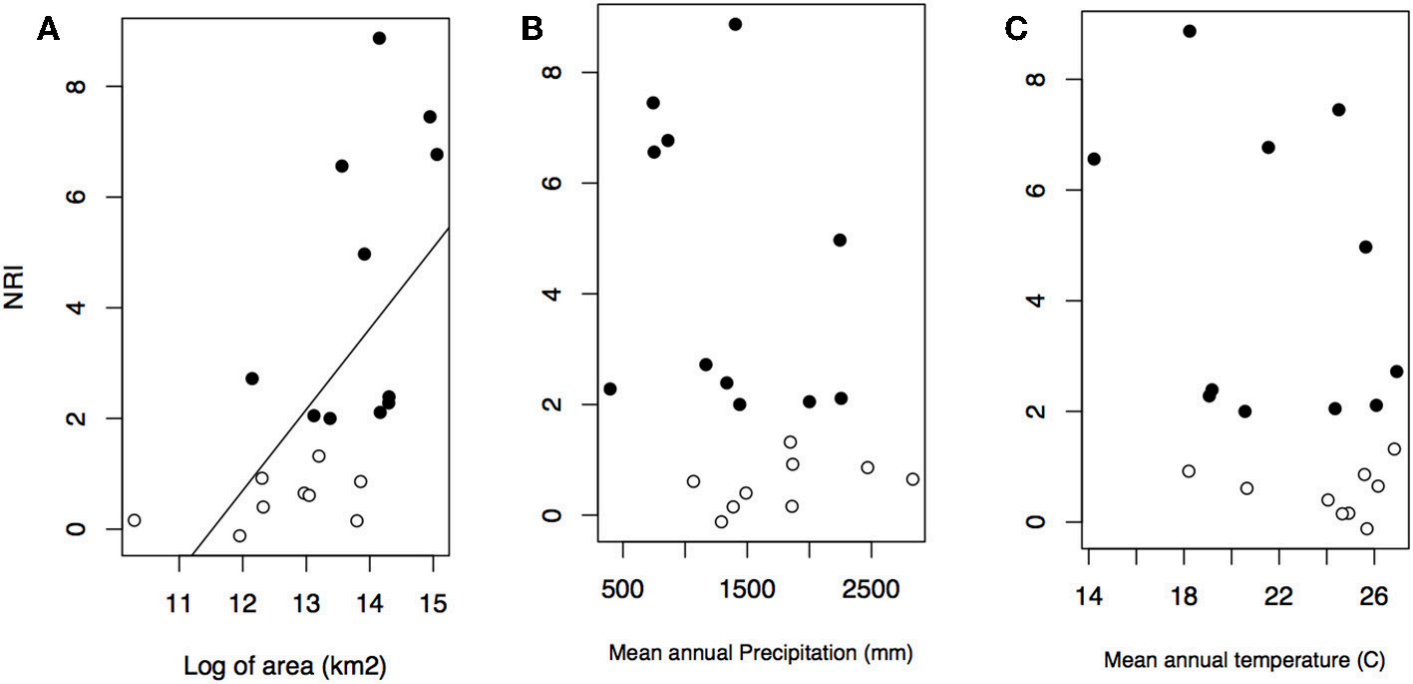
Relationship between NRI and bioregion area in kilometer square **(A)**, mean annual precipitation **(B)**, and mean annual temperature **(C)**. Black dots represent significant values of NRI.

Geographical variables better explained bioregions phylogenetic beta diversity and structure than environmental ones. Larger bioregions had a greater contribution to the turnover component (*r*^2^ = 0. 20, *p* = 0.05; Figure 4), and were more phylogenetically clustered than smaller ones (*r*^2^ = 0. 37, *p* = 0.004; Figure 5A). However, most bioregions were phylogenetically clustered (Table 1). NRI was not significantly related with precipitation (*r*^2^ = 0.15, *p* = 0.08, Figure 5B) and temperature (*r*^2^ = 0.16, *p* = 0.07; Figure 5C). NTI was significant in fewer cases but tended to corroborate NRI. NTI was not related with area (*r*^2^ = 0.13, *p* = 0.12), temperature (*r*^2^ = 0.05, *p* = 0.34) or precipitation (*r*^2^ = 0.05, *p* = 0.3).

### Clade Overabundance in Bioregions

**Figure 6:**
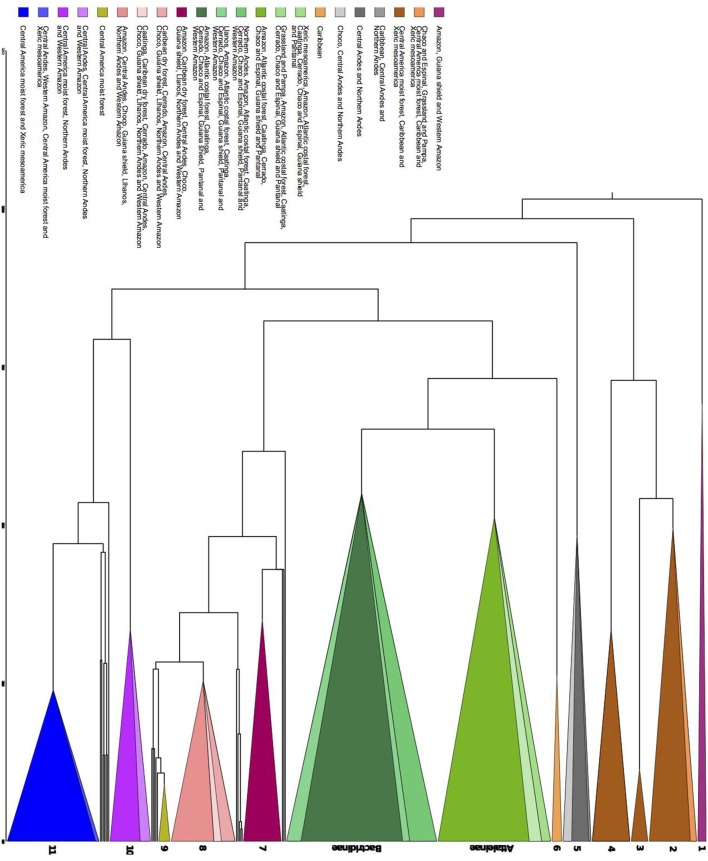
Chronogram representing the major clades that are significantly over abundant in the Nodesig analysis. The groups are as it follows, 1–Raphiinae, Mauritiinae, and Lepidocaryeae, 2–Crysophileae, detail of *Trithrinax*, 3–*Sabal*, 4–Unplaced Trachycarpeae, 5–Detail of *Pseudophoenix*, detail of Phytelepheae, Ceroxyloideae and Cyclospatheae, and detail of *Aphandra, Ammandra*, and *Phytelephas*, 6–*Roystonea*, Subtribe Attaleinae, detail of *Butia*, Subtribe Bactridinae detail of *Aiphanes, Bactris* and *Desmoncus*, 7–Euterpeae, 8–*Geonoma*, 9–*Calyptrogyne*, 10–Iriarteeae, detail of *Socratea* and *Iriartella*, 11–Chamadoreeae. Color represents sets of biomes where those major clades were overabundant (Appendix S5).

Regardless of the geographical distance or environment, bioregions shared similar over abundant clades (Figure 6). Seven areas with shared overabundant clades are adjacent and 13 are non-adjacent. For example, Atlantic Coastal forest (ACF) shared *Butia* with Cerrado (CER), Grassland and Pampa (GRP), and Chaco and Espinal (CHE), *Syagrus* + *Lytocaryum* + *Allagoptera* with Caatinga (CAA), Chaco and Espinal (CHE), and Cerrado (CER), but also shared clades with non-adjacent bioregions such as Amazonia (AMA) and Pantanal (PAN). CAA and CER shared clades with each other, but CAA shared *Desmoncus* + *Acrocomia* with the non-adjacent Pantanal (PAN) and Llanos (LLA) and part of *Geonoma* with Caribbean dry forests (CDF). Amazonia (AMA) also shared clades with adjacent bioregions such as Western Amazonia (WAM), Guiana Shield (GSH) and Caatinga (CAA), but also with non-adjacent ones such as Caribbean dry forest (CDF), Northern Andes (NAN), Choco (CHO), and Central Andes (CAN). Grasslands and Pampas (GRP) also shared the same species of *Trithrinax* with Caribbean (CAR), which had the most idiosyncratic clades, but also shared overabundant ones with non-adjacent bioregions such as Southern United States (SEU), Xeric Mesoamerica (XMA), and dry Tropical America (TAM; Appendix S5).

The Bactridinae palm lineage was overabundant across several South American bioregions (ACF, AMA, CAA, CER, CHE, GSH, PAN, and WAM) regardless of the adjacency. Few clades were overabundant in only one bioregion: *Aiphanes* (Northen Andes, NAN), a clade within *Bactris* and *Desmoncus* (Llanos, LLA), *Calyptrogyne* (Central America moist forest, CMF), *Roystonea* (Caribbean, CAR), and the clades *Socratea* and *Iriartella* (Central Andes, CAN).

### Shifts Between TRF and Non-TRF Bioregions

**Figure 7:**
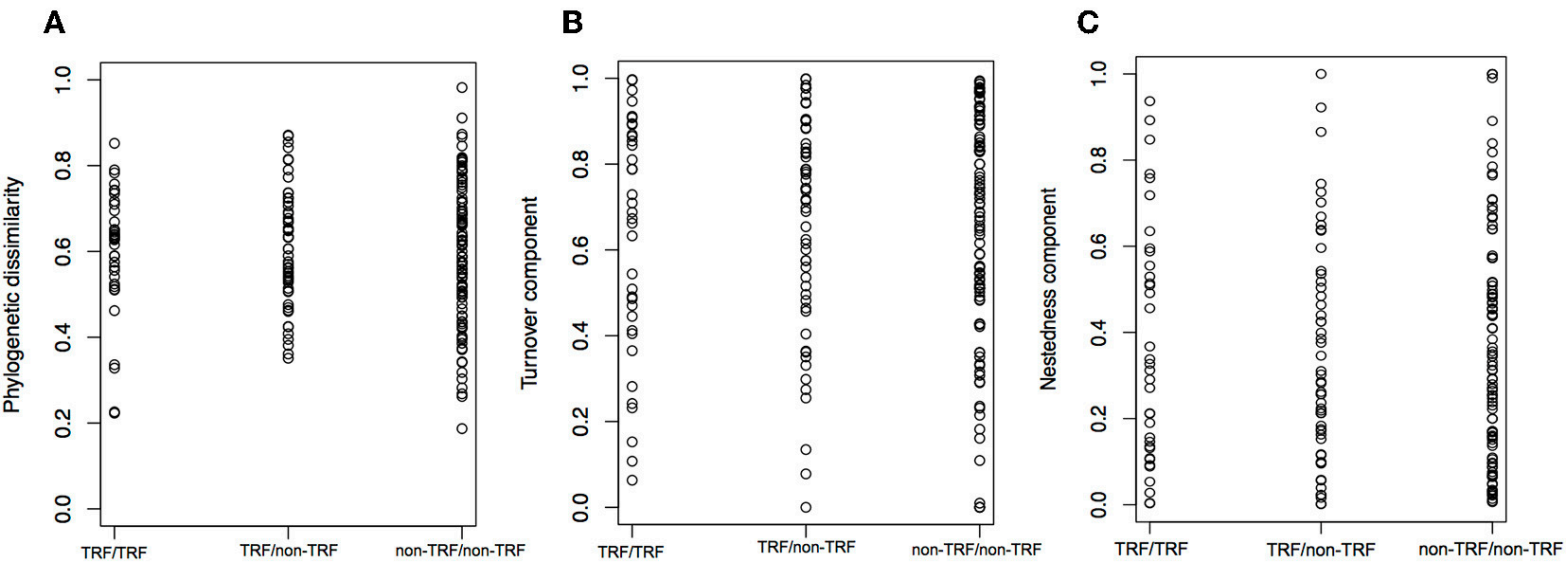
Distribution of phylogenetic dissimilarity **(A)**, turnover component **(B)**, and nestedness **(C)** between bioregion categories. TRF, tropical rain forest bioregions; non-TRF, non-tropical rain forest bioregions. The three comparisons are between TRF and TRF, TRF and non-TRF, and non-TRF and non-TRF bioregions. Each dot is a pairwise comparison.

**Figure 8:**
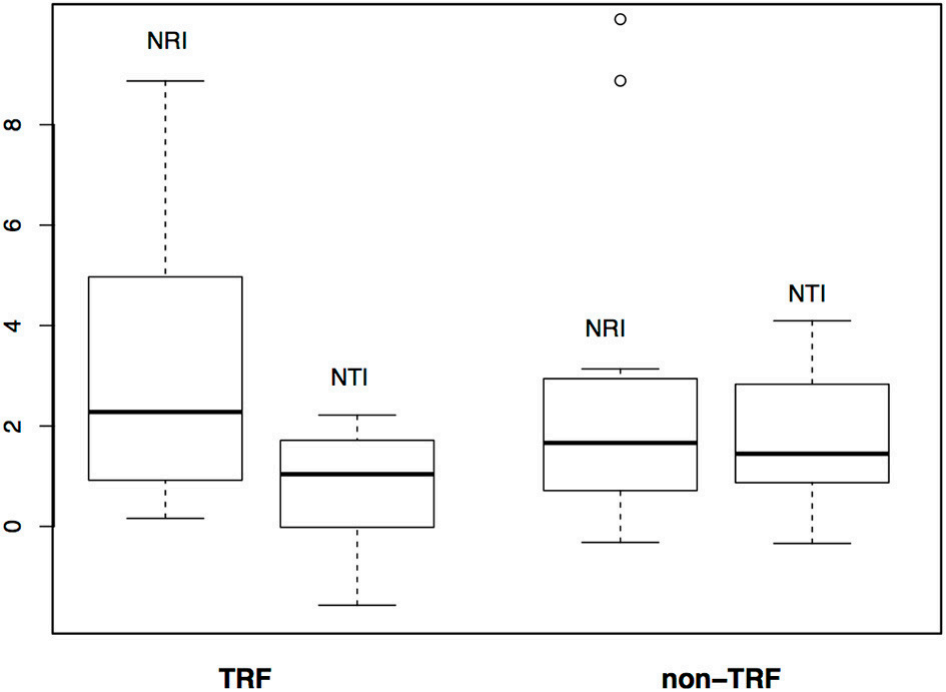
Comparison of phylogenetic structure between tropical rain forest (TRF) and non-tropical rain forest (non-TRF) bioregions based on NRI (Net Relatedness Index) and NTI (Nearest Taxon Index) values.

The mean annual temperature in TRF bioregions is 25°C (*SD* = 1.6°C), while for non-TRFs is 21°C (*SD* = 2.9°C), and the mean annual precipitation is 1,975.5 mm (*SD* = 557 mm) for TRF, and 1,175.1 mm (*SD* = 413 mm) for non-TRF (Figures S2, S3 in Appendix S3). We found no significant correlation between the phylogenetic dissimilarity (*r* = −0.022, *p* = 0.592, Figure 7A), turnover (*r* = −0.004, *p* = 0.483, Figure 7B) or nestedness (*r* = 0.004, *p* = 0.500, Figure 7C) and classes of bioregions (TRF and TRF, TRF and non-TRF and non-TRF and non-TRF). Finally, there was no significant difference in NRI (t = 0.83, *p* = 0.410) or NTI (*t* = −1.20, *p* = 0.240) between TRF and non-TRF classes (Figure 8).

### Ancestral Area Reconstruction and Lineage Shifts

**Figure 9:**
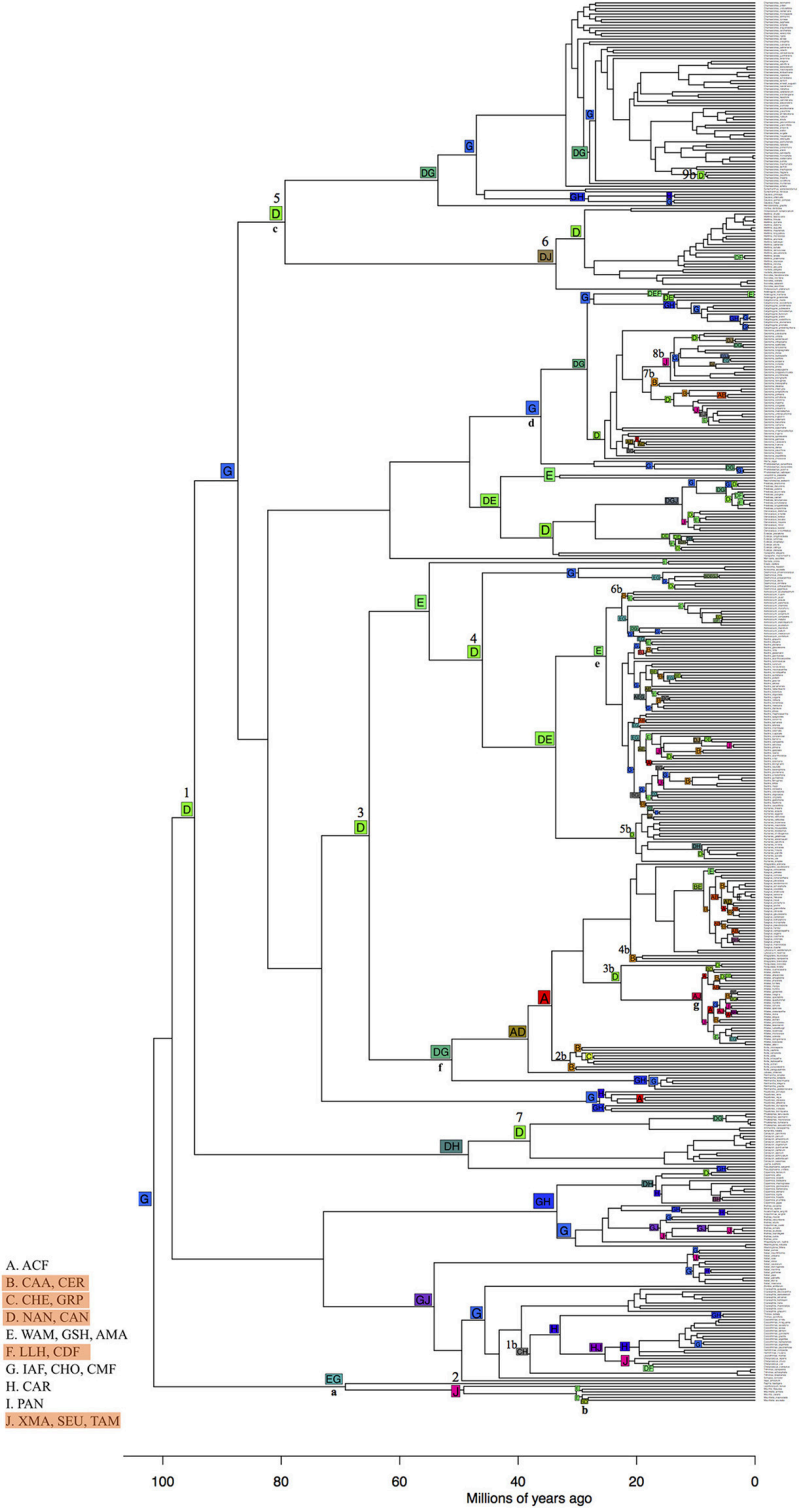
Ancestral bioregions inferred with BioGeoBears showing the most likely global optimization of 10 bioregions (total likelihood = −1,318). Dispersal parameter d = 0 and extinction parameter e = 0.062. non-TRF bioregions are highlighted in orange in legends. Numbers from 1 to 7 on top of the node squares represent transitions from TRF to non-TRF bioregions before 40 Ma. Numbers 1–9 followed by the letter “b” represent transitions from TRF to non-TRF after 40 Ma. Small bold letters (a–g) represent palm clades: a and b, Mauritiinae; c, Chamaedoreeae, Geonomateae and Iriarteae; d, Geonomateae; f, Attaleinae; g, *Attalea*. Capital letters in nodes correspond to the bioregions that were grouped according to the legends and numbers are clades described in the text. ACF, Atlantic Coastal forest; CAA, Caatinga; CER, Cerrado; CHE, Chaco and Espinal; GRP, Grasslands and Pampa; NAN, Northern Andes; CAN, Central Andes; WAM, Western Amazonia; GSH, Guiana shield; AMA, Amazonia; LLH, Llanos; CDF, Caribbean dry forest; IAF, Inter-Andean forest; CHO, Choco; CMF, Central American moist forest; CAR, Caribbean; PAN, Pantanal; XMA, Xeric Mesoamerica; SEU, Southeastern United States; TAM, Dry Tropical America.

TRFs such as the Central American moist forest (CMF), Inter-Andean forest (IAF) and Choco (CHO, G) are the most likely ancestral bioregions of Neotropical palms (Figure 9). We found more transitions from different bioregions, such as TRF to non-TRF bioregions (31) and non-TRF to TRF (24) than to similar bioregions, like TRF to TRF (11) and non-TRF to non-TRF (9). From 100 to 40 Ma there were seven transitions from TRF bioregions to non-TRF (clades 1–7 in Figure 9). The most ancient clade of Neotropical palms comprising *Mauritia* and *Mauritiella* spread out of the center of origin (G) northwards to more xeric regions and southwards into Amazonia (Figure 9, clade a) with later colonization of other xeric areas within South America (Caatinga and Cerrado; clade b). Chamaedoreeae, Geonomateae and Iriarteae originated in the Northern and Central Andes, non-TRF bioregions, more than 80 Ma (D; clade c, Figure 9), and later colonized the Inter-Andean forests, Choco and Central American moist forests (G), and drier areas in North (J) and north of South America (F). Geonomateae, mainly represented by *Geonoma*, originated in the Inter-Andean, Central American moist forest and Choco (G; clade d) colonized later the Atlantic forest (A) and Cerrado and Caatinga (B). Cocoseae originated in the Amazonia (E) and Northern and Central Andes (D; clade x). Bactridinae also originated in Amazonia (E; clade e) and recently (*c*. 20 Ma) colonized the Atlantic Coastal forest (A), and non-TRFs such as Cerrado, Caatinga (B), Northern and Central Andes (D) and the xeric regions of North America (J). Attaleinae (clade f) originated in the Central America moist forest, Inter-Andean forest and Choco (G) and Northern and Central Andes (D) and later (*c*. 40 Ma) colonized the Atlantic Coastal forest (A) and Cerrado and Caatinga (B), represented by *Allagoptera*, and into Grasslands and Pampa (C) represented by *Butia*. And finally, *Attalea* had a recent and widespread colonization pattern (clade g).

### Shifts in Diversification Rates

**Figure 10:**
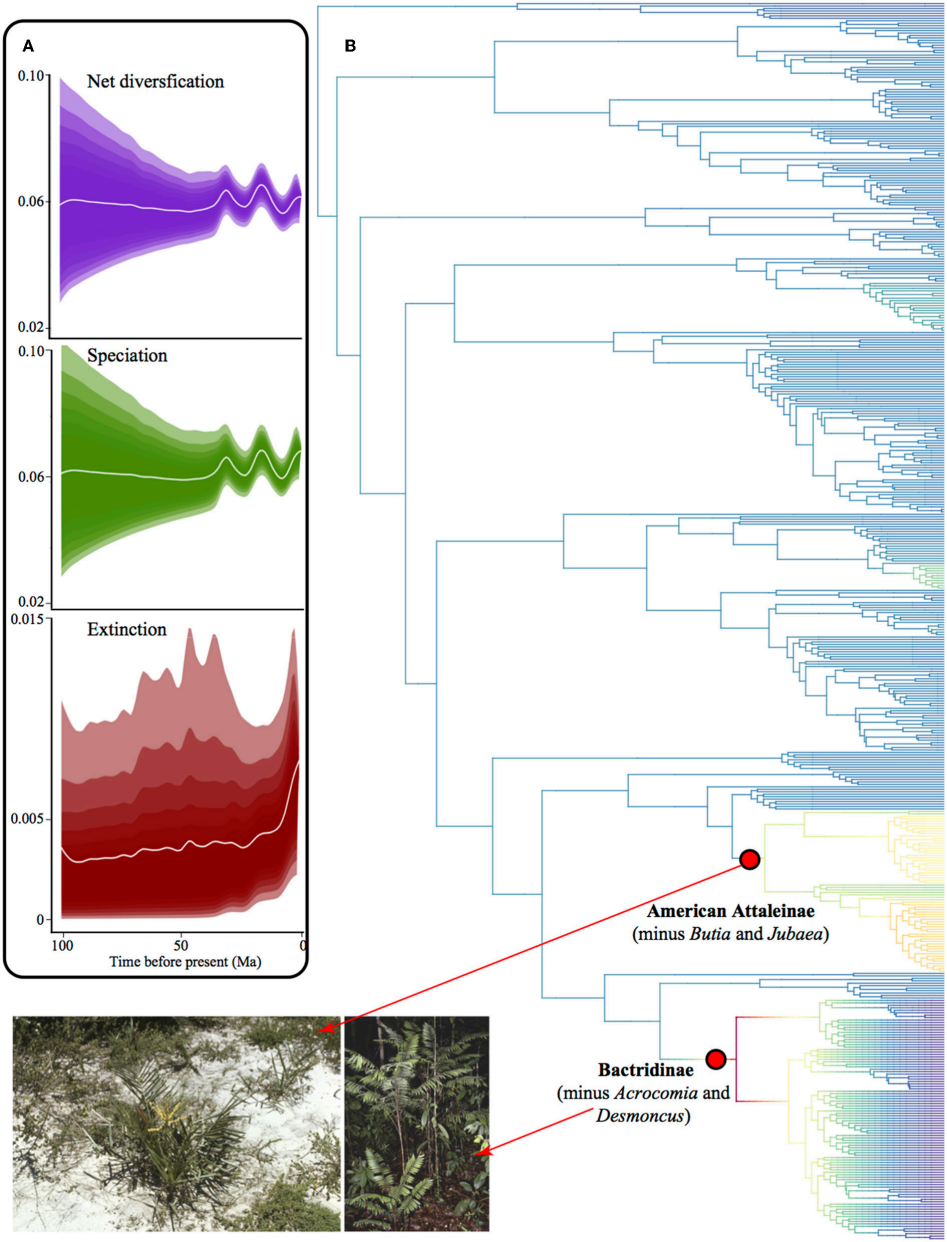
Diversification in American palms. **(A)** Phylorate plot of net diversification rates showing rates through time for net diversification, speciation, and extinction, where the shades comprise 95% of the data. **(B)** Phylogenetic tree of American palms depicting clades with shifts in diversification rates. Red dots represent the most probable rate shift configuration in the phylogenetic tree and indicate a rate increase. Photographs from palmweb.org showing the diversity of the clades involved in rate shifts, such as the species-rich genera *Bactris* and *Syagrus* that represent drastically different bioregions.

We found no difference between results from the Maximum Clade Credibility tree (MCC) and the random tree drawn from the 1,000 trees. Thus, we show here only the MCC tree. We detected only two diversification rate shifts, both rate increase although with low posterior probability for the shift configuration (0.30 posterior probability, Figure 10). One rate shift corresponds to the clade of American Attaleinae (excluding *Butia* and Jubaea) and the other Bactridinae species (excluding *Acrocomia* and *Desmoncus*). These clades are overabundant in several bioregions, including both TRF and non-TRF. Diversification rate through time was very similar to speciation rate because extinction rate was close to zero (Figure 10A)

## Discussion

Our findings show that geographical variables such as area and adjacency play an important role in the assembly of Neotropical palm communities. We corroborated our first hypothesis (Table 1, H1) that shifts to new bioregions are independent of environment differences as no correlation between phylogenetic turnover and environment was found. Although we found phylogenetic clustering in most bioregions, the phylogenetic clustering was correlated with geography. We also corroborated our second hypothesis (Table 1, H2) since we found strong evidence for larger bioregions having higher turnover and higher phylogenetic clustering than smaller ones. Additionally, phylogenetic dissimilarity and turnover increased with geographical distance, and the complimentary nestedness was higher between nearby bioregions, which indicates more shifts among adjacent bioregions. Moreover, our third hypothesis (Table 1, H3) was partially supported since we found no relationship between turnover or nestedness components and classes of bioregions, but we found some cases where lineages from non-TRF bioregions are nested in TRF, and TRF and non-TRF bioregions have no difference in phylogenetic structure. We also found several shifts from TRF to non-TRF bioregions in ancestral reconstruction analysis, corroborating our hypotheses that lineages shifts occurred more from TRF bioregions to non-TRF bioregions and that shifts are independent of environment differences.

Turnover was dominant over the nestedness component and larger bioregions harbor more closely related lineages than expected by chance, likely due to *in situ* diversification. The difference in species and lineage composition across bioregions was not related to the environment (e.g., temperature and mean annual precipitation). However, other variables not measured here might play a role as shown in Eiserhardt et al. (2013), although these authors did not also find effect of temperature and precipitation in phylogenetic beta diversity in palms. Environmental variables seem to play a role in palm species richness and composition at the continental scale (Kristiansen et al., 2011; Eiserhardt et al., 2013) and we cannot rule out the influence of these environmental variables (e.g., edaphic, topographic conditions and vegetation structure; Eiserhardt et al., 2013) in the phylogenetic assemblage of palm species. Edaphic and topographic variables are known to influence plant assembly in many habitats (Vormisto et al., 2004a,b).

Palm lineage assembly may be linked to stochastic events controlling the probability of colonization, speciation and extinction, the main drivers of the species-area relationship (e.g., MacArthur and Wilson, 1967; Hubbell, 2001), although species richness was not related to area (*r* = 0.30, *p* = 0.12, Figure S4 in Appendix S3). In our work, we found no evidence of differential diversification rates in different bioregions. In fact, only Attaleinae and Bactridinae clades showed shifts in diversification rates, and they are widespread and overabundant in many different bioregions, including TRF and non-TRF. In the same way, smaller bioregions showed slightly higher proportion of nestedness, which could indicate lineage shifts into a new climatic zone. Additionally, geographically distant bioregions tend to be more phylogenetically and taxonomically dissimilar and have higher turnover component of dissimilarity. Dispersal limitation could be responsible for this pattern (Nekola and White, 1999), but we did not assess species trait differences. Differences in speciation rates due to functional traits in different bioregions may have affected our results. In the Neotropics, seed traits related to dispersal, such as size and mass, may be related to speciation rate, together with geography (Onstein et al., 2017). At the continental scale, inconspicuous barriers, such as the drier Cerrado in between the moist Amazonia and the Atlantic forest might limit species dispersal and in turn cause phylogenetic turnover (Eiserhardt et al., 2013). However, the evidence of shifts coupled with the significant nestedness component found implies that the restrictions in niche evolution might not be as strong as previously thought. The differences found between this study and Eiserhardt et al. (2013) might also be due to the different spatial scales (e.g., Swenson et al., 2006).

We did not find a significant relationship between nestedness and distance, but we illustrate how nestedness was more likely to happen among adjacent bioregions supporting our finding that shifts primarily occur among adjacent areas despite differences in environment. For instance, Caatinga, Chaco, Grassland and Pampas, and Pantanal are nested within the Atlantic Coastal forest. In the case of Cerrado, Atlantic Coastal forest and Amazonia, the turnover was higher than the nestedness, but the dissimilarity was very low, meaning that Cerrado bioregions share lineages with TRF bioregions. A similar pattern was identified for Cerrado clades that have independently evolved fire-adaptations, where sister clades are found in adjacent fire-free bioregions (e.g., Amazonia and the Atlantic forest; Simon et al., 2009). Another example of how proximity might impact species assembly more than environmental constraints is the greater interchange of avian species between the Cerrado and adjacent bioregions, such as Amazonia, Atlantic Coastal forest, and Chaco, rather than with the far distant Amazonian savannas (Silva, 1995). Pennington et al. (2009) also pointed out the difference in the level of interchange between some bioregions and suggested that different processes dominate the assembly of biomes and that biomes differ in their permeability to successful colonization.

Regardless of the geographical distance or environment, bioregions shared similar overabundant clades indicating an underlying exchange of lineages. For example, Atlantic Coastal forest shared clades with adjacent smaller bioregions such as Grassland and Pampa and Chaco and Espinal, adjacent larger ones such as Cerrado, and also with both larger and smaller non-adjacent bioregions such as Amazonia and Pantanal. Caatinga shared clades with bioregions as far as Llanos and as close as Cerrado. Therefore, although turnover was dominant over nestedness, the contribution of nestedness together with similar overabundant clades are evidence that the Neotropical palm assembly is the result of both *in situ* diversification and shifts toward different climatic zones.

The lineage turnover pattern was widespread in all bioregions, both TRF and non-TRF, indicating a possible independent evolution of lineages within bioregions. Palms are suggested to have originated in TRFs (Couvreur et al., 2011) and our findings corroborate that, placing the origin of American palms in TRF bioregions such as the Inter-Andean forest, Choco, and the Central American moist forest. Further, we found evidence for shifts to have occurred preferentially between TRF and non-TRF bioregions, and non-TRF bioregions (e.g., Caatinga, Chaco and Espinal, Grassland and Pampa and Pantanal) are nested in TRF bioregions (Atlantic Coastal forest). Our results on phylogenetic beta diversity also shows examples of low dissimilarity between TRF and non-TRF bioregions (Table S6 in Appendix S4), indicating that the majority of clades occurring in non-TRFs also occur in TRF bioregions, and both present few exclusive clades.

Diversity patterns in palms are historically linked to tropical conditions (Dransfield et al., 2008) and to environment variables such as seasonality in precipitation (Kristiansen et al., 2011), humidity and temperature (Bjorholm et al., 2006). However, here we show that geographical variables also significantly affect the Neotropical palm, as it was also shown for some subfamilies (Bjorholm et al., 2005). Moreover, even though palms are iconic elements of humid forests, our results show multiple shifts between humid and dry environments, likely incurring adaptations to drier areas. Even though the overall pattern was skewed to lineage turnover among bioregions, we did find evidence of recent bioregional shifts. We argue that over evolutionary time, some species may have shifted to different bioregions, evading strong environmental constrains, potentially through adaptations to these new environments. Indeed, Townsend Peterson (2011) pointed out that the effects of niche conservatism in deeper evolutionary time are expected to decline. Diversity patterns are bounded to the environment, and palms are confined to tropical bioregions (Dransfield et al., 2008) and its richness patterns are linked with environmental variables such as seasonality in precipitation (Kristiansen et al., 2011). However, we show that, at a continental scale, palms are also influenced by geographical variables and some lineages have overcome this environmental filter while colonizing and spreading across Neotropical biomes throughout their evolutionary history.

The evidence for niche conservatism is overwhelming between tropical and temperate biotas (Wiens and Donoghue, 2004), mangroves (Ricklefs et al., 2006), grasslands (Hughes and Eastwood, 2006) and seasonally dry forests (Schrire et al., 2005; Pennington et al., 2009). Palms are also thought to be textbook examples of niche conservatism due to their restriction to tropical areas and its strong phylogenetic turnover (Eiserhardt et al., 2013). However, there are also numerous examples of plant lineages that show shifts to different habitats (Pennington et al., 2004; Alcantara et al., 2014; Donoghue and Edwards, 2014; Souza-Neto et al., 2016). Here we highlight how factors such as area and adjacency are also important drivers of the assembly of this iconic rainforest clade within the Neotropics, and how shifts into drier areas provide evidence for lesser environmental control than previously thought.

## Author Contributions

RC and CB conceived the study. CF and AS-N analyzed the data. RC, CB, and CF wrote the manuscript. All authors approved the final version of the manuscript.

### Conflict of Interest Statement

The authors declare that the research was conducted in the absence of any commercial or financial relationships that could be construed as a potential conflict of interest.
