# Supplementary material for: Adjacency and Area Explain Species Bioregional Shifts in Neotropical Palms
Source: Front Plant Sci. 2019 Feb 5;10:55. doi: 10.3389/fpls.2019.00055 (PMC6370682; doi:10.3389/fpls.2019.00055)
Supplement: Supplementary file 1 [file Data_Sheet_1.docx]

**Adjacency and area explain species bioregional shifts in Neotropical palms**

Cintia G. Freitas, Christine D. Bacon, Advaldo C. Souza-Neto, Rosane G. Collevatti

**Appendix S1.** GenBank numbers and sequences for the 337 species of American palms for chloroplast matK orf.

>Acoelorraphe_wrightii_HQ720485

GGAGATGCTACTCCTTTTACTGATGTCACAGTAAGTAAATACATAAAACCTTCATGCAAGCTTTACCTGA

GAAGCGTGTCATAATTTAACCTGCTATAAATGCTGGAGCTTTTTTGCTTATTCAGTTTAAGAATTCCTAG

TCACTGTATCTGATGTTAAAGAACATTAAGGAAAGTTTCTTTTGTTCTGCTCCAGGAGGAAGTAATTTTT

GCATTTGTCTAGCTAAAAGACATTGTTGATAGCTTATACAAAAACAGAAGGTTATTCAATTTTCATTACC

ATTGTGGATTTGCATCAGATATGGTTTAGTTAATTATAATCCTTCCAACTTCATACCTGGATTTGATTTT

CTTTTGAGGGGAGCTTTTGCCTTTGGAGTTCATATTTTCCTCTGGTTTAGTTAATTATATTCCTGTTATT

TGAAGTCTACTTTGAATATCTCTTTTGAAGTTTTTGAGATGTAAGTTTACCCTGTAGACAGAAACGTGGC

TTTGAAGAATGACTTTGGACGAGTGTAAGTTATTCTGATGATCCTAAACTTGTATAAGTTTCAAGACATA

CTATTTTGACTAAGGTCTCTTCTAAATTATTAGTAGACCATCTCCTTGTAACTGGCCTGTGACTTGGATT

TTCCAATTCGATGATCTATCGGGAGTTCTATCCTAAATACTGTTTCTAACATTGATTTCAGATGCATTCT

GATATAGGAGGTCAATATGTAATTGTTCTTTTGTAATTTCCCCATTACAAAAAATACTTATATCGTCACA

AATTTCCTAGGTGGACAATAT

>Acoelorraphe_wrightii_HQ720486.1

TCCTTTTACTGATGTCACAGTAAGTAAATACATAAAACCTTCATGCAAGCTTTACCTGAGAAGCGTGTCA

TAATTTAACCTGCTATAAATGCTGGAGCTTTTTTGCTTATTCAGTTTAAGAATTCCTAGTCACTGTATCT

GATGTTAAAGAACATTAAGGAAAGTTTCTTTTGTTCTGCTCCAGGAGGAAGTAATTTTTGCATTTGTCTA

GCTAAAAGACATTGTTGATAGCTTATACAAAAACAGAAGGTTATTCAATTTTCATTACCATTGTGGATTT

GCATCAGATATGGTTTAGTTAATTATAATCCTTCCAACTTCATACCTGGATTTGATTTTCTTTTGAGGGG

AGCTTTTGCCTTTGGAGTTCATATTTTCCTCTGGTTTAGTTAATTATATTCCTGTTATTTGAAGTCTACT

TTGAATATCTCTTTTGAAGTTTTTGAGATGTAAGTTTACCCTGTAGACAGAAACGTGGCTTTGAAGAATG

ACTTTGGACGAGTGTAAGTTATTCTGATGATCCTAAACTTGTATAAGTTTCAAGACATACTATTTKGACT

AAGGTCTCTTSTAAATTATTAGTAGACCATCTCCTTGTAACTGGCCTGTGACTTGGATTTTCCAATTCGA

TGATCTATCGGGAGTTCTATCCTAAATACTGTTTCTAACATTGATTTCAGATGCATTCTGATATAGGAGG

KCAATATGTAATTGTTCTTTTGTAATTTCCCCATTACAAAAAATACTTATATCGTCACAAATT

>Acoelorraphe_wrightii_EU215508.1

TTATTGAGTGCATCATGGGAAAGGTTGCTGCCCATATGGGAAAGGAGGGAGATGCTACTCCTTTTACTGA

TGTCACAGTAAGTAAATACATAAAACCTTCATGCAAGCTTTACCTGAGAAGCGTGTCATAATTTAACCTG

CTATAAATGCTGGAGCTTTTTTGCTTATTCAGTTTAAGAATTCCTAGTCACTGTATCTGATGTTAAAGAA

CATTAAGGAAAGTTTCTTTTGTTCTGCTCCAGGAGGAAGTAATTTTTGCATTTGTCTAGCTAAAAGACAT

TGTTGATAGCTTATACAAAAACAGAAGGTTATTCAATTTTCATTACCATTGTGGATTTGCATCAGATATG

GTTTAGTTAATTATAATCCTTCCAACTTCATACCTGGATTTGATTTTCTTTTGAGGGGAGCTTTTGCCTT

TGGAGTTCATATTTTCCTCTGGTTTAGTTAATTATATTCCTGTTATTTGAAGTCTACTTTGAATATCTCT

TTTGAAGTTTTTGAGATGTAAGTTTACCCTGTAGACAGAAACGTGGCTTTGAAGAATGACTTTGGACGAG

TGTAAGTTATTCTGATGATCCTAAACTTGTATAAGTTTCAAGACATACTATTTTGACTAAGGTCTCTTCT

AAATTATTAGTAGACCATCTCCTTGTAACTGGCCTGTGACTTGGATTTTCCAATTCGATGATCTATCGGG

AGTTCTATCCTAAATACTGTTTCTAACATTGATTTCAGATGCATTCTGATATAGGAGGTCAATATGTAAT

TGTTCTTTTGTAATTTCCCCATTACAAAAAATACTTATATCGTCACNNATTTCCTAGGTGGACAATATAA

GCAAAGCTCTTCATAAGTGTGGATATCAGA

>Acrocomia_aculeata_AJ830151.1

ATGCTACTCCTTTTACTGACGTCACAGTAAGTAAATCCATGGAACCTTCATGCAAGCTTTACCTGAGAAG

CATGTCTCAATTTAACTTGCTATAAATGCTGGAGTTTTTTTGCTTATTCAGTTTAAGAATTCCAAGGCAC

TGTATCTGATGTTAAAGAACATTATTTAAAGTTTCTTTTGTTCTGCTCGAGGAGGAAGTATTTTTTTTCC

ATGTGTCTAGCTAAAAGACATTGTTCATAGCTCGGACAAAATCACATGGTTATTCAATTTGCATTACCAT

TAAGGATTTCTACTAATATGGTTTAATAAATATGAATGAAACTGTTACCCGGAGTTTGCCTTTTTCGTAT

TTTCCTCTGGATTAGTTAATTATAATTCGGTTATTTGAAGTCTACTTTGAATCTCTCTTTTGAAGTCCTT

GATATGTAGGTTTACCCTGTAGACAAACGTGGCTCTGAACTCTGAGGAATGACCTTGGATGAGTGTCTTG

TATGTTATTCTGATGACCCTAAACTTGTATAAGTTTCAAGGCATGCTATTTTGACTAAGGTCTCTCCTAA

ATTATATCATTGACTATCTCCTTGTAACTGACCTGTGACTTGGTTTTTCCAATTCAATGACCTATCAGGA

GTTCTATCCTAAATAATGTTTCTAACATTGATTTCAGATATTTTCTGATGTAGGAGTTTAATATGTAATG

TACTTTTGTAGTTGCCCCATTACAGAAAATGCTTATATTATCACAAATTTCCTAGGTGGCCCACTCCACT

GA

>Acrocomia_crispa_HQ265621.1

GGGAANGGAGGNAGANGCTACTCCTTTTACTGACGTCACAGTAAGTAAATCCATGGAACCTTCATGCAAG

CTTTACCTGAGAAGCATGTCTCAATTTAACTTGCTACAAATGCTGGAGTTTTTTGCTTATTCAGTTTAAG

AATTCCAAGGCACTGTTATCTGATGTTNAAGAACATTATTTAAAGTTTCTTTTGTTCTGCTCAAGGAGGA

AGTATTTTTTTTTCCATGTGTCTAGCTAAAAGACATTGTTCATAGCTCGGACAAAAACACATGGTTATTC

AATTTGCATTACCATTAAGGATTTCTACTAATATGGTTTAATAAATATGAATGAAACTGTTACCCTTTGC

CTTTGGAGTTCATATTTTCCTCTGGATTAGTTAATTATAATTCGGTTATTTGAAGTCTACTTTGAATCTC

TCTTTTGAAGTCCTTGATATGTAGGTTTACCCAGTAGACAAACGTGGCTCTGAACTCTGAGGAATGACCT

TGGATGAGTGTCTTGTATGTTATTCTGATGACCCTAAACTTGTATAAGTTTCAAGGCATGCTATTTTGAC

TAAGGTCTCTCCTAAATTATATCATTGACTATCTCCTTGTAACTGACCTGTGACTTGGTTTTTCCAATTC

AATGACCTATCAGGAGTTCTATCCTAAATAATGTTTCTAACATTGATTTCAGATATTTTCTGATGTAGGA

GTTTAATATGTAATGTACNGNTTGTAGTTGCCCCATTACAGAAAATGCTTATATTATCACAAATTCCAGG

GGACA

>Acrocomia_aculeata_HQ265620.1

GGGAANGGAGGGAGANGCTACTCCTTTTACTGACGTCACAGTAAGTAAATCCATGGAACCTTCATGCAAG

CTTTACCTGAGAAGCATGTCTCAATTTAACTTGCTATAAATGCTGGAGTTTTTTTGCTTATTCAGTTTAA

GAATTCCAAGGCACTGTATCTGATGTTAAAGAACATTATTTAAAGTTTCTTTTGTTCTGCTCGAGGAGGA

AGTATTTTTTTTCCATGTGTCTAGCTAAAAGACATTGTTCATAGCTCGGACAAAATCACATGGTTATTCA

ATTTGCATTACCATTAAGGATTTCTACTAATATGGCTTAATAAATATGAATGAAACTGTTACCCTTTGCC

TTTGGAGTTCGTATTTTCCTCTGGATTAGTTAATTATAATTCGGTTATTTGAAGTCTACTTTGAATCTCT

CTTTTGAAGTCCTTGATATGTAGGTTTACCCTGTAGACAAACGTGGCTCTGAACTCTGAGGAATGACCTT

GGATGAGTGTCTTGTATGTTATTCTGATGACCCTAAACTTGTATAAGTTTCAAGGCATGCTATTTTGACT

AAGGTCTCTCCTAAATTATATCATTGACTATCTCCTTGTAACTGACCTGTGACTTGGTTTTTCCAATTCA

ATGACCTATCAGGAGTTCTATCCTAAATAATGTTTCTAACATTGATTTCAGATATTTTCTGATGTAGGAG

TTTAATATGTAATGNACGGNTGTAGTTGCCCCATTACAGAAAATGCTTATATTATCACAAATTCCTAGGG

GACA

>Aiphanes_horrida_EF491155.1

GCCCTCCTTTTACTGATGTCACAGTAAGTAAATCCATGGAACCTTCATGCAAGCTTTACCTGAGAAGCAT

ATCACAATTTAACTTGCTATAAATGCTGGAGCTTTTTTGGTTATTCAGTTTAAGAATCCCAAGGCACGGT

ATCGGATGTTAAAGAACATTATGTAAAGTTTCTTTTGTTCTGCTTGAGGAGGAATTATCTTTTTCCATGT

GTCTAGCTAAAAGACATTGTTGATAGCTCATACAACAACACATGGTTATTCAATTTGCATTACCATGAAG

GATGTCTACTGATATGGTTTAATAAATATGATTAGAACTGTTATCCTTTGCCTTTGGAGTTCATATTTTC

CTCTGGATTAGTTAATTATAATTCAGTTATTTGAAGTCTACTTTGAATCTCTTTTGAAGTCGTTGATCCG

TAAGTTTACCCTGTAGAATGACCTTGGACGAGTGTCTTGTATGTTATTCTGATGACTCTATACTTGTATA

AGTTTCAAGACATACTATTTTGACTAAGGTCTCTTATAAATTATATTATTGACCATCTCCTTGTAACTGA

CTTGTGACTTGGTTTTTCCAATTCAATGATCTATCTGGAGTTCTATCCTAAACAATGTTTTTAACATTGA

TTTCTGATATTTTCTGATGTAGGAGTTTCATATCTAATGTCTTTTTGTGGTTGCCTCAT

>Aiphanes_ulei_HQ265629.1

CGAATTCGCCCTTCAACTTATTGAGTGCATCATGGGAAAAGTTGCCGCCCATATGGGAAAGGAGGGAGAC

GCTACTCCTTTTACTGATGTCACAGTAAGTAAATCCATGGAACCTTCATGCAAGCTTTACCTGAGAAGCA

TATCACAATTTAACTTGCTATAAATGCTGGAGCTTTTTTGCTTATTCAGTTTAAGAATTCCAAGGCACAG

TATCGGATGTTAAAGAACATTATGTAAAGTTTCTTTTGTTCTGCTTGAGGAGGAAGTATTTTTTTCCATG

TGTCTAGCTAAAAGACATTGTTGATAGCTCATACAACAACACATGGTTATTCAATTTGCATTACCATGAA

GGATTTCTACTGATATGGTTTAATAAATATGATTAGAACTGTTACCCTTTGCCTTTGGAGTTCATATTTT

CCTCTGGATTAGTTAATTATAATTCAGTTATTTGAAGTCTACTTTGAATCTCTTTTGAAGTCGTTGATTC

GTAAGTTTACCCTGTAGAATGACCTTGGATGAGTGTCTTGTATGTTATTCTGATGACTCTATACTTGTAT

AAGTTTCAAGACATACTGTTTTGACTAAGGTCTCTTATAAATTATATTATTGACCATCTCCTTGTAACTG

ACTTGTGACTTGGTTTATCCAATTCAATGATCTATCTGGAGTTCTATCCTAAACAATGTTTCTAACATTG

ATTTCTGATATTTTCTGATGTAGGAGTTTCATATCTAATGTCTTTTTGTAGTTGCCTCATTACAAAAAAT

GCTTATATTATCACAAATTTCCTAGGTGGACAATATCAGCAAAGCTCTTCATAAGTGTGGATATCAGATG

CGTGGAAGGGCGAATCG

>Aiphanes_minima_HQ265627.1

GCATCANGGGAAAAGTTGCCGCCCATATGGGAAAGGAGGGAGACGCCACTCCTTTTACTGATGTCACAGG

TAAGTAAATCCATGGAACCTTCATGCAAGCTTTACCTGAGAAGCATATCACAATTTAACTTGCTATAAAT

GCTGGAGCTTTTTTGGTTATTCAGTTTAAGAATCCCAAGGCATGGTATCGGATGTTAAAGAACATTATGT

AAAGTTTCTTTTGTTCTGCTTGAGGAGGAAGTATCTTTTTCCATGTGTCTAGCTAAAAGACATTGTTGAT

AGCTCATACAACAACACATGGTTATTCAATTTGCATTACCATGAAGGATGTCTACTGATATGGTTTAATA

AATATGATTAGAACTGTTACCCTTTGCCTTTGGAGTTCATATTTTCCTCTGGATTAGTTAATTATAATTC

AGTTATTTGAAGTCTACTTTGAATTTCTTTTGAAGTCGTTGATCCGTAAGTTTACCCTGTAGAATGACCT

TGGACGAGTGTCTTGTATGTTATTCTGATGACTCTATACTTGTATAAGTTTCAAGACATACTATTTTGAC

TAAGGTCTCTTATAAATTATATTATTGACCATCTCCTTGTAACTGACTTGTGACTTGGTTTTTCCAATTC

AATGATCTATCTGGAGTTCTATCCTAAACAATGTTTTTAACATTGATTTCTGATATTTTCTGATGTAGGA

GTTTCATATCTAATGTCTTTTTGTGGTTGCCTCATTACAAAAAATGCTTATATTATCACAAATTTCCTAG

GTGGAAACATCAGCAAAGCTCTTCATAAGTGTGGATATCAGA

>Aiphanes_hirsuta_subsp_HQ265625.1

GCATCATGGGAAAAGTTGCCGCCCATATGGGAAAGGAGGGAGACGCTACTCCTTTTACTGATGTCACAGT

AAGTCAATCCATGGAACCTTCATGCAAGCTTTACCTGAGAAGCATATCACAATTTAACTTGCTATAAATG

CTGGAGCTTTTTTGCTTATTCAGTTTAAGAATTCAAAGGCACGGTATCGGATGTTAAAGAACATTATGTA

AAGTTTCTTTTGTTCTGCTTGAGGAGGAAGTATTTTTTTCCATGTGTCTAGCTAAAAGACATTGTTGATA

GCTCATACAACAACACATGGTTATTCAATTTGCATTACCATGAAGGATTTCTACTGATATGGTTTAATAA

ATATGATTAGAACTGTTACCCTTTGCCTTTGGAGTTCATATTTTCCTCTGGATTAGTTAATGATAATTCA

GTTATTTGAAGTCTACTTTGAATCTCTTTTGAAGTCGTTGATCCGTAAGTTTACCCTGTAGAATGACCTT

GGACGAGTGTCTTGTATGTTATTCTGATGACTCTATACTTGTATAAGTTTCAAGACATACTGTTTTGACT

AAGGTCTCTTATAAATTATATTATTGACCATCTCCTTGTAACTGACTTGTGATTTGGTTTTTCCAATTCA

ATGATCTGTCCGGAGTTCTATCCTAAACAATGTTTCTAACATTGATTTCTGATATTTTCTGATGTAGGAG

TTTCATATCTAATGTCTTTTTGTAGTTGCCTCATTACAAAAAATGCTTATAYTATCACAAATTTCCTAGG

TGGACAATATCAGCAAAGCTCTTCATAAGTGTGG

>Aiphanes_erinacea_HQ265623.1

TGGGAAAAGTTGCCGCCCATATGGGAAAGGAGGGAGACGCTACTCCTTTTACTGATGTCACAGTAAGTAA

ATCCATGGAACCTTCATGCAAGCTTTACCTGAGAAGCATATCACAATTTAACTTGCTATAAATGCTGGAG

YTTTTTTGCTTATTCAGTTTAAGAWTTCCAAGGCACGGTATCGGATGTTAAAGAACATTATGTAAAGTTT

CTTTTGTTCTKCTTGAGGAGGAAGTATTTTTTTCCATGTGTCTAGCTAAAAGACATTGTTGATAGCTCRT

ACAACAACACATGGTTATTCAATTTGCATTACCATGAAGGATTTCTACTGATATGGTTTAATAAATATGA

TTAGAACTGTTACCCTTTGCCKTTGGAGTTCATATTTTCCTCTGGATTAGTTAATTATAATTCAGTTATT

TGAAGTCTACTTTGAATCTCTCTTTTGAAGTCGTTGATCCGTAAGTTTACCCTGTAGAATGACCTTGGAC

GAGTGTCTTGTATGTTATTCTGATGACTCTATACTTGTATAAGTTTCAAGACATACTRTTTTGACTAAGG

TCTCTTATAAATTATATTATTGACSATCTCCTTGYAACTGACTTGTGACTTGGTTTTTCCAATTCAATGA

TCTATCMGGAGTTCTATCCTAAACAATGTTTCTACCATTGATTTCTGATATTTTCTSAGTGTAGGAGTTT

CATATCTAATGTCTTYTWGTAGYTGCCTCATTACAAAAAATGCTTATATYATCACAAATTCCTAGGTGGA

CAATATCAGCAAAGCTCTCCATAAGTGTG

>Aiphanes_spicata_HQ265628.1

CGAATTCGCCCTTCAACTTATTGAGTGCATCATGGGAAAAGTTGCCGCCCATATGGGAAAGGAGGGAGAC

GCTACTCCTTTTACTGATGTCACAGTAAGTAAATCCATGGAACCTTCATGCAAGCTTTTATCACAATTTA

ACTTGCTATAAATGCTGGAGCTTTTTTGCTTATTCAGTTTAAGAATTCCAAGGCACGGTATCGGATGTTA

AAGAACATTATGTAAAGTTTCTTTTGTTCTGCTTGAGGAGGAAGTATTTTTTTCCATGTGTCTAGCTAAA

AGACATTGTTGATAGCTCATACAACAACACATGGTTATTCAATTTGCATTACCATGAAGGATTTCTACTG

ATATTGTTTAATAAATATGATTAGAACTGTTACCCTTTGCCTTTGGAGTTCATATTTTCCTCTGGATTAG

TTAATTATAATTCAGTTATTTGAAGTCTACTTTGAATCTCTTTTGAAGTCGTTGATCCGTAAGTTTACCC

TGTAGAATGACCTTGGACGAGTGTCTTGTATGTTATTCTGATGACTCTATACTTGTATAACTGTTTTGAC

TAAGGTCTCTTATAAATTATATTATTGACCATCTCCTTGTAACTGACTTGTGACTTGGTTTTTCCAATTC

AATGATCTATCCGGAGTTCTATCCTAAACAATGTTTCTAACATTGATTTCTGATATTTTCTGATGTAGGA

GTTTCATATCTAATGACTTTTTGTAGTTGCCTCATTACAAAAAATGCTTATATTATCACAAATTTCCTAG

GTGGACAATATCAGCAAAGCTCTTCATAAGTGTGGATATCAGATGCGTGGAARGGCGAATTCG

>Aiphanes_killipiiHQ265626.1

CCCATATGGGAAAGGAGGGAGACGCTACTCCTTTTACTGATGTCACAGTAAGTAAATCCATGGAACCTTC

ATGCAAGCTTTACCTGAGAAGCGTGTCACAATTTAACTTGCTATAAAATGCTGGAGCTTTTTTGCTTATT

CAGTTTAAGAATACCAGGGCACTGTATTGGATGTTACAGAACATTATGTAAAGTTTCTTTTGTTCTGCTT

AAGGAGGAAGTATTTTTTCCATGGGTCTAGCTAAAAGACATTGTTGATAGCTTGTACAACAACACATGGT

TATTCAATTTGCATTACCATTAAAGATTTCTACTGATATGGTTTAATAAATATGATTGAAACTGTTACCC

TTTGCCTTTGGAGTTCATGTTTTCCTCTGGATTAGTTAATTATAATTCAATTATTTTAAGTCTACTTTGA

ATCTCTCTTTTGAAGTCCTTGATACGTAAGTTTACCCTGTAGACAGAGATGTGGCTCTGAAGAATGACCT

TGGACGAGCATCTTGTATGTTATTCTGATGACTCTATACTTATATAAGTTTCAAGACATACTATTTTGAC

TAAGGTCTCTTATAAATTATATTATTGACCATCTCCTTGTAACTGACCTGTGACTTGGTTTTTCCAATTC

AATGATCGATAGGAGTTCTATCCTAAACAATGTTTCTAACATTGATTTCAGATATTTTCTGATGTAGGAG

TTTAATATGTAATGTCTTTTTGTAGTTGCCTCATTACAGAAAATGCTTATATTATCACAAATTTCCTAGG

TGGACA

>Aiphanes_grandis_HQ265624.1

TGGGAAAAGTTGCCGCCCATATGGGAAAGGAGGGAGACGCTACTCCTTTTACTGATGTCACAGTAAGTAA

ATCCATGGAACCTTCATGCAAGCTTTACCTGAGAAGCATATCACAATTTAACTTGCTATAAATGCTGGAG

YTTTTTTGCTTATTCAGTTTAAGAWTTCCAAGGCACGGTATCGGATGTTAAAGAACATTATGTAAAGTTT

CTTTTGTTCTKCTTGAGGAGGAAGTATTTTTTTCCATGTGTCTAGCTAAAAGACATYGTTGATAGCTCRT

ACAACAACACATGGTTATTCAATTTGCATTACCATGAAGGATTTCTACTGATRTKGTTTAATAAATATGA

TTAGAACTGTTACCCTTTGCCKTTGGAGTTCATATTTTCCTCTGGATTAGTTAATTATAATTCAGTTATT

TGAAGTCTACTTTGAATCTCTCTTTTGAAGTCGTTGATCCGTAAGTTTACCCTGTAGAATGACCTTGGAC

GAGTGTCTTGTATGTTATTCTGATGACTCTATACTTGTATAAGTTTCAAGACATACTRTTTTGACTAAGG

TCTCTTATAAATTATATTATTGACSATCTCCTTGTAACTGATTTGTGACTTGGTTTTTCCAATTCAATGA

TCTATCCGGAGTTCTATCCTAAACAATGTTTCTAACATTGATTTCTGATATTTTCTGATGTAGGAGTTTC

ATATCTAATGTCTTTTTGTAGTTGCCTCATTACAAAAAATGCTTATATTATCACAAATTTCCTAGGTGGA

CAATATCAGCAAAGCTCTTCATAAGT

>Aiphanes_horrida_HQ265622.1

CCCATATGGGAAAGGAGGGAGACGCCACTCCTTTTACTGATGTCACAGGTAAGTAAATCCATGGAACCTT

CATGCAAGCTTTACCTGAGAAGCATATCACAATTTAACTTGCTATAAATGCTGGAGCTTTTTTGGTTATT

CAGTTTAAGAATCCCAAGGCACGGTGTATCGGATGTTAAAGAACATTATGTAAAGTTTCTTTTGTTCTGC

TTGAGGAGGAATTATCTTTTTCCATGTGTCTAGCTAAAAGACATTGTTGATAGCTCATACAACAACACAT

GGTTATTCAATTTGCATTACCATGAAGGATGTCTACTGATATGGTTTAATAAATATGATTAGAACTGTTA

TCCTTTGCCTTTGGAGTTCATATTTTCCTCTGGATTAGTTAATTATAATTCAGTTATTTGAAGTCTACTT

TGAATCTCTTTTGAAGTCGTTGATCCGTAAGTTTACCCTGTAGAATGACCTTGGACGAGTGTCTTGTATG

TTATTCTGATGACTCTATACTTGTATAAGTTTCAAGACATACTATTTTGACTAAGGTCTCTTATAAATTA

TATTATTGACCATCTCCTTGTAACTGACTTGTGACTTGGTTTTTCCAATTCAATGATCTATCTGGAGTTC

TATCCTAAACAATGTTTTTAACATTGATTTCTGATATTTTCTGATGTAGGAGTTTCATATCTAATGTCTT

TTTGTGGTTGCCTCATTACAAAAAATGCTTATATTATCACAAATTTCCTAGGTGGACA

>Allagoptera_arenaria_AJ830152.1

ATGCTACTCCTTTTACCGATGTCACAGTAAGTAAATCCATAGAACCTTCATGCAAACTTTACCTGAGAAG

CATGTCACAATTTAACTTGCTATAAATGCTGGAGCTTGTTTGCTTATTCAGTCTAAGAATTCCAAGTCAC

TGTATCTGATGTTAAAGAACATCATGGAAAATTTCTTTTGTTCTGCTCGAGGAGGAAGTAAGTTTTGCAT

GTGTCTGGCTAAATGACGTTGTTAGCTCATACAAAAATGCATGGTTATTCAATTTGCATTACCACTACCA

GATATGGTTTAATTAATATGTTTGAGTTGCATTACCATTAAGGATTTGTGCTAGATATGGTTTAATAAAT

ATGGGTGAGTTGCATTACCCAAGATGCGGGGGGGGCGAGGTGTTGTATAAAACCCTTCAACTACATACCC

GATTTTTTTTTTTTTTTTTTTTTTTTTTTTTTTTTTTTGGGGGTTGTTCAAATAACAGGATTATATTAAA

CTAATCCAGGTATGAAGTTGATGATATGTTAGTTTACCCTGTAGACAGAAACATGGCTCTGAAGATTAAC

CTTGGACGAGTGTCTTGTAAGTTATTCTGATGATCCTAAACTTGTATAAGTTTCAAGACGTACTATTTTG

ACTAAGGTCTCTCCTAAATTATGTCATTGACTATCTCCTTGTAATTGACCTGTGGCTTGGATTTTCCAAT

TCAATGATCTATCAGTTCTATCCTAAATAATGTTTCTGATATTGATTTCAGATATATTCTGATGTAGGAG

TTTAATATGTAATTGTACTTTTGTAGTTGCTCCATTACAAAAATGCTTATATTATCCCAAATT

>Polyandrococos_Allagoptera_JQ821997.1

GAAAGGAGGGAGATGCTACTCCTTTTACTGATGTCACAGTAAGTAAATCCATAGAACCTTCTTCCAAACT

TTACCTGAGAAGCATGTCACAATTTAACTTGCTATAAATGCTGGGGCTTGTTTGCTTATTCAGTCTAAGA

ATTCCAAGTCACTGTATCTGATGTTAAAGAACATCATGGAAAATTTCTTTTGTTCTGCTCGAGGAGGAAG

TAAGTTTTGCATGTGTCTGGCTAAATGACGTTGTTAGCTCATACAAAAATGCATGGTCATTCAATTTGCA

TTACCATTAAGGATTTGTACCAGATATGGTTTAGTTAATATGTTTGAGTTGCATTACCATTAAGGATTTG

TACTAGATATGGTTTAATAAATATGGTTGAGTTGCATTACCCAAGATGCGCGGGGGGGCGGTGGGGGAGG

GTTGGTGTTGATACCTGAATTTTTTATTTTTATTTTTTTGTGGGGGTGGTGGGGGTGGGGGAGCCTTTGC

CTTTGGAGTTCATATTTTCCTCTGGATTAGTTTATTATAATCCTGTTATTTGAAGTCTACTTTGAATATC

TCTTTTGAAGTTGATGATATGTTAGTTTACCCTGTAGACAGAAACATGGCTCTGAAGATTGACCTTGGAT

GAGTGTCTTGTAAGTTATTCTGGTGATCCTAAACTTGTATAAGTTTCAAGATGTACTATTTTGACTAAGG

TCTCTCCTAAATTATGTCATTGACTATCTCCTTGTAATTGACCTGTGGCTTGGATTTTCCAATTCAATGA

TCTATCAGGAGTTCTATCCTAAATAATGTTTCTAACATTGATTTCAGATATATTCCGATGTAGGAGTTTA

ATATGTAATTGTACTTCTGTAGTTGCTCCACTACAAAAATGCTTATATTATCACAAATTTCCTAGGTGGA

>Ammandra_decasperma_AY543096.1

GATGCTACTCCTTTTACTGATGTCACAGTAAGTAAATCCATGGAAGCTTCATGCAAGCTTTACCTGAGAA

GCATGTCACAATTTAATTTGCTATAAATGCTGGTGCTTTTTTTGCTTATTCAGTTTTAGAATTTCGAGGC

ACTGTATCTGATGTTAAAGAACACTAAGGAAGTTTTCTTTCATTCTGCTCCAGGAGGAAGTAATTTTTGC

ATTTGTCTAGCTAAAAGATATTTTGGATAGCTTGTACAAAAACACAAGGTTATTGAACTTGCATTACACC

TACACCATCGGCCCGCCCCTGCGGTGGGGGCCCATGCAGTGTCTAGAACCTTTCAACTTCATACCTGGAT

TTGTGTTTTTTATGGGGAGCTTTTTCTTATAAGCTATTCTGATGGTTCTAAACTTGTATAAGTTTCAGGA

CATACTATTTTGAGTAAGGTCTCTCCAAAATTATAGTGGACCATCTCCTTGTAACTGGCCTGTGACTTTG

ATTTTCCAATGCAATGATCTATCAGGAGTTCTATCCTAAATAATGTTTCTAACATTGATTTCAGATACAT

TCTGATGTACGAGTTTAATATGTAATTGTATTTTTGTAATTGCCCCATTACAAAAAATACTTATATTATC

>Ammandra_decasperma_EF128401.1

GTAAATCCATGGAAGCTTCATGCAAGCTTTACCTGAGAAGCATGTCACAATTWAATTTGCTATAAATGCT

GGTGCTTTTTTTGCTTATTCAGTTTTAGAATTTCGAGGCACTGTATCTGATGTTAAAGAACACTAAGGAA

GTTTTCTTTCATTCTGCTCCAGGAGGAAGTAATTTTTGCATTTGTCTAGCTAAAAGATATTTTGGATAGC

TTGTACAAAAACACAAGGTTATTGAACTTGCATTACACCTACACCATCGGCCCGCCCCTGCGGTGGGGGC

CCATGCAGTGTCTAGAACCTTTCAACTTCATACCTGGATTTGTGTTTTTTATGGGGAGCTTTTTCTTATA

AGCTATTCTGATGGTTCTAAACTTGTATAAGTTTCAGGACATACTATTTTGACTAAGGTCTCTCCAAAAT

TATAGTGGACCATCTCCTTGTAACTGGCCTGTGACTTTGATTTTCCAATGCAATGATCTATCAGGAGTTC

TATCCTAAATAATGTTTCTAACATTGATTTCAGATACATTCTGATGTACGAGTTTAATAGTAATTGTATT

TTTGTAATTGCCCCA

>Aphandra_natalia_AY779384.1

TTGCCTTTAGAGTTCTTATTTTCCTCTGGTTTAGTTAGTTATAATCCTGTAATTTGAAGTGTGCTTGAAT

ATCTCCTTTGAAGTTCTTGAGATGTGAGTTTACCCTGTACTGAAATGTGGCTTTGAAGAATGACCTTGGA

CGAGTGTCTTGTAAGTTATTTTGATGATCCTAAAGTTGTATTAGTTTCAAGACATGACATACTATTTTGA

CTAAGGCCTCTTCTAAATTATATAGTAGAGTATCTCCTTGTAACTGGCCTGTCACTTAGATTTTCCTATT

CAATGATCTATCAGGAGTTCTATGCTAAATAATGTTTCTAATGTTGATTTCTGATGTGTTCTGATGTAGG

AGTTTAATATGTAAGTGTACTTTTATAATTGTCCCATTGCTAAAAATACTTATATTATCACAAATTTCCT

AGGTGGACAATATCAGCAAAGCTCTTCATAAG

>Aphandra_natalia_EF128402.1

GTAAATCCATGGAAGCTTCATGCAAGCTTTACCTGAGAAGCATGTCACAATTTAATTTGCTATAAATGCT

GGTGCTTTTTTTGCTTATTCAGTTTTAGAATTTCGAGGCACTGTATCTGATGTTAAAGAACACTAAGGAA

GTTTTCTTTCATTCTGCTCCAGGAGGAAGTAATTTTTGCATTTGTCTAGCTAAAAGATATTTTGGATAGC

TTGTACAAAAACACAAGGTTATTGAACTTGCATTACACCTACACCATCGGCCCGCCCCTGCGGTGGGGGC

CCATGCAGCGTCTAGAACCTTTCAACTTCATACCTGGATTTGTGTTTTTTATGGGGAGCTTTTTCTTATA

AGCTATTCTGATGGTTCTAAACTTGTATAAGTTTCAGGACATACTATTTTGACTAAGGTCTCTCCAAAAT

TATAGTGGACCATCTCCTTGTAACTGGCCTGTGACTTCGATTTTCCAATGCAATGATCTATCAGGAGTTC

TATCCTAAATAATGTTTCTAACATTGATTTCAGATACATTCTGATGTACGAGTTTCATATATAATTGTAT

TTTTGTAATTGCCCCA

>Aphandra_natalia_AJ830153.1

CATCATGGAAAGGTTGCTGCCCATATGGGAAAGGAGGGAGATGCTACTCCTTTTACTGATGTCACAGTAA

GTAAATCCATGGAAGCTTCATGCAAGCTTTACCTGAGAAGCATGTCACAATTTAATTTGCTATAAATGCT

GGTGCTTTTTTTGCTTATTCAGTTTTAGAATTTCGAGGCACTGTATCTGATGTTAAAGAACACTAAGGAA

GTTTTCTTTCATTCTGCTCCAGGAGGAAGTAATTTTTGCATTTGTCTAGCTAAAAGATATTTTGGATAGC

TTGTACAAAAACACAAGGTTATTGAACTTGCATTACACCTACACCATCGGCCCGCCCCTGCGGTGGGGGC

ACATGCAGTGTCTAGAACCTTTCAACTTCATACCTGGATTTGTGTTTTTTATGGGGAGCTTTTTCTTATA

AGCTATTCTGATGGTTCTAAACTTGTATAAGTTTCAGGACATACTATTTTGACTAAGGTCTCTCCAAAAT

TATAGTGGACCATCTCCTTGTAACTGGCCTGTGACTTTGATTTTCCAATGCAATGATCTATCAGGAGTTC

TATCCTAAATAATGTTTCTAACATTGATTTCAGATACATTCTGATGTACGAGTTTAATATGTAATTGTAT

TTTTGTAATTGCCCCATTACAAAAAATACTTATATTATCACAAATTTCCTAGGTGGACAATATCAGCAAA

GCTCTTCATAAGTGTGA

>Asterogyne_guianensis_JQ417486.1

TTGCCTTTAGAGTTCATATTTTCCTTTGGATTAGTTAATTATAATTATGTTATTTGAAGTATACTTTAAA

TATCTCTTTTGAAGTTCTTGATATGTAAGTTTTCCGTAACGTGGCTTGAAGAATGACTTTGGATGAGTGT

CTTGTAAGTTATTCTGATGATCCTAAACTTGTATAAAGTTTCAGGACATACTATTTTGACCGATCTCTCT

CCTAAATTATATAATAGACTATCTCCTTGTAACTGACCTGTGACTTGGCTTTTCCAGTTCAATGATCTAT

CAGGAGTTCTGTCCTAAATAATGTTTCTAACATTGATTTCAGATATATTCTGATGTAGGAGTTTAATAGG

TAATGGTACTTTTCTAATTTCCCCATTGCAAAAAATGCTTATATTATCACAAATTTCCTAGGTGGACAAT

ATCAGCAAAGCTCTTCATAAG

>Asterogyne_martiana_AJ830154.1

CACAGTAAGTAAATCCATAGAACCTTCATGCAAGCTTTGCCTGAGAAGCATGTCACAATTTAACTTGCTA

TAAATGSTGGAGCTTTTTTGSTTATTCAGTTTAAGAATTCCAAGGSACTGTACCTGATGTTAAAGAACAT

CATGGAAAGTTTCTTTTGTTCTGCTCCAGGAGGAGGTAATTTTGCATATGTCTAGCTAAAAGACATTGTT

GATAGCTTATAGAAAAACACATGGTTATTCAATTTGCATTACCATTAAGGATTTGTACTAGATATGGTTT

AATTAATATGTTTGAGAAACAAGGGGGTCGTCTAGAACCCTTCAACTTCATACCTGGATTTTTTTTTTTT

GAGGGGGAGCACTTGCCTTTAGAGTTCATATTTTCCTTCGGATTAGTTAATTATAATCATGTTATTTGAA

GTATTCTTTAAATATCTCTTTTGAAGTTCTTGATATGTAAGTTTACTGTAACGTGGCTTGAAGAATGACT

TTGGACGAGTGTCTTGTAAGTTATTCTGATGATCCTAAACTTGTATAAAGTTTCAGGACATACTATTTGA

CCGATCTCTCTCCTAAATTATATAATAGACTATCTCCTTGTAACTGACCTGTGACTTGGCTTTTCCAGTT

CAATGATCTATCAGGAGTTCTGTCCTAAATAATGTTTCTAACATTGATTTCAGATATATTCTGATGTAGG

AGNTTAATAGGNNGGGGTACTTTTCTAATTTCCCCATTGCAAAAAANNGCTTATATTATCACAAATTTC

>Asterogyne_martiana_AY779370.1

TTGCCTTTAGAGTTCATATTTTCCTTCGGATTAGTTATTTATAATCATGTTATTTGAAGTATTCTTTAAA

TATCTCTTTTGAAGTTCTTGATATGTAAGTTTACTGTAACGTGGCTTGAAGAATGACTTTGGACGAGTGT

CTTGTAAGTTATTCTGATGATCCTAAACTTGTATAAAGTTTCAGGACATACTATTTTGACCGATCTCTCT

CCTAAATTATATAATAGACTATCTCCTTGTAACTGACCTGTGACTTGGCTTTTCCGGTTCAATGATCTAT

CAGGAGTTCTGTCCTAAATAATGTTTCTAACATTGATTTCAGATATATTCTGATGTAGGAGTTTAATAGG

TGATGGTACTTTTCTAATTTCCCCATTGCAAAAAATGCTTATATTATCACAAATTTCCTAGGTGGACAAT

ATCAGCAAAGCTCTTCATAAG

>Astrocaryum_sociale_JQ821995.1

GAAAGGAGGGAGATGCTACTCCTTTTACTGATGTCACAGTAAGTAAATCCATGGAACCTTCATGCAAGCT

TTACCTGAGAAGCATGTTACAATTTAACTTGCTATAAATGCTGGAGCTTTTTTGCTTATTCAGTTTAAGA

ATTCCAAGGCACTGCATCGGATGTTAAAGAATATTATGTAAAGTTTCTTTTGTTCTGCTTGAGGAGGAAG

TATTTTTTCCATGTGTCTAGCTAAAAGACATTGTTGATAGCTCGTACAATAACACATGGTTATTCAATTT

GCATTTCCATTAAGGATTTCTACTGATATGGTTTAATAAATATGATTGAAACTGTTACCCTTTGCCTTTG

GAGTTCATATTTTCCTCTGGATTAGTTAATTATAATTCAGTTATTTGAAGTCTACTTTGAATCTCTCTTT

TGACGTCCTTGATACGCAAGTTTACCCTGTAGACAGAAATGTGGCTCTGCAGAATGACCTTGGATGAGCG

TCTTGTATGTTATTCTGATGACCCTAAACTTGTATAAGTTTCAAGACATACTATTTTGACCAAGGTCTCT

TCTCAATTATATCATTGACCATCTCCTTGTAACTGATCTGTGACTTGGTTTTTCCAATTCAATGATCTAT

CAGGAGTTCTATCCTAAATAATGTTTCTAACATTGATTTTAGATATTTTCTGATGTAGGAGTTTAATATG

TAATGTCCTTTTGTAGTTGCCTCATTACAGAAAATGCTTATATTATCACCAATTCCTAG

>Astrocaryum_sciophilum_JQ821993.1

GAAAGGAGGGAGATGCTACTCCTTTTACTGATGTCACAGTAAGTAAATCCATGGAACCTTCATGCAAGCT

TTACCTGAGAAGCATGTTACAATTTAACTTGCTATAAATGCTGGAGCTTTTTTGCTTATTCAGTTTAAGA

ATTCCAAAGGCACTGCATCGGATGTTAAAGAATATTATGTAAAGTTTCTTTTGTTCTGCTTGAGGAGGAA

GTATTTTTTCCATGTGTCTAGCTAAAAGACATTGTTGATAGCTCGTACAATAACACATGGTTATTCAATT

TGCATTTCCATTAAGGATTTCTACTGATATGGTTTAATAAATATGATTGAAACTGTTACCCTTTGCCTTT

GGAGTTCATATTTTCCTCTGGATTAGTTAATTATAATTCAGTTATTTGAAGTCTACTTTGAATCTCTCTT

TTGACGTCCTTGATACGCAAGTTTACCCTGTAGACAGAAATGTGGCTCTGCAGAATGACCTTGGATGAGC

GTCTTGTATGTTATTCTGATGACCCTAAACTTGTATAAGTTTCAAGACATACTATTTTGACCAAGGTCTC

TTCTCAATTATATCATTGACCATCTCCTTGTAACTGATCTGTGACTTGGTTTTTCCAATTCAATGATCTA

TCAGGAGTTCTATCCTAAATAATGTTTCTACCATTGATTTTAGATATTTTCTGATGTAGGAGTTTAATAT

GTAATGTCCTTTTGTAGTTGCCTCATTACAGAAAATGCTTATATTATCACCAATTTCCTAGGTGGA

>Astrocaryum_perangustatum_JQ821991.1

GAAAGGAGGGAGATGCTACTCCTTTTACTGATGTCACAGTAAGTAAATCCATGGAACCTTCATGCAAGCT

TTACCTGAGAAGCATGTTACAATTTAACTTGCTATAAATGCTGGAGCTTTTTTGCTTATTCAGTTTAAGA

ATTCCAAGGCACTGCATCGGATGTTAAAGAATATTATGTAAAGTTTCTTTTGTTCTGCTTGAGGAGGAAG

TATTTTTTCCATGTGTCTAGCTAAAAGACATTGTTGATAGCTCGTACAATAACACATGGTTATTCAATTT

GCATTTCCATTAAGGATTTCTACTGATATGGTTTAATAAATATGATTGAAACTGTTACCCTTTGCCTTTG

GAGTTCATATTTTCCTCTGGATTAGTTAATTATAATTCAGTTATTTGAAGTCTACTTTGAATCTCTCTTT

TGACGTCCTTGATACGCAAGTTTACCCTGTAGACAGAAATGTGGCTCTGCAGAATGACCTTGGATGAGCG

TCTTGTATGTTATTCTGATGACCCTAWACTTGTATAAGTTTCAAGACATACTATTTTGACCAAGGTCTCT

TCTCAATTATATCATTGACCATCTCCTTGTAACTGATCTGTGACTTGGTTTTTCCAATTCAATGATCTAT

CAGGAGTTCTATCCTAAATAATGTTTCTAACATTGATTTCAGATATTTTCTGATGTAGGAGTTTAATATG

TAATGTCCTTTTGTAGTTGCCTCATTACAGAAAATGCTTATATTATCACCAATTCCTAGGTGGA

>Astrocaryum_macrocalyx_JQ821989.1

GAAAGGAGGGAGATGCTACTCCTTTTACTGATGTCACAGTAAGTAAATCCATGGAACCTTCATGCAAGCT

TTACCTGAGAAGCATGTTACAATTTAACTTGCTATAAATGCTGGAGCTTTTTTGCTTATTCAGTTTAAGA

ATTCCAAGGCACTGCATCGGATGTTAAAGAATATTATGTAAAGTTTCTTTTGTTCTGCTTGAGGAGGAAG

TATTTTTTCCATGTGTCTAGCTAAAAGACATTGTTGATAGCTCGTACAATAACACATGGTTATTCAATTT

GCATTTCCATTAAGGATTTCTACTGATATGGTTTAATAAATATGATTGAAACTGTTACCCTTTGCCTTTG

GAGTTCATATTTTCCTCTGGATTAGTTAATTATAATTCAGTTATTTGAAGTCTACTTTGAATCTCTCTTT

TGACGTCCTTGATACGCAAGTTTACCCTGTAGACAGAAATGTGGCTCTGCAGAATGACCTTGGATGAGCG

TCTTGTATGTTATTCTGATGACCCTAAACTTGTATAAGTTTCAAGACATACTATTTTGACCAAGGTCTCT

TCTCAATTATATCATTGACCATCTCCTTGTAACTGATCTGTGACTTGGTTTTTCCAATTCAATGATCTAT

CAGGAGTTCTATCCTAAATAATGTTTCTAACATTGATTTCAGATATTTTCTGATGTAGGAGTTTAATATG

TAATGTCCTTTTGTAGTTGCCTCATTACAGAAAATGCTTATATTATCACCAATTCCTAG

>Astrocaryum_jauari_JQ821987.1

GAAAGGAGGGAGATGCTACTCCTTTTACTGATGTCACAGTAAGTAAATCCATGGAACCTTCATGCAAGCT

TTACCTGAGAAGCATGTTGCAATTTAACTTGCTATAAATGCTGGAGCTTTTTTGCTTATTCAGTTTAGGA

ATTCCAAGGCACTGTATCGGATGTTAAAGAATATTATGTAAAGTTTCTTTTGTTCTGCTCGAGGAGGAAG

TATTTTTTCCATATGTCTAGCTAAAGGACATTGTTGATAGCTCGTACAACAACACATGGTTATTCATTTT

GCATTTCCATTAAGGATTTCTACTGATATGGTTTAATAAATATGATTGAAACTGTTACCCTTTGCCTTTG

GAGTTCATATTTTCCTCTGGATTAGTTAATTATAATTCAGTTATTTGAAGTCTACTTTGAATCTCTCTTT

TGAAGTCCTTGATACGTAAGTTTACCCTGTAGACAGAAATGTGGCTCTGAAGAATGACCTTGGATGAGCG

TCTTGTATGTTATTCTAATGACCCTAAACTTGTATAAGTTTCAAGACATACTATTTTGACCAAGGTCTCT

TCTCAATTATATCATTGACCATCTCCTTGTAACTGATCTGTGACTTGGTTTTTTCAATTCAATGATCTAT

CAGGAGTTCTATCCTAAATAATGTTTCTAACATTGATTTCAGATATTTTCTGATGTAGGAGTTTAATATG

TAATGTCCTTTTGTAGTTGCCTCATTACAGAAAATGCTTATATTATCACAAATTCCTAG

>Astrocaryum_gratum_JQ821985.1

GAAAGGAGGGAGATGCTACTCCTTTTACTGATGTCACAGTAAGTAAATCCATGGAACCTTCATGCAAGCT

TTACCTGAGAAGCATGTTACAATTTAACTTGCTATAAATGCTGGAGCTTTTTTGCTTATACAGTTTAAGA

ATTCCAAGGCACCGCATCGGATGTTAAAGAATATTATGTAAAGTTTCTTTTGTTCTGCTTGAGGAGGAAG

TATTTTTTTCCATGTGTCTAGCTAAAAGACATTGTTGATAGCTCGTACAATAACACATGGTTATTCAATT

TGCATATCCATTAAGGATTTCTACTGATATGGTTTAATAAATATGATTGAAACTGTTACCCTTTGCCTTT

GGAGTTCATATTTTCCTCTGGATTAGTTAATTATAATTCAGTTATTTGAAGTCTACTTTGAATCTCTCTT

TTGACGTCCTTGATACGCAAGTTTACCCTGTAGACAGAAATGTGGCTCTGCAGAATGACCTTGGATGAGC

GTCTTGTATGTTATTCTGATGACCCTAAACTTGTATAAGTTTCAAGACATACTATTTTGACCAAGGTCTC

TTCTCAATTATATCATTGACCATCTCCTTGTAACTGATCTGTGACTTGGTTTTTCCAATTCAATGATCTA

TCAGGAGTTCTATCCTAAATAATGTTTCTAACATTGATTTCAGATATTTTCTGATGTAGGAGTTTAATAT

GTAATGTCCTTTTGTAGTTGCCTCATTACAGAAAATGCTTATATTATCACCAATTTCCTAGGTGGA

>Astrocaryum_farinosum_JQ821983.1

GAAAGGAGGGAGATGCTACTCCTTTTACTGATGTCACAGTAAGTAAATCCATGGAACCTTCATGCAAGCT

TTACCTGAGAAGCATGTTACAATTTAACTTGCTATAAATGCTGGAGCTTTTTTGCTTATTCAGTTTAAGA

ATTCCAAGGCACTGCATCGGATGTTAAAGAATATTATGTAAAGTTTCTTTTGTTCTGCTTGAGGAGGAAG

TATTTTTTCCATGTGTCTAGCTAAAAGACATTGTTGATAGCTCGTACAATAACACATGGTTATTCAATTT

GCATTTCCATTAAGGATTTCTACTGATATGGTTTAATAAATATGATTGAAACTGTTACCCTTTGCCTTTG

GAGTTCATATTTTCCTCTGGATTAGTTAATTATAATTCAGTTATTTGAAGTCTACTTTGAATCTCTCTTT

TGACGTCCTTGATACGCAAGTTTACCCTGTAGACAGAAATGTGGCTCTGCAGAATGACCTTGGATGAGCG

TCTTGTATGTTATTCTGATGACCCTAAACTTGTATAAGTTTCAAGACATACTATTTTGACCAAGGTCTCT

TCTCAATTATATCATTGACCATCTCCTTGTAACTGATCTGTGACTTGGTTTTTCCAATTCAATGATCTAT

CAGGAGTTCTATCCTAAATAATGTTTCTAACATTGATTTTAGATATTTTCTGATGTAGGAGTTTAATATG

TAATGTCCTTTTGTAGTTGCCTCATTACAGAAAATGCTTATATTATCACCAATTCCTAGGTGGA

>Astrocaryum_chonta_JQ821981.1

CTGATGTCACAGTAAGTAAATCCATGGAACCTTCATGCAAGCTTTACCTGAGAAGCATGTTACAATTTAA

CTTGCTATAAATGCTGGAGCTTTTTTGCTTATTCAGTTTAAGAATTCCAAGGCACTGCATCGGATGTTAA

AGAATATTATGTAAAGTTTCTTTTGTTCTGCTTGAGGAGGAAGTATATTTTCCATGTGTCTAGCTAAAAG

ACATTGTTGATAGCTCGTACAATAACACATGGTTATTCAATTTGCATTTCCATTAAGGATTTCTACTGAT

ATGGTTTAATAAATATGATTGAAACTGTTACCCTTTGCCTTTGGAGTTCATATTTTCCTCTGGATTAGTT

AATTATAATTCAGTTATTTGAAGTCTACTTTGAATCTCTCTTTTGACGTCCTTGATACGCAAGTTTACCC

TGTAGACAGAAATGTGGCTCTGCAGAATGACCTTGGATGAGCGTCTTGTATGTTATTCTGATGACCCTAA

ACTTGTATAAGTTTCAAGACATACTATTTTGACCAAGGTCTCTTCTCAATTATATCATTGACCATCTCCT

TGTAACTGATCTGTGACTTGGTTTTTCCAATTCAATGATCTATCAGGAGTTCTATCCTAAATAATGTTTC

TAACATTGATTTCAGATATTTTCTGATGTAGGAGTTTAATATGTAATGTCCTTTTGTAGTTGCCTCATTA

CAGAAAATGCTTATATTATCACCAATT

>Astrocaryum_carnosum_JQ821979.1

GGAGATGCTACTCCTTTTACTGATGTCACAGTAAGTAAATCCATGGAACCTTCATGCAAGCTTTACCTGA

GAAGCATGTTACAATTTAACTTGCTATAAATGCTGGAGCTTTTTTGCTTATTCAGTTTAAGAATTCCAAG

GCACTGCATCGGATGTTAAAGAATATTATGTAAAGTTTCTTTTGATCTGCTTGAGGAGGAAGTATTTTTT

CCATGTGTCTAGCTAAAAGACATTGTTGATAGCCCGTACAATAACACATGGTTATTCAATTTGCATTTCC

ATTAAGGATTTCTACTGATATGGTTTAATAAATATGATTGAAACTGTTACCCTTTGCCTTTGGAGTTCAT

ATTTTCCTCTGGATTAGTTAATTATAATTCAGTTATTTGAAGTCTACTTTGAATCTCTCTTTTGACGTCC

TTGATACGCAAGTTTACCCTGTAGACAGAAATGTGGCTCTGCAGAATGACCTTGGATGAGCGTCTTGTAT

GTTATTCTGATGACCCTAAACTTGTATAAGTTTCAAGACATACTATTTTGACCAAGGTCTCTTCTCAATT

ATATCATTGACCATCTCCTTGTAACTGATCTGTGACTTGGTTTTTCCAATTCAATGATCTATCAGGAGTT

CTATCCTAAATAATGTTTCTAACATTGATTTCAGATATTTTCTGATGTAGGAGTTTAATATGTAATGTCC

TTTTGTAGTTGCCTCATTACTAGAAAATGCTTATATTATCACCAATTCCTAGGTG

>Astrocaryum_aculeatum_JQ821977.1

GAAAGGAGGGAGATGCTACTCCTTTTACTGATGTCACAGTAAGTAAATCCATGGAACCTTCATGCAAGCT

TTACCTGAGAAGCATGTTACAATTTAACTTGCTATAAATGCTGGAGCTTTTTTGCTTATTCAGTTTAAGA

ATTCCAAGGCACTGCATCGGATGTTAAAGAATATTATGTAAAGTTTCTTTTGTTCTGCTTGAGGAGGAAG

TATTTTTTCCATGTGTCTAGCTAAAAGACATTGTTGATAGCTCGTACAATAACACATGGTTATTCAATTT

GCATTTCCATTAAGGATTTCTACTGATATGGTTTAATAAATATGATTGAAACTGTTACCCTTTGCCTTTG

GAGTTCATATTTTCCTCTGGATTAGTTAATTATAATTCAGTTATTTGAAGTCTACTTTGAATCTCTCTTT

TGACGTCCTTGATACGCAAGTTTACCCTGTAGACAGAAATGTGGCTCTGCAGAATGACCTTGGATGAGTG

TCTTGTATGTTATTCTGATGACCCTAAACTTGTATAAGTTTCAAGACATACTATTTTGACCAAGGTCTCT

TCTCAATTATATCATTGACCATCTCCTTGTAACTGATCTGTGACTTGGTTTTTCCAATTCAATGATCTAT

CAGGAGTTCTATCCTAAATAATGTTTCTAACATTGATTTCAGATATTTTCTGATGTAGGAGTTTAATATG

TAATGTCCTTTTGTAGTTGCCTCACTACAGAAAATGCTTATATTATCACCAATTTCCTAGGTGGA

>Astrocaryum_ulei_JQ821996.1

GAAAGGAGGGAGATGCTACTCCTTTTACTGATGTCACAGTAAGTAAATCCATGGAACCTTCATGCAAGCT

TTACCTGAGAAGCGTGTTACAATTTAACTTGCTATAAATGCTGGAGCTTTTTTGCTTATTCAGTTTAAGA

ATTCCAAGGCACTGCATCGGATGTTAAAGAATATTATGTAAAGTTTCTTTTGTTCTGCTTGAGGAGGAAG

TATATTTTCCATGTGTCTAGCTAAAAGACATTGTTGATAGCTCGTACAATAACACATGGTTATTCAATTT

GCATTTCCATTAAGGATTTCTACTGATATGGTTTAATAAATATGATTGAAACTGTTACCCTTTGCCTTTG

GAGTTCATATTTTCCTCTGGATTAGTTAATTATAATTCAGTTATTTGAAGTCTACTTTGAATCTCTCTTT

TGACGTCCTTGATACGCAAGTTTACCCTGTAGACAGAAATGTGGCTCTGCAGAATGACCTTGGATGAGCG

TCTTGTATGTTATTCTGATGACCCTAAACTTGTATAAGTTTCAAGACATACTATTTTGACCAAGGTCTCT

TCTCAATTATATCATTGACCATCTCCTTGTAACTGATCTGTGACTTGGTTTTTCCAATTCAATGATCTAT

CAGGAGTTCTATCCTAAATAATGTTTCTAACATTGATTTCAGATATTTTCTGATGTAGGAGTTTAATATG

TAATGTCCTTTTGTAGTTGCCTCATTACAGAAAATGCTTATATTATCACCAATTTCCTAGGTGGA

>Astrocaryum_scopatum_JQ821994.1

GAGATGCTACTCCTTTTACTGATGTCACAGTAAGTAAATCCATGGAACCTTCATGCAAGCTTTACCTGAG

AAGCATGTTACAATTTAACTTGCTATAAATGCTGGAGCTTTTTTGCTTATTCAGTTTAAGAATTCCAAGG

CACTGCATCGGATGTTAAAGAATATTATGTAAAGTTTCTTTTGTTCTGCTTGAGGAGGAAGTATATTTTC

CATGTGTCTAGCTAAAAGACATTGTTGATAGCTYGTACAATAACACATGGTTATTCAATTTGCATTTCCA

TTAAGGATTTCTACTGATATGGTTTAATAAATATGATTGAAACTGTTACCCTTTGCCTTTGGAGTTCATA

TTTTCCTCTGGATTAGTTAATTATAATTCAGTTATTTGAAGTCTACTTTGAATCTCTCTTTTGACGTCCT

TGATACGCAAGTTTACCCTGTAGACAGAAATGTGGCTCTGCAGAATGACCTTGGATGAGCGTCTTGTATG

TTATTCTGATGACCCTAAACTTGTATAAGTTTCAAGACATACTATTTTGACCAAGGTCTCTTCTCAATTA

TATCATTGACCATCTCCTTGTAACTGATCTGTGACTTGGTTTTTCCAATTCAATGATCTATCAGGAGTTC

TATCCTAAATAATGTTTCTAACATTGATTTCAGATATTTTCTGATGTAGGAGTTTAATATGTAATGTCCT

TTTGTAGTTGCCTCATTACAGAAAATGCTTATATTATCACCAATTCCTAGTG

>Astrocaryum_rodriguesii_JQ821992.1

AGCTTTTTTGCTTATTCAGTTTAAGAATTCCAAGGCACTGTATCGGATGTTAAAGAATATTATGTAAAGT

TTCTTTTGTTCTGCTCGAGGAGGAAGTATTTTTTCCATGTGTCTAGCTAAAAGACATTGTTGATAGCTTG

TACAACAACACATGGTTATTCAAGTTGCATTTCCATTAAGGATTTCTACTGATATGGTTTAATAAATATG

ATTGAAACTGTTACCCTTTGCCTTTGGAGTTCATATTTTCCTCTGGATTAGTTAATTATAATTCAGTTAT

TTGAAGTCTACTTTGAATCTCTCTTTTGAAGTCCTTGGTACGCAAGTTTACCCTGTAGACAGAAATGTGG

CTCTGCAGAATGACCTTGGATGAGCGTCTTGTATGTTATTCTGATGACCCTAAACTTGTATAAGTTTCAA

GACATACTATTTTGACCAAGGTCTCTTCTCAATTATATCATTGACCATCTCCTTGTAACTGATCTGTGAC

TTGGTTTTTCCAATTCAATGATCTATCAGGAGTTCTATCCTAAATAATGTTTCTAACATTGATTTCAGAT

ATTTTCTGATGTAGGAGTTTAATATGTAATGTCCTTTTGTAGTTGCCTCATTACAGAAAATGCTTATATT

ATCACCAATTTCCTAGGTGGA

>Astrocaryum_paramaca_JQ821990.1

GAAAGGAGGGAGTTGCTACTCCTTTTACTGATGTCACAGTAAGTAAATCCATGGAACCTTCATGCAAGCT

TTACCTGAGAAGCATGTTACAATTTAACTTGCTATAAATGCTGGAGCTTATTTGCTTATTCAGTTTAAGA

ATTCCAAGGCACTGTATCGGATGTTAAAGAATATTATGTAAGGTTTCTTTTGTTCTGCTCGAGGAGGAAG

TATTTTTTCCATGTGTCTAGCTAAAAGACATTGTTGATAGCTTGTACAACAACACATGGTTATTCAAGTT

GCATTTCCATTAAGGATTTCTACTGATATGGTTTAATAAATATGATTGAAACTGTTACCCTCTGCCTTTG

GAGTTCATATTTTCCTCTGGATTAGTTAATTATAATTCAGTTATTTGAAGTCTACTTTGAATCTCTCTTT

TGAAGTCCTTGGTACGCAAGTTTACCCTGTAGACAGAAATGTGGCTCTGCAGAATGACCTTGGATGAGCG

TCTTGTATGTTATTCTGATGACCCTAAACTTGTATAAGTTTCAAGACATACTATTTTGACCAAGGTCTCT

TCTCAATTATATCATTGACCATCTCCTTGTAACTGATCTGTGACTTGGTTTTTCCAATTCAATGATCTAT

CAGGAGTTCTATCCTAAATAATGTTTCTAACATTGATTTCAGATATTTTCTGATGTAGGAGTTTAATATG

TAATGTCCTTTTGTAGTTGCCTCATTACAGAAAATGCTTATATTATCACCAATTTCCTAGGTGGA

>Astrocaryum_javarense_JQ821988.1

GAAAGGAGGGAGANGCTACTCCTTTTACTGATGTCACAGTAAGTAAATCCATGGAACCTTCATNCAAGCT

TTACCTGAGAAGCATGTTACAATTTAACTTGCTATAAATGCTGGAGCTTTTTNGCTTATTCAGTTTAAGA

ATTCCAAGGCACCGCATCGGATGTTAAAGAATATTATGTAAAGTTTCTTTTGTTCTGCTTGAGGAGGAAG

TATTTTTTTCCATGTGTCTAGCTAAAAGACATTGTTGATAGCTCGTACAATAACACATGGTTATTCAATT

TGCATATCCATTAAGGATTTCTACTGATATGGTTTAATAAATATGATTGAAACTGTTACCCTTTGCCTTT

GGAGTTCATATTTTCCTCTGGATTAGTTAATTATAATTCAGTTATTTGAAGTCTACTTTGAATCTCTCTT

TTGACGTCCTTGATACGCAAGTTTACCCTGTAGACAGAAATGTGGCTCTGCAGAATGACCTTGGATGAGC

GTCTTGTATGTTATTCTGATGACCCTAAACTTGTATAAGTTTCAAGACATACTATTTTGACCAAGGTCTC

TTCTCAATTATATCATTGACCATCTCCTTGTAACTGATCTGTGACTTGGTTTTTCCAATTCAATGATCTA

TCAGGAGTTCTATCCTAAATAATGTTTCTAACATTGATTTCAGATATTTTCTGATGTAGGAGTTTAATAT

GTAATGTCCTTTTGTAGTTGCCTCATTACAGAAAATGCTTATATTATCACCAATTTCCTAGGTGGA

>Astrocaryum_huicungo_JQ821986.1

CCTGAGAAGCATGTTACAATTTAACTTGCTATAAATGCTGGAGCTTTTTTGCTTATTCAGTTTAAGAATT

CCAAGGCACTGCATCGGATGTTAAAGAATATTATGTAAAGTTTCTTTTGTTCTGCTTGAGGAGGAAGTAT

TTTTTCCATGTGTCTAGCTAAAAGACATTGTTGATAGCTCGTACAATAACACATGGTTATTCAATTTGCA

TTTCCATTAAGGATTTCTACTGATATGGTTTAATAAATATGATTGAAACTGTTACCCTTTGCCTTTGGAG

TTCATATTTTCCTCTGGATTAGTTAATTATAATTCAGTTATTTGAAGTCTACTTTGAATCTCTCTTTTGA

CGTCCTTGATACGCAAGTTTACCCTGTAGACAGAAATGTGGCTCTGCAGAATGACCTTGGATGAGCGTCT

TGTATGTTATTCTGATGACCCTATACTTGTATAAGTTTCAAGACATACTATTTTGACCAAGGTCTCTTCT

CAATTATATCATTGACCATCTCCTTGTAACTGATCTGTGACTTGGTTTTTCCAATTCAATGATCTATCAG

GAGTTCTATCCTAAATAATGTTTCTAACATTGATTTCAGATATTTTCTGATGTAGGAGTTTAATATGTAA

TGTCCTTTTGTAGTTGCCTCATTACAGAAAATGCTTATATTATCACCAATTCCTAG

>Astrocaryum_ferrugineum_JQ821984.1

GAAAGGAGGGAGATGCTACTCCTTTTACTGATGTCACAGTAAGTAAATCCATGGAACCTTCATGCAAGCT

TTACCTGAGAAGCATGTTACAATTTAACTTGCTATAAATGCTGGAGCTTTTTTGCTTATTCAGTTTAAGA

ATTCCAAGGCACTGCATCGGATGTTAAAGAATATTATGTAAAGTTTCTTTTGTTCTGCTTGAGGAGGAAG

TATTTTTTCCATGTGTCTAGCTAAAAGACATTGTTGATAGCTCGTACAATAACACATGGTTATTCAATTT

GCATTTCCATTAAGGATTTCTACTGATATGGTTTAATAAATATGATTGAAACTGTTACCCTTTGCCTTTG

GAGTTCATATTTTCCTCTGGATTAGTTAATTATAATTCAGTTATTTGAAGTCTACTTTGAATCTCTCTTT

TGACGTCCTTGATACGCAAGTTTACCCTGTAGACAGAAATGTGGCTCTGCAGAATGACCTTGGATGAGYG

TCTTGTATGTTATTCTGATGACCCTAAACTTGTATAAGTTTCAAGACATACTATTTTGACCAAGGTCTCT

TCTCAATTATATCATTGACCATCTCCTTGTAACTGATCTGTGACTTGGTTTTTCCAATTCAATGATCTAT

CAGGAGTTCTATCCTAAATAATGTTTCTAACATTGATTTCAGATATTTTCTGATGTAGGAGTTTAATATG

TAATGTCCTTTTGTAGTTGCCTCATTACAGAAAATGCTTATATTATCACCAATTCCTAGGTGGA

>Astrocaryum_faranae_JQ821982.1

GAAAGGAGGGAGATGCTACTCCTTTTACTGATGTCACAGTAAGTAAATCCATGGAACCTTCATGCAAGCT

TTACCTGAGAAGCATGTTACAATTTAACTTGCTATAAATGCTGGAGCTTTTTTGCTTATTCAGTTTAAGA

ATTCCAAGGCACCGCATCGGATGTTAAAGAATATTATGTAAAGTTTCTTTTGTTCTGCTTGAGGAGGAAG

TATTTTTTTCCATGTGTCTAGCTAAAAGACATTGTTGATAGCTCGTACAATAACACATGGTTATTCAATT

TGCATATCCATTAAGGATTTCTACTGATATGGTTTAATAAATATGATTGAAACTGTTACCCTTTGCCTTT

GGAGTTCATATTTTCCTCTGGATTAGTTAATTATAATTCAGTTATTTGAAGTCTACTTTGAATCTCTCTT

TTGACGTCCTTGATACGCAAGTTTACCCTGTAGACAGAAATGTGGCTCTGCAGAATGACCTTGGATGAGC

GTCTTGTATGTTATTCTGATGACCCTAAACTTGTATAAGTTTCAAGATATACTATTTTGACCAAGGTCTC

TTCTCAATTATATCATTGACCATCTCCTTGTAACTGATCTGTGACTTGGTTTTTCCAATTCAATGATCTA

TCAGGAGTTCTATCCTAAATAATGTTTCTAACATTGATTTCAGATATTTTCTGATGTAGGAGTTTAATAT

GTAATGTCCTTTTGTAGTTGCCTCATTACAGAAAATGCTTATATTATCACCAATTTCCTAGGTGGA

>Astrocaryum_chambira_JQ821980.1

AGATGCTACTCCTTTTACTGATGTCACAGTAAGTAAATCCATGGAACCTTCATGCAAGCTTTACCTGAGA

AAGCAATGTTACAAATTTAACTTGCTATAAAATGCTGGAGCTTTTTTGCTTATTCAGTTTAAGAATTCCA

AGGCACTGTATCGGATGTTAAAGAATATTATGTAAAGTTTCTTTTGTTCTGCTTGAGGAGGAAGTATTTT

TTCCATGTGTCTAGCTAAAAGACATTGTTGATAGCTCGTACAACAACACATGGTTATTCAATTTGCATTT

CCATTAAGGATTTCTACTGATATGGTTTAATAAATATGATTGAAACTGTTACCCTTTGCCTTTGGAGTTC

ATATTTTCCTCTGGATTAGTTAATTATAATTCAGTTATTTGAAGTCTACTTTGAATCTCTCTTTTGAAGT

CCTTGATACGCAAGTTTACCCTGTAGACAGAAATGTGGCTCTGCAGAATGACCTTGGATGAGCGTCTTGT

ATGTTATTCTGATGACCCTAAACTTGTATAAGTTTCAAGACATACTATTTTGACCAAGGTCTCTTCTCAA

TTATATCATTGACCATCTCCTTGTAACTGATCTGTGACTTGGTTTTTCCAATTCAATGATCTATCAGGAG

TTCTATCCTAAATAATGTTTCTAACATTGATTTCAGATATTTTCTGATGTAGGAGTTTAATATGTAATGT

CCTTTTGTAGTTGCCTCATTACAGAAAATGCTTATATTATCACCAATTTCCTAGGTGGA

>Astrocaryum_campestre_JQ821978.1

GAAAGGAGGGAGATGCTACTCCTTTTACTGATGTCACAGTAAGTAAATCCATGGAACCTTCATGCAACAT

TTACCTGAGAAGCATGTTACAATTTAACTTGCTATAAATGCTGGAGCTTTTTTGCTTATTCAGTTTAAGA

ATTCCAAGGCACTGCATCGGATGTTAAAGAATATTATGTAAAGTTTCTTTTGTTCTGCTTGAGGAGGAAG

TATTTTTTCCATGTGTCTAGCTAAAAGACATTGTTGATAGCTCGTACAATAACACATGGTTATTCAATTT

GCATTTCCATTAAGGATTTCTACTGATATGGTTTAATAAATATGATTGAAACTGTTACCCTTTGCCTTTG

GAGTTCATATTTTCCTCTGGATTAGTTAATTATAATTCAGTTATTTGAAGTCTACTTTGAATCTCTCTTT

TGACGTCCTTGATACGCAAGTTTACCCTGTAGACAGAAATGTGGCTCTGCAGAATGACCTTGGATGAGCG

TCTTGTATGTTATTCTGATGACCCTAAACTTGTATAAGTTTCAAGACATACTATTTTGACCAAGGTCTCT

TCTCAATTATATCATTGACCATCTCCTTGTAACTGATCTGTGACTTGGTTTTTCCAATTCAATGATCTAT

CAGGAGTTCTATCCTAAATAATGTTTCTAACATTGATTTCAGATATTTTCTGATGTAGGAGTTTAATATG

TAATGTCCTTTTGTAGTTGCCTCATTACAGAAAATGCTTATGTTATCACCAATTTCCTAGGTGGA

>Astrocaryum_acaule_JQ821976.1

AGCTTTACCTGAGAAGCATGTTGCAATTTAACTTGCTATAAATGAGGGAGCTTTTTTGCTTATTCAGTTT

TWAGAATTCCCAAGGCACTGTATCGGATGTTAAAGAATATTATGTAAAGTTTCTTTTGTTCTGCTCGAGG

AGGAAGTATTTTTTCCATATGTCTAGCTAAAAGACATTGTTGATAGCTCGTACAACAACACATGGTTATT

CATTTTGCATTTCCATTAAGGATTTCTACTGATATGGTTTAATAAATATGATTGAAACTGTTACCCTTTG

CCTTTGGAGTTCATATTTTCCTCTGGATTAGTTAATTATAATTCAGTTATTTGAAGTCTACTTTGAATCT

CTCTTTTGAAGTCCTTGATACGTAAGTTTACCCTGTAGACAGAAATGTGGCTCTGAAGAATGACCTTGGA

TGAGCGTCTTGTATGTTATTCTAATGACCCTAAACTTGTATAAGTTTCAAGACATACTATTTTGACCAAG

GTCTCTTCTCAATTATATCATTGACCATCTCCTTGTAACTGATCTGTGACTTGGTTTTTCCAATTCAATG

ATCTATCAGGAGTTCTATCCCAAATAATGTTTCTAACATTGATTTCAGATATTTTCTGATGTAGGAGTTT

AATATGTAATGTCCTTTTGTAGTTGCCTCATTACAGAAAATGCTTATATTATCACAAATTCCTAGG

>Astrocaryum_mexicanum_EF491154.1

CTACTCCTTTTACTGATGTCACAGTAAGTAAATCCATGGAACCTTCATGCAAGCTTTACCTGAGAAGCAT

GTCACAATTTAACTTGCTATAAATGCTGGAGCTTTTTTGCTTATTCAGTTTAAGAATTCCAAGGCACTGT

ATCGGATGTTAAAGTATATTATGTAAAGTTTCTTTTGTTCTGCTCGAGGAGGAAGTATTTTTTCCATGTG

TCTAGCTAAAAGACATTGTTGATAGCTCGTACAACAACACATGGTTATTCAATTTGCATTTCCATTAAGG

ATTTCTACTGATATGGTTTAATAAATATGATTGAAACTGTTAACCTTTGCCTTTGGAGTTCATATTTTCC

TCTGGATTAGTTAATTATAATTCAGTTATTTGAAGTCTACTTTGAATCTCTCTTTTGAAGTCCTTGATAC

GTAAGTTTACCCTGTAGACAGAAATGTGGCTCTGAAGAATGACCTTGGATGAGCGTCTTGTATGATATTC

TGATGACCCTAAACTTGTATAAGTTTCAAGACATGCTATTTTGACCAAGGTCTCTTCTCAATTATATCAT

TGACCATCTCCTTGTAACTGATCTGTGACTTGGTTTTTCCAATTCAATGATCTATCAGGAGTTCTATCCT

AAATAATGTTTCTAACATTGATTTCAGATATTTTCTGATGTAGGAGTTTAATATGTAATGTCCTTTTGTA

GTTGCCTCAT

>Astrocaryum_huaimi_EF491159.1

CTACTCCTTTTACTGATGTCACAGTAAGTAAATCCATGGAACCTTCATGCAAGCTTTACCTGAGAAGCAT

GTTGCAATTTAACTTGCTATAAATGCTGGAGCTTTTTTGCTTATTCAGTTTAAGAATTCCAAGGCACTGT

ATCGGATGTTAAAGAATATTATGTAAAGTTTCTTTTGTTCTGCTCGAGGAGGAAGTATTTTTTCCATATG

TCTAGCTAAAAGACATTGTTGATAGCTCGTACAACAACACATGGTTATTCATTTTGCATTTCCATTAAGG

ATTTCTACTGATATGGTTTAATAAATATGATTGAAACTGTTACCCTTTGCCTTTGGAGTTCATATTTTCC

TCTGGATTAGTTAATTATAATTCAGTTATTTGAAGTCTACTTTGAATCTCTCTTTTGAAGTCCTTGATAC

GTAAGTATACCCTGTAGACAGAAATGTGGCTCTGAAGAATGACCTTGGATGAGCGTCTTGTATGTTATTC

TAATGACCCTAAACTTGTATAAGTTTCAAGACATACTATTTTGACCAAGGTCTCTTCTCAATTATATCAT

TGACCATCTCCTTGTAACTGATCTGTGACTTGGTTTTTCCAATTCAATGATCTATCAGGAGTTCTATCCC

AAATAATGTTTCTAACATTGATTTCAGATATTTTCTGATGTAGGAGTTTAATATGTAATGTCCTTTTGTA

GTTGCCTCAT

>Astrocaryum_vulgare_HQ265643.1

TCATGGGAAAAGTTGCTGCCCATATGGGAAAGGAGGGAGATGCTACTCCTTTTACTGATGTNCCAGTAAG

TAAATCCATGGAACCTTCANTGCAAGCTTTACCTGAGGAGCATGTTACAATTTAACTTGCNTATAAATGC

NTGGAGCTTTTTTGCTTATTCAGTTTAAGAATTCCAAGGCACTGCATCGGATGTTAAAGAATATTATGTA

AAGTTTCTTTTGTTCTGCTTGAGGAGGAAGTATTTTTTCCATGTGTCTAGCTAAAAGACATTGTTGATAG

CTCGTACAATAACACATGGTTATNCAATTTGCATTTCCATTAAGGATTTCTACTGATATGGTTTAATAAA

TATGATTGAAACTGTTACCCTTTGCCTTTGGAGTTCATATTTTCCTCTGGATTAGTTAATTATAANTCAG

TTATTTGAAGTCTACTTTGAATCTCTCTTTTGACGTCCTTGATACGCAAGTTTACCCTGTAGACAGAAAT

GNGGCTCTGCNGAATGACCTTGGATGAGCGTCTTGTATGTTATTCTGATGACCCTAAACTTGTATAAGTT

TCAAAACATACTATTTTGACCAAGGTCTCTTCTCAATTATATCATTGACCATCTCCTTGTAACTGATCTG

TGACTTGGTTTTTCCAATTCAATGATCTATCAGGAGTTCTATCCTAAATAATGTTTCTAACATTGATTTC

AGATATTTTCTGATGTAGGAGTTTAATATGTAATGTCCTTTTGTAGTTGCCTCATTACAGAAAATGCTTA

TATTATCACCAATTTCCTAGGTGGACAACATCAGCAA

>Astrocaryum_standleyanum_HQ265641.1

GGGAGANGCTACTCCTTTTNCTGATGTCACAGNTAAGTAAATCTATGGAACCTTCATGCAAGCTTTACCT

GAGAAGCATGTTACAATTTAACTTGCTATAAATGCTGGAGCTTTTTTGCTTATTCAGTTTAAGAATTCCA

AGGCACTGCATCGGATGTTAAAGAATATTATGTAAAGTTTCTTTTGTTCTGCTTGAGGAGGAAGTATTTT

TTCCATGTGTCTAGCTAAAAGACATTGTTGATAGCTCGTACAATAACACATGGTTATTCAATTTGCATTT

CCATTAAGGATTTCTACTGATATGGTTTAATAAATATGATTGAAACTGTTACCCTTTGCCTTTGGAGTTC

ATATTTTCCTCTGGATTAGTTAATTATAATTCAGTTATTTGAAGTCTACTTTGAATCTCTCTTTTGACGT

CCTTGATGCGCAAGTTTACCCTGTAGACAGAAATGTGGCTCTGCAGAATGACCTTGGATGAGCATCTTGT

ATGTTATTCTGATGACCCTAAACTTGTATAAGTTTCAAGACATACTATTTTGACCAAGGTCTCTTCTCAA

TTATATCATTGACCATCTCCTTGTAACTGATCTGTGACTTGGTTTTTCCAATTCAATGATCTATCAGGAG

TTCTATCCTAAATAATGTTTCTAACATTGGATTTCAGATATTTTCTGATGTAGGAGTTTAATATGTAATG

TCCNTTTGTAGNTGCCTCATTACAGAAAATGCTTATATTATCACCAATTTCCTAGGNGGACAACATCAGC

AA

>Astrocaryum_rodriguesii_HQ265639.1

GGGAAAGGAGGGAGATGCTACTCCTTTTACNGATGTCCCAGTAAGTAAATCCAGGGACCCTTCATGCAAG

CTTTACCTGAGAAGCATGTTACAATTTAACTTGCTATAAATGCNGGAGCTTTTTTGCTTATTCAGTTTAA

GAATTCCAAGGCACTGCATCGGATGTTAAAGAATATTATGTAAAGTTTCTTTTGTTCTGCTTGAGGAGGA

AGTATTTTTTCCATGTGTCTAGCTAAAAGACATTGTTGATAGCTCGTACAATAACACATGGTTATTCAAT

TTGCATTTCCATTAAGGATTTCTACTGATATGGTTTAATAAATATGATTGAAACTGTTACCCTTTGCCTT

TGGAGTTCATATTTTCCTCTGGATTAGTTAATTATAATTCAGTTATTTGAAGTCTACTTTGAATCTCTCT

TTTGACGTCCTTGATACGCAAGTTTACCCTGTAGACAGAAATGTGGCTCTGCAGAATGACCTTGGATGAG

CGTCTTGTATGTTATTCTGATGACCCTAAACTTGTATAAGTTTCAAGACATACTATTTTGACCAAGGTCT

CTTCTCAATTATATCATTGACCATCTCCTTGTAACTGATCTGTGACTTGGTTTTTCCAATTCAATGATCT

ATCAGGAGTTCTATCCTAAATAATGTTTCTAACATTGATTTCAGATATTTTCTGATGTAGGAGTTTA

>Astrocaryum_murumuru_HQ265637.1

TGCATCATGGGAAAAGTTGCTGCCCATATGGGAAAGGAGGGAGATGCTACTCCTTTTACTGATGTCACAG

TAAGTAAATCCATGGAACCTTCATGCAAGCTTTACCTGAGAAGCATGTTACAATTTAACTTGCTATAAAT

GCTGGAGCTTTTTTGCTTATTCAGTTTAAGAATTCCAAGGCACCGCATCGGATGTTAAAGAATATTATGT

AAAGTTTCTTTTGTTCTGCTTGAGGAGGAAGTATTTTTTTCCATGTGGCTAGCTAAAAGACATTGTTGAT

AGCTCGTACAATAACACATGGTTATTCAATTTGCATATCCATTAAGGATTTCTACTGATATGGTTTAATA

AATATGATTGAAACTGTTACCCTTTGCCTTTGGAGTTCATATTTTCCTCTGGATTAGTTAATTATAATTC

AGTTATTTGAAGTCTACTTTGAATCTCTCTTTTGACGTCCTTGATACGCAAGTTTACCCTGTAGACAGAA

ATGTGGCTCTGCAGAATGACCTTGGATGAGCGTCTTGTATGTTATTCTGATGACCCTAAACTTGTATAAG

TTTCAAGACATACTATTTTGACCAAGGTCTCTTCTCAATTATATCATTGACCATCTCCTTGTAACTGATC

TGTGACTTGGTTTTTCCAATTCAATGATCTATCAGGAGTTCTATCCTAAATAATGTTTCTAACATTGATT

TCAGATATTTTCTGATGTAGGAGTTTAATATGTAATGTCCTTTTGTAGCTGCCTCATTACAGAAAAATGC

TTATATTATCACCAATTTCCTAGGTGGACAACATCAGCAAAGCTCTTCATAAGTGTGATA

>Astrocaryum_mexicanum_HQ265635.1

TCATGGGAAAAGTTGCTGCCCATATGGGAAAGGAGGGAGATGCTACTCCTTTTACTGATGTCACAGTAAG

TAAATCCATGGAACCTTCATGCAAGCTTTACCTGAGAAGCATGTCACAATTTAACTTGCTATAAATGCTG

GAGCTTTTTTGCTTATTCAGTTTAAGAATTCCAAGGCACTGTATCGGATGTTAAAGTATATTATGTAAAG

TTTCTTTTGTTCTGCTCGAGGAGGAAGTATTTTTTCCATGTGTCTAGCTAAAAGACATTGTTGATAGCTC

GTACAACAACACATGGTTATTCAATTTGCATTTCCATTAAGGATTTCTACTGATATGGTTTAATAAATAT

GATTGAAACTGTTAACCTTTGCCTTTGGAGTTCATATTTTCCTCTGGATTAGTTAATTATAATTCAGTTA

TTTGAAGTCTACTTTGAATCTCTCTTTTGAAGTCCTTGATACGTAAGTTTACCCTGTAGACAGAAATGTG

GCTCTGAAGAATGACCTTGGATGAGCGTCTTGTATGATATTCTGATGACCCTAAACTTGTATAAGTTTCA

AGACATGCTATTTTGACCAAGGTCTCTTCTCAATTATATCATTGACCATCTCCTTGTAACTGATCTGTGA

CTTGGTTTTTCCAATTCAATGATCTATCAGGAGTTCTATCCTAAATAATGTTTCTAACATTGATTTCAGA

TATTTTCTGATGTAGGAGTTTAATATGTAATGTCCTTTTGTAGTTGCCTCATTACAGAAAATGCTTATAT

TATCACAAATTCCAGGTGACA

>Astrocaryum_jauari_HQ265633.1

CATCANGGGAAAAGTTGCTGCCCATATGGGAAAGGAGGGAGATGCTACTCCTTTTACTGATGTCACAGTA

AGTAAATCCCATGGAACCTTCATGCAAGCTTTACCTGAGAAGCATGTTGCAATTTAACTTGCGATAAATG

CTGGAGCTTTTTTGCTTATTCAGTTTAAGAATTCCAAGGCACTGTATCGGATGTTACAGAATATTATGTA

AAGTTTCTTTTGTTCTGCTCGAGGAGGAAGTATTTTTTCCATATGTCTAGCTAAAAGACATTGTTGATAG

CTCGTACAACAACACATGGTTATTCATTTTGCATTTCCATTAAGGATTTCTACTGATATGGTTTAATAAA

TATGATTGAAACTGTTACCCTTTGCCTTTGGAGTTCATATTTTCCTCTGGATTAGTTAATTATAATTCAG

TTATTTGAAGTCTACTTTGAATCTCTCTTTTGAAGTCCTTGATACGTAAGTTTACCCTGTAGACAGAAAT

GTGGCTCTGAAGAATGACCTTGGATGAGCGTCTTGTATGTTATTCTAATGACCCTAAACTTGTATAAGTT

TCAAGACATACTATTTTGACCAAGGTCTCTTCTCAATTGTATCATTGACCATCTCCTTGTAACTGATCTG

TGACTTGGTTTTTCCAATTCAATGATCTATCAGGAGTTCTATCCTAAATAATGTTTCTAACATTGATTTC

AGATATTTTCTGATGTAGGAGTTTAMATATGTAATGTCCCTTTGGAAGTTGCCCCCATTACCGAAAAATG

CTTATATTATCACCAAATTTCCTAGGTGGGACAACATCAGCAAAGCTCTTTCATAAG

>Astrocaryum_chambira_HQ265631.1

GGGNAAGGAGGGAGATGCTACTCCTTTTACTGATGTCACAGTAAGTAAATCCATGGAACCTTCATGCAAG

CTTTACCTGAGAAGCATGTTACAATTTAACTTGCTATAAATGCTGGAGCTTTTTTGCTTATTCAGTTTAA

GAATTCCAAGGCACTGTATCGGATGTTAAAGAATATTATGTAAAGTTTCTTTTGTTCTGCTTGAGGAGGA

AGTATTTTTTCCATGTGTCTAGCTAAAAGACATTGTTGATAGCTCGTACAACAACACATGGTTATTCAAT

TTGCATTTCCATTAAGGATTTCTACTGATATGGTTTAATAAATATGATTGAAACTGTTACCCTTTGCCTT

TGGAGTTCATATTTTCCTCTGGATTAGTTAATTATAATTCAGTTATTTGAAGTCTACTTTGAATCTCTCT

TTTGAAGTCCTTGATACGCAAGTTTACCCTGTAGACAGAAATGTGGCTCTGCAGAATGACCTTGGATGAG

CGTCTTGTATGTTATTCTGATGACCCTAAACTTGTATAAGTTTCAAGACATACTATTTTGACCAAGGTCT

CTTCTCAATTATATCATTGACCATCTCCTTGTAACTGATCTGTGACTTGGTTTTTCCAATTCAATGATCT

ATCAGGAGTTCTATCCTAAATAATGTTTCTAACATTGATTTCAGATATTTTCTGATGTAGGAGTTTAATA

TGTAATGTCCTTTTGTAGTTGCCTCATTACAGAAAATGCTTATATTATCACCAATTTCCTAGGTGGACAA

CATCAGCAAAGCTCTTCATAAGTGTGGATA

>Astrocaryum_urostachys_HQ265642.1

GGGAAAGGAGGGAGATGCTACTCCTTTTACTGATGTCACAGTTAAGTAAATCCATGGAACCTTCATGCAA

GCTTTACCTGAGAAGCATGTTACAATTTAACTTGCTATAAATGCTGGAGCTTTTTTGCTTATTCAGTTTA

NGAATTCCAAGGCACTGCATCGGATGTTAAAGAATATTATGTAAAGTTTCTTTTGTTCTGCTTGAGGAGG

AAGTATTTTTTCCATGTGTCTAGCTAAAAGACATTGTTGATAGCTCGTACAATAACACATGGTTATTCAA

TTTGCATTTCCATTAAGGATTTCTACTGATATGGTTTAATAAATATGATTGAAACTGTTACCCTTTGCCT

TTGGAGTTCATATTTTCCTCTGGATTAGTTAATTATAATTCAGTTATTTGAAGTCTACTTTGAATCTCTC

TTTTGACGTCCTTGATACGCAAGTTTACCCTGTAGACAGAAATGTGGCTCTGCAGAATGACCTTGGATGA

GCGTCTTGTATGTTATTCTGATGACCCTAAACTTGTATAAGTTTCAAGACATACTATTTTGACCAAGGTC

TCTTCTCAATTATATCATTGACCATCTCCTTGTAACTGATCTGTGACTTGGTTTTTCCAATTCAATGATC

TATCAGGAGTTCTATCCTAAATAATGTTTCTAACATTGATTTCAGATATTTTCTGATGTAGGAGTTTAAT

ATGTAATGTCCTTTTGTAGNTGCCTCATTACAGAAAATGCTTATATTATCACCAATTTCC

>Astrocaryum_sciophilum_HQ265640.1

GGAAAAGTTGCTGCCCATATGGGAAAGGAGGGAGATGCTACTCCTTTTACTGATGTCACAGTAAGTAAAT

CCATGGAACCTTCATGCAAGCTTTACCTGAGAAGCATGTTACAATTTAACTTGCTATAAATGCTGGAGCT

TTTTTGCTTATTCAGTTTAAGAATTCCAAGGCACTGCATCGGATGTTAAAGAATATTATGTAAAGTTTCT

TTTGTTCTGCTTGAGGAGGAAGTATTTTTTCCATGTGTCTAGCTAAAAGACATTGTTGATAGCTCGTACA

ATAACACATGGTTATTCAATTTGCATTTCCATTAAGGATTTCTACTGATATGGTTTAATAAATATGATTG

AAACTGTTACCCTTTGCCTTTGGAGTTCATATTTTCCTCTGGATTAGTTAATTATAATTCAGTTATTTGA

AGTCTACTTTGAATCTCTCTTTTGACGTCCTTGATACGCAAGTTTACCCTGTAGACAGAAATGTGGCTCT

GCAGAATGACCTTGGATGAGCGTCTTGTATGTTATTCTGATGACCCTAAACTTGTATAAGTTTCAAGACA

TACTATTTTGACCAAGGTCTCTTCTCAATTATATCATTGACCATCTCCTTGTAACTGATCTGTGACTTGG

TTTTTCCAATTCAATGATCTATCAGGAGTTCTATCCTAAATAATGKTTCTACCATTGATTTTAGATATTT

TCTGATGTAGGAGTTTAMATATGTAATGTCCTTTTGTAGGTGCCTCATTACAGAAAAATGCTTATATTAT

CACCAATTTCC

>Astrocaryum_paramaca_HQ265638.1

GCATCATGGGAAAAGTTGCTGCCCATATGGGGAAGGAGGGAGATGCTACTCCTTTTACTGATGTCACAGT

AAGTAAAATCCATGGRACCTTCATGCAAGCTTTACCTTGAGAAGCATGTTACAATTTAACTTGCTATAAA

TGCTGGAGCTTTTTTGCTTATTCAGTTTAAGAATTCCAAGGCACTGTATCGGATGTTAAAGAATATTATG

TAAAGTTTCTTTTGTTCTGCTCGAGGAGGAAGTATTTTTTCCATGTGTCTAGCTAAAAGACATTGTTGAT

AGCTTGTACAACAACACATGGTTATTCAAGTTGCATTTCCATTAAGGATTTCTACTGATATGGTTTAATA

AATATGATTGAAACTGTTACCCTTTGCCTTTGGAGTTCATATTTTCCTCTGGATTAGTTAATTATAATTC

AGTTATTTGAAGTCTACTTTGAATCTCTCTTTTGAAGTCCTTGGTACGCAAGTTTACCCTGTAGACAGAA

ATGTGGCTCTGCAGAATGACCTTGGATGAGCGTCTTGTATGTTATTCTGATGACCCTAAACTTGTATAAG

TTTCAAGACATACTATTTTGACCAAGGTCTCTTCTCAATTATATCATTGACCATCTCCTTGTAACTGATC

TGTGACTTGGTTTTTCCAATTCAATGATCTATCAGGAGTTCTATCCTAAATAATGTTTCTAACATTGATT

TCAGATATTTTCTGATGTAGGAGTTTAATATGTAATGTCCTTTTGTAGTTGCCTCATTACAGAAAATGCT

TATATTATCACCAATTCCTAGGTGGACAACATCAGCAAAGCTCTTCATAAGTGTGG

>Astrocaryum_minus_HQ265636.1

CCCCGGWAGGAAATYCMAGGGACCCTCCAGCCAGCCTTACCTGAGAAGCATGTTACAATTTAWCTTGCTA

TAAATGCWGGAGCGTTTTTTGCTTACTTCAGTTTAAGAATTCCAAGGCACCGCATCGGATGTTAAAGAAT

ATTATGTAAAGTTTCTTTTGTTCTGCTTGAGGAGAAAGTATTTTTTCCATGTGGCTAGCTAAAAGACATT

GTTGATAGCTCGTACAATAACACATGGTTATTCAATTTGCATATCCATTAAGGATTTCTACTGATATGGT

TTAATAAATATGATTGAAACTGTTACCCTTTGCCTTTGGAGTTCATATTTTCCTCTGGATTAGTTAATTA

TAATTCAGTTATTTGAAGTCTACTTYGAATCTCTCTTTTGACGTCCTTGATACGCAAGTTTACCCTGTAG

ACAGAAATGTGGCTCTGCAGAATGACCTTGGATGAGCGTCTTGTATGTTATTCTGATGACCCTAAACTTG

TATAAGTTTCAAGACATACTATTTTGACCAAGGTCTCTTCTCAATTATATCATTGACCATCTCCTTGTAA

CTGATCTGTGACTTGGTTTTTCCAATTCAATGATCTATCAGGAGTTCTATCCTAAATAATGTTTCTAACA

TTGATTTCAGATATTTTCTGATGTAGGAGTTTAMTACTGTAATGTCCTCTTGTAGGTGCCTCATTACMGA

AAATGCTTATATMTCACCAATTCC

>Astrocaryum_malybo_HQ265634.1

CGAANTCGCCCTTCNACTTATTGAGTGCATCATGGGAAAAGTTGCTGCCCATATGGGAAAGGAGGGAGAT

GCTACTCCTTTTACTGATGTCACAGTAAGTAAATCCATGGAACCTTCATGCAAGCTTTACCTGAGAAGCA

TGTTACAATTTAACTTGCTATAAATGCTGGAGCTTTTTTGCTTATTCAGTTTAAGAATTCCAAGGCACTG

CATCGGATGTTAAAGAATATTATGTAAAGTTTCTTTTGGTTCTGCTTGAGGAGGAAGTATTTTTTCCATG

TGTCTAGCTAAAAGACATTGTTGATAGCTCGTACAATAACACATGGTTATTCAATTTGCATTTCCATTAA

GGATTTCTACTGATATGGTTTAATAAATATGATTGAAACTGTTACCCTTTGCCTTTGGAGTTCATATTTT

CCTCTGGATTAGTTAATTATAATTCAGTTATTTGAAGTCTACTTTGAATCTCTCTTTTGACGTCCTTGAT

ACGCAAGTTTACCCTGTAGACAGAAATGTGGCTCTGCAGAATGACCTTGGATGAGCGTCTTGTATGTTAT

TCTGATGACCCTAAACTTGTATAAGTTTCAAGACATACTATTTTGACCAAGGTCTCTTCTCAATTATATC

ATTGACCATCTCCTTGTAACTGATCTGTGACTTGGTTTTTCCAATTCAATGATCTATCAGGAGTTCTATC

CTAAATAATGTTTCTAACATTGATTTCAGATATTTTCTGATGTAGGAGTTTAATATGTAATGTCCTTTTG

TAGTTGCCTCATTACAGAAAATGCTTATATTATCACCAATTTCCTAGGTGGACAACATCAGCAAAGCTCT

TCAT

>Astrocaryum_gynacanthum_HQ265632.1

GCATCANGGGAAAAGTTGCTGCCCATATGGGGAAGGAGGGAGATGCTACTCCTTTTACTGATGTCACAGT

AAGTAAATCCATGGAACCTTCATGCAAGCTTTACCTGAGAAGCATGTTACAATTTAACTTGCTATAAATG

CTGGAGCTTTTTTGCTTATTCAGTTTAAGAATTCCAAGGCACTGCATCGGATGTTAAAGAATATTATGTA

AAGTTTCTTTTGTTCTGCTTGAGGAGGAAGTATTTTTTCCATGTGTCTAGCTAAAAGACATTGTTGATAG

CTCGTACAATAACACATGGTTATTCAATTTGCATTTCCATTAAGGATTTCTACTGATATGGTTTAATAAA

TATGATTGAAACTGTTACCCTTTGCCTTTGGAGTTCATATTTTCCTCTGGATTAGTTAATTATAATTCAG

TTATTTGAAGTCTACTTTGAATCTCTCTTTTGACGTCCTTGATACGCAAGTTTACCCTGTAGACAGAAAT

GTGGCTCTGCAGAATGACCTTGGATGAGCGTCTTGTATGTTATTCTGATGACCCTAAACTTGTATAAGTT

TCAAGACATACTATTTTGACCAAGGTCTCTTCTCAATTATATCATTGACCATCTCCTTGTAACTGATCTG

TGACTTGGTTTTTCCAATTCAATGATCTATCAGGAGTTCTATCCTAAATAATGTTTCTAACATTGATTTC

AGATATTTTCTGATGTAGGAGTTTAATATGTAATGTCCTTTTGTAGTTGCCTCATTACAGAAAAATGCTT

ATATTATCACCAATTTCCTAGGTGGACAACATCAGCAAAGCTCTTCATAAGTGTGGATA

>Astrocaryum_alatum_HQ265630.1

CAACTTTTTGAGTGCATCANGGGAAAAGTTGCNGCCCATATGGGAAAGGAGGGAGATGCTACTCCTTTTA

CTGATGTCACAGTTAAGTAAATCCCATGGAACCTTCATGCAAGCTTTACCTGAGAAGCATGTCACAATTT

AACTTGCTATAAATGCTGGAGCTTTTTTGCTTATTCAGTTTAAGAATTCCAAGGCACTGTATCGGATGTT

AAAGAATATTATGTAAAGTTTCTTTTGTTCTGCTCGAGGAGGAAGTATTTTTTCCATGTGTCTAGCTAAA

AGACATTGTTGATAGCTCGTACAACAACACATGGTTATTCAATTTGCATTTCCATTAAGGATTTCTACTG

ATATGGTTTAATAAATATGATTGAAACTGTTACCCTTTGCCTTTGGAGTTCATATTTTCCTCTGGATTAG

TTAATTACAATTCAGTTATTTGAAGTCTACTTTGAATCTCTCTTTTGAAGTCCTTGATACGTAAGTTTAC

CCTGTAGACAGAAATGTGGCTCTGAAGAATGACCTTGGATGAGTGTCTTGTATGTTATTCTGATGACCCT

AAACTTGTATAAGTTTCAAGACATGCTATTTTGACCAAGGTCTCTTCTCAATTATATCATTGACCATCTC

CTTGTAACTGATCTGTGACTTGGTTTTTCCAATTCAATGATCTATCAGGAGTTCTATCCTAAATAATGTT

TCTAACATTGATTTCAGATATTTTCTGATGTAGGAGTTTAATATGTAATGTCCTTTTGTAGTTGCCTCAT

TACAGAAAATGCTTATATTATCACAAATTCCAGGGGACA

>Attalea_phalerata_HQ265644.1

GGGAGGAAGTAATTTTTGCATGTCTGCCTAAAAAGACATTGTTGTTAGCTCATACAAAAATGCAGGGTTA

TTCAATTTGCATTACCATTAAGGATTTGTAACTAGATGTGGTTTAAATTAATATGTTTGAGTTGCATTAC

CATTAAGGATTTGTACTAGATATGGTTTAATAAATATGGTTGAGTTGCATTACCCAAGATGCGGGGGGGG

GGGGGGGTAAANCCCTTCAACTTCATACCTGAAATTTTTTTTTTCCTNTGGGGTNGNGGGGGGTGGGGTG

TGGCTTTTGCCTNTGGAGTTCATATTNTCCTGTGGATTAGTTAATTATAATCCTGTTATNTGAAGTCTAC

TGNGAATATCTCTTTTGAAGTTGGTGATATGNNAGTNTACCCNGNAGACAGANACATGGCTCTGAAGAAT

GACCTTAGACGAGTGTCTTGTAAGTTATTCTGATGATCCTAAACTTGTANAAGTTTCAAGACATACT

>Attalea_allenii_AJ830207.1

GTGAGCACTTGCCTTTAGAGTTCATATTTTCCTGTTGATTAGTTAATTATAATCCTGTTATTTGAAGTCT

ACTGTGAATATCTCTTTTGAAGTTGGTGATATGTTAGTTTACCCTGTAGACAGAAACATGGCTCTGAAGA

ATGACCTTAGACGAGTGTCTTGTAAGTTATTCTGATGATCCTAAACTTGTATAAGTTTCAAGACATACTA

TTTTGACTAAGGTCTCTCCTAAATTATGTCATTGACTATCTCCTTGTGCCTGACCTGTGGCTTGGATTTT

CCAATTCAATGATCTATCGGGAGTTCTATCCTAAATAATGTTTCTAACATTGAATTCAGATATATTCTGA

TGTAGGGGTTTAATATGTAATTGTACTTTTGTAGTTGCTCCATTACAAAAATGCTTATATTATCACAAAT

TTCCTAGGTGGACAATATCAGCAAAGCTCTTCATAAGTGTGG

>Bactris_riparia_HQ265655.1

ATTCGCCCTTCAACTTATTGAGTGCATCATGGGAAAAGTTGTTGCCCATATGGGAAAGGAGGGAGATGCT

ACTCCTTTTACTGATGTCACAGTAAGTAAATCCATGGAACCTTCATGCAAGCTTTACCTGAGAAGCATGT

CACAATTTAACTTGCTATAAATGCTGGAGCTTTTTTGCTTATTCAGTTTAAGAATTCCAAGGCACTGTAT

CGGATGTTAAAGAATATTATGTAAAGTTTCTTTTGTTCTGCTCAAAGAGGAAGTATTTTTGCCATGTGTC

TAGCTAAAAGACATCGTTGATAGCTCGTACAACAGCACATGGTTATTCAATTTGCATTTCCATTAAGGAT

TTCTACTGACAAGGTTTAATAAATATGATTGAAACTGTTACCCTTTGCCTGTGGAGTTCATATTTTCCTC

CGGATTAGTTAATTATAATTCAGTTATTTGAAGTCTACTTTGAATCTCTCTTTTGAAGTCCTTGATACGT

AAGTTTACCCTGTAGACAGAAATGTCAAATGTGGCTCTGAACAATGACCTTGGACTAGAGCGTCTTGTAT

GTTATTCTGATGACCGTAAACTTGTATAAGTTTCAAGACATACTATTTTGACCAAAGGTCTCTTCTCAAT

TATATCATTTTGACCATCTCCTTGTTAACTGATCTGTGACTTGGTTTTTCCAATTCAATGATCTATCAGG

AGTTCTATCCTGAATAATGTTTCTAACATGATTTCGGATATTTTCTGATGTAGGAGTTTAATATGTAATG

TCCTTTTGTAGTTGCCTCATTACAGAAAATGCTTATGTTATCGCAAATTTCCTAGGTGGACAATATCAGC

AAAGCTCTTCATAAGTGTGGATATCAGATGCGTGGAAGGGCGAATTCG

>Bactris_maraja_HQ265653.1

ACTTATTGAGTGCATCATGGGAAAAGTTGCTGCCCATATGGGAAAGGAGGGAGATGCTACTCCTTTTACT

GATGTCACAGTAAGTAAATCCATGGAACCTTCATGCAAGCTTTACCTGAGAAGCATGTCACAATTTAACT

TGCTATAAATGTTGGAGCTTTTTTGCTTATTCAGTTTAAGAATTCCAAGGCACTGTATCGGATGTTAAAG

AATATTATGTAAAGTTTCTTTTGTTCTGCTCAAGGAGGAAGTATTTTTGCCATGTGTCTAGTTAAAAGAC

ATCGTTGATAGCTCGTACAACAGCACATGGTTATTCAATTTGCATTTCCATTAAGGATTTCTACTGGCAA

GGTTTAATAAATATGATTGAAACTGTTACCCTTTGCCTGTGGAGTTCATATTTTCCTCTGGATTAGTTAA

TTATAATTCAGTTATTTGAAGTCTACTTTGAATCTCTCTTTTGAAGTCCTTGATACTTAAGTTTACCCTG

TAGACAGAAATGTGGCTCTGAAGAATGACCTTGGACGAGAGCGTCTTGTATGTTATTCTGATGACCCTAA

ACTTGTTTTAAGTTTCAAGACATACTATTTTGACCAAAGGTCTCTTCTCAATTATATCACTTTGACCATC

TCCTTGTTAACTGATCTGTGACTTGGTTTTTCCAATTCAATGATCTATCAGGAGTTCTATCCTAAATAAT

GTTTCTAACATGATTTCAGATATCTTCTGATGTAGGAGTTTAATATGTAATGCCCTTTKGTAGTTGCCTC

ATTACAGAAAATGCTTATGTTATCACAAATTTCCTAGGTGGACAATATCAGCAAAGCTCTTCATAAGTGT

GGATATCAGA

>Bactris_killipii_HQ265651.1

AGCTTTACCTGAGAAGCATGTCACAATTTAACTTGCTATAAATGCTGGAGCTTTTTGGCTTATTCAGTTT

AAGAATTCCAAGGCACTGTATCGGATGTTAAAGAANATTATGTAAAGTTTCTTTTGTTCTGCTCAAGGAG

GAAGTATTTTTGCCATGTGTCTAGCTAAAAGACATCGTTGATAGCTCGTACAACAGCACATGGKTATTCA

ATTTGCATTTCCATTAAGGATTTCTACTGACAAGGTTTAATAAATATGATTGAAACTGTTACCCTTTGCC

TGTGGAGTTCATATTTTCCTCTGGATTAGTTAATTATAATTCAGTTATTTGAAGTCTACTTTGAATCTCT

CTTTTGAAGTCCTTGATACTTAAGTTTACCCCGTAGACAGAAATGTGGCTCTGAAGAATGACCTTGGACG

AGAGCGTCTTGTATGTTATTCTGATGACCCTAAACTTGTTTAAGTTTCAAGACATACTATTTTGACCAAA

GGTCTCTTCTCAATTATATCATTTTGACCATCTCCTTGTNAACTGATCTGTGACTTGGTTTTTCCAATTC

AATGATCTATCAGGAGTTCTATCCTAAATAATGTGTCNAACATGATTTCAGATATCTTCCGATGTAGCAG

TGNTAATATGTAATGTCCTTTTGTAGTCGCCTCATTNCAGAAAANGCTTAGGTATCACAAATTCCTAGGG

ACA

>Bactris_ferruginea_HQ265649.1

GGGAAAAGTTGCTGCCCATATGGGAAAGGAGGGAGATGCTACTCCTTTTACTGATGTCACAGTAAGTAAA

TCCATGGAACCTTCATGCAAGCTTTACCTGAGAAGCATGTCACAATTTAACTTGCTATAAATGCTGGAGC

TTTTTTGCTTATTCAGTTTAAGAATTCCAAGGCACTGTATCGGATGTTAAAGAATATTATGTAAAGTTTC

TTTTGTTCTGCTCAAGGAGGAAGTATTTTTGCCATGTGTCTAGCTAAAAGACATCGTTGATAGCTCGTAC

AACAGCACATGGTTATTCAATTTGCATTTCCGTTAAGGATTTCTGCTGACAAGGTTTAATAAATATGATT

GAAACTGTTACCCTTTGCCTGTGGAGTTCATATTTTCCTCTGGATAAGTTAATTATAATTCAGTTATTTG

AAGTCTACTTTGAATCTCTCTTTTGAAGTCCTNGATACTNAAGTTTACCCTGTAGACAGAAATGNGGCTC

TGAAGAATGNCCTNGGACGAGAGCATCTTGTATGTTATTCTGATGACCCTAAACTTGTNTAAGTTTCAAG

ACATACTATTTTGACCAAAGGTCTCTTCTCAATTATATCANTTTGACCATCTCCNTGTTAACTGATNTGT

GACNTGGTTTTTCCAATTCAATGATCTATCAGGAGTTCTATCCTAAATAATGTTTCTAACA

>Bactris_campestris_HQ265647.1

ATGGGAAAAGTTGCTGCCCATATGGGAAAGGAGGGAGATGCTACTCCTTTTACTGATGTCACAGTAAGTA

AATCCCATGGAACCTTCATGCAAGCTTTACCTGAGAAGCATGTCACAATTTAACTTGCTATAAATGCTGG

ACCTTTTTTGCTTATTCAGTTTAAGAATTCCAAGGCACTGTATCGGATGTTAAAGAATATTATTGTAAAG

TTTCTATTGTTCTGCTCAAGGAGGAAGTATTTTTGCCATGTGTCTAGCTAAAAGACATCGTTGATAGCTC

GTACAACAGCACATGGTAATTCAATTTGCATTTCCGTTAAGGATATCTACTGACAAGGTTTAATAAATAT

GATTGAAACTGTTACCCTTTGCCTGTGGAGTTCATATTTTCCTCTGGATTAGTTAATTATAATTCAGTTA

TTTGAAGTCTACTTTGAATCTCTCTTTTGAAGTCCTTGATACTTAAGTTTACCCTGTAGACAGAAATGTG

GCTCTGAAGAATGACCTTGGACGAGAGCGTCTTGTATGTTATTCTGATGACCCTAAACTTGTTTAAGTTT

CAGGACATACTATTTTGCCCAAAGGTCTCTTCTCAATTATATCATTTTGACCATCTCCTTGTTAACTGAT

CTGTGACTTGGTTTTTCCAATTCAATGATCTATCAGGAGTTCTATCCTAAATAATATTTCTAACATGATT

TCCAGATATCTTCCTGATGTA

>Bactris_acanthocarpa_HQ265645.1

CGAATTCGCCCTTCNACTTATTGAGTGCATCATGGGAAAAGTTGCTGCCCATATGGGAAAGGAGGGAGAT

GCTACTCCTTTTACTGATGTCACAGTAAGTAAATCCATGGAACCTTCATGCAAGCTTTACCTGAGAAGCA

TGTCACAATTTAACTTGCCATAATGCTGGAGCTTTTTTGCTTATTCAGTTTAAGAATTCCAAGGCACTGT

ATCGGATGTTGAAGAATATTATGTAAAGTTTCTTTTGTTCTGCTCAAGGGGGAAGTATTTTTGCCATGTG

TCTAGTTAAAAGACATCATTGATAGCTCGTACAACAGCACATGGTTATTCAATTTGCATTTCCATTAAGG

ATTTCTACTGACAAGGTTTAATAAATATGATTGAAACTGTTACCCTTTGCCTGTGGAGTTCATATTTTCC

TCTGGATTAGTTAATTATAATTCAGTTATTTGAAGTCTACTTTGAATCTCTCTTTTGAAGTCCTTGATAC

TTAAGTTTACCCTGTAGACAGAAATGTGGCTCTGAAGAATGACCTTGGACGAGAGCGTCTTGTATGTTAT

TCTGATGACCCTAAACTTGTTTAAGTTTCAAGACATACTATTTTGACCAAGGGTCTCTTCTCAATTATAT

CATTTTGACCATCTCCTTGTTAACTGATCTGTGACTTGGTTTTTCCAATTCAATGATCTATCAGGAGTTC

TATCCTAAATAATGTTTCTAACATGATTTCAGATATCTTCTGATGCAGGAGTTTAATATGTAATGTCCTT

TTGTAGTTGCCTCATTACAGAAAATGCTTATGTTATCACAAATTTCCTAGGTGGACAATATCAGCAAAGC

TCTTCATAAGTGTGGATATCAGATGCGTGGAAGGGGCGAATTCG

>Bactris_pliniana_HQ265654.1

GTTGCGGCCCATATGGGAAAGGAGGGAGATGCTACTCCTTTTACTGATGTCACAGTAAGTAAATCCATGG

AACCTTCATGCAAGCTTTACCTGAGAAGCATGTCACAATTTAACTTGCTATAAATGCTGGAGCTTTTTTG

CTTATTCAGTTTAAGAATTCCAAGGCACTGTATCGGATGTTAAAGAATATTATGTAAAGTTTCTATTGTT

CTGCTCAAGGAGGAAGTATTTTTGCCATGTGTCTAGCTAAAAGACATCGTTGATAGCTCGTACAACAGCA

CATGGTAATTCAATTTGCATTTCCATTAAGGATATCTACTGACAAGGTTTAATAAATATGATTGAAACTG

TTACCCTTTGCCTGTGGAGTTCATATTTTCCTCTGGATTAGTTAATTATAATTCAGTTATTTGAAGTCTA

CTTTGAATCTCTCTTTTGAAGTCCTTGATACTTAAGTTTACCCTGTAGACATAAATGTGGCTCTGAAGAA

TGACCTTGGACGAGAGCGTCTTGTATGTTATTCTGATGACCCTAAACTTGTTTAAGTTTCAAGACATACT

ATTTTGCCCAAAGGTCTCTTCTCAATTATATCATTTTGACCATCTCCTTGTTAACTGATCTGTGACTTGG

TTTTTCCCAATTCAATGATCTATTAGGAGTTCTATCCTAAATAATATTTCTAACATGATTTCAGATATCT

TCTGATGTAGGAGTTTAATATGTAATGTCCTTTNGTAGTTGCCTCATTACAGAAAATGCTTATGTNTCAC

AAATT

>Bactris_major_HQ265652.1

GGGAGATGCTACTCCTTTTACTGATGTCACAGTTAAGATAAATCCATGGAACCTTCATGCAAGCTTTACC

TGAGAAGCATGTCACAATTTAACTTGCTATAAATGCTGGAGCTTTTTTGCTTATTCAGTTTAAGAATTCC

AAGGCACTGTATCAGATGTTAAAGAATATTATGTAAAGTTTCTTTTGTTCTGCTCAAGGAGGAAGTATTT

TTGCCATGTGTCTAGTTAAAAGACATCGTTGATGGCTCGTACAACAGCACATGGTTATTCAATTTGCATT

TCCATTAAGGATTTCTACTGACAAGGTTTAATAAATATGATTGAAACTGTTACCCTTTGCCTGTGGAGTT

CATATTTTCCTCTGGATTAGTTAATTATAATTCAGTTATTTGAAGTCTACTTTGAATCTCTCTTTTGAAG

TCCTTGATACTTAAGTTTACCCTGTAGACAGAAATGTGGCTCTGAAGAATAACCTTGGACGAGAGCGTCT

TGTATGTTATTCTGATGACCCTAAACTTGTTTAAGTTTCAAGACATACTATTTTGACCGAAGGTCTCTTC

TCAATTATATCATTTTGACCATCTCCTTGTTAACTGATCTGTGACTTGGTTTTTCCAATTCAATGATCTA

TCAGGAGTTCTATCCTAAATAATGTTTCTAACATGATTTCAGATATCTTCTGATGTAGGAGTTTAATATG

TAATGTCCTTTTGTAGTTGCCTCATTACAGAAAAATGCTTATGTTATCACAAATTTCNTAGGTGGACAAT

ATCAGCAANGCTCTTCATAAGTGGGA

>Bactris_gasipaes_HQ265650.1

AACTTGCTATAAATGCTGGAGCTTTTTTGCTTATTCAGTTTAAGAATTCCTAAGGCACTGTATCGGATGT

TAAAGAATATTATGTAAAGCTTTCTTTTGTTCTGCTCAANGAGGAAGTATTTTTGCCATGTGTCTAGCTA

AAAGACATCGTTGATAGCTCGTACAACAGCACATGGTTATTCAATTTGCATTTCCATTAAGGATTTCTAC

TGACAAGGTTTAATAAATATGATTGAAACTGTTACCCTTTGCCTGTGGAGTTCATATTTTCCTCCGGATT

AGTTAATTATAATTCAGTTATTTGAAGTCTACTTTGAATCTCTCTTTTGAAGTCCTTGATACTTAAGTTT

ACCCTGTAGACAGAAATGTGGCATGTGAACTAATGACCTATGGACCGAGAGCSTCTTGYATGTTATTCTG

ATGACCNGAAACTTGTATAAGTTTCAAGACATACTATTTTGCCCAAAGGTCTCTTCTCAATTATATCATT

TTGACCATCTCCTTGTTAACTGATCTGTGACTTGGTTTTTCCAATTCAATGATCTATCAGGAGTTCTATC

CTGAATAATGTTTCTAACATGATTTCGGNTATCTTCTGATGTAGGAGTTTAATATGTAATGTCCTTTNGT

AGTNGCCTCANTACAGAAAATGC

>Bactris_constanciae_HQ265648.1

CCCATATGGGAANGGAGGGAGATGCTACTCCTTTTACTGATGTCACAGGTAAGTAAATCCATGGAACCTT

CATGCAAGCTTTACCTGAGAAACATGTCACAATTTAACTTGCTATAAATGCTGGAGCTTTTTTGCTTATT

CAGTTTAAGAATTCCAAGGCACTGTATCGGATGTTAAAGAATATTATGTAAAGTTTCTTTTGTTCTGCTC

AAGGAGGAAGTATTTTTTGCCATGTGTCTAGCTAAAAGACACCGTTGATAGCTCGTACAACAGCACATGG

TTATTCAATTTGCATTTCCATTAAGGATTTCTACTGACAAGGTTTAATAATTATGATTGAAACTGTTACC

CTTTGCCTGTGGAGTTCATATTTTCCTTTGGATTAGTTAATTATAATTCAGTTATTTGAAGTCTACTTTG

AATCTCTCTTTTGAAGTCCTTGATACTTAAGTTTACCCTGTAGACAGAAATGTGGGTCTGAAGAATGACC

TTGGATGAGAGCGTCTTGTATGTTATTCTGATGACCCTAAACTTGTTTAGGTTTCAAGACATACTATTTT

GACCAAAGGTCTCTTCTCAATTATATCATTTTGACCATATCCTTGTTAACTGATCTGTGACTTGGTTTTT

CCAATTCAATGATCTATCAGGAGTTCTATCCYAAATAATGTTTCTAACATGATTTCAGATATCTTCTGAT

GTAGGAGTTTAATATGTAATGTCCTTTTGTAGTTGCCTCATTACAGAAAATGCTTATGTATCACAAATT

>Bactris_bifida_HQ265646.1

GGGAAAAGTTGCTGCCCATATGGGAAAGGAGGGAGATGCTACTCCTTTTACTGATGTCACAGATAAGTAA

ATCCATGGAACCTTCATGCAAGCTTTACCTGAGAAGCATGTCACAATTTAACTTGCTATAAATGCTGGAG

CTTTTTTGCTTATTCAGTTTAAGAATTCCAAGGCACTGTATCGGATGTTAAAGAATATTATGTAAAGTTT

CTTTTGTTCTGCTCAAGGAGGAAGTATTTTTGCCATGTGTCTAGTTAAAAGACATCGTTGATGGCTCGTA

CAACAGCACATGGTTATTCAATTTGCATTTCCATTAAGGATTTCTACTGACAAGGTTTAATAAATATGAT

TGAAACTGTTACCCTTTGCCTGTGGAGTTCATATTTTCCTCTGGATTAGTTAATTATAATTCAGTTATTT

GAAGTCTACTTTGAATCTCTCTTTTGAAGTCCTTGATACTTAAGTTTACCCTGTAGACAGAAATGTGGCT

CTGAAGAATGACCTTGGACGAGAGCGTCTTGTATGTTATTCTGATGACCCTAAACTTGTTTAAGTTTCAA

GACATACTATTTTGACCGAAGGTCTCTTCTCAATTATATCATTTTGACCATCTCCTTGTTAACTGATCTG

TGACTTGGTTTTTCCAATTCAATGATCTATCAGGAGTTCTATCCTAAATAATGTTTCTAACATGATTTCA

GATATCTTCTGATGTAGGAGTTTAATATGTAATGTCCTTTTGTAGTTGCCTCATTACAGAAAATGCTTAT

GTTATCACAAATTTCCTAGG

>Barcella_odora_EF491158.1

CTACTCCTTTTACTGATGTCACAGTGAGTAAATCTATAGAACCTTCATGCAAGCTTTACCTGAGAAGCAT

GTCACAATTTAACTTGCTATAAATGCTGGAGCTTTTTTGCTTATTCAGTTTAAGAATTCCAAGGTACTGT

ATCTGATGTTAAAAAACATTATGTAAAGTTTCTTCTGTTCTGCTCGAGGAGGAAGTATTTTTTTCCGTAT

GCCTAGCTAAAAGACATTGTTGATAGCTCGTACATAAACACATGGTTATTCAATTTGCATTACCATTAAG

GATTTGTACTAATATGGTTTAATAAATATGATGGAGAACTGTTATCCTTTGCCTTTGGAGTTCATATTTT

CCTCTGGATTAGTTAATTATAATTCGGTTATTTGAAGTCTACTTTGATTCTCTCTTTTGAAGTTCTTGAT

ATGTAAATTTACCCTGTAGACAGAAACGTGGCTCTGAAGGATGACCTTGGACGAGTGTCTTGCATGTTAT

TCTGATGATCCTAAACTTGTATAAGTTTCAAGATGTGACTAAGGTCTCTCCTAAATTATATCATTGACTA

TCTCCTTGTAACTGACCTGTGACTTAGTTTTTCCAATTCAATGATCTATCAGGAGTTCTATCCTAAATAA

TGTTTCTAACATTGATTTCAGATATTTTCTGATGTAGGAGTTTAATATGTAATGTACTTTGGTAGTTGTC

TCAT

>Barcella_odora_JQ821998.1

GGAGATGCTACTCCTTTTACTGATGTCACAGTGAGTAAATCTATAGAACCTTCATGCAAGCTTTACCTGA

GAAGCAATGTCACAAATTTAACTTGCTATAAATGCTGGAGCTTTTTTGCTTATTCAGTTTAAGAATTCCA

AGGTACTGTATCTGATGTTAAAAAACATTATGTAAAGTTTCTTCTGTTCTGCTCGAGGAGGAAGTATTTT

TTTCCGTATGCCTAGCTAAAAGACATTGTTGATAGCTCGTACATAAACACATGGTTATTCAATTTGCATT

ACCATTAAGGATTTGTACTAATATGGTTTAATAAATATGATGGAGAACTGTTATCCTTTGCCTTTGGAGT

TCATATTTTCCTCTGGATTAGTTAATTATAATTCGGTTATTTGAAGTCTACTTTGATTCTCTCTTTTGAA

GTTCTTGATATGTAAATTTACCCTGTAGACAGAAACGTGGCTCTGAAGGATGACCTTGGACGAGTGTCTT

GCATGTTATTCTGATGATCCTAAACTTGTATAAGTTTCAAGATGTGACTAAGGTCTCTCCTAAATTATAT

CATTGACTATCTCCTTGTAACTGACCTGTGACTTAGTTTTTCCAATTCAATGATCTATCAGGAGTTCTAT

CCTAAATAATGTTTCTAACATTGATTTCAGATATTTTCTGATGTAGGAGTTTAATATGTAATGTACTTTT

GTAGTTGTCTCATTACAAAAAAAAATGCTTATATTATCACAAATTTCCTAGGTGGA

>Brahea_dulcis_HQ720493.1

TCCTTTTACTGATGTCACAGTAAGTAAATACATAAAACCTCCATGGAAGCTTTACCTGAGAAGCGTGTCA

TAATTTAACCTGCTATAAATGCTGGAGCTTTTTTCCTTATTCAGTTTAAGAATTTTGAGTCACTATATCT

GATGTTAAAGAACATTAAGGAAAGTTTCTTTTGTTCTGCTCCAGGAGGAAGTAATTTTTGCATATGTCTA

GCTAAAAGACATTGTTGATAGCTTATGCAAAAACAGAAGGTTATTCAATTTTCATTACCATTAAGGATTT

GTATTAGATATGGTTTAGTTAATTATAATCCTTTCAACTTCATACCTGGATTTGATTTTCTTTTGAGGGG

AGCTTTTGCCTTTGGAGTTCATATTTTCCTCTGGTTTAGTTAATTATAATCATGTTATTTGAAGTCTACT

TTGAATATCTCTTTTGAAGTTCTTGAGATGTAAGTTTACCCTGTAGACAGAAACGTGGCTTTGGAGAATG

ACTTTGGATGAGTGTAAGTTATTCTGATGATCCTAAACTTGTATAAGTTTCAAGACATACTATTTTGACT

AAGGTCTCTTCTAAATTATATAGTAGACTATCTCTTTGTAACTGGCCTGTGACTTRGATTTTCCAATTCG

ATGATCTATCAGGAGTTCTATCCTAAATAATGTTTCTAACATTGATTTCAGATGCATTCTGATATAGGAG

TTCAATATGTAATTGTTCTTTTGTAATTTCCTCATTACAAAAAATACTTATATTATC

>Brahea_dulcis_HQ720491.1

AATGCTGGAGCTTTTTTCCTTATTCAGTTTAAGAATTTTGAGTCACTATATCTGATGTTAAAGAACATTA

AGGAAAGTTTCTTTTGTTCTGCTCCAGGAGGAAGTAATTTTTGCATATGTCTAGCTAAAAGACATTGTTG

ATAGCTTATGCAAAAACAGAAGGTTATTCAATTTTCATTACCATTAAGGATTTGTATTAGATATGGTTTA

TAATCCTTTCAACTTCATACCTGGATTTGATTTTCTTTTGAGGGGAGCTTTTGCCTTTGGAGTTCATATT

TTCCTCTGGTTTAGTTAATTATAATCATGTTATTTGAAGTCTACTTTGAATATCTCTTTTGAAGTTCTTG

AGATGTAAGTTTACCCTGTAGACMGAAACGTGGCTTTGGAGAATGACTTTGGATGAGTGTAAGTTATTCT

GATGATCCTAAACTTGTATAAGTTTCAAGACATACTATTTTGACTAAGGTCTCTTCTAAATTATATAGTA

GACTATCTCTTTGTAACTGGCCTGTGACTTGGATTTTCCAATTCGATGATCTATCAGGAGTTCTATCCTA

AATAATGTTTCTAACATTGATTTCAGATGCATTCTGATATAGGAGTTCAATATGTAATTGTTCTTTTGTA

ATTTCCCCATTACAAAAAATACTTATATTATCAC

>Brahea_armata_HQ720489.1

GAAAGGTTGCTGCCCATATGGGAAAGGAGGGAGATGCTACTCCTTTTACTGATGTCACAGTAAGTAAAGA

CATAAAACCTTCATGCAAGCTACCTGAGAAGCGTGTCATAATTTAACCTGCTATAAATGCTGGAGCTTTT

TTGCTTATTCAGTTTAAGAATTCCGAGTCACTGTATGTTAAAGAACATTAAGGAAAGTTTCTTTTGTTCT

GCTCCAGGAGGAAGTAATTTTTGCATATGTCTAGCTAAAAGACATTGTTGATAGCTTATACAAGAACAGA

AGGTTATTCAATTTTCATTACCATTAAGGATTTGTATTAGATATGGTTTAGTTAATTATAATCCTTTCAA

CTTCATACCTGGATTTGATTTTCTTTTGAGGGGAGCTTTTGCCTTTGGAGTTCATATTTTCCTCTGGTTT

AGTTAATTATAATCCTGTTATTTGAAGTCTACTTTGAATATCTCTTTTGAAGTTCTTGAGATGTAAGCTT

ACCCTGTAGACAGAAACGTGGCTCTGAAGAATGACTTTGGACGAGTGTAAGTTATTCTGATGATCCTAAA

CTTGTATAAGTTTCAAGACATACTATTTTGACTAAGGTCTCTTCTAAATTATATAGTAGACTATCTCCTT

GTAACTGGCCTGTGACTTGGATTTTCCAATTCGATGATCTATCAGTAGTTCTATCCTAAATAATGTTTCT

AACATTGATTTCAGATGCATTCTGATATAGGAGTTCAATATGTAATTGTTCTTTTGTAATTTCCCCATTA

CAAAAAATACTTATATTATCACAAATTTCCTAGGTGGACAATATCAGCAAAGCTCTTCATAAGT

>Brahea dulcis_HQ720492.1

ATACATAAAACCTCCATGGAAGCTTTACCTGAGAAGCGTGTCATAATTTAACCTGCTATAAATGCTGGAG

CTTTTTTCCTTATTCAGTTTAAGAATTTTGAGTCACTATATCTGATGTTAAAGAACATTAAGGAAAGTTT

CTTTTGTTCTGCTCCAGGAGGAAGTAATTTTTGCATATGTCTAGCTAAAAGACATTGTTGATAGCTTATG

CAAAAACAGAAGSTTATTCAATTTTCATTACCATTAAGGATTTGTATTAGATATGGTTTAGTTAATTATA

ATCCTTTCAACTTCATACCTGGATTTGATTTTCTTTTGAGGGGAGCTTTTGCCTTTGGAGTTCATATTTT

CCTCTGGTTTAGTTAATTATAATCATGTTATTTGAAGTCTACTTTGAATATCTCTTTTGAAGTTCTTGAG

ATGTAAGTTTACCCTGTAGACAGAAACGTGGCTTTGGAGAATGACTTTGGATGAGTGTAAGTTATTCTGA

TGATCCTAAACTTGTATAAGTTTCAAGACATACTATTTTGACTAAGGTCTCTTCTAAATTATATAGTAGA

CTATCTCTTTGTAACTGGCCTGTGACTTGGATTTTCCAATTCGATGATCTATCAGGAGTTCTATCCTAAA

TAATGTTTCTAACATTGATTTCAGATGCATTCTGATATAGGAGTTCAATATGTAATTGTTCTTTTGTAAT

TTCCCCATTACAAAAAATACTTATATTATCACAAATT

>Brahea_brandegeei_HQ720490.1

GAAAGGTTGCTGCCCATATGGGAAAGGAGGGAGATGCTACTCCTTTTACTGATGTCACAGTAAGTAAAGA

CATAAAACCTTCATGCAAGCTACCTGAGAAGCGTGTCATAATTTAACCTGCTATAAATGCTGGAGCTTTT

TTGCTTATTCAGTTTAAGAATTCCGAGTCACTGTATCTGATGTTAAAGAACATTAAGGAAAGTTTCTTTT

GTTCTGCTCCAGGAGGAAGTAATTTTTGCATATGTCTAGCTAAAAGACATTGTTGATAGCTTACACAAGA

ACAGAAGGTTATTCAATTTTCATTACCATTAAGGATTTTTATTAGATATGGTTTAGTTAATTATAATCCT

TTCAACTTCATACCTGGATTTGATTTTCTTTTGAGGGGAGCTTTTGCCTTTGGAGTTCATATTTTCCTCT

GGTTTAGTTAATTATAATCCTGTTATTTGAAGTCTACTTTGAATATCTCTTTTGAAGTTCTTGAGATGTA

AGCTTACCCTGTAGACAGAAACGTGGCTCTGAAGAATGACTTTGGACGAGTGTAAGTTATTCTGATGATC

CTAAACTTGTATAAGTTTCAAGACATACTATTTTGACTAAGGTCTCTTCTAAATTATATAGTAGACTATC

TCCTTGTAACTGGCCTGTGACTTGGATTTTCCAATTCGATGATCTATCAGTAGTTCTATCCTAAATAATG

TTTCTAACATTGATTTCAGATGCATTCTGATATAGGAGTTCAATATGTAATTGTTCTTTTGTAATTTCCC

CATTACAAAAATTACTTATATTATCACAAATTTCCTA

>Brahea_aculeata_HQ720488.1

ATTTAACCTGCTATAAATGCTGGAGCTTTTTTGCTTATTCAGTTTAAGAATTCCGAGTCACTGTATSTKA

TGTTAAAGAACATTAAGGAAAGTTTCTTTTGTTCTGCTCCAGGAGGAAGTAATTTTTGCATATGTCTAGC

TAAAAGACATTGTTGATAGCTTATRCAAGAACAGAAGGTTATTCAATTTTCATTACCATTAAGGATTTGT

ATTAGATATGGTTTAGTTAATTATAATCCTTTCAACTTCATACCTGGATTTGATTTTCTTTTGAGGGGAG

CTTTTGCCTTTGGAGTTCATATTTTCCTCTGGTTTAGTTAATTATAATCCTGTTATTTGAAGTCTACTTT

GAATATCTCTTTTGAAGTTCTTGAGATGTAAGKTTACCCTGTAGACAGAAACGTGGCTCTGAAGAATGAC

TTTGGACGAGTGTAAGTTATTCTGATGATCCTAAACTTGTATAAGTTTCAAGACATACTATTTTGACTAA

GGTCTCTTCTAAATTATATAGTAGACTATCTCCTTGTAACTGGCCTGTGACTTGGATTTTCCAATTCGAT

GATCTATCAGTAGTTCTATCCTAAATAATGTTTCTAACATTGATTTCAGATGCATTCTGATATAGGAGTT

CAATATGTAATTGTACTGTTGTAATTTCCCCATTACAAAAAATAC

>Butia_capitata_EF491157.1

CTACTCCTTTTCTGATGTCACAGTAAGTAAATCCATAGAACCTTCATGCAAGCTTTACCTGACAAGCATG

TCACAATTTAACTTGCTGTAAATGCTGGAGCTTTTATGCATATACAGTTTAAGAATTCCAAGTCACTGTA

TCTGATGTTAAAGAATATCTTGGAAAGTTTCTTTTGTTCTGCCCGAGGAGGAAGTAATTTTTGCATGTGT

CTGGCTAAAAGACATTGTTGTTAGCTCATACAAAAATGCACGGTTATTCAATTTGCATTACCATTAAGGA

TTTGTACTAGATATGGTTTAATTAATATGTTTGAGTTGCATTACCATTAAGGATTTGTACTAGATATGGT

TCAATAAATATGGTTGAGTTGCATTACCCAAGCTCCCGGGGGGGGGGGAGGGGGGTTTTTTAAAAAACCC

CTCCACTTTTAACCCGCAAATTTTTTTTTTTTTTTTTTTTGGGTTTGGGTGGGGGTGGGGGGCACGGGGG

GTAGCTTTTGCCTTTGGAGTTCATATTTTCCTCTGGATTAGTTAATTATAATCCTGTTATTTGAAGTCTA

CTTTGAATATCTCTTTTGAAGTTGGTGATATGTTAGTTTACCCTGTAGACAGAAACGTGGCTCTGAAGAA

TGACCTTGGACGAGTGTCTTTTAAGTTATTCTGATGATCCTAAACTTGTATAAGTTTCAAGACATACTAT

TTTGACTAAGGTCTCTTTTAAATTATGTCATTGACTATCTCCTTGTAATGACCTGTGGCTTGGATTTTCC

AATTCAATGATCTATCAGGAGTTCTATCCTAAATAATGTTTCTAACATTGATTTCAGATATATTCTGATG

TAGGAGTTTAATATGTAATTGTACTTTGTAGTTGCTCCAT

>Calyptrogyne_trichostachys_JQ417494.1

TTGCCTTTAGAGTTCATATTTTCCTTTGGATTAGTTAATTATAATCCTGTTATTTGAAGTCTACTTTAAA

TATCTCTTTTGAAGTTCTTGATTTGTAAGTTTACCGTAATGTGGCTTTGAAGAATGACTTTTGACGAGTG

TCTTGTAAGTTAGTCTGATGATCCTAAACTTGTATAAAGTTTCAAGACATACTATTTTGACCAATCTCTC

TCCTAAATTATATAATAGACTACCTCCTTGTAACTGACCTGTGACTTGGATTTTCCAGTTCAATGATCTA

TCGGGAGTTCTGTCCTAAATAATGTTTCTAACATTGATTTCAGCTATATTCTGATGTAGGAGTTTAATAG

GTAATGGTACTTTTATAATTTCCCCATTACAAAAAATGCTTATATTATCACAAATTTCCCAGGTGGACAA

TATCAGCAAAGCTCTTCATAAG

>Calyptrogyne_panamensis_JQ417492.1

TTGCCTTTAGAGTTCATATTTTCCTTTGGATTAGTTAATTATAATCCTGTTATTTGAAGTCTACTTTAAA

TATCTCTTTTGAAGTTCTTGATTTGTGAGTTTACCGTAATGTGGCTTTGAAGAATGACTTTGGACGAGTG

TCTTGTAAGTTAGTCTGATGATCCTAAACTTGTATAAAGTTCAAGACATACTATTTTGACCAATCTCTCT

CCTAAATTATATAATAGACTACCTCCTTGTAACTGACCTGTGACTTGGATTTTCCAGTTCAATGATCTAT

TGGGAGTTCTGTCCTAAATAATGTTTCTAACATTGATTTCAGCTATATTCTGATGTAGGAGTTTAATAGG

TAATGGTACTTTTATAATTTCCCCATTACAAAAAATGCTTATATTATCGCAAATTTCCTAGGTGGACAAT

ATCAGCAAAGCTCTTCATAAG

>Calyptrogyne_fortunensis_JQ417490.1

TTGCCTTTAGAGTTCATATTTTCCTTTGGATTAGTTAATTATAATCCTGTTATTTGAAGTCTACTTTAAA

TATCTCTTTTGAAGTTCTTGATTTGTRAGTTTACCGTAATGTGGCTTTGAAGAATGACTTTGGACGAGTG

TCTTGTAAGTTAGTCTGATGATCCTAAACTTGTATAAAGTTTCAAGACATACTATTTTGACCAATCTCTC

TCCTAAATTATATAATAGACTACCTCCTTGTAACTGACCTGTGACTTGRATTTTCCAGTTCAATGATCTA

TCGGGAGTTCTGTCCTAAATAATGTTTCTAACATTGATTTCAGCTATATTCTGA

>Calyptrogyne_anomala_JQ417488.1

TTGCCTTTAGAGTTCATATTTTCCTTTGGATTAGTTAATTATAATCCTGTTATTTGAAGTCTACTTTAAA

TATCTCTTTTGAAGTTCTTGATTTGTGAGTTTACCGTAATGTGGCTTTGAAGAATGACTTTGGACGAGTG

TCTTGTAAGTTAGTCTGATGATCCTAAACTTGTATAAAGTTTCAAGACATACTATTTTGACCAATCTCTC

TCCTAAATTATATAATAGACTACCTCCTTGTAACTGACCTGTGACTTGGATTTTCCAGTTCAATGATCTA

TCGGGAGTTCTGTCCTAAATAATGTTTCTAACATTGATTTCAGCTATATTCTGATGTAGGAGTTTAATAG

GTAATGGTACTTTTATAATTTCCCCATTACAAAAAATGCTTATATTATCACAAATTTCCTAGGTGGACAA

TATCAGCAAAGCTCTTCATAAG

>Calyptrogyne_tutensis_JQ417495.1

TTGCCTTTAGAGTTCATATTTTCCATTGGATTAGTTAATTATAATCCTGTTATTTGAAGTCTACTTTAAA

TATCTCTTTTGAAGTTCTTGATTTGTGAGTTTACCGTAATGTGGCTTTGAAGAATGACTTTGGACGAGTG

TCTTGTAAGTTAGTCTGATGATCCTAAACTTGTATAAAGTTTCAAGACATACTATTTTGACCAATCTCTC

TCCTAAATTATATAATAGACTACCTCCTTGTAACTGACCTGTGACTTGGATTTTCCAGTTCAATGATCTA

TCGGGAGTTCTGTCCTAAATAATGTTTCTAACATTGATTTCAGCTATATTCTGATGTAGGAGTTTAATAG

GTAATGGTACTTTTATAATTTCCCCATTACAAAAAATGCTTATATTATCACAAATTTCCTAGGTGGACAA

TATCAGCAAAGCTCTTCATAAG

>Calyptrogyne_panamensis_JQ417493.1

TTGCCTTTAGGGTTCATATTTTCCTTTGGATTAGTTAATTATAATCCTGTTATTTGAAGTCTACTTTAAA

TATCTCTTTTGAAGTTCTTGATTTGTAAGTTTACCGTAATGTGGCTTTGAAGAATGACTTTGGACGAGTG

TCTTGTAAGTTAGTCTGATGATCCTAAACTTGTATAAAGTTTCAAGACATACTATTTTGACCAATCTCTC

TCCTAAATTATATAATAGACTACCTCCTTGTAACTGACCTGTGACTTGGATTTTCCAGTTCGATGATCTA

TCGGGAGTTCTGTCCTAAATAATGTTTCTAACATTGATTTCAGCTATATTCTGATGTAGGAGTTTAATAG

GTAATGGTACTTTTATAATTTCCCCATTACAAAAAATGCTTATATTATCACAAATTTCCTAGGTGGACAA

TATCAGCAAAGCTCTTCATAAG

>Calyptrogyne_osensis_JQ417491.1

TTGCCTTTAGAGTTCATATTTTCCTTTGGATTAGTTAATTATAATCCTGTTATTTGAAGTCTACTTTAAA

TATCTCTTTTGAAGTTCTTGATTTGTAAGTTTACCGTAATGTGGCTTTGAAGAATGACTTTGGACGAGTG

TCTTGTAAGTTAGTCTGATGATCCTAAACTTGTATAAAGTTTCAAGACATACTATTTTGACCAATCTCTC

TCCTAAATTATATAATAGACTACCTCCTTGTAACTGACCTGTGACTTGGATTTTCCAGTTCAATGATCTA

TCGGGAGTTCTGTCCTAAATAATGTTTCTAACATTGATTTCAGCTATATTCTGATGTAGGAGTTTAATAG

GTAATGGTACTTTTATAATTTCCCCATTACAAAAAATGCTTATATTATCACAAATTTCCTAGGTGGACAA

TATCAGCAAAGCTCTTCATAAG

>Calyptrogyne_brachystachys_JQ417489.1

TTGCCTTTAGAGTTCATATTTTCCTTTGGATTAGTTAATTATAATCCTGTTATTTGAAGTCTACTTTAAA

TATCTCTTTTGAAGTTCTTGATTTGTAAGTTTACCGTAATGTGGCTTTGAAGAATGACTTTGGACGAGTG

CCTTGTAAGTTAGTCTGATGATCCTAAACTTGTATAAAGTTTCAAGACATACTATTTTGACCAATCTCTC

TCCTAAATTATATAATAGACTACCTCCTTGTAACTGACCTGTGACTTGGATTTTCCAGTTCAATGATCTA

TCGGGAGTTCTGTCCTAAATAATGTTTCTAACATTGATTTCAGCTATATTCTGATGTAGGAGTTTAATAG

GTAATAGTACTTTTATAATTTCCCCATTACAAAAAATGCTTATATTATCACAAATTTCCCAGGTGGACAA

TATCAGCAAAGCTCTTCATAAG

>Calyptrogyne_allenii_JQ417487.1

TTGCCTTTAGAGTTCATATTTTCCTTTGGATTAGTTAATTATAATCCTGTTATTTGAAGTCTACTTTAAA

TATCTCTTTTGAAGTTCTTGATTTGTGAGTTTACCGTAATGTGGCTTTGAAGAATGACTTTGGACGAGTG

TCTTGTAAGTTAGTCTGATGATCCTAAACTTGTATAAAGTTTCAAGACATACTATTTTGACCAATCTCTC

TCCTAAATTATATAATAGACTACCTCCTTGTAACTGACCTGTGACTTGGATTTTCCAGTTCAATGATCTA

TCGGGAGTTCTGTCCTAAATAATGTTTCTAACATTGATTTCAGCTATATTCTGATGTAGGAGTTTAATAG

GTAATGGTACTTTTATAATTTCCCCATTACAAAAAATGCTTATATTATCACAAATTTCCTAGGTGGACAA

TATCAGCAAAGCTCTTCATAAG

>Calyptrogyne_costatifrons_AJ830208.1

TGAGCCCTTGCCTTTAGAGTTCATATTTTCCTTTGGATTAGTTAATTATAATCCTGTTATTTGAAGTCTA

CTTTAAATATCTCTTTTGAAGTTCTTGATTTGTGAGTTTACCGTAATGTGGCTTTGAAGAATGACTTTGG

ACGAGTGTCTTGTAAGTTAGTCTGATGATCCTAAACTTGTATAAAGTTTCAAGACATACTATTTTGACCA

ATCTCTCTCCTAAATTATATAATAGACTACCTCCTTGTAACTGACCTGTGACTTGGATTTTCCAGTTCAA

TGATCTATCGGGAGTTCTGTCCTAAATAATGTTTCTAACATTGATTTCAGCTATATTCTGATGTAGGAGT

TTAATAGGTAATGGTACTTTTATAATT

>Calyptrogyne_ghiesbreghtiana_AY779364.1

TTGCCTTTAGAGTTCATATTTTCCTTTGGATTAGTTAATTATAATCCTGTTATTTGAAGTCTACTTTAAA

TATCTCTTTTGAAGTTCTTGATTTGTGAGTTTACCGTAATGTGGCTTTGAAGAATGACTTTGGACGAGTG

TCTTGTAAGTTAGTCTGATGATCCTAAACTTGTATAAAGTTTCAAGACATACTATTTTGACCAATCTCTC

TCCTAAATTATATAATAGACTACCTCCTTGTAACTGACCTGTGACTTGAATTTTCCAGTTCAATGATCTA

TCGGGAGTTCTGTCCTAAATAATGTTTCTAACATTGATTTCAGCTATATTCTGATGTAGGAGTTTAATAG

GTAATGGTACTTTTATAATTTCCCCATTACAAAAAATGCTTATATTATCACAAATTTCCTAGGTGGACAA

TATCAGCAAAGCTCTTCATAAG

>Calyptronoma_plumeriana_AY779367.1

TTGCCTTTAGAGTTCATATTTTCCTTTGGATTAGTTAATTATAATCCTGTTATTTGAAGTCTGCTGTAAA

TATCTCTTTTGAAGTTCTTGATATGTAAGTTTACCATAACGTGGCTTTGAAGAATGACTTTGGACGAGTG

TCTTGTAAGTTATTCTGATGATCCTAAACTTGTATAAAGTTTCAAGACATACTATTTTGACCAATCTCTC

TCCTAAATTATATAATAGACTACCTCCTTGTAACTGACCTGTGACTTGGATTTTCCAGTTCAATGATCTA

TCAGGAGTTCTGTCCTAAATAATGTTTCTAACATTGATTTCAGATATATTCTGATGTAGGAGTTTAATAG

GTAATGGTACTTTTATAATTTCCCCATTACAAAAAATGCTTATATTATCACAAATTTCCTAGGTGGACAA

TATCAGCAAAGCTCTTCATAAG

>Calyptronoma_occidentalis_AY779365.1

TTGCCTTTAGAGTTCATATTTTCCTTTGGATTAGTTAATTATAATCCTGTTATTTGAAGTCTACTTTAAA

TATCTCTTTTGAAGTTCTTGATATGTAAGTTTACCGTAATGTGGCTTTGAAGAATGACTTTGGACGAGTG

TCTTGTAAGTTATTCTGATGATCCTAAACTTGTATAAAGTTTCAAGACATACTATTTTGACCAATCTCTC

TCCTAAATTATATAATAGACTACCTCCTTGTAACTGACCTGTGACTTGGATTTTCCAGTTCAATGATCTG

TCGGGAGTTCTGTCCTAAATAGTGTTTCTAACATTGATTTCAGATATATTCTGATGTAGGAGTTTAATAG

GTAATGGTACTTTTATAATTTTCCCATAACAAAAAATGCTTATATTATCACAAATTTCCTAGGGGGACAA

TATCAGCAAAGCTCTTCATAAG

>Calyptronoma_rivalis_AY779366.1

TTGCCTTTAGAGTTCATATTTTCCTTTGGATTAGTTAATTATAATCCTGTTATTTGAAGTCTACTTTAAA

TATCTCTTTTGAAGTTCTTGATATGTAAGTTTACCGTAATGTGGCTTTGAAGAATGACTTTGGACGAGTG

TCTTGTAAGTTATTCTGATGATCCTAAACTTGTATAAAGTTTCAAGACATACTATTTTGACCAATCTCTC

TCCTAAATTATATAATAGACTACCTCCTTGTAACTGACCTGTGACTTGGATTTTCCAGTTCAATGATCTA

TCGGGAGTTCTGTCCTAAATAATGTTTCTAACATTGATTTCAGATATATTCTGATGTAGGAGTTTAATAG

GTAATGGTACTTTTATAATTTCCCCATTACAAAAAATGCTTATATTATCACAAATTTCCTAGG

>Calyptronoma_plumeriana_AJ830209.1

GAGCACTTGCCTTTAGAGTTCATATTTTCCTTTGGATTAGTTAATTATAATCCTGTTATTTGAAGTCTAC

TTTAAATATCTCTTTTGAAGTTCTTGATTTGTGAGTTTACCGTAATGTGGCTTTGAAGAATGACTTTGGA

CGAGTGTCTTGTAAGTTAGTCTGATGATCCTAAACTTGTATAAAGTTTCAAGACATACTATTTTGACCAA

TCTCTCTCCTAAATTATATAATAGACTACCTCCTTGTAACTGACCTGTGACTTGGATTTTCCAGTTCAAT

GATCTATCGGGAGTTCTGTCCTAAATAATGTTTCTAACATTGATTTCAGCTATATTCTGATGTAGGAGTT

TAATAGGTAATGGTACTTTTATAATTTCCCCATTACAAAAAATGCTTATATTATCACAAATTTCCTAGGT

GGACAATATCAGCAAAGCTCTTCATAAGTGTGG

>Ceroxylon_quindiuense_AJ830162.1

AAGGAGGGAGATGCTACTCCTTTTACTGATGTCACAGTAAGTAAATCCATAGAAGCTTCATGCAAGCTTT

ACCTGAGAAGCATGTCACAATTTAATTTGCTATAATGCTGGAGCTTTTTTTGCTTATTCGGTTTTAGAAT

TTCGAGGCACTGTATCTGATGTTAAAGAACACTAAGGAAGTTTTCTTTCATTCTGCTCCAGGAGGGAGTA

ACTTTTGCATATATCTAGCTAAAAGATATTGTGCATAGCTTATACAAAAACACAAGGTTATTGAATTTGC

ATTATGCCTACCTCCCCCTCCCATGCGGGGTGTCTAGAACCTTTCAACTTCATACCTGGATTTGTTTTTC

TTTATGGGGAGCTTTTGCCTTTGGAGTTCATATTTTCCTCTGGATCAGTTAATTATAATCCTGTTATTTG

AAGTCTACTTTTGATATCTCTGTTGAAGTTGTTGAGATGTAAGTTTACCCTGTAGACAGAAACGTGGCTT

TGAAGAATGACCTCGGACGAGTGTCTTGTAAGCTTTCTGATGATCCTAAACTTGTATAAGTTTCAGGACA

TACTATTTTGACTAAGGTCTCTCCAAAATTATATTAGTGGACTATCTCCTTGTAACTAGCCTGTGACTTA

GATTTTCCAATTCCATGATCTATCAGGAGTTCTATCCTAAGTAATGTTTCTAACATTGATTCTGGATATA

TTCTGATGTAGGAGTTTAATATGTAATTGTACTTTTGTAATTGCCCCATTAAAAAAATACTTATATCACA

AATTTCCTAGGTGGACAA

>Ceroxylon_quindiuense_AJ830160.1

AAGGAGGGAGATGCTACTCCTTTTACTGATGTCACAGTAAGTAAATCCATAGAAGCTTCATGCAAGCTTT

ACCTGAGAAGCATGTCACAATTTAGTTTGCTATAATGCTGGAGCTTTTTTTGCTTATTCAGTTTTAGAAT

TTCGAGGCACTGTATCTGATGTTAAAGAACACTAAGGAAGTTTTCTTTCATTCTGCTCCAGGAGGGAGTA

ACTTTTGCATATATCTAGCTAAAAGATATTGTGCATAGCTTATACAAAAACACAAGGTTATTGAATTTGC

ATTATGCCTACCTCCCCCTCCCATGCGGGGTGTCTAGGACCTTTCAACTTCATACCTGGATTTGTTTTTT

TTATGGGGAGCTTTTGCCTTTGGAGTTCATATTTTCCTCTGGATCAGTTAATTATAATCCTGTTATTTGA

AGTCTACTTTTGATATCTCTGTTGAAGTTGTTGAGATGTAAGTTTACCCTGTAGACAGAAACGTGGCTTT

GAAGAATGACCTCGGACGAGTGTCTTGTAAGCTTTCTGATGATCCTAAACTTGTATAAGTTTCGGGACAT

ACTATTTTGACTAAGGTCTCTCCAAAATTATATTAGTGGACTATCTCCTTGTAACTAGCCTGTGACTTAG

ATTTTCCAATTCCATGATCTATCAGGAGTTCTATCCTAAATAATGTTTCTAACATTGATTTTGGATATAT

TCTGATGTAGGAGTTTAATATGTAATTGTACTTTTGTAATTGCCCCATTAAAAAAATACTTATATCACAA

ATTTCCTAGGTGGACAA

>Ceroxylon_quindiuense_AJ830158.1

GGAGGGAGATGCTACTCCTTTTACTGATGTCACAGTAAGTAAATCCATAGAAGCTTCATGCAAGCTTTAC

CTGAGAAGCATGTCACAGTTTAATTTGCTATAATGCTGGAGCTTTTTTTGCTTATTCAGTTTTAGAATTT

CGAGGCACTGTATCTGATGTTAAAGAACACTAAGGAAGTTTTCTTTCATTCTGCTCCAGGAGGGAGTAAC

TTTTGCATATATCTAGCTAAAAGATATTGTGCATAGCTTATACAAAAACACAAGGTTATTGAATTTGCAT

TATGCCTACCTCCCCCTCCCATGCGGGGTGTCTAGAACCTTTCAACTTCATACCTGGATTTGTTTTTTTT

TATGGGGAGCTTTTGCCTTTGGAGTTCATATTTTCCTCTGGATCAGTTAATTATAATCCTGTTATTTGAA

GTCTACTTTTGATATCTCTGTTGAAGTTGTTGAGATGTAAGTTTACCCTGTAGACAGAAACGTGGCTTTG

AAGAATGACCTCGGACGAGTGTCTTGTAAGCTTTCTGATGATCCTAAACTTGTATAAGTTTCAGGACATA

CTATTTTGACTAAGGTCTCTCCAAAATTATATTAGTGGACTGTCTCCTTGTAACTAGCCTGTGACTTAGA

TTTTCCAATTCCATGATCTATCAGGAGTTCTATCCTAAATAATGTTTCTAACATTGATTTTGGATATATT

CTGATGTAGGAGTTTAATATGTAATTGTACTTTTGTAATTGCCCCATTAAAAAAATACTTATATCACAAA

TTTCCTAGGTGGACAATATC

>Ceroxylon_quindiuense_AJ830161.1

AAGGAGGGAGATGCTACTCCTTTTACTGATGTCACAGTAAGTAAATCCATAGAAGCTTCATGCAAGCTTT

ACCTGAGAAGCATGTCACAATTTAATTTGCTATAATGCTGGAGCTTTTTTTGCTTATTCAGTTTTAGAAT

TTCGAGGCACTGTATCTGATGTTAAAGAACACTAAGGAAGTTTTCTTTCATTCTGCTCCAGGAGGGAGTA

ACTTTTGCATATATCTAGCTAAAAGATATTGTGCATAGCTTATACAAAAACACAAGGTTATTGAATTTGC

ATTATGCCTACCTCCCCCTCCCATGCGGGGTGTCTAGAACCTTTCAACTTCATACCTGGATTTGTTTTTT

TTTATGGGGAGCTTTTGCCTTTGGAGTTCATATTTTCCTCTGGATCGGTTAATTATAATCCTGTTATTTG

AAGTCTACTTTTGATATCTCTGTTGAAGTTGTTGAGATGTAAGTTTACCCTGTAGACAGAAACGTGGCTT

TGAAGAATGACCTCGGACGAGTGTCTTGTAAGCTTTCTGATGATCCTAAACTTGTATAAGTTTCAGGACA

TACTATTTTGACTAAGGTCTCTCCAAAATTATATTAGTGGACTATCTCCTTGTAACTAGCCTGTGACTTA

GATTTTCCAATTCCATGATCTATCAGGAGTTCTATCCTAAATAATGTTTCTAACATTGATTTTGGATATA

TTCTGATGTAGGAGTTTAATATGTAATTGTACTTTTGTAATTGCCCCATTAAAAAAAATACTTATATCAC

AAATTTCCTAGGTGGACA

>Ceroxylon_quindiuense_AJ830159.1

AAGGAGGGAGATGCTACTCCTTTTACTGATGTCACAGTAAGTAAATCCATAGAAGCTTCATGCAAGCTTT

ACCTGAGAAGCATGTCACAATTTAATTTGCTATAATGCTGGAGCTTTTTTTGCTTATTCAGTTTTAGAAT

TTCGAGGCACTGTATTTGATGTTAAAGAACACTAAGGAAGTTTTCTTTCATTCTGCTCCAGGAGGGAGTA

ACTTTTGCATATATCTAGCTAAAAGATATTGTGCATAGCTTATACAAAAACACAAGGTTATTGAATTTGC

ATTATGCCTACCTCCCCCTCCCATGCGGGGTGTCTAGAACCTTTCAACTTCATACCTGGATTTGTTTTTT

TTTATGGGGAGCTTTTGCCTTTGGAGTTCATATTTTCCTCTGGATCAGTTAATTATAATCCTGTTATTTG

AAGTCTACTTTTGATATCTCTGTTGAAGTTGTTGAGATGTAAGTTTACCCTGTAGACAGAAACGTGGCTT

TGAAGAATGACCTCGGACGAGTGTCTTGTAAGCTTTCTGATGATCCTAAACTTGTATAAGTTTCAGGACA

TACTATTTTGACTAAGGTCTCTCCAAAATTATATTAGTGGACTATCTCCTTGTAACTAGCCTGTGACTTA

GATTTTCCAATTCCATGATCTATCAGGAGTTCTATCCTAAATAATGTTTCTAACATTGATTTTGGATATA

TTCTGATGTAGGAGTTTAATATGTAATTGTACTTTTGTAATTGCCCCATTAAAAAAAATACTTATATCAC

AAATTTCCTAGGTGGACAA

>Ceroxylon_quindiuense_AJ830157.1

AAGGAGGGAGATGCTACTCCTTTTACTGATGTCACAGTAAGTAAATCCATAGAAGCTTCATGCAAGCTTT

ACCTGAGAAGCATGTCACAATTTAATTTGCTATAATGCTGGAGCTTTTTTTGCTTATTCAGTTTTAGAAT

TTCGAGGCACTGTATCTGATGTTAAAGAACACTAAGGAAGTTTTCTTTCATTCTGCTCCAGGAGGGAGTA

ACTTTTGCATATATCTAGCTAAAAGATATTGTGCATAGCTTATACAAAAACACAAGGTTATTGAATTTGC

ATTATGCCTACCTCCCCCTCCCATGCGGGGTGTCTAGAACCTTTCAACTTCATACCTGGATTTGTTTTTT

TTTATGGGGAGCTTTTGCCTTTGGAGTTCATATTTTCCTCTGGATCAGTTAATTATAATCCTGTTATTTG

AAGTCTACTTTTGATATCTCTGTTGAAGTTGTTGAGATGTAAGTTTACCCTGTAGACAGAAACGTGGCTT

TGAAGAATGACCTCGGACGAGTGTCTTGTAAGCTTTCTGATGATCCTAAACTTGTATAAGTTTCAGGACA

TACTATTTTGACTAAGGTCTCTCCAAAATTATATTAGTGGACTATCTCCTTGTAACTAGCCTGTGACTTA

GATTTTCCAATTCCATGATCTATCAGGAGTTCTATCCTAAATAATGTTTCTAACATTGATTTTGGATATA

TTCTGATGTAGGAGTTTAATATGTAATTGTACTTTTGTAATTGCCCCATTAAAAAAATACTTATATCACA

AATTTCCTAGGTGGACAATATCA

>Ceroxylon_vogelianum_EF128433.1

GTAAATCCATAGAAGCTTCATGCAAGCTTTACCTGAGAAGCATGTCACAATTTAATTTGCTATAATGCTG

GAGCTTTTTTTGCTTATTCAGTTTTAGAATTTCGAGGCACTGTATCTGATGTTAAAGAACATAAGGAAGT

TTTCTTTCATTCTGCTCCAGGAGGGAGTAACTTTTGCATATATCTAGCTAAAAGATATTGTGCATAGCTT

ATACAAAAACACAAGGTTATTGAATTTGCATTATGCCTACCTCCCCCTCCCATGCGGGGTGTCTAGAACC

TTTCAACTTCATACCTGGATTTGTTTTTTTTTATGGGGAGCTTTTGCCTTTGGAGTTCATATTTTCCTCT

GGATCAGTTAATTATAATCCTGTTATTTGAAGTCTACTTTTGATATCTCTGTTGAAGTTGTTGAGATGTA

AGTTTACCCTGTAGACAGAAACGTGGCTTTGAAGAATGACCTCGGACGAGTGTCTTGTAAGCTTTCTGAT

GATCCTAAACTTGTATAAGTTTCAGGACATACTATTTTGACTAAGGTCTCTCCAAAATTATATTAGTGGA

CTATCTCYTTGTAACTAGCCTGTGACTTAGATTTTCCAATTCCATGATCTATCAGGAGTTCTATCCTAAA

TAATGTTTCTAACATTGATTTTGGATATATTCTGATGTAGGAGTTTAATATGTAATTGTACTTTTGTAAT

TGCCCCA

>Ceroxylon_ventricosum_EF128431.1

GTAAATCCATAGAAGCTTCATGCAAGCTTTACCTGAGAAGCATGTCACAATTTAATTTGCTATAATGCTG

GAGCTTTTTTTGCTTATTCAGTTTTAGAATTTCGAGGCACTGTATCTGATGTTAAAGAACACTAAGGAAG

TTTTCTTTCATTCTGCTCCAGGAGGGAGTAACTTTTGCATATATCTAGCTAAAAGATATTGTGCATAGCT

TATACAAAAACACAAGGTTATTGAATTTGCATTATGCCTACCTCCCCCTCCCATGCGGGGTGTCTAGAAC

CTTTCAACTTCATACCTGGATTTGTTTTTTTTTATGGGGAGCTTTTGCCTTTGGAGTTCATATTTTCCTC

TGGATCAGTTAATTATAATCCTGTTATTTGAAGTCTACTTTTGATATCTCTGTTGAAGTTGTTGAGATGT

AAGTTTACCCTGTAGACAGAAACGTGGCTTTGAAGAATGACCTCGGACGAGTGTCTTGTAAGCTTTCTGA

TGATCCTAAACTTGTATAAGTTTCAGGACATACTATTTTGACTAAGGTCTCTCCAAAATTATATTAGTGG

ACTATCTCCTTGTAACTAGCCTGTGACTTAGATTTTCCAATTCCATGATCTATCAGGAGTTCTATCCTAA

ATAATGTTTCTAACATTGATTTTGGATATATTCTGATGTAGGAGTTTAATATGTAATTGTACTTTTGTAA

TTGCCCCA

>Ceroxylon_parvum_EF128429.1

GTAAATCCATAGAAGCTTCATGCAAGCTTTACCTGAGAAGCATGTCACAATTTAATTTGCTATAATGCTG

GAGCTTTTTTTGCTTATTCAGTTTTAGAATTTCGAGGCACTGTATCTGATGTTAAAGAACACTAAGGAAG

TTTTCTTTCATTCTGCTCCAGGAGGGAGTAACTTTTGCATATATCTAGCTAAAAGATATTGTGCATAGCT

TATACAAAAACACAAGGTTATTGAATTTGCATTATGCCTACCTCCCCCTCCCATGCGGGGTGTCTAGAAC

CTTTCAACTTCATACCTGGATTTGTTTTTTTTTATGGGGAGCTTTTGCCTTTGGAGTTCATATTTTCCTC

TGGATCAGTTAATTATAATCCTGTTATTTGAAGTCTACTTTTGATTTCTCTGTTGAAGTTGTTGAGATGT

AAGTTTACCCTGTAGACAGAAACGTGGCTTTGAAGAATGACCTCGGACGAGTGTCTTGTAAGCTTTCTGA

TGATCCTAAACTTGTATAAGTTTCAGGACATACTATTTTGACTAAGGTCTCTCCAAAATTATATTAGTGG

ACTATCTCCTTGTAACTAGCCTGTGACTTAGATTTTCCAATTCCATGATCTATCAGGAGTTCTATCCTAA

ATAATGTTTCTAACATTGATTTTGGATATATTCTGATGTAGGAGTATAATATGTAATTGTACTTTTGTAA

TTGCCCCG

>Ceroxylon_parvifrons_EF128427.1

GTAAATCCATAGAAGCTTCATGCAAGCTTTACCTGAGAAGCATGTCACAATTTAATTTGCTATAATGCTG

GAGCTTTTTTTGCTTATTCAGTTTTAGAATTTCGAGGCACTGTATCTGATGTTAAAGAACACTAAGGAAG

TTTTCTTTCATTCTGCTCCAGGAGGGAGTAACTTTTGCATATATCTAGCTAAAAGATATTGTGCATAGCT

TATACAAAAACACAAGGTTATTGAATTTGCATTATGCCTACCTCCCCCTCCCATGCGGGGTGTCTAGAAC

CTTTCAACTTCATACCTGGATTTGTTTTTTTAATGGGGAGCTTTTGCCTTTGGAGTTCATATTTTCCTCT

GGATCAGTTAATTATAATCCTGTTATTTGAAGTCTACTTTTGATATCTCTGTTGAAGTTGTTGAGATGTA

AGTTTACCCTGTAGACAGAAACGTGGCTTTGAAGAATGACCTCGGACGAGTGTCTTGTAAGCTTTCTGAT

GATCCTAAACTTGTATAAGTTTCAGGACATACTATTTTGACTAAGGTCTCTCCAAAATTATATTAGTGGA

CTATCTCCTTGTAACTAGCCTGTGACTTAGATTTTCCAATTCCATGATCTATCAGGAGTTCTATCCTAAA

TAATGTTTCTAACATTGATTTTGGATATATTCTGATGTAGGAGTTTAATATGTAATTGTACTTTTGTAAT

TGCCCCA

>Ceroxylon_echinulatum_EF128425.1

GTAAATCCATAGAAGCTTCATGCAAGCTTTACCTGAGAAGCATGTCACAATTTAATTTGCTATAATGCTG

GAGCTTTTTTTGCTTATTCAGTTTTAGAATTTCGAGGCACTGTATCTGATGTTAAAGAACACTAAGGAAG

TTTTCTTTCATTCTGCTCCAGGAGGGAGTAACTTTTGCATATATCTAGCTAAAAGATATTGTGCATAGCT

TATACAAAAACACAAGGTTATTGAATTTGCATTATGCCTACCTCCCCCTCCCATGCGGGGTGTCTAGAAC

CTTTCAACTTCATACCTGGATTTGTTTTTTTTTATGGGGAGCTTTTGCCTTTGGAGTTCATATTTTCCTC

TGGATCAGTTAATTATAATCCTGTTATTTGAAGTCTACTTTTGATATCTCTGTTGAAGTTGTTGAGATGT

AAGTTTACCCTGTAGACAGAAACGTGGCTTTGAAGAATGACCTCGGACGAGTGTCTTGTAAGCTTTCTGA

TGATCCTAAACTTGTATAAGTTTCAGGACATACTATTTTGACTAAGGTCTCTCCAAAATTATATTAGTGG

ACTATCTCCTTGTAACTAGCCTGTGACTTAGATTTTCCAATTCCATGATCTATCAGGAGTTCTATCCTAA

ATAATGTTTCTAACATTGATTTTGGATATATTCTGATGTAGGAGTTTAATATGTAATTGTACTTTTGTAA

TTGCCCCA

>Ceroxylon_amazonicum_EF128423.1

GTAAATCCATAGAAGCTTCATGCAAGCTTTACCTGAGAAGCATGTCACAATTTAATTTGCTATAATGCTG

GAGCTTTTTTTGCTTATTCAGTTTTAGAATTKCGAGGCACTGTATCTGATGTTAAAGAACACTAAGGAAG

TTTTCTTTCATTCTGCTCCAGGAGGGAGTAACTTTTGCATATATCTAGCTAAAAGATATTGTGCATAGCT

TATACAAAAACACAAGGTTATTGAATTTGCATTATGCCTACCTCCCCCTCCCATGCGGGGTGTCTAGAAC

CTTTCAACTTCATACCTGGATTTGTTTTTTTTTATGGGGAGCTTTTGCCTTTGGAGTTCATATTTTCCTA

TGGATCAGTTAATTATAATCCTGTTATTTGAAGTCTACTTTTGATATCTCTGTTGAAGTTGTTGAGATGT

AAGTTTACCCTGTAGACAGAAACGTGGCTTTGAAGAATGACCTCGGACGAGTGTCTTGTAAGCTTTCTGA

TGATCCTAAACTTGTATAAGTTTCAGGACATACTATTTTGACTAAGGTCTCTCCAAAATTATATTAGTGG

ACTATCTCCTTGTAACTAGCCTGTGACTTAGATTTTCCAATTCCATGATCTATCAGGAGTTCTATCCTAA

ATAATGTTTCTAACATTGATTTTGGATATATTCTGATGTAGGAGTTTAATATGTAATTGTACTTTTGTAA

TTGCCCCA

>Ceroxylon_alpinum_subsp._EF128421.1

GTAAATCCATAGAAGCTTCATGCAAGCTTTACCTGAGAAGCATGTCACAATTTAATTTGCTATAATGCTG

GAGCTTTTTTTGCTTATTCAGTTTTAGAATTTCGAGGCACTGTATCTGATGTTAAAGAACACTAAGGAAG

TTTTCTTTCATTCTGCTCCAGGAGGGAGTAACTTTTGCATATATCTAGCTAAAAGATATTGTGCATAGCT

TATACAAAAACACAAGGTTATTGAATTTGCATTATGCCTACCTCCCCCTCCCATGCGGGGTGTCTAGAAC

CTTTCAACTTCATACCTGGATTTGTTTTTTTTTATGGGGAGCTTTTGCCTTTGGAGTTCTTATTTTCTCT

GGATCAGTTAATTATAATCCTGTTATTTGAAGTCTACTTTTGATATCTCTGTTGAAGTTGTTGAGATGTA

AGTTTACCCTGTAGACAGAAACGTGGCTTTGAAGAATGACCTCGGACGAGTGTCTTGTAAGCTTTCTGAT

GATCCTAAACTTGTATAAGTTTCAGGACATACTATTTTGACTAAGGTCTCTTTAAAATTATATTAGTGGA

CTATCTCCTTGTAACTAGCCTGTGACTTAGATTTTCCAATTCCATGATCTATCAGGAGTTCTATCCTAAA

TAATGTTTCTAACATTGATTTTGGATATATTCTGATGTAGGAGTTTAATATGTAATTGTACTTTTGTAAT

TGCCCCA

>Ceroxylon_vogelianum_EF128432.1

GTAAATCCATAGAAGCTTCATGCAAGCTTTACCTGAGAAGCATGTCACAATTTAATTTGCTATAATGCTG

GAGCTTTTTTTGCTTATTCAGTTTTAGAATTTCGAGGCACTGTATCTGATGTTAAAGAACACTAAGGAAG

TTTTCTTTCATTCTGCTCCAGGAGGGAGTAACTTTTGCATATATCTAGCTAAAAGATATTGTGCATAGCT

TATACAAAAACACAAGGTTATTGAATTTGCATTATGCCTACCTCCCCCTCCCATGCGGGGTGTCTAGAAC

CTTTCAACTTCATACCTGGATTTGTTTTTTTTTATGGGGAGCTTTTGCCTTTGGAGTTCATATTTTCCTC

TGGATCAGTTAATTATAATCCTGTTATTTGAAGTCTACTTTTGATATCTCTGTTGAAGTTGTTGAGATGT

AAGTTTACCCTGTAGACAGAAACGTGGCTTTGAAGAATGACCTCGGACGAGTGTCTTGTAAGCTTTCTGA

TGATCCTAAACTTGTATAAGTTTCAGGACATACTATTTTGACTAAGGTCTCTCCAAAATTATATTAGTGG

ACTATCTCCTTGTAACTAGCCTGTGACTTAGATTTTCCAATTCCATGATCTATCAGGAGTTCTATCCTAA

ATAATGTTTCTAACATTGATTTTGGATATATTCTGATGTAGGAGTTTAATATGTAATTGTACTTTTGTAA

TTGCCCCA

>Ceroxylon_parvum_EF128430.1

GTAAATCTATAGAAGCTTCATGCAAGCTTTACCTGAGAAGCAGGTCACAATTTAATTTGCTATAATGCTG

GAGCTTTTTTTGCTTATTCAGTTTTAGAATTTCGAGGCACTGTATCTGATGTTAAAGAACACTAAGGAAG

TTTTCTTTCATTCTGCTCCAGGAGGGAGTAACTTTTGCATATATCTAGCTAAAAGATATTGTGCATAGCT

TATACAAAAACACAAGGTTATTGAATTTGCATTATGCCTACCTCCCCCTCCCATGCGGGGTGTCTAGAAC

CTTTCAACTTCATACCTGGATTTGTTTTTTTTTTATGGGGAGCTTTTGCCTTTGGAGTTCATATTTTCCT

CTGGATCAGTTAATTATAATCCTGTTATTTGAAGTCTACTTTTGATATCTCTGTTGAAGTTGTTGAGATG

TAAGTTTACCCTGTAGACAGAAATGTGGCTTTGAAGAATGACCTCGGACGAGTGTCTTGTAAGCTTTCTG

ATGATCCTAAACTTGTATAAGTTTCAGGACATACTATTTTGACTAAGGTCTCTCCAAAATTATATTAGTG

GACTATCTCCTTGTAACTAGCCTGTGTCTTAGATTTTCCAATTCCATGATCTATCAGGAGTTCTATCCTA

AATAATGTTTCTA

>Ceroxylon_parvifrons_EF128428.1

GTAAATCCATAGAAGCTTCATGCAAGCTTTACCTGAGAAGCATGTCACAATTTAATTTGCTATAATGCTG

GAGCTTTTTTTGCTTATTCAGTTTTAGAATTTCGAGGCACTGTATCTGATGTTAAAGAACACTAAGGAAG

TTTTCTTTCATTCTGCTCCAGGAGGGAGTAACTTTTGCATATATCTAGCTAAAAGATATTGTGCATAGCT

TATACAAAAACACAAGGTTATTGAATTTGCATTATGCCTACCTCCCCCTCCCATGCGGGGTGTCTAGAAC

CTTTCAACTTCATACCTGGATTTGTTTTTTTAATGGGGAGCTTTTGCCTTTGGAGTTCATATTTTCCTCT

GGATCAGTTAATTATAATCCTGTTATTTGAAGTCTACTTTTGATATCTCTGTTGAAGTTGTTGAGATGTA

AGTTTACCCTGTAGACAGAAACGTGGCTTTGAAGAATGACCTCGGACGAGTGTCTTGTAAGCTTTCTGAT

GATCCTAAACTTGTATAAGTTTCAGGACATACTATTTTGACTAAGGTCTCTCCAAAATTATATTAGTGGA

CTATCTCCTTGTAACTAGCCTGTGACTTAGATTTTCCAATTCCATGATCTATCAGGAGTTCTATCCTAAA

TAATGTTTCTAACATTGATTTTGGATATATTCTGATGTAGGAGTTTAATATGTAATTGTACTTTTGTAAT

TGCCCCA

>Ceroxylon_echinulatum_EF128426.1

GTAAATCCATAGAAGCTTCATGCAAGCTTTACCTGAGAAGCATGTCACAATTTAATTTGCTATAATGCTG

GAGCTTTTTTTGCTTATTCAGTTTTAGAATTTCGAGGCACTGTATCTGATGTTAAAGAACACTAAGGAAG

TTTTCTTTCATTCTGCTCCAGGAGGGAGTAACTTTTGCATATATCTAGCTAAAAGATATTGTGCATAGCT

TATACAAAAACACAAGGTTATTGAATTTGCATTATGCCTACCTCCCCCTCCCATGCGGGGTGTCTAGAAC

CTTTCAACTTCATACCTGGATTTGTTTTTTTTTATGGGGAGCTTTTGCCTTTGGAGTTCATATTTTCCTC

TGGATCAGTTAATTATAATCCTGTTATTTGAAGTCTACTTTTGATATCTCTGTTGAAGTTGTTGAGATGT

AAGTTTACCCTGTAGACAGAAACGTGGCTTTGAAGAATGACCTCGGACGAGTGTCTTGTAAGCTTTCTGA

TGATCCTAAACTTGTATAAGTTTCAGGACATACTATTTTGACTAAGGTCTCTCCAAAATTATATTAGTGG

ACTATCTCCTTGTAACTAGCCTGTGACTTAGATTTTCCAATTCCATGATCTATCAGGAGTTCTATCCTAA

ATAATGTTTCTAACATTGATTTTGGATATATTCTGATGTAGGAGTTTAATATGTAATTGTACTTTTGTAA

TTGCCCCA

>Ceroxylon_echinulatum_EF128424.1

GTAAATCCATAGAAGCTTCATGCAAGCTTTACCTGAGAAGCATGTCACAATTTAATTTGCTATAATGCTG

GAGCTTTTTTTGCTTATTCAGTTTTAGAATTTCGAGGCACTGTATCTGATGTTAAAGAACACTAAGGAAG

TTTTCTTTCATTCTGCTCCAGGAGGGAGTAACTTTTGCATATATCTAGCTAAAAGATATTGTGCATAGCT

TATACAAAAACACAAGGTTATTGAATTTGCATTATGCCTACCTCCCCCTCCCATGCGGGGTGTCTAGAAC

CTTTCAACTTCATACCTGGATTTGTTTTTTTTTATGGGGAGCTTTTGCCTTTGGAGTTCWTATTTTCTCT

GGATCAGTTAATTATAATCCTGTTATTTGAAGTCTACTTTTGATATCTCTGTTGAAGTTGTTGAGATGTA

AGTTTACCCTGTAGACAGAAACGTGGCTTTGAAGAATGACCTCGGACGAGTGTCTTGTAAGCTTTCTGAT

GATCCTAAACTTGTATAAGTTTCAGGACATACTATTTTGACTAAGGTCTCTCCAAAATTATATTAGTGGA

CTATCTCCTTGTAACTAGCCTGTGACTTAGATTTTCCAATTCCATGATCTATCAGGAGTTCTATCCTAAA

TAATGTTTCTAACATTGATTTTGGATATATTCTGATGTAGGAGTTTAATATGTAATTGTACTTTTGTAAT

TGCCCCA

>Ceroxylon_alpinum_EF128422.1

GTAAATCCATAGAAGCTTCATGCAAGCTTTACCTGAGAAGCATGTCACAATTTAATTTGCTATAATGCTG

GAGCTTTTTTTGCTTATTCAGTTTTAGAATTTCGAGGCACTGTATCTGATGTTAAAGAACACTAAGGAAG

TTTTCTTTCATTCTGCTCCAGGAGGGAGTAACTTTTGCATATATCTAGCTAAAAGATATTGTGCATAGCT

TATACAAAAACACAAGGTTATTGAATTTGCATTATGCCTACCTCCCCCTCCCATGCGGGGTGTCTAGAAC

CTTTCAACTTCATACCTGGATTTGTTTTTTTTTATGGGGAGCTTTTGCCTTTGGAGTTCTTATTTTCTCT

GGATCAGTTAATTATAATCCTGTTATTTGAAGTCTACTTTTGATATCTCTGTTGAAGTTGTTGAGATGTA

AGTTTACCCTGTAGACAGAAACGTGGCTTTGAAGAATGACCTCGGACGAGTGTCTTGTAAGCTTTCTGAT

GATCCTAAACTTGTATAAGTTTCAGGACATACTATTTTGACTAAGGTCTCTTTAAAATTATATTAGTGGA

CTATCTCCTTGTAACTAGCCTGTGACTTAGATTTTCCAATTCCATGATCTATCAGGAGTTCTATCCTAAA

TAATGTTTCTAACATTGATTTTGGATATATTCTGATGTAGGAGTTTAATATGTAATTGTACTTTTGTAAT

TGCCCCA

>Chamaedorea_microspadix_AJ830166.1

CCCATATGGGAAAGGAGGGAGATGCTACTCCTTTTACTGATGTCACAGTAAGTAAATCCATAGAACCTTC

TTGCAAGCTTTACCTGAGAAGCCTGTTGCAATTTAACTTGCTATGCTGGAACTTTTCTGCTTATTCAGTT

TAAGAATTCTGAGGCACTGTATCTGATGTTAAAGAAGATTAAGCTTACTGTTAAGGATTTGTACTAGATA

TGGTTTAATAAATATGTTTGAGAACAGTTACCCCCCACCCCCACCCCCCTTCCCTCTCTGTCTAGAACCT

TTCGACTTCTTACCTGGATTTGATTTTTTTGAGGGGAGATTTTGCCTTTCAAGTTCATTTTTTTCCTCTG

GATTAGTTTAATTATAATACTATTATTTGTTGTCTGCTTTGAATATCTCTTTTGAAGTTCTTGCTGTGTA

AGTTTACCCTGTAGGCAGAAACATGGCTTTGAAGAATGACCTTGGATGAGTGTCTTGTAAGTTATTCTGA

TGATCCTAAACTTGTATTAAGTTTCAAGACATACTATTTTAACTTAGTCTCTCCTAAATTATATAGTAGA

CTATCCCCTTGTAATTGACCTTTGACTTGGATTTTCCAATTCAATGATCTATCAGGAGTTCTATCCTAAA

TAATGTTTCTAGCATTGATTTCAGATATATTCTGATGTAGGAGTTAAATATGTAATTGTACTTTTGTAAT

TGCCCCATTTCAAGAAATGCTTATATTATCAAAAATTTCCTAGGTGGACAATATCAGGGCAAAAGGCCTC

CTTTCAT

>Chamaedorea_pinnatifrons_EF491136.1

CTACTCCTTTTACTGATGTCACAGTAAGTAAATCCATAGAACCTTCTTGCAAGCTTTACCTGAGAAGCCT

GTCGCAATTTAACTTGCTATGCTGGAACTTTTCTGCTTATTCAGTTTAAGAATTCTGAGGCACTGTATCT

GATGTTAAAGAAGATTAAGCTTACTGTTAAGGATTTGTACTAGATATGGTTTAATAAATATGTTTGAGAA

CAGTTAACCCCCCACCCCCACCCCCCTTCCCTCTCTGTCTAGAACCTTTCGACTTCTTACCTGGATTTGA

TTTTTTTGAGGGGTGTTTTTGCCTTTCAAGTTCATTTTTTTCCTCTGGATTAGTTAATTATAATACTATT

ATTTGTTGTCTGCTTTGAATATCTCTTTTGAAGTTCTTGCTGTGCAAGTTTACCCTGTAGACAGAAACAT

GGCTTTGAAGAATGACCTTGGACGAGTGTCTTGTAAGTTATTCTGATGATCCTAAACTTGTATTAAGTTT

CAAGACATACTATTTTAACTTAGTCTCTCCTAAATTATATAGTAGACTATCTCCTTGTAATTGACCTTTG

ACTTGGATTTTCCAATTCAATGATCTATCAGGAGTTCTATCCTAAATAATGTTTCTAACATTGATTTCAG

ATATATTCTGATGTAGGAGTTTAATATGTAATTGTACTTTTGTAATTGCCCCAT

>Chamaedorea_linearis_EF491134.1

CTACTCCTTTTACTGATGTCACAGTAAGTAATTCCATAGAATCTTCTTGCAAGCTTTACCTGAGAAGCCT

GTTGCAATTTAACTTGCTATGTTGGAACTTTTCTGCTTATTCAGTTTAAGAATTCTGAGGCACTGTATCT

GATGTTAAAGAAGATTAAGCTTACTGTTAAGGATTTGTACTAGATATGGTTTAATAAATATGTTTGAGAA

CAGTTACCCCCCACCCCCACCCCCCTTCCCTCTCTGTCTAGAACCTTTCAACTTCTTACCTGGATTTGAT

TTTTTTGAGGGGAGTTTTTGCTTTTCAAGTTCATTTTTTTCCTCTGGATTAGTTAATTATAATACTATTA

TTTGTTGTCTGCTTTGAATATCTCTTTTGAAGTTCTTGCTGTGTAAGTTTACCCTGTAGACAGAAACATG

GCTTTGAAGAATGACCTTGGACGAGTGTCTTGTAAGTTATTCTGATGATCCTAAACTTGTATTAAGTTTC

AAGACATAATATTTTAACTTAGTCTCTTTTAAATTATATAGTAGACTATCTCCTTGTAATTGACCTTTGA

CTTGGATTTTCCAATTCAATGATCTATCAGGAGTTCTATCCTAAATAATGTTTCTAACATTGATTTCAGA

TATATTCTGATGTTGGAGTTTAATATGTAATTGTACTTTTGTAATTGCTCCAT

>Chamaedorea_alternans_EF491132.1

CTACTCCTTTTACTGATGTCACAGTAAGTAAATCCATAGAACCTTCTTGCAAGCTTTACCGGAGAAGCCT

GTCGCAATTTAACTTGCTATGCTGGAACTTTTCTGCTTATTCAGTTTAAGAATTCTGAGGCACTGTATCT

GATGTTAAAGAAGATTAAGCTTACTGTTAAGGATTGTACTAGATATGGTTTAATAAATATGTTTGAGAAC

AGTTGCCCCCCACCCCCACCCCCCTTCCCTCTCTGTCTAGAACCTTTCGACTTCTTACCTGGATTTGATT

TTTTTGAGGGGAGTTTTTGCCTTTCAAGTTCATTTTTTTCCTCTGGATTAGTTAATTATAATACTATTAT

TTGTTGTCTGCTTTGAATATCTCTTTTGAAGTTCTTGTTGTGTAAGTTTACCCTGTAGACAGAAACATGG

CTTTGAAGAATGACCTTGGACGAGTGTCTTGGAAGTTATTCTGATGATCCTAAACTTGTATTAAGTTTCA

AGACATACTATTTTAACTTAATCTCTCCTAAATTATATAGTAGACTATCTCCTTGTAATTGACCTTTGAC

TTGGATTTTCCAATTCAATGATCGATCAGGAGTTCTATCCTAAATAATGTTTCTAACATTGATTTCAGAT

ATATTCTGATGTAGGAGTTTAATATGTAATTGTACTTTTGTAATTGCCCCAT

>Chamaedorea_glaucifolia_EF491130.1

CTACTCCTTTTACTGATGTCACAGTAAGTAAATCCATAGAACCTTCTTGCAAGCTTTACCTGAGAAGCCT

GTCGCAATTTAACTTGCTATGCTGGAACTTTTCTGCTTATTCAGTTTAAGAATTCTGAGGCACTGTATCT

GATGTTAAAGAAGATTAAGCTTACTGTTAAGGATTTGTACTAGATATGGTTTAATAAATATGTTTGAGAA

CAGTTACCCCCCACCCCCACCCCCCTTCCCTCTCTGTCTAGAACCTCTCGACTTCTTACCTGGATTTGAT

TTTTTTGAGGGGAGTTTTTGCCTTTCAAGTTCATTTTTTTCCTCTGGATTAGTTAATTATAATACTATTA

TTTGTTGTCTGCTTTGAATATCTCTTTTGAAGTTCTTGCTGTGTAAGTTTACCCTGTAGACAGAAACATG

GCTTTGAAGAATGACCTTGGACGAGTGTCTTGTAAGTTATTCTGATGATCCTAAACTTGTATTAAGTTTC

AAGACATACTATTTTAACTTAGTCTCTCCTAAATTATATAGTAGACTATCTCCTTGTAATTGACCTTTGA

CTTGGATTTTCCAATTCAATGATCTATCAGGAGTTCTATCCTAAATAATGTTTCTAACATTGATTTCAGA

TATATTCTGATGTAGGAGTTTAATATGTAATTGTACTTTTGTAATTGCCCCAT

>Chamaedorea_pochutlensis_EF491128.1

CTACTCCTTTTACTGATGTCACAGTAAGTAAATCCATAGAACCTTCTTGCAAGCTTTACCTGAGAAGCCT

GTCGCAATTTAACTTGCTATGCTGGAACTTTTCTGCTTATTCAGTTTAAGAATTCTGAGGCACTGTATCT

GATGTTAAAGAAGATTAAGCTTACTGTTAAGGATTTGTACTAGATATGGTTTAATAAATATGTTTGAGAA

CAGTTACCCCCCACCGCCACCCCCCTTCCCTCTCTGTCTAGAACTTTTCGACTTCTTACCTGGATTTGAT

TTTTTTGAGGGGAGTTTTTGCCTTTCAAGTTCATTTTTTTCCTCTGGATTAGTTAATTATAATACTATTA

TTTGTTGTCTGCTTTGAATATCTCTTTTGAAGTTATTGCTGTGTAAGTTTACCCTGTAGACAGAAACATG

GCTTTGAAGAATGACCTTGGACGAGTGTCTTGTAAGTTATTCTGATGATCCTAAACTTGTATTAAGTTTC

AAGACATACTATTTTAACTTAGTCTCTCCTAAATTATATAGTAGACTATCTCCTTGTAATTGACCTTTGA

CTTGGATTTTCCAATTCAATGATCTATCAGGAGTTCTATCCTAAATAATGTTTCTAACATTGATTTCAGA

TATATTCTGATGTAGGAGTTTCATATGTAATTGTACTTTTGTAATTGCCCCAT

>Chamaedorea_pedunculata_EF491126.1

CTACTCCTTTTACTGATGTCACAGTAAGTAAATCCATAGAACCTTCTTGCAAGCTTTACCTGAGAAGCCT

GTCGCAATTTAACTTGCTATGCTGGAACTTTTCTGCTTATTCAGTTTAAGAATTCTGAGGCACTGTATCT

GATGTTAAAGAAGATTAAGCTTACTGTTAAGGATTTGTACTAGATATGGTTTAATAAATATGTTTGAGAA

CAGTTACCCCCACCCCCACCCCCCTTCCCTCTCTGTCTAGAACCTTTCGACTTCTTACCTGGATTTGATT

TTTTTGAGGGGAGTTTTTGCCTTTCAAGTTCATTTTTTTCCTCTGGATTAGTTAATTATAATACTATTAT

TTGTTGTCTGCTTTGAATATCTCTTTTGAAGTTCTTGCTGTGTAAGTTTACCCTGTGGACAGAAACATGG

CTTTGAAGAATGACCTTGGACGAGTGTCTTGTAAGTTATTCTGATGATCCTAAACTTGTATTAAGTTTCA

AGACATACTATTTTAACTTAGTCTCTCCTAAATTATATAGTAGACTATCTCCTTGTAATTGACCTTTGAC

TTGGATTTTCCAATTCAATGATCTCTCAGGAGTTCTATCCTAAATAATGTTTCTAACATTGATTTCAGAT

ATATTCTGATGTAGGAGTTTAATATGTAATTGTACTTTTGTAATTGCCCCAT

>Chamaedorea_seifrizii_EF491124.1

CTACTCCTTTTACTGATGTCACAGTAAGTAAATCCATAGAACCTTCTTGCAAGTTTTACCCGAGAAGCCT

GTCGCAATTTAACTTGCTATGCTGGAACTTTTCTGCTTATTCAGTTTAAGAATTCTGAGGCACTGTATCT

GATGTTAAAGAAGATTAAGCTTACTGTTAAGGATTTGTACTATATATGGTTTAATAAATATGTTTGAGAA

CAGTTACCCCCCACCTCCACCCCCCTTCCTTCTCTGTCTAGAACCTTTCGACTTCTTACCTGGATTTAAT

TTTTTTAAGGGGAGTTTTTGCCTTTCAAGTTCATTTTTTTCTTCTGGATTAGTTAATTATAATACTATTA

TTTGTTGTCTGCTTTGAATATCTCTTTTGAAGTTCTTGATGTGTAAGTTTACCCTGTAGACAGAAACATG

GCTTTGAAGAATGACCTTGGACGAGTGTCTTGTAAGTTATTCTGATGATCCTAAACTTGTATTTCAGTTT

CAAGACATACTATTTTAACTTAGTCTCTCCTAAATTATATAGTAGACTTTCTCCTTGTAATTGACCTTTG

ACTTGGATTTTCCAATTCAATGATCTATCAGGAGTTCTATCCTAAATAATGTTTCTAACATTGATTTCAG

ATATATTCTGATGTTGGAGTTTAATATGTAATTGTACTTTTGTAATTGCTCCAT

>Chamaedorea_stolonifera_EF491122.1

CTACTCCTTTTACCGATGTCACAGTAAGTAAATCCATAGAACCTTCTTGCAAGCTTTACCTGAGAAGCCT

GTCCAAATTTAACTTGCTATGCTGGAACTTTTCTGATTATTCAGTTTAAGAATTCCGAGGCACTGTATCT

GATGTTAAAGAAGATTAAGCTTACTGTTAAGGATTTGTACTAGATATGGTTTAATAAATATGTTTGAGAA

CAGTTACCCCCCAGCGCCACCCCCCTTCCCTCTCTGTCTAGAACCTTTCGACTTCTTACCTGGATTTGAT

TTTTTTGAGGGGAGTTTTTGCCTTTCAAGTTCATTTTTTTCCTCTGGATTAGTTGATTATAATACCATTA

TTTGTTGTCTGCTTTGAATATCTCTTTTGAAGTTCTTGCTGTGTAAGTTTACCCTGTAGACAGAAACATG

TCTTTGAAGAATGACCTTGGACGAGTGTCTTGTAAGTTATTCTGATGATCCTAAACTTGTATTAAGTTTC

AAGACATACTATTTTAACTTAGTCTCTCCTAAATTATATAGTAGACTATCTCCTTGTAATTGACCTTTGA

CTTGGATTTTCCAATTCAATGATCTATCAGGAGTTCTATCCTTAATAATGTTTCTAACATTGATTTCAGA

TATATTCTGATGTAGGAGTTTAATATGTAATTGTACTTTTGTAATTGCCCCAT

>Chamaedorea_adscendens_EF491120.1

CTCTCCTTTTCTGATGTCACAGTAAGTAAATCCATAGAACCTTCTTGCAAGCTTTACCTGAGAAGCCTGT

CGCAATTTAACTTGCTATGCTGGAACTTTTCTGCTTATTCAGNTTAAGAATTCCGAGGCACTGTATCTGA

TGGTAAAGAAGANTAAGCTTACTGKTAAGGATTTGTACTAGATATGGTTTAATAAATANGNTTGAGAACA

GTTACCCCCCCACCCCCCTTCCCTCTCTGTCTAGAACCTTTCGACTTCTTACCTGGATTTGATTTTTTTG

AGGGGAGTTTTTGCCTTTCAAGTTCATTTTTTTCCTCTGGATTAGTTAATTATAATACTATTATTTGTTG

TCTGCTTTGAATATCTCTTTTGAAGTCCTTGCTGTGTAAGTTTACCCTGTAGACAGAAACATGGCTTTGA

AGAATGACCTTGGACGAGTGTCTTGTAAGTTATTCTGATGATCCTAAACTTGTTTTAAGTTTCAAGACAT

ACTATTTTAACTTAGTCTCTCCTAAATTATATGGTAGACTATCTCCTTGTAATTGACCTTTGACTTGGAT

TTTCCAATTCAATGATCTATCAGGAGTTCTATCCTAAATAATGTTTCTAACGTTGATTTCAGATATATTC

TGAAGTATGAGTTTAATATGTAATTGTACTTTCGTAATTGCCCCAT

>Chamaedorea_dammeriana_EF491118.1

CTCTCCTTTTACTGATGTCACAGTAAGTAAATCCATAGAACCTTCTTGCAAGCTTTACCTGAGAAGCCTG

TCGCAATTTAACTTGCTATGCTGGAACTTTTCTGCTTATTCAGTTTAAGAATTCCGAGGCACTGTATCTG

ATGTTAAAGAAGATTAAGCTTACTGTTAAGGATTTGTACTAGATATGGTTTAATAAATATGTTTGTGAAC

AGTTACCCCCCACCCCCACCCCCACCCCCCTTCCCTCTCTGTCTAGAACCTTTCGACTTCTTACCTGGAT

TTGATTTTTTTGAGGGGAGTTTTTTCCTTTCAAGTTCATTTTTTTCCTCTGGATTAGTTAATTATAATAC

TATTATTTGTTGTCTGCTTTGAATATCTCTTTTGAAGTTCTTGCTGTGTAAGTTTACCCTGTAGACAGAA

ACATGGCTTTGAAGAATGACATTGGACGAGTGTCTTGTAAGTTATTCTGATGATCCTAAAATTGTTTTAA

GTTTCAAGACATACTATTTTAACTTAGTCTCTCCTAAATTATATGGTAGACTATCTCCTTGTAATTGACC

TTTGACTTGGATTTTCCAATTCAATGATCTATCAGGAGTTCTATCCTAAATAATGTTTCTAACATTGATT

TCAGATATATTCTGATGTAGGAGTTTAATATGTAATTGTACTTTTGTAATTGCCCCAT

>Chamaedorea_ernesti_augustii_EF491116.1

CTACTCCTTTTACTGATGTCACAGTAAGTAAATCCATAGAACCTTCTTGCAAGCTTTACCTGAGAAGCCC

GTGTCAATTTAACTTGCTATGCGGGAACTTTTCTGCTTATTCAGTTTAAGAATTCTGAGGCGCCGTATCT

GATGTTAAAGAAGATTAAGCTTACTGTTAAGGATTTGTNCTATATATGGTTTAATAAATATGTTTGAGAA

CAGTTACCCCCCAGCGCCACCCCCCTTCCCTCTCTGTCTAGAACCTTTCGACTTCTTACCTGGATTTGAT

TTTTTTGAGGGGAGTTTTTGCCTTTCAAGTTCATTTTTTTCCTCTGGATTAGTTAATTATAATACTATTA

TTTGTTGTCTGCTTTGAATATCTCTTTTGAAGTTCTTGCTGTGTAAGTCTACCCTGTAGACAGAAACATG

GCTTTGAAGAATGACCTTGGACGAGTGTCTTGTAAGTTATTCTGATGATCCTAAACTTGTATTAAGTTTC

AAGACATACTATTTTAACTTAGTCTCTCCTAAATTATATAGTAGACTATCTCCTTGTAATTGACCTTTGA

CTTGGATTTTCCAATTCAATGATCTATCAGGAGTTCTATCCTTAATAATGTTTCTAACATTGATTTCAGA

TATATTCTGATGTAGGAGTTTAATATGTAATTGTACTTTTGTAATTGCCCCAT

>Chamaedorea_oblongata_EF491114.1

CTACTCCTTTTACTGATGTCACAGTAAGTAAATCCATAGAACCTTCTTGCAAGCTTTACCTGAGAAGCCT

GTCGCAATTTAACTTGCTATGCTGGAACTTTTCTGCTTATTCAGTTTAAGAATTCTGAGGCACTGTATCT

GATGTTARAGAAGATTAAGCTTACTGTTAAGGATTTGTACTAGATATGGTTTAATAAATATGTTTGAGAA

CAGTTACCCCCCACCCCCACCCCCCTTCCCTCTCTGTCTAGAACCTTTCGACTTCTTACCTGGATTTGAT

TTTTTTGAGGGGAGTTTTTGCCTTTCAAGTTCATTTTTTTCCTCTGGATTAGTTAATTATAATACTATTA

TTTGTTGTCTGCTTTGAATATCTCTTTTGAAGTTCTTGCTGTGTAAGTTTACCCTGTAGACAGAAACATG

GCTTTGAAGAATGACCTTGGACGAGTGTCTTGTAAGTTATTCTGATGATCCTAAACTTGTATTAAGTTTC

AAGACATACTATTTTAACTTAGTCTCTCCTAAATTATATAGTAGACTATSTCCTTGTAATTGACCTTTGA

CTTGGATTTTCCAATTCAATGATCTATCAGGAGTTCTATCCTAAATAATGTTTCTAACATTGATTTCAGA

TATATTCTGATGTAGGAGTTTAATATGTAATTGTACTTTTGTAATTGCCCCAT

>Chamaedorea_tepejilote_EF491137.1

CTACTCCTTTTACTGATGTCACAGTAAGTAAATCCATAGAACCTTCTTGCAAGCTTTACCGGAGAAGCCT

GTCGCAATTTAACTTGCTATGCTGGAACTTTTCTGCTTATTCAGTTTAATAATTCTGAGGCACTGTATCT

GATGTTAAAGAAGATTAAGCTTACTGTTAAGGATTGTACTAGATATGGTTTAATAAATATGTTTGAGAAC

AGTTGCCCCCCACCCCCACCCCCCTTCCCTCTCTGTCTAGAACCTTTCGACTTCTTACCTGGATTTGATT

TTTTTGAGGGGAGTTTTTGCCTTTCAAGTTCATTTTTTTCCTCTGGATTAGTTAATTATAATACTATTAT

TTGTTGTCTGCTTTGAATATCTCTTTTGAAGTTCTTGCTGTGTAAGTTTACCCTGTAGACAGAAACATGG

CTTTGAAGAATGACCTTGGACGAGTGTCTTGTAAGTTATTCTGATGATCCTAAACTTGTATTAAGTTTCA

AGACATATTATTTTAACTTAATCTCTCCTAAATTATATAGTAGACTATCTCCTTGTAATTGACCTTTGAC

TTGGATTTTCCAATTCAATGATCTATCAGGAGTTCTATCCTAAATAATGTTTCTAACATTGATTTCAGAT

ATATTATGATGTAGGAGTTTAATATGTAACTGTACTTTTGTAATTGCCCCAT

>Chamaedorea_metallica_EF491135.1

CTACTCCTTTTACTGATGTCACAGTAAGTAAATCCATAGAACCTTCTTGCAAGCTTGACCTGAGAAGCTT

GTCGCAATTTAACTTGCTATGCTGGAACTTTTCTGATTATTCAGTTTAAGAATTCTGAGGCACTGTATCT

GGTGTAAAAGATGATTAAGCTTACTGTTAAGGATTTGTACTAGATATGGTTTAATAAATATGTTTGAGAA

CAGTTACCCCCCAGCGCCACCCCCCTTCCCTCTCTGTCTAGAACCTTTCGACTTCTTACCTGGATTTGAT

TTTTTTGAGGGGAGTTTTTGCCTTTCAAGTTCATTTTTTTCCTCTGGATTAGTTAATTATAATACTATTA

TTTGTTGTCTGCTTTGAATATCTCTTTTGAAGTTCTTGCTGTGTAAGTTTACCCTGTAGACAGAAACATG

GCTTTGAAGAATGACCTTGGACGAGTGTCTTGTAAGTTATTCTGATGATCCTAAACTTGTATTAAGTTTC

AAGACATACTATTTTAACTTAGTCTCTCCTAAATTATATAGTAGACTATCGACCTTTGACTTGGATTTTC

CAATTCAATGATCTGTCAGGAGTTCTATCCTTAATAATGTTTCTAACATTGATTTCAGATATATTCTGAT

GTAGGAGTTTAATATGTAATTGTACTTTTGTAATTGCCCCAT

>Chamaedorea_elatior_EF491133.1

CTACTCCTTTTACTGATGTCACAGTAAGTAAATCCATAGAACCTTCTTGCAAGCTTTACCTGAGAAGCCT

GTCGCAATTTAACTTGCTATGCTGGAACTTTTCTGCTTATTCAGTTTAAGAATTCTGAGGCACTGTATCT

GATGTTAAAGAAGATTAAGCTTACTGTTAAGGATTTGTACTAGATATGGTTTAATAAATATGTTTGASAA

CAGTTACCCCCCACCCCCACCCCCCTTCCCTCTCTGTCTAGAACCTTTCGACTTCTTACCTGGATTTGAT

TTTTTTGAGGGGAGTTTTTGCCTTTCAAGTTCATTTTTTTCCTCTGGATTAGTTAATTATAATACTATTA

TTTGTTGTCTGCTTTGAATATCTCTTTTGAAGTTCTTGCTGTGTAAGTTTACCCTGTAGACAGAAACATG

GCTTTGAAGAATGACCTTGGACGAGTGTCTTGTAAGTTATTCTGATGATCCTAAACTTGTATTAAGTTTC

AAGACATACTATTTTAACTTAGTCTCTCCTAAATTATATAGTAGACTATCTCCTTGTAATTGACCTTTGA

CTTGGATTTTCCAATTCAATGATCCATCAGGAGTTCTATCCTAAATAATGTTTCTAACATTGATTTCAGA

TATATTCTGATGTAGGAGTTTAATATGTAATTGTACTTTTGTAATTGCCCCAT

>Chamaedorea_costaricana_EF491131.1

CTACTCCTTTTACTGATGTCACAGTAAGTAAATCCATAGAACCTTCTTGCAAGCTTTACCTGAGAAGCCT

GTCGCAATTTAACTTGCTATGCTGGAACTTTTCTGCTTATTCAGTTTAAGAATTCTGAGGCACTGTATCT

GATGTTAAAGAAGATTAAGCTTACTGTTAAGGATTTGTACTAGATATGGTTTAATAAATATGTTTGAGAA

CAGTTACCCCCCACCCCCACCCCCCTTCCCTCTCTGTCTAGAACCTTTCGACTTCTTACCTGGATTTGAT

TTTTTTGAGGGGAGTTTTTGCCTTTCAAGTTCATTTTTTTCCTCTGGATTAGTTAATTATAATACTATTA

TTTGTTGTCTGCTTTGAATATCTCTTTTGAAGTTCTTGCTGTGTAAGTTTACCCTGTAGACAGAAACATG

GCTTTGAAGAATGACCTTGGACGAGTGTCTTGTAAGTTATTCTGATGATCCTAAACTTGTTTTAAGTTTC

AAGACATACTATTTTAACTTAGTCTCTCCTAAATTATATGGTAGACTATCTCCTTGTAATTGACCTTTGA

CTTGGATTTTCCAATTCAATGATCTATCAGGAGTTCTATCCTAAATAATGTTTCTAACATTGATTTCAGA

TATATTCTGATGTAGGAGTTTAATATGTAATTGTACTTTTGTAATTGCCCCAT

>Chamaedorea_elegans_EF491129.1

CTACTCCTTTTACTGATGTCACAGTAAGTAAATCCATAGAACCTTCTTGCAAGCTTTACCTGAGAAGTCT

GTCGCAATTTAACTTGTTATGTTGGAACTTTTCTGCTCATTCAGTTTAAGAATTCTGAGGCACTGTATCT

GATGTTAAAGAAGATTAAGCTTACTGTTAAGGATTTGTACTAGATATGGTTTAATAAATATGTTTGAGAA

CAGTTACCCCCCACCCCCACCCCCACCCCCACCCCCCTTCCCTCTCTGTCTAGAACCTTTCGGCTTCTTA

CCTGGATTTGATTTTTTTGAGGGGAGTTTTTGCCTTTCAAGTTCATTTTTTTCCTCTGGATTAGTTAATT

ATAATACTATTATTTGTTGTCTGCTTTGAATRTCTCTTTTGAAGTTCTTGCTGTGTAAGTTTACCCTGTA

GACAGAAACATGGCTTTGAAGAATGACKTTGGACGAGTGTCTTGTAAGTTATTCTGATGATCCTAAACTT

GTATTAAGTTTCAAGACATACTATTTTAACTTAGTCTCTCCTAAATTATATGGTAGACTATCTCCTTGTA

ATTGACCTTTGACTTGGATTTTCCAATTCAATGATCTATCAGGAGTTCTATCCTAAATAATGTTTCTAAC

ATTGATTTCAGATATATTCTGATGTAGGAGTTTAATATGTAATTGTACTTTTGTAATTGCCCCAT

>Chamaedorea_fragrans_EF491127.1

CTACTCCTTTTACTGATGTCACAGTAAGTAAATCCATAGAACCTTCTTGCAAGCTTTACCTGAGAAGCCT

GTTGCAATTTAACTTGCTATGCTGGAACTTTTCTGCTTATTCAGTTTAAGAATTCTGAGGCACTGTATCT

GATGTTAAAGAAGATTAAGCTTACTGTTAAGGATTTGTACTAGATATGGTTTAATAAATATGTTTGAGAA

CAGTTACCCCCCACCCCCACCCCCCTTCCTTCTCTGTCTAGAACCTTTCGACTTCTTACCTGGATTTGAT

TTTTTTGAGGGGAGTTTTTGCTTTTCAAGTTCATTTTTTTCCTCTGGATTAGTTAATTATAATACTATTA

TTTGTTGTCTGCTTTGAATATCTCTTTTGAAGTTCTTGCTGTGTAAGTTTACCCTGTAGACAGAAACATG

GCTTTGAAGAATGACCTTGGACGAGTGTCTTGTAAGTTATTCTGATGATCCTAAACTTGTATTAAGTTTC

AAGACATACTATTTTAACTTAGTCTCTTTTAAATTATATAGTAGACTATCTCCTTGTAATTGACCTTTGA

CTTGGATTTTGCAATTCAATGATCTATCAGTTCTATCCTAAATAATGTTTCTAACATTGATTTCAGATAT

ATTCTGATGTTGGAGTTTAATATGTAATTGTACTTTTGTAATTGCTCCAT

>Chamaedorea_brachypoda_EF491125.1

CTACTCCTTTTACTGATGTCACAGTAAGTAAATCCATAGAACCTTCTTGCAAGCTTTACCTGAGAAGCCT

GTCGCAATTTAACTTGCTATGCTGGAACTTTTCTGCTTATTCAGTTTAAGAATTCTGAGGCACTGTATCT

GATGTTAAAGAAGATTAAGCTTACTGTTAAGGATTTGTACTATATATGGTTTAATAAATATGTTTGAGAA

CAGTTACCCCCCACCTCCACCCCCCTTCCTTCTCTGTCTAGAACCTTTCGACTTCTTACCTGGATTTAAT

TTTTTTAAGGGGAGTTTTTGCCTTTCAAGTTCATTTTTTTCTTCTGGATTAGTTAATTATAATACTATTA

TTTGTTGTCTGCTTTGAATCTCTCTTTTGAAGTTCTTGCTGTGTAAGTTTACCCTGTAGACAGAAACATG

GCTTTGAAGAATGACCTTGGACGAGTGTCTTGTAAGTTATTCTGATGATCGTAAACTTGTATTAAGTTTC

AAGACATACTATTTTAACTTTGTCTCTCCTAAATTATATAGCAGACTATGTCCTTGTAATTGACCTTTGA

CTTGGATTTTCCAATTCAATGATCTATCAGGAGTTCTATCCTAAATAATGTTTCTAACATTGATTTCAGA

TATATTCTGATGTTGGAGTTTAATATGTAATTGTACTTTTGTAATTTCTCCAT

>Chamaedorea_nationsiana_EF491123.1

CTACTCCTTTTACTGATGTCACAGTAAGTAAATCCATAGAACCTTCTTGCAAGCTTTACCGGAGAAGCCT

GTCACAATTTAACTTGCTATGCTGGAACTTTTCTGCTTATTCAGTTTAAGAATTCTGAGGCACTGTATCT

GATGTTAAAGAAGATTTAGCTTACTGTTAAGGATTGTACTAGATATGGTTTAATAAATATGTTTGAGAAC

AGTTGCCCCCCACCCCACCCCCCTTCCCTCTCTGTCTAGAACCTTTCGACTTCTTACCTGGATTTGATTT

TTTTGAGGGGAGTTTTTGCCTTTCAAGTTCATTTTTTTCCTCTGGATTAGTTAATTATAATACTATTATT

TGTTGTCTGCTTTGAATATCTCTTTTGAAGTTCTTGCTGTGTAAGTTTACCCTGTAGACAGAAACATGGC

TTTGAAGAATGACCTTGGACGAGTGTCTTGTAAGTTATTCTGATGATCCTAAACTTGTATTAAGTTTCAA

GACATACTATTTTAACTTAATCTCTCCTAAATTATATAGTAGACTATCTCCTTGTAATTGAACTTTGACT

TGGATTTTCCAATTCAATGATCTATCAGGAGTTCTATCCTAAATAATGTTTCTAACATTGATTTCAGATA

TAATCTGATGTAGGAGTTTAATATGTAATTGTACTTTTGTAATTGCCCCAT

>Chamaedorea_hooperiana_EF491121.1

CTACTCCTTTTACTGATGTCACAGTAAGTAAATCCATAGAACCTTCTTGCAAGCTTTACCTGAGAAGCCT

GTCGCAATTTAACTTGCTATGCTGGAACTTTTCTGCTTATTCAGTTTAGGAATTCTGAGGCACTGTATCT

GATGTTAAAGAAGATTAAGCTTACTGTTAAGGATTTGTACTAGATATGGTTTAATAAATATGTTTGAGAA

CAGTTTCCCCCCACCCCCACCCCCCTTCCCTCTCTGTTTAGAACCTTTCGACTTCTTACCTGGATTTGAT

TTTTTTGAGGGGAGTTTTTGCCTTTCAAGTTCATTTTTTTCCTCTGGATTAGTTAATTATAATACTATTA

TTTGTTGTCTGCTTTGAATATCTCTTTTGAAGTTCTTGCTGTGTAAGTTTACCCTGTAGACAGAAACATG

GCTTTGAAGAATGACCTTGGACGAGTGTCTTGTAAGTTATTCTGATGATCCTAAACTTGTATTAAGTTTC

AAGACATACTATTTTAACTTAGTCTCTCCTAAATTATATAGTAGACTATCTCCTTGTAATTGACCTTTGA

CTTGGATTTTCCAATTCAATGATCTATCAGGAGTTCTATCCTAAATAATGTTTCTAACATTGATTTCAGA

TATATTCTGATGTAGGAGTTTAATATGTAATTGTACTTTTGTAATTGCCCCAT

>Chamaedorea_crucensis_EF491119.1

TTACTGATGTCACAGTAAGTAAATCCATAGAACCTTCTTGCAAGCTTTACCGGAGAAGCCTGTCGCAATT

TAACTTGCTATGCTGGAACTTTTCTGCTTATTCAGTTTAAGAATTCTAAGGCACTGTATCTGATGTTAAA

GAAGATTAAGCTTACTGTTAAGGATTGTACTAGATATGGTTTAATAAATATGTTTGAGAACAGTTGCCCC

CCCACCCCCACCCCCCTTCCCTCTCTGTCTAGAACCTTTCGACTTCTTGCCTGGATTTGATTTTTTTGAG

GGGAGTTTTTGCCTTTCAAGTTCATTTTTTTCCTCTGGATTAGTTAATTATAATACTATTATTTGTTGTC

TGCTTTGAATATCTCTTTTGAAGTTCTTGCTGTGTAAGTTTACCCTGTAGACAGAAACATGGCTTTGAAG

AATGACCTTGGACGAGTGTCTTGTAAGTTATTCTGATGATCCTAAACTTGTATTAAGTTTCAAGACATAC

TATTTTAACTTAATCTCTCCTAAATTATATAGTAGACTATCTCCTTGTAATTGACCTTTGACTTGGATTT

TCCAATTCAATGATCTATCAGGAGTTCTATCCTAAATAATGTTTCTAACATTGATTTCAGATATATTCTG

ATGTAGGAGTTTAATATGTAATTGTACTTTTGTAATTGCCCAT

>Chamaedorea_geonomiformis_EF491117.1

CTACTCCTTTTACTGATGTCACAGTAAGTAAATCCTTAGAAACTTCTTGCAAGCTTTACCTGAGAAGCCT

GTCGCAATTTAACTTGCTATGCTGTTACTTTTCTGCTTATTCAGTTTAAGAATCCTGAGGCACTGTATCT

GATGTTAAAGAAGATTAAGCTTACTGTTAAGGATTTRTACTAGATATGGTTTAATAAATATGTTGAGAAC

AGTTACCCCCCACCCCCGCCCCCATCCCCCTTCCCTCTCTGTCTAGAACCTTTCGACTTCTTACCTGGAT

TTGATTTTTTTGAGGGGAGTTTTTGCCTTTCAAGTTCATTTTTTTCCTCTGGATTAGTTAATTATAATAC

TATTATTTGTTGTCTGCTTTGAATATCTCTTTTGAAGTTCTTGCTGTGTAAGTTTACCCTGTAGACAGAA

ACATGGCTTTGAAGAATGACCTTGGACGAGTGTCTTGTAAGTTATTCTGATGATCCTAAACTTGTATTAA

GTTTCAAGACATACTATTTTAACTTAGTCTCTCCTAAATTATATAGTAGACTATCTCCTTGTAATTGACC

TTTGACTTGGATTTTCCAATTCAATGATCTATCAGGAGTTCTATCCTAAATAATGTTTCTAACATTGATT

TCAGATATATTCTGATGTAGGAGTTTAATATGTAATTGTACTTTTGTAATTGCCCCAT

>Chamaedorea_sartorii_EF491115.1

CTACTCCTTTTACTGATGTCACAGTAAGTAAATCCATAGAACCTTCTTGCAAGCTACCTGAGAAGCCTGT

CGCAATTTAACTTGCTATGCTGGAACTTTTCTGCTTATTCAGTTTAAGAATTCTGAGGCACCGTATCTGA

TGTTAAAGAAGATTAAGCTTACTGTTAAGGATTTGTACTAGATATGGTTTAATAAATATGTTTGAGAACA

GTTAACCCCCCAGCGCCACCCCCCTTCCCTCTCTGTCTAGAACCTTTCGACTTCTTACCTGGATTTGATT

TTTTTGAGGGGAGTTTTTGCCTTTCAAGTTCATTTTTTCCCTCTGGATTAGTTAATTATAATACTATTAT

TTGTTGTCTGCTTTGAATATCTCTTTTGAAGTTCTTGCTGTGTAAGTTTACCCTGTAGACAGAAACATGG

CTTTGAAGAATGACCTTGGACGAGTGTCTTGTAAGTTATTCTGATGATCCTAAACTTGTATTAAGTTTCA

AGACATACTATTTTAACTTAGTCTCTCCTAAATTATATAGTAGACTATCTCCTTGTAATTGACCTTTGAC

TTGGATTTTCCAATTCAATGATCTATCAGGAGTTCTATCCTTAATAATGTTTCTAACATTGATTTCAGAT

ATATTCTGATGTAGGAGTTTAATATGTAATTGTACTTTTGTAATTGCCCCAT

>Chamaedorea_anemophila_DQ177755.1

CTACTCCTTTTACTGATGTCACAGTAAGTAAATCCATAGAACCTTCTTGCAAGCTTTACCTGAGAAGCCT

GTCGCAATTTAACTTGCTATGCTGGAACTTTTCTGCTTATTCAGTTTAAGAATTCTGAGGCACTGTATCT

GATGTTAAAGAAGATTAAGCTTACTGTTAAGGATTTGTACTAGATATGGTTTAATAAATATGTTTGAGAA

CAGTTAACCCCCCACCCCCACCCCCCTTCCCTCTCTGTCTAGAACCTTTCGACTTCTTACCTGGATTTGA

TTTTTTTTAGGGGAGTTTTTGCCTTTCAAGTTCATTTTTTCCCTCTGGATTAGTTAATTATAATACTATT

ATTTGTTGTCTGCTTTGAATATCTCTTTTGAAGTTCTTGCTGTGCAAGTTTACCCTGTAGACAGAAACAT

GGCTTTGAAGAATGACCTTGGACGAGTGTCTTGTAAGTTATTCTGATGATCCTAAACTTGTTTTAAGTTT

CAAGACATACTATTTTAACTTAGTCTCTCCTAAATTATATGGTAGACTATCTCCTTGTAATTGACCTTTG

ACTTGGATTTTCCAATTCAATGATCTATCAGGGGTTCTATCCTAAATAATGTTTCTAACATTGATTTCAG

ATATATTCTGATGTAGGAGTTTAATATGTAATTGTACTTTGTAATTGCCCCATTT

>Chamaedorea_vulgata_DQ177753.1

CTACTCCTTTTACTGATGTCACAGTAAGTAAATCCATAGAACCTTCTTGCAAGCTTTACCTGAGAAGCCT

GTCGCAATTTAACTTGCTATGCTGGAACTTTTCTGCTTATTCAGTTTAAGAATTCTGAGGCACTGTATCT

GATGTTAAAGAAGATTAAGCTTACTGTTAAGGATTTGTACTAGATATGGTTTAATAAATATGTTTGAGAA

CAGTTACCCCCCACCCCCACCCCCCTTCCCTCTCTGTCTAGAACCTTTCGACTTCTTACCTGGATTTGAT

TTTTTTGAGGGGAGTTTTTGCCTTTCAAGTTCATTTTTTTCCTCTGGATTAGTTAATTATAATACTATTA

TTTGTTGTCTGCTTTGAATATCTCTTTTGAAGTTCTTGCTGTGTAAGTTTACCCTGTAGACAGAAACATG

GCTTTGAAGAATGACCTTGGACGAGTGTCTTGTAAGTTATTCTGATGATCCTAAACTTGTTTTAAGTTTC

AAGACATACTATTTTAACTTAGTTTCTCCTAAATTATATAGTAGACTATCTCCTTGTAATTGACCTTTGA

CTTGGATTTTCCAATTCAATGATCTATCAGGAGTTCTATCCTAAATAATGTTTCTAACATTGATTTCAGA

TATATTCTGATGTAGGAGTTTAATATGTAATTGTACTTTTGTAATTGCCCCATTT

>Chamaedorea_rossteniorum_DQ177751.1

CTACTCCTTTTACTGATGTCACAGTAAGTAAATCCATAGAACCTTCTTGCAAGCTTTACCTGAGAAGCCT

GTCGCAATTTAACTTGCTATGCTGGAACTTTTCTGCTTATTCAGTTTAAGAATTCTGAGGCACTGTATCT

GATGTTAAAGAAGATTAAGCTTACTGTTAAGGATTTGTACTAGATATGGTTTAATAAATATGTTTGAGAA

CAGTTACCCCCCACCCCCACCCCCCTTCACTCTCTGTCTAGAACCTTTCGACTTCTTACCTGGATTTGAT

TTTTTTGAGGGGAGTTTTTGCCTTTCAAGTTCATTTTTTTCCTCTGGATTAGTTAATTATAATACTATTA

TTTGTTGTCTGCTTTGAATATCTCTTTTGAAGTTCTTGCTGTGTAAGTTTACCTTGTAGACAGAAACATG

GCTTTGAAGAATGACCTTGGACGAGTGTCTTGTAAGTTATTCTGATGATCCTAAACTTGTATTAAGTTTC

AAGACATACTATTTTAACTTAGTCTCTCCTAAATTATATAGTAGACTATCTCCTTGTAATTGACCTTTGA

CTTGGATTTTCCAATTCAATGATCTATCAGGAGTTCTATCCTAAATAATGTTTCTAACATTGATTTCAGA

TATATTCTGATGTAGGAGTTTAATATGTAATTGTACTTTTGTAATTGCCCCATTT

>Chamaedorea_parvisecta_DQ177749.1

CTACTCCTTTTACTGATGTCACAGTAAGTAAATCCATAGAACCTTCTTGCAAGCTTTACCTGAGAAGCCT

GTCGCAATTTAACTTGCTATGCTGGAACTTTTCTGCTTATTCAGTTTAAGAATTCTGAGGCACTGTGTCT

GATGTTAAAGAAGATTAAGCTTACTGTTAAGGATTTGTACTAGATATGGTTTAATAAATATGTTTGAGAA

CAGTTACCCCCCACCCCCACCCCCCTTCCCTCTCTGTCTAGAACCTTTCGACTTCTTACCTGGATTTGAT

TTTTTTGAGGGGAGTTTTTGCCTTTCAAGTTCATTTTTTTCCTCTGGATTAGTTAATTATAATACTATTA

TTTGTTGTCTGCTTTGAATATCTCTTTTGAAGTTCTTGCTGTGTAAGTTTACCCTGTAGACAGAAACATG

GCTTTGGAGAATGACCTTGGACGAGTGTCTTGTAAGTTATTCTGATGATCCTAAACTTGTTTTAAGTTTC

AAGACATACTATTTTAACTTAGTCTCTCCTAAATTATATGGTAGACTATCTCCTTGTAATTGACCTTTGA

CTTGGATTTTCCAATTCAATGATCTATCAGGAGTTCTATCCTAAATAATGTTTCTAACATTGATTTCAGA

TATATTCTGATGTAGGAGATTAATATGTAATTGTACTTTTGTAATTGCCCCATTT

>Chamaedorea_frondosa_DQ177747.1

CTACTCCTTTTACTGATGTCACAGTAAGTAAATCCATAGAACCTTCTTGCAAGCTTTACCTGAGAAGCCT

GTCGCAATTTAACTTGCTATGCTGGAACTTTTCTGCTTATTCAGTTTAAGAATTCTGAGGCACTGTATCT

GATGTTAAAGAAGATTAAGCTTACTGTTAAGGATTTGTACTAGATATGGTTTAATAAATATGTTTGAGAA

CAGTTACCCCCCACCCCCACCCCCACCCCCACCCCCCTTCCCTCTCTGTCTAGAACCTTTCGACTTCTTA

CCTGGATTTGATTTTTTTGAGGGGAGTTTTTGCCTTTCAAGTTCATTTTTTTCCTCTGGATTAGTTAATT

ATAATACTATTATTTGTTGTCTGCTTTGAATATCTCTTTTGAAGTTCTTGCTGTGTAAGTTTACCCTGTA

GACAGAAACATGGCTTTGAAGAATGACCTTGGACGAGTGTCTTGTAAGTTATTCTGATGATCCTAAACTT

GTATTAAGTTTCAAGACATACTATTTTAACTTAGTCTCTCCTAAATTATATAGTAGACTATCTCCTTGTA

ATTGACCTTTGACTTGGATTTTCCAATTCAATGATCTATCAGGAGTTCTATCCTAAATAATGTTTCTAAC

ATTGATTTCAGATATATTCTGATGTAGGAGTTTAATATGTAATTGTACTTTTGTAATTGCCCCATTT

>Chamaedorea_nubium_DQ177745.1

CTACTCCTTTTACTGATGTCACAGTAAGTAAATCCATAGAACCTTCTTGCAAGCTTTACCTGAGAAGCCT

GTCGCAATTTAACTTGCTATGCTGGAACTTTTCTGCTCATTCAGTTTAAGAATTCTGAGGCACTGTATCT

GATGTTAAAGAAGATTAAGCTTACTGTTAAGGATTTGTACTAGATATGGTTTAATAAATATGTTTGAGAA

CAGTTACCCCCCACCCCCACCCCCCTTCCCTCTCTGTCTAGAACCTTTCGACTTCTTACCTGGATTTGAT

TTTTTTGAGGGGAGTTTTTGCCTTTCAAGTTCATTTTTTYCCTCTGGATTAGTTAATTATAATACTATTA

TATGTTGTCTGCTTTGAATATCTCTTTTGAAGTTCTTGCTGTGTAAGTTTACCCTGTAGACAGAAACATG

GCTTTGAAGAATGACCTTGGACGAGTGTCTTGTAAGTTATTCTGATGRTCCTAAACTTGTATTAAGTTTC

AAGACATACTATTTWAACTTAGTCTCTCCTAAATTATATAGTAGACTATCTCCTTGTAATTGACCTTTGA

CTTGGATTTTCCAATTCAATGATCTATCAGGAGTTCTATCCTAAATAATGTTTCTAACATTGATTTCAGA

TATATTCTGATGTAGGAGTTTAATATGTAATTGTACTTTTGTAATTGCCCCATTT

>Chamaedorea_ibarrae_DQ177743.1

CTACTCCTTTTACTGATGTCACAGTAAGTAAATCCATAGAACCTTCTTGTAAGCTTTACCTGAGAAGCCT

GTCGCAATTTAACTTGCTATGCTGGAACTTTTCTGCTTATTCAGTTTAAGAATTCTGAGGCACTGTATCT

GATGTTAAAGAAGATTAAGCTTACTGTTAAGGATTTGTACTAGATATGGTTTAATAAATATGTTTGAGAA

CAGTTACCCCCCACCCCCCCACCCCTTCCCTCTCTGTCTAGAACCTTTCGACTTCTTACCTGGATTTGAT

TTTTTTGAGGGGAGTTTTTGCCTTTCAAGTTCATTTTTTTCCTCTGGATTAGTTAATTATAATACTATTA

TTTGTTGTCTGCTTTGAATATCTCTTTTGAAGTTCTTGCTGTGTAAGTTTACCCTGTAGACAGAAACATG

GCTTTGAAGAATGACCTTGGACGAGTGTCTTGTAAGTTATTCTGATGATCCTAAACTTGTATTAAGTTTC

AAGACATACTATTTTAACTTAGTCTCTCCTAAATTATATAGTAGACTATCTCCTTGTAATTGACCTTTGA

CTTGGATTTTCCAATTCAATGATCTATCAGGAGTTCTATCCTAAATAATGTTTCTAACATTGATTTCAGA

TATATTCTGATGTAGGAGTTTAATATGTAATTGTACTTTTGTAATTGCCCCATTT

>Chamaedorea_brachyclada_DQ177741.1

CTACTCCTTTTACTGATGTCACAGTAAGTAAATCCATAGAACCTTCTTGCAAGCTTTACCTGAGAAGCCT

GTCGCAATTTAACTTGCTATGCTGGAACTTTTCTGCTTATTCAGTTTAAGAATTCTGAGGCACTGTATCT

GATGTTAAAGAAGATTAAGCTTACTGTTAAGGATTTGTACTAGATATGGTTTAATAAATATGTTTGAGAA

CAGTTATCCCCCACCCCCTTCCCTCTCTGTCTAGAACCTTTCGACTTCTTACCTGGATTTGATTTTTTTG

AGGGGAGTTTTTGCCTTTCAAGTTCATTTTTTTCCTCTGGATTAGTTAATTATAATACTATTATTTGTTG

TCTGCTTTGAATATCTCTTTTGAAGTTCTTGCTGTGTAAGTTTACCCTGTAGACAGAAACATGGCTTTGA

AGAATGACCTTGGACGAGTGTCTTGTAAGTTATTCTGATGATCCTAAACTTGTTTTAAGTTTCAAGACAT

ACTATTTTAACTTAGTCTCTCCTAAATTATATGGTAGACTATCTCCTTGTAATTGACCTTTGACTTGGAT

TTTCCAATTCAATGATCTATCAGGAGTTCTATCCTAAATAATGTTTCTAACATTGATTTCAGATATATTC

TGATGTAGGAGTTTAATATGTAATTGTACTTTTGTAATTGCCCCATTT

>Chamaedorea_pumila_DQ177739.1

CTACTCCTTTTACTGATGTCACAGTAAGTAAATCCATAGAACCTTCTTGCAAGCTTTACCTGAGAAGCCT

GTCGCAATTTAACTTGCTATGCTGGAACTTTTCTGCTTATTCAGTTTAAGAATTCTGAGGCACTGTATCT

GATGTTAAAGAAGATTAAGCTTACTGTTAAGGATTTGTACTAGATATGGTTTAATAAATATGTTTGAGAA

CAGTTATCCCCCACCCCCACCCCCCTTCCCTCTCTGTCTAGAACCTTTCGACTTCTTACCTGGATTTGAT

TTTTTTGAGGGGAGTTTTTGCCTTTCAAGTTCATTTTTTTCCTCTGGATTAGTTAATTATAATACTATTA

TTTGTTGTCTGCTTTGAATATCTCTTTTGAAGTTCTTGCTGTGTAAGTTTAACCTGTAGACAGAAACATG

GCTTTGAAGAATGACCTTGGACGAGTGTCTTGTAAGTTATTCTGATGATCCTAAACTTGTTTTAAGTTTC

AAGACATACTATTTTAACTTAGTCTCTCCTAAATTATATGGTAGACTATCTCCTTGTAATTGACCTTTGA

CTTGGATTTTCCAATTCAATGATCTATCAGGAGTTCTATCCTAAATAATGTTTCTAACATTGATTTCAGA

TATATTCTGATGTAGGAGTTTAATATGTAATTGTACTTTTGTAATTGCCCCATTT

>Chamaedorea_woodsoniana_DQ177737.1

CTACTCCTTTTACTGATGTCACAGTAAGTAAATCCATAGAACCTTCTTGCAAGCTTTACCTGAGAAGCCT

GTCGCAATTTAACTTGCTATGCTGGAACTTTTCTGCTTATTCAGTTTAAGAATTCTGAGGCACTGTATCT

GATGTTAAAGAAGATTAAGCTTACTGTTAAGGATTTGTACTAGATATGGTTTAATAAATATGTTTGAGAA

CAGTTACCCCCCACCCCCACCCCCCTTCCCTCTCTGTCTAGAACCTTTGGACTTCTTACCTGGATTTGAT

TTTTTTGAGGGGAGTTTTTGCCTTTCAAGTTCATTTTTTTCCTCTGGATTAGTTAATTATAATACTATTA

TTTGTTGTCTGCTTTGAATATCTCTTTTGAAGTTCTTGCTGTGTAAGTTTACCCTGTAGACAGAAACATG

GCTTTGAAGAATGACCTTGGACGAGTGTCTTGTAAGTTATTCTGATGATCCTAAACTTGTATTAAGTTTC

AAGACATACTATTTTAACTTAGTCTCTCCTAAATTATATAGTAGACTATCTCCTTGTAATTGACCTTTGA

CTTGGATTTTCCAATTCAATGATCTATCAGGAGTTCTATCCTAAATAATGTTTCTAACATTGATTTCAGA

TATATTCTGATGTAGGAGTTTAATATGTAATTGTACTTTTGTAATTGCCCCATTT

>Chamaedorea_linearis_DQ177735.1

CTACTCCTTTTACTGATGTCACAGTAAGTAAATCCATAGAACCTTCTTGCAAGCTTTACCTGAGAAGCCT

GTTGCAATTTAACTTGCTATGTTGGAACTTTTCTGCTTATTCAGTTTAAGAATTCTGAGGCACTGTATCT

GATGTTAAAGAAGATTAAGCTTACTGTTAAGGATTTGTACTAGATATGGTTTAATAAATATGTTTGAGAA

CAGTTACCCCCCACCCCCACCCCCCTTCCCTCTCTGTCTAGAACCTTTCGACTTCTTACCTGGATTTGAT

TTTTTTGAGGGGAGTTTTTGCTTTTCAAGTTCATTTTTTCCCTCTGGATTAGTTAATTATAGTACTATTA

TTTGTTGTCTGCTTTGAATATCTCTTTTGAAGTTCTTGCTGTGTAAGTTTACCCTGTAGACAGAAACATG

GCTTTGAAGAATGACCTTGGACGAGTGTCTTGTAAGTTATTCTGATGATCCTAAACTTGTATTAAGTTTC

AAGACATAATATTTTAACTTAGTCTCTTTTAAATTATATAGTAGACTATCTCCTTGTAATTGACCTTTGA

CTTGGATTTTCCAATTCAATGATCTATCAGGAGTTCTATCCTAAATAATGTTTCTAACATTGATTTCAGA

TATATTCTGATGTTGGAGTTTAATATGTAATTGTACTTTTGTAATTGCTCCATTT

>Chamaedorea_pauciflora_DQ177733.1

CTACTCCTTTTACTGATGTCACAGTAAGTAAATCCATAGGACCTTCTTTCAAGCTTTACCTGAGAAGCCT

GTTGCAATTTAACTTGCTATGTTGGAACTTTTCTGCTTATTCAGTTTAAGAATTCTGAGGCACTGTATCT

GATGTTAAAGAAGATTAAGCTTACTGTTAAGGATTTGTACTAGATATGGTTTAATAAATATGTTTGAGAA

CAGTTACCCCCCACCCCCACCCCCCTTCCTTCTCTGTCTAGAACCTTTCGACTTCTTACCTGGATTTGAT

TTTTTTGAGGGGAGTTTTTGCTTTTCAAGTTCATTTTTTTCCTCTGGATTAGTTAATTATAATACTATTA

TTTGTTGTCTGCTTTGAATATCTCTTTTCAAGTTCTTGCTGTGTAAGTTTACCCTGTAGACAGAAACATG

GCTTTGAAGAATGACCTTGGACGAGTGTCTTGTAAGTTATTCTGATGATCCTAAACTTGTATTAAGTTCA

AGACATAATATTTTAACTTAGTCTCTTTTAAATTATATAGTAGACTATCTCCTTGTAATTGACCTTTGAC

TTGGATTTTCCAATTCAATGATCTATCAGGAGTTCTATACTAAATAATGTTTCTAACATTGATTTCAGAT

ATATTCTGATGTTGGAGTTTAATATATAATTGTACTTTTGTAATTGCTCCATTT

>Chamaedorea_pinnatifrons_DQ177731.1

CTACTCCTTTTACTGATGTCACAGTAAGTAAATCCATAGAACCTTCTTGCAAGCTTTACCTGAGAAGCCT

GTCGCAATTTAACTTGCTATGCTGGAACTTTTCTGCTTATTCAGTTTAAGAATTCTGAGGCACTGTATCT

GATGTTAAAGAAGATTAAGCTTACTGTTAAGGATTTGTACTAGATATGGTTTAATAAATATGTTTGAGAA

CAGTTAACCCCCCACCCCCACCCCCCTTCCCTCTCTGTCTAGAACCTTTCGACTTCTTACCTGGATTTGA

TTTTTTTGAGGGGAGTTTTTGCCTTTCAAGTTCATTTTTTTCCTCTGGATTAGTTAATTATAATACTATT

ATTTGTTGTCTGCTTTGAATATCTCTTTTGAAGTTCTTGCTGTGCAAGTTTACCCTGTAGACAGAAACAT

GGCTTTGAAGAATGACCTTGGACGAGTGTCTTGTAAGTTATTCTGATGATCCTAAACTTGTATTAAGTTT

CAAGACATACTATTTTAACTTAGTCTCTCCTAAATTATATAGTAGACTATCTCCTTGTAATTGACCTTTG

ACTTGGATTTTCCAATTCAATGATCTATCAGGAGTTCTATCCTAAATAATGTTTCTAACATTGATTTCAG

ATATATTCTGATGTAGGAGTTTAATATGTAATTGTACTTTTGTAATTGCCCCATTT

>Chamaedorea_whitelockiana_DQ177729.1

CTACTCCTTTTACTGATGTCACAGTAAGTAAATCCATAGAACCTTCTTGCAAGCTTTACCTGAGAAGCCT

GTCGCAATTTAACTTGCTATGCTGGAACTTTTCTGCTTATTCAGTTTAAGAATTCTGAAGCACTGTATCT

GATGTTAAAGAAGATTAAGCTTACTGTTAAGGATTTGTACTAGATATGGTTTAATAAATATGTTTGAGAA

CAGTTACCCCCCACCCCCACCCCCCTTCCCTCTCTGTCTAGAACCTTTCGACTTCTTACCTGGATTTGAT

TTTTTTGAGGGGAGTTTTTGCCTTTCAAGTTCATTTTTTTCCTCTGGATTAGTTAATTATAATACTATTA

TTTGTTGTCTGCTTTGAATATCTCTTTTGAAGTTCWTGCTGTGTAAGTTTACCCTGTAGACAGAAACATG

GCTTTGAAGAATGACCTTGGACGAGTGTCTTGTAAGTTATTCTGATGATCCTAAACTTGTATTAAGTTTC

AAGACATACTATTTTAACTTAGTCTCTCCTAAATTATATAGTAGACTATCTCCTTGTAATTGACCTTTGA

CTTGGATTTTCCAATTCAATGATCTATCAGGAGTTCTATCCTAAATAATGTTTCTAACATTGATTTCAGA

TATATTCTGATGTAGGAGTTTAATATGTAATTGTACTTTTGTAATTGCCCCATTT

>Chamaedorea_parvifolia_DQ177727.1

CTACTCCTTTTACTGATGTCACAGTAAGTAAATCCATAGAACCTTCTTGCAAGCTTTACCTGAGAAGCCT

GTCGCAATTTAACTTGCTATGCTGGAACTTTTCTGCTTATTCAGTTTAAGAATTCTGAGGCACTGTATCT

GATGTTAAAGAAGATTAAGCTTACTGTTAAGGATTTGTACTAGATATGGTTTAATAAATATGTTTGAGAA

CAGTTACCCCCACCCCCACCCCCACCCCCCTTCCCTCTCTGTCTAGAACCTTTCGACTTCTTACCTGGAT

TTGATTTTTTTAGGGGAGTTTTTGCCTTTCAAGTTCATTTTTTTCCTCTGGATTAGTTAATTATAATACT

ATTATTTGTTGTCTGCTTTGAATATCTCTTTTGAAGTTCTTGCTGTGTAAGTTTACCCTGTAGACAGAAA

CATGGCTTTGAAGAATGACCTTGGACGAGTGTCTTGTAAGTTATTCTGATGATCCTAAACTTGTATTAAG

TTTCAAGACATACTATTTTAACTTAGTCTCTCCTAAATTATATAGTAGACTATCTCCTTGTAATTGACCT

TTGACTTGGATTTTCCAATTCAATGATCTATCAGGAGTTCTATCCTAAATAATGTTTCTAACATTGATTT

CAGATATATTCTGATGTAGGAGTTTAATATGTAATTGTACTTTTGTAATTGCCCCATTT

>Chamaedorea_elegans_DQ177725.1

CTACTCCTTTTACTGATGTCACAGTAAGTAAATCCATAGAACCTTCTTGCAAGCTTTACCTGAGAAGTCT

GTCGCAATTTAACTTGTTATGTTGGAACTTTTCTGCTCATTCAGTTTAAGAATTCTGAGGCACTGTATCT

GATGTTAAAGAAGATTAAGCTTACTGTTAAGGATTTGTACTAGATATGGTTTAATAAATATGTTTGAGAA

CAGTTACCCCCCACCCCCACCCCCACCCCCACCCCCCTTCCCTCTCTGTCTAGAACCTTTCGGCTTCTTA

CCTGGATTTGATTTTTTTGAGGGGAGTTTTTGCCTTTCAAGTTCATTTTTTTCCTCTGGATTAGTTAATT

ATAATACTATTATTTGTTGTCTGCTTTGAATRTCTCTTTTGAAGTTCTTGCTGTGTAAGTTTACCCTGTA

GACAGAAACATGGCTTTGAAGAATGACKTTGGACGAGTGTCTTGTAAGTTATTCTGATGATCCTAAACTT

GTATTAAGTTTCAAGACATACTATTTTAACTTAGTCTCTCCTAAATTATATGGTAGACTATCTCCTTGTA

ATTGACCTTTGACTTGGATTTTCCAATTCAATGATCTATCAGGAGTTCTATCCTAAATAATGTTTCTAAC

ATTGATTTCAGATATATTCTGATGTAGGAGTTTAATATGTAATTGTACTTTTGTAATTGCCCCATTA

>Chamaedorea_metallica_DQ177723.1

CTACTCCTTTTACTGATGTCACAGTAAGTAAATCCATAGAACCTTCTTGCAAGCTTGACCTGAGAAGCTT

GTCGCAATTTAACTTGCTATGCTGGAACTTTTCTGATTATTCAGTTTAAGAATTCTGAGGCACTGTATCT

GGTGTWAAAGATGATTAAGCTTACTGTTAAGGATTTGTACTAGATATGGTTTAATAAATATGTTTGAGAA

CAGTTACCCCCCAGCGCCACCCCCCTTCCCTCTCTGTCTAGAACCTTTCGACTTCTTACCTGGATTTGAT

TTTTTTGAGGGGAGTTTTTGCCTTTCAAGTTCATTTTTTTCCTCTGGATTAGTTAATTATAATACTATTA

TTTGTTGTCTGCTTTGAATATCTCTTTTGAAGTTCTTGCTGTGTAAGTTTACCCTGTAGACAGAAACATG

GCTTTGAAGAATGACCTTGGACGAGTGTCTTGTAAGTTATTCTGATGATCCTAAACTTGTATTAAGTTTC

AAGACATACTATTTTAACTTAGTCTCTCCTAAATTATATAGTAGACTATCGACCTTTGACTTGGATTTTC

CAATTCAATGATCTGTCAGGAGTTCTATCCTTAATAATGTTTCTAACATTGATTTCAGATATATTCTGAT

GTAGGAGTTTAATATGTAATTGTACTTTTGTAATTGCCCCATCT

>Chamaedorea_arenbergiana_DQ177721.1

CTACTCCTTTTACTGATGTCACAGTAAGTAAATCCATAGAACCTTCTTGCAAGCTTTACCGGAGAAGCCT

GTCACAATTTAACTTGCTATGCTGGAACTTTTCTGCTTATTCAGTTTAAGAATTCTGAGGCACTGTATCT

GATGTTAAAGAAGATTTAGCTTACTGTTAAGGATTGTACTAGATATGGTTTAATAAATATGTTTGAGAAC

AGTTGCCCCCCACCCCCACCCCCCTTCCCTCTCTGTCTAGAACCTTTCGACTTCTTACCTGGATTTGATT

TTTTTGAGGGGAGTTTTTGCCTTTCAAGTTCATTTTTTTCCTCTGGATTAGTTAATTATAATACTATTAT

TTGTTGTCTGCTTTGAATATCTCTTTTGAAGTTCTTGCTGTGTAAGTTTACCCTGTAGACAGAAACATGG

CTTTGAAGAATGACCTTGGACGAGTGTCTTGTAAGTTATTCTGATGATCCTAAACTTGTATTAAGTTTCA

AGACATACTATTTTAACTTAATCTCTCTTAAATTATATAGTAGACTATCTCCTTGTAATTGAACTTTGAC

TTGGATTTTCCAATTCAATGATCTATCAGGAGTTCTATCCTAAATAATGTTTCTAACATTGATTTCAGAT

ATAATCTGATGTAGGAGTTTAATATGTAATTGTACTTTTGTAATTGCCCCATTT

>Chamaedorea_pochutlensis_DQ177719.1

CTACTCCTTTTACTGATGTCACAGTAAGTAAATCCATAGAACCTTCTTGCAAGCTTTACCTGAGAAGCCT

GTCGCAATTTAACTTGCTATGCTGGAACTTTTCTGCTTATTCAGTTTAAGAATTCTGAGGCACTGTATCT

GATGTTAAAGAAGATTAAGCTTACTGTTAAGGATTTGTACTAGATATGGTTTAATAAATATGTTTGAGAA

CAGTTACCCCCCACCGCCACCCCCCTTCCCTCTCTGTCTAGAACTTTTCGACTTCTTACCTGGATTTGAT

TTTTTTGAGGGGAGTTTTTGCCTTTCAAGTTCATTTTTTTCCTCTGGATTAGTTAATTATAATACTATTA

TTTGTTGTCTGCTTTGAATATCTCTTTTGAAGTTATTGCTGTGTAAGTTTACCCTGTAGACAGAAACATG

GCTTTGAAGAATGACCTTGGACGAGTGTCTTGTAAGTTATTCTGATGATCCTAAACTTGTATTAAGTTTC

AAGACATACTATTTTAACTTAGTCTCTCCTAAATTATATAGTAGACTATCTCCTTGTAATTGACCTTTGA

CTTGGATTTTCCAATTCAATGATCTATCAGGAGTTCTATCCTAAATAATGTTTCTAACATTGATTTCAGA

TATATTCTGATGTAGGAGTTTCATATGTAATTGTACTTTTGTAATTGCCCCATTT

>Chamaedorea_tenella_DQ177717.1

CTACTCCTTTTACTGATGTCACAGTAAGTAAATCCATAGAACCTTCTTGCAAGCTTTACCTGAGAAGCCT

GTCGCAATTTAACTTGCTATGCTGGAACTTTTCTGCTTATTCAGTTTAAGAATTCTGAGGCACTGTATCT

GATGTTAAAGAWGATTAAGCTTACTGTTAAGGATTTGTACTAGATATGGTTTAATAAATATGTTTGAGAA

CAGTTAACCCCCCACCCCCACCCCCACCCCCACCCCCCTTCCCTCTCTGTCTAGAACCTTTCGACTTCTT

ACCTGGATTTGATTTTTTTTTGAGGGGAGTTTTTGCCTTTCAAGTTCATTTTTTTCCTCTGGATTAGTTA

ATTATAATACTATTATTTGTTGTCTGCTTTGAATATCTCTTTTRAAGTTCTTGCTGTGCAAGTTTACCCT

GTAGACAGAAACATGGCTTTGAAGAATGACCTTGGACGAGTGTCTTGTAAGTTATTCKGATGATCCTAAA

CTTGTATTAAGTTTCAAGACATACTATTTTAACTTAGTCTCTCCTAAATTATATAGTAGACTATCTCCTT

GTAATTGACCTTTGACTTGGATTTTCCAATTCAATGATCTATCAGGAGTTCTATCCTAAATAATGTTTCT

AACATTGATTTCAGATATATTCTGATGTAGGAGTTTAATATGTAATTGTACTTTTGTAATTGCCCCATTT

>Chamaedorea_stolonifera_DQ177715.1

CTACTCCTTTTACCGATGTCACAGTAAGTAAATCCATAGAACCTTCTTGCAAGCTTTACCTGAGAAGCCT

GTCCAAATTTAACTTGCTATGCTGGAACTTTTCTGATTATTCAGTTTAAGAATTCCGAGGCACTGTATCT

GATGTTAAAGAAGATTAAGCTTACTGTTAAGGATTTGTACTAGATATGGTTTAATAAATATGTTTGAGAA

CAGTTACCCCCCAGCGCCACCCCCCTTCCCTCTCTGTCTAGAACCTTTCGACTTCTTACCTGGATTTGAT

TTTTTTGAGGGGAGTTTTTGCCTTTCAAGTTCATTTTTTTCCTCTGGATTAGTTGATTATAATACTATTA

TTTGTTGTCTGCTTTGAATATCTCTTTTGAAGTTCTTGCTGTGTAAGTTTACCCTGTAGACAGAAACATG

KCTTTGAAGAATGACCTTGGACGAGTGTCTTGTAAGTTATTCTGATGATCCTAAACTTGTATTAAGTTTC

AAGACATACTATTTTAACTTAGTCTCTCCTAAATTATATAGTAGACTATCTCCTTGTAATTGACCTTTGA

CTTGGATTTTCCAATTCAATGATCTATCAGGAGTTCTATCCTTAATAATGTTTCTAACATTGATTTCAGA

TATATTCTGATGTAGGAGTTTAATATGTAATTGTACTTTTGTAATTGCCCCATTT

>Chamaedorea_sartorii_DQ177713.1

CTACTCCTTTTACTGATGTCACAGTAAGTAAATCCATAAGAACCTTCTTGCAAGCTACCTGAGAAGCCTG

TCGCAATTTAACTTGCTATGCTGGAACTTTTCTGCTTATTCAGTTTAAGAATTCTGAGGCACCGTATCTG

ATGTTAAAGAAGATTAAGCTTACTGTTAAGGATTTGTACTAGATATGGTTTAATAAATATGTTTGAGAAC

AGTTAACCCCCCAGCGCCACCCCCCTTCCCTCTCTGTCTAGAACCTTTCGACTTCTTACCTGGATTTGAT

TTTTTTGAGGGGAGTTTTTGCCTTTCAAGTTCATTTTTTCCCTCTGGATTAGTTAATTATAATACTATTA

TTTGTTGTCTGCTTTGAATATCTCTTTTGAAGTTCTTGCTGTGTAAGTTTACCCTGTAGACAGAAACATG

GCTTTGAAGAATGACCTTGGACGAGTGTCTTGTAAGTTATTCTGATGATCCTAAACTTGTATTAAGTTTC

AAGACATACTATTTTAACTTAGTCTCTCCTAAATTATATAGTAGACTATCTCCTTGTAATTGACCTTTGA

CTTGGATTTTCCAATTCAATGATCTATCAGGAGTTCTATCCTTAATAATGTTTCTAACATTGATTTCAGA

TATATTCTGATGTAGGAGTTTAATATGTAATTGTACTTTTGTAATTGCCCCATTT

>Chamaedorea_tuerckheimii_DQ177711.1

CTACTCCTTTTACTGATGTCACAGTAAGTAAATCCATAGAACCTTCTTGCAAGCTTGACCTGAGAAGCCT

GTCGCAATTTAACTTGCTATGCTGGAACTTTTCTGATTATTCAGTTTAAGAATTCTGAGGCACTGTATCT

GATGTTAAAGAAGATTAAGCTTACTGTTAAGGATTTGTACTAGATATGGTTTAATAAATATGTTTGAGAA

CAGTTACCCCCCAGCGCCACCCCCCTTCCCTCTCTGTCTAGAACCTTTCGACTTCTTGCCTGGATTTGAT

TTTTTTGAGGGGAGTTTTTGCCTTTCAAGTTCATTTTTTTCCTCTGGATTAGTTAATTATAATACTATTA

TTTGTTGTCTGCTTTGAATATCTCTTTTGAAGTTCTTGCTGTGTAAGTTTACCCTGTAGACAGAAACATG

GCTTTGAAGAATGACCTTGGACGAGTGTCTTGTAAGTTATTCTGATGATCCTAAACTTGTATTAAGTTTC

AAGACATACTATTTTAACTTAGTCTCTCCTAAATTATATAGTAGACTATCTCCTTGTAATTGACCTTTGA

CTTGGATTTTCCAATTCAATGATCTATCAGGAGTTCTATCCTTAATAATGTTTCTAACATTGATTTCAGA

TATATTCTGATGTAGGAGTTTAATATGTAATTGTACTTTTGTAATTGCCCCATCT

>Chamaedorea_nationsiana_DQ177709.1

CTACTCCTTTTACTGATGTCACAGTAAGTAAATCCATAGAACCTCTTGCAAGCTTTACCGGAGAAGCCTG

TCACAATTTAACTTGCTATGCTGGAACTTTTCTGCTTATTCAGTTTAAGAATTCTGAGGCACTGTATCTG

ATGTTAAAGAAGATTTAGCTTACTGTTAAGGATTGTACTAGATATGGTTTAATAAATATGTTTGAGAACA

GTTGCCCCCCACCCCCACCCCCCTTCCCTCTCTGTCTAGAACCTTTCGACTTCTTACCTGGATTTGATTT

TTTTGAGGGGAGTTTTTGCCTTTCAAGTTCATTTTTTTCCTCTGGATTAGTTAATTATAATACTATTATT

TGTTGTCTGCTTTGAATATCTCTTTTGAAGTTCTTGCTGTGTAAGTTTACCCTGTAGACAGAAACATGGC

TTTGAAGAATGACCTTGGACGAGTGTCTTGTAAGTTATTCTGATGATCCTAAACTTGTATTAAGTTTCAA

GACATACTATTTTAACTTAATCTCTCTTAAATTATATAGTAGACTATCTCCTTGTAATTGAACTTTGACT

TGGATTTTCCAATTCAATGATCTATCAGGAGTTCTATCCTAAATAATGTTTCTAACATTGATTTCAGATA

TAATCTGATGTAGGAGTTTAATATGTAATTGTACTTTTGTAATTGCCCCATTT

>Chamaedorea_brachypoda_DQ177707.1

CTACTCCTTTTACTGATGTCACAGTAAGTAAATCCATAGAACCTTCTTGCAAGCTTTACCTGAGAAGCCT

GTCGCAATTTAACTTGCTATGCTGGAACTTTTCTGCTTATTCAGTTTAAGAATTCTGAGGCACTGTATCT

GATGTTAAAGAAGATTAAGCTTACTGTTAAGGATTTGTACTATATATGGTTTAATAAATATGTTTGAGAA

CAGTTACCCCCCACCTCCACCCCCCTTCCTTCTCTGTCTAGAACCTTTCGACTTCTTACCTGGATTTAAT

TTTTTTAAGGGGAGTTTTTGCCTTTCAAGTTCATTTTTTTCTTCTGGATTAGTTAATTATAATACTATTA

TTTGTTGTCTGCTTTGAATCTCTCTTTTGAAGTTCTTGCTGTGTAAGTTTACCCTGTAGACAGAAACATG

GCTTTGAAGAATGACCTTGGACGAGTGTCTTGTAAGTTATTCTGATGATCGTAAACTTGTATTAAGTTTC

AAGACATACTATTTTAACTTTGTCTCTCCTAAATTATATAGCAGACTATGTCCTTGTAATTGACCTTTGA

CTTGGATTTTCCAATTCAATGATCTATCAGGAGTTCTATCCTAAATAATGTTTCTAACATTGATTTCAGA

TATATTCTGATGTTGGAGTTTAATATGTAATTGTACTTTTGTAATTTCTCCATTT

>Chamaedorea_macrospadix_DQ177705.1

CTACTCCTTTTACTGATGTCACAGTAAGTAAATCCATAGAACCTTCTTGCAAGCTTTACCTGAWAAGCCT

GTCGCAATTTAACTTGCTATGCTGGAACTTTTCTGCTTATTCAGTTTAAGAATTCTGAGGCACTGTATCT

GATGTTAAAGAAGATTAAGCTTACTGTTAAGGATTTGTACTAGATATGGTTTAATAAATATGTTTGAGAA

CAGTTACCCCCACCCCCACCCCCCTTCCCTCTCTGTCTAGAACCTTTYGACTTCTTACCTGGATTTGATT

TTTTTGAGGGGAGTTTTTGCCTTTCAAGTTCATTTTTTTCCTCTGGATTAGTTAATTATAATACTATTAT

TTGTTGTCTGCTTTGAATATCTCTTTTGAAGTTCTTGCTGTGTAAGTTTACCCTGTAGACAGAAACATGG

CTTTGAAGAATGACCTTGGACGAGTGTCTTGTAAGTTATTCTGATGATCCTAAACTTGTATTAAGTTTCA

AGACATACTATTTTAACTTAGTCTCTCCTAAATTATATAGTAGACTATCTCCTTGTAATTGACCTTTGAC

TTGGATTTTCCAATTCAATGATCTATCAGGAGTTCTATCCTAAATAATGTTTCTAACATTGATTTCAGAT

ATATTCTGATGTAGGAGTTTAATATGCAATTGTACTTTTGTAATTGCCCCATTT

>Chamaedorea_seifrizii_DQ177703.1

CTACTCCTTTTACTGATGTCACAGTAAGTAAATCCATAGAACCTTCTTGCAAGTTTTACCCGAGAAGCCT

GTCGCAATTTAACTTGCTATGCTGGAACTTTTCTGCTTATTCAGTTTAAGAATTCTGAGGCACTGTATCT

GATGTTAAAGAAGATTAAGCTTACTGTTAAGGATTTGTACTATATATGGTTTAATAAATATGTTTGAGAA

CAGTTACCCCCCACCTCCACCCCCCTTCCTTCTCTGTCTAGAACCTTTCGACTTCTTACCTGGATTTAAT

TTTTTTAAGGGGAGTTTTTGCCTTTCAAGTTCATTTTTTTCTTCTGGATTAGTTAATTATAATACTATTA

TTTGTTGTCTGCTTTGAATATCTCTTTTGAAGTTCTTGATGTGTAAGTTTACCCTGTAGACAGAAACATG

GCTTTGAAGAATGACCTTGGACGAGTGTCTTGTAAGTTATTCTGATGATCCTAAACTTGTATTTCAGTTT

CAAGACATACTATTTTAACTTAGTCTCTCCTAAATTATATAGTAGACTTTCTCCTTGTAATTGACCTTTG

ACTTGGATTTTCCAATTCAATGATCTATCAGGAGTTCTATCCTAAATAATGTTTCTAACATTGATTTCAG

ATATATTCTGATGTTGGAGTTTAATATGTAATTGTACTTTTGTAATTGCTCCATTT

>Chamaedorea_hooperiana_DQ177701.1

CTACTCCTTTTACTGATGTCACAGTAAGTAAATCCATAGAACCTTCTTGCAAGCTTTACCTGAGAAGCCT

GTCGCAATTTAACTTGCTATGCTGGAACTTTTCTGCTTATTCAGTTTAAGAATTCTGAGGCACTGTATCT

GATGTTAAAGAAGATTAAGCTTACTGTTAAGGATTTGTACTAGATATGGTTTAATAAATATGTTTGAGAA

CAGTTACCCCCCACCCCCACCCCCCTTCCCTCTCTGTCTAGAACCTTTCGACTTCTTACCTGGATTTGAT

TTTTTTGAGGGGAGTTTTTGCCTTTCAAGTTCATTTTTTTCCTCTGGATTAGTTAATTATAATACTATTA

TTTGTTGTCTGCTTTGAATATCTCTTTTGAAGTTCTTGCTGTGTAAGTTTACCCTGTAGACAGAAACATG

GCTTTGAAGAATGACCTTGGACGAGTGTCTTGTAAGTTATTCTGATGATCCTAAACTTGTTTTAAGTTTC

AAGACATACTATTTTAACTTAGTCTCTCCTAAATTATATGGTAGACTATCTCCTTGTAATTGACCTTTGA

CTTGGATTTTCCAATTCAATGATCTATCAGGAGTTCTATCCTAAATAATGTTTCTAACATTGATTTCAGA

TATATTCTGATGTAGGAGTTTAATATGTAATTGTACTTTTGTAATTGCCCCATTT

>Synechanthus_warscewiczianus_DQ177758.1

CTACTCCTTTTACTGATGTCACAGTAAGTAAATCCATAGAACCTTCTTGCAAGCTTTACCTGAGAAGCAT

CTCGCAATTCAACTTGCTATGCTGGAACTTTTTTGCTTATTCAGTTTAAGAATTCTGAGGCACTATATCT

GATGTTAAAGAAGATTACGGAATGCTTCGTTTGTGCTGCTCCATGAGGAAGTAATTTTGCATATGTCTAT

CTAAAAGACGTTGATAGCTTATACAAAAACACATGGTTATTCAATTTTCATTACTGTTAAGGATTTGTAC

TAGATATGGTTTAATAAATATGTTTGAGCACTGYTACCCGCCCCCCCCCCCCACCCCCTTCCCTCTCACT

CTGTCTGGAACCTTTCAACTTCTTACCTGGATTTGATTTTTTTTTAGGGGAGTTTTTTCCTTTCGAGTTC

ATATTTTTCTTCTGGATTAGTTAATTATAATCCTATTATTTGTAGTCTGCTTTGAATATCTCTTTTGAAG

TTCTTGCTGTGTAAGTTCACCCTGTAGACAGAAACATGGCTTTGAAGAATGACCTTGGACAAGTGTCTTG

TAAATTATTCTGATGATCCTAAACTTGTATAAGTTTCAAGACTTACTATTTTGACTAAGGTCCCTCCTAA

ATTATATAGTAGACTATCTCCTTGTAATTGACCTTTGACTTGGATTTTCCAAGTCAATGATCTATCAGGA

GTTCTATCCTAAATAATGTTTCTAACATTGATTACAGATCTATTCTGATGTAGGAGTTTAATATGTAATT

GTACTTTTGTAATTGCCCTATTA

>Hyophorbe_lagenicaulis_DQ177756.1

CTACTCCTTTTACTGATGTCACAGTAAGTAGTTGCAAGCTTTACCTGAGAAGCATGTAGCAATTTAATTT

GCTATGCTGGAGCTTTTTTGCTTATTCAGTTTAAGAATTCTGAGGCACTGTATCTGTTGTTAATGAAGAT

TAAGGAAAGCTTCTTTTATTCTGCTTGAGGAGGGAGTAATTATGCATATGTCTAGCTAAAAGACGTTGTT

GATAGCTTATACAAAAACACATGGTTATTCAATTAAGGATCCCCACCGCGCCCCCCCCCCTYTYTATYTA

GAACCYTTYAACTTYTTACCYGGRWTTGAWTTTTTTTTTTTTTTGAGGGGAGATTTTGCCTTTCAAGTTC

ATATTTTCCTCTGGATTAGTTAATTATAATCCTATTATTTGTAGTCTGCTTTGAATATCTCTTTTGAAGT

TCTTGATGTGTAAGTTTACCCTGTTGACAGAAACATGGCTTTGAAGAATGACCTTGGACGAGTGTCTTGT

TAATTATTCTGATGATCCTAAACTTGTATAAGTTTCAAGACATACTATTTGAGACAGGTCTCTCCTAAAT

TATATAGTAGGCTCTCTCCTTGTAACTGACCTGTGACTCGGATTTTCCAATTCAATGATCTATCAGGAGT

TCTAATCTAAATAATGTTTCTAACATTGATTTCAGATTCTGATGTTGGAGTGTAATATGTAATTGTACTT

TTGTAATTGCCCCAYTA

>Chamaedorea_oblongata_DQ177754.1

CTACTCCTTTTACTGATGTCGCAGTAAGTAAATCCATAGAACCTTCTTGCAAGCTTTACCTGAGAAGCCT

GTCGCAATTTAACTTGCTATGCTGGAACTTTTCTGCTTATTCAGTTTAAGAATTCTGAGGCACTGTATCT

GATGTTAAAGAAGATTAAGCTTACTGTTAAGGATTTGTACTAGATATGGTTTAATAAATATGTTTGAGAA

CAGTTACCCCCCACCCCCACCCCCCTTCCCTCTCTGTCTAGAACCTTTCGACTTCTTACCTGGATTTGAT

TTTTTTGAGGGGAGTTTTTGCCTTTCAAGTTCATTTTTTTCCTCTGGATTAGTTAATTATAATACTATTA

TTTGTTGTCTGCTTTGAATATCTCTTTTGAAGTTCTTGCTGTGTAAGTTTACCCTGTAGACAGAAACATG

GCTTTGAAGAATGACCTTGGACGAGTGTCTTGTAAGTTATTCTGATGATCCTAAACTTGTATTAAGTTTC

AAGACATACTATTTTAACTTAGTCTCTCCTAAATTATATAGTAGACTATCTCCTTGTAATTGACCTTTGA

CTTGGATTTTCCAATTCAATGATCTATCAGGAGTTCTATCCTAAATAATGTTTCTAACATTGATTTCAGA

TATATTCTGATGTAGGAGTTTAATATGTAATTGTACTTTTGTAATTGCCCCATTT

>Chamaedorea_schippii_DQ177752.1

CTACTCCTTTTACTGATGTCACAGTAAGTAAATCCATAGAACCTTCTTGCAAGCTTTACCTGAGAAGCCT

GTTGCAATTTAACTTGCTATGCTGGAACTTTTCTGCTTATTCAGTTTAAGAATTCTGAGGCACTGTATCT

GATGTTAAAGAAGATTAAGCTTACTGTTAAGGATTTGTACTAGATATGGTTTAATAAATATGTTTGAGAA

CAGTTACCCCCCACCCCCACCCCCCTTCCCTCTCTGTCTAGAACCTTTCGACTTCTTACCTGGATTTGAT

TTTTTTGAGGGGAGTTTTTGCCTTTCAAGTTCATTTTTTTCCTCTGGATTAGTTAATTATAATACTATTA

TTTGTTGTCTGCTTTGAATATCTCTTTTGATGTTCTTGCTGTGTAAGTTTACCCTGTAGACAGAAACATG

GCTTTGAASAATGACCTTGGACGAGTGTCTTGTAAGTTATTCTGATGATCCTAAACTTGTATTAAGTTTC

AAGACATACTATTTTAACTTAGTCTCTCCTAAATTATATAGTAGACTATCTCCTTGTAATTGACCTTTGA

CTTGGATTTTCCAATTCAATGATCTATCAGGAGTTCTATCCTAAATGATGTTTCTAACATTGATTTCAGA

TATATTCTGATGTAGGAGTTTAATATGTAATTGTACTTTTGTAATTGCCCCATTT

>Chamaedorea_plumosa_DQ177750.1

CTACTCCTTTTACTGATGTCACAGTAAGTAAATCCATAGAACCTTTTTGCAAGCTTTACCTGAGAAGCCT

GTCGCAATTTAACTTGCTATGTTGGAACTTTTCTGCTTATTCAGTTTAAGAATTCTGAGGCACTGTATCT

GATGTTAAAGAAGATTAATCTTACTGTTAAGGATTTGTACTAGATATGGTTTAATAAATATGTTTGAGAA

CAGTTACCCCCCACCCCCACCCCCACCCCCCTTCCCTCTCTGTCTAGAACCTTTCGGCTTCTTACCTGGA

TTTGATTTTTTTGAGGGGAGTTTTTGCCTTTCAAGTTCATTTTTTCCCTCTGGATTAGTTAATTATAATA

CTATTATTTGTTGTATGCTTTGAATATCTCTTTTGAAGTTCTTGCTGTGTAAGTTTACCCTGTAGACAGA

AACATGGCTTTGAAGAATGACTTTGGACGAGTGTCTTGTAAGTTATTCTGATGATCCTAAACTTGTATTA

AGTTTCAAGACATACTATTTTAACTTAGTATCTCCTAAATTATATAGTAGACTATCTCCTTGTAATTGAC

CTTTGACTTGGATTTTCCAATTCAATGATCTATCAGGAGTTCTATCCTAACTAATGTTTCTAACATTGAT

TTCAGATATATTCTGATGTAGGAGTTTAATATGTAATTGTACTTTTGTAATTGCCCCATTA

>Chamaedorea_microphylla_DQ177748.1

CTACTCCTTTTACTGATGTCACAGTAAGTAAATCCGTAGAACCTTCTTGCAAGCTTTACCTGAGAAGCCT

GTCGCAATTTAACTTGCTATGCTGGAACTTTTCTGCTTATTCAGTTTAAGAATTCTGAGGCACTGTATCT

GATGTTAAAGAAGATTAAGCTTACTGTTAAGGATTTGTACTAGATATGGTTTAATAAATATGTTTGAGAA

CAGTTACCCCCCACCCCCACCCCCCTTCCCTCTCTGTCTAGAACCTTTCGACTTCTTACCTGGATTTGAT

TTTTTTGAGGGGAGTTTTTGCCTTTCAAGTTCATTTTTTTCCTCTGGATTAGTTAATTATAATACTATTA

TTTGTTGTCTGCTTTGAATATCTCTTTTGAAGTTCTTGCTGTGTAAGTTTACCCTGTAGACAGAAACATG

GCTTTGAAGAATGACCTTGGACGAGTGTCTTGTAAGTTATTCTGATGATCCTAAACTTGTTTTAAGTTTC

AAGACATACTATTTTAACTTAGTCTCTCCTAAATTATATGGTAGACTATCTCCTTGTAATTGACCTTTGA

CTTGGATTTTCCAATTCAATGATCTATCAGGAGTTCTATCCTAAATAATGTTTCTAACATTGATTTCAGA

TATATTCTGATGTAGGAGTTTAATATGTAATTGTACTTTGTAATTGCCCCATTT

>Chamaedorea_rojasiana_DQ177746.1

CTACTCCTTTTACTGATGTCACAGTAAGTAAATCCATAGAACCTTCTTGCAAGCTTTACCTGAGAAGCCT

GTCGCAATTTAACTTGCTATGCTGGAACTTTTCTGCTTATTCAGTTTAAGAATTCTGAGGCACTGTATCT

GATGTTAAAGAAGATTAAGCTTACTGTTAAGGATTTGTACTAGATATGGTTTAATAAATATGTTTGAGAA

CAGTTAACCCCCCACCCCCACCCCCTTCCCTCTCTGTCTAGAACCTTTCGACTTCTTACCTGGATTTGAT

TTTTTTGAGGGGAGTTTTTGCCTTTCAAGTTCATTTTTTTCCTCTGGATTAGTTAATTATAATACTATTA

TTTGTTGTCTGCTTTGAATATCTCTTTTGAAGTTCTTGCTGTGCAAGTTTACCCTGTAGACAGAAACATG

GCTTTGAAGAATGACCTTGGACGAGTGTCTTGTAAGTTATTCTGATGATCCTAAACTTGTATTAAGTTTC

AAGACATACTATTTTAACTTAGTCTCTCCTAAATTATATAGTAGACTATCTCCTTGTAATTGACCTTTGA

CTTGGATTTTCCAATTCAATGATCTATCAGGAGTTCTATCCTAAATAATGTTTCTAACATTGATTTCAGA

TATATTCTGATGTAGGAGTTTAATATGTAATTGTACTTTTGTAATTGCCCCATTT

>Chamaedorea_geonomiformis_DQ177744.1

TTCTTGCAAGCTTTACCTGAGAAGCCTGTCGCAATTTAACTTGCTATGCTGGAACTTTTCTGCTTATTCA

GTTTAAGAATTCTGAGGCACTGTATCTGATGTTAAAGAAGATTAAGCTTACTGTTAAGGATTTGTACTAG

ATATGGTTTAATAAATATGTTTGAGAACAGTTAACCCCCCACCCCCACCCCCACCCCCACCCCCACCCCC

CTTCCCTCTCTGTCTAGAACATTTCGACTTCTTACCTGGATTTGATTTTTTTTTGAGGGGAGTTTTTGCC

TTTCAAGTTCATTTTTTTCCTCTGGATTAGTTAATTATAATACTATTATTTGTTGTCTGCTTTGAATATC

TCTTTTGAAGTTCTTGCTGTGCAAGTTTACCCTGTAGACAGAAACATGGCTTTGAAGAATGACCTTGGAC

GAGTGTCTTGTAAGTTATTCTGATGATCCTAAACTTGTATTAAGTTTCAAGACATACTATTTTAACTTAG

TCTCTCCTAAATTATATAGTAGACTATCTCCTTGTAATTGACCTTTGACTTGGATTTTCCAATTCAATGA

TCTATCAGGAGTTCTATCCTAAATAATGTTTCTAACATTGATTTCAGATATATTCTGATGTAGGAGTTTA

ATATGTAATTGTACTTTTGTAATTGCCCCATTT

>Chamaedorea_warscewiczii_DQ177742.1

CTACTCCTTTTACTGATGTCACAGTAAGTAAATCCATAGAACCTTCTTGCAAGCTTTACCTGAGAAGCCT

GTCGCAATTTAACTTGCTATGCTGGAACTTTTCTGCTTATTCAGTTTAAGAATTCTGAGGCACTGTATCT

GATGTTAAAGAAGATTAAGCTTACTGTTAAGGATTTGTACTAGATATGGTTTAATAAATATGTTTGAGAA

CAGTTACCCCCACCCCCACCCCCCTTCCCTCTCTGTCTAGAACCTTTCGACTTCTTACCTGGATTTGATT

TTTTTGAGGGGAGTTTTTGCCTTTCAAGTTCATTTTTTTCCTCTGGATTAGTTAATTATAATACTATTAT

TTGTTGTCTGCTTTGAATATCTCTTTTGAAGTTCTTGCTGTGTAAGTTTACCCTGTAGACAGAAACATGG

CTTTGAAGAATGACCTTGGACGAGTGTCTTGTAAGTTATTCTGATGATCCTAAACTTGTATTAAGTTTCA

AGACATACTATTTTAACTTAGTCTCTCCTAAATTATATAGTAGACTATCTCCTTGTAATTGACCTTTGAC

TTGGATTTTCCAATTCAATGATCTATCAGGAGTTCTATCCTAAATAATGTTTCTAACGTTGATTTCAGAT

ATATTCTGATGTAGGAGTTTAATATGTAATTGTACTTTTGTAATTGCCCCATTT

>Chamaedorea_serpens_DQ177740.1

CTACTCCTTTTACTGATGTCACAGTAAGTAAATCCATAGAACCTTCTTGCAAGCTTTACCTGAGAAGCCT

GTCGCAATTTAACTTGCTATGCTGGAACTTTTCTGCTTATTCAGTTTAAGAATTCTGAGGCACTGTATCT

GATGTTAAAGAAGATTAAGCTTACTGTTAAGGATTTGTACTAGATATGGTTTAATAAATATGTTTGAGAA

CAGTTAACCCCCCACCCCCACCCCCCTTCCCTCTCTGTCTAGAACCTTTCGACTTCTTACCTGGATTTGA

TTTTTTTGAGGGGAGTTTTTGCCTTTCAAGTTCATTTTTTTCCTCTGGATTAGTTAATTATAATACTATT

ATTTGTTGTCTGCTTTGAATATCTCTTTTGAAGTTCTTGCTGTGCAAGTTTACCCTGTAGACAGAAACAT

GGCTTTGAAGAATGACCTTGGACGAGTGTCTTGTAAGTTATTCTGATGATCCTAAACTTGTATTAAGTTT

CAAGACATACTATTTTAACTTAGTCTCTCCTAAATTATATAGTAGACTATCTCCTTGTAATTGACCTTTG

ACTTGGATTTTCCAATTCAATGATCTATCAGGAGTTCTATCCTAAATAATGTTTCTAACATTGATTTCAG

ATATATTCTGATGTAGGAGTTTAATATGTAATTGTACTTTTGTAATTGCCCCATTT

>Chamaedorea_quezalteca_DQ177738.1

CTACTCCTTTTACTGATGTCACAGTAAGTAAATCCATAGAACCTTCTTGCAAGCTTTACCTGAGAAGCCT

GTCGCAATTTAACTTGCTATGCTGGAACTTTTCTGCTTATTCAGTTTAAGAATTCTGAGGCACTGTGTCT

GATGTTAAAGAAGATTAAGCTTACTGTTAAGGATTTGTACTAGATATGGTTTAATAAATATGTTTGAGAA

CAGTTACCCCCCACCCCCACCCCCCTTCCCTCTCTGTCTAGAACCTTTCGACTTCTTACCTGGATTTGAT

TTTTTTGAGGGGAGTTTTTGCCTTTCAAGTTCATTTTTTTCCTCTGGATTAGTTAATTATAATACTATTA

TTTGTTGTCTGCTTTGAATATCTCTTTTGAAGTTCTTGCTGTGTAAGTTTACCCTGTAGACAGAAACATG

GCTTTGGAGAATGACCTTGGACGAGTGTCTTGTAAGTTATTCTGATGATCCTAAACTTGTTTTAAGTTTC

AAGACATACTATTTTAACTTAGTCTCTCCTAAATTATATGGTAGACTATCTCCTTGTAATTGACCTTTGA

CTTGGATTTTCCAATTCAATGATCTATCAGGAGTTCTATCCTAAATAATGTTTCTAACATTGATTTCAGA

TATATTCTGATGTAGGAGATTAATATGTAATTGTACTTTTGTAATTGCCCCATTT

>Chamaedorea_coralliformis_DQ177736.1

CTACTCCATTTACTGATGTCACAGTAAGTAAATCCATAGAACCTTCTTGCAAGCTTTACCGGAGAAGCCT

GTCGCAATTTAACTTGCTATGCTGGAACTTTTCTGCTTATTCAGTTTAAGAATTCTAAGGCACTGTATCT

GATGTTAAAGAAGATTAAGCTTACTGTTAAGGATTGTACTAGATATGGTTTAATAAATATGTTTGAGAAC

AGTTGCCCCCCCACCCCCACCCCCYTTCCCTCTCTGTCTAGAACCTTTCGACTTCTTGCCTGGATTTGAT

TTTTTTGAGGGGAGTTTTTGCCTTTCAAGTTCATTTTTTTCCTCTGGATTAGTTAATTATAATACTATTA

TTTGTTGTCTGCTTTGAATATCTCTTTTGAAGTTCTTGCTGTGTAAGTTTACCCTGTAGACAGAAACATG

GCTTTGAAGAATGACCTTGGACGAGTGTCTTGTAAGTTATTCTGATGATCCTAAACTTGTATTAAGTTTC

AAGACATACTATTTTAACTTAATCTCTCCTAAATTATATAGTAGACTATCTCCTTGTAATTGACCTTTGA

CTTGGATTTTCCAATTCAATGATCTATCAGGAGTTCTATCCTAAATAATGTTTCTAACATTGATTTCAGA

TATATTCTGATGTAGGAGTTTAATATGTAATTGTACTTTTGTAATTGCCCATTT

>Chamaedorea_elatior_DQ177734.1

CTACTCCTTTTACTGATGTCACAGTAAGTAAATCCATAGAACCTTCTTGCAAGCTTTACCTGAGAAGCCT

GTCGCAATTTAACTTGCTATGCTGGAACTTTTCTGCTTATTCAGTTTAAGAATTCTGAGGCACTGTATCT

GATGTTAAAGAAGATTAAGCTTACTGTTAAGGATTTGTACTAGATATGGTTTAATAAATATGTTTGASAA

CAGTTACCCCCCACCCCCACCCCCCTTCCCTCTCTGTCTAGAACCTTTCGACTTCTTACCTGGATTTGAT

TTTTTTGAGGGGAGTTTTTGCCTTTCAAGTTCATTTTTTTCCTCTGGATTAGTTAATTATAATACTATTA

TTTGTTGTCTGCTTTGAATATCTCTTTTGAAGTTCTTGCTGTGTAAGTTTACCCTGTAGACAGAAACATG

GCTTTGAAGAATGACCTTGGACGAGTGTCTTGTAAGTTATTCTGATGATCCTAAACTTGTATTAAGTTTC

AAGACATACTATTTTAACTTAGTCTCTCCTAAATTATATAGTAGACTATCTCCTTGTAATTGACCTTTGA

CTTGGATTTTCCAATTCAATGATCCATCAGGAGTTCTATCCTAAATAATGTTTCTAACATTGATTTCAGA

TATATTCTGATGTAGGAGTTTAATATGTAATTGTACTTTTGTAATTGCCCCATTT

>Chamaedorea_costaricana_DQ177732.1

CTACTCCTTTTACTGATGTCACAGTAAGTAAATCCATAGAACCTTCTTGCAAGCTTTACCTGAGAAGCCT

GTCGCAATTTAACTTGCTATGCTGGAACTTTTCTGCTTATTCAGTTTAAGAATTCTGAGGCACTGTATCT

GATGTTAAAGAAGATTAAGCTTACTGTTAAGGATTTGTACTAGATATGGTTTAATAAATATGTTTGAGAA

CAGTTACCCCCCACCCCCACCCCCCTTCCCTCTCTGTCTAGAACCTTTCGACTTCTTACCTGGATTTGAT

TTTTTTGAGGGGAGTTTTTGCCTTTCAAGTTCATTTTTTTCCTCTGGATTAGTTAATTATAATACTATTA

TTTGTTGTCTGCTTTGAATATCTCTTTTGAAGTTCTTGCTGTGTAAGTTTACCCTGTAGACAGAAACATG

GCTTTGAAGAATGACCTTGGACGAGTGTCTTGTAAGTTATTCTGATGATCCTAAACTTGTTTTAAGTTTC

AAGACATACTATTTTAACTTAGTCTCTCCTAAATTATATGGTAGACTATCTCCTTGTAATTGACCTTTGA

CTTGGATTTTCCAATTCAATGATCTATCAGGAGTTCTATCCTAAATAATGTTTCTAACATTGATTTCAGA

TATATTCTGATGTAGGAGTTTAATATGTAATTGTACTTTTGTAATTGCCCCATTT

>Chamaedorea_stenocarpa_gDQ177730.1

CTACTCCTTTTACTGATGTCACAGTAAGTAAATCCATAGAACCTTCTTGCAAGCTTTACCTGAGAAGCCT

GTCGCAATTTAACTTGCTATGCTGGAACTTTTCTGCTTATTCAGTTTAAGAATTCTGAGGCACTGTGTCT

GATGTTAAAGAAGATTAAGCTTACTGTTAAGGATTTGTACTAGATATGGTTTAATAAATATGTTTGAGAA

CAGTTACCCCCCACCCCCACCCCCCTTCCCTCTCTGTCTAGAACCTTTCGACTTCTTACCTGGATTTGAT

TTTTTTGAGGGGACTTTTTGCCTTTCAAGTTCATTTTTTTCCTCTGGATTAGTTAATTATAATACTATTA

TTTGTCGTCTGCTTTGAATATCTCTTTTGAAGTTCTTGCTGTGTAAGTTTACCCTGTAGACAGAAACATG

GCTTTGAAGAATGACCTTGGACGAGTGTCTTGTAAGTTATTCTGATGACCCTAAACTTGTTTTAAGTTTC

AAGACATACTATTTTAACTTAGTCTCTCCTAAATTATATGGTAGACTATCTCCTTGTAATTGACCTTTGA

CTTGGATTTTCCAATTCAATGATCTATCAGGAGTTCTATCCTAAATAATGTTTCTAACATTGATTTCAGA

TATATTCTGATGTAGGAGATTAATATGCAATTGTACTTTTGTAATTGCCCCATTT

>Chamaedorea_graminifolia_DQ177728.1

CTACTCCTTTTACTGATGTCACAGTAAGTAAATCCATGGAACCTTCTTGCAAGCTTTACCTGAGAAGCCT

GTCGCAATTTAGCTTGCTATGCCGGAACTTTTCTGCTTATTCAGTTTAAGAATTCTGAGGCACTGTATCT

GATGTTAAAGAAGATTAAGCTTACTGTTAAGGATTTGTACTAGATATGGTTTAATAAATATGTTTGAGAA

CAGTTACCCCCCACCCCCCTTCCCTCTCTGTCTAGAACCTTTCGACTTCTTACCTGGATTTGATTTTTTT

GAGGGGAGTTTTTGCCTTTCAAGTTCATTTTTTTCCTCTGGATTAGTTAATTATAATACTATTATTTGTT

GTCTGCTTTGAATATGTCTTTTGAAGTTCTTGCTGTGTAAGTTTACCCTGTAGACAGAAACATGGCTTTG

AAGAATGACCTTGGACGAGTGTCTTGTAAGTTATTCTGATGATCCTAAACTTGTATTAAGTTTCAAGACA

TACTATTTAGTCTCTCCTAAATTATATAGTAGACTATCTCCTTGTAATTGATCTTTGACTTGGATTTTCC

AATTCAATGATCTATCAGGAGTTCTATCCTAAATAATGTTTCTAACATCGATTTCAGATATCTTCTGATG

TAGGAGTTTAATATGTAATTGTACTTGTGTAATTGCCCCATTT

>Chamaedorea_amabilis_DQ177726.1

CTACTCCTTTTACTGATGTCACAGTAAGTAAATCCATAGAACCTTCTTGCAAGCTTTACCTGAGAAGCCT

GTCGCAATTTACCTTGCTATGCTGGAACTTTTCTGCTTATTCAGTTTAAGAATTCTGAGGCACTGTATCT

GATGTTAAAGAAGATTAAGCTTGCTGTTAAGGATTTGTACTAGATATGGTTTAATAAATATGTTTGAGAA

CAGTTACCCCCCACCCCCAACCCCCTTCCCTCTCTGTCTAGAACCTTTCGACTTCTTACCTGGATTTGAT

TTTTTTTGAGGGGAGTTTTTGCCTTTCAAGTTAATTTTTTTCCTCTGGATTAGTTAATTATAATACTATT

ATTTGTTGTCTGCTTTGAATATCTCTTTTGAAGTTCTTGCTGTGTAAGTTTACCCTGTAAACAGAAACAT

GGCTTTGAAGAATGACCTTGGACGAGTGTCTTGTAAGTTATTCTGATGATCCTAAACTTGTATTAAGTTT

CAAGACATACTATTTTAACTTAGTCTCTCCTAAATTATATAGTAGACTATCTCCTTGTAATTGATCTTTG

ACTTGGATTTTCCAATTCAATGATCTACCAGGAGTTCTATCCTAAATAATGTTTCTAACATTGATTTCAG

ATATATTCTGATGTAGGAGTTTAATATGTAATTGTACTTTTGTAATTGCCCCATTT

>Chamaedorea_radicalis_DQ177724.1

CTACTCCTTTTACTGATGTCACAGTAAGTAAATCCATAGAACCTTCTTGCAAGCTTTACCTGAGAAGCCT

GTCGCAATTTAACTTGCTATGCTTGAACTTTTCTGCTTATTCAGTTTAAGAATTCTGAGGCACTGTATCT

GATGTTAAAGAAGATTAAGCTTACTGTTAAGGATTTGTACTAGATATGGTTTAATAAATAAGTTTGAGAA

CAGTTACCCCCCACCCCCACCCCCCTTCCCTCTCTGTCTAGAACCTTTCGACTTCTTACCTGGTTTTGAT

TTTTTTGAGGGGAGTTTTTGCCTTTCAAGTTCATTTTTTTCCTCTGGATTAGTTAATTATAATACTATTA

TTTGTTGTCTGCTCTGAATATCTCTTTTGAAGTTCTTGCTGTGTAAGTTTACCCTGTAGACAGAAACATG

GCTTTGAAGAATGACCTTGGACGAGTGTCTTGTAAGTTATTCTGATGATCCTAAACTTGTATTAAGTTTC

AAGACATACTATTTTAACTTAGTCTCTCCTAAATTATATAGTAGACTATCTCCTTGTAATTGACCTTTGA

CTTGGATTTTCCAATTCAATGATCTATCAGGAGTTCTATCCTAAATAATGTTTCTAACATTGATTTCAGA

TATATTCTGATGTAGGAGTTTAATATGTATTTGTACTTTTGTAATTGCCCCATTT

>Chamaedorea_allenii_DQ177722.1

CTACTCCTTTTACTGATGTCACAGTAAGTAAATCCATAGAACCTTCTTGCAAGCTTTACCGGAGAAGCCT

GTCGCAATTTAACTTGCTATGCTGGAACTTTTCTGCTTATTCAGTTTAAGAATTCTGAGGCACTGTATCT

GATGTTAAAGAAGATTAAGCTTACTGTTAAGGATTGTACTAGATATGGTTTAATAAATATGTTTGAGAAC

AGTTGCCCCCCACCCCCACCCCCCTTCCCTCTCTGTCTAGAACCTTTCGACTTCTTACCTGGATTTGATA

TTTTTGAGGGGAGTTTTTGCCTTTCAAGTTCATTTTTTTCCTCTGGATTAGTTAATTATAATACTATTAT

TTGTTGTCTGCTTTGAATATCTCTTTTGAAGTTCTTGCTGTGTAAGTTTACCCTGTAGACAGAAACATGG

CTTTGAAGAATGACCTTGGACGAGTGTCTTGTAAGTTATTCTGATGATCCTAAACTTGTATTAAGTTTCA

AGACATACTATTTTAACTTAATCTCTCCTAAATTATATAGTAGACTATCTCCTTGTAATTGACCTTTGAC

TTGGATTTTCCAATTCAATGATCTATCAGGAGTTCTATCCTAAATAATGTTTCTAACATTGATTTCAGAT

ATATTATGATGTAGGAGTTTAATATGTAATTGTACTTTTGTAATTGCCCCATTT

>Chamaedorea_stricta_DQ177720.1

CTACTCCTTTTACTGATGTCACAGTAAGTAAATCCATAGAACCTTCTTGCAAGCTTTACCTGAGAAGCCT

GTCGCAATTTAACTTGCTATGCTGGAACTTTTCTGCTTATTCAGTTTAAGAATTCTGAGGCACTGTATCT

GATGTTAAAGAAGATTAAGCTTACTGTTAAGGATTTGTACTAGATATGGTTTAATAAATATGTTTGAGAA

CAGTTACCCCCCACCCCCACCCCCCTTCCGTCTCTGTCTAGAACCTTTCGACTTCTTACCTGGATTTGAT

TTTTTTGAGGGGAGTTTTTGCCTTTCAAGTTCATTTTTTTCCTCTGGATTAGTTAATTATAATACTATTA

TATGTTGTCTGCTTTGAATATCTCTTTTGAAGTTCTTGCTGTGTAAGTTTACCCTGTAGACAGAAACATG

GCTTTGAAGAATGACCTTGGACGAGTGTCTTGTAAGTTATTCTGATGATCCTAAACTTGTATTAAGTTTC

AAGACATACTATTTTAACTTAGTCTCTCCTAAATTATATAGTAGACTATCTCCTTGTAATTGACCTTTGA

CTTGGATTTTCCAATTCAATGATCTATCAGGAGTTCTATCCTAAATAATGTTTCTAACATTGATTTCAGA

TATATTCTGATGTAGGAGTTTAATATGTAATTGTACTTTTGTAATTGCCCCATTT

>Chamaedorea_dammeriana_DQ177718.1

CTACTCCTTTTACTGATGTCACAGTAAGTAAATCCATAGAACCTTCTTGCAAGCTTTACCTGAGAAGCCT

GTCGCAATTTAACTTGCTATGCTGGAACTTTTCTGCTTATTCAGTTTAAGAATTCCGAGGCACTGTATCT

GATGTTAAAGAAGATTAAGCTTACTGTTAAGGATTTGTACTAGATATGGTTTAATAAATATGTTTGAGAA

CAGTTACCCCCCACCCCCCTTCCCTCTCTGTCTAGAACCTTTCGACTTCTTACCTGGATTTGATTTTTTT

GAGGGGAGTTTTTGCCTTTCAAGTTCATTTTTTTCCTCTGGATTAGTTAATTATTATACTATTATTTGTT

GTCTGCTTTGAATATCTCTTTTGAAGTTCTTGCTGTGTAAGTTTACCCTGTAGACAGAAACATGGCTTTG

AAGAATGACATTGGACGAGTGTCTTGTAAGTTATTCTGATGATCCTAAACTTGTTTTAAGTTTCAAGACA

TACTATTTTAACTTAGTCTCTCCTAAATTATATGGTAGACTATCTCCTTGTAATTGACCTTTGACTTGGA

TTTTCCAATTCAATGATCTATCAGGAGTTCTATCCTAAATAATGTTTCTAACATTGATTTCAGATATATT

CTGATGTAGGAGTTTAATATGTAATTGTACTTTTGTAATTGCCCCATTT

>Chamaedorea_glaucifolia_DQ177716.1

CTACTCCTTTTACTGATGTCACAGTAAGTAAATCCATAGAACCTTCTTGCAAGCTTTACCTGAGAAGCCT

GTCGCAATTTAACTTGCTATGCTGGAACTTTTCTGCTTATTCAGTTTAAGAATTCTGAGGCACTGTATCT

GATGTTAAAGAAGATTAAGCTTACTGTTAAGGATTTGTACTAGATATGGTTTAATAAATATGTTTGAGAA

CAGTTACCCCCCACCCCCACCCCCCTTCCCTCTCTGTCTAGAACCTCTCGACTTCTTACCTGGATTTGAT

TTTTTTGAGGGGAGTTTTTGCCTTTCAAGTTCATTTTTTTCCTCTGGATTAGTTAATTATAATACTATTA

TTTGTTGTCTGCTTTGAATATCTCTTTTGAAGTTCTTGCTGTGTAAGTTTACCCTGTAGACAGAAACATG

GCTTTGAAGAATGACCTTGGACGAGTGTCTTGTAAGTTATTCTGATGATCCTAAACTTGTATTAAGTTTC

AAGACATACTATTTTAACTTAGTCTCTCCTAAATTATATAGTAGACTATCTCCTTGTAATTGACCTTTGA

CTTGGATTTTCCAATTCAATGATCTATCAGGAGTTCTATCCTAAATAATGTTTCTAACATTGATTTCAGA

TATATTCTGATGTAGGAGTTTAATATGTAATTGTACTTTTGTAATTGCCCCATTT

>Chamaedorea_crucensis_DQ177714.1

CTACTCCATTTACTGATGTCACAGTAAGTAAATCCATAGAACCTTCTTGCAAGCTTTACCGGAGAAGCCT

GTCGCAATTTAACTTGCTATGCTGGAACTTTTCTGCTTATTCAGTTTAAGAATTCTAAGGCACTGTATCT

GATGTTAAAGAAGATTAAGCTTACTGTTAAGGATTGTACTAGATATGGTTTAATAAATATGTTTGAGAAC

AGTTGCCCCCCCMMCCCCCMCCCCCYTYCCYYYYYKKYYWRRAMCYTTTSRVYTYYTKSCYKGRWTTKRT

TTTTTTTGAGGGGAGTTTTTGCCTTTCAAGTTCATTTTTTTCCTCTGGATTAGTTAATTATAATACCATT

ATTTGTTGTCTGCTTTGAATATCTCTTTTGAAGTTCTTGCTGTGTAAGTTTACCCTGTAGACAGAAACAT

GGCTTTGAAGAATGACCTTGGACGAGTGTCTTGTAAGTTATTCTGATGATCCTAAACTTGTATTAAGTTT

CAAGACATACTATTTTAACTTAATCTCTCCTAAATTATATAGTAGACTATCTCCTTGTAATTGACCTTTG

ACTTGGATTTTCCAATTCAATGATCTATCAGGAGTTCTATCCTAAATAATGTTTCTAACATTGATTTCAG

ATATATTCTGATGTAGGAGTTTAATATGTAATTGTACTTTTGTAATTGCCCATTT

>Chamaedorea_adscendens_DQ177712.1

CTACTCCTTTTACTGATGTCACAGTAAGTAAATCCATAGAACCTTCTTGCAAGCTTTACCTGAGAAGCCT

GTCGCAATTTAACTTGCTATGCTGGAACTTTTCTGCTTATTCAGTTTAAGAATTCTGAGGCACTGTATCT

GATGTTAAAGAAGATTAAGCTTACTGTTAAGGATTTGTACTAGATATGGTTTAATAAATATGTTTGAGAA

CAGTTACCCCCCCACCCCCCTTCCCTCTCTGTCTAGAACCTTTCGACTTCTTACCTGGATTTGATTTTTT

TGAGGGGAGTTTTTGCCTTTCAAGTTCATTTTTTTCCTCTGGATTAGTTAATTATAATACTATTATTTGT

TGTCTGCTTTGAATATCTCTTTTGAAGTCCTTGCTGTGTAAGTTTACCCTGTAGACAGAAACATGGCTTT

GAAGAATGACCTTGGACGAGTGTCTTGTAAGTTATTCTGATGATCCTAAACTTGTTTTAAGTTTCAAGAC

ATACTATTTTAACTTAGTCTCTCCTAAATTATATGGTAGACTATCTCCTTGTAATTGACCTTTGACTTGG

ATTTTCCAATTCAATGATCTATCAGGAGTTCTATCCTAAATAATGTTTCTAACGTTGATTTCAGATATAT

TCTGAAGTATGAGTTTAATATGTAATTGTACTTT

>Chamaedorea_cataractarum_DQ177710.1

CTACTCCTTTTACTGATGTCACAGTAAGTAAATCCATTGAACCTTCTTGCAAGCTTTACCGGAGAAGCCT

GTCGCAATTTAACTTGCTATGCTGGAACTTTTCTGCTTATTCAGTTTAAGAATTCTGAGGCACTGTATCT

GATGTTAAAGAAGATTAAGCTTACTGTTAAGGATTGTACTAGATATGGTTTAATAAATATGTTTGAGAAC

AGTTGCCCCCCACCCCCACCCCCCTTCCCTCTCTGTCTAGAACCTTTTGACTTCTTACCTGGATTTGATT

TTTTTGAGGGGAGTTTTTGCCTTTCAAGTTCATTTTTTTCCTCTGGATTAGTTAATTATAATACCATTAT

ATGTTGTCTGCTTTGAATATCTCTTTTGAAGTTCTTGCTGTGTAAGTTTACCCTGTAGACAGAAACATGG

CTTTGAAGAATGACCTTGGACGAGTGTCTTGTAGGTTATTCTGATGATCCTAAACTTGTATTAAGTTTCA

AGACATACTATTTTAACTTAATCTCTCCTAAATTATATAGTAGACTATCTCCTTGTAATTGACCTTTGAC

TTGGATTTTCCAATTCAATGATCTATCAGGATTTCTATCCTAAATAATGTTTCTAACATTGATTTCAGAT

ATATTCTGATGTAGGAGTTTAATATGTAATTGTACTTTTGTAATTGCCCCATTT

>Chamaedorea_fragrans_DQ177708.1

CTACTCCTTTTACTGATGTCACAGTAAGTAAATCCATAGAACCTTCTTGCAAGCTTTACCTGAGAAGCCT

GTTGCAATTTAACTTGCTATGCTGGAACTTTTCTGCTTATTCAGTTTAAGAATTCTGAGGCACTGTATCT

GATGTTAAAGAAGATTAAGCTTACTGTTAAGGATTTGTACTAGATATGGTTTAATAAATATGTTTGAGAA

CAGTTACCCCCCACCCCCACCCCCCTTCCTTCTCTGTCTAGAACCTTTCGACTTCTTACCTGGATTTGAT

TTTTTTGAGGGGAGTTTTTGCTTTTCAAGTTCATTTTTTTCCTCTGGATTAGTTAATTATAATACTATTA

TTTGTTGTCTGCTTTGAATATCTCTTTTGAAGTTCTTGCTGTGTAAGTTTACCCTGTAGACAGAAACATG

GCTTTGAAGAATGACCTTGGACGAGTGTCTTGTAAGTTATTCTGATGATCCTAAACTTGTATTAAGTTTC

AAGACATACTATTTTAACTTAGTCTCTTTTAAATTATATAGTAGACTATCTCCTTGTAATTGACCTTTGA

CTTGGATTTTGCAATTCAATGATCTATCAGTTCTATCCTAAATAATGTTTCTAACATTGATTTCAGATAT

ATTCTGATGTTGGAGTTTAATATGTAATTGTACTTTTGTAATTGCTCCATTT

>Chamaedorea_neurochlamys_DQ177706.1

GTAAATCCATAGAACCTTCTTGCAAGCTTTACCTGAGAAGCCTGTCGCAATTTAACTTGCTATGCTGGAA

CTTTTCTGCTTATTCAGTTTAAGAATTCTGAGGCACTGTATCTGATGTTAAAGAAGATTAAGCTTACTGT

TAAGGATTTGTACTAGATATGGTTTAATAAATATGTTTGAGAACAGTTAACCCCCCACCCCCACCCCCAC

CCCCACCCCCACCCCCCTTCCCTCTCTGTCTAGAACCTTTCGACTTCTTACCTGGATTTGATTTTTTTGA

GGGGAGTTTTTGCCTTTCAAGTTCATTTTTTTCCTCTGGATTAGTTAATTATAATACTATTATTTGTTGT

CTGCTTTGAATATCTCTTTTGAAGTTCTTGCTGTGCAAGTTTACCCTGTAGACAGGAACATGGCTTTGAA

GAATGACCTTGGACGAGTGTCTTGTAAGTTATTCTGATGATCCTAAACTTGTATTAAGTTTCAAGACATA

CTATTTTAACTTAGTCTCTCCTAAATTATATAGTAGACTATCTCCTTGTAATTGACCTTTGACTTGGATT

TTCCAATTCAATGATCTATCAGGAGTTCTATCCTAAATAATGTTTCTAACATTGATTTCAGATATATTCT

GATGTAGGAGTTTAATATGTAATTGTACTTTTGTAATTGCCCCATTT

>Chamaedorea_tepejilote_DQ177704.1

CTACTCCTTTTACTGATGTCACAGTAAGTAAATCCATAGAACCTTCTTGCAAGCTTTACCGGAGAAGCCT

GTCGCAATTTAACTTGCTATGCTGGAACTTTTCTGCTTATTCAGTTTAAGAATTCTGAGGCACTGTATCT

GATGTTAAAGAAGATTAAGCTTACTGTTAAGGATTGTACTAGATATGGTTTAATAAATATGTTTGAGAAC

AGTTGCCCCCCACCCCCACCCCCACCCCCCTTCCCTCTCTGTCTAGAACCTTTCGACTTCTTACCTGGAT

TTGATTTTTTTGAGGGGAGTTTTTGCCTTTCAAGTTCATTTTTTTCCTCTGGATTAGTTAATTATAATAC

TATTATTTGTTGTCTGCTTTGAATATCTCTTTTGAAGTTCTTGCTGTGTAAGTTTACCCTGTAGACAGAA

ACATGGCTTTGAAGAATGACCTTGGACGAGTGTCTTGTAAGTTATTCTGATGATCCTAAACTTGTATTAA

GTTTCAAGACATACTATTTTAACTTAATCTCTCCTAAATTATATAGTAGACTATCTCCTTGTAATTGACC

TTTGACTTGGATTTTCCAATTCAATGATCTATCAGGAGTTCTATCCTAAATAATGTTTCTAACATTGATT

TCAGATATATTCTGATGTAGGAGTTTAATATGTAATTGTACTTTTGTAATTGCCCCATTT

>Chamaedorea_klotzschiana_DQ177702.1

CTACTCCTTTTACTGATGTCACAGTAAGTAAATCCATAGAACCTTCTTGCAAGCTTTACCTGAGAAGCCT

GTCGCAATTTAACTTGCTATGCTGGAACTTTTCTGCTTATTCAGTTTAAGAATTCTGAGGCACTGTATCT

GATGTTAAAGAAGATTAAGCTTACTGTTAAGGATTTGTACTAGATATGGTTTAATAAATATGTTTGAGAA

CAGTTACCCCCCAATCCACCCCCCTTCCCTCTCTGTCTAGAACCTTTCGACTTCTTACCTGGATTTGATT

TTTTTGAGGGGAGTTTTTGTCTTTCAAGTTCATTTTTTTCCTCTGGATTAGTTAATTATAATACTATTAT

TTGTTGTCTGCTTTGAATATCTCTTTTGAAGTTCTTGCTGTGTAAGTTTACCCTGTAGACAGAAACATGG

CTTTGAAGAATGACCTTGGACGAGTGTCTTGTAAGTTATTCTGATGATCCTAAACTTGTATTAAGTTTCA

AGACATACTATTTTAACTTAGTCTCTCCTAAATTATATAGTAGACTATCTCCTTGTAATTGACCTTTGAC

TTGGATTTTCCAATTCAATGATCTATCAGGAGTTCTATCCTAAATAATGTTTCTAACATTGATTTCAGAT

ATATTCTGATGTAGGAGTTTAATATGTAATTGTACTTTTGTAATTGCCCCATTT

>Gaussia_maya_DQ177815.1

GTCTTGAGAGCATCAAAGCTAGCATTGGAGCTCGAAAGCTCGACTTTGATGCTTATGTTGGTATGTCACT

CTGCCACGCTGCCTAGTTTTTAGTACTTGAAGAATTGGCAGAGCGTTCATTTGTTGGTATAATATAGAGC

AATTTCATTAGTTTTGATTGCCGAATGTAGTAATATACTTCAATATCTATCAAATGCTTCATTAAAAAAT

AAAAAAATAAAAAAACTCACTGACAAAGTATCTGATCCATGAGTCAAATAATGCACAAAACATACTGGAT

ACCTTTTTCTGAAAAACGAAAGAGGGAGAGCAAACCCCACCCGATTCATGGAGATAATGAGACAATTTTA

TGTCTCATGTTCTTTGGATATCAGAATACATGTCTTTTGTCTTTAGCGCTCTTGGATTGCTGACAGCTTA

TAGATTGCTACCCTTGGCAGACCTGCAGAAGCAATATGCTGATGTCGTGATCGAAGTTTTGCCGACACAA

TTAATTCCTGACGACAATGAAAGGAAGGTGCTCAGAGTTCGATTGGTGATGAAGGAAGGGGTGAAGTATT

GCAATCCAGTTTACCTCTTTGACGAAGGCTCCACCG

>Chamaedorea_oblongata_DQ177813.1

GTCTTGAGTGCATCAAAGCCAGCATTGGAGCTCGAAAGCTCGACTTTGATGCTTATGTTGGTATGTTTCT

TTGCCACGGTGCCTAGTTATTGGTACTTGAAGAATTGGCAGAGCATTCATTTGTTGGTATAATATAGAGC

AATTTCATTAGTTTTGATTGCCAAATATAGTACTATACTTCAATATCTAGCAAATGCTTCATTAAAAAAA

GAACTCGCTAACAAAGTATCTGATCCATAAGTCAAATAATGCACAAAACATACTGGACTAAAAATATAAT

CACTCCATGAGAAATACTTTTGGATACCTTTTTCTGAAAAAAAGAAAGAGGGAGAGCAAACCCCAACCAA

TTCATTGAGATAATGAGACAATTTTATGTGTGGAGTCTCATGTTCTTCAGATATCAGAATACATGTCTTT

TTCTTTAGCACTCTTGGATTGCTGGCAGCTTATTGATTGCTATCCTTGGCAGACCTGCAGAAGCAATATG

CTGATGTCGTGATCGAAGTTTTACCGACACAATTAATTCCTGATGACAATGAAAGGAAGGTGGTGAGAGT

TCGATTGGTGATGAAGGAAGGGGTGAAGTATTGCAATCCAGTTTATCTCTTAGATGAAGGCTCCACCG

>Chamaedorea_subjectifolia_DQ177811.1

GTCTTGAGTGCATCAAAGCCAGCATTGGAGCTCGAAAGCTCGACTTTGATGCTTATGTTGGTATGTTTCT

TTGCCACGGTGCCTAGTTATTGGTACTTGAAGAATTGGCAGAGCATTCATTTGTTGGTATAATATAGAGC

AATTTCATTAGTTTTGATTGCCAAATATAGTACTATACTTCAATATCTAGCAAATGCTTCATTAAAAAAA

GAACTCGCTAACAAAGTATCTGATCCATAAGTCAAATAATGCACAAAACATACTGGACTAAAAATATAAT

CACTCCATGAGAAATACTTTTGGATACCTTTTTCTGAAAAAAAGAAAGAGGGAGAGCAAACCCCAACCAA

TTCATTGAGATAATGAGACAATTTTATGTGTGGAGTCTCATGTTCTTCAGATATCAGAATACATGTCTTT

TTCTTTAGCACTCTTGGATTGCTGGCAGCTTATTGATTGCTATCCTTGGCAGACCTGCAGAAGCAATATG

CTGATGTCGTGATCTAAGTTTTACCGACACTATTAATTCCTGATGACAATGAAAGGAAGGTGCTGAGAGT

TCGATTGGTGATGAAGGAAGGGGTGAAGTATTGCAATCCAGTTTATCTCTTAGATGAAGGCTCCACCG

>Chamaedorea_rossteniorum_DQ177809.1

GTCTTGAGTGCATCAAAGCCAGCATTGGAGCTCGAAAGCTCGACTTTGATGCTTATGTTGGTATGTTTCT

TTGCCACGGTGCCTAGTTATTGGTACTTGAAGAATTGGCAGAGCATTCATTTGTTGGTATAATATAGAGC

AATTTCATTAGTTTTGATTGCCAAATATAGTACTATACTTCAATATCTAGCAAATGCTTCATTAAAAAAA

GAACTCGCTAACAAAGTATCTGATCCATAAGTCAAATAATGCACAAAACATACTGGACTAAAAATATAAT

CACTCCATGAGAAATACTTTTGGATACCTTTTTCTGAAAAAAAGAAAGCGGGAGAGCAAACCCCAGCCAA

TTCATTGAGATAATGAGACAATTTTATGTGTGGAGTCTCATGTTCTTCAGATATCAGAATACATGTCTTT

TTCTTTAGCACTCTTGGATTGCTGGCAGCTTATTGATTGCTATCCTTGGCAGACCTGCAGAAGCAATATG

CTGATGTCGTGATCGAAGTTTTACCGACACAATTAATTCCTGATGACAATGAAAGGAAGGTGCTGAGAGT

TCGATTGGTGATGAAGGAAGGGGTGAAGTATTGCAATCCAGTTTATCTCTTAGATGAAGGCTCCACCG

>Chamaedorea_parvisecta_DQ177807.1

GTCTTGAGTGCATCAAAGCCAGCATTGGAGCTCGAAAGCTCGACTTTGATGCTTATGTTGGTATGTTTCT

TTGCCACAGTGCCTAGTTATTGGTACTTGAAGAATTGGCAGAGCATTCATTTGTTGGTATAATATAGAGC

AATTTCATTAGTTTTGATTGCCAAATATAGTACTATACTTCAATATCTAGCAAATGCTTCATTAAAAAAA

GAACTCACTAACAAAGTATCTGATCCATAAGTCAAATAATGCACAAAACATACTGGACTAAAAATATAAT

CACTCCATGAGAAATACTTTTGGATACCTTTTTCTGAAAAAAAGAAAGAGGGAGAGCAAACCCCAACCAA

TTCATTGAGATAATGAGATAATTTTATGTGTGGAGTCTCATGTTCTTCAGATATCAGAATACATGTCTTT

TTCTTTAGCACTCTTGGATTGCTGGCAGCTTATAGATTGCTACCCTTGGCAGACCTGCAGAAGCAATATG

CTGATGTCGTGATCTCGAAGTTTTACCGACACAATTAATTCCTGATGACAATGAAAGGAAGGTGCTGAGA

GTTCGATTGGTGATGAAGGAAGGGGTGAAGTATTGCAATCCAGTTTACCTCTTAGATGAAGGCTCCACCG

>Chamaedorea_foveata_DQ177805.1

GTCTTGAGTGCATCAAAGCCAGCATTGGAGCTCGAAAGCTCGACTTTGATGCTTATGTTGGTATGTTTCT

TTGCCACAGTGCCTAGTTATTGGTACTTGAAGAATTGGCAGAGCATTCCTTTGTTGGTATAATATAGAGC

AATTTCATTAGTTTTGATTGCCAAATATAGTACTATACTTCAATATCTAGCAAATGCTTCATTAAAAAAA

GAACTCACTAACAAAGTATCTGATCCATAAGTCAAATAATGCACAAAACATACTGGACTAAAAATATAAT

CACTCCATGAGAAATACTTTTGGATACCTTTTTCTGAAAAAAAGAAAGAGGGAGAGCAAACCCCAACCAA

TTCATTGAGACAATGAGACAATTTTATGTGTGGAGTCTCATGTTCTTCAGATATCAGAATACATGTCTTT

TTCTTTAGCACTCTTGGATTGCTGGCAGCTTATAGATTGCTGGCAGCTTATTGATTGCTACCCTTGGCAG

ACCTGCAGAAGCAATATGCTGATGTCGTGATCGAAGTTTTACCGACACAATTAATTCCTGATGACAATGA

AAGGAAGGTGCTGAGAGTTCGATTGGTGATGAAGGAAGGGGTGAAGTATTGCAATCCAGTTTACCTCTTA

GATGAAGGCTCCACCG

>Chamaedorea_nubium_DQ177803.1

GTCTTGAGTGCATCAAAGCCAGCATTGGAGCTCGAAAGCTCGACTTTGATGCTTATGTTGGTATGTTTCT

TTGCCACAGTGCCTAGTTATTGGTACTTGAAGAATTGGCAGAGCATTCATTTGTTGGTATAATATAGAGC

AATTTCATTAGTTTTGATTGCCAAATATAGTACTATACTTCAATATCTAGCAAATGCTTCATTAAAAAAA

GAACTCGTTAACAAAGTATCTGATCCATAAGTCAAATAATGCACAAAACATACTGGACTAAAAATATAAT

CACTCCATGAGAAATACTTTTGGATACCTTTTTCTGAAAAAAAGAAAGAGGGAGAGCAAACCCCAACCAA

TTCATTGAGATAATGAGACAATTTTATGTGTGGAGTCTCATGTTCTTCAGATATCAGAATACATGTCTTT

TTCTTTAGCACTCTTGGATGGCTGGCAGCTTATAGATTGCTACCCTTGGCAGATCTGCAGAAGCAATATG

CTGATGTCGTGATCGAAGTTTTACCGACACAATTAATTCCTGATGACAATGAAAGGAAGGTGCTGAGAGT

TCGATTGGTGATGAAGGAAGGGGTGAAGTATTGCAATCCAGTTTACCTCTTAGATGAAGGCTCCACCG

>Chamaedorea_ernesti_augustii_DQ177801.1

GTCTTGAGTGCATCAAAGCCAGCATTGGAGCGCGAAAGCTCGACTTTGATGCTTATGTTGGTATGTTTCT

TTGCCACAGTGCCTAGTTATTGGTACTTGAAGAATTGGCAGGGCATTCATTTGTTGGTATAATATAGAGC

AATTTCATTAGTTTTGATTGCCAAATATAGTACTATACTTCAATATCTAGCAAATGCTTCATTAAAAAAA

AAAACTCACTAACAAAGTATCTGATCCATAAGTCAAATAATGCACAAAACATACTGAACTAAAAATATAA

TCACTCCATGAGAAATACTTTTGGATACCTTTTTCTGAAAAAAAGAAAGAGGGAGAGCAAACCCCAACCA

ATTCATTGAGATAATGAGACAATTTTATGTGTGGAGTCTCATGTTCTTCAGATATCAGAATACATGTCTT

TTTCTTTAGCACTCTTGGATTGCTGGCAGCTTATAGATTGCTACCCTTGGCAGACCTGCAGAAGCAATAT

GCTGATGTCGTGATCGAAGTTTTACCGACACAATTAATTCCTGATGACAATGAAAGGAAGGTGCTGAGAG

TTCGATTGGTGATGAAGGAAGGGGTGAAGTATTGCAATCCAGTTTACCTCCTTAGATGAAGGCTCCACCG

>Chamaedorea_warscewiczii_DQ177799.1

GTCTTGAGTGCATCAAAGCCAGCATTGGAGCTCGAAAGCTCGACTTTGATGCTTATGTTGGTATGTTTCT

TTGCCACGGTGCCTAGTTATTGGTACTTGAAGAATTGGCAGAGCATTCATTTGTTGGTATAATATAGAGC

AATTTCATTAGTTTTGATTGCCAAATATAGTACTATACTTCAATATCTAGCAAATGCTTCATTGAAAAAA

GAACTCGCTAACAAAGTATCTGATCCATAAGTCAAATAATGCACAAAACATACTGGACTAAAAATATAAT

CACTCCATGAGAAATACTTTTGTATACCTTTTTCTGAAAAAAAGAAAGAGGGAGAGCAAACCCCAACCAA

TTCATTGAGATAATGAGACAATTTTATGTGTGGAGTCTCATGTTCTTCAGATATCAGAATACATGTCTTT

TTCTTTAGCACTCTTGGATTGCTGGCAGCTTATTGATTGCTATCCTTGGCAGACCTGCAGAAGCAATATG

CTGATGTCGTGATCGAAGTTTTACCGACACAATTAATTCCTGATGACAATGAAAGGAAGGTGCTGGGAGT

TCGATTGGTGATGAAGGAAGGGGTGAAGTATTGCAATCCAGTTTATCTCTTAGATGAAGGCTCCACCG

>Chamaedorea_pumila_DQ177797.1

GTCTTGAGTGCATCAAAGCCAGCATTGGAGCTCGAAAGCTCGACTTTGATGCTTATGTTGGTATGTTTCT

TTGCCACAGTGCCTAGTTATTGGTACTTGAAGAATTGGCAGAGCATTCATTTGTTGGTAAAATATAGAGC

AATTTCATTAGTTTTGATTGCCAAATATAGTACTATACTTCAATATCTAGCAAATGCTTCATTAAAAAAA

GAACTCACTAACAAAGTATCTGATCCATAAGTCAAATAATGCACAAAACATACTGGACTAAAAATATAAT

CACTCCATGAGAAATACTTTTGGATACCTTTTTCTGAAAAAAAGAAAGAGGGAGAGCAAACTCCAACCAA

TTCATTGAGATAATGAGATAATTTTATGTGTGGAGTCTCGTGTTCTTCAGATATCAGAAAACATGTCTTT

TTCTTTAGCACTCTTGGATTGCTGGCAGCTTATAGATTCCTACCCTTGGCAGACCTGCAGAAGCAATATG

CTGATGTCGTGATCGAAGTTTTACCGACACAATTAATTCCTGATGACAATGAAAGGAAGGTGCTGAGAGT

TCGATTGGTGATGAAGGAAGGGGTGAAGTATTGCAATCCAGTTTACCTCTTAGATGAAGGCTCCACCG

>Chamaedorea quezalteca_DQ177795.1

GTCTTGAGTGCATCAAAGCCAGCATTGGAGCTCGAAAGCTCGACTTTGATGCTTATGTTGGTATGTTTCT

TTGCCACAGTGCCTAGTTATTGGTACTTGAAGAATTGGCAGAGCATTCATTTGTTGGTATAATATAGAGC

AATTTCATTAGTTTTGATTGCCAAATATAGTACTATACTTCAATATCTAGCAAATGCTTCATTAAAAAAA

GAACTCACTAACAAAGTATCTGATCCATAAGTCAAATAATGCACAAAACATACTGGACTAAAAATATAAT

CACTCCATGAGAAATACTTTTGGATACCTTTTTCTGAAAAAAAGAAAGAGGGAGAGCAAACCCCAACCAA

TTCATTGAGATAATGAGATAATTTTATGTGTGGAGTCTCATGTTCTTCAGATATCAGAATACATGTCTTT

TTCTTTAGCACTCTTGGATTGCTGGCAGCTTATAGATTGCTACCCTTGGCAGACCTGCAGAAGCAATATG

CTGATGTCGTGATCTCGAAGTTTTACCGACACAATTAATTCCTGATGACAATGAAAGGAAGGTGCTGAGA

GTTCGATTGGTGATGAAGGAAGGGGTGAAGTATTGCAATCCAGTTTACCTCTTAGATGAAGGCTCCACCG

>Chamaedorea_coralliformis_DQ177793.1

GTCTTGAGTGCATCAAAGCCAGCATTGGAGCTCGAAAGCTAGACTTTGATGCTTATGTTGGTATGTTTTT

TTGCCACAGTGCCTAGTTATTGGTACTTGAAGAATTGGCAGAGCATTTATTTGTTGGTATAATATAGAAC

AATTTCATTAGTTTTGATTGCCAAATATAGTACTATACTTCAATATCTAGCAAACGCTTCATTAAAAAAA

GAACTCGCTAACAAAGTATCTGATCCATAAGTCAAATAATGCACAAAACATACTGGACTAAAAATATAAT

CACTCCATGAGAAATACTTTTGGATACCTTTTTCTGGAAAARAGAAAGAGGGAGAGCAAACCCCAACCAA

TTCATTGAGATAATTAGACAATTTTATGTGTGGAGTCTCGTGTTCTTCAGATATCAGAATACATGTCTTT

TTCTTTAGCACTCTTGGATTGCTGGCAGCTTATAGATTGCTGGCAGCTTATTGATTGCTACCCTTGGCAG

ACCTGCAGAAGCAATATGCTGATGTCGTGATCGAAGTTTTACCGACACAATTAATTCCTGATGACAATGA

AAGGAAGGTGCTGAGAGTTCGATTGGTGATGAAGGAAGGGGTGAAGTATTGCAATCCAGTTTACCTCTTA

GATGAAGGCTCCACCG

>Chamaedorea_linearis_DQ177791.1

GTCTTGAGTGCATCAAAGCCAGCATTGGAGCTCGAAAGCTCGACTTTGATGCTTATGTTGGTATGTTTCT

TTGCCACAGTGCCTAGTTATTGGTACTTGAAGAATTGGCAGAGCATTCATTTGTTGGTATAATATAGAGC

AATTTCATTAGTTTTGATTGCCAAATATAGTACTATACTTCAATATCTAGCAAATGCTTCATTAAAAAAA

GAACTCGCTAACAAAGTATCTGATCCATAAGTCAAATAATGCACAAAACATACTGGACTAAAAAAATAAT

CACTCCATGAGAAATACTTTTGGATACCTTTTTCTGAAAAAAAGAAAGAGGGAGAGCAAACCCCAACCAA

TTCATTGAGATAATGAGACAATTTTATGTGTGGAGTCTCGTGTTCTTCAGATATCAGAATACACGTCTTT

TTCTTTAGCACTCTTGGATTGCTGGCAGCTTATAGATTGCTACCCTTGGCAGACCTGCAGAAGCAATATG

CTGATGTCGTGATCGAAGTTTTACCGACACAATTAATTCCTGATGACAATGAAAGGAAGGTGCTGAGAGT

TCGATTGGTGATGAAGGAAGGGGTGAAGTATTGCAATCCAGTTTACCTCTTAGATGAAGGCTCCACCG

>Chamaedorea_pauciflora_DQ177789.1

GTCTTGAGTGCATCAAAGCCAGCATTGGAGCTCGAAAGGTCGACTTTGATGCTTATGTTGGTATGTTTCT

TTGCCACAGTGCCTAGTTATTGGTACTTGAAGAATTGGCAGAGCATTCATTTGTTGGTATAATATAGAGC

AATTTCATTAGTTTTGATTGCCAAATATAGTACTACACTTCAATATCTAGCAAATGCTTCATTAAAAAAA

GAACTCGCTAACAAAGTATCTGATCCATAAGTCAAATAATGCACAAAACATACTGGACTAAAAATATAAT

CACTCCATGAGAAATACTTTTGGATACCTTTTTCTGAAAAAAAGAAAGAGGGAGAGCAAACCCCAACCAA

TTCATTGAGATAATGAGACAATTTTATGTGTGGAGTCTCATGTTCTTCAGATATCAGAATACATGTCTTT

TTCTTTAGCACTCTTGGATTGCTGGCAGCTTATAGATTGCTACCCTTGGCAGACCTGCAGAAGCAATATG

CTGATGTCGTGATCGAAGTTTTACCGACACAATTAATTCCTGATGACAATGAAAGGAAGGTGCTGAGAGT

TCGATTGGTGATGAAGGAAGGGGTGAAGTATTGCAATCCAGTTTACCTCTTAGATGAAGGCTCCACCG

>Chamaedorea_pinnatifrons_DQ177787.1

GTCTCGAGTGCATCAAAGCCAGCATTGGAGCTCGAAAGCTCGACTTTGATGCTTATGTTGGTATGTTTCT

TTGCCACAGTGCCTAGTTATTGGTACTTGAAGAATTGGCAGAGCATTCATTTGTTGGTATAATATAGAGC

AATTTCATTAGTTTTGATTGCCAAATATAGTACTATACTTCAATATCTAGCAAATGCTTCATTAAAAAAA

GAACTCACTAACAAAGTATCTGATCCATAAGTCAAATAATGCACAAAACATACTGGACTAAAAATATAAT

CACTCCATGAGAAATACTTTTGGATACCTTTTTCTGAAAGAAAGAAAGAGGGAGAGCAAACCCCAACCAA

TTCATTGAGATAATGAGATAATTTTATGTGTGGAGTCTCGTGTTCTTCAGATATCAGAAAACATGTCTTT

TTCTTTAGCACTCTTGGATTGCTGGCAGCGTATAGATTGCTACCCTTGGCAGACCTGCAGAAGCAATATG

CTGATGTCGTGATCGAAGTTTTACCGACACAATTAATTCCTGATGACAATGAAAGGAAGGTGCTGAGAGT

TCGATTGGTGATGAAGGAAGGGGTGAAGTATTGCAATCCAGTTTACCTCTTAGATGAAGGCTCCACCG

>Chamaedorea_graminifolia_DQ177785.1

GTCTTGAGTGCATCAAAGCCAGCATTGGAGCTCGAAAGCTCGACTTTGATGCTTATATTGGTATGTTTCT

TTGCCACAGTGCCTAGTTATTGGTACTTGAAGAATTGGCAGAGCATTCATTTGTTGGTATAATATAGAGC

AATTTCATTAGTTTTGATTGCCAAATATAGTACTATACTTCAATATCTAGCAAATGCTTCATTAAAAAAA

GAACTCACTAACAAAGTATCTGATCCATAAGTCAAATAATGCACAAAACATACTGGACTAAAAATATAAT

CACTCCATGAGAAATACTTTTGGATACCTTTTTCTGAAAAAAAGAAAGAGGGAGAGCAAACCCCAACCAA

TTCATTGAGATAATGAGATAATTTTATGTGTGGAGTCTCATGTTCTTCAGATATCAGAATACATGTCTTT

TTCTTTAGCACTCTTGGATTGCTGGCAGCTTATAGATTGCTACCCTTGGCAGACCTGCAGAAGCAATATG

CTGATGTCGTGATCGAAGTTTTACCGACACAATTAATTCCTGATGACAATGAAAGGAAGGTGCTGAGAGT

TCGATTGGTGATGAAGGAAGGGGTGAAGTATTGCAATCCAGTTTACCTCTTAGATGAAGGCTCCACCG

>Chamaedorea_amabilis_DQ177783.1

GTCTTGAGTGCATCAAAGCCAGCATTGGAGCTCGAAAGCTCGACTTTGATGCTTATGTTGGTATGTTTCT

TTGCCACAGTGCCTAGTTATTGGTACTTGAAGAATTGGCAGAGCATTCATTTGTTGGTATAATATAGAGC

AATTTCATTAGTTTTGATTGCCAAATATAGTACTATACTTCAATATCTAGCAAATGCTTCATTAAAAAAA

GAACTCGCTAACAAAGTATCTGATCCATAAGTCAAATAATGCACAAAACATACTGGACTAAAAATATAAT

CACTCCATGAGAAATACATTTGGATACCTTTTTCTGAAAAAAAGAAAGAGGGAGAGCAAACCCAAACCAA

TTCATTGAGATAATGACACAATTTTATGTGTGGAGTCTCATGTTCTTCAGATATCAGAATACATGTCTTT

TTCTTTAGCACTCTTGGATTGCTGGCAGCTTATAGATTGCTACCCTTGGCAGACCTGCAGAAGCAATATG

CTGATGTCGTGATCGAAGTTTTACCGACACAATTAATTCCTGATGACAATGAAAGGAAGGTGCTGAGAGT

TCGATTGGTGATGAAGGAAGGGGTGAAGCATTGCAATCCAGTTTACCTCTTAGATGAAGGCTCCACCG

>Chamaedorea_radicalis_DQ177781.1

GTCTTGAGTGCATCAAAGCCAGCATTGGAGCTCGAAAGCTCGACTTTGATGCTTATGTTGGTATGTCACT

TTGCCACAGTGCCTAGTTGTTAGTACTTGAAGAATTGGCAGAGCATTCATTTGTTGGTATAATATAGAGC

AATTTCATTAGTTTTGATTGCCAAATATAGTACTATACTTCAATATCTAGCAAATGCTTCATTAAAAAAA

AACTCACTAACAAAGTATCTGATCCATAGGTCAAATAATGCACAAAACATACTGGACTAAAAATATAATC

ACTCCATGAGAAATACTTTTGGATACCTTTTTCTGAGAAAGAGAAAGAGGGAGAGCAAACCCCAACCAAT

TCATTGAGATAATGAGACAATTTTATGTGTGGAGTCTCATGTTCTTCAGATATCAGAATACATGTCTTTT

TCTTTAGCACTCTTGGATTGCTGGCAGCTTATAGACAGCTACCCTTGGCAGACCTGCAGAAGCAATATGC

TGATGTCGTGATCGAAGTTTTACCGACACAATTAATTCCTGATGACAATGAAAGGAAGGTGCTGAGAGTT

CGATTGGTGATGAAGGAAGGGGTGAAGTATTGCAATCCAGTTTACCTCTTAGATGAAGGCTCCACCG

>Chamaedorea_allenii_DQ177779.1

GTCTTGAGTGCATCAAAGCCAGCATTGGAGCTCGAAAGCTAGACTTTGATGCTTATGTTGGTATGTTTTT

TTGCCACAGTGCCTAGTTATTGGTACTTGAAGAATTGGCAGAGCATTTATTTGTTGGTATAATATAGASC

AATTTCATTAGTTTTGATTGCCAAATACAGTACTATACTTCAATATCTAGCAAACGCTTCATTAAAAAAA

GAACTCGCTAACAAAGTATCTGATCCATAAGTCAAATAATGCACAAAACATACTGGACTAAAAATATAAT

CACTCCATGAGAAATACTTTTGGATACCTTTTTCTGGAAAAAAGAAAGAGGGAGAGCAAACCCCAACCAA

TTCATTGAGATAATTAGACAATTTTATGTGTGGAGTCTCGTGTTCTTCAGATATCAGAATACATGTCTTT

TTCTTTAGCACTCTTGGATTGCTGGCAGCTTATAGATTGCTGGCAGCTTATTGATTGCTACCCTTGGCAG

ACCTGCAGAAGCAATATGCTGATGTCGTGATCGAAGTTTTACCGACACAATTAATTCCTGATGACAATGA

AAGGAAGGTGCTGAGAGTTCGATTGGTGATGAAGGAAGGGGTGAAGTATTGCAATCCAGTTTACCTCTTA

GATGAAGGCTCCACCG

>Chamaedorea_stricta_DQ177777.1

GTCTTGAGTGCATCAAAGCCAGCATTGGAGCTCGAAAGCTCGACTTTGATGCTTATGTTGGTATGTTTCT

TTGCCACAGTGCCTAGTTATTGGTACTTGAAGAATTGGCAGAGCATTCCTTTGTTGGTATAATATAGAGC

AATTTCATTAGTTTTGATTGCCAAATATAGTACTATACTTCAATATCTAGCAAATGCTTCATTACAAAAA

GAACTCACTAACAAAGTATCTGATCCATAAGTCAAATAATGCACAAAACATACTGGACTAAAAATATAAT

CACTCCATGAGAAATACTTTTGGATACCTTTTTCTGAAAAAAAGAAAGAGGGAGAGCAAACCCCAACCAA

TTCATTGAGATAATGAGACAATTTTATGTGTGGAGTCTCATGTTCTTCAGATATCAGAATACATGTCTTT

TTCTTTAGCACTCTTGGATTGCTGGCAGCTTATTGATTGCTACCCTTGGCAGACCTGCAGAAGCAATATG

CTGATGTCGTGATCGAAGTTTTACCGACACAATTAATTCCTGATGACAATGAAAGGAAGGTGCTGAGAGT

TCGATTGGTGATGAAGGAAGGGGTGAAGTATTGCAATCCAGTTTACCTCTTAGATGAAGGCTCCACCG

>Chamaedorea_dammeriana_DQ177775.1

ACAGCCAGCATTGGAGCTCGAAAGCTCGACTTTGATGCTTATGTTGGTATGTCACTTTGCCACAGTGCCT

AGTTATTGGTACTTGAAGAATTGGCAGAGCATTCATTTGTTGGTATAATATAGAGCAATTTCATTAGTTT

TGATTGCCAAATATAGTACTATACTTCAATATCTAGCAAATGCTTCATTAAAAAAAGAACTCGCTAACAA

AGTATCTGATCCATAAGTCAAATAATGCACAAAACATACTGGACTAAATATATAATCACTCCATGAGAAA

TACTTTTGGATACCTTTTTCTGAAAAAAAGAAAGAGGGAGAGCAAACCCCAACCAATTCATTGAGATAAT

GAGACAATTTTATGTGTGGAGTCTCATGTTCTTCAGATATCAGAATACATGTCTTTTTTATTTAGCACTC

TTGGATTGATGGCAGCTTATAGATTGCTACCCTTGGCAGACCTGCAGAAGCAATATGCTGATGTCGTGAT

CGAAGTTTTACCGACACAATTAATTCCTGATGACAATGAAAGGAAGGTGCTGAGAGTTCGATTGGTGATG

AAGGAAGGGGTGAAGTATTGCAATCCAGTTTACCTCTTAGATGAAGGCTCCACCG

>Chamaedorea_glaucifolia_DQ177773.1

GTCTTGAGTGCATCAAAGCCAGCATTGGAGCTCGAAAGCTCGACTTTGATGCTTATGTTGGTATGTTTCT

TTGCCACGGTGCCTAGTTATTGGTACTTGAAGAATTGGCAGAGCATTCATTTGTTGGTATAATATAGAGC

AATTTCATTAGTTTTGATTGCCAAATATAGTACTATACTTCAATATCTAGCAAATGCTTCATTAAAAAAA

GAACTCGCTAACAAAGTATCTGATCCATAAGTCAAATAATGCACAAAACATACTGGACTAAAAGTATAAT

CACTCCATGAGAAATACTTTTGGATACCTTTTTCTGAAAAAAAGAAAGAGGGAGAGCAAACCCCAACCAA

TTCATTGAGATAATGAGACAATTTTATGTGTGGAGTCTCATGTTCTTCAGATATCAGAATACATGTCTTT

TTCTTTAGCACTCTTGGATTGCTGGCAGCTTATTGATTGCTATCCTTGGCAGACCTGCAGAAGCAATATG

CTGATGTCGTGATCGAAGTTTTACCGACACAATTAATTCCTGATGACAATGAAAGGAAGGTGCTGAGAGT

TCGATTGGTGATGAAGAAAGGGGTGAAGTATTGCAATCCAGTTTATCTCTTAGATGAAGGCTCCACCG

>Chamaedorea_crucensis_DQ177771.1

GTCTTGAGTGCATCAAAGCCAGCATTGGAGCTCGAAAGCTAGACTTTGATGCTTATGTTGGTATGTTTTT

TTGCCACAGTGCCTAGTTATTGGTACTTGAAGAATTGGCAGAGCATTTATTTGTTGGTATAATATAGAAC

AATTTCATTAGTTTTGATTGCCAAATATAGTACTATACTTCAATATCTAGCAAACGCTTCATTAAAAAAA

GAACTCGCTAACAAAGTATCTGATCCATAAGTCAAATAATGCACAAAACATACTGGACTAAAAATATAAT

CACTCCATGAGAAATACTTTTGGATACCTTTTTCTGGAAAAAAGAAAGAGGGAGAGCAAACCCCAACCAA

TTCATTGAGATAATTAGACAATTTTATGTGTGGAGTCTCGTGTTCTTCAGATATCAGAATACATGTCTTT

TTCTTTAGCACTCTTGGATTGCTGGCAGCTTATAGATTGCTGGCAGCTTATTGATTGCTACCCTTGGCAG

ACCTGCAGAAGCAATATGCTGATGTCGTGATCGAAGTTTTACCGACACAATTAATTCCTGATGACAATGA

AAGGAAGGTGCTGAGAGTTCGATTGGTGATGAAGGAAGGGGTGAAGTATTGCAATCCAGTTTACCTCTTA

GATGAAGGCTCCACCG

>Chamaedorea_adscendens_DQ177769.1

GTCTTGAGTGCATCAAAGCCAGCATTGGAGCTCGAAAGCTCGACTTTGATGCTTATGTTGGTATGTTTCT

TTGCCACAGTGCCTAGTTATTGGTACTTGAAGAATTGGCAGAGCATTCATTTGTTGGTATAATATAGAGC

AATTTCATTAGTTTTGATTGCCAAATATAGTACTATACTTCAATATCTAGCAAATGCTTCATTAAAAAAA

GAACTCGCTAACAAAGTATCTGATCCATAAGTCAAATAATGCACAAAACATACTGGACTAAAAATATAAT

CACTCCATGAGAAATACATTTGGATACCTTTTTCTGAAAAAAAGAAAGAGGGAGAGCAAACCCAAACCAA

TTCATTGAGATAATGACACAATTTTATGTGTGGAGTCTCATGTTCTTCAGATATCAGAATACATGTCTTT

TTCTTTAGCACTCTTGGATTGCTGGCAGCTTATAGATTGCTACCCTTGGCAGACCTGCAGAAGCAATATG

CTGATGTCGTGATCGAAGTTTTACCGACACAATTAATTCCTGATGACAATGAAAGGAAGGTGCTGAGAGT

TCGATTGGTGATGAAGGAAGGGGTGAAGCATTGCAATCCAGTTTACCTCTTAGATGAAGGCTCCACCG

>Chamaedorea_cataractarum_DQ177767.1

AAGCCAGCATTGGAGCTCGAAAGCTCGACTTTGATGCTTATGTTGGTATGTTTCTTTGCCACAGTGCCTA

GTTATTGGTAGAGCATTTATTTGTTGGTATAATATAGAGCAATTTCATTAGTTTTGATTGCCAAATATAG

TACTATACTTCAATATCTAGCAAATGCTTCATTAAAAAAAGAACTCGCTAACAAAGTATCTGATCCATAA

GTCAAATAATGCACAAAACATACTGGACTAAAAATATAATCACTCCATGAGAAATACTTTTGGATACCTT

TTTCTGGAAAAAAGAAAAAGGGAGAGCAAACCCCAACCAATTCATTGAGATAATTAGACAATTTTATGTG

TGGAGTCTCCATGTTCTTCAGATATCAGAATACATGTCTTTTTCTTTAGCACTCTTGGATTGCTGGCAGC

TTATTGATTGCTACCCTTGGCAGACCTGCAGAAGCAATATGCTGATGTCGTGATCGAAGTTTTACCGACA

CAATTAATTCCTGATGACAATGAAAGGAAGGTGCTGAGAGTTCGATTGGTGATGAAGGAAGGGGTGAAGT

ATTGCAATCCAGTTTACCTCTTAGATGAAGGCTCCACCG

>Chamaedorea_fragrans_DQ177765.1

GTCTTGAGTGCATCAAAGCCAGCATTGGAGCTCGAAAGCTCGACTTTGATGCTTATGTTGGTATGTCACT

TTGCCACAGTGCCTAGTTATTGGTACTTGAAGAATTGGCAGAGCATTCATTTGTTGGTATAATATAGAGC

AATTTCATTAGTTTTGATTGCCAAATATAGTACTATACTTCAATATCTAGCAAATGCTTCATTAAAAAAA

GAACTCGCTAACAAAGTATCTGATCCATAAGTCAAATAATGCACAAAACATACTGGACTAAATATATAAT

CACTCCATGAGAAATACTTTTGGATACCTTTTTCTGAAAAAAAGAAAGAGGGAGAGCAAACCCCAACCAA

TTCATTGAGATAATGAGACAATTTTATGTGTGGAGTCTCATGTTCTTCAGATATCAGAATACATGTCTTT

TTTMTTTAGCACTCTTGGATTGATGGCAGCTTATAGATTGCTACCCTTGGCAGACCTGCAGAAGCAATAT

GCTGATGTCGTGATCGAAGTTTTACCGACACAATTAATTCCTGATGACAATGAAAGGAAGGTGCTGAGAG

TTCGATTGGTGATGAAGGAAGGGGTGAAGTATTGCAATCCAGTTTACCTCTTAGATGAAGGCTCCACCG

>Chamaedorea_neurochlamys_DQ177763.1

GTCTTGAGTGCATCAAAGCCAGCATTGGAGCTCGAAAGCTCGACTTTGATGCTTATGTTGGTATGTTTCT

TTGCCACGGTGCCTAGTTATTGGTACTTGAAGAATTGGCAGAGCATTCATTTGTTGGTATAATATAGAGC

AATTTCATTAGTTTTGATTGCCAAATATAGTACTATACTTCAATATCTAGCAAATGCTTCATTAAAAAAA

GAACTTGCTAACAAAGTATCTGATCCATAAGTCAAATAATGCACAAAACATACTGGACTAAAAATATAAT

CACTCCATGAGAAATACTTTTGGATACCTTTTCTGAAAAAAAGAAAGAGGGAGAGCAAACCCCAACCAAT

TCATTGAGATAATGAGACAATTTTATGTGTGGAGTCTCATGTTCTTCAGATATCAGAATACATGTCTTTT

TCTTTAGCACTCTTGGATTGCTGGCAGCTTATTGATTGCTATCCTTGGCAGACCTGCAGAAGCAATATGC

TGATGTCGTGATCGAAGTTTTACCGACACAATTAATTCCTGATGACAATGAAAGGAAGGTGCTGAGAGTT

CGATTGGTGATGAAGGAAGGGGTGAAGTATTGCAATCCAGTTTATCTCTTAGATGAAGGCTCCACCG

>Chamaedorea_seifrizii_DQ177761.1

GTCTTGAGTGCATCAAAGCCAGCATTGGAGCTCGAAAGCTTGACTTTGATGCTTATGTTGGTATGTTTCT

TTGCCACAGTCCCTAGTTATTGTTACTTGAAGAATTGGCAGAGCATTCATTTGTTGGTATAATATAGAGC

AATTTCATTAGTTTTGATAGCCAAATATGGTACTATACTTCAATATCTAGCAAATGCTTCATTAAGAAAA

AAACTCACTAACAAAGTATCTGATCCATAAGTCAAATAATGCACAAAACATTCTGGACTAAAAATATAAT

CACTCCATGAGAAATACTTTTGGATACCTTTTTCTGAAAAAAAGAAAGAGGGAGAGCAAACCCCAACCAA

TTCATTGAGATAATGAGACAAATTTATGTGTGGAGTCTCATGTTCTTCAGATATCAGAATACATGTCTTT

TTCTTTAGCACTCTTGGATTGCTGGCAGCTTATAGATTGCTACCCTTGGCAGACCTGCAGAAGCAATATG

CTGATGTCGTGATCGAAGTTTTACCGACACAATTAATTACTGATGACAATGAAAGGAAGGTGCTGAGAGT

TCGATTGGTGATGAAGGAAGGGGTGAAGTATTGCAATCCAGTTTACCTCTTAGATGAAGGCTCCACCG

>Chamaedorea_hooperiana_DQ177759.1

GTCTTGAGTGCATCAAAGCCAGCATTGGAGCTCGAAAGCTCGACTTTGATGCTTATGTTGGTATGTTTCT

TTGCCACAGTGCCTAGTTATTGGTACTTGAAGAATTGGCAGAGCATTCATTTGTTGGTATAATATAGAGC

AATTTCATTAGTTTTGATTGCCAAATATAGTACTATACTTCAATATCTAGCAAATGCTTCATTAAAAAAA

GAACTCACTAACAAAGTATCTGATCCATAAGTCAAATAATGCACAAAACATACTGGACTAAAAATATAAT

CACTCCATGAGAAATACTTTTGGATACCTTTTTCTGAAAAAAAGAAAGAGGGAGAGCAAACCCCAACCAA

TTCATTGAGATAATGAGATAATTTTATGTGTGGAGTCTCATGTTCTTCAGATATCAGAATACATGTCTTT

TTCTTTAGCACTCTTGGATTGCTGGCAGCTTATAGATTGCTACCCTTGGCAGACCTGCAGAAGCAATATG

CTGATGTCGTGATCTCGAAGTTTTACCGACACAATTAATTCCTGATGACAATGAAAGGAAGGTGCTGAGA

GTTCGATTGGTGATGAAGGAAGGGGTGAAGTATTGCAATCCAGTTTACCTCTTAGATGAAGGCTCCACCG

>Chamaedorea_benziei_DQ177814.1

GTCTTGAGTGCATCAAAGCCAGCATTGGAGCTCGAAAGCTCGACTTTGATGCTTATGTTGGTATGTTTCT

TTGCCACGGTGCCTAGTTATTGGTACTTGAAGAATTGGCAGAGCATTCATTTGTTGGTATAATATAGAGC

AATTTCATTAGTTTTGATTGCCAAATATAGTACTATACTTCAATATCTAGCAAATGCTTCATTAAAAAAA

GAACTCGCTAACAAAGTATCTGATCCATAAGTCAAATAATGCACAAAACATACTGGACTAAAAATATAAT

CACTCCATGAGAAATACTTTTGGATACCTTTTTCTGAAAAAAAGAAAGAGGGAGAGCAAACCCCAACCAA

TTCATTGAGATAATGAGACAATTTTATGTGTGGAGTCTCATGTYCTTCAGATATCAGAATACATGTCTTT

TTCTTTAGCACTCTTGGATTGCTGGCAGCTTATTGATTGCTATCCTTGGCAGACCTGCAGAAGCAATATG

CTGATGTCGTGATCGAAGTTTTACCGACACAATTAATTCCTGATGACAATGAAAGGAAGGTGCTGAGAGT

TCGATTGGTGATGAAGGAAGGGGTGAAGTATTGCAATCCAGTTTATCTCTTAGATGAAGGCTCCACCG

>Chamaedorea_vulgata_DQ177812.1

GTCTTGAGTGCATCAAAGCCAGCATTGGAGCTCGAAAGCTCAACTTTGATGCTTATGTTGGTATGTTTCT

TTGCCACAGTGCCTAGTTATTGGTACTTGAAGAATTGGCAGAGCATTCATTTGTTGGTATAATATAGAGC

AATTTCATTAGTTTTGATTGCCAAATATAGTACTATACTTCAATATCTAGCAAATGCTTCATTAAAAAAA

GAACTCGCTAACAAAGTATCTGATCCATAAGTCAAATAATGCACAAAACATACTGGACTAAAAATATAAT

CACTCCATGAGAAATACTTTTGGATACCTTTTTCTGAAAAAAAGAAAGAGGGAGAGCAAACCCCAACCAA

TTCATTGAGATAATGAGATAATTTTATGTGTGGAGTCTCGTGTTCTTCAGATATCAGAAAACATGTCTTT

TTCTTTACCACTCTTGGATTGCTGGCAGCTTATAGATTGCTACCCTTGGCAGACCTGCAGAAGCAATATG

CTGATGTCGTGATCGAAGTTTTACGGACACAATTAATTCCTGATGACAATGAAAGGAAGGGGCTGAGAGT

TCGATTGGTGATGAAGGAAGGGGTGAAGTATTGCAATCCAGTTTACCTCTTAGATGAAGGCTCCACCG

>Chamaedorea_schippii_DQ177810.1

GTCTTGAGTGCATCAAAGCCAGCATTGGAGCTCGAAAGCTCGACTTTGATGCTTATGTTGGTATGTTTCT

TTGCCACGGTGCCTAGTTATTGGTACTTGAAGAATTGGCAGAGCATTCATTTGTTGGTATAATATAGAGC

AATTTCATTAGTTTTGATTGCCAAATATAGTACTATACTTCAATATCTAGCAAATGCTTCATTAAAAAAA

GAACTCGCTAACAAAGTATCTGATCCATAAGTCAAATAATGCACAAAACATACTGGACTAAAAATATAAT

CACTCCATGAGAAATACTTTTGGATACCTTTTTCTGAAAAAAAGAAAGAGGGAGAGCAAACCCCAACCAA

TTCATTGAGATAATGAGACAATTTTATGTGTGGAGTCTCATGTTCTTCAGATATCAGAATACATGTCTTT

TTCTTTAGCACTCTTGGATTGCTGGCAGCTTATTGATTGCTATCCTTGGCAGACCTGCAGAAGCAATATG

CTGATGTCGTGATCGAAGTTTTACCGACACTATTAATTCCTGATGACAATGAAAGGAAGGTGCTGAGAGT

TCGATTGGTGATGAAGGAAGGGGTGAAGTATTGCAATCCAGTTTATCTCTTAGATGAAGGCTCCACCG

>Chamaedorea_plumosa_DQ177808.1

GTCTTGAGTGCATCAAAGCCAGCATTGGAGCTCGAAAGCTCGACTTTGATGCTTATGTTGGTATGTTTCT

TTGCCACAGTGCCTAGTTATTGGTACTTGAAGAATTGGCAGAGCATTCATTTGTTGGTATAATATAGAGC

AATTTCATTAGTTTTGATTGCCAAATATAATACTATACTTCAATATCTAGCAAATGCTTCATTAAAAAAA

AAACTCACTAACAAAGTATCTGATCCATAAGTCAAATAATGCACAAAACATACTGGACTAAAAATATAAA

TCACTCCATGAGAAATACTTTTTGATACCTTTTTCTGAAAAAAAGAAAGAGGGAGAGCAAACCCCAACCA

ATTCATTGAGATAATGAGACAATTTTATGTGTGGAGTCTCATGTTCTTCAGATATCAGAATACATGTCTT

TTTCTTTAGCACTCTTGGATTGCTGGCAGCTTATAGATTGCTACCCTTGGCAGACCTGCAGAAGCAATAT

GCTGATGTCGTGATCGAAGTTTTACCGACACAATTAATTCCTGATGACAATGAAAGGAAGGTGCTGAGAG

TTCGATTGGTGATGAAGGAAGGGGTGAAGTATTGCAATCCAGTTTACCTCTTAGATGAAGGCTCCACCG

>Chamaedorea_frondosa_DQ177806.1

GTCTTGAGTGCATCAAGCCAGCATTGGAGCTCGAAAGCTCGACTTTGATGCTTATGTTGGTATGTTTCTT

TGCCACGGTGCCTAGTTATTGGTACTTGAAGAATTGGCAGAGCATTCATTTGTTGGTATAATATAGAGCA

ATTTCATTAGTTTTGATTGCCAAATATAGTACTATACTTCAATATCTAGCAAATGCTTCATTAAAAAAAG

AACTCGCTAACAAAGTATCTGATCCATAAGTCAAATAATGCACAAAACATACTGGACTAAAAATATAATC

ACTCCATGAGAAATACTTTTGGATACCTTTTTCTGAAAAAAAGAAAGAGGGAGAGCAAACCCCAACCAAT

TCATTGAGATAATGAGACAATTTTATGTGTGGAGTCTCATGTTCTTCAGATATCAGAATACATGTCTTTT

TCTTTAGCACTCTTGGATTGCTGGCAGCTTATTGATTGCTATCCTTGGCAGACCTGCAGAAGCAATATGC

TGATGTCGTGATTGAAGTTTTACCGACACAATTAATTCCTGATGACAATGAAAGGAAGGTGATGAGAGTT

CGATTGGTGATGAAGGAAGGGGTGAAGTATTGCAATCCAGTTTATCTCTTAGATGAAGGCTCCACCG

>Chamaedorea_rojasiana_DQ177804.1

GTCTTGAGTGCATCAAAGCCAGCATTGGAGCTCGAAAGCTCGACTTTGATGCTTATGTTGGTATGTTTCT

TTGCCACGGTGCCTAGTTATTGGTACTTGAAGAATTGGCAGAGCATTCATTTGTTGGTATAATATAGAGC

AATTTCATTAGTTTTGATTGCCAAATATAGTACTATACTTCAATATCTAGCAAATGCTTCATTGAAAAAA

GAACTCGCTAACAAAGTATCTGATCCATAAGTCAAATAATGCACAAAACATACTGGACTAAAAATATAAT

CACTCCATGAGAAATACTTTTGTATACCTTTTTCTGAAAAAAAGAAAGAGGGAGAGCAAACCCCAACCAA

TTCATTGAGATAATGAGACAATTTTATGTGTGGAGTCTCATGTTCTTCAGATATCAGAATACATGTCTTT

TTCTTTAGCACTCTTGGATTGCTGGCAGCTTATTGATTGCTATCCTTGGCAGACCTGCAGAAGCAATATG

CTGATGTCGTGATCGAAGTTTTACCGACACAATTAATTCCTGATGACAATGAAAGGAAGGTGCTGGGAGT

TCGATTGGTGATGAAGGAAGGGGTGAAGTATTGCAATCCAGTTTATCTCTTAGATGAAGGCTCCACCG

>Chamaedorea_geonomiformis_DQ177802.1

GTCTTGAGTGCATCAAAGCCAGCATTGGAGCTCGAAAGCTCGACTTTGATGCTTATGTTGGTATGTTTCT

TTGCCACGGTGCCTAGTTATTGGTACTTGAAGAATTGGCAGAGCATTCATTTGTTGGTATAATATAGAGC

AATTTCATTAGTTTTGATTGCCAAATATAGTACTATACTTCAATATCTAGCAAATGCTTCATTAAAAAAA

GAACTCGCTAACAAAGTATCTGATCCATAAGTCAAATAATGCACAAAACATACTGGACTAAAAATATAAT

CACTCCATGAGAAATACTTTTGGATACCTTTTTCTGAAAAAAAGAAAGAGGGAGAGCAAACCCCAACCAA

TTCATTGAGATAATGAGACAATTTTATGTGTGGAGTCTCATGTTCTTCAGATATCAGAATACATGTCTTT

TTCTTTAGCACTCTTGGATTGCTGGCAGCTTATTGATTGCTATCCTTGGCAGACCTGCAGAAGCAATATG

CTGATGTCGTGATCGAAGTTTTACCGACACAATTAATTCCTGATGACAATGAAAGGAAGGTGCTGAGAGT

TCGATTGGTGATGAAGGAAGGGGTGAAGTATTGCAATCCAGTTTATCTCTTAGATGAAGGCTCCACCG

>Chamaedorea_ibarrae_DQ177800.1

GTCTTGAGTGCATCAAAGCCAGCATTGGAGCTCGAAAGCTCGACTTTGATGCTTATGTTGGTATGTTTCT

TTGCCACGGTGCCTAGTTATTGGTACTTGAAGAATTGGCAGAGCATTCATTTGTTGGTATAATATAGAGC

AATTTCATTAGTTTTGATTGCCAAATATAGTACTATACTTCAATATCTAGCAAATGCTTCATTAAAAAAA

GAACTCGCTAACAAAGTATCTGATCCATAAGTCAAATAATGCACAAAACATACTGGACTAAAAATATAAT

CACTCCATGAGAAATACTTTTGGATACCTTTTTCTGAAAAAAAGAAAGAGGGAGAGCAAACCCCAACCAA

TTCATTGAGATAATGAGACAATTTTATGTGTGGAGTCTCATGTYCTTCAGATATCAGAATACATGTCTTT

TTCTTTAGCACTCTTGGATTGCTGGCAGCTTATTGATTGCTATCCTTGGCAGACCTGCAGAAGCAATATG

CTGATGTCGTGATCGAAGTTTTACCGACACAATTAATTCCTGATGACAATGAAAGGAAGGTGCTGAGAGT

TCGATTGGTGATGAAGGAAGGGGTGAAGTATTGCAATCCAGTTTATCTCTTAGATGAAGGCTCCACCG

>Chamaedorea_guntheriana_DQ177798.1

GTCTTGAGTGCATCAAAGCCAGCATTGGAGCTCGAAAGCTCGACTTTGATGCTTATGTTGGTATGTTTCT

TTGCCACGGTGCCTAGTTATTGGTACTTGAAGAATTGGCAGAGCATTCATTTGTTGGTATAATATAGAAC

AATTTCATTAGTTTTGATTGCCAAATATAGTACTATACTTCAATATCTAGCAAATGCTTCATTGAAAAAA

GAACTCGCTAACAAAGTATCTGATCCATAAGTCAAATAATGCACAAAACATACTGGACTAAAAATATAAT

CACTCCATGAGAAATACTTTTGGATACCTTTTTCTGAAAAAAAGAAAGAGGGAGAGCAAACCCCAACCAA

TTCATTGAGATAATGAGACAATTTTATGTGTGGAGTCTCATGTTCTTCAGATATCAGAATACATGTCTTT

TTCTTTAGCACTCTTTGATTGCTGGCAGCTTATTGATTGCTATCCTTGGCAGACCTGCAGAAGCAATATG

CTGATGTCGTGATCGAAGTTTTACCGACACAATTAATTCCTGATGACAATGAAAGGAAGGTGCTGAGAGT

TCGATTGGTGATGAAGGAAGGGGTGAAGTATTGCAATCCAGTTTATCTCTTAGATGAAGGCTCCACCG

>Chamaedorea_rhizomatosa_DQ177796.1

GTCTTGAGTGCATCAAAGCCAGCATTGGAGCGCAAAAGCTCGACTTTGATGCTTATGTTGGTATGTTTCT

TTGCCACAGTGCCTAGTTATTGGTACTTGAAGAATTGGCAGGGCATTCATTTGTTGGTATAATATAGAGC

AATTTCATTAGTTTTGATTGCCAAATATAGTACTATACTTCAATATCTAGCAAATGCTTCATTAAAAAAA

AAAACTCACGAACAAAGTATCTGATCCATAAGTCAAATAATGCACAAAACATACTGGACTAAAAATATAA

TCACTCCATGAGAAATACTTTTGGATACCTTTTTCTGAAAAAAAGAAAGAGGGAGAGCAAACCCCAACCA

ATTCATTGAGATAATGAGACAATTTTATGTGTGGAGTCTCATGTTCTTCAGATATCAGAATACATGTCTT

TTTCTTTAGCACTCTTGGATTGCTGGCAGCTTATAGATTGCTACCCTTGGCAGACCTGCAGAAGCAATAT

GCTGATGTCGTGATCGAAGTTTTACCGACACAATTAATTCCTGATGACAATGAAAGGAAGGTGCTGAGAG

TTCGATCGGTGATGAAGGAAGGGGTGAAGTATTGCAATCCAGTTTACCTCTTAGATGAAGGCTCCACCG

>Chamaedorea_woodsoniana_DQ177794.1

GTCTTGAGTGCATCAAAGCCAGCATTGGAGCTCGAAAGCTCGACTTTGATGCTTATGTTGGTATGTTTCT

TTGCCACAGTGCCTAGTTATTGGTACTTGAAGAATTGGCAGAGCATTCCTTTGTTGGTATAATATAGAGC

AATTTCATTAGTTTTGATTGCCAAATATAGTACTATACTTCAATATCTAGCAAATGCTTCATTACAAAAA

GAACTCACTAACAAAGTATCTGATCCATAAGTCAAATAATGCACAAAACATACTGGACTAAAAATATAAT

CACTCCATGAGAAATACTTTTGGATACCTTTTTCTGAAAAAAAGAAAGAGGGAGAGCAAACCCCAACCAA

TTCATTGAGATAATGAGACAATTTTATGTGTGGAGTCTCATGTTCTTCAGATATCAGAATACATGTCTTT

TTCTTTAGCACTCTTGGATTGCTGGCAGCTTATTGATTGCTACCCTTGGCAGACCTGCAGAAGCAATATG

CTGATGTCGTGATCGAAGTTTTACCGACACAATTAATTCCTGATGACAATGAAAGGAAGGTGCTGAGAGT

TCGATTGGTGATGAAGGAAGGGGTGAAGTATTGCAATCCAGTTTACCTCTTAGATGAAGGCTCCACCG

>Chamaedorea_alternans_DQ177792.1

GTCTTGAGTGCATCAAAGCCAGCATTGGAGCTCGAAAGCTCGACTTTGATGCTTATGTTCGTATGTTTCT

TTGCCACAGTGCCTAGTTATTGGTACTTGAAGAATTGGCAGAGCATTTATTTGTTGGTATAATATAGAGC

AATTTCATTAGTTTTGATTGCCAAATATAGTACTATACTTCAATATCTAGCAAATGCTTCATTAAAAAAA

GAACTCGCTAACAAAGTATCTGATCCATAAGTCAAATAATGCACAAAACATACTGAACTAAAAATATAAT

CACTCCATGAGAAATACTTTTAGATACCTTTTTCTGGAAAAAAGAAAGAGGGAGAGCAAACCCCAACCAA

TTCATTGAGATAATTAGACAATTTTATGTGTGGAGTCTCATGTTCTTCAGATATCAGAATACATGTCTTT

TTCTTTAGCACTCTTGGATTGCTGGCAGCTTATAGATTGCTGGCAGCTTATTGATTGCTACCCTTGGCAG

ACCTGCAGAAGCAATATGCTGATGTCGTGATCGAAGTTTTACCGACACAATTAATTCCTGATGACAATGA

AAGGAAGGTGCTGAGAGTTCGATTGGTGATGAAGGAAGGGGTGAAGTATTGCAATCCAGTTTACCTCTTA

GATGAAGGCTCCACCG

>Chamaedorea_elatior_DQ177790.1

GTCTTGAGTGCATCAAAGCCAGCATTGGAGCTCGAAAGCTCGACTTTGATGCTTATGTTGGTATGTTTCT

TTGCCACGGTGCCTAGTTATTGGTACTTGAAGAATTGGCAGAGCATTCATTTGTTGGTATAATATAGAGC

AATTTCATTAGTTTTGATTGCCAAATATAGTACTATACTTCAATATCTAGCAAATGCTTCATTAAAAAAA

GAACTCGCTAACAAAGTATCTGATCCATAAGTCAAATAATGCACAAAACATACTGGACTAAAAATATAAT

CACTCCATGAGAAATACTTTTGGATACCTTTWTCTGAAAAAAAGAAAGAGGGAGAGCAAACCCCAACCAA

TTCATTGAGATAATGAGACAATTTTATGTGTGGAGTCTCATGTTCTTCAGATATCAGAATACATGTCTTT

TTCTTTAGCACTCTTGGATTGCTGGCAGCTTATTGATTGCTATCCTTGGCAGACCTGCAGAAGCAATATG

CTGATGTCGTGATCGAAGTTTTACCGACACAATTAATTCCTGATGACAATGAAAGGAAGGTGCTGAGAGT

TCGATTGGTGATGAAGGAAGGGGTGAAGTATTGCAATCCAGTTTATCTCTTAGATGAAGGCTCCACCG

>Chamaedorea_costaricana_DQ177788.1

GTCTTGAGTGCATCAAAGCCAGCATTGGAGCTCGAAAGCTCGACTTTGATGCTTATGTTGGTATGTTTCT

TTGCCACAGTGCCTAGTTATTGGTACTTGAAGAATTGGCAGAGCATTCATTTGTTGGTATAATATAGAGC

AATTTCATTAGTTTTGATTGCCAAATATAGTACTATACTTCAATATCTAGCAAATGCTTCATTAAAAAAA

GAACTCACTAACAAAGTATCTGATCCATAAGTCAAATAATGCACAAAACATACTGGACTAAAAATATAAT

CACTCCATGAGAAATACTTTTGGATACCTTTTTCTGAAAAAAAGAAAGAGGGAGAGCAAACCCCAACCAA

TTCATTGAGATAATGAGATAATTTTATGTGTGGAGTCTCATGTTCTTCAGATATCAGAATACATGTCTTT

TTCTTTAGCACTCTTGGATTGCTGGCAGCTTATAGATTGCTACCCTTGGCAGACCTGCAGAAGCAATATG

CTGATGTCGTGATCTCGAAGTTTTACCGACACAATTAATTCCTGATGACAATGAAAGGAAGGTGCTGAGA

GTTCGATTGGTGATGAAGGAAGGGGTGAAGTATTGCAATCCAGTTTACCTCTTAGATGAAGGCTCCACCG

>Chamaedorea_stenocarpa_DQ177786.1

GTCTCGAGTGCATCAAAGCCAGCATTGGAGCTCGAAAGCTCGACTTTGATGCTTATGTTGGTATGTTTCT

TTGCCACAGTGCCTAGTTATTGGTACTTGAAGAATTGGCAGAGCATTCATTTGTTGGTATAATATAGAGC

AATTTCATTAGTTTTGATTGCCAAATATAGTACTATACTTCAATATCTAGCAAATGCTTCATTAAAAAAA

GAACTCACTAACAAAGTATCTGATCCATAAGTCAAATAATGCACAAAACATACTGGACTAAAAATATAAT

CACTCCATGAGAAATACTTTTGGATACCTTTTTCTGAAAGAAAGAAAGAGGGAGAGCAAACCCCAACCAA

TTCATTGAGATAATGAGATAATTTTATGTGTGGAGTCTCGTGTTCTTCAGATATCAGAAAACATGTCTTT

TTCTTTAGCACTCTTGGATTGCTGGCAGCGTATAGATTGCTACCCTTGGCAGACCTGCAGAAGCAATATG

CTGATGTCGTGATCGAAGTTTTACCGACACAATTAATTCCTGATGACAATGAAAGGAAGGTGCTGAGAGT

TCGATTGGTGATGAAGGAAGGGGTGAAGTATTGCAATCCAGTTTACCTCTTAGATGAAGGCTCCACCG

>Chamaedorea_parvifolia_DQ177784.1

GTCTTGAGTGCATCAAAGCCAGCATTGGAGCTCGAAAGCTCGACTTTGATGCTTATGTTGGTATGTTTCT

TTGCCACGGTGCCTAGTTATTGGTACTTGAAGAATTGGCAGAGCATTCATTTGTTGGTATAATATAGAAC

AATTTCATTAGTTTTGATTGCCAAATATAGTACTATACTTCAATATCTAGCAAATGCTTCATTGAAAAAA

GAACTCGCTAACAAAGTATCTGATCCATAAGTCAAATAATGCACAAAACATACTGGACTAAAAATATAAT

CACTCCATGAGAAATACTTTTGGATACCTTTTTCTGAAAAAAAGAAAGAGGGAGAGCAAACCCCAACCAA

TTCATTGAGATAATGAGACAATTTTATGTGTGGAGTCTCATGTTCTTCAGATATCAGAATACATGTCTTT

TTCTTTAGCACTCTTGGATTGCTGGCAGCTTATTGATTGCTATCCTTGGCAGACCTGCAGAAGCAATATG

CTGATGTCGTGATCGAAGTTTTACCGACACAATTAATTCCTGATGACAATGAAAGGAAGGTGCTGAGAGT

TCGATTGGTGATGAAGGAAGGGGTGAAGTATTGCAATCCAGTTTATCTCTTAGATGAAGGCTCCACCG

>Chamaedorea_elegans_DQ177782.1

GTCTTGAGGGCATCAAAGCCCGCATTGGAGCTCGAAAGCTCGACTTTGATGCTTATGTTGGTATGTTTCT

TTGCCACAGTGCCTAGTTATTGGTACTTGAAGAATTGGCAGAGCATTCATTTGTTGGTATAATATAGAGC

AATTTCATTAGTTTTGATTGCAAAATATAGTACTATACTTCAATATCTAGCAAATGCTTCATTAAAAAAA

AAACTCACTAACAAAGTATCTGATCCATAAGTCAAATAATGCACAAAACATTCTGGACTAAAAATATAAT

CACTCCATGAGAAATTCTTTTGGATACCTTTTTCTGAAAAAAAGAAAGAGGGAGAGCAAACCCCAACCAA

TTCATTGAGATAATGAGACAATTTTATGTGTGGAGTCTCGTGTTCTTCAGATATCAGAATACATGTCTTT

TTCTTTAGCACTCTTGGATTGCTGGCAGCTTATAGATTGCTACCCTTGGCAGACCTGCAGAAGCAATATG

CTGATGTCGTGATCGAAGTTTTACCGACACAATTAATTCCTGATGACAATGAAAGGAAGGTGCTGAGAGT

TCGATTGGTGACGAAGGAAGGGGTGAAGTATTGCAATCCAGTTTACCTCTTAGATGAAGGCTCCACCG

>Chamaedorea_metallica_DQ177780.1

GTCTTGAGTGCATCAAAGCCAGCATTGGAGCGCGAAAGCTCGACTTTGATGCTTATGTTGGTATGTTTCT

TTGCCACAGTGCCTAGTTATTGGTACTTGAAGAATTGGCAGGGCATTCATTTGTTGGTATAATATAGAGC

AATTTCATTAGTTTTGATTGCCAAATATAGTACTATACTTCAATATCTAGCAAATGCTTCATTAAAAAAA

GAAACTCACTAACAAAGTATCTGATCCATAAGTCAAATAATGCACAAAACATACTGGACTAAAAATATAA

TCACTCCATGAGAAATACTTTTGGATACCTTTTTCTGAAAAAAAGAAAGAGGGAGAGCAAACCCCAACCA

ATTCATTGAGATAATGAGACAATTTTATGTGTGGAGTCTCATGTTCTTCAGATATCAGAATACTTGTCTT

TTTCTTTAGCACTCTTGGATTGCTGGCAGCTTATATAGATTGCTACCCTTGGCAGACCTGCAGAAGCAAT

ATGCTGATGTCGTGATCGAAGTTTTACCGACACAATTAATTCCTGATGACAATGAAAGGAAGGTGCTGAG

AGTTCGATTGGTGATGAAGGAAGGGGTGAAGTATTGCAATCAAGTTTACCTCTTAGATGAAGGCTCCACC

G

>Chamaedorea_arenbergiana_DQ177778.1

GTCTTGAGTGCATCAAAGCCAGCATTGGAGCTCGAAAGCTAGACTTTGATGCTTATGTTGGTATGTTTCT

TTGCCACAGTGCCTAGTTATTGGTACTTGAAGAATTGGCAGAGCATTTATTTGTTGGTATAATATAGAGC

AATTTCATTAGTTTTGATTGCCAAATATAGTACTATACTTCAATATCTAGCAAATGCTTCATTAAAAAAA

GAACTCGCTAACAAAGTATCTGATCCATAAGTCAAATAATGCACAAAACATACTGGACTAAAAATATAAT

CACTCCATGAGAAATACTTTTGGATACCTTTTTCTGGAAAAAAGAAAGAGGGAGAGCAAACCCCAACCAA

TTCATTGAGATAATTAGACAATTTTATGTGTGGAGTCTCATGTTCTTCAGATATCAGAATACATGTCTTT

TTCTTTAGCACTCTTGGATTGCTGGCAGCTTATAGATTGCTGGCAGCTTATTGATTGCTACCCTTGGCAG

ACCTGCAGAAGCAATATGCTGATGTCGTGATCGAAGTTTTACCGACACAATTAATTCCTGATGACAATGA

AAGGAAGGTGCTGAGAGTTCGATTAGTGATGAAGGAAGGGTGAAGTATTGCAATCCAGTTTACCTCTTAG

ATGAAGGCTCCACCG

>Chamaedorea_pochutlensis_DQ177776.1

GTCTTGAGTGCATCAAAGCCAGCATTGGAGCTCGAAAGCTCGACTTTGATGCTTATGTTGGTATGTTTCT

TTGCCACGGTGCCTAGTTATTGGTACTTGAAGAATTGGCAGAGCATTCATTTGTTGGTATAATATAGAGC

AATTTCATTAGTTTTGATTGCCAAATATAGTACTATACTTCAATATCTAGCAAATGCTTCATTAAAAAAA

GAACTCGCTAACAAAGTATCTGATCCATAAGTCAAATAATGCACAAAACCTACTGGACTAAAAATATAAT

CACTCCATGAGAAATACTTTTGGATACCTTTTTCTGAAAAAAAGAAAGAGGGAGAGCAAACCCCAACCAA

TTCATTGAGATAATGAGACAATTTTATGTGTGGAGTCTCATGTTCTTCAGATATCAGAATACATGTCTTT

TTCTTTAGCACTCTTGGATTGCTGGCAGCTTATTGATTGCTATCCTTGGCAGACCTGCAGAAGCAATATG

CTGATGTCGTGATCGAAGTTTTACCGACACAATTAATTCCTGATGACAATGAAAGGAAGGTGCTGAGAGT

TCGATTGGTGATGAAGGAAGGGGTGAAGTATTGCAATCCAGTTTATCTCTTAGATGAAGGCTCCACCG

>Chamaedorea_tenella_DQ177774.1

GTCTTGAGTGCATCAAAGCCAGCATTGGAGCTCGAAAGCTCGACTTTGATGCTTATGTTGGTATGTTTCT

TTGCCACGGTGCCTAGTTATTGGTACTTGAAGAATTGGCAGAGCATTCATTTGTTGGTATAATATAGAGC

AATTTCATTAGTTTTGATTGCCAAATATAGTACTATACTTCAATATCTAGCAAATGCTTCATTAAAAAAA

GAACTCGCTAACAAAGTATCTGATCCATAAGTCAAATAATGCACAAAACATACTGGACTAAAAATATAAT

CACTCCATGAGAAATACTTTTGGATACCTTTTTCTGAAAAAAAGAAAGAGGGAGAGCAAACCCCAACCAA

TTCATTGAGATAATGAGACAATTTTATGTGTGGAGTCTCATGTTCTTCAGATATCAGAATACATGTCTTT

TTCTTTAGCACTCTTGGATTGCTGGCAGCTTATTGATTGCTATCCTTGGCAGACCTGCAGAAGCAATATG

CTGATGTCGTGATCGAAGTTTTACCGACACAATTAATTCCTGATGACAATGAAAGGAAGGTGCTGAGAGT

TCGATTGGTGATGAAGGAAGGGGTGAAGTATTGCAATCCAGTTTATCTCTTAGATGAAGGCTCCACCG

>Chamaedorea_stolonifera_DQ177772.1

GTCTTGAGTGCATCAAAGCCAGCATTGGAGCGCAAAAGCTCGACTTTGATGCTTATGTTGGTATGTTTCT

TTGCCACAGTGCCTAGTTATTGGTACTTGAAGAATTGGCAGGGCATTCATTTGTTGGTATAATATAGAGC

AATTTCATTAGTTTTGATTGCCAAATATAGTACTATACTTCAATATCTAGCAAATGCTTCATTAAAAAAA

AAAACTCACGAACAAAGTATCTGATCCATAAGTCAAATAATGCACAAAACATACTGGACTAAAAATATAA

TCACTCCATGAGAAATACTTTTGGATACCTTTTTCTGAAAAAAAGAAAGAGGGAGAGCAAACCCCAACCA

ATTCATTGAGATAATGAGACAATTTTATGTGTGGAGTCTCATGTTCTTCAGATATCAGAATACATGTCTT

TTTCTTTAGCACTCTTGGATTGCTGGCAGCTTATAGATTGCTACCCTTGGCAGACCTGCAGAAGCAATAT

GCTGATGTCGTGATCGAAGTTTTACCGACACAATTAATTCCTGATGACAATGAAAGGAAGGTGCTGAGAG

TTCGATTGGTGATGAAGGAAGGGGTGAAGTATTGCAATCCAGTTTACCTCTTAGATGAAGGCTCCACCG

>Chamaedorea_sartorii_DQ177770.1

GTCTTGAGTGCATCAAAGCCAGCATTGGAGCGCGAAAGCGCGACTTTGATGCTTATGTTGGTATGTTTCT

TTGCCACAGTGCCTAGTTATTGGTACTTGAAGAATTGGCAGGGCATTCATTTGTTGGTATAATATAGAGC

AATTTCATTAGTTTTGATTGCCAAATATAGTACTATACTTCAATATCTAGCAAATGCTTCATTAAAAAAA

AAACTCACTAACAAAGTATCTGATCCATAAGTCAAATAATGCACAAAACATACTGGACTAAAAATATAAT

CACTCCATGAGATATACTTTTGGATACCTTTTTCTGAAAAAAAGAAAGAGGGAGAGCAAACCCCAACCAA

TTCATTGAGATAATGAGACAATTTTATGTGTGGAGTCTCATGTTCTTCAGATATCAGAATACATGTCTTT

TTCTTTAGCACTCTTGGATTGCTGGCAGCTTATAGATTGCTACCCTTGGCAGACCTGCAGAAGCAATATG

CTGATGTCGTGATCGAAGTTTTACCGACACAATTAATTCCTGATGACAATGAAAGGAAGGTGCTGAGAGT

TCGATTGGTGATGAAGGAAGGGGTGAAGTATTGCAATCCAGTTTACCTCTTAGATGAAGGCTCCACCG

>Chamaedorea_tuerckheimii_DQ177768.1

GTCTTGAGTGCATCAAAGCCAGCATTGGAGCTCGAAAGCTCGACTTTGATGCTTATGTTGGTATGTTTCT

TTGCCACAGTGCCTAGTTATTGGTACTTGAACAATTGGCAGAGCATTTATTTGTTGGTATAATATAGAGC

AATTTCATTAGTTTTGATTGCCAAATATAGTACTATACTTCAATATCTAGCAAATGCTTCATTAAAAAAA

GAACTCGCTAACAAAGTATCTGATCCATAAGTCAAATAATGCACAAAACATACTGGACTAAAAATATAAT

CACTCCATGAGAAATACTTTTGGATACCTTTTTCTGGAAAAAAGAAAAAGGGAGAGCAAACCCCAACCAA

TTCATTGAGATAATTAGACAATTTTATGTGTGGAGTCTCCATGTTCTTCAGATATCAGAATACATGTCTT

TTTCTTTAGCACTCTTGGATTGCTGGCAGCTTATTGATTGCTACCCTTGGCAGACCTGCAGAAGCAATAT

GCTGATGTCGTGATCGAAGTTTTACCGACACAATTAATTCCTGATGACAATGAAAGGAAGGTGCTGAGAG

TTCGATTGGTGATGAAGGAAGGGGTGAAGTATTGCAATCCAGTTTACCTCTTAGATGAAGGCTCCACCG

>Chamaedorea_nationsiana_DQ177766.1

GTCTTGAGTGCATCAAAGCCAGCATTGGAGCTCGAAAGCTAGACTTTGATGCTTATGTTGGTATGTTTCT

TTGCCACAGTGCCTAGTTATTGGTACTTGAAGAATTGGCAGAGCATTTATTTGTTGGTATAATATAGAGC

AATTTCATTAGTTTTGATTGCCAAATATAGTACTATACTTCAATATCTAGCAAATGCTTCATTAAAAAAA

GAACTCSCTAACAAAGTATCTGATCCATAAGTCAAATAATGCACAAAACATACTGGACTAAAAATATAAT

CACTCCATGAGAAATACTTTTGGATACCTTTTTCTGGAAAAAAGAAAGAGGGAGAGCAAACCCCAACCAA

TTCATTGAGATAATTAGACAATTTTATGTGTGGAGTCTCATGTTCTTCAGATATCAGAATACATGTCTTT

TTCTTTAGCACTCTTGGATTGCTGGCAGCTTATAGATTGCTGGCAGCTTATTGATTGCTACCCTTGGCAG

ACCTGCAGAAGCAATATGCTGATGTCGTGATCGAAGTTTTACSGACACAATTAATTCCTGATGACAATGA

AAGGAAGGTGCTGAGAGTTCGATTAGTGATGAAGGAAGGGGTGAAGTATTGCAATCCAGTTTACCTCTTA

GATGAAGGCTCCACCG

>Chamaedorea_brachypoda_DQ177764.1

GTCTTGAGTCTATCAAAGCCAGCATTGGAGCTCGAAAGCTTGACTTTGATGCTTATGTTGGTATGTTTCT

TTGCCACAGTGCCTAGTTATTGGTACTTGAAGAATTGGCAGAGCATTCATTTGTTGGTATAATATAGAGC

AATTTCATTAGTTTTGATAGCCAAATATAGTACTATACTTCAATATCTAGTAAATGCTTCATTAAAAAAA

AAACTCACTAACAAAGTATCTGATCCATAAGTCAAATAATGCACAAAACATTCTGGACTAAAAATATAAT

CACTCCATGAGAAATACTTTTGGATACCTTTTTCTGAAAAAAAGAAAGAGGGAGAGCAAACCCCAACCAA

TTCATTGAGATAATGAGACAATTTTATGTGTGGAGTCTCATGTTCTTCAGATGTCAGAATACATGTCTTT

TTCTTTAGCACTCTTGGATTGCTGGCAGCTTATAGATTCCTACCCTTGGCAGACCTGCAGAAGCAATATG

CTGATGTCGTGATCGAAGTTTTACCGACACAATTAATTCCTGATGACAATGAAAGGAAGGTGCTGAGAGT

TAGATTGGTGATGAAGGAAGGGGTGAAGTATTGCAATCCAGTTTACCTCTTAGATGAAGGCTCCACG

>Chamaedorea_tepejilote_DQ177762.1

GTCTTGAGTGCATCAAAGCCAGCATTGGAGCTCGAAAGCTCGACTTTGATGCTTATGTTGGTATGTTTCT

TTGCCACAGTGCCTAGTTATTGGTACTTGAACAATTGGCAGAGCATTTATTTGTTGGTATAATATAGAGC

AATTTCATTAGTTTTGATTGCCAAATATAGTACTATACTTCAATATCTAGCAAATGCTTCATTAAAAAAA

GAACTCGCTAACAAAGTATCTGATCCATAAGTCAAATAATGCACAAAACATACTGGACTAAAAATATAAT

CACTCCATGAGAAATACTTTTGGATACCTTTTTCTGGAAAAAAGAAAAAGGGAGAGCAAACCCCAACCAA

TTCATTGAGATAATTAGACAATTTTATGTGTGGAGTCTCCATGTTCTTCAGATATCAGAATACATGTCTT

TTTCTTTAGCACTCTTGGATTGCTGGCAGCTTATTGATTGCTACCCTTGGCAGACCTGCAGAAGCAATAT

GCTGATGTCGTGATCGAAGTTTTACCGACACAATTAATTCCTGATGACAATGAAAGGAAGGTGCTGAGAG

TTCGATTGGTGATGAAGGAAGGGGTGAAGTATTGCAATCCAGTTTACCTCTTAGATGAAGGCTCCACCG

>Chelyocarpus_ulei_EU215491.1

TTATTGAGTGCATCATGGGAAAGGTTGCTGCCCATATGGGAAAAGAGGGAGATGCTACTCCTTTTACTGA

TGTCACAGTAAGTAAATCCATGGAACCTTAATGCAAGCTTTACCTGAGTAGCATGTCACAATTTAACTTG

CTATAAATGCTGGAGCTTCTTTGCTTATTCAGTTTAAGAATTCCAAGTCAGTGTATCTGATGTCAAAGAA

CATTTTGGAAGGTTGCTTTTGTTCTGCTCCAGGCGGAAGGAATATTTGCATGTCTAGCTAAAAGACATCG

TTGGTAGCTTATACAAAAACACATGGTTATCCAATTTGCATTACCATTAAGGATTTGTACTAGATACGGT

TTAATAAATATGTTTGAGAAAGAGCATTTGCCTTTGGAGTTCATATTTTCATCTGAATTAGTTAATTATA

ATCCTGCTATTTGAAGTCTACTTTAGATGTCTCTTTTGAAGTTCTTGATATGTAAGTTTACCCTATAGAC

AGAAATGTGGCTTTGAAGAATGACCTTGGACAAGTGTCTTGTAAGTTATTCCGATGATCCTAAACTTGTA

CAAGTTTCATGGCATACTATTTTGACTGAGGTCTCTCCTAAATTATATAATAGACTCTCTCCTTGTGACT

GACCTGTGACATGGGTTTTCCGGTTCAATGATATATCAGGAGTTCCATCCTAAATAATGTTTCTAACATT

GATTTCAGATATATTCTGATGTAGGAGTTTAATACGTAATTGTCTTTTTGTAATTTCCCCATTACAGAAA

ATGCTTATATTATCACAAATTTCCTAGGTGGACAATATCAGCAAAGCTCTTCGTAAGTGTGGATATCAGA

>Chelyocarpus_dianeurus_JQ417485.1

TTGCCTTTGGAGTTCATTTTTTCCTCTGGTTTACTTAATTCTAATCCTGTTATTTGAGTCTACTTTGAAT

GTCTCTTTTGAAGTTCTTGAGATGTAAGTTTACCCTGTAGACAGAAACGTGGCTTTGAAAAATGACCTTG

GATGAGTGTCTTGTAAGTTATTCTGATGATCCTAAACTTGTACAAGTTTCAAGACATATTATCTTGACTA

AGGTCTCTTTTAAATTATATAGTAGACTATCTCCTTGTAACTGGCCTGTGACTTGGATTTTCCAGTTCAA

TGATCTATCAGGAGTTCTATCCTAAATAATGTTTCTAACATTGATTTCAGATGTATTCTGATGTAGGAGT

TTAATATGTAATTGTTCTTTTGTAATTGTCCCATTACAAAAAATACTTATATTATCAGGTGGACAA

>Coccothrinax_borhidiana_gEU215510.1

TTATTGAGTGCATCATGGGAAAGGTTGCTGCCCATATGGGAAAAGAGGGAGATGCTACTCCTTTTACTGA

TGTCACAGTATTTAAATCCATAAAACCTTCATGTGAGCTTTACCTGTGAAGCATGTCACAATTTAACTTG

CTATAGATGCTGGAGCTTGTTTGCTTATTCAGTTTAAGAATTCCGAGGCACTGTGTCTGATGATGTTAAA

GAACATTAAGGAAAGTTTCTTTTGTTCTGCTCCAGGAGGAAGTAGTTTTTGAAGATGTCTAGCTAAAAGA

CATTGTTGATAGCTTATACAAGACAGAAGGTTATCCAATTTGCATTACCATTAAGGATTTGTACTAGATA

TGGTTCAATAAATATGTTTGAGAACTGGAGCTTTTGCCTTTGTAGTTCATTTTTTCCTCTGGCTTACTTA

ATTCTAAATTTCTAATCCTGTTATTTGAGTCTACTTTGAGTATCTCTTTTGAAGTTCTTGAGATGTAAGT

TTACCCTGTAGACAGAAACGTGGCTTTGAAGAATGACCTTGGATGAGTGTCTTGTAAGTTATTCTGATGA

TCCTAAACTTGTACAAGTTTCAAGACATACTGTCTTGACTAAGGTCTCTTCTAAATTATATAGTAGACTA

TGACTAAGGTCTCTTCTAAATTATATAGTAGACTATCTCCTCGTAACCGGCCTGTGACTTGGATTTTCCA

ATTCAATGATCTATCAGGAGTTCTATCCTAAATAATGTGTCTAACATTGATTTCAGATGTATTCTGATGT

AGGAGTTTAATATGTAATTGTTCTTTTGTAATTGCCCCATTACAAAAAATACTTATTTTATCAGGTGGAC

AATATAAGCAAAGCTCTTCATAAGTGTGGATATCAGA

>Coccothrinax_crinita_subsp._crinita_EU215506.1

TTATTGAGTGCATCATGGGAAAGGTTGCTGCCCATATGGGAGAAGAGGGAGATGCTACTCCTTTTACTGA

TGTCACAGTAAGTAAATCCATAAAACCTTCATGTTAGCTTTACCTGTGAAGCATGTCACAATTTAACTTG

CTATAGATGCTGGTGCTTGTTTGCCTATTCAGTTTAAGAATTCCGAGGCACTGTGTCTGATGATGTTAAA

GAACATTAAGGAAAGTTTCTTTTGTTCTGCTCCAGGAGGAAGTAGTTTTTGCAGATGTCTAGCTAAAAGA

CATTGTTGATAGCTTATACAAGACAGAAGGTTATCCAATTTGCATTACCATTAAGGATTTGTACTAGATA

TGGTTCAATAAATATGTTTGAGAACTGGAGCCTTTGCCTTTGTAGTTCGTTTTTTCCTCTGGCTTACTTA

ATTCTAAATTTCTAATCCTGTTATTTGAGTCTACTTTGAGTATCTCTTTTGAAGCTCTTGAGATGTAAGT

TTACCCTGTAGACAGAAACGTGGCTTTGAAGAATGACCTTGGATGAGTGTCTTGTAAGTTATTCTGATGA

TCCTAAACTTGTACAAGTTTCAAGACATACTGTCTTGACTAAGGTCTCTTCTAAATTATACAGTAGACTA

TGACCAAGGTCTCTTCTAAATTATATAGTAGACTACCTCCTCGTAACCGGCCTGTGACTTGGATCTTCCA

ATTCAATGATCTATCAGGAGTTCTATCCTAAATAATGTGTCTAACATTGATTTCAGATGTATTCTGATGT

AGGAGTTTAATATGTAATTGTTCTTTTGTAATTGCCCCATTACAAAAAGTACTTATTTTATCAGGCGGAC

AATATAAGCAAAGCTCTTCATAAGTGTGGATATCAGA

>Coccothrinax_crinita_subsp._brevicrinis_EU215504.1

TTATTGAGTGCATCATGGGAAAGGTTGCTGCCCATATGGGAAAAGAGGGAGATGCTACTCCTTTTACTGA

TGTCACAGTAAGTAAATCCATAAAACCTTCATGTTAGCTTTACCTGTGAAGCATGTCACAATTTAACTTG

CTATAGATGCTGGTGCTTGTTTGCTTATTCAGTTTAAGAATTCCGAGGCACTGTGTCTGATGATGTTAAA

GAACATTAAGGAAAGTTTCTTTTGTTCTGCTCCAGGAGGAAGTAGTTTTTGCAGATGTCTAGCTAAAAGA

CATTGTTGACAGCTTATACAAGACAGAAGGTTATCCAATTTGCATTACCATTAAGGATTTGTACTAGATA

TGGTTCAATAAATATGTTTGAGAACTGTCACAATCCTCCTCCCCCCACCCAAAAAAAAAAAAAAAAAAAA

CCAAAAACACACACACACCCACCCAAAACCCAAAATAAAAGAAAGAAGAAGAAGAAGAAAAACCCCTGCC

CGGCAAGGCGCCACGTGTGCTGTGGCTTGTTTAGAACTTTTTTTCAACTTCACACCTGGATTTTTATTTT

TATTTTTGAGGGGAGCTTTTGCCTTTGTAGTTCATTTTTTCCTCTGGCTTACTTAATTCTAAATTTCTAA

TCCTGTTATTTGAGTCTACTTTGAGTATCTCTTTTGAAGTTCTTGAGATGTAAGTTTACCCTGTAGACAG

AAACGTGGCTTTGAAGAATGACCTTGGATGAGTGTCTTGTAAGTTATTCTGATGATCCTAAACTTGTACA

AGTTTCAAGACATACTGTCTTGACTAAGGTCTCTTCTAAATTATATAGTAGACTATGACTAAGGTCTCTT

CTAAATTATATAGTAGACTATCTCCTCGTAACCGGCCTGTGACTTGGATTTTCCAATTCAATGATCTATC

AGGAGTTCTATCCTAAATAATGTGTCTAACATTGATTTCAGATGTATTCTGATGTAGGAGTTTAATATGT

AATTGTTCTTTTGTAATTGCCCCATTACAAAAAATACTTATTTTATCAGGTGGACAATATAAGCAAAGCT

CTTCATAAGTGTGGATATCAGA

>Coccothrinax_inaguensis_EU215502.1

TTATTGAGTGCATCATGGGAAAGGTTGCTGCCCGTATGGGAAAAGAGGGAGATGCTACTCCTTTTACTGA

TGTCACAGTAAGTAAATCCATAAAACCTTCATGTGAGCTTTACCTGTGAAGCATGTCACAATTTAACTTG

CTATAGATGCTGGAGCTTGTTTGCTTATTCAGTTTAAGAATTCCGAGGCACTGTGTCTGATGATGTTAAA

GAACATTAAGGAAAGTTTCTTTTGTTCTGCTCCAGGAGGAAGTAGTTTTTGCAGATGTCTAGCTAAAAGA

CATTGTTGATAGCTTATACAAGACAGAAGGTTATCCAATTTGCATTACCATTAAGGATTTGTACTAGATA

TGGTTCAATAAATATGTTTGAGAACTGGAGCTTTTGCCTTCGTAGTTCATTTTTTCCTCTGGCTTACTTA

ATTCTAAATTTCTAATCCTGTTATTTGAGTCTACTTTGAGTATCTCTTTTGAAGTTCTTGAGATGTAAGT

TAACCCTGTAGACAGAGACGTGGCTTTGAAGAATGACCTTGGATGAGTGTCTTGTAAGTTATTCTGATGA

TCCTAAACTTGTACAAGTTTCAAGACATACTGTCTTGACTAAGGTCTCTTCTAAATTATATAGTAGACTA

TGACTAAGGTCTCTTCTAAATTATATAGTAGACTATCTCCTCGTAACCCGCCTGTGACTTGGATTTTCCA

ATTCAATGATCTATCAGGAGTTCTATCCTAAATAATGTGTCTAACATTGATTTCAGATGTATTCTGATGT

AGGAGTTTAATATGTAATTGTTCTTTTGTAATTGCCCCATTACAAAAAATACTTATTTTATCAGGTGGAC

AATATAAGCAAAGCTCTTCATAGGTGTGGATATCAGA

>Coccothrinax_salvatoris_EU215500.1

TTATTGAGTGCATCATGGGAAAGGTTGCTGCCCATATGGGAAAAGAGGGAGATGCTACTCCTTTTACTGA

TGTCACAGTAAGTAAATCCATAAAACCTTCATGTGAGCTTTACCCGTGAAGCATGTCACAATTTAACTTG

CTATAGATGCTGGAGCTTGTTTGCTTATTCAGTTTAAGAATTCCGAGGCACTGTGTCTGATGATGTTAAA

GAACATTAAGGAAAGTTTCTTTTGTTCTGCTCCAGGAGGAAGTAGTTTTTGCAGATGTCTAGCTAAAAGA

CATTGTTGATAGCTTATACAAGACAGAAGGTTATCCAATATGCATTACCATTAAGGATTTGTACTAGATA

TGGTTTAATAAATATGTTTGAGAACTGGAGCTTTTGCCTTTGTAGTTCATTTTTTCCTCTGGCTTACTTA

ATTCTAAATTTCTAATCCTGTTATTTGAGTCTACTTTGAGTATCTCTTTTGAAGTTCTTGAGATGTAAGT

TTACCCTGTAGACAGAAACGTGGCTTTGAAGAATGACCTTGGATGAGTGTCTTGTAAGTTATTCTGATGA

TCCTAAACCTGTACAAGTTTCAAGACATACTGTCTTGACTAAGGTCTCTTCTAAATTATATAGTAGACTA

TGACTAAGGTCTCTTCTAAATTATATAGTAGACTATCTCCTCGTAACCGGCCTGTGACTTGGATTTTCCA

ATTCAATGATCTATCAGGAGTTCTATCCTAAATAATGTGTCTAACATTGATTTCAGATGTATTCTGATGT

AGGAGTTTAATATGTAATTGTTCTTTTGTAATTGCCCCATTACGAAAAATACTTATTTTATCAGGTGGAC

AATATAAGCAAAGCTCTTCATAAGTGTGGATATCAGA

>Coccothrinax_argentea_EU215507.1

TTATTGAGTGCATCATGGGAAAGGTTGCTGCCCATATGGGAAAAGAGGGAGATGCTACTCCTTTTACTGA

TGTCACAGTAAGTAAATCTATAAAACCTTCATGTGAGCTTTACCTGTGAAGCATGTCACAATTTAACTTG

CTATAGATGCTGGAGCTTGTTTGCTTATTCAGTTTAAGAATTCCGAGGCACTGTGTCTGATGATGTTAAA

GAACATTAAGGAAAGTTTCTTTTGTTCTGCTCCAGGAGGAAGTAGTTTTTGCAGATGTCTAGCTAAAAGA

CATTGTTGATAGCTTATACAAGACAGAAGGTTATCCAATTTGCATTACCATTAAGGATTTGTACTAGATA

TGGTTCAATAAATATGTTTGAGAACTGGAGCTTTTGCCTTTGTAGTTCATTTTTTCCTCTGGCTTACTTA

ATTCTAAATTTCTAATCCTGTTATTTGAGTCTACTTTGAGTATCTCTTTTGAAGTTCTTGAGATGTAAGT

TAACCCTGTAGACAGAAACGTGGCTTTGAAGAATGACCTTGGATGAGTGTCTTGTAAGTTATTCTGATGA

TCCTAAACTTGTACAAGTTTCAAGACATACTGTCTTGACTAAGGTCTCTTCTAAATTATATAGTAGACTA

TGACTAAGGTCTCTTCTAAATTATATAGTAGACTATCTCCTCGTAACCGGCCTGTGACTTGGATTTTCCA

ATTCAATGATCTATCAGGAGTTCTATCCTAAATAATGTGTCTAACATTGATTTCAGATGTATTCTGATGT

AGGAGTTTAATATGTAATTGTTCTTTTGTAATTGCCCCATTACAAAAAATACTTATTTTATCAGGTGGAC

AATATAAGCAAAGCTCTTCATAAGTGTGGATATCAGA

>Coccothrinax_spissa_EU215505.1

TTATTGAGTGCATCATGGGAAAGGTTGCTGCCCATATGGGAAAAGAGGGAGATGCTACTCCTTTTACTGA

TGTCACAGTAAGTAAATCTATAAAACCTTCATGTGAGCTTTACCTGTGGAGCATGTCACAATTTAACTTG

CTACAGATGCTGGAGCTTGTTTGCTTATTCAGTTTAAGAATTCCGAGGCACTGTGTCTGATGTTAAAGAA

CATTAAGGAAAGTTTCTTTTGTTCTGCTCCAGGAGGAAGTAGTTTTTGCAGATGTCTAGCTAAAAGACAT

TGTTGATAGCTTATACAAGACAGAAGGTTATCCAATTTGCATTACCATTAAGGATTTGTACTAGATATGG

TTCAATAAATATGTTTGAGAACTGGAGCTTTTGCCTTTGGAGTTCATTTTTTCCTCTGGTTTACTTAATC

CTAAATTTCTAATCCTGTTATTTGAGTCTACTTTGAGCATCTCCTTTGAAGTTCTTGAGATGTAAGTTTA

CCCTGTAGACAGAAACGTGGCTTTGAAGAATGACCTTGGATGAGTGTCTTGTAAGTTATTCTGATGATCC

TAAACTTGTACAAGTTTCAAGACATACTGTCTTGACTAAGGTCTCTTCTAAATTATATAGTAGACTATGA

CTAAGGTCTCTTCTAAATTATATAGTAGACTATCTCCTCGTAACCGGCCTGTGACTTGGATTTTCCAGTT

CAATGATCTATCAGGAGTTCTATCCTAAATAATGTTTCTAACATTGATTTCAGATGTATTCTGATGTAGG

AGTTTAATATGTAATTGTTCTTTTGTAATTGCCCCATTACAAAAAATACTTATTTTATCAGGTGGACAAT

ATAAGCAAAGCTCTTCATAAGTGTGGATATCAGA

>Coccothrinax_barbadensis_EU215503.1

TTATTGAGTGCATCATGGGAAAGGTTGCTGCCCATATGGGAAAAGAGGGAGATGCTACTCCTTTTACTGA

TGTCACAGTAAGTAAATCCATAAAACCTTCATGTGAGCTTTACCTGTGAAGCATGTCACAATTTAACTTG

CTATAGATGCTGGAGCTTGTTTGCTTATTCAGTTTAAGAATTCCGAGGCACTGTGTCTGATGTTAAAGAA

CATTAAGGAAAGTTTCTTTTGTTCTGCTCCGGGAGGAAGTAGTTTTTGCAGATGTCTAGCTAAAGGCATT

GTTGATAGCTTATACAACACAGAAGGTTATCCAATTTGCATTACCATTAAGGATTTGTACTAGATATGGT

TCAATAAATATGTTTGAGTACTGGAGCTTTTGCCTTTGGAGTTCATTTTTTCCTCTGGTTTACTTAATTC

TAAATTTCTAATCCTGTTATTTGAGTCTACTTTGAGTATCTCTTTTGAAGTTCTTGAGATGTAAGTTTAC

CCTGTAGACAGAAACGTGGCTTTGAAGAATGACCTTGGATGAGTGTCTTGTAAGTTATTCTGATGATCCT

AAACTTGTACCAGTTTCAAGACATACTGTCTTGACTAAGGTCTCTTCTAAATTATATAGTAGACTATGAC

TAAGGTCTCTTCTAAATTATATAGTAGACTATCTCCTCGTAACCGGCCTGTGACTTGGATTTTCCAATTC

AATGATCTATCAGGAGTTCTATCCTAAATAATGTTTCTAACATTGATTTCAGATGTATTCTGATGTAGGA

GTTTAATATGTAATTGTTCTTTTGTAATTGCCCCATTACAAAAAATACTTATTTTATCAGGTGGACAATA

TAAGCAAAGCTCTTCATAAGTGTGGATATCAGA

>Coccothrinax_miraguama_subsp._miraguama_EU215501.1

TTATTGAGTGCATCATGGGAAAGGTTGCTGCCCATATGGGAAAAGAGGGAGATGCTACTCCTTTTACTGA

TGTCACAGTAAGTAAATCCATAAAACCTTCATGTGAGCTTTACCTGTGAAGCATGTCACAATTTAACTTG

CTATAGATGCTGGAGCTTGTTTGCTTATTCAGTTTAAGAATTCCGAGGCACTGTGTCTGATGATGTTAAA

GAACATTAAGGAAAGTTTCTTTTGTTCTGCTCCAGGAGGAAGTAGTTTTTGCAGATGTCTAGCTAAAAAA

CATTGTTGATAGCTTATACAAGACAGAAGGTTATCCAATTTGCATTACCATTAAGGATTTGTACTAGATA

TGGTTCAATAAATATGTTTGAGAACTGGAGCTTTTGCCTTTGTAGTTCATTTTGTCCTCTGGCTTACTTA

ATTCTAAATTTCTAATCCTGTTATTTGAGTCTACTTTGAGTATCTCTTTTGAAGTTCTTGAGATGTAAGT

TTACCCTGTAGACGGAAACGTGGCTTTGAAGAATGACCTTGGATGAGTGTCTTGTAAGTTATTCTGATGA

TCCTAAACTTGTACAAGTTTCAAGACATACTGTCTTGACTAAGGTCTCTTCTAAATTATATAGTAGACTA

TGACTAAGGTCTCTTCTAAATTATATAGTAGACTATCTCCTCGTAACCGGCCTGTGACTTGGATTTTCCA

ATTCAATGATCTATCAGGAGTTCTATCCTAAATAATGTGTCTAACATTGATTTCAGATGTATTCTGATGT

AGGAGTTTAATGTGTAATTGTTCTTTTGTAATTGCCCCATTACAAAAAATACTTATTTTATCAGGTGGAC

AATATAAGCAAAGCTCTTCATAAATGTGGATATCAGA

>Coccothrinax_argentata_AM903107.1

GCCGTGGGAAAGAGGGAGATGCTACTCCTTTTACTGATSTCACAGTAAGTAAATCCATAAAACCTTCATG

CGAGCTTTACCTGTGAAGCATGTCACAATTTAACTTGCTATAGATGCTGGAGCTTGTTTGCTTATTCAGT

TTAAGAATTCCGAGGCACTGTGTCTGATGTTAAAGAACATTAAGGAAAGTTTCTTTTGTTCTGCTCCAGG

AGGAAGTAGTTTTTGCAGATGTCTAGCTAAAAGGCATTGTTGATAGCTTATACAACACAGAAGGTTATCC

AATTTGCATTACCATTAAGGATTTGTACTAGATATGGTTCAATAAATATGTTTGAGAACTGTCACAATCC

CCCCCCCCCCCAAAAAAAAAAACAAAAAAACCCCCCCCCCCCCCCAAACCCCAAAAMMMAAAAAAAAAAA

AAAAAAGAAAGAAGAAGAAGAAGAAAAACCCCTGCCCTGCAAGGCGCCACGTGTGCTGTGGCTTGTTTAG

AACTTTTTTTCAACTTCACACCTGGATTTTTATTTTTGAGGGGAGCTTTTGCCTTTGGAGTTCATTTTTT

CCTCTGGTTTACTTAATTCTAAATTTCTAATCCTGTTATTTGAGTCTACTTTGAGTATCTCTTTTGAAGT

TCTTGAGATGTAAGTTTACCCTGTAGACAGAAACGTGGCTTTGAAGAATGACCTTGGATGAGTGTCTTGT

AAGTTATTCTGATGATCCTAAACTTGTACCAGTTTCAAGACATACTGTCTTGACTAAGGTCTCTTCTAAA

TTATATAGTAGACTATGACTAAGGTCTCTTCTAAATTATATAGTAGACTATCTCCTCGTAACCGGCCTGT

GACTTGGATTTTCCAATTCAATGATCTATCAGGAGTTCTATCCTAAATAATGTTTCTAACATTGATTTCA

GATGTATTCTGATGTAGGAGTKTAATATGTAATTGTTCTTTTGTAATTGCCCCATTACAAAAAATACTTA

TDTGATCAGGTGGACAATAAC

>Cocos_nucifera_EF491150.1

CTACTCCTTTTACTGATGTCACAGTAAGTAAATCCATAGAACCTTCATGCAAGCTTTACCTGAGAAGCAT

GTCACAATTTAACTTGCTATAAATGCTGGAGCTTTTTCGCTTATTCAGTTTAAGAATTCCAAGTCACTGT

ATCTGATGTTATAGAACATCATGGAAAGTTTCTTTTGTTCTGCTCGAGAAGGAAGTAATTTTTGCATGTC

TTGCTAAAAGACATTGTTGTTAGCTCATACAAAAATGCATGGTTATTCAATTTGCATTACCATTAAGGAT

TTGTACTAGATATGGTTTAATTAATATGTTTGAGTTGCATTACCATTAAGAATTTGTACTAGACATACTA

TTTTGACTAAGGTCTCTCCTAAATTATGTCATTGACCATCTCCTTGTACCTGACCTGTGGCTTGGATTTT

CCAATTCAATGATCTATCAGGAGTTCTATCCTAAATAATGTTTCTAACATTGAATTCAGATATATTCTGA

TGTAGGGGTTTAATATGTAATTGTACTTTTGTAGTTGCTCCAT

>Colpothrinax_wrightii_HQ720497.1

GAGATGCTACTCCTTTTACTGATGTCACAGKAAGTAAATTCATGAAACCTTCATGCAAGCTTTACCTGAG

AAGCATGTCACAATTTAACTTGCTATAAATGCTGGAGCTTTTTTGCTTATTCAGTTTAAGAATTCTGAGT

CACTGTATCTGATGTTAAAGAACATTAAGGAAAGTTTCTTTTGTGCTGCTCCAGGAGGAAGTAATTTTTG

TGTATGTCTAGCTAGAAGACATTGTTGATAGCTTGTACAAAAACAGAAGGTTATTCAATTTGCATTACCA

TTAAGGATTTGTACTAGATATGGTTTAATAAATATGTTAAATATGCTTGAGAACCACAACACCCCCCCCC

CCCCCCCCCTACTCTGTAAACAGAAACGTGGCTTAGCAGAAGGACTTTGGACGGGTGTCTTGTTAGTTAT

TCTGATGATCCTAAACTTGTATAAGTTTCAAGACATACTATTTTGGCTATTTTAACTTGTATAGTAGACT

ATCTCCTTGTAACTGGCCTGTGACTTGGATTTTCCAATTCAATGATCTATCAGGAGTTCTATCCTAAATA

ATGTTTCTAACATTGATTTCAGATGTATTCTGATGTTGGAGTTTAATATGTAATTGTTCTTTTGTAATTG

CCCCATTACCAAAAAATACTTATATTATCAC

>Colpothrinax_wrightii_HQ720498.1

AAACCCTCATGCAAGCTTTACCTGAGAAGCATGTCACAATTTAACTTGCTATAAATGCTGGAGCTTTTTT

GCTTATTCAGTTTAAGAATTCTGAGTCACTGTATCTGATGTTAAAGAACATTAAGGAAAGTTTCTTTTGT

GCTGCTCCAGGAGGAAGTAATTTTTGTGTATGTCTAGCTAGAAGACATTGTTGATAGCTTGTACAAAAAC

AGAAGGTTATTCAATTTGCATTACCATTAAGGATTTGTACTAGATATGGTTTAATAAATATGTTAAATAT

GCTTGAGAACCACAACACCCCCCCCCCCCCCCCCCCCTACTCTGTAAACAGAAACGTGGCTTAGCAGAAG

GACTTTGGACGGGTGTCTTGTTAGTTATTCTGATGATCCTAAACTTGTATAAGTTTCAAGACATACTATT

TTGGCTATTTTAACTTGTATAGTAGACTATCTCCTTGTAACTGGCCTGTGACTTGGATTTTCCAATTCAA

TGATCTATCAGGAGTTCTATCCTAAATAATGTTTCTAACATTGATTTCAGATGTATTCTGATGTTGGAGT

TTAATATGTAATTGTTCGGTTGTAATTGCCCCATTACCAAAAAATAC

>Colpothrinax_aphanopetala_HQ720496.1

CTACTCCTTTTACTGATGTCACAGKAAGTAAAGACATAAAACCTTCATGCAAGCTACCTGAGAAGCGTGT

CATAATTTAACCTGCTATAAATGCTGGAGCTTTTTTGCTTATTCAGTTTAAGAATTCCGAGTCACTGTAT

GTGATGTTAAAGAACATTAAGGAAAGTTTCTTTTGTTCTGCTCCAGGAGGAAGTAATTTTTGCATATGTC

TAGCTAAAAGACATTGTTGATAGCTTATRCAAGAACAGAAGGTTATTCAATTTTCATTACCATTAAGGAT

TTGTATTAGATATGGTTTAGTTAATTATAATCCTTTCAACTTCATACCTGGATTTGATTTTCTTTTGAGG

GGAGCTTTTGCCTTTGGAGTTCATATTTTCCTCTGGTTTAGTTAATTATAATCCTGTTATTTGAAGTCTA

CTTTGAATATCTCTTTTGAAGTTCTTGAGATGTAAGYTTACCCTGTAGACAGAAACGTGGCTCTGAAGAA

TGACTTTGGACGAGTGTAAGTTATTCTGATGATCCTAAACTTGTATAAGTTTCAAGACATACTATTTTGA

CTAAGGTCTCTTCTAAATTATATAGTAGACTATCTCCTTGTAACTGGCCTGTGACTTGGATTTTCCAATT

CGATGATCTATCAGTAGTTCTATCCTAAATAATGTTTCTAACATTGATTTCAGATGCATTCTGATATAGG

AGTTCAATATGTAATTGTTCTTTTGTAATTTCCCCATTACAAAAAATACTTATATTATC

>Colpothrinax_wrightii_EU215499.1

TTATTGAGTGCATCATGGGAAAGGTTGCTGCCCATATGGGAAAGGAGGGAGATGCTACTCCTTTTACTGA

TGTCACAGTAAGTAAATACATAAAACCTTCATGCAAGCTTTACCTGAGAAGCGTGTCATAATTTAACCTG

CTATAAATGCTGGAGCTTTTTTGCTTATTCAGTTTAAGAATTCCTAGTCACTGTATCTGATGTTAAAGAA

CATTAAGGAAAGTTTCTTTTGTTCTGCTCCAGGAGGAAGTAATTTTTGCATTTGTCTAGCTAAAAGACAT

TGTTGATAGCTTATACAAAAACAGAAGGTTATTCAATTTTCATTACCATTGTGGATTTGCATCAGATATG

GTTTAGTTAATTATAATCCTTCCAACTTCATACCTGGATTTGATTTTCCTTTGAGGGGAGCTTTTGCCTT

TGGAGTTCATATTTTCCTCTGGTTTAGTTAATTATATTCCTGTTATTTGAAGTCTACTTTGAATATCTCT

TTTGAAGTTTTTGAGATGTAAGTTTACCCTGTAGACAGAAACGTGGCTTTGAAGAATGACTTTGGACGAG

TGTAAGTTATTCTGATGATCCTAAACTTGTATAAGTTTCAAGACATACTATTTTGACTAAGGTCTCTTCT

AAATTATTAGTAGACCATCTCCTTGTAACTGGCCTGTGACTTGGATTTTCCAATTCGATGATCTATCGGG

AGTTCTATCCTAAATACTGTTTCTAACATTGATTTCAGATGCATTCTGATATAGGAGGTCAATATGTAAT

TGTTCTTTTGTAATTTCCCCATTACAAAAAATACTTATATCGTCACAAATTTCCTAGGTGGACAATATAA

GCAAAGCTCTTCATAAGTGTGGATATCAGA

>Copernicia_yarey_HQ720513.1

ATGTCACAGTAAGTAAATACATAAAMCCTTCATGCAAGCTTTACCTGAGAAGCATATCATAGTTTAACCT

GCTATAAATGCTTGAGCTTTTTTGTTTATTCAGTTTAAGAATTCTGAGTCACTGTATCCGATGTTGTTGA

ACATTAAGGAAAGTTTCTTTTGTTCTGCTCCAGGAGGAATTAATTTTTGCATATGTCTAGGTAAAAGACA

TTGTTGATAGCTTAGACAGAAGGTTATTCAATTTGCATTACCATTAAGGATTTGTATTAGATATGGTTTG

GTTAATTATAATCCTTTCAACTTCATACATGGATTTGATTTTCTTTTGAGGGGAGCTTTTGCCTTTGGAG

TTCATACTTTCCTCTGGTTTAGTTAATTATACTCCTGTTATTTGAACTSTACTTTGAATGTCTCTTTTTG

AAGTTCTTGAGATGTAAGTTTACCCTGTAGACAGAAACGTGGCTTTTCAGAATGACTTTGGACGAATGTC

TTGCAAGTTGTTATGATGATCCTAAACTTGTATAAGTTTCAGACATACTATTTTGACTAAGGTCTCTTCT

AAATTATATAGTGGACTATCTTCTTGTAACTGGCCTATGACTTGGATTTTCCAATTTAATKATCTATCAG

GAGTTCTATAATAAGAATGTTTCTAACATTGTTTTCAGATGCATTCTGATATAGGAGTTCAATATGTAAT

CGTTCTTTTGTAATTTTCCCATTGCAAAAAATACTTATATTATCACAAAT

>Copernicia_rigida_HQ720511.1

GGAGACGCTACTCCTTTTACTGATGTCACAGTAAGTAAATACATAAAACCTTCATGCAAGCTTTACCTGA

GAAGCATATCATAGTTTAACCTGCTATAAATGCTTGAGCTTTTTTGTTTATTCAGTTTAAGAATTCTGAG

TCACTGTATCCGATGTTGTTGAACATTAAGGAAAGTTTCTTTTGTTCTGCTCCAGGAGGAATTAATTTTT

GCATATGTCTAGGTAAAAGACATTGTTGATAGCTTAGACAGAAGGTTATTCAATTTGCATTACCATTAAG

GATTTGTATTAGATATGGTTTGGTTAATAATAATCCTTTCAACTTCATACATGGATTTGATTTTCTTTTG

AGGGGAGCTTTTGCCTTTGGAGTTCATACTTTCCTCTGGTTTAGTTAATTATACTCCTGTTATTTGAACT

CTACTTTGAATGTCTCTTTTTGAAGTTCTTGAGATGTAAGTTTACCCTGTAGACAGAAACGTGGCTTTTC

AGAATGACTTTGGACGAATGTCTTGCAAGTTGTTATGATGATCCTAAACTTGTATAAGTTTCAGACATAC

TATTTTGACTAAGGTCTCTTCTAAATTATATAGTGGACTATCTTCTTGTAACTGGCCTATGACTTGGATT

TTCCAATTTAATGATCTATCAGGAGTTCTATAATAAGAATGTTTCTAACATTGTTTTCAGATGCATTCTG

ATATAGGAGTTCAATATGTAATCGTTCTTTTGTAATTTTCCCATTGCAAAAAATACTTATATTATC

>Copernicia_macroglossa_HQ720509.1

GAAAGGTTGCTGCCCATATGGGAAAGGAGGGAGACGCTACTCCTTTTACTGATGTCACAGTAAGTAAATA

CATAAAACCTTCATGCAAGCTTTACCTGAGAAGCATATCATAGTTTAACCTGCTATAAATGCTTGCGCTT

TTTTGTTTATTCAGTTTAAGAATTCTGAGTCACTGTATCCGATGTTGTTGAACATTAAGGAAAGTTTCTT

TTGTTCTGCTCCAGGAGGAATTAATTTTTGCATATGTCTAGGTAAAAGACATTGTTGATAGCTTAGACAG

AAGGTTATTCAATTTGCATTACCATTAAGGATTTGTATTAGATATGGTTTGGTTAATTATAATCCTTTCA

ACTTCATACATGGATTTGATTTTCTTTTGAGGGGAGCTTTTGCCTTTGGAGTTCATACTTTCCTCTGGTT

TAGTTAATTATACTCCTGTTATTTGAACTCTACTTTGAATGTCTCTTTTTGAAGTTCTTGAGATGTAAGT

TTACCCTGTAGACAGAAACGTGGCTTTTCAGAATGACTTTGGACGAATGTCTTGCAAGTTGTTATGATGA

TCCTAAACTTGTATAAGTTTCAGACATACTATTTTGACTAAGGTCTCTTCTAAATTATATAGTGGACTAT

CTTCTTGTAACCGGCCAATGACTTGGATTTTCCAATTTAATGATCTATCAGGAGTTATATAATAAGAATG

TTTCTAACATTGTTTTCAGATGCATTCTGATATAGGAGCTCAATATGTAATCGTTCTTTTGTAATTTTCC

CATTGCAAAAAATACTTATATTATCACAAATTTCCTAGGTGGACAATATCAGCAAAGCTCTTCATAAGT

>Copernicia_hospita_HQ720507.1

AGGAGGGAGACGCTACTCCTTTTACTGATGTCACAGTAAGTAAATACATAAAACCTTCATGCAAGCTTTA

CCTGAGAAGCATATCATAGTTTAACCTGCTATAAATGCTTGAGCTTTTTTGTTTATTCAGTTTAAGAATT

CTGAGTCACTGTATCCGATGTTGTTGAACATTAAGGAAAGTTTCTTTTGTTCTGCTCCAGGAGGAATTAA

TTTTTGCATATGTCTAGGTAAAAGACATTGTTGATAGCTTAGACAGAAGGTTATTCAATTTGCATTACCA

TTAAGGATTTGTATTAGATATGGTTTGGTTAATTATAATCCTTTCAACTTCATACATGGATTTGATTTTC

TTTTGAGGGGAGCTTTTGCCTTTGGAGTTCATACTTTCCTCTGGTTTAGTTAATTATACTCCTGTTATTT

GAACTGTACTTTGAATGTCTCTTTTTGAAGTTCTTGAGTTGTAAGTTTACCCTGTAGACAGAAACGTGGC

TTTTCAGAATGACTTTGGACGAATGTCTTGCAAGTTGTTATGATGATCCTAAACTTGTATAAGTTTCAGA

CATACTATTTTGACTAAGGTCTCTTCTAAATTATATAGTGGACTATCTTCTTGTAACTGGCCTATGACTT

GGATTTTCCAATTTAATGATCTATCAGGAGTTCTATAATAAGAATGTTTCTAACATTGTTTTCAGATGCA

TTCTGATATAGGAGTTCAATATGTAATCGTTCTTTTGTAATTTTCCCATTGCAAAAAATACTTATATTAT

CACAAATTTCCTAGGTGG

>Copernicia_glabrescens_HQ720505.1

GCTTTACCTGAGAAGCATATCATAGTTTAACCTGCTATAAATGCTCGAGCTTTTTTGTTTATTCAGTTTA

AGAATTCTGAGTCACTGTATCCGATGTTGTTGAACATTAAGGAAAGTTTCTTTTGTTCTGCTCCAGGAGG

AATTAATTTTTGCATATGTCTAGGTAAAAGACATTGTTGATAGCTTTGACAGAAGGTTATTCAATTTGCA

TTACCATTAGGATTTGTATTAGATATGGTTTGGTTAATTATAATCCTTTCAACTTCATACAKGGATTTGA

TTTTCTTTTGAGGGGAGCTTTTGCCTTTGGAGTTCATACTTTCCTCTGGTTTAGTTAATTATACTCCTGT

TATTTGAACTCTACTTGGAAGGTCTCTTTTKGAAGTTCTTGAGATGTAAGTTTACCCTGTAGACAGAAAC

GTGGCTTTTCAGAATGACTTKGGACGAATGTCTTGCAAGTTGTTATGATGATCCTAAACTTGTATAAGTT

TCAGACATACTATTTTGACTAAGGTCTCTTCTAAATTATA

>Copernicia_ekmanii_HQ720503.1

TTTACTGATGTCACAGTAAGTAAATACATAAAACCTTCATGCAAGCTTTACCTGAGAAGCATATCATAGT

TTAACCTGCTATAAATGCTTGAGCTTTTTTGTTTATTCAGTTTAAGAATTCTGAGTCACTGTATCCGATG

TTGTTGAACATTAAGGAAAGTTTCTTTTGTTCTGCTCCAGGAGGAATTAATTTTTGCATATGTCTAGGTA

AAAGACATTGTTGATAGCTTAGACAGAAGGTTATTCAATTTGCATTACCATTAAGGATTTGTATTAGATA

TGGTTTGGTTAATTATAATCCTTTCAACTTCATACATGGATTTGATTTTCTTTTGAGGGGAGCTTTTGCC

TTTGGAGTTCATACTTTCCTCTGGTTTAGTTAATTATACTCCTGTTATTTGAACTCTACTTTGAATGTCT

CTTTTTGAAGTTCTTGAGATGTAAGTTTACCCTGTAGACAGAAACGTGGCTTTTCAGAATGACTTTGGAC

GAATGTCTTGCAAGTTATTATGATGATCCTAAACTTGTATAAGTTTCAGACATACTATTTTGACTAAGGT

CTCTTCTAAATTATATAGTGGACTATCTTCTTGTAACTGGCCTATGACTTGGATTTTCCAATTTAATGAT

CTATCAGGAGTTCTATAATAAGAATGTTTCTAACATTGTTTTCAGATGCATTCTGATATAGGAGTTCAAT

ATGTAATCGTTCTTTTGTAATTTTCCCATTGCAAAAAATACTTATATTATCACAAATT

>Copernicia_berteroana_HQ720501.1

GCTACTCCTTTTACTGATGTCACAGTAAGTAAATACATAAAACCTTCATGCAAGCTTTACCTGAGAAGCA

TATCATAGTTTAACCTGCTATAAATGCTTGAGCTTTTTTGTTTATTCAGTTTAAGAATTCTGAGTCACTG

TATCCGATGTTGTTGAACATTAAGGAAAGTTTCTTTTGTTCTGCTCCAGGAGGAATTAATTTTTGCATAT

GTCTAGGTAAAAGACATTGTTGATAGCTTAGACAGAAGGTTATTCAATTTGCATTACCATTAAGGATTTG

TATTAGATATGGTTTGGTTAATTATAATCCTTTCAACTTCATACATGGATTTGATTTTCTTTTGAGGGGA

GCTTTTGCCTTTGGAGTTCATACTTTCCTCTGGTTTAGTTAATTATACTCCTGTTATTTGAACTCTACTT

TGAATGTCTCTTTTTGAAGTTCTTGAGATGTAAGTTTACCCTGTAGACAGAAACGTGGCTTTTCAGAATG

ACTTTGGAYGAATGTCTTGCAAGTTGTTATGATGATCCTAAACTTGTATAAGTTTCAGACATACTATTTT

GACTAAGGTCTCTTCTAAATTATATAGTGGACTATCTTCTTGTAACTGGCCTATGACTTGGATTTTCCAA

TTTAACGATCTATCAGGAGTTCTATAATAAGAATGTTTCTAACATTGTTTTCAGATGCATTCTGATATAG

GAGTTCAATATGTAATCGTTCTTTTGTAATTTTCCCATTGCAAAMAATACTTATATTATCACAAATT

>Copernicia_alba_HQ720499.1

GAAAGGCTGCTGCCCATATGGGAAAGGAGGGAGATGCTACTCCTTTTACTGATGTCACAGTAAGTAAATA

CATAAAACCTTCATGCAAGCTTTACCTGAGAAGCATATCATAGTTTAACCTGCTATAAATGCTTGAGCTT

TCTTGTTTATTCAGTTTAAGAATTCTGAGTCACTGTATCCGATGTTGTTGAACATTAAGGAAAGTTTCTT

TTGTTCTGCTCCAGGAGGAATTAATTTTTGCATATGTCTAGGTAAAAGACATTGTTGATAGCTTGGACAT

AAGGTTATTCAATTTGCATTACCATTAAGGATTTGTATTAGATATGGTTTGGTTGATTATAATCCTTTCA

ACTTCATACGTGGATTTGATTTTCTTTTGAGGGGAGCTTTTGCCTTTGGAGTTCATACTTTCCTCTGGTT

TAGTTAATTATACTCCTGTTATTTGAACTCTACTTTGAATGTCTCTTTTTGAAGTTCTTGAGATGTAAGT

TTACCCTGTAGACAGAAACGTGGCTTTTCAGAATGACTTTGGACGAATGTCTTGCAAGTTGTTATGATGA

TCCTAAACTTGTATAAGTTTCAGACATACTATTTTGACTGAGGTCTCTTCTAAATTATATAGTGGACTAT

CTTCTTGTAACTGGCCTATGACTTGGATTTTCCAATTTAATGATCTATCAGGAGTTCTATAGTAAGAATG

CTTCTAACATTGTTTTCAGACGCATTCTGATATAGGAGTTCAATATGTAATCGTTATTTTGTAATTTTC

>Copernicia_tectorum_HQ720512.1

GGAGATGCTACTCCTTTTACTGATGTCACAGTAAGTAAATACATAAAACCTTCATGCAAGCTTTACCTGA

GAAGCATATCATAGTTTAACCTGCTATAAATGCTTGAGCTTTCTTGTTTATTCAGTTTAAGAATTCTGAG

TCACTGTATCCGATGTTGTTGAACATTAAGGAAAGTTTCTTTTGTTCTGCTCCAGGAGAAATTAATTTTT

GCATATGTCTAGGTAAAAGACATTGTTGATAGCTTGGATATAAGGTTATTCAATTTGCATTACCATTAAG

GATTTGTATTAGATATGGTTTGGTTGATTATAATCCTTTCAACTTCATACGTGGATTTGATTTTCTTTTG

AGGGGAGCTTTTGCCTTTGGAGTTCATACTTTCCTCTGGTTTAGTTAATTATACTCCTGTTATTTGAACT

CTACTTTGAATGTCTCTTTTTGAAGTTCTTGAGATGTAAGTTTACCCTGTAGACAGAAACGTGGGGCTTT

TCAGAATGACTTTGGACGAATGTCTTGCAAGTTGTTATGATGATCCCAAACTTGTATAAGTTTCAGACAT

CCTATTTTGACTGAGGTCTCTTCTAAATTATATAGTGGACTATCTTCTTGTAACTGGGCTATGACTTGGA

TTTCCAATTTAATGATCTATCAGGAGTTCTATAATAAGAATGTTTCTAACATTGTTTTCAGACGCATTCT

GATATAGGAGTTCAATATGTAATTGTTCTTTTGTAATTTTCCCATTGCAAAAAATACTTATATTATCACA

AATT

>Copernicia_prunifera_HQ720510.1

AGGGAGATGCTACTCCTTTTACTGATGTCACAGTAAGTAAATACATAAAACCTTCATGCAAGCTTTACCT

GAGAAGCATATCATAGTTTAACCTGCTATAAATGCTTGAGCTTTCTTGTTTATTCAGTTTAAGAATTCTG

AGTCACTGTATCCGATGTTGTTGAACATTAAGGAAAGTTTCTTTTGTTCTGCTCCAGGAGGAATTAATTT

TTGCATATGTCTAGGTAAAAGACATCATTGATAGCTTGGACATAAGGTTATTCAATTTGCATTACCATTA

AGGATTTGTATTTGATATGGTTTGGTTGATTATAATCCTTTCAACTTCATACGTGGATTTGATTTTCTTT

TGAGGGGAGCTTTTGCCTTTGGAGTTCATACTTTCCTCTGGTTTAGTTAATTATACTCCTGTTATTTGAA

CTCTACTTTGAATGTCTCTTTTTGAAGTTCTTGAGATGTAAGTTTACCCTGTAGACAGAAACGTGGCTTT

TCAGAATGACTTTGGACGAATGTCTTGCAAGTTGTTATGATGATCCCAAACTTGTATAAGTTTCAGACAT

CCTATTTTGACTGAGGTCTCTTCTAAATTATATAGTGGACTATCTTCTTGTGACTGGCCTATGACTTGGA

TTTTCCAATTTAATGATCTATCAGGAGTTCTATAATAAGAATGTTTCTAACATTGTTTTCAGACGCATTC

TGATATAGGAGTTCAATATGTAATCATTCTTTTGTAATTTTCCCATTGCAAAAAGTACTTATATTATCAC

AAATTTCCTAGGTG

>Copernicia_macroglossa_HQ720508.1

CCATGCAAGCTTTACCTGAGAAGCATATCATAGTTTAACCTGCTATAAATGCTTGAGCTTTTTTGTTTAT

TCAGTTTAAGAATTCTGAGTCACTGTATCCGATGTTGTTGAACATTAAGGAAAGTTTCTTTTGTTCTGCT

CCAGGAGGAATTAATTTTTGCATATGTCTAGGTAAAAGACATTGTTGATAGCTTAGACAGAAGGTTATTC

AATTTGCATTATCATTAAGGATTTGTATTAGATATGGTTTGGTTAATTATAATCCTTTCAACTTCATACA

TGGATTTGATTTTCTTTTGAGGGGAGCTTTTGCCTTTGGAGTTCATACTTTCCTCTGGTTTAGTTAATTA

TACTCCTGTTATTTGAACTCTACTTTGAATGTCTCTTTTTGAAGTTCTTGAGATGTAAGTTTACCCTGTA

GACAGAAACGTGGCTTTTCAGAATGACTTTGGACGAATGTCTTGCAAGTTGTTATGATGATCCTAAACTT

GTATAAGTTTCAGACATACTATTTTGACTAAGGTCTCTTCTAAATTATATAGTGGACTATCTTCTTGTAA

CTGGCCTATGGCTTGGATTTTCCAATTTAATGATCTATCAGGAGTTCTATAATAAGAATGTTTCTAACAT

TGTTTTCAGATGCATTCTGATATAGGAGTTCAATATGTAATC

>Copernicia_hospita_HQ720506.1

AAATACATAAAACCTTCATGCAAGCTTTACCTGAGAAGCATATCATAGTTTAACCTGCTATAAATGCTTG

AGCTTTTTTGTTTATTCAGTTTAAGAATTCTGAGTCACTGTATCCGATGTTGTTGAACATTAAGGAAAGT

TTCTTTTGTTCTGCTCCAGGAGGAATTAATTTTTGCATATGTCTAGGTAAAAGACATTGTTGATAGCTTA

GACAGAAGGTTATTCAATTTGCATTACCATTAAGGATTTGTATTAGATATGGTTTGGTTAATTATAATCC

TTTCAACTTCATACATGGATTTGATTTTCTTTTGAGGGGAGCTTTTGCCTTTGGAGTTCATACTTTCCTC

TGGTTTAGTTAATTATACTCCTGTTATTTGAACTCTACTTTGAATGTCTCTTTYTGAAGTTCTTGAGATG

TAAGTTTACCCTGTAGACAGAAACGTGGCTTTTCAGAATGWCTTTGGACGAATGTCTTGCAAGTTGTWWT

GATGATCCTAAACTTGTATAAGTKTCAGACATACTATTTTGACTRAGGTCTCTTCTAAATTATATAGTGG

ACTATSKTCTTGTAACTGGCCTATGACTTGGATTTTCCAATTTAATGATCTATCAGGAGTTCTATAATAA

GAATGTTTCTAACATTGTTTTCAGAYGCATTCTGATATAGGAGTTCAATATGTAATCGTTYTTTTGTAAT

TTTCCC

>Copernicia_fallaensis_HQ720504.1

TAGTTTAACCTGCTATAAATGCTTGAGCTTTTTTGTTTATTCAGTTTAAGAATTCTGAGTCACTGTATCC

GATGTTGTTGAACATTAAGGAAAGTTTCTTTTGTTCTGCTCCAGGAGGAMTTAATTTTTGCATATGTCTA

GGTAAAAGACATTGTTGATAGCTTAGACAGAAGGTTATTCAATTTGCATTACCATTAAGGATTTGTATTA

GATATGGTTTGGTTAATTATAATCCTTTCAACTTCATACATGGATTTGATTTTCTTTTGAGGGGAGCTTT

TGCCTTTGGAGTTCATACTTTCCTCTGGTTTAGTTAATTATACTCCTGTTATTTGAACTCTACTTTGAAT

GTCTCTTTTTGAAGTTCTTGAGATGTAAGTTTACCCTGTAGACAGAAACGTGGCTTTTCAGAATGACTTT

GGACGAATGTCTTGCAAGTTGTTATGATGATCCTAAACTTGTATAAGTTTCAGACATAYTATTTTGACTA

AGGTCTCTTCTAAATTATATAGTGGACTATCTTCTTGTAACTGGCCTATGACTTGGATTTTCC

>Copernicia_curtissii_HQ720502.1

CTCCTTTTACTGATGTCACAGTAAGTAAATACATAAAACCTTCATGCAAGCTTTACCTGAGAAGCATATC

ATAGTTTAACCTGCTATAAATGCTTGAGCTTTTTTGTTTATTCAGTTTAAGAATTCTGAGTCACTGTATC

CGATGTTGTTGAACATTAAGGAAAGTTTCTTTTGTTCTGCTCCAGGAGGAATTAATTTTTGCATATGTCT

AGGTAAAAGACATTGTTGATAGCTTAGACAGAAGGTTATTCAATTTGCATTACCATTAAGGATTTGTATT

AGATATGGTTTGGTTAATTATAATCCTTTCAACTTCATACATGGATTTGATTTTCTTTTGAGGGGAGCTT

TTGCCTTTGGAGTTCATACTTTCCTCTGGTTTAGTTAATTATACTCCTGTTATTTGAACTCTACTTTGAA

TGTCTCTTTTTGAAGTTCTTGAGATGTAAGTTTACCCTGTAGACAGAAACGTGGCTTTTCAGAATGACTT

TGGACGAATGTCTTGCAAGTTGTTATGATGATCCTAAACTTGTATAAGTTTCAGACATACTATTGTGACT

AAGGTCTCTTCTAAATTATATAGTGGACTATCTTCTTGTAACYGGCCTATGACTTGGATTTTCCAATTTA

ATGATCTATCAGGAGTTATATAATAAGAATGTTTCTAACATTGTTTTCAGATGCATTCTGATATAGGAGY

TCAATATGTAATCGTTCTTTTGTAATTTTCCCATTGCAAAAAATACTTATATTATCACAAATT

>Copernicia_baileyana_HQ720500.1

TATGGGAAAGGAGGGAGACGCTACTCCTTTTACTGATGTCACAGTAAGTAAATACATAAAACTTTCATGC

AAGCTTTACCTGAGAAGCATATCATAGTTTAACCTGCTATAAATGCTTGAGCTTTTTTGTTTATTCAGTT

TAAGAATTCTGAGTCACTGTATCCGATGTTGTTGAACATTAAGGAAAGTTTCTTTTGTTCTGCTCCAGGA

GGACTTAATTTTTGCATATGTCTAGGTAAAAGACATTGTTGATAGCTTAGACAGAAGGTTATTCAATTTG

CATTACCATTAAGGATTTGTATTAGATATGGTTTGGTTAATTATAATCCTTTCAACTTCATACATGGATT

TGATTTTCTTTTGAGGGGAGCTTTTGCCTTTGGAGTTCATACTTTCCTCTGGTTTAGTTAATTATACTCC

TGTTATTTGAACTCTACTTTGAATGTCTCTTTTTGAAGTTCTTGAGATGTAAGTTTACCCTGTAGACAGA

AACGTGGCTTTTCAGAATGACTTTGGACGAATGTCTTGCAAGTTGTTATGATGATCCTAAACTTGTATAA

GTTTCAGACATACTATTTTGACTAAGGTCTCTTCTAAATTATATAGTGGGCTATCTTCTTGTAACTGGCC

TATGGCTTGGGTTTTCCAATTTTATGATCTATCAGGAGTTCTATAATAAGAATGTTTCTTACATTGTTTT

CAGATGCATTCTGATATAGGAGTTCAATATTGTATCGTTCTTTTGTAATTTTCCCATTGCAAAAAATACT

TATATTATCACAATTTTCCT

>Copernicia_prunifera_EU215513.1

TTATTGAGTGCATCATGGGAAAGGTTGCTGCCCATATGGGAAAGGAGGGAGACGCTACTCCTTTTACTGA

TGTCACAGTAAGTAAATACATAAAACCTTCATGCAAGCTTTACCTGAGAAGCATATCATAGTTTATCCTG

CTATAAATGCTTGAGCTTTTTTGTTTATTCAGTTTAAGAATTCTGAGTCACTGTATCCGATGTTGTTGAA

CATTAAGGAAAGTTTCTTTTGTTCTGCTCCAGGAGGAATTAATTTTTGCATATGTCTAGGTAAAAGACAT

TGTTGATAGCTTAGACAGAAGGTTATTCAATTTGCATTACCATTAAGGGTTTGTATTAGATATGGTTTGG

TTAATTATAATCCTTTCAACTTCATACATGGATTTGATTTTCTTTTGAGGGGAGCTTTTGCCTTTGGAGT

TCATACTTTCCTCTGGTTTAGTTAATTATACTCCTGTTATTTGAACTCTACTTTGAATGTCTCTTTTGAA

GTTCTTGAGATGTAAGTTTACCCTGTAGACAGAAACGTGGCTTTTCAGAATGACTTTGGACGAATGTCTT

GCAAGTTGTATTGATGATCCTAAACTTGTATAAGTTTCAGACATACTATTTTGACTAAGGTCTCTTCTAA

ATTATATAGTGGACTATCTTCTTGTAACTGGCCTATGACTTGGATTTTCCAATTTAATGATCTATCAGGA

GTTCTATAATAAGAATGTTTCTAACATTGTTTTCAGATGCATTCTGATATAGGAGTTCAATATGTAATCG

TTCTTTTGTAATTTTCCCATTGCAAAAAATACTTATATTATCACAAATTTCCTAGGTGGACAATATCAGC

AAAGCTCTTCATAAGTGTGGATATCAGA

>Cryosophila_stauracantha_EU215492.1

TTATTGAGTGCATCATGGGAAAGGTTGCTGCTCATATGGGAAAAGAGGGAGATGCTACTCCTTTTACTGA

TGCCACAGTAAGTAAATCCGTAAAAACTTTATGCGAGCTTTACCTGTGAAGCATGTCGCAGTTTAACTTG

CTATAGATGCTGGAGCTTGTTTGCTTATTCAGTTTAAGAATTCCGAGGCACTGTCTGATGTTAAAGAACA

TTAACGAAAGTTTCTTTTGTTCTGCTCGAGGAGGAAGTAGTTTTTGTATGCGTCTAGCTAAAAGACATCG

TTGATAGCTTACACAAAACAGAGGGTTATTCAATTTGCATTACCATTAAGGATTTGTACTAGATATGGTT

CAATAAATATGTTTGAGAACTGGAGCTTTTGCGTTTAGTGTTCATTTTTTTCTCTGGTTTACTTAATTCT

AATCCTGTTATTTGAGTCTACTTTGAATATCGCTTTTGAAGTTCTTGAGATGTTAGTTTACCCTGTAGAC

AGAAACGTT

>Desmoncus_orthacanthos_EF491156.1

CTACTCCTTTTACTGATGTCACAGTAAGTAAATCCATGGAACCTTCGTGCAAGCTTTACCTGAGAAGCAT

GTCTCAATTTAACTTGCTAATTGCTATAAATGCTGGAGTTTTTCTGCTTATTCAGTTTAAGAATTCCAAG

GCACTGTATCTGATGTTAAACAACATTATGTAAAGTTTCTTTTGTTCTGCTCGAGGAGGAAGTATTTTTT

TCATGTGTCTAGCTAAAAGACATTGTTGATAGCTCGTACCAAAACACATAGTTATTCAATTTGCATTACC

ATTAAGGACTTCTACTAATATGGTTTCATAAATATGATTGAAACTGTTACCCTTTGCCTTTGGAGTTCAT

ATTTTCCTCTGGATTAGTTAATTATAATTCAGTTATTTGAAGTCTACTTTGAATCTCTCTTTTGAAGTCC

TTAATATGTAGGTTTACCCTGTAGATAGAAACGTGGGTCTGAACTTTGTGGAACGAACTTGGATGAGCAT

CTTGTTTGTTGTTCTGATGACCATAAACTTGTATAAGTTTCAAGGCATGCTATTTTGACTAAGGTCTTTC

CTAAATTATATCATTGACTATCTCCTTGTAACTGACCTGTGACTTGGTTTTTCCAATTCAATGATCTATC

AGGAGTTCTATCCTAAATAATGTTTCTAACATTCATTTCAGATATTTTCTGATGTAGGAGTTTAATATGT

AATGTACTTTTGTAGTTGCCCCAT

>Desmoncus_orthacanthos_HQ265659.1

GCATCATGGGAAAAGTTGCTGCCCATATGGGAAAGGAGGGAGATGCTACTCCTTTTACTGATGTCACAGT

AAGTAAATCCATGGAACCTTCGTGCAAGCTTTACCTGAGAAGCATGTCTCAATTTAACTTGCTAATTGCT

ATAAATGCTGGAGTTTTTCTGCTTATTCAGTTTAAGAATTCCAAGGCACTGTATCTGATGTTAAACAACA

TTATGTAAAGTTTCTTTTGTTCTGCTCGAGGAGGAAGTATTTTTTTCATGTGTCTAGCTAAAAGACATTG

TTGATAGCTCGTACAAAAACACATAGTTATTCAATTTGCATTACCATTAAGGACTTCTACTAATATGGTT

TCATAAATAGGATTGAAACTGTTACCCTTTGCCTTTGGAGTTCATATTTTCCTCTGGATTAGTTAATTAT

AATTCGGTTATTTGAAGTCTACTTTGAATCTCTCTTTTGAAGTCCTTAATATGTAGGTTTACCCTGTAGA

TAGAAACGTGGGTCTGAACTTTGTGGAACGACCTTGGATGAGCATCTTGTTTGTTGTTCTGATGACCATA

AACTTGTATCAGTTTCAAGGCATGCTATTTTGACTAAGGTCTTCCTAAATTATATCATTGACTATCTCCT

TGTAACTGACCTGTGACTTGGTTTTTCCAATTCAATGATCTATCAGGAGTTCTATCCTAAGTAATGTTTC

TAACATTGATTTCAGATATTTTCTGATGTAGGAGTTTAATATGTAATGTACTTTTGTAGTTGCCCCATTA

CTGAAAATGCTTATATTATCACAAATTTCGTAGGTGGACAAATCATCAGCAAAGCTCTTCATAAGTGTGG

A

>Desmoncus_cirrhifer_HQ265657.1

ATTCGCCCTTCNACTTATTGAGTGCATCATGGGAAAAGTTGCTGCCCATATGGGAAAGGAGGGAGATGCT

ACTCCTTTTACTGATGTCACAGTAAGTAAATCCATGGAACCTTCGTGCAAGCTTTACCTGAGAAGCATGT

CTCAATTTAACTTGCTAATTGCTATAAATGCTGGAGTTTTTCTGCTTATTCAGTTTTAGAATTCCAAGGT

ACTGTATCTGATGTTAAACAACATTATGTAAAGTTTCTTTTGTTCTGCTCGAGGAGGAAGTATTTTTTTC

ATGTGTCTAGCTAAAAGACATTGTTGATAGCTCATACAAAAACACATAGTTATTCAATTTGCATTACCAT

TAAGGACTTCTACTAATATGGTTTCATAAATATGATTGAAACTGTTACCCTTTGCCTTTGGAGTTCATAT

TTTCCTCTGGATTAGTTAATTATAATTCGGTTATTTGAAGTCTACTTTGAATCTCTCTTTGGAAGTCCTT

AATATGCAGGTTTACCCTGTAGATAGAAACGTGGGTCTGAACTTTGTGGAACGACCTTGGATGAGCATCT

TGTTTGTTGTTCTGATGACCATAAACTTGTATAAGTTTCAAGGCATGCTATTTTGACTAAGGTCTTTCCT

AAATTATATCATTGACTATCTCCTTGTAACTGACCTGTGACTTGGTTTTTCCAATTCAATGATCTATCAG

GAGTTCTATCCTAAATAATGTTTCTAACATTGATTTCAGATATTTTCTGATGTAGGAGTTTAATATGTAA

TGTACTTTTGTAGTTGCCCCATTACTGAAAATGCTTATATTATCACAAATTTCCTAGGTGGACAAGATCA

GCAAAGCTCTTCATAAGCAGGAGTTCTATCCTAAATAATGTTCTAACATTGATTTCAGATATTTTCTGAT

GTAGGAGTTTAATATGTAATGTACTTTTGTAGTTGCCCCATTACTGAAAATGCTTATATTATCACAAATT

TCCTAGGTGGACAATATCAGCAAAGCTCTTCATAAGTGTGGATATCAGATGCGTGGAAGGGCGAATCG

>Desmoncus_polyacanthos_HQ265660.1

TGCATCATGGGAAAAGTTGCTGCCCATATGGGAAAGGAGGGAGATGCTACTCCTTTTACTGATGTCACAG

TAAGTAAATCCATGGAACCTTCATGCAAGCTTTACCTGAGAAGCATGTCTCAATTTAACTTGCTAATTGC

TATAAATGCTGGAGTTTTTCTGCTTATTCAGTTTAAGAATTCCAAGGCACTGTATCTGATGTTAAACAAC

ATTATGTAAAGTTTCTTTTGTTCTGCTCGAGGAGGAAGTATTTTTTTCATGTGTCTAGCTAAAAGACATT

GTTGATAGCTCGTACAAAAACACATAGTTATTCAATTTGCATTACCATTAAGGACTTTTACTAATATGGT

TTCATAAATATGATTGAAACTGTTACCCTTTGCCTTTGGAGTTCATATTTTCCTCTGGATTAGTTAATTA

TAATTCGGTTATTTGAAGTCTACTTTGAATCTCTCTTTTGAAGTCCTTAGTATGTAGGTTTACCCTGTAG

ATAGAAACGTGGGTCTGAACTTTGTGGAACGACCTTGGATGAGCATCTTGTTTGTTGTTCTGATGACCAT

AAACTTGTATAAGTTTCAAGGCATGCTATTTTGACTAAGGTCTTCCTAAATTATATCATTGACTATCTCC

TTGTAACTGACCTGTGACTTGGTTTTTCCAATTCAATGATCTATCAGGGATTCTATCCTAAATAATGTTT

CTAACATTGATTTCAGATATTTTCTGATGTAGGAGTTTAACTATNTAGATGCACNNNAGTAGCNGCCCCA

NNACTGAAAACGCTTATANTATCACAAAT

>Desmoncus_mitis_HQ265658.1

GGGANNGGAGGNAGANGCTACTCCTTTTACTGATGTCACAGTAAGTAAATCCATGGAACCTCCATGCAAG

CTTTACCTGAGAAGCATGTCTCAATTTAACTTGCTAATTGCTATAAATGCTGGAGTTTTTCTGCTTATTC

AGTTTAAGAATTCCAAGGCACTGTATCTGATGTTAAACAACATTATGTAAAGTTTCTTGTGTTCTGCTCG

AGGAGGAAGTATTTTTTTCATGTGTCTAGCTAAAAGACATTGTTGATAGCTCGTACAAAAACACATAGTT

ATTCAATTTGCATTACCATTAAGGACTTCTACTAATATGGTTTCATAAATATGATCGAAACTGTTACCCT

TTGCCTTTGGAGTTCATATTTTCCTCTGGATTAGTTAATTATAATTCGGTTATTTGAAGTCTACTTTGAA

TCTCTCTTTTGAAGTCCTTAGTATGTAGGTTTACCCTGTAGATAGAAACGTGGGTCTGAACTTTGTGGAA

CGACCTTGGATGAGCATCTTGTTTGTTGTTCTGATGACCATAAACTTGTATAAGTTTCAAGGCATGCTAT

TTTGACTAAGGTCTTCCTAAATTATATCATTGACTATCTCCTTGTAACTGACCTGTGACTTGGTTTTTCC

AATTCAATGATCTATCAGGAATTCTATCCTAAATAATGTTTCTAACATTGATTTCAGATATTTTCTGATG

TAGGAGTTTAATATGTAATGTACTTTTGTAGTTGCCCCATTACTGAAAATGCTTATATTATCACAAATTC

CAGGTGACA

>Elaeis_oleifera_AJ830163.1

TGGGAAAGGAGGGAGATGCTACTCCTTTTACTGATGTCACAGTAAGTAAATCCATAGAACCTTCATGCAA

GCTTTACCTGAGAAGCATGTCACAATTTAACTTGCTAGAAATGCTGGAGCTTTTTTGCTTATTCAGTTTA

AGAATTCCAAGGTACTGTATCTGATGTTAAAGAACATTATGTAAAGTTTCTTCTGTTCTGCTCGAGGAGG

AAGTATTTTTTCCGTATGTCTAGCTAAAAGACGTTGTTGATAGCTCGTACATAAACACATGGTTATTCAA

TTTGCATTACCATTAAGGATTTGTACTAATATGGTTTAATAAATATGATGGAGAACTGTTATCCTTTGCC

TTTGGAGTTCATATTTTCCTCTGGATTAGTTAATTATAATTCGGTTATTTGAAGTCTACTTTGATTCTCT

CTTTTGAAGTTCTTGATATGTAAATTTACCCTGTAGACAGAAACGTGGCTCTGAAGAATGACCTTGGACG

AGTGTCTTGCATGTTATTCTGATGATCCTAAACTTGTATCAGTTTCAAGATGTGACTAAGGTCTCTCCTA

AATTATATCATTGACTATCTCCTTGTAACTGACCTGTGACTTAGTTTTTCCAATTCAATGATCTATCAGG

AGTTCTATCCTAAATAATGTTTCTAACATTGATTTCAGATATTTTCTGATGTAGGAGTTTAATATGTAAT

GTACTTTTGTAGTTGCCTCATTACAAAAAAAATGCTTATATTATCACAAATTTCCTAGGTGGACAATATC

AGC

>Elaeis_oleifera_HQ265662.1

AGATGCTNCTCCTTTTACTGATGTCACAGTAAGGTAAATCCATAGAACCTTCATGCAAGCTTTACCTGAG

AAGCATGTCACAATTTAACTTGCTAGAAATGCTGGAGCTTTTTTGCTTATTCAGTTTAAGAATTCCAAGG

TACTGTATCTGATGTTAAAGAACATTATGTAAAGTTTCTTCTGTTCTGCTCGAGGAGGAAGTATTTTTTC

CGTATGTCTAGCTAAAAGACGTTGTTGATAGCTCGTACATAAACACATGGTTATTCAATTTGCATTACCA

TTAAGGATTTGTACTAATATGGTTTAATAAATATGATGGAGAACTGTTATCCTTTGCCTTTGGAGTTCAT

ATTTTCCTCTGGATTAGTTAATTATAATTCGGTTATTTGAAGTCTACTTTGATTCTCTCTTTTGAAGTTC

TTGATATGTAAATTTACCCTGTAGACAGAAACGTGGCTCTGAAGAATGACCTTGGACGAGTGTCTTGCAT

GTTATTCTGATGATCCTAAACTTGTATCAGTTTCAAGATGTGACTAAGGTCTCTCCTAAATTATATCATT

GACTATCTCCTTGTAACTGACCTGTGACTTAGTTTTTCCAATTCAATGATCTATCAGGAGTTCTATCCTA

AATAATGTTTCTAACATTGATTTCAGATATTTTCTGATGTAGGAGTTTAATATGTAATGTACTTTTGTAG

TTGCCTCATTACAAAAAAAAATGCTTATATTATCACAAATTTCCTAGG

>Elaeis_guineensis_HQ265661.1

AATCCCATAGACCCTTCATGCAAGCTTTTCCCNGAGAAGCATGNCCACAATTTAACTTGCTAGAAATGCN

GGAGCTTTTTTGGCTTATTCAGTTTAAGAATTCCAAGGTACTGTATCTGATGTTAAAGAACATTATGTAA

AGTTTCTTTTGTTCTGCTTGAGGAGGAAGTATTTTTTCCATGTGTCTAGCTAAAAGACATTGTTGATAGC

TCRTACAWWAACACATGGTTATTCAATTTGCATTTCCATTAAGGATTTSTACTRATATGGTTTAATAAAT

ATGATKGARAACTGTTAYCCTTTGCCTTTGGAGTTCATATTTTCCTCTGGATYAGTTAATTATAATTTGG

TTATTTGAAGTCTACTTTGATTCTCTCTTTTGAAGTTCTTGATATGTAAATTTACCCTGTAGAGAGAAAC

GTGGCTCTGAAGAATGACCTTGGACGAGTGTCTTGTATGTTATTCTGATGATCCTAAACTTGAATAAGTT

TCAAGACATACTATTGTGACGAAGGTCTCTCCTAAATTATATCATTGACTATCTCCTTGTAACTGACCTG

TGACTTAGTTTTTTTCCAATTCCATGATCTATCAGGAGTTCTATCCTAAATAATGTTTCTAACATTGATT

TCAGATATTTTTCTGATGTAGAAGTTTAATATGTAATGTACTTTTTGTAGTTTGCCTCATTACAAAAAAA

ATGCTTATATTATCACAAATTT

>Gaussia_maya_DQ177757.1

CTACTCCTTTTACTGATGTCACAGTAAGTAAATCCATAGAACCTTCTTGCAAGCATTACCTGAGAAGCCT

GTCGCAATTTAACTTGCTATGCTGGAACTTTTTTGCTCATTCAATTTAAGAATTCTGAGGCACTGTATCT

GATGTTAAAGAAGATTAAGGAAAGTTTCGTTTGTGCTGCTCCAGGAGGAAGTAATTTTGCATATGTCTAG

CTAAAAGACATTGATAGCTTATACAAAAACACATGGTTATTCAATTTGCATTACTGTTAAGGATTTGTAC

TAGATATGGTTTAATAAATATGTTTGAGAACTGTTACCCCCCCCCCCCCCAACCACCACCACCCCCCTTC

CCTCTCTCTCCCTGTCTAGAACCTTTCAACTTCTTACCTGGATTTGATTTTTTTTGAGGGGAGTTTTTGC

CTTTCAAGTTCATATTTTTCCTCTGGATTAGTTAATTATAATCCTATTATTTGTAGTCTGCTTTGAATAT

CTCTTTTGAAGTTCTTGCTGTGTAAGCTTACCCTGTAGACAGAAACATGGCTTTGAAGAATGACCTTGGA

CGAGTGTCTTGTAAGTTATTCTGATGATCCTAAACTTGTATAAGTTTCAAGACATTACTATTTTGACTAA

GGTCTCTCCTAAATCATATAGTAGACAATCTCCTTGTAATTGACCTTTGACTTGGATTTTCCAATTCAAT

GATCTATCAGGAGTTCTATCCTAAATAATGTTTCTAACATTGATTTCAGATATATTCTGATGTAGTTGTT

TAATATGTAATTGTACTTTTGTAATTGCCCCATTA

>Gaussia_attenuata_EF491138.1

CTACTCCTTTTACTGATGTTACAGTAAGTAAATCCATAGAACCTTCTTGCAAGCATTACCTGAGAAGCCT

GTCGCAATTTAACTTGCTATGCTGGAACTTTTTTGCTCATTCAATTTAAGAATTCTGAGGCACTGTATCT

GATGTTAAAGAAGATTAAGGAAAGTTTCGTTTGTGCTGCTCCAGGAGGAAGAAATTTTGCATATGTCTAG

CTAAAAGACGTTGATAGCTTATACAAAAACACTTGGTTATTCAATTTGCATTACTGTTAAGGATTTGTAC

TAGATATGGTTTGATAAACATGTTTGAGAACTGTTAAACCCCACCCACCCCCACCCCCCTTCCCTCTCTC

TCTCTGTCTAGAACCTTTCAACTTCTTACCTGGATTTGATTTTTTTGAGGGGAGTTTTTGCCTTTCAAGT

TCATATTTTTCCTCTGGATTAGTTAATTATAATCCTATTATTTGTAGTCTGCTTTGAATATCTCTTTTGG

AGTTCTTGCTGTGTAAGTTTACCCTGTAGACAGAAACATGGCTTTGAAGAATGACCTTGGACGAGTGTCT

TGTAAGTTATTCTGATGATCCTAAACTCGTATGAGTTTCAAGACATTACTATTTTGACTAAGGTCTCTCC

TAAATCATATAGTAGACAATCTCCTTGTAATTGACCTTTGACTTGGATTTTCCAATTCAATGATCTATCA

GGAGTTCTATCCTAAATAATGTTTCTAACATTGATTTCAGATATATTCTGATGTAGGAGTTTAATATGTA

ATTGTACTTTTGTAATCGCCCCAT

>Gaussia_maya_EF491140.1

CTACTCCTTTTACTGATGTCACAGTAAGTAAATCCATAGAACCTTCTTGCAAGCATTACCTGAGAAGCCT

GTCGCAATTTAACTTGCTATGCTGGAACTTTTTTGCTCATTCAATTTAAGAATTCTGAGGCACTGTATCT

GATGTTAAAGAAGATTAAGGAAAGTTTCGTTTGTGCTGCTCCAGGAGGAAGTAATTTTGCATATGTCTAG

CTAAAAGACATTGATAGCTTATACAAAAACACATGGTTATTCAATTTGCATTACTGTTAAGGATTTGTAC

TAGATATGGTTTAATAAATATGTTTGAGAACTGTTACCCCCCCCCCCCCCAACCACCACCACCCCCCTTC

CCTCTCTCTCCCTGTCTAGAACCTTTCAACTTCTTACCTGGATTTGATTTTTTTTGAGGGGAGTTTTTGC

CTTTCAAGTTCATATTTTTCCTCTGGATTAGTTAATTATAATCCTATTATTTGTAGTCTGCTTTGAATAT

CTCTTTTGAAGTTCTTGCTGTGTAAGCTTACCCTGTAGACAGAAACATGGCTTTGAAGAATGACCTTGGA

CGAGTGTCTTGTAAGTTATTCTGATGATCCTAAACTTGTATAAGTTTCAAGACATTACTATTTTGACTAA

GGTCTCTCCTAAATCATATAGTAGACAATCTCCTTGTAATTGACCTTTGACTTGGATTTTCCAATTCAAT

GATCTATCAGGAGTTCTATCCTAAATAATGTTTCTAACATTGATTTCAGATATATTCTGATGTAGTTGTT

TAATATGTAATTGTACTTTTGTAATTGCCCCAT

>Gaussia_spirituana_EF491142.1

AAGTAAATCCATAGAACCTTCTTGCAAGCATTACCTGAGAAGCCTGTCGCAATTTATCTTGCTATGCTGG

AACTTTTTTGCTCATTCAATTTAAGAATTCTGAGGCACTGTATCTGATGTTAAAGAAGATTAAGGAAAGT

TTCGTTTGNGCTGCTCCAGGAGGAAGAAATTTTGCATATGTCTAGCTAAAAGACGTTGATAGCTTATACA

AAAACACTTGGTTATTCAATTTGCATTACTGTTAAGGATTTGTACTAGATATGGTTTGATAAACATGTTT

GAGAACTGTTAAACCCCCACCACCCCCACCCCCCTTCCCTCTCTCTCTGTGTCTAGAACCTTTCAACTTC

TTACCTGCATTTGATTTTTTTGAGGGGAGTTTTGCCTTTCAAGTTCATATTTTTCCTCTGGATTAGTTAA

TTATAATCCTATTATTTGTAGTCTGCTTTGAATATCTCTTTTGGAGTTCTTGCTGTGTAAGTTTACCCTG

TAGACAGAAACATGGCTTTGAAGAATGACCTTGGACGAGTGTCTTGTAAGTTATTCTGATGATCCTAAAC

TCGTATGAGTTTCAAGACATTACTATTTTGACTAAGGTCTCTCCTAAATCATATAGTAGACAATCTCCTT

GTAATTGACCTTTGACTTGGATTTTCCAATTCAATGATCTATCAGGAGTTCTATCCTAAATAATGTTTCT

AACATTGATTTCAGATATATTCTGATGTAGGAGTTTAATATGTAATTGTACTTTTGTAATCGCCCCAT

>Gaussia_gomez_pompae_EF491139.1

CTACTCCTTTTACTGATGTCACAGTAAGTAAATCCATAGAACCTTCTTGCAAGCATTACCTGAGAAGCCT

GTCGCAATTTAACTTGCTATGCTGGAACTTTTTTGCTCATTCAATTTAAGAATTCTGAGGCACTGTATCT

GATGTTAAAGAAGATTAAGGAAAGTTTCGTTTGTGCTGCTCCAGGAGGAAGTAATTTTGCATATGTCTAG

CTAAAAGACATTGATAGCTTATACAAAAACACATGGTTATTCAATTTGCATTACTGTTAAGGATTTGTAC

TAGATATGGTTTAATAAATATGTTTGAGAACTGTTACCCCCCCCCCCCCAACCACCACCACCCCCCTTCC

CTCTCTCTCCCTGTCTAGAACCTTTCAACTTCTTACCTGGATTTGATTTTTTGTGAGGGGAGTTTTTGCC

TTTCAAGTTCATATTTTTCCTCTGGATTAGTTAATTATAATCCTATTATTTGTAGTCTGCTTTGAATATC

TCTTTTGAAGTTCTTGCTGTGTAAGCTTACCCTGTAGACAGAAACATGGCTTTGAAGAATGACCTTGGAC

GAGTGTCTTGTAAGTTATTCTGATGATCGTAAACTTGTATAAGTTTCAAGACATTACTATTTTGACTAAG

GTCTCTCNTAAATCATATAATAGACAATCTCCTTGTAATTGACCTTTGACTTGGATTTTCCAATTCAATG

ATCTATCAGGAGTTCTATCCTAAATAATGTTTCTAGCATTGATTTCAGATATATTCTGATGTAGGAGTTT

AATATGTAATTGTACTTTTGTAATTGCCCCAT

>Gaussia_maya_AJ830165.1

ATGCTACTCCTTTTACTGATGTCACAGCAAGTAAATCCATAGAACCCTTCTTGCAAGCATTACCTGAGAA

GCCTGTCGCAATTTAACTTGCTATGCTGGAACTTTTTTGCTCATTCAATTTAAGAATTCTGAGGCACTGT

ATCTGATGTTAAAGAAGATTAAGGAAAGTTTCGTTTGTNNTGCTCCAGGAGGAAGTAATTTTGCATATGT

CTAGCTAAAAGACATTGATAGCNTATACAAAAACACATGGTTATTCAATTTGCATTACTGTTAAGGATTT

GTACTAGATATGGTTTAATAAATATGTTTGAGAACTGTTACCCCCCCCCCCCCCAACCACCACCACCCCC

CTTCCCTCTCTCTCCCTGTCTAGAACCTTTCAACTTTTTACCTGGATTTGATTTTTTTTGAGGGGAGTTT

TTGCCTTTCAAGTTCATATTTTTCCTCTGGATTAGTTAATTATAATCCTATTATTTGTAGTCTGCTTTGA

ATATCTCTTTTGAAGTTCTTGCTGTGTAAGCTTACCCTGTAGACAGAAACATGGCTNTGAAGAATGACCT

TGGACGAGTGTCTTGTAAGTTATTCTGATGATCCTAAACTTGTATAAGTTTCAAGACATTACTATTTTGA

CTAAGGTCTCTCCTAAATCATATAGTAGACAATCTCCTTGTAATTNNCCTTTGACTTGGATTTTCCAATT

CAATGATCTATCAGGAGTTCTATCCTAAATAATGTTTCTAACATTGATTTCAGATATATTCTGATGTAGT

TGTTTAATATGTAATTGTACTTTTGTAATTGCCCCATTATAAGAAAT

>Gaussia_princeps_EF491141.1

CTACTCCTTTTACTGATGTCACAGTAAGTAAATCCATAGAACCTTCTTGCAAGCATTACCTGAGAAGCCT

GTCGCAATTTAACTTGCTATGCTGGAACTTTTTTGCTCATTCAATTTAAGAATTCTGAGGCACTGTATCT

GATGTTAAAGAAGATTAAGGAAAGTTTCGTTTGTGCTGCTCCAGGAGGAAGAAATTTTGCATATGTCTAG

CTAAAAGACGTTGATAGCTTNTACAAAAACACTTGGTTATTCAATTTGCATTACTGTTAAGGATTTGTAC

TAGATATGGTTTGATAAACATGTTAGAGAACTGTTAACCCCCCCCCCCCAACCCCCCCMCAACACCACCC

ACCCACCCCCCACCCCCCTTCCCTCTCTCTCTCTGTCTAGAACCTTTCAACTTCTTACCTGGATTTGATT

TTTTTGAGGGGAGTTTTTGCCTTTCAAGTTCATATTTTTCCTCTGGATTAGTTAATTATAATCCTATTAT

TTGTAGTCTGCTTTGAATATCTCTTTTGGAGTTCTTGCTGTGTAAGTTTACCCTGTAGACAGAAACATGG

CTTTGAAGAATGACCTTGGACGAGTGTCTTGTAAGTTATTCTGATGAACCTAAACTCGTATGAGTTTCAA

GACATTACTATTTTGACTAAGGTCTCTCCTAAATCATATAGTAGACAATCTCCTTGTAATTGACCTTTGA

CTTGGATTTTCCAATTCAATGATCTATCAGGAGTTCTATCCTAAATAATGTTTCTAACATTGATTTCAGA

TATATTCTGATGTAGGAGTTTAATATGTAATTGTACTTTTGTAATCGCCCCAT

>Geonoma_undata_JQ417531.1

TTGTCTTTAGAGTTCATATTTTTTCCTTTGGATTAGTTTATTATAATCCTGTTATTTGAAGTCTACTTTA

AATATCTCTTTTGAAGTTCTTGATATGTAAGTTTACTGTAACGTGGCTTTGAAGAATGACTTTGGACGAG

TGTCTCGTAAGTTATTCTGATGATCCTAAACTTGTATAAAGTTTCAAGACATACATTTTTGACCGATCTC

TCTCCTAAATTATATAATAGACTATCTCTTGTAACTGACCTGTGACTTGGATTTTCCAGTTCAATGATCT

ATCAGGAGTTCTGTCCTAAATAATGTTTCTAACATTGATTTCAGAYATATTC

>Geonoma_undata_subsp.skovii_JQ417529.1

TTGCCTTTAGAGTTCATATTTTTTCCTTTGGATTAGTTAATTATAATCATGTTATTTGAAGTCTACTTTA

AATATCTCTTTTGAAGTTCTTGATATGTAAGTTTACCGTAACGTGGCTTTGAAGAATGACTTTGGACGAG

TGTCTCGTAAGTTATTCTGATGATCCTAAACTTGTATAAAGTTTCAAGACATACATTTTTGACCGATCTC

TCTCCTAAATTATATAATAGACTATCTCTTGTAACTGACCTGTGACTTGGATTTTCCAGTTCAATGATCT

ATCAGGAGTTCTGTCCTAAATAATGTTTCTAACATTGATTTCAGATATATTCTGATGTAGGAGTTTAATA

GGTATTGGTACTTTTATAATTTCCCCATTACAAAATATGCTTATATTATCACAAATTTCCTAGGTGGACA

ATATCAGCAAAGCTCTTCATAAG

>Geonoma_schottiana_JQ417527.1

TTGCCTTTAGAGTTCATATTTTTTTCCTTTGGATCAGTTAATTATAATCCTGTTATTTGAAGTCTACTTT

AAATATCTCTTTTGAAGTTCTTGATATGTAAGTTTACCGTAAYGTGACTTTGAAGAATGACTTTGGACGA

GTGTCTTGTAAGTTATTCTGATGATCCTAAACTTGTATAAAGTTTCAAGACATACTTTTTTGACCGATCT

CTCTCCTAAATTATATAATAGACTATCTCCTTGTAACTGACCTGTGACTTGGATTTTCCAGTTCAATGAT

CTATCAGGAGTTCTGTCCTAAATAATGTTTCTAACATTGATTTCAGATATATTCTGATGTAGGAGTTTAA

TAGGTAATGGTACTTTTATAATTTCCCCA

>Geonoma_pohliana_subsp.rubescens_JQ417525.1

TTGCCTTTAGAGTTCATATTTTTTTCCTTTGGATTAGTTAATTATAATCCTGTTATTTGAAGTMTACTTT

AAATATCTCTTTTGAAGTTCTTGATATGTAAGTTTACCGTAACGTGACTTTGAAGAATGACTTTGGACGA

GTGTCTTGTAAGTTATTCTGATGATCCTAAACTTGTATAAAGTTTCAAGACATACTTTTTTGACCAATCT

CTCTCCTAAATTATATAATAGACTATCTCCTTGTAACTGACCTGTGACTTGGATTTTCCAGTTCAATGAT

CTATCAGGAGTTCTGTCCTAAATAATGTTTCTAACATTGATTTCAGATATATTCTGATGTAGGAGTTTAA

TAGGTAATGGTACTTTTATAATTTCCCCATT

>Geonoma_pohliana_JQ417521.1

TTGCCTTTAGAGTTCATATTTTTTTCCTTTGGATTAGTTAATTATAATCCTGTTATTTGAAGTATACTTT

AAATATCTCTTTTGAAGTTCTTGATATGTAAGTTTACCGTAACGTGACTTTGAAGAATGACTTTGGACGA

GTGTCTTGTAAGTTATTCTGATGATCCTAAACTTGTATAAAGTTTCAAGACATACTTTTTTGACCAATCT

CTCTCCTAAATTATATAATAGACTATCTCCTTGTAACTGACCTGTGACTTGGATTTTCCAGTTCAATGAT

CTATCAGGAGTTCTGTCCTAAATAATGTTTCTAACATTGATTTCAGATATATTCTGATGTAGGAGTTTAA

TAGGTAATGGTACTTTTATAATTTC

>Geonoma_maxima_JQ417519.1

TTGCCTTTAGAGTTCATATTTTTTCCTTTGGATTAGTTAATTATAATCCTGTTATTTGAAGTCTACTTTA

AATCTCTCTTTTGAAGTTCTTGATATGTAAGTTTACTGTAACGTGGCTTTGAAGAATGACTTTGGACGAG

TGTCTTGTAAGTTATTCTGATGATCCTAACCTTGTATAAAGTTTCAAGACATACTTTTTTGACCGATCTC

TCTCCTAAATTATATAATAGACTATCTCCTTGTAACTGACCTGTGACTTGGATTTTCCAGTTCAATGATC

TATCAGGAGTTCTGTCCCAAATAATGTTTCTAACATTGATTTCAGATATATTCTGATGTAGGAGTTTAAT

AGGTAATGGTACTTTTATAATTTCCCCATTACAAAAAATGCTTATATTATCACAAATTCCTAGGT

>Geonoma_longivaginata_JQ417517.1

TTGCCTTTAGAGTTCATATTTTTTCCTTTGGATTAGTTAATTATAATCCTGTTATTTGAAGTCTACTTTA

AATATCTCTTTTGAAGTTCTTGATATGTAAGTTTACCGTAACGTGGCTTTGAAGAATGACTTTGGACGAG

TGTCTTGTAAGTTATTCTGATGATCCTAAACTTGTATAAAGTTTCAAGACATACATTTTTGACCGATCTC

TCTCCTACATTATATAATAGACTATCTCTTGTAACTGACCTGTGACTTGGATTTTCCAGTTCAATGATCT

ATCAGGAGTTCTGTCCTAAATAATGTTTCTAACATTGATTTCAGATATATTCTGATGTAGGAGTTTAATA

GGTAATGGTACTTTTATAATTTCCCCATTACAAAAAATGCTTATATTATCACAAATTTCCTAGGTGGACA

ATATCAGCAAAGCTCTTCATAAA

>Geonoma_ferruginea_JQ417515.1

TTGCCTTTAGAGTTCATATTTTTTCCTTTGGATTAGTTAATTATAATCCTGTTATTTGAAGTCTACTTTA

AATATCTCTTTTGAAGTTCTTGATATGTAAGTTTACCGTAACGTGGCTTTGAAGAATGACTTTGGACGAG

TGTCTTGTAAGTTATTCTGATGATCCTAAACTTGTATAAAGTTTCAAGACATACATTTTTGACCGATCTC

TCTCCTAAATTATATAATAGACTATCTCTTGTAACTGACCTGTGACTTGGATTTTCCAGTTCAATGATCT

ATCAGGAGTTCTGTCCTAAATAATGTTTCTAACATTGATTTCAGATATATTCTGATGTAGGAGTTTAATA

GGTAATGGTACTTTTATAATTTCCCCATTACAAAAAATGCTTATATTATCACAAATTTCCTAGGTGGACA

ATATCAGCAAAGCTCTTCATAAA

>Geonoma_leptospadix_JQ417513.1

TTGCCTTTAGAGTTCATATTTTTTTCCTTTGGATTAGTTAATTATAATCCTGTTATTTGAAGTCTACTTT

AAATATCTCTTTTGAAGTTCTTGATATGTAAGTTTACCGTAACGTGGCTTTGAAGAATGACTTTGGACGA

GTGTCTTGTAAGTTATTCTGATGATCCTAAACTTGTATAAAGTTTCAAGACATACTTTTTTGACCGATCT

CTCTACTAAATTATATAATAGACTATCTCCTTGTAACTGACCTGTGACTTGGATTTTCCAGTTCAATGAT

CTATCAGGAGTTCTGTCCTAAATAATGTTTCTAACATTGATTTCAGATATATTCTGATGTAGGAGTTTAA

TAGGTAATGGTACTTTTATAATTTCCCCATTACAAAAAATGCTTATATTATCACAAATTCCTAGGTGGAC

AA

>Geonoma_cuneata_subsp.irena_JQ417511.1

TTGCCTTTAGAGTTCATATTTTTTCCTTTGGATTAGTTAATTATAATCCTGTTATTTGAAGTCTACTTTA

AATATCTCTTTTGAAGTTCTTGATATGTAAGTTTACCGTAACGTGGCTTTGAAGAATGACTTTGGACAAG

TGTCTTGTAAGTTATTCTGATGATCCTAAACTTGTATAAAGTTTCAAGACATACATTTTTGACCGATCTC

TCTCCTAAATTATATAATAGACTATCTCTTGTAACTGACCTGTGACTTGGATTTTCCAGTTCAATGATCT

ATCAGGAGTTCTGTCCTAAATAATGTTTCTAACATTGATTTCAGATATATTCTGATGTAGGAGTTTAATA

GGTAATGGTACTTTTATAATTTCCCCATTACAAAAAATGCTTATATTATCACAAATTTCCTAGGTGGACA

ATATCAGCAAAGCTCTTCATAAG

>Geonoma_interrupta_JQ417509.1

TTGCCTTTAGAGTTCATTTTTTTTCCTTTGGATTAGTTAATTATAATCCTGTTATTTGAAGTCTATTTTA

AATATCTCTTTTAAAGTTCTTGATATGTAAGTTTACCGTAACGTGGCTTTGAAGAATGACTTTGGACGAG

TGTCTTGTAAGTTATTCTGATGATCCTAAACTTGTATAAAGTTTCAAGACATACTTTTTTGACCGATCTC

TCTCCTAAATTATATAATAGATTATCTCCTTGTAACTGACCCGTGACTTGGATTTTCCAGTTCAATGATC

TATCAGGAGTTCTGTCCTAAATAATGTTTCTAACATTGATTTCAGATATATTCTGATGTAGGAGTTTAAT

AGGTAATGGTACTTTTATAATTTCCCCATTACAAAAAATGCTTATATTATCACAAATTTCGTAGGTGGAC

AATATTAGCAAAGCTCTTCATAAG

>Geonoma_frontinensis_JQ417507.1

TTGCCTTTAGAGTTCATATTTTTTTCCTTTGGATTAGTTAATTATAATCCTGTTATTTGAAGTCTACTTT

AAATATCTCTTTTAAAGTTCTTGATATGTAAGCTTACCGTAACGTGGCTTTGAAGAATGACTTTGGACGA

CTGTCTTGTAAGTTATTCTGATGATCCTAAACTTGTATAAAGTTTCAAGACATACTTTTTTGACCGATCT

CTCTCCTAAATTATATAATAGATTATCTCCTTGTAACTGACCTGTGACTTGGATTTTCCAGTTCAATGAT

CTATCAGGAGTTCTGTCCTAAATAATGTTTCTAACATTGATTTCAGATATATTCTGATGTAGGAGTTTAA

TAGGTAATGGTACTTTTATAATTTCCCCATTACAAAAAATGCTTATATTATCACAAATTTCGTAGGTGGA

CAA

>Geonoma_macrostachys_var.acaulis_JQ417505.1

TTGCCTTTAGAGTTCATATTTTTTCCTTTGGATTAGTTAATTATAATCCTGTTATTTGAAGTCTACTTTA

AATCTCTCTTTTGAAGTTCTTGATATGCAAGTTTACTGTAACGTGGCTTTGAAGAATGACTTTGGACGAG

TGTCTTGTAAGTTATTCTGATGATCCTAAACTTGTATAAAGTTTCAAGACATACTCTTTTGACCGATCTC

TCTCCTAAATTATATAATAGACTATCTCCTTGTAACTGACCTGTGACTTGGATTTTCCAGTTCAATGATC

TATCAGGAGTTCTGTCCTAAATAATGTTTCTAACATTGATTTCAGATATATTCTGATGTAGGAGTTCTGT

CCTAAATAATGTTTCCCCATTACAAAAAATGCTTATATTATCACAAGTTTCCTAGGTGGACAATATCAGC

AAAGCTCTTCATAAG

>Geonoma_deversa_subsp.deversa_JQ417503.1

TTGCCTTTAGAGTTCATATTTTTTTCCTTTGGATTAGTTAATTATAATCCTGTTATTTGAAGTCTACTTT

AAATATCTCTTTTGAAGTTCTTGATATGTAAGTTTACCGTAACGTGGCTTTGAAGAATGACTTTGGACGA

GTGTCTTGTAAGTTATTCTGATGATCCTAAACTTGTATAAAGTTTCAAGACATACTTTTTTGACCGATCT

CTCTCCTAAATTATATAATAGACTATCTCCTTGTAACTGACCTGTGACTTGGATTTTCCAGTTCAATGAT

CTATCAGGAGTTCTGTCCTAAATAATGTTTCTAACATTGATTTCAGATATATTCTGATGTAGGAGTTTAA

TAGGTAATGGTACTTTTATAATTTCCCCATTACAAAAAATGCTTATATTATCACAAATTTCCTAGGTGGA

CAATATCAGCAAAGCTCTTCATAAG

>Geonoma_congesta_JQ417501.1

TTGCCTTTAGAGTTCATACTTTTTCCTTTGGATTAGTTAATTATAATCCTGTTATTTGAAGTCTGCTTTA

AATCTCTCTTTTGAAGTTCTTGATATGCAAGTTTACTGTAACGTGGCTTTGAAGAATGACTTTGGACGAG

TGTCTTGTAAGTTATTCTGATGATCCTAAACTTGTATAAAGTTTCAAGGCATACTTTTTTGACCGTTCTC

TCTCCTAAATTATATAATAGACTATCTCCTTGTAACTGACCTGTGACTTGGATTTTCCAGTTCAATGATC

TATCAGGAGTTCTGTCCTAAATAATGTTTCTAACATTGATTTCAGATATATTCTGATGTAGGAGTTTAAT

AGGTAATGGTACTTTTATAATTTCTCCATTACAAAAAATGCTTATATTATCACAAATTTCCTAGGTGGAC

AATATCAGCAAAGCTCTTCATAAG

>Geonoma_brenesii_JQ417499.1

TTGCCTTTAGAGTTCATATTTTTTCCTTTGGATTAGTTAATTATAATCCTGTTATTTGAAGTCTACTTTA

AATATCTCTTTTGAAGTTCTTGATATGTAAGTTTACCGTAACGTGGCTTAGAAGAATGACTTTGGACGAG

TGTCTTGTAAGTTATTCTGATGATCCTAAACTTGTATAAAGTTTCAAGACATACATTTTTGACCGATCTC

TCTCCTAAATTATATAATAGACTATCTCTTGTAACTGACCTGTGACTTGGATTTTCCAGTTCAATGATCT

ATCAGGAGTTCTGTCCTAAATAATGTTTCTAACATTGATTTCAGATATATTCTGATGTAGGAGTTTAATA

GGTAATGGTACTTTTATAATTTCCCC

>Geonoma_atrovirens_JQ417497.1

TTGCCTTTAGAGTTCATATTTTTTCCTTTGGATTAGTTAATTATAATCCTGTTATTTGAAGTCTACTTTA

AATCTCTCTTTTGAAGTTCTTGATATGCAGTTTACTGTAACGTGGCTTTGAAGAATGACTTTGGACGAGT

GTCTTGTAAGTTATTCTGATGATCCTAAACTTGTATAAAGTTTCAAGACATACTTTTTTGACCGATCTCT

CTCCTAAATTATATAATAGACTATCTCCTTGTAACTGACCTGTGACTTGGATTTTCCAGTTCAATGATCT

ATCAGGAGTTCTGTCCTAAATAATGTTTCTAACATTGATTTCAGATATATTCTGATGTAGGAGTTTAATA

GGTAATGGTACTTTTATAATTTCCCCATTACAAAAAATGCTTATATTATCACAAATTTCCTAGGTGGACA

ATATCAGCAAAGCTCTTCACAAG

>Geonoma_orbignyana_subsp.hoffmanniana_JQ417532.1

TTGCCTTTAGAGTTCATATTTTTTCCTTTGGATTAGTTAATTATAATCCTGTTATTTGAAGTCTACTTTA

AATATCTCTTTTGAAGTTCTTGATATGTAAGTTTACCGTAACGTGGCTTTGAAGAATGACTTTGGACGAG

TGTCTCGTAAGTTATTCTGATGATCCTAAACTTGTATAAAGTTTCAAGACATACATTTTTGACCGATCTC

TCTCCTAAATTATATAATAGACTATCTCTTGTAACTGACCTGTGACTTGGATTTTCCAGTTCAATGATCT

ATCAGGAGTTCTGTCCTAAATAATGTTTCTAACATTGATTTCAGATATATTCTGATGTAGGAGTTTAATA

GGTATTGGTACTTTTATAATTTCCCCATTACAAAATATGCTTATATTATCACAAATTTCCTAGGTGGAC

>Geonoma_supracostata_JQ417530.1

TTGCCTTTAGAGTTCATATTTTTTCCTTTGGATTAGTTAATTATAATCCTGTTATTTGAAATCTACTTTA

AATCTCTCTTTTGAAGTTCTTGATATGCAAGTTTACTGTAACGTGGCTTTGAAGAATGACTTTGGACGAG

TGTCTTGTAAGTTATTCTGATGATCCTAAACTTGTATAAAGTTTCAAGACACTTTTTTGACCGATCTCTC

TCCTAAATTATATAATAGACTATCTCCTTGTAACTGACCTGTGACTTGGATTTTCCAGTTCAATGATCTA

TCAGGAGTTCTGTCCTAAATAATGTTTCTAACATTGATTTCAGATATATTCTGATGTAGGAGTTTAATAG

GTAATGGTACTTTTATAATTTCCCCATTACAAAAAATACTTATATTATCACAAATTTCCTAGGTGGACAA

TATCAGCAAAGCTCTTCATAAG

>Geonoma_simplicifrons_JQ417528.1

TTTTCTTTAGAGTTCATATTTTTTTCCTTTGGATTAGTTAATTATAATCCTGTTATTTGAAGTCTACTTT

AAATATCTCTTTTAAAGTTCTTGATATGTAAGTTTACCGTAACGTGGCTTTGAAGAATGACTTTGGACGA

GTGTCTTGTAAGTTATTCTGATGATCCTAAACTTGTATAAAGTTTCAAGACATACTTTTTTGACCGATCT

CTCTCCTAAATTATATAATAGACTATCTCCTTGTAACTGACCTGTGACTTGGGTTTTCCAGTTCAATGAT

CTATCAGGAGTTCTGTCCTAAATAATGTTTCTAACATTGATTTCAGATATATTCTGATGTAGGAGTTTAA

TAGGTAATGGTACTTTTATAATTTGCCCATTACAAAAATGCTTATATTATCACAAATTCCTAGGTGGACA

A

>Geonoma_schottiana_JQ417526.1

TTGCCTTTAGAGTTCATATTTTTTTCCTTTGGATTAGTTAATTATAATCCTGTTATTTGAAGTCTACTTT

AAATATCTCTTTTGAAGTTCTTGATATGTAAGTTTACCGTAACGTGACTTTGAAGAATGACTTTGGACGA

GTGTCTTGTAAGTTATTCTGATGATCCTAAACTTGTATAAAGTTTCAAGACATACTTTTTTGACCGATCT

CTCTCCTAAATTATATAATAGACTATCTCCTTGTAACTGACCTGTGACTTGGATTTTCCAGTTCAATGAT

CTATCAGGAGTTCTGTCCTAAATAATGTTTCTAACATTGATTTCAGATATATTCTGATGTAGGAGTTTAA

TAGGTAATGGTACTTTTATAATTTCCCCATTACAAAAAATGCTTATATTATCACAAATTTCCTAGGTGGA

CAATATCAGCAAAGCTCTTCATAAG

>Geonoma_pohliana_subsp.kuhlmannii_JQ417524.1

TTGCCTTTAGAGTTCATATTTTTTTCCTTTGGATTAGTTAATTATAATCCTGTTATTTGAAGTCTACTTT

AAATATCTCTTTTGAAGTTCTTGATATGTAAGTTTACCGTAACGTGACTTTGAAGAATGACTTTGGACGA

GTGTCTTGTAAGTTATTCTGATGATCCTAAACTTGTATAAAGTTTCAAGACATACTTTTTTGACCAATCT

CTCTCCTAAATTATATAATAGACTATCTCCTTGTAACTGACCTGTGACTTGGATTTTCCAGTTCAATGAT

CTATCAGGAGTTCTGTCCTAAATAATGTTTCTAACATTGATTTCAGATATATTCTGATGTAGGAGTTTAA

TAGGTAATGG

>Geonoma_undata_JQ417522.1

TTGCCTTTAGAGTTCATATTTTTTCCTTTGGATTAGTTAATTATAATCCTGTTATTTGAAGTCTACTTTA

AATATCTCTTTTGAAGTTCTTGATATGTAAGTTTACCATAACGTGGCTTTGAAGAATGACTTTGGACGAG

TGTCTCGTAAGTTATTCTGATGATCCTAAACTTGTATAAAGTTTCAAGACATACATTTTTGACCGATCTC

TCTCCTAAATTATATAATAGACTATCTCTTGTAACTGACCTGTGACTTGGATTTTCCAGTTCAATGATCT

ATCAGGAGTTCTGTCCTAAATAATGTTTCTAACATTGATTTCAGATATATTCTGATGTAGGAGTTTAATA

GGTATTGGTACTTTTATAATTTCCCCATTACAAAATATGCTTATATTATCACAAATTTCCTAGGTGGACA

ATATCAGCAAAGCTCTTCATAAG

>Geonoma_orbignyana_JQ417520.1

TTGCCTTTAGAGTTCATATTTTTTCCTTTGGATTAGTTAATTATAATCCTGTTATTTGAAGTCTACTTTG

AATATCTCTTTTGAAGTTCTTGATATGTAAGTTTACCGTAACGTGGCTTTGAAGAATGACTTTGGACGAG

TGTCTCGTAAGTTATTCCGATGATCCTAAACTTGTATAAAGTTTCAAGACATACATTTTTGACCGATCTC

TCTCCTAAATTATATAATAGACTATCTCTTGTAACTGACCTGTGACTTGGATTTTCCAGTTCAATGATCT

ATCAGGAGTTCTGTCCTAAATAATGTTTCTAACATTGATTTCAGATATATTCTGATGTAGGAGTTTAATA

GGTATTGGTACTTTTATAATTTCCCCATTACAAAATATGCTTATATTATCACAAATTTCCTAGGTGGACA

ATATCAGCAAAGCTCTTCATAAG

>Geonoma_longivaginata_JQ417518.1

TTGCCTTTAGAGTTCATATTTTTTCCTTTGGATTAGTTAATTATAATCCTGTTATTTGAAGTCTACTTTA

AATATCTCTTTTGAAGTTCTTGATATGTAAGTTTACCGTAACGTGGCTTTGAAGAATGACTTTGGACGAG

TGTCTTGTAAGTTATTCTGATGATCCTAAACTTGTATAAAGTTTCAAGACATACATTTTTGACCGATCTC

TCTCCTAAATTATATAATAGACTATCTCTTGTAACTGACCTGTGACTTGGATTTTCCAGTTCAATGATCT

ATCAGGAGTTCTGTCCTAAATAATGTTTCTAACATTGATTTCAGATATATTCTGATGTAGGAGTTTAATA

GGTAATGGTACTTTTATAACTTCCCCATTACAAAAAATGCTTATATTATCACAAATTTCCTAGGTGGACA

ATATCAGCAAAGCTCTTCATAAG

>Geonoma_ferruginea_JQ417516.1

TTGCCTTTAGAGTTCATATTTTTTCCTTTGGATTAGTTAATTATAATCCTGTTATTTGAAGTCCACTTTA

AATATCTCTTTTGAAGTTCTTGATATGTAAGTTTACCGTAACGTGGCTTTGAAGAATGACTTTGGACGAG

TGTCTTGTAAGTTATTCTGATGATCCTAAACTTGTATAAAGTTTCAAGACATACATTTTTGACCGATCTC

TCTCCTAAATTATATAATAGACTATCTCTTGTAACTGACCTGTGACTTGGATTTTCCAGTTCAATGATCT

ATCAGGAGTTCTGTCCTAAATAATGTTTCTAACATTGATTTCAGATATATTCTGATATAGGAGTTTAATA

GGTAATGGTACTTTTATAATTTCCCCATTACAAAAAATGCTTATATTATCACAAATTTCCTAGGTGGACA

ATATCAGCAAAGCTCTTCATAGAG

>Geonoma_cuneata_subsp.linearis_JQ417514.1

TTGCCTTTAGAGTTCATATTTTTTCCTTTGGATTAGTTAATTATAATCCTGTTATTTGAAGTCTACTTTA

AATATCTCTTTTGAAGTTCTTGATATGTAAGTTTACCGTAACGTGGCTTTGAAGAATGACTTTGGACAAG

TGTCTTGTAAGTTATTCTGATGATCCTAAACTTGTATAAAGTTTCAAGACATACATTTTTGACCGATCTC

TCTCCTAAATTATATAATAGACTATCTCTTGTAACTGACCTGTGACTTGGATTTTCCAGTTCAATGATCT

ATCAGGAGTTCTGTCCTAAATAATGTTTCTAACATTGATTTCAGATATATTCTGATGTAGGAGTTTAATA

GGTAATGGTACTTTTATAATTTCCCCATTACAAAAAATGCTTATATTATCACAAATTTCCTAGGTGGACA

ATATCAGCAAAGCTCTTCATAAG

>Geonoma_laxiflora_JQ417512.1

TTGCCTTTAGAGTTCATATTTTTTCCTTTGGATTAGTTAATTATAATCCTGTTATTTGAAGTCTACTTTA

AATATCTCTTTTGAAGTTCTTGATATGTAAGTTTACCGTAACGTGGCTTTGAAGAATGACTTTGGACGAG

TGTCTTGTAAGTTATTCTGATGATCCTAAACTTGTATAAAGTTTCAAGACATACATTTTTGACCGATCTC

TCTCCTAAATTATATAATAGACCATCTCTTGTAACTGACCTGTGACTTGGATTTTCCAGTTCAATGATCT

ATCAGGAGTTCTGTCCTAAATAATGTTTCTAACATTGATTTCAGATATATTCTGATGTAGGAGTTTAATA

GGTAATGGTACTTTTATAATTTCCCCATTACAAAAAATGCTTATATTATCACAAATTCCTAGGTGGACAA

>Geonoma_interrupta_JQ417510.1

TTGCCTTTAGAGTTCATATTTTTTTCCTTTGGATTAGTTAATTATAATCCTGTTATTTGAAGTCTACTTT

AAATATCTCTTTTAAAGTTCTTGATATGTAAGTTTACCGTAACGTGGCTTTGAAGAATGACTTTGGACGA

GTGTCTTGTAAGTTATTCTGATGATCCTAAACTTGTATAAAGTTTCAAGACATACTTTTTTGACCGATCT

CTCTCCTAAATTATATAATAGACTATCTCCTTGTAACTGACCTGTGACTTGGATTTTCCAGTTCAATGAT

CTATCAGGAGTTCTGTCCTAAATAATGTTTCTAACATTGATTTCAGATATATTCTGATGTAGGAGTTTAA

TAGGTAATGGTACTTTTATAATTTCCCCATTACAAAAAATGCTTATATTATCACAAATTTCCTAGGTGGA

CAATATCAGCAAAGCTCTTCATAAG

>Geonoma_hollinensis_JQ417508.1

TTGCCTTTAGAGTTCATATTTTTTCCTTTGGATTAGTTAATTATAATCCTGTTATTTGAAGTCTACTTTA

AATATCTCTTTTGAAGTTCTTGATATGTAAGTTTACCGTAACGTGGCTTTGAAGAATGACTTTGGACGAG

TGTCTTGTAAGTTATTCTGATGATCCTAAACTTGTATAAAGTTTCAAGACATACATTTTTGACTGATCTC

TCCTAAATTATATAATAGACTTTCTCTTGTAACTGACCTGTGACTTGGATTTTCCAGTTCAATGATCTAT

CAGGAGTTGTCCTAAATAATGTTTCTAACATTGATTTCAGATATATTCTGATGTAGGAGTTTAATAGGTA

ATGGTACTTTTATAATTTCCCCATTACAAAAAATGCTTATATTATCACAAATTTCCTAGGTGGACAATAT

CAGCAAAGCTCTTCGTAAG

>Geonoma_elegans_JQ417506.1

TTGCCTTTAGAGTTCATATTTTTTTCCTTTGGATTAGTTAATTATAATCCTGTTATTTGAAGTCTACTTT

AAATATCTCTTTTGAAGTTCTTGATATGTAAGTTTACCGTAACGTGGCTTTGAAGAATGACTTTGGACGA

GTGTCTTGTAAGTTATTCTGATGATCCTAAACTTGTATAAAGTTTCAAGACATACTTTTTTGACCGTTCT

CTCTCCTAAATTATATAATAGACTATCTCCTTGTAACTGACCTGTGACTTGGATTTTCCAGTTCAATGAT

CTATCAGGAGTTCTGTCCTAAATAATGTTTCTAACATTGATTTCAGATATATTCTGATGTAGGAGTTTAA

TAGGTAATGGTACTTTTATAATTTCCCCATTACAAAAAATGCTTATATTATCACAAATTCCTAG

>Geonoma_divisa_JQ417504.1

TTGCTTTTAGAGTTCATATTTTTTCCTTTGGATTAGTTAATTATAATCCTGTTATTTGAAGTCTGCTTTA

AATATATCTTTTGAAGTTCTTGATATGTAAGTTTACCGTAACGTGGCTTTGAAGAATGACTTTGGACGAG

TGTCTTGTAAGTTATTCTGATGATCCTAAACTTGTATAAAGTTTCAAGACATACATTTTTGACCGATCTC

TCTCCTAAATTATATAATAGACTATCTCTTGTAACTGACCTGTGACTTGGATTTTCCAGTTCAGTGATCT

ATCAGGAGTTCTGTCCTAAATAATGTTTCTAACATTGATTTCAGATATATTCTGATGTAGGAGTTTAATA

GGTAATGGTACTTTTATAATTTCCCCATTACAAAAAATGCTTATATTATCACAAATTCCTAGGTGGACAA

>Geonoma_cuneata_subsp.cuneata_JQ417502.1

TTGCCTTTAGAGTTCATATTTTTTCCTTTGGATTAGTTAATTATAATCCTGTTATTTGAAGTCTACTTTA

AATATCTCTTTTGAAGTTCTTGATATGTAAGTTTACCGTAACGTGGCTTTGAAGAATGACTTTGGACAAG

TGTCTTGTAAGTTATTCTGATGATCCTAAACTTGTATAAAGTTTCAAGACATACATTTTTGACCGATCTC

TCTCCTAAATTATATAATAGACTATCTCTTGTAACTGACCTGTGACTTGGATTTTCCAGTTCAATGATCT

ATCAGGAGTCCTGTCCTAAATAATGTTTCTAACATTGATTTCAGATATATTCTGATGTAGGAGTTTAATA

GGTAATGGTACTTTTATAATTTCCCCATTACAAAAAATGCTTATATTATCACAAATTTCCTAGGTGGACA

ATATCAGCAAAGCTCTTCATAAG

>Geonoma_bernalii_JQ417498.1

TTGCCTTTAGAGTTCATATTTTTTTCCTTTGGATTAGTTAATTATAATCCTGTTATTTGAAGTCTACTTT

AAATATCTCTTTTGAAGTTCTTGATATGTAAGTTTACCGTAACGTGGCTTTGAAGAATGACTTTGGACGA

GTGTCTTGTAAGTTATTCTGATGATCCTAAACTTGTATAAAGTTTCAAGACATACTTTTTTGACCGATCT

CTCTCCTAAATTATATAATAGACTATCTCCTTGTAACTGACCTGTGACTTGGATTTTCCAGTTCAATGAT

CTATCAGGAGTTCTGTCCTAAATAATGTTTCTAACATTGATTTCAGATATATTCTGATGTAGGAGTTTAA

TAGGTAATGGTACTTTTATAATTTCCCCATTACAAAAAATGCTTATATTATCACAAATTCCTAGGTGGAC

AA

>Geonoma_stricta_subsp.arundinacea_JQ417496.1

TTGCCTTTAGAGTTCATATTTTTTCCTTTGGATTAGTTAATTATAATCCTGTTATTTGAAGTCTACTTTA

AATATCTCTTTTGAAGTTCTTGATATGTAAGTTTACCGTAACGTGGCTTTGAAGAATGACTTTGGACGAG

TGTCTTGTAAGTTATTCTGATGATCCTAAACTTGTATAAAGTTTCAAGACATACATTTTTGACCGATCTC

TCTCCTAAATTATATAATAGACTATCTCTTGTAACTGACAGTTCAATGATCTATCAGGAGTTCTGTCCTA

AATAATGTTTCTAACATTGATTTCAGATATATTCTGATGTAGGAGTTTAATAGGTAATGGTACTTTTATA

ATTTCCCCATTACAAAAAATGCTTATATTATCACAAATTCCTAGGTGGACA

>Geonoma_edulis_HM140615.1

TTGCCTTTAGAGTTCATATTTTTTCCTTTGGATTAGTTAATTATAATCCTGTTATTTGAAGTCTACTTTA

AATATCTCTTTTGAAGTTCTTGATATGTAAGTTTACCGTAACGTGGCTTTGAAGAATGACTTTGGACGAG

TGTCTCGTAAGTTATTCTGATGACCCTAAACTTGTATAAAGTTTCAAGACATACATTTTTGACCGATCTC

TCTCCTAAATTATATAATAGACTATCTCTTGTAACTGACCTGTGACTTGGATTTTCCAGTTCAATGATCT

ATCAGGAGTTCTGTCCTAAATAATGTTTCTAACATTGATTTCAGATATATTCTGATGTAGGAGTTTAATA

GGTATTGGTACTTTTATAATTTCCCCATTACAAAATATGCTTATATTATCACAAATTTCCTAGGTGGACA

ATATCAGCAAAGCTCTTCATAAG

>Geonoma_umbraculiformis_HM140613.1

TTGCCTTTAGAGTTCATATTTTTTCCTTTGGATTAGTTAATTATAATCCTGTTATTTGAAGTCTACTTTA

AATCTCTCTTTTGAAGTTCTTGATATGCAAGTTTACTGTAACGTGGCTTTGAAGAATGACTTTGGACGAG

TGTCTTGTAAGTTATTCTGATGATCCTAAACTTGTATAAAGTTTCAAGACAATACTTTTTTGACCGATCT

CTCTCCTAAATTATATAATAGACTATCTCCTTGTAACTGACCTGTGACTTGGATTTTCCAGTTCAATGAT

CTATCAGGAGTTCTGTCCTAAATAATGTTTCTAACATTGATTTCAGATATATTCTGATGTATGAGTTTAA

TAGGTAATGGTACTTTTATAATTTCCCCATTACAAAAAATGCTTATATTATCACAAATTTCCTAGGTGGA

CAATATCAGCAAAGCTCTTCATAAG

>Geonoma_scoparia_HM140611.1

TTGCCTTTAGAGTTCATATTTTTTCCTTTGGATTAGTTAATTATAATCCTGTTATTTGAAGTCTACTTTA

AATATCTCTTTTGAAGTTCTTGATATGTAAGTTTACCGTAACGTGGCTTTGAAGAATGACTTTGGACGAG

TGTCTTGTAAGTTATTCTGATGATCCTAAACTTGTATAAAGTTTCAAGACATACATTTTTGACCGATCTC

TCTCCTAAATTATATAATAGACTATCTCTTGTAACTGACCTGTGACTTGGATTTTCCAGTTCAATGATCT

ATCAGGAGTTCTGTCCTAAATAATGTTTCTAACATTGATTTCAGATATATTCTGATGTAGGAGTTTAATA

GGTAATGGTACTTTTATAATTTCCCCATTACAAAAAATGCTTATATTATCACAAATTTCCTAGGTGGACA

ATATCAGCAAAGCTCTTCATAAG

>Geonoma_monospatha_HM140609.1

TTGCCTTTAGAGTTCATATTTTTTCCTTTGGATTAGTTAATTATAATCCTGTTATTTGAAGTCTACTTTA

AATATCTCTTTTGAAGTTCTTGATATGTAAGTTTACCGTAACGTGGCTTTGAAGAATGACTTTGGACGAG

TGTCTTGTAAGTTATTCTGATGATCCTAAACTTGTATAAAGTTTCAAGACATACATTTTTGACCGATCTC

TCTCCTAAATTATATAATAGACTATCTCTTGTAACTGACCTGTGACTTGGATTTTCCAGTTCAATGATCT

ATCAGGAGTTCTGTCCTAAATAATGTTTCTAACATTGATTTCAGATATATTCTGATGTAGGAGTTTAATA

GGTAATGGTACTTTTATAATTTCCCCATTACAAAAAATGCTTATATTATCACAAATTTCCTAGGTGGACA

ATATCAGCAAAGCTCTTCATAAG

>Geonoma_epetiolata_HM140607.1

TTGCCTTTAGAGTTCATATTTTTTCCTTTGGATTAGTTAATTATAATCCTGTTATTTGAAGTCTACTTTA

AATATCTCTTTTGAAGTTCTTGATATGTAAGTTTACCGTAACGTGGCTTTGAAGAATGACTTTGGACGAG

TGTCTTGTAAGTTATTCTGATGATCCTAAACTTGTATAAAGTTTCAAGACATACATTTTTGACCGATCTC

TCTCCTAAATTATATAATAGACTATCTCTTGTAACTGACCTGTGACTTGGATTTTCCAGTTCAATGATCT

ATCAGGAGTTCTGTCCTAAATAATGTTTCTAACATTGATTTCAGATATATTCTGATGTAGGAGTTTAATA

GGTAATGGTACTTTTATAATTTCCCCATTACAAAAAATGCTTATATTATCACAAATTTCCTAGGTGGACA

ATATCAGCAAAGCTCTTCATAAG

>Geonoma_longevaginata_HM140605.1

TTGCCTTTAGAGTTCATATTTTTTCCTTTGGATTAGTTAATTATAATCCTGTTATTTAAAGTCTACTTTA

AATATCTCTTTTGAAGTTCTTGATATGTAAGTTTACCGTAACGTGGCTTTGAAGAATGACTTTGGACGAG

TGTCTTGTAAGTTATTCTGATGATCCTAAACTTGTATAAAGTTTCAAGACATACATTTTTGACCGATCTC

TCTCCTAAATTATATAATAGACTATCTCTTGTAACTGACCTGTGACTTGGATTTTCCAGTTCAATGATCT

ATCAGGAGTTCTGTCCTAAATAATGTTTCTAACATTGATTTCAGATATATTCTGATGTAGGAGTTTAATA

GGTAATGGTACTTTTATAATTTCCCCATTACAAAAAATGCTTATATTATCACAAATTTCCTAGGTGGACA

ATATCAGCAAAGCTCTTCATAAG

>Geonoma_concinna_HM140603.1

TTGCCTTTAGAGTTCATATTTTTTCCTTTGGATTAGTTAATTATAATCCGGTTATTTGAAGTCTACTTTA

AATCTCTCTTTGGAAGTTCTTGATATGTAAGTTTACTGTAACGTGGCTTTGAAGAATGACTTTGGACGAG

TGTCTCGTAAGTTATTCTGATGATCCTAAACTTGTATAAAGTTTCAAGACATACTTTTTTGACCGATCTC

TCTCCTAAATTATATAATAGACTATCTCCTTGTAACTGACCTGTGACTTGGATTTTCCAGTTCAATGATC

TATCAGGAGTTCTGTCCTAAATAATGTTTCTAACATTGATTTCAGATATATTCTGATGTAGGAGTTTAAT

AGGTAATGGTACTTTTATAATTTCCCCATTACAGAAAATGCTTATATTATCACAAATTTCCTAGGTGGAC

AATATCAGCAAAGCTCTTCATAAG

>Geonoma_baculifera_HM140601.1

TTGTCTTTAGAGTTCATATTTTTTCCTTTGGATTAGTTAATTATAATCCTGTTATTTGAAGTCTACTTTA

AATCTCTCTTTTGAAGTTCTTGATATGCAAGTTTACTGTAACGTGGCTTTGAAGAATGACTTTGGACGAG

TGTCTTGTAAGTTATTCTGATGATCCTAAACTTGTATAAAGTTTCAAGACATACTTTTTTGACCGATCTC

TCTCCTAAATTATATAATAGACTATCTCCTTGTAACTGACCTGTGACTTGGATTTTCCAGTTCAATGATC

TATCAGGAGTTCTGTCCTAAATAATGTTTCTAACATTGATTTCAGATATATTCTGATGTAGGAGTTTAAT

AGGTAATGGTACTTTTATAATTTCCCCATTACAAAAAATGCTTATATTATCACAAATTTCCTAGGTGGAC

AATATCAGCAAAGCTCTTCATAAG

>Geonoma_pohliana_FJ716264.1

AACGAATTCGCCCTTCACTTATTGAGTGCATCATGGGAAAGGTTGCTGCCCATATGGGAAAGGAGGGAGA

TGCTACTCCTTTTACTGATGTCACAGTAAGTAAATCCATAGAACCTTCATGCAAGCTTTGCCTGAGAAGC

ATGTCACAATTTAACTTGCTATAAATGCTGGAGCTTTTTTGCTTATTCAGTTTAAGAACTCCAAGGCACT

GTATCTGATGTTAAAGAACATCATGGAAAGTTTCTTTTGTTCTGCCCCAGGAGGAAGTAATTTTGCATAT

GTCTAGCTAAAAGACATTGTTGATAGCTTATACAAAAACACATGGTTATTCAATTTGCATTACCATTAAG

GATTTGTACTAGATATGGTTTAATAAATATGTTTGTGAAACACGGGGGTCGTCTAGAACCCTTCAACTTC

ATACCTGGATTTTTTTTTTTTGAGGGGAGCACTTGCCTTTAGAGTTCATATTTTTTTCCTTTGGATTAGT

TAATTATAATCCTGTTATTTGAAGTCTACTTTAAATATCTCTTTTGAAGTTCTTGATATGTAAGTTTACC

GTAACGTGACTTTGAAGAATGACTTTGGACGAGTTTCTTGTAAGTTATTCTGATGATCCTAAACTTGTAT

AAAGTTTCAAGACATACTTTTTTGACCGATCTCTCTCCTAAATTATATAATAGACTATCTCCTTGTAACT

GACCTGTGACTTGGATTTTCCAGTTCAATGATCTATCAGGAGTTCTGTCCTAAATAATGTTTCTAACATT

GATTTCAGATATATTCTGATGTAGGAGTTTAATAGGTAATGGTACTTTTATAATTTCCCCATTACAAAAA

ATGCTTATATTATCACAAATTTCCTAGGTGGACAATATCAGCAAAGCTCTTCATAAGTGTGGATATCAGA

TGCGTGGAAGGGCGAAT

>Geonoma_jussieuana_FJ716262.1

ATTCGCCCTTCACTTATTGAGTGCATCATGGGAAAGGTTGCTGCCCATATGGGAAAGGAGGGAGATGCTA

CTCCTTTTACTGATGTCACAGTAAGTAAATCCATAGAACCTTCATGCAAGCTTTGCCTGAGAAGCATGTC

ACAATTTAACTTGCTATAAATGCTGGAGCTTTTTTGCTTATTCAGTTTAAGAACTCCAAGGCACTGTATC

TGATGTTAAAGAACATCATGGAAAGTTTCTTTTGTTCTGCTCCAGGAGGAAGTAATTTTTGCATATGTCT

AGCTAAAAGACATTGTTGATAGCTTATACAAAAACACATGGTTATTCAATTTGCATTACCATTAAGGATT

TGTACTAGATATGGTTTAATAAATATGTTTGAGAAACACGGGGGTCGTCTAGAACCCTTCAACTTCATAC

CTGGATTTTTTTAAATTTTTTTTTGAGGGGAGCACTTGCCTTTAGAGTTCATATTTTTTCCTTTGGATTA

GTTAATTATAATCCTGTTATTTGAAGTCTACTTTAAATATCTCTTTTGAAGTTCTTGATATGTAAGTTTA

CCGTAACGTGGCTTTGAAGGATGACTTTGGACGAGTGTCTCGTAAGTTATTCTGATGATCCTAAACTTGT

ATAAAGTTTCAAGACATACATTTTTGACCGATCTCTCTCCTAAATTATATAATAGACTATCTCTTGTAAC

TGACCTGTGACTTGGATTTTCCAGTTCAATGATCTATCAGGAGTTCTGTCCTAAATAATGTTTCTAACAT

TGATTTCAGATATATTCTGATGTAGGAGTTTAATAGGTATTGGTACTTTTATAATTTCCCCATTACAAAA

TATGCTTATATTATCACAAATTTCCTAGGTGGACAATATCAGCAAAGCTCTTCATAAGTGTGGATATCAG

ATGCGTGAAGGGCGAATCGTA

>Geonoma_schottiana_FJ716260.1

ATTCGCCCTTCACTTATTGAGTGCATCATGGGAAAGGTTGCTGCCCATATGGGAAAGGAGGGAGATGCTA

CTCCTTTTACTGATGTCACAGTAAGTAAATCCATAGAACCTTCATGCAAGCTTTGCCTGAGAAGCATGTC

ACAATTTAACTTGCTATAAATGCTGGAGCTTTTTTGCTTATTCAGTTTAAGAACTCCAAGGCACTGTATC

TGATGTTAAAGAACATCATGGAAAGTTTCTTTTGTTCTGCCCCAGGAGGAAGTAATTTTGCATATGTCTA

GCTAAAAGACATTGTTGATAGCTTATACAAAAACACATGGTTATTCAATTTGCATTACCATTAAGGATTT

GTACTAGATATGGTTTAATAAATATGTTTGTGAAACACGGGGGTCGTCTAGAACCCTTCAACTTCATACC

TGGATTTTTTTTTTTGAGGGGAGCACTTGCCTTTAGAGTTCATATTTTTTTCCTTTGGATTAGTTAATTA

TAATCCTGTTATTTGAAGTCTACTTTAAATATCTCTTTTGAAGTTCTTGATATGTAAGTTTACCGTAACG

TGACTTTGAAGAATGACTTTGGACGAGTGTCTTGTAAGTTATTCTGATGATCCTAAACTTGTATAAAGTT

TCAAGACATACTTTTTTGACCGATCAATCTCCTAAATTATATAATAGACTATCTCCTTGTAACTGACCTG

TGACTTGGATTTTCCAGTTCAATGATCTATCAGGAGTTCTGTCCTAAATAATGTTTCTAACATTGATTTC

AGATATATTCTGATGTAGGAGTTTAATAGGTAATGGTACTTTTATAATTTCCCCATTACAAAAAATGCTT

ATATTATCACAAATTTCCTAGGTGGACAATATCAGCAAAGCTCTTCATAAGTGTGGATATCAGATGCGTG

AAGGGCGAATCG

>Geonoma_weberbaueri_HM140616.1

TTGCCTTTAGAGTTCATATTTTTTCCTTTGGATTAGTTAATTATAATCCTGTTATTTGAAGTCTACTTTG

AATATCTCTTTTGAAGTTCTTGATATGTAAGTTTACCGTAACGTGGCTTTGAAGAATGACTTTGGACGAG

TGTCTCGTAAGTTATTCCGATGATCCTAAACTTGTATAAAGTTTCAAGACATACATTTTTGACCGATCTC

TCTCCTAAATTATATAATAGACTATCTCTTGTAACTGACCTGTGACTTGGATTTTCCAGTTCAATGATCT

ATCAGGAGTTCTGTCCTAAATAATGTTTCTAACATTGATTTCAGATATACTC

>Geonoma_undata_HM140614.1

TTGCCTTTAGAGTTCATATTTTTTCCTTTGGATTAGTTAATTATAATCCTGTTATTTGAAGTCTACTTTA

AATATCTCTTTTGAAGTTCTTGATATGTAAGTTTACCGTAACGTGGCTTTGAAGAATGACTTTGGACGAG

TGTCTCGTAAGTTATTCTGATGATCCTAAACTTGTATAAAGTTTCAAGACATACATTTTTGACCGATCTC

TCTCCTAAATTATATAATAGACTATCTCTTGTAACTGACCTGTGACTTGGATTTTCCAGTTCAATGATCT

ATCAGGAGTTCTGTCCTAAATAATGTTTCTAACATTGATTTCAGATATATTCTGATGTAGGAGTTTAATA

GGTATTGGTACTTTTATAATTTCCCCATTACAAAATATGCTTATATTATCACAAATTTCCTAGGTGGACA

ATATCAGCAAAGCTCTTCATAAG

>Geonoma_stricta_HM140612.1

TTGCCTTTAGAGTTCATATTTTTTGCTTTGGATTAGTTAATTATAATCCTGTTATTTGAAGTCTACTTTA

AATATCTCTTTTGAAGTTATTGATATGTAAGTTTACCGTAACGTGGCTTTGAAGAATGACTTTGGACGAG

TGTCTTGTAAGTTATTCTGATGATCCTAAACTTGTATAAAGTTTCAAGACATACATTTTTGACCGATCTC

TCTCCTAAATTATATAATAGACTATCTCTTGTAGCTGACCTGTGACTTGGATTTTCCAGTTCAATGATCT

ATCAGGAGTTTTGTCCTAAATAATGTTTCTAACATTGATTTCAGATATATTCTGATGTAGGAGTTTAATA

GGTAATGGTACTTTTATAATTTCCCCATTACAAAAAATGCTTATATTATCACAAATTTCCTAGGTGGACA

ATATCAGCAAAGCTCTTCATAAG

>Geonoma_oldemanii_HM140610.1

TTGTCTTTAGAGTTCATATTTTTTCCTTTGGATTAGTTAATTATAATCCTGTTATTTGAAGTCTACTTTA

AATCTCTCTTTTGAAGTTCTTGATATGCAAGTTTACTGTAACGTGGCTTTGAAGAATGACTTTGGACGAG

TGTCTTGTAAGTTATTCTGATGATCCTAAACTTGTATAAAGTTTCAAGACATACTTTTTTGACCGATCTC

TCTCCTAAATTATATAATAGACTATCTCCTTGTAACTGACCTGTGACTTGGATTTTCCAGTTCAATGATC

TATCAGGAGTTCTGTCCTAAATAATGTTTCTAACATTGATTTCAGATATATTCTGATGTAGGAGTTTAAT

AGGTAATGGTACTTTTATAATTTCCCCATTACAAAAAATGCTTATATTATCACAAATTTCCTAGGTGGAC

AATATCAGCAAAGCTCTTCATAAG

>Geonoma_hugonis_HM140608.1

TTGCCTTTAGAGTTCATATTTTTTCCTTTGGATTAGTTAATTATAATCCTGTTATTTGAAGTCTACTTTA

AATATCTCTTTTGAAGTTCTTGATATGTAAGTTTACCGTAACGTGGCTTTGAAGAATGACTTTGGACGAG

TGTCTTGTAAGTTATTCTGATGATCCTAAACTTGTATAAAGTTTCAAGACATACATTTTTGACCGATCTC

TCTCCTAAATTATATAATAGACTATCTCTTGTAACTGACCTGTGACTTGGTTTTTCCAGTTCAATGATCT

ATCAGGAGTTCTGTCCTAAATAATGTTTCTAACATTGATTTCAGATATATTCTGATGTAGGAGTTTAATA

GGTAATGGTACTTTTATAATTTCCCCATTACAAAAAATGCTTATATTATCACAAATTTCCTAGGTGGACA

ATATCAGCAAAGCTCTTCATAAG

>Geonoma_cuneata_var.procumbens_HM140606.1

TTGCCTTTAGAGTTCATATTTTCTCCTTTGGATTAGTTAATTATAATCCTGTTATTTGAAGTCTGCTTTA

AATATCTCTTTTGAAGTTCTTGATATGTAAGTTTACCGTAACGTGGCTTTGAAGAATGACTTTGGACAAG

TGTCTTGTAAGTTATTCTGATGATCCTAAACTTGTATAAAATTTCAAGACATACATTTTTGACCGATCTC

TCTCCTAAATTATATAATAGACTATCTCTTGTAACTGACCTGTGACTTGGATTTTCCAGTTCAATGATCT

ATCAGGAGTTCTGTCCTAAATAATGTTTCTAACATTGATTTTAGATATATTCTGATGTAGGAGTTTAATA

GGTAATGGTACTTTTATAATTTCCCCATTACAAAAAATGCTTATATTATCACAAATTTCCTAGGTGGACA

ATATCAGCAAAGCTCTTCATAAG

>Geonoma_congesta_HM140604.1

TTGCCTTTAGAGTTCATATTTTTTCCTTTGGATTAGTTAATTATAATCCTGTTATTTGAAGTCTACTTTA

AATCTCTCTTTTGAAGTTCTTGATATGCAAGTTTACTGTAACGTGGCTTTGAAGAATGACTTTGGACGAG

TGTCTTGTAAGTTATTCTGATGATCCTAAACTTGTATAAAGTTTCAAGACATACTTTTTTGACCGATCTC

TCTCCTAAATTATATAATAGACTATCTCCTTGTAACTGACCTGTGACTTGGATTTTCCAGTTCAATGATC

TATCAGGAGTTCTGTCCTAAATAATGTTTCTAACATTGATTTCAGATATATTCTGATGTAGGAGTTTAAT

AGGTAATGGTACTTGTATAATTTCCCCATTACAAAAAATGCTTATATTATCACAAATTTCCTAGGTGGAC

AATATCAGCAAAGCTCTTCATAAG

>Geonoma_brevispatha_FJ716263.1

ATTCGCCCTTCACTTATTGAGTGCATCATGGGAAAGGTTGCTGCCCATATGGGAAAGGAGGGAGATGCTA

CTCCTTTTACTGATGTCACAGTAAGTAAATCCATAGAACCTTCATGCAAGCTTTGCCTGAGAAGCATGTC

ACAGTTTAACTTGCTATAAATGCTGGAGCTTTTTTGCTTATTCAGTTTAAGAACTCCAAGGCACTGTATC

TGATGTTAAAGAACATCATGGAAAGTTTCTTTTGTTCTGCTCCAGGAGGAAGTAATTTTGCATATGTCTA

GCTAAAAGACATTGTTGATAGCTTATACAAAAACACATGGTTATTCAATTTGCATTACCATTAAGGATCT

GTACTAGATATGGTTTAATAAATATGTTTGAGAAACATGGGGGTCGTCTAGAACCCTTCAACTTCATACC

TGGATTTTTTTTTTTTTTTTTTTTGAGGGGAGCACTTGCCTTTAGAGTTCATATTTTTTTCCTTTGGATT

AGTTAATTATAATCCTGTTATTTGAAGTCTACTTTAAATATCTCTTTTGAAGTTCTTGATATGTAAGTTT

ACCGTAACGTGGCTTTGAAGAATGACTTTGGACGAGTGTCTTGTAAGTTATTCTGATGATCCTAAACTTG

TATAAAGTTTCAAGACATACTTTTTTGACCGATCTCTCTCCTAAATTATATAATAGACTATCTCCTTGTA

ACTGACCTGTGACTTGGATTTTCCAGTTCAATGATCTATCAGGAGTTCTGTCCTAAATAATGTTTCTAAC

ATTGATTTCAGATATATTCTGATGTAGGAGTTTAATAGGTAATGGTACTTTTATAATTTCCCCATTACAA

AAAATGCTTATATTATCACAAATTTCCTAGGTGGACAATATCAGCAAAGCTCTTCATAAGTGTGGATATC

AGATGCGTGAAGGGCGAATC

>Geonoma_pauciflora_FJ716261.1

ATTCGCCCTTCACTTATTGAGTGCATCATGGGAAAGGTTGCTGCCCATATGGGAAAGGAGGGAGATGCTA

CTCCTTTTACTGATGTCACAGTAAGTAAATCCATAGAACCTTCATGCAAGCTTTGCCTGAGAAGCATGTC

ACAATTTAACTTGCTATAAATGCTGGAGCTTTTTTGCTTATTCAGTTTAAGAACTCCAAGGCATTGTATC

TGATGTTAAAGAACGTAATGGAAAGTTTCTTTTGTTCTGCTCCAGGAGGAAGTAATTTTGCATATGTCTA

GCTAAAAGACATTGTTGATAGCTTATACAAAAACACATGGTTATTCAATTTGCATTACCATTAAGGATTT

GTACTAGATATGGTTTAATAAATATGTTTGAGAAACACGGGGGTCGTCTAGAACCCTTCAACTTCATACC

TGGATTTTTTTTTTTTTTTTGAGGGGAGCACTTGCCTTTAGAGTTCATATTTTTTTCCTTTGGATTAGTT

AATTATAATCCTGTTATTTGAAGTCTACTTTAAATATCTCTTTTGAAGTTCTTGATATGTAAGTTTACCG

TAACGTGGCTTTGAAGAATGACTTTGGACGAGTGTCTTGTAAGTTATTCTGATGATCCTAAACTTGTATA

AAGTTTCAAGACATACTTTTTTGATCGATCTCTCTCCTAAATTATATAATAGACTATCTCCTTGTAACTT

ACCTGTGACTTGGATTTTCCAGTTCAATGATCTATCAGGAGTTCTGTCCTAAATAATGTTTCTAACATTG

ATTTCAGATATATTCTGATGTAGGAGTTTAATAGGTAATGGTACTTTTATAATTTCCCCATTACAAAAAA

TGCTTATATTATCACAAATTTCCTAGGTGGACAATATCAGCAAAGCTCTTCATAAGTGTGGATATCAGAT

GCGTGAAGGGCGAATC

>Geonoma_pauciflora_FJ716259.1

ATTCGCCCTTCACTTATTGAGTGCATCATGGGAAAGGTTGCTGCCCATATGGGAAAGGAGGGAGATGCTA

CTCCTTTTACTGATGTCACAGTAAGTAAATCCATAGAACCTTCATGCAAGCTTTGCCTGAGAAGCATGTC

ACAATTTAACTTGCTATAAATGCTGGAGCTTTTTTGCTTATTCAGTTTAAGAACTCCAAGGCATTGTATC

TGATGTTAAAGAACATCATGGAAAGTTTCTTTTGTTCTGCTCCAGGAGGAAGTAATTTTGCATATGTCTA

GCTAAAAGTCATTGTTGATAGCTTATACAAAAACACATGGTTATTCAATTTGCATTACCATTAAGGATTT

GTACTAGATATGGTTTAATAAATATGTTTGAGAAACACGGGGGTCGTCTAGAACCCTTCAACTTCATACC

TGGATTTTTTTTTTTTTTTTTTTGAGGGGAGCACTTGCCTTTAGAGTTCATATTTTTTTCCTTTGGATTA

GTTAATTATAATCCTGTTATTTGAAGTCTACTTTAAATATCTCTTTTGAAGTTCTTGATATGTAAGTTTA

CCGTAACGTGGCTTTGAAGAATGACTTTGGACGAGTGTCTTGTAAGTTATTCTGATGATCCTAAACTTGT

ATAAAGTTTCAAGACATAACTTTTTTGACCGATCTCTCTCCTAAATTATATAATAGACTATCTCCTTGTA

ACTGACCTGTGACTTGGATTTTCCAGTTCAATGATCTATCAGGAGTTCTGTCCTAAATAATGTTTCTAAC

ATTGATTTCAGATATATTCTGATGTAGGAGTTTAATAGGTAATGGTACTTTTATAATTTCCCCATTACAA

AAGATGCTTATATTATCACAAATTTCCTAGGTGGACAATATCAGCAAAGCTCTTCATAAGTGTGGATATC

AGATGCG

>Geonoma_deversa_AJ830210.1

TTTTTTTTTTTTGTTTTTTGAGGGGGGCACTTGCCTTTAGAGTTCATATTTTTTTCCTTTGGATTAGTTA

ATTATAATCCTGTTATTTGAAGTCTACTTTAAATATCTCTTTTGAAGTTCTTGATATGTAAGTTTACCGT

AACGTGGCTTTGAAGAATGACTTTGGACGAGTGTCTTGTAAGTTATTCTGATGATCCTAAACTTGTATAA

AGTTTCAAGACATACTTTTTTGACCGATCTCTCTCCTAAATTATATAATAGACTATCTCCTTGTAACTGA

CCTGTGACTTGGATTTTCCAGTTCAATGATCTATCAGGAGTTCTGTCCTAAATAATGTTTCTAACATTGA

TTTCAGATATATTCTGATGTAGGAGTTTAATAGGTAATGGTACTTTTATAATTTCCCCATTACAAAAAAT

GCTTATATTATCACAAATTTCCTAGGTGGACAATATCAGCAAAGCTCTTCATAAGTGT

>Geonoma_longevaginata_AY779362.1

TTGCCTTTAGAGTTCATATTTTTTCCTTTGGATTAGTTAATTATAGTCCTGTTATTTGAAGTCTACTTTA

AATATCTCTTTTGAAGTTCTTGATATGTAAGTTTACCGTAACGTGGCTTAGAAGAATGACTTTGGACGAG

TGTCTTGTAAGTTATTCTGATGATCCTAAACTTGTATAAAGTTTCAAGACATACATTTTTGACCGATCTC

TCTCCTAAATTATATAATAGACTATCTCTTGTAACTGACCTGTGACTTGGATTTTCCAGTTCAATGATCT

ATCAGGAGTTCTGTCCTAAATAATGTTTCTAACATTGATTTCAGATATATTCTGATGTAGGAGTTTAATA

GGTAATGGTACTTTTATAATTTCCCCATTACAAAAAATGCTTATATTATCACAAATTTCCTAGGTGGACA

ATATCAGCAAAGCTCTTCATAAG

>Geonoma_interrupta_AY779360.1

TCCTTTGGATTAGTTAATTTAATCCTGTTATTTGAAGTCTACTTTAAATATCTCTTTTAAAGTTCTTGAT

ATGTAAGTTTACCGTAACGTGGCTTTGAAGAATGACTTTGGACGACTGTCTTGTAAGTTATTCTGATGAT

CCTAAACTTGTATAAAGTTTCAAGACATACTTTTTTGACCGATCTCTCTCCTAAATTATATAATAGATTA

TCTCCTTGTAACTGACCTGTGACTTGGATTTTCCAGTTCAATGATCTATCAGGAGTTCTGTCCTAAATAA

TGTTTCTAACATTGATTTCAGATATATTCTGATGTAGGAGTTTAATAGGTAATGGTACTTTTATAATTTC

CCCATTACAAAAAATGCTTATATTATCACAAATTTCGTAGGTGGACAATATCAGCAAAGCTCTTCATAAG

>Geonoma_triglochin_AY779358.1

TTGCCTTTAGAGTTCATATTTTTTCCTTTGGATTAGTTAATTATAATCCTGTTATTTGAAGTCTACTTTA

AATCTCTCTTTTGAAGTTCTTGATATGCAAGTTTACTGTAACGTGGCTTTGAAGAATGACTTTGGACGAG

TGTCTTGTAAGTTATTCTGATGATCCTAAACTTGTATAAAGTTTCAAGACAATACTTTTTTGACCGATCT

CTCTCCTAAATTATATAATAGACTATCTCCTTGTAACTGACCTGTGACTTGGATTTTCCAGTTCAATGAT

CTATCAGGAGTTCTGTCCTAAATAATGTTTCTAACATTGATTTCAGATATATTCTGATGTATGAGTTTAA

TAGGTAATGGTACTTTTATAATTTCCCCATTACAAAAAATGCTTATATTATCACAAATTTCCTAGGTGGA

CAA

>Geonoma_camana_AY779356.1

TTGCCTTTAGAGTTCATATTTTTTCCTTTGGATTAGTTAAATATAATCCTGTTATTTGAAGTCTACTTTA

AATCTCTCTTTTGAAGTTCTTGATATGCAAGTTTACTGTAACGTGGCTTTGAAGAATGACTTTGGACGAG

TGTCTTGTAAGTTATTCTGATGATCCTAAACTTGTATAAAGTTTCAAGACATACTTTTTTGACCGATCTC

TCTCCTAAATTATATAATAGACTATCTCCTTGTAACTAACCTGTGACTTGGATTTTCCAGTTCAATGATC

TATCAGGAGTTCTGTCCTAAATAATGTTTCTAACATTGATTTCAGATATATTCTGATGTAGGAGTTTAAT

AGGTAATGGTACTTTTATAATTTCTCCATTACAAAAAATACTTATATTATCACAAATTTCCTAGGTGGAC

AATATCAGCAAAGCTCTTCATAAG

>Geonoma_macrostachys_var.macrostachys_AY779354.1

TTGCCTTTAGAGTTCATATTTTTTCCTTTGGATTAGTTAATTATAATCCTGTTATTTGAAGTCTACTTTA

AATCTCTCTTTTGAAGTTCTTGATATGCAAGTTTACTGTAACGTGGCTTTGAAGAATGACTTTGGACGAG

TGTCTTGTAAGTTATTCTGATGATCCTAAACTTGTATAAAGTTTCAAGACATACTTTTTTGACCGATCTC

TCTCCTAAATTATATAATAGACTATCTCCTTGTAACTGACCTGTGACTTGGATTTTCCAGTTCAATGATC

TATCAGGAGTTCTGTCCTAAATAATGTTTCTAACATTGATTTCAGATATATTCTGATGTAGGAGTTTAAT

AGGTAATGGTACTTTTATAATTTCCCCATTACAAAAAATGCTTATATTATCACAAATTCCCTAGGTGGAC

A

>Geonoma_macrostachys_var.acaulis_AY779352.1

TTGCCTTTAGAGTTCATTTTTTTTCCTTTGGATTAGTTAATTATAATCCTGTTATTTGAAGTCTACTTTA

AATCTCTCTTTTGAAGTTCTTGATATGCAAGTTTACTGTAACGTGGCTTTGAAGAATGACTTTGGACGAG

TGTCTTGTAAGTTATTCTGATGATCCTAAACTTGTATAAAGTTTCAAGACATACTTTTTTGACCGATCTC

TCTCCTAAATTATATAATAGACTATCTCCTTGTAACTGACCTGTGACTTGGATTTTCCAGTTCAATGATC

TATCAGGAGTTCTGTCCTAAATAATGTTTCTAACATTGATTTCAGATATATTCTGATGTAGGAGTTTAAT

AGGTAATGGTACTTTTATAATTTCCCCATTACAAAAAATGCTTATATTATCACAAATTCCCTAGGTGGAC

AATATCAGCAAAGCTCTTCATAAG

>Geonoma_leptospadix_AY779350.1

TTGCCTTTAGAGTTCATATTTTTTCCTTTGGATTAGTTAATTATAATCCTGTTATTTGAAGTCTACTTTA

AATATCTCTTTTGAAGTTCTTGATATGTAAGTTTACCGTAACGTGGCTTTGAAGAATGACTTTGGACGAG

TGTCTTGTAAGTTATTCTGATGATCCTAAACTTGTATAAAGTTTCAAGACATACATTTTTGACCGATCTC

TCTCCTAAATTATATAATAAACTATCTCTTGTAACTGACCTGTGACTTGGATTTTCCAGTTCAATGATCT

ATCAGGAGTTCTGTCCTAAATAATGTTTCTAACATTGATTTCAGATATATTCTGATGTAGGAGTTTAATA

GGTAATGGTACTTTTATAATTTCCCCATTACAAAAAAATGCTTATATTATCACAAATTTCCTAGGTGGAC

AATATCAGCAAAGCTCTTCATAAG

>Geonoma_cuneata_AY779348.1

TTGCCTTTAGAGTTCATTTTTTTCCTTTGGATTAGTTAATTATAATCCTGTTATTTGAAGTCTACTTTAA

ATATCTCTTTTGAAGTTCTTGATATGTAAGTTTACCGTAACGTGGCTTTGAAGAATGACTTTGGACAAGT

GTCTTGTAAGTTATTCTGATGATCCTAAACTTGTATAAAGTTTCAAGACATACATTTTTGACCGATCTCT

CTCCTAAATTATATAATAGACTATCTCTTGTAACTGACCTGTGACTTGGATTTTCCAGTTCAATGATCTA

TCAGGAGTTGTGTCCTAAATAATGTTTCTAACATTGATTTCAGATATATTCTGATGTAGGAGTTTAATAG

GTAATGGTACTTTTATAATTTCCCCATTACAAAAAATGCTTATATTATCACAAATTTCCTAGGTGGACAA

TATCAGCAAAGCTCTTCATAAG

>Geonoma_tenuissima_AY779346.1

TTGCCTTTAGAGTTCATATTTTTTCCTTTGGATTAGTTAATTAAAATCCTGTTATTTGAAGTCTACTTTA

AATATCTCTTTTGAAGTTCTTGATATGTAAGTTTACTGTAACGTGGCTTTGAAGAATGACTTTGGACGAG

TGTCTTGTAAGTTATTCTGATGATCGTAAACTTGTATAAAGTTTCAAGACATACATTTTTGACCGATCTC

TCTCCTAAATTATATAATAGACTATCTCTTGTAACTGACCTGTGACTTGGATTTTCCAGTTCAATGATCT

ATCAGGAGTTCTGTCCTAAATAATGTTTCTAACATTGATTTCAGATATATTCTGATGTAGGAGTTTAATA

GGTAATGGTACTTTTATAATTTCCCCATTACAAAAAATGCTTATATTATCACAAATTTCCTAGGTGGACA

ATATCAGCAAAGCTCTTCATAAG

>Geonoma_congesta_AY779344.1

TTGCCTTTAGAGTTCATATTTTTTCCTTTGGATTAGTTAATTATAATCCTGTTATTTGAAGTCTACTTTA

AATCTCTCTTTTGAAGTTCTTGATATGCAAGTTTACTGTAACGTGGCTTTGAAGAATGACTTTGGACGAG

TGTCTTGTAAGTTATTCTGATGATCCTTAACTTGTATAAAGTTTCAAGACATACTTTTTTGACCGATCTC

TCTCCTAAATTATATAATAGACTATCTCCTTGTAACTGACCTGTGACTTGGATTTTCCAGTTCAATGATC

TATCAGGAGTTCTGTCCTAAATAATGTTTCTAACATTGATTTCAGATATATTCTGATGTAGGAGTTTAAT

AGGTAATGGTACTTTTATAATTTCCCCATTACAAAAAATGCTTATATTATCACAAATTTCCTAGG

>Geonoma maxima_AY779342.1

TTGCCTTTAGAGTTCATATTTTTTCCTTTGGATTAGTTAATTATAATCCTGTTATTTGAAGTCTACTTTA

AATCTCTCTTTTGAAGTTCTTGATATGTAAGTTTACTGTAACGTGGCTTTGAAGAATGACTTTGGACGAG

TGTCTTGTAAGTTATTCTGATGATCCTAAACTTGTATAAAGTTTCAAGACATACTTTTTTGACCGATCTC

TCTCCTAAATTATATAATAGACTATCTCCTTGTAACTGACCTGTGACTTGGATTTTCCAGTTCAATGATC

TATCAGGAGTTCTGTCCTAAATAATGTTTCTAACATTGATTTCAGATATATTCTGATGTAGGAGTTTAAT

AGGTAATGGTTCTTTTATAATTTCTCCATTACAAAAAATGCTTATATTATCACCAATTTCCTAGGTGGAC

AATATCAGCAAAGCTCTTCATAAG

>Geonoma_orbignyana_AY779363.1

TTGCCTTTAGAGTTCATATTTTTTCCTTTGGATTAGTTAATTATAATCCTGTTATTTGAAGTCTACTTTG

AATATCTCTTTTGAAGTTCTTGATATGTAAGTTTACCGTAACGTGGCTTTGAAGAATGACTTTGGACGAG

TGTCTCGTAAGTTATTCTGATGATCCTAAACTTGTATAAAGTTTCAAGACATACATTTTTGACCGATCTC

TCTCCTAAATTATATAATAGACTCTCTCTTGTAACTGACCTGTGACTTGGATTTTCCAGTTCAATGATCT

ATCAGGAGTTCTGTCCTAAATAATGTTTCTAACATTGATTTCAGATATATTCTGATGTAGGAGTTTAATA

GGTATTGGTACTTTTATAATTTCCCCATTACAAAATATGCTTATATTATCACAAATTTCTTAGGTGGACA

ATATCAGCAAAGCTCTTCATAAG

>Geonoma_interrupta_AY779361.1

TTTTTTTCCTTTGGATTAGTTAATTATAATCCTGTTATTTGAAGTCTACTTTAAATATCTCTTTTAAAGT

TCTTGATATGTAAGTTTACCGTAACGTGGCTTTGAAGAATGACTTTGGACGAGTGTCTTGTAAGTTATTC

TGATGATCCTAAACTTGTATAAAGTTTCAAGACATACTTTTTTGACCGATCTCTCTCCTAAATTATATAA

TAGATTATCTCCTTGTAACTGACCCGTGACTTGGATTTTCCAGTTCAATGATCTATCAGGAGTTCTGTCC

TAAATAATGTTTCTAACATTGATTTCAGATATATTCTGATGTAGGAGTTTAATAGGTAATGGTACTTTTA

TAATTTCCCCATTACAAAAAATGCTTATATTATCACAAATTTCGTAGGTGGACAATATCAGCAAAGCTCT

TCATAAG

>Geonoma_polyandra_AY779359.1

TTGCCTTTAGAGTTCATATTTTTTCCTTTGGATTAGTTAATTATAATCCTGTTATTTGAAGTCTACTTTA

AATCTCTCTTTTGAAGTTCTTGATATGCAAGTTTACTGTAACGTGGCTTTGAAGAATGACTTTGGACGAG

TGTCTTGTAAGTTATTCTGATGATCCTAAACTTGTATAAAGTTTCAAGACATACTTTTTTGACCGATCTC

TCTCCTAAATTATATAATAGACTATCTCCTTGTAACTGACCTGTGACTTGGATTTTCCAGTTCTATGATC

TATCAGGAGTTCTGTCCTAAATAATGTTTCTAACATTGATTTCAGATATATTCTGATGTAGGAGTTTAAT

AGGTAATGGTACTTTTATAATTTCCCCATTACAAAAAATGCTTATATTATCACAAATTTCCTAGGTGGAC

AATATCAGCAAAGCTCTTCATAAG

>Geonoma_undata_AY779357.1

TTGCCTTTAGAGTTCATATTTTTTCCTTTGGATTAGTTAATTATAATCCTGTTATTTGAAGTCTACTTTA

AATATCTCTTTTGAAGTTCTTGATATGTAAGTTTACCGTAACGTGGCTTTGAAGAATGACTTTGGACGAG

TGTCTCGTAAGTTATTCTGATGATCCTAAACTTGTATAAAGTTTCAAGACATACATTTTTGACCGATCTC

TCTCCTAAATTATATAATAGACTATCTCTTGTAACTGACCTGTGACTTGGATTTTCCAGTTCAATGATCT

ATCAGGAGTTCTGTCCTAAATAATGTTTCTAACATTGATTTCAGATATATTCTGATGTAGGAGTTTAATA

GGTATTGGTACTTTTATAATTTCCCCATTACAAAATATGCTTATATTATCACAAATTTCCTAGGTGGACA

ATATCA

>Geonoma_longepedunculata_AY779355.1

TTGCCTTTAGAGTTCATATTTTTTCCTTTGGATTAGTTAATTATAATCCTGTTATTTGAAGCCTACTTTA

AATATCTCTTTTGAGGTTCTTGATATGTAAGTTTACCGTAACGTGGCTTTGAAGAATGACTTTGGACGAG

TATCTTGTAAGTTATTCTGATGATCCTAAGCTTGTATAAAGTTTCAAGACATACATTTTTGACCGATCTC

TCTCCTAAATTATATAATAGACTATCTCTTGTAACTGACAGTTCAATGATCTATCAGGAGTTCTGTCCTA

AATAATGTTTCTAACATTGATTTCAGATATGTTCTGATGTAGGGGCTTAATAGGTAATGGTACTTTTATA

ATTTCCCCATTACAAAAAAATGCTTATATTATCACAAATTTCCTAGGTGGACAATATCAGCAAAGCTCTT

CATAAG

>Geonoma_arundinacea_AY779353.1

TTGCCTTTAGAGTTCATATTTTTTCCTTTGGATTAGTTAATTATAATCCTGTTATTTGAAGTCTACTTTA

AATATCTCTTTTGAAGTTCTTGATATGTAAGTTTACCGTAACGTGGCTTTGAAGAATGACTTTGGACGAG

TATCTTGTAAGTTATTCTGATGATCCTAAACTTGTATAAAGTTTCAAGACATACATTTTTGACCGATCTC

TCTCCTAAATTATATAATAGACTATCTCTTGTAACTGACAGTTCAATGATCTATCAGGAGTTCTGTCCTA

AATAACGTTTCTAACATTGATTTCAGATATGTTCTGATGTAGGGGTTTAATAGGTAATGGTACTTTTATA

ATTTCCCCATTACAAAAAAATGCTTATATTATCACAAATTTCCTAGGTGGACAATATCAGCAAAGCTCTT

CATAAG

>Geonoma_brongniartii_AY779351.1

TTGCCTTTAGAGTTCATATTTTTTCCTTTGGATTAGTTAATTATAATCCTGTTATTTGAAGTCTACTTTA

AATGTCTCTTTTGAAGTTCTTGATATGTAAGTTTACCGTAACGTGGCTTTGAAGAATGACTTTGGACGAG

TGTCTTGTAAGTTATTCTGATGATCCTAAACTTGTATAAAGTTTCAAGACATACATTTTTGACCGATCTC

TCTCCTAAATTATATAATAGACTATCTCTTGTAACTGACCTGTGACTTGGATTTTCCAGTTCAATGATCT

ATCAGGAGTTCTGTCCTAAATAATGTTTCTAACATTGATTTCAGATATATTCTGATGTAGGAGTTTAATA

GGTAATGGTACTTTTATAATTTCCCCATTACAAAAAATGCTTATATTATCACAAATTTCCTAGGTGGACA

ATATCAGCAAAGCTCTTCATAAG

>Geonoma_stricta_AY779349.1

TTGCCTTTAGAGTTCATATTTTTTCCTTTGGATTAGTTAATTATAATCTTGTTATTTGAAGTCTACTTTA

AATATCTCTTTTGAAGTTCTTGATATGTAAGTTTACCGTAACGTGGCTTTGAAGAATGACTTTGGACGAG

TGTCTTGTAAGTTATTCTGATGATCCTAAACTTGTATAAAGTTTCAAGACATACATTTTTGACCGATCTC

TCTCCTAAATTATATAATAGACTATCTCTTGTAACTGACCCGTGACTTGGATTTTCCAGTTCAATGATCT

ATCAGGAGTTCTGTCCTAAATAATGTTTCTAACATTGATTTCAGATGTATTCTGATGTAGGAGTTTAATA

GGTAATGGTACTTTTATAATTTCCCCATTACAAAAAATGCTTATATTATCACAAATTTCCTAGGTGGACA

ATATCAGCAAAGCTCTTCATAAG

>Geonoma_poeppigiana_AY779347.1

TTGCCTTTAGAGTTCATATTTTTTCCTTTGGATTAGTTAATTATAATCTTGTTATTTGAAGTCTACTTTA

AATTTCTCTTTTGAAGTTCTTGATATGTAAGCTTACCGTAACGTGGCTTTGAAGAATGACTTTGGACGAG

TGTCTTGTAAGTTATTCTGATGATCCTAAACTTGTATAAAGTTTCAAGACATACATTTTTGACCGATCTC

TCTCCTAAATTATATAATAGACTATCTCTTGTAACTGACCTGTGACTTGGATTTTCCAGTTCAATGATCT

ATCAGGAGTTCTGTCCTAAATAATGTTTCTAACATTGATTTCAGATATATTCTGATGTAGGAGTTTAATA

GGTAATGGTACTTTTATAATTTCCCCATTACAAAAAATGCTTATATTATCACAAATTTCCTAGGTGGACA

ATATCAGCAAAGCTCTTCATAAG

>Geonoma_congesta_AY779345.1

TTGCCTTTAGAGTTCATATTTTTTCCTTTGGATTAGTTAATTATAATCCTGTTATTTGAAGTCTACTTTA

AATCTCTCTTTTGAAGTTCTTGATATGCAAGTTTACTGTAACGTGGCTTTGAAGAATGACTTTGGACGAG

TGTCTTGTAAGTTATTCTGATGATCCTTAACTTGTATAAAGTTTCAAGACATACTTTTTTGACCGATCTC

TCTCCTAAATTATATAATAGACTATCTCCTTGTAACTGACCTGTGACTTGGATTTTCCAGTTCAATGATC

TATCAGGAGTTCTGTCCTAAATAATGTTTCTAACATTGATTTCAGATATATTCTGATGTAGGAGTTTAAT

AGGTAATGGTACTTTTATAATTTCCCCATTACAAAAAATGCTTATATTATCACAAATTTCCTAGGTGGAC

AATATCAGCAAAGCTCTTCATAAG

>Geonoma_jussieuana_AY779343.1

TTGCCTTTAGAGTTCATATTTTTTCCTTTGGATTAGTTAATTATAATCCTGTTATTTGAAGTCTACTTTA

AATATCTCTTTTGAAGTTCTTGATATGTAAGTTTACCTTAACGTGGCTTTGAAGAATGACTTTGGACGAG

TGCCTCGTAAGTTATTCTGATGATCCTAAACTTGTATAAAGTTTCAAGACATACATTTTTGACCGATCTC

TCTCCTAAATTATATAATAGACTATCTCTTGTAACTGACCTGTGACTTGGATTTTCCAGTTCAATGATCT

ATCAGGAGTTCTGTCCTAAATAATGTTTCTAACATTGATTTCAGATATATTCTGATGTAGGAGTTTAATA

GGTATTGGTACTTTTATAATTTCCCCATTACAAAATATGCTTATATTATCGCAAATTTCCTAGGTGGACA

ATATCAGCAAAGCTCTTCATAAG

>Geonoma_maxima_var.chelidonura_AY779341.1

TTGCCTTTAGAGTTCATATTTTTTCCTTTGGATTAGTTAATTATAATCCTGTTATTTGAAGTCTACTTTA

AATCTCTCTTTTGAAGCTCTTGATATGTAAGTTTACTGTAACGTGGCTTTGAAGAATGACTTTGGACGAG

TGTCTTGTAAGTTATTCTGATGATCCTAAACTTGTATAAAGTTTCAAGACATACTTTTTTGACCGATCTC

TCTCCTAAATTATATAATAGACTATCTCCTTGTAACTGACCTGTGACTTGGATTTTCCAGTTCAATGATC

TATCAGGAGTTCTGTCCCAAATAATGTTTCTAACATTGATTTCAGATATATTCTGATGTAGGAGTTTAAT

AGGTAATGGTACTTTTATAATTTCCCCATTACAAAAAATGCTTATATTATCACAAATTTCCTAGGTGGAC

AATATCAGCAAAGCTCTTCATAAG

>Hemithrinax_rivularis_EU215511.1

TTATTGAGTGCATCATGGGGAAGGTTGCTGCCCATATGGGAAAAGAGGGGGATGCTACTCCCTTTACTGA

TGTCACAGTAAGTAAATCCATAAAACCTTCATGTGAGCTTTACCTGTGAAGCATGTCACAATTTAACTTG

CTATAGATGCTGGAGCTTGTTTGCTTATTCAGTTTAAGAATTCCGAGGCACTGTGTCTGATGTTAAAGAA

CATTAAGGAAGGTTTCTTTTGTTCTGCTTCAGGAGGAAGTAGTTTTTGCAGATGTCTAGCTAAAAGACAT

TGTTGATAGCTTATACAAAACAGAAGGTTATTCAATTTGCATTACCATTAAGGATTTGTACTAGATATGG

TTCAATAAATATGTTAAATACGTTTGAGAACTGGAGCTTTTGCCTTTGGAGTTCATTTTTTCCTCTGGTT

TGCTTAATTCTAATCCTGTTATTTGAGTCTACCTTGAATATCTCTTTTGAAGTTCTTGAGATGTAAGTTT

ACCCTGTAGACAGAAACGTGGCTTTGAAGAATGACCTTGGATGAGTGTCCTGTAAGTTATTGTGAGGATC

CTAAACTTGTACAAGTTTCAAGACATACTGTCTTGACTAAGGTCTCTTCTAAATTATATAGTAGACTATC

TCCTCGTAACCGGCCTGTGACTTGGATTTTCCAATTCGAGTTCTATCCTAAATAATGTTTCTAACATTGG

TTTCAGATGTATTCTGATGTAGGAGTTTAATATGTAATTGTTCTTTTGTAATTGCCCCATTACAAAGAAC

ACTTATTTTATCAGGTGGACAATATAAGCAAAGCTCTTCATAAGTGTGGATATCAGA

>Hemithrinax_compacta_EU215498.1

TTATTGAGTGCATCATGGGAAAGGTTGCTGCCCATATGGGAAAAGAGGGAGATGCTACTCCTTTTACTGA

TGTCACAGTAAGTAAATCCATAAAACCTTCATGTGAGCTTTACCTGTGAAGCATGTCACAATTTAACTTG

CTATAGATGCTGGAGCTTGTTTGCTTATTCAGTTTAAGAATTCCGAGGCACTGTGTCTGATGTTAAAGAA

CATTAAGGAAAGTTTCTTTTGTTCTGCTTCAGGAGGAAGTAGTTTTTGCAGATGTCTAGCTAAAAGACAT

TGTTGATAGCTTATACAAAACAGAAGGTTATTCAATTTGCATTACCATTAAGGATTTGTACTAGATATGG

TTCAATAAATATGTTAAATATGTTTGAGAACTGGAGCTTTTGCCTTTGGAGTTCATTTTTTCCTCTGGTT

TACTTAATTCTAATCCTGTTATTTGAGTCTACCTTGAATATCTCTTTTGAAGTTCTTGAGATGTAAGTTT

ACCCTGTAGACAGAAATGTGGCTTTGAAGAATGACCTTGGATGAGTGTCTTGTAAGTTATTGTGAGGATC

CTAAACTTGTACAAGTTTCAAGACATACTGTCTTGACTAAGGTCTCTTCTAAATTATATAGTAGACTATC

TCCTCGTAACCGGCCTGTGACTTGGATTTTCCAATTCGAGTTCTATCCTAAATAATGTTTCTAACATTGA

TTTCAGATGTATTCTGATGTAGGAGTTTAATATGTAATTGTTCTTTTGTAATTGCCCCATTACAAAAAAC

ACTTATTTTATCAGGTGGACAATATAAGCAAAGCTCTTCATAAGTGTGGATATCAGA

>Hyospathe_macrorachis_AJ830169.1

CCCCTTTTACTGATGCCCAGTAAGTAAATCCATAGAACCTTCATGCNNNCTTTACCTGAGAAGCATGCCA

CAATTTAACTTGCCATAAATGCTGGATCTTTTTTGCTTATTCAGTTTAAGAGTTCCAAGGCACTCTATCT

GATGTTAAAGAACATTATGGACAGTTTTTTTTGTTCTGCACCAGGAGCAAGTAATTTGTGCATATGTCTA

AGCTAAAAGACATTGTTCATAGCTTATACAAAAACACATGGTTTTTCAATTTGCATTCCCATTAAGGATT

TGTACTAGATATGGTTTAATAAATATGTTTGAGAAACACGGGGGTTCATCTAGAACCTTTCTACTTCATA

CCTGGATTTTTTTTTTTTTGAGGGGAGCATTTGCCTTTGGAGTTCATATTTTCCTCTGGATTAGTTAATT

ATAATCCTGTTATTTGAAGTCTACTTTAAATCTCTCTTTTGAAGTTCTTGATATGTAAGTTTACCCTGTA

GATAGAAACGTGGCTTTGAAGAATGACCTTGGACGAGTGTCTTGTAAGTTATTCTGATGATCCTAAACTT

GTATAAAGTTTCAAGACATACTATTTTGACTGAGGTCTCTCCTAAATTATATAATAGACTATCTCTTTGT

AACTGACCTGTGACTTGGATTTTCCAGTTCAATGATCTATCAGGAGTTCCATCCTAAATAATGTTTCTAA

CTTTGATTTCAGATATATTCTGATGTAGGAGTTTAATACGNAATTGTACTTTTGTAATTTCCCC

>Iriartea_deltoidea_EF491149.1

CTACTCCTTTTACTGATGTCACAGTAAGTAAATCCATAGAACCTTCATGCAGGCTTTACCTGAGAAGCAT

GTCACAATTTAACTTGCTATAAATGCTGGAGCCTTTTTGCTTATTCAGTTTAAGAATTCTGAGGCACTGT

ATCTGATGTTAAAGAACGTTAAGGAAAGTTTCTTTTGTTCTGCTCCAGGAGGAAGTAATTTTTGCATGTC

TAGCTAAAAGACATTGTTGATAGCTTATACAAAAACACATGATTATTCAATTTGCATTACCATTAAGGAT

TTGTACTAGATATGGTTTAATAAATGTGTTTGAGAACTGTTACCCCCCCCCCCTCGGGTCTAGAACCTTC

CAACTTCATACCTGGATTTTATATTTTTGGAGGGGAGCTTTTGCCTTTGGAGTTCATATTTTCCTCTGAA

TTAGCTAATTATAATCCTGTTATTTGTAGTCTACTTTGAATATCTCTTTTGAAGTTCTTGATATGTAAGT

TTACCATGTAGACAGAAACATGGCTTTGAAGAATGACCTTGGACGAGTGTCTTGTAAGTTATTCTGATGA

TCCTACACTTGTATAAGTTTCAAGACATTATTTTGACTAAGGTCTCTCCTAAATTATATTGTAGACTATC

TCCTCGTAACTGACCTGTGACTTGGATTTTCCAATTCAATGACCTATCAGGAGTCCTATCCTAAATAATG

TTTCTAACATTGATTTCAGATATATTCTGATGTAGGAGTTTAATATGTAATTGTACTTTTGTAATTGTCC

CAT

>Itaya_amicorum_EU215485.1

TTATTGAGTGCATCATGGGAAAGGTTGCTGCCCATATGGGAAAGGAGGGAGATGCTACTCCTTTTACTGA

TGTCACAGTAAGTAGGTCCATAAAACCTTCATGTGAGCTTTACCTGTGAAGCATGTCACAATTTAACTTG

CTATAGATGCTTTAGCTTGTTTGCTTATTCAGTTTAAGAACTCTGAGGCACTGTGCCTGATGTTAAAGAA

CATTAAGGAAAGTTTCTTTTGTTCTGCTCCAGGAGGAAGTAGTTTTTGCATGTCTTAACTACAAGACATT

GTTGATAGCTTATACAAAGACGGAAGGTTATTCAATTTGCATTACCATTAAGGATTTATACTAGATATGG

GAGCTTTTGCCTTTGGGGTTCATTTTTTCCTCTGGTTTAGTTATTTATAATCCTGTTATTTGACTCTACT

TTGAATATCTCTTTTGAAGTTCTTGAGATGTAAGTTTACCCTGTAGACAGAAACGTGGCTTTGAAGAATG

ACCTTGGACAAGTGTCTTGTAAGTTATTCTGATGATCCTAAACTTGTACAAGTTTCAAGACATACCATCT

TGACTAAGGTCTCTTCTAAATTGTATAGTAGACTATCTCCTTGTAACTGTCCTGTGACTTGGATTTTCCA

ATTCAATGATCTGTCAGGAGTTCTATCCTAAATAATGTTTCTAGCATTGATTTCAGATGTATTCTGATGT

AGGAGTTTAATATGTAATTGTTCTTTTGTAATTGCCCCATTACAAAAAATACTTATTATCAGGTGGACAA

TATCAGCAAAGCTCTACATAAGTGTGGATATCAGA

>Juania_australis_EF128420.1

GTAAATCCATAGAAGCTTCATGCAAGCTTTACCTGAGAAGCATGTCACAATTTAATTTGCTATAATGCTG

GAGCTTTTTTTGCTTATTCAGTTTTAGAATWTCGAGGCACTGTATCTGATGTTAAAGAACACCAAGGAAG

TTTTCATTCATTCTGCTCCAGGAGGGAGTAACTTTTGCATATATCTAGCTAAAAGATATTGTGCATAGCT

TATACAAAAACACAAGGTTATTGAATTTGCATTATGCCTACCTCCCCCTCCCATGCGTGGTCTTTCAACT

TCATACCTGGATTTGTTTTTTTTTATGGGGAGCTTTTGCCTTTGGAGTTCATATTTTCCTCTGGATCAGT

TAATTATAATCCTGTTATTTGAAGTCTACTTTTGATATCTCTGTTGAAGTTGTTGAGATGTAAGTTTACC

CTGTAGACAGAAACGTGGCTTTGAAGAATGACCTCGGACGAGTGTCTTGTAAGCTTTCTGATGATCCTAA

ACTTGTATAAGTTTAGGACATACTATTTTGACTAAGGTCTCTCCAAAATTATATAGTGGACTATCTCCTT

GTAACTAGCCTGTGACTTAGATTTTCCAATTCAATGATCTATCAGGAGTTCTATCCTAAATAATGTTTCT

AACATTGATTTCGGATATATTCTGATGTAGGAGTTTAATATGTAATTGTACTTTTGTAATTGCCCCA

>Leopoldinia_pulchra_AY543102.1

AGATGCTACTCCTTTTACTGATGTCACAGTAAGCAAATCCATAGAACCTTCATGCAAGCTTTACCTGAGA

AGCATGTCACAATTTAACTTGCTATTAAATGCTGGAGCTTTTTTGTTCAGTTTAAGAATTCCAAGGCACT

GTATCTGATGTTAAAAAAACATTATGAAAAGTTTCTTCTGTTCTGCTTCAGGAGGAAGTAATTTTTGTAT

ATGTCTAGCTAAAAGACATTGTTGATAGCTTATACGAAAACACATGGTTATTCAATTTGCATTACCTTTA

AGGATTTGTACTATATATGGTTTAATAAATATGTTTGAGACACACAAGGGGGTCGTCTAGAACACTTCAA

CTTCATACCTGGATTCTTTTTTTTTTTTTTTTTTTCCTTTTTTTTTTTGAGGGGACCATTTGCCTTTGGA

GTTTATATTTTCCCCTGGATTAGTTAATCATAATTCTATTTTTGAAGTCCACTTTAAATATCTCTTTTGA

AGTTCTTGATATGTAAGTTTACCCTGTAGACAGAAACGTAGCTTTGAAGAATGACCTTGGACGAGTGTCT

TGTAAGTTATTCTGGTGATCCTAAACTTGTATAAAGTTTCAAGACGTTATTTTGACTGAGGTCTCTCCTA

AATTATATAATAGACGTCTCCTTGTAACTGACCTGTGACTTGGATTTTCCAGTTCAATGATCTCTCAGGA

CTTCCATCCTAAATAATGTTTCTAACATCGATTTCAGATATATTCTGATGTAGGAGTTTAATACGTTTTT

GTACTTTTGTAATTTCCCCATTACAAAAAATGCTTATATTATCACAAATTTTCTAGGTGGACAATATCA

>Lytocaryum_weddellianum_JQ821999.1

GAAAGGAGGGAGATGCTACTCCTTTTACTGATGTCACAGTAAGTAAATCCATAGAACCTTCATGCAAACT

TTACCTGAGAAGCATGTCACAATTTAACTTGCTATAAGTGCTGGAGCTTGTTTGCTTATTCAGTCTAAGA

ATTCCAAGTCACTGTATCTGATGTTAAAGAACATCATGGAAAATTTCTTTGTTCTGCTCGAGGAGGAAGT

AAGTTTTGCATGTGTCTGGCTAAATGACGTTGTTAGCTCATACAAAAATGCATGGTTATTCAATTTGCAT

TACCATTAAGGATTTGTACCAGATATGGTTTAATTAATATGTTTGAGTTGCATTACCATTAAGGATTTGT

ACTAGATATGGTTTAATAAATATGGTTGAGTTGCATTACCCAAGATGCAGGGGGGTGTTGTATAAAACCC

TTCAACTTCATACCTGAATTTTTTTTTTTTTTTTTTGGTGGAGGAGGGGGGGTGGGGGAGCTTTTGCCTT

TGGAGTTCATATTTTCCTCTGGATTAGTTTATTATAATCCTGTTATTTGAAGTCTACTTTGAATATCTCT

TTTGAAGTTGATGATATGTTAGTTTACCCTGTAGACAGAAACATGGCTCTGAAGATTGACCTTGGACGAG

TGTCTTGAAAGTTATTCTGATGATCCTAAACTTGTATAAGTTTCAAGACGTACTATTTTGACTAAGGTCT

CTCCTAAATTATGTCATTGACTATCTCCTTGTAATTGACCTGTGGCTTGGATTTTCCAATTCAATGATCT

ATCAGGAGTTCTATCCTAAATAATGTTTCTAATATTGATTTCAGATATATTCTGATGTAGGAGTTTAATA

TGTAATTGTACTTTTGTAGTTGCTCCATTACAAAAATGCTTATATTATCACAAATTTCCTAGGTGGA

>Manicaria_saccifera_AJ830173.1

GAGATGCTACTCCTTTTACTGATGTCACAGTAAGTAAATCCATAGAACCTGCATGCAAGCTTTACCTGAG

AAGCATGTCACAATTTAACTTGCTATAAATGCTGGAACTTTTTAGCTTATTCAGTTTAAGAATTCCAAGG

CACCGTATCTGATGTTAAAGAACATTAAGGAAAGGTTCTTTTGTTCTGCTTCAGGAGGAAGTAATTGTTG

CATATGTGTAGCTAAAGGGCATTGTTGATAGCTAATACAAAAACACATGGTTATTCAATTTGCATTACCA

TTAAGGATTTGTACTAGATATGGTTTGATAAATATGTTTGAGAACTGGCGGTCGTCTAGAACCCTTCAAC

TTCATACCTGGATTTTATTTTTTTTGAGGGGAGCTTTTGCCTTTGGAGTTCATATTTTCGCCTGGATTAG

TTAATTATAATCTTGTTATTTGAAGTCTACTTTGAATATCTCTTTTGGAGTTCTTGATATGTAAGTTTAC

CCAGTAGACAGAAATGTGGCTTTGAAGAATGACCTTGGACGAGCCTCTTGTAAGTTATTCTGATGATCCT

GAACTTGTATAAGTTTCAAGACATACTATTTTGACTTAGGTCTTTCCTAAATTATATAATAGACTATCTC

CTTGTAACTGACCTGTGACTTGGATTTTCCAATTCAATGATCTATCAGGAGTTCTATCCTAAATAATGTT

TCTAACGTTGATTTCAGATATATTCTGATGTAGGAGTTTAATATGTAATTGTACTTCTTGTAATTTCCCC

ATTACAAAGAAAGCTTATATTATCACAAATTTCCTAGGTGGACACATATC

>Manicaria_saccifera_AY779374.1

TTGCCTTTAGAGTTCATATTTTCGCCTGGATTAGTTAATTATAATCTTGTTATTTGAAGTCTACTTTGAA

TATCTCTTTTGGAGTTCTTGATATGTAAGTTTACCCAGTAGACAGAAATGTGGCTTTGAAGAATGACCTT

GGACGAGCCTCTTGTAAGTTATTCTGATGATCCTGAACTTGTATAAGTTTCAAGACATCCTATTTTGACT

TAGGTCTTTCCTAAATTATATAATAGACTATCTCCTTGTAACTGACCTGTGACTTGGATTTTCCAATTCA

ATGATCTATCAGGAGTTCTATCCTAAATAATGTTTCTAACGTTGATTTCAGATATATTCTGATGTAGGAG

TTTAATATGTAATTGTACTTTTGTAATTTCCCCATTACAAAAAAAGCTTATATTATCACAAATTTCCTAG

GTGGACAATATCAGCAAAGCTCTTCATAAG

>Manicaria_saccifera_AY543103.1

GAGATGCTACTCCTTTTACTGATGTCACAGTAAGTAAATCCATAGAACCTGCATGCAAGCTTTACCTGAG

AAGCATGTCACAATTTAACTTGCTATAAATGCTGGAACTTTTTAGCTTATTCAGTTTAAGAATTCCAAGG

CACCGTATCTGATGTTAAAGAACATTAAGGAAAGGTTCTTTTGTTCTGCTTCAGGAGGAAGTAATTGTTG

CATATGTGTAGCTAAAGGGCATTGTTGATAGCTAATACAAAAACACATGGTTATTCAATTTGCATTACCA

TTAAGGATTTGTACTAGATATGGTTTGATAAATATGTTTGAGAACTGGCGGTCGTCTAGAACCCTTCAAC

TTCATACCTGGATTTTATTTTTTTTGAGGGGAGCTTTTGCCTTTGGAGTTCATATTTTCGCCTGGATTAG

TTAATTATAATCTTGTTATTTGAAGTCTACTTTGAATATCTCTTTTGGAGTTCTTGATATGTAAGTTTAC

CCAGTAGACAGAAATGTGGCTTTGAAGAATGACCTTGGACGAGCCTCTTGTAAGTTATTCTGATGATCCT

GAACTTGTATAAGTTTCAAGACATACTATTTTGACTTAGGTCTTTCCTAAATTATATAATAGACTATCTC

CTTGTAACTGACCTGTGACTTGGATTTTCCAATTCAATGATCTATCAGGAGTTCTATCCTAAATAATGTT

TCTAACGTTGATTTCAGATATATTCTGATGTAGGAGTTTAATATGTAATTGTACTTCTTGTAATTTCCCC

ATTACAAAGAAAGCTTATATTATCACAAATTTCCTAGGTGGACACATATC

>Neonicholsonia_watsonii_AJ830172.1

CTCCTTTTACTGATGCCACAGTAAGTAAATCCATAAAACCTTCATGCNAGCTTTACCTGAGAAGCATGTC

ACAATTTAACTTGCTATAGATGCTGGATCTTTTTTGCTTATTCAGTTTAAGAATTTCAAGGCACTCTATC

TGATGTTAAAGAACATTATGGAAAGTTTCTTTTGTTCTGCTCCAGGAGGAAGTAATTTTTGCATGTCTAG

ATAAAAGACATTGTTCATAGCTTATACAAAAACACATGGTTATTCAATTTGCATTACCATTAAGGATTTG

TACTAGATATGGTTTAATAAATATGTTTGAGCAACACAGGAGGGCCATCTAGAACCCTTCAACTTCATAC

CTGGATTTTATTTATTTATTTTTTGGAGGGGAGCATTTGCCTTTGGAGTTCATATTTTCCTCTGGATTAG

TTAATTATAATCCTGTTATTTGAAGTCTACTTTAAATATCTCTTTTGAAATTCTTGATATGTAAGTTTAC

CCTGTAGACAGAAACGTGACTTTGAAGAATGACCTTGGACGAGTGTCTTGTAAGTTATTCTGATGATCGT

AAACTTGTATAAAGTTTCAAGACATACTATTTTGACTGAGGTCTCTCCTAAATTATGTGATAGACTATCT

CCTTGTAACTGACCTGTGACTTGGATTTTCCAGTTCAATGATCTGTCAGGAGTTCCATCCTAAATAATGT

TTCTAACTTTGATTTTAGATATATTCTGATTTAGGAGTTTAGTACGCAATCGTGCTTTTGTAATTTCCCC

ATTACAAANNAATGCTTATATTATCACAAA

>Pholidostachys_kalbreyeri_JQ417533.1

TTGCCTTGAGAGTTCATATTTTCCTTTGCATTAGTTAATTATAATCTTGTTATTTGAAGTCTAATTTAAA

TATCTCTTTTGAAGTTCTTGATATGTAAGTTTACCGTAATGTGGCTTTGAAGAATGACTTTGGACTAGTG

CCTTGTAAGTTATTCGGATGATCCTAAACTTGTATAAAGTTTCAAGACATACTATTTTGACCGATCTCTC

TCCTAAATTATATAATAGACTATCTCCTTGTAACTGACCTGTGACTTGGATTTTCCAGTTCAATGATCTA

TCAGGAGTTCTGTCCTAAATAATGTTTCTAACATTGATTTCAGATATATTCTGATGTAGGAGTTTAATAG

GTAATGGTACTTTTGTTATTTCCCCATTACAAAAAATGCTTATATTATCACAATTTTCCTAGGTGGACAA

TATCAGCAAAGCTCTTCACAAG

>Pholidostachys_synanthera_JQ417534.1

TCATATTTTCCTTTGCATTAGTTAATTATAATCTTGTTATTTGAAGTCTAATTTAAATATCTCTTTTGAA

GTTCTTGATATGTAAGTTTACCGTAACGTGGCTTTGAAGAATGACTTTGGACTAGTGTCTTGTAAGTTAT

TCTGATGATCCTAAACTTGTATAAAGTTTCAAGACATACTATTTTGACCGATCTCTCTCCTAAATTATAT

AATAGACTATCTCCTTGTAACTGACCTGTGACTTGGATTTTCCAGTTCAATGATCTATCAGGAGTTCTGT

CCTAAATAATGTTTCTAACATTGATTTCAGATATATTCTGATGTAGGAGTTTAATAGGTAATGGTACTTT

TGTTATTTCCCCATTACAAAAAATGCTTATATTATCACAATTTTCCTAGGTGGACAATATCAGCAAAGCT

CTTCACAAG

>Pholidostachys_pulchra_AJ830211.1

TTTCTTTCTTCTTTTTTTCTTTTCTATTTTTTTGAGGGGAGCACTTGCCTTGAGAGTTCATATTTTCCTT

TGCATTAGTTAATTATAATCTTGTTATTTGAAGTCTAATTTAAATATCTCTTTTGAAGTTCTTGATATGT

AAGTTTACCGTAATGTGGCTTTGAAGAATGACTTTGGACTAGTGTCTTGTAAGTTATTCGGATGATCCTA

AACTTGTATAAAGTTTCAAGACATACTATTTTGACCGATCTCTCTCCTAAATTATATAATAGACTATCTC

CTTGTAACTGACCTGTGACTTGGATTTTCCAGTTCAATGATCTATCAGGAGTTCTGTCCTAAATAATGTT

TCTAACATTGATTTCAGATATATTCTGATGTAGGAGTTTAATAGGTAATGGTACTTTTGTTATTTCCCCA

TTACAAAAAATGCTTATATTATCACAATTTTCCTAGGTGGACAATATCAGCAAAGCTCTTCACAAGTGT

>Pholidostachys_dactyloides_AY779368.1

TTGCCTTGAGAGTTCATATTTTCCTTTGCATTAGTTAATTATAATCTTGTTATTTGAAGTCTAATTTAAA

TATCTCTTTTGAAGTTCTTGATATGTAAGTTTACCGTAATGTGGCTTTGAAGAATGACTTTGGACTAGTG

TCTTGTAAGTTATTCGGATGATCCTAAACTTGTATAAAGTTTCAAGACATACTATTTTGACCGATCTCTC

TCCTAAATTATATAATAGACTATCTCCTTGTAACTGACCTGTGACTTGGATTTTCCAGTTCAATGATCTA

TCAGGAGTTCTGTCCTAAATAATGTTTCTAACATTGATTTCAGATATATTCTGATGTAGGAGTTTAATAG

GTAATGGTACTTTTGTTATTTCCCCATTACAAAAAATGCTTATATTATCACAATTTTCCTAGGTGGACAA

TATCAGCAAAGCTCTTCACAAG

>Pholidostachys_synanthera_AY779369.1

TCATATTTTCCTTTGCATTAGTTAATTATAATCTTGTTATTTGAAGTCTAATTTAAATATCTCTTTTGAA

GTTCTTGATATGTAAGTTTACCGTAACGTGGCTTTGAAGAATGACTTTGGACTAGTGTCTTGTAAGTTAT

TCTGATGATCCTAAACTTGTATAAAGTTTCAAGACATACTATTTTGACCGATCTCTCTCCTAAATTATAT

AATAGACTATCTCCTTGTAACTGACCTGTGACTTGGATTTTCCAGTTCAATGATCTATCAGGAGTTCTGT

CCTAAATAATGTTTCTAACATTGATTTCAGATATATTCTGATGTAGGAGTTTAATAGGTAATGGTACTTT

TGTTATTTCCCCATTACAAAAAATGCTTATATTATCACAATTTTCCTAGGTGGACAATATCAGCAAAGCT

CTTCACAAG

>Phytelephas_macrocarpa_AJ830179.1

CATCATGGGAAAGGTTGCTGCCCATATGGGAAAGGAGGGAGATGCTACTCCTTTTACTGATGTCACAGTA

AGTAAATCCATAGAAGCTTCATGCAAGCTTTACCTGAGAAGCATGTCACAATTTAATTTGCTATAAATGC

TGGTGCTTTTTTTGCTTATTCAGTTTTAGAATTTCGAGGCACTGTATCTGATGTTAAAGAACACTAAGGA

AGTTTTCTTTCATTCTGCTCCAGGAGGAAGTAATTTTTTGCATTTGTCTAACTAAAAGATATTTTGGATA

GCTTATACAAAAACACAAGGTTATTGAACTTGCATTACACCTACCCCCCGGTGGGGGCCCATGCAGTGTC

TAGAACCTTTCAACTTCATACCTGGATTTGTGTTTTTTATGTGGAGCTTTTGCTTGTAAGCTATTCTGAT

GGTGCTAAACTTGTATAGGTTTCAGGACATACTATTTTGACTAAGGTCTCTCCAAAATTATATAGTGGAC

CATCTCCTTGTAACTGGCCTGTGACTTAGATTTTCCAATGCAATGATCTATCAGGAGTTCTATCCTAAAT

AATGTTTCTAACATTGATTTCAGATGCATTCTGATGTAGGAGTTTAATATGTAATTGTACTTTTGTAATT

GCCCCATTACAAAAAATACTTATATTATCACAAATTTCCTAGGTGGACAATATCAGCAAAGCTCTTCATA

ATGTGA

>Phytelephas_aequatorialis_AJ830178.1

CATCATGGGAAAGGTTGCTGCCCATATGGAAAGGAGGGAGATGCTACTCCTTTTACTGATGTCACAGTAA

GTAAATCCATAGAAGCTTCATGCAAGCTTTACCTGAGAAGCATGTCACAATTTAATTTGCTATAAATGCT

GGTGCTTTTTTGGCTTATTCAGTTTTAGAATTTCGAGGCACTGTATCTGATGTTAAAGAACACTAAGGAA

GTTTTCTTTCATTCTGCTCCAGGAGGAAGTAATTTTTTGCATTTGTCTAACTAAAAGATATTTTGGATAG

CTTATACAAAAACACAAGGTTATTGAACTTGCATTACACCTACCCCCCGGTGGGGGCCCATGCAGTGTCT

AGAACCTTTCAACTTCATACCTGGATTTGTGTTTTTTATGGGGAGCTTTTGCTTGTAAGCTATTCTGATG

GTGCTAAACTTGTATAGGTTTCAGGACATACTATTTTGACTAAGGTCTCTCCAAAATTATATAGTGGACC

ATCTCCTTGTAACTGGCCTGTGACTTAGATTTTCCAATGCAATGATCTATCAGGAGTTCTATCCTAAATA

ATGTTTCTAACATTGATTTCAGATGCATTCTGATGTAGGAGTTTAATATGTAATTGTACTTTTGTAATTG

CCCCATTACAAAAAATACTTATATTATCACAAATTTCCTAGGTGGACAATATCAGCAAAGCTCTTCATAA

TGTGA

>Phytelephas_tumacana_EF128409.1

GTAAATCCATAGAAGCTTCATGCAAGCTTTACCTGAGAAGCATGTCACAATTTAATTTGCTATAAATGCT

GGTGCTTTTTTTGCTTATTCAGTTTTAGAATTTCGAGGCACTGTATCTGATGTTAAAGAACACTAAGGAA

GTTTTCTTTCATTCTGCTCCAGGAGGAAGTAATTTTTTGCATTTGTCTAACTAAAAGATATTTTGGATAG

CTTATACAAAAACACAAGGTTATTGAACTTGCATTACACCTACCCGCCGGTGGGGGCCCATGCAGTGTCT

AGAACCTTTCAACTTCATACCTGGATTTGTGTTTTTTATGGGGAGCTTTTGCTTGTAAGCTATTCTGATG

GTGCTAAACTTGTATAGGTTTCAGGACATACTATTTTGACTAAGGTCTCTCCAAAATTATATAGTGGACC

ATCTCCTTGTAACTGGCCTGTGACTTAGATGTTCCAATGCAATGATCTATCAGGAGTTCTATCCTAAATA

ATGTTTCTAACATTGATTTCAGATSCATTCTGATGTAGGAGTTTAATATGTAATTGTACTTTTGTAATTG

CCCCA

>Phytelephas_tenuicaulis_EF128407.1

GTAAATCCATAGAAGCTTCATGCAAGCTTTACCTGAGAAGCATGTCACAATTTAATTTGCTATAAATGCT

GGTGCTTTTTTTGCTTATTCAGTTTTAGAATTTCGAGGCACTGTATCTGATGTTAAAGAACACTAAGGAA

GTTTTCTTTCATTCTGCTCCAGGAGGAAGTAATTTTTTGCATTTGTCTAACTAAAAGATATTTTGGATAG

CTTATACAAAAACACAAGGTTATTGAACTTGCATTACACCTACCCCCCGGTGGGGGCCCATGCAGTGTCT

AGAACCTTTCAACTTCATACCTGGATTTGTGTTTTTTATGGGGAGCTTTTGCTTGTAAGCTATTCTGATG

GTGCTAAACTTGTATAGGTTTCAGGACATACTATTTTGACTAAGGTCTCTCCAAAATTATATAGTGGACC

ATCTCCTTGTAACTGGCCTGTGACTTAGATTTTCCAATGCAATGATCTATCAGGAGTTCTATCCTAAATA

ATGTTTCTAACATTGATTTCAGATGCATTCTGATGTAGGAGTTTAATATGTAATTGTACTTTTGTAATTG

CCCCA

>Phytelephas_schottii_EF128405.1

GTAAATCCATAGAAGCTTCATGCAAGCTTTACCTGAGAAGCATGTCACAATTTAATTTGCTATAAATGCT

GGTGCTTTTTTTGCTTATTCAGTTTTAGAATTTCGAGGCACTGTATCTGATGTTAAAGAACACTAAGGAA

GTTTTCTTTCATTCTGCTCCAGGAGGAAGTAATTTTTTGCATTTGTCTAACTAACAGATATTTTGGATAG

CTTATACAAAAACACAAGGTTATTGAACTTGCATTACACCTACCCCCCGGTGGGGGCCCATGCAGTGTCT

AGAACCTTTCAACTTCATACCTGGATTTGTGTTTTTTATGGGGAGCTTTTGCTTGTAAGCTATTCTGATG

GTGCTAAACTTGTATAGGTTTCAGGACATACTATTTTGACTAAGGTCTCTCCAAAATTATATAGTGGACC

ATCTCCTTGTAACTGGCCTGTGACTTAGATTTTCCAATGCAATGATCTATCAGGAGTTCTATCCTAAATA

ATGTTTCTAACATTGATTTCAGATGCATTCTGATGTAGGAGTTTAATATGYAATTGTACTTKGTCAATTG

CCCCA

>Phytelephas_macrocarpa_EF128403.1

GTAAATCCATAGAAGCTTCATGCAAGCTTTACCTGAGAAGCATGTCACAATTTAATTTGCTATAAATGCT

GGTGCTTTTTTTGCTTATTCAGTTTTAGAATTTCGAGGCACTGTATCTGATGTTAAAGAACACTAAGGAA

GTTTTCTTTCATTCTGCTCCAGGAGGAAGTAATTTTTTGCATTTGTCTAACTAAAAGATATTTTGGATAG

CTTATACAAAAACACAAGGTTATTGAACTTGCATTACACCTACCCCCCGGTGGGGGCCCATGCAGTGTCT

AGAACCTTTCAACTTCATACCTGGATTTGTGTTTTTTATGTGGAGCTTTTGCTTGTAAGCTATTCTGATG

GTGCTAAACTTGTATAGGTTTCAGGACATACTATTTTGACTAAGGTCTCTCCAAAATTATATAGTGGACC

ATCTCCTTGTAACTGGCCTGTGACTTAAATTTTCCAATGCAATGATCTATCAGGAGTTCTATCCTAAATA

ATGTTTCTAACATTGATTTCAGATGCATTCTGATGTAGGAGTTTAATATGTAATTGTACTTTTGTAATTG

CCCCA

>Phytelephas_seemannii_EF128406.1

GTAAATCCATAGAAGCTTCATGCAAGCTTTACCTGAGAAGCATGTCACAATTTAATTTGCTATAAATGCT

GGTGCTTTTTTTGCTTATTCAGTTTTAGAATTTCGAGGCACTGTATCTGATGTTAAAGAACACTAAGGAA

GTTTTCTTTCATTCTGCTCCAGGAGGAAGTAATTTTTTGCATTTGTCTAACTAACAGATATTTTGGATAG

CTACAAAAACACAAGGTTATTGAACTTGCATTACACCTACCCCCCGGTGGGGGCCCATGCAGTGTCTAGA

ACCTTTCAACTTCATACCTGGATTTGTGTTTTTATGGGGAGCTTTTGCTTGTAAACTATTCTGATGGTGC

TAAACTTGTATAGGTTTCAGGACATACTATTTTGACTAAGGTCTCTCCAAAATTATATAGTGGACCATCT

CCTTGTAACTGGCCTGTGACTTAGATTTTCCAATGCGATGATCTATCAGGAGTTCTATCCTAAATAATGT

TTCTAACATTGATTTCAGATGCATTCTGATGTAGGAGTTTAATATGTAATTGTACTTTTGTAATTGCCCC

A

>Phytelephas_tenuicaulis_EF128408.1

GTAAATCCATAGAAGCTTCATGCAAGCTTTACCTGAGAAGCATGTCACAATTTAATTTGCTATAAATGCT

GGTGCTTTTTTTGCTTATTCAGTTTTAGAATTTCGAGGCACTGTATCTGATGTTAAAGAACACTAAGGAA

GTTTTCTTTCATTCTGCTCCAGGAGGAAGTAATTTTTTGCATTTGTCTAACTAAAAGATATTTTGGATAG

CTTATACAAAAACACAAGGTTATTGAACTTGCATTACACCTACCCCCGGTGGGGGCCCATGCAGTGTCTA

GAACCTTTCAACTTCATACCTAGATTTGTGTTTTTTATGGGGAGCTTTTGCTTGTAAGCTATTCTGATGG

TGCTAAACTTGTATAGGTTTCAGGACATACTATTTTGACTAAGGTCTCTCCAAAATTATATAGTGGACCA

TCTCCTTGTAACTGGCCTGTGACTTAGATTTTCCAGTGCAATGATCTATCAGGAGTTCTATCCTAAATAA

TGTTTCTAACATTGATTTCAGATGCATTCTGATGTAGGAGTTTAATATGTAATTGTACTTTTGTAATTGC

CCCA

>Phytelephas_schottii_EF128404.1

GTAAATCCATAGAAGCTTCATGCAAGCTTTACCTGAGAAGCATGTCACAATTTAATTTGCTATAAATGCT

GGTGCTTTTTTTGCTTATTCAGTTTTAGAATTTCGAGGCACTGTATCTGATGTTAAAGAACACTAAGGAA

GTTTTCTTTCATTCTGCTCCAGGAGGAAGTAATTTTTTGCATTTGTCTAACTAACAGATATTTTGGATAG

CTTATACAAAAACACAAGGTTATTGAACTTGCATTACACCTACCCCCCGGTGGGGGCCCATGCAGTGTCT

AGAACCTTTCAACTTCATACCTGGATTTGTGTTTTTTATGGGGAGCTTTTGCTTGTAAGCTATTCTGATG

GTGCTAAACTTGTATAGGTTTCAGGACATACTATTTTGACTAAGGTCTCTCCAAAATTATATAGTGGACC

ATCTCCTTGTAACTGGCCTGTGACTTAGATTTTCCAATGCAATGATCTATCAGGAGTTCTATCCTAAATA

ATGTTTCTAACATTGATTTCAGATGCATTCTGATGTAGGAGTTTAATATGYAATTGTACTTKGTCAATTG

CCCCA

>Pseudophoenix_vinifera_AJ830181.1

AAGGAGGGAGATGCTACTCCTTTTACTGATGTCACAGTAAGTAAATCCATAGAAGCTTCATGCAAGCTTT

ACTTGAGAAGCATGTCACAACTTAATTTGCTACAAATGCTGGAGCTTTTTTTTTTTCCCTCATTCAGATA

AAAACATGAAGGAAGTTTTCTTTCATTCTGCTCCAGGAGTAAAATTTTTGCATATGTCTAGCTAAAAGAT

GTTGTAGATAGCTTATACAAAAACACAAGGTTATTGAATTTGCGTTGCCTACCTTCCCCATCCTGTGGCC

CAGATTTTTAAGTAAGATCCGGTTATTTGTGGGGAGCTTTTGCCTTCAGAGTTCATATGTTCCTCTGGAT

TAGTTAATTATAATCCTGTTATTTGAAGTCTACTTTTGATATCTCTTTTGAAGTCATTGAGATGTAAGTT

TACCCTGTAGACAGAAACACGGCTTTGAAGAATGACCTTGGACAAGTGTCTTCTAAGCTATTATGTTGAT

CCTAAACTTGTTTAAGTTTCAGGACATACTATTTTGATGAAGGTCTCTCCACAATTATATAGCAGACTAT

CTCCTTGTAACTAGCCTGTGACTTGGATTTTCTAATTCAATGACCTATCAGGAGTTCTATCCTAAATAAT

GTTCTAACGTTGATTTCAGATATATYCTGATATAGGAGTTTAGTATGTAATTGTACTTTTGTAATTGCCC

CATTCCACAAAATACTTATATTATCAAAAAATTCCTAGGTGGAC

>Pseudophoenix_sargentii_var.navassana_EF128399.1

GTAAATCCATAGAAGCTTCATGCAAGCTTTACTTGAGAAGCATGTCACAACTTAATTTGCTACAAATGCT

GGAGCTTTTTTTTTTTCCCTTATTCAAATAAAAACATGAAGGAAGTTTTCTTTCATTCCGCTCCAGGAGT

AAAATTTTTGCATATGTCTACCTAAAAGATGTTGTAAATAGCTTATACAAAAACACAAGGTTATTGAATT

TGCGTTACGCCTACCTTCCCCATCCTGTGGCCCARATTTTTAAGTAAGATCCGGTTATTTGTGGGGAGCT

TTCGCCTTCAGAGTTCATATGTTCCTCTGGATTAGTTAATTATAATCCTGTTATTTGAAGTCTACTTTTG

ATATCTCTTTTGAAGTCATTGAGATGTAAGTTTTCCCTGTAGACAGAAACACGGCTTTGAAGAATGACCT

TGGACAAGTGTCTTCTAAGCTATTATGTTGATCCTAAACTTGTTTAAGTTTCAGGACATACTATTTTGAT

GAAGGTCTCTCCACAATTATATAGCAGACTATCTCCTTGTAACTAGCCTGTGACTTGGATTTTCTAATTC

AATGACCTATCAGTTCTATCCTAAATAATGTTCTAACGTTGATTTCAGATATATCCTGATATAGGAGTTT

AGTAAGTAATTGTACTTTTGTAATT

>Pseudophoenix_ekmanii_EF128397.1

GTAAATCCATAGGAGCTTTTTTTTTTCCCTWATTCAGATAAAAACATGAAGGAGGTTTTCTTTCATGCCG

CTCCAGGAGTAAAATTTTTGCATATGTCTAGCTAAAAGATGTTGTAGATAGCTTATACAAAAACACAAGG

TTATTGAATTTGCGTTACGCCTACCTTCCCCATCCTGTGGCCCAGATTTTTAAGTATGATCCGGTTATTT

GTGGGGAGCTTTKGCCTTCGGAGTTCATATGTTCCTCTGGATTAGTTAATTATAATCCTGTTATTTGAAG

TCTACTTTTGATATCTCTTTTGAAGTCATTGAGATGTAAGTTTACCCTGTAGACAGAAACACGGCTTTGA

AGAATGACCTTGGACAAGTGTCTTCTAAGCTATTATGTTGATCCTAAACTTGTTTAAGTTTCAGGACATA

CTATTTTGATGAAGGTCTCTCCACAATTATATAGCAGACTATCTCCTTGTAACTAGCCTGTGACTTGGAT

TTTCTAATTCAATGGCCTATCAGGAGTTCTATCCTAAATAATGTTCTAACATTGATTTCAGATATATCCT

GATGTAGGAGTTTAGTATGTAATTGTACTTTTGTAATTGCCCCA

>Pseudophoenix_sargentii_var.saonae_EF128400.1

GTAAATCCATAGAAGCTTCATGCAAGCTTTACTTGAGAAGCATGTCACAACTTAATTTGCTACAAATGCT

GGAGCTTTTTTTTTTTCCCTGTATTCAGATAAAAACATGAAGGAAGTTTTCTTKCATYCCGCTCCAGGAG

TAAAATTTTGGCATATGTCTAGCTAAAAGATGTTGTAGATAGCTTATACAAAAACACAAGGTTATTGAAT

TTGCGTTACGCCTACCTTCCCCATCCTGTGGCCCAGATTTTTAAGTAAGATCCGGTTATTTGTGGGGAGC

TTTCGCCTTCAGAGTTCATATGTTCCTCTGGATTAGTTAATTATAATCCTGTTATTTGAAGTCTACTTTT

GATATCTCTTTTGAAGTCATTGAGATGTAAGTTTACCCTGTAGACAGAAACACGGCTTTGAAGAATGACC

TTGGACAAGTGTCTTCTAAGCTATTATGTTGATCCTAAACTTGTTTAAGTTTCAGGACATACTATTTTGA

TGAAGGTCTCTCCACAATTATATAGCAGASTATCTCCTTGTAAMTAGCCTGTGACTTGGATTTTCTAATT

CAATGACCTATCAGGAGTTCTATCCTAAATAATGKTCTAACGTTGATTTCAGATATATCCTGATATAGGA

GTTTAGTATGTAATTGTACTTTTGTAATTGCCCCA

>Pseudophoenix_lediniana_EF128398.1

ACTTGAGAAGCATGTCACAACTTAATTTGCTACAAATGCTGGAGCTTTTTTTTTTCCCTTATTCAGATAA

AAACATGAAGGAAGTTTTCTTTCATTCCGCTCCAGGAGTAAAATTTTTGCATATGTCTAGCTAAAAGATG

TTGTAGATAGCTTATACAAAAACACAAGGTTATTGAATTTGCGTTACGCCTACCTTCCCCATCCTGTGGC

CCAGATTTTTAAGTATGTTCCGGTTATTTGTGGGGAGCTTTTGCCTTCAGAGTTCATATGTTCCTCTGGA

TTAGTTAATTATAATCCTGTTATTTGAAGTCTACTTTTGATATCTCTTTTGAAGTCATTGAGATGTAAGT

TTACCCTGTAGACAGAAACACGGCTTTGAAGAATGACCTTGGACAAGTGTCTTCTAAGCTATTATGTTGA

TCCTAAACTTGTTTAAGTTTCAGGACATACTATTTTGATGAAGGTCTCTCCACAATTATATAGCAGACTA

TCTCCTTGTAACTAGCCTGTGACTTGGATTTTCTAATTCAATGGCCTATCAGGAGTTCTATCCTAAATAA

TGTTCTAACATTGATTTCAGATATATCCTGATGTAGGAGTTTAGTA

>Reinhardtia_gracilis_AJ830182.1

GGGAGATGCTACTCCTTTTACTGATGTCACAGTAAGTAAATCCATAGAACCTTCATGCAAGCTTTACCTG

AGAAGCATGTCACAATTTAACTTGTTATAAATGTTGGAGCTTTTTTGCTTATTCAGTTTAAGAATTCCAA

GGCTCTGTATCTGATGTTACAGAACATTATGGAAAGTTTCTTTTGATCTGCTTGAGCAATTAATTTTTGC

ATGTCTAGCTAAAAGACATTGTTGGTAGCTCGTACAAGAATGCATGGTAGTCGTACAGAATGCCTTTGGA

GTTCATATTTTCCTCTGGATTAGTTAATTATAAGCATGTTATTTGAAGTCTACTTTGAGCATCTCTTTTG

AAGTTGGTGATATGTTAGTTTACCCTGTAGACAGAAAGTAGCTCTGAAGAATGACCTTGGACGGGTGTCT

TGTAAGTTATTCTGATGATCCTAAACTTGTGTAAGTTTCAAGACATACTATTTTGACTAAGGTCTCTCCT

AAATTATGTCATTGACTATCTCCTTGTAACTGACCTGTGACTTGGATTTTCCAATTCAATGATCTATTAG

GAGTTATCCCAAATAATATTTCTAACATTGATTTCAGATGTATACTGATGTAGGAGTTGAATATGTAATT

GTACTTTTGTAGTTGCCCCATTACAAAAAATGCTTATATTATCACAAATTTCCTAGG

>Reinhardtia_simplex_AJ830183.1

CATCATGGAAAGGTTGCTGCCCATATGGGAAAGGAGGGAGATGCTACTCCTTTACTGATGTCACAGTAAG

TAAATCCATAGAACCTTCATGCAAGCTTTACCTGAGAAGCATGTCACAATTTAACTTGTTATAAATGTTG

GAGCTTTTTTGCTTATTCAGTTTAAGAATTCCAAGGCTCTGTATCTGATGTTACAGAACATTATGGAAAG

TTTCTTTTGATCTGCTTGAGCAAATAATTTTTGCATGTCTTGCTAAAAGACATTTTTGGTAGCTCGTACA

AGAATGCATGGTAGATCGTACAAGAATGCCTTTGGAGTTCATATTTTCCTCTGGATTAGTTAATTATAAG

CATGTTATTTGAAGTCTACTTTGAGCATCTCTTTTGAAGTTGGTGATATGTTAGTTTACCCTGTAGACAG

AAAGTAGCTCTGAAGAATGACCTTGGACGGGTGTCTTGTAAGTTATTCTGATGATCCTAAACTTGTGTAA

GTTTCAAGACATACTATTTTGACTAAGGTCTCTCCTAAATTATGTCATTGACTATCTCCTTGTAACTGAC

CTGTGACTTGGATTTTCCAATTCAATGATCTATTAGGAGTTATCCCAAATAATATTTCTAACATTGATTT

CAGATGTATACTGATGTAGGAGTTGAATATGTAATTGTACTTTTGTAGTTGCCCCATTACAAAAAATGCT

TATATTATCACAAATTTCCTAGGTGGACAA

>Reinhardtia_gracilis_var.rostrata_HQ265664.1

GCATCATGGGAAAGGTTGCTGCCCATATGGGAAAGGAGGGAGATGCTACTCCTTTTACTGATGTCACAGT

AAGTAAATCCATAGAACCTTCATGCAAGCTTTACCTGAGAAGCATGTCACAATTTAACTTGTTATAAATG

TTGGAGCTTTTTTGCTTATTCAGTTTAAGAATTCCAAGGCTCTGTATCTGATGTTACAGAACATTATGGA

AAGTTTCTTTTGATCTGCTTGAGCAATTAATTTTTGCATGTCTAGCTAAAAGACATTGTTGGTAGCTCGT

ACAAGAATGCATGGTAGCTCGTACAAGAATGCCTTTGGAGTTCATATTTTCCTCTGGATTAGTTAATTAT

AAGCATGTTATTTGAAGTCTACTTTGAGCATCTCTTTTGAAGTTGGTGATATGTTAGTTTACCCTGTAGA

CAGAAAGTAGCTCTGAAGAATGACCTTGGACGGGTGTCTTGTAAGTTATTCTGATGATCCTAAACTTGTG

TAAGTTTCAAGACATACTATTTTGACTAAGGTCTCTCCTAAATTATGTCATTGACTATCTCCTTGTAACT

GACCTGTGACTTGGATTTTCCAATTCAATGATCTATTAGGAGTTATCCCAAATAATATTTCTAACATTGA

TTTCAGATGTATACTGATGTAGGAGTTGAATATGTAATTGTACTTTTGTAGTTGCCCCATTACAAAAAAT

GCTTATATTATCACAAATTTCCTAGGTGGACAATATAAGCAAAGCTCTTCATAAGTGTGGATA

>Reinhardtia_simplex_HQ265665.1

GCATCATGGGAAAGGTTGCTGCCCATATGGGAAAGGAGGGAGATGCTACTCCTTTTACTGATGTCACAGT

AAGTAAATCCATAGAACCTTCATGCAAGCTTTACCTGAGAAGCATGTCACAATTTAACTTGTTATAAATG

TTGGAGCTTTTTTGCTTATTCAGTTTAAGAATTCCAAGGCTCTGTATCTGATGTTACAGAACATTATGGA

AAGTTTCTTTTGATCTGCTTGAGCAAATAATTTTTGCATGTCTTGCTAAAAGACATTTTTGGTAGCTCGT

ACAAGAATGCATGGTAGATCGTACAAGAATGCCTTTGGAGTTCATATTTTCCTCTGGATTAGTTAATTAT

AAGCATGTTATTTGAAGTCTACTTTGAGCATCTCTTTTGAAGTTGGTGATATGTTAGTTTACCCTGTAGA

CAGAAAGTAGCTCTGAAGAATGACCTTGGACGGGTGTCTTGTAAGTTATTCTGATGATCCTAAACTTGTG

TAAGTTTCAAGACATACTATTTTGACTAAGGTCTCTCCTAAATTATGTCATTGACTATCTCCTTGTAACT

GACCTGTGACTTGGATTTTCCAATTCAATGATCTATTAGGAGTTATCCCAAATAATATTTCTAACATTGA

TTTCAGATGTATACTGATGTAGGAGTTGAATATGTAATTGTACTTTTGTAGTTGCCCCATTACAAAAAAT

GCTTATATTATCACAAATTTCCTAGGTGGACAATATAAAGCAAAGCTCTTCATAAGTGTGGATA

>Reinhardtia_simplex_AY779372.1

TTGCCTTTAGAGTTCATATTTTCCTCTGGATTAGTTAATTATAAGCATGTTATTTGAAGTCTACTTTGAG

CATCTCTTTTGAAGTTGGTGATATGTTAGTTTACCCTGTAGACAGAAAGTAGCTCTGAAGAATGACCTTG

GACGGGTGTCTTGTAAGTTATTCTGATGATCCTAAACTTGTGTAAGTTTCAAGACATACTATTTTGACTA

AGGTCTCTCCTAAATTATGTCATTGACTATCTCCTTGTAACTGACCTGTGACTTGGATTTTCCAATTCAA

TGATCTATTAGGAGTTATCCCAAATAATATTTCTAACATTGATTTCAGATGTATACTGATGTAGGAGTTG

AATATGTAATTGTACTTTTGTAGTTGCCCCATTACAAAAAATGCTTATATTATCACAAATTTCCTAGGTG

GACAATATAAGCAAAGCTCTTCATAAG

>Reinhardtia_gracilis_AY543106.1

GGGAGATGCTACTCCTTTTACTGATGTCACAGTAAGTAAATCCATAGAACCTTCATGCAAGCTTTACCTG

AGAAGCATGTCACAATTTAACTTGTTATAAATGTTGGAGCTTTTTTGCTTATTCAGTTTAAGAATTCCAA

GGCTCTGTATCTGATGTTACAGAACATTATGGAAAGTTTCTTTTGATCTGCTTGAGCAATTAATTTTTGC

ATGTCTAGCTAAAAGACATTGTTGGTAGCTCGTACAAGAATGCATGGTAGCTCGTACAAGAATGCCTTTG

GAGTTCATATTTTCCTCTGGATTAGTTAATTATAAGCATGTTATTTGAAGTCTACTTTGAGCATCTCTTT

TGAAGTTGGTGATATGTTAGTTTACCCTGTAGACAGAAAGTAGCTCTGAAGAATGACCTTGGACGGGTGT

CTTGTAAGTTATTCTGATGATCCTAAACTTGTGTAAGTTTCAAGACATACTATTTTGACTAAGGTCTCTC

CTAAATTATGTCATTGACTATCTCCTTGTAACTGACCTGTGACTTGGATTTTCCAATTCAATGATCTATT

AGGAGTTATCCCAAATAATATTTCTAACATTGATTTCAGATGTATACTGATGTAGGAGTTGAATATGTAA

TTGTACTTTTGTAGTTGCCCCATTACAAAAAATGCTTATATTATCACAAATTTCCTAGG

>Roystonea_regia_AJ830189.1

GGAGGGAGATGCTACTCCTTTTACTGATGTCACAGTAAGTAAATCCATAGAACCTTCATGCAAGCTTTAC

CTGAGAAGCATGTCACAGTTTAGCTTGCTATAAATGCTGGAGCTTTTTTGCTTATTCGGTTTAAGAATTC

CAAGGCACTGTATCTGATGTTAAAGAACATTATGGAAAGTTTCTTTTGTTCTGCTCGAGCAGGAAGTAAT

TTTTGCATTTGTCTAGCTAAAAGACATTGTTGTAGCTTGTTAAAAAACTCATGGTTATTCAATTTTTATT

ACCATCAAGGATTTGTACTAGATATTGGTTTAGGGTAGACAGAAACGTGGCTTTGAAGAATGACCTTGGA

TGAGTGTCTAGGAAGTTATTCTGATGATCCTAAACTTGTTTAAGTTTCAAGACATACTATTTTGACTAAG

GTCTCCCCTAAATTATATAATAGACTATCTCCTTTTAACTGACCCGTGACTTAAATTTTCCAATTCAATG

ATCTATCAGCTATCCTAAATAATGTTTCCAATAGACTATCTCCTTGTAACTGAACTGTTGCTTGGAATTT

CCAATTCAATGATCTATCAGGAGTTCTATCCTGAATAATGTTTCTAACATTGATTTCAGATATATTCTGA

TGTAGGAGTTTAATATGTAAGTGTACCTTTGTAATTGCTCCATTACAAAAGATGCTTATATTATCACAA

>Roystonea_regia_AJ830187.1

GGGAGATGCTACTCCTTTTACTGATGTCACAGTAAGTAAATCCATAGAACCTTCATGCAAGCTTTACCTG

AGAAGCATGTCACAGTTTAGCTTGCTATAAATGCTGGAGCTTTTTTGCTTATTCGGTTTAAGAATTCCAA

GGCACTGTATCTGATGTTAAAGAACATTATGGAAAGTTTCTTTTGTTCTGCTCGAGCAGGAAGTAATTTT

TGCATTTGTCTAGCTAAAAGACATTGTTGTAGCTTGTTAAAAAACTCATGGTTATTCAATTTTTATTACC

ATCAAGGATTTGTACTAGATATTGGTTTAGGGTAGACAGAAACGTGGCTTTGAAGAATGACCTTGGATGA

GTGTCTAGGAAGTTATTCTGATGATCCTAAACTTGTTTAAGTTTCAAGACATACTATTTTGACTAAGGTC

TCCCCTAAATTATATAATAGACTATCTCCTTTTAACTGACCCGTGACTTAAATTTTCCAATTCAATGATC

TATCAGCTATCCTAAATAATGTTTCCAATAGACTATCTCCTTGTAACTGAACTGTTGCTTGGAATTTCCA

ATTCAATGATCTATCAGGAGTTCTATCCTGAATAATGTTTCTAACATTGATTTCAGATATATTCTGATGT

AGGAGTTTAATATGTAAGTGTACCTTTGTAATTGCTCCATTACAAAAGATGCTTATATTATCACAAATTG

CCTAGGTGGACAATATCAGCAAGCTCTC

>Roystonea_regia_AJ830185.1

GTGCTGCCCATATGGGAAGGAGGGAGATGCTACTCCTTTTACTGATGTCACAGTAAGTAAATCCATAGAA

CCTTCATGCAAGCTTTACCTGAGAAGCATGTCACAGTTTAGCTTGCTATAAATGCTGGAGCTTTTTTGCT

TATTCGGTTTAAGAATTCCAAGGCACTGTATCTGATGTTAAAGAACATTATGGAAAGTTTCTTTTGTTCT

GCTCGAGCAGGAAGTAATTTTTGCATTTGTCTAGCTAAAAGACATTGTTGTAGCTTGTTAAAAAACTCAT

GGTTATTCAATTTTTATTACCATCAAGGATTTGTACTAGATATTGGTTTAGGGTAGACAGAAACGTGGCT

TTGAAGAATGACCTTGGATGAGTGTCTAGGAAGTTATTCTGATGATCCTAAACTTGTTTAAGTTTCAAGA

CATACTATTTTGACTAAGGTCTCCCCTAAATTATATAATAGACTATCTCCTTTTAACTGACCCGTGACTT

AAATTTTCCAATTCAATGATCTATCAGCTATCCTAAATAATGTTTCCAATAGACTATCTCCTTGTAACTG

AACTGTTGCTTGGAATTTCCAATTCAATGATCTATCAGGAGTTCTATCCTGAATAATGTTTCTAACATTG

ATTTCAGATATATTCTGATGTAGGAGTTTAATATGTAAGTGTACCTTTGTAATTGCTCCATTACAAAAGA

TGCTTATATTATCACAAATTGCCTAGGTGGA

>Roystonea_oleracea_AJ830184.1

TGCTGCGCATATGGGAAAGGAGGGAGATGCTACTCCTTTTACTGATGTCACAGTAAGTAAATCCATAGAA

CCTTCATGCAAGCTTTACCTGAGAAGCATGTCAGAGTTTAGCTTGCTATAAATGCTGGAGCTTTTTTGCT

TATTCGGTTTAAGAATTCCAAGGCACTGTATCTGATGTTAAAGAACATTATGGAAAGTTTCTTTTGTTCT

GCTCGAGCAGGAAGTAATTTTTGCGTTTGTCTAGCTAAAAGACATTGTTGTAGCTTGTACAAAAACTCAT

GGTTATTCAATTTTTATTACCATCAAGGATTTGTACTAGATATTGGTTTAGGGTAGACAGAAACGTGGCT

TTGAAGAATGACCTTGGATGAGTGTCTAGGAAGTTATTCTGATGATCCTAAACTTGTTTAAGTTTCAAGA

CATACTATTTTGACTAAGGTCTCCCCTAAATTATATAATAGACTATCTCCTTTTAACTGACCTGTGACTT

AAATTTTCCAATTCAATGATCTATCAGGAGTTCTATCCTAAATAATGTTTCCAATAGACTATCTCCTTGT

AACTGAACTGTTGCTTGGAATTTCCAATTCAATGATCTATCAGGAGTTCTATCCTGAATAATGTTTCTAA

CATTGATTTCAGATATATTCTGATGTAGGAGTTTAATATGTAAGTGTACCTTTGTAATTGCTCCATTACA

AAGATGCTTATATTATCACAAATTGCCTAGGTGGACAATATCAGCAAAGCTCTTCATAAGTGTG

>Roystonea_regia_AJ830188.1

CATATGGGAAAGGAGGGAGATGCTACTCCTTTTACTGATGTCACAGTAAGTAAATCCATAGAACCTTCAT

GCAAGCTTTACCTGAGAAGCATGTCACAGTTTAGCTTGCTATAAATGCTGGAGCTTTTTTGCTTATTCGG

TTTAAGAATTCCAAGGCACTGTATCTGATGTTAAAGAACATTATGGAAAGTTTCTTTTGTTCTGCTCGAG

CAGGAAGTAATTTTTGCATTTGTCTAGCTAAAAGACATTGTTGTAGCTTGTTAAAAAACTCATGGTTATT

CAATTTTTATTACCATCAAGGATTTGTACTAGATATTGGTTTAGGGTAGACAGAAACGTGGCTTTGAAGA

ATGACCTTGGATGAGTGTCTAGGAAGTTATTCTGATGATCCTAAACTTGTTTAAGTTTCAAGACATACTA

TTTTGACTAAGGTCTCCCCTAAATTATATAATAGACTATCTCCTTTTAACTGACCCGTGACTTAAATTTT

CCAATTCAATGATCTATCAGCTATCCTAAATAATGTTTCCAATAGACTATCTCCTTGTAACTGAACTGTT

GCTTGGAATTTCCAATTCAATGATCTATCAGGAGTTCTATCCTGAATAATGTTTCTAACATTGATTTCAG

ATATATTCTGATGTAGGAGTTTAATATGTAAGTGTACCTTTGTAATTGCTCCATTACAAAAGATGCTTAT

ATTATCACAAATTGCCTAGGTGGACAATATCAGCAAGCTCTCATAG

>Roystonea_regia_AJ830186.1

GGAGATGCTACTCCTTTTACTGATGTCACAGTAAGTAAATCCATAGAACCTTCATGCAAGCTTTACCTGA

GAAGCATGTCACAGTTTAGCTTGCTATAAATGCTGGAGCTTTTTTGCTTATTCGGTTTAAGAATTCCAAG

GCACTGTATCTGATGTTAAAGAACATTATGGAAAGTTTCTTTCGTTCTGCTCGAGCAAGAAGTAATTTTT

GCATTTGTCTAGCTAAAAGACATTGTTGTAGCTTGTTAAAAAACTCATGGTTATTCAATTTTTATTACCA

TCAAGGATTTGTACTAGATATTGGTTTAGGGTAGACAGAAACGTGGCTTTGAAGAATGACCTTGGATGAG

TGTCTAGGAAGTTATTCTGATGATCCTAAACTTGTTTAAGTTTCAAGACATACTATTTTGACTAAGGTCT

CCCCTAAATTATATAATAGACTATCTCCTTTTAACTGACCCGTGACTTAAATTTTCCAATTCAATGATCT

ATCAGCTATCCTAAATAATGTTTCCAATAGACTATCTCCTTGTAACTGAACTGTTGCTTGGAATTTCCAA

TTCAATGATCTATCAGGAGTTCTATCCTGAATAATGTTTCTAACATTGATTTCAGATATATTCTGATGTA

GGAGTTTAATATGTAAGTGTACCTTTGTAATTGCTCCATTACAAAAGATGCTTATATTATCACAAATTG

>Rhapidophyllum_hystrix_EU215488.1

ATATGCCAAAGGAGGGAGATGCTACTCCTTTTACTGATGTCACAGTAAGTAAATACATAAAACCTTCGTG

CAAGCTTTACCTGAGAAGCGTGCCATAATTTAACCTGCTATAAATGCTGGAGCTTTTTTGATTATTCAGT

TTAAGAATTCCGAGTCACTGTATCTGATGTTAAAGAACATTAGGAAAGTTTCTTTTGTTCTGCTCCAGGA

GGAAGTAATTTTTGCATATGTCTAGCTAAAAGACATTGTTGATAGCTTATACGAAAACAGAAGGTTATTC

AATTCTCTTTACCATTAAGGATTTGTATTAGATATGGTTGAGCGTTTGCCTTTGGAGTTCATATTTTCCT

CTGGTTTAGTTAATTATAATCCTGTTATTTGAAGTGTACTTTGAATATCTCTTTTGAAGTTCTTGAGATG

TAAGTTTACCCTGTAGACAGAAACGTGGCTTTGAAGAACGACTTTGGACGAGTGTAAGTTATTCTGATGA

TCCTAAACTTGTATAAGTTTCAAGACATACTATTTTGACTAAGGTCTCTTTTAAATTATATAGTAGACTA

TCTCCTTGTAACTGGCCTGTGACTTGGGTTTTCCAATTCGATGATCTATCAGGAGTTCTATCCTAAATAA

TGTTTCTAACATTGATTTCAGATGCATTCTGATATAGGAGTTCCATATGTAATTGTTCTTTTGTAATTTC

CCCATAACAAAAAATACTATATTATCATAAATTTCCTAGGTGGACAATATCAGCAAAGCTCTT

>Rhapidophyllum_hystrix_HQ720571.1

CTCCTTTTACTGATGTCACAGTAAGTAAATACATAAAACCTTCGTGCAAGCTTTACCTGAGAAGCGTGCC

ATAATTTAACCTGCTATAAATGCTGGAGCTTTTTTGATTATTCAGTTTAAGAATTCCGAGTCACTGTATC

TGATGTTAAAGAACATTAGGAAAGTTTCTTTTGTTCTGCTCCAGGAGGAAGTAATTTTTGCATATGTCTA

GCTAAAAGACATTGTTGATAGCTTATACGAAAACAGAAGGTTATTCAATTCTCTTTACCATTAAGGATTT

GTATTAGATATGGTTTAGTTAATTATAATCCTTTCAACTTCATACCTGGATTTGATTTTCTTTTGAGGAG

AGCGTTTGCCTTTGGAGTTCATATTTTCCTCTGGTTTAGTTAATTATAATCCTGTTATTTGAAGTGTACT

TTGAATATCTCTTTTGAAGTTCTTGAGATGTAAGTTTACCCTGTAGACAGAAACGTGGCTTTGAAGAACG

ACTTTGGACGAGTGTAAGTTATTCTGATGATCCTAAACTTGTATAAGTTTCAAGACATACTATTTTGACT

AAGGTCTCTTTTAAATTATATAGTAGACTATCTCCTTGTAACTGGCCTGTGACTTGGGTTTTCCAATTCG

ATGATCTATCAGGAGTTCTATCCTAAATAATGTTTCTAACATTGATTTCAGATGCATTCTGATATAGGAG

TTCCATATGTAATTGTTCTTTTGTAATTTCCCCATAACAAAAAATACTTATATTATCATAAATT

>Sabal_palmetto_HQ720578.1

TTTACTGATGTCACAGTAAGTAATTCCATAAAACCTTCATGCAAGCTTTACCTGAGAAGCATGTCACAAT

TTAACTTGCTATAGATGCTGGAGCTTTTTTGCTTATTCAGTTTAAGAAAGTGTCTGGTGTTAAAGAACAT

TGAGGAAAGTTTCTTTTGTTCTGCTCCTGGAGGAAGTAGTTTTTGCATGTCTAGCTAAAAGACATCGTTG

ATAGCTTATACAAAAGCAGAAAGTTATTCAATTTGCATTACCATTAAGGATTTGTACTAGATATAGTTAA

TTATATCTATATCTAGAACCTTTCAACTCCATACCTGAATTTTGAGGGGAGCTTTTGCCTTTGGAGTTCA

TTTTTTCCTCTGATTTAGTTAATTATAATCCTGTTATTGGAGTCTACTTTGAATATCTCTTTTGAAGTTC

CTGAGATGTAAGTTTACCCTGTAGACAGAAACGTGGCTTTGAAGAATGACCTTGGACGAGTGTCGTGCAA

GTTATTCTGATGATCCTAAACTTGTACAAGTTTCAAGACATACTATCTTGACTAAGTTCTCTTCTAAATT

ATATAGTAGACTATCTCCTTGTAACTGGCTTGTGACTTGGATTTTCCAATTCAACGATCTATCTGGAGTT

CTATACTAAATAATGTTTCTAACATTGATTTCAGATGTATTCTGATGTAGTAATTGTTCTTTTGTAATTG

CTCCATTACAAAAAATACTTATATTATCACAAATTTCCTAGGTGGACAATATCAGCAAAGCTCTTCATAA

GT

>Sabal_bermudana_EU215512.1

TTATTGAGTGCATCATGGGAAAGGTTGCTGCCCATATGGGAAAGGAGGGAGATGCTACTCCCTTTACTGA

TGTCACAGTAAGTAAGTCCATAAAACCTTCATGCAAGCTTTACCTGAGAAGCATGTCACAATTTAACTTG

CTATAGATGCTGGAGCTTTTTTGCTTATTCAGTTTAAGAAAGTGTCTGGTGTTAAAGAACATTGAGGCAA

GTTTCTTTTGTTCTGCTCCTGGAGGAAGTAGTTTTTGCATGTCTAGCTAAAAGACATCGTTGATAGCTTA

TACAAAAGCAGAAAGTTATTCAATTTGCATTACCATTAAGGATTTGTACTAGATATGGTTTAATAAATAT

GTTAAATATGTTTGACAACTGTCACCCTGCCCCCTCCCCGCACACCATCTCTCCCCCGCCCCCCCCGCCG

CGGTGGCTTGACTAGAACCTTTCAACTCCATACCTGAATTTGTTTTTTTTTGTTTTTTGTTTTTGTTTTT

GTTTTTTTTTTTTTTTGAGGGGAGCTTTTGCCTTTGGAGTTCATTTTTTCCTCTGATTTAGTTAATTATA

ATCCTGTTATTGGAGTCTACTTTGAATATCTCTTTTGAAGTTCCTGAGATGTAAGTTTACCCTGTAGACA

GAAACATGGCATTGAAGAATGACCTTGGACGAGTGTCTTGCAAGTTATTCTGATGATCCTAAACTTGTAC

AAGTTTCAAGACATACTATCTTGACTAAGGTTTCCTCTAAATTATATAATAGACTATCTCCTTGTAACTG

GCCTGTGACTTGGATTTTCCAATTCAATGGTCTATCAGGAGTTCTATCCTAAATAATGTTTCTAACATTG

ATTTCAGATGTATTCTGATGTAGTAATTGTTCTTTTGTAATTGCCCCATTACAAAAAATACTTATATTAT

CACAAATTTCCTAGGTGGACAATATCAGCAAANGCCTTCANAAGTGTGGAGANCA

>Schippia_concolor_EU215486.1

TTATTGAGTGCATCATGGGAAAGGTTGCTGCACATATGGGAAAAGAGGGAGATGCTACTCCTTTTACTGA

TGTCACAGTAAGTAAATCCATAAAACCTTCATGTGAGCTTTACCTGTGAAGCACGTCACAATTTAACTTG

CTATAGATGCTGGAGCTTGTTTGCTTATTCAGTTTAAGAATCTGAGGCACTGTGTCTGGTGTTAAAGAAC

ATTAAGGAAAGTTTCTTTTGTTCTGCTCCAGGAGGAAGTAGTTTTTGCACGTCTAGCTAAAAGACATTGT

TGATAGCTTATACAAAACAGAGGGTTATTCAATTTGCATTACCATTAAGGATTTGTACTAGTATGGTTCA

ATAAATATGTTAAATATGTTTGAGAACTGGAGCTTTTGCCTTTGGAGCTCATTTTTTCCTCTGGTTTACT

TAATTCTAATCCTGTTATTTGAGTCTACTTTGAATATCTCTTTTGAAGTTCTTGAGAGGTAAGTTTACCC

TGTAGACAGAAACGTGGCTTTGAAGAATGACCTTGGATGAGTGTCTTGTAAGTTATTCTGATGATCCTAA

ACTTGTACAAGTTTCGAGGCATACTATCTTGACTAAGGTCTCTTCTAAATTATATAGTGATGATCCTAAA

CTTGTACAAGTTTCAAGGCATACTATCTTGACTAAGGTCTCTTCTAAATTATATAGTGATGATCCTAAAC

TTGTGCAAGTTTCAAGGCATACTATCTTGACTAAGGTCTCTTCTAAATTATATAGTAGAGTATCTCCTCG

TAACTAGCCTGTGACTTGGATTTTCCTATTCAATGATCTATCAGGAGTTCTATCCTAAATAATGTTTCTA

ACGTTGATTTCAGATGTATTCTGATGTAGGAGTTTAATATGTAATTGTTCTTTTGTAATTGCCCCATTAC

AAAAAATACTTATATTATCAGGTGGACAATATCAGCAAGGCTCTTCATAAGTGTGGATATCAGA

>Serenoa_repens_HQ720585.1

CTACTCCTTTTACTGATGTCACAGTAAGTAAATACATAAAACCTTCATGCAAGCTTTACCTGAGAAGCGT

GTCATAATTTAACCTGCTATAAATGCTGGAGCTTTTTTGCTTATTCAGTTTAAGAATTCCGAGTCACTGT

ATCTGATGTTAAAGAACATTAAGGAAAGTTTCTTTTGTTCTGCTCCAGGAGGAAGTAATTTTTGCATTTG

TCTAGCTAAAAGACATTGTTGATAGCTTATACAAAAACAGAAGGTTATTCAATTTTCATTACCATTAAGG

ATTTGTATTAGATATGGTTTAGTTAATTATAATCCTTCCAACTTCATACCTGGATTTGATTTTCTTTTGA

GGGGAGCTTTTGCCTTTGGAGTTCATATTTTCCTCTGGTTTAGTTAATTATAATCCTGTTATTTGAAGTC

TACTTTGAATCTCTTTTGAAGTTTTTGAGATGTAAGCTTATCCTGTAGACAGAAACGTGGCTTTGAAGAA

TGACTTTGGACGAGTGTAAGTTATTCTGATGATCCTAAACTTGTATAAGTTTCAAGACATACTATTTTGA

CTAAGGTCTCTTCTAAATTATATAGTAGACTATCTCCTTGTAACTGGCCTGTGACTTGGATTTTCCAATT

TGATGATCTATCAGGAGTTCTATCCTAAATAATGTTTCTAACATTGATTTCAGATGCATTCTGATATAGA

AGGTCAATATGTAATTGTTCTTTTGTAATTTCCCCATTACAAAAAATACTTATATCATCACAAATTTCCT

AGGTGGACNAATATAAGCAAAGCTCTTCATAAGT

>Serenoa_repens_HQ720586.1

GAAAGGTTGCTGCCCATATGGGAAAGGAGGGAGATGCTACTCCTTTTACTGATGTCACAGTAAGTAAATA

CATAAAACCTTCATGCAAGCTTTACCTGAGAAGCGTGTCATAATTTAACCTGCTATAAATGCTGGAGCTT

TTTTGCTTATTCAGTTTAAGAATTATGAGTCACTGTATCTGATGTTAAAGAACATTAAGGAAAGTTTCTT

TTGTTCTGCTCCAGGAGGAAGTAATTTTTGCATTTGTCTAGCTAAAAGACATTGTTGATAGCTTATACAA

AAACAGAAGGTTATTCAATTTTCATTACCATTAAGGATTTGTATTAGATATGGTTTAGTTAATTATAATC

CTTCCAACTTCATACCTGGATTTGATTTTCTTTTGAGGGGAGCTTTTGCCTTTGGATTTCATATTTTCCT

CTGGTTTAGTTAATTATAATCCTGTTATTTGAAGTCTACTTTGAATATCTCTTTTGAAGTTGTTGAGATG

TAAGTTTACCCTGTAGACAGAAACGTGGCTTTGAAGAATGACTTTGGACGAGTGTAAGTTATTCTGATGA

TCCTAAACTTGTGTAAGTTTCAAGACATACTATTTTGACTAAGGTCTCTTCTAAATTATATAGTAGACTA

TCTCCTTGTAACTGGCATGTGACTTGGATTTTCCAATTCGATGATCTCTCAGGAGTTCTATCCGAAATAA

TGTTTCTAACATTGATTTCAGATGCATTCTGATATAGGAGGTCAATATGTAATTGTTCTTTTGTAATTTC

CCCATTACAAAAAATACTTATATCATCACAAATTTCCTAGGTGGACAATATCAGCAAAGCTCTTCATAAG

T

>Serenoa_repens_EU215494.1

TTATTGAGTGCATCATGGGAAAGGTTGCTGCCCATATGGGAAAGGAGGGAGATGCTACTCCTTTTACTGA

TGTCACAGTAAGTAAATACATAAAACCTTCATGCAAGCTTTACCTGAGAAGCGTGTCATAATTTAACCTG

CTATAAATGCTGGAGCTTTTTTGCTTATTCAGTTTAAGAATTATGAGTCACTGTATCTGATGTTAAAGAA

CATTAAGGAAAGTTTCTTTTGTTCTGCTCCAGGAGGAAGTAATTTTTGCATTTGTCTAGCTAAAAGACAT

TGTTGATAGCTTATACAAAAACAGAAGGTTATTCAATTTTCATTACCATTAAGGATTTGTATTAGATATG

GTTTAGTTAATTATAATCCTTCCAACTTCATACCTGGATTTGATTTTCTTTTGAGGGGAGTTTTTGCCTT

TGGATTTCATATTTTCCTCTGGTTTAGTTAATTATAATCCTGTTATTTGAAGTCTACTTTGAATATCTCT

TTTGAAGTTGTTGAGATGTAAGTTTACCCTGTAGACAGAAACGTGGCTTTGAAGAATGACTTTGGACGAG

TGTAAGTTATTCTGATGATCCTAAACTTGTGTAAGTTTCAAGACATACTATTTTGACTAAGGTCTCTTCT

AAATTATATAGTAGACTATCTCCTTGTAACTGGCATGTGACTTGGATTTTCCAATTCGATGATCTCTCAG

GAGTTCTATCCGAAATAATGTTTCTAACATTGATTTCAGATGCATTCTGATATAGGAGGTCAATATGTAA

TTGTTCATTTGTAATTTCCCCATTACAAAAAATACTTATATCATCACAAATTTCCTAGGTGGACAATATC

AGCAAAGCTCTTCATAAGTGTGGATATCAGA

>Socratea_exorrhiza_AY543108.1

ATTGCAGGAGATGCTACTCCTTTTACTGATGTCACAGTAAGTAAATCCTTAGAACCTTCATGCAAGCTTT

ACCTGAGAAGCACGTCACAATTTAACTTGCTATAAAATGCTGGAGCTTTTTTGCTTATTCAGTTTAAGAA

TTCTGAGGCACTGTATCTGAGGTTAAAGAACGTTAAGGAAAGTTTCTTTTGTTCTGCTCCAGGAGGAAGT

AATTTTTGCATATGTCTAGCTAAAAGACATTGTTGATAGCTTATACAAAAACACATGATTATTCAATTTG

CATTACCATTTAGGATTTGTACTAGATATGGTTTAATAAATGTGTTTGAGAACTGTTACCCCCCCCTCCG

GTCTAGAACCTTCCAACTTCATACCTGGATTTGATATTTTTAGAGGGGAGCTTTTGCCTTTGGGGTTCAT

ATTTTCCTCTGGATTAGCTAATTATAATCCTGTTATTTGAAGTCTACTTTGAATCTCTCTTTTGAAGTTC

TTGATATGTAAGTTTACCGTGTAGACAGAAACATGGCTTTGAAGAATGACCTTGGACGAGTGTCTTGTAA

GTTATTCTGAAGATCCTGCACGTGTATAAGTTTCAAGACATATTATTTCGACTAAGGTCTAAGGTCTCTC

CTAAATTATATTGTAGACTATCTCCTTGTAACTGACCTGTGACTTGGATTTTCCAATTCAATGATCTATC

AGGAGTCCTATCCTAAATAATGTTTCGAACATTGATTTCAGATATATTCTGATGTAGGAGTTTAATATGT

AATTGTACTTTTGTAATTGTCCCATTACAAAAAATGCTGATATTATCACAAATTTCTAGGTGGACAAT

>Syagrus_smithii_HQ265666.1

TCMCAATTTTACCTGCTATAAGGGCTGGAGCTTGTTTGCTTATTCACTCTAAGAATTCCAGGTCACTGTA

TCTGATGCTTAAAGAACATCATGGAAAATTTCTTTTGTTCTGCTCGAGGAGGAAGTAAGTATTGCATGTG

TCTGGCTAAATGATGTAGTTAGCTCATACAAAAATGCATGGTTATTCAATTTGCATTACCATTAAGGATT

TGTACCAGATATGGTTTAATTAATATGTTTGAGTTGCATTACCATTAAGGATTTGTACTAGATATGGTTT

AATAAATATGGTTGAGTTGCATTGCCCAAGATGCAGGGGGATGTTGTATAAAACCCTTCAACTTCATACC

TGAATTTTTTTTTTTTTTTGGTGGGGGAGGGGGTGTGGGGGAGCTTTTGCCTTTGGAGTTCATATTTTCC

CCTGGATTAGTTTATTATAATCCTGGTATTTGAAGTCTACTTTGAATATCTCTTTTGAAGTTGATGATAT

GTTAGTTTACCCTGTAGACAGAAACATGGCTCTGAAGATTGACCTTGGACGAGTGTCTTGAAAGTTATTC

TGATGATCCTAAACTTGTATAAGTTTCAAGACGTACTATTTTGACTAAGGTCTCTCCTAAATTATGTCAT

TGACTATCTCCTNGTAATTGACCTGTGGCTTGGATTTTCCAATTCAATGATCTATCAGGGTTCTATCCTA

AATAATGTTTCTAACATTGATTTCAGATATATTCTGATGTAGGAGTTTAACATGTAATTGTACTTTTGTA

GTTGCTCCATTACAAAAATGCTTATGTTATCACAAATTCTC

>Syagrus_smithii_AY779378.1

TGCCTTTAGAGTTCATATTTTCCCCTGGATTAGTTTATTATAATCCTGGTATTTGAAGTCTACTTTGAAT

ATCTCTTTTGAAGTTGATGATATGTTAGTTTACCCTGTAGACAGAAACATGGCTCTGAAGATTGACCTTG

GACGAGTGTCTTGAAAGTTATTCTGATGATCCTAAACTTGTATAAGTTTCAAGACGTACTATTTTGACTA

AGGTCTCTCCTAAATTATGTCATTGACTATCTCCTTGTAATTGACCTGTGGCTTGGATTTTCCAATTCAA

TGATCTATCAGGGTTCTATCCTAAATAATGTTTCTAACATTGATTTCAGATATATTCTGATGTAGGAGTT

TAATATGTAATTGTACTTTTGTAGTTGCTCCATTACAAAAATGCTTATGTTATCACAAATTTCCTAGGTG

GACAATATCAGCAAAG

>Synechanthus_fibrosus_EF491143.1

CTACTCCTTTTACTGATGTCACAGTAAGTAAATCCATAGAACCTTCTTGCAAGCTTTACCTGAGAAGCAT

CTCGCAATTTAACTTGCTATGCTGGAACTTTTTTGCGTATTCAGTTTAAGAATTCTGAGGCACTGTATCT

GATGTTAAAGAAGATTACGGAAAGCTTCGTTTGTGCTGCTCCATGAGGAAGTAATTTTGCATATGTCTAG

CTAAAAGATGTTGATTGCTTATACAAAAACACATGGTTATTCAATTTGCATTACTGTTAAGGATTTGTAC

TACATATGGTTTGATAAATATGTTTGAGCACTGTTACCCACCCACACCCCCTTCCCTCTCTCTTTCTGGA

ACCTTTCAACTTCTTACCTGGATTTGATTTTTTTTGTGGGGAGTTTTTTCCTTTTGAGTTCATATTTTTC

CTCTGGATTAGTTTATTATAATCCTATTATTTGTAGTCTGCTTTGAATATCTCTTTTGAAGTTCTTGCTG

TGTAAGTTCACCCTGTAGACAGAAACATGGCTTTGAAGAATGACCTTGACAAGTGTCTTGTAAGTTATTC

TGATGATCTTAAACTTGTATAAGTTTCAAGACTTACTATTTTGACTAAGGTCTCTCCTAAATTATATAGT

GGACTATCTCCTTGTAATTGACCTTTGACTTGGATTTTCCAAGTCAATGATCTATCAGGAGTTCTATCCT

AAATAATGTTTCTAACATTGATTACAGATCTATTCTGATGTAGGAGTTTAATATGTAATTGTACTTTTGT

AATTGCCCTAT

>Synechanthus_warscewiczianus_EF491144.1

CTACTCCTTTTACTGATGTCACAGTAAGTAAATCCATAGAACCTTCTTGCAAGCTTTACCTGAGAAGCAT

CTCGCAATTCAACTTGCTATGCTGGAACTTTTTTGCTTATTCAGTTTAAGAATTCTGAGGCACTATATCT

GATGTTAAAGAAGATTACGGAATGCTTCGTTTGTGCTGCTCCATGAGGAAGTAATTTTGCATATGTCTAT

CTAAAAGACGTTGATAGCTTATACAAAAACACATGGTTATTCAATTTTCATTACTGTTAAGGATTTGTAC

TAGATATGGTTTAATAAATATGTTTGAGCACTGYTACCCGCCCCCCCCCCCCACCCCCTTCCCTCTCACT

CTGTCTGGAACCTTTCAACTTCTTACCTGGATTTGATTTTTTTTTAGGGGAGTTTTTTCCTTTCGAGTTC

ATATTTTTCTTCTGGATTAGTTAATTATAATCCTATTATTTGTAGTCTGCTTTGAATATCTCTTTTGAAG

TTCTTGCTGTGTAAGTTCACCCTGTAGACAGAAACATGGCTTTGAAGAATGACCTTGGACAAGTGTCTTG

TAAATTATTCTGATGATCCTAAACTTGTATAAGTTTCAAGACTTACTATTTTGACTAAGGTCCCTCCTAA

ATTATATAGTAGACTATCTCCTTGTAATTGACCTTTGACTTGGATTTTCCAAGTCAATGATCTATCAGGA

GTTCTATCCTAAATAATGTTTCTAACATTGATTACAGATCTATTCTGATGTAGGAGTTTAATATGTAATT

GTACTTTTGTAATTGCCCTAT

>Synechanthus_warscewiczianus_DQ177758.1

CTACTCCTTTTACTGATGTCACAGTAAGTAAATCCATAGAACCTTCTTGCAAGCTTTACCTGAGAAGCAT

CTCGCAATTCAACTTGCTATGCTGGAACTTTTTTGCTTATTCAGTTTAAGAATTCTGAGGCACTATATCT

GATGTTAAAGAAGATTACGGAATGCTTCGTTTGTGCTGCTCCATGAGGAAGTAATTTTGCATATGTCTAT

CTAAAAGACGTTGATAGCTTATACAAAAACACATGGTTATTCAATTTTCATTACTGTTAAGGATTTGTAC

TAGATATGGTTTAATAAATATGTTTGAGCACTGYTACCCGCCCCCCCCCCCCACCCCCTTCCCTCTCACT

CTGTCTGGAACCTTTCAACTTCTTACCTGGATTTGATTTTTTTTTAGGGGAGTTTTTTCCTTTCGAGTTC

ATATTTTTCTTCTGGATTAGTTAATTATAATCCTATTATTTGTAGTCTGCTTTGAATATCTCTTTTGAAG

TTCTTGCTGTGTAAGTTCACCCTGTAGACAGAAACATGGCTTTGAAGAATGACCTTGGACAAGTGTCTTG

TAAATTATTCTGATGATCCTAAACTTGTATAAGTTTCAAGACTTACTATTTTGACTAAGGTCCCTCCTAA

ATTATATAGTAGACTATCTCCTTGTAATTGACCTTTGACTTGGATTTTCCAAGTCAATGATCTATCAGGA

GTTCTATCCTAAATAATGTTTCTAACATTGATTACAGATCTATTCTGATGTAGGAGTTTAATATGTAATT

GTACTTTTGTAATTGCCCTATTA

>Thrinax_ekmaniana_EU215509.1

TTATTGAGAGCATCATGGGAAAGGTTGCTGCCCATATGGGAAAAGAGGGAGATGCTACTCCTTTTACTGA

TGTCACAGTAAGTAAATCCATAAAACCTTCATGTGAGCTTTACCTGTGAAGCATCTCACAATTTAACTTG

CTATAGATGCTGGAGCTTGTTTGCTTATTCAGTTTAAGAATTCCGAGGCACTGTATCTGATGTTAAAGAA

CATTAAGGAAAGTTTCTTTTGTTCTGCTCCAGGAGGAAGTAGTTTTTGCAGATGTCTAGCTAAAAGACAT

TGTTGATAGCTTATACAAAACAGAAGGTTATTCAATTTGCATTACCATTAAGGATTTGCACTAGATATGG

TTCAATAAATATGTTAAATATGTTTGGAGCTCTTGCCTTTGGAGTTCATTTTTTCCTCTGGTTTACTTAA

TTCTAATCCTGTTATTTGAGTCTACCTTGAATATCTCTTTTGAAGTTCTTGAGATGTAAGTTTACCCTGT

AGACAGAAACGTGGCTTTGAAGAATGACCTTCGATGAGTGTCTTGTAAGTTATTGTGAGGATCCTAAACT

TGTTCAAGTTTCAAGACATACTGTCTTGACTAAGGTCTCTTCTAAATTATATAGTAGACTATCTCCTCGT

AACCGGCCTGTGACTTGGATTTTCAATTCAATGATCTATCAGGAGTTCTATCCTAAATAATGTTTCTAAC

ATTGATTTCAGATGTATTCTGATGTAGGAGTTTAATATGTAATTGTTCTTCTGTAATTGCCCCATTACGA

AAAACACTTATTTTATCAGGTGGACAATATAAGCAAAGCTCTTCATAAGTGTGGATATCAGA

>Thrinax_radiata_EU215495.1

TTATTGAGTGCATCATGGGAAAGGTTGCTGCCCATATGGGAAAAGAGGGAGATGCTACTCCTTTTACTGA

TGTCACAGTAAGTAAATCCATAAAACCTTCATGTGAGCTTTACCTGTGAAGCATGTCACAATTTAACTTG

CTATAGATGCTGGAGCTTGTTTGCTTATTCAGTTTAGGAATTCTGAGGCACTGTGTCTGATGTTAAAGAA

CATTAAGGAAAGTTTCTTTTGTCCTGCCCCAGGAGGAAGTAGTTTTTGCATATGTCTAGCTAAAAGACAT

TGTTGATAGCTTATACAAAACAGAAGGTTATTCAATTTGCATTACCATTAAGGATTTGTACTAGATATGG

TTCAATAAATATGTTAAATATGTTTGAGAACTGGAGCTTTTGCCTTTGGAGTTCATTTCTTCCTCTGGTT

TACTTAATTCTAATCCTGTTATTTGAGTCTACTTTGAATATCTCTTTTGAAGTTCTTGAGATGTAAGTTT

ACCCTGTAGACAGAAACGTGACTTTGAAGAATGACCTTGGATGAGTGTCTTGTAAGTTATTCTGATGATC

CTAAACTTGTACAAGTTTCAAGACATACTATCTTGACTAAGGTCTCTTCTAAATTATATAGTAGACTCTC

TCCTCGTAACTGGCCTGTGACTTGGATTTTCCAATTCAATGATCTACCAGGAGTTCTATCCTAAATAATG

TTTCTAACATTGATTTCAGATGTATTCTGATGTAGGAGTTTAATATGTAATTGTTCTTTTGTAATTGCCC

CATTACAAAAAATACTTATATTATCAGGTGGACAATATCAGCAAAGCTCTTCATAAGTGTGGATATCAGA

>Thrinax_excelsa_EU215489.1

TTATTGAGTGCATCATGGGAAAGGTTGCTGCCCATATGGGAAAAGAGGGAGATGCTACTCCTTTTACTGA

TGTCACAGTAAGTAAATCCATAAAACCTTCATGTGAGCTTTACCTGTGAAGCATGTCACAATTTAACTTG

CTATAGATGCTGGAGCTTGTTTGCTTATTCAGTTTAAGAATTCTGAGGCACTGTGTCTGATGTTAAAGAA

CATTAAGGAAAGTTTCTTTTGTCCTGCCCCAGGAGGAAGTAGTTTTTGCATATGTCTAGCTAAAAGACAT

TGTTGATAGCTTATACAAAACAGAAGGTTATTCAATTTGCATTACCATTAAGGATTTGTACTAGATATGG

TTCAATAAATATGTTAAATATGTTTGAGAACTGGAGCTTTTGCCTTTGGAGTTCATTTCTTCCTCTGGTT

TACTTAATTCTAATCCTGTTATTTGAGTCTACTTTGAATATCTCTTTTGAAGTTCTTGAGATGTAAGTTT

ACCCTGTAGACAGAAACGTGGCTTTGAAGAATGACCTTGGATGAGTGTCTTGTAAGTTATTCTGATGATC

CTAAACTTGTACAAGTTTCAAGACATACTATCTTGACTAAGGTCTCTTCTAAATTATATAGTAGACTCTC

TCCTCGTAACTGGCCTGTGACTTGGATTTTCCAATTCAATGATCTATCAGGAGTTCTATCCTAAACAATG

TTTCTAACATTGATTTCAGATGTATTCTGATGTAGGAGTTTAATATGTAATTGTTCTTTTGTAATTGCCC

CATTACAAAAAATACTTATATTATCAGGTGGACAATATCAGCAAAGCTCTTCATAAGTGTGGATATCAGA

>Thrinax_excelsa_EU215487.1

TTATTGAGTGCATCACGGGAAAGGTTGCTGCCCATATGGGAAAAGAGGGAGATGCTACTCCTTTTACTGA

TGTCACAGTAAGTAAATCCATAAAACCTTCATGTGAGCTTTACCTGTGAAGCATGTCACAGTTTAACTTG

CTATAGATGCTGGAGCTTGTTTGCTTATTCAGTTTAAGAATTCTGAGGCACTGTGACTGATGTTAAAGAA

CATTAAGGAAAGTTTCTTTTGTCCTGCCCCAGGAGGAAGTAGTTTTTGCATATGTCTAGCTAAAAGACAT

TGTTGATAGCTTATACAAAACAGAAGGTTATTCAATTTGCATTACCATTAAGGATTTGTACTAGATATGG

TTCAATAAATATGTTAAATATGTTTGAGAACTGTCACAACACCCCCCCAACACCCGCCTGGCATGGCGCC

GTGTGCGCGGTGGCTTGTTTAGAACTTTTTTTCAACTTCATACCTGGATTATTATTATTATTATTATTAT

TATTATTATTATTATTATTATTGAGGGGAGCTTTTGCCTTTGGAGTTCATTTCTTCCTCTGGTTTACTTA

ATTCTAATCCTGTTATTTGAGTCTACTTTGAATATCTCTTTTGAAGTTCTTGAGATGTAAGTTTACCCTG

TAGACAGAAACGTGGCTTTGAAGAATGACCTTGGATGAGTGTCTTGTAAGTTATTCTGATGATCCTAAAC

TTGTACAAGTTTCAAGACATACTATCTTGACTAAGGTCTCTTCTAAATTATATAGTAGACTCTCTCCTCG

TAACTGGCCTGTGACTTGGATTTTCCAATTCAATGATCTATCAGGAGTTCTATCCTAAATAATGTTTCTA

ACATTGATTTCAGATGTATTCTGATGTAGGAGTTTAATATGTAATTGTTCTTTTGTAATTGCCCCATTAC

AAAAAATACTTATATTATCAGGTGGACAATATCAGCAAAGCTCTTCATAAGTGTGGATATCAGA

>Thrinax_parviflora_subsp.parviflora_EU215496.1

TTATTGAGTGCATCATGGGAAAGGTTGCTGCCCATATGGGAAAAGAGGGAGATGCTACTCCTTTTACTGA

TGTCACAGTAAGTAAATCCATAAAACCTTCATGTGAGCTTTACCTGTGAAGCATGTCACAATTTAACTTG

CTATAGATGCTGGAGCTTGTCTGCTTATTCAGTTTAAGAATTCTGAGGCACTGTGTCTGATGTTAAAGAA

CATTAAGGAAAGTTTCTTTTGTCCTGCCCCAGGAGGAAGTAGTTTTTGCATATGTCTASCTAAAAGACAT

TGTTGATAGCTTATACAAAACAGAAGGTTATTCAATTTGCATTACCATTAAGGATCTGTACTAGATATGG

TTCAATAAATATGTTAAATATGTTTGAGAACTGGAGCTTTTGCCTTTGGAGTTCATTTCTTCCTCTGGTT

TACTTAATTCTAATCCTGTTATTTGAGTCTACTTTGAATATCTCTTTTGAAGTTCTTGAGATGTAAGTTT

ACCCTGTAGACAGAAACGTGGCTTTGAAGAATGACCTTGGATGAGTGTCTTGTAAGTTATTCTGATGATC

CTAAACTTGTACAYGTTTCAAGACATACTATCTTGACTAAGGTCTCTTCTAAATTATATAGTAGACTCTC

TCCTCGTAACTGGCCTGTGACTTGGATTTTCCAATTCAATGATCTATCAGGAGTTCTATCCTAAATAATG

TTTCTAACAYTGATTTCAGATGTATTCTGATGTAGGAGTTTAATATGTAATTGTTCTTTTGTAATTGCCC

CAYTACAAAAAATACTTATATTATCAGGTGGACAATATCAGCAAAGCTCTTCATAAGTGTGGATATCAGA

>Thrinax_radiata_EU215490.1

TTATTGAGTGCATCATGGGAAAGGTTGCTGCCCATATGGGAAAAGAGGGAGATGCTACTCCTTTTACTGA

TGTCACAGCAAGTAAATCCATAAAACCTTCATGTGAGCTTTGCCTGTGAAGCATGTCACAATTTAACTTG

CTATAGATGCTGGAGCTTGTTTGCTTATTCAGTTTAAGAATTCTGAGGCACTGTGTCTGATGTTAAAGAA

CATTAAGGAAAGTTTCTTTTGTCCTGCCCCTGGAGGAAGTAGTTTTTGCATATGTCTAGCTAAAAGACAT

TGTTGATAGCTTATACAAAACAGAAGGTTATTCAATTTGCATTACCATTAAGGATTTGTACTAGATATGG

TTCAATAAATATGTTAAATATGTTCGAGAACTGGAGCTTTTGCCTTTGGAGTTCATTTCTTCCTCTGGTT

TACTTAATTCTAATCCTGTTATTTGAGTCTACTTTGAATATCTCTTTTGAAGTTCTTGAGATGTAAGTTT

ACCCTGTAGACAGAAACGTGGCTTTGAAGAATGACCTTGGATGAGTGTCTTGTAAGTTATTCTGATGATC

CTAAACTTGTACAAGTTTCAAGACATACTATCTTGACTAAGGTCTCTTCTAAATTATATAGTAGACTCTC

TCCTCGTAGCTGGCCTGTGACTTGGATTTTCCAATTCAATGATCTATCAGGAGTTCTATCCTAAATAATG

TTTCTAACATTGATTTCAGATGTATTCTGATGTAGGAGTTTAATATGTAATTGTTNGTTAGTAATTGCCC

CATTACAAAAAATACTTATATTATCAGGTGGACAATATCAGCAAAGCTCTTCATAAGTGTGGATATCAGA

>Thrinax_morrisii_EU215493.1

TTATTGAGTGCATCATGGGAAAGGTTGCTGCCCATATGGGAAAAGAGGGAGATGCTACTCCTTTTACTGA

TGTCACAGTAAGTAAATCCATAAAACCTTCATGTGAGCTTTACCTGTGAAGCATGTCACAATTTAACTTG

CTATAGATTCTGGAGCTTGTTTGCTTATTCAGTTTAAGAAATCCGAGGCACTGTGTCTGATGTTAAAGAA

CATTAAGGAAAGTTTCTTCTGTTCTGCTCCAGGAGGAAGTAGTTTTTGCAGATGTCTAGCTAAAAGACAT

TGTTGATAGCTTATACAAAACAGAAGGTTATTCAATTTGCATTACCATTAAGGATTTGTACTAGATATGG

TTCAATAAATATGTTAAATATGTTTGAGAACTGGAGCTTTTGCCTTTGGAGTTCATTTTTTCCTCTGGTT

TACTTAATTCTAATCCTGTTATTTGAGTCTACTTTGAATATCTCTTTTGAAGTTCTTGAGATGTAGGTTT

ACCCTGTAGACAGAAACGTGGCTTTGAAGAATGACCTTGGATGAGTGTCTTGTAAGTTATTCTGATGATC

CTAAACTTGTACAAGTTTCAGGGCATACTGCCTTGACTAAGATCTCTTCTAAATTGTATAGTAGGCTATT

CCTTGTAACCGGCCTGTGACTTGGATTTTCCAATTCAATGATCTATCAGGAGTTCCATCCTAAATAATGT

TTCTAACATTGATTTCAGATGTATTCTGATGTAGGAGTTTAATATGTAATTGTTCTTTTGTAATTGCTCC

ATTACAAAAAATACATTTTTTAACAGGTGGACAATATAAGCAAAGCTCTTCATAAGTGTGGATATCAGA

>Thrinax_morrisii_EU215514.1

TTATTGAGTGCATCATGGGAAAGGTTGCTGCCCATATGGGAAAAGAGGGAGATGCTACTCCTTTTACTGA

TGTCACAGTAAGTAAATCCATAAAACCTTCATGTGAGCTTTACCTGTGAAGCATGTCACAATTTAACTTG

CTATAGATTCTGGAGCTTGTTTGCTTATTCAGTTTAAGAAATCCGAGGCACTGTGTCTGATGTTAAAGAA

CATTAAGGAAAGTTTCTTTTGTTCTGCTCCAGGAGGAAGTAGTTTTTGCAGATGTCTAGCTAAAAGACAT

TGTTGATAGCTTATACAAAACAGAAGGTTATTCAATTTGCATTACCATTAAGGATTTGTACTAGATATGG

TTCAATAAATATGTTAAATATGTTTGAGAACTGGAGCTTTTGCCTTTGGAGTTCATTTTTTCCTCTGGTT

TACTTAATTCTAATCCTGTTATTTGAGTCTACTTTGAATATCTCTTTTGAAGTTCTTGAGATGTAAGCTT

ACCCTGTAGACAGAAACGTGGCTTTGAAGAATGACCTTGGATGAGTGTCTTGTAAGTTATTCTGATGATC

CTAAACTTGTACAAGTTTCAAGGCATACTGCCTTGACTAAGATCTCTTCTAAATTGTATAGTAGACTATT

CCTTGTAACCGGCCTGTGACTTGGATTTTCCAATTCAATGATCTATCAGGAGTTCCATCCTAAATAATGT

TTCTAACATTGATTTCAGATGTATTCTGATGTAGGAGTTTAATATGTAATTGTTCGTTNGTAATTGCTCC

ATTACAAAAAATACATTTTTTAACAGGTGGACAATATAAGCAAAGCTCTTCATAAGTGTGGATATCAGA

>Washingtonia_robusta_HQ720593.1

AGGGAGATGCTACTCCTTTTACTGATGTCACAGTAAGTAAATACAGAAAACCTTCATGCAAGCTTTACCT

GAGAAGTGTGTCATAATTTAACCTGCTATAAATGCTGGAGCTTTTTTGCTTATTCAGTTTAAGAATTCCG

AGTCACTGTATCTGATGTTAAAGAACATTAAGGAAAGTTTCTTTTGTTCTGCTCCAGGAGGAAGTAATTT

TTTGCGTATGTCTAGCTAAAAGACATTGTTGATAGCTTATACAAAAACAGAAGGTTATTCAATTTTCATT

ACCATTAAGGATTTGTATTAGATATGGTTTAGTTAATTATAATCCTTTCAACTTCATACCTGGATTTGAT

TTTCTTTTGAGGGGAGCTTTTGCCTTTGGAGTTCATATTTTCCTCTGGTTTAGTTAATTATAATCCTGTT

ATTTGAAGTCTACTTTGAATATCTCTTTTGAAGTTCTTGAGATGTAAGTTTACCCTGTAGACAGAAACGT

GGCTTTGAAGAATGACTTTGGACGAGTGTAAGTTATTCTGATGATCCTAAACTTGTATAAGTTTCAAGGC

ATACTATTTTGACTAAAGTCTCTTCTAAATTATATAGTAGACTATCTCCTTGTAACTGGCCTGTGACTTG

GATTTTCCAATTCGATGATCTATCAGGAGTTCTATCCTAAATAATGTTTCTAACATTGATTTCAGATGCA

TTCTGATATAGGAGTTCAATATGTAATTGTTCTTTTGTAATTTCCCCATTACAAAAAATACTTATATTAT

CACAAATTTCCTAGGTGGACAATATC

>Washingtonia_filifera_HQ720591.1

GAAAGGTTGCTGCCCATATGGGAAAGGAGGGAGATGCTACTCCTTTTACTGATGTCACAGTAAGTAAATA

CAGAAAACCTTCATGCAAGCTTTACCTGAGAAGTGTGTCATAATTTAACCTGCTATAAATGCTGGAGCTT

TTTTGCTTATTCAGTTTAAGAATTCCGAGTCACTGTATCTGATGTTAAAGAACATTAAGGAAAGTTTCTT

TTGTTCTGCTCCAGGAGGAAGTAATTTTTTGCGTATGTCTAGCTAAAAGACATTGTTGATAGCTTATACA

AAAACAGAAGGTTATTCAATTTTCATTACCATTAAGGATTTGTATTAGATATGGTTTAGTTAATTATAAT

CCTTTCAACTTCATACCTGGATTTGATTTTCTTTTGAGGGGAGCTTTTGCCTTTGGAGTTCATATTTTCC

TCTGGTTTAGTTAATTATAATCCTGTTATTTGAAGTCTACTTTGAATATCTCTTTTGAAGTTCTTGAGAT

GTAAGTTTACCCTGTAGACAGAAACGTGGCTTTGAAGAATGACTTTGGACGAGTGTAAGTTATTCTGATG

ATCCTAAACTTGTATAAGTTTCAAGGCATACTATTTTGACTAAAGTCTCTTCTAAATTATATAGTAGACT

ATCTCCTTGTAACTGGCCTGTGACTTGGATTTTCCAATTCGATGATCTATCAGGAGTTCTATCCTAAATA

ATGTTTCTAACATTGATTTCAGATGCATTCTGATATAGGAGTTCAATATGTAATTGTTCTTTTGTAATTT

CCCCATTACAAAAAATACTTATATTATCACAAATTTCCTAGGTGGACAATATC

>Washingtonia_filifera_HQ720592.1

CTGCCCATATGGGAAAGGAGGGAGATGCTACTCCTTTTACTGATGTCACAGTAAGTAAATACAGAAAACC

TTCATGCAAGCTTTACCTGAGAAGTGTGTCATAATTTAACCTGCTATAAATGCTGGAGCTTTTTTGCTTA

TTCAGTTTAAGAATTCCGAGTCACTGTATCTGATGTTAAAGAACATTAAGGAAAGTTTCTTTTGTTCTGC

TCCAGGAGGAAGTAATTTTTTGCGTATGTCTAGCTAAAAGACATTGTTGATAGCTTATACAAAAACAGAA

GGTTATTCAATTTTCATTACCATTAAGGATTTGTATTAGATATGGTTTAGTTAATTATAATCCTTTCAAC

TTCATACCTGGATTTGATTTTCTTTTGAGGGGAGCTTTTGCCTTTGGAGTTCATATTTTCCTCTGGTTTA

GTTAATTATAATCCTGTTATTTGAAGTCTACTTTGAATATCTCTTTTGAAGTTCTTGAGATGTAAGTTTA

CCCTGTAGACAGAAACGTGGCTTTGAAGAATGACTTTGGACGAGTGTAAGTTATTCTGATGATCCTAAAC

TTGTATAAGTTTCAAGGCATACTATTTTGACTAAAGTCTCTTCTAAATTATATAGTAGACTATCTCCTTG

TAACTGGCCTGTGACTTGGATTTTCCAATTCGATGATCTATCAGGAGTTCTATCCTAAATAATGTTTCTA

ACATTGATTTCAGATGCATTCTGATATAGGAGTTCAATATGTAATTGTTCTTTTGTAATTTCCCCATTAC

AAAAAATACTTATATTATCACAAATTTCCTAGGTGGACAATATCAGC

>Welfia_regia_JQ417535.1

TTGCCTTTAGAGTTCATATTTTCCTTTGGATTAGTTAATTATAATCCTGTTATTTGAAGTCTACTTTAAA

TATCTCTTTTGAAGTTCTTGATATGTAAGTTTACCGTAAAGTGGCTTTGAAGAATGACTTTGGACGAGTG

TCTTGTAAGTTATTCTGATGATCCTAAACTTGTATAAAGTTTCAAGACATACTATTTTGACCGATCTCTC

TCCTAAATTATATAATAGACTCTCTCCTTGTAACTGACCTGTGACTTGGATTTTCCAGTTCAATGATCTA

TCAGGAGTTCTGTCCTAAATAATGTTTCTAACATTGATTTCAGATATATTCTGATGTAGGAGTTTAATAG

GTAATGGTACTTTTGTAATTTCCCTATTACAAAAAATGCTTATATTATCACAAATTTCCTAGGTGGAC

>Welfia_regia_AY779371.1

TTGCCTTTAGAGTTCATATTTTCCTTTGGATTAGTTAATTATAATCCTGTTATTTGAAGTCTGCTTTAAA

TATCTCTTTTGAAGTTCTTGATATGTAAGTTTACCGTAAAGTGGCTTTGAAGAATGACTTTGGACGAGTG

TCTTGTAAGTTATTCTGATGATCCTAAACTTGTATAAAGTTTCAAGACATACTATTTTGACCGATCTCTC

TCCTAAATTATATAATAGACTCTCTCCTTGTAACTGACCTCTGACTTGGATTTTCCAGTTCAATGATCTA

TCAGGAGTTCTGTCCTAAATAATGTTTCTAACGTTGATTTCAGATATATTCTGATGTAGGAGTTTAATAG

GTAATGGTACTTTTGTAATTTCCCTATTACAAAAAATGCTTATATTATCACAAATTTCCTAGGTGGACAA

TATCAGCAAAGCTCTTCATAAG

>Wendlandiella_gracilis_AJ830167.1

CATCATGGGAAAGGTTGCTGCCCATATGGGAAAGGAGGGAGATGCTACTCCTTTTACTGATGTCACAGTA

AGTAAATCCATAGAATCTTCTTGCAAGCTTTACCTGAGAAGCTTGTTGCAATATAACTTGCTATGCTGGA

ACTTTTTTTGCTTATTCAGTTTAAGAATTCGGAGGCACTGTATCTGATGTTAAAGAAGATTAAGGAAAGT

TTCTTTTGTGCTGCTCCAGAAGTAATTTTGCATGTATAGCTAAAAGACGTTGTCTATAGCTTATACAAAA

ACACATGGTTATTCAAATTGCATTACCATTAAGAATTTGTACTGGATATGGTTTAATAAATATGTTTGAG

AACTGTTATTGCCGCCCCTCCCCCCTCTCTCCCTTTTTCTCTGTCTAGAATCTTTCAACTTCTTACCTGG

ATTTGATTTTTTTAAGGGGAGCTTTTGCCTTTCAAGTTCATATTTTCCTCTGGATTAGTTAATTATAATC

CCATTATTTGTAGTCTGCTTTGAATCTCTTTTGAAGTTCTTGCTGTGTAAGTTTACTCTGTAGACAGAAA

CATGGCTTTGAAGAATGACCTTGGATGAGTGTCTTATAAGTTATTCTGATGATCCTAAACTTTTTTAAGT

TTCAAGACATACTATTTTGACTAAGGTCTCTCCTAAATTATATAGCAGACTATCTCCTTGTAATTGACCT

GTGACCTGGATTTTCCAAGTCAATGATCTATCAGGAGTTCTATCCTAAATAATGTTTCTAACGTTGATTT

CAGATATAATCTGATGTAGGAGTTTAATATGTAATTGTACTTTTGTAATTGCCCCATTACAAGATATGCT

TATATCATCAAAAATTTTCCTAGGTGGACAATATCAGCAAAGCTTCTTCCATAAGT

>Wendlandiella_gracilis_var.polyclada_EF491145.1

CTACTCCTTTTACTGATGTCACAGTAAGTAAATCCATAGAATCTTCTTGCAAGCTTTACCTGAGAAGCTT

GTTGCAATATAACTTGCTATGCTGGAACTTTTTTGCTTATTCAGTTTAAGAATTCGGAGGCACTGTATCT

GATGTTAAAGAAGATTAAGGAAAGTTTCTTTTGTGCTGCTCCAGAAGTAATTTTGCATGTATAGCTAAAA

GACGTTGTCTATAGCTTATACAAAAACACATGGTTATTCAAATTGCATTACCATTAAGAATTTGTACTGG

ATATGGTTTAATAAATATGTTTGAGAACTGTTATTGCCGCCCCTCCCCCCTCTCTCCCTTTTTCTCTGTC

TAGAATCTTTCAACTTCTTACCTGGATTTGATTTTTTTAAGGGGAGCTTTTGCCTTTCAAGTTCATATTT

TCCTCTGGATTAGTTAATTATAATCCCATTATTTGTAGTCTGCTTTGAATCTCTTTTGAAGTTCTTGCTG

TGTAAGTTTACTCTGTAGACAGAAACATGGCTTTGAAGAATGACCTTGGATGAGTGTCTTATAAGTTATT

CTGATGATCCTAAACTTTTTTAAGTTTCAAGACATACTATTTTGACTAAGGTCTCTCCTAAATTATATAG

CAGACTATCTCCTTGTAATTGACCTGTGACCTGGATTTTCCAAGTCAATGATCTATCAGGAGTTCTATCC

TAAATAATGTTTCTAACGTTGATTTCAGATATAATCTGATGTAGGAGTTTAATATGTAATTGTACTTTTG

TAATTGCCCCAT

>Wettinia_hirsuta_AJ830191.1

TCCTTTTACTGATGTCACAGTAAGTAAATCCATAGAACCTTCATGCAAGCTTTACCTGAGAAGCATGTCA

CAATTTAACTTGCTATAAATGCTGGAGCTTTTTTGCTTATTCAGTTTAAGAATTCTGAGGAACTGTATCT

GATGTTAAAGAACGTTAAGTTTCTTTTGTTCTGCTCCAGGAGGAAGTAATTTTTGCATATGTCTAGCTAA

AAGACATTGTTGATAGCTTGTACAAAAACACATGATTATTCAATTTGCATTACCATTAAGGATTTGTACT

AGGTATGGTTTAATAAATTTGTTTGAGAACTGTTACCCCCCCTTCCGGTCTAAAACCTTCCAATTGCATA

CGTGGATTTGATATTTTTTGAGGGGATCATATTTTCCTCTGGATTAGCTAATTATAATCCTGTTATTTGA

AGTCTACTTTGAATCTCTTTTGAAGTTCTTGATATGTAAGTTTACCGTGTAGACAGAAACATGGCTTTGA

AGAATGACCTTGGACGAGTGTCTTGTAAGTTATTCTGATGATCCTACACTTGTATAAGTTTCAAGACATT

ATTTTGACTAAGGCCTCTCCTACATTATATTGTAGACTATCTCCTTGTAACTGACCTGTGACTTGGATTT

TCCAATTCAATGATCTATCAGGAGTCCTATCCTAAATAATGTTTCTATCATTGATTTCAGATATATTCTG

ATGTAGGAGTTTAATATGCAATTGTACTTTTGTAATTGTCCCATTACAAAAAATGCTGATATCATCACAA

ATT

>Wettinia_hirsuta_AJ830192.1

TTTACTGATGTCACAGCTAAGTAAATCCATAGAACCTTCATGCAAGCTTTACCTGAGAAGCATGTCACAA

TTTAACTTGCTATAAATGCTGGAGCTTTTTTGCATATTCAGTTTAAGAATTCTGAGGAACTGTATCTGAT

GTTAAAGAACGTTAAGTTTCTTTTGTTCTGCTCCAGGAGGAAGTAATTTTTGCATATGTCTAGCTAAAAG

ACATTGTTGATAGCTTGTACAAAAACACATGATTATTCAATTTGCATTACCATTAAGGATTTGTACTAGG

TATGGTTTAATAAATTTGTTTGAGAACTGTTACCCCCCCTTCCGGTCTAAAACCTTCCAATTGCATACGT

GGATTTGATATTTTTTGAGGGGATCATATTTTCCTCTGGATTAGCTAATTATAATCCTGTTATTTGAAGT

CTACTTTGAATCTCTTTTGAAGTTCTTGATATGTAAGTTTACCGTGTAGACAGAAACATGGCTTTGAAGA

ATGACCTTGGACGAGTGTCTTGTAAGTTATTCTGATGATCCTACTCTTGTATAAGTTTCAAGACATTATC

TTGACTAAGGCCTCTCCTACATTATATTGTAGACTATCTCCTTGTAACTGACCTGTGACTTGGATTTTCC

AATTCAATGATCTATCAGGAGTCCTATCCTAAATAATGTTTCTATCATTGATTTCAGATATATTCTGATG

TAGGAGTTTAATATGCAATTGTACTTTTGTAATTGTCCCATTACAAAAAATGCTGATATCATCACAAATT

>Zombia_antillarum_EU215515.1

TTATTGAGTGCATCATGGGAAAGGTTGCTGCCCATATGGGAAAAGAGGGAGATGCTACTCCTTTTACTGA

TGTCACAGTAAGTAAATCCATAAAACCTTCATGTGAGCTTTATCTGTGAAGCATGTCACAATTTAACTTG

CTATAGATGCTGGAGCTTGTTTGCTTATTCAGTTCAAGAATTCCAAGGCACTGTGTCTGATGTTAAAGAA

CATTAAGGAAAGTTTCTTTTGTTCTGCTCCAGGAGGTAGTAATTTTTGCATATGTCTAGCTAAAAGACAT

TGTTGATAGCTTATACAAAACAGATGGTTATTCAATTTGCATTACCATTAAGGATTTGTACTAGATATGG

TTCAATAAATATGTTAAATATGTTTGAGAACTGGAGCTTTCGCCTTTGGAGTTCATTTTTTCCTCTGGTT

TTCTTAATTCTAATTTTCCTCTGGTTTACTTAATTCTAATCCTGTTATTTGAGACTACTTTGAATATCTC

TTTTGAAGTTCTTGAGATGTAAGTTTACCCTGTAGACAGAAACGTGGCTTTGAAGAATGACCTTGGATGA

GTATCTTGTAAGTTATTCTGATGATCCTAAACTTGTACAAGTTTCAAGACATACTATCTTGACTAAGGTC

TCTTCTAAATTATATAGTAGACCATCTCCTCGTAACTGGCCTGTGACTTGGATTTTCCAATTCAATGATC

TATCAGGAGTTCTATCCTAAATAATGTTTCTAACATTGATTTCAGATGTATTCTGATGTAGGAGTTTAAT

TAGTAATTGTTCTTTTGTAATTGCCCCATTACAAAAAATACTTATATTACCAGGTGGACAATATCAGCAA

AGCTCTTCATAAGTGTGGATATCAGA

>Zombia_antillarum_EU215497.1

TTATTGAGTGCATCATGGGAAAGGTTGCTGCCCATATGGGAAAAGAGGGAGATGCTACTCCTTTTACTGA

TGTCACAGTAAGTAAATCCATAAAACCTTCATGTGAACTTTATCTGTGAAGCATGTCACAATTTAACTTG

CTATAGATGCTGGAGCTTGTTTGCTTATTCAGCTTAAGAATTCCAAGGCACTGTGTCTGATGTTAAAGAA

CATTAAGGAAAGTTTCTTTTGTTCTGCTCCAGGAGGTAGTAATCTTTGCATATGTCTAGCTAAAAGACAT

TGTTGATAGCTTATACAAAACAGATGGTTATTCAATTTGCATTACCATTAAGGATTTGTACTAGATATGG

TTCAATAAATATGTTAAATATGTTTGAGGACTGGAGCTTTCGCCTTTGGAGTTCATTTTTTCCTCTGGTT

TTCTTAATTCTAATTTTCCTCTGGTTTACTTAATTCTAATCCTATTATCTGAGACTACTTTGAATATCTC

TTTTGAAGTTCTTGAGATGTAAGTTTACCCTGTAGACAGAAACGTGGCTTTGAAGAATGACCTTGGATGA

GTATCTTGTAAGTTATTCTGATGATCCTAAACTTGTACAAGTTTCAAGACATACTATCTTGACTAAGGTC

TCCTCTAAATTATATAGTAGACCATCTCCTCGTAACTGGCCTGTGACTTGGATTTTCCAATTCAGTGATC

TATCAGGAGTTCTATCCTAAATAATGTTTCTAACATTGATTTCAGATGTATTCTGATGTAGGAGTTTAAT

TAGTAATTGTTCTTTTGTAATTGCCCCATTACAAAAAATACTTATACTACCAGGTGGGCAATATCAGCAA

AGCTCTTCATAAGTGTGGATATCAGA
