# Supplementary material for: Adjacency and Area Explain Species Bioregional Shifts in Neotropical Palms
Source: Front Plant Sci. 2019 Feb 5;10:55. doi: 10.3389/fpls.2019.00055 (PMC6370682; doi:10.3389/fpls.2019.00055)
Supplement: Supplementary file 2 [file Data_Sheet_2.docx]

**Adjacency and area explain species bioregional shifts in Neotropical palms**

Cintia G. Freitas, Christine D. Bacon, Advaldo C. Souza-Neto, Rosane G. Collevatti

**Appendix S2.** GenBank numbers and sequences for the 337 species of American palms for RPB2 nuclear gene.

>Acoelorraphe_wrightii_HQ720242.1

AAATGTAAATGGAAGAATTACAAGGATATTTAGAAAGAGATAGATCTCTGCAACAACACTTTCTATATCC

GCTTCTCTTTAAGGAGTATATTTACACATTTCTTCATGATCGTGGTTTAAATGGTTCGATTTTTTACGAA

TCCACGGAAATTTTTGGTTATGACAATAAATCTAGTTCAGTACTTGTGAAACGTTCAATTATTCGAATGT

ATCGACAGAATTATTTGATTTATTCGGTTAATGATTCTAACCAAAATCGATTCGTTGGGCACAACAATTA

TTTTGATTTTCATTTTTATTCTCAGATGATATTGGAAGGTTTTGCAGTCATTGTGGAAATTCCATTCTTG

CTGCGATTGGTATCTTCCCTCGAAGAAAAAAAAATACCAAAATCTCAGAATTTGAATTTACGATCTATTC

ATTCAATATTTCCCTTTTTGGAGGACAAATTATCGCATTTAAATTATGTGTCAGATATACTAATACCTTA

TCCCATCCATCTGAAAATCTTGGTTCAAATCCTTCAATTCTGGATCCAAGATGTTCCTTCTTTACATTTA

TTGCGATTCTTTCTTCACGAATATCATAATTGGAATAGTCTTATTACTCCGAATAATTCTATTTTTCTTT

TTTCAAAAGAAAATAAAAGACTATTTCGGTTCCCATATAATTCTTATGTATCTGAATGCGAATTTGTATT

AGTTTTTCTTCGTAAACAATCTTCTTATTTACGATTAACATCTTCTGGAGCTTTTCTTGAGCGAACACAT

TTCTATGGAAAAATAGAATATCGTATAGTAGTGCGCCGTAATTATTTTCAGAAGAAGACCCTATGGTTTT

TCAAGGATCCCTTCATGCATTATGTTCGATATCAAGGAAAAGCAATTCTGGTTTCAAAGGGGACTCATCT

TCTGATGAAGAAATGGAAATGTCACCTTGTCAATTTCTGGCAATATTATTTTCACTTTTGGTCTCAACCG

TACAGGATTCATATAAACCAATTATCAAACTGTTCTTTCTATTTTCTAGGTTATCTTTCAAGTGTACTAA

TAAATCCTTCGGCGGTAAGGAATCAAATGCTAGAGAATTCATTTCTAATAGATACTGTTATTAAAAAATT

CGATACCAGAGTCCCAGTTATTCCTCTTATTAGATCATTGTCTAAAGCTAAATTTTGTACCGTATCGGGG

CATCCTATTAGTAAGCCGATCTGGACCGATTTATCAGATTGCGATATTATTGATCGATTTGGTCGGATAT

GTAGAAATCTTTCTCATTATCACAGTGGATCCTCAAAAAAACAGAGTTTGTATCGAATAAAGTATATACT

TCGGTTTTCGTGTGCTAGAACTTTGGCTCGTAAACATAAAAGTATGGTACGCGCTTTTTTGCAAAGATTA

GGTTCGGGATTATTAGAAGAATTTTTTATGGAAGAAGAACAAGTTGTTTCTTTGATCTTCCCAAAAACAA

CTTCTTTTTCTTTACATGAATCACATATAGAACGTATTTGGTATTTGGATATTATCCGTATCAATGACCT

GGTGAATTATTCATAATTGGTTTGTTGACGTGATGAGACTTATGAATAGAATAGAAATGATCTATAAATG

ATCAAGAGAGAAAAAAATTCATGAATTTTCATTCTGAAATGCTCATTGCAGTAGTGTAGTGGTTGAATCA

ACTGAGTAGTC

>Acoelorraphe_wrightii_HQ720241.1

GGAAGAATTACAAGGATATTTAGAAAGAGATAGATCTCTGCAACAACACTTTCTATATCCGCTTCTCTTT

AAGGAGTATATTTACACATTTCTTCATGATCGTGGTTTAAATGGTTCGATTTTTTACGAATCCACGGAAA

TTTTTGGTTATGACAATAAATCTAGTTCAGTACTTGTGAAACGTTCAATTATTCGAATGTATCGACAGAA

TTATTTGATTTATTCGGTTAATGATTCTAACCAAAATCGATTCGTTGGGCACAACAATTATTTTGATTTT

CATTTTTATTCTCAGATGATATTGGAAGGTTTTGCAGTCATTGTGGAAATTCCATTCTTGCTGCGATTGG

TATCTTCCCTCGAAGAAAAAAAAATACCAAAATCTCAGAATTTGAATTTACGATCTATTCATTCAATATT

TCCCTTTTTGGAGGACAAATTATCGCATTTAAATTATGTGTCAGATATACTAATACCTTATCCCATCCAT

CTGAAAATCTTGGTTCAAATCCTTCAATTCTGGATCCAAGATGTTCCTTCTTTACATTTATTGCGATTCT

TTCTTCACGAATATCATAATTGGAATAGTCTTATTACTCCGAATAATTCTATTTTTCTTTTTTCAAAAGA

AAATAAAAGACTATTTCGGTTCCCATATAATTCTTATGTATCTGAATGCGAATTTGTATTAGTTTTTCTT

CGTAAACAATCTTCTTATTTACGATTAACATCTTCTGGAGCTTTTCTTGAGCGAACACATTTCTATGGAA

AAATAGAATATCGTATAGTAGTGCGCCGTAATTATTTTCAGAAGAAGACCCTATGGTTTTTCAAGGATCC

CTTCATGCATTATGTTCGATATCAAGGAAAAGCAATTCTGGTTTCAAAGGGGACTCATCTTCTGATGAAG

AAATGGAAATGTCACCTTGTCAATTTCTGGCAATATTATTTTCACTTTTGGTCTCAACCGTACAGGATTC

ATATAAACCAATTATCAAACTGTTCTTTCTATTTTCTAGGTTATCTTTCAAGTGTACTAATAAATCCTTC

GGCGGTAAGGAATCAAATGCTAGAGAATTCATTTCTAATAGATACTGTTATTAAAAAATTCGATACCAGA

GTCCCAGTTATTCCTCTTATTAGATCATTGTCTAAAGCTAAATTTTGTACCGTATCGGGGCATCCTATTA

GTAAGCCGATCTGGACCGATTTATCAGATTGCGATATTATTGATCGATTTGGTCGGATATGTAGAAATCT

TTCTCATTATCACAGTGGATCCTCAAAAAAACAGAGTTTGTATCGAATAAAGTATATACTTCGGTTTTCG

TGTGCTAGAACTTTGGCTCGTAAACATAAAAGTATGGTACGCGCTTTTTTGCAAAGATTAGGTTCGGGAT

TATTAGAAGAATTTTTTATGGAAGAAGAACAAGTTGTTTCTTTGATCTTCCCAAAAACAACTTCTTTTTC

TTTACATGAATCACATATAGAACGTATTTGGTATTTGGATATTATCCGTATCAATGACCTGGTGAATTAT

TCATAATTGGTTTGTTGACGTGATGAGACTTATGAATAGAATAGAAATGATCTATAAATGATCAAGAGAG

AAAAAAATTCATGAATTTTCATTCTGAAATGCTCATTGCAGTAGTGTAGTGGTTGAATCAAC

>Acoelorraphe_wrightii_AM114579.1

GACCATATTGCACTATGTATCATTTGATAACCCAAAAAATGAAATAGGTCCCGCCTCTGGTTCAAGTAGA

AATGTAAATGGAAGAATTACAAGGATATTTAGAAAGAGATAGATCTCTGCAACAACACTTTCTATATCCG

CTTCTCTTTAAGGAGTATATTTACACATTTCTTCATGATCGTGGTTTAAATGGTTCGATTTTTTACGAAT

CCACGGAAATTTTTGGTTATGACAATAAATCTAGTTCAGTACTTGTGAAACGTTCAATTATTCGAATGTA

TCGACAGAATTATTTGATTTATTCGGTTAATGATTCTAACCAAAATCGATTCGTTGGGCACAACAATTAT

TTTGATTTTCATTTTTATTCTCAGATGATATTGGAAGGTTTTGCAGTCATTGTGGAAATTCCATTCTTGC

TGCGATTGGTATCTTCCCTCGAAGAAAAAAAAATACCAAAATCTCAGAATTTGAATTTACGATCTATTCA

TTCAATATTTCCCTTTTTGGAGGACAAATTATCGCATTTAAATTATGTGTCAGATATACTAATACCTTAT

CCCATCCATCTGAAAATCTTGGTTCAAATCCTTCAATTCTGGATCCAAGATGTTCCTTCTTTACATTTAT

TGCGATTCTTTCTTCACGAATATCATAATTGGAATAGTCTTATTACTCCGAATAATTCTATTTTTCTTTT

TTCAAAAGAAAATAAAAGACTATTTCGGTTCCCATATAATTCTTATGTATCTGAATGCGAATTTGTATTA

GTTTTTCTTCGTAAACAATCTTCTTATTTACGATTAACATCTTCTGGAGCTTTTCTTGAGCGAACACATT

TCTATGGAAAAATAGAATATCGTATAGTAGTGCGCCGTAATTATTTTCAGAAGAAGACCCTATGGTTTTT

CAAGGATCCCTTCATGCATTATGTTCGATATCAAGGAAAAGCAATTCTGGTTTCAAAGGGGACTCATCTT

CTGATGAAGAAATGGAAATGTCACCTTGTCAATTTCTGGCAATATTATTTTCACTTTTGGTCTCAACCGT

ACAGGATTCATATAAACCAATTATCAAACTGTTCTTTCTATTTTCTAGGTTATCTTTCAAGTGTACTAAT

AAATCCTTCGGCGGTAAGGAATCAAATGCTAGAGAATTCATTTCTAATAGATACTGTTATTAAAAAATTC

GATACCAGAGTCCCAGTTATTCCTCTTATTAGATCATTGTCTAAAGCTAAATTTTGTACCGTATCGGGGC

ATCCTATTAGTAAGCCGATCTGGACCGATTTATCAGATTGCGATATTATTGATCGATTTGGTCGGATATG

TAGAAATCTTTCTCATTATCACAGTGGATCCTCAAAAAAACAGAGTTTGTATCGAATAAAGTATATACTT

CGGTTTTCGTGTGCTAGAACTTTGGCTCGTAAACATAAAAGTATGGTACGCGCTTTTTTGCAAAGATTAG

GTTCGGGATTATTAGAAGAATTTTTTATGGAAGAAGAACAAGTTGTTTCTTTGATCTTCCCAAAAACAAC

TTCTTTTTCTTTACATGAATCACATATAGAACGTATTTGGTATTTGGATATTATCCGTATCAATGACCTG

GTGAATTATTCATAATTGGTTTGTTGACGTGATGAGACTTATGAATAGAATAGAAATGATCTATAAATGA

TCAAGAGAGAAAAAAATTCATGAATTTTCATTCTGAAATGCTCATTGCAGTAGTGTAGTGGTTGAATCAA

CTGAGTAGTCAAAATTATTATACTTTCTTCTCGGGACCCAAGTTTTATATTATACATAGGTAAAGTC

>Acrocomia_crispa_HQ265525.1

TGGGTCCTGCCTCTGGTTCAGTAGAAATGGAAATGGAAGAATTACAAGGATATTTAGAAAAAGATAGATC

TCGGCAACAACACTTTCTATATCCGCTTCTCTTTAAGGAGTATATTTACACATTTGCTCATGATCGTGGT

TTAAATGGTTCGATTTTTTACGAATCCACGGAAATTTTTGGTTATGACAATAAATCTAGTTCAGTACTTG

TGAAACGTTCAATTATTCGAATGTATCAACAGAATTATTTGATTTATTCGGTTAATGATTCTAACCAAAA

TCGATTCGTTGGGCACAACAATTATTTTTATTTTCATTTTTATTCTCAGATGATATTGGAAGGTTTTGCA

GTCATTGTGGAAATTCCATTCTTGCTGCGATTAGTATCTTCCCTCGAAGAAAAAAAAATACCAAAATCTC

AGAATTTGAATTTACGATCTATTCATTCAATATTTCCCTTTTTGGAGGACAAATTATCGCATTTAAATTA

TGTGTCAGATATACTAATACCTTATCCCATCCATCTGAAAATCTTGGTTCAAATCCTTCAATGCTGGATC

CAAGATGTTCCTTCTTTACATTTATTGCGATTCTTTCTTCACGAATATCATAATTGGAATAGTCTTATTA

CTCCGAATAATTCTATTTTTTTTTCAAAAGAAAATAAAAGACTATTTCGGTTCCCATATAATTCTTATGT

ATCTGAATGCGAATTTGTATTAGTTTTTCTTCGTAAACAATCTTCTTATTTACGATTAACATCTTCTGGA

GCTTTTCTTGAGCGAACACATTTCTATGGAAAAATAGAACATCTTATACTTATAGTAGTGCGCCGTAATT

ATTTTCAGAAGACCCTATGGTTCTTCAAGGATCCCTTCATGCATTATGTTCGATATCAAGGAAAAGCAAT

TCTGGTTTCAAAGGGGACTCATCTTCTGATGAAGAAATGGAAATGTCATCTTGTCAATTTCTGGCAATAT

TATTTTCACTTTTGGTCTCAACCGTACAGGATCCATATAAACCAATTATCAAGCTGTTCTTTCTATTTTC

TAGGTTATCTTTCAAGTGTACTAATAAATCCTTCGGCGGTAAGGAATCAAATGCTAGAGAATTCATTTCT

AATAGATACTGTTATTAAAAAATTCGATACCAGAGTCCCCGTTATTCCTCTTATTGGATCATTGTCTAAA

GCTAAATTTTGTACCGTATCGGGGCATCCTATTAGTAAGCCGATCTGGACCAATTTATCAGATTGCGATA

TTATTGATCGATTTGGTCGGATATGTAGAAATCTTTCTCATTATCACAGTGGATCCTCAAAAAAACAGAG

TTTGTATCGAATAAAGTATATACTTCGATTTTCGTGTGCTAGAACTTTGGCTCGTAAACATAAAAGTATG

GTACGCGCTTTTTTGCAAAGATTAGGTTCGGGATTATTAGAAGAATTCTTTACGGAAGAAGAACAAGTTG

TTTCTTTGATCTTCCCAAAAACAACTTCTTTTTCTTTACATGAATCACATATAGAACGTATTTGGTATTT

GGATATTATCCGTATCAATGACCTGGTGAATTATTCATAATGGGTTTGGTGACGTGATGAGACTTATGAA

TAGAATAGAAATGATCTATAAATGATCAAGAGAGAAAAAAATTCATGAATTTTCATTCTGAAATGCTCNT

TGCAGTAGTGTACGT

>Aiphanes_ulei_HQ265533.1

GACCATATTGCACTATGTATCATTTGATAACCCCAAAAATGAAATGGGTCCTGTCTCTGGTTCAAGTAGA

AATGGAAATGGAAGAATTACAAGGATATTTAGAAAAAGATAGATCTCGGCAACAACACTTTCTATATCCG

CTTCTCTTTAAGGAGTATATTTACACATTTGCTCATGATCGTGGTTTAAATGGTTCGATTTTTTACGAAT

CCACGGAAATTTTTGGTTATGACAATAAATCTAGTTCAGTACTTGTGAAACGTTTAATTATTCGAATGTA

TCAACAGAATTATTTGATTTATTCGGTTAATGATTCTAACCAAAATCGATTCGTTGGGCACAACAATTAT

TTTTATTTTCATTTTTATTCTCAGATGATATTGGAAGGTTTTGCAGTCATTGTGGAAATTCCATTCTTGC

TGCGATTAGTATCTTCCCWCGAAGAAAAAAAAATACCAAAATCTCAGAATTTGAATTTACGATCTATTCA

TTCAATATTTCCCTTTTTGGAGGACAAATTATCGCATTTAAATTATGTGTCAGATATACTAATACCTTAT

CCCATCCATCTGAAAATCTTGGTTCAAATCCTTCAATGCTGGATCCAAGATGTTCCTTCTTTACATTTAT

TGCGATTCTTTCTTCACGAATATCATAATTGGAATAGTCTTATTACTCCGAATAATTCTATTTTTTTTTC

AAAAGAAAATAAAAGACTATTTCGGTTCCCATATAATTCTTATGTATCTGAATGCGAATTTGTATTAGTT

TTTCTTCGTAAACAATCTTCTTATTTACGATTAACATCTTCTGGAGCTTTTCTTGAGCGAACACATTTCT

ATGGAAAAATAGAACATCTTATACTTATAGTAGTGCGCCGTAATTATTTTCAGAAGACCCTATGGTTCTT

CAAGGATCCCTTCATGCATTATGTTCGATATCAAGGAAAAGCAATTCTGGTTTCAAAGGGGACTCATCTT

CTGATGAAGAAATGGAAATGTCATCTTGTCAATTTCYGGCAAWATTATTTTCMCTTTTGGTCTCAACCGT

ACAGGATCCATATAAACCAATTATCAAGCTGTTCTTTCTATTTTCTAGGTTATCTTTCAAGTGTACTAAT

AAATCCTTCGGCGGTAAGGAATCAAATGCTAGAGAATTCATTTCTAATAGATACTGTTATTAAAAAATTC

GATACCAGAGTCCCTGTTATTCCTCTTATTGGATCATTGTCTAAAGCTAAATTTTGTACCGTATCGGGGC

ATCCTATTAGTAAGCCCATCTGGACCAATTTATCAGATTGCGATATTATTGATCGATTTGGTCGGATATG

TAGAAATCTTTCTCATTATCACAGTGGATCCTCAAAAAAACAGAGTTTGTATCGAATAAAGTATATACTT

CGATTTTCGTGTGCTAGAACTTTGGCTCGTAAACATAAAAGTATGGTACGCGCTTTTTTGCAAAGATTAG

GTTCGGGATTATTAGAAGAATTCTTTACGGAAGAAGAACAAGTTGTTTCTTTGATCTTCCCAAAAACAAC

TTCTTTTTCTTTACATGAATCACATATAGAACGTATTTGGTATTTGGATATTATCCGTATCAATGACCTG

GTGAATTATTCATAATGGGTTTGGTGACGTGATGAGACTTATGAATAGAATAGAAATGATCTATAAATGA

TCAAGAGAGAAAAAAATTCATGAATTTTCATTCTGAAATGCTCATTGCAGTAGTGTAGTGGTTGAATCAA

CTGAGTAGTCAAAATTATTATACTTTCTTCTCGGGACCCAAGTTTTATATTATACATAGGTAAAGTCGTG

TGCAATGAAAAATGCAAGCACGGTTTGGGGAGGGATCTTTTTCCTCTATTCCAACAAAGAAAAGTTATCT

ACTCCATCC

>Aiphanes_minima_HQ265531.1

GGGTCCTGTCTCTGGTTCAGTAGAAATGGAAATGGAAGAATTACAAGGATATTTAGAAAAAGATGGATCT

CGGCAACAACACTTTCTATATCCGCTTCTCTTTAAGGAGTATATTTACACATTTGCTCATGATCGTGGTT

TAAATGGTTCGATTTTTTACGAATCCACGGAAANTTTTGGTTATGACAATAAATCTAGTTCAGTACTTGT

GAAACGTTTAATTATTCGAATGTNTCAACAGAATTATTTGATTTATTCGGTTAATGATTCTAACCAAAAT

CGATTCGTTGGGCACAACAATTATTTTTATTTTCATTTTTATTCTCAGATGATATTGGAAGGTTTTGCAG

TCATTGTGGAAATTCCATTCTTGCTGCGATTAGTATCTTCCCTCGAAGAAAAAAAAATACCAAAATCTCA

GAATTTGAATTTACGATCTATTCATTCAATATTTCCCTTTTTGGAGGACAAATTATCGCATTTAAATTAT

GTGNCAGATATACTAATACCTTATCCCATCCATCTGAAAATCTTGGTTCAAATCCTTCAATGCTGGATCC

AAGATGTTCCTTCTTTACATTTATTGCGATTCTTTCTTCACGAATATCATAATTGGAATAGTCTTATTAC

TCCGAATAATTCTATTTTTTTTTCAAAAGAAAATAAAAGACTATTTCGGTTCCCATATAATTCTTATGTA

TCTGAATGCGAATTTGTATTAGTTTTTCTTCGTAAACAATCTTCTTATTTACGATTAACATCTTCTGGAG

CTTTTCTTGAGCGAACACATTTCTATGGAAAAATAGAACATCTTATACTTATAGTAGTGCGCCGTAATTA

TTTTCAGAAGACCCTATGGTTCTTCAAGGATCCCTTCATGCATTATGTTCGATATCAAGGAAAAGCAATT

CTGGTTTCAAAGGGGACTCATCTTCTGATGAAGAAATGGAAATGTCATCTTGTCAATTTCTGGCAATATT

ATTTTCACTTTTGGTCTCAACCGTACAGGATCCATATAAACCAATTATCAAGCTGTTCTTTCTATTTTCT

AGGTTATCTTTCAAGTGTACTAATAAATCCTTCGGCGGTAAGGAATCAAATGCTAGAGAATTCATTTCTA

ATGGATACTGTTATTAAAAAATTCGATACCAGAGTCCCTGTTATTCCTCTTATTGGATCATTGTCTAAAG

CTAAATTTTGTACCGTATCGGGGCATCCTATTAGTAAGCCCATCTGGACCAATTTATCAGATTGCGATAT

TCTTGATCGATTTGGTCGGATATGTAGAAATCTTTCTCATTATCACAGTGGATCCTCAAAAAAACAGAGT

TTGTATCGAATAAAGTATATACTTCGATTTTCGTGTGCTAGAACTTTGGCTCGTAAACATAAAAGTATGG

TACGCGCTTTTTTGCAAAGATTAGGTTCGGGATTATTAGAAGAATTCTTTACGGAAGAAGAACAAGTTGT

TTCTTTGATCTTCCCAAAAACAACTTCTTTTTCTTTACATGAATCACATATAGAACGTATTTGGTATTTG

GATATTATCCGTATCAATGACCTGGTGAATTATTCATAATGGGTTTGGTGATGTGATGAGACTTATGAAT

AGAATAGAAATGATCTATAAATGATCAAGAGAGAAAAAAATTCATGAATTTTCATT

>Aiphanes_hirsuta_subsp._fosteriorum_HQ265529.1

CCATATTGCACTATGTATCATTTGATAACCCCAAAAATGAAATGGGTCCTGTCTCTGGTTCAAGTAGAAA

TGGAAATGGAAGAATTACAAGGATATTTAGAAAAAGATAGATCTCGGCAACAACACTTTCTATATCCGCT

TCTCTTTAAGGAGTATATTTACATATTTGCTCATGATCGTGGTTTAAATGGTTCGATTTTTTACGAATCC

ACGGAAATTTTGGGTTATGACAATAAATCTAGTTCAGTACTTGTGAAACGTTTAATTATTCGAATGTATC

AACAGAATTATTTGATTTATTCGGTTAATGATTCTAACCAAAATCGATTCGTTGGGCACAACAATTATTT

TTATTTTCATTTTTATTCTCAGATGATATTGGAAGGTTTTGCAGTCATTGTGGAAATTCCATTCTTGCTG

CGATTAGTATCTTCCCWCGAAGAAAAAAAAATACCAAAATCTCAGAATTTGAATTTACGATCTATTCATT

CAATATTTCCCTTTTTGGAGGACAAATTATCGCATTTAAATTATGTGTCAGATATACTAATACCTTATCC

CATCCATCTGAAAATCTTGGTTCAAATCCTTCAATGCTGGATCCAAGATGTTCCTTCTTTACATTTATTC

CGATTCTTTCTTCACGAATATCATAATTGGAATAGTCTTATTACTCCGAATAATTCTATTTTATTTTCAA

AAGAAAATAAAAGACTATTTCGGTTCCCATATAATTCTTATGTATCTGAATGCGAATTTGTATTAGTTTT

TCTTCGTAAACAATCTTCTTATTTACGATTAACATCTTTTGGAGCTTTTCTTGAGCGAACACATTTCTAT

GGAAAAATAGAACATCTTATACTTATAGTAGTGCGCCGTAATTATTTTCAGAAGACCCTATGGTTCTTCA

AGGATCCCTTCATGCATTATGTTCGATATCAAGGAAAAGCAATTCTGGTTTCAAAGGGGACTCATCTTCT

GATGAAGAAATGGAAATGTCATCTTGTCAATTTCTGGCAATATTATTTTCACTTTTGGTCTCAACCGTAC

AGGATCCATATAAACCAATTATCAAGCTGTTCTTTCTATTTTCTAGGTTATCTTTCAAGTGTACTAATAA

ATCCTTCGGCGGTAAGGAATCAAATGCTAGAGAATTCATTTCTAATAGATACTGTTATTAAAAAATTCGA

TACCAGAGTCCCTGTTATTCCTCTTATTGGATCATTGTCTAAAGCTAAATTTTGTACCGTATCGGGGCAT

CCTATTAGTAAGCCCATCTGGACCAATTTATCAGATTGCGATATTATTGATCGATTTGGTCGGATATGTA

GAAATCTTTCTCATTATCACAGTGGATCCTCAAAAAAACAGAGTTTGTATCGAATAAAGTATATACTTCG

ATTTTCGTGTGCTAGAACTTTGGCTCGTAAACATAAAAGTATGGTACGCGCTTTTTTGCAAAGATTAGGT

TCGGGATTATTAGAAGAATTCTTTACGGAAGAAGAAGAAGTTGTTTCTTTGATCTTCCCAAAAACAACTT

CTTTTTCTTTACATGAATCACATATAGAACGTATTTGGTATTTGGATATTATCCGTATCAATGACCTGGT

GAATTATTCATAATGGGTTTGGTGACGTGATGAGACTTATGAATAGAATAGAAATGATCTATAAATGATC

AAGAGAGAAAAAATTCATGAATTTTCATTCTGAAATGCTCATTGCAGTAGTGTAGTGGTTGAATCAACTG

AGTAGTCAAAATTATTATACTTTCTTCTCGGGACCCAAGTTTTATATTATACATAGGTAAAGTCGTGTGC

AATGAAAAATGCAAGCACGGTTTGGGGAGGGATCTTTTTCCTCTATTCCAACAAAGAAAAGTTATCTACT

CC

>Aiphanes_erinacea_HQ265527.1

CATATTGCACTATGTATCATTTGATAACCCCAAAAATGAAATGGGTCCTGTCTCTGGTTCAAGTAGAAAT

GGAAATGGAAGAATTACAAGGATATTTAGAAAAAGATAGATCTCGGCAACAACACTTTCTATATCCGCTT

CTCTTTAAGGAGTATATTTACACATTTGCTCATGATCGTGGTTTAAATGGTTCGATTTTTTACGAATCCA

CGGAAATTTTTGGTTATGACAATAAATCTAGTTCAGTACTTGTGAAACGTTTAATTATTCGAATGTATCA

ACAGAATTATTTGATTTATTCGGTTAATGATTCTAACCAAAATCGATTCGTTGGGCACAACAATTATTTT

TATTTTCATTTTTATTCTCAGATGATATTGGAAGGTTTTGCAGTCATTGTGGAAATTCCATTCTTGCTGC

GATTAGTATCTTCCCWCGAAGAAAAAAAAATACCAAAATCTCAGAATTTGAATTTACGATCTATTCATTC

AATATTTCCCTTTTTGGAGGACAAATTATCGCATTTAAATTATGTGTCAGATATACTAATACCTTATCCC

ATCCATCTGAAAATCTTGGTTCAAATCCTTCAATGCTGGATCCAAGATGTTCCTTCTTTACATTTATTGC

GATTCTTTCTTCACGAATATCATAATTGGAATAGTCTTATTACTCCGAATAATTCTATTTTTTTTTCAAA

AGAAAATCAAAGACTATTTCGGTTCCCATATAATTCTTATGTATCTGAATGCGAATTTGTATTAGTTTTT

CTTCGTAAACAATCTTCTTATTTACGATTAACATCTTCTGGAGCTTTTCTTGAGCGAACACATTTCTATG

GAAAAATAGAACATCTTATACTTATAGTAGTGCGCCGTAATTATTTTCAGAAGACCCTATGGTTCTTCAA

GGATCCCTTCATGCATTATGTTCGATATCAAGGAAAAGCAATTCTGGTTTCAAAGGGGACTCATCTTCTG

AAGAAGAAATGGAAATGTCATCTTGTCAATTTCTGGCAATATTATTTTCACTTTTGGTCCCAACCGTACA

GGATCCATATAAACCAATTATCAAGCTGTTCTTTCTATTTTCTAGGTTATCTTTCAAGTGTACTAATAAA

CCCTTCGGCGGTAAGGAATCAAATGCTAGAGAATTCATTTCTAATAGATACTGTTATTAAAAAATTCGAT

ACCAGAGTCCCTGTTATTCCTCTTATTGGATCATTGTCTAAAGCTAAATTTTGTACCGTATCGGGGCATC

CTATTAGTAAGCCCATCTGGACCAATTTATCAGATTGTGATATTATTGATCGATTTGGTCGGATATGTAG

AAACCTTTCTCATTATCACAGTGGATCCTCAAAAAAACAGAGTTTGTATCGAATAAAGTATATACTTCGA

TTTTCGTGTGCTAGAACTTTGGCTCGTAAACATAAAAGTATGGTACGCGCTTTTTTGCAAAGATTAGGTT

CGGGATTATTAGAAGAATTCTTTACGGAAGAAGAACAAGTTGTTTCTTTGATCTTCCAAAAAACAACTTC

TTTTTCTTTACATGAATCACATATAGAACGTATTTGGTATTTGGATATTATCCGTATCAATGACCTGGTG

AATTATTCATAATGGGTTTGGTGACGTGATGAGACTTATGAATAGAATAGAAATGATCTATAAATGATCA

AGAGAGAAAAAAATTCATGAATTTGCATTCTGAAATGCTCATTGCAGTAGTGTAGTGGTTGAATCAACTG

AGTAGTCAAAATTATTATACTTTCTTCTCGGGACCCAAGTTTTATATTATACATAGGTAAAGTCGTGTGC

AATGAAAAATGCAAGCACGGTTTGGGGAGGGATCTTTTTCCTCTATTCCAACAAAGAAAAGTTATCTACT

CC

>Aiphanes_spicata_HQ265532.1

AATGGGTCCTGTCTCTGGTTCAGTAGAAATGGAAATGGAAGAATTACAAGGATATTTAGAAAAAGATAGA

TCTCGGCAACAACACTTTCTATATCCGCTTCTCTTTAAGGAGTATATTTACACATTTGCTCATGATCGTG

GTTTAAATGGTTCGATTTTTTACGAATCCACGGAAATTTTTGGTTATGACAATAAATCTAGTTCAGTACT

TGTGAAACGTTTAATTATTCGAATGTATCAACAGAATTATTTGATTTATTCGGTTAATGATTCTAACCAA

AATCGATTCGTTGGGCACAACAATTATTTTTATTTTCATTTTTATTCTCAGATGATATTGGAAGGTTTTG

CAGTCATTGTGGAAATTCCATTCTTGCTGCGATTAGTATCTTCCCTCGAAGAAAAAAAAATACCAAAATC

TCAGAATTTGAATTTACGATCTATTCATTCAATATTTCCCTTTTTGGAGGACAAATTATCGCATTTAAAT

TATGTGNCAGATATACTAATACCTTATCCCATCCATCTGAAAATCTTGGTTCAAATCCTTCAATGCTGGA

TCCAAGATGTTCCTTCTTTACATTTATTGCGATTCTTTCTTCACGAATATCATAATTGGAATAGTCTTAT

TACTCCGAATAATTCTATTTTTTTTTCAAAAGAAAATAAAAGACTATTTCGGTTCCCATATAATTCTTAT

GTATCTGAATGCGAATTTGTATTAGTTTTTCTTCGTAAACAATCTTCTTATTTACGATTAACATCTTCTG

GAGCTTTTCTTGAGCGAACACATTTCTATGGAAAAATAGAACATCTTATACTTATAGTAGTGCGCCGTAA

TTATTTTCAGAAGACCCTATGGTTCTTCAAGGATCCCTTCATGCATTATGTTCGATATCAAGGAAAAGCA

ATTCTGGTTTCAAAGGGGACTCATCTTCTGATGAAGAAATGGAAATGTCATCTTGTCAATTTCTGGCAAT

ATTATTTTCACTTTTGGTCTCAACCGTACAGGATCCATATAAACCAATTATCAAGCTGTTCTTTCTATTT

TCTAGGTTATCTTTCAAGTGTACTAATAAATCCTTCGGCAGTAAGGAATCAAATGCTAGAGAATTCATTT

CTAATGGATACTGTTATTAAAAAATTCGATACCAGAGTCCCTGTTATTCCTCTTATTGGATCATTGTCTA

AAGCGAAATTTTGTACCGTATCGGGGCATCCTATTAGTAAGCCCATCTGGACCAATTTATCAGATTGCGA

TATTATTGATCGATTTGGTCGGATATGTAGAAATCTTTCTCATTATCACAGTGGATCCTCAAAAAAACAG

AGTTTGTATCGAATAAAGTATATACTTCGATTTTCGTGTGCTAGAACTTTGGCTCGTAAACATAAAAGTA

TGGTACGCGCTTTTTTGCAAAGATTAGGTTCGGGATTATTAGAAGAATTCTTTACGGAAGAAGAACAAGT

TGTTTCTTTGATCTTCCCAAAAACAACTTCTTTTTCTTTACATGAATCACATATAGAACGTATTTGGTAT

TTGGATATTATCCGTATCAATGACCTGGTGAATTATTCATAATGGGTTTGGTGACGTGATGAGACTTATG

AATAGAATAGAAATGATCTATAAATGATCAAGAGAGAAAAAAATTCATGAATTTTCATTCTGAAATGCTC

ATTGCAGTAGTGTAGTAGTTGAATCAACTGAGTAGTCAAAATTATTATACTTTCTTCTCGGGACCCAAGT

CTTATATTATACATAGGTAAAGTCGTGTGCAATGAAAAATGCAAGCACGGTTTGGGGAGGGATCTTTTTC

CTCTATTCCAACAAAGAAAAGTTATCTACTCCNTCCGACTAGTTAA

>Aiphanes_killipii_HQ265530.1

TGGGTCCTGTCTCTGGTTCAGTAGAAATGGAAATGGAAGAATTACAAGGATATTTAGAAAAAGATAGATC

TCGGCAACAACACTTTCTATATCCGCTTCTCTTTAAGGAGTATATTTACACATTTGCTCATGATCGTGGT

TTAAATGGTTCGATTTTTTACGAATCCACGGAAATTTTTGGTTATGACAATAAATCTAGTTCAGTACTTG

TGAAACGTTCAATTATTCGAATGTATCAACAGAATTATTTGATTTATTCGGTTAATGATTCTAACCAAAA

TCGATTCGTTGGGCACAACAATTATTTTTATTTTCATTTTTATTCTCAGATGATATTGGAAGGTTTTGCA

GTCATTGTGGAAATTCCATTCTTGCTGCGATTAGTATCTTCCCTCGAAGAAAAAAAAATACCAAAATCTC

AGAATTTGAATTTACGATCTATTCATTCAATATTTCCCTTTTTGGAGGACAAATTATCGCATTTAAATTA

TGTGTCAGATATACTAATACCTTATCCCATCCATCTGAAAATCTTGGTTCAAATCCTTCAATGCTGGATC

CAAGATGTTCCTTCTTTACATTTATTGCGATTCTTTCTTCACGAATATCATAATTGGAATAGTCTTATTA

CTCCGAATAATTCTATTTTTTTTTCAAAAGAAAATAAAAGACTATTTCGGTTCCCATATAATTCTTATGT

ATCTGAATGCGAATTTGTATTAGTTTTTCTTCGTAAACAATCTTCTTATTTACGATTAACATCTTCTGGA

GCTTTTCTTGAGCGAACACATTTCTATGGAAAAATAGAACATCTTATAGTAGTGCGCCGTAATTATTTTC

AGAAGACCCTATGGTTCTTCAAGGATCCCTTCATGCATTATGTTCGATATCAAGGAAAAGCAATTCTGAT

TTCAAAGGGGACTCATCTTCTGATGAAGAAATGGAAATGTCATCTTGTCAATTTCTGGCAATATTATTTT

CACTTTTGGTCTCAACCGTACAGGATCCATATAAACCAATTATCAAGCTGTTCTTTCTATTTTCTAGGTT

ATCTTTCAAGTGTACTAATAAATCCTTCGGCGGTAAGGAATCAAATGCTAGAGAATTCATTTCTAATAGA

TACTGTTATTAAAAAATTCGATACCAGAGTCCCTGTTATTCCTCTTATTGGATCATTGTCTAAAGCTAAA

TTTTGTACCGTATCGGGGCATCCTATTAGTAAGCCCATCTGGACCAATTTATCAGATTGCGATATTATTG

ATCGATTTGGTCGGATATGTAGAAATCTTTCTCATTATCACAGTGGATCCTCAAAAAAACAGAGTTTGTA

TCGAATAAAGTATATACTTCGATTTTCGTGTGCTAGAACTTTGGCTCGTAAACATAAAAGTATGGTACGC

GCTTTTTTGCAAAGATTAGGTTCGGGATTATTAGAAGAATTCTTTACGGAAGAAGAACAAGTTGTTTCTT

TGATCTTCCCAAAAACAACTTCTTTTTCTTTACATGAATCACATATAGAACGTATTTGGTATTTGGATAT

TATCCGTATCAATGACCTGGTGAATTATTCATAATGGGTTTGGTGACGTGATGAGACTTATGAATAGAAT

AGAAATGATCTATAAATGATCAAGAGAGAAAAAAATTCATGAATTTTCATTCTGAAATGCTCATTGCAGT

AGTGTAGTGGTTGAATCAACTGAGTAGTCAAAATTATTATACTTTCTTCTCGGGACCCAAGTTTTATATT

ATACATAGGTAAAGTTCGTGTGCAATGAAAAATGCAAGCACGGTTTGGGGAGGGATCTTTTTCCTCNATT

CCAACAAAGAAAAGTTATCTACTCCNNCCCACTAGTTAA

>Aiphanes_grandis_HQ265528.1

CCATATTGCACTATGTATCATTTGATAACCCCAAAAATGAAATGGGTCCTGTCTCTGGTTCAAGTAGAAA

TGGAAATGAAAGAATTACAAGGATATTTAGAAAAAGATAGATCTCGGCAACAACACTTTCTATATCCGCT

TCTCTTTAAGGAGTATATTTACACATTTGCTCATGATCGTGGTTTAAATGGTTCGATTTTTTACGAATCC

ACGGAAATTTTTGGTTATGACAATAAATCTAGTTCAGTACTTGTGAAACGTTCAATTATTCGAATGTATC

AACAGAATTATTTGATTTATTCGGTTAATGATTCTAACCAAAATCGATTCGTTGGGCACAACAATTATTT

TTATTTTCATTTTTATTCTCAGATGATATTGGAAGGTTTTGCAGTCATTGTGGAAATTCCATTCTTGCTG

CGATTAGTATCTTCCCWCGAAGAAAAAAAAATACCAAAATCTCAGAATTTGAATTTACGATCTATTCATT

CAATATTTCCCTTTTTGGAGGACAAATTATCGCATTTAAATTATGTGTCAGATATACTAATACCTTATCC

CATCCATCTGAAAATCTTGGTTCAAATCCTTCAATGCTGGATCCAAGATGTTCCTTCTTTACATTTATTG

CGATTCTTTCTTCACGAATATCATAATTGGAATAGTCTTATTACTCCGAATAATTCTATTTTTTTTTCAA

AAGAAAATAAAAGACTATTTCGGTTCCCATATAATTCTTATGTATCTGAATGCGAATTTGTATTAGTTTT

TCTTCGTAAACAATCTTCTTATTTACGATTAACATCTTCTGGAGCTTTTCTTGAGCGAACACATTTCTAT

GGAAAAATAGAACATCTTATACTTATAGTAGTGCGCCGTAATTATTTTCAGAAGACCCTATGGTTCTTCA

AGGATCCCTTCATGCATTATGTTCGATATCAAGGAAAAGCAATTCTGGTTTCAAAGGGGACTCATCTTCT

GATGAAGAAATGGAAATGTCATCTTGTCAATTTCTGGCAATATTATTTTCACTTTTGGTCTCAACCGTAC

AGGATCCATATAAACCAATTATCAAGCTGTTCTTTCTATTTTCTAGGTTATCTTTCAAGTGTACTAATAA

ATCCTTCGGCGGTAAGGAATCAAATGCTAGAGAATTCATTTCTAATAGATACTGTTATTAAAAAATTCGA

TACCAGAGTCCCTGTTATTCCTCTTATTGGATCATTGTCTAAAGCTAAATTTTGTACCGTATCGGGGCAT

CCTATTAGTAAGCCCATCTGGACCAATTTATCAGATTGCGATATTATTGATCGATTTGGTCGGATATGTA

GAAATCTTTCTCATTATCACAGTGGATCCTCAAAAAAACAGAGTTTGTATCGAATAAAGTATATACTTCG

ATTTTCGTGTGCTAGAACTTTGGCTCGTAAACATAAAAGTATGGTACGCGCTTTTTTGCAAAGATTAGGT

TCGGGATTATTAGAAGAATTCTTTACGGAAGAAGAACAAGTTGTTTCTTTGATCTTCCCAAAAACAACTT

CTTTTTCTTTACATGAATCACATATAGAACGTATTTGGTATTTGGATATTATCCGTATCAATGACCTGGT

GAATTATTCATAATGGGTTTGGTGACGTGATGAGACTTATGAATAGAATAGAAATGATCTATAAATGATC

AAGAGAGAAAAAAATTCATGAATTTTCATTCTGAAATGCTCATTGCAGTAGTGTACGTGGTTGAATCAAC

TGAGTAGTCAAAATTATTATACTTTCTTCTCGGGACCACAAGTCTTATATTATACATAGGTAAAGTCGTG

TGCAATGAAAAATGCAAGCACGGTTTGGGGAGGGATCTTTTTCCTCTATTCCAACAAAGAAAAGTTATCT

ACTC

>Aiphanes_horrida_HQ265526.1

CTCTGGTTCAGTAGAAATGGAAATGGAAGAATTACAAGGATATTTAGAAAAAGATGGATCTCGGCAACAA

CACTTTCTATATCCGCTTCTCTTTAAGGAGTATATTTACACATTTGCTCATGATCGTGGTTTAAATGGTT

CGATTTTTTACGAATCCACGGAAATTTTTGGTTATGACAATAAATCTAGTTCAGTACTTGTGAAACGTTT

AATTATTCGAATGTATCAACAGAATTATTTGATTTATTCGGTTAATGATTCTAACCAAAATCGATTCGTT

GGGCACAACAATTATTTTTATTTTCATTTTTATTCTCAGATGATATTGGAAGGTTTTGCAGTCATTGTGG

AAATTCCATTCTTGCTGCGATTAGTATCTTCCCTCGAAGAAAAAAAAATACCAAAATCTCAGAATTTGAA

TTTACGATCTATTCATTCAATATTTCCCTTTTTGGAGGACAAATTATCGCATTTAAATTATGTGTCAGAT

ATACTAATACCTTATCCCATCCATCTGAAAATCTTGGTTCAAATCCTTCAATGCTGGATCCAAGATGTTC

CTTCTTTACATTTATTGCGATTCTTTCTTCACGAATATCATAATTGGAATAGTCTTATTACTCCGAATAA

TTCTATTTTTTTTTCAAAAGAAAATAAAAGACTATTTCGGTTCCCATATAATTCTTATGTATCTGAATGC

GAATTTGTATTAGTTTTTCTTCGTAAACAATCTTCTTATTTACGATTAACATCTTCTGGAGCTTTTCTTG

AGCGAACACATTTCTATGGAAAAATAGAACATCTTATACTTATAGTAGTGCGCCGTAATTATTTTCAGAA

GACCCTATGGTTCTTCAAGGATCCCTTCATGCATTATGTTCGATATCAAGGAAAAGCAATTCTGGTTTCA

AAGGGGACTCATCTTCTGATGAAGAAATGGAAATGTCATCTTGTCAATTTCTGGCAATATTATTTTCACT

TTTGGTCTCAACCGTACAGGATCCATATAAACCAATTATCAAGCTGTTCTTTCTATTTTCTAGGTTATCT

TTCAAGTGTACTAATAAATCCTTCGGCGGTAAGGAATCAAATGCTAGAGAATTCATTTCTAATGGATACT

GTTATTAAAAAATTCGATACCAGAGTCCCTGTTATTCCTCTTATTGGATCATTGTCTAAAGCTAAATTTT

GTACCGTATCGGGGCATCCTATTAGTAAGCCCATCTGGACCAATTTATCAGATTGCGATATTCTTGATCG

ATTTGGTCGGATATGTAGAAATCTTTCTCATTATCACAGTGGATCCTCAAAAAAACAGAGTTTGTATCGA

ATAAAGTATATACTTCGATTTTCGTGTGCTAGAACTTTGGCTCGTAAACATAAAAGTATGGTACGCGCTT

TTTTGCAAAGATTAGGTTCGGGATTATTAGAAGAATTCTTTACGGAAGAAGAACAAGTTGTTTCTTTGAT

CTTCCCAAAAACAACTTCTTTTTCTTTACATGAATCACATATAGAACGTATTTGGTATTTGGATATTATC

CGTATCAATGACCTGGTGAATTATTCATAATGGGTTTGGTGACGTGATGAGACTTATGAATAGAATAGAA

ATGATCTATAAATGATCAAGAGAGAAAAAAATTCATGAATTTTCATTATGAAATGCTCACTTGCAGTAGT

GTAGTGGTTGAATCAACTGAGTAGTCAAAATTATTATACTTTCTTCTCGGGACCCAAGTTTTATATTATA

CATAGGTAAAGTCGTGTGCAATGAAAAATGCAAGCACGGTTTGGGGAGGGATCTTTTTCCTCTATTCCAA

CAAAGAAAAGTTATCTACTCC

>Aiphanes_aculeata_AM114641.1

GACCATATTGCACTATGTATCATTTGATAACCCCAAAAATGAAATGGGTCCTGTCTCTGGTTCAAGTAGA

AATGGAAATGGAAGAATTACAAGGATATTTAGAAAAAGATGGATCTCGGCAACAACACTTTCTATATCCG

CTTCTCTTTAAGGAGTATATTTACACATTTGCTCATGATCGTGGTTTAAATGGTTCGATTTTTTACGAAT

CCACGGAAATTTTTGGTTATGACAATAAATCTAGTTCAGTACTTGTGAAACGTTTAATTATTCGAATGTA

TCAACAGAATTATTTGATTTATTCGGTTAATGATTCTAACCAAAATCGATTCGTTGGGCACAACAATTAT

TTTTATTTTCATTTTTATTCTCAGATGATATTGGAAGGTTTTGCAGTCATTGTGGAAATTCCATTCTTGC

TGCGATTAGTATCTTCCCTCGAAGAAAAAAAAATACCAAAATCTCAGAATTTGAATTTACGATCTATTCA

TTCAATATTTCCCTTTTTGGAGGACAAATTATCGCATTTAAATTATGTGTCAGATATACTAATACCTTAT

CCCATCCATCTGAAAATCTTGGTTCAAATCCTTCAATGCTGGATCCAAGATGTTCCTTCTTTACATTTAT

TGCGATTCTTTCTTCACGAATATCATAATTGGAATAGTCTTATTACTCCGAATAATTCTATTTTTTTTTC

AAAAGAAAATAAAAGACTATTTCGGTTCCCATATAATTCTTATGTATCTGAATGCGAATTTGTATTAGTT

TTTCTTCGTAAACAATCTTCTTATTTACGATTAACATCTTCTGGAGCTTTTCTTGAGCGAACACATTTCT

ATGGAAAAATAGAACATCTTATACTTATAGTAGTGCGCCGTAATTATTTTCAGAAGACCCTATGGTTCTT

CAAGGATCCCTTCATGCATTATGTTCGATATCAAGGAAAAGCAATTCTGGTTTCAAAGGGGACTCATCTT

CTGATGAAGAAATGGAAATGTCATCTTGTCAATTTCTGGCAATATTATTTTCACTTTTGGTCTCAACCGT

ACAGGATCCATATAAACCAATTATCAAGCTGTTCTTTCTATTTTCTAGGTTATCTTTCAAGTGTACTAAT

AAATCCTTCGGCGGTAAGGAATCAAATGCTAGAGAATTCATTTCTAATGGATACTGTTATTAAAAAATTC

GATACCAGAGTCCCTGTTATTCCTCTTATTGGATCATTGTCTAAAGCTAAATTTTGTACCGTATCGGGGC

ATCCTATTAGTAAGCCCATCTGGACCAATTTATCAGATTGCGATATTCTTGATCGATTTGGTCGGATATG

TAGAAATCTTTCTCATTATCACAGTGGATCCTCAAAAAAACAGAGTTTGTATCGAATAAAGTATATACTT

CGATTTTCGTGTGCTAGAACTTTGGCTCGTAAACATAAAAGTATGGTACGCGCTTTTTTGCAAAGATTAG

GTTCGGGATTATTAGAAGAATTCTTTACGGAAGAAGAACAAGTTGTTTCTTTGATCTTCCCAAAAACAAC

TTCTTTTTCTTTACATGAATCACATATAGAACGTATTTGGTATTTGGATATTATCCGTATCAATGACCTG

GTGAATTATTCATAATGGGTTTGGTGACGTGATGAGACTTATGAATAGAATAGAAATGATCTATAAATGA

TCAAGAGAGAAAAAAATTCATGAATTTTCATTATGAAATGCTCATTGCAGTAGTGTAGTGGTTGAATCAA

CTGAGTAGTCAAAATTATTATACTTTCTTCTCGGGACCCAAGTTTTATATTATACATAGGTAAAGTC

>Allagoptera_arenaria_AM114635.1

GACCATATTGCACTATGTATCATTTGATAACCCAAAAAATGAAATGGGTCCTGTCTCTGGTTCAAGTAGA

AATGTAAATGGAAGAATTACAAGGATATTTAGAAAAAGATAGATCTCGGCAACAACACTTTCTATATCCG

CTTCTCTTTAAGGAGTATATTTACACATTTGCTCATGATCGTGGTTTAAATGGTTCGATTTTTTACGAAT

CCACGGAAATTTTTGGTTATGACAATAAATCTAGTTCAGTACTTGTGAAACGTTCAATTATTCGAATGTA

TCAACAGAATTATTTGATTTATTCGGTTAATGATTCTAACCAAAATCGATTCGTTGGGCACAACAATTAT

TTTGATTTTCATTTTTATTCTCAGATGATATTGGAAGGTTTTGCAGTCATTGTGGAAATTCCATTCTTGC

TGCGATTAGTATCTTCCCCCGAAGAAAAAAAAATACCAAAATCTCAGAATTTGAATTTACGATCTATTCA

TTCAATATTTCCCTTTTTGGAGGACAAATTATCGCATTTAAATTATGTGTCAGATATACTAATACCTTAT

CCCATCCATCTGAAAATCTTGGTTCAAATCCTTCAATGCTGGATCCAAGATGTTCCTTCTTTACATTTAT

TGCGATTCTTTCTTCACGAATATCATAATTGGAATAGTCTTATTACTCCGAATAATTCTATTTTTTTTTC

AAAAGAAAATAAAAGACTATTTCGGTTCCCATATAATTCTTATGTATCTGAATGCGAATTTGTATTAGTT

TTTCTTCGTAAACAATCTTCTTATTTACGATTAACATCTTCTGGAGCTTTTCTTGAGCGAACACATTTCT

ATGGAAAAATAGAACATCTTATAGTAGTGCGCCGTAATTATTTTCAGAAGACCCTATGGTTCTTCAAGGA

TCCCTTCATGCATTATGTTCGATATCAAGGAAAAGCAATTCTGGTTTCAAAGGGGACTCATCTTCTGATG

AAGAAATGGAAATGTCATCTTGTCAATTTCTGGCAATATTATTTTCACTTTTGGTCTCAACCGTACAGGA

TTCATATAAACCAATTATCAAGCTGTTCTTTCTATTTTCTAGGTTATCTTTCAAGTGTACTAATAAATCC

TTCGGCGGTAAGGAATCAAATGCTAGAGAATTCATTTCTAATAGATACTGTTATTAAAAAATTCGATACC

AGAGTCCCAGTTATTCCTCTTATTGGATCATTGTCTAAAGCTAAATTTTGTACCGTATCGGGGCATCCTA

TTAGTAAGCCGATCTGGACCAATTTATCAGATTGCGATATTATTGATCGATTTGGTCGGATATGTAGAAA

TCTTTCTCATTATCACAGTGGATCCTCAAAAAAACAGAGTTTGTATCGAATAAAGTATATACTTCGATTT

TCGTGTGCTAGAACTTTGGCTCGTAAACATAAAAGTATGGTACGCGCTTTTTTGCAAAGATTAGGTTCGG

GATTATTAGAAGAATTCTTTACGGAAGAAGAAGAAGTTGTTTCTTTGATCTTCCAAAAAACAACCTCTTT

TTCTTTACATGAATCACATATAGAACGTATTTGGTATTTGGATATTATCCGTATCAATGACCTGGTGAAT

TATTCATAATGGGTTTGGTGACGTGATGAGACTTATGAATAGAATAGAAATGATCTATAAATGATCAAGA

GAGAAAAAAATTCATGAATTTTCATTCTGAAATGCTCATTGCAGTAGTGTAGTGGTTGAATCAACTGAGT

AGTCAAAATTATTATACTTTCTTCTCGGGACCCAAGTTTTCTATTATACATAGGTAAAGTC

>Ammandra_decasperma_AM114611.1

GACCATATTGCACTATGTATCATTTGATAACCCAAAAAATGAAATGGGTCCTGCCTCTGGTTCAAGTAGA

AATGTAAATGGAAGAATTACAAGGATATTTAGAAAAAGATAGATCTCGGCAACAACACTTTCTATATCCG

CTTCTCTTTAAGGAATATATTTACACATTTGCTCATGATCGTGGTTTAAATGGTTCGATTTTTTACGAAT

CCACGGAAATTTTTGGTTATGACAATAAATCTAGTTCAGTACTTGTGAAACGTTCAATTATTCGAATGTA

TCAACAGAATTATTTGATTTATTCGGTTAATGATTCTAACCAAAATCGATTCGTTGGGCACAACAATTAT

TTTTATTTTCATTTTTATTCTCAGATGATATTGGAAGGTTTTGCAGTCATTGTGGAAATTCCATTCTTGC

TGCGATTAGTATCTTCCCTCGAAGAAAAAAAAATACCAAAATCTCAGAATTTGAATTTACGATCTATTCA

TTCAATATTTCCCTTTTTGGAGGACAAATTATCGCATTTAAATTATGTGTCAGATATACTAATACCTTAT

CCCATCCATCTGAAAATCTTGGTTCAAATCCTTCAATGCTGGATCCAAGATGTTCCTTCTTTACATTTAT

TGCGATTCTTTCTTCACGAATATCATAATTGGAATAGTCTTATTACTCCGAATAATTCTATTTTTCTTTT

TCAAAATAAAAGACTATTTCGGTTCCCATATAATTCTTATGTATCTGAATGTGAATTTGTATTAGTTTTT

CTTCGTAAACAATCTTCTTATTTACGATTAACATCTTCTGGAGCTTTTCTTGAGCGAACACATTTCTATG

GAAAAATAGAACATCTTATAGTAGTGCGCCGTAATTATTTTCAGAAGACCCTATGGTTCTTCAAGGATCC

CTTCATGCATTATGTTCGATATCAAGGAAAAGCAATTCTGGTTTCAAAGGGGACTCATCTTCTGATGAAG

AAATGGAAATGTCACCTTGTCAATTTCTGGCAATATTATTTTCACTTTTGGTCTCAACCGTACAGGATCC

ATATAAACCAATTATCAAACTGTTCTTTCTATTTTCTAGGTTATCTTTCAAGTGTACTAATAAATCCTTC

GGCGGTAAGGAATCAAATGCTAGAGAATTCATTTCTAATAGATACTGTTATTAAAAAATTCGATACCAGA

GTCCCAGTTATTCCTCTTATTGGATCATTATCTAAAGCTAAATTTTGTACCGTATCGGGGCATCCTATTA

GTAAGCCGATCTGGACCAATTTATCAGATTGCGATATTATTGATCGATTTGGTCGGATATGTAGAAATCT

TTCTCATTATCACAGTGGATCCTCAAAAAAACAGAGTTTGTATCGAATAAAGTATATACTTCGATTTTCG

TGTGCTAGAACTTTGGCTCGTAAACATAAAAGTATGGTACGCGCTTTTTTGCAAAGATTAGGTTCGGGAT

TATTAGAAGAATTCTTTACGGAAGAAGAACAAGTTGTTTCTTTGATCTTCCCAAAAACAACTTCTTTTTC

TTTACATGAATCACATATAGAACGTATTTGGTATTTGGATATTATCCGTATCAATGACCTGGTTAATTAT

TCATAATGGGTTTGGTGACGTGATGAGACTTATGAATAGAATAGAAATGATCTATAAATGATCAAGAGAG

AAAAAAATTCATGAATTTTCATTCTGAAATACTCATTGCAGTAGTGTAGTGGTTGAATCAACTGAGTAGC

CAAAATTATTATACTTTCTTCTCGGGACCCAAGTTTTATATTATACATAGGTAAAGTC

>Ammandra_decasperma_EF128232.1

AACCCAAAAAATGAAATGGGTCCTGCCTCTGGTTCAAGTAGAAATGTAAATGGAAGAATTACAAGGATAT

TTAGAAAAAGATAGATCTCGGCAACAACACTTTCTATATCCGCTTCTCTTTAAGGAATATATTTACACAT

TTGCTCATGATCGTGGTTTAAATGGTTCGATTTTTTACGAATCCACGGAAATTTTTGGTTATGACAATAA

ATCTAGTTCAGTACTTGTGAAACGTTCAATTATTCGAATGTATCAACAGAATTATTTGATTTATTCGGTT

AATGATTCTAACCAAAATCGATTCGTTGGGCACAACAATTATTTTTATTTTCATTTTTATTCTCAGATGA

TATTGGAAGGTTTTGCAGTCATTGTGGAAATTCCATTCTTGCTGCGATTAGTATCTTCCCTCGAAGAAAA

AAAAATACCAAAATCTCAGAATTTGAATTTACGATCTATTCATTCAATATTTCCCTTTTTGGAGGACAAA

TTATCGCATTTAAATTATGTGTCAGATATACTAATACCTTATCCCATCCATCTGAAAATCTTGGTTCAAA

TCCTTCAATGCTGGATCCAAGATGTTCCTTCTTTACATTTATTGCGATTCTTTCTTCACGAATATCATAA

TTGGAATAGTCTTATTACTCCGAATAATTCTATTTTTTTTTTTCAAAATAAAAGACTATTTCGGTTCCCA

TATAATTCTTATGTATCTGAATGTGAATTTGTATTAGTTTTTCTTCGTAAACAATCTTCTTATTTACGAT

TAACATCTTCTGGAGCTTTTCTTGAGCGAACACATTTCTATGGAAAAATAGAACATCTTATAGTAGTGCG

CCGTAATTATTTTCAGAAGACCCTATGGTTCTTCAAGGATCCCTTAAATTCGATACCAGAGTCCCAGTTA

TTCCTCTTATTGGATCATTATCTAAAGCTAAATTTTGTACCGTATCGGGGCATCCTATTAGTAAGCCGAT

CTGGACCAATTTATCAGATTGCGATATTATTGATCGATTTGGTCGGATATGTAGAAATCTTTCTCATTAT

CACAGTGGATCCTCAAAAAAACAGAGTTTGTATCGAATAAAGTATATACTTCGATTTTCGTGTGCTAGAA

CTTTGGCTCGTAAACATAAAAGTATGGTACGCGCTTTTTTGCAAAGATTAGGTTCGGGATTATTAGAAGA

ATTCTTTACGGAAGAAGAACAAGTTGTTTCTTTGATCTTCCCAAAAACAACTTCTTTTTCTTTACATGAA

TCACATATAGAACGTATTTGGTATTTGGATATTATCCGTATCAATGACCTGGTTAATTATTCATAATGGG

TTTGGTGACGTGATGAGACTTATGAATAGAATAGAAATGATCTATAAATGATCAAGAGAGAAAAAAATTA

ATGAATTTTCATTCTGAAATGCTCATTGCAGTAGTGTAGTGGTTGAATCAACTGAGTAGCCAAAATTATT

ATACTTTC

>Asterogyne_martiana_AM114654.1

GGGTCCTGCCTCTGGTTCAAGTAGAAATGTAAATGGAAGAATTACAAGGATATTTAGAAAAAGATAGATC

TCGGCAACAACACTTTCTATATCCGCTTCTCTTTAAGGAGTATATTTACACATTTGCTCATGATCGGGGT

TTAAATGGTTCGATTTTTTACGAATCCACGGAAATTTTTGGTTATGACAATAAATCTAGTTCAGTACTTG

TGAAACGTTCAATTATTCGAATGTATCAACAGAATTATTTGATTTATTCGGTTAACGATTCTAACCAAAA

TCGATTCGTTGGGCACAACAATTATTTTTATTTTCATTTTTATTCTCAGATGATATTGGAAGGTTTTGCA

GTCATTGTGGAAATTCCATTCTTGCTGCGATTAGTATCTTCCCTCGAAGAAAAAAAAATACCAAAATCTC

AGAATTTGAATTTACGATCTATTCATTCAATATTTCCCTTTTTGGAGGATAAATTATCGCATTTAAATTA

TGTGTCAGATATACTAATACCTTATCCCATCCATCTGAAAATCTTGGTTCAAATCCTTCAATGCTGGATC

CAAGATGTTCCTTCTTTACATTTATTGCGATTCTTTCTTCACGAATATCATAATTGGAATAGTCTTATTA

CTCCGAATAATTCTATTTTTTTTTTTTCAAAAGAAAATAAAAGACTATTTCGGTTCCCATATAATTCTTA

TGTATCTGAATGCGAATTTGTATTAGTTTTTCTTCGTAAACAATCTTCTTATTTACGATTAACATCTTCT

GGAGCTTTTCTTGAGCGAACACATTTCTATGGAAAAATAGAACATCTTATAGTAGTGCGCCGTAATTATT

TTCAGAAGACCCTATGGTTCTTCAAGGATCCCTTCATGCATTATGTTCGATATCAAGGAAAAGCAATTCT

GGTTTCAAGGGGGACTCATCTTCTGATGAAGAAATGGAAATGTCACCTTGTCAATTTCTGGCAATATTAT

TTTCACTTTTGGTCTCAACCGTACAGGATCCATATAAACCAATTATCAAGCTGTTCTTTCTATTTTCTAG

GTTATCTTTCAAGTGTACTAATAAATCCTTCGGCGGTAAGGAATCAAATGCTAGAGAATTCATTTCTAAT

AGATACTGTTATTAAAAAATTCGATACCAGAGTCCCAGTTATTCCTCTTATTGGATCATTGTCTAAAGCT

AAATTTTGTACCGTATCGGGGCATCCTATTAGTAAGCCGATCTGGACCAATTTATCAGATTGCGATATTA

TTGATCGATTTGGTCGGATATGTAGAAATCTTTCTCATTATCACAGTGGATCCTCAAAAAAACAGAGTTT

GTATCGAATAAAGTATATACTTCGATTTTCGTGTGCTAGAACTTTGGCTCGTAAACATAAAAGTATGGTA

CGCGCTTTTTTGCAAAGATTAGGTTCGGGATTATTAGAAGAATTCTTTACGGAAGAAGAACAAGTTGTTT

CTTTGATCTTCCCAAAAACTACTTCTTTTTCTTTACATGAATCACATATAGAACGTATTTGGTATTTGGA

TATTATCCGTATCAATGACCTGGTGAATTATTCATAATGGGTTTGGTGACGTGATGAGACTTATGAATAG

AATAGAAATGATCTATAAATGATCAAGAGAGAAAAAAATTCATTAATTTTCATTCTGAAATGCTCATTGC

AGTAGTGTAGTGGTTGAATCAACTGAGTAGTCAAAATTATTATACTTTCTTCTCGGGACCCAAGTTTTAT

ATTATACATAGGTAAAGTC

>Astrocaryum_sciophilum_JQ626555.1

TACCTTATCCCATCCATCTGAAAATCTTGGTTCAAATCCTTCAATGCTGGATCCAAGATGTTCCTTCTTT

ACATTTATTGCGATTCTTTCTTCACGAATATCATAATTGGAATAGTCTTATTACTCCGAATAATTCTATT

TTTTTTTCAAAAGAAAATAAAAGACTATTTCGGTTCCCATATAATTCTTATGTATCTGAATGCGAATTTG

TATTAGTTTTTCTTCGTAAACAATCTTCTTATTTACGATTAACATCTTCTGGAGCTTTTCTTGAGCGAAC

ACATTTCTATGGAAAAATAGAACATCTTATAGTAGTGCGCCGTAATTATTTTCAGAAGACCCTATGGTTC

TTCAAGGATCCCTTCATGCATTATGTTCGATATCAAGGAAAAGCAATTCTGGTTTCAAAGGGGACTCATC

TTCTGATGAAGAAATGGAAATGTCATCTTGTCAATTTCTGGCAATATTATTTTCACTTTTGGTCTCAACC

GTACAGGATCCATATAAACCAATTATCAAGCTGTTCTTTCTATTTTCTAGGTTATCTTTCAAGTGTACTA

ATAAATCCTTCGGCGGTAAGGAATCAAATGCTAGAGAATTCATTTCTAATAGATACTGTTATTAAAAAAT

TCGATACCAGAGTCCCTGTTATTCCTCTTATTGGATCATTGTCTAAAGCTAAATTTTGTACCGTATCGGG

GCATCCTATTAGTAAGCCGATCTGGACCAATTTATCAGATTGCGATATTATTGATCGATTTGGTCGGATA

TGTAGAA

>Astrocaryum_alatum_JQ586688.1

CCGAATAATTCTATTTTTTTTTCAAAAGAAAATAAAAGACTATTTCGGTTCCCATATAATTCTTATGTAT

CTGAATGCGAATTTGTATTAGTTTTTCTTCGTAAACAATCTTCTTATTTACGATTAACATCTTCTGGAGC

TTTTCTTGAGCGAACACATTTCTATGGAAAAATAGAACATCTTATAGTAGTGCGCCGTAATTATTTTCAG

AAGACCCTATGGTTCTTCAAGGATCCCTTCATGCATTATGTTCGATATCAAGGAAAAGCAATTCTGGTTT

CAAAGGGGACTCATCTTCTGATGAAGAAATGGAAATGTCATCTTGTCAATTTCTGGCAATATTATTTTCA

CTTTTGGTCTCAACCGTACAGGATCCATATAAACCAATTATCAAGCTGTTCTTTCTATTTTCTAGGTTAT

CTTTCAAGTGTACTAATAAATCCTTCGGCGGTAAGGAATCAAATGCTAGAGAATTCATTTCTAATAGATA

CTGTTATTAAAAAATTCGATACCAGAGTCCCTGTTATTCCTCTTATTGGATCATTGTCTAAAGCTAAATT

TTGTACCGTATCGGGGCATCCTATTAGTAAGCCGATCTGGACCAATTTATCAGATTGCGATATTATTGAT

CGATTTGGTCGGATATGTAGAAATCTTTCTCATTATCACAG

>Astrocaryum_alatum_JQ586689.1

TTATTGCGATTCTTTCTTCACGAATATCATAATTGGAATAGTCTTATTACTCCGAATAATTCTATTTTTT

TTTCAAAAGAAAATAAAAGACTATTTCGGTTCCCATATAATTCTTATGTATCTGAATGCGAATTTGTATT

AGTTTTTCTTCGTAAACAATCTTCTTATTTACGATTAACATCTTCTGGAGCTTTTCTTGAGCGAACACAT

TTCTATGGAAAAATAGAACATCTTATAGTAGTGCGCCGTAATTATTTTCAGAAGACCCTATGGTTCTTCA

AGGATCCCTTCATGCATTATGTTCGATATCAAGGAAAAGCAATTCTGGTTTCAAAGGGGACTCATCTTCT

GATGAAGAAATGGAAATGTCATCTTGTCAATTTCTGGCAATATTATTTTCACTTTTGGTCTCAACCGTAC

AGGATCCATATAAACCAATTATCAAGCTGTTCTTTCTATTTTCTAGGTTATCTTTCAAGTGTACTAATAA

ATCCTTCGGCGGTAAGGAATCAAATGCTAGAGAATTCATTTCTAATAGATACTGTTATTAAAAAATTCGA

TACCAGAGTCCCTGTTATTCCTCTTATTGGATCATTGTCTAAAGCTAAATTTTGTACCGTATCGGGGCAT

CCTATTAGTAAGCCGATCTGGACCAATTTATCAGATTGCGATATTATTGATCGATTTGGTCGGATATGTA

GAAATCTTTCTCATTATCACAGTGGATCCTCAAAAAAACAGAGTTTGTATCGAATAA

>Astrocaryum_alatum_JQ586687.1

GAATAATTCTATTTTTTTTTCAAAAGAAAATAAAAGACTATTTCGGTTCCCATATAATTCTTATGTATCT

GAATGCGAATTTGTATTAGTTTTTCTTCGTAAACAATCTTCTTATTTACGATTAACATCTTCTGGAGCTT

TTCTTGAGCGAACACATTTCTATGGAAAAATAGAACATCTTATAGTAGTGCGCCGTAATTATTTTCAGAA

GACCCTATGGTTCTTCAAGGATCCCTTCATGCATTATGTTCGATATCAAGGAAAAGCAATTCTGGTTTCA

AAGGGGACTCATCTTCTGATGAAGAAATGGAAATGTCATCTTGTCAATTTCTGGCAATATTATTTTCACT

TTTGGTCTCAACCGTACAGGATCCATATAAACCAATTATCAAGCTGTTCTTTCTATTTTCTAGGTTATCT

TTCAAGTGTACTAATAAATCCTTCGGCGGTAAGGAATCAAATGCTAGAGAATTCATTTCTAATAGATACT

GTTATTAAAAAATTCGATACCAGAGTCCCTGTTATTCCTCTTATTGGATCATTGTCTAAAGCTAAATTTT

GTACCGTATCGGGGCATCCTATTAGTAAGCCGATCTGGACCAATTTATCAGATTGCGATATTATTGATCG

ATTTGGTCGGATATGTAGAAATCTTTCTCATTATCACAGTGGATCCTCAAAAAAACAGAGTTTGTATCGA

ATAA

>Astrocaryum_scopatum_JF758213.1

GATCAAAGAAACAACTTGTTCTTCTTCCGTAAAGAATTCTTCTAATAATCCCGAACCTAATCTTTGCAAA

AAAGCGCGTACCATACTTTTATGTTTACGAGCCAAAGTTCTAGCACACGAAAATCGAAGTATATACTTTA

TTCGATACAAACTCTGTTTTTTTGAGGATCCACTGTGATAATGAGAAAGATTTCTACATATCCGACCAAA

TCGATCAATAATATCGCAATCTGATAAATTGGTCCAGATCGGCTTACTAATAGGATGCCCCGATACGGTA

CAAAATTTAGCTTTAGACAATGATCCAATAAGAGGAATAACAGGGACTCTGGTATCGAATTTTTTAATAA

CAGTATCTATTAGAAATGAATTCTCTAGCATTTGATTCCTTACCGCCGAAGGATTTATTAGTACACTTGA

AAGATAACCTAGAAAATAGAAAGAACAGCTTGATAATTGGTTTATATGGATCCTGTACGGTTGAGACCAA

AAGTGAAAATAATATTGCCAGAAATTGACAAGATGACATTTCCATTTCTTCATCAGAAGATGAGTCCCCT

TTGAAACCAGAATTGCTTTTCCTTGATATCGAACATAATGCATGAAGGGATCCTTGAAGAACCATAGGGT

CTTCTGAAAATAATTACGGCGCACTACTATAAGATGTTCTATTTTTCCATAGAAATGTGTTCGCTCAAGA

AAAGCTCCAGAAGATGTTAATCGTAAATAAGAAGATTGTTTACGAAGAAAAACTAATACAAATTCGCATT

CAGATACATAAGAATTATATGGGAACCGAAATAGTTCTTTATTTTCTTTTGAAAAAAAAAAAATAGAATT

ATTCGGAGTAATAAGACTATTCCAATTATGATATTCGTGAAGAAAGAATCGCAATAAATGTAAAGAAGGA

ACATCTTGGATCCAGCATTGAAGGATTTGAACCAAGATTTTCAGATGGATGGGATAAGGTATTAGTATAT

CTGACACATAATTTAAATGCGATAATTTGTCCTCCAAAAAGGGAAATATTGAATGAATAGATCGTAAATT

CAAATTCTGAGATTTTGGTATTTTTTTTTCTTCGAGGGAAGATACTAATCGCAGCAAGAATGGAATTTCC

ACAATGACTGCAAAACCTTCCAATATCATCTGAGAATAAAAATGAAAATAAAAATAATTGTTGTGCCCAA

CGAATCGATTTTGGTTAGAATCATTAACCGAATAAATCAAATAATTCTGTTGATACATTCGAATAATTGA

ACGTTTCACAAGTACTGAACTAGATTTATTGTCATAACCAAAAATTTCCGTGGATTCGTAAAAAATCGAA

CCATTTAAACCACGATCATGAGCAAATGTGTAAATATACTCCTTAAAGAGAAGCGGATATAGAAAGTGTT

GTTGCCGAGATCTATCTTTTTCTAAATATCCTTGTAATTCTTCCAT

>Astrocaryum_vulgare_HQ265547.1

GTCCTGCCTCTGGTTCAGTAGAAATGGAAATGGAAGAATTACAAGGATATTTAGAAAAAGATAGATCTCG

GCAACAACACTTTCTATATCCGCTTCTCTTTAAGGAGTATATTTACACATTTGCTCATGATCGTGGTTTA

AATGGTTCGATTTTTTACGAATCCACGGAAATTTTTGGTTATGACAATAAATCTAGTTCAGTACTTGTGA

AACGTTCAATTATTCGAATGTATCAACAGAATTATTTGATTTATTCGGTTAATGATTCTAACCAAAATCG

ATTCGTTGGGCACAACAATTATTTTTATTTTCATTTTTATTCTCAGATGATATTGGAAGGTTTTGCAGTC

ATTGTGGAAATTCCATTCTTGCTGCGATTAGTATCTTCCCTCGAAGAAAAAAAAATACCAAAATCTCAGA

ATTTGAATTTACGATCTATTCATTCAATATTTCCCTTTTTGGAGGACAAATTATCGCATTTAAATTATGT

GTCAGATATACTAATACCTTATCCCATCCATCTGAAAATCTTGGTTCAAATCCTTCAATGCTGGATCCAA

GATGTTCCTTCTTTACATTTATTGCGATTCTTTCTTCACGAATATCATAATTGGAATAGTCTTATTACTC

CGAATAATTCTATTTTTTTTTCNAAAGAAAATAAAAGACTATTTCGGTTCCCATATAATTCTTATGTATC

TGAATGCGAATTTGTATTAGTTTTTCTTCGTAANCAATCTTCTTATTTACGATNAACNTCTTCTGGAGCT

TTTCTTGAGCGAACACNTTTCTAKGSAAAAANAGANCATCTTATAGTAGTGCGCCGTAATTNTTTTCAGA

AGACCCTATGGTTCTTCANGGATCCCTTCATGCATTATGTTCGATATCAAGGAAAAGCAATTCTGGTTTC

AAAGGGGACTCATCTTCTGATGAAGAAATGGAAATGTCATCTTGTCAATTTCTGGCAATATTATTTTCAC

TTTTGGTCTCAACCGTNCNGGATCCATATAAACCAATTATCAAGCTGTTCTTTCTATTTTCTNGGTTATC

TTNCAAGTGTACTAATAAANCCTTCGGCGGTAAGGAATCAAATGCTAGAGAATNCATTTCTAATAGATAC

TGTTATTAAAAAATTCGATACCAGAGTCCCTGTTATTCCTCTTATTGGATCATTGTCTAAAGCTAAATTT

TGTACCGTATCGGGGCATCCTATTAGTAAGCCGATCTGGACCAATTTATCAGATTGCGATATTATTGATC

GATTTGGTCGGATATGTAGAAATCTTTCTCATTATCACAGTGGATCCTCAAAAAAACAGAGTTTGTATCG

AATAAAGTATATACTTCGATTTTCGTGTGCTAGAACTTTGGCTCGTAAACATAAAAGTATGGTACGCGCT

TTTTTGCAAAGATTAGGTTCGGGATTATTAGAAGAATTCTTTACGGAAGAAGAACAAGTTGTTTCTTTGA

TCTYCCMAAAAACAACTTCTTTTTCNTTMCATGAATCACATATAGAACGTATTTGGTATTTGGATATTAT

CCGTATCAATGACCTGGTGAATTATTCATAATGGGTTTGGTGACGTGATGAGACTTATGAATAGAATAGA

AATGATCTATAAATGATCAAGAGAGAAAAAAATTCATGAATTTTCATTCTGAAATGCTCATTGCAGTAGT

GTAGTGGTTGAATCAACTGAGTAGTCAAAATTATTATACTTTCTTCTCGGGACCCAAGTTTTATATTATA

CATAGGTAAAGTCGTGTGCAATGAAAAATGCAAGCACCGGTTTGGGGAGGGATC

>Astrocaryum_standleyanum_HQ265545.1

GTCCTGCCTCTGGTTCAGTAGAAATGGAAATGGAAGAATTACAAGGATATTTAGAAAAAGATAGATCTCG

GCAACAACACTTTCTATATCCGCTTCTCTTTAAGGAGTATATTTACACATTTGCTCATGATCGTGGTTTA

AATGGTTCGATTTTTTACGAATCCACGGAAATTTTTGGTTATGACAATAAATCTAGTTCAGTACTTGTGA

AACGTTCAATTATTCGAATGTATCAACAGAATTATTTGATTTATTCGGTTAATGATTCTAACCAAAATCG

ATTCGTTGGGCACAACAATTATTTTTATTTTCATTTTTATTCTCAGATGATATTGGAAGGTTTTGCAGTC

ATTGTGGAAATTCCATTCTTGCTGCGATTAGTATCTTCCCTCGAAGAAAAAAAAATACCAAAATCTCAGA

ATTTGAATTTACGATCTATTCATTCAATATTTCCCTTTTTGGAGGACAAATTATCGCATTTAAATTATGT

GTCAGATATACTAATACCTTATCCCATCCATCTGAAAATCTTGGTTCAAATCCTTCAATGCTGGATCCAA

GATGTTCCTTCTTTACATTTATTGCGATTCTTTCTTCACGAATATCATAATTGGAATAGTCTTATTACTC

CGAATAATTCTATTTTTTTTTCAAAAGAAAATAAAAGACTATTTCGGTTCCCATATAATTCTTATGTATC

TGAATGCGAATTTGTATTAGTTTTTCTTCGTAAACAATCTTCTTATTTACGATTAACATCTTCTGGAGCT

TTTCTTGAGCGAACACATTTCTATGGAAAAATAGAACATCTTATAGTAGTGCGCCGTAATTATTTTCAGA

AGACCCTATGGTTCTTCAAGGATCCCTTCATGCATTATGTTCGATATCAAGGAAAAGCAATTCTGGTTTC

AAAGGGGACTCATCTTCTGATGAAGAAATGGAAATGTCATCTTGTCAATTTCTGGCAATATTATTTTCAC

TTTTGGTCTCAACCGTACAGGATCCATATAAACCAATTATCAAGCTGTTCTTTCTATTTTCTAGGTTATC

TTTCAAGTGTACTAATAAATCCTTCGGCGGTAAGGAATCAAATGCTAGAGAATTCATTTCTAATAGATAC

TGTTATTAAAAAATTCGATACCAGAGTCCCTGTTATTCCTCTTATTGGATCATTGTCTAAAGCTAAATTT

TGTACCGTATCGGGGCATCCTATTAGTAAGCCGATCTGGACCAATTTATCAGATTGCGATATTATTGATC

GATTTGGTCGGATATGTAGAAATCTTTCTCATTATCACAGTGGATCCTCAAAAAAACAGAGTTTGTATCG

AATAAAGTATATACTTCGATTTTCGTGTGCTAGAACTTTGGCTCGTAAACATAAAAGTATGGTACGCGCT

TTTTTGCAAAGATTAGGTTCGGGATTATTAGAAGAATTCTTTACGGAAGAAGAACAAGTTGTTTCTTTGA

TCTTCCCAAAAACAACTTCTTTTTCTTTACATGAATCACATATAGAACGTATTTGGTATTTGGATATTAT

CCGTATCAATGACCTGGTGAATTATTCATAATGGGTTTGGTGACGTGATGAGACTTATGAATAGAATAGA

AATGATCTATAAATGATCAAGAGAGAAAAAAATTCATGAATTTTCATTCTGAAATGCTCATTGCAGTAGT

GTAGTGGTTGAATCAACTGAGTAGTCAAAATTATTATACTTTCTTCTCGGGACCCAAGTTTTATATTATA

CATAGGTAAAGTCGTGTGCAATGAAAAATGCAAGCACGGTTTGGGGAGGGATCTTTTTCCTCTATTCCAA

CAAAGAAAAGTTATCTACTCCNTCCGACTAGTTAA

>Astrocaryum_rodriguesii_HQ265543.1

GGTCCTGCCTCTGGTTCAGTAGAAATGGAAATGGAAGAATTACAAGGATATTTAGAAAAAGATAGATCTC

GGCAACAACACTTTCTATATCCGCTTCTCTTTAAGGAGTATATTTACACATTTGCTCATGATCGTGGTTT

AAATGGTTCGATTTTTTACGAATCCACGGAAATTTTTGGTTATGACAATAAATCTAGTTCAGTACTTGTG

AAACGTTCAATTATTCGAATGTATCAACAGAATTATTTGATTTATTCGGTTAATGATTCTAACCAAAATC

GATTCGTTGGGCACAACAATTATTTTTATTTTCATTTTTATTCTCAGATGATATTGGAAGGTTTTGCAGT

CATTGTGGAAATTCCATTCTTGCTGCGATTAGTATCTTCCCTCGAAGAAAAAAAAATACCAAAATCTCAG

AATTTGAATTTACGATCTATTCATTCAATATTTCCCTTTTTGGAGGACAAATTATCGCATTTAAATTATG

TGTCAGATATACTAATACCTTATCCCATCCATCTGAAAATCTTGGTTCAAATCCTTCAATGCTGGATCCA

AGATGTTCCTTCTTTACATTTATTGCGATTCTTTCTTCACGAATATCATAATTGGAATAGTCTTATTACT

CCGAATAATTCTATTTTTTTTTCAAAAGAAAATAAAAGACTATTTCGGTTCCCATATAATTCTTATGTAT

CTGAATGCGAATTTGTATTAGTTTTTCTTCGTAAACAATCTTCTTATTTACGATTAACATCTTCTGGAGC

TTTTCTTGAGCGAACACATTTCTATGGAAAAATAGAACATCTTATAGTAGTGCGCCGTAATTATTTTCAG

AAGACCCTATGGTTCTTCAAGGATCCCTTCATGCATTATGTTCGATATCAAGGAAAAGCAATTCTGGTTT

CAAAGGGGACTCATCTTCTGATGAAGAAATGGAAATGTCATCTTGTCAATTTCTGGCAATATTATTTTCA

CTTTTGGTCTCAACCGTACAGGATCCATATAAACCAATTATCAAGCTGTTCTTTCTATTTTCTAGGTTAT

CTTTCAAGTGTACTAATAAATCCTTCGGCGGTAAGGAATCAAATGCTAGAGAATTCATTTCTAATAGATA

CTGTTATTAAAAAATTCGATACCAGAGTCCCTGTTATTCCTCTTATTGGATCATTGTCTAAAGCTAAATT

TTGTACCGTATCGGGGCATCCTATTAGTAAGCCGATCTGGACCAATTTATCAGATTGCGATATTATTGAT

CGATTTGGTCGGATATGTAGAAATCTTTCTCATTATCACAGTGGATCCTCAAAAAAACAGAGTTTGTATC

GAATAAANTATATACTTCGATNTTCGTGTGCCAGAACNNCGNCTCGTAAACATAAANGTATGGTACGCGC

TTTTTTGCAAAGATTAGGTTCGGGATTATTAGAAGAATTCTTTACGGAAGAAGAACAAGTTGTTTCTTTG

ATCTTCCCAAAAACAACTTCTTTTTCTTTACATGAATCACATATAGAACGTATTTGGTATTTGGATATTA

TCCGTATCAATGACCTGGTGAATTATTCATAATGGGTTTGGTGACGTGATGAGACTTATGAATAGAATAG

AAATGATCTATAAATGATCAAGAGAGAAAAAAATTCATGAATTTTCATTCTGAAATGCTCATTGCAGTAG

TGTAGTGGTTGAATCAACTGAGTAGTCAAAATTATTATACTTTCTTCTCGGGACCCAAGTTTTATATTAT

ACATAGGTAAAGTCGTGTGCAATGAAAAATGCAAGCACGGTTTGGGGAGGGATCTTTTTCCTCTATTCCA

ACAAAGAAAAGTTATCTACTCCATCCGGACTAGTTAA

>Astrocaryum_murumuru_HQ265541.1

GTCCTGCCTCTGGTTCAAGTAGAAATGGAAATGGAAGAATTACAAGGATATTTAGAAAAAGATAGATCTC

GGCAACAACACTTTCTATATCCGCTTCTCTTTAAGGAGTATATTTACACATTTGCTCATGATCGTGGTTT

AAATGGTTCGATTTTTTACGAATCCACGGAAATTTTTGGTTATGACAATAAATCTAGTTCAGTACTTGTG

AAACGTTCAATTATTCGAATGTATCAACAGAATTATTTGATTTATTCGGTTAATGATTCTAACCAAAATC

GATTCGTTGGGCACAACAATTATTTTTATTTTCATTTTTATTCTCAGATGATATTGGAAGGTTTTGCAGT

CATTGTGGAAATTCCATTCTTGCTGCGATTAGTATCTTCCCTCGAAGAAAAAAAAATACCAAAATCTCAG

AATTTGAATTTACGATCTATTCATTCAATATTTCCCTTTTTGGAGGACAAATTATCGCATTTAAATTATG

TGTCAGATATACTAATACCTTATCCCATCCATCTGAAAATCTTGGTTCAAATCCTTCAATGCTGGATCCA

AGATGTTCCTTCTTTACATTTATTGCGATTCTTTCTTCACGAATATCATAATTGGAATAGTCTTATTACT

CCGAATAATTCGATTTTTTTTTCAAAAGAAAATAAAAGACTATTTCGGTTCCCATATAATTCTTATGTAT

CTGAATGCGAATTTGTATTCGTTTTTCTTCGTAAACAATCTTCTTATTTACGATTAACATCTTCTGGAGC

TTTTCTTGAGCGAACACATTTCTATGGAAAAATAGAACATCTTATAGTAGTGCGCCGTAATTATTTTCAG

AAGACCCTATGGTTCTTCAAGGATCCCTTCATGCATTATGTTCGATATCAAGGAAAAGCAATTCTGGTTT

CAAAGGGGACTCATCTTCTGATGAAGAAATGGAAATGTCATCTTGTCAATTTCTGGCAATATTATTTTCA

CTTTTGGTCTCAACCGTACAGGATCCATATAAACCAATTATCAAGCTGTTCTTTCTATTTTCTAGGTTAT

CTTTCAAGTGTACTAATAAATCCTTCGGCGGTAAGGAATCAAATGCTAGAGAATTCATTTCTAATAGATA

CTGTTATTAAAAAATTCGATACCAGAGTCCCTGTTATTCCTCTTATTGGATCATTGTCTAAAGCTAAATT

TTGTACCGTATCGGGGCATCCTATTAGTAAGCCGATCTGGACCAATTTATCAGATTGCGATATTATTGAT

CGATTTGGTCGGATATGTAGAAATCTTTCTCATTATCACAGTGGATCCTCAAAAAAACAGAGTTTGTATC

GAATAAAGTATATACTTCGATTTTCGTGTGCTAGAACTTTGGCTCGTAAACATAAAAGTATGGTACGCGC

TTTTTTGCAAAGATTAGGTTCGGGATTATTAGAAGAATTCTTTACGGAAGAAGAACAAGTTGTTTCTTTG

ATCTTCCCAAAAACAACTTCTTTTTCTTTACACGAATCACATATAGAACGTATTTGGTATTTGGATATTA

TCCGTATCAATGACCTGGTGAATTATTCATAATGGGTTTGGTGACGTGATGAGACTTATGAATAGAATAG

AAATGATCTATAAATGATCAAGAGAGAAAAAAATTCATGAATTTTCATTCTGAAATGCTCATTGCAGTAG

TGTAGTGGTTGAATCAACTGAGTAGTCAAAATTATTATACTTTCTTCTCGGGACCCAAGTTTTATATTAT

ACATAGGTAAAGTCGTGTGCAATGAAAAATGCAAGCACGGTTTGGGGAGGGATCTTTTTCCTCTATTCCA

ACAAAGAAAAGTTATCTACTCCACCNAACTAGTTAA

>Astrocaryum_mexicanum_HQ265539.1

TCCTGCCTCTGGTTCAGTAGAAATGGAAATGGAAGAATTACANGGATATTTAGAAAAAGATAGATCTCGG

CAACAACACTTTCTATATCCACTTCTCTTTAAGGAGTATATTTACNCATTTGCTCATGATCGTGGTTTAA

ATGGTTCGATTTTTTACGAATCCACGGAAATTTTTGGTTATGACAATAAATCTAGTTCAGTACTTGTGAA

ACGTTCAATTATTCGAATGTATCAACAGAATTATTTGATTTATTCGGTTAATGATTCTAACCAAAATCGA

TTCGTTGGGCACAACAATTATTTTTATTTTCATTTTTATTCTCAGATGATATTGGAAAGTTTTGCAGTCA

TTGTGGAAATTCCATTCTTGCTGCGATTAGTATCTTCCCTCGAAGAAAAAAAAATACCAAAATCTCAGAA

TTTGAATTTACGATCTATTCATTCAATATTTCCCTTTTTGGAGGACAAATTATCGCATTTAAATTATGTG

TCAGATATACTAATACCTTATCCCATCCATCTGAAAATCTTGGTTCAAATCCTTCAATGCTGGATCCAAG

ATGTTCCTTCTTTACATTTATTGCGATTCTTTCTTCACGAATATCATAATTGGAATAGTCTTATTACTCC

GAATAATTCTATTTTTTTTTCAAAAGAAAATAAAAGACTATTTCGGTTCCCATATAATTCTTATGTATCT

GAATGCGAATTTGTATTAGTTTTTCTTCGTAAACAATCTTCTTATTTACGATTAACATCTTCTGGAGCTT

TTCTTGAGCGAACACATTTCTATGGAAAAATAGAACATCTTATAGTAGTGCGCCGTAATTATTTTCAGAA

GACCCTATGGTTCTTCAAGGATCCCTTCATGCATTATGTTCGATATCAAGGAAAAGCAATTCTGGTTTCA

AAGGGGACTCATCTTCTGATGAAGAAATGGAAATGTCATCTTGTCAATTTCTGGCAATATTATTTTCACT

TTTGGTCTCAACCGTACAGGATCCATATAAACCAATTATCAAGCTGTTCTTTCTATTTTCTAGGTTATCT

TTCAAGTGTACTAATAAATCCTTCGGCGGTAAGGAATCAAATGCTAGAGAATTCATTTCTAATAGATACT

GTTATTAAAAAATTCGATACCAGAGTCCCTGTTATTCCTCTTATTGGATCATTGTCTAAAGCTAAATTTT

GTACCGTATCGGGGCATCCTATTAGTAAGCCGATCTGGACCAATTTATCAGATTGCGATATTATTGATCG

ATTTGGTCGGATATGTAGAAATCTTTCTCATTATCACAGTGGATCCTCAAAAAAACAGAGTTTGTATCGA

ATAAAGTATATACTTCGATTTTCGTGTGCTAGAACTTTGGCTCGTAAACATAAAAGTATGGTACGCGCTT

TTTTGCAAAGATTAGGTTCGGGATTATTAGAAGAATTCTTTACGGAAGAAGAACAAGTTGTTTCTTTGAT

CTTCCCAAAAACAACTTCTTTTTCTTTACATGAATCACATATAGAACGTATTTGGTATTTGGATATTATC

CGTATCAATGACCTGGTGAATTATTCATAATGGGTTTGGTGACGTGATGAGACTTATGAATAGAATAGAA

ATGATCTATAAATGATCAAGAGAGAAAAAAATTCATGAATTTTCATTCTGAAATGCTCATTGCAGTAGTG

TAGTGGTTGAATCAACTGAGTAGTCAAAATTATTATACTTTCTTCTCGGGACCCAAGTTTTATATTATAC

ATAGGTAAAGTCGTGTGCAATGAAAAATGCAAGCACGGTTTGGGGAGGGATCTTTTTCCTCTATTCCAAC

AAAGAAAAGTTATCTACTCCNTCCCACTAGTTAAA

>Astrocaryum_jauari_HQ265537.1

TCTGGGTCCAGTAGAATGGAATGGAGGAATTCCANGGATATTNAGAAAAAGANAGATCTCGGCANCANCN

CTTTCTATATCCNGNTCTCNTTAAGGAGNATATTTACNCATTNGCTCANGNTCGGGGTTNAAANNGGTCG

GATTTTTACGGATCCNCGGNAANTTTTGGGTATGACANTAAATCTAGTNCAGTACTTGTGAAACGTTCAA

TTATTCGAATGTATCAACAGAATTATTTGATTTATTCGGTTAATGATTCTAACCAAAATCGATTCGTTGG

GCACAACAATTATTTTTATTTTCATTTTTATTCTCAGATGATATTGGAAGGTTTTGCAGTCATTGTGGAA

ATTCCATTCTTGCTGCGATTAGTATCTTCCCTCGAAGAAAAAAAAATACCAAAATCTCAGAATTTGAATT

TACGATCTATTCATTCAATATTTCCCTTTTTGGAGGACAAATTATCGCATTTAAATTATGTGTCAGATAT

ACTAATACCTTATCCCATCCATCTGAAAATCTTGGTTCAAATCCTTCAATGCTGGATCCAAGATGTTCCT

TCTTTACATTTATTGCGATTCTTTCTTCACGAATATCATAATTGGAATAGTCTTATTACTCCGAATAATT

CTATTTTTTTTTCAAAAGAAAATAAAAGACTATTTCGGTTCCCATATAATTCTTATGTATCTGAATGCGA

ATTTGTATTAGTTTTTCTTCGTAAACAATCTTCTTATTTACGATTAACATCTTCTGGAGCTTTTCTTGAG

CGAACACATTTCTATGGAAAAATAGAACATCTTATAGTAGTGCGCCGTAATTATTTTCAGAAGACCCTAT

GGTTCTTCAAGGATCCCTTCATGCATTATGTTCGATATCAAGGAAAAGCAATTCTGGTTTCAAAGGGGAC

TCATCTTCTGATGAAGAAATGGAAATGTCATCTTGTCAATTTCTGGCAATATTATTTTCACTTTTGGTCT

CAACCGTACAGGATCCATATAAACCAATTATCAAGCTGTTCTTTCTATTTTCTAGGTTATCTTTCAAGTG

TACTAATAAATCCTTCGGCGGTAAGGAATCAAATGCTAGAGAATTCATTTCTAATAGATACTGTTATTAA

AAAATTCGATACCAGAGTCCCTGTTATTCCTCTTATNGGATCATTGTCTAAAGCTAAATTTTGTACCGTA

TCGGGGCATCCTATTAGTAAGCCGATCTGGACCAATTTATCAGATTGCGATATTATTGATCGATTTGGTC

GGATATGTAGAAATCTTTCTCATTATCACAGTGGATCCTCAAAAAAACAGAGTTTGTATCGAATAAAGTA

TATACTTCGATTTTCGTGTGCTAGAACTTTGGCTCGTAAACATAAAAGTATGGTACGCGCTTTTTTGCAA

AGATTAGGTTCGGGATTATTAGAAGAATTCTTTACGGAAGAAGAACAAGTTGTTTCTTTGATCTTCCCAA

AAACAACTTCTTTTTCTTTACATGAATCACATATAGAACGTATTTGGTATTTGGATATTATCCGTATCAA

TGACCTGGTGAATTATTCATAATGGGTTTGGTGACGTGATGAGACTTATGA

>Astrocaryum_chambira_HQ265535.1

GGGTCCTGCCTCTGGTTCAGTAGAAATGGAAATGGAAGAATTACAAGGATATTTAGAAAAAGATAGATCT

CGGCAACAACACTTTCTATATCCGCTTCTCTTTAAGGAGTATATTTACACATTTGCTCATGATCGTGGTT

TAAATGGTTCGATTTTTTACGAATCCACGGAAATTTTTGGTTATGACAATAAATCTAGTTCAGTACTTGT

GAAACGTTCAATTATTCGAATGTATCAACAGAATTATTTGATTTATTCGGTTAATGATTCTAACCAAAAT

CGATTCGTTGGGCACAACAATTATTTTTATTTTCATTTTTATTCTCAGATGATATTGGAAGGTTTTGCAG

TCATTGTGGAAATTCCATTCTTGCTGCGATTAGTATCTTCCCTCGAAGAAAAAAAAATACCAAAATCTCA

GAATTTGAATTTACGATCTATTCATTCAATATTTCCCTTTTTGGAGGACAAATTATCGCATTTAAATTAT

GTGTCAGATATACTAATACCTTATCCCATCCATCTGAAAATCTTGGTTCAAATCCTTCAATGCTGGATCC

AAGATGTTCCTTCTTTACATTTATTGCGATTCTTTCTTCACGAATATCATAATTGGAATAGTCTTATTAC

TCCGAATAATTCTATTTTTTTTTCAAAAGAAAATAAAAGACTATTTCGGTTCCCATATAATTCTTATGTA

TCTGAATGCGAATTTGTATTAGTTTTTCTTCGTAAACAATCTTCTTATTTACGATTAACATCTTCTGGAG

CTTTTCTTGAGCGAACACATTTCTATGGAAAAATAGAACATCTTATAGTAGTGCGCCGTAATTATTTTCA

GAAGACCCTATGGTTCTTCAAGGATCCCTTCATGCATTATGTTCGATATCAAGGAAAAGCAATTCTGGTT

TCAAAGGGGACTCATCTTCTGATGAAGAAATGGAAATGTCATCTTGTCAATTTCTGGCAATATTATTTTC

ACTTTTGGTCTCAACCGTACAGGATCCATATAAACCAATTATCAAGCTGTTCTTTCTATTTTCTAGGTTA

TCTTTCAAGTGTACTAATAAATCCTTCGGCGGTAAGGAATCAAATGCTAGAGAATTCATTTCTAATAGAT

ACTGTTATTAAAAAATTCGATACCAGAGTCCCTGTTATTCCTCTTATTGGATCATTGTCTAAAGCTAAAT

TTTGTACCGTATCGGGGCATCCTATTAGTAAGCCGATCTGGACCAATTTATCAGATTGCGATATTATTGA

TCGATTTGGTCGGATATGTAGAAATCTTTCTCATTATCACAGTGGATCCTCAAAAAAACAGAGTTTGTAT

CGAATAAAGTATATACTTCGATTTTCGTGTGCTAGAACTTTGGCTCGTAAACATAAAAGTATGGTACGCG

CTTTTTTGCAAAGATTAGGTTCGGGATTATTAGAAGAATTCTTTACGGAAGAAGAACAAGTTGTTTCTTT

GATCTTCCCAAAAACAACTTCTTTTTCTTTACATGAATCACATATAGAACGTATTTGGTATTTGGATATT

ATCCGTATCAATGACCTGGTGAATTATTCATAATGGGTTTGGTGACGTGATGAGACTTATGAATAGAATA

GAAATGATCTATAAATGATCAAGAGAGAAAAAAATTCATGAATTTTCATTCTGAAATGCTCATTGCAGTA

GTGTAGTGGTTGAATCAACTGAGTAGTCAAAATTATTATACTTTCTTCTCGGGACCCAAGTTTTATATTA

TACATAGGTAAAGTCGTGTGCAATGAAAAATGCAAGCACGGTTTGGGGAGGGATCTTTTTCCTCTATTCC

AACAAAGAAAAGTTATCTACTCCATCCGNACTAGTTAAA

>Astrocaryum_urostachys_HQ265546.1

GTCCTGCCTCTGGTTCAGTAGAAATGGAAATGGAAGAATTACAAGGATATTTAGAAAAAGATAGATCTCG

GCAACAACACTTTCTATATCCGCTTCTCTTTAAGGAGTATATTTACACATTTGCTCATGATCGTGGTTTA

AATGGTTCGATTTTTTACGAATCCACGGAAATTTTTGGTTATGACAATAAATCTAGTTCAGTACTTGTGA

AACGTTCAATTATTCGAATGTATCAACAGAATTATTTGATTTATTCGGTTAATGATTCTAACCAAAATCG

ATTCGTTGGGCACAACAATTATTTTTATTTTCATTTTTATTCTCAGATGATATTGGAAGGTTTTGCAGTC

ATTGTGGAAATTCCATTCTTGCTGCGATTAGTATCTTCCCTCGAAGAAAAAAAAATACCAAAATCTCAGA

ATTTGAATTTACGATCTATTCATTCAATATTTCCCTTTTTGGAGGACAAATTATCGCATTTAAATTATGT

GTCAGATATACTAATACCTTATCCCATCCATCTGAAAATCTTGGTTCAAATCCTTCAATGCTGGATCCAA

GATGTTCCTTCTTTACATTTATTGCGATTCTTTCTTCACGAATATCATAATTGGAATAGTCTTATTACTC

CGAATAATTCGATTTTTTTTTCAAAAGAAAATAAAAGACTATTTCGGTTCCCATATAATTCTTATGTATC

TGAATGCGAATTTGTATTCGTTTTTCTTCGTAAACAATCTTCTTATTTACGATTAACATCTTCTGGAGCT

TTTCTTGAGCGAACACATTTCTATGGAAAAATAGAACATCTTATAGTAGTGCGCCGTAATTATTTTCAGA

AGACCCTATGGTTCTTCAAGGATCCCTTCATGCATTATGTTCGATATCAAGGAAAAGCAATTCTGGTTTC

AAAGGGGACTCATCTTCTGATGAAGAAATGGAAATGTCATCTTGTCAATTTCTGGCAATATTATTTTCAC

TTTTGGTCTCAACCGTACAGGATCCATATAAACCAATTATCAAGCTGTTCTTTCTATTTTCTAGGTTATC

TTTCAAGTGTACTAATAAATCCTTCGGCGGTAAGGAATCAAATGCTAGAGAATTCATTTCTAATAGATAC

TGTTATTAAAAAATTCGATACCAGAGTCCCTGTTATTCCTCTTATTGGATCATTGTCTAAAGCTAAATTT

TGTACCGTATCGGGGCATCCTATTAGTAAGCCGATCTGGACCAATTTATCAGATTGCGATATTATTGATC

GATTTGGTCGGATATGTAGAAATCTTTCTCATTATCACAGTGGATCCTCAAAAAAACAGAGTTTGTATCG

AATAAAGTATATACTTCGATTTTCGTGTGCTAGAACTTTGGCTCGTAAACATAAAAGTATGGTACGCGCT

TTTTTGCAAAGATTAGGTTCGGGATTATTAGAAGAATTCTTTACGGAAGAAGAACAAGTTGTTTCTTTGA

TCTTCCCAAAAACAACTTCTTTTTCTTTACATGAATCACATATAGAACGTATTTGGTATTTGGATATTAT

CCGTATCAATGACCTGGTGAATTATTCATAATGGGTTTGGTGACGTGATGAGACTTATGAATAGAATAGA

AATGATCTATAAATGATCAAGAGAGAAAAAAATTCATGAATTTTCATTCTGAAATGCTCATTGCAATAGT

GTAGTGGTTGAATCAACTGAGTAGTCAAAATTATTATACTTTCTTCTCGGGACCCAAGTTTTATATTATA

CATAGGTAAAGTCGTGTGCAATGAAAAATGCAAGCACGGTTTGGGGAGGGATCTTTTTCCTCTATTCCAA

CAAAGAAAAGTTATCTACTCCATCCNAACTAGTTAA

>Astrocaryum_sciophilum_HQ265544.1

GGTCCTGCCTCTGGNTCAGCTAGAAATGGAAATGGAAGAATTACAAGGATATTTAGAAAAAGATAGATCT

CGGCAACAACACTTTCTATATCCGCTTCTCTTTAAGGAGTATATTTACACATTTGCTCATGATCGTGGTT

TAAATGGTTCGATTTTTTACGAATCCACGGAAATTTTTGGTTATGACAATAAATCTAGTTCAGTACTTGT

GAAACGTTCAATTATTCGAATGTATCAACAGAATTATTTGATTTATTCGGTTAATGATTCTAACCAAAAT

CGATTCGTTGGGCACAACAATTATTTTTATTTTCATTTTTATTCTCAGATGATATTGGAAGGTTTTGCAG

TCATTGTGGAAATTCCATTCTTGCTGCGATTAGTATCTTCCCTCGAAGAAAAAAAAATACCAAAATCTCA

GAATTTGAATTTACGATCTATTCATTCAATATTTCCCTTTTTGGAGGACAAATTATCGCATTTAAATTAT

GTGTCAGATATACTAATACCTTATCCCATCCATCTGAAAATCTTGGTTCAAATCCTTCAATGCTGGATCC

AAGATGTTCCTTCTTTACATTTATTGCGATTCTTTCTTCACGAATATCATAATTGGAATAGTCTTATTAC

TCCGAATAATTCTATTTTTTTTTCAAAAGAAAATAAAAGACTATTTCGGTTCCCATATAATTCTTATGTA

TCTGAATGCGAATTTGTATTAGTTTTTCTTCGTAAACAATCTTCTTATTTACGATTAACATCTTCTGGAG

CTTTTCTTGAGCGAACACATTTCTATGGAAAAATAGAACATCTTATAGTAGTGCGCCGTAATTATTTTCA

GAAGACCCTATGGTTCTTCAAGGATCCCTTCATGCATTATGTTCGATATCAAGGAAAAGCAATTCTGGTT

TCAAAGGGGACTCATCTTCTGATGAAGAAATGGAAATGTCATCTTGTCAATTTCTGGCAATATTATTTTC

ACTTTTGGTCTCAACCGTACAGGATCCATATAAACCAATTATCAAGCTGTTCTTTCTATTTTCTAGGTTA

TCTTTCAAGTGTACTAATAAATCCTTCGGCGGTAAGGAATCAAATGCTAGAGAATTCATTTCTAATAGAT

ACTGTTATTAAAAAATTCGATACCAGAGTCCCTGTTATTCCTCTTATTGGATCATTGTCTAAAGCTAAAT

TTTGTACCGTATCGGGGCATCCTATTAGTAAGCCGATCTGGACCAATTTATCAGATTGCGATATTATTGA

TCGATTTGGTCGGATATGTAGAAATCTTTCTCATTATCACAGTGGATCCTCAAAAAAACAGAGTTTGTAT

CGAATAAAGTATATACTTCGATTTTCGTGTGCTAGAACTTTGGCTCGTAAACATAAAAGTATGGTACGCG

CTTTTTTGCAAAGATTAGGTTCGGGATTATTAGAAGAATTCTTTACGGAAGAAGAACAAGTTGTTTCTTT

GATCTTCCCAAAAACAACTTCTTTTTCTTTACATGAATCACATATAGAACGTATTTGGTATTTGGATATT

ATCCGTATCAATGACCTGGTGAATTATTCATAATGGGTTTGGTGACGTGATGAGACTTATGAATAGAATA

GAAATGATCTATAAATGATCAAGAGAGAAAAAAATTCATGAATTTTCATTCTGAAATGCTCATTGCAGTA

GTGTAGTGGTTGAATCAACTGAGTAGTCAAAATTATTATACTTTCTTCTCGGGACCCAAGTTTTATATTA

TACATAGGTAAAGTCGTGTGCAATGAAAAATGCAAGCACGGTTTGGGGAGGGATCTTTTTCCTCTATTCC

AACAAAGAAAAGTTATCTACTCCATCCCACTAGTTAAA

>Astrocaryum_paramaca_HQ265542.1

TCCTGCCTCTGGTTCAGTAGAAATGGAAATGGAAGAATTACAAGGATATTTAGAAAAAGATAGATCTCGG

CAACAACACTTTCTATATCCGCTTCTCTTTAAGGAGTATATTTACACATTTGCTCATGATCGTGGTTTAA

ATGGTTCGATTTTTTACGAATCCACGGAAATTTTTGGTTATGACAATAAATCTAGTTCAGTACTTGTGAA

ACGTTCAATTATTCGAATGTATCAACAGAATTATTTGATTTATTCGGTTAATGATTCTAACCAAAATCGA

TTCGTTGGGCACAACAATTATTTTTATTTTCATTTTTATTCTCAGATGATATTGGAAGGTTTTGCAGTCA

TTGTGGAAATTCCATTCTTGCTGCGATTAGTATCTTCCCTCGAAGAAAAAAAAATACCAAAATCTCAGAA

TTTGAATTTACGATCTATTCATTCAATATTTCCCTTTTTGGAGGACAAATTATCGCATTTAAATTATGTG

TCAGATATACTAATACCTTATCCCATCCATCTGAAAATCTTGGTTCAAATCCTTCAATGCTGGATCCAAG

ATGTTCCTTCTTTACATTTATTGCGATTCTTTCTTCACGAATATCATAATTGGAATAGTCTTATTACTCC

GAATAATTCTATTTTTTTTTCAAAAGAAAATAAAAGACTATTTCGGTTCCCATATAATTCTTATGTATCT

GAATGCGAATTTGTATTAGTTTTTCTTCGTAAACAATCTTCTTATTTACGATTAACATCTTCTGGAGCTT

TNCTTGAGCGAACACATTTCTATGGAAAAATAGAACATCTTATAGTAGTGCGCCGTAATTATTTTCAGAA

GACCCTATGGTTCTTCAAGGATCCCTTCATGCATTATGTTCGATATCAAGGAAAAGCAATTCTGGTTTCA

AAGGGGACTCATCTTCTGATGAAGAAATGGAAATGTCATCTTGTCAATTTCTGGCAATATTATTTTCACT

TTTGGTCTCAACCGTACAGGATCCATATAAACCAATTATCAAGCTGTTCTTTCTATTTTCTAGGTTATCT

TTCAAGTGTACTAATAAATCCTTCGGCGGTAAGGAATCAAATGCTAGAGAATTCATTTCTAATAGATACT

GTTATTAAAAAATTCGATACCAGAGTCCCTGTTATTCCTCTTATTGGATCATTGTCTAAAGCTAAATTTT

GTACCGTATCGGGGCATCCTATTAGTAAGCCGATCTGGACCAATTTATCAGATTGCGATATTATTGATCG

ATTTGGTCGGATATGTAGAAATCTTTCTCATTATCACAGTGGATCCTCAAAAAAACAGAGTTTGTATCGA

ATAAAGTATATACTTCGATTTTCGTGTGCTAGAACTTTGGCTCGTAAACATAAAAGTATGGTACGCGCTT

TTTTGCAAAGATTAGGTTCGGGATTATTAGAAGAATTCTTTACGGAAGAAGAACAAGTTGTTTCTTTGAT

CTTCCAAAAAACAACTTCTTTTTCTTTACATGAATCACATATAGAACGTATTTGGTATTTGGATATTATC

CGTATCAATGACCTGGTGAATTATTCATAATGGGTTTGGTGACGTGATGAGACTTATGAATAGAATAGAA

ATGATCTATAAATGATCAAGAGAGAAAAAAATTCATGAATTTTCATTCTGAAATGCTCATTGCAGTAGTG

TAGTGGTTGAATCAACTGAGTAGTCAAAATTATTATACTTTCTTCTCGGGACCCAAGTTTTATATTATAC

ATAGGGTAAAGTCGTGTGCHATGAAAAATGCAAGCACGGTTTGGGGAGGGRATCTTTTTCCCCTATTCCC

ACCCAGAAAAGTTATCCTACTCC

>Astrocaryum_minus_HQ265540.1

GGTCCTGCCTCTGGGTTCAGTAGAAATGGAAATGGAAGAATTACAAGGATATTTAGAAAAAGATAGATCT

CGGCAACAACACTTTCTATATCCGCTTCTCTTTAAGGAGTATATTTACACATTTGCTCATGATCGTGGTT

TAAATGGTTCGATTTTTTACGAATCCACGGAAATTTTTGGTTATGACAATAAATCTAGTTCAGTACTTGT

GAAACGTTCAATTATTCGAATGTATCAACAGAATTATTTGATTTATTCGGTTAATGATTCTAACCAAAAT

CGATTCGTTGGGCACAACAATTATTTTTATTTTCATTTTTATTCTCAGATGATATTGGAAGGTTTTGCAG

TCATTGTGGAAATTCCATTCTTGCTGCGATTAGTATCTTCCCTCGAAGAAAAAAAAATACCAAAATCTCA

GAATTTGAATTTACGATCTATTCATTCAATATTTCCCTTTTTGGAGGACAAATTATCGCATTTAAATTAT

GTGTCAGATATACTAATACCTTATCCCATCCATCTGAAAATCTTGGTTCAAATCCTTCAATGCTGGATCC

AAGATGTTCCTTCTTTACATTTATTGCGATTCTTTCTTCACGAATATCATAATTGGAATAGTCTTATTAC

TCCGAATAATTCGATTTTTTTTTCAAAAGAAAATAAAAGACTATTTCGGTTCCCATATAATTCTTATGTA

TCTGAATGCGAATTTGTATTCGTTTTTCTTCGTAAACAATCTTCTTATTTACGATTAACATCTTCTGGAG

CTTTTCTTGAGCGAACACATTTCTATGGAAAAATAGAACATCTTATAGTAGTGCGCCGTAATTATTTTCA

GAAGACCCTATGGTTCTTCAAGGATCCCTTCATGCATTATGTTCGATATCAAGGAAAAGCAATTCTGGTT

TCAAAGGGGACTCATCTTCTGATGAAGAAATGGAAATGTCATCTTGTCAATTTCTGGCAATATTATTTTC

ACTTTTGGTCTCAACCGTACAGGATCCATATAAACCAATTATCAAGCTGTTCTTTCTATTTTCTAGGTTA

TCTTTCAAGTGTACTAATAAATCCTTCGGCGGTAAGGAATCAAATGCTAGAGAATTCATTTCTAATAGAT

ACTGTTATTAAAAAATTCGATACCAGAGTCCCTGTTATTCCTCTTATTGGATCATTGTCTAAAGCTAAAT

TTTGTACCGTATCGGGGCATCCTATTAGTAAGCCGATCTGGACCAATTTATCAGATTGCGATATTATTGA

TCGATTTGGTCGGATATGTAGAAATCTTTCTCATTATCACAGTGGATCCTCAAAAAAACAGAGTTTGTAT

CGAATAAAGTATATACTTCGATTTTCGTGTGCTAGAACTTTGGCTCGTAAACATAAAAGTATGGTACGCG

CTTTTTTGCAAAGATTAGGTTCGGGATTATTAGAAGAATTCTTTACGGAAGAAGAACAAGTTGTTTCTTT

GATCTTCCCAAAAACAACTTCTTTTTCTTTACACGAATCACATATAGAACGTATTTGGTATTTGGATATT

ATCCGTATCAATGACCTGGTGAATTATTCATAATGGGTTTGGTGACGTGATGAGACTTATGAATAGAATA

GAAATGATCTATAAATGATCAAGAGAGAAAAAAATTCATGAATTTTCATTCTGAAATGCTCATTGCAGTA

GTGTAGTGGTTGAATCAACTGAGTAGTCAAAATTATTATACTTTCTTCTCGGGACCCAAGTTTTATATTA

TACCATAGGTAAAGTCGTGTGCAATGAAAAATGCAAGCACGGTTTGGGGAGGGATCTTTTTCCTCTATTC

CAACAAAGAAAAGTTATCTACTCCATCCNNACTAGTTAA

>Astrocaryum_malybo_HQ265538.1

GTCCTGCCTCTGGTTCAGTAGAAATGGAAATGGAAGAATTACAAGGATATTTAGAAAAAGATAGATCTCG

GCAACAACACTTTCTATATCCGCTTCTCTTTAAGGAGTATATTTACACATTTGCTCATGATCGTGGTTTA

AATGGTTCGATTTTTTACGAATCCACGGAAATTTTTGGTTATGACAATAAATCTAGTTCAGTACTTGTGA

AACGTTCAATTATTNGAATGTATCAACAGAATTATTTGATTTATTCGGTTAATGATTCTAACCAAAATCG

ATTCGTTGGGCACAACAATTATTTTTATTTTCATTTTTATTCTCAGATGATATTGGAAGGTTTTGCAGTC

ATTGTGGAAATTCCATTCTTGCTGCGATTAGTATCTTCCCTCGAAGAAAAAAAAATACCAAAATCTCAGA

ATTTGAATTTACGATCTATTCATTCAATATTTCCCTTTTTGGAGGACAAATTATCGCATTTAAATTATGT

GTCAGATATACTAATACCTTATCCCATCCATCTGAAAATCTTGGTTCAAATCCTTCAATGCTGGATCCAA

GATGTTCCTTCTTTACATTTATTGCGATTCTTTCTTCACGAATATCATAATTGGAATAGTCTTATTACTC

CGAATAATTCTATTTTTTTTTCAAAAGAAAATAAAAGACTATTTCGGTTCCCATATAATTCTTATGTATC

TGAATGCGAATTTGTATTAGTTTTTCTTCGTAAACAATCTTCTTATTTACGATTAACATCTTCTGGAGCT

TTTCTTGAGCGAACACATTTCTATGGAAAAATAGAACATCTTATAGTAGTGCGCCGTAATTATTTTCAGA

AGACCCTATGGTTCTTCAAGGATCCCTTCATGCATTATGTTCGATATCAAGGAAAAGCAATTCTGGTTTC

AAAGGGGACTCATCTTCTGATGAAGAAATGGAAATGTCATCTTGTCAATTTCTGGCAATATTATTTTCAC

TTTTGGTCTCAACCGTACAGGATCCATATAAACCAATTATCAAGCTGTTCTTTCTATTTTCTAGGTTATC

TTTCAAGTGTACTAATAAATCCTTCGGCGGTAAGGAATCAAATGCTAGAGAATTCATTTCTAATAGATAC

TGTTATTAAAAAATTCGATACCAGAGTCCCTGTTATTCCTCTTATTGGATCATTGTCTAAAGCTAAATTT

TGTACCGTATCGGGGCATCCTATTAGTAAGCCGATCTGGACCAATTTATCAGATTGCGATATTATTGATC

GATTTGGTCGGATATGTAGAAATCTTTCTCATTATCACAGTGGATCCTCAAAAAAACAGAGTTTGTATCG

AATAAAGTATATACTTCGATTTTCGTGTGCTAGAACTTTGGCTCGTAAACATAAAAGTATGGTACGCGCT

TTTTTGCAAAGATTAGGTTCGGGATTATTAGAAGAATTCTTTACGGAAGAAGAACAAGTTGTTTCTTTGA

TCTTCCCAAAAACAACTTCTTTTTCTTTACATGAATCACATATAGAACGTATTTGGTATTTGGATATTAT

CCGTATCAATGACCTGGTGAATTATTCATAATGGGTTTGGTGACGTGATGAGACTTATGAATAGAATAGA

AATGATCTATAAATGATCAAGAGAGAAAAAAATTCATGAATTTTCATTCTGAAATGCTCATTGCAGTAGT

GTAGTGGTTGAATCAACTGAGTAGTCAAAATTATTATACTTTCTTCTCGGGACCCAAGTYTTATACTTAT

ACATAGGTAMAGTCGTGTGCAATGAAAAATGCAAGCACCGGTTTGGGG

>Astrocaryum_standleyanum_GQ981942.1

TCAGATATACTAATACCTTATCCCATCCATCTGAAAATCTTGGTTCAAATCCTTCAATGCTGGATCCAAG

ATGTTCCTTCTTTACATTTATTGCGATTCTTTCTTCACGAATATCATAATTGGAATAGTCTTATTACTCC

GAATAATTCTATTTTTTTTTCAAAAGAAAATAAAAGACTATTTCGGTTCCCATATAATTCTTATGTATCT

GAATGCGAATTTGTATTAGTTTTTCTTCGTAAACAATCTTCTTATTTACGATTAACATCTTCTGGAGCTT

TTCTTGAGCGAACACATTTCTATGGAAAAATAGAACATCTTATAGTAGTGCGCCGTAATTATTTTCAGAA

GACCCTATGGTTCTTCAAGGATCCCTTCATGCATTATGTTCGATATCAAGGAAAAGCAATTCTGGTTTCA

AAGGGGACTCATCTTCTGATGAAGAAATGGAAATGTCATCTTGTCAATTTCTGGCAATATTATTTTCACT

TTTGGTCTCAACCGTACAGGATCCATATAAACCAATTATCAAGCTGTTCTTTCTATTTTCTAGGTTATCT

TTCAAGTGTACTAATAAATCCTTCGGCGGTAAGGAATCAAATGCTAGAGAATTCATTTCTAATAGATACT

GTTATTAAAAAATTCGATACCAGAGTCCCTGTTATTCCTCTTATTGGATCATTGTCTAAAGCTAAATTTT

GTACCGTATCGGGGCATCCTATTAGTAAGCCGATCTGGACCAATTTATCAGATTGCGATATTATTGATCG

ATTTGGTCGGATATGTAGAAATCTTTCTCATTATCACAGTGGATCCTCAAAAAAACAGAGTTTGTATCGA

ATAAAGT

>Astrocaryum_alatum_HQ265534.1

TCCTGCCTCTGGTTCAAGTAGAAATGGAAATGGAAGAATTACAAGGATATTTAGAAAAAGATAGATCTCG

GCAACAACACTTTCTATATCCGCTTCTCTTTAAGGAGTATATTTACACATTTGCTCATGATCGTGGTTTA

AATGGTTCGATTTTTTACGAATCCACGGAAATTTTTGGTTATGACAATAAATCTAGTTCAGTACTTGTGA

AACGTTCAATTATTCGAATGTATCAACAGAATTATTTGATTTATTCGGTTAATGATTCTAACCAAAATCG

ATTCGTTGGGCACAACAATTATTTTTATTTTTATTCTCAGATGATATTGGAAGGTTTTGCAGTCATTGTG

GAAATTCCATTCTTGCTGCGATTAGTATCTTCCCTCGAAGAAAAAAAAATACCAAAATCTCAGAATTTGA

ATTTACGATCTATTCATTCAATATTTCCCTTTTTGGAGGACAAATTATCGCATTTAAATTATGTGTCAGA

TATACTAATACCTTATCCCATCCATCTGAAAATCTTGGTTCAAATCCTTCAATGCTGGATCCAAGATGTT

CCTTCTTTACATTTATTGCGATTCTTTCTTCACGAATATCATAATTGGAATAGTCTTATTACTCCGAATA

ATTCTATTTTTTTTTCAAAAGAAAATAAAAGACTATTTCGGTTCCCATATAATTCTTATGTATCTGAATG

CGAATTTGTATTAGTTTTTCTTCGTAAACAATCTTCTTATTTACGATTAACATCTTCTGGAGCTTTTCTT

GAGCGAACACATTTCTATGGAAAAATAGAACATCTTATAGTAGTGCGCCGTAATTATTTTCAGAAGACCC

TATGGTTCTTCAAGGATCCCTTCATGCATTATGTTCGATATCAAGGAAAAGCAATTCTGGTTTCAAAGGG

GACTCATCTTCTGATGAAGAAATGGAAATGTCATCTTGTCAATTTCTGGCAATATTATTTTCACTTTTGG

TCTCAACCGTACAGGATCCATATAAACCAATTATCAAGCTGTTCTTTCTATTTTCTAGGTTATCTTTCAA

GTGTACTAATAAATCCTTCGGCGGTAAGGAATCAAATGCTAGAGAATTCATTTCTAATAGATACTGTTAT

TAAAAAATTCGATACCAGAGTCCCTGTTATTCCTCTTATTGGATCATTGTCTAAAGCTAAATTTTGTACC

GTATCGGGGCATCCTATTAGTAAGCCGATCTGGACCAATTTATCAGATTGCGATATTATTGATCGATTTG

GTCGGATATGTAGAAATCTTTCTCATTATCACAGTGGATCCTCAAAAAAACAGAGTTTGTATCGAATAAA

GTATATACTTCGATTTTCGTGTGCTAGAACTTTGGCTCGTAAACATAAAAGTATGGTACGCGCTTTTTTG

CAAAGATTAGGTTCGGGATTATTAGAAGAATTCTTTACGGAAGAAGAACAAGTTGTTTCTTTGATCTTCC

CAAAAACAACTTCTTTTTCTTTACATGAATCACATATAGAACGTATTTGGTATTTGGATATTATCCGTAT

CAATGACCTGGTGAATTATTCATAATGGGTTTGGTGACGTGATGAGACTTATGAATAGAATAGAAATGAT

CTATAAATGATCAAGAGAGAAAAAAATTCATGAATTTTCATTCTGAAATGCTCATTGCAGTAGTGTAGTG

GTTGAATCAACTGAGTAGTCAAAATTATTATACTTTCTTCTCGGGACCCAAGTTTTATATTATACATAGG

TAAAGTCGTGTGCAATGAAAAATGCAAGCACGGTTTGGGGAGGGATCTTTTTCCTCTATTCCAACAAAGA

AAAGTTATCTACTCCTCCCAACTAGTTAAA

>Astrocaryum_gynacanthum_HQ265536.1

AAATGAATGGGTCCTGCCTCTGGTTCAGTAGAAATGGAAATGGAAGAATTACAAGGATATTTAGAAAAAG

ATAGATCTCGGCAACAACACTTTCTATATCCGCTTCTCTTTAAGGAGTATATTTACACATTTGCTCATGA

TCGTGGTTTAAATGGTTCGATTTTTTACGAATCCACGGAAATTTTTGGTTATGACAATAAATCTAGTTCA

GTACTTGTGAAACGTTCAATTATTCGAATGTATCAACAGAATTATTTGATTTATTCGGTTAATGATTCTA

ACCAAAATCGATTCGTTGGGCACAACAATTATTTTTATTTTCATTTTTATTCTCAGATGATATTGGAAGG

TTTTGCAGTCATTGTGGAAATTCCATTCTTGCTGCGATTAGTATCTTCCCTCGAAGAAAAAAAAATACCA

AAATCTCAGAATTTGAATTTACGATCTATTCATTCAATATTTCCCTTTTTGGAGGACAAATTATCGCATT

TAAATTATGNGNCAGATATACTAATACCTTATCCCATCCATCTGAAAATCTTGGTTCAAATCCTTCAATG

CTGGATCCAAGATGTTCCTTCTTTACATTTATTGCGATTCTTTCTTCACGAATATCATAATTGGAATAGT

CTTATTACTCCGAATAAATCTATTTTTTTTTCAAAAGAAAATAAAAGACTATTTCGGTTCCCATATAATT

CTTATGTATCTGAATGCGAATTTGTATTAGTTTTTCTTCGTAAACAATCTTCTTATTTACGATTAACATC

TTCTGGAGCTTTTCTTGAGCGAACACATTTCTATGGAAAAATAGAACATCTTATAGTAGTGCGCCGTAAT

TATTTTCAGAAGACCCTATGGTTCTTCAAGGATCCCTTCATGCATTATGTTCGATATCAAGGAAAAGCAA

TTCTGGTTTCAAAGGGGACTCATCTTCTGATGAAGAAATGGAAATGTCATCTTGTCAATTTCTGGCAATA

TTATTTTCACTTTTGGTCTCAACCGTACAGGATCCATATAAACCAATTATCAAGCTGTTCTTTCTATTTT

CTAGGTTATCTTTCAAGTGTACTAATAAATCCTTCGGCGGTAAGGAATCAAATGCTAGAGAATTCATTTC

TAATAGATACTGTTATTAAAAAATTCGATACCAGAGTCCCTGTTATTCCTCTTATTGGATCATTGTCTAA

AGCTAAATTTTGTACCGTATCGGGGCATCCTATTAGTAAGCCGATCTGGACCAATTTATCAGATTGCGAT

ATTATTGATCGATTTGGTCGGATATGTAGAAATCTTTCTCATTATCACAGTGGATCCTCAAAAAAACAGA

GTTTGTATCGAATAAAGTATATACTTCGATTTTCGTGTGCTAGAACTTTGGCTCGTAAACATAAAAGTAT

GGTACGCGCTTTTTTGCAAAGATTAGGTTCGGGATTATTAGAAGAATTCTTTACGGAAGAAGAACAAGTT

GTTTCTTTGATCTTCCCAAAAACAACTTCTTTTTCTTTACATGAATCACATATAGAACGTATTTGGTATT

TGGATATTATCCGTATCAATGACCTGGTGAATTATTCATAATGGGTTTGGTGACGTGATGAGACTTATGA

ATAGAATAGAAATGATCTATAAATGATCAAGAGAGAAAAAAATTCATGAATTTTCATTCTGAANATGCTC

ATTGCAGTAGTGTACGTAACTTGAATCNAACTGAGTAGTCAAAATTATTATACTTTCTTCTCGGGACCCA

AGTTTTATATTATACATAGGTAAAGTCGTGTGCAATGAAAAATGCAAGCACGGTTTGGGGAGGGATCTTT

TTCCTCTATTCCAACAAAGAAAAGTTATCTACTCCATCCGGACTAGTTAA

>Astrocaryum_mexicanum_EU004872.1

CGTTCTGACCATATTGCACTATGTATCATTTGATAACCCAAAAAATGAAATGGGTCCTGCCTCTGGTTCA

AGTAGAAATGGAAATGGAAGAATTACAAGGATATTTAGAAAAAGATAGATCTCGGCAACAACACTTTCTA

TATCCACTTCTCTTTAAGGAGTATATTTACACATTTGCTCATGATCGTGGTTTAAATGGTTCGATTTTTT

ACGAATCCACGGAAATTTTTGGTTATGACAATAAATCTAGTTCAGTACTTGTGAAACGTTCAATTATTCG

AATGTATCAACAGAATTATTTGATTTATTCGGTTAATGATTCTAACCAAAATCGATTCGTTGGGCACAAC

AATTATTTTTATTTTCATTTTTATTCTCAGATGATATTGGAAAGTTTTGCAGTCATTGTGGAAATTCCAT

TCTTGCTGCGATTAGTATCTTCCCTCGAAGAAAAAAAAATACCAAAATCTCAGAATTTGAATTTACGATC

TATTCATTCAATATTTCCCTTTTTGGAGGACAAATTATCGCATTTAAATTATGTGTCAGATATACTAATA

CCTTATCCCATCCATCTGAAAATCTTGGTTCAAATCCTTCAATGCTGGATCCAAGATGTTCCTTCTTTAC

ATTTATTGCGATTCTTTCTTCACGAATATCATAATTGGAATAGTCTTATTACTCCGAATAATTCTATTTT

TTTTTCAAAAGAAAATAAAAGACTATTTCGGTTCCCATATAATTCTTATGTATCTGAATGCGAATTTGTA

TTAGTTTTTCTTCGTAAACAATCTTCTTATTTACGATTAACATCTTCTGGAGCTTTTCTTGAGCGAACAC

ATTTCTATGGAAAAATAGAACATCTTATAGTAGTGCGCCGTAATTATTTTCAGAAGACCCTATGGTTCTT

CAAGGATCCCTTCATGCATTATGTTCGATATCAAGGAAAAGCAATTCTGGTTTCAAAGGGGACTCATCTT

CTGATGAAGAAATGGAAATGTCATCTTGTCAATTTCTGGCAATATTATTTTCACTTTTGGTCTCAACCGT

ACAGGATCCATATAAACCAATTATCAAGCTGTTCTTTCTATTTTCTAGGTTATCTTTCAAGTGTACTAAT

AAATCCTTCGGCGGTAAGGAATCAAATGCTAGAGAATTCATTTCTAATAGATACTGTTATTAAAAAATTC

GATACCAGAGTCCCTGTTATTCCTCTTATTGGATCATTGTCTAAAGCTAAATTTTGTACCGTATCGGGGC

ATCCTATTAGTAAGCCGATCTGGACCAATTTATCAGATTGCGATATTATTGATCGATTTGGTCGGATATG

TAGAAATCTTTCTCATTATCACAGTGGATCCTCAAAAAAACAGAGTTTGTATCGAATAAAGTATATACTT

CGATTTTCGTGTGCTAGAACTTTGGCTCGTAAACATAAAAGTATGGTACGCGCTTTTTTGCAAAGATTAG

GTTCGGGATTATTAGAAGAATTCTTTACGGAAGAAGAACAAGTTGTTTCTTTGATCTTCCCAAAAACAAC

TTCTTTTTCTTTACATGAATCACATATAGAACGTATTTGGTATTTGGATATTATCCGTATCAATGACCTG

GTGAATTATTCATAATGGGTTTGGTGACGTGATGAGACTTATGAATAGAATAGAAATGATCTATAAATGA

TCAAGAGAGAAAAAAATTCATGAATTTTCATTCTGAAATGCTCATTGCAGTAGTGTAGTGGTTGAATCAA

CTGAGTAGTCAAAATTATTATACTTTCTTCTCGGGACCCAAGTTTTATATTATACATAGGTAAAGTCGGG

TGCAATGA

>Astrocaryum_huaimi_EU004871.1

TTGCACTATGTATCATTTGATAACCCAAAAAATGAAATGGGTCCTGCCTCTGGTTCAAGTAGAAATGGAA

ATGGAAGAATTACAAGGATATTTAGAAAAAGATAGATCTCGGCAACAACACTTTCTATATCCGCTTCTCT

TTAAGGAGTATATTTACACATTTGCTCATGATCGTGGTTTAAATGGTTCGATTTTTTACGAATCCACGGA

AATTTTTGGTTATGACAATAAATCTAGTTCAGTACTTGTGAAACGTTCAATTATTCGAATGTATCAACAG

AATTATTTGATTTATTCGGTTAATGATTCTAACCAAAATCGATTCGTTGGGCACAACAATTATTTTTATT

TTCATTTTTATTCTCAGATGATATTGGAAGGTTTTGCAGTCATTGTGGAAATTCCATTCTTGCTGCGATT

AGTATCTTCCCTCGAAGAAAAAAAAATACCAAAATCTCAGAATTTGAATTTACGATCTATTCATTCAATA

TTTCCCTTTTTGGAGGACAAATTATCGCATTTAAATTATGTGTCAGATATACTAATACCTTATCCCATCC

ATCTGAAAATCTTGGTTCAAATCCTTCAATGCTGGATCCAAGATGTTCCTTCTTTACATTTATTGCGATT

CTTTCTTCACGAATATCATAATTGGAATAGTCTTATTACTCCGAATAATTCTATTTTTTTTTCAAAAGAA

AATAAAAGACTATTTCGGTTCCCATATAATTCTTATGTATCTGAATGCGAATTTGTATTGGTTTTTCTTC

GTAAACAATCTTCTTATTTACGATTAACATCTTCTGGAGCTTTTCTTGAGCGAACACATTTCTATGGAAA

AATAGAACATCTTATAGTAGTGCGCCGTAATTATTTTCAGAAGACCCTATGGTTCTTCAAGGATCCCTTC

ATGCATTATGTTCGATATCAAGGAAAAGCAATTCTGGTTTCAAAGGGGACTCATCTTCTGATGAAGAAAT

GGAAATGTCATCTTGTCAATTTCTGGCAATATTATTTTCACTTTTGGTCTCAACCGTACAGGATCCATAT

AAACCAATTATCAAGCTGTTCTTTCTATTTTCTAGGTTATCTTTCAAGTGTACTAATAAATCCTTCGGCG

GTAAGGAATCAAATGCTAGAGAATTCATTTCTAATAGATACTGTTATTAAAAAATTCGATACCAGAGTCC

CTGTTATTCCTCTTATTGGATCATTGTCTAAAGCTAAATTTTGTACCGTATCGGGGCATCCTATTAGTAA

GCCGATCTGGACCAATTTATCAGATTGCGATATTATTGATCGATTTGGTCGGATATGTAGAAATCTTTCT

CATTATCACAGTGGATCCTCAAAAAAACAGAGTTTGTATCGAATAAAGTATATACTTCGATTTTCGTGTG

CTAGAACTTTGGCTCGTAAACATAAAAGTATGGTACGCGCTTTTTTGCAAAGATTAGGTTCGGGATTATT

AGAAGAATTCTTTACGGAAGAAGAACAAGTTGTTTCTTTGATCTTCCCAAAAACAACTTCTTTTTCTTTA

CATGAATCACATATAGAACGTATTTGGTATTTGGATATTATCCGTATCAATGACCTGGTGAATTATTCAT

AATGGGTTTGGTGACGTGATGAGACTTATGAATAGAATAGAAATGATCTATAAATGATCAAGAGAGAAAA

AAATTCATGAATTTTCATTCTGAAATGCTCATTGCAGTAGTGTAGTGGTTGAATCAACTGAGTAGTCAAA

ATTATTATACTTTCTTCTCGGGACCC

>Astrocaryum_murumuru_EU004873.1

TTGCACTATGTATCATTTGATAACCCAAAAAATGAAATGGGTCCTGCCTCTGGTTCAAGTAGAAATGGAA

ATGGAAGAATTACAAGGATATTTAGAAAAAGATAGATCTCGGCAACAACACTTTCTATATCCGCTTCTCT

TTAAGGAGTATATTTACACATTTGCTCATGATCGTGGTTTAAATGGTTCGATTTTTTACGAATCCACGGA

AATTTTTGGTTATGACAATAAATCTAGTTCAGTACTTGTGAAACGTTCAATTATTCGAATGTATCAACAG

AATTATTTGATTTATTCGGTTAATGATTCTAACCAAAATCGATTCGTTGGGCACAACAATTATTTTTATT

TTCATTTTTATTCTCAGATGATATTGGAAGGTTTTGCAGTCATTGTGGAAATTCCATTCTTGCTGCGATT

AGTATCTTCCCTCGAAGAAAAAAAAATACCAAAATCTCAGAATTTGAATTTACGATCTATTCATTCAATA

TTTCCCTTTTTGGAGGACAAATTATCGCATTTAAATTATGTGTCAGATATACTAATACCTTATCCCATCC

ATCTGAAAATCTTGGTTCAAATCCTTCAATGCTGGATCCAAGATGTTCCTTCTTTACATTTATTGCGATT

CTTTCTTCACGAATATCATAATTGGAATAGTCTTATTACTCCGAATAATTCTATTTTTTTTTCAAAAGAA

AATAAAAGACTATTTCGGTTCCCATATAATTCTTATGTATCTGAATGCGAATTTGTATTAGTTTTTCTTC

GTAAACAATCTTCTTATTTACGATTAACATCTTCTGGAGCTTTTCTTGAGCGAACACATTTCTATGGAAA

AATAGAACATCTTATAGTAGTGCGCCGTAATTATTTTCAGAAGACCCTATGGTTCTTCAAGGATCCCTTC

ATGCATTATGTTCGATATCAAGGAAAAGCAATTCTGGTTTCAAAGGGGACTCATCTTCTGATGAAGAAAT

GGAAATGTCATCTTGTCAATTTCTGGCAATATTATTTTCACTTTTGGTCTCAACCGTACAGGATCCATAT

AAACCAATTATCAAGCTGTTCTTTCTATTTTCTAGGTTATCTTTCAAGTGTACTAATAAATCCTTCGGCG

GTAAGGAATCAAATGCTAGAGAATTCATTTCTAATAGATACTGTTATTAAAAAATTCGATACCAGAGTCC

CTGTTATTCCTCTTATTGGGTCATTGTCTAAAGCTAAATTTTGTACCGTATCGGGGCATCCTATTAGTAA

GCCGATCTGGACCAATTTATCAGATTGCGATATTATTGATCGATTTGGTCGGATATGTAGAAATCTTTCT

CATTATCACAGTGGATCCTCAAAAAAACAGAGTTTGTATCGAATAAAGTATATACTTCGATTTTCGTGTG

CTAGAACTTTGGCTCGTAAACATAAAAGTATGGTACGCGCTTTTTTGCAAAGATTAGGTTCGGGATTATT

AGAAGAATTCTTTACGGAAGAAGAACAAGTTGTTTCTTTGATCTTCCCAAAAACAACTTCTTTTTCTTTA

CATGAATCACATATAGAACGTATTTGGTATTTGGATATTATCCGTATCAATGACCTGGTGAATTATTCAT

AATGGGTTTGGTGACGTGATGAGACTTATGAATAGAATAGAAATGATCTATAAATGATCAAGAGAGAAAA

AAATTCATGAATTTTCATTCTGAAATGCTCATTGCAGTAGTGTAGTGGTTGAATCAACTGAGTAGTCAAA

ATTATTATACTTTCTTCTCGGGACCC

>Attalea_butyracea_JQ586690.1

CCTTCTTTACATTTATTGCGATTCTTTCTTCACGAATATCATAATTGGAATAGTCTTATTACTCCGAATA

ATTCTATTTTTTTTTCAAAAGAAAATAAAAGACTATTTCGGTTCCCATATAATTCTTATGTATCTGAATG

CGAATTTGTATTAGTTTTTCTTCGTAAACAATCTTCTTATTTACGATTAACATCTTCTGGAGCTTTTCTT

GAGCGAACACATTTCTATGGAAAAATAGAACATCTTATAGTAGTGCGCCGTAATTATTTTCAGAAGACCC

TATGGTTCTTCAAGGATCCCTTCATGCATTATGTTCGATATCAAGGAAAAGCAATTCTGGTTTCAAAGGG

GACTCATCTTCTGATGAAGAAATGGAAATGTCATCTTGTCAATTTCTGGCAATATTATTTTCACTTTTGG

TCTCAACCGTACAGGATCCATATAAACCAATTATCAAGCTGTTCTTTCTATTTTCTAGGTTATCTTTCAA

GTGTACTAATAAATCCTTCGGCGGTAAGGAATCAAATGCTAGAGAATTCATTTCTAATAGATACTCTTAT

TAAAAAATTCGATACCAGAGTCCCAGTTATTCCTCTTATTGGATCATTGTCTAAAGCTAAATTTTGTACC

GTATCGGGGCATCCTATTAGTAAGCCGACCTGGACCAATTTATCAGATTGCGATATTATTGATCGATTTG

GTCGGATATGTAGAAATCTTTCTCATTATCACAGTGGATCCTCAAAAAAACAGAGTTTGTATCGAATAA

>Attalea_butyracea_JQ586691.1

CCTTCTTTACATTTATTGCGATTCTTTCTTCACGAATATCATAATTGGAATAGTCTTATTACTCCGAATA

ATTCTATTTTTTTTTCAAAAGAAAATAAAAGACTATTTCGGTTCCCATATAATTCTTATGTATCTGAATG

CGAATTTGTATTAGTTTTTCTTCGTAAACAATCTTCTTATTTACGATTAACATCTTCTGGAGCTTTTCTT

GAGCGAACACATTTCTATGGAAAAATAGAACATCTTATAGTAGTGCGCCGTAATTATTTTCAGAAGACCC

TATGGTTCTTCAAGGATCCCTTCATGCATTATGTTCGATATCAAGGAAAAGCAATTCTGGTTTCAAAGGG

GACTCATCTTCTGATGAAGAAATGGAAATGTCATCTTGTCAATTTCTGGCAATATTATTTTCACTTTTGG

TCTCAACCGTACAGGATCCATATAAACCAATTATCAAGCTGTTCTTTCTATTTTCTAGGTTATCTTTCAA

GTGTACTAATAAATCCTTCGGCGGTAAGGAATCAAATGCTAGAGAATTCATTTCTAATAGATACTCTTAT

TAAAAAATTCGATACCAGAGTCCCAGTTATTCCTCTTATTGGATCATTGTCTAAAGCTAAATTTTGTACC

GTATCGGGGCATCCTATTAGTAAGCCGACCTGGACCAATTTATCAGATTGCGATATTATTGATCGATTTG

GTCGGATATGTAGAAATCTTTCTCATTATCACAGTGGATCCTCAAAAAAACAGAGTTTGTATCGAATAA

>Attalea_phalerata_HQ265548.1

AAATGAATGGGTCCTGTCTCTGGTTCAGTAGAAATGTAAATGGAAGAATTACAAGGATATTTAGAAAAAG

ATAGATCTCGGCAACAACACTTTCTATATCCGCTTCTCTTTAAGGAGTATATTTACACATTTGCTCATGA

TCGTGGTTTAAATGGTTCGATTTTTTACGAATCCACGGAAATTTTTGGTTATGACAATAAATCTAGTTCA

GTACTTGTGAAACGTTCAATTATTCGAATGTATCAACAGAATTATTTGATTTATTCGGTTAATGATTCTA

ACCAAAATCGATTCGTTGGGCACAACAATTATTTTGATTTTCATTTTTATTCTCAGATGATATTGGAAGG

TTTTGCAGTCATTGTGGAAATTCCATTCTTGCTGCGATTAGTATCTTCCCCCGAAGAAAAAAAAATACCA

AAATCTCAGAATTTGAATTTACGATCTATTCATTCAATATTTCCCTTTTTGGAGGACAAATTATCGCATT

TAAATTATGTGTCAGATATACTAATACCTTATCCCATCCATCTGAAAATCTTGGTTCAAATCCTTCAATG

CTGGATCCAAGATGTTCCTTCTTTACATTTATTGCGATTCTTTCTTCACGAATATCATAATTGGAATAGT

CTTATTACTCCGAATAATTCTATTTTTTTTTCAAAAGAAAATAAAAGACTATTTCGGTTCCCATATAATT

CTTATGTATCTGAATGCGAATTTGTATTAGTTTTTCTTCGTAAACAATCTTCTTATTTACGATTAACATC

TTCTGGAGCTTTTCTTGAGCGAACACATTTCTATGGAAAAATAGAACATCTTATAGTAGTGCGCCGTAAT

TATTTTCAGAAGACCCTATGGTTCTTCAAGGATCCCTTCATGCATTATGTTCGATATCAAGGAAAAGCAA

TTCTGGTTTCAAAGGGGACTCATCTTCTGATGAAGAAATGGAAATGTCATCTTGTCAATTTCTGGCAATA

TTATTTTCACTTTTGGTCTCAACCGTACAGGATCCATATAAACCAATTATCAAGCTGTTCTTTCTATTTT

CTAGGTTATCTTTCAAGTGTACTAATAAATCCTTCGGCGGTAAGGAATCAAATGCTAGAGAATTCATTTC

TAATAGATACTCTTATTAAAAAATTCGATACCAGAGTCCCAGTTATTCCTCTTATTGGATCATTGTCTAA

AGCTAAATTTTGTACCGTATCGGGGCATCCTATTAGTAAGCCGACCTGGACCAATTTATCAGATTGCGAT

ATTATTGATCGATTTGGTCGGATATGTAGAAATCTTTCTCATTATCACAGTGGATCCTCAAAAAAACAGA

GTTTGTATCGAATAAAGTATATACTTCGATTTTCGTGTGCTAGAACTTTGGCTCGTAAACATAAAAGTAT

GGTACGCGCTTTTTTGCAAAGATTAGGTTCGGGATTATTAGAAGAATTCTTTACGGAAGAAGAAGAAGTT

GTTTCTTTGATCTTCCCAAAAACAACCTCTTTTTCTTTACATGAATCACATATAGAACGTATTTGGTATT

TGGATATTATCCGTATCAATGACCTGGTGAATTATTCATAATGGGTTTGGTGACGTGATGAGACTTATGA

ATAGAATAGAAATGATCTATAAATGATCAAGAGAGAAAAAAATTCATGAATTTTCATTCTGAAATGCTCA

TTGCAGTAGTGTAGTGGTTGAATCAACTGAGTAGTCAAAATTATTATACTTTCTTCTCGGGACCCAAGTT

TTATATTATACATAGGTAAAGTCGTGTGCAATGAAAAATGCAAGCACGGTTTGGGGAGGGATCTTTTTCC

TCTATTCCAACAAAGAAAAGTTATCTACTCCATCCNNACTAGTTAA

>Attalea_rostrata_GQ981943.1

GTGTCAGATATACTAATACCTTATCCCATCCATCTGAAAATCTTGGTTCAAATCCTTCAATGCTGGATCC

AAGATGTTCCTTCTTTACATTTATTGCGATTCTTTCTTCACGAATATCATAATTGGAATAGTCTTATTAC

TCCGAATAATTCTATTTTTTTTTCAAAAGAAAATAAAAGACTATTTCGGTTCCCATATAATTCTTATGTA

TCTGAATGCGAATTTGTATTAGTTTTTCTTCGTAAACAATCTTCTTATTTACGATTAACATCTTCTGGAG

CTTTTCTTGAGCGAACACATTTCTATGGAAAAATAGAACATCTTATAGTAGTGCGCCGTAATTATTTTCA

GAAGACCCTATGGTTCTTCAAGGATCCCTTCATGCATTATGTTCGATATCAAGGAAAAGCAATTCTGGTT

TCAAAGGGGACTCATCTTCTGATGAAGAAATGGAAATGTCATCTTGTCAATTTCTGGCAATATTATTTTC

ACTTTTGGTCTCAACCGTACAGGATCCATATAAACCAATTATCAAGCTGTTCTTTCTATTTTCTAGGTTA

TCTTTCAAGTGTACTAATAAATCCTTCGGCGGTAAGGAATCAAATGCTAGAGAATTCATTTCTAATAGAT

ACTCTTATTAAAAAATTCGATACCAGAGTCCCAGTTATTCCTCTTATTGGATCATTGTCTAAAGCTAAAT

TTTGTACCGTATCGGGGCATCCTATTAGTAAGCCGACCTGGACCAATTTATCAGATTGCGATATTATTGA

TCGATTTGGTCGGATATGTAGAAATCTTTCTCATTATCACAGTGGATCCTCAAAAAAACCAGAGTTTGTA

TCGAATAA

>Attalea_allenii_AM114636.1

GACCATATTGCACTATGTATCATTTGATAACCCAAAAAATGAAATGGGTCCTGTCTCTGGTTCAAGTAGA

AATGTAAATGGAAGAATTACAAGGATATTTAGAAAAAGATAGATCTCGGCAACAACACTTTCTATATCCG

CTTCTCTTTAAGGAGTATATTTACACATTTGCTCATGATCGTGGTTTAAATGGTTCGATTTTTTACGAAT

CCACGGAAATTTTTGGTTATGACAATAAATCTAGTTCAGTACTTGTGAAACGTTCAATTATTCGAATGTA

TCAACAGAATTATTTGATTTATTCGGTTAATGATTCTAACCAAAATCGATTCGTTGGGCACAACAATTAT

TTTGATTTTCATTTTTATTCTCAGATGATATTGGAAGGTTTTGCAGTCATTGTGGAAATTCCATTCTTGC

TGCGATTAGTATCTTCCCCCGAAGAAAAAAAAATACCAAAATCTCAGAATTTGAATTTACGATCTATTCA

TTCAATATTTCCCTTTTTGGAGGACAAATTATCGCATTTAAATTATGTGTCAGATATACTAATACCTTAT

CCCATCCATCTGAAAATCTTGGTTCAAATCCTTCAATGCTGGATCCAAGATGTTCCTTCTTTACATTTAT

TGCGATTCTTTCTTCACGAATATCATAATTGGAATAGTCTTATTACTCCGAATAATTCTATTTTTTTTTC

AAAAGAAAATAAAAGACTATTTCGGTTCCCATATAATTCTTATGTATCTGAATGCGAATTTGTATTAGTT

TTTCTTCGTAAACAATCTTCTTATTTACGATTAACATCTTCTGGAGCTTTTCTTGAGCGAACACATTTCT

ATGGAAAAATAGAACATCTTATAGTAGTGCGCCGTAATTATTTTCAGAAGACCCTATGGTTCTTCAAGGA

TCCCTTCATGCATTATGTTCGATATCAAGGAAAAGCAATTCTGGTTTCAAAGGGGACTCATCTTCTGATG

AAGAAATGGAAATGTCATCTTGTCAATTTCTGGCAATATTATTTTCACTTTTGGTCTCAACCGTACAGGA

TCCATATAAACCAATTATCAAGCTGTTCTTTCTATTTTCTAGGTTATCTTTCAAGTGTACTAATAAATCC

TTCGGCGGTAAGGAATCAAATGCTAGAGAATTCATTTCTAATAGATACTCTTATTAAAAAATTCGATACC

AGAGTCCCAGTTATTCCTCTTATTGGATCATTGTCTAAAGCTAAATTTTGTACCGTATCGGGGCATCCTA

TTAGTAAGCCGACCTGGACCAATTTATCAGATTGCGATATTATTGATCGATTTGGTCGGATATGTAGAAA

TCTTTCTCATTATCACAGTGGATCCTCAAAAAAACAGAGTTTGTATCGAATAAAGTATATACTTCGATTT

TCGTGTGCTAGAACTTTGGCTCGTAAACATAAAAGTATGGTACGCGCTTTTTTGCAAAGATTAGGTTCGG

GATTATTAGAAGAATTCTTTACGGAAGAAGAAGAAGTTGTTTCTTTGATCTTCCCAAAAACAACCTCTTT

TTCTTTACATGAATCACATATAGAACGTATTTGGTATTTGGATATTATCCGTATCAATGACCTGGTGAAT

TATTCATAATGGGTTTGGTGACGTGATGAGACTTATGAATAGAATAGAAATGATCTATAAATGATCAAGA

GAGAAAAAAATTCATGAATTTTCATTCTGAAATGCTCATTGCAGTAGTGTAGTGGTTGAATCAACTGAGT

AGTCAAAATTATTATACTTTCTTCTCGGGACCCAAGTTTTATATTATACATAGGTAAAGTC

>Bactris_simplicifrons_HQ265561.1

TTCACTTTTGGTCTCAACCGTACAGGATCCATATAAACCAATTATCAAGCTGTTCTTTCTATTTTCTAGG

TTATCTTTCAAGTGTACTAATAAATCCTTCGGCGGTAAGGAATCAAATGCTAGAGAATTCATTTCTAATA

GATACTGTTATTAAAAAATTCGATACCAGAGTCCCTGTTATTCCTCTTATTGGATCATTGTCTAAAGCGA

AATTTTGTACCGTATCGGGGCATCCTATTAGTNATCCGATCTGGNCCANTTTATCAGATTGCGATATTAT

TGATCGATTTGGTCGGATATGTAGAAATCTNTCTCATNANCACAGTGGATCCTCAAAAAAACAGAGTTCG

NANCGAATAAAGTATATACNTCGNTNTTCGTGNGCTAGNNCNNTGGNTCNNNNACATNNAANTATGNTCC

GCGCTTTTTTGCAAAGATTAGGTTCGGGATTATTAGAAGAATTCTTTACGGAAGAAGAACAAGTTGTTTC

TTTGATCTTCCCAAAAACAACTTCTTTTTCTTTACATGAATCACATATAGAACGTATTTGGTATTTGGAT

ATTATCCGTATCAATGACCTGGTGAATTATTCATAATGGGTTTGGTGACGTGATGAGACTTATGAATAGA

ATAGAAATGATCTATAAATGATCAAGAGAGAAAAAAATTCATGAATTTTCTTTCTGAAATGCTCATTGCA

GTAGTGTAGTGGTTGAATCAACTGAGTAGTCAAAATTATTATACTTTCTTCTCGGGACCCAAGTTTTATA

TTATACATAGGTAAAGTCGTGTGCAATGAAAAATGCAAGCACGGTTTGGGGAGGGATCTTTTTCCTCTAT

TCCAACAAAGAAAAGTTATCTACTCCATCCGAACTAGTTAA

>Bactris_riparia_HQ265559.1

GGGTCCTGCCTCTGGTTCAAGTAGAAATGGAAATGGAAGAATTACAAGGATATTTAGAAAAAGATAGATC

TCGGCAACAACACTTTCTATATCCGCTTCTCTTTAAGGAGTATATTTACACATTTGCTCATGATCGTGGT

TTAAATGGTTCGATTTTTTACGAATCCACGGAAATTTTTGGTTATGACAATAAATCTAGTTCAGTACTTG

TGAAACGTTCAATTATTCGAATGTATCAACAGAATTATTTGATTTATTCGGTTAATGATTCTAACCAAAA

TCGATTCGTTGGGCACAACAATTATTTTGATTTTCATTTTTATTCTCAGATGATATTGGAAGGTTTTGCA

GTCATTGTGGAAATTCCATTCTTGCTGCGATTAGTATCTTCCCTCGAAGAAAAAAAAATACCAAAATCTC

AGAATTTGAATTTACGATCTATTCATTCAATATTTCCCTTTTTGGAGGACAAATTATCGCATTTAAATTA

TGTGTCAGATATACTAATACCTTATCCCATCCATCTGAAAATCTTGGTTCAAATCCTTCAATGCTGGATC

CAAGATGTTCCTTCTTTACATTTATTGCGATTCTTTCTTCACGAATATCATAATTGGAATAGTCTTATTA

CTCCGAATAATTCTATTTTTTTTTCAAAAGAAAATAAAAGACTATTTCGGTTCCCATATAATTCTTATGT

ATCTGAATGCGAATTTGTATTAGTTTTTCTTCGTAAACAATCTTCTTATTTACGATTAACATCTTCTGGA

GCTTTTCTTGAGCGAACACATTTCTATGGAAAAATAGAACATCTTATAGTAGTGCGCCGTAATTATTTTC

AGAAGACCCTATGGTTCTTCAAGGATCCCTTCATGCATTATGTTCGATATCAAGGAAAGGCAATTCTGGT

TTCAAAGGGGACTCATCTTCTGATGAAGAAATGGAAATGTCATCTTGTCAATTTCTGGCAATATTATTTT

CACTTTTGGTCTCAACCGTACAGGATCCATATAAACCAATTATCAAGCTGTTCTTTCTATTTTCTAGGTT

ATCTTTCAAGTGTACTAATAAATCCTTCGGCGGTAAGGAATCAAATGCTAGAGAATTCATTTCTAATAGA

TACTGTTATTAAAAAATTCGATACCAGAGTCCCTGTTATTCCTCTTATTGGATCATTGTCTAAAGCTAAA

TTTTGTACCGTATCGGGGCATCCTATTAGTAAGCCGATCTGGACCAATTTATCAGATTGCGATATTATTG

ATCGATTTGGTCGGATATGTAGAAATCTTTCTCATTATCACAGTGGATCCTCAAAAAAACAGAGTTTGTA

TCGAATAAAGTATATACTTCGATTTTCGTGTGCTAGAACTTTGGCTCGTAAACATAAAAGTATGGTACGC

GCTTTTTTGCAAAGATTAGGTTCGGGATTATTAGAAGAATTCTTTACGGAAGAAGAACAAGTTGTTTCTT

TGATCTTCCCAAAAACAACTTCTTTTTCTTTACATGAATCACATATAGAACGTATTTGGTATTTGGATAT

TATCCGTATCAATGACCTGGTGAATTATTCATAATGGGTTTGGTGACGTGATGAGACTTATGAATAGAAT

AGAAATGATCTATAAATGATCAATAGAGAAAAAAATTCATGAATTTTCATTCTGAAATGCTCATTGCAGT

AGTGTAGTGGTTGAATCAACTGAGTAGTCAAAATTATTATACTTTCTTCTCGGGACCCAAGTTTTATATT

ATACCATAGGTAAAGTCGTGTGCAATGAAAAATGCAAGCACCGGTTTGGGGAGGG

>Bactris_maraja_HQ265557.1

TGAATGGGTCCTGCCTCTGGTTCAGTAGAAATGGAAATGGAAGAATTACAAGGATATTTAGAAAAAGATA

GATCTCGGCAACAACACTTTCTATATCCGCTTCTCTTTAAGGAGTATATTTACACATTTGCTCATGATCG

TGGTTTAAATGGTTCGATTTTTTACGAATCCACGGAAATTTTTGGTTATGACAATAAATCTAGTTCAGTA

CTTGTGAAACGTTCAATTATTCGAATGTATCAACAGAATTATTTGATTTATTCGGTTAATGATTCTAACC

AAAATCGATTCGTTGGGCACAACAATTATTTTTATTTTCATTTTTATTCTCAGATGATATTGGAAGGTTT

TGCAGTCATTGTGGAAATTCCATTCTTGCTGCGATTAGTATCTTCCCTCGAAGAAAAAAAAATACCAAAA

TCTCAGAATTTGAATTTACGATCTATTCATTCAATATTTCCCTTTTTGGAGGACAAATTATCGCATTTAA

ATTATGNGNCAGATATACTAATACCTTATCCCATCCATCTGAAAATCTTGGTTCAAATCCTTCAATGCTG

GATCCAAGATGTTCCTTCTTTACATTTATTGCGATTCTTTCTTCACGAATATCATAATTGGAATAGTCTT

ATTACTCCGAATAATTCTATTTTTTTTTCAAAAGAAAATAAAAGACTATTTCGGTTCCCATATAATTCTT

ATGTATCTGAATGCGAATTTGTATTAGTTTTTCTTCGTAAACAATCTTCTTATTTACGATTAACATCTTC

TGGAGCTTTTCTTGAGCGAACACATTTCTATGGAAAAATGGAACATCTTATAGTAGTGCGCCGTAATTAT

TTTCAGAAGACCCTATGGTTCTTCAAGGATCCCTTCATGCATTATGTTCGATATCAAGGAAAGGCAATTC

TGGTTTCAAAGGGGACTCATCTTCTGATGAAGAAATGGAAATGTCATCTTGTCAATTTCTGGCAATATTA

TTTTCACTTTTGGTCTCAACCGTACAGGATCCATATAAACCAATTATCAAGCTGTTCTTTCTATTTTCTA

GGTTATCTTTCAAGTGTACTAATAAATCCTTCGGCGGTAAGGAATCAAATGCTAGAGAATTCATTTCTAA

TAGATACTGTTATTAAAAAATTCGATACCAGAGTCCCTGTTATTCCTCTTATTGGATCATTGTCTAAAGC

TAAATTTTGTACCGTATCGGGGCATCCTATTAGTAAGCCGATCTGGACCAATTTATCAGATTGCGATATT

ATTGATCGATTTGGTCGGATATGTAGAAATCTTTCTCATTATCACAGTGGATCCTCAAAAAAACAGAGTT

TGTATCGAATAAAGTATATACTTCGATTTTCGTGTGCTAGAACTTTGGCTCGTAAACATAAAAGTATGGT

ACGCGCTTTTTTGCAAAGATTAGGTTCGGGATTATTAGAAGAATTCTTTACGGAAGAAGAACAAGTTGTT

TCTTTGATCTTCCCAAAAACAACTTCTTTTTCTTTACATGAATCACATATAGAACGTATTTGGTATTTGG

ATATTATCCGTATCAATGACCTGGTGAATTATTCATAATGGGTTTGGTGACGTGATGAGACTTATGAATA

GAATAGAAATGATCCATAAATGATCAATAGAGAAAAAAATTCATGAATTTTCATTCTGAAATGCTCATTG

CAGTAGTGTAGTGGTTGAATCAACTGAGTAGTCAAAATTATTATACTTTCTTCTCGGGACCCAAGTTTTA

TATTATACATAGGTAAAGTCGTGTGCAATGAAAAATGCAAGCACGGTTTGGGGAGGGATCTTTTTCCTCT

ATTCCAACAAAGAAAAGTTATCTACTCCATCCGACTAGTTAA

>Bactris_killipii_HQ265555.1

GGGTCCTGCCTCTGGTTCAGTAGAAATGGAAATGGAAGAATTACAAGGATATTTAGAAAAAGATAGATCT

CGGCAACAACACTTTCTATATCCGCTTCTCTTTAAGGAGTATATTTATACATTTGCTCATGATCGTGGTT

TAAATGGTTCGATTTTTTACGAATCCACGGAAATTTTTGGTTATGACAATAAATCTAGTTCAGTACTTGT

GAAACGTTCAATTATTCGAATGTATCAACAGAATTATTTGATTTATTCGGTTAATGATTCTAACCAAAAT

CGATTCGTTGGGCACAACAATTATTTTTATTTTCATTTTTATTCTCAGATGATATTGGAAGGTTTTGCAG

TCATTGTGGAAATTCCATTCTTGCTGCGATTAGTATCTTCCCTCGAAGAAAAAAAAATACCAAAATCTCA

GAATTTGAATTTACGATCTATTCATTCAATATTTCCCTTTTTGGAGGACAAATTATCGCATTTAAATTAT

GTGTCAGATATACTAATACCTTATCCCATACATCTGAAAATCTTGGTTCAAATCCTTCAATGCTGGATCC

AAGATGTTCCTTCTTTACATTTATTGCGATTCTTTCTTCACGAATATCATAATTGGAATAGTCTTATTAC

TCCGAATAATTCTATTTTTTTTTCAAAAGAAAATAAAAGACTATTTCGGTTCCCATATAATTCTTATGTA

TCTGAATGCGAATTTGTATTAGTTTTTCTTCGTAAACAATCTTCTTATTTACGATTAACATCTTCTGGAG

CTTTTCTTGAGCGAACACATTTCTATGGAAAAATAGAACATCTTATAGTAGTGCGCCGTAATTATTTTCA

GAAGACCCTATGGTTCTTCAAGGATCCCTTCATGCATTATGTTCGATATCAAGGAAAGGCAATTCTGGTT

TCAAAGGGGACTCATCTTCTGATGAAGAAATGGAAATGTCATCTTGTCAATTTCTGGCAATATTATTTTC

ACTTTTGGTCTCAACCGTACAGGATCCATATAAACCAATTATCAAGCTGTTCTTTCTATTTTCTAGGTTA

TCTTTCAAGTGTACTAATAAATCCTTCGGCGGTAAGGAATCAAATGCTAGAGAATTCATTTCTAATAGAT

ACTGTTATTAAAAAATTCGATACCAGAGTCCCTGTTATTCCTCTTATTGGATCATTGTCTAAAGCTAAAT

TTTGTACCGTATCGGGGCATCCTATTAGTAAGCCGATCTGGACCAATTTATCAGATTGCGATATTATTGA

TCGATTTGGTCGGATATGTAGAAATCTTTCTCATTATCACAGTGGATCCTCAAAAAAACAGAGTTTGTAT

CGAATAAAGTATATACTTCGATTTTCGTGTGCTAGAACTTTGGCTCGTAAACATAAAAGTATGGTACGCG

CTTTTTTGCAAAGATTAGGTTCGGGATTATTAGAAGAATTCTTTACGGAAGAAGAACAAGTTGTTTCTTT

GATCTTCCCAAAAACAACTTCTTTTTCTTTACATGAATCACATATAGAACGTATTTGGTATTTGGATATT

ATCCGTATCAATGACCTGGTGAATTATTCATAATGGGTTTGGTGWCGTGATGAGACTTATGAATAGAATA

GAAATGATCTATAAATGATCAATAGAGAAAAAAATTCATGAATTTTCATTCTGAAATGCTCATTGCAGTA

GTGTAGTGGTTGAATCAACTGAGTAGTCAAAATTATTATACTTTCTTCTCGGGACCCAAGTTTTATATTA

TACATAGGTAAAGTCGTGTGCAATGAAAAATGCAAGCACGGTTTGGGGAGGGATCTTTTTCCTCTATTCC

AACAAAGAAAAGTTATCTACTCCATCCNAACTAGTTAA

>Bactris_ferruginea_HQ265553.1

AAATGAATGGGTCCTGCCTCTGGTTCAGTAGAAATGGAAATGGAAGAATTACAAGGATATTTAGAAAAAG

ATAGATCTCGGCAACAACACTTTCTATATCCGCTTCTCTTTAAGGAGTATATTTACACATTTGCTCATGA

TCGTGGTTTAAATGGTTCGATTTTTTACGAATCCACGGAAATTTTTGGTTATGACAATAAATCTAGTTCA

GTACTTGTGAAACGTTCAATTATTCGAATGTATCAACAGAATTATTTGATTTATTCGGTTAATGATTCTA

ACCAAAATCGATTCGTTGGGCACAACAATTATTTTTATTTTCATTTTTATTCTCAGATGATATTGGAAGG

TTTTGCAGTCATTGTGGAAATTCCATTCTTGCTGCGATTAGTATCTTCCCTCGAAGAAAAAAAAATACCA

AAATCTCAGAATTTGAATTTACGATCTATTCATTCAATATTTCCCTTTTTGGAGGACAAATTATCGCATT

TAAATTATGTGTCAGATATACTAATACCTTATCCCATCCATCTGAAAATCTTGGTTCAAATCCTTCAATG

CTGGATCCAAGATGTTCCTTCTTTACATTTATTGCGATTCTTTCTTCACGAATATCATAATTGGAATAGT

CTTATTACTCCGAATAATTCTATTTTTTTTTCAAAAGAAAATAAAAGACTATTTCGGTTCCCATATAATT

CTTATGTATCTGAATGCGAATTTGTATTAGTTTTTCTTCGTAAACAATCTTCTTATTTACGATTAACATC

TTCTGGAGCTTTTCTTGAGCGAACACATTTCTATGGAAAAATAGAACATCTTATAGTAGTGCGCCGTAAT

TATTTTCAGAAGACCCTATGGTTCTTCAAGGATCCCTTCATGCATTATGTTCGATATCAAGGAAAGGCAA

TTCTGGTTTCAAAGGGGACTCATCTTCTGATGAAGAAATGGAAATGTCATCTTGTCAATTTCTGGCAATA

TTATTTTCACTTTTGGTCTCAACCGTACAGGATCCATATAAACCAATTATCAAGCTGTTCTTTCTATTTT

CTAGGTTATCTTTCAAGTGTACTAATAAATCCTTCGGCGGTAAGGAATCAAATGCTAGAGAATTCATTTC

TAATAGATACTGTTATTAAAAAATTCGATACCAGAGTCCCTGTTATTCCTCTTATTGGATCATTGTCTAA

AGCGAAATTTTGTACCGTATCGGGGCATCCTATTAGTAAGCCGATCTGGACCAATTTATCAGATTGCGAT

ATTATTGATCGATTTGGTCGGATATGTAGAAATCTTTCTCATTATCACAGTGGATCCTCAAAAAAACAGA

GTTTGTATCGAATAAAGTATATACTTCGATTTTCGTGTGCTAGAACTTTGGCTCGTAAACATAAAAGTAT

GGTACGCGCTTTTTTGCAAAGATTAGGTTCGGGATTATTAGAAGAATTCTTTACGGAAGAAGAACAAGTT

GTTTCTTTGATCTTCCCAAAAACAACTTCTTTTTCTTTACATGAATCACATATAGAACGTATTTGGTATT

TGGATATTATCCGTATCAATGACCTGGTGAATTATTCATAATGGGTTTGGTGACGTGATGAGACTTATGA

ATAGAATAGAAATGATCTATAAATGATCAAGAGAGAAAAAAATTCATGAATTTTCATTCTGAAATGCTCA

TTGCAGTAGTGTAGTGGTTGAATCAACTGAGTAGTCAAAATTATTATACTTTCTTCTCGGGACCCAAGCT

TTATATTATACATAGGTAAAGTCGTGTGCAATGAAAAATGCAAGCACGGTTTGGGGAGGGATCTTTTTCC

TCTATTCCAACAAAGAAAAGTTATCTACTCCATCCNNACTAGTTAA

>Bactris_campestris_HQ265551.1

ATGAATGGGTCCTGCCTCTGGTTCAGTAGAAATGGAAATGGAAGAATTACAAAGATATTTAGAAAAAGAT

AGATCTCGGCAACAACACTTTCTATATCCGCTTCTCTTTAAGGAGTATATTTACACATTTGCTCATGATC

GTGGTTTAAATGGTTCGATTTTTTACGAATCCACGGAAATTTTTGGTTATGACAATAAATCTAGTTCAGT

ACTTGTGAAACGTTCAATTATTCGAATGTATCAACAGAATTATTTGATTTATTCGGTTAATGATTCTAAC

CAAAATCGATTCGTTGGGCACAACAATTATTTTTATTTTCATTTTTATTCTCAGATGATATTGGAAGGTT

TTGCAGTCATTGTGGAAATTCCATTCTTGCTGCGATTAGTATCTTCCCTCGAAGAAAAAAAAATACCAAA

ATCTCAGAATTTAAATTTACGATCTATTCATTCAATATTTCCCTTTTTGGAGGACAAATTATCGCATTTA

AATTATGTGTCAGATATACTAATACCTTATCCCATCCATCTGAAAATCTTGGTTCAAATCCTTCAATGCT

GGATCCAAGATGTTCCTTCTTTACATTTATTGCGATTCTTTCTTCACGAATATCATAATTGGAATAGTCT

TATTACTCCGAATAATTCTATTTTTTTTTCAAAAGAAAATAAAAGACTATTTCGGTTCCCATATAATTCT

TATGTATCTGAATGCGAATTTGTATTAGTTTTTCTTCGTAAACAATCTTCTTATTTACGATTAACATCTT

CTGGAGCTTTTCTTGAGCGAACACATTTCTATGGAAAAATAGAACATCTTATAGTAGTGCGCCGTAATTA

TTTTCAGAAGACCCTATGGTTCTTCAAGGATCCCTTCATGCATTATGTTCGATATCAAGGAAAGGCAATT

CTGGTTTCAAAGGGGACTCATCTTCTGATGAAGAAATGGAAATGTCATCTTGTCAATTTCTGGCAATATT

ATTTTCACTTTTGGTCTCAACCATATAGGATCCATATAAACCAATTATCAAGCTGTTCTTTCTATTTTCT

AGGTTATCTTTCAAGTGTACTAATAAATCCTTCGGCGGTAAGGAATCAAATGCTAGAGAATTCATTTCTA

ATAGATACTGTTATTAAAAAATTCGATACCAGAGTCCCTGTTATTCCTCTTATTGGATCATTGTCTAAAG

CTAAATTTTGTACCGTATCGGGGCATCCTATTAGTAAGCCGATCTGGACCAATTTATCAGATTGCGATAT

TATTGATCGATTTGGTCGGATATGTAGAAATCTTTCTCATTATCACAGTGGATCCTCAAAAAAACAGAGT

TTGTATCGAATAAAGTATATACTTCGATTTTCGTGTGCTAGAACTTTGGCTCGTAAACATAAAAGTATGG

TACGCGCTTTTTTGCAAAGATTAGGTTCGGGATTATTAGAAGAATTCTTTACGGAAGAAGAACAAGTTGT

TTCTTTGATCTTCCCAAAAACAACTTCTTTTTCTTTACATGAATCACATATAGAACGTATTTGGTATTTG

GATATTATCCGTATCAATGACCTGGTGAATTATTCATAATGGGTTTGGTGACGTGATGAGACTTATGAAT

AGAATAGAAATGATCTATAAATGATCAATAGAGAAAAAAATTCATGAATTTTCATTCTGAAATGCTCATT

GCAGTAGTGTAGTGGTTGAATCAACTGAGTAGTCAAAATTATTATACTTTCTTCTCGGGACCCAAGTTTT

ATATTATACATAGGTAAAGTCGTGTGCAATGAAAAATGCAAGCACGGTTTGGGGAGGGATCTTTTTCCTC

TATTCCAACAAAGAAAAGTTATCTACTCCATCCNAACTAGTTAA

>Bactris_acanthocarpa_HQ265549.1

GAATGGGTCCTGCCTCTGGTTCAGTAGAAATGGAAATGGAAGAATTACAAGGATATTTAGAAAAAGATAG

ATCTCGGCAACAACACTTTCTATATCCGCTTCTCTTTAAGGAGTATATTTACACATTTGCTCATGATCGT

GGTTTAAATGGTTCGATTTTTTACGAATCCACGGAAATTTTTGGTTATGACAATAAATCTAGTTCAGTAC

TTGTGAAACGTTCAATTATTCGAATGTATCAGCAGAATTATTTGATTTATTCGGTTAATGATTCTAACCA

AAATCGATTCGTTGGGCACAACAATTATTTTTATTTTCATTTTTATTCTCAGATGATATTGGAAGGTTTT

GCAGTCATTGTGGAAATTCCATTCTTGCTGCGATTAGTATCTTCCCTCGAAGAAAAAAAAATACAAAAAT

CTCAGAATTTGAATTTACGATCTATTCATTCAATATTTCCCTTTTTGGAGGACAAATTATCGCATTTAAA

TTATGNGTCAGATATACTAATACCTTATCCCATCCATCTGAAAATCTTGGTTCAAATCCTTCAATGCTGG

ATCCAAGATGTTCCTTCTTTACATTTATTGCGATTCTTTCTTCACGAATATCATAATTGGAATAGTCTTA

TTACTCCGAATAATTCTATTTTTTTTTCAAAAGAAAATAAAAGACTATTTCGGTTCCCATATAATTCTTA

TGTATCTGAATGCGAATTTGTATTAGTTTTTCTTCGTAAACAATCTTCTTATTTACGATTAACATCTTCT

GGAGCTTTTCTTGAGCGAACACATTTCTATGGAAAAATAGAACATCTTATAGTAGTGCGCCGTAATTATT

TTCAGAAGACCCTATGGTTCTTCAAGGATCCCTTCATGCATTATGTTCGATATCAAGGAAAGGCAATTCT

GGTTTCAAAGGGGACTCATCTTCTGATGAAGAAATGGAAATGTCATCTTGTCAATTTCTGGCAATATTAT

TTTCACTTTTGGTCTCAACCGTACAGGATCCATATAAACCAATTATCAAGCTGTTCTTTCTATTTTCTAG

GTTATCTTTCAAGTGTACTAATAAATCCTTCGGCGGTAAGGAATCAAATGCTAGAGAATTCATTTCTAAT

AGATACTGTTATTAAAAAATTCGATACCAGAGTCCCTGTTATTCCTCTTATTGGATCATTGTCTAAAGCT

AAATTTTGTACCGTATCGGGGCATCCTATTAGTAAGCCGATCTGGNCCAATTTATCAGATTGCGATATTA

TTGATCGATTTGGTCGGATATGTAGAAATCTTTCTCATTATCACAGTGGATCCTCAAAAAAACAGAGTTT

GTATCGAATAAAGTATATACTTCGATTTTCGTGTGCTAGAACTTTGGCTCGTAAACATAAAAGTATGGTA

CGCGCTTTTTTGCAAAGATTAGGTTCGGGATTATTAGAAGAATTCTTTACGGAAGAAGAACAAGTTGTTT

CTTTGATCTTCCCAAAAACAACTTCTTTTTCTTTACATGAATCACATATAGAACGTATTTGGTATTTGGA

TATTATCCGTATCAATGACCTGGTGAATTATTCATAATGGGTTTGGTGACGTGATGAGACTTATGAATAG

AATAGAAATGATCTATAAATGATCAATAGAGAAAAAAATTCATGAATTTTCATTCTGAAATGCTCATTGC

AGTAGTGTAGTGGTTGAATCAACTGAGTAGTCAAAATTATTATACTTTCTTCTCGGGACCCAAGTTTTAT

ATTATACATAGGTAAAGTCGTGTGCAATGAAAAATGCAAGCACGGTTTGGGGAGGGATCTTTTTCCTCTA

TTCCAACAAAGAAAAGTTATCTACTCCATCCGGACTAGTTAA

>Bactris_setulosa_HQ265560.1

CCTGCCTCTGGCNTCAAGATAGAAATGGAAATGGAAGAATTACAAAGATATTTAGAAAAAGATAGATCTA

GGCAACAACACTTTCTATATCCGCTTCTCTTTAAGGAGTATATTTACACATTTGCTCATGATCGTGGTTT

AAATGGTTCGATTTTTTACGAATCCACGGAAATTTTTGGTTATGACAATAAATCTAGTTCAGTACTTGTG

AAACGTTCAATTATTCGAATGTATCAACAGAATTATTTGATTTATTCGGTTAATGATTCTAACCAAAATC

GATTCGTTGGGCACAACAATTATTTTGATTTTCATTTTTATTCTCAGATGATATTGGAAGGTTTTGCAGT

CATTGTGGAAATTCCATTCTTGCTGCGATTAGTATCTTCCCTCGAAGAAAAAAAAATACCAAAATCTCAG

AATTTGAATTTACGATCTATTCATTCAATATTTCCCTTTTTGGAGGACAAATTATCGCATTTAAATTATG

TGTCAGATATACTAATACCTTATCCCTCCCATCTGAAAATCTTGGTTCAAATCCTTCAATGCTGGATCCA

AGATGTTCCTTCTTTACATTTATTGCGATTCTTTCTTCACGAATATCATAATTGGAATAGTCTTATTACT

CCGAATAATTCTATTTTTTTTTCAAAAGAAAATAAAAGACTATTTCGGTTCCCATATAATTCTTATGTAT

CTGAATGCGAATTTGTATTAGTTTTTCTTCGTAAACAATCTTCTTATTTACGATTAACATCTTCTGGAGC

TTTTCTTGAGCGAACACATTTCTATGGAAAAATAGAACATCTTATAGTAGTGCGCCGTAATTATTTTCAG

AAGACCCTATGGTTCTTCAAGGATCCCTTCATGCATTATGTTCGATATCAAGGAAAGGCAATTCTGGTTT

CAAAGGGGACTCATCTTCTGATGAAGAAATGGAAATGTCATCTTGTCAATTTCTGGCAATATTATTTTCA

CTTTTGGTCTCAACCGTAYAGGATCCATATAAACCAATTATCAAGCTGTTCTTTCTATTTTCTAGGTTAT

CTTTCAAGTGTACTAATAAATCCTTCGGCGGTAAGGAATCAAATGCTAGAGAATTCATTTCTAATAGATA

CTGTTATTAAAAAATTCGATACCAGAGTCCCTGTTATTCCTCTTATTGGATCATTGTCTAAAGCTAAATT

TTGTACCGTATCGGGGCATCCTATTAGTAAGCCGATCTGGACCAATTTATCAGATTGCGATATTATTGAT

CGATTTGGTCGGATATGTAGAAATCTTTCTCATTATCACAGTGGATCCTCAAAAAAACAGAGTTTGTATC

GAATAAAGTATATACTTCGATTTTCGTGTGCTAGAACTTTGGCTCGTAAACATAAAA

>Bactris_pliniana_HQ265558.1

CCTGCCTCTGGCNTCAAGATAGAAATGGAAATGGAAGAATTACAAAGATATTTAGAAAAAGATAGATCTC

GGCAACAACACTTTCTATATCCGCTTCTCTTTAAGGAGTATATTTACACATTTGCTCATGATCGTGGTTT

AAATGGTTCGATTTTTTACGAATCCACGGAAATTTTTGGTTATGACAATAAATCTAGTTCAGTACTTGTG

AAACGTTCAATTATTCGAATGTATCAACAGAATTATTTGATTTATTCGGTTAATGATTCTAACCAAAATC

GATTCGTTGGGCACAACAATTATTTTTATTTTCATTTTTATTCTCAGATGATATTGGAAGGTTTTGCAGT

CATTGTGGAAATTCCATTCTTGCTGCGATTAGTATCTTCCCTCGAAGAAAAAAAAATACCAAAATCTCAG

AATTTGAATTTACGATCTATTCATTCAATATTTCCCTTTTTGGAGGACAAATTATCGCATTTAAATTATG

TGTCAGATATACTAATACCTTATCCCTCCCATCTGAAAATCTTGGTTCAAATCCTTCAATGCTGGATCCA

AGATGTTCCTTCTTTACATTTATTGCGATTCTTTCTTCACGAATATCATAATTGGAATAGTCTTATTACT

CCGAATAATTCTATTTTTTTTTCAAAAGAAAATAAAAGACTATTTCGGTTCCCATATAATTCTTATGTAT

CTGAATGCGAATTTGTATTAGTTTTTCTTCGTAAACAATCTTCTTATTTACGATTAACATCTTCTGGAGC

TTTTCTTGAGCGAACACATTTCTATGGAAAAATAGAACATCTTATAGTAGTGCGCCGTAATTATTTTCAG

AAGACCCTATGGTTCTTCAAGGATCCCTTCATGCATTATGTTCGATATCAAGGAAAGGCAATTCTGGTTT

CAAAGGGGACTCATCTTCTGATGAAGAAATGGAAATGTCATCTTGTCAATTTCTGGCAATATTATTTTCA

CTTTTGGTCTCAACCGTATAGGATCCATATAAACCAATTATCAAGCTGTTCTTTCTATTTTCTAGGTTAT

CTTTCAAGTGTACTAATAAATCCTTCGGCGGTAAGGAATCAAATGCTAGAGAATTCATTTCTAATAGATA

CTGTTATTAAAAAATTCGATACCAGAGTCCCTGTTATTCCTCTTATTGGATCATTGTCTAAAGCTAAATT

TTGTACCGTATCGGGGCATCCTATTAGTAAGCCGATCTGGACCAATTTATCAGATTGCGATATTATTGAT

CGATTTGGTCGGATATGTAGAAATCTTTCTCATTATCACAGTGGATCCTCAAAAAAACAGAGTTTGTATC

GAATAAAGTATATACTTCGATTTTCGTGTGCTAGAACTTTGGCTCGTAAACATAAAAGTATGGTACGCGC

TTTTTTGCAAAGATTAGGTTCGGGATTATTAGAAGAATTCTTTACGGAAGAAGAACAAGTTGTTTCTTTG

ATCTTCCCAAAAACAACTTCTTTTTCTTTACATGAATCACATATAGAACGTATTTGGTATTTGGATATTA

TCCGTATCAATGACCTGGTGAATTATTCATAATGGGTTTGGTGACGTGATGAGACTTATGAATAGAATAG

AAATGATCTATAAATGATCAATAGAGAAAAAAATTCATGAATTTTCATTCTGAAATGCTCATTGCAGTAG

TGTAGTGGTTGAATCAACTGAGTAGTCAAAATTATTATACTTTCTTCTCGGGACCCAAGTTTTATATTAT

ACATAGGTAAAGTCGTGTGCAATGAAAAATGCAAGCACGGTTTGGGGAGGGATCTTTTTCCTCTATTCCA

ACAAAGAAAAGTTATCTACTCCATCCNNACTAGTTAA

>Bactris_major_HQ265556.1

AAATGAATGGGTCCTGCCTCTGGTTCAGTAGAAATGGAAATGGAAGAATTACAAGGATATTTAGAAAAAG

ATAGATCTCGGCAACAACACTTTCTATATCCGCTTCTCTTTAAGGAGTATATTTACACATTTGCTCATGA

TCGTGGTTTAAATGGTTCGATTTTTTACGAATCCACGGAAATTTTTGGTTATGACAATAAATCTAGTTCA

GTACTTGTGAAACGTTCAATTATTCGAATGTATCAACAGAATTATTTGATTTATTCGGTTAATGATTCTA

ACCAAAATCGATTTGTTGGGCACAACAATTATTTTTATTTAAATTTTTATTCTCAGATGATATTGGAAGG

TTTTGCAGTCATTGTGGAAATTCCATTCTTGCTGCGATTAGTATCTTCCCTCGAAGAAAAAAAAATACCA

AAATCTCAGAATTTGAATTTACGATCTATTCATTCAATATTTCCCTTTTTGGAGGACAAATTATCGCATT

TAAATTATGTGTCAGATATACTAATACCTTATCCCATCCATCTGAAAATCTTGGTTCAAATCCTTCAATG

CTGGATCCAAGATGTTCCTTCTTTACATTTATTGCGATTCTTTCTTCACGAATATCATAATTGGAATAGT

CTTATTACTCCGAATAATTCTATTTTTTTTTCAAAAGAAAATAAAAGACTATTTCGGTTCCCATATAATT

CTTATGTATCTGAATGCGAATTTGTATTAGTTTTTCTTCGTAAACAATCTTCTTATTTACGATTAACATC

TTCTGGAGCTTTTCTTGAGCGAACACATTTCTATGGAAAAATAGAACATCTTATAGTAGTGCGCCGTAAT

TATTTTCAGAAGACCCTATGGTTCTTCAAGGATCCCTTCATGCATTATGTTCGATATCAAGGAAAGGCAA

TTCTGGTTTCAAAGGGAACTCATCTTCTGATGAAGAAATGGAAATGTCATCTTGTCAATTTCTGGCAATA

TTATTTTCACTTTTGGTCTCAACCGTACAGGATCCATATAAACCAATTATCAAGCTGTTCTTTCTATTTT

CTAGGTTATCTTTCAAGTGTACTAATAAATCCTTCGGCGGTAAGGAATCAAATGCTAGAGAATTCATTTC

TAATAGATACTGTTATTAAAAAATTCGATACCAGAGTCCCTGTTATTCCTCTTATTGGATCATTGTCTAA

AGCGAAATTTTGTACCGTATCGGGGCATCCTATTAGTAAGCCGATCTGGACCAATTTATCAGATTGCGAT

ATTATTGATCGATTTGGTCGGATATGTAGAAATCTTTCTCATTATCACAGTGGATCCTCAAAAAAACAGA

GTTTGTATCGAATAAAGTATATACTTCGATTTTCGTGTGCTAGAACTTTGGCTCGTAAACATAAAAGTAT

GGTACGCGCTTTTTTGCAAAGATTAGGTTCGGGATTATTAGAAGAATTCTTTACGGAAGAAGAACAAGTT

GTTTCTTTGATCTTCCCAAAAACAACTTCTTTTTCTTTACATGAATCACATATAGAACGTATTTGGTATT

TGGATATTATCCGTATCAATGACCTGGTGAATTATTCATAATGGGTTTGGTGACGTGATGAGACTTATGA

ATAGAATAGAAATGATCTATAAATGATCAAGAGAGAAAAAAATTCATGAATTTTCATTCTGAAATGCTCA

TTGCAGTAGTGTAGTGGTTGAATCAACTGAGTAGTCAAAATTATTATACTTTCTTCTCGGGACCCAAGTT

TTATATTATACATAGGTAAAGTTCGTGTGCAATGAAAAATGCAAGCACGGTTTGGGGAGGGATCTTTTTC

CTCTATTCCAACAAAGAAAAGTTATCTACTCCATCCGAACTAGTTAA

>Bactris_gasipaes_HQ265554.1

GTCCTGCCTCTGGTTCAGTAGAAATGGAAATGGAAGAATTACAAGGATATTTAGAAAAAGATAGATCTCG

GCAACAACACTTTCTATATCCACTTCTCTTTAAGGAGTATATTTACACATTTGCTCATGATCGTGGTTTA

AATGGTTCGATTTTTTACGAATCCACGGAAATTTTTGGTTATGACAATAAATCTAGTTCAGTACTTGTGA

AACGTTCAATTATTCGAATGTATCGACAGAATTATTTGATTTATTCGGTTAATGATTCTAACCAAAATCG

ATTCGTTGGTCACAACAATTATTTTGATTTTCATTTTTATTCTCAGATGATATTGGAAGGTTTTGCAGTC

ATTGTGGAAATTCCATTCTTGCTGCGATTAGTATCTTCCCTCGAAGAAAAAAAAATACCAAAATCTCAGA

ATTTGAATTTACGATCTATTCATTCAATATTTCCCTTTTTGGAGGACAAATTATCGCATTTAAATTATGT

GTCAGATATACTAATACCTTATCCCATCCATCTGAAAATCTTGGTTCAAATCCTTCAATGCTGGATCCAA

GATGTTCCTTCTTTACATTTATTGCGATTCTTTCTTCACGAATATCATAATTGGAATAGTCTTATTACTC

CGAATAATTCTATTTTTTTTTCAAAAGAAAATAAAAGACTATTTCGGTTCCCATATAATTCTTATGTATC

TGAATGCGAATTTGTATTAGTTTTTCTTCGTAAACAATCTTCTTATTTACGATTAACATCTTCTGGAGCT

TTTCTTGAGCGAACACATTTCTATGGAAAAATAGAACATCTTATAGTAGTGCGCCGTAATTATTTTCAGA

AGACCCTATGGTTCTTCAAGGATCCCTTCATGCATTATGTTCGATATCAAGGAAAGGCAATTCTGGTTTC

AAAGGGGACTCATCTTCTGATGAAGAAATGGAAATGTCATCTTGTCAATTTCTGGCAATATTATTTTCAC

TTTTGGTCTCAACCGTACAGGATCCATATAAACCAATTATCAAGCTGTTCTTTCTATTTTCTAGGTTATC

TTTCAAGTGTACTAATAAATCCTTCGGCGGTAAGGAATCAAATGCTAGAGAATTCATTTCTAATAGATAC

TGTTATTAAAAAATTCGATACTAGAGTCCCTGTTATTCCTCTTATTGGATCATTGTCTAAAGCTAAATTT

TGTACCGTATCGGGGCATCCTATTAGTAAGCCGATCTGGACCAATTTATCAGATTGCGATATTATTGATC

GATTTGGTCGGATATGTAGAAATCTTTCTCATTATCACAGTGGATCCTCAAAAAAACAGAGTTTGTATCG

AATAAAGTATATACTTCGATTTTCGTGTGCTAGAACTTTGGCTCGTAAACATAAAAGTATGGTACGCGCT

TTTTTGCAAAGATTAGGTTCGGGATTATTAGAAGAATTCTTTACGGAAGAAGAACAAGTTGTTTCTTTGA

TCTTCCCAAAAACAACTTCTTTTTCTTTACATGAATCACATATAGAACGTATTTGGTATTTGGATATTAT

CCGTATCAATGACCTGGTGAATTATTCATAATGGGTTTGGTGACGTGATGAGACTTATGAATAGAATAGA

AATGATCTATAAATGATCAATAGAGAAAAAAATTCATGAATTTTCATTCTGAAATGCTCATTGCAGTAGT

GTAGTGGTTGAATCAACTGAGTAGTCAAAATTATTATACTTTCTTCTCGGGACCCAAGTTTTATATTATA

CATAGGTAAAGTCGTGTGCAATGAAAAATGCAAGCACGGTTTGGGGAGGGATCTTTTTCCTGTATTCCAA

CAAAGAAAAGTTATCTACTCCATCCNAACTAGTTAA

>Bactris_constanciae_HQ265552.1

TGAATGGGTCCTGCCTCTGGTTCAGTAGAAATGGAAATGGAAGAATTACAAGGATATTTAGAAAAAGATA

GATCTCGGCAACAACACTTTCTATATCCGCTTCTCTTTAAGGAGTATATTTACACATTTGCTCATGATCG

TGGTTTAAATGGTTCGATTTTTTACGAATCCACGGAAATTTTTGGTTATGACAATAAATCTAGTTCAGTA

CTTGTGAAACGTTCAATTATTCGAATGTATCAACAGAATTATTTGATTTATTCGGTTAATGATTCTAACC

AAAATCGATTCGTTGGGCACAACAATTATTTTTATTTTCATTTTTATTCTCAGATGATATTGGAAGGTTT

TGCAGTCATTGTGGAAATTCCATTCTTGCTGCGATTAGTATCTTCCCTCGAAGAAAAAAAAATACCAAAA

TCTCAGAATTTGAATTTACGATCTATTCATTCAATATTTCCCTTTTTGGAGGACAAATTATCGCATTTAA

ATTATGTGTCAGATATACTAATACCTTATCCCATCCATCTGAAAATCTTGGTTCAAATCCTTCAATGCTG

GATCCAAGATGTTCCTTCTTTACATTTATTGCGATTCTTTCTTCACGAATATCATAATTGGAATAGTCTT

ATTACTCCGAATAATTCTATTTTTTTTTCAAAAGAAAATAAAAGACTATTTCGGTTCCCATATAATTCTT

ATGTATCTGAATGCGAATTTGTATTAGTTTTTCTTCGTAAACAATCTTCTTATTTACGATTAACATCTTC

TGGAGCTTTTCTTGAGCGAACACATTTCTATGGAAAAATAGAACATCTTATAGTAGTGCGCCGTAATTAT

TTTCAGAAGACCCTATGGTTCTTCAAGGATCCCTTCATGCATTATGTTCGATATCAAGGAAAGGCAATTC

TGGTTTCAAAGGGGACTCATCTTCTGATGAAGAAATGGAAATGTCATCTTGTCAATTTCTGGCAATATTA

TTTTCACTTTTGGTCTCAACCGTACAGGATCCATATAAACCAATTATCAAGCTGTTCTTTCTATTTTCTA

GGTTATCTTTCAAGTGTACTAATAAATCCTTCGGCGGTAAGGAATCAAATGCTAGAGAATTCATTTCTAA

TAGATACTGTTATTAAAAAATTCGATACCAGAGTCCCCGTTATTCCTCTTATTGGATCATTGTCTAAAGC

TAAATTTTGTACCGTATCGGGGCATCCTATTAGTAAGCCGATCTGGACCAATTTATCAGATTGCGATATT

ATTGATCGATTTGGTCGGATATGTAGAAATCTTTCTCATTATCACAGTGGATCCTCAAAAAAACAGAGTT

NGTATCGAATAAAGTATATACTTCGATTTTCGTGTGCTAGAACTTTGGCTCGTAAACATAAAAGTATGGT

ACGCGCTTTTTTGCAAAGATTAGGTTCGGGATTATTAGAAGAATTCTTTACGGAAGAAGAACAAGTTGTT

TCTTTGATCTTCCCAAAAACAACTTCTTTTTCTTTACATGAATCACATATAGAACGTATTTGGTATTTGG

ATATTATCCGTATCAATGACCTGGTGAATTATTCATAATGGGTTTGGTGACGTGATGAGACTTATGAATA

GAATAGAAATGATCTATAAATGATCAATAGAGAAAAAAATTCATGAATTTTCATTCTGAAATGCTCATTG

CAGTAGTGTAGTGGTTGAATCAACTGAGTAGTCAAAATTTTTATACTTTCTTCTCGGGACCCAAGTTTTA

TATTATACATAGGTAAAGTCGTGTGCAATGAAAAATGCAAGCACGGTTTGGGGAGGGATCTTTTTCCTCT

ATTCCAACAAAGAAAAGTTATCTACTCCATCCNAACTAGTTAAA

>Bactris_bifida_HQ265550.1

TCCTGCCTCTGGTTCAGTAGAAATGGAAATGGAAGAATTACAAGGATATTTAGAAAAAGATAGATCTCGG

CAACAACACTTTCTATATCCGCTTCTCTTTAAGGAGTATATTTACACATTTGCTCATGATCGTGGTTTAA

ATGGTTCGATTTTTTACGAATCCACGGAAATTTTTGGTTATGACAATAAATCTAGTTCAGTACTTGTGAA

ACGTTCAATTATTCGAATGTATCAACAGAATTATTTGATTTATTCGGTTAATGATTCTAACCAAAATCGA

TTTGTTGGGCACAACAATTATTTTTATTTAAATTTTTATTCTCAGATGATATTGGAAGGTTTTGCAGTCA

TTGTGGAAATTCCATTCTTGCTGCGATTAGTATCTTCCCTCGAAGAAAAAAAAATACCAAAATCTCAGAA

TTTGAATTTACGATCTATTCATTCAATATTTCCCTTTTTGGAGGACAAATTATCGCATTTAAATTATGTG

TCAGATATACTAATACCTTATCCCATCCATCTGAAAATCTTGGTTCAAATCCTTCAATGCTGGATCCAAG

ATGTTCCTTCTTTACATTTATTGCGATTCTTTCTTCACGAATATCATAATTGGAATAGTCTTATTACTCC

GAATAATTCTATTTTTTTTTCAAAAGAAAATAAAAGACTATTTCGGTTCCCATATAATTCTTATGTATCT

GAATGCGAATTTGTATTAGTTTTTCTTCGTAAACAATCTTCTTATTTACGATTAACATCTTCTGGAGCTT

TTCTTGAGCGAACACATTTCTATGGAAAAATAGAACATCTTATAGTAGTGCGCCGTAATTATTTTCAGAA

GACCCTATGGTTCTTCAAGGATCCCTTCATGCATTATGTTCGATATCAAGGAAAGGCAATTCTGGTTTCA

AAGGGAACTCATCTTCTGATGAAGAAATGGAAATGTCATCTTGTCAATTTCTGGCAATATTATTTTCACT

TTTGGTCTCAMCCGTACAGGATCCATATAAACCAATTATCAAGCTGTTCTTTCTATTTTCTAGGTTATCT

TTCAAGTGTACTAATAAATCCTTCGGCGGTAAGGAATCAAATGCTAGAGAATTCATTTCTAATAGATACT

GTTATTAAAAAATTCGATACCAGAGTCCCTGTTATTCCTCTTATTGGATCATTGTCTAAAGCGAAATTTT

GTACCGTATCGGGGCATCCTATTAGTAAGCCGATCTGGACCAATTTATCAGATTGCGATATTATTGATCG

ATTTGGTCGGATATGTAGAAATCTTTCTCATTATCACAGTGGATCCTCAAAAAAACAGAGTTTGTATCGA

ATAAAGTATATACTTCGATTTTCGTGTGCTAGAACTTTGGCTCGTAAACATAAAAGTATGGTACGCGCTT

TTTTGCAAAGATTAGGTTCGGGATTATTAGAAGAATTCTTTACGGAAGAAGAACAAGTTGTTTCTTTGAT

CTTCCCAAAAACAACTTCTTTTTCTTTACATGAATCACATATAGAACGTATTTGGTATTTGGATATTATC

CGTATCAATGACCTGGTGAATTATTCATAATGGGTTTGGTGACGTGATGAGACTTATGAATAGAATAGAA

ATGATCTATAAATGATCAAGAGAGAAAAAAATTCATGAATTTTCATTCTGAAATGCTCATTGCAGTAGTG

TAGTGGTTGAATCAACTGAGTAGTCAAAATTATTATACTTTCTTCTCGGGACCCAAGTTTTATATTATAC

ATAGGTAAAGTCGTGTGCAATGAAAAATGCAAGCACGG

>Bactris_major_GQ981945.1

TCCTTCTTTACATTTATTGCGATTCTTTCTTCACGAATATCATAATTGGAATAGTCTTATTACTCCGAAT

AATTCTATTTTTTTTTCAAAAGAAAATAAAAGACTATTTCGGTTCCCATATAATTCTTATGTATCTGAAT

GCGAATTTGTATTAGTTTTTCTTCGTAAACAATCTTCTTATTTACGATTAACATCTTCTGGAGCTTTTCT

TGAGCGAACACATTTCTATGGAAAAATAGAACATCTTATAGTAGTGCGCCGTAATTATTTTCAGAAGACC

CTATGGTTCTTCAAGGATCCCTTCATGCATTATGTTCGATATCAAGGAAAGGCAATTCTGGTTTCAAAGG

GAACTCATCTTCTGATGAAGAAATGGAAATGTCATCTTGTCAATTTCTGGCAATATTATTTTCACTTTTG

GTCTCAACCGTACAGGATCCATATAAACCAATTATCAAGCTGTTCTTTCTATTTTCTAGGTTATCTTTCA

AGTGTACTAATAAATCCTTCGGCGGTAAGGAATCAAATGCTAGAGAATTCATTTCTAATAGATACTGTTA

TTAAAAAATTCGATACCAGAGTCCCTGTTATTCCTCTTATTGGATCATTGTCTAAAGCGAAATTTTGTAC

CGTATCGGGGCATCCTATTAGTAAGCCGATCTGGACCAATTTATCAGATTGCGATATTATTGATCGATTT

GGTCGGATATGTAGAAATCTTTCTCATTATCACAGTGGATCCTCAAAAAAACAGAGTTTGTATCGAATAA

AGT

>Bactris_barronis_GQ981944.1

GTGTCAGATATACTAATACCTTATCCCATCCATCTGAAAATCTTGGTTCAAATCCTTCAATGCTGGATCC

AAGATGTTCCTTCTTTACATTTATTGCGATTCTTTCTTCACGAATATCATAATTGGAATAGTCTTATTAC

TCCGAATAATTCTATTTTTTTTTCAAAAGAAAATAAAAGACTATTTCGGTTCCCATATAATTCTTATGTA

TCTGAATGCGAATTTGTATTAGTTTTTCTTCGTAAACAATCTTCTTATTTACGATTAACATCTTCTGGAG

CTTTTCTTGAGCGAACACATTTCTATGGAAAAATAGAACATCTTATAGTAGTGCGCCGTAATTATTTTCA

GAAGACCCTATGGTTCTTCAAGGATCCCTTCATGCATTATGTTCGATATCAAGGAAAGGCAATTCTGGTT

TCAAAGGGGACTCATCTTCTGATGAAGAAATGGAAATGTCATCTTGTCAATTTCTGGCAATATTATTTTC

ACTTTTGGTCTCAACCGTATAGGATCCATATAAACCAATTATCAAGCTGTTCTTTCTATTTTCTAGGTTA

TCTTTCAAGTGTACTAATAAATCCTTCGGCGGTAAGGAATCAAATGCTAGAGAATTCATTTCTAATAGAT

ACTGTTATTAAAAAATTCGATACCAGAGTCCCTGTTATTCCTCTTATTGGATCATTGTCTAAAGCTAAAT

TTTGTACCGTATCGGGGCATCCTATTAGTAAGCCGATCTGGACCAATTTATCAGATTGCGATATTATTGA

TCGATTTGGTCGGATATGTAGAAATCTTTCTCATTATCAC

>Bactris_gasipaes_AM114642.1

GACCATATTGCACTATGTATCATTTGATAACCCCAAAAATGAAATGGGTCCTGCCTCTGGTTCAAGTAGA

AATGGAAATGGAAGAATTACAAGGATATTTAGAAAAAGATAGATCTCGGCAACAACACTTTCTATATCCG

CTTCTCTTTAAGGAGTATATTTACACATTTGCTCATGATCGTGGTTTAAATGGTTCGATTTTTTACGAAT

CCACGGAAATTTTTGGTTATGACAATAAATCTAGTTCAGTACTTGTGAAACGTTCAATTATTCGAATGTA

TCAACAGAATTATTTGATTTATTCGGTTAATGATTCTAACCAAAATCGATTCGTTGGGCACAACAATTAT

TTTGATTTTCATTTTTATTCTCAGATGATATTGGAAGGTTTTGCAGTCATTGTGGAAATTCCATTCTTGC

TGCGATTAGTATCTTCCCTCGAAGAAAAAAAAATACCAAAATCTCAGAATTTGAATTTACGATCTATTCA

TTCAATATTTCCCTTTTTGGAGGACAAATTATCGCATTTAAATTATGTGTCAGATATACTAATACCTTAT

CCCATCCATCTGAAAATCTTGGTTCAAATCCTTCAATGCTGGATCCAAGATGTTCCTTCTTTACATTTAT

TGCGATTCTTTCTTCACGAATATCATAATTGGAATAGTCTTATTACTCCGAATAATTCTATTTTTTTTTT

CAAAAGAAAATAAAAGACTATTTCGGTTCCCATATAATTCTTATGTATCTGAATGCGAATTTGTATTAGT

TTTTCTTCGTAAACAATCTTCTTATTTACGATTAACATCTTCTGGAGCTTTTCTTGAGCGAACACATTTC

TATGGAAAAATAGAACATCTTATAGTAGTGCGCCGTAATTATTTTCAGAAGACCCTATGGTTCTTCAAGG

ATCCCTTCATGCATTATGTTCGATATCAAGGAAAGGCAATTCTGGTTTCAAAGGGGACTCATCTTCTGAT

GAAGAAATGGAAATGTCATCTTGTCAATTTCTGGCAATATTATTTTCACTTTTGGTCTCAACCGTACAGG

ATCCATATAAACCAATTATCAAGCTGTTCTTTCTATTTTCTAGGTTATCTTTCAAGTGTACTAATAAATC

CTTCGGCGGTAAGGAATCAAATGCTAGAGAATTCATTTCTAATAGATACTGTTATTAAAAAATTCGATAC

CAGAGTCCCTGTTATTCCTCTTATTGGATCATTGTCTAAAGCTAAATTTTGTACCGTATCGGGGCATCCT

ATTAGTAAGCCGATCTGGACCAATTTATCAGATTGCGATATTATTGATCGATTTGGTCGGATATGTAGAA

ATCTTTCTCATTATCACAGTGGATCCTCAAAAAAACAGAGTTTGTATCGAATAAAGTATATACTTCGATT

TTCGTGTGCTAGAACTTTGGCTCGTAAACATAAAAGTATGGTACGCGCTTTTTTGCAAAGATTAGGTTCG

GGATTATTAGAAGAATTCTTTACGGAAGAAGAACAAGTTGTTTCTTTGATCTTCCCAAAAACAACTTCTT

TTTCTTTACATGAATCACATATAGAACGTATTTGGTATTTGGATATTATCCGTATCAATGACCTGGTGAA

TTATTCATAATGGGTTTGGTGACGTGATGAGACTTATGAATAGAATAGAAATGATCTATAAATGATCAAT

AGAGAAAAAAATTCATGAATTTTCATTCTGAAATGCTCATTGCAGTAGTGTAGTGGTTGAATCAACTGAG

TAGTCAAAATTATTATACTTTCTTCTCGGGACCCAAGTTTTATATTATACATAGGTAAAGTC

>Barcella_odora_EU004874.1

TTGCACTATGTATCATTTGATAACCCAAAAAATGAAATGGGTCCTGCCTCTGGTTCAAGTAGAAATGGAA

ATGGAAGAATTACAAGGATATTTAGAAAAAGATAGATCTCGGCAACAACACTTTCTATATCCGCTTCTCT

TTAAGGAGTATATTTACACATTTGCTCATGATCGTGGTTTAAATGGTTCGATTTTTTACGAATCCACGGA

AATTTTTGGTTATGACAATAAATCTAGTTCAGTACTTGTGAAACGTTCAATTATTCGAATGTATCAACAG

AATTATTTGATTTATTCGGTTAATGATTCTAACCAAAATCGATTCGTTGGGCACAACAATTATTTTGATT

TTCATTTTTATTATCAGATGCTATTGGAAGGTTTTGCAGTCATTGTGGAAATTCCAATCTTGCTGCGATT

AGTATCTTCCCTCGAAGAAAAAAAAATACCAAAATCTCAGAATTTGAATTTACGATCTATTCATTCAATA

TTTCCCTTTTTGGAGGACAAATTATCGCATTTAAATTATGTGTCAGATATACTAATACCTTATCCCATCC

ATCTGAAAATCTTGGTTCAAATCCTTCAATGCTGGATCCAAGATGTTCCTTCTTTACATTTATTGCGATT

CTTTCTTCACGAATATCATAATTGGAATAGTCTTATTACTCCGAATAATTCTATTTTTTTTTCAAAAGAA

AATAAAAGACTATTTCGGTTCCCATATAATTCTTATGTATCTGAATGCGAATTTGTATTAGTTTTTCTTC

GTAAACAATCTTCTTATTTACGATTAACATCTTCTGGAGCCTTTCTTGAGCGAACACATTTCTATGGAAA

AATAGAACATCTTATAGTAGTGCGCCGTAATTATTTTCAGAAGACCCTATGGTTCTTCAAGGATCCCTTC

ATGCATTATGTTCGATATCAAGGAAAAGCAATTCTGGTTTCAAAGGGGACTCATCTTCTGATGAAGAAAT

GGAAATGTCATCTTGTCAATTTCTGGCAATATTATTTTCACTTTTGGTCTCAACCGTACAGGATCCATAT

AAACCAATTATCAAGCTGTTCTTTCTATTTTCTAGGTTATCTTTCAAGTGTACTAATAAATCCTTCGGCG

GTAAGGAATCAAATGCTAGAGAATTCATTTCTAATAGATACTGTTATTAAAAAATTCGATACCAGAGTCC

CTGTTATTCCTCTTATTGGATCATTGTCTAAAGCTAAATTTTGTACCGTATCGGGGCATCCTATTAGTAA

GCCGATCTGGACCAATTTATCAGATTGCGATATTATTGATCGATTTGGTCGGATATGTAGAAATCTTTCT

CATTATCACAGTGGATCCTCAAAAAAACAGAGTTTGTATCGAATAAAGTATATACTTCGATTTTCGTGTG

CTAGAACTTTGGCTCGTAAACATAAAAGTATGGTACGCGCTTTTTTGCAAAGATTAGGTTCGGGATTATT

AGAAGAATTCTTTACGGAAGAAGAACAAGTTGTTTCTTTGATCTTCCCAAAAACAACTTCTTTTTCTTTA

CATGAATCACATATAGAACGTATTTGGTATTTGGATATTATCCGTATCAATGACCTGGTGAATTATTCAT

AATGGGTTTGGTGACGTGATGAGACTTATGAATAGAATAGAAATGATCTATAAATGATCAAGAGAGAAAA

AAATTCATGAATTTTCATTCTGAAATGCTCATTGCAGTAATGTAGTGGTTGAATCAACTGAGTAGTCAAA

ATTATTATCTTCTCGGGACCC

>Barcella_odora_HQ265562.1

GGGTCCTGCCTCTGGTTCAGTAGAAATGGAAATGGAAGAATTACAAGGATATTTAGAAAAAGATAGATCT

CGGCAACAACACTTTCTATATCCGCTTCTCTTTAAGGAGTATATTTACACATTTGCTCATGATCGTGGTT

TAAATGGTTCGATTTTTTACGAATCCACGGAAATTTTTGGTTATGACAATAAATCTAGTTCAGTACTTGT

GAAACGTTCAATTATTCGAATGTATCAACAGAATTATTTGATTTATTCGGTTAATGATTCTAACCAAAAT

CGATTCGTTGGGCACAACAATTATTTTGATTTTCATTTTTATTATCAGATGCTATTGGAAGGTTTTGCAG

TCATTGTGGAAATTCCAATCTTGCTGCGATTAGTATCTTCCCTCGAAGAAAAAAAAATACCAAAATCTCA

GAATTTGAATTTACGATCTATTCATTCAATATTTCCCTTTTTGGAGGACAAATTATCGCATTTAAATTAT

GTGTCAGATATACTAATACCTTATCCCATCCATCTGAAAATCTTGGTTCAAATCCTTCAATGCTGGATCC

AAGATGTTCCTTCTTTACATTTATTGCGATTCTTTCTTCACGAATATCATAATTGGAATAGTCTTATTAC

TCCGAATAATTCTATTTTTTTTTCAAAAGAAAATAAAAGACTATTTCGGTTCCCATATAATTCTTATGTA

TCTGAATGCGAATTTGTATTAGTTTTTCTTCGTAAACAATCTTCTTATTTACGATTAACATCTTCTGGAG

CCTTTCTTGAGCGAACACATTTCTATGGAAAAATAGAACATCTTATAGTAGTGCGCCGTAATTATTTTCA

GAAGACCCTATGGTTCTTCAAGGATCCCTTCATGCATTATGTTCGATATCAAGGAAAAGCAATTCTGGTT

TCAAAGGGGACTCATCTTCTGATGAAGAAATGGAAATGTCATCTTGTCAATTTCTGGCAATATTATTTTC

ACTTTTGGTCTCAACCGTACAGGATCCATATAAACCAATTATCAAGCTGTTCTTTCTATTTTCTAGGTTA

TCTTTCAAGTGTACTAATAAATCCTTCGGCGGTAAGGAATCAAATGCTAGAGAATTCATTTCTAATAGAT

ACTGTTATTAAAAAATTCGATACCAGAGTCCCTGTTATTCCTCTTATTGGATCATTGTCTAAAGCTAAAT

TTTGTACCGTATCGGGGCATCCTATTAGTAAGCCGATCTGGACCAATTTATCAGATTGCGATATTATTGA

TCGATTTGGTCGGATATGTAGAAATCTTTCTCATTATCACAGTGGATCCTCAAAAAAACAGAGTTTGTAT

CGAATAAAGTATATACTTCGATTTTCGTGTGCTAGAACTTTGGCTCGTAAACATAAAAGTATGGTACGCG

CTTTTTTGCAAAGATTAGGTTCGGGATTATTAGAAGAATTCTTTACGGAAGAAGAACAAGTTGTTTCTTT

GATCTTCCCAAAAACAACTTCTTTTTCTTTACATGAATCACATATAGAACGTATTTGGTATTTGGATATT

ATCCGTATCAATGACCTGGTGAATTATTCATAATGGGTTTGGTGACGTGATGAGACTTATGAATAGAATA

GAAATGATCTATAAATGATCAAGAGAGAAAAAAATTCATGAATTTTCATTCTGAAATGCTCATTGCAGTA

ATGTAGTGGTTGAATCAACTGAGTAGTCAAAATTATTATCTTCTCGGGACCCAAGTTTTATATTATACAT

AGGTAAAGTCGTGTGCAATGAAAAATGCAAGCACGGTTTGGGGAGGGATCTTTTCCTCTATTCCAACAAA

GAAAAGTTATCTACTCCACNNAACTAGTTAA

>Brahea_dulcis_HQ720250.1

AGAAATGTAAATGGAAGAATTACAAGGATATTTAGAAAGAGATAGATCTCTGCAACAACACTTTCTATAT

CCGCTTCTCTTTAAGGAGTATATTTACACATTTCTTCATGATCGTGGTTTAAATGGTTCGATTTTTTACG

AATCCACGGAAATTTTTGGTTATGACAATAAATCTAGTTCAGTACTTGTGAAACGTTCAATTATTCGAAT

GTATCAACAGAATTATTTGATTTATTCGGTTAATGATTCTAACCAAAATCGATTCGTTGGGCACAACAAT

TATTTTTATTTTCATTTTTATTCTCAGATGATATTGGAAGGTTTTGCAGTCATTGTGGAAATTCCATTCT

TGCTGCGATTGGTATCTTCCCTCGAAGAAAAAAAAATACCAAAATCTCAGAATTTGAATTTACGATCTAT

TCATTCAATATTTCCCTTTTTGGAGGACAAATTATCGCATTTAAATTATGTGTCAGATATACTAATACCT

TATCCCATCCATCTGAAAATCTTGGTTCAAATCCTTCAATTCTGGATCCAAGATGTTCCTTCTTTACATT

TATTGCGATTCTTTCTTCACGAATATCATAATTGGAATAGTCTTATTACTCCGAATAATTCTATTTTTCT

TTTTTCAAAAGAAAATAAAAGACTATTTCGGTTCCCATATAATTCTTATGTATCTGAATGCGAATTTGTA

TTAGTTTTTCTTCGTAAACAATCTTCTTATTTACGATTAACATCTTCTGGAGCTTTTCTTGAGCGAACAC

ATTTCTATGGAAAAATAGAATATCGTATAGTAGTGCGCCGTAATTATTTTCAGAAGACCCTATGGTTTTT

CAAGGATCCCCTCATGCATTATGTTCGATATCAAGGAAAAGCAATTCTGGTTTCAAAGGGGACTCATCTT

CTGATGAAGAAATGGAAATGTCACCTTGTCAATTTCTGGCAATATTATTTTCACTTTTGGTCTCAACCGT

ACAGGATTCATATAAACCAATTATCAAACTGTTCTTTCTATTTTCTAGGTTATCTTTCAAGTGTACTAAT

AAATCCTTCGGCGGTAAGGAATCAAATGCTAGAGAATTCATTTCTAATAGATACTGTTATTAAAAAATTC

GATACCGGAGTCCCAGTTATTCCTCTTATTAGATCATTGTCTAAAGCTAAATTTTGTACCGTATCGGGGC

ATCCTATTAGTAAGCCGATCTGGACCGATTTATCAGATTGCGATATTATTGATCGATTTGGTCGGATATG

TAGAAATCTTTCTCATTATCACAGTGGATCCTCAAAAAAACAGAGTTTGTATCGAATAAAGTATATACTT

CGATTTTCGTGTGCTAGAACTTTGGCTCGTAAACATAAAAGTATGGTACGCGCTTTTTTGCAAAGATTAG

GTTCGGGATTATTAGAAGAATTTTTTATGGAAGAAGAACAAGTTGTTTCTTTGATCTTCCCAAAAACAAC

TTCTTTTTCTTTACATGAATCACATATAGAACGTATTTGGTATTTGGATATTATCCGTATCAATGACCTG

GTGAATTATTCATAATTGGTTTGTTGACGTGATGAGACTTATGAATAGAATAGAAATGATCTATAAATGA

TCAAGAGAGAAAAAAATTCATGAATTTTCATTCTGAAATGCTCATTGCAGTAGTGTAGTGGTTGAATCAA

CTGAGTAGTCAAAATT

>Brahea_dulcis_HQ720248.1

GGAAGAATTACAAGGATATTTAGAAAGAGATAGATCTCTGCAACAACACTTTCTATATCCGCTTCTCTTT

AAGGAGTATATTTACACATTTCTTCATGATCGTGGTTTAAATGGTTCGATTTTTTACGAATCCACGGAAA

TTTTTGGTTATGACAATAAATCTAGTTCAGTACTTGTGAAACGTTCAATTATTCGAATGTATCAACAGAA

TTATTTGATTTATTCGGTTAATGATTCTAACCAAAATCGATTCGTTGGGCACAACAATTATTTTTATTTT

CATTTTTATTCTCAGATGATATTGGAAGGTTTTGCAGTCATTGTGGAAATTCCATTCTTGCTGCGATTGG

TATCTTCCCTCGAAGAAAAAAAAATACCAAAATCTCAGAATTTGAATTTACGATCTATTCATTCAATATT

TCCCTTTTTGGAGGACAAATTATCGCATTTAAATTATGTGTCAGATATACTAATACCTTATCCCATCCAT

CTGAAAATCTTGGTTCAAATCCTTCAATTCTGGATCCAAGATGTTCCTTCTTTACATTTATTGCGATTCT

TTCTTCACGAATATCATAATTGGAATAGTCTTATTACTCCGAATAATTCTATTTTTCTTTTTTCAAAAGA

AAATAAAAGACTATTTCGGTTCCCATATAATTCTTATGTATCTGAATGCGAATTTGTATTAGTTTTTCTT

CGTAAACAATCTTCTTATTTACGATTAACATCTTCTGGAGCTTTTCTTGAGCGAACACATTTCTATGGAA

AAATAGAATATCGTATAGTAGTGCGCCGTAATTATTTTCAGAAGACCCTATGGTTTTTCAAGGATCCCCT

CATGCATTATGTTCGATATCAAGGAAAAGCAATTCTGGTTTCAAAGGGGACTCATCTTCTGATGAAGAAA

TGGAAATGTCACCTTGTCAATTTCTGGCAATATTATTTTCACTTTTGGTCTCAACCGTACAGGATTCATA

TAAACCAATTATCAAACTGTTCTTTCTATTTTCTAGGTTATCTTTCAAGTGTACTAATAAATCCTTCGGC

GGTAAGGAATCAAATGCTAGAGAATTCATTTCTAATAGATACTGTTATTAAAAAATTCGATACCGGAGTC

CCAGTTATTCCTCTTATTAGATCATTGTCTAAAGCTAAATTTTGTACCGTATCGGGGCATCCTATTAGTA

AGCCGATCTGGACCGATTTATCAGATTGCGATATTATTGATCGATTTGGTCGGATATGTAGAAATCTTTC

TCATTATCACAGTGGATCCTCAAAAAAACAGAGTTTGTATCGAATAAAGTATATACTTCGATTTTCGTGT

GCTAGAACTTTGGCTCGTAAACATAAAAGTATGGTACGCGCTTTTTTGCAAAGATTAGGTTCGGGATTAT

TAGAAGAATTTTTTATGGAAGAAGAACAAGTTGTTTCTTTGATCTTCCCAAAAACAACTTCTTTTTCTTT

ACATGAATCACATATAGAACGTATTTGGTATTTGGATATTATCCGTATCAATGACCTGGTGAATTATTCA

TAATTGGTTTGTTGACGTGATGAGACTTATGAATAGAATAGAAATGATCTATAAATGATCAAGAGAGAAA

AAAATTCATGAATTTTCATTCTGAAATGCTCATTGCAGTAGTGTAGTGGTTGAATCAACTGAGTAGTCAA

A

>Brahea_armata_HQ720246.1

CCGCCTCTGGTTCAAGTAGAAATGTAAATGGAAGAATTACAAGGATATTTAGAAAGAGATAGATCTCTGC

AACAACACTTTCTATATCCGCTTCTCTTTAAGGAGTATATTTACACATTTCTTCATGATCGTGGTTTAAA

TGGTTCGATTTTTTACGAATCCACGGAAATTTTTGGTTATGACAATAAATCTAGTTCAGTACTTGTGAAA

CGTTCAATTATTCGAATGTATCAACAGAATTATTTGATTTATTCGGTTAATGATTCTAACCAAAATCGAT

TCGTTGGGCACAACAATTATTTTTATTTTCATTTTTATTCTCAGATGATATTGGAAGGTTTTGCAGTCAT

TGTGGAAATTCCATTCTTGCTGCGATTGGTATCTTCCCTCGAAGAAAAAAAAATACCAAAATCTCAGAAT

TTGAATTTACGATCTATTCATTCAATATTTCCCTTTTTGGAGGACAAATTATCGCATTTAAATTATGTGT

CAGATATACTAATACCTTATCCCATCCATCTGAAAATCTTGGTTCAAATCCTTCAATTCTGGATCCAAGA

TGTTCCTTCTTTACATTTATTGCGATTCTTTCTTCACGAATATCATAATTGGAATAGTCTTATTACTCCG

AATAATTCTATTTTTCTTTTTTCAAAAGAAAATAAAAGACTATTTCGGTTCCCATATAATTCTTATGTAT

CTGAATGCGAATTTGTATTAGTTTTTCTTCGTAAACAATCTTCTTATTTACGATTAACATCTTCTGGAGC

TTTTCTTGAGCGAACACATTTCTATGGAAAAATAGAATATCGTATAGTAGTGCGCCGTAATTATTTTCAG

AAGACCCTATGGTTTTTCAAGGATCCCTTCATGCATTATGTTCGATATCAAGGAAAAGCAATTCTGGTTT

CAAAGGGGACTCATCTTCTGATGAAGAAATGGAAATGTCACCTTGTCAATTTCTGGCAATATTATTTTCA

CTTTTGGTCTCAACCGTACAGGATTCATATAAACCAATTATCAAACTGTTCTTTCTATTTTCTAGGTTAT

CTTTCAAGTGTACTAATAAATCCTTCGGCGGTAAGGAATCAAATGCTAGAGAATTCATTTCTAATAGATA

CTGTTATTAAAAAATTCGATACCAGAGTCCCAGTTATTCCTCTTATTAGATCATTGTCTAAAGCTAAATT

TTGTACCGTATCGGGGCATTCTATTAGTAAGCCGATCTGGACCGATTTATCAGATTGCGATATTATTGAT

CGATTTGGTCGGATATGTAGAAATCTTTCTCATTATCACAGTGGATCCTCAAAAAAACAGAGTTTGTATC

GAATAAAGTATATACTTCGATTTTCGTGTGCTAGAACTTTGGCTCGTAAACATAAAAGAATGGTACGCGC

TTTTTTGCAAAGATTAGGTTCGGGATTATTAGAAGAATTTTTTATGGAAGAAGAACAAGTTGTTTCTTTG

ATCTTCCCAAAAACAACTTCTTTTTCTTTACATGAATCACATATAGAACGTATTTGGTATTTGGATATTA

TCCGTATCAATGACCTGGTGAATTATTCATAATTGGTTTGTTGACGTGATGAGACTTATGAATAGAATAG

AAATGATCTATAAATGATCAAGAGAGAAAAAAATTCATGAATTTTCATTCTGAAATGCTCATTGCAGTAG

TGTAGTGGTTGAATCAACTGAGTAGTCAAA

>Brahea_dulcis_HQ720249.1

GAAATGTAAATGGAAGAATTACAAGGATATTTAGAAAGAGATAGATCTCTGCAACAACACTTTCTATATC

CGCTTCTCTTTAAGGAGTATATTTACACATTTCTTCATGATCGTGGTTTAAATGGTTCGATTTTTTACGA

ATCCACGGAAATTTTTGGTTATGACAATAAATCTAGTTCAGTACTTGTGAAACGTTCAATTATTCGAATG

TATCAACAGAATTATTTGATTTATTCGGTTAATGATTCTAACCAAAATCGATTCGTTGGGCACAACAATT

ATTTTTATTTTCATTTTTATTCTCAGATGATATTGGAAGGTTTTGCAGTCATTGTGGAAATTCCATTCTT

GCTGCGATTGGTATCTTCCCTCGAAGAAAAAAAAATACCAAAATCTCAGAATTTGAATTTACGATCTATT

CATTCAATATTTCCCTTTTTGGAGGACAAATTATCGCATTTAAATTATGTGTCAGATATACTAATACCTT

ATCCCATCCATCTGAAAATCTTGGTTCAAATCCTTCAATTCTGGATCCAAGATGTTCCTTCTTTACATTT

ATTGCGATTCTTTCTTCACGAATATCATAATTGGAATAGTCTTATTACTCCGAATAATTCTATTTTTCTT

TTTTCAAAAGAAAATAAAAGACTATTTCGGTTCCCATATAATTCTTATGTATCTGAATGCGAATTTGTAT

TAGTTTTTCTTCGTAAACAATCTTCTTATTTACGATTAACATCTTCTGGAGCTTTTCTTGAGCGAACACA

TTTCTATGGAAAAATAGAATATCGTATAGTAGTGCGCCGTAATTATTTTCAGAAGACCCTATGGTTTTTC

AAGGATCCCCTCATGCATTATGTTCGATATCAAGGAAAAGCAATTCTGGTTTCAAAGGGGACTCATCTTC

TGATGAAGAAATGGAAATGTCACCTTGTCAATTTCTGGCAATATTATTTTCACTTTTGGTCTCAACCGTA

CAGGATTCATATAAACCAATTATCAAACTGTTCTTTCTATTTTCTAGGTTATCTTTCAAGTGTACTAATA

AATCCTTCGGCGGTAAGGAATCAAATGCTAGAGAATTCATTTCTAATAGATACTGTTATTAAAAAATTCG

ATACCGGAGTCCCAGTTATTCCTCTTATTAGATCATTGTCTAAAGCTAAATTTTGTACCGTATCGGGGCA

TCCTATTAGTAAGCCGATCTGGACCGATTTATCAGATTGCGATATTATTGATCGATTTGGTCGGATATGT

AGAAATCTTTCTCATTATCACAGTGGATCCTCAAAAAAACAGAGTTTGTATCGAATAAAGTATATACTTC

GATTTTCGTGTGCTAGAACTTTGGCTCGTAAACATAAAAGTATGGTACGCGCTTTTTTGCAAAGATTAGG

TTCGGGATTATTAGAAGAATTTTTTATGGAAGAAGAACAAGTTGTTTCTTTGATCTTCCCAAAAACAACT

TCTTTTTCTTTACATGAATCACATATAGAACGTATTTGGTATTTGGATATTATCCGTATCAATGACCTGG

TGAATTATTCATAATTGGTTTGTTGACGTGATGAGACTTATGAATAGAATAGAAATGATCTATAAATGAT

CAAGAGAGAAAAAAATTCATGAATTTTCATTCTGAAATGCTCATTGCAGTAGTGTAGTGGTTGAATCAAC

TGAGTAGTCAAAA

>Brahea_brandegeei_HQ720247.1

AAATGTAAATGGAAGAATTACAAGGATATTTAGAAAGAGATAGATCTCTGCAACAACACTTTCTATATCC

GCTTCTCTTTAAGGAGTATATTTACACATTTCTTCATGATCGTGGTTTAAATGGTTCGATTTTTTACGAA

TCCACGGAAATTTTTGGTTATGACAATAAATCTAGTTCAGTACTTGTGAAACGTTCAATTATTCGAATGT

ATCAACAGAATTATTTGATTTATTCGGTTAATGATTCTAACCAAAATCGATTCGTTGGGCACAACAATTA

TTTTTATTTTCATTTTTATTCTCAGATGATATTGGAAGGTTTTGCAGTCATTGTGGAAATTCCATTCTTG

CTGCGATTGGTATCTTCCCTCGAAGAAAAAAAAATACCAAAATCTCAGAATTTGAATTTACGATCTATTC

ATTCAATATTTCCCTTTTTGGAGGACAAATTATCGCATTTAAATTATGTGTCAGATATACTAATACCTTA

TCCCATCCATCTGAAAATCTTGGTTCAAATCCTTCAATTCTGGATCCAAGATGTTCCTTCTTTACATTTA

TTGCGATTCTTTCTTCACGAATATCATAATTGGAATAGTCTTATTACTCCGAATAATTCTATTTTTCTTT

TTTCAAAAGAAAATAAAAGACTATTTCGGTTCCCATATAATTCTTATGTATCTGAATGCGAATTTGTATT

AGTTTTTCTTCGTAAACAATCTTCTTATTTACGATTAACATCTTCTGGAGCTTTTCTTGAGCGAACACAT

TTCTATGGAAAAATAGAATATCGTATAGTAGTGCGTCGTAATTATTTTCAGAAGACCCTATGGTTTTTCA

AGGATCCCTTCATGCATTATGTTCGATATCAAGGAAAAGCAATTCTGGTTTCAAAGGGGACTCATCTTCT

GATGAAGAAATGGAAATGTCACCTTGTCAATTTCTGGCAATATTATTTTCACTTTTGGTCTCAACCGTAC

AGGATTCATATAAACCAATTATCAAACTGTTCTTTCTATTTTCTAGGTTATCTTTCAAGTGTACTAATAA

ATCCTTCGGCGGTAAGGAATCAAATGCTAGAGAATTCATTTCTAATAGGTACTGTTATTAAAAAATTCGA

TACCAGAGTCCCAGTTATTCCTCTTATTAGATCATTGTCTAAAGCTAAATTTTGTACCGTATCGGGGCAT

TCTATTAGTAAGCCGATCTGGACCGATTTATCAGATTGCGATATTATTGATCGATTTGGTCGGATATGTA

GAAATCTTTCTCATTATCACAGTGGATCCTCAAAAAAACAGAGTTTGTATCGAATAAAGTATATACTTCG

ATTTTCGTGTGCTAGAACTTTGGCTCGTAAACATAAAAGAATGGTACGCGCTTTTTTGCAAAGATTAGGT

TCGGGATTATTAGAAGAATTTTTTATGGAAGAAGAACAAGTTGTTTCTTTGATCTTCCCAAAAACAACTT

CTTTTTCTTTACATGAATCACATATAGAACGTATTTGGTATTTGGATATTATCCGTATCAATGACCTGGT

GAATTATTCATAATTGGTTTGTTGACGTGATGAGACTTATGAATAGAATAGAAATGATCTATAAATGATC

AAGAGAGAAAAAAATTCATGAATTTTCATTCTGAAATGCTCATTGCAGTAGTGTAGTGGTTGAATCAACT

GAGTAGTCAAAA

>Brahea aculeata_HQ720245.1

AAATGTAAATGGAAGAATTACAAGGATATTTAGAAAGAGATAGATCTCTGCAACAACACTTTCTATATCC

GCTTCTCTTTAAGGAGTATATTTACACATTTCTTCATGATCGTGGTTTAAATGGTTCGATTTTTTACGAA

TCCACGGAAATTTTTGGTTATGACAATAAATCTAGTTCAGTACTTGTGAAACGTTCAATTATTCGAATGT

ATCAACAGAATTATTTGATTTATATTTATTCGGTTAATGATTCTAACCAAAATCGATTCGTTGGGCACAA

CAATTATTTTTATTTTCATTTTTATTCTCAGATGATATTGGAAGGTTTTGCAGTCATTGTGGAAATTCCA

TTCTTGCTGCGATTGGTATCTTCCCTCGAAGAAAAAAAAATACCAAAATCTCAGAATTTGAATTTACGAT

CTATTCATTCAATATTTCCCTTTTTGGAGGACAAATTATCGCATTTAAATTATGTGTCAGATATACTAAT

ACCTTATCCCATCCATCTGAAAATCTTGGTTCAAATCCTTCAATTCTGGATCCAAGATGTTCCTTCTTTA

CATTTATTGCGATTCTTTCTTCACGAATATCATAATTGGAATAGTCTTATTACTCCGAATAATTCTATTT

TTCTTTTTTCAAAAGAAAATAAAAGACTATTTCGGTTCCCATATAATTCTTATGTATCTGAATGCGAATT

TGTATTAGTTTTTCTTCGTAAACAATCTTCTTATTTACGATTAACATCTTCTGGAGCTTTTCTTGAGCGA

ACACATTTCTATGGAAAAATAGAATATCGTATAGTAGTGCGCCGTAATTATTTTCAGAAGACCCTATGGT

TTTTCAAGGATCCCTTCATGCATTATGTTCGATATCAAGGAAAAGCAATTCTGGTTTCAAAGGGGACTCA

TCTTCTGATGAAGAAATGGAAATGTCACCTTGTCAATTTCTGGCAATATTATTTTCACTTTTGGTCTCAA

CCGTACAGGATTCATATAAACCAATTATCAAACTGTTCTTTCTATTTTCTAGGTTATCTTTCAAGTGTAC

TAATAAATCCTTCGGCGGTAAGGAATCAAATGCTAGAGAATTCATTTCTAATAGATACTGTTATTAAAAA

ATTCGATACCAGAGTCCCAGTTATTCCTCTTATTAGATCATTGTCTAAAGCTAAATTTTGTACCGTATCG

GGGCATTCTATTAGTAAGCCGATCTGGACCGATTTATCAGATTGCGATATTATTGATCGATTTGGTCGGA

TATGTAGAAATCTTTCTCATTATCACAGTGGATCCTCAAAAAAACAGAGTTTGTATCGAATAAAGTATAT

ACTTCGATTTTCGTGTGCTAGAACTTTGGCTCGTAAACATAAAAGAATGGTACGCGCTTTTTTGCAAAGA

TTAGGTTCGGGATTATTAGAAGAATTTTTTATGGAAGAAGAACAAGTTGTTTCTTTGATCTTCCCAAAAA

CAACTTCTTTTTCTTTACATGAATCACATATAGAACGTATTTGGTATTTGGATATTATCCGTATCAATGA

CCTGGTGAATTATTCATAATTGGTTTGTTGACGTGATGAGACTTATGAATAGAATAGAAATGATCTATAA

ATGATCAAGAGAGAAAAAAATTCATGAATTTTCATTCTGAAATGCTCATTGCAGTAGTGTAGTGGTTGAA

TCAACTGAGTAGTCAAAATTATTA

>Brahea_berlandieri_AM114580.1

GACCATATTGCACTATGTATCATTTGATAACCCAAAAAATGAAATAGGTCCCGCCTCTGGTTCAAGTAGA

AATGTAAATGGAAGAATTACAAGGATATTTAGAAAGAGATAGATCTCTGCAACAACACTTTCTATATCCG

CTTCTCTTTAAGGAGTATATTTACACATTTCTTCATGATCGTGGTTTAAATGGTTCGATTTTTTACGAAT

CCACGGAAATTTTTGGTTATGACAATAAATCTAGTTCAGTACTTGTGAAACGTTCAATTATTCGAATGTA

TCAACAGAATTATTTGATTTATTCGGTTAATGATTCTAACCAAAATCGATTCGTTGGGCACAACAATTAT

TTTTATTTTCATTTTTATTCTCAGATGATATTGGAAGGTTTTGCAGTCATTGTGGAAATTCCATTCTTGC

TGCGATTGGTATCTTCCCTCGAAGAAAAAAAAATACCAAAATCTCAGAATTTGAATTTACGATCTATTCA

TTCAATATTTCCCTTTTTGGAGGACAAATTATCGCATTTAAATTATGTGTCAGATATACTAATACCTTAT

CCCATCCATCTGAAAATCTTGGTTCAAATCCTTCAATTCTGGATCCAAGATGTTCCTTCTTTACATTTAT

TGCGATTCTTTCTTCACGAATATCATAATTGGAATAGTCTTATTACTCCGAATAATTCTATTTTTCTTTT

TTCAAAAGAAAATAAAAGACTATTTCGGTTCCCATATAATTCTTATGTATCTGAATGCGAATTTGTATTA

GTTTTTCTTCGTAAACAATCTTCTTATTTACGATTAACATCTTCTGGAGCTTTTCTTGAGCGAACACATT

TCTATGGAAAAATAGAATATCGTATAGTAGTGCGCCGTAATTATTTTCAGAAGACCCTATGGTTTTTCAA

GGATCCCCTCATGCATTATGTTCGATATCAAGGAAAAGCAATTCTGGTTTCAAAGGGGACTCATCTTCTG

ATGAAGAAATGGAAATGTCACCTTGTCAATTTCTGGCAATATTATTTTCACTTTTGGTCTCAACCGTACA

GGATTCATATAAACCAATTATCAAACTGTTCTTTCTATTTTCTAGGTTATCTTTCAAGTGTACTAATAAA

TCCTTCGGCGGTAAGGAATCAAATGCTAGAGAATTCATTTCTAATAGATACTGTTATTAAAAAATTCGAT

ACCGGAGTCCCAGTTATTCCTCTTATTAGATCATTGTCTAAAGCTAAATTTTGTACCGTATCGGGGCATC

CTATTAGTAAGCCGATCTGGACCGATTTATCAGATTGCGATATTATTGATCGATTTGGTCGGATATGTAG

AAATCTTTCTCATTATCACAGTGGATCCTCAAAAAAACAGAGTTTGTATCGAATAAAGTATATACTTCGA

TTTTCGTGTGCTAGAACTTTGGCTCGTAAACATAAAAGTATGGTACGCGCTTTTTTGCAAAGATTAGGTT

CGGGATTATTAGAAGAATTTTTTATGGAAGAAGAACAAGTTGTTTCTTTGATCTTCCCAAAAACAACTTC

TTTTTCTTTACATGAATCACATATAGAACGTATTTGGTATTTGGATATTATCCGTATCAATGACCTGGTG

AATTATTCATAATTGGTTTGTTGACGTGATGAGACTTATGAATAGAATAGAAATGATCTATAAATGATCA

AGAGAGAAAAAAATTCATGAATTTTCATTCTGAAATGCTCATTGCAGTAGTGTAGTGGTTGAATCAACTG

AGTAGTCAAAATTATTATACTTTCTTCTCGGGACCCAAGTTTTTTTTATATTATACATAGGTAAAGTC

>Butia_yatay_AB088794.1

ATGGAAGAATTACAAGGATATTTAGAAAAAGATAGATCTCGGCAACAACACTTTCTATATCCGCTTCTCT

TTAAGGAGTATATTTACACATTTGCTCATGATCGTGGTTTAAATGGTTCGATTTTTTACGAATCCACGGA

AATTTTTGGTTATGACAATAAATCTAGTTCAGTACTTGTGAAACGTTCAATTATTCGAATGTATCAACAG

AATTATTTGATTTATTCGGTTAATGATTCTAACCAAAATCGATTCGTTGGGCACAACAATTATTTTGATT

TTCATTTTTATTCTCAGATGATATTGGAAGGTTTTGCAGTCATTGTGGAAATTCCATTCTTGCTGCGATT

AGTATCTTCCCTCGAAGAAAAAAAAATACCAAAATCTCAGAATTTGAATTTACGATCTATTCATTCAATA

TTTCCCTTTTTGGAGGACAAATTATCGCATTTAAATTATGTGTCAGATATACTAATACCTTATCCCATCC

ATCTGAAAATCTTGGTTCAAATCCTTCAATGCTGGATCCAAGATGTTCCTTCTTTACATTTATTGCGATT

CTTTCTTCACGAATATCATAATTGGAATAGTCTTATTACTCCGAATAATTCTATTTTTTTTTCAAAAGAA

AATAAAAGACTATTTCGGTTCCCATATAATTCTTATGTATCTGAATGCGAATTTGTATTAGTTTTTCTTC

GTAAACAATCTTCTNATTTACGATNAACATCTTCTGGAGCTTTTCTTGGGCGACCACATTTCTATGGAAA

AATTGAACATCTTATAGTAGTGCCCCCGNATTATTTTCAGAAGACCCTATGGTTCTTCAAGGATCCCTTC

ATGCATTATGTTCGATATCAAGGAAAAGCAATTCTGGTTTCAAAGGGGACTCATCTTCTGATGAAGAAAT

GGAAATGTCATCTTGTCAATTTCTGGCAATATTATTTTCACTTTTGGTCTCAACCGTACAGGATCCATAT

AAACCAATTATCAAGCTGTTCTTTCTATTTTCTAGGTTATCTTTCAAGTGTACTAATAAATCCTTCGGCG

GTAAGGAATCAAATGCTAGAGAATTCATTTCTAATAGATACTGTTATTAAAAAATTCGATACCAGAGTCC

CAGTTATTCCTCTTATTGGATCATTGTCTAAAGCTAAATTTTGTACCGTATCGGGGCATCCTATTAGTAA

GCCGATCTGGACCAATTTATCAGATTGCGATATTATTGATCGATTTGGTCGGATATGTAGAAATCTTTCT

CATTATCACAGTGGATCCTCAAAAAAACAGAGTTTGTATCGAATAAAGTATATACTTCGATTTTCGTGTG

CTAGAACTTTGGCTCGTAAACATAAAAGTATGGGACGCGCTTTTTTGCAAAGATTAGGTTCGGGATTATT

AGAAGAATTCTTTACGGAAGAAGAAGAAGTTGTTTCTTTGATCTTCCCAAAAACAACTTCTTTTTCTTTA

CATGAATCACATATAGAACGTATTTGGTATTTGGATATTATCCGNATCAATGACCTGGTGAATTATTCAT

AATGGGTTTGGTGACGTGATGAGA

>Butia_capitata_EU004870.1

CGTTCTGACCATATTGCACTATGTATCATTTGATAACCCAAAAAATGAAATGGGTCCTGTCTCTGGTTCA

AGTAGAAATGTAAATGGAAGAATTACAAGGATATTTAGAAAAAGATAGATCTCGGCAACAACACTTTCTA

TATCCGCTTCTCTTTAAGGAGTATATTTACACATTTGCTCATGATCGTGGTTTAAATGGTTCGATTTTTT

ACGAATCCACGGAAATTTTTGGTTATGACAATAAATCTAGTTCAGTACTTGTGAAACGTTCAATTATTCG

AATGTATCAACAGAATTATTTGATTTATTCGGTTAATGATTCTAACCAAAATCGATTCGTTGGGCACAAC

AATTATTTTGATTTTCATTTTTATTCTCAGATGATATTGGAAGGTTTTGCAGTCATTGTGGAAATTCCAT

TCTTGCTGCGATTAGTATCTTCCCTCGAAGAAAAAAAAATACCAAAATCTCAGAATTTGAATTTACGATC

TATTCATTCAATATTTCCCTTTTTGGAGGACAAATTATCGCATTTAAATTATGTGTCAGATATACTAATA

CCTTATCCCATCCATCTGAAAATCTTGGTTCAAATCCTTCAATGCTGGATCCAAGATGTTCCTTCTTTAC

ATTTATTGCGATTCTTTCTTCACGAATATCATAATTGGAATAGTCTTATTACTCCGAATAATTCTATTTT

TTTTTCAAAAGAAAATAAAAGACTATTTCGGTTCCCATATAATTCTTATGTATCTGAATGCGAATTTGTA

TTAGTTTTTCTTCGTAAACAATCTTCTTATTTACGATTAACATCTTCTGGAGCTTTTCTTGAGCGAACAC

ATTTCTATGGAAAAATAGAACATCTTATAGTAGTGCGCCGTAATTATTTTCAGAAGACCCTATGGTTCTT

CAAGGATCCCTTCATGCATTATGTTCGATATCAAGGAAAAGCAATTCTGGTTTCAAAGGGGACTCATCTT

CTGATGAAGAAATGGAAATGTCATCTTGTCAATTTCTGGCAATATTATTTTCACTTTTGGTCTCAACCGT

ACAGGATCCATATAAACCAATTATCAAGCTGTTCTTTCTATTTTCTAGGTTATCTTTCAAGTGTACTAAT

AAATCCTTCGGCGGTAAGGAATCAAATGCTAGAGAATTCATTTCTAATAGATACTGTTATTAAAAAATTC

GATACCAGAGTCCCAGTTATTCCTCTTATTGGATCATTGTCTAAAGCTAAATTTTGTACCGTATCGGGGC

ATCCTATTAGTAAGCCGATCTGGACCAATTTATCAGATTGCGATATTATTGATCGATTTGGTCGGATATG

TAGAAATCTTTCTCATTATCACAGTGGATCCTCAAAAAAACAGAGTTTGTATCGAATAAAGTATATACTT

CGATTTTCGTGTGCTAGAACTTTGGCTCGTAAACATAAAAGTATGGTACGCGCTTTTTTGCAAAGATTAG

GTTCGGGATTATTAGAAGAATTCTTTACGGAAGAAGAAGAAGTTGTTTCTTTGATCTTCCCAAAAACAAC

TTCTTTTTCTTTACATGAATCACATATAGAACGTATTTGGTATTTGGATATTATCCGTATCAATGACCTG

GTGAATTATTCATAATGGGTTTGGTGACGTGATGAGACTTATGAATAGAATAGAAATGATCTATAAATGA

TCAAGAGAGAAAAAAATTCATGAATTTTCATTCTGAAATGCTCATTGCAGTAGTGTAGTGGTTGAATCAA

CTGAGTAGTCAAAATTATTATACTTTCTTCTCGGGACCCAAGTTTTATATTATACATAGGTAAAGTCGTG

TGCAATGA

>Calyptrogyne_ghiesbreghtiana_AM114652.1

GACCATATTGCACTATGTATCATTTGATAACCCCAAAAATGAAATGGGTCCTGCCTCTGGTTCAAGTAGA

AATGTAAATGGAAGAATTACAAGGATATTTAGAAAAAGATAGATCTCGGCAACAACACTTTCTATATCCG

CTTCTCTTTAAGGAGTATATTTACACATTTGCTCATGATCGTGGTTTAAATGGTTCGATTTTTTACGAAT

CCACGGAAATTTTTGGTTATGACAATAAATCTAGTTCAGTACTTGTGAAACGTTCAATTATTCGAATGTA

TCAACAGAATTATTTGATTTATTCGGTTAACGATTCTAACCAAAATCGATTCGTTGGGCACAACAATTAT

TTTTATTTTCATTTTTATTCTCAGATGATATTGGAAGGTTTTGCAGTCATTGTGGAAATTCCATTCTTGC

TGCGATTAGTATCTTCCCTCGAAGAAAAAAAAATACCAAAATCTCAGAATTTGAATTTACGATCTATTCA

TTCAATATTTCCCTTTTTGGAGGACAAATTATCGCATTTAAATTATGTGTCAGATATACTAATACCTTAT

CCCATCCATCTGAAAATCTTGGTTCAAATCCTTCAATGCTGGATCCAAGATGTTCCTTCTTTACATTTAT

TGCGATTCTTTCTTCACGAATATCATAATTGGAATAGTCTTATTACTCCGAAAAATTCTATTTTTTTTTT

TTCAAAAGAAAATAAAAGACTATTTCGGTTCCCATATAATTCTTATGTATCTGAATGCGAATTTGTATTA

GTTTTTCTTCGTAAACAATCTTCTTATTTACGATTAACATCTTCTGGAGCTTTTCTTGAGCGAACACATT

TCTATGGAAAAATAGAACATCTTATAGTAGTGCGCCGTAATTATTTTCAGAAGACCCTATGGTTCTTCAA

GGATCCCTTCATGCATTATGTTCGATATCAAGGAAAAGCAATTCTGGTTTCAAGGGGGACCCATCTTCTG

ATGAAGAAATGGAAATGTCACCTTGTCAATTTCTGGCAATATTATTTTCACTTTTGGTCTCAACCGTACA

GGATCCATATAAACCAATTATCAAGCTGTTCTTTCTATTTTCTAGGTTATCTTTCAAGTGTACTAATAAA

TCCTTCGGCGGTAAGGAATCAAATGCTAGAGAATTCATTTCTAATGGATACTGTTATTAAAAAATTCGAT

ACCAGAGTCCCAGTTATTCCTCTTATTGGATCATTGTCTAAAGCTAAATTTTGTACCGTATCGGGGCATC

CTATTAGTAAGCCGATCTGGACCAATTTATCAGATTGCGATATTATTGATCGATTTGGTCGGATATGTAG

AAATCTTTTTCATTATCACAGTGGATCCTCAAAAAAACAGAGTTTGTATCGAATAAAGTATATACTTCGA

TTTTCGTGTGCTAGAACTTTGGCTCGTAAACATAAAAGTATGGTACGCGCTTTTTTGCAAAGATTAGGTT

CGGGATTATTAGAAGAATTCTTTACGGAAGAAGAACAAGTTGTTTCTTTGATCTTCCCAAAAACGACTTC

TTTTTCTTTACATGAATCACATATAGAACGTATTTGGTATTTGGATATTATCCGTATCAATGACCTGGTG

AATTATTCATAATGGGTTTGGTGACGTGATGAGACTTATGAATAGAATAGAAATGATCTATAAATGATCA

AGAGAGAAAAAAATTCATGAATTTTCATTCTGAAATGCTCATTGCAGTAGTGTAGTGGTTGAATCAACTG

AGTAGTCAAAATTATTATACTTTCTTCTCGGGACCCAAGTTTTATACTATACATAGGTAAAGTC

>Calyptronoma_occidentalis_AM114653.1

AATGAAATGGGTCCTGCCTCTGGTTCAAGTAGAAATGTAAATGGAAGAATTACAAGGATATTTAGAAAAA

GATAGATCTCGGCAACAACACTTTCTATATCCGCTTCTCTTTAAGGAGTATATTTACACATTTGCTCATG

ATCGTGGTTTAAATGGTTCGATTTTTTACGAATCCACGGAAATTTTTGGTTATGACAATAAATCTAGTTC

AGTACTTGTGAAACGTTCAATTATTCGAATGTATCAACAGAATTATTTGATTTATTCGGTTAACGATTCT

AACCAAAATCGATTCGTTGGGCACAACAATTATTTTTATTTTCATTTTTATTCTCAGATGATATTGGAAG

GTTTTGCAGTCATTGTGGAAATTCCATTCTTGCTGCGATTAGTATCTTCCCTCGAAGAAAAAAAAATACC

AAAATCTCAGAATTTGAATTTACGATCTATTCATTCAATATTTCCCTTTTTGGAGGACAAATTATCGCAT

TTAAATTATGTGTCAGATATACTAATACCTTATCCCATCCATCTGAAAATCTTGGTTCAAATCCTTCAAT

GCTGGATCCAAGATGTTCCTTCTTTACATTTATTGCGATTCTTTCTTCACGAATATCATAATTGGAATAG

TCTTATTACTCCGAATAATTCTATTTTTTTTTTTTCAAAAGAAAATAAAAGACTATTTCGGTTCACATAT

AATTCTTATGTATCTGAATGCGAATTTGTATTAGTTTTTCTTCGTAAACAATCTTCTTATTTACGATTAA

CATCTTCTGGAGCTTTTCTTGAGCGAACACATTTCTATGGAAAAATAGAACATCTTATAGTAGTGCGCCG

TAATTATTTTCAGAAGACCCTATGGTTCTTCAAGGATCCCTTCATGCATTATGTTCGATATCAAGGAAAA

GCAATTCTGGTTTCAAGGGGGACCCATCTTCTGATGAAGAAATGGAAATGTCACCTTGTCAATTTCTGGC

AATATTATTTTCACTTTTGGTCTCAACCGTACAGGATCCATATAAACCAATTATCAAGCTGTTCTTTCTA

TTTTCTAGGTTATCTTTCAAGTGTACTAATAAATCCTTCGGCGGTAAGGAATCAAATGCTAGAGAATTCA

TTTCTAATAGATACTGTTATTAAAAAATTCGATACCAGAGTCCCAGTTATTCCTCTTATTGGATCATTGT

CTAAAGCTAAATTTTGTACCGTATCGGGGCATCCTATTAGTAAGCCGATCTGGACCAATTTATCAGATTG

CGATATTATTGATCGATTTGGTCGGATATGTAGAAATCTTTCTCATTATCACAGTGGATCCTCAAAAAAA

CAGAGTTTGTATCGAATAAAGTATATACTTCGATTTTCGTGTGCTAGAACTTTGGCTCGTAAACATAAAA

GTATGGTACGCGCTTTTTTGCAAAGATTAGGTTCGGGATTATTAGAAGAATTCTTTACGGAAGAAGAACA

AGTTGTTTCTTTGATCTTCCCAAAAACTACTTCTTTTTCTTTACATGAATCACATATAGAACGTATTTGG

TATTTGGATATTATCCGTATCAATGACCTGGTGAATTATTCATAATGGGTTTGGTGACGTGATGAGACTT

ATGAATAGAATAGAAATGATCTATAAATGATCAAGAGAGAAAAAAATTCATGAATTTTCATTCTGAAATG

CTCATTGCAGTAGTGTAGTGGTTGAATCAACTGAGTAGTCAAAATTATTATACTTTCTTCTCGGGACCCA

AGTTTTATACTATACATAGGTAAAGTC

>Ceroxylon_vogelianum_EF128264.1

AACCCMAAAAATTAAATGGGTCCTGCCTCTGGTTCAAGTAGAAATGTAAATGGAAGAATTACAAGGATAT

TTAGAAAAAGATAGATCTCGGCAACAACACTTTCTATATCCGCTTCTCTTTAAGGAGTATATTTACACAT

TTGCTCATGATCGTGGTTTAAATGGTTCGATTTTTTACGAATCCACGGAAATTTTTTGTTATGACAATAA

ATCTAGTTCAGTACTTGTGAAACGTTCAATTATTCGAATGTATCAACAGAATTATTTGATTTATTCGGTT

AATGATTCTAACCAAAATCGATTCGTTGGGCACAACAATTATTTTCATTTTTATTCTCAGATGATATTGG

AAGGTTTTGCAGTCATTGTGGAAATTCCATTCTTGCTGCGATTAGTATCTTCCCWCGAAGAAAAAAAAAT

ACCAAAATCTCAGAATTTGAATTTACGATCTATTCATTCAATATTTCCCTTTTTGGAGGACAAATTATCG

CATTTAAATTATGTGTCAGATATACTAATACCTTATCCCATCCATCTGAAAATCTTGGTTCAAATCCTTC

AATGCTGGATCCAARATGTTCCTTCTTTACATTTATTGCGATTCTTTCTTCACGAATATCATAATTGGAA

TAGTCTTATTACTCCGAATAATTCTATTTTTTTTTTTTCAAAAGAAAATAAAAGACTATTTCGGTTCCCA

TATAATTCTTATGTATCTGAATGCGAATTTGTATTAGTTTTTCTTCGTAAACAATCTTCTTATTTACGAT

TAACATCTTCTGGAGCTTTTCTTGAGCGAACACATTTCTATGGAAAAATAGAACATCTTATAGTAGTGCG

CCGTAATTATTTTCAGAAGACCCTATGGTTCTTCAAGGATCCCTTCATGCATTATGTTCGATATCAAGGA

AAAGCAATTCTGGTCTCAAAGGGGACTCATCTTCTGATGAAGAAATGGAAATGTCACCTTGTCAATTTCT

GGCAATATTATTTTCACTTTTGGTCTCAACCGYACAGGATCCATATAAACCAATTATCAAACTGTTCTTT

CTATTTTCTAGGTTATCTTTCAAGTGTACTAATAAATCCTTCGGCGGTAAGGAATCAAATGCTAGAGAAT

TCATTTCTAATAGATACTGTTATTAAAAAATTCGATACCAGAGTCCCAGTTATTCCTCTTATTGGATCAT

TGTCTAAAGCTAAATTTTGTACCGTATCGGGGCATCCTATTAGTAAGCCGATCTGGACCAATTTATCAGA

TTGCGATATTATTGATCGATTTGGTCGGATATGTAGAAATCTTTCTCATTATCACAGTGGATCCTCAAAA

AAACAGAGTTTGTATCGAATAAAGTATATACTTCGATTTTCGTGTGCTAGAACTTTGGCTCGTAAACATA

AAAGTATGGTACGCGCTTTTTTGCAAAGATTAGGTTCGGGATTATTAGAAGAATTCTTTACGGAAGAAGA

ACAAGTTGTTTCTTTGATCTTCCCAAAAACAACTTCTTTTTCTTTACATGAATCACATATAGAACGTGTT

TGGTATTTGGATATTATCCGTATCAATGACCTGGTGAATTATTCATAATGGGTTTGGTGACGTGACGAGA

CTTATGAATAGAATGGAAATGATCTATAAATGATCAAGAGAGAAAAAAATTCATGAATTTTCATTCTGAA

ATGCTCATTGCAGTAGTGTAGTGGTTGAATCAACTGAGTAGCCAAAATTATTATACTTTC

>Ceroxylon_parvum_EF128262.1

AACCCAAAAAATGAAATGGGTCCTGCCTCTGGTTCAAGTAGAAATGTAAATGGAAGAATTACAAGGATAT

TTAGAAAAAGATAGATCTCGGCAACAACACTTTCTATATCCGCTTCTCTTTAAGGAGTATATTTACACAT

TTGCTCATGATCGTGGTTTAAATGGTTCGATTTTTTACGAATCCACGGAAATTTTTTGTTATGACAATAA

ATCTAGTTCAGTACTTGTGAAACGTTCAATTATTCGAATGTATCAACAGAATTATTTGATTTATTCGGTT

AATGATTCTAACCAAAATCGATTCGTTGGGCACAACAATAATTTTTATTTTCATTTTTATTCTCAGATGA

TATTGGAAGGTTTTGCAGTCATTGTGGAAATTCCATTCTTGCTGCGATTAGTATCTTCCCTCGAAGAAAA

AAAAATACCAAAATCTCAGAATTTGAATTTACGATCTATTCATTCAATATTTCCCTTTTTGGAGGACAAA

TTATCGCATTTAAATTATGTGTCAGATATACTAATACCTTATCCCATCCATCTGAAAATCTTGGTTCAAA

TCCTTCAATGCTGGATCCAAGATGTTCCTTCTTTACATTTATTGCGATTCTTTCTTCACGAATATCATAA

TTGGAATAGTCTTATTACTCCGAATAATTCTATTTTTTTTTTTTCAAAAGAAAATAAAAGACTATTTCGG

TTCCCATATAATTCTTATGTATCTGAATGCGAATTTGTATTAGTTTTTCTTCGTAAACAATCTTCTTATT

TACGATTAACATCTTCTGGAGCTTTTCTTGAGCGAACACATTTCTATGGAAAAATAGAACATCTTATAGT

AGTGCGCCGTAATTATTTTCAGAAGACCCTATGGTTCTTCAAGGATCCCTTCATGCATTATGTTCGATAT

CAAGGAAAAGCAATTCTGGTCTCAAAGGGGACTCATCTTCTGATGAAGAAATGGAAATGTCACCTTGTCA

ATTTCTGGCAATATTATTTTCACTTTTGGTCTCAACCGTACAGGATCCATATAAACCAATTATCAAACTG

TTCTTTCTATTTTCTAGGTTATCTTTCAAGTGTACTAATAAATCCTTCGGCGGTAAGGAATCAAATGCTA

GAGAATTCATTTCTAATAGATACTGTTATTAAAAAATTCGATACCAGAGTCCCAGTTATTCCTCTTATTG

GATCATTGTCTAAAGCTAAATTTTGTACCGTATCGGGGCATCCTATTAGTAAGCCGATCTGGACCAATTT

ATCAGATTGCGATATTATTGATCGATTTGGTCGGATATGTAGAAATCTTTCTCATTATCACAGTGGATCC

TCAAAAAAACAGAGTTTGTATCGAATAAAGTATATACTTCGATTTTCGTGTGCTAGAACTTTGGCTCGTA

AACATAAAAGTATGGTACGCGCTTTTTTGCAAAGATTAGGTTCGGGATTATTAGAAGAATTCTTTACGGA

AGAAGAACAAGTTGTTTCTTTGATCTTCCCAAAAACAACTTCTTTTTCTTTACATGAATCACATATAGAA

CGTGTTTGGTATTTGGATATTATCCGTATCAATGACCTGGTGAATTATTCATAATGGGTTTGGTGACGTG

ACGAGACTTATGAATAGAATGGAAATGATCTATAAATGATCAAGAGAGAAAAAAATTCATGAATTTTCAT

TCTGAAATGCTCATTGSAGTAGTGTAGTGGTTGAATCAACTGAGTAGCCAAAATTATTATACTTTC

>Ceroxylon_parvifrons_EF128260.1

AACCCAAAAAATGAAATGGGTCCTGCCTCTGGTTCAAGTAGAAATGTAAATGGAAGAATTACAAGGATAT

TTAGAAAAAGATAGATCTCGGCAACAACACTTTCTATATCCGCTTCTCTTTAAGGAGTATATTTACACAT

TTGCTCATGATCGTGGTTTAAATGGTTCGATTTTTTACGAATCCACGGAAATTTTTTGTTATGACAATAA

ATCTAGTTCAGTACTTGTGAAACGTTCAATTATTCGAATGTATCAACAGAATTATTTGATTTATTCGGTT

AATGATTCTAACCAAAATCGATTCGTTGGGCACAACAATTATTTTTATTTTCATTTTTATTCTCAGATGA

TATTGGAAGGTTTTGCAGTCATTGTGGAAATTCCATTCTTGCTGCGATTAGTATCTTCCCTCGAAGAAAA

AAAAATACCAAAATCTCAGAATTTGAATTTACGATCTATTCATTCAATATTTCCCTTTTTGGAGGACAAA

TTATCGCATTTAAATTATGTGTCAGATATACTAATACCTTATCCCATCCATCTGAAAATCTTGGTTCAAA

TCCTTCAATGCTGGATCCAAGATGTTCCTTCTTTACATTTATTGCGATTCTTTCTTCACGAATATCATAA

TTGGAATAGTCTTATTACTCCGAATAATTCTATTTTTTTTTTTTCAAAAGAAAATAAAAGACTATTTCGG

TTCCCATATAATTCTTATGTATCTGAATGCGAATTTGTATTAGTTTTTCTTCGTAAACAATCTTCTTATT

TACGATTAACATCTTCTGGAGCTTTTCTTGAGCGAACACATTTCTATGGAAAAATAGAACATCTTATAGT

AGTGCGCCGTAATTATTTTCAGAAGACCCTATGGTTCTCCAAGGATCCCTTCATGCATTATGTTCGATAT

CAAGGAAAAGCAATTCCGGTCTCAAAGGGGACTCATCTTCTGATGAAGAAATGGAAATGTCACCTTGTCA

ATTTCTGGCAATATTATTTTCACTTTTGGTCTCAACCGTACAGGATCCATATAAACCAATTATCAAACTG

TTCTTTCTATTTTCTAGGTTATCTTTCAAGTGTACTAATAAATCCTTCGGCGGTAAGGAATCAAATGCTA

GAGAATTCATTTCTAATAGATACTGTTATTAAAAAATTCGATACCAGAGTCCCAGTTATTCCTCTTATTG

GATCATTGTCTAAAGCTAAATTTTGTACCGTATCGGGGCATCCTATTAGTAAGCCGATCTGGACCAATTT

ATCAGATTGCGATATTATTGATCGATTTGGTCGGATATGTAGAAATCTTTCTCATTATCACAGTGGATCC

TCAAAAAAACAGAGTTTGTATCGAATAAAGTATATACTTCGATTTTCGTGTGCTAGAACTTTGGCTCGTA

AACATAAAAGTATGGTACGCGCTTTTTTGCAAAGATTAGGTTCGGGATTATTAGAAGAATTCTTTACGGA

AGAAGAACAAGTTGTTTCTTTGATCTTCCCAAAAACAACTTCTTTTTCTTTACATGAATCACATATAGAA

CGTGTTTGGTATTTGGATATTATCCGTATCAATGACCTGGTGAATTATTCATAATGGGTTTGGTGACGTG

ACGAGACTTATGAATAGAATGGAAATGATCTATAAATGATCAAGAGAGAAAAAAATTCATGAATTTTCAT

TCTGAAATGCTCATTGCAGTAGTGTAGTGGTTGAATCAACTGAGTAGCCAAAATTATTATACTTTC

>Ceroxylon_echinulatum_EF128258.1

AACCCAAAAAATGAAATGGGTCCTGCCTCTGGTTCAAGTAGAAATGTAAATGGAAGAATTACAAGGATAT

TTAGAAAAAGATAGATCTCGGCAACAACACTTTCTATATCCGCTTCTCTTTAAGGAGTATATTTACACAT

TTGCTCATGATCGTGGTTTAAATGGTTCGATTTTTTACGAATCCACGGAAATTTTTTGTTATGACAATAA

ATCTAGTTCAGTACTTGTGAAACGTTCAATTATTCGAATGTATCAACAGAATTATTTGATTTATTCGGTT

AATGATTCTAACCAAAATCGATTCGTTGGGCACAACAATTATTTTTATTTTCATTTTTATTCTCAGATGA

TATTGGAAGGTTTTGCAGTCATTGTGGAAATTCCATTCTTGCTGCGATTAGTATCTTCCCTCGAAGAAAA

AAAAATACCAAAATCTCAGAATTTGAATTTACGATCTATTCATTCAATATTTCCCTTTTTGGAGGACAAA

TTATCGCATTTAAATTATGTGTCAGATATACTAATACCTTATCCCATCCATCTGAAAATCTTGGTTCAAA

TCCTTCAATGCTGGATCCAAGATGTTCCTTCTTTACATTTATTGCGATTCTTTCTTCACGAATATCATAA

TTGGAATAGTCTTATTACTCCGAATAATTCTATTTTTTTTTTTTCAAAAGAAAATAAAAGACTATTTCGG

TTCCCATATAATTCTTATGTATCTGAATGCGAATTTGTATTAGTTTTTCTTCGTAAACAATCTTCTTATT

TACGATTAACATCTTCTGGAGCTTTTCTTGAGCGAACACATTTCTATGGAAAAATAGAACATCTTATAGT

AGTGCGCCGTAATTATTTTCAGAAGACCCTATGGTTCTTCAAGGATCCCTTCATGCATTATGTTCGATAT

CAAGGNAAAGCAATTCTGGTCTCAAAGGGGACTCATCTTCTGATGAAGAAATGGAAATGTCACCTTGTCA

ATTTCTGGCAATATTATTTTCACTTTTGGTCTCAWCCGTACAGGATCCATATAAACCAATTATCAAACTG

TTCTTTCTATTTTCTAGGTTATCTTTCAAGTGTACTAATAAATCCTTCGGCGGTAAGGAATCAAATGCTA

GAGAATTCATTTCTAATAGATACTGTTATTAAAAAATTCGATACCAGAGTCCCAGTTATTCCTCTTATTG

GATCATTGTCTAAAGCTAAATTTTGTACCGTATCGGGGCATCCTATTAGTAAGCCGATCTGGACCAATTT

ATCAGATTGCGATATTATTGATCGATTTGGTCGGATATGTAGAAATCTTTCTCATTATCACAGTGGATCC

TCAAAAAAACAGAGTTTGTATCGAATAAAGTATATACTTCGATTTTCGTGTGCTAGAACTTTGGCTCGTA

AACATAAAAGTATGGTACGCGCTTTTTTGCAAAGATTAGGTTCGGGATTATTAGAAGAATTCTTTACGGA

AGAAGAACAAGTTGTTTCTTTGATCTTCCCAAAAACAACTTCTTTTTCTTTACATGAATCACATATAGAA

CGTGTTTGGTATTTGGATATTATCCGTATCAATGACCTGGTGAATTATTCATAATGGGTTTGGTGACGTG

ACGAGACTTATGAATAGAATGGAAATGATCTATAAATGATCAAGAGAGAAAAAAATTCATGAATTTTCAT

TCTGAAATGCTCATTGCAGTAGTGTAGTGGTTGAATCAACTGAGTAGCCAAAATTATTATACTTTC

>Ceroxylon_echinulatum_EF128256.1

AACCCAAAAAATGAAATGGGTCCTGCCTCTGGTTCAAGTAGAAATGTAAATGGAAGAATTACAAGGATAT

TTAGAAAAAGATAGATCTCGGCAACAACACTTTCTATATCCGCTTCTCTTTAAGGAGTATATTTACACAT

TTGCTCATGATCGTGGTTTAAATGGTTCGATTTTTTACGAATCCACGGAAATTTTTTGTTATGACAATAA

AKCKAGTTCAGKACTTGTGAAACGTKCAATTATTCGAATGTATCAACAGAATTATTTGATTTATTCGGTT

AATGATTCTAACCAAAATCGATTCGTTGGGCACAACAATTATTTTTATTTTAATTTTTATTCTCAGATGA

TATTGGAAGGTTTTGCAGTCATTGTGGAAATTCCATTCTTGCTGCGATTAGTATCTTCCCTCGAAGAAAA

AAAAATACCAAAATCTCAGAATTTGAATTTACGATCTATTCATTCAATATTTCCCTTTTTGGAGGACAAA

TTATCGCATTTAAATTATGTGTCAGATATACTAATACCTTATCCCATCCATCTGAAAATCTTGGTTCAAA

TCCTTCAATGCTGGATCCAAGATGTTCCTTCTTTACATTTATTGCGATTCTTTCTTCACGAATATCATAA

TTGGAATAGTCTTATTACTCCGAATAATTCTATTTTTTTTTTTCAAAATAAAAGACTATTTCGGTTCCCA

TATAATTCTTATGTATCTGAATGTGAATTTGTATTAGTTTTTCTTCGTAAACAATCTTCTTATTTACGAT

TAACATCTTCTGGAGCTTTTCTTGAGCGAACACATTTCTATGGAAAAATAGAACATCTTATAGTAGTGCG

CCGTAATTATTTTCAGAAGACCCTATGGTTCTTCAAGGATCCCTTCATGCATTATGTTCGATATCAAGGA

AAAGCAATTCTGGTCTCAAAGGGGACTCATCTTCTGATGAAGAAATGGAAATGTCACCTTGTCAATTTCT

GGCAATATTATTTTCACTTTTGGTCTCAACCGTACAGGATCCATATAAACCAATTATCAAACTGTTCTTT

CTATTTTCTAGGTTATCTTTCAAGTGTACTAATAAATCCTTCGGCGGTAAGGAATCAAATGCTAGAGAAT

TCATTTCTAATAGATACTGTTATTAAAAAATTCGATACCAGAGTCCCAGTTATTCCTCTTATTGGATCAT

TGTCTAAAGCTAAATTTTGTACCGTATCGGGGCATCCTATTAGTAAGCCGATCTGGACCAATTTATCAGA

TTGCGATATTATTGATCGATTTGGTCGGATATGTAGAAATCTTTCTCATTATCACAGTGGATCCTCAAAA

AAACAGAGTTTGTATCGAATAAAGTATATACTTCGATTTTCGTGTGCTAGAACTTTGGCTCGTAAACATA

AAAGTATGGTACGCGCTTTTTTGCAAAGATTAGGTTCGGGATTATTAGAAGAATTCTTTACGGAAGAAGA

ACAAGTTGTTTCTTTGATCTTCCCAAAAACAACTTCTTTTTCTTTACATGAATCACATATAGAACGTGTT

TGGTATTTGGATATTATCCGTATCAATGACCTGGTGAATTATTCATAATGGGTTTGGTGACGTGACGAGA

CTTATGAATAGAATGGAAATGATCTATAAATGATCAAGAGAGAAAAAAATTCATGAATTTTCATTCTGAA

ATGCTCATTGCAGTAGTGTAGTGGTTGAATCAACTGAGTAGCCAAAATTATTATACTTTC

>Ceroxylon_alpinum_subsp.ecuadorense_EF128254.1

AACCCAAAAAATGAAATGGGTCCTGCCTCTGGTTCAAGTAGAAATGTAAATGGAAGAATTACAAGGATAT

TTAGAAAAAGATAGATCTCGGCAACAACACTTTCTATATCCGCTTCTCTTTAAGGAGTATATTTACACAT

TTGCTCATGATCGTGGTTTAAATGGTTCGATTTTTTACGAATCCACGGAAATTTTTTGTTATGACAATAA

ATCTAGTTCAGTACTTGTGAAACGTTCAATTATTCGAATGTATCAACAGAATTATTTGATTTATTCGGTT

AATGATTCTAACCAAAATCGATTCGTTGGGCACAACAATTATTTTTATTTTAATTTTTATTCTCAGATGA

TATTGGAAGGTTTTGCAGTCATTGTGGAAATTCCATTCTTGCTGCGATTAGTATCTTCCCACGAAGAAAA

AAAAATACCAAAATCTCAGAATTTGAATTTACGATCTATTCATTCAATATTTCCCTTTTTGGAGGACAAA

TTATCGCATTTAAATTATGTGTCAGATATACTAATACCTTATCCCATCCATCTGAAAATCTTGGTTCAAA

TCCTTCAATGCTGGATCCAAGATGTTCCTTCTTTACATTTATTGCGATTCTTTCTTCACGAATATCATAA

TTGGAATAGTCTTATTACTCCGAATAATTCTATTTTTTTTTTTTCAAAAGAAAATAAAAGACTATTTCGG

TTCCCATATAATTCTTATGTATCTGAATGCGAATTTGTATTAGTTTTTCTTCGTAAACAATCTTCTTATT

TACGATTAACATCTTCTGGAGCTTTTCTTGAGCGAACACATTTCTATGGAAAAATAGAACATCTTATAGT

AGTGCGCCGTAATTATTTTCAGAAGACCCTATGGTTCCTNCAAGGATCCCNTCATGCATTATGTTCGATA

TCAAGGAAAAGCAATTCTGGTCTCAAAGGGGACTCATCTTCTGATGAAGAAATGGAAATGTCACCTTGTC

AATTTCTGGCAATATTATTTTCACTTTTGGTCTCAACCGTACAGGATCCATATAAACCAATTATCAAACT

GTTCTTTCTATTTTCTAGGTTATCTTTCAAGTGTACTAATAAATCCTTCGGCGGTAAGGAATCAAATGCT

AGAGAATTCATTTCTAATAGATACTGTTATTAAAAAATTCGATACCAGAGTCCCAGTTATTCCTCTTATT

GGATCATTGTCTAAAGCTAAATTTTGTACCGTATCGGGGCATCCTATTAGTAAGCCGATCTGGACCAATT

TATCAGATTGCGATATTATTGATCGATTTGGTCGGATATGTAGAAATCTTTCTCATTATCACAGTGGATC

CTCAAAAAAACAGAGTTTGTATCGAATAAAGTATATACTTCGATTTTGTAAACATAAAAGTATGGTACGC

GCTTTTTTGCAAAGATTAGGTTCGGGATTATTAGAAGAATTCTTTACGGAAGAAGAACAAGTTGTTTCTT

TGATCTTCCCAAAAACAACTTCTTTTTCTTTACATGAATCACATATAGAACGTGTTTGGTATTTGGATAT

TATCCGTATCAATGACCTGGTGAATTATTCATAATGGGTTTGGTGACGTGACGAGACTTATGAATAGAAT

GGAAATGATCTATAAATGATCAAGAGAGAAAAAAATTCATGAATTTTCATTCTGAAATGCTCATTGCAGT

AGTGTAGTGGTTGAATCAACTGAGTAGCCAAAATTATTATACTTTC

>Ceroxylon_vogelianum_EF128265.1

AACCCAAAAAATGAAATGGGTCCTGCCTCTGGTTCAAGTAGAAATGTAAATGGAAGAATTACAAGGATAT

TTAGAAAAAGATAGATCTCGGCAACAACACTTTCTATATCCGCTTCTCTTTAAGGAGTATATTTACACAT

TTGCTCATGATCGTGGTTTAAATGGTTCGATTTTTTACGAATCCACGGAAATTTTTTGTTATGACAATAA

ATCTAGTTCAGTACTTGTGAAACGTTCAATTATTCGAATGTATCAACAGAATTATTTGATTTATTCGGTT

AATGATTCTAACCAAAATCGATTCGTTGGGCACAACAATTATTTTTATTTTAATTTTTATTCTCAGATGA

TATTGGAAGGTTTTGCAGTCATTGTGGAAATTCCATTCTTGCTGCGATTAGTATCTTCCCTCGAAGAAAA

AAAAATACCAAAATCTCAGAATTTTAATTTACGATCTATTCATTCAATATTTCCCTTTTTGGAGGACAAA

TTATCGCATTTAAATTATGTGTCAGATATACTAATACCTTATCCCATCCATCTGAAAATCTTGGTTCAAA

TCCTTCAATGCTGGATCCAAGATGTTCCTTCTTTACATTTATTGCGATTCTTTCTTCACGAATATCATAA

TTGGAATAGTCTTATTACTCCGAATAATTCTATTTTTTTTTTTTCAAAAGAAAATAAAAGACTATTTCGG

TTCCCATATAATTCTTATGTATCTGAATGCGAATTTGTATTAGTTTTTCTTCGTAAACAATCTTCTTATT

TACGATTAACATCTTCTGGAGCTTTTCTTGAGCGAACACATTTCTATGGAAAAATAGAACATCTTATAGT

AGTGCGCCGTAATTATTTTCAGAAGACCCTATGGTTCTTCAAGGATCCCTTCATGCATTATGTTCGATAT

CAAGGAAAAGCAATTCTGGTCTCAAAGGGGACTCATCTTCTGATGAAGAAATGGAAATGTCACCTTGTCA

ATTTCTGGCAATATTATTTTCACTTTTGGTCTCAACCGYACAGGATCCATATAAACCAATTATCAAACTG

TTCTTTCTATTTTCTAGGTTATCTTTCAAGTGTACTAATAAATCCTTCGGCGGTAAGGAATCAAATGCTA

GAGAATTCATTTCTAATAGATACTGTTATTAAAAAATTCGATACCAGAGTCCCAGTTATTCCTCTTATTG

GATCATTGTCTAAAGCTAAATTTTGTACCGTATCGGGGCATCCTATTAGTAAGCCGATCTGGACCAATTT

ATCAGATTGCGATATTATTGATCGATTTGGTCGGATATGTAGAAATCTTTCTCATTATCACAGTGGATCC

TCAAAAAAACAGAGTTTGTATCGAATAAAGTATATACTTCGATTTGTAAACATAAAAGTATGGTACGCGC

TTTTTTGCAAAGATTAGGTTCGGGATTATTAGAAGAATTCTTTACGGAAGAAGAACAAGTTGTTTCTTTG

ATCTTCCCAAAAACAACTTCTTTTTCTTTACATGAATCACATATAGAACGTGTTTGGTATTTGGATATTA

TCCGTATCAATGACCTGGTGAATTATTCATAATGGGTTTGGTGACGTGACGAGACTTATGAATAGAATGG

AAATGATCTATAAATGATCAAGAGAGAAAAAAATTCATGAATTTTCATTCTGAAATGCTCATTGCAGTAG

TGTAGTGGTTGAATCAACTGAGTAGCCAAAATTATTATACTTTC

>Ceroxylon_ventricosum_EF128263.1

AACCCAAAAAATGAAATGGGTCCTGCCTCTGGTTCAAGTAGAAATGTAAATGGAAGAATTACAAGGATAT

TTAGAAAAAGATAGATCTCGGCAACAACACTTTCTATATCCGCTTCTCTTTAAGGAGTATATTTACACAT

TTGCTCATGATCGTGGTTTAAATGGTTCGATTTTTTACGAATCCACGGAAATTTTTTGTTATGACAATAA

ATCTAGTTCAGTACTTGTGAAACGTTCAATTATTCGAATGTATCAACAGAATTATTTGATTTATTCGGTT

AATGATTCTAACCAAAATCGATTCGTTGGGCACAACAATTATTTTTATTTTCATTTTTATTCTCAGATGA

TATTGGAAGGTTTTGCAGTCATTGTGGAAATTCCATTCTTGCTGCGATTAGTATCTTCCCTCGAAGAAAA

AAAAATACCAAAATCTCAGAATTTGAATTTACGATCTATTCATTCAATATTTCCCTTTTTGGAGGACAAA

TTATCGCATTTAAATTATGTGTCAGATATACTAATACCTTATCCCATCCATCTGAAAATCTTGGTTCAAA

TCCTTCAATGCTGGATCCAAGATGTTCCTTCTTTACATTTATTGCGATTCTTTCTTCACGAATATCATAA

TTGGAATAGTCTTATTACTCCGAATAATTCTATTTTTTTTTTTTCAAAAGAAAATAAAAGACTATTTCGG

TTCCCATATAATTCTTATGTATCTGAATGCGAATTTGTATTAGTTTTTCTTCGTAAACAATCTTCTTATT

TACGATTAACATCTTCTGGAGCTTTTCTTGAGCGAACACATTTCTATGGAAAAATAGAACATCTTATAGT

AGTGCGCCGTAATTATTTTCAGAAGACCCTATGGTTCTTCAAGGATCCCTTCATGCATTATGTTCGATAT

CAAGGAAAAGCAATTCTGGTCTCAAAGGGGACTCATCTTCTGATGAAGAAATGGAAATGTCACCTTGTCA

ATTTCTGGCAATATTATTTTCACTTTTGGTCTCAACCGTACAGGATCCATATAAACCAATTATCAAACTG

TTCTTTCTATTTTCTAGGTTATCTTTCAAGTGTACTAATAAATCCTTCGGCGGTAAGGAATCAAATGCTA

GAGAATTCATTTCTAATAGATACTGTTATTAAAAAATTCGATACCAGAGTCCCAGTTATTCCTCTTATTG

GATCATTGTCTAAAGCTAAATTTTGTACCGTATCGGGGCATCCTATTAGTAAGCCGATCTGGACCAATTT

ATCAGATTGCGATATTATTGATCGATTTGGTCGGATATGTAGAAATCTTTCTCATTATCACAGTGGATCC

TCAAAAAAACAGAGTTTGTATCGAATAAAGTATATACTTCGATTTTCGTGTGCTAGAACTTTGGCTCGTA

AACATAAAAGTATGGTACGCGCTTTTTTGCAAAGATTAGGTTCGGGATTATTAGAAGAATTCTTTACGGA

AGAAGAACAAGTTGTTTCTTTGATCTTCCCAAAAACAACTTCTTTTTCTTTACATGAATCACATATAGAA

CGTGTTTGGTATTTGGATATTATCCGTATCAATGACCTGGTGAATTATTCATAATGGGTTTGGTGACGTG

ACGAGACTTATGAATAGAATGGAAATGATCTATAAATGATCAAGAGAGAAAAAAATTCATGAATTTTCAT

TCTGAAATGCTCATTGCAGTAGTGTAGTGGTTGAATCAACTGAGTAGCCAAAATTATTATACTTTC

>Ceroxylon_parvum_EF128261.1

AACCCAAAAAATGAAATGGGTCCTGCCTCTGGTTCAAGTAGAAATGTAAATGGAAGAATTACAAGGATAT

TTAGAAAAAGATAGATCTCGGCAACAACACTTTCTATATCCGCTTCTCTTTAAGGAGTATATTTACACAT

TTGCTCATGATCGTGGTTTAAATGGTTCGATTTTTTACGAATCCACGGAAATTTTTTGTTATGACAATAA

ATCTAGTTCAGTACTTGTGAAACGTTCAATTATTCGAATGTATCAACAGAATTATTTGATTTATTCGGTT

AATGATTCTAACCAAAATCGATTCGTTGGGCACAACAATTATTTTTATTTTCATTTTTATTCTCAGATGA

TATTGGAAGGTTTTGCAGTCATTGTGGAAATTCCATTCTTGCTGCGATTAGTATCTTCCCTCGAAGAAAA

AAAAATACCAAAATCTAAGAATTTGAATTTACGATCTATTCATTCAATATTTCCCTTTTTGGAGGACAAA

TTATCGCATTTAAATTATGTGTCAGATATACTAATACCTTATCCCATCCATCTGAAAATCTTGGTTCAAA

TCCTTCAATGCTGGATCCAAGATGTTCCTTCTTTACATTTATTGCGATTCTTTCTTCACGAATATCATAA

TTGGAATAGTCTTATTACTCCGAATAATTCTATTTTTTTTTTTTCAAAAGAAAATAAAAGACTATTTCGG

TTCCCATATAATTCTTATGTATCTGAATGCGAATTTGTATTAGTTTTTCTTCGTAAACAATCTTCTTATT

TACGATTAACATCTTCTGGAGCTTTTCTTGAGCGAACACATTTCTATGGAAAAATAGAACATCTTATAGT

AGTGCGCCGTAATTATTTTCAGAAGACCCTATGGTTCTTCAAGGATCCCTTCATGCATTATGTTCGATAT

CAAGGAAAAGCAATTCTGGTCTCAAAGGGGACTCATCTTCTGATGAAGAAATGGAAATGTCACCTTGTCA

ATTTCTGGCAATATTATTTTCACTTTTGGTCTCAACCGTACAGGATCCATATAAACCAATTATCAAACTG

TTCTTTCTATTTTCTAGGTTATCTTTCAAGTGTACTAATAAATCCTTCGGCGGTAAGGAATCAAATGCTA

GAGAATTCATTTCTAATAGATACTGTTATTAAAAAATTCGATACCAGAGTCCCAGTTATTCCTCTTATTG

GATCATTGTCTAAAGCTAAATTTTGTACCGTATCGGGGCATCCTATTAGTAAGCCGATCTGGACCAATTT

ATCAGATTGCGATATTATTGATCGATTTGGTCGGATATGTAGAAATCTTTCTCATTATCACAGTGGATCC

TCAAAAAAACAGAGTTTGTATCGAATAAAGTATATACTTCGATTTTCGTGTGCTAGAACTTTGGCTCGTA

AACATAAAAGTATGGTACGCGCTTTTTTGCAAAGATTAGGTTCGGGATTATTAGAAGAATTCTTTACGGA

AGAAGAACAAGTTGTTTCTTTGATCTTCCCAAAAACAACTTCTTTTTCTTTACATGAATCACATATAGAA

CGTGTTTGGTATTTGGATATTATCCGTATCAATGACCTGGTGAATTATTCATAATGGGTTTGGTGACGTG

ACGAGACTTATGAATAGAATGGAAATGATCTATAAATGATCAAGAGAGAAAAAAATTCATGAATTTTCAT

TCTGAAATGCTCATTGCAGTAGTGTAGTGGTTGAATCAACTGAGTAGCCAAAATTATTATACTTTC

>Ceroxylon_parvifrons_EF128259.1

ACAACACTTTCTATATCCGCTTCTCTTTAAGGAGTATATTTACACATTTGCTCATGATCGTGGTTTAAAT

GGTTCGATTTTTTACGAATCCACGGAAATTTTTTGTTATGACAATAAATCTAGTTCAGTACTTGTGAAAC

GTTCAATTATTCGAATGTATCAACAGAATTATTTGATTTATTCGGTTAATGATTCTAACCAAAATCGATT

CGTTGGGCACAACAATTATTTTTATTTTCATTTTTATTCTCAGATGATATTGGAAGGTTTTGCAGTCATT

GTGGAAATTCCATTCTTGCYGCGATTAGKATCTTCCCTCGAAGAAAAAAAAATACCMAAATCTCAGAATT

TGAATTTACGATCTATTCATTCMATATTTCCCTTTTTGGAGGACAAATTATCGCATTTAAATTATGTGTC

AGATATACTAATACCYTATCCCATCCATCTGAAAATCTTGGTTCAAATCCTTCAATGCTGGATCCAAGAT

GTTCCTTCTTTACATTTATTGCGATTCTTTCTTCACGAATATCATAATTGGAATAGTCTTATTACTCCGA

ATAATTCTATTTTTTTTTTTTCAAAAGAAAATAAAAGACTATTTCGGTTCCCATATAATTCTTATGTATC

TGAATGCGAATTTGTATTAGTTTTTCTTCGTAAACAATCTTCTTATTTACGATTAACATCTTCTGGAGCT

TTTCTTGAGCGAACACATTTCTATGGAAAAATAGAACATCTTATAGTAGTGCGCCGTAATTATTTTCAGA

AGACCCTATGGTTCTCCAAGGATCCCTTCATGCATTATGTTCGATATCAAGGAAAAGCAATTCTGGTCTC

AAAGGGGACTCATCTTCTGATGAAGAAATGGAAATGTCACCTTGTCAATTTCTGGCAATATTATTTTCAC

TTTTGGTCTCAWCCGYACAGGATCCATATAAACCAATTATCAAACTGTTCTTTCTATTTTCTAGGTTATC

KTTCAAGTGTACTAATAAATCCTTCGGCGGTAAGGAATCAAATGCTAGAGAATTCATTTCTAATAGATAC

TGTTATTAAAAAATTCGATACCAGAGTCCCAGTTATTCCTCTTATTGGATCATTGTCTAAAGCTAAATTT

TGTACCGTATCGGGGCATCCTATTAGTAAGCCGATCTGGACCAATTTATCAGATTGCGATATTATTGATC

GATTTGGTCGGATATGTAGAAATCTTTCTCATTATCACAGTGGATCCTCAAAAAAACAGAGTTTGTATCG

AATAAAGTATATACTTCGATTTTCGTGTGCTAGAACTTTGGCTCGTAAACATAAAAGTATGGTACGCGCT

TTTTTGCAAAGATTAGGTTCGGGATTATTAGAAGAATTCTTTACGGAAGAAGAACAAGTTGTTTCTTTGA

TCTTCCCAAAAACAACTTCTTTTTCTTTACATGAATCACATATAGAACGTGTTTGGTATTTGGATATTAT

CCGTATCAATGACCTGGTGAATTATTCATAATGGGTTTGGTGACGTGACGAGACTTATGAATAGAATGGA

AATGATCTATAAATGATCAAGAGAGAAAAAAATTCATGAATTTTCATTCTGAAATGCTCATTGCAGTAGT

GTAGTGGTTGAATCAACTGAGTAGCCAAAATTATTATACTTTC

>Ceroxylon_echinulatum_EF128257.1

AACCCAAAAAATGAAATGGGTCCTGCCTCTGGTTCAAGTAGAAATGTAAATGGAAGAATTACAAGGATAT

TTAGAAAAAGATAGATCTCGGCAACAACACTTTCTATATCCGCTTCTCTTTAAGGAGTATATTTACACAT

TTGCTCATGATCGTGGTTTAAATGGTTCGATTTTTTACGAATCCACGGAAATTTTTTGTTATGACAATAA

ATCTAGTTCAGTACTTGTGAAACGTTCAATTATTCGAATGTATCAACAGAATTATTTGATTTATTCGGTT

AATGATTCTAACCAAAATCGATTCGTTGGGCACAACAATTATTTTTATTTTAATTTTTATTCTCAGATGA

TATTGGAAGGTTTTGCAGTCATTGTGGAAATTCCATTCTTGCTGCGATTAGTATCTTCCCWCGAAGAAAA

AAAAATACCAAAATCTCAGAATTTGAATTTACGATCTATTCATTCAATATTTCCCTTTTTGGAGGACAAA

TTATCGCATTTAAATTATGTGTCAGATATACTAATACCTTATCCCATCCATCTGAAAATCTTGGTTCAAA

TCCTTCAATGCTGGATCCAAGATGTTCCTTCTTTACATTTATTGCGATTCTTTCTTCACGAATATCATAA

TTGGAATAGTCTTATTACTCCGAATAATTCTATTTTTTTTTTTTCAAAAGAAAATAAAAGACTATTTCGG

TTCCCATATAATTCTTATGTATCTGAATGCGAATTTGTATTAGTTTTTCTTCGTAAACAATCTTCTTATT

TACGATTAACATCTTCTGGAGCTTTTCTTGAGCGAACACATTTCTATGGAAAAATAGAACATCTTATAGT

AGTGCGCCGTAATTATTTTCAGAAGACCCTATGGTTCTTCAAGGATCCCTTCATGCATTATGTTCGATAT

CAAGGAAAAGCAATTCTGGTCTCAAAGGGGACTCATCTTCTGATGAAGAAATGGAAATGTCACCTTGTCA

ATTTCTGGCAATATTATTTTCACTTTTGGTCTCAACCGTACAGGATCCATATAAACCAATTATCAAACTG

TTCTTTCTATTTTCTAGGTTATCTTTCAAGTGTACTAATAAATCCTTCGGCGGTAAGGAATCAAATGCTA

GAGAATTCATTTCTAATAGATACTGTTATTAAAAAATTCGATACCAGAGTCCCAGTTATTCCTCTTATTG

GATCATTGTCTAAAGCTAAATTTTGTACCGTATCGGGGCATCCTATTAGTAAGCCGATCTGGACCAATTT

ATCAGATTGCGATATTATTGATCGATTTGGTCGGATATGTAGAAATCTTTCTCATTATCACAGTGGATCC

TCAAAAAAAACAGAGTTTGTATCGAATAAAGTATATACTTCGATTTTSGTGNGCTAGAACTTNGGCTCGT

AAACATAAAAGTATGGTACGCGCTTTTTTGCAAAGATTAGGTTCGGGATTATTAGAAGAATTCTTTACGG

AAGAAGAACAAGTTGTTTCTTTGATCTTCCCAAAAACAACTTCTTTTTCTTTACATGAATCACATATAGA

ACGTGTTTGGTATTTGGATATTATCCGTATCAATGACCTGGTGAATTATTCATAATGGGTTTGGTGACGT

GACGAGACTTATGAATAGAATGGAAATGATCTATAAATGATCAAGAGAGAAAAAAATTCATGAATTTTCA

TTCTGAAATGCTCATTGCAGTAGTGTAGTGGTTGAATCAACTGAGTAGCCAAAATTATTATACTTTC

>Ceroxylon_amazonicum_EF128255.1

AACCCAAAAAATGAAATGGGTCCTGCCTCTGGTTCAAGTAGAAATGTAAATGGAAGAATTACAAGGATAT

TTAGAAAAAGATAGATCTCGGCAACAACACTTTCTATATCCGCTTCTCTTTAAGGAGTATATTTACACAT

TTGCTCATGATCGTGGTTTAAATGGTTCGATTTTTTACGAATCCACGGAAATTTTTTGTTATGACAATAA

ATCTAGTTCAGTACTTGTGAAACGTTCAATTATTCGAATGTATCAACAGAATTATTTGATTTATTCGGTT

AATGATTCTAACCAAAATCGATTCGTTGGGCACAACAATTATTTTTATTTTCATTTTTATTCTCAGATGA

TATTGGAAGGTTTTGCAGTCATTGTGGAAATTCCATTCTTGCTGCGATTAGTATCTTCCCTCGAAGAAAA

AAAAATACCAAAATCTCAGAATTTGAATTTACGATCTATTCATTCAATATTTCCCTTTTTGGAGGACAAA

TTATCGCATTTAAATTATGTGTCAGATATACTAATACCTTATCCCATCCATCTGAAAATCTTGGTTCAAA

TCCTTCAATGCTGGATCCAAGATGTTCCTTCTTTACATTTATTGCGATTCTTTCTTCACGAATATCATAA

TTGGAATAGTCTTATTACTCCGAATAATTCTATTTTTTTTTTTTCAAAAGAAAATAAAAGACTATTTCGG

TTCCCATATAATTCTTATGTATCTGAATGCGAATTTGTATTAGTTTTTCTTCGTAAACAATCTTCTTATT

TACGATTAACATCTTCTGGAGCTTTTCTTGAGCGAACACATTTCTATGGAAAAATAGAACATCTTATAGT

AGTGCGCCGTAATTATTTTCAGAAGACCCTATGGTTCTTCAAGGATCCCTTCATGCATTATGTTCGATAT

CAAGGAAAAGCAATTCTGGTCTCAAAGGGGACTCATCTTCTGATGAAGAAATGGAAATGTCACCTTGTCA

ATTTCTGGCAATATTATTTTCACTTTTGGTCTCAACCGTACAGGATCCATATAAACCAATTATCAAACTG

TTCTTTCTATTTTCTAGGTTATCTTTCAAGTGTACTAATAAATCCTTCGGCGGTAAGGAATCAAATGCTA

GAGAATTCATTTCTAATAGATACTGTTATTAAAAAATTCGATACCAGAGTCCCAGTTATTCCTCTTATTG

GATCATTGTCTAAAGCTAAATTTTGTACCGTATCGGGGCATCCTATTAGTAAGCCGATCTGGACCAATTT

ATCAGATTGCGATATTATTGATCGATTTGGTCGGATATGTAGAAATCTTTCTCATTATCACAGTGGATCC

TCAAAAAAACAGAGTTTGTATCGAATAAAGTATATACTTCGATTTTCGTGTGCTAGAACTTTGGCTCGTA

AACATAAAAGTATGGTACGCGCTTTTTTGCAAAGATTAGGTTCGGGATTATTAGAAGAATTCTTTACGGA

AGAAGAACAAGTTGTTTCTTTGATCTTCCCAAAAACAACTTCTTTTTCTTTACATGAATCACATATAGAA

CGTGTTTGGTATTTGGATATTATCCGTATCAATGACCTGGTGAATTATTCATAATGGGTTTGGTGACGTG

ACGAGACTTATGAATAGAATGGAAATGATCTATAAATGATCAAGAGAGAAAAAAATTCATGAATTTTCAT

TCTGAAATGCTCATTGCAGTAGTGTAGTGGTTGAATCAACTGAGTAGCCAAAATTATTATACTTTC

>Ceroxylon_alpinum_subsp.ecuadorense_EF128253.1

AACCCAAAAAATGAAATGGGTCCTGCCTCTGGTTCAAGTAGAAATGTAAATGGAAGAATTACAAGGATAT

TTAGAAAAAGATAGATCTCGGCAACAACACTTTCTATATCCGCTTCTCTTTAAGGAGTATATTTACACAT

TTGCTCATGATCGTGGTTTAAATGGTTCGATTTTTTACGAATCCACGGAAATTTTTTGTTATGACAATAA

ATCTAGTTCAGTACTTGTGAAACGTTCAATTATTCGAATGTATCAACAGAATTATTTGATTTATTCGGTT

AATGATTCTAACCAAAATCGATTCGTTGGGCACAACAATTATTTTTATTTTAATTTTTATTCTCAGATGA

TATTGGAAGGTTTTGCAGTCATTGTGGAAATTCCATTCTTGCTGCGATTAGTATCTTCCCTCGAAGAAAA

AAAAATACCAAAATCTCAGAATTTGAATTTACGATCTATTCATTCAATATTTCCCTTTTTGGAGGACAAA

TTATCGCATTTAAATTATGTGTCAGATATACTAATACCTTATCCCATCCATCTGAAAATCTTGGTTCAAA

TCCTTCAATGCTGGATCCAAGATGTTCCTTCTTTACATTTATTGCGATTCTTTCTTCACGAATATCATAA

TTGGAATAGTCTTATTACTCCGAATAATTCTATTTTTTTTTTTTCAAAAGAAAATAAAAGACTATTTCGG

TTCCCATATAATTCTTATGTATCTGAATGCGAATTTGTATTAGTTTTTCTTCGTAAACAATCTTCTTATT

TACGATTAACATCTTCTGGAGCTTTTCTTGAGCGAACACATTTCTATGGAAAAATAGAACATCTTATAGT

AGTGCGCCGTAATTATTTTCAGAAGACCCTAGGTTCCNCCAAGGATCCCNTCATGCATTATGTTCGATAT

CAAGGAAAAAGCAATTCTGGTCTCAAAGGGGACTCATCTTCTGATGAAGAAATGGAAATGTCACCTTGTC

AATTTCTGGCAATATTATTTTCACTTTTGGTSTCAACCGTACAGGATCCATATAAACCAATTATCAAACT

GTTCTTTCTATTTTMTAGGTTATCTTTCAAGTGTACTAATAAATCCTTCGGCGGTAAGGAATCAAATGCT

AGAGAATTCATTTCTAATAGATACTGTTATTAAAAAATTCGATACCAGAGTCCCAGTTATTCCTCTTATT

GGATCATTGTCTAAAGCTAAATTTTGTACCGTATCGGGGCATCCTATTAGTAAGCCGATCTGGACCAATT

TATCAGATTGCGATATTATTGATCGATTTGGTCGGATATGTAGAAATCTTTCTCATTATCACAGTGGATC

CTCAAAAAAACAGAGTTTGTATCGAATAAAGTATATACTTCGATTTTCGTGTGCTAGAACTTTGGCTCGT

AAACATAAAAGTATGGTACGCGCTTTTTTGCAAAGATTAGGTTCGGGATTATTAGAAGAATTCTTTACGG

AAGAAGAACAAGTTGTTTCTTTGATCTTCCCAAAAACAACTTCTTTTTCTTTACATGAATCACATATAGA

ACGTGTTTGGTATTTGGATATTATCCGTATCAATGACCTGGTGAATTATTCATAATGGGTTTGGTGACGT

GACGAGACTTATGAATAGAATGGAAATGATCTATAAATGATCAAGAGAGAAAAAAATTCATGAATTTTCA

TTCTGAAATGCTCATTGCAGTAGTGTAGTGGTTGAATCAACTGAGTAGCCAAAATTATTATACTTTC

>Ceroxylon_quindiuense_AM114607.1

GACCATATTGCACTATGTATCATTTGATAACCCAAAAAATGAAATGGGTCCTGCCTCTGGTTCAAGTAGA

AATGTAAATGGAAGAATTACAAGGATATTTAGAAAAAGATAGATCTCGGCAACAACACTTTCTATATCCG

CTTCTCTTTAAGGAGTATATTTACACATTTGCTCATGATCGTGGTTTAAATGGTTCGATTTTTTACGAAT

CCACGGAAATTTTTTGTTATGACAATAAATCTAGTTCAGTACTTGTGAAACGTTCAATTATTCGAATGTA

TCAACAGAATTATTTGATTTATTCGGTTAATGATTCTAACCAAAATCGATTCGTTGGGCACAACAATTAT

TTTTATTTTCATTTTTATTCTCAGATGATATTGGAAGGTTTTGCAGTCATTGTGGAAATTCCATTCTTGC

TGCGATTAGTATCTTCCCTCGAAGAAAAAAAAATACCAAAATCTCAGAATTTGAATTTACGATCTATTCA

TTCAATATTTCCCTTTTTGGAGGACAAATTATCGCATTTAAATTATGTGTCAGATATACTAATACCTTAT

CCCATCCATCTGAAAATCTTGGTTCAAATCCTTCAATGCTGGATCCAAGATGTTCCTTCTTTACATTTAT

TGCGATTCTTTCTTCACGAATATCATAATTGGAATAGTCTTATTACTCCGAATAATTCTATTTTTTTTTT

TTCAAAAGAAAATAAAAGACTATTTCGGTTCCCATATAATTCTTATGTATCTGAATGCGAATTTGTATTA

GTTTTTCTTCGTAAACAATCTTCTTATTTACGATTAACATCTTCTGGAGCTTTTCTTGAGCGAACACATT

TCTATGGAAAAATAGAACATCTTATAGTAGTGCGCCGTAATTATTTTCAGAAGACCCTATGGTTCTTCAA

GGATCCCTTCATGCATTATGTTCGATATCAAGGAAAAGCAATTATGGTTTCAAAGGGGACTCATCTTCTG

ATGAAGAAATGGAAATGTCACCTTGTCAATTTCTGGCAATATTATTTTCACTTTTGGTCTCAACCGTACA

GGATCCATATAAACCAATTATCAAACTGTTCTTTCTATTTTCTAGGTTATCTTTCAAGTGTACTAATAAA

TCCTTCGGCGGTAAGGAATCAAATGCTAGAGAATTCATTTCTAATAGATACTGTTATTAAAAAATTCGAT

ACCAGAGTCCCAGTTATTCCTCTTATTGGATCATTGTCTAAAGCTAAATTTTGTACCGTATCGGGGCATC

CTATTAGTAAGCCGATCTGGACCAATTTATCAGATTGCGATATTATTGATCGATTTGGTCGGATATGTAG

AAATCTTTCTCATTATCACAGTGGATCCTCAAAAAAACAGAGTTTGTATCGAATAAAGTATATACTTCGA

TTTTCGTGTGCTAGAACTTTGGCTCGTAAACATAAAAGTATGGTACGCGCTTTTTTGCAAAGATTAGGTT

CGGGATTATTAGAAGAATTCTTTACGGAAGAAGAACAAGTTGTTTCTTTGATCTTCCCAAAAACAACTTC

TTTTTCTTTACATGAATCACATATAGAACGTGTTTGGTATTTGGATATTATCCGTATCAATGACCTGGTG

AATTATTCATAATGGGTTTGGTGACGTGACGAGACTTATGAATAGAATGGAAATGATCTATAAATGATCA

AGAGAGAAAAAAATTCATGAATTTTCATTCTGAAATGCTCATTGCAGTAGTGTAGTGGTTGAATCAACTG

AGTAGCCAAAATTATTATACTTTCTTCTCGGGACCCAAGTTTTATATTATACATAGGTAAAGTC

>Chamaedorea_seifrizii_HQ180858.1

ATGGAAGAATTACAAGGATATTTAGAAAAAGATAGATCTCGGCAACAACACTTTCTATATCCGCTTCTCT

TTAAGGAGTATATTTACACATTTGCTCATGATCGTGGTTTAAATGGTTCGATTTTTTACGAATCCACGGA

AATTTTTGGTTATGACAATAAATCTAGTTCAGTACTTGTGAAACGTTCAATTATTCGAATGTATCAACAG

AATTTTTTGATTTATTCGGTTAATGATTCTAACCAAAATCGATTCGTTGGGCACAATTATTTTTTTTATT

TTCATTTTTATTCTCAGATGATATTGGAAGGTTTTGCAGTCATTGTGGAAATTCCATTCTTGCTGCGATT

AGTATCTTCTCTCGAAGAAAAAAAAATACCCAAATTTCAGAATTTGAATTTACGATCTATTCATTCAATA

TTTCCCTTTTTGGAGGACAAATTATCACATTTAAATTATGTGTCAGATATACTAATACCTTATCCCATCC

ATCTGAAAATCTTGGTTCAAATCCTTCAATGCTGGATCCAAGATGTTCCTTCTTTACATTTATTGCGATT

CTTTCTTCACGAATATCATAATTGGAATAGGAATAATTCGATTTTTTTTTCAAAAGAAAATAAAAGACTA

TTTCGGTTCCCATATAATTATTATGTATCTGAATGCGAATTTGTATTAGTTTTTCTTCGTAAACAATCTT

CTTATTTACGATTAACATCTTCTGGAGCTTTTCTTGAGCGAACACATTTCTATGGAAAGATAGAACATCT

TATAGTAGTGCGCCGTAATTATTTTCAGAGGACCCTATGGTTCTTCAAGGATCCCTTCATGCATTATGTT

CGATATCAAGGAAAAGCAATTATGGTTTCAAAGGGGACTCATCTTCTGATGAAGAAATGGAAATGTCACC

TTGTCCATTTCTGGCAATATTATTTTCATTTTTGGTCTCAACCGTACAGGATCCATATAAACCAATTATC

AAGCTGTTCTTTCCATTTTCTAGGTTATCTTTCAAGTGTACTAATAAATCCTTCGGCGGTAAGGAATCAA

ATGCTAGAGAATTCATTTCTAATGGATACTGTTATTAAAAAATTCGATACCAGAGTCCCAGTTATTCCTC

TTATTGGATCATTGTCTAAAGCTAAATTTTGTACCGTATCGGGGCATCCTATTAGTAAGCCGATCTGGAC

CAATTTATCAGATTGTGATATTATTGATCGATTTGGTCGGATATGTAGAAATCTTTCTCATTATCACAGT

GGATCCTCAAAAAAACAGAGTTTGTATCGAATAAAGTATATACTTCGATTTTCGTGTGCTAGAACTTTGG

CTCGTAAACATAAAAGTACGGTACGCACTTTTTTGCAAAGATTAGGTTCGGGATTATTAGAAGAATTCTT

TACGGAAGAAGAACAAGTTGTTTCTTTGATCTTCCCAAAAGCAACTTCTTTTTCTTTACATGAATCGCAT

ATAGAACGTATTTGGTATTTGGATATTATCCGTATCAATAACCTGGTGAATTATTCATAA

>Chamaedorea_seifrizii_GU135024.1

ATTTAAATTATGTGTCAGATATACTAATACCTTATCCCATCCATCTGAAAATCTTGGTTCAAATCCTTCA

ATGCTGGATCCAAGATGTTCCTTCTTTACATTTATTGCGATTCTTTCTTCACGAATATCATAATTGGAAT

AGGAATAATTCGATTTTTTTTTCAAAAGAAAATAAAAGACTATTTCGGTTCCCATATAATTATTATGTAT

CTGAATGCGAATTTGTATTAGTTTTTCTTCGTAAACAATCTTCTTATTTACGATTAACATCTTCTGGAGC

TTTTCTTGAGCGAACACATTTCTATGGAAAGATAGAACATCTTATAGTAGTGCGCCGTAATTATTTTCAG

AGGACCCTATGGTTCTTCAAGGATCCCTTCATGCATTATGTTCGATATCAAGGAAAAGCAATTATGGTTT

CAAAGGGGACTCATCTTCTGATGAAGAAATGGAAATGTCACCTTGTCCATTTCTGGCAATATTATTTTCA

TTTTTGGTCTCAACCGTACAGGATCCATATAAACCAATTATCAAGCTGTTCTTTCCATTTTCTAGGTTAT

CTTTCAAGTGTACTAATAAATCCTTCGGCGGTAAGGAATCAAATGCTAGAGAATTCATTTCTAATGGATA

CTGTTATTAAAAAATTCGATACCAGAGTCCCAGTTATTCCTCTTATTGGATCATTGTCTAAAGCTAAATT

TTGTACCGTATCGGGGCATCCTATTAGTAAGCCGATCTGGACCAATTTATCAGATTGTGATATTATTGAT

CGATTTGGTCGGATATGTAGAAATCTTTCTCATTAT

>Chamaedorea_tepejilote_GQ981962.1

AAGATGTTCCTTCTTTACATTTATTGCGATTCTTTCTTCACGAATATCATAATTGGAATAGGAATAATTC

TATTTTTTTTTTTTCAAAAAAAAATAAAAAACTATTTCGGTTCCCATATAATTATTATGTATCTGAAGGC

GAATTTGTATTAGTTTTTCTTCGTAAACAATCTTCTTATTTACRATTAACATCTTCGGGAGCTTTTCTTG

AGCGAACACWTTTCTATGGAAAAATARAACATCTTATAGWAGKGCGCCGWAATTATTTTCARAGGACCCT

ATGGTTCTTCAAGGATCCCTTCATGCATTATGTTCRATATCAAGGAAAAGCAATTCTGGTTTCAAAGGGR

ACTCATCTTCTGATGAARAAAKGGAAAWRWMACCTTGTCCATTTCKGGCAWTWTTATTTTCACTTTTGGT

CTCAACCGTACAGGATCCATATAAACCAATTATCAAGCTGTTCTTTCCATTTTCWAGGTTATCTTTCAAG

TGTACTAATAAATCCTTCGGCGGTAAGGAATCAAATGCTAGAGAATTCATTTCTAATGGATACTGTTATT

AAAAAATTCGATACCAGAGTCCCAGTTATTCCTCTTATTGGATCATTGTCTAAAGCTAAATTTTGTACCG

TATCGGGGCATCCTATTAGTAAGCCGATCTGGACCAATTTATCAGATTGTGATATTATTGATCGATTTGG

TCGGATATGTAGAAATCTTTCTCATTATCACAGTGGATCCTCAAAAAAACCAGAGTTTGTATCGAATAAA

>Chamaedorea_tenella_DQ401368.1

CGTGGTTTAAATGGTTCAATTTTTTACGAATCCACGGAAATTTTTGGTTATGACAATAAATCTAGTTCAA

TACTTGTGAAACGTTCAATTATTCGAATGTATCAACAGAATTATTTGATTTATTCGGTTAATGATTCTAA

CCAAAATCGATTCGTTGGGCACAACCATTTTTTTTATTTTCATTTTTATTCTCAGATGATATTGGAAGGT

TTTGCAGTCATTGTGGAAATTCCATTCTTGCTGCGATTAGTATCTTCTCTCGAAAAAAAAAAAATACCAA

AATATCAGAATTTGAATTTACGATCTATTCATTCAATATTTCCCTTTTTGGAGGACAAATTATCGCATTT

AAATTATGTGTCAGATATACTAATACCTTATCCCATCCATCTGAAAATCTTGGTTCCAATCCTTCAATGC

TGGATCCAAGATGTTCCTTCTTTACATTTATTGCGATTCTTTCTTCACGAATATCATAATTGGAATAGGA

ATAATTCTATTTTTTTTTCAAAAGAAAATAAAAGACTATTTCGGTTCCCATATAATTATTATGTATCTGA

ATGCGAATTTTTATTAGTTTTTCTTCGTAAACAATCCTCTTATTTACGATTAACATCTTCTGGAGCTTTT

CTTGAGCGAACACATTTCTATGGAAAAATAGAACATCTTATAGTAGTGCGCCGTAATTATTTTCAGAGGA

CCCTATGGTTCTTCAAGGATCCCTTCATGCATTATGTTCGATATCAAGGAAAAGCAATTCTGGTTTCAAA

GGGGACTCATCTTCTGATGAAGAAATGGAAATGTCACCTTGTCCATTTCTGGCAATATTATTTTCACTTT

TGGTCTCAACCGTACAGGATCCATATAAACCAATTATCAAGCTGTTCTTTCCATTTTCTAGGTTATCTTT

CAAGTGTACTAATAAATCCTTCGGCGGTAAGGAATCAAATGCTAGAGAATTCATTTCTAATGGATACTGT

TATTAAAAAATTCGATACCAGAGTCCCAGTTATTCCTCTTATTGGATCATTGTCTAAAGCTAAATTTTGT

ACCGTATCGGGGCATCCTATTAGTAAGCCGATCTGGACCAATTTATCAGATTGTGATATTATTGATCGAT

TTGGTCGGATATGTAGAAATCTTTCTCATTATCACAGTGGATCCTCAAAAAAACAGAGTTTGTATCGAAT

CAAGTATATACTTCGATTTTCGTGTGCTAGAACTTTGGCTCGTAAACATAAAAGTACGGTACGCACTTTT

TTGCAAAGATTAGGTTCGGAATTATTAGAAGAATTCTTTACGGAAGAAGAACAAGTTGTTTCTTTGATCT

TCCCAAAAGCAACTTCTTTTTCTTTACATGAATCACATATAGAACGTATTTGGTCTTTGGA

>Chamaedorea_tepejilote_DQ178691.1

CGTTCTGACCATATTGCACTATGTATCATTTGATAACCCCAAAAATGAAATGTGTCCTGCCTCTGGTTCA

AGTAGAAATGTAAATGGAAGAATTACAAGGATATTTAGAAAAAGATAAATCTCGGCAACAACACTTTCTA

TATCCGCTTCTCTTTAAGGAGTATATTTACACATTTGCTCATGATCGTGGTTTAAATGGTTCGATTTTTT

ACGAATCCACGGAAATTTTTGGTTATGACAATAAATCTAGTTCAGTACTTGTGAAACGTTCAATTATTCG

AATGTATCAACAGAATTATTTGATTTATTCGGTTAATGATTCTAACCAAAATCGATTCGTTGGGCACAAC

CATTTTTTTTATTTTTATTTTTATTCTCAGATGATATTGGAAGGTTTTGCAGTCATTGTGGAAATTCCAT

TCTTGCTGCGATTAGTATCCTCTCTCGAAGAAAAAAAAATACCAAAATATCATAATTTAAATTTACGATC

TATTCATTCAATATTTCCCTTTTTGGAGGACAAATTATCACATTTAAATTATGTGTCAGATATACTAATA

CCTTATCCCATCCATCTGAAAATCTTGGTTCAAATCCTTCAATGCTGGATCCAAGATGTTCCTTCTTTAC

ATTTATTGCGATTCTTTCTTCACGAATATCATAATTGGAATAGGAATAATTCTATTTTTTTTTCAAAAGA

AAATAAAAGACTATTTCGGTTCCCATATAATTATTATGTATCTGAATGCGAATTTGTATTAGTTTTTCTT

CGTAAACAATCTTCTTATTTACGATTAACATCTTCTGGAGCTTTTCTTGAGCGAACACATTTCTATGGAA

AAATAGAACATCTTATAGTAGTGCGCCGTAATTATTTTCAGAGGACCCTATGGTTCTTCAAGGATCCCTT

CATGCATTATGTTCGATATCAAGGAAAAGCAATTCTGGTTTCAAAGGGGACTCATCTTCTGATGAAGAAA

TGGAAATGTCACCTTGTCCATTTCTGGCAATATTATTTTCACTTTTGGTCTCAACCGTACAGGATCCATA

TAAACCAATTATCAAGCTGTTCTTTCCATTTTCTAGGTTATCTTTCAAGTGTACTAATAAATCCTTCGGC

GGTAAGGAATCAAATGCTAGAGAATTCATTTCTAATGGATACTGTTATTAAAAAATTCGATACCAGAGTC

CCAGTTATTCCTCTTATTGGATCATTGTCTAAAGCTAAATTTTGTACCGTATCGGGGCATCCTATTAGTA

AGCCGATCTGGACCAATTTATCAGATTGTGATATTATTGATCGATTTGGTCGGATATGTAGAAATCTTTC

TCATTATCACAGTGGATCCTCAAAAAAACAGAGTTTGTATCGAATAAAGTATATACTTCGATTTTCGTGT

GCTAGAACTTTGGCTCGTAAACATAAAAGTACGGTACGCACTTTTTTGCAAAGATTAGGTTCGGGATTAT

TAGAAGAATTCTTTACGGAAGAAGAACAAGTTGTTTCTTTGATCTTCCAAAAAGCAACTTCTTTTTCTTT

ACATGAATCACATATAGAACGTATTTGGTATTTGGATATTATCCGTATCAATGACCTGGTGAATTATTCA

TAATAGGTTTGTTTGGTGACGTGATGAGACTTATGAATAGTCTGGAAATGATCTATAAATGATCAAGAGA

GAAAAAAATTCATGAATTTTCATTCTGAAATGCTCATTGCAGTAGTGTAGTGGTTGAATCAACTGAGTAG

TCAAAATTATTATACTTTCTTCTCGGGACCCAAGTTTTCTATTATACATAGGTAAAGTCGTGTGCAATG

>Chamaedorea_seifrizii_DQ178689.1

CGTTCTGACCATATTGCACTATGTATCATTTGATAACCCCAAAAATGAAATGGGTCCTGCCTCTGGTTCA

AGTAGAAATGTAAATGGAAGAATTACAAGGATATTTAGAAAAAGATAGATCTCGGCAACAACACTTTCTA

TATCCGCTTCTCTTTAAGGAGTATATTTACACATTTGCTCATGATCGTGGTTTAAATGGTTCGATTTTTT

ACGAATCCACGGAAATTTTTGGTTATGACAATAAATCTAGTTCAGTACTTGTGAAACGTTCAATTATTCG

AATGTATCAACAGAATTTTTTGATTTATTCGGTTAATGATTCTAACCAAAATCGATTCGTTGGGCACAAT

TATTTTTTTTATTTTCATTTTTATTCTCAGATGATATTGGAAGGTTTTGCAGTCATTGTGGAAATTCCAT

TCTTGCTGCGATTAGTATCTTCTCTCGAAGAAAAAAAAATACCCAAATTTCAGAATTTGAATTTACGATC

TATTCATTCAATATTTCCCTTTTTGGAGGACAAATTATCACATTTAAATTATGTGTCAGATATACTAATA

CCTTATCCCATCCATCTGAAAATCTTGGTTCAAATCCTTCAATGCTGGATCCAAGATGTTCCTTCTTTAC

ATTTATTGCGATTCTTTCTTCACGAATATCATAATTGGAATAGGAATAATTCGATTTTTTTTTCAAAAGA

AAATAAAAGACTATTTCGGTTCCCATATAATTATTATGTATCTGAATGCGAATTTGTATTAGTTTTTCTT

CGTAAACAATCTTCTTATTTACGATTAACATCTTCTGGAGCTTTTCTTGAGCGAACACATTTCTATGGAA

AGATAGAACATCTTATAGTAGTGCGCCGTAATTATTTTCAGAGGACCCTATGGTTCTTCAAGGATCCCTT

CATGCATTATGTTCGATATCAAGGAAAAGCAATTATGGTTTCAAAGGGGACTCATCTTCTGATGAAGAAA

TGGAAATGTCACCTTGTCCATTTCTGGCAATATTATTTTCATTTTTGGTCTCAACCGTACAGGATCCATA

TAAACCAATTATCAAGCTGTTCTTTCCATTTTCTAGGTTATCTTTCAAGTGTACTAATAAATCCTTCGGC

GGTAAGGAATCAAATGCTAGAGAATTCATTTCTAATGGATACTGTTATTAAAAAATTCGATACCAGAGTC

CCAGTTATTCCTCTTATTGGATCATTGTCTAAAGCTAAATTTTGTACCGTATCGGGGCATCCTATTAGTA

AGCCGATCTGGACCAATTTATCAGATTGTGATATTATTGATCGATTTGGTCGGATATGTAGAAATCTTTC

TCATTATCACAGTGGATCCTCAAAAAAACAGAGTTTGTATCGAATAAAGTATATACTTCGATTTTCGTGT

GCTAGAACTTTGGCTCGTAAACATAAAAGTACGGTACGCACTTTTTTGCAAAGATTAGGTTCGGGATTAT

TAGAAGAATTCTTTACGGAAGAAGAACAAGTTGTTTCTTTGATCTTCCCAAAAGCAACTTCTTTTTCTTT

ACATGAATCGCATATAGAACGTATTTGGTATTTGGATATTATCCGTATCAATAACCTGGTGAATTATTCA

TAATAGGTTTGTTTGGTGACGTGATGAGACTTATGAATAGTCTGGAAATGATCTATAAATGATCAAGAGA

GAAAAAAATTCATGAATTTTCATTCTGAAATGCTCATTGCAGTAGTGTAGTGGTTGAATCAACTGAGTAG

TCAAAATTATTATACTTTCTTCTCGGGACCCAAGTTTTATATTATACATAGGTAAAGTCGTGTGCAATG

>Chamaedorea_pochutlensis_DQ178687.1

CGTTCTGACCATATTGCACTATGTATCATTTGATAACCCCAAAAATGAAATGGGTCCTGCCTCTGGTTCA

AGTAGAAATGTAAATGGAAGAATTACAAGGATATTTAGAAAAAGATAGATCTCGGCAACAACACTTTCTA

TATCCGCTTCTCTTTAAGGAGTATATTTACACATTTGCTCATGATCGTGGTTTAAATGGTTCGATTTTTT

ACGAATCCACGGAAATTTTTGGTTATGACAAGAAATCTAGTTCAGTACTTGTGAAACGTTCAATTATTCG

AATGTATCAACAGAATTATTTGATTTATTCGGTTAATGATTCTAACCAAAATCGATTCGTTGGGCACAAC

AATTTTTTTTATTTTCATTTTTATTCTCAGATGATATTGGAAGGTTTTGCAGTCATTGTGGAAATTCCAT

TCTTGCTGCGATTAGTATCTTCTCTCGAAGAAAAAAAAATACCAAAATATCAGAATTTGAATTTACGATC

TATTCATTCAATATTTCCCTTTTTGGAGGACAAATTATCACATTTAAATTATGTGTCAGATATACTAATA

CCTTATCCCATCCATCTGAAAATCTTGGTTCAAATCCTTCAATGCTGGATCCAAGATGTTCCTTCTTTAC

ATTTATTGCGATTCTTTCTTCACGAATATCATAATTGGAATAGGAATAATTCTATTTTTTTTTCAAAAGA

AAATAAAAGACTATTTCGGTTCCCATATAATTATTATGTATCTGAATGCGAATTTGTATTAGTTTTTCTT

CGTAAACAATCTTCTTATTTACGATTAACATCTTCTGGAGCTTTTCTTGAGCGAACACATTTCTATGGAA

AAATAGAACATCTTATAGTAGTGCGCCGTAATTATTTTCAGAGGACCCTATGGTTCTTCAAGGATCCCTT

CATGCATTATGTTCGATATCAAGGAAAAGCAATTCTGGTTTCAAAGGGGACTCATCTTCTGATGAAGAAA

TGGAAATGTCACCTTGTCCATTTCTGGCAATATTATTTTCACTTTTGGTCTCAACCGTACAGGATCCATA

TAAACCAATTATCAAGCTGTTCTTTCCATTTTCTAGGTTATCTTTCAAGTGTACTAATAAATCCTTCGGC

GGTAAGGAATCAAATGCTAGAGAATTCATTTCTAATGGATACTGTTATTAAAAAATTCGATACCAGAGTC

TCAGTTATTCCTCTTATTGGATCATTGTCTAAAGCTAAATTTTGTACCGTATCGGGGCATCCTATTAGTA

AGCCGATCTGGACCAATTTATCAGATTGTGATATTATTGATCGATTTGGTCGGATATGTAGAAATCTTTC

TCATTATCACAGTGGATCCTCAAAAAAACAGAGTTTGTATCGAATAAAGTATATACTTCGATTTTCGTGT

GCTAGAACTTTGGCTCGTAAACATAAAAGTACGGTACGCACTTTTTTGCAAAGATTAGGTTCGGGATTAT

TAGAAGAATTCTTTACGGAAGAAGAACAAGTTGTTTCTTTGATCTTCCCAAAAGCAACTTCTTTTTCTTT

ACATGAATCACATATAGAACGTATTTGGTATTTTGATATTATCCGTATCAATGACCTGGTGAATTATTCA

TAATAGGTTTGTTTGGTGACGTGATGAGACTTATGAATAGTCTGGAAATGATCTATAAATGATCAAGAGA

GAAAAAAATTCATGAATTTTCATTCTGAAATGCTCATTGCAGTAGTGTAGTGGTTGAATCAACTGAGTAG

TCAAAATTATTATACTTTCTTCTCGGGACCCAAGTTTTATATTATACATAGGTAAAGTCGTGTGCAATG

>Chamaedorea_pinnatifrons_DQ178685.1

CGTTCTGACCATATTGCACTATGTATCATTTGATAACCCCAAAAATGAAATGGGTCCTGCCTCTGGTTCA

AGTAGAAATGTAAATGGAAGAATTACAAGGATATTTAGAAAAAGATAGATCTCGGCAACAACACTTTCTA

TATCCGCTTCTCTTTAAGGAGTATATTTACACATTTGCTCATGATCGTGGTTCAATTTTTTACGAATCCA

CGGAAATTTTTGGTTATGACAATAAATCTAGTTCAGTACTTGTGAAACGTTCAATTATTCGAATGTATCA

ACAGAATTATTTGATTTATTCGGTTAATGATTCTAACCAAAATCGATTCGTTGGGCACAACAATTTTTTT

TATTTTCATTTTTATTCTCAGATGATATTGGAAGGTTTTGCAGTCATTGTGGAAATTCCATTCTTGCTGC

GATTAGTATCTTCTCTCGAAGAAAAAAAAATACCAAAATATCAGAATTTGAATTTACGATCTATTCATTC

AATATTTCCCTTTTTGGAGGACAAATTATCACATTTAAATTATGTGTCAGATATACTAATACCTTATCCC

ATCCATCTGAAAATCTTGGTTCAAATCCTTCAATGCTGGATCCAAGATGTTCCTTCTTTACATTTATTGC

GATTCTTTCTTCACGAATATCATAATTGGAATAGGAATAATTCTATTTTTTTTTCAAAAGAAAATAAAAG

ACTATTTCGGTTCCCATATAATTATTATGTATCTGAATGCGAATTTGTATTAGTTTTTCTTCGTAAACAA

TCTTCTTATTTACGATTAACATCTTCTGGAGCTTTTCTTGAGCGAACACATTTCTATGGAAAAATAGAAC

ATCTTATAGTAGTGCGCCGTAATTATTTTCAGAGGACCCTATGGTTCTTCAAGGATCCCTTCATGCATTA

TGTTCGATATCAAGGAAAAGCAATTCTGGTTTCAAAGGGGACTCATCTTCTGATGAAGAAATGGAAATGT

CACCTTGTCCATTTCTGGCAATATTATTTTCACTTTTGGTCTCAACCGTACAGGATCCATATAAACCAAT

TATCAAGCTGTTCTTTCCATTTTCTAGGTTATCTTTCAAGTGTACTAATAAATCCTTCGGCGGTAAGGAA

TCAAATGCTAGAGAATTCATTTCTAATGGATACTGTTATTAAAAAATTCGATACCAGAGTCCCAGTTATT

CCTCTTATTGGATCATTGTCTAAAGCTAAATTTTGTACCGTATCGAGGCATCCTATTAGTAAGCCGATCT

GGACCAATTTATCAGATTGTGATATTATTGATCGATTTGGTCGGATATGTAGAAATCTTTCTCATTATCA

CAGTGGATCCTCAAAAAAACAGAGTTTGTATCGAATAAAGTATATANTTCGATTTTTGTGTGCTAGAACT

CTGGCTCGTAAACATAAAAGTACGGTACGCACTTTTTTGCAAAGATTAGGTTCGGGATTATTAGAAGAAT

TCTTTACGGAAGAAGAACAAGTTGTTTCTTTGATCTTCCCAAAAGCAACTTCTTTTTCTTTACATGAATC

ACATATAGAACGTATTTGGTATTTGGATATTATCCGTATCAATGACCTGGTGAATTATTCATAATAGGTT

TGTTTGGTGACGTGATGAGACTTATGAATAGTCTGGAAATGATCTATAAATGATCAAGAGAGAAAAAAAT

TCATGAATTTTCATTCTGAAATGCTCATTGCAGTAGTGTAGTGGTTGAATCAACTGAGTAGTCAAAATTA

TTATACTTTCTTCTCGGGACCCAAGTTTTCTATTATACATAGGTAAAGTCGTGTGCAATG

>Chamaedorea_oblongata_DQ178683.1

CGTTCTGACCATATTGCACTATGTATCATTTGATAACCCCAAAAATGAAATGGGTCCTGCCTCTGGTTCA

AGTAGAAATGTAAATGGAAGAATTACAAGGATATTTAGAAAAAGATAGATCTCGGCAACAACACTTTCTA

TATCCGCTTCTCTTTAAGGAGTATATTTACACATTTGCTCATGATCGTGGTTTAAATGGTTCGATTTTTT

ACGAATCCACGGAAATTTTTGGTTATGACAATAAATCTAGTTCAGTACTTGTGAAACGTTCAATTATTCG

AATGTATCAACAGAATTATTTGATTTATTCGGTTAATGATTCTAACCAAAATCGATTCGTTGGGCACAAC

AATTTTTTTTATTTTCATTTTTATTCTCAGATGATATTGGAAGGTTTTGCAGTCATTGTGGAAATTCCAT

TCTTGCTGCGATTAGTATCTTCTCTCGAAGAAAAAAAAATACCAAAATATCAGAATTTGAATTTACGATC

TATTCATTCAATATTTCCCTTTTTGGAGGACAAATTATCACATTTAAATTATGTGTCAGATATACTAATA

CCTTATCCCATCCATCTGAAAATCTTGGTTCAAATCCTTCAATGCTGGATCCAAGATGTTCCTTCTTTAC

ATTTATTGCGATTCTTTCTTCACGAATATCATAATTGGAATAGGAATAATTCTATTTTTTTTTTTTCAAA

AGAAAATAAAAGACTATTTCGGTTCCCATATAATTATTATGTATCTGAATGCGAATTTGTATTAGTTTTT

CTTCGTAAACAATCTTCTTATTTACGATTAACATCTTCTGGAGCTTTTCTTGAGCGAACACATTTCTATG

GAAAAATAGAACATCTTATAGTAGTGCGCCGTAATTATTTTCAGAGGACCCTATGGGTCTTCAAGGATCC

CTTCATGCATTATGTTCGATATCAAGGAAAAGCAATTCTGGTTTCAAAGGGGACTCATCTTCTGATGAAG

AAATGGAAATGTCACCTTGTCCATTTCTGGCAATATTATTTTCATTTTTGGTCTCAACCGTACAGGATCC

ATATAAACCAATTATCAAGCTGTTCTTTCCATTTTCTAGGTTATCTTTCAAGTGTACTAATAAATCCTTC

GGCGGTAAGGAATCAAATGCTAGAGAATTCATTTCTAATGGATACTGTTATTAAAAAATTCGATACCAGA

GTCCCAGTTATTCCTCTTATTGGATCATTGTCTAAAGCTAAATTTTGTACCGTATCGGGGCATCCTATTA

GTAAGCCGATCTGGACCAATTTATCAGATTGTGATATTATTGATCGATTTGGTCGGATATGTAGAAATCT

TTCTCATTATCACAGTGGATCCTCAAAAAAACAGAGTTTGTATCGAATAAAGTATATACTTCGATTTTCG

TGTGCTAGAACTTTGGCTCGTAAACATAAAAGTACGGTACGCACTTTTTTGCAAAGATTAGGTTCGGGAT

TATTAGAAGAATTCTTTACGGAAGAAGAACAAGTTCTTTCTTTGATCTTCCCAAAAGCAACTTCTTTTTC

TTTACATGAATCACATATAGAACGTATTTGGTATTTGGATATTATCCGTATCAATGACCTGGTGAATTAT

TCATAATAGGTTTGTTTGGTGACGTGATGAGACTTATGAATAGTCTGGAAATGATCTATAAATGATCAAG

AGAGAAAAAAATTCATGAATTTTCATTCTGAAATGCTCATTGCAGTAGTGTAGTGGTTGAATCAACTGAG

TAGTCAAAATTATTATACTTTCTTCTCGGGACCAAAGTTTTATATTATACATAGGTAAAGTCGTGTGCAA

TG

>Chamaedorea_nationsiana_DQ178681.1

CGTTCTGACCATATTGCACTATGTATCATTTGATAACCCCAAAAATGAAATGGGTCCTGCCTCTGGTTCA

AGTAGAAATGTAAATGGAAGAATTACAAGGATATTTAGAAAAAGATAGATCTCGGCAACAACACTTTCTA

TATCCGCTTCTCTTTAAGGAGTATATTTACACATTTGCTCATGATCGTGGTTTAAATGGTTCGATTTTTT

ACGAATCCACGGAAATTTTTGGTTATGACAATAAATCTAGTTCAGTACTTGTGAAACGTTCAATTATTCG

AATGTATCAACAGAATTATTTGATTTATTCGGTTAATGATTCTAACCAAAATCGATTAGTTGGGCACAAC

CATTTTTTTTATTTTCATTTTTATTCTCAGATGATATTGGAAGGTTTTGCAGTCATTGTGGAAATTCCAT

TCTTGCTGCGATTAGTATCCTCTCTCGAAGAAAAAAAAATACCAAAATATCAGAATTTGAATTTACGATC

TATTCATTCAATATTTCCCTTTTTGGAGGACAAATTATCACATTTAAATTATGTGTCAGATATACTAATA

CCTTATCCCATCCATCTGAAAATCTTGGTTCAAATCCTTCAATGCTGGATCCAAGATGTTCCTTCTTTAC

ATTTATTGCGATTCTTTCTTCACGAATATCATAATTGGAATAGGAATAATTCTATTTTTTTTTCAAAAGA

AAATAAAAGACTATTTCGGTTCCCATATAATTATTATGTATCTGAATGCGAATTTGTATTAGTTTTTCTT

CGTAAACAATCTTCTTATTTACGATTAACATCTTCTGGAGCTTTTCTTGAGCGAACACATTTCTATGGAA

AAATAGAACATCTTATAGTAGTGCGCCGTAATTATTTTCAGAGGACCCTATGGTTCTTCAAGGATCCCTT

CATGCATTATGTTCGATATCAAGGAAAAGCAATTCTGGTTTCAAAGGGGACTCATCTTCTGATGAAGAAA

TGGAAATGTCACCTTGTCCATTTCTGGCAATATTATTTTCACTTTTGGTCTCAACCGTACAGGATCCATA

TAAACCAATTATCAAGCTGTTCTTTCCATTTTCTAGGTTATCTTTCAAGTGTACTAATAAATCCTTCGGC

GATAAGGAATCAAATGCTAGAGAATTCATTTCTAATGGATACTGTTATTAAAAAATTCGATACCAGAGTC

CCAGTTATTCCTCTTATTGGATCATTGTCTAAAGCTAAATTTTGTACCGTATCGGGGCATCCTATTAGTA

AGCCGATCTGGACCAATTTATCAGATTGTGATATTATTGATCGATTTGGTCGGATATGTAGAAATCTTTC

TCATTATCACAGTGGATCCTCAAAAAAACAGAGTTTGTATCGAATAAAGTATATACTTCGATTTTCGTGT

GCTAGAACTTTGGCTCGTAAACATAAAAGTACGGTACGCACTTTTTTGCAAAGATTAGGTTCGGGATTAT

TAGAAGAATTCTTTACGGAAGAAGAACAAGTTGTTTCTTTGATCTTCCAAAAAGCAACTTCTTTTTCTTT

ACATGAATCACATATAGAACGTATTTGGTATTTGGATATTATCCGTATCAATGACCTGGTGAATTATTCA

TAATAGGTTTGTTTGGTGACGTGATGAGACTTATGAATAGTCTGGAAATGATCTATAAATGATCAAGAGA

GAAAAAAATTCATGAATTTTCATTCTGAAATGCTCATTGCAGTAGTGTAGTGGTTGAATCAACTGAGTAG

TCAAAATTATTATACTTTCTTCTCGGGACCCAAGTTTTATATTATACATAGGTAAAGTCGTGTGCAATG

>Chamaedorea_linearis_DQ178679.1

CGTTCTGACCATATTGCACTATGTATCATTTGATAACCCAAAAAATGAAATGGGTCCTGCCTCTGGTTCA

AGTAGAAATGTAAATGGAAGAATTACAAGGATATTTAGAAAAAGATAGATCTCGGCAACAACACTTTCTA

TATCCGCTTCTCTTTAAGGAGTATATTTACACATTTGCTCATGATCGTGGTTTAAATGGTTCGATTTTTT

ACGAATCCACGGAAATTTTTGGTTATGACAATAAATCTAGTTCAGTACTTGTGAAACGTTCAATTATTCG

AATGTATCAACAGAATTATTTGATTTATTCGGTTAATGATTCTAACCAAAATCGATTCGTTGGGCACAAC

AATTTTTTTTATTTTCATTTTTATTCTCAGATGATATTGGAAGGTTTTGCAGTCATTGTGGAAATTCCAT

TCTTGCTGCGATTAGTATCTTCTCTCGAAGAAAAAAAAATACCAAAATATCAGAATTTGAATTTACGATC

TATTCATTCAATATTTCCCTTTTTGGAGGACAAATTATCGCATTTAAATTATGTGTCAGATATACTAATA

CCTTATCCCATCCATCTGAAAATCTTGGTTCAAATCCTTCAATGCTGGATCCAAGATGTTCCTTCTTTAC

ATTTCTTGCGATTCCTTCTTCACGAATATCATAATTGGAATAGGAATAATTCCATTTTTTTTTTTTCAAA

AGAAAAAAAAAGACTATTTCGGTTCCCATATAATTATTATGTATCTGAATGCGAATTTGTATTAGTTTTT

CTTCGTAAACAATCTTCTTATTTACGATTAACATCTTCTGGAGCTTTTCTTGATCGAACACATTTCTATG

GAAAAATGGAACATCTTATAGTAGTGCGCCGTAATTATTTTCAGAGGACCCTATGGTTCTTCAAGGATCC

CTTCATGCATTATGTTCGATATCAAGGAAAAGCAATTCTGGTTTCAAAGGGGACTCATCTTCTGATGAAG

AAATGGAAATGTCACCTTGTCCATTTCTGGCAATATTATTTTCACTTTTGGTCTCAACCGTACAGGATCC

ATATAAACCAATTATCAAGCTGTTCTTTCCATTTTCTAGGTTATCTTTCAAGTGTACTAATAAATCCTTT

GGCGGTAAGGAATCAAATGCTAGAGAATTCATTTCTAATGGATACTGTTATTAAAAAATTCGATACCAGA

GTCCCAGTTATTCCTCTTATTGGATCATTGTCTAAAGCTAAATTTTGTACCGTATCGGGGCATCCTATTA

GTAAGCCGATCTGGACCAATTTATCAGATTGTGATATTATTGATCGATTTGGTCGGATANGTAGAAATCT

TTCTCATTATCACAGTGGATCCTCAAAAAAACAGAGTTTGTATCGAATAAAGTATATACTTCGATTTTCG

TGTGCTAGAACTTTGGCTCGTAAACATAAAAGTACAGTACGCACTTTTATGCAAAGATTAGGTTCGGGAT

TATTAGAAGAATTTTTTACGGAAGAAGAACAAGTTGTTTCTTTGATCTTCCCAAAAGCAACTTCTTTTTC

TTTACATGAATCACATATAGAACGTATTTGGTATTTGGATATTATCCGTATCAATGACCTGGTGAATTAT

TCATAATAGGTTTGTTTGGTGACGTGATGAGACTTATGAATAGTCTGGAAATGATCTATAAATGATCAAG

AGAGAAAAAAATTCATGAATTTTCATTCTGAAATGCTCATTGCAGTAGTGTAGTGGTTGAATCAACTGAG

TAGTCAAAATTATTATACTTTCTTCTCGGGACCCAAGTTTTATATTATACATAGGTAAAGTCGTGTGCAA

TG

>Chamaedorea_hooperiana_DQ178677.1

CGTTCTGACCATATTGCACTATGTATCATTTGATAACCCCAAAAATGAAATGGGTCCTGCCTCTGGTTCA

AGTAGAAATGTAAATGGAAGAATTACAAGGATATTTAGAAAAAGATAGATCTCGGCAACAACACTTTCTA

TATCCGCTTCTCTTTAAGGAGTATATTTACACATTTGCTCATGATCGTGGTTTAAATGGTTCGATTTTTT

ACGAATCCACGGAAATTTTTGGTTATGACAATAAATCTAGTTCAGTACTTGTGAAACGTTCAATTATTCG

AATGTATCAACAGAATTATTTGATTTATTCGGTTAATGATTCTAACCAAAATCGATTCGTTGGGCACAAC

AATTTTTTTTATTTTCATTTTTATTCTCAGATGATATTGGAAGGTTTTGCAGTCATTGTGGAAATTCCAT

TCTTGCTGCGATTAGTATCTTCTCTCGAAGAAAAAAAAATACCAAAATATCAGAATTTGAATTTACGATC

TATTCATTCAATATTTCCCTTTTTGGAGGACAAATTATCACATTTAAATTATGTGTCAGATATACTAATA

CCTTATCCCATCCATCTGAAAATCTTGGTTCAAATCCTTCAATGCTGGATCCAAGATGTTCCTTCTTTAC

ATTTATTGCGATTCTTTCTTCACGAATATCATAATTGGAATAGGAATAATTCTATTTTTTTTTTTTCAAA

AGAAAATAAAAGACTATTTCGGTTCCCATATAATTATTATGTATCTGAATGCGAATTTGTATTAGTTTTT

CTTCGTAAACAATCTTCTTATTTACGATTAACATCTTCTGGAGCTTTTCTTGAGCGAACACATTTCTATG

GAAAAATAGAACATCTTATAGTAGTGCGCCGTAATTATTTTCAGAGGACCCTATGGGTCTTCAAGGATCC

CTTCATGCATTATGTTCGATATCAAGGAAAAGCAATTCTGGTTTCAAAGGGGACTCATCTTCTGATGAAG

AAATGGAAATGTCACCTTGTCCATTTCTGGCAATATTATTTTCATTTTTGGTCTCAACCGTACAGGATCC

ATATAAACCAATTATCAAGCTGTTCTTTCCATTTTCTAGGTTATCTTTCAAGTGTACTAATAAATCCTTC

GGCGGTAAGGAATCAAATGCTAGAGAATTCATTTCTAATGGATACTGTTATTAAAAAATTCGATACCAGA

GTCCCAGTTATTCCTCTTATTGGATCATTGTCTAAAGCTAAATTTTGTACCGTATCGGGGCATCCTATTA

GTAAGCCGATCTGGACCAATTTATCAGATTGTGATATTATTGATCGATTTGGTCGGATATGTAGAAATCT

TTCTCATTATCACAGTGGATCCTCAAAAAAACAGAGTTTGTATCGAATAAAGTATATACTTCGATTTTCG

TGTGCTAGAACTTTGGCTCGTAAACATAAAAGTACGGTACGCACTTTTTTGCAAAGATTAGGTTCGGGAT

TATTAGAAGAATTCTTTACGGAAGAAGAACAAGTTGTTTCTTTGATCTTCCCAAAAGCAACTTCTTTTTC

TTTACATGAATCACATATAGAACGTATTTGGTATTTGGATATTATCCGTATCAATGACCTGGTGAATTAT

TCATAATAGGTTTGTTTGGTGACGTGATGAGACTTATGAATAGTCTGGAAATGATCTATAAATGATCAAG

AGAGAAAAAAATTCATGAATTTTCATTCTGAAATGCTCATTGCAGTAGTGTAGTGGTTGAATCAACTGAG

TAGTCAAAATTATTATACTTTCTTCTCGGGACCAAAGTTTTATATTATACATAGGTAAAGTCGTGTGCAA

TG

>Chamaedorea_geonomiformis_DQ178675.1

CGTTCTGACCATATTGCACTATGTATCATTTGATAACCCCAAAAATGAAATGGGTCCTGCCTCTGGTTCA

AGTAGAAATGTAAATGGAAGAATTACAAGGATATTTAGAAAAAGATAGATCTCGGCAACAACACTTTCTA

TATCCGCTTCTCTTTAAGGAGTATATTTACACATTTGCTCATGATCGTGGTTTAAATGGTTCGATTTTTT

ACGAATCCACGGAAATTTTTGGTTATGACAATAAATCTAGTTCAGTACTTGTGAAACGTTCAATTATTCG

AATGTATCAACAGAATTATTTGATTTATTCGGTTAATGATTCTAACCAAAATCGATTCGTTGGGCACAAC

AATTTTTTTTATTTTCATTTTTATTCTCAGATGATATTGGAAGGTTTTGCAGTCATTGTGGAAATTCCAT

TCTTGCTGCGATTAGTATCTTCTCTCGAAGAAAAAAAAATACCAAAATATCAGAATTTGAATTTACGATC

TATTCATTCAATATTTCCCTTTTTGGAGGACAAATTATCACATTTAAATTATGTGTCAGATATACTAATA

CCTTATCCCATCCATCTGAAAATCTTGGTTCAAATCCTTCAATGCTGGATCCAAGATGTTCCTTCTTTAC

ATTTATTGCGATTCTTTCTTCACGAATATCATAATTGGAATAGGAATAATTCTATTTTTTTTTTTTCAAA

AGAAAATAAAAGATTATTTCGGTTCCCATATAATTATTATGTATCTGAATGCGAATTTGTATTAGTTTTT

CTTCGTAAACAATCTTCTTATTTACGATTAACATCTTCTGGAGCTTTTCTTGAGCGAACACATTTCTATG

GAAAAATAGAACATCTTATAGTAGTGCGCCGTAATTATTTTCAGAGGACCCTATGGTTCTTCAAGGATCC

CTTCATGCATTATGTTCGATATCAAGGAAAAGCAATTCTGGTTTCAAAGGGGACTCATCTTCTGATGAAG

AAATGGAAATGTCACCTTGTCCATTTCTGGCAATATTATTTTCATTTTTGGTCTCAACCGTACAGGATCC

ATATAAACCAATTATCAAGCTGTTCTTTCCATTTTCTAGGTTATCTTTCAAGTGTACTAATAAATCCTTC

GGCGGTAAGGAATCAAATGCTAGAGAATTCATTTCTAATGGATACTGTTATTAAAAAATTCGATACCAGA

GTCCCAGTTATTCCTCTTATTGGATCATTGTCTAAAGCTAAATTTTGTACCGTATCGGGGCATCCTATTA

GTAAGCCGATCTGGACCAATTTATCAGATTGTGATATTATTGATCGATTTGGTCGGATATGTAGAAATCT

TTCTCATTATCACAGTGGATCCTCAAAAAAACAGAGTTTGTATCGAATAAAGTATATACTTCGATTTTCG

TGTGCTAGAACTTTGGCTCGTAAACATAAAAGTACGGTACGCACTTTTTTGCAAAGATTAGGTTCGGGAT

TATTAGAAGAATTCTTTACGGAAGAAGAACAAGTTGTTTCTTTGATCTTCCCAAAAGCAACTTCTTTTTC

TTTACATGAATCACATATAGAACGTATTTGGTATTTGGATATTATCCGTATCAATGACCTGGTGAATTAT

TCATAATAGGTTTGTTTGGTGACGTGATGAGACTTATGAATAGTCTGGAAATGATCTATAAATGATCAAG

AGAGAAAAAAATTCATGAATTTTCATTCTGAAATGCTCATTGCAGTAGTGTAGTGGTTGAATCAACTGAG

TAGTCAAAATTATTATACTTTCTTCTCGGGACCAAAGTTTTATATTATACATAGGTAAAGTCGTGTGCAA

TG

>Chamaedorea_ernesti_augustii_DQ178673.1

CGTTCTGACCATATTGCACTATGTATCATTTGATAACCCAAAAAATGAAATGGGTCCTGCCTCTGGTTCA

AGTAGAAATGTAAATGGAAGAATTACAAGGATATTTAGAAAAAGATAGATCTCGGCAACAACACTTTCTA

TATCCGCTTCTCTTTAAGGAGTATATTTACACATTTGCTCATGATCGTGGTTTAAATGGTTCGATTTTTT

ACGAATCCACGGAAATTTTTGGTTATGACAATAAATCTAGTTCAATACTTGTGAAACGTTCAATTATTCG

AATGTATCAACAGAATTATTTGATTTATTCGGTTAATGATTCTAACCAAAATCGATTCGTTGGGCACAAC

CATTTTTTTTATTTTCATTTTTATTCTCAGATGATATTGGAAGGTTTTGCAGTCATTGTGGAAATTCCAT

TCTTGCTGCGATTAGTATCTTCTCTCGAAGAAAAAAAAATACCAAAATATAAGAATTTGAATTTACGATC

TATTCATTCAATATTTCCCTTTTTGGAGGACAAATTATCGCATTTAAATTATGTGTCAGATATACTAATA

CCTTATCCCATCCATCTGAAAATATTGGTTCAAATCCTTCAATGCTGGATCCAAGATGTTCCTTCTTTAC

ATTTATTGCGATTCTTTCTTCACGAATATCATAATTGGAATAGGAATAATTCTATTTTTTTTTCAAAAGA

AAATAAAAGACTATTTCGGTTCCCATATAATTATTATGTATCTGAATGCGAATTTTTATTAGTTTTTCTT

CGTAAACAATCCTCTTATTTACGATTAACATCTTCTGGAGCTTTTCTTGAGCGAACACATTTCTATGGAA

AAATAGAACATCTTATAGTAGTGCGCCGTAATTATTTTCAGAGGACCCTATGGTCCTTCAAGGATCCCTT

CATGCATTATGTTCGATATCAAGGAAAAGCAATTCTGGTTTCAAAGGGGACTCATCTTCTGATGAAGAAA

TGGAAATGTCACCTTGTCCATTTCTGGCAATATTATTTTCACTTTTGGTCTCAACCGTACAGGATCCATA

TAAACCAATTATCAAGCTGTTCTTTCCATTTTCTAGGTTATCTTTCAAGTGTACTAATAAATCCTTCGGC

GGTAAGGAATCAAATGCTAGAGAATTCATTTCTAATGGATACTGTTATTAAAAAATTCGATACCAGAGTC

CCAGTTATTCCTCTTATTGGATCATTGTCTAAAGCTAAATTTTGTACCGTATCGGGGCATCCTATTAGTA

AGCCGATCTGGACCAATTTATCAGATTGTGATATTATTGATCGATTTGGTCGGATATGTAGAAATCTTTC

TCATTATCACAGTGGATCCTCAAAAAAACAGAGTTTGTATCGAATAAAGTATATACTTCGATTTTCGTGT

GCTAGAACTTTGGCTCGTAAACATAAAAGTACGGTACGCACTTTTTTGCAAAGATTAGGTTCGGAATTAT

TAGAAGAATTCTTTACGGAAGAAGAACAAGTTGTTTCTTTGATCTTCCAAAAAGCAACTTCTTTTTCTTT

ACATGAATCACATATAGAACGTATTTGGTATTTGGATATTATCCGTATCAATGACCTGGTGAATTATTCA

TAATAGGTTTGTTTGGTGACGTGATGAGACTTATGAATAGTCTGGAAATGATCTATAAATGATCAAGAGA

TAAAAAAATTCATGAATTTTCATTCTGAAATGCTCATTGCAGTAGTGTAGTGGTTGAATCAACTGAGTAG

TCAAAATTATTATACTTTCTTCTCGGGACCCAAGTTTTATATTATACATAGGTAAAGTCGTGTGCAATG

>Chamaedorea_crucensis_DQ178671.1

CGTTCTGACCATATTGCACTATGTATCATTTGATAACCCCAAAAATGAAATGGGTCCTGCCTCTGGTTCA

AGTAGAAATGTAAATGGAAGAATTACAAGGATATTTAGAAAAAGATAGATCTCGGCAACAACACTTTCTA

TATCCGCTTCTCTTTAAGGAGTATATTTACACATTTGCTCATGATCGTGGTTCAATTTTTTACGAATCCA

CGGAAATTTTTGGTTATGACAATAAATCTAGTTCAGTACTTGTGAAACGTTCAATTATTCGAATGTATCA

ACAGAATTATTTGATTTATTCGGTTAATGATTCTAACCAAAATCGATTCGTTGGGCACAACAATTTTTTT

TATTTTCATTTTTATTCTCAGATGATATTGGAAGGTTTTGCAGTCATTGTGGAAATTCCATTCTTGCTGC

GATTAGTATCTTCTCTCGAAGAAAAAAAAATACCAAAATATCAGAATTTGAATTTACGATCTATTCATTC

AATATTTCCCTTTTTGGAGGACAAATTATCACATTTAAATTATGTGTCAGATATACTAATACCTTATCCC

ATCCATCTGAAAATCTTGGTTCAAATCCTTCAATGCTGGATCCAAGATGTTCCTTCTTTACATTTATTGC

GATTCTTTCTTCACGAATATCATAATTGGAATAGGAATAATTCTATTTTTTTTTCAAAAGAAAATAAAAG

ACTATTTCGGTTCCCATATAATTATTATGTATCTGAATGCGAATTTGTATTAGTTTTTCTTCGTAAACAA

TCTTCTTATTTACGATTAACATCTTCTGGAGCTTTTCTTGAGCGAACACATTTCTATGGAAAAATAGAAC

ATCTTATAGTAGTGCGCCGTAATTATTTTCAGAGGACCCTATGGTTCTTCAAGGATCCCTTCATGCATTA

TGTTCGATATCAAGGAAAAGCAATTCTGGTTTCAAAGGGGACTCATCTTCTGATGAAGAAATGGAAATGT

CACCTTGTCCATTTCTGGCAATATTATTTTCACTTTTGGTCTCAACCGTACAGGATCCATATAAACCAAT

TATCAAGCTGTTCTTTCCATTTTCTAGGTTATCTTTCAAGTGTACTAATAAATCCTTCGGCGGTAAGGAA

TCAAATGCTAGAGAATTCATTTCTAATGGATACTGTTATTAAAAAATTCGATACCAGAGTCCCAGTTATT

CCTCTTATTGGATCATTGTCTAAAGCTAAATTTTGTACCGTATCGGGGCATCCTATTAGTAAGCCGATCT

GGACCAATTTATCAGATTGTGATATTATTGATCGATTTGGTCGGATATGTAGAAATCTTTCTCATTATCA

CAGTGGATCCTCAAAAAAACAGAGTTTGTATCGAATAAAGTATATACTTCGATTTTCGTGTGCTAGAACT

TTGGCTCGTAAACATAAAAGTACGGTACGCACTTTTTTGCAAAGATTAGGTTCGGGATTATTAGAAGAAT

TCTTTACGGAAGAAGAACAAGTTGTTTCTTTGATCTTCCCAAAAGCAACTTCTTTTTCTTTACATGAATC

ACATATAGAACGTATTTGGTATTTGGATATTATCCGTATCAATGACCTGGTGAATTATTCATAATAGGTT

TGTTTGGTGACGTGATGAGACTTATGAATAGTCTGGAAATGATCTATAAATGATCAAGAGAGAAAAAAAT

TCATGAATTTTCATTCTGAAATGCTCATTGCAGTAGTGTAGTGGTTGAATCAACTGAGTAGTCAAAATTA

TTATACTTTCTTCTCGGGACCCAAGTTTTCTATTATACATAGGTAAAGTCGTGTGCAATG

>Chamaedorea_brachypoda_DQ178669.1

CGTTCTGACCATATTGCACTATGTATCATTTGATAATCCCAAAAATGAAATGGGTCCTGCCTCTGGTTCA

AGTAGAAATGTAAATGGAAGAATTACAAGGATATTTAGAAAAAGATAGATCTCGGCAACAACACTTTCTA

TATCCGCTTCTCTTTAAGGAGTATATTTACACATTTGCTCATGATCGTGGTTTAAATGGTTCGATTTTTT

ACGAATCCACGGAAATTTTTGGTTATGACAATAAATCTAGTTCAGTACTTGTGAAACGTTCAATTATTCG

AATGTATCAACAGAATTATTTGATTTATTCGGTTAATGATTCTAACCAAAATCGATTCGTTGGGCACAAC

AATTTTTTTTATTTTCATTTTTATTCTCAGATGATATTGGAAGGTTTTGCAGTCATTGTGGAAATTCCAT

TCTTGCTGCGATTAGTATCTTCTCTCGAAGAAAAAAAAATACCAAAATATCAGAATTTTAATTTACGATC

TATTCATTCAATATTTCCCTTTTTGGAGGACAAATTATCACATTTAAATTATGTGTCAGATATACTAATA

CCTTATCCCATCCATCTGAAAATCTTGGTTCAAATCCTTCAATGCTGGATCCAAGATGTTCCTTCTTTAC

ATTTATTGCGATTCTTTCTTCACGAATATCATAATTGGAATAGGAATAATTCTATTTTTTTTTCAAAAGA

AAATAAAAGACTATTTCGGTTCCCATATAATTATTATGTATCTGAATGCGAATTTGTATTAGTTTTTCTT

CGTAAAAAATCTTCTTATTTACGATTAACATCTTCTGGAGCTTTTCTTGAGCGAACACATTTCTATGGAA

AAATAGAACATCTTATAGTAGTGCGCCGTAATTATTTTCAGAGGACCCTATGGTTCTTCAAGGATCCCTT

CATGCATTATGTTCGATATCAAGGAAAAGCAATTCTGGTTTCAAAGGGGACTCATCTTCTGATGAAGAAA

TGGAAATGTCACCTTGTCCATTTCTGGCAATATTATTTTCACTTTTGGTCTCAACCGTACAGGATCCATA

TAAACCAATTATCAAGCTGTTCTTTCCATTTTCTAGGTTATCTTTCAAGTGTACTAATAAATCCTTCGGC

GGTAAGGAATCAAATGCTAGAGAATTCATTTCTAATAGATACTGTTATTAAAAAATTCGATACCAGAGTC

CCAGTTATTCCTCTTATTGGATCATTGTCTAAAGCTAAATTTTGTACCGTATCGGGGCATCCTGTTAGTA

AGCCGATCTGGACCAATTTATCAGATTGTGATATTATTGATCGATTTGGTCGGATATGTAGAAATCTTTC

TCATTATCACAGTGGATCCTCAAAAAAACAGAGTTTGTATCGAATAAAGTATATACTTCGATTTTCGTGT

GCTAGAACTTTGGCTCGTAAACATAAAAGTACGGTACGCACTTTTTTGCAAAGATTAGGTTCGGGATTAT

TAGAAGAATTCTTTACGGAAGAAGAACAAGTTGTTTCTTTGATCTTCCCAAAAGCAACTTCTTTTTCTTT

ACATGAATCACATATAGAACGTATTTGGTATTTTGATATTATCCGTATCAATGACCTGGTGAATTATTCA

TAATAGGTTTGTTTGGTGACGTGATGAGACTTATGAATAGTCTGGAAATGATCTATAAATGATCAAGAGA

GAAAAAAATTCATGAATTTTCATTCTGAAATGCTCATTGCAGTAGTGTAGTGGTTGAATCAACTGAGTAG

TCAAAATTATTATACTTTCTTCTCGGGACCCAAGTTTTATATTACACAT

>Chamaedorea_allenii_DQ178667.1

CGTTCTGACCATATTGCACTATGTATCATTTGATAACCCCAAAAATGAAATGGGTCCTGCCTCTGGTTCA

AGTAGAAATGTAAATGGAAGAATTACAAGGATATTTAGAAAAAGATAGATCTCGGCAACAACACTTTCTA

TATCCGCTTCTCTTTAAGGAGTATATTTACACATTTGCTCATGATCGTGGTTCAATTTTTTACGAATCCA

CGGAAATTTTTGGTTATGACAATAAATCTAGTTCAGTACTTGTGAAACGTTCAATTATTCGAATGTATCA

ACAGAATTATTTGATTTATTCGGTTAATGATTCTAACCAAAATCGATTCGTTGGGCACAACAATTTTTTT

TATTTTCATTTTTATTCTCAGATGATATTGGAAGGTTTTGCAGTCATTGTGGAAATTCCATTCTTGCTGC

GATTAGTATCTTCTCTCGAAGAAAAAAAAATACCAAAATATCAGAATTTGAATTTACGATCTATTCATTC

AATATTTCCCTTTTTGGAGGACAAATTATCACATTTAAATTATGTGTCAGATATACTAATACCTTATCCC

ATCCATCTGAAAATCTTGGTTCAAATCCTTCAATGCTGGATCCAAGATGTTCCTTCTTTACATTTATTGC

GATTCTTTCTTCACGAATATCATAATTGGAATAGGAATAATTCTATTTTTTTTTCAAAAGAAAATAAAAG

ACTATTTCGGTTCCCATATAATTATTATGTATCTGAATGCGAATTTGTATTAGTTTTTCTTCGTAAACAA

TCTTCTTATTTACGATTAACATCTTCTGGAGCTTTTCTTGAGCGAACACATTTCTATGGAAAAATAGAAC

ATCTTATAGTAGTGCGCCGTAATTATTTTCAGAGGACCCTATGGTTCTTCAAGGATCCCTTCATGCATTA

TGTTCGATATCAAGGAAAAGCAATTCTGGTTTCAAAGGGGACTCATCTTCTGATGAAGAAATGGAAATGT

CACCTTGTCCATTTCTGGCAATATTATTTTCACTTTTGGTCTCAACCGTACAGGATCCATATAAACCAAT

TATCAAGCTGTTCTTTCCATTTTCTAGGTTATCTTTCAAGTGTACTAATAAATCCTTCGGCGGTAAGGAA

TCAAATGCTAGAGAATTCATTTCTAATGGATACTGTTATTAAAAAATTCGATACCAGAGTCCCAGTTATT

CCTCTTATTGGATCATTGTCTAAAGCTAAATTTTGTACCGTATCGGGGCATCCTATTAGTAAGCCGATCT

GGACCAATTTATCAGATTGTGATATTATTGATCGATTTGGTCGGATATGTAGAAATCTTTCTCATTATCA

CAGTGGATCCTCAAAAAAACAGAGTTTGTATCGAATAAAGTATATACTTCGATTTTCGTGTGCTAGAACT

TTGGCTCGTAAACATAAAAGTACGGTACGCACTTTTTTGCAAAGATTAGGTTCGGGATTATTAGAAGAAT

TCTTTACGGAAGAAGAACAAGTTGTTTCTTTGATCTTCCCAAAAGCAACTTCTTTTTCTTTACATGAATC

ACATATAGAACGTATTTGGTATTTGGATATTATCCGTATCAATGACCTGGTGAATTATTCATAATAGGTT

TGTTTGGTGACGTGATGAGACTTATGAATAGTCTGGAAATGATCTATAAATGATCAAGAGAGAAAAAAAT

TCATGAATTTTCATTCTGAAATGCTCATTGCAGTAGTGTAGTGGTTGAATCAACTGAGTAGTCAAAATTA

TTATACTTTCTTCTCGGGACCCAAGTTTTCTATTATACATAGGTAAAGTCGTGTGCAATG

>Chamaedorea_elatior_DQ178702.1

ATTGCACTATGTATCATTTGATAACCCAAAAATGAAATGGGTCCTGCCTCTGGTTCAAGTAGAAATGTAA

ATGGAAGAATTACAAGGATATTTAGAAAAAGATAGATCTCGGCAACAACACTTTCTATATCCGCTTCTCT

TTAAGGAGTATATTTACACATTTGCTCATGATCGTGGTTTAAATGGTTCGATTTTTTACGAATCCACGGA

AATTTTTGGTTATGACAATAAATCTAGTTCAGTACTTGTGAAACGTTCAATTATTCGAATGTATCAACAG

AATTATTTGATTTATTCGGTTAATGATTCTAACCAAAATCGATTCGTTGGGCACAACAATTTTTTTTATT

TTCATTTTTATTCTCAGATGATATTGGAAGGTTTTGCAGTCATTGTGGAAATTCCATTCTTGCTGCGATT

AGTATCTTCTCTCGAAGAAAAAAAAATACCAAAATATCNGAATTTGAATTTNNGATCTATTCANTCAATA

TTTCCCTTTTTGGAGGACAAANTATCACATTTAAATTATGTGTCAGATATACTAATACCTTATCCCATCC

ATCTGAAAATCTTGGTTCAAATCCNTCAATGCTGGATCCAAGATGTTCCTTCTTTACATTTATTGCGATT

CTTTCTTCACGAATATCATAATTGGAATAGGAATAATTCTATTTTTTTTTTTTCAAAAGAAAATAAAAGA

CTATTTCGGTTCCCATATAATTATTATGTATCTGAATGCGAATTTTTATTAGTTTTTCTTCGTAAACAAT

CTTCTTATTTACGATTAACATCTTCNGGAGCTTTTCTTGAGCGAACACATTTCTATGGAAAAATAGAACA

TCTTATAGTAGTGCGCCGTAATTATTTTCAGAGGACCCTATGGGTCTTCAAGGATCCCTTCATGCATTAT

GTTCGATATCAAGGAAAAGCAATTCTGGTTTCAAAGGGGACTCATCTTCTGATGAAGAAATGGAAATGTC

ACCTTGTCCATTTCTGGCAATATTATTTTCATTTTTGGTCTCAACCGTACAGGATCCATATAAACCAATT

ATCAAGCTGTTCTTTCCATTTTCTAGGTTATCTTTCAAGTGTACTAATAAATCCTTCGGCGGTAAGGAAT

CAAATGCTAGAGAATTCATTTCTAATGGATACTGTTATTAAAAAATTCGATACCAGAGTCCCAGTTATTC

CTCTTATTGGATCATTGTCTAAAGCTAAATTTTGTACCGTATCGGGGCATCCTATTAGTAAGCCGATCTG

GACCAATTTATCAGATTGTGATATTATTGATCGATTTGGTCGGATATGTAGAAATCTTTCTCATTATCAC

AGTGGATCCTCAAAAAAACAGAGTTTGTATCGAATAAAGTATATACTTCGATTTTCGTGTGCCAGAACTT

TGGCTCGTAAACATNAAAGTACGGTACGCACTTTTTTGCAAAGATTAGGTTCGGGATTATTAGAAGAATT

CTTTACGGAAGAAGAACAAGTTGTTTCTTTGATCTTCCCAAAAGCAACTTCTTTTTCTTTACATGAATCA

CATATAGAACGTATTTGGTATTTGGATATTATCCGTATCAATGACCTGGTGAATTATTCATAATAGGTTT

GTTTGGTGACGTGATGAGACTTATGAATAGTCTGGAAATGATCTATAAATGATCAAGAGAGAAAAAAATT

CATGAATTTTCATTCTGAAATGCTCATTGCAGTAGTGTAGTGGTTGAATCAACTGAGTAGTCAAAATTAT

TATACTTTCTTCTCGGGACCAAAGTTTTATATTATACATAGGTAAAGT

>Chamaedorea_stolonifera_DQ178690.1

CGTTCTGACCATATTGCACTATGTATCATTTGATAACCCAAAAAATGAAATGGGTCCTGCCTCTGGTTCA

AGTAGAAATGTAAATGGAAGAATTACAAGGATATTTAGAAAAAGATAGATCTCGGCAACAACACTTTCTA

TATCCGCTTCTCTTTAAGGAGTATATTTACACATTTGCTCATGATCGTGGTTTAAATGGTTCGATTTTTT

ACGAATCCACGGAAATTTTTGGTTATGACAATAAATCTAGTTCAATACTTGTGAAACGTTCAATTATTCG

AATGTATCAACAGAATTATTTGATTTATTCGGTTAATGATTCTAACCAAAATCGATTCGTTGGGCACAAC

CATTTTTTTTATTTTCATTTTTATTCTCAGATGATATTGGAAGGTTTTGCAGTCATTGTGGAAATTCCAT

TCTTGCTGCGATTAGTATCTTCTCTCGAAGAAAAAAAAATACCAAAATATAAGAATTTGAATTTACGATC

TATTCATTCAATATTTCCCTTTTTGGAGGACAAATTATCGCATTTAAATTATGTGTCAGATATACTAATA

CCTTATCCCATCCATCTGAAAATATTGGTTCAAATCCTTCAATGCTGGATCCAAGATGTTCCTTCTTTAC

ATTTATTGCGATTCTTTCTTCACGAATATCATAATTGGAATAGGAATAATTCTATTTTTTTTTCAAAAGA

AAATAAAAGACTATTTCGGTTCCCATATAATTATTATGTATCTGAATGCGAATTTTTATTAGTTTTTCTT

CGTAAACAATCCTCTTATTTACGATTAACATCTTCTGGAGCTTTTCTTGAGCGAACACATTTCTATGGAA

AAATAGAACATCTTATAGTAGTGCGCCGTAATTATTTTCAGAGGACCCTATGGTCCTTCAAGGATCCCTT

CATGCATTATGTTCGATATCAAGGAAAAGCAATTCTGGTTTCAAAGGGGACTCATCTTCTGATGAAGAAA

TGGAAATGTCACCTTGTCCATTTCTGGCAATATTATTTTCACTTTTGGTCTCAACCGTACAGGATCCATA

TAAACCAATTATCAAGCTGTTCTTTCCATTTTCTAGGTTATCTTTCAAGTGTACTAATAAATCCTTCGGC

GGTAAGGAATCAAATGCTAGAGAATTCATTTCTAATGGATACTGTTATTAAAAAATTCGATACCAGAGTC

CCAGTTATTCCTCTTATTGGATCATTGTCTAAAGCTAAATTTTGTACCGTATCGGGGCATCCTATTAGTA

AGCCGATCTGGACCAATTTATCAGATTGTGATATTATTGATCGATTTGGTCGGATATGTAGAAATCTTTC

TCATTATCACAGTGGATCCTCAAAAAAACAGAGTTTGTATCGAATAAAGTATATACTTCGATTTTCGTGT

GCTAGAACTTTGGCTCGTAAACATAAAAGTACGGTACGCACTTTTTTGCAAAGATTAGGTTCGGAATTAT

TAGAAGAATTCTTTACGGAAGAAGAACAAGTTGTTTCTTTGATCTTCCAAAAAGCAACTTCTTTTTCTTT

ACATGAATCACATATAGAACGTATTTGGTATTTGGATATTATCCGTATCAATGACCTGGTGAATTATTCA

TAATAGGTTTGTTTGGTGACGTGATGAGACTTATGAATAGTCTGGAAATGATCTATAAATGATCAAGAGA

TAAAAAAATTCATGAATTTTCATTCTGAAATGCTCATTGCAGTAGTGTAGTGGTTGAATCAACTGAGTAG

TCAAAATTATTATACTTTCTTCTCGGGACCCAAGTTTTATATTATACATAGGTAAAGTCGTGTGGCAAT

>Chamaedorea_schiedeana_DQ178688.1

CGTTCTGACCATATTGCACTATGTATCATTTGATAACCCAAAAAATGAAATGGGTCCTGCCTCTGGTTCA

AGTAGAAATGTAAATGGAAGAATTACAAGGATATTTAGAAAAAGATAGATCTCGGCAACAACACTTTCTA

TATCCGCTTCTCTTTAAGGAGTATATTTACACATTTGCTCATGATCGTGGTTTAAATGGTTCGATTTTTT

ACGAATCCACGGAAATTTTTGGTTATGACAATAAATCTAGTTCAATACTTGTGAAACGTTCAATTATTCG

AATGTATCAACAGAATTATTTGATTTATTCGGTTAATGATTCTAACCAAAATCGATTCGTTGGGCACAAC

CATTTTTTTTATTTTCATTTTTATTCTCAGATGATATTGGAAGGTTTTGCAGTCATTGTGGAAATTCCAT

TCTTGCTGCGATTAGTATCTTCTCTCGAAGAAAAAAAAATACCAAAATATCAGAATTTGAATTTACGATC

TATTCATTCAATATTTCCCTTTTTGGAGGACAAATTATCGCATTTAAATTATGTGTCAGATATACTAATA

CCTTATCCCATCCATCTGAAAATATTGGTTCAAATCCTTCAATGCTGGATCCAAGATGTTCCTTCTTTAC

ATTTATTGCGATTCTTTCTTCACGAATATCATAATTGGAATAGGAATAATTCTATTTTTTTTTCAAAAGA

AAATAAAAGACTATTTCGGTTCCCATATAATTATTATGTATCTGAATGCGAATTTTTATTAGTTTTTCTT

CGTAAACAATCCTCTTATTTACGATTAACATCTTCTGGAGCTTTTCTTGAGCGAACACATTTCTATGGAA

AAATAGAACATCTTATAGTAGTGCGCCGTAATTATTTTCAGAGGACCCTATGGTTCTTCAAGGATCCCTT

CATGCATTATGTTCGATATCAAGGAAAAGCAATTCTGGTTTCAAAGGGGACTCATCTTCTGATGAAGAAA

TGGAAATGTCACCTTGTCCATTTCTGGCAATATTATTTTCACTTTTGGTCTCAACCGTACAGGATCCATA

TAAACCAATTATCAAGCTGTTCTTTCCATTTTCTAGGTTATCTTTCAAGTGTACTAATAAATCCTTCGGC

GGTAAGGAATCAAATGCTAGAGAATTCATTTCTAATGGATACTGTTATTAAAAAATTCGATACCAGAGTC

CCAGTTATTCCTCTTATTGGATCATTGTCTAAAGCTAAATTTTGTACCGTATCGGGTCATCCTATTAGTA

AGCCGATCTGGACCAATTTATCAGATTGTGATATTATTGATCGATTTGGTCGGATATGTAGAAATCTTTC

TCATTATCACAGTGGATCCTCAAAAAAACAGAGTTTGTATCGAATAAAGTATATACTTCGATTTTCGTGT

GCTAGAACTTTGGCTCGTAAACATAAAAGTACGGTACGCACTTTTTTGCAAAGATTAGGTTCGGAATTAT

TAGAAGAATTCTTTACGGAAGAAGAACAAGTTGTTTCTTTGATCTTCCCAAAAGCAACTTCTTTTTCTTT

ACATGAATCACATATAGAACGTATTTGGTATTTGGATATTATCCGTATCAATGACCTGGTGAATTATTCA

TAATAGGTTTGTTTGGTGACGTGATGAGACTTATGAATAGTCTGGAAATGATCTATAAATGATCAAGAGA

GAAAAAAATTCATGAATTTTCATTCTGAAATGCTCATTGCAGTAGTGTAGTGGTTGAATCAACTGAGTAG

TAAAAATTATTATACTTTCTTCTCGGGACCCAAGTTTTATATTATACATAGGTAAAGTCGTGTGCAATG

>Chamaedorea_plumosa_DQ178686.1

CGTTCTGACCATATTGCACTATGTATCATTTGATAACCCCAAAAATGAAATGGGTCCTGCCTCTGGTTCA

AGTAGAAATGTAAATGGAAGAATTACAAGGATATTTAGAAAAAGATAGATCTCGGCAACAACACTTTCTA

TATCCGCTTCTCTTTAAGGAGTATATTTACACATTTGCTCATGATCGTGGTTTAAATGGTTCGATTTTTT

ACGAATCCACGGAAATTTTTGGTTATGACAATAAATCTAGTTCAGTACTTGTGAAACGTTCAATTATTCG

AATGTATCAACAGAATTATTTGATTTATTCGGTTAATGATTCTAACCAAAATCGATTCGTTGGGCACAAC

CATTTTTTTTATTTTCATTTTTATTCTCAGATGATATTGGAAGGTTTTGCAGTCATTGTGGAAATTCCAT

TCTTGCTGCGATTAGTATCTTCTCTCGAAGAAAAAAAAATACCAAAATATCAGAATTTGAATTTACGATC

TATTCATTCAATATTTCCCTTTTTGGAGGACAAATTATCACATTTAAATTATGTGTCAGATATACTAATA

CCTTATCCCATCCATCTGAAAATCTTGGTTCAAATCCTTCAATGCTGGATCCAAGATGTTCCTTCTTTAC

ATTTATTGCGATTCTTTCTTCACGAATATCATAATTGGAATAGGAATAATTCTATTTTTTTTTCAAAAGA

AAATAAAAGACTATTTCGGTTCCCATATAATTATTATGTATCTGAATGCGAATTTGTATTAGTTTTTCTT

CGTAAACAATCTTCTTATTTACGATTAACATCTTCTGGAGCTTTTCTTGAGCGAACACATTTCTATGGAA

AAATAGAACATCTTATAGTAGTGCGCCGTAATTATTTTCAGAGGACCCTATGGTTCTTCAAGGATCCCTT

CATGCATTATGTTCGATATCAAGGAAAAGCAATTCTGGTTTCAAAGGGGACTCATCTTCTGATGAAGAAA

TGGAAATGTCACCTTGTCCATTTCTGGCAATATTATTTTCACTTTTGGTCTCAACCGTACAGGATCCATA

TAAACCAATTATCAAGCTGTTCTTTCCATTTTCTAGGTTATCTTTCAAATGTACTAATAAATCCTTCGGC

GGTAAGGAATCAAATGCTAGAGAATTCATTTCTAATGGATACTGTTATTAAAAAATTCGATACCAGAGTC

CCAGTTATTCCTCTTATTGGATCATTGTCTAAAGCTAAATTTTGTACCGTATCGGGGCATCCTATTAGTA

AGCCGATCTGGACCAATTTATCAGATTGTGATATTATTGATCGATTTGGTCGGATATGTAGAAATCTTTC

TCATTATCACAGTGGATCCTCAAAAAAACAGAGTTTGTATCGAATAAAGTATATACTTCGATTTTCGTGT

GCTAGAACTTTGGCTCGTAAACATAAAAGTACGGTACGCACTTTTTTGCAAAGATTAGGTTCGGGATTAT

TAGAAGAATTCTTTACGGAAGAAGAACAAGTTGTTTCTTTGATCTTCCCAAAAGCAACTTCTTTTTCTTT

ACATGAATCACATATAGAACGTATTTGGTATTTGGATATTATCCGTATCAATGACCTGGTGAATTATTCA

TAATAGGTTTGTTTGGTGACGTGATGAGACTTATGAATAGTCTGGAAATGATCTATAAATGATCAAGAGA

GAAAAAAATTCATGAATTTTCATTCTGAAATGCTCATTGCAGTAGTGTAGTGGTTGAATCAACTGAGTAG

TCAAAATTATTATACTTTCTTCTCGGGACCCAAGTTTTATATTATACATAGGTAAAGTCGTGTGCAATG

>Chamaedorea_pedunculata_DQ178684.1

CGTTCTGACCATATTGCACTATGTATCATTTGATAACCCCAAAAATGAAATGGGTCCTGCCTCTGGTTCA

AGTAGAAATGTAAATGGAAGAATTACAAGGATATTTAGAAAAAGATAGATCTCGGCAACAACACTTTCTA

TATCCGCTTCTCTTTAAGGAGTATATTTACACATTTGCTCATGATCGTGGTTCAATTTTTTACGAATCCA

CGGAAATTTTTGGTTATGACAATAAATCTAGTTCAGTACTTGTGAAACGTTCAATTATTCGAATGTATCA

ACAGAATTATTTGATTTATTCGGTTAATGATTCTAACCAAAATCGATTCGTTGGGCACAACAATTTTTTT

TATTTTCATTTTTATTCTCAGATGATATTGGAAGGTTTTGCAGTCATTGTGGAAATTCCATTCTTGCTGC

GATTAGTATCTTCTCTCGAAGAAAAAAAAATACCAAAATATCAGAATTTGAATTTACGATCTATTCATTC

AATATTTCCCTTTTTGGAGGACAAATTATCACATTTAAATTATGTGTCAGATATACTAATACCTTATCCC

ATCCATCTGAAAATCTTGGTTCAAATCCTTCAATGCTGGATCCAAGATGTTCCTTCTTTACATTTATTGC

GATTCTTTCTTCACGAATATCATAATTGGAATAGGAATAATTCTATTTTTTTTTCAAAAGAAAATAAAAG

ACTATTTCGGTTCCCATATAATTATTATGTATCTGAATGCGAATTTGTATTAGTTTTTCTTCGTAAACAA

TCTTCTTATTTACGATTAACATCTTCTGGAGCTTTTCTTGAGCGAACACATTTCTATGGAAAAATAGAAC

ATCTTATAGTAGTGCGCCGTAATTATTTTCAGAGGACCCTATGGTTCTTCAAGGATCCCTTCATGCATTA

TGTTCGATATCAAGGAAAAGCAATTCTGGTTTCAAAGGGGACTCATCTTCTGATGAAGAAATGGAAATGT

CACCTTGTCCATTTCTGGCAATATTATTTTCACTTTTGGTCTCAACCGTACAGGATCCATATAAACCAAT

TATCAAGCTGTTCTTTCCATTTTCTAGGTTATCTTTCAAGTGTACTAATAAATCCTTCGGCGGTAAGGAA

TCAAATGCTAGAGAATTCATTTCTAATAGATACTGTTATTAAAAAATTCGATACCAGAGTCCCAGTTATT

CCTCTTATTGGATCATTGTCTAAAGCTAAATTTTGTACCGTATCGGGGCATCCTATTAGTAAGCCGATCT

GGACCAATTTATCAGATTGTGATATTATTGATCGATTTGGTCGGATATGTAGAAATCTTTCTCATTATCA

CAGTGGATCCTCAAAAAAACAGAGTTTGTATCGAATAAAGTATATACTTCGATTTTCGTGTGCTAGAACT

TTGGCTCGTAAACATAAAAGTACGGTACGCACTTTTTTGCAAAGATTAGGTTCGGGATTATTAGAAGAAT

TCTTTACGGAAGAAGAACAAGTTGTTTCTTTGATCTTCCCAAAAGCAACTTCTTTTTCTTTACATGAATC

ACATATAGAACGTATTTGGTATTTGGATATTATCCGTATCAATGACCTGGTGAATTATTCATAATAGGTT

TGTTTGGTGACGTGATGAGACTTATGAATAGTCTGGAAATGATCTATAAATGATCAAGAGAGAAAAAAAT

TCATGAATTTTCATTCTGAAATGCTCATTGCAGTAGTGTAGTGGTTGAATCAACTGAGTAGTCAAAATTA

TTATACTTTCTTCTCGGGACCCAAGTTTTCTATTATACATAGGTAAAGTCGTGTGCAATG

>Chamaedorea_neurochlamys_DQ178682.1

CGTTCTGACCATATTGCACTATGTATCATTTGATAACCCCAAAAATGAAATGGGTCCTGCCTCTGGTTCA

AGTAGAAATGTAAATGGAAGAATTACAAGGATATTTAGAAAAAGATAGATCTCGGCAACAACACTTTCTA

TATCCGCTTCTCTTTAAGGAGTATATTTACACATTTGCTCATGATCGTGGTTTAAATGGTTCGATTTTTT

ACGAATCCACGGAAATTTTTGGTTATGACAATAAATCTAGTTCAGTACTTGTGAAACGTTCAATTATTCG

AATGTATCAACAGAATTATTTGATTTATTCGGTTAATGATTCTAACCAAAATCGATTCGTTGGGCACAAC

AATTTTTTTTATTTTCATTTTTATTCTCAGATGATATTGGAAGGTTTTGCAGTCATTGTGGAAATTCCAT

TCTTGCTGCGATTAGTATCTTCTCTCGAAGAAAAAAAAATACCAAAATATCAGAATTTGAATTTACGATC

TATTCATTCAATATTTCCCTTTTTGGAGGACAAATTATCACATTTAAATTATGTGTCAGATATACTAATA

CCTTATCCCATCCATCTGAAAATCTTGGTTCAAATCCTTCAATGCTGGATCCAAGATGTTCCTTCTTTAC

ATTTATTGCGATTCTTTCTTCACGAATATCATAATTGGAATAGGAATAATTCTATTTTTTTTTTTTCAAA

AGAAAATAAAAGATTATTTCGGTTCCCATATAATTATTATGTATCTGAATGCGAATTTGTATTAGTTTTT

CTTCGTAAACAATCTTCTTATTTACGATTAACATCTTCTGGAGCTTTTCTTGAGCGAACACATTTCTATG

GAAAAATAGAACATCTTATAGTAGTGCGCCGTAATTATTTTCAGAGGACCCTATGGTTCTTCAAGGATCC

CTTCATGCATTATGTTCGATATCAAGGAAAAGCAATTCTGGTTTCAAAGGGGACTCATCTTCTGATGAAG

AAATGGAAATGTCACCTTGTCCATTTCTGGCAATATTATTTTCATTTTTGGTCTCAACCGTACAGGATCC

ATATAAACCAATTATCAAGCTGTTCTTTCCATTTTCTAGGTTATCTTTCAAGTGTACTAATAAATCCTTC

GGCGGTAAGGAATCAAATGCTAGAGAATTCATTTCTAATGGATACTGTTATTAAAAAATTCGATACCAGA

GTCCCAGTTATTCCTCTTATTGGATCATTGTCTAAAGCTAAATTTTGTACCGTATCGGGGCATCCTATTA

GTAAGCCGATCTGGACCAATTTATCAGATTGTGATATTATTGATCGATTTGGTCGGATATGTAGAAATCT

TTCTCATTATCACAGTGGATCCTCAAAAAAACAGAGTTTGTATCGAATAAAGTATATACTTCGATTTTCG

TGTGCTAGAACTTTGGCTCGTAAACATAAAAGTACGGTACGCACTTTTTTGCAAAGATTAGGTTCGGGAT

TATTAGAAGAATTCTTTACGGAAGAAGAACAAGTTGTTTCTTTGATCTTCCCAAAAGCAACTTCTTTTTC

TTTACATGAATCACATATAGAACGTATTTGGTATTTGGATATTATCCGTATCAATGACCTGGTGAATTAT

TCATAATAGGTTTGTTTGGTGACGTGATGAGACTTATGAATAGTCTGGAAATGATCTATAAATGATCAAG

AGAGAAAAAAATTCATGAATTTTCATTCTGAAATGCTCATTGCAGTAGTGTAGTGGTTGAATCAACTGAG

TAGTCAAAATTATTATACTTTCTTCTCGGGACCAAAGTTTTATATTATACATAGGTAAAGTCGTGTGCAA

TG

>Chamaedorea_metallica_DQ178680.1

CGTTCTGACCATATTGCACTATGTATCATTTGATAACCCAAAAAATGAAATGGGTCCTGCCTCTGGTTCA

AGTAGAAATGTAAATGGAAGAATTACAAGGATATTTAGAAAAAGATAGATCTCGGCAACAACACTTTCTA

TATCCGCTTCTCTTTAAGGAGTATATTTACACATTTGCTCATGATCGTGGTTTAAATGGTTCAATTTTTT

ACGAATCCACGGAAATTTTTGGTTATGACAATAAATCTAGTTCAATACTTGTGAAACGTTCAATTATTCG

AATGTATCAACAGAATTATTTGATTTATTCGGTTAATGATTCTAACCAAAATCGATTCGTTGGGCACAAC

CATTTTTTTTATTTTCATTTTTATTCTCAGATGATATTGGAAGGTTTTGCAGTCATTGTGGAAATTCCAT

TCTTGCTGCGATTAGTATCTTCTCTCGAAAAAAAAAAAATACCAAAATATCAGAATTTGAATTTACGATC

TATTCATTCAATATTTCCCTTTTTGGAGGACAAATTATCGCATTTAAATTATGTGTCAGATATACTAATA

CCTTATCCCATCCATCTGAAAATATTGGTTCAAATCCTTCAATGCTGGATCCAAGATGTTCCTTCTTTAC

ATTTATTGCGATTCTTTCTTCACGAATATCATAATTGGAATAGGAATAATTCTATTTTTTTTTCAAAAGA

AAATAAAAGACTATTTCGGTTCCCATATAATTATTATGTATCTGAATGCGAATTTTTATTAGTTTTTCTT

CGTAAACAATCCTCTTATTTACGATTAACATCTTCTGGAGCTTTTCTTGAGCGAACACATTTCTATGGAA

AAATAGAACATCTTANAGTAGTGCGCCGTAATTATTTTCAGAGGACCCTATGGTTCTTCAAGGATCCCTT

CATGCATTATGTTCGATATCAAGGAAAAGCAATTCTGGTTTCAAAGGGGACTCATCTTCTGATGAAGAAA

TGGAAATGTCACCTTGTCCATTTCTGGCAATATTATTTTCACTTTTGGTCTCAACCGTACAGGATCCATA

TAAACCAATTATCAAGCTGTTCTTTCCATTTTCTAGGTTATCTTTCAAGTGTACTAATAAATCCTTCGGC

GGTAAGGAATCAAATGCTAGAGAATTCATTTCTAATGGATACTGTTATTAAAAAATTCGATACCAGAGTC

CCAGTTATTCCTCTTATTGGATCATTGTCTAAAGCTAAATTTTGTACCGTATCGGGGCATCCTATTAGTA

AGCCGATCTGGACCAATTTATCAGATTGTGATATTATTGATCGATTTGGTCGGATATGTAGAAATCTTTC

TCATTATCACAGTGGATCCTCAAAAAAACAGAGTTTGTATCGAATCAAGTATATACTTCGATTTTCGTGT

GCTAGAACTTTGGCTCGTAAACATAAAAGTACGGTACGCACTTTTTTGCAAAGATTAGGTTCGGAATTAT

TAGAAGAATTCTTTACGGAAGAAGAACAAGTTGTTTCTTTGATCTTCCCAAAAGCAACTTCTTTTTCTTT

ACATGAATCACATATAGAACGTATTTGGTATTTGGATATTATCCGTATCAATGACCTGGTGAATTATTCA

TAATAGGTTTGTTTAGTGACGTGATGAGACTTATGAATAGTCTGGAAATGATCTATAAATGATCAAGAGA

GAAAAAAATTCATGAATTTTCATTCTGAAATGCTCATTGCAGTAGTGTAGTGGTTGAATCAACTGAGTAG

TCAAAATTATTATACTTTCTTCTCGGGACCCAAGTTTTATATTATACATAGGTAAAGTCGTGTGCAA

>Chamaedorea_klotzschiana_DQ178678.1

CGTTCTGACCATATTGCACTATGTATCATTTGATAACCCAAAAAATGAAATGGGTCCTGCCTCTGGTTCA

AGTAGAAATGTAAATGGAAGAATTACAAGGATATTTAGAAAAAGATAGATCTCGGCAACAACACTTTCTA

TATCCGCTTCTCTTTAAGGAGTATATTTACACATTTGCTCATGATCGTGGTTTAAATGGTTCGATTTTTT

ACGAATCCACGGAAATTTTTGGTTATGACAATAAATCTAGTTCAATACTTGTGAAACGTTCAATTATTCG

AATGTATCAACAGAATTATTTGATTTATTCGGTTAATGATTCTAACCAAAATCGATTCGTTGGGCACAAC

CATTTTTTTTATTTTCATTTTTATTCTCAGATGATATTGGAAGGTTTTGCAGTCATTGTGGAAATTCCAT

TCTTGCTGCGATTAGTATCTTCTCTCGAAGAAAAAAAAATACCAAAATATCAGAATTTGAATTTACGATC

TATTCATTCAATATTTCCCTTTTTGGAGGACAAATTATCGCATTTAAATTATGTGTCAGATATACTAATA

CCTTATCCCATCCATCTGAAAATATTGGTTCAAATCCTTCAATGCTGGATCCAAGATGTTCCTTCTTTAC

ATTTATTGCGATTCTTTCTTCACGAATATCATAATTGGAATAGGAATAATTCTATTTTTTTTTCAAAAGA

AAATAAAAGACTATTTCGGTTCCCATATAATTATTATGTATCTGAATGCGAATTTTTATTAGTTTTTCTT

CGTAAACAATCCTCTTATTTACGATTAACATCTTCTGGAGCTTTTCTTGAGCGAACACATTTCTATGGAA

AAATAGAACATCTTATAGTAGTGCGCCGTAATTATTTTCAGAGGACCCTATGGTTCTTCAAGGATCCCTT

CATGCATTATGTTCGATATCAAGGAAAAGCAATTCTGGTTTCAAAGGGGACTCATCTTCTGATGAAGAAA

TGGAAATGTCACCTTGTCCATTTCTGGCAATATTATTTTCACTTTTGGTCTCAACCGTACAGGATCCATA

TAAACCAATTATCAAGCTGTTCTTTCCATTTTCTAGGTTATCTTTCAAGTGTACTAATAAATCCTTCGGC

GGTAAGGAATCAAATGCTAGAGAATTCATTTCTAATGGATACTGTTATTAAAAAATTCGATACCAGAGTC

CCAGTTATTCCTCTTATTGGATCATTGTCTAAAGCTAAATTTTGTACCGTATCGGGTCATCCTATTAGTA

AGCCGATCTGGACCAATTTATCAGATTGTGATATTATTGATCGATTTGGTCGGATATGTAGAAATCTTTC

TCATTATCACAGTGGATCCTCAAAAAAACAGAGTTTGTATCGAATAAAGTATATACTTCGATTTTCGTGT

GCTAGAACTTTGGCTCGTAAACATCAAAGTACGGTACGCACTTTTTTGCAAAGATTAGGTTCGGAATTAT

TAGAAGAATTCTTTACGGAAGAAGAACAAGTTGTTTCTTTGATCTTCCCAAAAGCAACTTCTTTTTCTTT

ACATGAATCACATATAGAACGTATTTGGTATTTGGATATTATCCGTATCAATGACCTGGTGAATTATTCA

TAATAGGTTTGTTTGGTGACGTGATGAGACTTATGAATAGTCTGGAAATGATCTATAAATGATCAAGAGA

GAAAAAAATTCATGAATTTTCATTCTGAAATGCTCATTGCAGTAGTGTAGTGGTTGAATCAACTGAGTAG

TAAAAATTATTATACTTTCTTCTCGGGACCCAAGTTTTATATTATACATAGGTAAAGTCGTGTGCAATG

>Chamaedorea_glaucifolia_DQ178676.1

CGTTCTGACCATATTGCACTATGTATCATTTGATAACCCCAAAAATGAAATGGGTCCTGCCTCTGGTTCA

AGTAGAAATGTAAATGGAAGAATTACAAGGATATTTAGAAAAAGATAGATCTCGGCAACAACACTTTCTA

TATCCGCTTCTCTTTAAGGAGTATATTTACACATTTGCTCATGATCGTGGTTTAAATGGTTCGATTTTTT

ACGAATCCACGGAAATTTTTGGTTATGACAATAAATCTAGTTCAGTACTTGTGAAACGTTCAATTATTCG

AATGTATCAACAGAATTATTTGATTTATTCGGTTAATGATTCTAACCAAAATCGATTCGTTGGGCACAAC

AATTTTTTTTATTTTCATTTTTATTCTCAGATGATATTGGAAGGTTTTGCAGTCATTGTGGAAATTCCAT

TCTTGCTGCGATTAGTATCTTCTCTCGAAGAAAAAAAAATACCAAAATATCAGAATTTGAATTTACGATC

TATTCATTCAATATTTCCCTTTTTGGAGGACAAATTATCACATTTAAATTATGTGTCAGATATACTAATA

CCTTATCCCATCCATCTGAAAATCTTGGTTCAAATCCTTCAATGCTGGATCCAAGATGTTCCTTCTTTAC

ATTTATTGCGATTCTTTCTTCACGAATATCATAATTGGAATAGGAATAATTCTATTTTTTTTTTTTCAAA

AGAAAATAAAAGATTATTTCGGTTCCCATATAATTATTATGTATCTGAATGCGAATTTGTATTAGTTTTT

CTTCGTAAACAATCTTCTTATTTACGATTAACATCTTCTGGAGCTTTTCTTGAGCGAACACATTTCTATG

GAAAAATAGAACATCTTATAGTAGTGCGCCGTAATTATTTTCAGAGGACCCTATGGTTCTGCAAGGATCC

CTTCATGCATTATGTTCGATATCAAGGAAAAGCAATTCTGGTTTCAAAGGGGACTCATCTTCTGATGAAG

AAATGGAAATGTCACCTTGTCCATTTCTGGCAATATTATTTTCATTTTTGGTCTCAACCGTACAGGATCC

ATATAAACCAATTATCAAGCTGTTCTTTCCATTTTCTAGGTTATCTTTCAAGTGTACTAATAAATCCTTC

GGCGGTAAGGAATCAAATGCTAGAGAATTCATTTCTAATGGATACTGTTATTAAAAAATTCGATACCAGA

GTCCCAGTTATTCCTCTTATTGGATCATTGTCTAAAGCTAAATTTTGTACCGTATCGGGGCATCCTATTA

GTAAGCCGATCTGGACCAATTTATCAGATTGTGATATTATTGATCGATTTGGTCGGATATGTAGAAATCT

TTCTCATTATCACAGTGGATCCTCAAAAAAACAGAGTTTGTATCGAATAAAGTATATACTTCGATTTTCG

TGTGCTAGAACTTTGGCTCGTAAACATAAAAGTACGGTACGCACTTTTTTGCAAAGATTAGGTTCGGGAT

TATTAGAAGAATTCTTTACGGAAGAAGAACAAGTTGTTTCTTTGATCTTCCCAAAAGCAACTTCTTTTTC

TTTACATGAATCACATATAGAACGTATTTGGTATTTGGATATTATCCGTATCAATGACCTGGTGAATTAT

TCATAATAGGTTTGTTTGGTGACGTGATGAGACTTATGAATAGTCTGGAAATGATCTATAAATGATCAAG

AGAGAAAAAAATTCATGAATTTTCATTCTGAAATGCTCATTGCAGTAGTGTAGTGGTTGAATCAACTGAG

TAGTCAAAATTATTATACTTTCTTCTCGGGACCAAAGTTTTATATTATACATAGGTAAAGTCGTGTGCAA

TG

>Chamaedorea_fragrans_DQ178674.1

CGTTCTGACCATATTGCACTATGTATCATTTGATAACCCAAAAAATGAAATGGGTCCTGCCTCTGGTTCA

AGTAGAAATGTAAATGGAAGAATTACAAGGATATTTAGAAAAAGATAGATCTCGGCAACAACACTTTCTA

TATCCGCTTCTCTTTAAGGAGTATATTTACACATTTGCTCATGATCGTGGTTTAAATGGTTCGATTTTTT

ACGAATCCACGGAAATTTTTGGTTATGACAATAAATCTAGTTCAGTACTTGTGAAACGTTCAATTATTCG

AATGTATCAACAGAATTATTTGATTTATTCGGTTAATGATTCTAACCAAAATCGATTCGTTGTGCACAAC

AATTTTTTTTATTTTCATTTTTATTCTCAGATGATATTGGAAGGTTTTGCAGTCATTGTGGAAATTCCAT

TCTTGCTGCGATTAGTATCTTCTCTCGAAGAAAAAAAAATACCAAAATATCAGAATTTGAATTTACGATC

TATTCATTCAATATTTCCCTTTTTGGAGGACAAATTATCGCATTTAAATTATGTGTCAGATATACTAATA

CCTTATCCCATCCATCTGAAAATCTTGGTTCAAATCCTTCAATGCTGGATCCAAGATGTTCCTTCTTTAC

ATTTCTTGCGATTATTTCTTCACGAATATCATAATTGGAATAGGAATAATTCCATTTTTTTTTTTCAAAA

GAAAAAAAAAAGACTATTTCGGTTCCCATATAATTATTATGTATCTGAATGCGAATTTGTATTAGTTTTT

CTTCGTAAACAATCTTCTTATTTACGATTAACATCTTCTGGAGCTTTTCTTGAGCGAACACATTTCTATG

GAAAAATGGAACATCTTATAGTAGTGCGCCGTAATTATTTTCAGAGGACCCTATGGTTCTTCAAGGATCC

CTTCATGCATTATGTTCGATATCAAGGAAAAGCAATTCTGGTTTCAAAGGGGACTCATCTTCTGATGAAG

AAATGGAAATGTCACCTTGTCCATTTCTGGCAATATTATTTTCACTTTTGGTCTCAACCGTACAGGATCC

ATATAAACCAATTATCAAGCTGTTCTTTCCATTTTCTAGGTTATCTTTCAAGTGTACTAATAAATCCTTT

GGCGGTAAGGAATCAAATGCTAGAGAATTCATTTCTAATGGATACTGTTATTAAAAAATTCGATACCAGA

GTCCCAGTTATTCCTCTTATTGGATCATTGTCTAAAGCTAAATTTTGTACCGTATCGGGGCATCCTATTA

GTAAGCCGATCTGGACCAATTTATCAGATTGTGATATTATTGATCGATTTGGTCGGATATGTAGAAATCT

TTCTCATTATCACAGTGGATCCTCAAAAAAACAGAGTTTGTATCGAATAAAGTATATACTTCGATTTTCG

TGTGCTAGAACTTTGGCTCGTAAACATAAAAGTACAGTACGCACTTTTATGCAAAGATTAGGTTCGGGAT

TATTAGAAGAATTCTTTACGGAAGAAGAACAAGTTGTTTCTTTGATCTTCCCAAAAGCAACTTCTTTTTC

TTTACATGAATCACATATAGAACGTATTTGGTATTTGGATATTATCCGTATCAATGACCTGGTGAATTAT

TCATAATAGGTTTGTTTGGTGACGTGATGAGACTTATGAATAGTCTGGAAATGATCTATAAATGATCAAG

AGAGAAAAAAATTCATGAATTTTCATTCTGAAATGCTCATTGCAGTAGTGTAGTGGTTGAATCAACTGAG

TAGTCAAAATTATTATACTTTCTTCTCGGGACCCAAGTTTTATATTATACATAGGTAAAGTCGTGTGCAA

TG

>Chamaedorea_dammeriana_DQ178672.1

ATCATTTGATAACCCCAAAAATGAAATGGGTCCTGCCTCTGGTTCAAGTAGAAATGTAAATGGAAGAATT

ACAAGGATATTTAGAAAAAGATAGATCTCGGCAACAACACTTTCTATATCCGCTTCTCTTTAAGGAGTAT

ATTTACACATTTGCTCATGATCGTGGTTTAAATGGTTCGATTTTTTACGAATCCACGGAAATTTTTGGTT

ATGACAATAAATCTAGTTCAGTACTTGTGAAACGTTCAATTATTCGAATGTATCAACAGAATTATTTGAT

TTATTCGGTTAATGATTCTAACCAAAATCGATTCGTTGGGCACAACCATTTTTTTTTTTTTTTTCATTTT

TATTCTCAGATGATATTGGAAGGTTTTGCAGTCATTGTGGAAATTCCATTCTTGCTGCGATTAGTATCTT

CTCTCGAAGAAAAAAAAATACCAAAATATCAGAATTTGAATTTACGATCTATTCATTCAATATTTCCCTT

TTTGGAAGACAAATTATCACATTTAAATTATGTGTCAGATATACTAATACCTTATCCCATCCATCTGAAA

ATCTTGGTTCAAATCCTTCAATGCTGGATCCAAGATGTTCCTTCTTTACATTTCTTGCGATTCTTTCTTC

ACGAATATCATAATTGGAATAGGAATAATTCTATTTTTTTTTCAAAAGAAAATAAAAGACTATTTTGGTT

CCCCTATAATTATTATGTATCTGAATGCGAATTTGTATTAGTTTTTCTTCGTAAACAATCTTCTTATTTA

CGATTAACATCTTCTGGAGCTTTTCTTGAGCGAACACATTTCTATGGAAAAATAGAACATCTTATACTTA

TAGTAGTACGCCGTAATTATTTTCAGAGGACCCTATGGTTCTTCAAGGATCCCTTCATGCATTATGTTCG

ATATCAAGGAAAAGCAATTCTGGTTTCAAAGGGGACTCATCTTCTGATGAAGAAATGGAAATGTCACCTT

GTCCATTTCTGGCAATATTATTTTCACTTTTGGTCTCAACCGTACAGGATCCATATAAACCAATTATCAA

GCTGTTCTTTCCATTTTCTAGGTTATCTTTCAAGTGTACTAATAAATCCTTCGGCGGTAAGGAATCAAAT

GCTAGAGAATTCATTTCTAATGGATACTGTTATTAAAAAATTCGATACCAGAGTCCCAGTTATTCCTCTT

ATTGGATCATTGTCTAAAGCTAAATTTTGTACCGTATCGGGGCATCCTATTAGTAAGCCGATCTGGACCA

ATTTATCAGATTGTGATATTATTGATCGATTTGGTCGGATATGTAGAAATCTTTCTCATTATCACAGTGG

ATCCTCAAAAAAACAGAGTTTGTATCGAATAAAGTATATACTTCGATTTTCGTGTGCTAGAACTTTGGCT

CGTAAACATAAAAGTACGGTACGCACTTTTTTGCAAAGATTAGGTTCGGGATTATTAGAAGAATTCTTTA

CGGAAGAAGAACAAGTTGTTTCTTTGATCTTCCCAAAAGCAACTTCTTTTTCTTTACATGAATCACATAT

AGAACGTATTTGGTATTTGGATATTATCCGTATCAATGACCTGGTGAATTATTCATAATAGGTTTGTTTG

GTGACGTGATGAGACTTATGAATAGTCTGGAAATGATCTATAAATGATCAAGAGAGAAAAAAATTCATGA

ATTTTCATTCTGAAATGCTCATTGCAGTAGTGTAGTGGTTGAATCAACTGAGTAGTCAAAATTATTATAC

TTTCTTCTCGGGACCCAAGTTTTATATTATACATAGGTAAAGTCGTGTGCAATG

>Chamaedorea_costaricana_DQ178670.1

CGTTCTGACCATATTGCACTATGTATCATTTGATAACCCCAAAAATGAAATGGGTCCTGCCTCTGGTTCA

AGTAGAAATGTAAATGGAAGAATTACAAGGATATTTAGAAAAAGATAGATCTCGGCAACAACACTTTCTA

TATCCGCTTCTCTTTAAGGAGTATATTTACACATTTGCTCATGATCGTGGTTCAATTTTTTACGAATCCA

CGGAAATTTTTGGTTATGACAATAAATCTAGTTCAGTACTTGTGAAACGTTCAATTATTCGAATGTATCA

ACAGAATTATTTGATTTATTCGGTTAATGATTCTAACCAAAATCGATTCGTTGGGCACAACAATTTTTTT

TATTTTCATTTTTATTCTCAGATGATATTGGAAGGTTTTGCAGTCATTGTGGAAATTCCATTCTTGCTGC

GATTAGTATCTTCTCTCGAAGAAAAAAAAATACCAAAATATCAGAATTTGAATTTACGATCTATTCATTC

AATATTTCCCTTTTTGGAGGACAAATTATCACATTTAAATTATGTGTCAGATATACTAATACCTTATCCC

ATCCATCTGAAAATCTTGGTTCAAATCCTTCAATGCTGGATCCAAGATGTTCCTTCTTTACATTTATTGC

GATTCTTTCTTCACGAATATCATAATTGGAATAGGAATAATTCTATTTTTTTTTCAAAAGAAAATAAAAG

ACTATTTCGGTTCCCATATAATTATTATGTATCTGAATGCGAATTTGTATTAGTTTTTCTTCGTAAACAA

TCTTCTTATTTACGATTAACATCTTCTGGAGCTTTTCTTGAGCGAACACATTTCTATGGAAAAATAGAAC

ATCTTATAGTAGTGCGCCGTAATTATTTTCAGAGGACCCTATGGTTCTTCAAGGATCCCTTCATGCATTA

TGTTCGATATCAAGGAAAAGCAATTCTGGTTTCAAAGGGGACTCATCTTCTGATGAAGAAATGGAAATGT

CACCTTGTCCATTTCTGGCAATATTATTTTCACTTTTGGTCTCAACCGTACAGGATCCATATAAACCAAT

TATCAAGCTGTTCTTTCCATTTTCTAGGTTATCTTTCAAGTGTACTAATAAATCCTTCGGCGGTAAGGAA

TCAAATGCTAGAGAATTCATTTCTAATGGATACTGTTATTAAAAAATTCGATACCAGAGTCCCAGTTATT

CCTCTTATTGGATCATTGTCTAAAGCTAAATTTTGTACCGTATCGGGGCATCCTATTAGTAAGCCGATCT

GGACCAATTTATCAGATTGTGATATTATTGATCGATTTGGTCGGATATGTAGAAATCTTTCTCATTATCA

CAGTGGATCCTCAAAAAAACAGAGTTTGTATCGAATAAAGTATATACTTCGATTTTCGTGTGCTAGAACT

TTGGCTCGTAAACATAAAAGTACGGTACGCACTTTTTTGCAAAGATTAGGTTCGGGATTATTAGAAGAAT

TATTTACGGAAGAAGAACAAGTTGTTTCTTTGATCTTCCCAAAAGCAACTTCTTTTTCTTTACATGAATC

ACATATAGAACGTATTTGGTATTTGGATATTATCCGTATCAATGACCTGGTGAATTATTCATAATAGGTT

TGTTTGGTGACGTGATGAGACTTATGAATAGTCTGGAAATGATCTATAAATGATCAAGAGAGAAAAAAAT

TCATGAATTTTCATTCTGAAATGCTCATTGCAGTAGTGTAGTGGTTGAATCAACTGAGTAGTCAAAATTA

TTATACTTTCTTCTCGGGACCCAAGTTTTCTATTATACATAGGTAAAGTCGTGTGCAATG

>Chamaedorea_alternans_DQ178668.1

CGTTCTGACCATATTGCACTATGTATCATTTGATAACCCCCAAAATGAAATGGGTCCTGCCTCTGGTTCA

AGTAGAAATGTAAATGGAAGAATTACAAGGATATTTAGAAAAAGATAGATCTCAGCAACAACACTTTCTA

TATCCGCTTCTCTTTAAGGAGTATATTTACACATTTGCTCATGATCGTGGTTTAAATGGTTCGATTTTTT

ACGAATCCACGGAAATTTTTGGTTATGACAATAAATCTAGTTCAGTACTTGTGAAACGTTCAATTATTCG

AATGTATCAACAGAATTATTTGATTTATTCGGTTAATGATTCTAACCAAAATCGATTCGTTGGGCACAAC

CATTTTTTTTATTTTCATTTTTATTCTCAGATGATATTGGAAGGTTTTGCAGTCATTGTGGAAATTCCAN

NNNNNNNGCGATTAGTATCTTCTCTCGAAGAAAAAAAAATACCAAAATATCAGAATTTGAATTTACGATC

TATTCATTCAATATTTCCCTTTTTGGAGGACAAATTATCACATTTAAATTATGTGTCAGATATACTAATA

CCTTATCCCATCCATCTGAAAATCTTGGTTCAAATCCTTCAATGCTGGATCCAAGATGTTCCTTCTTTAC

ATTTATTGCGATTCTTTCTTTACGAATATCATAATTGGAATAGGAATAATTCTATTTTTTTTTCAAAAGA

AAATAAAAGACTATTTCGGTTCCCATATAATTATTATGTATCTGAATGCGAATTTGTATTAGTTTTTCTT

CGTAAACAATCTTCTTATTTACGATTAACATCTTCTGGAGCTTTTCTTGAGCGAACACATTTCTATGGAA

AAATAGAACATCTTATAGTAGTGCGCCGTAATTATTTTCAGAGGACCCTATGGTTCTTCAAGGATCCCTT

CATGCATTATGTTCGATATCAAGGAAAAGCAATTCTGGTTTCAAAGGGGACTCATCTTCTGATGAAGAAA

TGGAAATGTCACCTTGTCCATTTCTGGCAATATTATTTTCACTTTTGGTCTCAACCGTACAGGATCCATA

TAAACCAATTATCAAGCTGTTCTTTCCATTTTCTAGGTTATCTTTCAAGTGTACTAATAAATCCTTCGGC

GGTAAGGAATCAAATGCTAGAGAATTCATTTCTAATGGATACTGTTATTAAAAAATTCGATACCAGAGTC

CCAGTTATTCCTCTTATTGGATCATTGTCTAAAGCTAAATTTTGTACCGTATCGGGGCATCCTATTAGTA

AGCCGATCTGGACCAATTTATCAGATTGTGATATTATTGATCGATTTGGTCGGATATGTAGAAATCTTTC

TCATTATCACAGTGGATCCTCAAAAAAACAGAGTTTGTATCGAATAAAGTATATACTTCGATTTTCGTGT

GCTAGAACTTTGGCTCGTAAACATAAAAGTACGGTACGCACTTTTTTGCAAAGATTAGGTTCGGGATTAT

TAGAAGAATTCTTTACGGAAGAAGAACAAGTTGTTTCTTTGATCTTCCCAAAAGCAACTTCTTTTTCTTT

ACATGAATCACATATAGAACGTATTTGGTATTTGGATATTATCCGTATCAATGACCTGGTGAATTATTCA

TAATAGGTTTGTTTGGTGACGTGATGAGACTTATGAATAGTCTGGAAATGATCTATAAATGATCAAGAGA

GAAAAAAATTCATGAATTTTCATTCTGAAATGCTCATTGCAGTAGTGTAGTGGTTGAATCAACTGAGTAG

TCAAAATTATTATACTTTCTTCTCGGGACCCAAGTTTTATATTATACATAGGTAAAGTCGTGTGCAATG

>Chamaedorea_adscendens_DQ178666.1

CGTTCTGACCATATTGCACTATGTATCATTTGATAACCCCAAAAATGAAATGGGTCCTGCCTCTGGTTCA

AGTAGAAATGTAAATGGAAGAATTACAAGGATATTTAGAAAAAGATAGATCTCGGCAACAACACTTTCTA

TATCCGCTTCTCTTTAAGGAGTATATTTACACATTTGCTCATGATCGTGGTTTAAATGGTTCGATTTTTT

ACGAATCCACGGAAATTTTTGGTTATGACAATAAATCTAGTTCAGTACTTGTGAAACGTTCAATTATTCG

AATGTATCAACAGAATTATTTGATTTATTCGGTTAATGATTCTAACCAAAATCGATTCGTTGGGCACAAC

AATTTTTTTTATTTTCATTTTTATTCTCAGATGATATTGGAAGGTTTTGCAGTCATTGTGGAAATTCCAT

TCTTGCTGCGATTAGTATCTTCTCTCGAAGAAAAAAAAATACCAAAATATCAGAATTTGAATTTACGATC

TATTCATTCAATATTTCCCTTTTTGGAGGACAAATTATCACATTTAAATTATGTGTCAGATATACTAATA

CCTTATCCCATCCATCTGAAAATCTTGGTTCAAATCCTTCAATGCTGGATCCAAGATGTTCCTTCTTTAC

ATTTATTGCGATTCTTTCTTCACGAATATCATAATTGGAATAGGAATAATTCTATTCTTTTTTCAAAAGA

AAATAAAAGACTATTTCGGTTCCCATATAATTATTATGTATCTGAATGCGAATTTGTATTTGTTTTTCTT

CGTAAACAATCTTCTTATTTACGATTAACATCTTCTGGAGCTTTTCTTGAGCGAACACATTTCTATGGAA

AAATAGAACTACTTATAGTTATAGTAGTGCGCCGTAATTATTTTCAGAGGACCCTATGGTTCTTCAAGGA

TCCCTTCATGCATTATGTTCGATATCAAGGAAAAGCAATTCTGGTTTCAAAGGGGACTCATCTTCTGATG

AAGAAATGGAAATGTCACCTTGTCCATTTCTGGCAATATTATTTTCACTTTTGGTCTCAACCGTATAGGA

TCCATATAAACCAATTATCAAGCTGTTCTTTCCATTTTCTAGGTTATCTTTCAAGTGTACTAATAAATCC

TTCGGCGGTAAGGAATCAAATGCTAGAGAATTCATTTCTAATGGATACTTTTATTAAAAAATTCGATACC

AGAGTCCCAGTTATTCCTCTTATTGGATCATTGTCTAAAGCTAAATTTTGTACCGTATCGGGGCATCCTA

TTAGTAAGCCGATCTGGACCAATTTATCAGATTGTGATATTATTGATCGATTTGGTCGGATATGTAGAAA

TCTTTCTCATTATCACAGTGGATCCTCAAAAAAACAGAGTTTGTATCGAATAAAGTATATACTTCGATTT

TCGTGTGCTAGAACTTTGGCTCGTAAACATAAAAGTACGGTACGCACTTTTTTGCAAAGATTAGGTTCGG

GATTATTAGAAGAATTCTTTACGGAAGAAGAACAAGTTGTTTCTTTGATCTTCCCAAAAGCAACTTATTT

TTCTTTACATGAATCACATATAGAACGTATTTGGTATTTGGATATTATCCGTATCAATGACCTGGTGAAT

TATTCATAATAGGTTTGTTTGGTGACGTGATGAGACTTATGAATAGTCTGGAAATGATCTATAAATGATC

AATAGAGAAAAAAATTCATGAATTTTCATTCTGAAATGCTCATTGCAGTAGTGTAGTGGTTGAATCAACT

GAGTATTCAAAATTATTATACTTTCTTCTCGGGACCCAAGTTTTATATTATACATAGGTAAAGTCGTGTG

CAATG

>Chamaedorea_microspadix_AM114623.1

GACCATATTGCACTATGTATCATTTGATAACCCAAAAAATGAAATGGGTCCTGCCTCTGGTTCAAGTAGA

AATGTAAATGGAAGAATTACAAGGATATTTAGAAAAAGATAGATCTCGGCAACAACACTTTCTATATCCG

CTTCTCTTTAAGGAGTATATTTACACATTTGCTCATGATCGTGGTTTAAATGGTTCGATTTTTTACGAAT

CCACGGAAATTTTTGGTTATGACAATAAATCTAGTTCAGTACTTGTGAAACGTTCAATTATTCGAATGTA

TCAACAGAATTATTTGATTTATTCGGTTAATGATTCTAACCAAAATCGATTCGTTGGGCACAACAATTTT

TTTTATTTTCATTTTTATTCTCAGATGATATTGGAAGGTTTTGCAGTCATTGTGGAAATTCCATTCTTGC

TGCGATTAGTATCTTCTCTCGAAGAAAAAAAAATACCAAAATATCAGAATTTGAATTTACGATCTATTCA

TTCAATATTTCCCTTTTTGGAGGACAAATTATCACATTTAAATTATGTGTCAGATATACTAATACCTTAT

CCCATCCATCTGAAAATCTTGGTTCAAATCCTTCAATGCTGGATCCAAGATGTTCCTTCTTTACATTTAT

TGCGATTCTTTCTTCACGAATATCATAATTGGAATAGGAATAATTCTATTTTTTTTTCAAAAGAAAATAA

AAGACTATTTCGGTTCCCATATAATTATTATGTATCTGAATGCGAATTTGTATTAGTTTTTCTTCGTAAA

CAATCTTCTTATTTACGATTAACATCTTCTGGAGCTTTTCTTGAGCGAACACATTTCTATGGAAAAATAG

AACATCTTATAGTAGTGCGCCGTAATTATTTTCAGAGGACCCTATGGTTCTTCAAGGATCCCTTCATGCA

TTATGTTCGATATCAAGGAAAAGCAATTCTGGTTTCAAAAGGGACTCATCTTCTGATGAAGAAATGGAAA

TGTCACCTTGTCCATTTCTGGCAATATTATTTTCATTTTTGGTCTCAACCGTACAGGATCCATATAAACC

AATTATCAAGCTGTTCTTTCCATTTTCTAGGTTATCTTTCAAGTGTACTAATAAATCCTTCGGCGGTAAG

GAATCAAATGCTAGAGAATTCATTTCTAATGGATACTGTTATTAAAAAATTCGATACCAGAGTCCCAGTT

ATTCCTCTTATTGGATCATTGTCTAAAGCTAAATTTTGTACCGTATCGGGGCATCCTATTAGTAAGCCGA

TCTGGACCAATTTATCAGATTGTGATATTATTGATCGATTTGGTCGGATATGTAGAAATCTTTCTCATTA

TCACAGTGGATCCTCAAAAAAACAGAGTTTGTATCGAATAAAGTATATACTTCGATTTTCGTGTGCTAGA

ACTTTGGCTCGTAAACATAAAAGTACGGTACGCACTTTTTTGCAAAGATTAGGTTCGGGATTATTAGAAG

AATTCTTTACGGAAGAAGAACAAGTTGTTTCTTTGATCTTCCCAAAAGCAACTTCTTTTTCTTTACATGA

ATCACATATAGAACGTATTTGGTATTTGGATATTATCCGTATCAATGACCTGGTGAATTATTCATAATAG

GTTTGTTTGGTGACGTGATGAGACTTATGAATAGTCTGGAAATGATCTATAAATGATCAAGAGAGAAAAA

AATTCATGAATTTTCATTCTGAAATGCTCATTGCAGTAGTGTAGTGGTTGAATCAACTGAGTAGTCAAAA

TTATTATACTTTCTTCTCGGGACCCAAGTTTTATATTATACATAGGTAAAGTC

>Chamaedorea_elegans_DQ336357.1

CGTTCTGACCATATTGCACTATGTATCATTTGATAACCCAAAAAATGAAATGGGTCCTGCCTCTGGTTCA

AGTAGAAATGTAAATGGAAGAATTACAAGGATATTTAGAAAAAGATAGATCTCGGCAACAACACTTTCTA

TATCCGCTTCTCTTTAAGGAGTATATTTACACATTTGCTCATGATCGTGGTTTAAATGGTTCGATTTTTT

ACGAATCCACGGAAATTTTTGGTTATGACAATAAATCTAGTTCAATACTTGTGAAACGTTCAATTATTCG

AATGTATCAACAGAATTATTTGATTTATTCGGTTAATGATTCTAACCAAAATCGATTCGTTGGGCACAAC

CATTTTTTTTATTTTCATTTTTATTCTCAGATGATATTGGAAGGTTTTGCAGTCATTGTGGAAATTCCAT

TCTTGCTGCGATTAGTATCTTCTCTCGAAGAAAAAAAAATACCAAAATATCAGAATTTGAATTTACGATC

TATTCATTCAATATTTCCCTTTTTGGAGGACAAATTATCGCATTTAAATTATGTGTCAGATATACTAATA

CCTTATCCCATCCATCTGAAAATATTGGTTCAAATCCTTCAATGCTGGATCCAAGATGTTCCTTCTTTAC

ATTTATTGCGATTCTTTCTTCACGAATATCATAATTGGAATAGGAATAATTCTATTTTTTTTTCAAAAGA

AAATAAAAGACTATTTCGGTTCCCATATAATTATTATGTATCTGAATGTGAATTTTTATTAGTTTTTCTT

CGTAAACAATCCTCTTATTTACGATTAACATCTTCTGGAGCTTTTCTTGAGCGAACACATTTCTATGGAA

AAATAGAACATCTTATAGTAGTGCGCCGTAATTATTTTCAGAGGACCCTATGGTTCTTCAAGGATCCCTT

CATGCATTATGTTCGATATCAAGGAAAAGCAATTCTGGTTTCAAAGGGGACTCATCTTCTGATGAAGAAA

TGGAAATGTCACCTTGTCCATTTCTGGCAATATTATTTTCACTTTTGGTCTCAACCGTACAGGATCCATA

TAAACCAATTATCAAGCTGTTCTTTCCATTTTCTAGGTTATCTTTCAAGTGTACTAATAAATCCTTTGGC

GGTAAGGAATCAAATGCTAGAGAATTCATTTCTAATGGATACTGTTATTAAAAAATTCGATACCAGAGTC

CCAGTTATTCCTCTTATTGGATCATTGTCTAAAGCTAAATTTTGTACCGTATCGGGGCATCCTATTAGTA

AGCCGATCTGGACCAATTTATCAGATTGTGATATTATTGATCGATTTGGTCGGATATGTAGAAATCTTTC

TCATTATCACAGTGGATCCTCAAAAAAACAGAGTTTGTATCGAATAAAGTATATACTTCGATTTTCGTGT

GCTAGAACTTTGGCTCGTAAACATAAAAGTACGGTACGCACTTTTTTGCAAAGATTAGGTTCGGAATTAT

TAGAAGAATTCTTTACGGAAGAAGAACAAGTTGTTTCTTTGATCTTCCCAAAAGCAACTTCTTTTTCTTT

ACATGAATCACATATGGAACGTATTTGGTATTTGGATATTATCCGTATCAATGACCTGGTGAATTATTCA

TAATAGGTTTGTTTGGTGACGTGATGAGACTTATGAATAGTCTGGAAATGATCTATAAATGATCAAGAGA

GAAAAAAATTCATGAATTTTCATTCTGAAATGCTCATTGCAGTAGTGTAGTGGTTGAATCAACTGAGTAG

TCAAAATTATTATACTTTCTTCTCGGGACCCAAGTTTTATATTATACATAGGTAAAGTCGTGTGCAATG

>Chelyocarpus_ulei_AM114562.1

GACCATATTGCACTATGTATCATTTGATAACCCCCAAAATGAAATGGGTTCCGCCTCTGGTTCAAGTAGA

AATGGAAATGGAAGAATTACAAGGATATTTAGAAAAAGATAGATCTCGGCAACAACACTTTCTATATTCG

CTTCTCTTTAAGGAGTATATTTACACATTTGCTCATGATCGTGGTTTAAATGGTTCGATTTTTTACGAAT

CCACGGAAATTTTTGGTTATGACAATAAATCTAGTTCAGTACTTGTGAAACGTTCAATTATTCGAATGTA

TCAACAGAATTATTTGATTTATTCGGTTAATGATTCTAACCAAAATCGATTCGTTGGGCACAACAATTAT

TTTTATTTTCATTTTTATTCTCAGATGATATTGGAAGGTTTTGCAGTCATTGTGGAAATTCCATTCTTGC

TACGATTAGTATCTTTCCTCGAAGAAAAAAAAATACCAAAATCTCAAAATTTGAATTTACGATCTATTCA

TTCAATATTTCCCTTTTTGGAGGACAAATTATCGCATTTAAATTATGTGTCAGATATACTAATACCTTAT

CCCATACATCTGAAAATCTTGGTTCAAATCCTTCAATTCTGGATCCAAGATGTTCCTTCTTTACATTTAT

TGCGATTCTTTCTTCACGAATATCATAATTGGAATAGTCTTATTACTCCGAATAATTCTATTTTTCCTTT

TTTACTTTTTTCAAAAGAAAATAAAAGACTATTTCGGTTCCCATATAATTCTTATGTATCTGAATGCGAA

TTTTTATTAGTTTTTCTTTGTAAACAATCTTCTTATTTACGATTAACATCTTCTGGAGCTTTTCTTGAGC

GAACACATTTCTATGGAAAAATAGAACATCTTATAGTAGTGCGCCGTAATTATTTTCAGAAGACCCTATG

GTTCTTCAAGGATCCCTTCATGCATTATGTTCGATATCAAGGAAAAGCAATTCTGGTTTCAAAGGGGACT

CATCTTCTGATGAAGAAATGGAAATGTCACCTTGTCAATTTCTGGCAATATTATTTTCACTTTTGGTCTC

AACCGTACAGGATCCATATAAAGCAATTATCAAACTGTTCTTTCTATTTTTTAGGTTATCTTTCAAGTGT

ACTAATAAATCCTTCGGCGGTAAGGAATCAAATGCTAGAGAATTCATTTCTAATAGATACTCTTATTAAA

AAATTCGATACCAGAGTCCCAGTTATTCCTCTTATTGGATCATTGTCTAAAGCTAAATTTTGTACCGTAT

CGGGGCATCCTATTAGTAAGCCGATCTGGACCGATTTATCAGATTGCGATATTATTGATCGATTTGGTCG

GATATGTAGAAATCTTTTTCATTATCACAGTGGATCCTCAAAAAAACAGAGTTTGTATCGAATAAAGTAT

ATACTTCGATTTTCGTGTGCTAGAACTTTGGCTCGTAAACATAAAAGTACGGTACGCGCTTTTTTGCAAA

GATTAGGTTCAGGATTATTAGAAGAATTTTTTATGGAAGAAAAAGAAGTTGTTTTTGGGAAGATCAAAGA

AACAACTTCTTTTTCTTTACATGAATCACATATAGAACGTATTTGGTATTTGGATATTATCCGTATCAAT

GACCTGGTGAATTATTCATAATTGGTTTGGTGACGTGATGAGACTTATGAATAGAAAATAGAATAGAAAT

GATCTATAAATGATCAAGAGAGAAAAAAATTCATGAATTTTCATTCTGAAATGCTCATTGCAGTAGTGTA

GTGGTTGAATCAACTGAGTAGTCAAAATTATTATACTTTCTTCTCGGGACCCAAGTTTTGTTTTATATTA

TACATAGGTAAAGTC

>Coccothrinax_argentata_AM114558.1

GACCATATTGCACTATGTATCATTTGATAACCCCAAAAATGAAATAGGTCCCGCCTCTGGTTCAAGTAGA

AATGGAAATGGAAGAATTACAAGGATATTTAGAAAAAGATAGATCTCGGCAACAACACTTTCTATATCCG

CTTCTCTTTAAGGAGTATATTTACACATTTGCTCATGATCGTGGTTTAAATGGTTCGATTTTTTACGAAT

CCACGGAAATTTTTGGTTATGACAATAAATCTAGTTCAGTACTTGTGAAACGTTCAATTATTCGAATGTA

TCAACAGAATTATTTGATTTATTCGGTTAATGATTCTAACCAAAATCGATTCGTTGGGCACAACAATTAT

TTTTATTTTCATTTTTATTCTCAGATGATATTGGAAGGTTTTGCAGTCATTGTGGAAATTCCATTCTTGC

TGCGATTAGTATCTTCCCTCGAAGAAAAAAAAATACCAAAATCTCAAAATTTGAATTTACGATCTATTCA

TTCAATATTTCCCTTTTTGGAGGACAAATTATCGCATTTAAATTATGTGTCAGATATACTAATACCTTAT

CCCATACATCTGAAAATCTTGGTTCAAATCCTTCAATTCTGGATCCAAGATGTTCCTTCTTTACATTTAT

TGCGATTCTTTCTTCACGAATATCATAATTGGAATAGTCTTATTACTCCGAATAATTCTATTTTTCCTTT

TTTACTTTTTTCAAAAGAAAATAAAAGACTATTTCGGTTCCCATATAATTCTTATGTATCTGAATGCGAA

TTTTTATTAGTTTTTCTTCGTAAACAATCTTCTTATTTACGATTAACATCTTCTGGAGCTTTTCTTGAGC

GAACACATTTCTATGGAAAAATAGAACATCTTATAGTAGTGCGCCGTAATTATTTTCAGAAGACCCTATG

GTTCTTCAAGGATCCCTTCATGCATTATGTTCGATATCAAGGAAAAGCAATTCTGGTTTCAAAGGGGACT

CATCTTCTGATGAAGAAATGGAAATGTCACCTTGTCAATTTCTGGCAATATTATTTTCACTTTTGGTCTC

AACCGTACAGGATCCATATAAAGCAATTATCAAACTGTTCTTTCTATTTTTTAGGTTATCTTTCAAGTGT

ACTAATAAATCCTTCGGCGGTAAGGAATCAAATGCTAGAGAATTCATTTCTAATAGATACTCTTATTAAA

AAATTCGATACCAGAGTCCCAGTTATTCCTCTTATTGGATCATTGTCTAAAGCTAAATTTTGTACCGTAT

CGGGGCATCCTATTAGTAAGCCGATCTGGACCGATTTATCAGATTGCGATATTATTGATCGATTTGGTCG

GATATGTAGAAATCTTTCTCATTATCACAGTGGATCCTCAAAAAAACAGAGTTTGTATCGAATAAAGTAT

ATACTTCGATTTTCGTGTGCTAGAACTTTGGCTCGTAAACATAAAAGTACGGTACGCGCTTTTTTGCAAA

GATTAGGTTCAGGATTATTAGAAGAATTTTTTATGGAAGAAGAAGAAGTTGTTTCTTTGATCTTCCCAAA

AACAACTTCTTTTTCTTTACATGAATCACATATAGAACGTATTTGGTATTTGGATATTATCCGTATCAAT

GACCTGGTGAATTATTCATAATTGGTTTGGTGACGTGATGAGACTTATGAATAGAAAATAGAATAGAAAT

GATCTATAAATGATCAAGAGAGAAAAAAATTCATGAATTTTCATTCTGAAATGCTCATTGCAGTAGTGTA

GTGGTTGAATCAACTGAGTAGTCAAAATTATTATACTTTCTTCTCGGGACCCAAGTTTTATATTATACAT

AGGTAAAGTC

>Cocos_nucifera_HQ265564.1

ATTACAAGGATATTTAGAAGAAGATGGATCTCGGCANCAACACTTTCTATATCCGCTTCTCTTAAAGGAG

TATATTTACNCATTTGCTCATGATCGTGGTTTAAATGGTTCGATTTTTTATGAATCCACGGAAATTTTTT

GTTATGACAATAAATCTAGTTCAGTACTTGTGAAACGTTCAATTATTCGAATGNATCAACAGAATTATTN

GATTTATTCGGTTAATGATTCTAACCAAAATCGATTCGTTGGGCACAACAATTATTTTGATTTTCATTTT

TATTCTCAGATGATATTGGAAGGTTTTGCAGTCATTGTGGAAATTCCATTCTTGCTGCGATTAGTATCTT

CCCCCGAAGAAAAAAAAATACCAAAATCTCAGAATTTGAATTTACGATCTATTCATTCAATATTTCCCTT

TTTGGAGGACAAATTATCGCATTTAAATTATGTGTCAGATATACTAATACCTTATCCCATCCATCTGAAA

ATCTTGGTTCAAATCCTTCAATGCTGGATCCAAGATGTTCCTTCTTTACATTTATTGCGATTCTTTCTTC

ACGAATATCATAATTGGAATAGTCTTATTACTCCGAATAATTCTATTTTTTTTTCAAAAGAAAATAAAAG

ACTATTTCGGTTCCCATATAATTCTTATGTATCTGAATGCGAATTTGTATTAGTTTTTCTTCGTAAACAA

TCTTCTTATTTACGATTAACATCTTCTGGAGCTTTTCTTGAGCGAACACATTTCTATGGAAAAATAGAAC

ATCTTATAGTAGTGCGCCGTAATTATTTTCAGAAGACCCTATGGTTCTTCAAGGATCCCTTCATGCATTA

TGTTCGATATCAAGGAAAAGCAATTCTGGTTTCAAAGGGGACTCATCTTCTGATGAAGAAATGGAAATGT

CATCTTGTCAATTTCTGGCAATATTATTTTCACTTTTGGTCTCAACCGTACAGGATCCATATAAACCAAT

TATCAAGCTGTTCTTTCTATTTTCTAGGTTATCTTTCAAGTGTACTAATAAATCCTTCGGCGGTAAGGAA

TCAAATGCTAGAGAATTCATTTCTAATAGATACTGTTATTAAAAAATTCGATACCAGAGTCCCAGTTATT

CCTCTTATTGGATCATTGTCTAAAGCTAAATTTTGTACCGTATCGGGGCATCCTATTAGTAAGCCGATCT

GGACCAATTTATCAGATTGCGATATTATTGATCGATTTGGTCGGATATGTAGAAATCTTTCTCATTATCA

CAGTGGATCCTCAAAAAAACAGAGTTTGTGNAAAATAAAGTATATACTTCGATTTTCGTGCGCNAGAACT

TTGGCTCGTAAACATAANAGTATGGTNCGCGCTTTTTTGCAAAGATTAGGTTCGGGATTATTAGAAGAAT

TCTTTACGGAAGAAGAAGAAGTTGTTTCTTTGATCTTCCAAAAAACAACCTCTTTTTCTTTACATGAATC

ACATATAGAACGTATTTGGTATTTGGATATTATCCGTATCAATGACCTGGTGAATTATTCATAATGGGTT

TGGTGACGTGATGAGACTTATGAATAGAATAGAAATGATCTATAAATGATCAAGAGAGAAAAAAATTCAT

GAATTTTCATTCTGAAATGCTCATTGCAGTAGTGTAGTGGTTGAATCAACTGAGTAGTCAAAATTATTAT

ACTTTCTTCTCGGGACCCAAGTTTTATATTATACATAGGTAAAGTCGTGTGCAATGAAAAATGCAAGCAC

GGTTTGGGGAGGGATCTTTTTCCTCTATTCCAACAAAGAAAAGTTATCTACTCCATCCGGACTAGTTAA

>Cocos_nucifera_AM114637.1

GACCATATTGCACTATGTATCATTTGATAACCCAAAAAATGAAATGGGTCCTGTCTCTGGTTCAAGTAGA

AATGTAAATGGAAGAATTACAAGGATATTTAGAAAAAGATAGATCTCGGCAACAACACTTTCTATATCCG

CTTCTCTTTAAGGAGTATATTTACACATTTGCTCATGATCGTGGTTTAAATGGTTCGATTTTTTATGAAT

CCACGGAAATTTTTTGTTATGACAATAAATCTAGTTCAGTACTTGTGAAACGTTCAATTATTCGAATGTA

TCAACAGAATTATTTGATTTATTCGGTTAATGATTCTAACCAAAATCGATTCGTTGGGCACAACAATTAT

TTTGATTTTCATTTTTATTCTCAGATGATATTGGAAGGTTTTGCAGTCATTGTGGAAATTCCATTCTTGC

TGCGATTAGTATCTTCCCCCGAAGAAAAAAAAATACCAAAATCTCAGAATTTGAATTTACGATCTATTCA

TTCAATATTTCCCTTTTTGGAGGACAAATTATCGCATTTAAATTATGTGTCAGATATACTAATACCTTAT

CCCATCCATCTGAAAATCTTGGTTCAAATCCTTCAATGCTGGATCCAAGATGTTCCTTCTTTACATTTAT

TGCGATTCTTTCTTCACGAATATCATAATTGGAATAGTCTTATTACTCCGAATAATTCTATTTTTTTTTC

AAAAGAAAATAAAAGACTATTTCGGTTCCCATATAATTCTTATGTATCTGAATGCGAATTTGTATTAGTT

TTTCTTCGTAAACAATCTTCTTATTTACGATTAACATCTTCTGGAGCTTTTCTTGAGCGAACACATTTCT

ATGGAAAAATAGAACATCTTATAGTAGTGCGCCGTAATTATTTTCAGAAGACCCTATGGTTCTTCAAGGA

TCCCTTCATGCATTATGTTCGATATCAAGGAAAAGCAATTCTGGTTTCAAAGGGGACTCATCTTCTGATG

AAGAAATGGAAATGTCATCTTGTCAATTTCTGGCAATATTATTTTCACTTTTGGTCTCAACCGTACAGGA

TCCATATAAACCAATTATCAAGCTGTTCTTTCTATTTTCTAGGTTATCTTTCAAGTGTACTAATAAATCC

TTCGGCGGTAAGGAATCAAATGCTAGAGAATTCATTTCTAATAGATACTGTTATTAAAAAATTCGATACC

AGAGTCCCAGTTATTCCTCTTATTGGATCATTGTCTAAAGCTAAATTTTGTACCGTATCGGGGCATCCTA

TTAGTAAGCCGATCTGGACCAATTTATCAGATTGCGATATTATTGATCGATTTGGTCGGATATGTAGAAA

TCTTTCTCATTATCACAGTGGATCCTCAAAAAAACAGAGTTTGTATCGAATAAAGTATATACTTCGATTT

TCGTGTGCTAGAACTTTGGCTCGTAAACATAAAAGTATGGTACGCGCTTTTTTGCAAAGATTAGGTTCGG

GATTATTAGAAGAATTCTTTACGGAAGAAGAAGAAGTTGTTTCTTTGATCTTCCAAAAAACAACCTCTTT

TTCTTTACATGAATCACATATAGAACGTATTTGGTATTTGGATATTATCCGTATCAATGACCTGGTGAAT

TATTCATAATGGGTTTGGTGACGTGATGAGACTTATGAATAGAATAGAAATGATCTATAAATGATCAAGA

GAGAAAAAAATTCATGAATTTTCATTCTGAAATGCTCATTGCAGTAGTGTAGTGGTTGAATCAACTGAGT

AGTCAAAATTATTATACTTTCTTCTCGGGACCCAAGTTTTATATTATACATAGGTAAAGTC

>Cocos_nucifera_GQ434293.1

GATATACTAATACCTTATCCCATCCATCTGAAAATCTTGGTTCAAATCCTTCAATGCTGGATCCAAGATG

TTCCTTCTTTACATTTATTGCGATTCTTTCTTCACGAATATCATAATTGGAATAGTCTTATTACTCCGAA

TAATTCTATTTTTTTTTCAAAAGAAAATAAAAGACTATTTCGGTTCCCATATAATTCTTATGTATCTGAA

TGCGAATTTGTATTAGTTTTTCTTCGTAAACAATCTTCTTATTTACGATTAACATCTTCTGGAGCTTTTC

TTGAGCGAACACATTTCTATGGAAAAATAGAACATCTTATAGTAGTGCGCCGTAATTATTTTCAGAAGAC

CCTATGGTTCTTCAAGGATCCCTTCATGCATTATGTTCGATATCAAGGAAAAGCAATTCTGGTTTCAAAG

GGGACTCATCTTCTGATGAAGAAATGGAAATGTCATCTTGTCAATTTCTGGCAATATTATTTTCACTTTT

GGTCTCAACCGTACAGGATCCATATAAACCAATTATCAAGCTGTTCTTTCTATTTTCTAGGTTATCTTTC

AAGTGTACTAATAAATCCTTCGGCGGTAAGGAATCAAATGCTAGAGAATTCATTTCTAATAGATACTGTT

ATTAAAAAATTCGATACCAGAGTCCCAGTTATTCCTCTTATTGGATCATTGTCTAAAGCTAAATTTTGTA

CCGTATCGGGGCATCCTATTAGTAAGCCGATCTGGACCAATTTATCAGATTGCGATATTATTGATCGATT

TGGTCGGATATGTAGAAATCTTTCTC

>Colpothrinax_wrightii_HQ720256.1

TAGAAATGTAAATGGAAGAATTACAAGGATATTTAGAAAGAGATAGATCTCTGCAACAACACTTTCTATA

TCCGCTTCTCTTTAAGGAGTATATTTACACATTTCTTCATGATCGTGGTTTAAATGGTTCGATTTTTTAC

GAATCCACGGAAATTTTTGGTTATGACAATAAATCTAGTTCAGTACTTGTGAAACGTTCAATTATTCGAA

TGTATCAACAGAATTATTTGATTTATTCGGTTAATGATTCTAACCAAAATCGATTCGTTGGGCACAACAA

TTATTTTTATTTTCATTTTTATTCTCAGATGATATTGGAAGGTTTTGCAGTCATTGTGGAAATTCCATTC

TTGCTGCGATTAGTATCTTCCCTCGAAGAAAAAAAAATACCAAAATCTCAGAATTTGAATTTACGATCTA

TTCATTCAATATTTCCCTTTTTGGAGGACAAATTATCGCATTTAAATTATGTGTCAGATATACTAATACC

TTATCCCATCCATCTGAAAATCTTGGTTCAAATCCTTCAATTCTGGATCCAAGATGTTCCTTCTTTACAT

TTATTGCGATTCTTTCTTCACGAATATCATAATTGGAATAGTCTTATTACTCCGAATAATTCTATTTTTC

TTTTTTCAAAAGAAAATAAAAGACTATTTCGGTTCCCATATAATTCTTATGTATCTGAATGCGAATTTGT

ATTAGTTTTTCTTCGTAAACAATCTTCTTATTTACGATTAACATCTTCTGGAGCTTTTCTTGAGCGAACA

CATTTCTATGGAAAAATAGAATATCGTATAGTAGTGCGCCGTAATTATTTTCAGAAGACCCTATGGTTTT

TCAAGGATCCCTTCATGCATTATGTTCGATATCAAGGAAAAGCAATTCTGGTTTCAAAGGGGACTCATCT

TCTGATGAAGAAATGGAAATGTCACCTTGTCAATTTCTGGCAATATTATTTTCACTTTTGGTCTCAACCG

TACAGGATTCATATAAACCAATTATCAAACTGTTCTTTCTATTTTCTAGGTTATCTTTCAAGTGTACTAA

TAAATCCTTCGGCGGTAAGGAATCAAATGCTAGAGAATTCATTTCTAATAGATACTGTTATTAAAAAATT

CGATACCAGAGTCCCAGTTATTCCTCTTATTAGATCATTGTCTAAAGCTAAATTTTGTACCGTATCGGGG

CATCCTATTAGTAAGCCGATCTGGACCGATTTATCAGATTGCGATATTATTGATCGATTTGGTCGGATAT

GTAGAAATCTTTCTCATTATCACAGTGGATCCTCAAAAAAACAGAGTTTGTATCGAATAAAGTATATACT

TCGATTTTCGTGTGCTAGAACTTTGGCTCGTAAACATAAAAGTATGGTACGCACTTTTTTGCAAAGATTA

GGTTCGGGATTATTAGAAGAATTTTTTATGGAAGAAGAACAAGTTGTTTCTTTGATCTTCCCAAAAACAA

CTTCTTTTTCTTTACATGAATCACATATAGAACGTATTTGGTATTTGGATATTATCCGTATCAATGACCT

GGTGAATTATTCATAATTGGTTTGTTGACGTGATGAGACTTATGAATAGAATAGAAATGATCTATAAATG

ATCAAGAGAGAAAAAAATTCATGAATTTTCATTCTGAAATGCTCATTGCAGTAGTGTAGT

>Colpothrinax_cookii_HQ720254.1

AGAAATGTAAATGGAAGAATTACAAGGATATTTAGAAAGAGATAGATCTCTGCAACAACACTTTCTATAT

CCGCTTCTCTTTAAGGAGTATATTTACACATTTCTTCATGATCGTGGTTTAAATGGTTCGATTTTTTACG

AATCCACGGAAATTTTTGGTTATGACAATAAATCTAGTTCAGTACTTGTGAAACGTTCAATTATTCGAAT

GTATCAACAGAATTATTTGATTTATTCGGTTAATGATTCTAACCAAAATCGATTCGTTGGGCACAACAAT

TATTTTTATTTTCATTTTTATTCTCAGATGATATTGGAAGGTTTTGCAGTCATTGTGGAAATTCCATTCT

TGCTGCGATTAGTATCTTCCCTCGAAGAAAAAAAAATACCAAAATCTCAGAATTTTAATTTACGATCTAT

TCATTCAATATTTCCCTTTTTGGAGGACAAATTATCGCATTTAAATTATGTGTCAGATATACTAATACCT

TATCCCATCCATCTGAAAATCTTGGTTCAAATCCTTCAATTCTGGATCCAAGATGTTCCTTCTTTACATT

TATTGCGATTCTTTCTTCACGAATATCATAATTGGAATAGTCTTATTACTCCGAATAATTCTATTTTTCT

TTTTTCAAAAGAAAATAAAAGACTATTTCGGTTCCCATATAATTCTTATGTATCTGAATGCGAATTTGTA

TTAGTTTTTCTTCGTAAACAATCTTCTTATTTACGATTAACATCTTCTGGAGCTTTTCTTGAGCGAACAC

ATTTCTATGGAAAAATAGAATATCGTATAGTAGTGCGCCGTAATTATTTTCAGAAGACCCTATGGTTTTT

CAAGGATCCCTTCATGCATTATGTTCGATATCAAGGAAAAGCAATTCTGGTTTCAAAGGGGACTCATCTT

CTGATGAAGAAATGGAAATGTCACCTTGTCAATTTCTGGCAATATTATTTTCACTTTTGGTCTCAACCGT

ACAGGATTCATATAAACCAATTATCAAACTGTTCTTTCTATTTTCTAGGTTATCTTTCAAGTGTACTAAT

AAATCCTTCGGCGGTAAGGAATCAAATGCTAGAGAATTCATTTCTAATAGATACTGTTATTAAAAAATTC

GATACCAGAGTCCCAGTTATTCCTCTTATTAGATCATTGTCTAAAGCTAAATTTTGTACCGTATCGGGGC

ATCCTATTAGTAAGCCGATCTGGACCGATTTATCAGATTGCGATATTATTGATCGATTTGGTCGGATATG

TAGAAATCTTTCTCATTATCACAGTGGATCCTCAAAAAAACAGAGTTTGTATCGAATAAAGTATATACTT

CGATTTTCGTGTGCTAGAACTTTGGCTCGTAAACATAAAAGTATGGTACGCACTTTTTTGCAAAGATTAG

GTTCGGGATTATTAGAAGAATTTTTTATGGAAGAAGAACAAGTTGTTTCTTTGATCTTCCCAAAAACAAC

TTCTTTTTCTTTACATGAATCACATATAGAACGTATTTGGTATTTGGATATTATCCGTATCAATGACCTG

GTGAATTATTCATAATTGGTTTGTTGACGTGATGAGACTTATGAATAGAATAGAAATGATCTATAAATGA

TCAAGAGAGAAAAAAATTCATGAATTTTCATTCTGAAATGCTCATTGCAGTAGTGTAGTGGTTGAATCAA

CTGAGTAGTCAAAATTAT

>Colpothrinax_aphanopetala_HQ720252.1

GGAAGAATTACAAGGATATTTAGAAAGAGATAGATCTCTGCAACAACACTTTCTATATCCGCTTCTCTTT

AAGGAGTATATTTACACATTTCTTCATGATCGTGGTTTAAATGGTTCGATTTTTTACGAATCCACGGAAA

TTTTTGGTTATGACAATAAATCTAGTTCAGTACTTGTGAAACGTTCAATTATTCGAATGTATCAACAGAA

TTATTTGATTTATTCGGTTAATGATTCTAACCAAAATCGATTCGTTGGGCACAACAATTATTTTTATTTT

CATTTTTATTCTCAGATGATATTGGAAGGTTTTGCAGTCATTGTGGAAATTCCATTCTTGCTGCGATTAG

TATCTTCCCTCGAAGAAAAAAAAATACCAAAATCTCAGAATTTTAATTTACGATCTATTCATTCAATATT

TCCCTTTTTGGAGGACAAATTATCGCATTTAAATTATGTGTCAGATATACTAATACCTTATCCCATCCAT

CTGAAAATCTTGGTTCAAATCCTTCAATTCTGGATCCAAGATGTTCCTTCTTTACATTTATTGCGATTCT

TTCTTCACGAATATCATAATTGGAATAGTCTTATTACTCCGAATAATTCTATTTTTCTTTTTTCAAAAGA

AAATAAAAGACTATTTCGGTTCCCATATAATTCTTATGTATCTGAATGCGAATTTGTATTAGTTTTTCTT

CGTAAACAATCTTCTTATTTACGATTAACATCTTCTGGAGCTTTTCTTGAGCGAACACATTTCTATGGAA

AAATAGAATATCGTATAGTAGTGCGCCGTAATTATTTTCAGAAGACCCTATGGTTTTTCAAGGATCCCTT

CATGCATTATGTTCGATATCAAGGAAAAGCAATTCTGGTTTCAAAGGGGACTCATCTTCTGATGAAGAAA

TGGAAATGTCACCTTGTCAATTTCTGGCAATATTATTTTCACTTTTGGTCTCAACCGTACAGGATTCATA

TAAACCAATTATCAAACTGTTCTTTCTATTTTCTAGGTTATCTTTCAAGTGTACTAATAAATCCTTCGGC

GGTAAGGAATCAAATGCTAGAGAATTCATTTCTAATAGATACTGTTATTAAAAAATTCGATACCAGAGTC

CCAGTTATTCCTCTTATTAGATCATTGTCTAAAGCTAAATTTTGTACCGTATCGGGGCATCCTATTAGTA

AGCCGATCTGGACCGATTTATCAGATTGCGATATTATTGATCGATTTGGTCGGATATGTAGAAATCTTTC

TCATTATCACAGTGGATCCTCAAAAAAACAGAGTTTGTATCGAATAAAGTATATACTTCGATTTTCGTGT

GCTAGAACTTTGGCTCGTAAACATAAAAGTATGGTACGCACTTTTTTGCAAAGATTAGGTTCGGGATTAT

TAGAAGAATTTTTTATGGAAGAAGAACAAGTTGTTTCTTTGATCTTCCCAAAAACAACTTCTTTTTCTTT

ACATGAATCACATATAGAACGTATTTGGTATTTGGATATTATCCGTATCAATGACCTGGTGAATTATTCA

TAATTGGTTTGTTGACGTGATGAGACTTATGAATAGAATAGAAATGATCTATAAATGATCAAGAGAGAAA

AAAATTCATGAATTTTCATTCTGAAATGCTCATTGCAGTAGTGTAGTGGTTGAATCAACTGAGTAGTCAA

AATTATTATACTTT

>Colpothrinax_wrightii_HQ720255.1

GGAAGAATTACAAGGATATTTAGAAAGAGATAGATCTCTGCAACAACACTTTCTATATCCGCTTCTCTTT

AAGGAGTATATTTACACATTTCTTCATGATCGTGGTTTAAATGGTTCGATTTTTTACGAATCCACGGAAA

TTTTTGGTTATGACAATAAATCTAGTTCAGTACTTGTGAAACGTTCAATTATTCGAATGTATCAACAGAA

TTATTTGATTTATTCGGTTAATGATTCTAACCAAAATCGATTCGTTGGGCACAACAATTATTTTTATTTT

CATTTTTATTCTCAGATGATATTGGAAGGTTTTGCAGTCATTGTGGAAATTCCATTCTTGCTGCGATTAG

TATCTTCCCTCGAAGAAAAAAAAATACCAAAATCTCAGAATTTGAATTTACGATCTATTCATTCAATATT

TCCCTTTTTGGAGGACAAATTATCGCATTTAAATTATGTGTCAGATATACTAATACCTTATCCCATCCAT

CTGAAAATCTTGGTTCAAATCCTTCAATTCTGGATCCAAGATGTTCCTTCTTTACATTTATTGCGATTCT

TTCTTCACGAATATCATAATTGGAATAGTCTTATTACTCCGAATAATTCTATTTTTCTTTTTTCAAAAGA

AAATAAAAGACTATTTCGGTTCCCATATAATTCTTATGTATCTGAATGCGAATTTGTATTAGTTTTTCTT

CGTAAACAATCTTCTTATTTACGATTAACATCTTCTGGAGCTTTTCTTGAGCGAACACATTTCTATGGAA

AAATAGAATATCGTATAGTAGTGCGCCGTAATTATTTTCAGAAGACCCTATGGTTTTTCAAGGATCCCTT

CATGCATTATGTTCGATATCAAGGAAAAGCAATTCTGGTTTCAAAGGGGACTCATCTTCTGATGAAGAAA

TGGAAATGTCACCTTGTCAATTTCTGGCAATATTATTTTCACTTTTGGTCTCAACCGTACAGGATTCATA

TAAACCAATTATCAAACTGTTCTTTCTATTTTCTAGGTTATCTTTCAAGTGTACTAATAAATCCTTCGGC

GGTAAGGAATCAAATGCTAGAGAATTCATTTCTAATAGATACTGTTATTAAAAAATTCGATACCAGAGTC

CCAGTTATTCCTCTTATTAGATCATTGTCTAAAGCTAAATTTTGTACCGTATCGGGGCATCCTATTAGTA

AGCCGATCTGGACCGATTTATCAGATTGCGATATTATTGATCGATTTGGTCGGATATGTAGAAATCTTTC

TCATTATCACAGTGGATCCTCAAAAAAACAGAGTTTGTATCGAATAAAGTATATACTTCGATTTTCGTGT

GCTAGAACTTTGGCTCGTAAACATAAAAGTATGGTACGCACTTTTTTGCAAAGATTAGGTTCGGGATTAT

TAGAAGAATTTTTTATGGAAGAAGAACAAGTTGTTTCTTTGATCTTCCCAAAAACAACTTCTTTTTCTTT

ACATGAATCACATATAGAACGTATTTGGTATTTGGATATTATCCGTATCAATGACCTGGTGAATTATTCA

TAATTGGTTTGTTGACGTGATGAGACTTATGAATAGAATAGAAATGATCTATAAATGATCAAGAGAGAAA

AAAATTCATGAATTTTCATTCTGAAATGCTCAT

>Colpothrinax_wrightii_AM114581.1

GACCATATTGCACTATGTATCATTTGATAACCCAAAAAATGAAATAGGTCCCGCCTCTGGTTCAAGTAGA

AATGTAAATGGAAGAATTACAAGGATATTTAGAAAGAGATAGATCTCTGCAACAACACTTTCTATATCCG

CTTCTCTTTAAGGAGTATATTTACACATTTCTTCATGATCGTGGTTTAAATGGTTCGATTTTTTACGAAT

CCACGGAAATTTTTGGTTATGACAATAAATCTAGTTCAGTACTTGTGAAACGTTCAATTATTCGAATGTA

TCAACAGAATTATTTGATTTATTCGGTTAATGATTCTAACCAAAATCGATTCGTTGGGCACAACAATTAT

TTTTATTTTCATTTTTATTCTCAGATGATATTGGAAGGTTTTGCAGTCATTGTGGAAATTCCATTCTTGC

TGCGATTAGTATCTTCCCTCGAAGAAAAAAAAATACCAAAATCTCAGAATTTGAATTTACGATCTATTCA

TTCAATATTTCCCTTTTTGGAGGACAAATTATCGCATTTAAATTATGTGTCAGATATACTAATACCTTAT

CCCATCCATCTGAAAATCTTGGTTCAAATCCTTCAATTCTGGATCCAAGATGTTCCTTCTTTACATTTAT

TGCGATTCTTTCTTCACGAATATCATAATTGGAATAGTCTTATTACTCCGAATAATTCTATTTTTCTTTT

TTCAAAAGAAAATAAAAGACTATTTCGGTTCCCATATAATTCTTATGTATCTGAATGCGAATTTGTATTA

GTTTTTCTTCGTAAACAATCTTCTTATTTACGATTAACATCTTCTGGAGCTTTTCTTGAGCGAACACATT

TCTATGGAAAAATAGAATATCGTATAGTAGTGCGCCGTAATTATTTTCAGAAGACCCTATGGTTTTTCAA

GGATCCCTTCATGCATTATGTTCGATATCAAGGAAAAGCAATTCTGGTTTCAAAGGGGACTCATCTTCTG

ATGAAGAAATGGAAATGTCACCTTGTCAATTTCTGGCAATATTATTTTCACTTTTGGTCTCAACCGTACA

GGATTCATATAAACCAATTATCAAACTGTTCTTTCTATTTTCTAGGTTATCTTTCAAGTGTACTAATAAA

TCCTTCGGCGGTAAGGAATCAAATGCTAGAGAATTCATTTCTAATAGATACTGTTATTAAAAAATTCGAT

ACCAGAGTCCCAGTTATTCCTCTTATTAGATCATTGTCTAAAGCTAAATTTTGTACCGTATCGGGGCATC

CTATTAGTAAGCCGATCTGGACCGATTTATCAGATTGCGATATTATTGATCGATTTGGTCGGATATGTAG

AAATCTTTCTCATTATCACAGTGGATCCTCAAAAAAACAGAGTTTGTATCGAATAAAGTATATACTTCGA

TTTTCGTGTGCTAGAACTTTGGCTCGTAAACATAAAAGTATGGTACGCACTTTTTTGCAAAGATTAGGTT

CGGGATTATTAGAAGAATTTTTTATGGAAGAAGAACAAGTTGTTTCTTTGATCTTCCCAAAAACAACTTC

TTTTTCTTTACATGAATCACATATAGAACGTATTTGGTATTTGGATATTATCCGTATCAATGACCTGGTG

AATTATTCATAATTGGTTTGTTGACGTGATGAGACTTATGAATAGAATAGAAATGATCTATAAATGATCA

AGAGAGAAAAAAATTCATGAATTTTCATTCTGAAATGCTCATTGCAGTAGTGTAGTGGTTGAATCAACTG

AGTAGTCAAAATTATTATACTTTCTTCTCGGGACCCAAGTTTTATATTATACATAGGTAAAGTC

>Colpothrinax_aphanopetala_HQ720253.1

GGAAGAATTACAAGGATATTTAGAAAGAGATAGATCTCTGCAACAACACTTTCTATATCCGCTTCTCTTT

AAGGAGTATATTTACACATTTCTTCATGATCGTGGTTTAAATGGTTCGATTTTTTACGAATCCACGGAAA

TTTTTGGTTATGACAATAAATCTAGTTCAGTACTTGTGAAACGTTCAATTATTCGAATGTATCAACAGAA

TTATTTGATTTATTCGGTTAATGATTCTAACCAAAATCGATTCGTTGGGCACAACAATTATTTTTATTTT

CATTTTTATTCTCAGATGATATTGGAAGGTTTTGCAGTCATTGTGGAAATTCCATTCTTGCTGCGATTAG

TATCTTCCCTCGAAGAAAAAAAAATACCAAAATCTCAGAATTTTAATTTACGATCTATTCATTCAATATT

TCCCTTTTTGGAGGACAAATTATCGCATTTAAATTATGTGTCAGATATACTAATACCTTATCCCATCCAT

CTGAAAATCTTGGTTCAAATCCTTCAATTCTGGATCCAAGATGTTCCTTCTTTACATTTATTGCGATTCT

TTCTTCACGAATATCATAATTGGAATAGTCTTATTACTCCGAATAATTCTATTTTTCTTTTTTCAAAAGA

AAATAAAAGACTATTTCGGTTCCCATATAATTCTTATGTATCTGAATGCGAATTTGTATTAGTTTTTCTT

CGTAAACAATCTTCTTATTTACGATTAACATCTTCTGGAGCTTTTCTTGAGCGAACACATTTCTATGGAA

AAATAGAATATCGTATAGTAGTGCGCCGTAATTATTTTCAGAAGACCCTATGGTTTTTCAAGGATCCCTT

CATGCATTATGTTCGATATCAAGGAAAAGCAATTCTGGTTTCAAAGGGGACTCATCTTCTGATGAAGAAA

TGGAAATGTCACCTTGTCAATTTCTGGCAATATTATTTTCACTTTTGGTCTCAACCGTACAGGATTCATA

TAAACCAATTATCAAACTGTTCTTTCTATTTTCTAGGTTATCTTTCAAGTGTACTAATAAATCCTTCGGC

GGTAAGGAATCAAATGCTAGAGAATTCATTTCTAATAGATACTGTTATTAAAAAATTCGATACCAGAGTC

CCAGTTATTCCTCTTATTAGATCATTGTCTAAAGCTAAATTTTGTACCGTATCGGGGCATCCTATTAGTA

AGCCGATCTGGACCGATTTATCAGATTGCGATATTATTGATCGATTTGGTCGGATATGTAGAAATCTTTC

TCATTATCACAGTGGATCCTCAAAAAAACAGAGTTTGTATCGAATAAAGTATATACTTCGATTTTCGTGT

GCTAGAACTTTGGCTCGTAAACATAAAAGTATGGTACGCACTTTTTTGCAAAGATTAGGTTCGGGATTAT

TAGAAGAATTTTTTATGGAAGAAGAACAAGTTGTTTCTTTGATCTTCCCAAAAACAACTTCTTTTTCTTT

ACATGAATCACATATAGAACGTATTTGGTATTTGGATATTATCCGTATCAATGACCTGGTGAATTATTCA

TAATTGGTTTGTTGACGTGATGAGACTTATGAATAGAATAGAAATGATCTATAAATGATCAAGAGAGAAA

AAAATTCATGAATTTTCATTCTGAAATGCTCATTGCAGTAGTGTAGTGGTTGAATCAACTGAGTA

>Copernicia_yarey_HQ720270.1

GAAATGTAAATGGAAGAATTACAAGGATATTTAGAAAGAGATAGATCTCTGCAACAACACTTTCTATATC

CGCTTCTCTTTAAGGAGTATATTTACACATTTCTTCATGATCGTGGTTTAAATGGTTCGATTTTTTACGA

ATCCACGGAAATTTTTGGTTATGACAATTTTGGTTATGACAATAAATCTAGTTCAGTACTTGTGAAACGT

TCAATTATTCGAATGTATCAACAGAATTATTTGATTTATTCGGTTAATGATTCTAACCAAAATCGATTCG

TTGGGCACAACAATTATTTTTATTTTCATTTTTATTCTCAGATGATATTGGAAGGTTTTGCAGTCATTGT

GGAAATTCCATTCTTGCTGCGATTAGTATCTTCCCTCGAAGAAAAAAAAATACCAAAATCTCAGAATTTG

AATTTACGATCTATTCATTCAATATTTCCCTTTTTGGAGGACAAATTATCGCATTTAAATTATGTGTCAG

ATATACTAATACCTTATCCCATCCATCTGAAAATCTTGGTTCAAATCCTTCAATTCTGGATCCAAGATGT

TCCTTCTTTACATTTATTGCGATTCTTTCTTCACGAATATCATAATTGGAATAGTCTTATTACTCCGAAT

AATTCTATTTTTCTTTTTTCAAAAGAAAATAAAAGACTATTTCGGTTCCCATATAATTCTTATGTATCTG

AATGCGAATTTGTATTAGTTTTTCTTCGTAAACAATCTTCTTATTTACGATTAACATCTTCTGGAGCTTT

TCTTGAGCGAACACATTTCTATGGAAAAATAGAATATCGTATAGTAGTGCGCCGTAATTATTTTCAGAAG

ACCCTATGGTTTTTCAAGGATCCCTTCATGCATTATGTTCGATATCAAGGAAAAGCAATTCTGGTTTCAA

AGGGGACTCATCTTCTGATGAAGAAATGGAAATGTCACCTTGTCAATTTCTGGCAATATTATTTTCACTT

TTGGTCTCAACCGTACAGGATTCATATAAACCAATTATCAAACTGTTCTTTCTATTTTCTAGGTTATCTT

TCAAGTGTACTAATAAATCCTTCGGCGGTAAGGAATCAAATGCTAGAGAATTCATTTCTAATAGATACTG

TTATTAAAAAATTCGATACTAGAGTCCCAGTTATTCCTCTTATTAGATCATTGTCTAAAGCTAAATTTTG

TACCGTATCGGGGCATCCCATTAGTAAGCCGATCTGGGCCGATTTATCAGATTGCGATATTATTGATCGA

TTTGGTCGGATATGTAGAAATCTTTCTCATTATCACAGTGGATCCTCAAAAAAACAGAGTTTGTATCGAA

TAAAGTATATACTTCGATTTTCGTGTGCTAGAACTTTGGCTCGTAAACATAAAAGTATGGTACGCGCTTT

TTTGCAAAGATTAGGTTCGGGATTATTAGAAGAATTTTTTATGGAAGAAGAACAAGTTGTTTCTTTGATC

TTCCCAAAAACAACTTCTTTTTCTTTACATGAATCACATATAGAACGTATTTGGTATTTGGATATTATCC

GTATCAATGACCTGGTGAATTATTCATAATTGGTTTGTTGACGTGATGAGACTTATGAATAGAATAGAAA

TGATCTATAAATGATCAAGAGAGAAAAAAATTCATGAATTTTAATTCTGAAATGTTCATTGCAGTAGTGT

AGT

>Copernicia_rigida_HQ720268.1

AAATGTAAATGGAAGAATTACAAGGATATTTAGAAAGAGATAGATCTCTGCAACAACACTTTCTATATCC

GCTTCTCTTTAAGGAGTATATTTACACATTTCTTCATGATCGTGGTTTAAATGGTTCGATTTTTTACGAA

TCCACGGAAATTTTTGGTTATGACAATTTTGGTTATGACAATAAATCTAGTTCAGTACTTGTGAAACGTT

CAATTATTCGAATGTATCAACAGAATTATTTGATTTATTCGGTTAATGATTCTAACCAAAATCGATTCGT

TGGGCACAACAATTATTTTTATTTTCATTTTTATTCTCAGATGATATTGGAAGGTTTTGCAGTCATTGTG

GAAATTCCATTCTTGCTGCGATTAGTATCTTCCCTCGAAGAAAAAAAAATACCAAAATCTCAGAATTTGA

ATTTACGATCTATTCATTCAATATTTCCCTTTTTGGAGGACAAATTATCGCATTTAAATTATGTGTCAGA

TATACTAATACCTTATCCCATCCATCTGAAAATCTTGGTTCAAATCCTTCAATTCTGGATCCAAGATGTT

CCTTCTTTACATTTATTGCGATTCTTTCTTCACGAATATCATAATTGGAATAGTCTTATTACTCCGAATA

ATTCTATTTTTCTTTTTTCAAAAGAAAATAAAAGACTATTTCGGTTCCCATATAATTCTTATGTATCTGA

ATGCGAATTTGTATTAGTTTTTCTTCGTAAACAATCTTCTTATTTACGATTAACATCTTCTGGAGCTTTT

CTTGAGCGAACACATTTCTATGGAAAAATAGAATATCGTATAGTAGTGCGCCGTAATTATTTTCAGAAGA

CCCTATGGTTTTTCAAGGATCCCTTCATGCATTATGTTCGATATCAAGGAAAAGCAATTCTGGTTTCAAA

GGGGACTCATCTTCTGATGAAGAAATGGAAATGTCACCTTGTCAATTTCTGGCAATATTATTTTCACTTT

TGGTCTCAACCGTACAGGATTCATATAAACCAATTATCAAACTGTTCTTTCTATTTTCTAGGTTATCTTT

CAAGTGTACTAATAAATCCTTCGGCGGTAAGGAATCAAATGCTAGAGAATTCATTTCTAATAGATACTGT

TATTAAAAAATTCGATACTAGAGTCCCAGTTATTCCTCTTATTAGATCATTGTCTAAAGCTAAATTTTGT

ACCGTATCGGGGCATCCCATTAGTAAGCCGATCTGGGCCGATTTATCAGATTGCGATATTATTGATCGAT

TTGGTCGGATATGTAGAAATCTTTCTCATTATCACAGTGGATCCTCAAAAAAACAGAGTTTGTATCGAAT

AAAGTATATACTTCGATTTTCGTGTGCTAGAACTTTGGCTCGTAAACATAAAAGTATGGTACGCGCTTTT

TTGCAAAGATTAGGTTCGGGATTATTAGAAGAATTTTTTATGGAAGAAGAACAAGTTGTTTCTTTGATCT

TCCCAAAAACAACTTCTTTTTCTTTACATGAATCACATATAGAACGTATTTGGTATTTGGATATTATCCG

TATCAATGACCTGGTGAATTATTCATAATTGGTTTGTTGACGTGATGAGACTTATGAATAGAATAGAAAT

GATCTATAAATGATCAAGAGAGAAAAAAATTCATGAATTTTCATTCTGAAATGTTCATTGCAGTAGTGTA

>Copernicia_macroglossa_HQ720266.1

GGTCCCGCCTCTGGTTCAAGTGGAAATGTAAATGGAAGAATTACAAGGATATTTAGAAAGAGATAGATCT

CTGCAACAACACTTTCTATATCCGCTTCTCTTTAAGGAGTATATTTACACATTTCTTCATGATCGTGGTT

TAAATGGTTCGATTTTTTACGAATCCACGGAAATTTTTGGTTATGACAATTTTGGTTATGACAATAAATC

TAGTTCAGTACTTGTGAAACGTTCAATTATTCGAATGTATCAACAGAATTATTTGATTTATTCGGTTAAT

GATTCTAACCAAAATCGATTCGTTGGGCACAACAATTATTTTTATTTTCATTTTTATTCTCAGATGATAT

TGGAAGGTTTTGCAGTCATTGTGGAAATTCCATTCTTGCTGCGATTAGTATCTTCCCTCGAAGAAAAAAA

AATACCAAAATCTCAGAATTTGAATTTACGATCTATTCATTCAATATTTCCCTTTTTGGAGGACAAATTA

TCGCATTTAAATTATGTGTCAGATATACTAATACCTTATCCCATCCATCTGAAAATCTTGGTTCAAATCC

TTCAATTCTGGATCCAAGATGTTCCTTCTTTACATTTATTGCGATTCTTTCTTCACGAATATCATAATTG

GAATAGTCTTATTACTCCGAATAATTCTATTTTTCTTTTTTCAAAAGAAAATAAAAGACTATTTCGGTTC

CCATATAATTCTTATGTATCTGAATGCGAATTTGTATTAGTTTTTCTTCGTAAACAATCTTCTTATTTAC

GATTAACATCTTCTGGAGCTTTTCTTGAGCGAACACATTTCTATGGAAAAATAGAATATCGTATAGTAGT

GCGCCGTAATTATTTTCAGAAGACCCTATGGTTTTTCAAGGATCCCTTCATGCATTATGTTCGATATCAA

GGAAAAGCAATTCTGGTTTCAAAGGGGACTCATCTTCTGATGAAGAAATGGAAATGTCACCTTGTCAATT

TCTGGCAATATTATTTTCACTTTTGGTCTCAACCGTACAGGATTCATATAAACCAATTATCAAACTGTTC

TTTCTATTTTCTAGGTTATCTTTCAAGTGTACTAATAAATCCTTCGGCGGTAAGGAATCAAATGCTAGAG

AATTCATTTCTAATAGATACTGTTATTAAAAAATTCGATACTAGAGTCCCAGTTATTCCTCTTATTAGAT

CATTGTCTAAAGCTAAATTTTGTACCGTATCGGGGCATCCCATTAGTAAGCCGATCTGGGCCGATTTATC

AGATTGCGATATTATTGATCGATTTGGTCGGATATGTAGAAATCTTTCTCATTATCACAGTGGATCCTCA

AAAAAACAGAGTTTGTATCGAATAAAGTATATACTTCGATTTTCGTGTGCTAGAACTTTGGCTCGTAAAC

ATAAAAGTATGGTACGCGCTTTTTTGCAAAGATTAGGTTCGGGATTATTAGAAGAATTTTTTATGGAAGA

AGAACAAGTTGTTTCTTTGATCTTCCCAAAAACAACTTCTTTTTCTTTACATGAATCACATATAGAACGT

ATTTGGTATTTGGATATTATCCGTATCAATGACCTGGTGAATTATTCATAATTGGTTTGTTGACGTGATG

AGACTTATGAATAGAATAGAAATGATCTATAAATGATCAAGAGAGAAAAAAATTCATGAATTTTCATTCT

GAAATGTTCATWGCAGTAGTGTAGTGGTTGAATCAACTGAGTAGTC

>Copernicia_hospita_HQ720264.1

GGTCCCGCCTCTGGTTCAAGTGGAAATGTAAATGGAAGAATTACAAGGATATTTAGAAAGAGATAGATCT

CTGCAACAACACTTTCTATATCCGCTTCTCTTTAAGGAGTATATTTACACATTTCTTCATGATCGTGGTT

TAAATGGTTCGATTTTTTACGAATCCACGGAAATTTTTGGTTATGACAATTTTGGTTATGACAATAAATC

TAGTTCAGTACTTGTGAAACGTTCAATTATTCGAATGTATCAACAGAATTATTTGATTTATTCGGTTAAT

GATTCTAACCAAAATCGATTCGTTGGGCACAACAATTATTTTTATTTTCATTTTTATTCTCAGATGATAT

TGGAAGGTTTTGCAGTCATTGTGGAAATTCCATTCTTGCTGCGATTAGTATCTTCCCTCGAAGAAAAAAA

AATACCAAAATCTCAGAATTTGAATTTACGATCTATTCATTCAATATTTCCCTTTTTGGAGGACAAATTA

TCGCATTTAAATTATGTGTCARATATACTAATACCTTATCCCATCCATCTGAAAATCTTGGTTCAAATCC

TTCAATTCTGGATCCAAGATGTTCCTTCTTTACATTTATTGCGATTCTTTCTTCACGAATATCATAATTG

GAATAGTCTTATTACTCCGAATAATTCTATTTTTCTTTTTTCAAAAGAAAATAAAAGACTATTTCGGTTC

CCATATAATTCTTATGTATCTGAATGCGAATTTGTATTAGTTTTTCTTCGTAAACAATCTTCTTATTTAC

GATTAACATCTTCTGGAGCTTTTCTTGAGCGAACACATTTCTATGGAAAAATAGAATATCGTATAGTAGT

GCGCCGTAATTATTTTCAGAAGACCCTATGGTTTTTCAAGGATCCCTTCATGCATTATGTTCGATATCAA

GGAAAAGCAATTCTGGTTTCAAAGGGGACTCATCTTCTGATGAAGAAATGGAAATGTCACCTTGTCAATT

TCTGGCAATATTATTTTCACTTTTGGTCTCAACCGTACAGGATTCATATAAACCAATTATCAAACTGTTC

TTTCTATTTTCTAGGTTATCTTTCAAGTGTACTAATAAATCCTTCGGCGGTAAGGAATCAAATGCTAGAG

AATTCATTTCTAATAGATACTGTTATTAAAAAATTCGATACTAGAGTCCCAGTTATTCCTCTTATTAGAT

CATTGTCTAAAGCTAAATTTTGTACCGTATCGGGGCATCCCATTAGTAAGCCGATCTGGGCCGATTTATC

AGATTGCGATATTATTGATCGATTTGGTCGGATATGTAGAAATCTTTCTCATTATCACAGTGGATCCTCA

AAAAAACAGAGTTTGTATCGAATAAAGTATATACTTCGATTTTCGTGTGCTAGAACTTTGGCTCGTAAAC

ATAAAAGTATGGTACGCGCTTTTTTGCAAAGATTAGGTTCGGGATTATTAGAAGAATTTTTTATGGAAGA

AGAACAAGTTGTTTCTTTGATCTTCCCAAAAACAACTTCTTTTTCTTTACATGAATCACATATAGAACGT

ATTTGGTATTTGGATATTATCCGTATCAATGACCTGGTGAATTATTCATAATTGGTTTGTTGACGTGATG

AGACTTATGAATAGAATAGAAATGATCTATAAATGATCAAGAGAGAAAAAAATTCATGAATTTTAATTCT

GAAATGTTCATTGCAGTAGTGTAGTGGTTGAATCAACTGAGTAGTCAAAATTATTATACTTTCTT

>Copernicia_fallaensis_HQ720262.1

AAATGAATAGGTCCCGCCTCTGGTTCGTAAATGGAAGAATTACAAGGATATTTAGAAAGAGATAGATCTC

TGCAACAACACTTTCTATATCCGCTTCTCTTTAAGGAGTATATTTACACATTTCTTCATGATCGTGGTTT

AAATGGTTCGATTTTTTACGAATCCACGGAAATTTTTGGTTATGACAATTTTGGTTATGACAATAAATCT

AGTTCAGTACTTGTGAAACGTTCAATTATTCGAATGTATCAACAGAATTATTTGATTTATTCGGTTAATG

ATTCTAACCAAAATCGATTCGTTGGGCACAACAATTATTTTTATTTTCATTTTTATTCTCAGATGATATT

GGAAGGTTTTGCAGTCATTGTGGAAATTCCATTCTTGCTGCGATTAGTATCTTCCCTCGAAGAAAAAAAA

ATACCAAAATCTCAGAATTTGAATTTACGATCTATTCATTCAATATTTCCCTTTTTGGAGGACAAATTAT

CGCATTTAAATTATGTGTCAGATATACTAATACCTTATCCCATCCATCTGAAAATCTTGGTTCAAATCCT

TCAATTCTGGATCCAAGATGTTCCTTCTTTACATTTATTGCGATTCTTTCTTCACGAATATCATAATTGG

AATAGTCTTATTACTCCGAATAATTCTATTTTTCTTTTTTCAAAAGAAAATAAAAGACTATTTCGGTTCC

CATATAATTCTTATGTATCTGAATGCGAATTTGTATTAGTTTTTCTTCGTAAACAATCTTCTTATTTACG

ATTAACATCTTCTGGAGCTTTTCTTGAGCGAACACATTTCTATGGAAAAATAGAATATCGTATAGTAGTG

CGCCGTAATTATTTTCAGAAGACCCTATGGTTTTTCAAGGATCCCTTCATGCATTATGTTCGATATCAAG

GAAAAGCAATTCTGGTTTCAAAGGGGACTCATCTTCTGATGAAGAAATGGAAATGTCACCTTGTCAATTT

CTGGCAATATTATTTTCACTTTTGGTCTCAACCGTACAGGATTCATATAAACCAATTATCAAACTGTTCT

TTCTATTTTCTAGGTTATCTTTCAAGTGTACTAATAAATCCTTCGGCGGTAAGGAATCAAATGCTAGAGA

ATTCATTTCTAATAGATACTGTTATTAAAAAATTCGATACTAGAGTCCCAGTTATTCCTCTTATTAGATC

ATTGTCTAAAGCTAAATTTTGTACCGTATCGGGGCATCCCATTAGTAAGCCGATCTGGGCCGATTTATCA

GATTGCGATATTATTGATCGATTTGGTCGGATATGTAGAAATCTTTCTCATTATCACAGTGGATCCTCAA

AAAAACAGAGTTTGTATCGAATAAAGTATATACTTCGATTTTCGTGTGCTAGAACTTTGGCTCGTAAACA

TAAAAGTATGGTACGCGCTTTTTTGCAAAGATTAGGTTCGGGATTATTAGAAGAATTTTTTATGGAAGAA

GAACAAGTTGTTTCTTTGATCTTCCCAAAAACAACTTCTTTTTCTTTACATGAATCACATATAGAACGTA

TTTGGTATTTGGATATTATCCGTATCAATGACCTGGTGAATTATTCATAATTGGTTTGTTGACGTGATGA

GACTTATGAATAGAATAGAAATGATCTATAAATGATCAAGAGAGAAAAAAATTCATGAATTTTCATTCTG

AAATGTTCATTGCAGTARTGTAGTGGTTGAATCAACTGAGTAGTCAAAATTATTATACTTTCTTCTCGGG

ACC

>Copernicia_curtissii_HQ720260.1

AAATGTAAATGGAAGAATTACAAGGATATTTAGAAAGAGATAGATCTCTGCAACAACACTTTCTATATCC

GCTTCTCTTTAAGGAGTATATTTACACATTTCTTCATGATCGTGGTTTAAATGGTTCGATTTTTTACGAA

TCCACGGAAATTTTTGGTTATGACAATTTTGGTTATGACAATAAATCTAGTTCAGTACTTGTGAAACGTT

CAATTATTCGAATGTATCAACAGAATTATTTGATTTATTCGGTTAATGATTCTAACCAAAATCGATTCGT

TGGGCACAACAATTATTTTTATTTTCATTTTTATTCTCAGATGATATTGGAAGGTTTTGCAGTCATTGTG

GAAATTCCATTCTTGCTGCGATTAGTATCTTCCCTCGAAGAAAAAAAAATACCAAAATCTCAGAATTTGA

ATTTACGATCTATTCATTCAATATTTCCCTTTTTGGAGGACAAATTATCGCATTTAAATTATGTGTCAGA

TATACTAATACCTTATCCCATCCATCTGAAAATCTTGGTTCAAATCCTTCAATTCTGGATCCAAGATGTT

CCTTCTTTACATTTATTGCGATTCTTTCTTCACGAATATCATAATTGGAATAGTCTTATTACTCCGAATA

ATTCTATTTTTCTTTTTTCAAAAGAAAATAAAAGACTATTTCGGTTCCCATATAATTCTTATGTATCTGA

ATGCGAATTTGTATTAGTTTTTCTTCGTAAACAATCTTCTTATTTACGATTAACATCTTCTGGAGCTTTT

CTTGAGCGAACACATTTCTATGGAAAAATAGAATATCGTATAGTAGTGCGCCGTAATTATTTTCAGAAGA

CCCTATGGTTTTTCAAGGATCCCTTCATGCATTATGTTCGATATCAAGGAAAAGCAATTCTGGTTTCAAA

GGGGACTCATCTTCTGATGAAGAAATGGAAATGTCACCTTGTCAATTTCTGGCAATATTATTTTCACTTT

TGGTCTCAACCGTACAGGATTCATATAAACCAATTATCAAACTGTTCTTTCTATTTTCTAGGTTATCTTT

CAAGTGTACTAATAAATCCTTCGGCGGTAAGGAATCAAATGCTAGAGAATTCATTTCTAATAGATACTGT

TATTAAAAAATTCGATACTAGAGTCCCAGTTATTCCTCTTATTAGATCATTGTCTAAAGCTAAATTTTGT

ACCGTATCGGGGCATCCCATTAGTAAGCCGATCTGGGCCGATTTATCAGATTGCGATATTATTGATCGAT

TTGGTCGGATATGTAGAAATCTTTCTCATTATCACAGTGGATCCTCAAAAAAACAGAGTTTGTATCGAAT

AAAGTATATACTTCGATTTTCGTGTGCTAGAACTTTGGCTCGTAAACATAAAAGTATGGTACGCGCTTTT

TTGCAAAGATTAGGTTCGGGATTATTAGAAGAATTTTTTATGGAAGAAGAACAAGTTGTTTCTTTGATCT

TCCCAAAAACAACTTCTTTTTCTTTACATGAATCACATATAGAACGTATTTGGTATTTGGATATTATCCG

TATCAATGACCTGGTGAATTATTCATAATTGGTTTGTTGACGTGATGAGACTTATGAATAGAATAGAAAT

GATCTATAAATGATCAAGAGAGAAAAAAATTCATGAATTTTCATTCTGAAATGTTCATTGCAGTAGTGTA

>Copernicia_baileyana_HQ720258.1

AAATGTAAATGGAAGAATTACAAGGATATTTAGAAAGAGATAGATCTCTGCAACAACACTTTCTATATCC

GCTTCTCTTTAAGGAGTATATTTACACATTTCTTCATGATCGTGGTTTAAATGGTTCGATTTTTTACGAA

TCCACGGAAATTTTTGGTTATGACAATTTTGGTTATGACAATAAATCTAGTTCAGTACTTGTGAAACGTT

CAATTATTCGAATGTATCAACAGAATTATTTGATTTATTCGGTTAATGATTCTAACCAAAATCGATTCGT

TGGGCACAACAATTATTTTTATTTTCATTTTTATTCTCAGATGATATTGGAAGGTTTTGCAGTCATTGTG

GAAATTCCATTCTTGCTGCGATTAGTATCTTCCCTCGAAGAAAAAAAAATACCAAAATCTCAGAATTTGA

ATTTACGATCTATTCATTCAATATTTCCCTTTTTGGAGGACAAATTATCGCATTTAAATTATGTGTCAGA

TATACTAATACCTTATCCCATCCATCTGAAAATCTTGGTTCAAATCCTTCAATTCTGGATCCAAGATGTT

CCTTCTTTACATTTATTGCGATTCTTTCTTCACGAATATCATAATTGGAATAGTCTTATTACTCCGAATA

ATTCTATTTTTCTTTTTTCAAAAGAAAATAAAAGACTATTTCGGTTCCCATATAATTCTTATGTATCTGA

ATGCGAATTTGTATTAGTTTTTCTTCGTAAACAATCTTCTTATTTACGATTAACATCTTCTGGAGCTTTT

CTTGAGCGAACACATTTCTATGGAAAAATAGAATATCGTATAGTAGTGCGCCGTAATTATTTTCAGAAGA

CCCTATGGTTTTTCAAGGATCCCTTCATGCATTATGTTCGATATCAAGGAAAAGCAATTCTGGTTTCAAA

GGGGACTCATCTTCTGATGAAGAAATGGAAATGTCACCTTGTCAATTTCTGGCAATATTATTTTCACTTT

TGGTCTCAACCGTACAGGATTCATATAAACCAATTATCAAACTGTTCTTTCTATTTTCTAGGTTATCTTT

CAAGTGTACTAATAAATCCTTCGGCGGTAAGGAATCAAATGCTAGAGAATTCATTTCTAATAGATACTGT

TATTAAAAAATTCGATACTAGAGTCCCAGTTATTCCTCTTATTAGATCATTGTCTAAAGCTAAATTTTGT

ACCGTATCGGGGCATCCCATTAGTAAGCCGATCTGGGCCGATTTATCAGATTGCGATATTATTGATCGAT

TTGGTCGGATATGTAGAAATCTTTCTCATTATCACAGTGGATCCTCAAAAAAACAGAGTTTGTATCGAAT

AAAGTATATACTTCGATTTTCGTGTGCTAGAACTTTGGCTCGTAAACATAAAAGTATGGTACGCGCTTTT

TTGCAAAGATTAGGTTCGGGATTATTAGAAGAATTTTTTATGGAAGAAGAACAAGTTGTTTCTTTGATCT

TCCCAAAAACAACTTCTTTTTCTTTACATGAATCACATATAGAACGTATTTGGTATTTGGATATTATCCG

TATCAATGACCTGGTGAATTATTCATAATTGGTTTGTTGACGTGATGAGACTTATGAATAGAATAGAAAT

GATCTATAAATGATCAAGAGAGAAAAAAATTCATGAATTTTCATTCTGAAATGTTCATTGCAGTAGTGTA

>Copernicia_tectorum_HQ720269.1

GAAATGTAAATGGAAGAATTACAAGGATATTTAGAAAGAGATAGATCTCTGCAACAACACTTTCTATATC

CGCTTCTCTTTAAGGAGTATATTTACACATTTCTTCATGATCGTGGTTTAAATGGTTCGATTTTTTACGA

ATCCACGGAAATTTTTGGTTATGACAATTTTGGTTATGACAATAAATCTAGTTCAGTACTTGTGAAACGT

TCAATTATTCGAATGTATCAACAGAATTATTTGATTTATTCGGTTAATGATTCTAACCAAAATCGATTCG

TTGGGCACAACAATTATTTTTATTTTCATTTTTTTTCTCAGATGATATTGGAAGGTTTTGCAGTCAGTGT

GGAAATTCCATTCTTGCTGCGATTARTATCTTCCCTCGAAGAAAAAAAAATACCAAAATCTCARAATTTG

AATTTACGATCTATTCATTCAATATTTCCCTTTTTGGAGGACAAATTATCGCATTTAAATTATGTGTCAG

ATATACTAATACCTTATCCCATCCATCTGAAAATCTTGGTTCAAATCCTTCAATTCTGGATCCAAGATGT

TCCTTCTTTACATTTATTGCGATTCTTTCTTCACGAATATCATAATTGGAATAGTCTTATTACTCCGAAT

AATTCTATTTTTCTTTTTTCAAAAGAAAATAAAAGACTATTTCGGTTCCCATATAATTCTTATGTATCTG

AATGCGAATTTGTATTAGTTTTTCTTCGTAAACAATCTTCTTATTTACRATTAACATCTTCTGGAGCTTT

TCTTGAGCGGACACATTTCTATGGAAAAATAGAATATCGTATAGTAGTGCGCCGTAATTATTTTCAGAAG

ACCCTATGGTTTTTCAAGGATCCCTTCATGCATTATGTTCGATATCAAGGAAAAGCAATTCTGGTTTCAA

AGGGGACTCATCTTCTGATGAAGAAATGGAAATGTCACCTTGTCAATTTCTGGCAATATTATTTTCACTT

TTGGTCTCAACCGTACAGGATTCATATAAACCAATTATCAAACTGTTCTTTCTATTTTCTAGGTTATCTT

TCAAGTGTACTAATAAATCCTTCGGCGGTAAGGAATCAAATGCTAGAGAATTCATTTCTAATAGATACTG

TTATTAAAAAATTCGATACCAGAGTCCCAGTTATTCCTCTTATTAGATCATTGTCTAAAGCTAAATTTTG

TACCGTATCGGGGCATCCCATTAGTAAGCCGATTTGGGCCGATTTATCAGATTGCGATATTATTGATCGA

TTTGGTCGGATATGTAGAAATCTTTCTCATTATCACAGTGGATCCTCAAAAAAACAGAGTTTGTATCGAA

TAAAGTATATACTTCGATTTTCGTGTGCTAGAACTTTGGCTCGTAAACATAAAAGTATGGTACGCGCTTT

TTTGCAAAGATTAGGTTCGGGATTATTAGAAGAATTTTTTATGGAAGAAGAACAAGTTGTTTCTTTGATC

TTCCCAAAAACAACTTCTTTTTCTTTACATGAATCACATATAGAACGTATTTGGTATTTGGATATTATCC

GTATCAATGACCTGGTGAATTATTCATAATTGGTTTGTTGACGTGATGAGACTTATGAATAGAATAGAAA

TGATCTATAAATGATCAAGAGAGAAAAAAATTCATGAATTTTCATTCTGAAATGTTCATTGCAGTAGTGT

A

>Copernicia_prunifera_HQ720267.1

GGAAATGTAAATGGAAGAATTACAAGGATATTTAGAAAGAGATAGATCTCTGCAACAACACTTTCTATAT

CCGCTTCTCTTTAAGGAGTATATTTACACATTTCTTCATGATCGTGGTTTAAATGGTTCGATTTTTTACG

AATCCACGGAAATTTTTGGTTATGACAATTTTGGTTATGACAATAAATCTAGTTCAGTACTTGTGAAACG

TTCAATTATTCGAATGTATCAACAGAATTATTTGATTTATTCGGTTAATGATTCTAACCAAAATCGATTC

GTCGGGCACAACAATTATTTTTATTTTCATTTTTATTCTCAGATGATATTGGAAGGTTTTGCAGTCAGTG

TGGAAATTCCATTCTTGCTGCGATTAGTATCTTCCCTCGAAGAAAAAAAAATACCAAAATATCAGAATTT

GAATTTACGATCTATTCATTCAATATTTCCCTTTTTGGAGGACAAATTATCGCATTTAAATTATGTGTCA

GATATACTAATACCTTATCCCATCCATCTGAAAATCTTGGTTCAAATCCTTCAATTCTGGATCCAAGATG

TTCCTTCTTTACATTTATTGCGATTCTTTCTTCACGAATATCATAATTGGAATAGTTTTATTACTCCGAA

TAATTCTATTTTTCTTTTTTCAAAAGAAAATAAAAGACTATTTCGGTTCCCATATAATTCTTATGTATCT

GAATGCGAATTTTTATTAGTTTTTCTTCGTAAACAATCTTCTTATTTACGATTAACATCTTCTGGAGCTT

TTCTTGAGCGAACACATTTCTATGGAAAAATAGAATATCGTATAGTAGTGCGCCGTAATTATTTTCAGAA

GACCCTATGGTTTTTCAAGGATCCCTTCATGCATTATGTTCGATATCAAGGAAAAGCAATTCTGGTTTCA

AAGGGGACTCATCTTCTGATGAAGAAATGGAAATGTCACCTTGTCAATTTCTGGCAATATTATTTTCACT

TTTGGTCTCAACCGTACAGGATTCATATAAACCAATTATCAAACTGTTCTTTCTATTTTCTAGGTTATCT

TTCAAGTGTACTAATAAATCCTTCGGCGGTAAGGAATCAAATGCTAGAGAATTCATTTCTAATAGATACT

GTTATTAAAAAATTCGATACCAGAGTCCCAGTTATTCCTCTTATTAGATCATTGTCTAAAGCTAAATTTT

GTACCGTATCGGGGCATCCCATTAGTAAGCCGATCTGGGCCGATTTATCAGATTGCGATATTTTTGATCG

ATTTGGTCGGATATGTAGAAATCTTTCTCATTATCACAGTGGATCCTCAAAAAAACAGAGTTTGTATCGA

ATAAAGTATATACTTCGATTTTCGTGTGCTAGAACTTTGGCTCGTAAACATAAAAGTATGGTACGCGCTT

GTTTGCAAAGATTAGGTTCGGGATTATTAGAAGAATTTTTTATGGAAGAAGAACAAGTTGTTTCTTTGAT

CTTCCCAAAAACAACTTCTTTTTCTTTACATGAATCACATATAGAACGTATTTGGTATTTGGATATTATC

CGTATCAATGACCTGGTGAATTATTCATAATTGGTTTGTTGACGTGATGAGACTTATGAATAGAATAGAA

ATGATCTATAAATGATCAAGAGAGAAAAAAATTCATGAATTTTCATTCTGAAATGTTCATTGCAGTAGTG

TAGTGGTTGAATCAACTGAGTAGTCAAAAT

>Copernicia_macroglossa_HQ720265.1

CCGCCTCTGGTTCAGTAAATGGAAGAATTACAAGGATATTTAGAAAGAGATAGATCTCTGCAACAACACT

TTCTATATCCGCTTCTCTTTAAGGAGTATATTTACACATTTCTTCATGATCGTGGTTTAAATGGTTCGAT

TTTTTACGAATCCACGGAAATTTTTGGTTATGACAATTTTGGTTATGACAATAAATCTAGTTCAGTACTT

GTGAAACGTTCAATTATTCGAATGTATCAACAGAATTATTTGATTTATTCGGTTAATGATTCTAACCAAA

ATCGATTCGTTGGGCACAACAATTATTTTTATTTTCATTTTTATTCTCAGATGATATTGGAAGGTTTTGC

AGTCATTGTGGAAATTCCATTCTTGCTGCGATTAGTATCTTCCCTCGAAGAAAAAAAAATACCAAAATCT

CAGAATTTGAATTTACGATCTATTCATTCAATATTTCCCTTTTTGGAGGACAAATTATCGCATTTAAATT

ATGTGTCAGATATACTAATACCTTATCCCATCCATCTGAAAATCTTGGTTCAAATCCTTCAATTCTGGAT

CCAAGATGTTCCTTCTTTACATTTATTGCGATTCTTTCTTCACGAATATCATAATTGGAATAGTCTTATT

ACTCCGAATAATTCTATTTTTCTTTTTTCAAAAGAAAATAAAAGACTATTTCGGTTCCCATATAATTCTT

ATGTATCTGAATGCGAATTTGTATTAGTTTTTCTTCGTAAACAATCTTCTTATTTACGATTAACATCTTC

TGGAGCTTTTCTTGAGCGAACACATTTCTATGGAAAAATAGAATATCGTATAGTAGTGCGCCGTAATTAT

TTTCAGAAGACCCTATGGTTTTTCAAGGATCCCTTCATGCATTATGTTCGATATCAAGGAAAAGCAATTC

TGGTTTCAAAGGGGACTCATCTTCTGATGAAGAAATGGAAATGTCACCTTGTCAATTTCTGGCAATATTA

TTTTCACTTTTGGTCTCAACCGTACAGGATTCATATAAACCAATTATCAAACTGTTCTTTCTATTTTCTA

GGTTATCTTTCAAGTGTACTAATAAATCCTTCGGCGGTAAGGAATCAAATGCTAGAGAATTCATTTCTAA

TAGATACTGTTATTAAAAAATTCGATACTAGAGTCCCAGTTATTCCTCTTATTAGATCATTGTCTAAAGC

TAAATTTTGTACCGTATCGGGGCATCCCATTAGTAAGCCGATCTGGGCCGATTTATCAGATTGCGATATT

ATTGATCGATTTGGTCGGATATGTAGAAATCTTTCTCATTATCACAGTGGATCCTCAAAAAAACAGAGTT

TGTATCGAATAAAGTATATACTTCGATTTTCGTGTGCTAGAACTTTGGCTCGTAAACATAAAAGTATGGT

ACGCGCTTTTTTGCAAAGATTAGGTTCGGGATTATTAGAAGAATTTTTTATGGAAGAAGAACAAGTTGTT

TCTTTGATCTTCCCAAAAACAACTTCTTTTTCTTTACATGAATCACATATAGAACGTATTTGGTATTTGG

ATATTATCCGTATCAATGACCTGGTGAATTATTCATAATTGGTTTGTTGACGTGATGAGACTTATGAATA

GAATAGAAATGATCTATAAATGATCAAGAGAGAAAAAAATTCATGAATTTTCATTCTGAAATGTTCATTG

CAGTAGTGTAGTGGTTGAATCAACTGAGTAGTC

>Copernicia_glabrescens_HQ720263.1

GAAATGTAAATGGAAGAATTACAAGGATATTTAGAAAGAGATAGATCTCTGCAACAACACTTTCTATATC

CGCTTCTCTTTAAGGAGTATATTTACACATTTCTTCATGATCGTGGTTTAAATGGTTCGATTTTTTACGA

ATCCACGGAAATTTTTGGTTATGACAATTTTGGTTATGACAATAAATCTAGTTCAGTACTTGTGAAACGT

TCAATTATTCGAATGTATCAACAGAATTATTTGATTTATTCGGTTAATGATTCTAACCAAAATCGATTCG

TTGGGCACAACAATTATTTTTATTTTCATTTTTATTCTCAGATGATATTGGAAGGTTTTGCAGTCATTGT

GGAAATTCCATTCTTGCTGCGATTAGTATCTTCCCTCGAAGAAAAAAAAATACCAAAATCTCAGAATTTG

AATTTACGATCTATTCATTCAATATTTCCCTTTTTGGAGGACAAATTATCGCATTTAAATTATGTGTCAG

ATATACTAATACCTTATCCCATCCATCTGAAAATCTTGGTTCAAATCCTTCAATTCTGGATCCAAGATGT

TCCTTCTTTACATTTATTGCGATTCTTTCTTCACGAATATCATAATTGGAATAGTCTTATTACTCCGAAT

AATTCTATTTTTCTTTTTTCAAAAGAAAATAAAAGACTATTTCGGTTCCCATATAATTCTTATGTATCTG

AATGCGAATTTGTATTAGTTTTTCTTCGTAAACAATCTTCTTATTTACGATTAACATCTTCTGGAGCTTT

TCTTGAGCGAACACATTTCTATGGAAAAATAGAATATCGTATAGTAGTGCGCCGTAATTATTTTCAGAAG

ACCCTATGGTTTTTCAAGGATCCCTTCATGCATTATGTTCGATATCAAGGAAAAGCAATTCTGGTTTCAA

AGGGGACTCATCTTCTGATGAAGAAATGGAAATGTCACCTTGTCAATTTCTGGCAATATTATTTTCACTT

TTGGTCTCAACCGTACAGGATTCATATAAACCAATTATCAAACTGTTCTTTCTATTTTCTAGGTTATCTT

TCAAGTGTACTAATAAATCCTTCGGCGGTAAGGAATCAAATGCTAGAGAATTCATTTCTAATAGATACTG

TTATTAAAAAATTCGATACTAGAGTCCCAGTTATTCCTCTTATTAGATCATTGTCTAAAGCTAAATTTTG

TACCGTATCGGGGCATCCCATTAGTAAGCCGATCTGGGCCGATTTATCAGATTGCGATATTATTGATCGA

TTTGGTCGGATATGTAGAAATCTTTCTCATTATCACAGTGGATCCTCAAAAAAACAGAGTTTGTATCGAA

TAAAGTATATACTTCGATTTTCGTGTGCTAGAACTTTGGCTCGTAAACATAAAAGTATGGTACGCGCTTT

TTTGCAAAGATTAGGTTCGGGATTATTAGAAGAATTTTTTATGGAAGAAGAACAAGTTGTTTCTTTGATC

TTCCCAAAAACAACTTCTTTTTCTTTACATGAATCACATATAGAACGTATTTGGTATTTGGATATTATCC

GTATCAATGACCTGGTGAATTATTCATAATTGGTTTGTTGACGTGATGAGACTTATGAATAGAATAGAAA

TGATCTATAAATGATCAAGAGAGAAAAAAATTCATGAATTTTCATTCTGAAATGTTCATTGCAGTAGTGT

AGT

>Copernicia_ekmanii_HQ720261.1

GTAAATGGAAGAATTACAAGGATATTTAGAAAGAGATAGATCTCTGCAACAACACTTTCTATATCCGCTT

CTCTTTAAGGAGTATATTTACACATTTCTTCATGATCGTGGTTTAAATGGTTCGATTTTTTACGAATCCA

CGGAAATTTTTGGTTATGACAATTTTGGTTATGACAATAAATCTAGTTCAGTACTTGTGAAACGTTCAAT

TATTCGAATGTATCAACAGAATTATTTGATTTATTCGGTTAATGATTCTAACCAAAATCGATTCGTTGGG

CACAACAATTATTTTTATTTTCATTTTTATTCTCAGATGATATTGGAAGGTTTTGCAGTCATTGTGGAAA

TTCCATTCTTGCTGCGATTAGTATCTTCCCTCGAAGAAAAAAAAATACCAAAATCTCAGAATTTGAATTT

ACGATCTATTCATTCAATATTTCCCTTTTTGGAGGACAAATTATCGCATTTAAATTATGTGTCAGATATA

CTAATACCTTATCCCATCCATCTGAAAATCTTGGTTCAAATCCTTCAATTCTGGATCCAAGATGTTCCTT

CTTTACATTTATTGCGATTCTTTCTTCACGAATATCATAATTGGAATAGTCTTATTACTCCGAATAATTC

TATTTTTCTTTTTTCAAAAGAAAATAAAAGACTATTTCGGTTCCCATATAATTCTTATGTATCTGAATGC

GAATTTGTATTAGTTTTTCTTCGTAAACAATCTTCTTATTTACGATTAACATCTTCTGGAGCTTTTCTTG

AGCGAACACATTTCTATGGAAAAATAGAATATCGTATAGTAGTGCGCCGTAATTATTTTCAGAAGACCCT

ATGGTTTTTCAAGGATCCCTTCATGCATTATGTTCGATATCAAGGAAAAGCAATTCTGGTTTCAAAGGGG

ACTCATCTTCTGATGAAGAAATGGAAATGTCACCTTGTCAATTTCTGGCAATATTATTTTCACTTTTGGT

CTCAACCGTACAGGATTCATATAAACCAATTATCAAACTGTTCTTTCTATTTTCTAGGTTATCTTTCAAG

TGTACTAATAAATCCTTCGGCGGTAAGGAATCAAATGCTAGAGAATTCATTTCTAATAGATACTGTTATT

AAAAAATTCGATACTAGAGTCCCAGTTATTCCTCTTATTAGATCATTGTCTAAAGCTAAATTTTGTACCG

TATCGGGGCATCCCATTAGTAAGCCGATCTGGGCCGATTTATCAGATTGCGATATTATTGATCGATTTGG

TCGGATATGTAGAAATCTTTCTCATTATCACAGTGGATCCTCAAAAAAACAGAGTTTGTATCGAATAAAG

TATATACTTCGATTTTCGTGTGCTAGAACTTTGGCTCGTAAACATAAAAGTATGGTACGCGCTTTTTTGC

AAAGATTAGGTTCGGGATTATTAGAAGAATTTTTTATGGAAGAAGAACAAGTTGTTTCTTTGATCTTCCC

AAAAACAACTTCTTTTTCTTTACATGAATCACATATAGAACGTATTTGGTATTTGGATATTATCCGTATC

AATGACCTGGTGAATTATTCAGAATTGGTTTGTTGACGTGATGAGACTTATGAATAGAATAGAAATGATC

TATAAATGATCAAGAGAGAAAAAAATTCATGAATTTTCATTCTGAAATGTTCATTGCAGTAGTG

>Copernicia_alba_HQ720257.1

GAAATGTAAATGGAAGAATTACAAGGATATTTAGAAAGAGATAGATCTCTGCAACAACACTTTCTATATC

CGCTTCTCTTTAAGGAGTATATTTACACATTTCTTCATGATCGTGGTTTAAATGGTTCGATTTTTTACGA

ATCCACGGAAATTTTTGGTTATGACAATTTTGGTTATGACAATAAATCTAGTTCAGTACTTGTGAAACGT

TCAATTATTCGAATGTATCAACAGAATTATTTGATTTATTCGGTTAATGATTCTAACCAAAATCGATTCG

TCGGGCACAACAATTATTTTTATTTTCATTTTTATTCTCAGATGATATTGGAAGGTTTTGCAGTCAGTGT

GGAAATTCCATTCTTGCTGCGATTAGTATCTTCCCTCGAAGAAAAAAAAATACCAAAATCTCAGAATTTG

AATTTACGATCTATTCATTCAATATTTCCCTTTTTGGAGGACAAATTATCGCATTTAAATTATGTGTCAG

ATATACTAATACCTTATCCCATCCATCTGAAAATCTTGGTTCAAATCCTTCAATTCTGGATCCAAGATGT

TCCTTCTTTACATTTATTGCGATTCTTTCTTCACGAATATCATAATTGGAATAGTTTTATTACTCCGAAT

AATTCTATTTTTCTTTTTTCAAAAGAAAATAAAAGACTATTTCGGTTCCCATATAATTCTTATGTATCTG

AATGCGAATTTGTATTAGTTTTTCTTCGTAAACAATCTTCTTATTTACGATTAACATCTTCTGGAGCTTT

TCTTGAGCGAACACATTTCTATGGAAAAATAGAATATCGTATAGTAGTGCGCCGTAATTATTTTCAGAAG

ACCCTATGGTTTTTCAAGGATCCCTTCATGCATTATGTTCGATATCAAGGAAAAGCAATTCTGGTTTCAA

AGGGGACTCATCTTCTGATGAAGAAATGGAAATGTCACCTTGTCAATTTCTGGCAATATTATTTTCACTT

TTGGTCTCAACCGTACAGGATTCATATAAACCAATTATCAAACTGTTCTTTCTATTTTCTAGGTTATCTT

TCAAGTGTACTAATAAATCCTTCGGCGGTAAGGAATCAAATGCTAGAGAATTCATTTCTAATAGATACTG

TTATTAAAAAATTCGATACCAGAGTCCCAGTTATTCCTCTTATTAGATCATTGTCTAAAGCTAAATTTTG

TACCGTATCGGGGCATCCCATTAGTAAGCCGATCTGGGCCGATTTATCAGATTGCGATATTATTGATCGA

TTTGGTCGGATATGTAGAAATCTTTCTCATTATCACAGTGGATCCTCAAAAAAACAGAGTTTGTATCGAA

TAAAGTATATACTTCGATTTTCGTGTGCTAGAACTTTGGCTCGTAAACATAAAAGTATGGTACGCGCTTG

TTTGCAAAGATTAGGTTCGGGATTATTAGAAGAATTTTTTATGGAAGAAGAACAAGTTGTTTCTTTGATC

TTCCCAAAAACAACTTCTTTTTCTTTACATGAATCACATATAGAACGTATTTGGTATTTGGATATTATCC

GTATCAATGACCTGGTGAATTATTCATAATTGGTTTGTTGACGTGATGAGACTTATGAATAGAATAGAAA

TGATCTATAAATGATCAAGAGAGAAAAAAATTCATGAATTTTCATTCTGAAATGTTCATTGCAGTAGTGT

AGT

>Copernicia_berteroana_HQ720259.1

AAATGTAAATGGAAGAATTACAAGGATATTTAGAAAGAGATAGATCTCTGCAACAACACTTTCTATATCC

GCTTCTCTTTAAGGAGTATATTTACACATTTCTTCATGATCGTGGTTTAAATGGTTCGATTTTTTACGAA

TCCACGGAAATTTTTGGTTATGACAATTTTGGTTATGACAATAAATCTAGTTCAGTACTTGTGAAACGTT

CAATTATTCGAATGTATCAACAGAATTATTTGATTTATTCGGTTAATGATTCTAACCAAAATCGATTCGT

TGGGCACAACAATTATTTTTATTTTCATTTTTATTCTCAGATGATATTGGAAGGTTTTGCAGTCATTGTG

GAAATTCCATTCTTGCTGCGATTAGTATCTTCCCTCGAAGAAAAAAAAATACCAAAATCTCAGAATTTGA

ATTTACGATCTATTCATTCAATATTTCCCTTTTTGGAGGACAAATTATCGCATTTAAATTATGTGTCAGA

TATACTAATACCTTATCCCATCCATCTGAAAATCTTGGTTCAAATCCTTCAATTCTGGATCCAAGATGTT

CCTTCTTTACATTTATTGCGATTCTTTCTTCACGAATATCATAATTGGAATAGTCTTATTACTCCGAATA

ATTCTATTTTTCTTTTTTCAAAAGAAAATAAAAGACTATTTCGGTTCCCATATAATTCTTATGTATCTGA

ATGCGAATTTGTATTAGTTTTTCTTCGTAAACAATCTTCTTATTTACGATTAACATCTTCTGGAGCTTTT

CTTGAGCGAACACATTTCTATGGAAAAATAGAATATCGTATAGTAGTGCGCCGTAATTATTTTCAGAAGA

CCCTATGGTTTTTCAAGGATCCCTTCATGCATTATGTTCGATATCAAGGAAAAGCAATTCTGGTTTCAAA

GGGGACTCATCTTCTGATGAAGAAATGGAAATGTCACCTTGTCAATTTCTGGCAATATTATTTTCACTTT

TGGTCTCAACCGTACAGGATTCATATAAACCAATTATCAAACTGTTCTTTCTATTTTCTAGGTTATCTTT

CAAGTGTACTAATAAATCCTTCGGCGGTAAGGAATCAAATGCTAGAGAATTCATTTCTAATAGATACTGT

TATTAAAAAATTCGATACTAGAGTCCCAGTTATTCCTCTTATTAGATCATTGTCTAAAGCTAAATTTTGT

ACCGTATCGGGGCATCCCATTAGTAAGCCGATCTGGGCCGATTTATCAGATTGCGATATTATTGATCGAT

TTGGTCGGATATGTAGAAATCTTTCTCATTATCACAGTGGATCCTCAAAAAAACAGAGTTTGTATCGAAT

AAAGTATATACTTCGATTTTCGTGTGCTAGAACTTTGGCTCGTAAACATAAAAGTATGGTACGCGCTTTT

TTGCAAAGATTAGGTTCGGGATTATTAGAAGAATTTTTTATGGAAGAAGAACAAGTTGTTTCTTTGATCT

TCCCAAAAACAACTTCTTTTTCTTTACATGAATCACATATAGAACGTATTTGGTATTTGGATATTATCCG

TATCAATGACCTGGTGAATTATTCAGAATTGGTTTGTTGACGTGATGAGACTTATGAATAGAATAGAAAT

GATCTATAAATGATCAAGAGAGAAAAAAATTCATGAATTTTCATTCTGAAATGTTCATTGCAGTAGTGTA

>Copernicia_prunifera_AM114582.1

GACCATATTGCACTATGTATCATTTGATAACCCCAAAAATGAAATAGGTCCCGCCTCTGGTTCAAGTGGA

AATGTAAATGGAAGAATTACAAGGATATTTAGAAAGAGATAGATCTCTGCAACAACACTTTCTATATCCG

CTTCTCTTTAAGGAGTATATTTACACATTTCTTCATGATCGTGGTTTAAATGGTTCGATTTTTTACGAAT

CCACGGAAATTTTTGGTTATGACAATTTTGGTTATGACAATAAATCTAGTTCAGTACTTGTGAAACGTTC

AATTATTCGAATGTATCAACAGAATTATTTGATTTATTCGGTTAATGATTCTAACCAAAATCGATTCGTT

GGGCACAACAATTATTTTTATTTTCATTTTTATTCTCAGATGATATTGGAAGGTTTTGCAGTCATTGTGG

AAATTCCATTCTTGCTGCGATTAGTATCTTCCCTCGAAGAAAAAAAAATACCAAAATCTCAGAATTTGAA

TTTACGATCTATTCATTCAATATTTCCCTTTTTGGAGGACAAATTATCGCATTTAAATTATGTGTCAGAT

ATACTAATACCTTATCCCATCCATCTGAAAATCTTGGTTCAAATCCTTCAATTCTGGATCCAAGATGTTC

CTTCTTTACATTTATTGCGATTCTTTCTTCACGAATATCATAATTGGAATAGTCTTATTACTCCGAATAA

TTCTATTTTTCTTTTTTCAAAAGAAAATAAAAGACTATTTCGGTTCCCATATAATTCTTATGTATCTGAA

TGCGAATTTGTATTAGTTTTTCTTCGTAAACAATCTTCTTATTTACGATTAACATCTTCTGGAGCTTTTC

TTGAGCGAACACATTTCTATGGAAAAATAGAATATCGTATAGTAGTGCGCCGTAATTATTTTCAGAAGAC

CCTATGGTTTTTCAAGGATCCCTTCATGCATTATGTTCGATATCAAGGAAAAGCAATTCTGGTTTCAAAG

GGGACTCATCTTCTGATGAAGAAATGGAAATGTCACCTTGTCAATTTCTGGCAATATTATTTTCACTTTT

GGTCTCAACCGTACAGGATTCATATAAACCAATTATCAAACTGTTCTTTCTATTTTCTAGGTTATCTTTC

AAGTGTACTAATAAATCCTTCGGCGGTAAGGAATCAAATGCTAGAGAATTCATTTCTAATAGATACTGTT

ATTAAAAAATTCGATACTAGAGTCCCAGTTATTCCTCTTATTAGATCATTGTCTAAAGCTAAATTTTGTA

CCGTATCGGGGCATCCCATTAGTAAGCCGATCTGGGCCGATTTATCAGATTGCGATATTATTGATCGATT

TGGTCGGATATGTAGAAATCTTTCTCATTATCACAGTGGATCCTCAAAAAAACAGAGTTTGTATCGAATA

AAGTATATACTTCGATTTTCGTGTGCTAGAACTTCGGCTCGTAAACATAAAAGTATGGTACGCGCTTTTT

TGCAAAGATTAGGTTCGGGATTATTAGAAGAATTTTTTATGGAAGAAGAACAAGTTGTTTCTTTGATCTT

CCCAAAAACAACTTCTTTTTCTTTACATGAATCACATATAGAACGTATTTGGTATTTGGATATTATCCGT

ATCAATGACCTGGTGAATTATTCATAATTGGTTTGTTGACGTGATGAGACTTATGAATAGAATAGAAATG

ATCTATAAATGATCAAGAGAGAAAAAAATTCATGAATTTTAATTCTGAAATGTTCATTGCAGTAGTGTAG

TGGTTGAATCAACTGAGTAGTCAAAATTATTATACTTTCTTCTCGGGACCCAAGTTTTATATTATACATA

GGTAAAGTC

>Cryosophila_warscewiczii_JQ586728.1

CCTTCTTTACATTTATTGCGATTCTTTCTTCACGAATATCATAATTGGAATAGTCTTATTACTCCGAATA

ATTCTATTTTTCCTTTTTTCCTTTTTTCAAAAGAAAATAAAAGACTATTTCGGTTCCCATATAATTCTTA

TGTATCTGAATGCGAATTTTTATTAGTTTTTCTTCGTAAACAATCTTCTTATTTACGATTAACATCTTCT

GGAGCTTTTCTTGAGCGAACACATTTCTATGGAAAAATAGAACATCTTATAGTAGTGCGCCGTAATTATT

TTCAGAAGACCCTATGGTTCTTCAAGGATCCCTTCATGCATTATGTTCGATATCAAGGAAAAGCAATTCT

GGTTTCAAAGGGGACTCATCTTCTGATGAAGAAATGGAAATGTCACCTTGTCAATTTCTGGCAATATTAT

TTTCACTTTTGGTCTCAACCGTACAGGATCCATATAAAGCAATTATCAAACTGTTCTTTCTATTTTTTAG

GTTATCTTTCAAGTGTACTAATAAATCCTTCGGCGGTAAGGAATCAAATGCTAGAGAATTCATTTCTAAT

AGATACTCTTATTAAAAAATTCGATACCAGAGTCCCAGTTATTCCTCTTATTGGATCATTGTCTAAAGCT

AAATTTTGTACCGTATCGGGGCATCCTATTAGTAAGCCGATCTGGACCGATTTATCAGATTGCGATATTA

TTGATCGATTTGGTCGGATATGTAGAAATCTTTCTCATTATCACAGTGGATCCTCAAAAAAACAGAGTTT

GTATCGAATAA

>Cryosophila_warscewiczii_JQ586729.1

CCTTCTTTACATTTATTGCGATTCTTTCTTCACGAATATCATAATTGGAATAGTCTTATTACTCCGAATA

ATTCTATTTTTCCTTTTTTCCTTTTTTCAAAAGAAAATAAAAGACTATTTCGGTTCCCATATAATTCTTA

TGTATCTGAATGCGAATTTTTATTAGTTTTTCTTCGTAAACAATCTTCTTATTTACGATTAACATCTTCT

GGAGCTTTTCTTGAGCGAACACATTTCTATGGAAAAATAGAACATCTTATAGTAGTGCGCCGTAATTATT

TTCAGAAGACCCTATGGTTCTTCAAGGATCCCTTCATGCATTATGTTCGATATCAAGGAAAAGCAATTCT

GGTTTCAAAGGGGACTCATCTTCTGATGAAGAAATGGAAATGTCACCTTGTCAATTTCTGGCAATATTAT

TTTCACTTTTGGTCTCAACCGTACAGGATCCATATAAAGCAATTATCAAACTGTTCTTTCTATTTTTTAG

GTTATCTTTCAAGTGTACTAATAAATCCTTCGGCGGTAAGGAATCAAATGCTAGAGAATTCATTTCTAAT

AGATACTCTTATTAAAAAATTCGATACCAGAGTCCCAGTTATTCCTCTTATTGGATCATTGTCTAAAGCT

AAATTTTGTACCGTATCGGGGCATCCTATTAGTAAGCCGATCTGGACCGATTTATCAGATTGCGATATTA

TTGATCGATTTGGTCGGATATGTAGAAATCTTTCTCATTATCACAGTGGATCCTCAAAAAAACAGAGTTT

GTATCGAATAA

>Cryosophila_warscewiczii_JQ586727.1

CCTTCTTTACATTTATTGCGATTCTTTCTTCACGAATATCATAATTGGAATAGTCTTATTACTCCGAATA

ATTCTATTTTTCCTTTTTTCCTTTTTTCAAAAGAAAATAAAAGACTATTTCGGTTCCCATATAATTCTTA

TGTATCTGAATGCGAATTTTTATTAGTTTTTCTTCGTAAACAATCTTCTTATTTACGATTAACATCTTCT

GGAGCTTTTCTTGAGCGAACACATTTCTATGGAAAAATAGAACATCTTATAGTAGTGCGCCGTAATTATT

TTCAGAAGACCCTATGGTTCTTCAAGGATCCCTTCATGCATTATGTTCGATATCAAGGAAAAGCAATTCT

GGTTTCAAAGGGGACTCATCTTCTGATGAAGAAATGGAAATGTCACCTTGTCAATTTCTGGCAATATTAT

TTTCACTTTTGGTCTCAACCGTACAGGATCCATATAAAGCAATTATCAAACTGTTCTTTCTATTTTTTAG

GTTATCTTTCAAGTGTACTAATAAATCCTTCGGCGGTAAGGAATCAAATGCTAGAGAATTCATTTCTAAT

AGATACTCTTATTAAAAAATTCGATACCAGAGTCCCAGTTATTCCTCTTATTGGATCATTGTCTAAAGCT

AAATTTTGTACCGTATCGGGGCATCCTATTAGTAAGCCGATCTGGACCGATTTATCAGATTGCGATATTA

TTGATCGATTTGGTCGGATATGTAGAAATCTTTCTCATTATCACAGTGGATCCTCAAAAAAACAGAGTTT

GTATCGAATAA

>Cryosophila_stauracantha_HQ720272.1

TAGAAATGGAAATGGAAGAATTACAAGGATATTTAGAAAAAGATAGATCTCGGCAACAACACTTTCTATA

TCCGCTTCTCTTTAAGGAGTATATTTACACATTTGCTCATGATCGTGGTTTAAATGGTTCGATTTTTTAC

GAATCCACGGAAATTTTTGGTTATGACAATAAATCTAGTTCAGTACTTGTGAAACGTTCAATTATTCGAA

TGTATCAACAGAATTATTTGATTTATTCGGTTAATGATTCTAACCAAAATCGATTCGTTGGGCACAACAA

TTATTTTTATTTTCATTTTTATTCTCAGATGATATTGGAAGGTTTTGCAGTCATTGTGGAAATTCCATTC

TTGCTGCGATTAGTATCTTCCCTCGAAGAAAAAAAAATACCAAAATCTCAAAATTTGAATTTACGATCTA

TTCATTCAATATTTCCCTTTTTGGAGGACAAATTATCGCATTTAAATTATGTGTCAGATATACTAATACC

TTATCCCATACATCTGAAAATCTTGGTTCAAATCCTTCAATTCTGGATCCAAGATGTTCCTTCTTTCCAT

TTATTGCGATTCTTTCTTCACGAATATCATAATTGGAATAGTCTTATTACTCCGAATAATTCTATTTTTC

CTTTTTTCCTTTTTTCAAAAGAAAATAAAAGACTATTTCGGTTCCCATATAATTCTTATGTATCTGAATG

CGAATTTTTATTAGTTTTTCTTCGTAAACAATCTTCTTATTTACGATTAACATCTTCTGGAGCTTTTCTT

GAGCGAACACATTTCTATGGAAAAATAGAACATCTTATAGTAGTGCGCCGTAATTATTTTCAGAAGACCC

TATGGTTCTTCAAGGATCCCTTCATGCATTATGTTCGATATCAAGGAAAAGCAATTCTGGTTTCAAAGGG

GACTCATCTTCTGATGAAGAAATGGAAATGTCACCTTGTCAATTTCTGGCAATATTATTTTCACTTTTGG

TCTCAACCGTACAGGATCCATATAAAGCAATTATCAAACTGTTCTTTCTATTTTTTAGGTTATCTTTCAA

GTGTACTAATAAATCCTTCGGCGGTAAGGAATCAAATGCTAGAGAATTCATTTCTAATAGATACTCTTAT

TAAAAAATTCGATACCAGAGTCCCAGTTATTCCTCTTATTGGATCATTGTCTAAAGCTAAATTTTGTACC

GTATCGGGGCATCCTATTAGTAAGCCGATCTGGACCGATTTATCAGATTGCGATATTATTGATCGATTTG

GTCGGATATGTAGAAATCTTTCTCATTATCACAGTGGATCCTCAAAAAAACAGAGTTTGTATCGAATAAA

GTATATACTTCGATTTTCGTGTGCTAGAACTTTGGCTCGTAAACATAAAAGTACGGTACGCGCTTTTTTG

CAAAGATTAGGTTCAGGATTATTAGAAGAATTTTTTATGGAAGAAGAAGAAGTTATTTCTTTGATCTTCC

CAAAAACAACTTCTTTTTCTTTACATGAATCACATATAGAACGTATTTGGTATTTGGATATTATCCGTAT

CAATGATCTGGTGAATTATTCATAATTGGTTTGGTGACGTGATGAGACTTATGAATAGAAAATAGAATAG

AAATGATCTATAAATGATCAAGAGAGAAAAAAATTCATGAATTTTCATTCTGAAATGCTCATTGCAGTGG

TGTAG

>Cryosophila_warscewiczii_AM114563.1

GACCATATTGCACTATGTATCATTTGATAACCCCAAAAATGAAATAGGTCCCGTCTCTGGTTCAAGTAGA

AATGGAAATGGAAGAATTACAAGGATATTTAGAAAAAGATAGATCTCGGCAACAACACTTTCTATATCCG

CTTCTCTTTAAGGAGTATATTTACACATTTGCTCATGATCGTGGTTTAAATGGTTCGATTTTTTACGAAT

CCACGGAAATTTTTGGTTATGACAATAAATCTAGTTCAGTACTTGTGAAACGTTCAATTATTCGAATGTA

TCAACAGAATTATTTGATTTATTCGGTTAATGATTCTAACCAAAATCGATTCGTTGGGCACAACAATTAT

TTTTATTTTCATTTTTATTCTCAGATGATATTGGAAGGTTTTGCAGTCATTGTGGAAATTCCATTCTTGC

TGCGATTAGTATCTTCCCTCGAAGAAAAAAAAATACCAAAATCTCAAAATTTGAATTTACGATCTATTCA

TTCAATATTTCCCTTTTTGGAGGACAAATTATCGCATTTAAATTATGTGTCAGATATACTAATACCTTAT

CCCATACATCTGAAAATCTTGGTTCAAATCCTTCAATTCTGGATCCAAGATGTTCCTTCTTTACATTTAT

TGCGATTCTTTCTTCACGAATATCATAATTGGAATAGTCTTATTACTCCGAATAATTCTATTTTTCCTTT

TTTCCTTTTTTCAAAAGAAAATAAAAGACTATTTCGGTTCCCATATAATTCTTATGTATCTGAATGCGAA

TTTTTATTAGTTTTTCTTCGTAAACAATCTTCTTATTTACGATTAACATCTTCTGGAGCTTTTCTTGAGC

GAACACATTTCTATGGAAAAATAGAACATCTTATAGTAGTGCGCCGTAATTATTTTCAGAAGACCCTATG

GTTCTTCAAGGATCCCTTCATGCATTATGTTCGATATCAAGGAAAAGCAATTCTGGTTTCAAAGGGGACT

CATCTTCTGATGAAGAAATGGAAATGTCACCTTGTCAATTTCTGGCAATATTATTTTCACTTTTGGTCTC

AACCGTACAGGATCCATATAAAGCAATTATCAAACTGTTCTTTCTATTTTTTAGGTTATCTTTCAAGTGT

ACTAATAAATCCTTCGGCGGTAAGGAATCAAATGCTAGAGAATTCATTTCTAATAGATACTCTTATTAAA

AAATTCGATACCAGAGTCCCAGTTATTCCTCTTATTGGATCATTGTCTAAAGCTAAATTTTGTACCGTAT

CGGGGCATCCTATTAGTAAGCCGATCTGGACCGATTTATCAGATTGCGATATTATTGATCGATTTGGTCG

GATATGTAGAAATCTTTCTCATTATCACAGTGGATCCTCAAAAAAACAGAGTTTGTATCGAATAAAGTAT

ATACTTCGATTTTCGTGTGCTAGAACTTTGGCTCGTAAACATAAAAGTACGGTACGCGCTTTTTTGCAAA

GATTAGGTTCAGGATTATTAGAAGAATTTTTTATGGAAGAAGAAGAAGTTATTTCTTTGATCTTCCCAAA

AACAACTTCTTTTTCTTTACATGAATCACATATAGAACGTATTTGGTATTTGGATATTATCCGTATCAAT

GATCTGGTGAATTATTCATAATTGGTTTGGTGACGTGATGAGACTTATGAATAGAAAATAGAATAGAAAT

GATCTATAAATGATCAAGAGAGAAAAAAATTCATGAATTTTCATTCTGAAATGCTCATTGCAGTAGTGTA

GTGGTTGAATCAACTGAGTAGTCAAAATTATTATACTTTCTTCTCGGGACCCAAGTTTTATATTATACAT

AGGTAAAGTC

>Desmoncus_polyacanthos_HQ265567.1

TGNATGGGTCCTGCCTCTGGTTCAAGTAGAAATGGAAATGGAAGAATTACAAGGATATTTAGAAAAAGAT

AGATCTCGGCAACAACACTTTCTATATCCGCTTCTCTTTAAGGAGTATATTTACACATTTGCTCATGATC

GTGGTTTAAATGGNTCGATTTTTTACGAATCCACGGANATTTTTGGTTATGACAATAAATCTAGTTCAGT

ACTTGTGAAACGNTCAATTATTCGAATGTATCAACAGAATTATTTGATTTATTCGGGTAATGATTCTAAC

CANAATCGANTCGNTGGGCACAACNANTATTTTTATTTTCATTTTTATTCTCAGATGATATTGGAAGGTT

TTGCNGCCATTGNGGAAATTCCCNTCTNGGGGCGANTAGNATCTTCCCTCGAAGAAAAAAAAANACCCAA

ATCTCCGAATTTGAATTTACGAGCTATTCNTNCNAAATTTCCCTTTTTGGNGGNCAAATTATCGCATTTN

NATTATGNGNCNGAANGACNAATACCTTATCCCNTCCCCCNGANAANCTTGGGTCNAAACCNTCAATGCN

GGANCCCAGAAGGTCCNTCTTTACATTTATTGCGATTCTTTCTTCACGAATATCACAATGGGAATAGTCT

TATTACTCCGAATAATTCTATTTTTTTTTCAAAAGAAAATAAAAGACTATTTCGGGGCCCATATAATTCT

TATGTATCTGAATGCGAATTTGTATTAGTTTTTCTTCGTAAACAATCTTCTTATTTACGATTAACATCTT

CTGGAGCTTTTCTTGAGCGAACACATTTCTATGGAAAAATAGAACATCTTATAGTAGTGCGCCGTAATTA

TTTTCAGAAGACCCTATGGTTCTTCAAGGATCCCTTCATGCATTATGTTCGATATCAAGGAAAAGCAATT

CTGGTTTCAAAGGGGACTCATCTTCTGATGAAGAAATGGAAATGTCATCTTGTCAATTTCTGGCAATATT

ATTTTCACTTTTGGTCTCAACCGNACAGGATCCATATAAACCAATTATCAAGCTGTTCTTTCTATTTTCT

AGGTTATCTTTCAAGTGTACTAATAAATCCTTCGGCGGTAAGGAATCAAATGCTAGAGAATTCATTTCTA

ATAGATACTGTTATTAAAAAATTCGATACCAGAGTCCCTGTTATTCCTCTTATTGGATCATTGTCTAAAG

CTAAATTTTGTACCGTATCGGGGCATCCTATTAGTAAGCCGATCTGGACCAATTTATCAGATTGCGATAT

TATTGATCGATTTGGTCGGATATGTAGAAATCTTTCTCATTATCACAGTGGATCCTCAAAAAAACAGAGT

TTGTANCGAATAAAGTATATACTTCGATTTTCGTGTGCTAGAACTTTGGCTCGTAAACATAAAAGTATGG

TNCGCGCTTTTTTGCAAAGATTAGGTTCGGGATTATTAGAAGAATTCTTTACGGAAGAAGAACAAGTTGT

TTCTTTGATCTTCCCAAAAACAACTTCTTTTTCTTTACATGAATCACATATAGAACGTATTTGGTATTTG

GATATTATCCGTATCAATGACCTGGTGAATTATTCATAATGGGTTTGGTGACGTGATGAGACTTATGAAT

AGAATAGAAATGATCTATAAATGATCAAGAGAGAAAAAAATTCATGAATTTTCATTCTGAAATGCTCATT

GCAGTAGTGTAGTGGTTGAATCAACTGAGTAGTCAAAATTATTATACTTTCTTCTCGGGACCCAAGTTTT

ATATTATACATAGGTAAAGTCGTGTGCAATGAAAAATGCAAGCACGGTTTGGGGAGGGATCTTTTTCCTC

TATTCCAACAAAGAAAAGTTATCTACTCCACCGGAACTAGTTAA

>Desmoncus_mitis_HQ265565.1

AATGAATGGGTCCTGCCTCTGGTTCAAGTAGAAATGGAAATGGAAGAATTACAAGGATATTTAGAAAAAG

ATAGATCTCGGCAACAACACTTTCTATATCCGCTTCTCNTTAAGGAGTATATTTACACATTTGCTCATGA

TCGTGGTTTAAATGGNTCGATTTTTTACGAATCCACGGNNATTTTTGGTTATGACAATAAATCTAGTTCA

GTACTTGTGAAACGNTCAATTATTCGNATGTATCAACAGAATTATTTGATTTATTCGGGTAATGATTCTA

ACCNAAATCGNTTCGNTGGGCACAACAANTATTTTTATTTTCATTTTTATTCTCNGATGATATTGGAAGG

NTTTGCAGCCATTGGGGAAATTCCCNTCTTGGGGCGATTAGTATCTTCCCTCGAAGAAAAAAAAATACCC

AAATCTCNGAATTTGAATTTACNANCNATTCATTCAATATTTCCCTTTTTGGNGGACAAATTATCGCATT

TNNATTATGGGNCAGANNNACTAANACCTTATCCCATCCNCCTGAAAANCNTGGGTCNAATCCTTCNATG

CNGGATCCCAGANGGTCCNTCNTTACNNTTNTTGCGATTCTTTCTTCCCGAANATCNTAANTGGGATAGT

CTTATTACTCCGAAAAATTCTATTTTTTTTTCAAAAGAAAATAAAAGACTATTTCGGTTCCCATATAATT

CTTATGTATCNGAATGCGAATTTGTATTAGTTTTTCTTCGTAAACAATCTTCTTATTTACGATTAACATC

TTCNGGAGCTTTTCTTGAGCGAACACATTTCTATGGAAAAATAGAACATCTTATAGTAGTGCGCCGTAAT

TATTTTCAGAAGACCCTATGGTTCTTCAAGGATCCCTTCATGCATTATGTTCGATATCAAGGAAAAGCAA

TTCTGGTTTCAAAGGGGACTCATCTTCTGATGAAGAAATGGAAATGTCATCTTGTCAATTTCTGGCAATA

TTATTTTCACTTTTGGTCTCAACCGTACAGGATCCATATAAACCAATTATCAAGCTGTTCTTTCTATTTT

CTAGGTTATCTTTCAAGTGTACTAATAAATCCTTCGGCGGTAAGGAATCAAATGCTAGAGAATTCATTTC

TAATAGATACTGTTATTAAAAAATTCGATACCAGAGTCCCTGTTATTCCTCTTATTGGATCATTGTCTAA

AGCTAAATTTTGTACCGTATCGGGGCATCCTATTAGTAAGCCGATCTGGACCAATTTATCAGATTGCGAT

ATTATTGATCGATTTGGTCGGATATGTAGAAATCTTTCTCATTATCACAGTGGATCCTCAAAAAAACAGA

GTTTGTATCGAATAAAGTATATACTTCGATTTTCGTGTGCTAGAACTTTGGCTCGTAAACATAAAAGTAT

GGTACGCGCTTTTTTGCAAAGATTAGGTTCGGGATTATTAGAAGAATTCTTTACGGAAGAAGAACAAGTT

GTTTCTTTGATCTTCCCAAAAACAACTTCTTTTTCTTTACATGAATCACATATAGAACGTATTTGGTATT

TGGATATTATCCGTATCAATGACCTGGTGAATTATTCATAATGGGTTTGGTGACGTGATGAGACTTATGA

ATAGAATAGAAATGATCTATAAATGATCAAGAGAGAAAAAAATTCATGAATTTTCATTCTGAAATGCTCA

TTGCAGTAGTGTAGTGGTTGAATCAACTGAGTAGTCAAAATTATTATACTTTCTTCTCGGGACCCAAGTT

TTATATTATACATAGGTAAAGTCGTGTGCAATGAAAAATGCAAGCACGGTTTGGGGAGGGATCTTTTTCC

TCTATTCCAACAAAGAAAAGTTATCTACTCCATCCGGACTAGTTAAA

>Desmoncus_orthacanthos_HQ265566.1

ATGGGTCCTGCCTCTGGTTCAGTAGAAATGGAAATGGAAGAATTACAAGGATATTTAGAAAAAGATAGAT

CTCGGCAACAACACTTTCTATATCCGCTTCTCTTTAAGGAGTATATTTACACATTTGCTCATGATCGTGG

TTTAAATGGTTCGATTTTTTACGAATCCACGGAAATTTTTGGTTATGACAATAAATCTAGTTCAGTACTT

GTGAAACGTTCAATTATTCGAATGTATCAACAGAATTATTTGATTTATTCGGTTAATGATTCTAACCAAA

ATCGATTCGTTGGGCACAACAATTATTTTTATTTTCATTTTTATTCTCAGATGATATTGGAAGGTTTTGC

AGTCATTGTGGAAATTCCATTCTTGCTGCGATTAGTATCTTCCCTCGAAGAAAAAAAAATACCAAAATCT

CAGAATTTGAATTTACGATCTATTCATTCAATATTTCCCTTTTTGGAGGACAAATTATCGCATTTAAATT

ATGTGTCAGATATACTAATACCTTATCCCATCCACCTGAAAATCTTGGTTCAAATCCTTCAATGCTGGAT

CCAAGATGTTCCTTCTTTACATTTATTGCGATTCTTTCTTCACGAATATCATAATTGGAATAGTCTTATT

ACTCCGAATAATTCTATTTTTTTTNCAAAAGAAAATAAAAGACTATTTCGGTTCCCATATAATTCTTATG

TATCTGAATGCGAATTTGTATTAGTTTTTCTTCGTAAACAATCTTCTTATTTACGATTAACATCTTCTGG

AGCTTTTCTTGAGCGAACACATTTCTATGGAAAAATAGAACATCTTATAGTAGTGCGCCGTAATTATTTT

CAGAAGACCCTATGGTTCTTCAAGGATCCCTTCATGCATTATGTTCGATATCAAGGAAAAGCAATTCTGG

TTTCAAAGGGGACTCATCTTCTGATGAAGAAATGGAAATGTCATCTTGTCAATTTCTGGCAATATTATTT

TCACTTTTGGTCTCAACCGTACAGGATCCATATAAACCAATTATCAAGCTGTTCTTTCTATTTTCTAGGT

TATCTTTCAAGTGTACTAATAAATCCTTCGGCGGTAAGGAATCAAATGCTAGAGAATTCATTTCTAATAG

ATACTGTTATTAAAAAATTCGATACCAGAGTCCCTGTTATTCCTCTTATTGGATCATTGTCTAAAGCTAA

ATTTTGTACCGTATCGGGGCATCCTATTAGTAAGCCGATCTGGACCAATTTATCAGATTGCGATATTATT

GATCGATTTGGTCGGATATGTAGAAATCTTTCTCATTATCACAGTGGATCCTCAAAAAAACAGAGTTTGT

ATCGAATAAAGTATATACTTCGATTTTCGTGTGCTAGAACTTTGGCTCGTAAACATAAAAGTATGGTACG

CGCTTTTTTGCAAAGATTAGGTTCGGGATTATTAGAAGAATTCTTTACGGAAGAAGAACAAGTTGTTTCT

TTGATCTTCCCAAAAACAACTTCTTTTTCTTTACATGAATCACATATAGAACGTATTTGGTATTTGGATA

TTATCCGTATCAATGACCTGGTGAATTATTCATAATGGGTTTGGTGACGTGATGAGACTTATGAATAGAA

TAGAAATGATCTATAAATGATCAAGAGAGAAAAAAATTCATGAATTTTCATTCTGAAATGCTCATTGCAG

TAGTGTAGTGGTTGAATCAACTGAGTAGTCAAAATTATTATACTTTCTTCTCGGGACCCAAGTTTTATAT

TATACATAGGTAAAGTCGTGTGCAATGAAAAATGCAAGCACGGTTTGGGGAGGGATCTTTTTCCTCTATT

CCAACAAAGAAAAGTTATCTACTCCATCCGNACTAGTTAA

>Desmoncus_orthacanthos_AM114643.1

GACCATATTGCACTATGTATCATTTGATAACCCCAAAAATGAAATGGGTCCTGCCTCTGGTTCAAGTAGA

AATGGAAATGGAAGAATTACAAGGATATTTAGAAAAAGATAGATCTCGGCAACAACACTTTCTATATCCG

CTTCTCTTTAAGGAGTATATTTACACATTTGCTCATGATCGTGGTTTAAATGGTTCGATTTTTTACGAAT

CCACGGAAATTTTTGGTTATGACAATAAATCTAGTTCAGTACTTGTGAAACGTTCAATTATTCGAATGTA

TCAACAGAATTATTTGATTTATTCGGTTAATGATTCTAACCAAAATCGATTCGTTGGGCACAACAATTAT

TTTTATTTTCATTTTTATTCTCAGATGATATTGGAAGGTTTTGCAGTCATTGTGGAAATTCCATTCTTGC

TGCGATTAGTATCTTCCCTCGAAGAAAAAAAAATACCAAAATCTCAGAATTTTAATTTACGATCTATTCA

TTCAATATTTCCCTTTTTGGAGGACAAATTATCGCATTTAAATTATGTGTCAGATATACTAATACCTTAT

CCCATCCATCTGAAAATCTTGGTTCAAATCCTTCAATGCTGGATCCAAGATGTTCCTTCTTTACATTTAT

TGCGATTCTTTCTTCACGAATATCATAATTGGAATAGTCTTATTACTCCGAATAATTCTATTTTTTTTTC

AAAAGAAAATAAAAGACTATTTCGGTTCCCATATAATTCTTATGTATCTGAATGCGAATTTGTATTAGTT

TTTCTTCGTAAACAATCTTCTTATTTACGATTAACATCTTCTGGAGCTTTTCTTGAGCGAACACATTTCT

ATGGAAAAATAGAACATCTTATAGTAGTGCGCCGTAATTATTTTCAGAAGACCCTATGGTTCTTCAAGGA

TCCCTTCATGCATTATGTTCGATATCAAGGAAAAGCAATTCTGGTTTCAAAGGGGACTCATCTTCTGATG

AAGAAATGGAAATGTCATCTTGTCAATTTCTGGCAATATTATTTTCACTTTTGGTCTCAACCGTACAGGA

TCCATATAAACCAATTATCAAGCTGTTCTTTCTATTTTCTAGGTTATCTTTCAAGTGTACTAATAAATCC

TTCGGCGGTAAGGAATCAAATGCTAGAGAATTCATTTCTAATAGATACTGTTATTAAAAAATTCGATACC

AGAGTCCCTGTTATTCCTCTTATTGGATCATTGTCTAAAGCTAAATTTTGTACCGTATCGGGGCATCCTA

TTAGTAAGCCGATCTGGACCAATTTATCAGATTGCGATATTATTGATCGATTTGGTCGGATATGTAGAAA

TCTTTCTCATTATCACAGTGGATCCTCAAAAAAACAGAGTTTGTATCGAATAAAGTATATACTTCGATTT

TCGTGTGCTAGAACTTTGGCTCGTAAACATAAAAGTATGGTACGCGCTTTTTTGCAAAGATTAGGTTCGG

GATTATTAGAAGAATTCTTTACGGAAGAAGAACAAGTTGTTTCTTTGATCTTCCCAAAAACAACTTCTTT

TTCTTTACATGAATCACATATAGAACGTATTTGGTATTTGGATATTATCCGTATCAATGACCTGGTGAAT

TATTCATAATGGGTTTGGTGACGTGATGAGACTTATGAATAGAATAGAAATGATCTATAAATGATCAAGA

GAGAAAAAAATTCATGAATTTTCATTCTGAAATGCTCATTGCAGTAGTGTAGTGGTTGAATCAACTGAGT

AGTCAAAATTATTATACTTTCTTCTCGGGACCAAAGTTTTATATTATACATAGGTAAAGTC

>Dictyocaryum_lamarckianumn_AM114616.1

GACCATATTGCACTATGTATCATTTGATAACCCCAAAAATGAAATGGGTCCTGCCTCTGGTTCAAGTAGA

AATGTAAATGGAAGAATTACAAGGATATTTAGAAAAAGATAGATCTCGGCAACAACACTTTCTATATCCG

CTTCTCTTTAAGGAGTATATTTACACATTTGCTCATGATCGTGGTTTAAATGGTTCGATTTTTTACGAAT

CCACGGAAATTTTTGGTTATGACAATAAATCTAGTTCAGTACTTGTGAAACGTTCAATTATTCGAATGTA

TCAACAGAATTTTTTGATTTATTCGGTTAATGATTCTAACCAAAATCGATTCGTTGGGCACAACAATTAT

TTTTATTTTCATTTTTATTCTCAGATGATATTGGAAGGTTTTGCAGTCATTGTGGAAATTCCATTCTTGC

TGCGATTAGTATCTTCCCTCGAAGAAAAAAAAATACCAAAATCTCAGAATTTGAATTTACGATCTATTCA

TTCAATATTTCCCTTTTTGGAGGACAAATTATCGCATTTAAATTATGTGTCAGATATACTAATACCTTAT

CCCATCCATCTGAAAATCTTGGTTCAAATCCTTCAATGCTGGATCCAAGATGTTCCTTCTTTACATTTAT

TGCGATTCTTTCTTCACGAATATCATAATTGGAATAGTCTTATTACTCCGAATAATTCTATTTTTTCAAA

AGAAAATAAAAGACTATTTCGGTTCCCATATAATTCTTATATATCTGAATGCGAATTTGTATTAGTTTTT

CTTCGTAAACAATCTTCTTATTTACGATTAACATCTTCTGGAGCTTTTCTTGAGCGAAAACATTTCTATG

GAAAAATAGAACATCTTATAGTAGTGCGCCGTAATTATTTTCAGAAGACCCTATGGTTCTTCAAGGATCC

CTTCATGCATTATGTTCGATATCAAGGAAAAGCAATTCTGGTTTCAAAGGGGACTCATCTTCTGATGAAG

AAATGGAAATGTCACCTTGTCAATTTCTGGCAATATTATTTTCACTTTTGGTCTCAACCGTACAGGATCC

ATATAAACCAATTATCAAACTGTTCTTTCTATTTTCTAGGTTATCTTTCAAGTGTACTAATAAATCCTTC

GGCGGTAAGGAATCAAATGCTAGAGAATTCATTTCTAATAGATACTGTTATTAAAAAATTCGATACCAGA

GTCCCAGTTATTCCTCTTATTGGATCATTGTCTAAAGCTAAATTTTGTACCGTATCGGGGCATCCTATTA

GTAAGCCGATCTGGACCAATTTATCAGATTGCGATATTATTGATCGATTTGGTCGGATATGTAGAAATCT

TTCTCATTATCACAGTGGATCCTCAAAAAAACAGAGTTTGTATCGAATAAAGTATATACTTCGATTTTCG

TGTGCTAGAACTTTGGCTCGTAAACATAAAAGTATGGTACGCGCTTTTTTGCAAAGATTAGGTTCGGGAT

TATTAGAAGAATTCTTTATGGAAGAAGAACAAGTTGTTTCTTTGATCTTCCCAAAAACAACTTCTTTTTC

TTTACATGAATCACATATAGAACGTATTTGGTATTTGGATATTATCCGTATCAATGACCTGGTGAATTAT

TCATAATGGGTTTGGTGACGTGATGAGACTTATGAATAGAATAGAAATGATCTATAAATGATCAAGAGAG

AAAAAAATTCATGAATTTTCATTCTGAAATGCTCATTGCAGTAGTGTAGTGGTTGAATCAACTGAGTAGT

CAAAATTATTATACTTTCTTCTCGGGACCCAAGTTTTATATTATACATAGGTAAAGTC

>Elaeis_oleifera_HQ265568.1

AAGTAGAAATGGAAATGGAAGAATTACAAGGATATTTAGAAAAAGATAGATCTCGGCAACAACACTTTCT

ATATCCGCTTCTCTTTAAGGAGTATATTTACGCATTTGCTCATGATCGTGGTTTAAATGGTTCGATTTTT

TACGAATCCACGGAAATTTTTGGTTATGACAATAAATCTAGTTCAGTACTTGTGAAACGTTCAATTATTC

GAATGTATCAACAGAATTATTTGATTTATTCGGTTAATGATTCTAACCAAAATCGATTCGTTGGGCACAA

CAATTATTTTGATTTTCATTTTNATTCTCAGATGATATTGGAAGGTTTTGCAGTCATTGTGGAAATTCCA

ATCTTGCTGCGATTAGTATCTTCCCTCGAAGAAAAAAAAATACCAAAATCTCAGAATTTGAATTTACGAT

CTATTCATTCAATATTTCCCTTTTTGGAGGACAANTTGTCGCATTTAAATTANGNGTCANATATACTAAT

ACCTTATCCCANCCATCTGAAAATCTNGGTTCAAATCCTNCAANGCGGGATCCAAGANGTCCCTTCNTTA

CATTTATGGGGATTCTTTCTCCANGAATNTCATAATGGGAATAGTCTTATTACTCNGANNAATTCTATTT

TTTTTTCAAAAGAAAATAAAAGACTATTTCGGTTCCCATATAATTCTTATGTATCTGAATGCGAATTTGT

ATTAGTTTTTCTTCGTAAACAATCTTCTTATTTACGATTAACATCTTCTGGAGCCTTTCTTGAGCGAACA

CATTTCTATGGAAAAATAGAACATCTTATAGTAGTGCGCCGTAATTATTTTCAGAAGACCCTATGGTTCT

TCAAGGATCCCTTCATGCATTATGTTCGATATCAAGGAAAAGCAATTCTGGTTTCAAAGGGGACTCATCT

TCTGATGAAGAAATGGAAATGTCATCTTGTCAATTTCTGGCAATATTATTTTCACTTTTGGTCTCAACCG

TACAGGATCCATATAAACCAATTATCAAGCTGTTCTTTCTATTTTCTAGGTTATCTTTCAAGTGTACTAA

TAAATCCTTCGGCGGTAAGGAATCAAATGCTAGAGAATNCATTTCTAATAGATACTGTTATTAAAAAATT

CGATACCAGAGTCCCTGTTATTCCTCTTATTGGATCATTGTCTAAAGCTAAATTTTGTACCGTATCGGGG

CATCCTATTAGTAAGCCGATCTGGACCAATTTATCAGATTGCGATATTATTGATCGATTTGGTCGGATAT

GTAGAAATCTTTCTCATTATCACAGTGGATCCTCAAAAAAACAGAGTTTGNGTCGNNTNAGGNANATGCT

TCGANTTNNGTGTGCGAGANCTNGGGCTCGNNNNCNTAAACGTANGGGTCGCGCTTTTTTGCAAAGATTA

GGTTCGGGATTATTAGAAGAATTCTTTACGGAAGAAGAACAAGTTGTTTCTTTGATCTTCCCAAAAACAA

CTTCTTTTTCTTTACATGAATCACATATAGAACGTATTTGGTATTTGGATATTATCCGTATCAATGACCT

GGTGAATTATTCATAATGGGTTTGGTGACGTGATGAGACTTATGAATAGAATAGAAATGATCTATAAATG

ATCAAGAGAGAAAAAAATTCATGAATTTTCATTCTGAAATGCTCATTGCAGTAACAGTAATGTAGTGGTT

GAATCAACTGAGTAGTCAAAATTATTATCTTCTCGGGACCCAAGTTTNATATTATACATAGGTAAAGTCG

TGTGCAATGAAAAATGCAAGCACGGTTTGGGGAGGGATC

>Elaeis_guineensis_AM114644.1

GACCATATTGCACTATGTATCATTTGATAACCCAAAAAATGAAATGGGTCCTGCCTCTGGTTCAAGTAGA

AATGGAAATGGAAGAATTACAAGGATATTTAGAAAAAGATAGATCTCGGCAACAACACTTTCTATATCCG

CTTCTCTTTAAGGAGTATATTTACACATTTGCTCATGATCGTGGTTTAAATGGTTCGATTTTTTACGAAT

CCACGGAAATTTTTGGTTATGACAATAAATCTAGTTCAGTACTTGTGAAACGTTCAATTATTCGAATGTA

TCAACAGAATTATTTGATTTATTCGGTTAATGATTCTAACCAAAATCGATTCGTTGGGCACAACAATTAT

TTTGATTTTCATTTTTATTCTCAGATGATATTGGAAGGTTTTGCAGTCATTGTGGAAATTCCAATCTTGC

TGCGATTAGTATCTTCCCTCGAAGAAAAAAAAATACCAAAATCTCAGAATTTGAATTTACGATCTATTCA

TTCAATATTTCCCTTTTTGGAGGACAAATTGTCGCATTTAAATTATGTGTCAGATATACTAATACCTTAT

CCCATCCATCTGAAAATCTTGGTTCAAATCCTTCAATGCTGGATCCAAGATGTTCCTTCTTTACATTTAT

TGCGATTCTTTCTTCACGAATATCATAATTGGAATAGTCTTATTACTCCGAATAATTCTATTTTTTTTTC

AAAAGAAAATAAAAGACTATTTCGGTTCCCATATAATTCTTATGTATCTGAATGCGAATTTGTATTAGTT

TTTCTTCGTAAACAATCTTCTTATTTACGATTAACATCTTCTGGAGCCTTTCTTGAGCGAACACATTTCT

ATGGAAAAATCGAACATCTTATAGTAGTGCGCCGTAATTATTTTCAGAAGACCCTATGGTTCTTCAAGGA

TCCCTTCATGCATTATGTTCGATATCAAGGAAAAGCAATTCTGGTTTCAAAGGGGACTCATCTTCTGATG

AAGAAATGGAAATGTCATCTTGTCAATTTCTGGCAATATTATTTTCACTTTTGGTCTCAACCGTACAGGA

TCCATATAAACCAATTATCAAGCTGTTCTTTCTATTTTCTAGGTTATCTTTCAAGTGTACTAATAAATCC

TTCGGCGGTAAGGAATCAAATGCTAGAGAATTCATTTCTAATAGATACTGTTATTAAAAAATTCGATACC

AGAGTCCCTGTTATTCCTCTTATTGGATCATTGTCTAAAGCTAAATTTTGTACCGTATCGGGGCATCCTA

TTAGTAAGCCGATCTGGACCAATTTATCAGATTGCGATATTATTGATCGATTTGGTCGGATATGTAGAAA

TCTTTCTCATTATCACAGTGGATCCTCAAAAAAACAGAGTTTGTATCGAATAAAGTATATACTTCGATTT

TCGTGTGCTAGAACTTTGGCTCGTAAACATAAAAGTATGGTACGCGCTTTTTTGCAAAGATTAGGTTCGG

GATTATTAGAAGAATTCTTTACGGAAGAAGAACAAGTTGTTTCTTTGATCTTCCCAAAAACAACTTCTTT

TTCTTTACATGAATCACATATAGAACGTATTTGGTATTTGGATATTATCCGTATCAATGACCTGGTGAAT

TATTCATAATGGGTTTGGTGACGTGATGAGACTTATGAATAGAATAGAAATGATCTATAAATGATCAAGA

GAGAAAAAAATTCATGAATTTTCATTCTGAAATGCTCATTGCAGTAATAGTAATGTAGTGGTTGAATCAA

CTGAGTAGTCAAAATTATTATCTTCTCGGGACCCAAGTTTTATATTATACATAGGTAAAGTC

>Euterpe_oleracea_JQ626542.1

TACCTTATCCCATCCATCTGAAAATCTTGGTTCAAATCCTTCAATGCTGGATCCAAGATGTTCCTTCTTT

ACATTTATTGCGATTCTTTCTTCACGAATATCATAATTGGAATAGTCTTATTACTCCGAATAATTCTATT

TTTTTTTTTTCAAAAGAAAATAAAAGACTATTTCGGTTCCTATATAATTCTTATGTATCTGAATGCGAAT

TTGTATTAGTTTTTCTTCGTAAACAATCTTCTTATTTACGATTAACATCTTCTGGAGCTTTTCTTGAGCG

AACACATTTCTATGGAAAAATAGAACATCTTATAGTAGTGCGCCGTAATTATTTTCAGAAGACCCTATGG

TTCTTCAAGGATCCCTTCATGCATTATGTTCGATATCAAGGAAAAGCAATTCTGGTTTCAAAGGGGACTC

ATCTTCTGATGAAGAAATGGAAATGTCACCTTGTCAATTTCTGGCAATATTATTTTCACTTTTGGTCTCA

ACCGTACAGGATCCATATAAACCAATTATCAAGCTGTTCTTTCTATTTTCTAGGTTATCTTTCAAGTGTA

CTAATAAATCCTTCGGCGGTAAGGAATCAAATGCTAGAGAATTCATTTCTAATAGATACTGTTATTAAAA

AATTCGATACCAGAGTCCCAGTTATTCCTCTTATTGGATCATTGTCTAAAGCTAAATTTTGTACCGTATC

GGGGCATCCTATTAGTAAGCCGATCTGGACCAATTTATCAGATTGCGATATTATTGATCGATTTGGTCGG

ATATGTAGAAATCTTTCTCATTATC

>Euterpe_oleracea_AM114647.1

GACCATATTGCACTATGTATCATTTGATAACCCCAAAAATGAAATGGGTCCAGCCTCTGGTTCAAGTAGA

AATGTAAATGGAAGAATTACAAGGATATTTAGAAAAAGATAGATCTCGGCAACAACACTTTCTATATCCG

CTTCTCTTTAAGGAGTATATTTACACATTTGCTCATGATCGTGGTTTAAATGGTTCGATTTTTTACGAAT

CCACGGAAATTTTTGGTTATGACAATAAATCTAGTTCAGTACTTGTGAAACGTTCAATTATTCGAATGTA

TCAACAGAATTATTTGATTTATTCGGTTAACGATTCTAACCAAAATCGATTCGTTGGGCACAACAATTAT

TTTTATTTTCATTTTTATTCTCAGATGATATTGGAAGGTTTTGCAGTCATTGTGGAAATTCCATTCTTGC

TGCGATTAGTATCTTCCCTCGAAGAAAAAAAAATACCAAAATCTCAGAATTTGAATTTACGATCTATTCA

TTCAATATTTCCCTTTTTGGAGGACAAATTATCGCATTTAAATTATGTGTCAGATATACTAATACCTTAT

CCCATCCATCTGAAAATCTTGGTTCAAATCCTTCAATGCTGGATCCAAGATGTTCCTTCTTTACATTTAT

TGCGATTCTTTCTTCACGAATATCATAATTGGAATAGTCTTATTACTCCGAATAATTCTATTTTTTTTTT

TTCAAAAGAAAATAAAAGACTATTTCGGTTCCTATATAATTCTTATGTATCTGAATGCGAATTTGTATTA

GTTTTTCTTCGTAAACAATCTTCTTATTTACGATTAACATCTTCTGGAGCTTTTCTTGAGCGAACACATT

TCTATGGAAAAATAGAACATCTTATAGTAGTGCGCCGTAATTATTTTCAGAAGACCCTATGGTTCTTCAA

GGATCCCTTCATGCATTATGTTCGATATCAAGGAAAAGCAATTCTGGTTTCAAAGGGGACTCATCTTCTG

ATGAAGAAATGGAAATGTCACCTTGTCAATTTCTGGCAATATTATTTTCACTTTTGGTCTCAACCGTACA

GGATCCATATAAACCAATTATCAAGCTGTTCTTTCTATTTTCTAGGTTATCTTTCAAGTGTACTAATAAA

TCCTTCGGCGGTAAGGAATCAAATGCTAGAGAATTCATTTCTAATAGATACTGTTATTAAAAAATTCGAT

ACCAGAGTCCCAGTTATTCCTCTTATTGGATCATTGTCTAAAGCTAAATTTTGTACCGTATCGGGGCATC

CTATTAGTAAGCCGATCTGGACCAATTTATCAGATTGCGATATTATTGATCGATTTGGTCGGATATGTAG

AAATCTTTCTCATTATCACAGTGGATCCTCAAAAAAACAGAGTTTGTATCGAATAAAGTATATACTTCGA

TTTTCGTGTGCTAGAACTTTGGCTCGTAAACATAAAAGTATGGTACGCGCTTTTTTGCAAAGATTAGGTT

CGGGATTATTAGAAGAATTCTTTACGGAAGAAGAACAAGTTGTTTCTTTGATCTTCCCAAAAACAACTTC

TTTTTCTTTACATGAATTACATATAGAACGTATTTGGTATTTGGATATTATCCGTATCAATGACCTGGTG

AATTATTCATAATGGGTTTGGTGACGTGATGAGACTTATGAATAGAATAGAAATGATCTATAAATGATCA

AGAGAGAAAAAAATTCATGAATTTTCATTCTGAAATGCTCATTGCAGTAGTGTAGTGGTTGAATCAACTG

AGTAGTCAAAATTATTATACTTTCTTCTCGGGACCCAAGTTTTATATTATACATAGGTAAAGTC

>Gaussia_spirituana_DQ178695.1

CGTTCTGACCATATTGCACTATGTATCATTTGATAACCAAAAAAATGAAATGGGTCCTGCCTCTGGTTCA

AGTAGAAATGTAAATGGAAGAATTACAAGGATATTTAGAAAAAGATAGATCTCGGCAACAACACTTTCTA

TATCCGCTTCTCTTTAAGGAGTATATTTACACATTTGCTCATGATCGTGGTTTAAATGGTTCGATTTTTT

ACGAATCCACGGAAATTTTTGGTTATGACAATAAATCTAGTTCAGTACTTGTGAAACGTTCAATTATTCG

AATGTATCAACAGAATTATTTGATTTATTCGGTTAATGATTCTAACCAAAATCGATTCGTTGGGCACAAC

AATTATTTTTATTTTCATTTTTATTCTCAGATGATATTGGAAGGTTTTGCAGTCATTGTGGAAATTCCAT

TCTTGCTGCGATTAGTATCTTCTCTCGAAGAAAAAAAAATACCAAAATCTCATAATTTTAATTTACGATC

TATTCATTCAATATTTCCCTTTTTGGAGGACAAATTATCGCATTTAAATTATGTGTCAGATATACTAATA

CCTTATCCCATCCATCTGAAAATCTTGGTTCAAATCCTTCAATGCTGGATCAAAGATGTTCCTTCTTTAC

ATTTATTGCGATTCTTTCTTCACGAATATCATAATTGGAATAGTCTTATTACTCCAAATAATTCTATTTT

TTTTTCAAAAGAAAATAAAAGACTATTTCGGTTCCCATATAATTCTTATGTATCTGAATGCGAATTTGTA

TTAGTTTTTCTTCGTAAACAATCCTCTTATTTACGATTAACATCTTCTGGAGCTTTTCTTGAGCGAATAC

ATTTCTATGGAAAAATAGAACATCTTATAGTAGTGCGCCGTAATTATTTTCAGAGGACCCTATGGTTCTT

CAAGGATCCCTTCATGCATTATGTTCGATATCAAGGAAAAGCAATTCTGGTTTCAAAGGGGACTCATCTT

CTGATGAAGAAATGGAAATGTCACCTTGTCAATTTCTGGCAATATTATTTTCACTTTTGGTCTCAACCGT

ACAGGATCCATATAAACCAATTATCAAGCTGTTCTTTCCATTTTCTAGGTTATCTTTCAAGTGTACTAAT

AAATCCTTCGGCGGTAAGGAATCAAATGCTAGAGAATTCATTTCTAATAGATACTGTTATTAAAAAATTC

GATACCAGAGTCCCAGTTATTCCTCTTATTGGATCATTGTCTAAAGCTAAATTTTGTACCGTATCGGGGC

ATCCTATTAGTAAGCCGATCTGGACCAATTTATCAGATTGTGATATTATTGATCGATTTGGTCGGATATG

TAGAAATCTTTCTCATTATCACAGTGGATCCTCAAAAAAACAGAGTTTGTATCGAATAAAGTATATACTT

CGATTTTCGTGTGCTAGAACTTTGGCTCGTAAACATAAAAGTACAGTACGCGCTTTTTTGCAAAGATTAG

GTTCGGGATTATTAGAAGAATTCTTTACGGAAGAAGAACAAGTTGTTTCTTTGATCTTCCCAAAAACAAC

TTCTTTTTCTTTACATGAATCACATATAGAACGTATTTGGTATTTGGATATTATCCGTATCAATGACCTA

GTGAATTATTCATAATGGGTTTGTTTGGTGACGTGATGAGACTTATGAATAGTCTGGAAATGATCTATAA

ATGATCAAGAGAGAAAAAAATTCATGAATTTTCATTCTGAAATGCTCATTGCAGTAGTGTAGTGGTTGAA

TCAACTGAGTAGTAAAAATTATTATACTTTCTTCTCGGGACCCAAGTTTTATATTATACATAGGTAAAGT

CGTGTGCAATG

>Gaussia_gomez_pompae_DQ178693.1

TTGCACTATGTATCATTTGATAACCCAAAAAATGAAATGGGTCCTGCCTCTGGTTCAAGTAGAAATGTAA

ATGGAAGAATTACAAGGATATTTAGAAAAAGATAGATCTCGGCAACAACACTTTCTATATCCGCTTCTCT

TTAAGGAGTATATTTACACATTTGCTCATGATCGTGGTTTAAATGGTTCGATTTTTTACGAATCCACGGA

AATTTTTGGTTATGACAATAAATCTAGTTCAGTACTTGTGAAACGTTCAATTATTCGAATGTATCAACAG

AATTATTTGATTTATTCGGTTAATGATTCTAACCAAAATCGATTCGTTGGGCACAACAATTATTTTTATT

TTCATTTTTATTCTCAGATGATATTGGAAGGTTTTGCAGTCATTGTGGAAATTCCATTCTTGCTGCGATT

AGTATCTTCTCTCGAAGAAAAAAAAATACCAAAATTTCAGAATTTGAATTTACGATCTATTCATTCAATA

TTTCCCTTTTTGGAGGACAAATTATCGCATTTAAATTATGTGTCAGATATACTAATACCTTATCCCATCC

ATCTGAAAATCTTGGTTCAAATCCTTCAATGCTGGATCCAAGATGTTCCTTCTTTACATTTATTGCGATT

CTTTCTTCACGAATATCATAATTGGAATAGTCTTATTACTCCAAATAATTCTATTTTTTTTTCAAAAGAA

AATAAAAGACTATTTCGGTTCCCATATAATTCTTATGTATCTGAATGCGAATTTGTATTAGTTTTTCTTC

GTAAACAATCCTCTTATTTACGATTAACATCTTCTGGAGCTTTTCTTGAGCGAATACATTTCTATGGAAA

AATAGAACATCTTATAGTAGTGCGCCGTAATTATTTTCAGAGGACCCTATGGTTCTTCAAGGATCCCTTC

ATGCATTATGTTCGATATCAAGGAAAAGCAATTCTGGTTTCAAAGGGGACTCATCTTCTGATGAAGAAAT

GGAAATGTCACCTTGTAAATTTCTGGCAATATTATTTTCACTTTTGGTCTCAACCGTACAGGATCCATAT

AAACCAATTATCAAGCTGTTCTTTCCATTTTCTAGGTTATCTTTCAAGTGTACTAATAAATCCTTCGGCG

GTAAGGAATCAAATGCTAGAGAATTCATTTCTAATAGATACTGTTATTAAAAAATTCGATACCAGAGTCC

CAGTTATTCCTCTTATTGGATCATTGTCTAAAGCTAAATTTTGTACCGTATCGGGGCATCCTATTAGTAA

GCCGATCTGGACCAATTTATCAGATTGTGATATTATTGATCGATTTGGTCGGATATGTAGAAATCTTTCT

CATTATCACAGTGGATCCTCAAAAAAACAGAGTTTGTATCGAATAAAGTATATACTTCGATTTTCGTGTG

CTAGAACTTTGGCTCGTAAACATAAAAGTACAGTACGCGCTTTTTTGCAAAGATTAGGTTCGGGATTATT

AGAAGAATTCTTTACGGAAGAAGAACAAGTTGTTTCTTTGATCTTCCCAAAAACAACTTCTTTTTCTTTA

CATGAATCACATATAGAACGTATTTGGTATTTGGATATTATCCGTATCAATGACCTGGTGAATTATTCAT

AATGGGTTTGTTTGGTGACGTGATGAGACTTATGAATAGTCTGGAAATGATCTATAAATGATCAAGAGAG

AAAAAAATTCATGAATTTTCATTCTGAAATGCTCATTGCAGTAGTGTAGTGGTTGAATCAACTGAGTAGT

AAAAATTATTATACTTTCTTCTCGGGACCCAAGTTTTATATTATACATAGGTAAAGTCGTGTGC

>Gaussia_princeps_DQ178694.1

CGTTCTGACCATATTGCACTATGTATCATTTGATAACCCAAAAAATGAAATGGGTCCTGCCTCTGGTTCA

AGTAGAAATGTAAATGGAAGAATTACAAGGATATTTAGAAAAAGATAGATCTCGGCAACAACACTTTCTA

TATCCGCTTCTCTTTAAGGAGTATATTTACACATTTGCTCATGATCGTGGTTTAAATGGTTTGATTTTTT

ACGAATCCACGGAAATTTTTGGTTATGACAATAAATCTAGTTCAGTACTTGTGAAACGTTCAATTATTCG

AATGTATCAACAGAATTATTTGATTTATTCGGTTAATGATTCTAACCAAAATCGATTCGTTGGGCACAAC

AATTATTTTTATTTTCATTTTTATTCTCAGATGATATTGGAAGGTTTTGCAGTCATTGTGGAAATTCCAT

TCTTGCTGCGATTAGTATCTTCTCTCGAAGAAAAAAAAATACCAAAATCTCATAATTTTAATTTACGATC

TATTCATTCAATATTTCCCTTTTTGGAGGACAAATTATCGCATTTAAATTATGTGTCAGATATACTAATA

CCTTATCCCATCCATCTGAAAATCTTGGTTCAAATCCTTCAATGCTGGATCAAAGATGTTCCTTCTTTAC

ATTTATTGCGATTCTTTCTTCACGAATATCATAATTGGAATAGTCTTATTACTCCAAATAATTCTATTTT

TTTTTCAAAAGAAAATAAAAGACTATTTCGGTTCCCATATAATTCTTATGTATCTGAATGCGAATTTGTA

TTAGTTTTTCTTCGTAAACAATCCTCTTATTTACGATTAACATCTTCTGGAGCTTTTCTTGAGCGAANAC

ATTTCTATGGAAAAATAGAACATCTTATAGTAGTGCGCCGTAATTATTTTCAGAGGACCCTATGGTTCTT

CAAGGATCCCTTCATGCATTATGTTCGATATCAAGGAAAAGCAATTCTGGTTTCAAAGGGGACTCATCTT

CTGATGAAGAAATGGAAATGTCACCTTGTCAATTTCTGGCAATATTATTTTCACTTTTGGTCTCAACCGT

ACAGGATCCATATAAACCAATTATCAAGCTGTTCTTTCCATTTTCTAGGTTATCTTTCAAGTGTACTAAT

AAATCCTTCGGCGGTAAGGAATCAAATGCTAGAGAATTCATTTCTAATAGATACTGTTATTAAAAAATTC

GATACCAGAGTCCCAGTTATTCCTCTTATTGGATCATTGTCTAAAGCTAAATTTTGTACCGTATCGGGGC

ATCCTATTAGTAAGCCGATCTGGACCAATTTATCAGATTGTGATATTATTGATCGATTTGGTCGGATATG

TAGAAATCTTTCTCATTATCACAGTGGATCCTCAAAAAAACAGAGTTTGTATCGAATAAAGTATATACTT

CGATTTTCGTGTGCTAGAACTTTGGCTCGTAAACATAAAAGTACAGTACGCGCTTTTTTGCAAAGATTAG

GTTCGGGATTATTAGAAGAATTCTTTACGGAAGAAGAACAAGTTGTTTCTTTGATCTTCCCAAAAACAAC

TTCTTTTTCTTTACATGAATCACATATAGAACGTATTTGGTATTTGGATATTATCCGTATCAATGACCTA

GTGAATTATTCATAATGGGTTTGTTTGGTGACGTGATGAGACTTATGAATAGTCTGGAAATGATCTATAA

ATGATCAAGAGAGAAAAAAATTCATGAATTTTCATTCTGAAATGCTCATTGCAGTAGTGTAGTGGTTGAA

TCAACTGAGTAGTAAAAATTATTATACTTTCTTCTCGGGACCCAAGTTTTATATTATACATAGGTAAAGT

CGTGTGCAATG

>Gaussia_maya_AM114624.1

GACCATATTGCACTATGTATCATTTGATAACCCAAAAAATGAAATGGGTCCTGCCTCTGGTTCAAGTAGA

AATGTAAATGGAAGAATTACAAGGATATTTAGAAAAAGATAGATCTCGGCAACAACACTTTCTATATCCG

CTTCTCTTTAAGGAGTATATTTACACATTTGCTCATGATCGTGGTTTAAATGGTTCGATTTTTTACGAAT

CCACGGAAATTTTTGGTTATGACAATAAATCTAGTTCAGTACTTGTGAAACGTTCAATTATTCGAATGTA

TCAACAGAATTATTTGATTTATTCGGTTAATGATTCTAACCAAAATCGATTCGTTGGGCACAACAATTAT

TTTTATTTTCATTTTTATTCTCAGATGATATTGGAAGGTTTTGCAGTCATTGTGGAAATTCCATTCTTGC

TGCGATTAGTATCTTCTCTCGAAGAAAAAAAAATACCAAAATCTCAGAATTTGAATTTACGATCTATTCA

TTCAATATTTCCCTTTTTGGAGGACAAATTATCGCATTTAAATTATGTGTCAGATATACTAATACCTTAT

CCCATCCATCTGAAAATCTTGGTTCAAATCCTTCAATGCTGGATCCAAGATGTTCCTTCTTTACATTTAT

TGCGATTCTTTCTTCACGAATATCATAATTGGAATAGTCTTATTACTCCAAATAATTCTATTTTTTTTTC

AAAAGAAAATAAAAGACTATTTCGGTTCCCATATAATTCTTATGTATCTGAATGCGAATTTGTATTAGTT

TTTCTTCGTAAACAATCCTCTTATTTACGATTAACATCTTCTGGAGCTTTTCTTGAGCGAATACATTTCT

ATGGAAAAATAGAACATCTTATAGTAGTGCGCCGTAATTATTTTCAGAGGACCCTATGGTTCTTCAAGGA

TCCCTTCATGCATTATGTTCGATATCAAGGAAAAGCAATTCTGGTTTCAAAGGGGACTCATCTTCTGATG

AAGAAATGGAAATGTCACCTTGTAAATTTCTGGCAATATTATTTTCACTTTTGGTCTCAACCGTACAGGA

TCCATATAAACCAATTATCAAGCTGTTCTTTCCATTTTCTAGGTTATCTTTCAAGTGTACTAATAAATCC

TTCGGCGGTAAGGAATCAAATGCTAGAGAATTCATTTCTAATAGATACTGTTATTAAAAAATTCGATACC

AGAGTCCCAGTTATTCCTCTTATTGGATCATTGTCTAAAGCTAAATTTTGTACCGTATCGGGGCATCCTA

TTAGTAAGCCGATCTGGACCAATTTATCAGATTGTGATATTATTGATCGATTTGGTCGGATATGTAGAAA

TCTTTCTCATTATCACAGTGGATCCTCAAAAAAACAGAGTTTGTATCGAATAAAGTATATACTTCGATTT

TCGTGTGCTAGAACTTTGGCTCGTAAACATAAAAGTACAGTACGCGCTTTTTTGCAAAGATTAGGTTCGG

GATTATTAGAAGAATTCTTTACGGAAGAAGAACAAGTTGTTTCTTTGATCTTCCCAAAAACAACTTCTTT

TTCTTTACATGAATCACATATAGAACGTATTTGGTATTTGGATATTATCCGTATCAATGACCTGGTGAAT

TATTCATAATGGGTTTGTTTGGTGACGTGATGAGACTTATGAATAGTCTGGAAATGATCTATAAATGATC

AAGAGAGAAAAAAATTCATGAATTTTCATTCTGAAATGCTCATTGCAGTAGTGTAGTGGTTGAATCAACT

GAGTAGTAAAAATTATTATACTTTCTTCTCGGGACCCAAGTTTTATATTATACATAGGTAAAGTC

>Gaussia_attenuata_DQ178692.1

GTTCTGACCATATTGCACTATGTATCATTTGATAACCCAAAAAATGAAATGGGTCCTGCCTCTGGTTCAA

GTAGAAATGTAAATGGAAGAATTACAAGGATATTTAGAAAAAGATAGATCTCGGCAACAACACTTTCTAT

ATCCGCTTCTCTTTAAGGAGTATATTTACACATTTGCTCATGATCGTGGTTTAAATGGTTCGATTTTTTA

CGAATCCACGGAAATTTTTGGTTATGACAATAAATCTAGTTCAGTACTTGTGAAACGTTCAATTATTCGA

ATGTATCAACAGAATTATTTGATTTATTCGGTTAATGATTCTAACCAAAATCGATTCGTTGGGCACAACA

ATTATTTTTATTTTCATTTTTATTCTCAGATGATATTGGAAGGTTTTGCAGTCATTGTGGAAATTCCATT

CTTGCTGCGATTAGTATCTTCTCTCGAAGAAAAAAAAATACCAAAATCTCATAATTTTAATTTACGATCT

ATTCATTCAATATTTCCCTTTTTGGAGGACAAATTATCGCATTTAAATTATGTGTCAGATATACTAATAC

CTTATCCCATCCATCTGAAAATCTTGGTTCAAATCCTTCAATGCTGGATCAAAGATGTTCCTTCTTTACA

TTTATTGCGATTCTTTCTTCACGAATATCATAATTGGAATAGTCTTATTACTCCAAATAATTCTATTTTT

TTTTCAAAAGAAAATAAAAGACTATTTCGGTTCCCATATAATTCTTATGTATCTGAATGCGAATTTGTAT

TAGTTTTTCTTCGTAAACAATCCTCTTATTTACGATTAACATCTTCTGGAGCTTTTCTTGAGCGAACACA

TTTCTATGGAAAAATAGAACATCTTATAGTAGTGCGCCGTAATTATTTTCAGAGGACCCTATGGTTCTTC

AAGGATCCCTTCATGCATTATGTTCGATATCAAGGAAAAGCAATTCTGGTTTCAAAGGGGACTCATCTTC

TGATGAAGAAATGGAAATGTCACCTTGTCAATTTCTGGCAATATTATTTTCACTTTTGGTCTCAACCGTA

CAGGATCCATATAAACCAATTATCAAGCTGTTCTTTCCATTTTCTAGGTTATCTTTCAAGTGTACTAATA

AATCCTTCGGCGGTAAGGAATCAAATGCTAGAGAATTCATTTCTAATAGATACTGTTATTAAAAAATTCG

ATACCAGAGTCCCAGTTATTCCTCTTATTGGATCATTGTCTAAAGCTAAATTTTGTACCGTATCGGGGCA

TCCTATTAGTAAGCCGATCTGGACCAATTTATCAGATTGTGATATTATTGATCGATTTGGTCGGATATGT

AGAAATCTTTCTCATTATCACAGTGGATCCTCAAAAAAACAGAGTTTGTATCGAATAAAGTATATACTTC

GATTTTCGTGTGCTAGAACTTTGGCTCGTAAACATAAAAGTACAGTACGCGCTTTTTTGCAAAGATTAGG

TTCGGGATTATTAGAAGAATTCTTTACGGAAGAAGAACAAGTTGTTTCTTTGATCTTCCCAAAAACAACT

TCTTTTTCTTTACATGAATCACATATAGAACGTATTTGGTATTTGGATATTATCCGTATCAATGACCTAG

TGAATTATTCATAATGGGTTTGTTTGGTGACGTGATGAGACTTATGAATAGTCTGGAAATGATCTATAAA

TGATCAAGAGAGAAAAAAATTCATGAATTTTCATTCTGAAATGCTCATTGCAGTAGTGTAGTGGTTGAAT

CAACTGAGTAGTAAAAATTATTATACTTTCTTCTCGGGACCCAAGTTTTATATTATACATAGGTAAAGTC

GTGTGCAATG

>Geonoma_congesta_AM114655.1

AACCCAAAAAATGAAATGGGTCCTGCCTCTGGTTCAAGTAGAAATGTAAATGGAAGAATTACAAGGATAT

TTAGAAAAAGATAGATCTCGGCAACAACACTTTCTATATCCGCTTCTCTTTAAGGAGTATATTTACACAT

TTGCTCATGATCGTGGTTTAAATGGTTCGATTTTTTACGAATCCACGGAAATTTTTGGTTATGACAATAA

ATCTAGTTCAGTACTTGTGAAACGTTCAATTATTCGAATGTATCAACAGAATTATTTGATTTATTCGGTT

AACGATTCTAACCAAAATCGATTCGTTGGACACAACAATTATTTTTATTTTCATTTTTATTCTCAGATGA

TATTGGAAGGTTTTGCAGTCATTGTGGAAATTCCATTCTTGCTGCAATTAGTATCTTCCCTCGAAGAAAA

AAAAATACCAAAATCTCAGAATTTGAATTTACGATCTATTCATTCAATATTTCCCTTTTTGGAGGACAAA

TTATCGCATTTAAATTATGTGTCAGATATACTAATACCTTATCCCATCCATCTGAAAATCTTGGTTCAAA

TCCTTCAATGCTGGATCCAAGATGTTCCTTCTTTACATTTATTGCGATTCTTTCTTCACGAATATCATAA

TTGGAATAGTCTTATTACTCCGAATAATTCTATTTTTTTTTTTTCAAAAGAAAATAAAAGACTATTTCGG

TTCCCATATAATTCTTATGTATCTGAATGCGAATTTTTATTAGTTTTTCTTCGTAAACAATCTTCTTATT

TACGATTAACATCTTCTGGAGCTTTTCTTGAGCGAACACATTTCTATGGAAAAATAGAACATCTTATAGT

AGTGCGCCGTAATTATTTTCAGAAGACCCTATGGTTCTTCAAGGATCCCTTCATGCATTATGTTCGATAT

CAAGGAAAAGCAATTCTGGTTTCAAGGGGGACTCATCTTCTGATGAAGAAATGGAAATGTCACCTTGTCA

ATTTCTGGCAATATTATTTTCACTTTTGGTCTCAACCGTACAGGATCCATATAAACCAATTATCAAGCTG

TTCTTTCTATTTTCTAGGTTATCTTTCAAGTGTACTAATAAATCCTTCGGCGGTAAGGAATCAAATGCTA

GAGAATTCATTTCTAATAGATACTGTTATTAAAAAATTCGATACCAGAGTCCCAGTTATTCCTCTTATTG

GATCATTGTCTAAAGCTAAATTTTGTACCGTATCGGGGCATCCTATTAGTAAGCCGATCTGGACCAATTT

ATCAGATTGCGATATTATTGATCGATTTGGTCGGATATGTAGAAATCTTTCTCATTATCACAGTGGATCC

TCAAAAAAACAGAGTTTGTATCGAATAAAGTATATACTTCGATTTTCGTGTGCTAGAACTTTGGCTCGTA

AACATAAAAGTATGGTACGCGCTTTTTTGCAAAGATTAGGTTCGGGATTATTAGAAGAATTCTTTACGGA

AGAAGAACAAGTTGTTTCTTTGATCTTCCCAAAAACTACTTCTTTTTCTTTACATGAATCACATATAGAA

CGTATTTGGTATTTGGATATTATCCGTATCAATGACCTGGTGAATTATTCATAATGGGTTTGGTGACGTG

ATGAGACTTATGAATAGAATAGAAATGATCTATAAATGATCAAGAGAGAAAAAAATTCATGAATTTTCAT

TCTGAAATGCTCATTGCAGTAGTGTAGTGGTTGAATCAACTGAGTAGTCAAAATTATTATACTTTCTTCT

CGGGACCCAAGTTTTATATTATACATAGGTAAAGTC

>Hemithrinax_compacta_AM114559.1

GACCATATTGCACTATGTATCATTTGATAACCCCAAAAATGAAATAGGTCCCGCCTCTGGTTCAAGTAGA

AATGGAAATGGAAGAATTACAAGGATATTTAAAAAAAGATAGATCTCGGCAACAACACTTTCTATATCCG

CTTCTCTTTAAGGAATATATTTACACATTTGCTCATGATCGTGGTTTAAATGGTTCGATTTTTTACGAAT

CCACGGAAATTTTTGGTTATGACAATAAATCTAGTTCAGTACTTGTGAAACGTTCAATTATTCGAATGTA

TCAACAGAATTATTTGATTTATTCGGTTAATGATTCTAACCAAAATCGATTCGTTGGGCACAACAATTAT

TTTTATTTTCATTTTTATTCTCAGATGATATTGGAAGGTTTTGCAGTCATTGTGGAAATTCCATTCTTGC

TGCGATTAGTATCTTCCCTCGAAGAAAAAAAAATACCAAAATCTCAAAATTTGAATTTACGATCTATTCA

TTCAATATTTCCCTTTTTGGAGGACAAATTATCGCATTTAAATTATGTGTCAGATATACTAATACCTTAT

CCCATACATCTGAAAATCTTGGTTCAAATCCTTCAATTCTGGATACAAGATGTTCCTTCTTTACATTTAT

TGCGATTCTTTCTTCACGAATATCATAATTGGAATAGTCTTATTACTCCGAATAATTCTATTTTTCCTTT

TTTACTTTTTTCAAAAGAAAATAAAAGACTATTTCGGTTCCCATATAATTCTTATGTATCTGAATGCGAA

TTTTTATTAGTTTTTCTTCGTAAACAATCTTCTTATTTACGATTAACATCTTCTGGAGCTTTTCTTGAGC

GAACACATTTCTATGGAAAAATAGAACATCTTATAGTAGTGCGCCGTAATTATTTTCAGAAGACCCTATG

GTTCTTCAAGGATCCCTTCATGCATTATGTTCGATATCAAGGAAAAGCAATTCTGGTTTCAAAGGGGACT

CATCTTCTGATGAAGAAATGGAAATGTCACCTTGTCAATTTCTGGCAATATTATTTTCACTTTTGGTCTC

AACCGTACAGGATCCATATAAAGCAATTATCAAACTGTTCTTTCTATTTTTTAGGTTATCTTTCAAGTGT

ACTAATAAATCCTTCGGCGGTAAGGAATCAAATGCTAGAGAATTCATTTCTAATAGATACTCTTATTAAA

AAATTCGATACCAGAGTCCCAGTTATTCCTCTTATTGGATCATTGTCTAAAGCTAAATTTTGTACCGTAT

CGGGGCATCCTATTAGTAAGCCGATCTGGACCGATTTATCAGATTGCGATATTATTGATCGATTTGGTCG

GATATGTAGAAATCTTTCTCATTATCACAGTGGATCCTCAAAAAAACAGAGTTTGTATCGAATAAAGTAT

ATACTTCGATTTTCGTGTGCTAGAACTTTGGCTCGTAAACATAAAAGTACGGTACGCGCTTTTTTGCAAA

GATTAGGTTCAGGATTATTAGAAGAATTTTTTATGGAAGAAGAAGAAGTTGTTTCTTTGATCTTCCCAAA

AACAACTTCTTTTTCTTTACATGAATCACATATAGAACGTATTTGGTATTTGGATATTATCCGTATCAAT

GACCTGGTGAATTATTCATAATTGGTTTGGTGACGTGATGAGACTTATGAATAGAAAATAGAATAGAAAT

GATCTATAAATGATCAAGAGAGAAAAAAATTCATGAATTTTCATTCTGAAATGCTCATTGCAGTAGTGTA

GTGGTTGAATCAACTGAGTAGTCAAAATTATTATACTTTCTTCTCGGGACCCAAGTTTTATATTATACAT

AGGTAAAGTC

>Hyospathe_macrorhachis_AM114646.1

GACCATATTGCACTATGTATCATTTGATAACCCCCAAAATGAAATGGGTCCTGCCTCTGGTTCAAGTAGA

AATGTAAATGGAAGAATTACAAGGATATTTAGAAAAAGATAGATCTCGGCAACAACACTTTCTATATCCG

CTTCTCTTTAAGGAGTATATTTACACATTTGCTCATGATCGTGGTTTAAATGGTTCGATTTTTTACGAAT

CCACGGAAATTTTTGGTTATGACAATAAATCTAGTTCAGTACTTGTGAAACGTTCAATTATTCGAATGTA

TCAACAGAATTATTTGATTTATTCGGTTAACGATTCTAACCAAAATCGATTCATTGGGCACAACAATTAT

TTTTATTTTTATTCTCAGATGATATTGGAAGGTTTTGCAGTCATTGTGGAAATTCCATTCTTGCTGCGAT

TAGTATCTTCCCTCGAAGAAAAAAAAATACCAAAATCTCAGAATTTGAATTTACGATCTATTCATTCAAT

ATTTCCCTTTTTGGAGGACAAATTATCACATTTAAATTATGTGTCAGATATACTAATACCTTATCCCATC

CATCTGAAAATCTTGGTTCAAATCCTTCAATGCTGGATCCAAGATGTTCCTTCTTTACATTTATTGCGAT

TCTTTCTTCACGAATATCATAATTGGAATAGTCTTATTACTCCGAATAATTCTATTTTTTATTTTTCAAA

AGAAAATAAAAGACTATTTCGGTTCCCATATAATTCTTATGTATCTGAATGCGAATTTTTATTCGTTTTT

CTTCGTAAACAATCTTCTTATTTACGATTAACATCTTCTGGAGCTTTTCTTGAGCGAACACATTTCTATG

GAAAAATGGAACATCTTATAGTAGTGCGCCGTAATTATTTTCAGAAGACCCTATGGTTCTTCAAGGATCC

CTTCATGCATTATGTTCGATATCAAGGAAAAGCAATTCTGGTTTCAAAGGGGACTCATCTTCTGATGAAG

AAATGGAAATGTCACCTTGTCAATTTCTGGCAATATTATTTTCACTTTTGGTCTCAACCGTACAGGATCC

ATATAAACCAATTATCAAGCTGTTCTTTCTATTTTCTAGGTTATCTTTCAAGTGTACTAATAAATCCTTC

GGCGGTAAGGAATCAAATGCTAGAGAATTTATTTCTAATAGATACTGTTATTAAAAAATTCGATACCAGA

GTCCCAGTTATTCCTCTTATTGGATCATTGTCTAAAGCTAAATTTTGTACCGTATCGGGGCATCCTATTA

GTAAGCCGATCTGGACCAATTTATCAGATTGCGATATTATTGATCGATTTGGTCGGATATGTAGAAATCT

TTCTCATTATCACAGCGGATCCTCAAAAAAACAGAGTTTGTATCGAATAAAGTATATACTTCGATTTTCG

TGTGCTAGAACTTTGGCTCGTAAACATAAAAGTATGGTACGCGCTTTTTTGCAAAGATTAGGTTCGGGAT

TATTAGAAGAATTCTTTACGGAAGAAGAACAAGTTGTTTCTTTGATCTTCCCCAAAAGAACTTCTTTTTC

TTTACATGAATCACATATAGAACGTATTTGGTATTTGGATATTATCCGTATCAATGACCTGGTGAATTAT

TCATAATGGGTTTGGTGACGTGATGAGACTTATGAATAGAATAGAAATGATCTATAAATGATCAAGAGAG

AAAAAAATTCATGAATTTTCATTCTGAAATGCTCATTGCAGTAGTGTAGTGGTTGAATCAACTGAGTAGT

CAAAATTATTATACTTTCTTCTCGGGACCCAAGTTTTATATTATACATAGGTAAAGTC

>Iriartea_deltoidea_AM114617.1

GACCATATTGCACTATGTATCATTTGATAACCCAAAAAATGAAATGGGTCCTGCCTCTGGTTCAAGTAGA

AATGTAAATGGAAGAATTACAAGGATATTTAGAAAAAGATAGATCTCGGCAACAACACTTTCTATATCCG

CTTCTCTTTAAGGAGTATATTTACACATTTGCTCATGATCGTGGTTTAAATGGTTCGATTTTTTACGAAT

CCACGGAAATTTTTGGTTATGACAATAAATCTAGTTCAGTACTTGTGAAACGTTCAATTATTCGAATGTA

TCAACAGAATTTTTTGATTTATTCGGTTAATGATTCTAACCAAAATCGATTCGTTGGGCACAACAATTAT

TTTTATTTTCATTTTTATTCTCAGATGATATTGGAAGGTTTTGCAGTCATTGTGGAAATTCCATTCTTGC

TGCGATTAGTATCTTCCCTCGAAGAAAAAAAAATACCAAAATCTCAGAATTTGAATTTACGATCTATTCA

TTCAATATTTCCCTTTTTGGAGGACAAATTATCGCATTTAAATTATGTGTCAGATATACTAATACCTTAT

CCCATCCATCTGAAAATCTTGGTTCAAATCCTTCAATGCTGGATCCAAGATGTTCCTTCTTTACATTTAT

TGCGATTCTTTCTTCACGAATATCATAATTGGAATAGTCTTATTACTCCGAATAATTCTATTTTTTCAAA

AGAAAATAAAAGACTATTTCGGTTCCCATATAATTCTTATGTATCTGAATGCGAATTTGTATTAGTTTTT

CTTCGTAAACAATCTTCTTATTTACGATTAACATCTTCTGGAGCTTTTCTTGAGCGAAAACATTTCTATG

GAAAAATAGAACATCTTATAGTAGTGCGCCGTAATTATTTTCAGAAGACCCTATGGTTCTTCAAGGATCC

CTTCATGCATTATGTTCGATATCAAGGAAAAGCAATTCTGGTTTCAAAGGGGACTCATCTTCTGATGAAG

AAATGGAAATGTCACCTTGTCAATTTCTGGCAATATTATTTTCACTTTTGGTCTCAACCGTACAGGATCC

ATATAAACCAATTATCAAACTGTTCTTTCTATTTTCTAGGTTATCTTTCAAGTGTACTAATAAATCCTTC

GGCGGTAAGGAATCAAATGCTAGAGAATTCATTTCTAATAGATACTGTTATTAAAAAATTCGATACCAGA

GTCCCAGTTATTCCTCTTATTGGATCATTGTCTAAAGCTAAATTTTGTACCGTATCGGGGCATCCTATTA

GTAAGCCGATCTGGACCAATTTATCAGATTGCGATATTATTGATCGATTTGGTCGGATATGTAGAAATCT

TTCTCATTATCACAGTGGATCCTCAAAAAAACAGAGTTTGTATCGAATAAAGTATATACTTCGATTTTCG

TGTGCTAGAACTTTGGCTCGTAAACATAAAAGTATGGTACGCGCTTTTTTGCAAAGATTAGGTTCGGGAT

TATTAGAAGAATTCTTTATGGAAGAAGAACAAGTTGTTTCTTTGATCTTCCCAAAAACAACTTCTTTTTC

TTTACATGAATCACATATAGAACGTATTTGGTATTTGGATATTATCCGTATCAATGACCTGGTGAATTAT

TCATAATGGGTTTGGTGACGTGATGAGACTTATGAATAGAATAGAAATGATCTATAAATGATCAAGAGAG

AAAAAAATTCATGAATTTTCATTCTGAAATGCTCATTGCAGTAGTGTAGTGGTTGAATCAACTGAGTAGT

CAAAATTATTATACTTTCTTCTCGGGACCCAAGTTTTATATTATACATAGGTAAAGTC

>Iriartella_stenocarpa_AM114615.1

GACCATATTGCACTATGTATCATTTGATAACCCCAAAAATGAAATGGATCCTGCCTCTGGTTCAAGTAGA

AATGTAAATGGAAGAATTACAAGGATATTTAGAAAAAGATAGATCTCGGCAACAACACTTTCTATATCCG

CTTTTCTTTAAGGAGTATATTTACACATTTGCTCATGATCGTGGTTTAAATTTAAATGGTTCGATTTTTT

ACGAATCCACGGAAATTTTTGGTTATGACAATAAATCTAGTTCAGTACTTGTGAAACGTTCAATTATTCG

AATGTATCAACAGAATTTTTTGATTTATTCGGTTAATGATTCTAACCAAAATCGATTCGTTGGGCACAAC

AATTATTTTTATTTTCATTTTTATTCTCAGATGATATTGGAAGGTTTTGCAGTCATTGTGGAAATTCCAT

TCTTGCTGCGATTAGTATCTTCCCTCGAAGAAAAAAAAATACCAAAATCTCAGAATTTGAATTTACGATC

TATTCATTCAATATTTCCCTTTTTGGAGGACAAATTATCGCATTTAAATTATGTGTCAGATATACTAATA

CCTTATCCCATCCATCTGAAAATCTTGGTTCAAATCCTTCAATGCTGGATCCAAGATGTTCCTTCTTTAC

ATTTATTGCGATTCTTTCTTCACGAATATCATAATTGGAATAGTCTTATTACTCCGAATAATTCTATTTT

TTCAAAAGAAAATAAAAGACTATTTCGGTTCCCATATAATTCTTATGTATCTGAATGCGAATTTGTATTA

GTTTTTCTTCGTAAACAATCTTCTTATTTACGATTAACATCTTCTGGAGCTTTTCTTGAGCGAAAACATT

TCTATGGAAAAATAGAACATCTTATAGTAGTGCTCCGTAATTATTTTCAGAAAACCCTATGGTTCTTCAA

GGATCCCTTCATGCATTATGTTCGATATCAAGGAAAAGCAATTCTGGTTTCAAAGGGGACTCATCTTCTG

ATGAAGAAATGGAAATGTCACCTTGTCAATTTCTGGCAATATTATTTTCACTTTTGGTCTCAACCGTACA

GGATCCATATAAACCAATTATCAAACTGTTCTTTCTATTTTCTAGGTTATCTTTCAAGTGTACTAATAAA

TCCTTCGGCGGTAAGGAATCAAATGCTAGAGAATTCATTTCTAATAGATACTGTTATTAAAAAATTCGAT

ACCAGAGTCCCAGTTATTCCTCTTATTGGATCATTGTCTAAAGCTAAATTTTGTACCGTATTGGGGCATC

CTATTAGTAAGCCGATCTGGACCAATTTATCAGATTGCGATATTATTGATCGATTTGGTCGGATATGTAG

AAATCTTTCTCATTATCACAGTGGATCCTCAAAAAAACAGAGTTTGTATCGAATAAAGTATATACTTCGA

TTTTCGTGTGCTAGAACTTTGGCTCGTAAACATAAAAGTATGGTACGCACTTTTTTGCAAAGATTAGGTT

CGGGATTATTAGAAGAATTCTTTATGAAAGAAGAACAAGCTCTTTCTTTGATCTTCCCAAAAACAACTTC

TTTTTCTTTACATGAATCACATATAGAACGTATTTGGTATTTGGATATTATCCGTATCAATGACCTGGTG

AATTATTCATAATGGGTTTGGTGACGTGATGAGACTTATGAATAGAATAGAAATGATCTATAAATGATCA

AGAGAGAAAAAAATTCATGAATTTTCATTCTGAAATGCTCATTGCAGTAGTGTAGTGGTTGAATCAACTG

AGTAGTCAAAATTATTATACTTTCTTCTCGGGACCCAAGTTTTATATTATACATAGGTAAAGCC

>Itaya_amicorum_AM114564.1

GACCATATTGCACTATGTATCATTTGATAACCCCAAAAATTAAATAGGTCCCGCCTCTGGTTCAAGTAGA

AATGGAAATGGAAATGGAAGAATTACAAGGATATTTAGAAAAAGATAGATCTCGGCAACAACACTTTCTA

TATCCGCTTCTCTTTAAGGAGTATATTTACACATTTGCTCATGATCGTGGTTTAAATGGTTCGATTTTTT

ACGAATCCACGGAAATTTTTGGTTATGACAATAAATCTAGTTCAGTACTTGTGAAACGTTCAATTATTCG

AATGTATCAACAGAATTATTTGATTTATTCGGTTAATGATTCTAACCAAAATCGATTCGTTGGGCACAAC

AATTATTTTTATTTTCATTTTTATTCTCAGATGATATTGGAAGGTTTTGCAGTCATTGTGGAAATTCCAT

TCTTGCTGCGATTAGTATCTTCCCTCGAAGAAAAAAAAATACCAAAATCTCAAAATTTGAATTTACGATC

TATTCATTCAATATTTCCCTTTTTGGAGGACAAATTATCGCATTTAAATTATGTGTCAGATATACTAATA

CCTTATCCCATACATCTGAAAATCTTGGTTCAAATCCTTCAATTCTGGATCCAAGATGTTCCTTCTTTAC

ATTTATTGCGATTCTTTCTTCACGAATATCATAATTGGAATAGTCTTATTACTCCGAATAATTCTATTTT

TCCTTTTTTACTTTTTTCAAAAGAAAATAAAAGACTATTTCGGTTCCCATATAATTCTTATGTATCTGAA

TGCGAATTTTTATTAGTTTTTCTTCGTAAACAATCTTCTTATTTACGATTAACATCTTCTGGAGCTTTTC

TTGAGCGAACACATTTCTATGGAAAAATAGAACATCTTATAGTAGTAGTGCGCCGTAATTATTTTCAGAA

GACCCTATGGTTCTTCAAGGATCCCTTCATGCATTATGTTCGATATCAAGGAAAAGCAATTCTGGTTTCA

AAGGGGACTCATCTTCTGATGAAGAAATGGAAATGTCACCTTGTCAATTTCTGGCAATATTATTTTCACT

TTTGGTCTCAACCGTACAGGATCCATATAAAGCAATTATCAAACTGTTCTTTCTATTTTTTAGGTTATCT

TTCAAGTGTACTAATAAATCCTTCGGCGGTAAGGAATCAAATGCTAGAGAATTCATTTCTAATAGATACT

GTTATTAAAAAATTCGATACCAGAGTCCCAGTTATTCCTCTTATTGGATCATTGTCTAAAGCTAAATTTT

GTACCGTATCGGGGCATCCTATTAGTAAGCCGATCTGGACCGATTTATCAGATTGCGATATTATTGATCG

ATTTGGTCGGATATGTAGAAATCTTTCTCATTATCACAGTGGATCCTCAAAAAAACAGAGTTTGTATCGA

ATAAAGTATATACTTCGATTTTCGTGTGCTAGAACTTTGGCTCGTAAACATAAAAGTACGGTACGCGCTT

TTTTGCAAAGATTAGGTTCAGGATTATTAGAAGAATTTTTTATGGAAGAAGAAGAAGTTGTTTCTTTGAT

CTTCCCAAAAACAACTTCTTTTTCTTTACATGAATCACATATAGAACGTATTTGGTATTTGGATATTATC

CGTATCAATGACCTGGTGAATTATTCATAATTGCTTTGGTGACGTGATGAGACTTATGAATAGAAAATAG

AATGGAAATGATCTATAAATGATCAAGAGAGAAAAAAATTCATGAATTTTCATTCTGAAATGCTCATTGC

AGTAGTGTAGTGGTTGAATCAACTGAGTAGTCAAAATTATTATACTTTCTTCTCGGGACCCATGTTTTAT

ATTATACATAGGTAAAGTC

>Juania_australis_AM114608.1

GACCATATTGCACTATGTATCATTTGATAACCCCAAAAATGAAATGGGTCCTGCCTCTGGTTCAAGTAGA

AATGTAAATGGAAGAATTACAAGGATATTTAGAAAAAGATAGATCTCGGCAACAACACTTTCTATATCCG

CTTCTCTTTAAGGAGTATATTTACACATTTGCTCATGATCGTGGTTTAAATGGTTCGATTTTTTACGAAT

CCACGGAAATTTTTTGTTATGACAAAAAATCTAGTTCAGTACTTGTGAAACGTTCAATTATTCGAATGTA

TCAACAGAATTATTTGATTTATTCGGTTAATGATTCTAACCAAAATCGATTCGTTGGGCACAACAATTTT

TTTTATTTTCATTTTTATTCTCAGATGATATTGGAAGGTTTTGCAGTCATTGTGGAAATTCCATTCTTGC

TGCGATTAGTATCTTCCCTCGAAGAAAAAAAAATACCAAAATCTCAGAATTTGAATTTACGATCTATTCA

TTCAATATTTCCCTTTTTGGAGGACAAATTATCGCATTTAAATTATGTGTCAGATATACTAATACCTTAT

CCCATCCATCTGAAAATCTTGGTTCAAATCCTTCAATGCTGGATCCAAGATGTTCCTTCTTTACATTTAT

TGCGATTCTTTCTTCACGAATATCATAATTGGAATAGTCTTATTACTCCGAATAATTCTATTTTTTTTTT

TTCAAAAGAAAATAAAAGACTATTTCGGTTCCCATATAATTCTTATGTATCTGAATGCGAATTTGTATTA

GTTTTTCTTCGTAAACAATCTTCTTATTTACGATTAACATCTTCTGGAGCTTTTCTTGAGCGAACACATT

TCTATGGAAAAATAGAACATCTTATAGTAGTGCGCCGTAATTATTTTCAGAAGACCCTATGGTTCTTCAA

GGATCCCTTCATGCATTATGTTCGATATCAAGGAAAAGCAATTCTGGTCTCAAAGGGGACTCATCTTCTG

ATGAAGAAATGGAAATGTCACCTTGTCAATTTCTGGCAATATTTTTTTCACTTTTGGTCTCAACCGTACA

GGATCCATATAAACCAATTATCAAACTGTTCTTTCTATTTTCTAGGTTATCTTTCAAGTGTACTAATAAA

TCCTTCGGCGGTAAGGAATCAAATGCTAGAGAATTCATTTCTAATAGATACTGTTATTAAAAAATTCGAT

ACCAGAGTCCCAGTTATTCCTCTTATTGGATCATTGTCTAAAGCTAAATTTTGTACCGTATCGGGGCATC

CTATTAGTAAGCCGATCTGGACCAATTTATCAGATTGCGATATTATTGATCGATTTGGTCGGATATGTAG

AAATCTTTCTCATTATCACAGTGGATCCTCAAAAAAACAGAGTTTGTATCGAATAAAGTATATACTTCGA

TTTTCGTGTGCTAGAACTTTGGCTCGTAAACATAAAAGTATGGTACGCGCTTTTTTGCAAAGATTAGGTT

CGGGATTATTAGAAGAATTCTTTACGGAAGAAGAACAAGTTGTTTCTTCGATCTTCCCAAAAACTACTTG

TTTTTCTTTACATGAATCACATATAGAACGTGTTTGGTATTTGGATATTATCCGTATCAATGACCTGGTG

AATTATTCATAATGGGTTTGGTGACGTGATGATACTTATGAATAGAATGGAAATGATCTATAAATGATCA

AGAGAGAAAAAAATTCATGAATTTTCATTCTGAAATGCTCATTGCAGTAGTGTAGTGGTTGAATCAACTG

AGTAGCCAAAATTATTATACTTTCTTCTCGGGACCCAAGTTTTATATTATACATAGGTAAAGTC

>Leopoldinia_pulchra_AM114656.1

GACCATATTGCACTATGTATCATTTGATAACCCCAAAAATGAAATGGGTCCTGCCTCTGGTTCAAGTAGA

AATGTAAATGGAAGAATTACAAGGATATTTAGAAAAAGATAGATCTCGGCAACAACACTTTCTATATCCG

CTTCTCTTTAAGGAGTATATTTACACATTTGCTCATGATCGTGGTTTAAATGGTTCGATTTTTTACGAAT

CCACGGAAATTTTTGGTTATGACAATAAATCTAGTTCAGTACTTGTGAAGCGTTCGATTATTCGAATGTA

TCAACAGAATTATTTGATTTATTCGGTTAACGATTCTAACCAAAATCGATTCGTTGGGCACAACAATTAT

TTTTATTTTCATTTTTATTCTCAGATGATATTGGAAGGTTTTGCAGTCATTGTGGAAATTCCATTCTTGC

TGCGATTAGTATCTTCCCTCGAAGAAAAAAAAATACCAAAATCTCAGAATTTGAATTTACGATCTATTCA

TTCAATATTTCCCTTTTTGGAGGACAAATTATCGCATTTAAATTATGTGTCAGATATACTAATACCTTAT

CCCATCCATCTGAAAATCTTGGTTCAAATCCTTCAATGCTGGATCCAAGATGTTCCTTCTTTACATTTAT

TGCGATTCTTTCTTCACGAATATCATAATTGGAATAGTCTTATTACTCCTAATAATTCTATTTTTTTTTC

AAAAGAAAATAAAAGACTATTTCGGTTCCCATATAATTCTTATGTATCTGAATGCGAATTTGTATTAGTT

TTTCTTCGTAAACAATCTTCTTATTTACGATTAACATCTTCTGGAGCTTTTCTTGAGCGAACACATTTCT

ATGGAAAAATAGAACATCTTATAGTAGTGCGCCGTAATTATTTTCAGAAGACCCTATGGTTCTTCAAGGA

TCCCTTCATGCATTATGTTCGATATCAAGGAAAAGCAATTCTGGTTTCAAAGGGGACTCATCTTCTGATG

AAGAAATGGAAATGTCACCTTGTCAATTTCTGGCAATATTATTTTCACTTTTGGTCTCAACCGTACAGGA

TCCATATAAACCAATTATCAAGCTGTTCTTTCTATTTTCTAGGTTATCTTTCAAGTGTACTAATAAATCC

TTCGGCGGTAAGGAATCAAATGCTAGAGAATTCATTTCTAATAGATACTGTTATTAAAAAATTCGATACC

AGAGTCCCAGTTATTCCTCTTATTGGATCATTGTCTAAAGCTAAATTTTGTACCGTATCGGGGCATCCTA

TTAGTAAGCCGATCTGGACCGATTTATCAGATTGCGATATTATTGATCGATTTGGTCGGATATGTAGAAA

TCTTTCTCATTATCACAGTGGATCCTCAAAAAAACAGAGTTTGTATCGAATAAAGTATATACTTCGATTT

TCGTGTGCTAGAACTTTGGCTCGTAAACATAAAAGTATGGTACGCGCTTTTTTGCAAAGATTAGGTTCGG

GATTATTAGAAGAATTCTTTACGGAAGAAGAACAAGTTGTTTCTTTGATCTTCCCAAAAACAACTTCTTT

TTCTTTACATGAATCACATATAGAACGTATTTGGTATTTGGATATTATCCGTATCAATGACCTGGTGAAT

TATTCATAATGGGTTTGGTGACGTGATGAGACTTATGAATAGAATAGAAATGATCTATAAATGATCAAGA

GAGAAAAAAATTCATGAATTTCCATTCTGAAATGCTCATTGCAGTAGTGTAGTGGTTGAATCAACTGAGT

AGTCAAAATTATTATACTTTCTTCTCGGGACCCAAGTTTTATATTATACATAGGAAAAGTC

>Manicaria_saccifera_AM114645.1

GACCATATTGCACTATGTATCATTTGATAACCCAAAAAATGAAATGGGTCCTGCCTCTGGTTCAAGTAGA

AATGTAAATGGAAGAATTACAAGGATATTTAGAAAAAGATAGATCTCGGCAACAACACTTTCTATATCCG

CTTCTCTTTAAGGAGTATATTTACACATTTGCTCATGATCGTGGTTTAAATGGTTCGATTTTTTACGAAT

CCACGGAAATTTTTGGTTATGACAATAAATCTAGTTCAGTACTTGTGAAACGTTCAATTATTCGAATGTA

TCAACAGAATTATTTGATTTATTCGGTTAACGATTCTAACCAAAATCGATTCGTTGGGCACAATTTTTAT

TTTCATTTTTATTCTCAGATGATATTGGAAGGTTTTGCAGTCATTGTGGAAATTCCATTCTTGCTGCGAT

TAGTATCTTCCCTCGAAGAAAAAAAAATACCAAAATCTCAGAATTTGAATTTACGATCTATTCATTCAAT

ATTTCCCTTTTTGGAGGACAAATTATCGCATTTAAATTATGTGTCAGATATACTAATACCTTATCCCATC

CATCTGAAAATCTTGGTTCAAATCCTTCAATGCTGGATCCAAGATGTTCCTTCTTTACATTTATTGCGAT

TCTTTCTTCACGAATATCATAATTGGAATAGTCTTATTACTCCGAATAATTCTATTTTCTTTTTTTCAAA

AGAAAATAAAAGACTATTTTGGTTCCCATATAATTCTTATGTATCTGAATGCGAATTTGTATTAGTTTTT

CTTCGTAAACAATCTTCTTATTTACGATTAACATCTTCTGGAGCTTTTCTTGAGCGAACACATTTCTATG

GAAAAATAGAACGTCTTATAGTAGTGCGCCATAATTATTTTCAGAAGACCCTATGGTTCTTCAAGGATCC

CTTCATGCATTATGTTCGATATCAAGGAAAAGCAATTCTGGTTTCAAAGGGGACTCATCTTCTGATGAAG

AAATGGAAATGTCACCTTGTCAATTTCTGGCAATATTATTTTCACTTTTGGTCTCAACCGTACAGGATCC

ATATAAACCAATTATCAAGCTGTTCTTTCTATTTTCTAGGTTATCTTTCAAGTGTACTAATAAATCCTTC

GGCGGTAAGGAATCAAATGCTAGAGAATTCATTTCTAATAGATACTGTTATTAAAAAATTCGATACCAGA

GTCCCAGTTATTCCTCTTATTGGATCATTGTCTAAAGCTAAATTTTGTACCGTATCGGGGCATCCTATTA

GTAAGCCGATCTGGACCGATTTATCAGATTGCGATATTATTGATCGATTTGGTCGGATATGTAGAAATCT

TTCTCATTATCACAGTGGATCCTCAAAAAAACAGAGTTTGTATCGAATAAAGTATATACTTCGATTTTCG

TGTGCTAGAACTTTGGCTCGTAAACATAAAAGTATGGTACGCGCTTTTTTGCAAAGATTAGGTTCGGGAT

TATTAGAAGAATTCTTTACGGAAGAAGAACAAGTTGTTTCTTTGATCTTCCCAAAAACAACTTCTTTTTC

TTTACATGAATCACATATAGAACGTATTTGGTATTTGGATATTATCCGTATCAATGACCTGGTGAATTAT

TCATAATAGGTTTGGTGACGTGATGAGACTTATGAATAGAATAGAAATGATCTATAAATGATCAAGAGAG

AAAAAAATTCATGAATTTCCATTCTGAAATGCTCATTGCAGTAGTGTAGTGGTTGAATCAACTGAGTAGT

CAAAATTATTATACTTTCTTCTCGGGACCCAAGTTTTATATTATACATAGGAAAAGTC

>Mauritia_flexuosa_AM114545.1

GACCATATTGCACTATGTATCATTTGATGACCCAAGAAATGGGTCCTGCCTCTGGTTCAATTAGAAATGT

AAATGGAAGAATTACAAGGATATTTAGAAAAAGATATATCTCGGCAACAACACTTCCTATATCCGCTTCT

CTTTAAGGAGTATATTTACACATTTGTTCATGATCGTGGTTTAAATGGTTCGATTTTTTACGAATCCGTG

GAAATTTTGGGTTATGACAATAAATCTAGTTTAGTACTTGTGAAACGTTTAATTATTCGAATGTATCAAC

AGAATTATTTGATTTATTCGGTTAATGATTCTAACCAAAATCGATTCGTTGGGTACTATTTTTATTTTCA

TTTTTATTCTCAGATAATATTGGAAGGTTTTGCAGTCATTGTGGAAATTCCATTCTTGCTGCGATTAGTA

TCTTCCCTCGAAGAAAAAATACCAAAATCTCAGAATTTGAATTTACGATCTATTCATTCAATATTTCCCT

TTTTAGAGGACAAATTATCGCATTTAAATTATGTGTCAGATATACTAATACCTTATCCCATCCATCTGGA

AATCTTGGTTCAAATCCTTCAATGCTGGATCCAAGATGTTCCCTCTTTACATTTATTGCGATTCTTTCTT

CACGAATATCATAATTGGAATAGTCTTATTACTCCGAATAATTCTATTTTTTTTTTTTCAAAAGAAAATA

AAAGACTATTTCGGTTCCTATATAATTCTTATGTATCTGAATGCGAATTTGTATTAGTTTTTCTTCGTAA

ACACTCTTCTTATTTACGATTAACATCTTCTGGAGCTTTTCTTGAGCGAACACATTTCTATGGAAAAATA

GAACATCTTATAGTAGTGCGCCATAATTATTTTCAGAGGACCCTATGGTTCTTCAAGGATCCTTTCATGC

ATTATGTTCGATATCAAGGAAAAGCAATTCTGGTTTCAAAGGGGGCTCATCTTCTGATGAAGAAATGGAA

ATGTCACCTTGTCAATTTCTGGCAATATTATTTTAACTTTTGGTCTCAACCGTACAGGATCCATATAGAC

CAATTATCAAACTGTTCTTTCTATTTTTTAGGTTATCTTTCAAGTGTATTAATAAATCTTTCGACGGTAA

GGAATCAAATGCTAGAGAATTCATTTCTAATGGATACTGTTACTAAAAAATTCGATACCAGAGTCCCAGT

TATTCCTCTTATTGAATCATTGTCTAAAGCTAAATTTTGTACCGTATCGGGGCACCCTATTAGTAAGCCG

ATCTGGACCGATTTATCAGATTGCGATATTATTGATCGATTTGGTCGGATATGTAGAAATCTTTCTCATT

ATCACAGTGGATCCTCAAAAAAACAGAGTTTGTATCGAATAAAGTATATACTTCGACTTTCGTGTGCTAG

AACTTTGGCTCGTAAACATAAAAGTACGGCACGCGCTTTTTTGCAAAGATTAGGCTCGGGATTATTAGAA

GAATTCTTTACGGAAGAAGAACAAGTTGTTTATTTGATCTTCCCCAAAACAACCTCTTTTTCTTTACATG

GATCACATAGAGAACGTATTTGGTATTTGGATATTATCCGTATCAATGACCTGGTGAATCATTCATAATT

GGTTTGGTGACGTGATGAGACTTATAAATAGAATAGAAATGATCTATAAATGATCAAGAGAGAAAAAAAT

TCATGAATTTTCATTCTGAAATGCTCATTGTAGTAGTGTAGTGGTTGAATCAACCGAGTAGTCAAAATTA

TTATACTTTCTTCTCGGGATGTAAGTTTTATATTATACATAGGGAAAGTC

>Neonicholsonia_watsonii_AM114649.1

GACCATATTGCACTATGTATCATTTGATAACCCCAAAAATGAAATGGGTCCAGCCTCTGGTTCAAGTAGA

AATGTAAATGGAAGAATTACAAGGATATTTAGAAAAAGATAGATCTCGGCAACAACACTTTCTATATCCG

CTTCTCTTTAAGGAGTATATTTACACATTTGCTCATGATCGTGGTTTAAATGGTTCGATTTTTTACGAAT

CCACGGAAATTTTTGGTTATGACAATAAATCTAGTTCAGTACTCGTGAAACGTTCAATTATTCGAATGTA

TCAACAGAATTATTTGATTTATTCGGTTAACGATTCTAACCAAAATCGATTCGTTGGGCACAACAATTAT

TTTTATTTTCATTTTTATTCTCAGATGATATTGGAAGGTTTTGCAGTCATTGTGGAAATTCCATTCTTGC

TGCGATTAGTATCTTCCCTCGAAGAAAAAAAAATACCAAAATCTCAGAATTTGAATTTACGATCTATTCA

TTCAATATTTCCCTTTTTGGAGGACAAATTATCGCATTTAAATTATGTGTCAGATATACTAATACCTTAT

CCCATCCATCTGAAAATCTTGGTTCAAATCCTTCAATGCTGGATCCAAGATGTTCCTTCTTTACATTTAT

TGCGATTCTTTCTTCACGAATATCATAATTGGAATAGTCTTATTACTCCGAATAATTCTATTTTTTTTTT

TTCAAAAGAAAATAAAAGACTATTTCGGTTCCCATATAATTCTTATGTATCTGAATGCGAATTTGTATTA

GTTTTTCTTCGTAAACAATCTTCTTATTTACGATTAACATCTTCTGGAGCTTTTCTTGAGCGAACACATT

TCTATGGAAAAATAGAACATCTTATAGTAGTGCGCCGTAATTATTTTCAGAAGACCCTATGGTTCTTCAA

GGATCCCTTCATGCATTATGTTCGATATCAAGGAAAAGCAATTCTGGTTTCAAAGGGGACTCATCTTCTG

ATGAAGAAATGGAAATGTCACCTTGTCAATTTCTGGCAATATTATTTTCACTTTTGGTCTCAACCGTACA

GGATCCATATAAACCAATTATCAAGCTGTTCTTTCTATTTTCTAGGTTATCTTTCAAGTGTACTAATAAA

TCCTTCGGCGGTAAGGAATCAAATGCTAGAGAATTCATTTCTAATAGATACTGTTATTAAAAAATTCGAT

ACCAGAGTCCCAGTTATTCTTCTTATTGGATCATTGTCTAAAGCTAAATTTTGTACCGTATCGGGGCATC

CTATTAGTAAGCCGATCTGGACCAATTTATCGGATTGCGATATTATTGATCGATTTGGTCGGATATGTAG

AAATCTTTCTCATTATCACAGTGGATCCTCAAAAAAACAGAGTTTGTATCGAATAAAGTATATACTTCGA

TTTTCGTGTGCTAGAACTTTGGCTCGTAAACATAAAAGTATGGTACGCACTTTTTTGCAAAGATTAGGTT

CGGGATTATTAGAAGAATTCTTTACGGAAGAAGAACAAGTTGTTTCTTTGATCTTCCCAAAAACAACTTC

TTTTTCTTTACATGAATCACATAAAGAACGTATTTGGTATTTGGATATTATCCGTATCAATGACCTGGTG

AATTATTCATAATGGGTTTGGTGACGTGATGAGACTTATGAATAGAATAGAAATGATCTATAAATGATCA

AGAGAGAAAAAAATTCATGAATTTTCATTCTGAAATGCTCATTGCAGTAGTGTAGTGGTTGAATCAACTG

AGTAGTCAAAATTATTATATTATACTTTCTTCTCGGGACCCAAGTTTTATATTATACATAGGTAAAGTC

>Oenocarpus_bacaba_JQ626537.1

ATATAATTTTTANGTATCTGAATGCGAATTTGTATTAGTTTTTCTTCGTAAACAATCCTCTTATTTACGA

TTAACATCTTCTGGAGCTTTTTCTGAGCGAACACATTTCTATGGAAAAATAGAACATCTTATAGTAGTGC

GCCGTAATTATTTTCAGAAGACCCTATGGTTCTTCAAGGATCCCTTCATGCATTATGTTCGATATCAAGG

AAAAGCAATTCTGGTTTCAAAGGGGACTCATCTTCTGATGAAGAAATGGAAATGTCACCTTGTCAATTTC

TGGCAATATTATTTTCACTTTTGGTCTCAACCGTACAGGATCCATATAAACCAATTATCAAGCTGTTCTT

TCTATTTTCTAGGTTATCTTTCAAGTGTACTAATAAATCCTTCGGCGGTAAGGAATCAAATGCTAGAGAA

TTCATTTCTAATAGATACTGTTATTAAAAAATTCGATACCAGAGTCCCAGTTATTCCTCTTATTGGATCA

TTGTCTAAAGCTAAATTTTGTACCGTATCGGGGCATCCTATTAGTAAGCCGATCTGGACCAATTTATCAG

ATTGCGATATTATTGATCGATTTGGTCGGAT

>Oenocarpus_bataua_JQ626533.1

TACCTTATCCCATCCATCTGAAAATCTTGGTTCAAATCCTTCAATGCTGGATCCAAGATGTTCCTTCTTT

ACATTTATTGCGATTCTTTCTTCACGAATATCATAATTGGAATAGTCTTATTACTCCGAATAATTCTATT

TTTTTTTTTTCAAAAGAAAATAAAAGACTATTCCGGTTCCCATATAATTCTTANGTATCTGAAGGCGAAT

TTGTATTAGTTTTTCTTCGAAAACAATCTTCTTATTTACGATTAACATCTTCGGGAGCTTTTCTTGAGCG

AACACATTTCTATGGAAAAATAGAACATCTTATAGTAGGGCGCCGAAATTATTTTCAAAAGACCCTAGGG

TTCTTCAGGGATCCCTTCAGGCATTANGTTCGATATCAAGGAAAAGCAATTCGGGTTTCAAAGGGGACTC

ATCTTCTGATGAAAAAAGGGAAAGGTCCCCTTGTCAATTTCGGGCAATATTATTTTCACTTTGGGTCTCA

ACCG

>Pholidostachys_pulchra_AM114651.1

CCTGCCTCTGGTTCAAGTAAAAATGAAAATGGAAGACTTACAAGGATATTTAGAAAAAGATAGATCTCGG

CGCCAACACTTTCTATATCCGCTTCTCTTTAAGGAGTATATTTACACATTTGCTCATGATCGTGGTTTAA

ATGGTTCGATTTTTTACGAATCCACGGAAATTTTTGGTTATGACAATAAATCTAGTTCAGTACTTGTGAA

ACGTTCAATTATTCGAATGTATCAACAGAATTATTTGATTTATTCGGTTAACGATTCTAACCAAAATCGA

TTCGTTGGGCACAATTTTTATTTTCATTTTTATTCTCAGATGATATTGGAAGGTTTTGCAGTCATTGTGG

AAATTCCATTCTTGCTGCGATTAGTATCTTCCCTCGAAGAAAAAAAAATACCAAAATCTCAGAATTTGAA

TTTACGATCTATTCATTCAATATTTCCCTTTTTGGAGGACAAATTATCGCATTTAAATTATGTGTCAGAT

ATACTAATACCTTATCCCATCCATCTGAAAATCTTGGTTCAAATCCTTCAATGCTGGATCCAAGATGTTC

CTTCTTTACATTTATTGCGATTCTTTCTTCACGAATATCATAATTGGAATAGTCTTATTACTCCGAATAA

TTCTATTTTCTTTTTTTCAAAAGAAAATAAAAGACTATTTTGGTTCCCATATAATTCTTATGTATCTGAA

TGCGAATTTGTATTAGTTTTTCTTCGTAAACAATCTTCTTATTTACGATTAACATCTTCTGGAGCTTTTC

TTGAGCGAACACATTTCTATGGAAAAATAGAACGTCTTATAGTAGTGCGCCATAATTATTTTCAGAAGAC

CCTATGGTTCTTCAAGGATCCCTTCATGCATTATGTTCGATATCAAGGAAAAGCAATTCTGGTTTCAAAG

GGGACTCATCTTCTGATGAAGAAATGGAAATGTCACCTTGTCAATTTCTGGCAATATTATTTTCACTTTT

GGTCTCAACCGTACAGGATCCATATAAACCAATTATCAAGCTGTTCTTTCTATTTTCTAGGTTATCTTTC

AAGTGTACTAATAAATCCTTCGGCGGTAAGGAATCAAATGCTAGAGAATTCATTTCTAATAGATACTGTT

ATTAAAAAATTCGATACCAGAGTCCCAGTTATTCCTCTTATTGGATCATTGTCTAAAGCTAAATTTTGTA

CCGTATCGGGGCATCCTATTAGTAAGCCGATCTGGACCGATTTATCAGATTGCGATATTATTGATCGATT

TGGTCGGATATGTAGAAATCTTTCTCATTATCACAGTGGATCCTCAAAAAAACAGAGTTTGTATCGAATA

AAGTATATACTTCGATTTTCGTGTGCTAGAACTTTGGCTCGTAAACATAAAAGTATGGTACGCGCTTTTT

TGCAAAGATTAGGTTCGGGATTATTAGAAGAATTCTTTACGGAAGAAGAACAAGTTGTTTCTTTGATCTT

CCCAAAAACAACTTCTTTTTCTTTACATGAATCACATATAGAACGTATTTGGTATTTGGATATTATCCGT

ATCAATGACCTGGTGAATTATTCATAATAGGTTTGGTGACGTGATGAGACTTATGAATAGAATAGAAATG

ATCTATAAATGATCAAGAGAGAAAAAAATTCATGAATTTCCATTCTGAAATGCTCATTGCAGTAGTGTAG

TGGTTGAATCAACTGAGTAGTCAAAATTATTATACTTTCTTCTCGGGACCCAAGTTTTATATTATACATA

GGAAAAGTC

>Phytelephas_tumacana_EF128240.1

AACCCAAAAAATGAAATGGGTCCTGCCTCTGGTTCAAGTAGAAATGTAAATGGAAGAATTACAAGGATAT

TTAGAAAAAGATAGATCTCGGCAACAACACTTTCTATATCCGCTTCTCTTTAAGGAATATATTTACACAT

TTGCTCATGATCGTGGTTTAAATGGTTCGATTTTTTACGAATCCACGGAAATTTTTGGTTATGACAATAA

ATCTAGTTCAGTACTTGTGAAACGTTCAATTATTCGAATGTATCAACAGAATTATTTGATTTATTCGGTT

AATGATTCTAACCAAAATCGATTCGTTGGGCACAACAATTATTTTTATTTTCATTTTTATTCTCAGATGA

TATTGGAAGGTTTTGCAGTCATTGTGGAAATTCCATTCTTGCTGCGATTAGTATCTTCCCTCGAAGAAAA

AAAAATACCAAAATCTCAGAATTTGAATTTACGATCTATTCATTCAATATTTCCCTTTTTGGAGGACAAA

TTATCGCATTTAAATTATGTGTCAGATATACTAATACCTTATCCCATCCATCTGAAAATCTTGGTTCAAA

TCCTTCAATGCTGGATCCAAGATGTTCCTTCTTTACATTTATTGCGATTCTTTCTTCACGAATATCATAA

TTGGAATAGTCTTATTACTCCGAATAATTCTATTTTTTTTTTTCAAAATAAAAGACTATTTCGGTTCCCA

TATAATTCTTATGTATCTGAATGTGAATTTGTATTAGTTTTTCTTCGTAAACAATCTTCTTATTTACGAT

TAACATCTTCTGGAGCTTTTCTTGAGCGAACACATTTCTATGGAAAAATAGAACATCTTATAGTAGTGCG

CCGTAATTATTTTCAGAAGACCCTATGGTTCTTCAAGGATCCCTTCATGCATTATGTTCGATATCAAGGA

AAAGCAATTCTGGTTTCAAAGGGGACTCATCTTCTGATGAAGAAATGGAAATGTCACCTTGTCAATTTCT

GGCAATATTATTTTCACTTTTGGTCTCAACCGTACAGGATCCATATAAACCAATTATCAAACTGTTCTTT

CTATTTTCTAGGTTATCTTTCAAGTGTACTAATAAATCCTTCGGCGGTAAGGAATCAAATGCTAGAGAAT

TCATTTCTAATAGATACTGTTATTAAAAAATTCGATACCAGAGTCCCAGTTATTCCTCTTATTGGATCAT

TATCTAAAGCTAAATTTTGTACCGTATCGGGGCATCCTATTAGTAAGCCGATCTGGACCAATTTATCAGA

TTGCGATATTATTGATCGATTTGGTCGGATATGTAGAAATCTTTCTCATTATCACAGTGGATCCTCAAAA

AAACAGAGTTTGTATCGAATAAAGTATATACTTCGATTTTCGTGTGCTAGAACTTTGGCTCGTAAACATA

AAAGTATGGTACGCGCTTTTTTGCAAAGATTAGGTTCGGGATTATTAGAAGAATTCTTTACGGAAGAAGA

ACAAGTTGTTTCTTTGATCTTCCCAAAAACAACTTCTTTTTCTTTACATGAATCACATATAGAACGTATT

TGGTATTTGGATATTATCCGTATCAATGACCTGGTTAATTATTCATAATGGGTTTGGTGACGTGATGAGA

CTTATGAATAGAATAGAAATGATCTATAAATGATCAAGAGAGAAAAAAATTCATGAATTTTCATTCTGAA

ATGCTCATTGCAGTAGTGTAGTGGTTGAATCAACTGAGTAGCCAAAATTATTATACTTTC

>Phytelephas_tenuicaulis_EF128238.1

AACCCAAAAAATGAAATGGGTCCTGCCTCTGGTTCAAGTAGAAATGTAAATGGAAGAATTACAAGGATAT

TTAGAAAAAGATAGATCTCGGCAACAACACTTTCTATATCCGCTTCTCTTTAAGGAATATATTTACACAT

TTGCTCATGATCGTGGTTTAAATGGTTCGATTTTTTACGAATCCACGGAAATTTTTGGTTATGACAATAA

ATCTAGTTCAGTACTTGTGAAACGTTCAATTATTCGAATGTATCAACAGAATTATTTGATTTATTCGGTT

AATGATTCTAACCAAAATCGATTCGTTGGGCACAACAATTATTTTTATTTTCATTTTTATTCTCAGATGA

TATTGGAAGGTTTTGCAGTCATTGTGGAAATTCCATTCTTGCTGCGATTAGTATCTTCCCTCGAAGAAAA

AAAAATACCAAAATCTCAGAATTTGAATTTACGATCTATTCATTCAATATTTCCCTTTTTGGAGGACAAA

TTATCGCATTTAAATTATGTGTCAGATATACTAATACCTTATCCCATCCATCTGAAAATCTTGGTTCAAA

TCCTTCAATGCTGGATCCAAGATGTTCCTTCTTTACATTTATTGCGATTCTTTCTTCACGAATATCATAA

TTGGAATAGTCTTATTACTCCGAATAATTCTATTTTTTTTTTTCAAAATAAAAGACTATTTCGGTTCCCA

TATAATTCTTATGTATCTGAATGTGAATTTGTATTAGTTTTTCTTCGTAAACAATCTTCTTATTTACGAT

TAACATCTTCTGGAGCTTTTCTTGAGCGAACACATTTCTATGGAAAAATAGAACATCTTATAGTAGTGCG

CCGTAATTATTTTCAGAAGACCCTATGGTTCTTCAAGGATCCCTTCATGCATTATGTTCGATATCAAGGA

AAAGCAATTCTGGTTTCAAAGGGGACTCATCTTCTGATGAAGAAATGGAAATGTCACCTTGTCAATTTCT

GGCAATATTATTTTCACTTTTGGTCTCAACCGTACAGGATCCATATAAACCAATTATCAAACTGTTCTTT

CTATTTTCTAGGTTATCTTTCAAGTGTACTAATAAATCCTTCGGCGGTAAGGAATCAAATGCTAGAGAAT

TCATTTCTAATAGATACTGTTATTAAAAAATTCGATACCAGAGTCCCAGTTATTCCTCTTATTGGATCAT

TATCTAAAGCTAAATTTTGTACCGTATCGGGGCATCCTATTAGTAAGCCGATCTGGACCAATTTATCAGA

TTGCGATATTATTGATCGATTTGGTCGGATATGTAGAAATCTTTCTCATTATCACAGTGGATCCTCAAAA

AAACAGAGTTTGTATCGAATAAAGTATATACTTCGATTTTCGTGTGCTAGAACTTTGGCTCGTAAACATA

AAAGTATGGTACGCGCTTTTTTGCAAAGATTAGGTTCGGGATTATTAGAAGAATTCTTTACGGAAGAAGA

ACAAGTTGTTTCTTTGATCTTCCCAAAAACAACTTCTTTTTCTTTACATGAATCACATATAGAACGTATT

TGGTATTTGGATATTATCCGTATCAATGACCTGGTTAATTATTCATAATGGGTTTGGTGACGTGATGAGA

CTTATGAATAGAATAGAAATGATCTATAAATGATCAAGAGAGAAAAAAATTCATGAATTTTCATTCTGAA

ATGCTCATTGCAGTAGTGTAGTGGTTGAATCAACTGAGTAGCCAAAATTATTATACTTTC

>Phytelephas_schottii_EF128236.1

AACCCAAAAAATGAAATGGGTCCTGCCTCTGGTTCAAGTAGAAATGTAAATGGAAGAATTACAAGGATAT

TTAGAAAAAGATAGATCTCGGCAACAACACTTTCTATATCCGCTTCTCTTTAAGGAATATATTTACACAT

TTGCTCATGATCGTGGTTTAAATGGTTCGATTTTTTACGAATCCACGGAAATTTTTGGTTATGACAATAA

ATCTAGTTCAGTACTTGTGAAACGTTCAATTATTCGAATGTATCAACAGAATTATTTGATTTATTCGGTT

AATGATTCTAACCAAAATCGATTCGTTGGGCACAACAATTATTTTTATTTTCATTTTTATTCTCAGATGA

TATTGGAAGGTTTTGCAGTCATTGTGGAAATTCCATTCTTGCTGCGATTAGTATCTTCCCACGAAGAAAA

AAAAATACCAAAATCTCAGAATTTGAATTTACGATCTATTCATTCAATATTTCCCTTTTTGGAGGACAAA

TTATCGCATTTAAATTATGTGTCAGATATACTAATACCTTATCCCATCCATCTGAAAATCTTGGTTCAAA

TCCTTCAATGCTGGATCCAAGATGTTCCTTCTTTACATTTATTGCGATTCTTTCTTCACGAATATCATAA

TTGGAATAGTCTTATTACTCCGAATAATTCTATTTTTTTTTTTCAAAATAAAAGACTATTTCGGTTCCCA

TATAATTCTTATGTATCTGAATGTGAATTTGTATTAGTTTTTCTTCGTAAACAATCTTCTTATTTACGAT

TAACATCTTCTGGAGCTTTTCTTGAGCGAACACATTTCTATGGAAAAATAGAACATCTTATAGTAGTGCG

CCGTAATTATTTTCAGAAGACCCTATGGTTCTTCAAGGATCCCTTCATGCATTATGTTCGATATCAAGGA

AAAGCAATTCTGGTTTCAAAGGGGACTCATCTTCTGATGAAGAAATGGAAATGTCACCTTGTCAATTTCT

GGCAATATTATTTTCACTTTTGGTCTCAACCGTACAGGATCCATATAAACCAATTATCAAACTGTTCTTT

CTATTTTCTAGGTTATCTTTCAAGTGTACTAATAAATCCTTCGGCGGTAAGGAATCAAATGCTAGAGAAT

TCATTTCTAATAGATACTGTTATTAAAAAATTCGATACCAGAGTCCCAGTTATTCCTCTTATTGGATCAT

TATCTAAAGCTAAATTTTGTACCGTATCGGGGCATCCTATTAGTAAGCCGATCTGGACCAATTTATCAGA

TTGCGATATTATTGATCGATTTGGTCGGATATGTAGAAATCTTTCTCATTATCACAGTGGATCCTCAAAA

AAACAGAGTTTGTATCGAATAAAGTATATACTTCGATTTTCGTGNGCTAGAACTTGGGCTCGTAAACATA

AAAGTATGGTACGCGCTTTTTTGCAAAGATTAGGTTCGGGATTATTAGAAGAATTCTTTACGGAAGAAGA

ACAAGTTGTTTCTTTGATCTTCCCAAAAACAACTTCTTTTTCTTTACATGAATCACATATAGAACGTATT

TGGTATTTGGATATTATCCGTATCAATGACCTGGTTAATTATTCATAATGGGTTTGGTGACGTGATGAGA

CTTATGAATAGAATAGAAATGATCTATAAATGATCAAGAGAGAAAAAAATTCATGAATTTTCATTCTGAA

ATGCTCATTGCAGTAGTGTAGTGGTTGAATCAACTGAGTAGCCAAAATTATTATACTTTC

>Phytelephas_macrocarpa_EF128234.1

AACCCAAAAAATGAAATGGGTCCTGCCTCTGGTTCAAGTAGAAATGTAAATGGAAGAATTACAAGGATAT

TTAGAAAAAGATAGATCTCGGCAACAACACTTTCTATATCCGCTTCTCTTTAAGGAATATATTTACACAT

TTGCTCATGATCGTGGTTTAAATGGTTCGATTTTTTACGAATCCACGGAAATTTTTGGTTATGACAATAA

ATCTAGTTCAGTACTTGTGAAACGTTCAATTATTCGAATGTATCAACAGAATTATTTGATTTATTCGGTT

AATGATTCTAACCAAAATCGATTCGTTGGGCACAACAATTATTTTTATTTTCATTTTTATTCTCAGATGA

TATTGGAAGGTTTTGCAGTCATTGTGGAAATTCCATTCTTGCTGCGATTAGTATCTTCCCACGAAGAAAA

AAAAATACCAAAATCTCAGAATTTGAATTTACGATCTATTCATTCAATATTTCCCTTTTTGGAGGACAAA

TTATCGCATTTAAATTATGTGTCAGATATACTAATACCTTATCCCATCCATCTGAAAATCTTGGTTCAAA

TCCTTCAATGCTGGATCCAAGATGTTCCTTCTTTACATTTATTGCGATTCTTTCTTCACGAATATCATAA

TTGGAATAGTCTTATTACTCCGAATAATTCTATTTTTTTTTTTCAAAATAAAAGACTATTTCGGTTCCCA

TATAATTCTTATGTATCTGAATGTGAATTTGTATTAGTTTTTCTTCGTAAACAATCTTCTTATTTACGAT

TAACATCTTCTGGAGCTTTTCTTGAGCGAACACATTTCTATGGAAAAATAGAACATCTTATAGTAGTGCG

CCGTAATTATTTTCAGAAGACCCTATGGTTCTTCAAGGATCCCTTCATGCATTATGTTCGATATCAAGGA

AAAGCAATTCTGGTTTCAAAGGGGACTCATCTTCTGATGAAGAAATGGAAATGTCACCTTGTCAATTTCT

GGCAATATTATTTTCACTTTTGGTCTCAACCGTACAGGATCCATATAAACCAATTATCAAACTGTTCTTT

CTATTTTCTAGGTTATCTTTCAAGTGTACTAATAAATCCTTCGGCGGTAAGGAATCAAATGCTAGAGAAT

TCATTTCTAATAGATACTGTTATTAAAAAATTCGATACCAGAGTCCCAGTTATTCCTCTTATTGGATCAT

TATCTAAAGCTAAATTTTGTACCGTATCGGGGCATCCTATTAGTAAGCCGATCTGGACCAATTTATCAGA

TTGCGATATTATTGATCGATTTGGTCGGATATGTAGAAATCTTTCTCATTATCACAGTGGATCCTCAAAA

AAACAGAGTTTGTATCGAATAAAGTATATACTTCGATTTGGCTCGTAAACATAAAAGTATGGTACGCGCT

TTTTTGCAAAGATTAGGTTCGGGATTATTAGAAGAATTCTTTACGGAAGAAGAACAAGTTGTTTCTTTGA

TCTTCCCAAAAACAACTTCTTTTTCTTTACATGAATCACATATAGAACGTATTTGGTATTTGGATATTAT

CCGTATCAATGACCTGGTTAATTATTCATAATGGGTTTGGTGACGTGATGAGACTTATGAATAGAATAGA

AATGATCTATAAATGATCAAGAGAGAAAAAAATTCATGAATTTTCATTCTGAAATGCTCATTGCAGTAGT

GTAGTGGTTGAATCAACTGAGTAGCCAAAATTATTATACTTTC

>Phytelephas_tenuicaulis_EF128239.1

AACCCAAAAAATGAAATGGGTCCTGCCTCTGGTTCAAGTAGAAATGTAAATGGAAGAATTACAAGGATAT

TTAGAAAAAGATAGATCTCGGCAACAACACTTTCTATATCCGCTTCTCTTTAAGGAATATATTTACACAT

TTGCTCATGATCGTGGTTTAAATGGTTCGATTTTTTACGAATCCACGGAAATTTTTGGTTATGACAATAA

ATCTAGTTCAGTACTTGTGAAACGTTCAATTATTCGAATGTATCAACAGAATTATTTGATTTATTCGGTT

AATGATTCTAACCAAAATCGATTCGTTGGGCACAACAATTATTTTTATTTTCATTTTTATTCTCAGATGA

TATTGGAAGGTTTTGCAGTCATTGTGGAAATTCCATTCTTGCTGCGATTAGTATCTTCCCTCGAAGAAAA

AAAAATACCAAAATCTCAGAATTTGAATTTACGATCTATTCATTCAATATTTCCCTTTTTGGAGGACAAA

TTATCGCATTTAAATTATGTGTCAGATATACTAATACCTTATCCCATCCATCTGAAAATCTTGGTTCAAA

TCCTTCAATGCTGGATCCAAGATGTTCCTTCTTTACATTTATTGCGATTCTTTCTTCACGAATATCATAA

TTGGAATAGTCTTATTACTCCGAATAATTCTATTTTTTTTTTTCAAAATAAAAGACTATTTCGGTTCCCA

TATAATTCTTATGTATCTGAATGTGAATTTGTATTAGTTTTTCTTCGTAAACAATCTTCTTATTTACGAT

TAACATCTTCTGGAGCTTTTCTTGAGCGAACACATTTCTATGGAAAAATAGAACATCTTATAGTAGTGCG

CCGTAATTATTTTCAGAAGACCCTATGGTTCTTCAAGGATCCCTTCATGCATTATGTTCGATATCAAGGA

AAAGCAATTCTGGTTTCAAAGGGGACTCATCTTCTGATGAAGAAATGGAAATGTCACCTTGTCAATTTCT

GGCAATATTATTTTCACTTTTGGTCTCAACCGTACAGGATCCATATAAACCAATTATCAAACTGTTCTTT

CTATTTTCTAGGTTATCTTTCAAGTGTACTAATAAATCCTTCGGCGGTAAGGAATCAAATGCTAGAGAAT

TCATTTCTAATAGATACTGTTATTAAAAAATTCGATACCAGAGTCCCAGTTATTCCTCTTATTGGATCAT

TATCTAAAGCTAAATTTTGTACCGTATCGGGGCATCCTATTAGTAAGCCGATCTGGACCAATTTATCAGA

TTGCGATATTATTGATCGATTTGGTCGGATATGTAGAAATCTTTCTCATTATCACAGTGGATCCTCAAAA

AAACAGAGTTTGTATCGAATAAAGTATATACTTCGATTTTCGTGTGCTAGAACTTTGGCTCGTAAACATA

AAAGTATGGTACGCGCTTTTTTGCAAAGATTAGGTTCGGGATTATTAGAAGAATTCTTTACGGAAGAAGA

ACAAGTTGTTTCTTTGATCTTCCCAAAAACAACTTCTTTTTCTTTACATGAATCACATATAGAACGTATT

TGGTATTTGGATATTATCCGTATCAATGACCTGGTTAATTATTCATAATGGGTTTGGTGACGTGATGAGA

CTTATGAATAGAATAGAAATGATCTATAAATGATCAAGAGAGAAAAAAATTCATGAATTTTCATTCTGAA

ATGCTCATTGCAGTAGTGTAGTGGTTGAATCAACTGAGTAGCCAAAATTATTATACTTTC

>Phytelephas_seemannii_EF128237.1

AACCCAAAAAATGAAATGGGTCCTGCCTCTGGTTCAAGTAGAAATGTAAATGGAAGAATTACAAGGATAT

TTAGAAAAAGATAGATCTCGGCAACAACACTTTCTATATCCGCTTCTCTTTAAGGAATATATTTACACAT

TTGCTCATGATCGTGGTTTAAATGGTTCGATTTTTTACGAATCCACGGAAATTTTTGGTTATGACAATAA

ATCTAGTTCAGTACTTGTGAAACGTTCAATTATTCGAATGTATCAACAGAATTATTTGATTTATTCGGTT

AATGATTCTAACCAAAATCGATTCGTTGGGCACAACAATTATTTTTATTTTCATTTTTATTCTCAGATGA

TATTGGAAGGTTTTGCAGTCATTGTGGAAATTCCATTCTTGCTGCGATTAGTATCTTCCCTCGAAGAAAA

AAAAATACCAAAATCTCAGAATTTGAATTTACGATCTATTCATTCAATATTTCCCTTTTTGGAGGACAAA

TTATCGCATTTAAATTATGTGTCAGATATACTAATACCTTATCCCATCCATCTGAAAATCTTGGTTCAAA

TCCTTCAATGCTGGATCCAAGATGTTCCTTCTTTACATTTATTGCGATTCTTTCTTCACGAATATCATAA

TTGGAATAGTCTTATTACTCCGAATAATTCTATTTTTTTTTTTCAAAATAAAAGACTATTTCGGTTCCCA

TATAATTCTTATGTATCTGAATGTGAATTTGTATTAGTTTTTCTTCGTAAACAATCTTCTTATTTACGAT

TAACATCTTCTGGAGCTTTTCTTGAGCGAACACATTTCTATGGAAAAATAGAACATCTTATAGTAGTGCG

CCGTAATTATTTTCAGAAGACCCTATGGTTCTTCAAGGATCCCTTCATGCATTATGTTCGATATCAAGGA

AAAGCAATTCTGGTTTCAAAGGGGACTCATCTTCTGATGAAGAAATGGAAATGTCACCTTGTCAATTTCT

GGCAATATTATTTTCACTTTTGGTCTCAACCGTACAGGATCCATATAAACCAATTATCAAACTGTTCTTT

CTATTTTCTAGGTTATCTTTCAAGTGTACTAATAAATCCTTCGGCGGTAAGGAATCAAATGCTAGAGAAT

TCATTTCTAATAGATACTGTTATTAAAAAATTCGATACCAGAGTCCCAGTTATTCCTCTTATTGGATCAT

TATCTAAAGCTAAATTTTGTACCGTATCGGGGCATCCTATTAGTAAGCCGATCTGGACCAATTTATCAGA

TTGCGATATTATTGATCGATTTGGTCGGATATGTAGAAATCTTTCTCATTATCACAGTGGATCCTCAAAA

AAACAGAGTTTGTATCGAATAAAGTATATACTTCGATTTTCGTGTGCTAGAACTTTGGCTCGTAAACATA

AAAGTATGGTACGCGCTTTTTTGCAAAGATTAGGTTCGGGATTATTAGAAGAATTCTTTACGGAAGAAGA

ACAAGTTGTTTCTTTGATCTTCCCAAAAACAACTTCTTTTTCTTTACATGAATCACATATAGAACGTATT

TGGTATTTGGATATTATCCGTATCAATGACCTGGTTAATTATTCATAATGGGTTTGGTGACGTGATGAGA

CTTATGAATAGAATAGAAATGATCTATAAATGATCAAGAGAGAAAAAAATTCATGAATTTTCATTCTGAA

ATGCTCATTGCAGTAGTGTAGTGGTTGAATCAACTGAGTAGCCAAAATTATTATACTTTC

>Phytelephas_schottii_EF128235.1

AACCCAAANNATGNAATGGGTCCNGCCTCTGGYTCNAGTAGAAATGTAAATGGAAGAANNACAAGGATAT

TTAGRAAAAGATAGATCTCGGCRACAACACTTTCTATATCCGCTTCTCTTTAAGGAATATATTTACACAT

TTGCTCATGATCGTGGTTTAAATGGTTCGATTTTTTACGAATCCACGGAAATTTTTGGTTATGACAATAA

ATCTAGTTCAGTACTTGTGAAACGTTCAATTATTCGAATGTATCAACAGAATTATTTGATTTATTCGGTT

AATGATTCTAACCAAAATCGATTCGTTGGGCACAACAATTATTTTTATTTTCATTTTTATTCTCAGATGA

TATTGGAAGGTTTTGCAGTCATTGTGGAAATTCCATTCTTGCTGCGATTAGTATCTTCCCACGAAGAAAA

AAAAATACCAAAATCTCAGAATTTGAATTTACGATCTATTCATTCAATATTTCCCTTTTTGGAGGACAAA

TTATCGCATTTAAATTATGTGTCAGATATACTAATACCTTATCCCATCCATCTGAAAATCTTGGTTCAAA

TCCTTCAANNCTGGATCNAANATGTTCCNTCTTNACATTTATNNCNATTCTTTCTTCACGAATATCATAA

TTGGAATAGTCTTATTACNCCGAATAATTCTATTTTTTTTTTTCAAAATAAAAGANTATTTCGGTTCCCA

TATAATTCTTATGTATCTGAATGTGAATTTGTATTAGTTTTTCTTCGTAAACAATCTTCTTATTTACGAT

TAACATCTTNTGGAGCTTTTCTTGAGCGAACACATTTCTATGGAAAAATAGAACATCTTATAGTAGTGCN

CCGTAATTATTTTCAGAAGACCCTATGGTTCTTCAAGGATCCCTTCATGCATTATGTTCGATATCAAGGA

AAAGCAATTCTGGTTTCAAAGGGACTCATCTTCTGATGAAGAAATGGAAATGTCACCTTGTCAATTTCTG

GCAATATTATTTTCACTTTTGGTCTCAACCGTACAGGATCCATATAAACCAATTATCAAACTGTTCTTTC

TATTTTCTAGGTTATCTTTCAAGTGTACTAATAAATCCTTCGGCGGTAAGGAATCAAATGCTAGAGAATT

CATTTCTAATAGATACTGTTATTAAAAAATTCGATACCAGAGTCCCAGTTATTCCTCTTATTGGATCATT

ATCTAAAGCTAAATTTTGTACCGTATCGGGGCATCCTATTAGTAAGCCGATCTGGACCAATTTATCAGAT

TGCGATATTATTGATCGATTTGGTCGGATATGTAGAAATCTTTCTCATTATCACAGTGGATCCTCAAAAA

AACAGAGTTTGTATCGAATAAAGTATATACTTCGATTTTNGTNNNCTAGAACTNNGGGTCGTAAACATNA

AAGTATGGTACGCGCTTTTTTGCAAAGATTAGGTTCGGGATTATTAGAAGAATTCTTTACGGAAGAAGAA

CAAGTTGTTTCTTTGATCTTCCCAAAAACAACTTCTTTTTCTTTACATGAATCACATATAGAACGTATTT

GGTATTTGGATATTATCCGTATCAATGACCTGGTTAATTATTCATAATGGGTTTGGTGACGTGATGAGAC

TTATGAATAGAATAGAAATGATCTATAAATGATCAAGAGAGAAAAAAATTCATGAATTTTCATTCTGAAA

TGCTCATTGCAGTAGTGTAGTGGTTGAATCAACTGAGTAGCCAAAATTATTATACTTTC

>Phytelephas_macrocarpa_AM114614.1

GACCATATTGCACTATGTATCATTTGATAACCCAAAAAATGAAATGGGTCCTGCCTCTGGTTCAAGTAGA

AATGTAAATGGAAGAATTACAAGGATATTTAGAAAAAGATAGATCTCGGCAACAACACTTTCTATATCCG

CTTCTCTTTAAGGAATATATTTACACATTTGCTCATGATCGTGGTTTAAATGGTTCGATTTTTTACGAAT

CCACGGAAATTTTTGGTTATGACAATAAATCTAGTTCAGTACTTGTGAAACGTTCAATTATTCGAATGTA

TCAACAGAATTATTTGATTTATTCGGTTAATGATTCTAACCAAAATCGATTCGTTGGGCACAACAATTAT

TTTTATTTTCATTTTTATTCTCAGATGATATTGGAAGGTTTTGCAGTCATTGTGGAAATTCCATTCTTGC

TGCGATTAGTATCTTCCCTCGAAGAAAAAAAAATACCAAAATCTCAGAATTTGAATTTACGATCTATTCA

TTCAATATTTCCCTTTTTGGAGGACAAATTATCGCATTTAAATTATGTGTCAGATATACTAATACCTTAT

CCCATCCATCTGAAAATCTTGGTTCAAATCCTTCAATGCTGGATCCAAGATGTTCCTTCTTTACATTTAT

TGCGATTCTTTCTTCACGAATATCATAATTGGAATAGTCTTATTACTCCGAATAATTCTATTTTTTTTTT

TCAAAATAAAAGACTATTTCGGTTCCCATATAATTCTTATGTATCTGAATGTGAATTTGTATTAGTTTTT

CTTCGTAAACAATCTTCTTATTTACGATTAACATCTTCTGGAGCTTTTCTTGAGCGAACACATTTCTATG

GAAAAATAGAACATCTTATAGTAGTGCGCCGTAATTATTTTCAGAAGACCCTATGGTTCTTCAAGGATCC

CTTCATGCATTATGTTCGATATCAAGGAAAAGCAATTCTGGTTTCAAAGGGGACTCATCTTCTGATGAAG

AAATGGAAATGTCACCTTGTCAATTTCTGGCAATATTATTTTCACTTTTGGTCTCAACCGTACAGGATCC

ATATAAACCAATTATCAAACTGTTCTTTCTATTTTCTAGGTTATCTTTCAAGTGTACTAATAAATCCTTC

GGCGGTAAGGAATCAAATGCTAGAGAATTCATTTCTAATAGATACTGTTATTAAAAAATTCGATACCAGA

GTCCCAGTTATTCCTCTTATTGGATCATTATCTAAAGCTAAATTTTGTACCGTATCGGGGCATCCTATTA

GTAAGCCGATCTGGACCAATTTATCAGATTGCGATATTATTGATCGATTTGGTCGGATATGTAGAAATCT

TTCTCATTATCACAGTGGATCCTCAAAAAAACAGAGTTTGTATCGAATAAAGTATATACTTCGATTTTCG

TGTGCTAGAACTTTGGCTCGTAAACATAAAAGTATGGTACGCGCTTTTTTGCAAAGATTAGGTTCGGGAT

TATTAGAAGAATTCTTTACGGAAGAAGAACAAGTTGTTTCTTTGATCTTCCCAAAAACAACTTCTTTTTC

TTTACATGAATCACATATAGAACGTATTTGGTATTTGGATATTATCCGTATCAATGACCTGGTTAATTAT

TCATAATGGGTTTGGTGACGTGATGAGACTTATGAATAGAATAGAAATGATCTATAAATGATCAAGAGAG

AAAAAAATTCATGAATTTTCATTCTGAAATGCTCATTGCAGTAGTGTAGTGGTTGAATCAACTGAGTAGC

CAAAATTATTATACTTTCTTCTCGGGACCCAAGTTTTATATTATACATAGGTAAAGTC

>Phytelephas_aequatorialis_AM114613.1

GACCATATTGCACTATGTATCATTTGATAACCCAAAAAATGAAATGGGTCCTGCCTCTGGTTCAAGTAGA

AATGTAAATGGAAGAATTACAAGGATATTTAGAAAAAGATAGATCTCGGCAACAACACTTTCTATATCCG

CTTCTCTTTAAGGAATATATTTACACATTTGCTCATGATCGTGGTTTAAATGGTTCGATTTTTTACGAAT

CCACGGAAATTTTTGGTTATGACAATAAATCTAGTTCAGTACTTGTGAAACGTTCAATTATTCGAATGTA

TCAACAGAATTATTTGATTTATTCGGTTAATGATTCTAACCAAAATCGATTCGTTGGGCACAACAATTAT

TTTTATTTTCATTTTTATTCTCAGATGATATTGGAAGGTTTTGCAGTCATTGTGGAAATTCCATTCTTGC

TGCGATTAGTATCTTCCCTCGAAGAAAAAAAAATACCAAAATCTCAGAATTTGAATTTACGATCTATTCA

TTCAATATTTCCCTTTTTGGAGGACAAATTATCGCATTTAAATTATGTGTCAGATATACTAATACCTTAT

CCCATCCATCTGAAAATCTTGGTTCAAATCCTTCAATGCTGGATCCAAGATGTTCCTTCTTTACATTTAT

TGCGATTCTTTCTTCACGAATATCATAATTGGAATAGTCTTATTACTCCGAATAATTCTATTTTTTTTTT

TCAAAATAAAAGACTATTTCGGTTCCCATATAATTCTTATGTATCTGAATGTGAATTTGTATTAGTTTTT

CTTCGTAAACAATCTTCTTATTTACGATTAACATCTTCTGGAGCTTTTCTTGAGCGAACACATTTCTATG

GAAAAATAGAACATCTTATAGTAGTGCGCCGTAATTATTTTCAGAAGACCCTATGGTTCTTCAAGGATCC

CTTCATGCATTATGTTCGATATCAAGGAAAAGCAATTCTGGTTTCAAAGGGGACTCATCTTCTGATGAAG

AAATGGAAATGTCACCTTGTCAATTTCTGGCAATATTATTTTCACTTTTGGTCTCAACCGTACAGGATCC

ATATAAACCAATTATCAAACTGTTCTTTCTATTTTCTAGGTTATCTTTCAAGTGTACTAATAAATCCTTC

GGCGGTAAGGAATCAAATGCTAGAGAATTCATTTCTAATAGATACTGTTATTAAAAAATTCGATACCAGA

GTCCCAGTTATTCCTCTTATTGGATCATTATCTAAAGCTAAATTTTGTACCGTATCGGGGCATCCTATTA

GTAAGCCGATCTGGACCAATTTATCAGATTGCGATATTATTGATCGATTTGGTCGGATATGTAGAAATCT

TTCTCATTATCACAGTGGATCCTCAAAAAAACAGAGTTTGTATCGAATAAAGTATATACTTCGATTTTCG

TGTGCTAGAACTTTGGCTCGTAAACATAAAAGTATGGTACGCGCTTTTTTGCAAAGATTAGGTTCGGGAT

TATTAGAAGAATTCTTTACGGAAGAAGAACAAGTTGTTTCTTTGATCTTCCCAAAAACAACTTCTTTTTC

TTTACATGAATCACATATAGAACGTATTTGGTATTTGGATATTATCCGTATCAATGACCTGGTTAATTAT

TCATAATGGGTTTGGTGACGTGATGAGACTTATGAATAGAATAGAAATGATCTATAAATGATCAAGAGAG

AAAAAAATTCATGAATTTTCATTCTGAAATGCTCATTGCAGTAGTGTAGTGGTTGAATCAACTGAGTAGC

CAAAATTATTATACTTTCTTCTCGGGACCCAAGTTTTATATTATACATAGGTAAAGTC

>Prestoea_acuminata_var.montana_HM446733.1

CTGTACGATGTTCCTTCTTTACATTTATTGCGATTCTTTCTTCACGAATATCATAATTGGAATAGTCTTA

TTACTCCGAATASTTYTSSTTTTTTTTTTTCAAAARAAAASAAAARACTATTTCGGTTCCCATATAATTC

TTATGTATCTGAATGCGAATTTGTATTAGTTTTTCTTCGTAAACAATCTTCTTATTTACGATTAACATCT

TCTGGAGCTTTTCTTGAGCGAACACATTTCTATGGAAAAATAGAACATCTTATAGTAGTGCGCCGTAATT

ATTTTCAGAAGACCCTATGGTTCTTCAAGGATCCCTTCATGCATTATGTTCGATATCAAGGAAAAGCAAT

TCTGGTTTCAAAGGGGACTCATCTTCTGATGAAGAAATRGAAATGTCACCTTGTCAATTTCTGGCAATAT

TATTTTCACTTTTGGTCTCAACCGTACAGGATCCATATAAACCAATTATCAAGCTGTTCTTTCTATTTTC

TAGGTTATCTTTCAAGTGTACTAATAAATCCTTCGGCGGTAAGGAATCAAATGCTAGAGAATTCATTTCT

AATAGATACTGTTATTAAAAAATTCGATACCAGAGTCCCAGTTATTCCTCTTATTGGATCATTGTCTAAA

GCTAAATTTTGTACCGTATCGGGGCATCCTATTAGTAAGCCGATCTGGACCAATTTATCAGATTGCGATA

TTATTGATCGATTTGGTCGGATATGTAGAAATCTTTTTCSTTATCACAGTGGATCCTCAAAAAAACAGAG

TTTGTATCGAATAAAGTATATG

>Prestoea_pubens_AM114648.1

GACCATATTGCACTATGTATCATTTGATAACCCAAAAAATGAAATGGGTCCAGCCTCTGGTTCAAGTAGA

AATGTAAATGGAAGAATTACAAGGATATTTAGAAAAAGATAGATCTCGGCAACAACACTTTCTATATCCG

CTTCTCTTTAAGGAGTATATTTACACATTTGCTCATGATCGTGGTTTAAATGGTTCGATTTTTTACGAAT

CCACGGAAATTTTTGGTTATGACAATAAATCTAGTTCAGTACTTGTGAAACGTTCAATTATTCGAATGTA

TCAACAGAATTATTTGATTTATTCGGTTAACGATTCTAACCAAAATCGATTCGTTGGGCACAACAATTAT

TTTTATTTTCATTTTTATTCTCAGATGATATTGGAAGGTTTTGCAGTCATTGTGGAAATTCCATTCTTGC

TGCGATTAGTATCTTCCCTCGAAGAAAAAAAAATACCAAAATCTCAGAATTTGAATTTACGATCTATTCA

TTCAATATTTCCCTTTTTGGAGGACAAATTATCGCATTTAAATTATGTGTCAGATATACTAATACCTTAT

CCCATCCATCTGAAAATCTTGGTTCAAGTCCTTCAATGCTGGATCCAAGATGTTCCTTCTTTACATTTAT

TGCGATTCTTTCTTCACGAATATCATAATTGGAATAGTCTTATTACTCCGAATAATTCTATTTTTTTTTT

TTCAAAAGAAAATAAAAGACTATTTCGGTTCCCATATAATTCTTATGTATCTGAATGCGAATTTGTATTA

GTTTTTCTTCGTAAACAATCTTCTTATTTACGATTAACATCTTCTGGAGCTTTTCTTGAGCGAACACATT

TCTATGGAAAAATAGAACATCTTATAGTAGTGCGCCGTAATTATTTTCAGAAGACCCTATGGTTCTTCAA

GGATCCCTTCATGCATTATGTTCGATATCAAGGAAAAGCAATTCTGGTTTCAAAGGGGACTCATCTTCTG

ATGAAGAAATGGAAATGTCATTTTGTCAATTTCTGGCAATATTATTTTCACTTTTGGTCTCAACCGCACA

GGATCCATATAAACCAATTATCAAGCTGTTCTTTCTATTTTCTAGGTTATCTTTCAAGTGTACTAATAAA

TCCTTCGGCGGTAAGGAATCAAATGCTAGAGAATTCATTTCTAATAGATACTGTTATTAAAAAATTCGAT

ACCAGAGTCCCAGTTATTCCTCTTATTGGATCATTGTCTAAAGCTAAATTTTGTACCGTATCGGGGCATC

CTATTAGTAAGCCGATCTGGACCAATTTATCAGATTGCGATATTATTGATCGATTTGGTCGGATATGTAG

AAATCTTTTTCATTATCACAGTGGATCCTCAAAAAAACAGAGTTTGTATCGAATAAAGTATATACTTCGA

TTTTCGTGTGCTAGAACTTTGGCTCGTAAACATAAAAGTATGGTACGCACTTTTTTGCAAAGATTAGGTT

CGGGATTATTAGAAGAATTCTTTACGGAAGAAGAACAAGTTGTTTCTTTGATCTTCCCAAAAACAACTTC

TTTTTCTTTACATGAATCACATAAAGAACGTATTTGGTATTTGGATATTATCCGTATCAATGACCTGGTG

AATTATTCATAATGGGTTTGGTGACGTGATGAGACTTATGAATAGAATAGAAATGATCTATAAATGATCA

AGAGAGAAAAAAATTCATGAATTTTCATTCTGAAATGCTCATTGCAGTAGTGTAGTGGTTGAATCAACTG

AGTAGTCAAAATTATTATACTTTCTTCTCGGGACCCAAGTTTTATATTATACATAGGTAAAGTC

>Pseudophoenix_sargentii_var.navassana_EF128230.1

AACCCAAAAAATGAAATGGGTCCTGCCCCTGGTTTAAGTAGAAATGTAAATGGAAGAATTACAAGGATAT

TTAGAAAAAGATAGATCTCGGCAACAACACTTTCTATATCCGCTTCTCTTTAAGGAGTATATTTACACAT

TTGCTCATGATCGTGGTTTAAATGGTTCGATTTTTTACGAATCCACAGAAATTTTTGGTTATGACAATAA

ATCTAGTTCAGTACTTGTGAAACGTTCAATTATTCGAATGTATCAACAGAATTATTTGATTTATTCGGTT

AATGATTCTAACCAAAATCGATTCGTTGGGCACAACAATTATTTTTATTTTCATTTTTATTTTCAGATGA

TATTGGAAGGTTTTGCAGTCATTGTGGAAATTCCATTCTTGCTGCGATTAGTATCTTCCCTCGAAGAAAA

AAAAATACCAAAATCTCAGAATTTGAATTTACGATCTATTCATTCAATATTTCCCTTTTTAGAGGACAAA

TTATCGCATTTAAATTATGTGTCAGATATACTAATACCTTATCCCATCCATCTGAAAATCTTGGTTCAAA

TCCTTCAATGCTGGATCCAAGATGTTCCTTCTTTACATTTATTGCGATTCTTTCTTCACGAATATCATAA

TTGGAATAGTCTTATTACTCCGAATAATTCTATTTTTTTTTTTCAAAATAAAAGACTATTTCGGTTCTCA

TATAATTCTTATGTATCTGAATGCGAATTTGTATTAGTTTTTCTTCGTAAACAATCTTCTTATTTACGAT

TAACATCTTCTGTAGCTTTTCTTGAGCGAACACATTTCTATGGAAAAATAGAACATCTTATAGTAGTGCG

CCGTAATTATTTTCAGAAGACCCTATGGTTCTTCAAGGATCCCTTCATGCATTATGTTCGATATCAAGGA

AAAGCAATTCTGGTTTCAAAGGGGACTCATCTTCTGATGAAGAAATGGAAATGTCACCTTGTCAATTTCT

GGCAATATTATTTTCACTTTTGGTCTCAACCGTACAGGATACATATAAACCAATTATCAAACTGTTCTTT

CTATTTTCTAGGTTATTTTTCAAGTGTACTAATAAATCCTTCGTCGGTAAGGAATCAAATGCTAGAGAAT

TCATTTCTAATAGATACTGTTATTAAAAAATTCGATACCAGAGTCCCAGTTATTCCTCTTATTGGATCAT

TGTATAAAGCTAAATTTTGTACCGTATCGGGGCATCCTATTAGTAAGCCGATCTGGACCCATTTATCAGA

TTGCGATATTATTGATCGATTTGGTCGGATATGTAGAAATCTTTCTCATTATCACAGTGGATCCTCAAAA

AAACAGGGTTTGTATCGAATAAAGTATATACTTCGATTTTCGTGTGCTAGAACTTTGGCTCGTAAACATA

AAAGTACGGTACGCGCTTTTTTGCAAAGATTAGGTTCGGGATTATTAGAAGAATTCTTTACGGAAGAAGA

AGAAGTTGTTTCTTTGATCTTCCAAAAAACAACTTCTTTTTCTTTACATGAATCACATATAGAACGTATT

TGGTATTTGGATATTATCCGTATCAATGACTTGGTGAATTATTCATAATGGGTTTGGTGACGTGATGAGA

CTTATGAATAGAATAGAATAGAAATGATCTATAAATGATCAAGAGAGAAAAAAATTCATGAATTTTCATT

CTGAAATGCTCATTGCAGTAGTGTAGTGGTTGAATCAACTGAGTAGCCAAAATTATTATACTTTC

>Pseudophoenix_ekmanii_EF128228.1

AACCCAAAAAATGAAATGGGTCCTGCCCCTGGTTTAAGTAGAAATGTAAATGGAAGAATTACAAGGATAT

TTAGAAAAAGATAGATCTCGGCAACAACACTTTCTATATCCGCTTCTCTTTAAGGAGTATATTTACACAT

TTGCTCATGATCGTGGTTTAAATGGTTCGATTTTTTACGAATCCACAGAAATTTTTGGTTATGACAATAA

ATCTAGTTCAGTACTTGTGAAACGTTCAATTATTCGAATGTATCAACAGAATTATTTGATTTATTCGGTT

AATGATTCTAACCAAAATCGATTCGTTGGGCACAACAATTATTTTTATTTTCATTTTTATTTTCAGATGA

TATTGGAAGGTTTTGCAGTCATTGTGGAAATTCCATTCTTGCTGCGATTAGTATCTTCCCTCGAAGAAAA

AAAAATACCAAAATCTCAGAATTTGAATTTACGATCTATTCATTCAATATTTCCCTTTTTAGAGGACAAA

TTATCGCATTTAAATTATGTGTCAGATATACTAATACCTTATCCCATCCATCTGAAAATCTTGGTTCAAA

TCCTTCAATGCTGGATCCAAGATGTTCCTTCTTTACATTTATTGCGATTCTTTCTTCACGAATATCATAA

TTGGAATAGTCTTATTACTCCGAATAATTCTATTTTTTTTTTTCAAAATAAAAGACTATTTCGGTTCTCA

TATAATTCTTATGTATCTGAATGCGAATTTGTATTAGTTTTTCTTCGTAAACAATCTTCTTATTTACGAT

TAACATCTTCTGTAGCTTTTCTTGAGCGAACACATTTCTATGGAAAAATAGAACATCTTATAGTAGTGCG

CCGTAATTATTTTCAGAAGACCCTATGGTTCTTCAAGGATCCCTTCATGCATTATGTTCGATATCAAGGA

CAAGCAATTCNGGTTTCAAAGGGGACTCATCTTCTGATGAAGAAATGGAAATGTCACCTTGTCAATTTCT

GGCAATATTATTTTCACTTTTGGTCTCAACCGTACAGGATACATATAAACCAATTATCAAACTGTTCTTT

CTATTTTCTAGGTTATTTTTCAAGTGTACTAATAAATCCTTCGTCGGTAAGGAATCAAATGCTAGAGAAT

TCATTTCTAATAGATACTGTTATTAAAAAATTCGATACCAGAGTCCCAGTTATTCCTCTTATTGGATCAT

TGTATAAAGCTAAATTTTGTACCGTATCGGGGCATCCTATTAGTAAGCCGATCTGGACCCATTTATCAGA

TTGCGATATTATTGATCGATTTGGTCGGATATGTAGAAATCTTTCTCATTATCACAGTGGATCCTCAAAA

AAACAGGGTTTGTATCGAATAAAGTATATACTTCGATTTTCGTGTGCTAGAACTTTGGCTCGTAAACATA

AAAGTACGGTACGCGCTTTTTTGCAAAGATTAGGTTCGGGATTATTAGAAGAATTCTTTACGGAAGAAGA

AGAAGTTGTTTCTTTGATCTTCCAAAAAACAACTTCTTTTTCTTTACATGAATCACATATAGAACGTATT

TGGTATTTGGATATTATCCGTATCAATGACTTGGTGAATTATTCATAATGGGTTTGGTGACGTGATGAGA

CTTATGAATAGAATAGAATAGAAATGATCTATAAATGATCAAGAGAGAAAAAAATTCATGAATTTTCATT

CTGAAATGCTCATTGCAGTAGTGTAGTGGTTGAATCAACTGAGTAGCCAAAATTATTATACTTTC

>Pseudophoenix_sargentii_var.saonae_EF128231.1

AACCCAAAAAATGAAATGGGTCCTGCCCCTGGTTTAAGTAGAAATGTAAATGGAAGAATTACAAGGATAT

TTAGAAAAAGATAGATCTCGGCAACAACACTTTCTATATCCGCTTCTCTTTAAGGAGTATATTTACACAT

TTGCTCATGATCGTGGTTTAAATGGTTCGATTTTTTACGAATCCACAGAAATTTTTGGTTATGACAATAA

ATCTAGTTCAGTACTTGTGAAACGTTCAATTATTCGAATGTATCAACAGAATTATTTGATTTATTCGGTT

AATGATTCTAACCAAAATCGATTCGTTGGGCACAACAATTATTTTTATTTTCATTTTTATTTTCAGATGA

TATTGGAAGGTTTTGCAGTCATTGTGGAAATTCCATTCNNNNNGCGANTAGKATCTTCCCTCGAAGAAAA

AAAAATACCAAAATCTCAGAATTTGAAKKTACGATCTATTCATTCMATATTTCCCTTTTTAGAGGACAAA

TTATCGCATTTAAATTATGTGTCAGATATACTAATACCTTATCCCATCCATCTGAAAATCTTGGTTCMAA

TCCTTCNANGCTGGATCCMAGATGTTCCTTCTTTACATTTATTGCGATTCTTTCTTCACGRATATCATAA

TTGGRATAGTCTTATTACTCCGAATAATTCTATTTTTTTTTTTCAAAATAAAAGACTATTTCGGTTCTCA

TATAATTCTTATGTATCTGAATGCGAATTTGTATTAGTTTTTCTTCGTAAACAATCTTCTTATTTACGAT

TAACATCTTCTGTAGCTTTTCTTGAGCGAACACATTTCTATGGAAAAATAGAACATCTTATAGTAGTGCG

CCGTAATTATTTTCAGAAGACCCTATGGTTCTTCAAGGATCCCTTCATGCATTATGNTCGATATCAAGGA

AAAGCAATTCTGGTTTCAAAGGGGACTCATCTTCTGATGAAGAAATGGAAATGTCACCTTGTCAATTTCT

GGCAATATTATTTTCACTTTTGGTCTCAACCGTACAGGATACATATAAACCAATTATCAAACTGTTCTTT

CTATTTTCTAGGTTATTTTTCAAGTGTACTAATAAATCCTTCGTCGGTAAGGAATCAAATGCTAGAGAAT

TCATTTCTAATAGATACTGTTATTAAAAAATTCGATACCAGAGTCCCAGTTATTCCTCTTATTGGATCAT

TGTATAAAGCTAAATTTTGTACCGTATCGGGGCATCCTATTAGTAAGCCGATCTGGACCCATTTATCAGA

TTGCGATATTATTGATCGATTTGGTCGGATATGTAGAAATCTTTCTCATTATCACAGTGGATCCTCAAAA

AAACAGGGTTTGTATCGAATAAAGTATATACTTCGATTTTCGTGTGCTAGAACTTTGGCTCGTAAACATA

AAAGTACGGTACGCGCTTTTTTGCAAAGATTAGGTTCGGGATTATTAGAAGAATTCTTTACGGAAGAAGA

AGAAGTTGTTTCTTTGATCTTCCAAAAAACAACTTCTTTTTCTTTACATGAATCACATATAGAACGTATT

TGGTATTTGGATATTATCCGTATCAATGACTTGGTGAATTATTCATAATGGGTTTGGTGACGTGATGAGA

CTTATGAATAGAATAGAATAGAAATGATCTATAAATGATCAAGAGAGAAAAAAATTCATGAATTTTCATT

CTGAAATGCTCATTGCAGTAGTGTAGTGGTTGAATCAACTGAGTAGCCAAAATTATTATACTTTC

>Pseudophoenix_lediniana_EF128229.1

AACCCAAAAAATGAAATGGGTCCTGCCCCTGGTTTAAGTAGAAATGTAAATGGAAGAATTACAAGGATAT

TTAGAAAAAGATAGATCTCGGCAACAACACTTTCTATATCCGCTTCTCTTTAAGGAGTATATTTACACAT

TTGCTCATGATCGTGGTTTAAATGGTTCGATTTTTTACGAATCCACAGAAATTTTTGGTTATGACAATAA

ATCTAGTTCAGTACTTGTGAAACGTTCAATTATTCGAATGTATCAACAGAATTATTTGATTTATTCGGTT

AATGATTCTAACCAAAATCGATTCGTTGGGCACAACAATTATTTTTATTTTCATTTTTATTTTCAGATGA

TATTGGAAGGTTTTGCAGTCATTGTGGAAATTCCATTCTTGCTGCGATTAGTATCTTCCCTCGAAGAAAA

AAAAATACCAAAATCTCAGAATTTGAATTTACGATCTATTCATTCAATATTTCCCTTTTTAGAGGACAAA

TTATCGCATTTAAATTATGTGTCAGATATACTAATACCTTATCCCATCCATCTGAAAATCTTGGTTCAAA

TCCTTCAATGCTGGATCCAAGATGTTCCTTCTTTACATTTATTGCGATTCTTTCTTCACGAATATCATAA

TTGGAATAGTCTTATTACTCCGAATAATTCTATTTTTTTTTTTCAAAATAAAAGACTATTTCGGTTCTCA

TATAATTCTTATGTATCTGAATGCGAATTTGTATTAGTTTTTCTTCGTAAACAATCTTCTTATTTACGAT

TAACATCTTCTGTAGCTTTTCTTGAGCGAACACATTTCTATGGAAAAATAGAACATCTTATAGTAGTGCG

CCGTAATTATTTTCAGAAGACCCTATGGTTCTTCAAGGATCCCTTCATGCATTANGNTCGATMTCAAGGA

CAAGCAATTNNGGTTTCAAAGGGGACTCATCTTCTGATGAAGAAATGGAAATGTCACCTTGTCAATTTCT

GGCAATATTATTTTCACTTTTGGTCTCAWCCGTACAGGATACATATAAACCAATTATCAAACTGTTCTTT

CTATTTTCTAGGTTATTTTTCAAGTGTACTAATAAATCCTTCGTCGGTAAGGAATCAAATGCTAGAGAAT

TCATTTCTAATAGATACTGTTATTAAAAAATTCGATACCAGAGTCCCAGTTATTCCTCTTATTGGATCAT

TGTATAAAGCTAAATTTTGTACCGTATCGGGGCATCCTATTAGTAAGCCGATCTGGACCCATTTATCAGA

TTGCGATATTATTGATCGATTTGGTCGGATATGTAGAAATCTTTCTCATTATCACAGTGGATCCTCAAAA

AAACAGGGTTTGTATCGAATAAAGTATATACTTCGATTTTCGTGTGCTAGAACTTTGGCTCGTAAACATA

AAAGTACGGTACGCGCTTTTTTGCAAAGATTAGGTTCGGGATTATTAGAAGAATTCTTTACGGAAGAAGA

AGAAGTTGTTTCTTTGATCTTCCAAAAAACAACTTCTTTTTCTTTACATGAATCACATATAGAACGTATT

TGGTATTTGGATATTATCCGTATCAATGACTTGGTGAATTATTCATAATGGGTTTGGTGACGTGATGAGA

CTTATGAATAGAATAGAATAGAAATGATCTATAAATGATCAAGAGAGAAAAAAATTCATGAATTTTCATT

CTGAAATGCTCATTGCAGTAGTGTAGTGGTTGAATCAACTGAGTAGCCAAAATTATTATACTTTC

>Pseudophoenix_vinifera_AM114606.1

GACCATATTGCACTATGTATCATTTGATAACCCAAAAAATGAAATGGGTCCTGCCTCTGGTTTAAGTAGA

AATGTAAATGGAAGAATTACAAGGATATTTAGAAAAAGATAGATCTCGGCAACAACACTTTCTATATCCG

CTTCTCTTTAAGGAGTATATTTACACATTTGCTCATGATCGTGGTTTAAATGGTTCGATTTTTTACGAAT

CCACAGAAATTTTTGGTTATGACAATAAATCTAGTTCAGTACTTGTGAAACGTTCAATTATTCGAATGTA

TCAACAGAATTATTTGATTTATTCGGTTAATGATTCTAACCAAAATCGATTCGTTGGGCACAACAATTAT

TTTTATTTTCATTTTTATTTTCAGATGATATTGGAAGGTTTTGCAGTCATTGTGGAAATTCCATTCTTGC

TGCGATTAGTATCTTCCCTCGAAGAAAAAAAAATACCAAAATCTCAGAATTTGAATTTACGATCTATTCA

TTCAATATTTCCCTTTTTAGAGGACAAATTATCGCATTTAAATTATGTGTCAGATATACTAATACCTTAT

CCCATCCATCTGAAAATCTTGGTTCAAATCCTTCAATGCTGGATCCAAGATGTTCCTTCTTTACATTTAT

TGCGATTCTTTCTTCACGAATATCATAATTGGAATAGTCTTATTACTCCGAATAATTCTATTTTTTTTTT

TTCAAAATAAAAGACTATTTCGGTTCTCATATAATTCTTATGTATCTGAATGCGAATTTGTATTAGTTTT

TCTTCGTAAACAATCTTCTTATTTACGATTAACATCTTCTGTAGCTTTTCTTGAGCGAACACATTTCTAT

GGAAAAATAGAACATCTTATAGTAGTGCGCCGTAATTATTTTCAGAAGACCCTATGGTTCTTCAAGGATC

CCTTCATGCATTATGTTCGATATCAAGGAAAAGCAATTCTGGTTTCAAAGGGGACTCATCTTCTGATGAA

GAAATGGAAATGTCACCTTGTCAATTTCTGGCAATATTATTTTCACTTTTGGTCTCAACCGTACAGGATA

CATATAAACCAATTATCAAACTGTTCTTTCTATTTTCTAGGTTATTTTTCAAGTGTACTAATAAATCCTT

CGTCGGTAAGGAATCAAATGCTAGAGAATTCATTTCTAATAGATACTGTTATTAAAAAATTCGATACCAG

AGTCCCAGTTATTCCTCTTATTGGATCATTGTATAAAGCTAAATTTTGTACCGTATCGGGGCATCCTATT

AGTAAGCCGATCTGGACCCATTTATCAGATTGCGATATTATTGATCGATTTGGTCGGATATGTAGAAATC

TTTCTCATTATCACAGTGGATCCTCAAAAAAACAGGGTTTGTATCGAATAAAGTATATACTTCGATTTTC

GTGTGCTAGAACTTTGGCTCGTAAACATAAAAGTACGGTACGCGCTTTTTTGCAAAGATTAGGTTCGGGA

TTATTAGAAGAATTCTTTACGGAAGAAGAAGAAGTTGTTTCTTTGATCTTCCAAAAAACAACTTCTTTTT

CTTTACATGAATCACATATAGAACGTATTTGGTATTTGGATATTATCCGTATCAATGACTTGGTGAATTA

TTCATAATGGGTTTGGTGACGTGATGAGACTTATGAATAGAATAGAATAGAAATGATCTATAAATGATCA

AGAGAGAAAAAAATTCATGAATTTTCATTCTGAAATGCTCATTGCAGTAGTGTAGTGGTTGAATCAACTG

AGTAGCCAAAATTATTATACTTTCTTCTCGGGACCCAAGTTTTATATTATACATAGGTAAAGTC

>Pseudophoenix_sargentii_AM114605.1

GACCATATTGCACTATGTATCATTTGATAACCCAAAAAATGAAATGGGTCCTGCCCCTGGTTTAAGTAGA

AATGTAAATGGAAGAATTACAAGGATATTTAGAAAAAGATAGATCTCGGCAACAACACTTTCTATATCCG

CTTCTCTTTAAGGAGTATATTTACACATTTGCTCATGATCGTGGTTTAAATGGTTCGATTTTTTACGAAT

CCACAGAAATTTTTGGTTATGACAATAAATCTAGTTCAGTACTTGTGAAACGTTCAATTATTCGAATGTA

TCAACAGAATTATTTGATTTATTCGGTTAATGATTCTAACCAAAATCGATTCGTTGGGCACAACAATTAT

TTTTATTTTCATTTTTATTTTCAGATGATATTGGAAGGTTTTGCAGTCATTGTGGAAATTCCATTCTTGC

TGCGATTAGTATCTTCCCTCGAAGAAAAAAAAATACCAAAATCTCAGAATTTGAATTTACGATCTATTCA

TTCAATATTTCCCTTTTTAGAGGACAAATTATCGCATTTAAATTATGTGTCAGATATACTAATACCTTAT

CCCATCCATCTGAAAATCTTGGTTCAAATCCTTCAATGCTGGATCCAAGATGTTCCTTCTTTACATTTAT

TGCGATTCTTTCTTCACGAATATCATAATTGGAATAGTCTTATTACTCCGAATAATTCTATTTTTTTTTT

TCAAAATAAAAGACTATTTCGGTTCTCATATAATTCTTATGTATCGGAATGCGAATTTGTATTAGTTTTT

CTTCGTAAACAATCTTCTTATTTACGATTAACATCTTCTGTAGCTTTTCTTGAGCGAACACATTTCTATG

GAAAAATAGAACATCTTATAGTAGTGCGCCGTAATTATTTTCAGAAGACCCTATGGTTCTTCAAGGATCC

CTTCATGCATTATGTTCGATATCAAGGAAAAGCAATTCTGGTTTCAAAGGGGACTCATCTTCTGATGAAG

AAATGGAAATGTCACCTTGTCAATTTCTGGCAATATTATTTTCACTTTTGGTCTCAACCGTACAGGATAC

ATATAAACCAATTATCAAACTGTTCTTTCTATTTTCTAGGTTATTTTTCAAGTGTACTAATAAATCCTTC

GTCGGTAAGGAATCAAATGCTAGAGAATTCATTTCTAATAGATACTGTTATTAAAAAATTCGATACCAGA

GTCCCAGTTATTCCTCTTATTGGATCATTGTATAAAGCTAAATTTTGTACCGTATCGGGGCATCCTATTA

GTAAGCCGATCTGGACCCATTTATCAGATTGCGATATTATTGATCGATTTGGTCGGATATGTAGAAATCT

TTCTCATTATCACAGTGGATCCTCAAAAAAACAGGGTTTGTATCGAATAAAGTATATACTTCGATTTTCG

TGTGCTAGAACTTTGGCTCGTAAACATAAAAGTACGGTACGCGCTTTTTTGCAAAGATTAGGTTCGGGAT

TATTAGAAGAATTCTTTACGGAAGAAGAAGAAGTTGTTTCTTTGATCTTCCAAAAAACAACTTCTTTTTC

TTTACATGAATCACATATAGAACGTATTTGGTATTTGGATATTATCCGTATCAATGACTTGGTGAATTAT

TCATAATGGGTTTGGTGACGTGATGAGACTTATGAATAGAATAGAATAGAAATGATCTATAAATGATCAA

GAGAGAAAAAAATTCATGAATTTTCATTCTGAAATGCTCATTGCAGTAGTGTAGTGGTTGAATCAACTGA

GTAGCCAAAATTATTATACTTTCTTCTCGGGACCCAAGTTTTATATTATACATAGGTAAAGTC

>Raphia_farinifera_AM114544.1

GACCATATTGCACTATGTATCATTTGATAACCCAAGAAATGGGTCCTGCCTCTGGTTCAATTAGAAATGT

AAATGGAAGAATTACAAGGATATTTAGAAAAAGATATATCTCGGCAACAACACTTCCTATATCCGCTTCT

CTTTAAGGAGTATATTTACACATTTGTTCATGATCGTGTTTTAAATGGTTCGATTTTTTACGAATCCGTG

GAAATTTTGGGTTATGACAATAAATCTAGTTCAGTACTTGTGAAACGTTTAATTATTCGAATGTATCAAC

AGAATTATTTGATTTATTCGGTTAATGATTCTAACCAAAATCGATTCGTTGGGTACAACAATTATTTTTA

TTTTAATTTTTATTCTCAGATAATATTGGAAGGTTTTGCAGTCATTGTGGAAATTCCATTCTTGCTGCGA

TTAGTATCTTCCCTCGAAGATAAAATACCAAAATCTCAGAATTTGAATTTACGATCTATTCATTCAATAT

TTCCCTTTTTAGAGGACAAATTATCGCATTTAAATTATGTGTCAGATATACTAATACCTTATCCCATCCA

TCTGGAAATCTTGGTTCAAATCCTTCAATGCTGGATCCAAGATGTTCCCTCTTTACATTTATTGCGATTC

TTTCTTCACGAATATCATAATTGGAATAGTCTTATTACTCCGAATAATTTTATTTTATTTTATTTTTCAA

AAGAAAATAAAAGACTATTTCGGTTCCTATATAATTCTTATGTATCTGAATGCGAATTTGTATTAGTTTT

TCTTCGTAAACAATCTTCTTATTTACGATTAACATCTTCTGGAGCTTTTCTTGAGCGAACACATTTCTAT

AGAAAAATAGAACATCTTATAGTAGTGCACCATAATTATTTTCAGAGGACCCTATGGTCCTTCAAGGATC

CTTTCATGCATTATGTTCGATATCAAGGAAAAGCAATTCTGGTTTCAAAGGGAGCTCATCTTCTGATGAA

GAAATGGAAATGTCACCTTGTCAATTTCTGGCAATATTATTTTCACTTTTGGTCTCAACCGTACAGGATC

CATATAGACCAATTATCAAACTGTTCTTTCTATTTTCTAGGTTATCTTTCAAGTGTATTAATAAATCTTT

CGACGGTAAGGAATCAAATGCTAGAGAATTCATTTCTAATGGATACTGTTACTAAAAAATTCGATACCAG

AGTCCCAGTTATTCCTCTTATTGAATCATTGTCTAAAGCTAAATTTTGTACCGTATCGGGGCACCCTATT

AGTAAGCCGATCTGGACCGATTTATCAGATTGCGATATTATTGATCGATTTGGTCGGATATGTAGAAATC

TTTCTCATTATCACAGTGGATCCTCAAAAAAACAGAGTTTGTATCGAATAAAGTATATACTTCGACTTTC

GTGTGCTAGAACTTTGGCTCGTAAACATAAAAGTACGGCACGCGCTTTTTTGCAAAGATTAGGCTCGGGA

TTATTAGAAGAATTCTTTACGGAAGAAGAACAAGTTGTTTATTTGATCTTCCCCAAAACAACCTCTTTTT

CTTTACATGGATCACATAGAGAACGTATTTGGTATTTGGATATTATACGTATCAATGACCTGGTGAATCA

TTCATAATTGGTTTGGTGACGTGATGAGACTTATAAATAGAATAGAAATGATCTATAAATGATCAAGAGA

GAATGAAAATTCATGAATTTTCATTCTGAAATGCTCATTGTAGTAGTGTAGTGGTTGAATCAACCGAGTA

GTCAAAATTATTATACTTTCTTCTCGGGATGTAAGTTTTATATTATACATAGGGAAAGTC

>Reinhardtia_gracilis_var.rostrata_HQ265570.1

TCCTGCCTCTGGTTCAGTAGAAATGTAAATGGAAGAATTACAAGGATATTTAGAAAAAGATAGATCTCGG

CAACAACACTTTCTATATCCGCTTCTCTTTAAGGAGTATATTTACACATTTGCTCATGATCGTGGTTTAA

ATGGTTCGATTTTTTACGAATCCACGGAAATTTTTGGTTATGACAATAAATCTAGTTTAGTACTTGTGAA

ACGTTCAATTATTCGAATGTATCAACAGAATTATTTGATTTATTCGGTTAATGATTCTAACCAAAATCGA

TTCGTTGGGCACAACAATTATTTTTATTCTCAGATGATATTGGAAGGTTTTGCAGTCATTGTGGAAATTC

CATTCTTGCTGCGATTAGTATCTTCTCTCGAAGAAAAAAAAATACCAAAATCTCAGAATTTGAATTTACG

ATCTATTCATTCAATATTTCCCTTTTTGGAGGACAAATTATCGCATTTAAATTATGTGTCAGATATACTA

ATACCTTATCCCATCCATCTGAAAATCTTGGTTCAAATCCTTCAATGCTGGATCCAAGATGTTCCTTCTT

TACATTTATTGCGATTCTTTCTTCACGAATATCATAATTGGAATAGTCTTATTACTCCGAATAATTCTAT

TTTTTTTTCAAAAGAAAATAAAAGACTATTTCGGTTCCCATATAATTCTTATGTATCTGAATGCGAATTT

GTATTAGTTTTTCTTCGTAAACAATCTTCTTATTTACGATTAACATCTTCTGGAGCTTTTCTTGAGCGAA

CACATTTCTATGGAAAAATAGAACATCTTATAGTAGTAGTAGTGCGCCATAATTATTTTCAGAAGACCCT

ATGGTTCTTCAAGGATCCCTTCATGCATTATGTTCGATATCAAGGAAAAGCAATTCTGGTTTCAAAGGGG

ACTCATCTTCTGATGAAGAAATGGAAATGTCATCTTGTCAATTTCTGGCAATATTATTTTCACTTTTGGT

CTCAACCGTACAGGATCCATATAAACCAATTATCAAGCTGTTCTTTCTATTTTCTAGGTTATCTTTCAAG

TGTACTAATAAATCCTTCGGCGGTAAGGAATCAAATGCTAGAGAATTCATTTCTAATAGATACTGTTATT

AAAAAATTCGATACCAGAGTCCCAGTTATTCCTCTTATTGGATCATTGTCTAAAGCTAAATTTTGTACCG

TATCGGGGCATCCTATTAGTAAGCCGATCTGGACCAATTTATCAGATTGCGATATTATTGATCGATTTGG

TCGGATATGTAGAAATCTTTCTCATTATCACAGTGGATCCTCAAAAAAACAGAGTTTGTATCGAATAAAG

TATATACTTCGATTTTCGTGTGCTAGAACTTTGGCTCGTAAACATAAAAGTATGGTACGCGCTTTTTTGC

AAAGATTAGGTTCGGGATTATTAGAAGAATTCTTTACGGAAGAAGAACAAGTTGTTTCTTTGATCTTCCC

AAAAACAACTTCTTTTTCTTTACATGAATCACATATAGAACGTATTTGGTATTTGGATATTATCCGTATC

AATGACCTGGTGAATTATTCATAATGGGTTTGGTGACGTGATGAGACTTATGAATAGAATAGAAATGATC

TATAAATGATCAAGAGAGAAAAAAATTCATGAATTTTCATTCTGAAATGCTCATTGCAGTAGTGTAGTGG

TTGAATCAACTGAGTAGTCAAAATTATTATACTTTCTTCTCGGGACCCAAGTTTTATATTATACATAGGT

AAAGTCGTGTGCAATGAAAAATGCAAGCACGGTT

>Reinhardtia_simplex_HQ265571.1

AAATGAAATGGCCCTGCCTCTGGTTCAAGTAGAAATGTAAATGGAAGAATTACAAGGATATTTAGAAAAA

GATAGATCTCGGCAACAACACTTTCTATATCCGCTTCTCTTTAAGGAGTATATTTACACATTTGCTCATG

ATCGTGGTTTAAATAGTTCGATTTTTTACGAATCCACGGAAATTTTTGGTTATGACAATAAATCTAGTTT

AGTACTTGTGAAACGTTCAATTATTCGAATGTATCGACAGAATTATTTGATTTATTCGGTTAATGATTCT

AACCAAAATCGATTCGTTGGGCACAACAATTATTTTGATTTTCATTTTTATTCTCAGATGATATTGGAAG

GTTTTGCAGTCATTGTGGAAATTCCATTCTTGCTGCGATTAGTATCTTCTCTCGAAGAAAAAAAAATACC

AAAATCTCAGAATTTGAATTTACGATCTATTCATTCAATATTTCCCTTTTTGGAGGACAAATTATCGCAT

TTAAATTATGTGTCAGATATACTAATACCTTATCCCATCCATCTGAAAATCTTGGTTCAAATCCTTCAAT

GCTGGATCCAAGATGTTCCTTCTTTACATTTATTGCGATTCTTTCTTCACGAATATCATAATTGGAATAG

TCTTATTACTCCGAATAATTCTATTTTTTTTTCAAAAGAAAATAAAAGACTATTTCGGTTCCCATATAAT

TCTTATGTATCTGAATGCGAATTTGTATTAGTTTTTCTTCGTAAACAATCTTCTTATTTACGATTAACAT

CTTCTGGAGCTTTTCTTGAGCGAACACATTTCTATGGAAAAATAGAACATCTTATAGTAGTAGTAGTGCG

CCATAATTATTTTCAGAAGACCCTATGGTTCTTCAAGGATCCCTTCATGCATTATGTTCGATATCAAGGA

AAAGCAATTCTGGTTTCAAAGGGGACTCATCTTCTGATGAAGAAATGGAAATGTCATCTTGTCAATTTCT

GGCAATATTATTTTCACTTTTGGTCTCAACCATACAGGATCCATATAAACCAATTATCAAGCTGTTCTTT

CTATTTTCTAGGTTATCTTTCAAGTGTACTAATAAATCCTTCGGCGGTAAGGAATCAAATGCTAGAGAAT

TCATTTCTAATAGATACTGTTATTAAAAAATTCGATACCAGAGTCCCAGTTATTCCTCTTATTGGATCAT

TGTCTAAAGCTAAATTTTGTACCGTATCGGGGCATCCTATTAGTAAGCCGATCTGGACCAATTTATCAGA

TTGCGATATTATTGATCGATTTGGTCGGATATGTAGAAATCTTTCTCATTATCACAGTGGATCCTCAAAA

AAACAGAGTTTGTATCAAATAAAGTATATACTTCGATTTTCGTGTGCTAGAACTTTGGCTCGTAAACATA

AAAGTATGGTACGCGCTTTTTTGCAAAGATTAGGTTCGGGATTATTAGAAGAATTCTTTACGGAAGAAGA

ACAAGTTGTTTCTTTGATCTTCCCAAAAACAACTTCTTTTTCTTTACATGAATCACATATAGAACGTATT

TGGTATTTGGATATTATCCGTATCAATGACCTGGTGAATTATTCATAATGGGTTTGGTGACGTGATGAGA

CTTATGAATAGAATAGAAATGATCTATAAATGATCAAGAGAGAAAAAAATTCATGAATTTTCATTCTGAA

ATGCTCATTGCAGTAGTGTAGTGGATGAATCAACTGAGTAGTCAAAATTATTATACTTTCTTCTCGGGAC

CCAAGTTTTATACTTATACATAGGTAAAGTTCGTGTGCAATGAAAAATGCAACGCACCGGTTAGGGGAGG

G

>Reinhardtia_simplex_AM114631.1

GACCATATTGCACTATGTATCATTTGATAACCCAAAAAATGAAATGGGCCCTGCCTCTGGTTCAAGTAGA

AATGTAAATGGAAGAATTACAAGGATATTTAGAAAAAGATAGATCTCGGCAACAACACTTTCTATATCCG

CTTCTCTTTAAGGAGTATATTTACACATTTGCTCATGATCGTGGTTTAAATAGTTCGATTTTTTACGAAT

CCACGGAAATTTTTGGTTATGACAATAAATCTAGTTTAGTACTTGTGAAACGTTCAATTATTCGAATGTA

TCGACAGAATTATTTGATTTATTCGGTTAATGATTCTAACCAAAATCGATTCGTTGGGCACAACAATTAT

TTTGATTTTCATTTTTATTCTCAGATGATATTGGAAGGTTTTGCAGTCATTGTGGAAATTCCATTCTTGC

TGCGATTAGTATCTTCTCTCGAAGAAAAAAAAATACCAAAATCTCAGAATTTGAATTTACGATCTATTCA

TTCAATATTTCCCTTTTTGGAGGACAAATTATCGCATTTAAATTATGTGTCAGATATACTAATACCTTAT

CCCATCCATCTGAAAATCTTGGTTCAAATCCTTCAATGCTGGATCCAAGATGTTCCTTCTTTACATTTAT

TGCGATTCTTTCTTCACGAATATCATAATTGGAATAGTCTTATTACTCCGAATAATTCTATTTTTTTTTC

AAAAGAAAATAAAAGACTATTTCGGTTCCCATATAATTCTTATGTATCTGAATGCGAATTTGTATTAGTT

TTTCTTCGTAAACAATCTTCTTATTTACGATTAACATCTTCTGGAGCTTTTCTTGAGCGAACACATTTCT

ATGGAAAAATAGAACATCTTATAGTAGTAGTAGTGCGCCATAATTATTTTCAGAAGACCCTATGGTTCTT

CAAGGATCCCTTCATGCATTATGTTCGATATCAAGGAAAAGCAATTCTGGTTTCAAAGGGGACTCATCTT

CTGATGAAGAAATGGAAATGTCATCTTGTCAATTTCTGGCAATATTATTTTCACTTTTGGTCTCAACCAT

ACAGGATCCATATAAACCAATTATCAAGCTGTTCTTTCTATTTTCTAGGTTATCTTTCAAGTGTACTAAT

AAATCCTTCGGCGGTAAGGAATCAAATGCTAGAGAATTCATTTCTAATAGATACTGTTATTAAAAAATTC

GATACCAGAGTCCCAGTTATTCCTCTTATTGGATCATTGTCTAAAGCTAAATTTTGTACCGTATCGGGGC

ATCCTATTAGTAAGCCGATCTGGACCAATTTATCAGATTGCGATATTATTGATCGATTTGGTCGGATATG

TAGAAATCTTTCTCATTATCACAGTGGATCCTCAAAAAAACAGAGTTTGTATCAAATAAAGTATATACTT

CGATTTTCGTGTGCTAGAACTTTGGCTCGTAAACATAAAAGTATGGTACGCGCTTTTTTGCAAAGATTAG

GTTCGGGATTATTAGAAGAATTCTTTACGGAAGAAGAACAAGTTGTTTCTTTGATCTTCCCAAAAACAAC

TTCTTTTTCTTTACATGAATCACATATAGAACGTATTTGGTATTTGGATATTATCCGTATCAATGACCTG

GTGAATTATTCATAATGGGTTTGGTGACGTGATGAGACTTATGAATAGAATAGAAATGATCTATAAATGA

TCAAGAGAGAAAAAAATTCATGAATTTTCATTCTGAAATGCTCATTGCAGTAGTGTAGTGGTTGAATCAA

CTGAGTAGTCAAAATTATTATACTTTCTTCTCGGGACCCAAGTTTTATATTATACATAGGTAAAGTC

>Roystonea_borinquena_HM446742.1

TGTAGTAGTAGTGCGCCGTAATTATTTTCAGAAGACCCTATGGTTCTTCAAGGATCCCCTCATGCATTAT

GTTCGATATCAAGGAAAAGCAATTCTAGTTTCAAAGGGGACTCATCTTCTGATGAAGAAAAAGAAATGTC

ACCTTGTCAATTTCTGGCAATATTATTTTCACTTTTGGTCTCAACCGTACAGGATCCATATAAACCAATT

ATCAAGCTGTTCTTTCTATTTTCTAGGTTATCTTTCAAGTGTACTAATAAATCCTTCGGCGGTAAGGAAT

CAAGTGCTAGAGAATTCATTTCTAATAGATACTGTTATTAAAAAATTCGATACCAGAGTCCCAGTTATTC

CTCTTATTGGATCATTGTCTAAAGCTAAATTTTGTACCGTATCGGGGCATCCTATTAGTAAGCCGATCTG

GACCAATTTATCAGATTGCGATATTATTGATCGATTTGGTCGGATATGTAGAAATCTTTCTCATTATCAC

AGTGGATCCTCAAAAAAACAGAGTTTGTATCGAATAAAGTATATA

>Roystonea_oleracea_AM114630.1

GACCATATTGCACTATGTATCATTTGATAACCCAAAAAATGAAATGGGTCCTGCCTCTGGTTCAAGTAGA

AATGTAAATGGAAGAATTACAAGGATATTTAGAAAAAGATAGATCTCGGCAACAACACTTTCTATATCCG

CTTCTCTTTAAGGAGTATATTTACACATTTGCTCATGATCGTGGTTTAAATGGTTCGATTTTTTACGAAT

CCACGGAAATTTTTGGTTATGACAATAAATCTAGTTCAGTACTTGTGAAACGTTCAATTATTCGAATGTA

TCAACAGAATTATTTGATTTATTCGGTTAATGATTCTAACCAAAATCGATTCGTTGGGCACAACAATTAT

TTTGATTTTCATTTTTATTCTCAGATGATATTGGAAGGTTTTGCAGTCATTGTGGAAATTCCATTCTTGC

TGCGATTAGTATCTTCCCTCGAAGAAAAAAAAATACCAAAATCTCAGAATTTGAATTTACGATCTATTCA

TTCAATATTTCCCTTTTTGGAGGACAAATTATCGCATTTAAATTATGTGTCAGATATACTAATACCTTAT

CCCATCCATCTGAAAATCTTGGTTCAAATCCTTCAATGCTGGATCCAAGATGTTCCTTCTTTACATTTAT

TGCGATTCTTTCTTCACGAATATCATAATTGGAATAGTCTTATTACTCCGAATAATTCTATTTTTTTTTT

TTCAAAAGAAAATAAAAGACTATTTCGGTTCCCATATAATTCTTATGTATCTGAATGCGAATTTGTATTA

GTTTTTCTTCGTAAACAATCTTCTTATTTACGATTAACATCTTCTGGAGCTTTTCTTGAGCGAACACATT

TCTATGGAAAAATAGAACATCTTATAGTAGTAGTAGTGCGCCGTAATTATTTTCAGAAGACCCTATGGTT

CTTCAAGGATCCCCTCATGCATTATGTTCGATATCAAGGAAAAGCAATTCTAGTTTCAAAGGGGACTCAT

CTTCTGATGAAGAAATGGAAATGTCACCTTGTCAATTTCTGGCAATATTATTTTCACTTTTGGTCTCAAC

CGTACAGGATCCATATAAACCAATTATCAAGCTGTTCTTTCTATTTTCTAGGTTATCTTTCAAGTGTACT

AATAAATCCTTCGGCGGTAAGGAATCAAGTGCTAGAGAATTCATTTCTAATAGATACTGTTATTAAAAAA

TTCGATACCAGAGTCCCAGTTATTCCTCTTATTGGATCATTGTCTAAAGCTAAATTTTGTACCGTATCGG

GGCATCCTATTAGTAAGCCGATCTGGACCAATTTATCAGATTGCGATATTATTGATCGATTTGGTCGGAT

ATGTAGAAATCTTTCTCATTATCACAGTGGATCCTCAAAAAAACAGAGTTTGTATCGAATAAAGTATATA

CTTCGATTTTCGTGTGCTAGAACTTTGGCTCGTAAACATAAAAGTATGGTACGCGCTTTTTTGCAAAGAT

TAGGTTCGGGATTATTAGAAGAATTCTTTACGGAAGAAGAACAAGTTGTTTCTTTGATCTTCCCAAAAAC

AACTTCTTTTTCTTTACATGAATCACATATAGAACGTATTTGGTATTTGGATATTATCCGTATCAATGAC

CTGGTGAATTATTCATAATGGGTTTGGTGACGTGATGAGACTTATGAATAGAATAGAAATGATCTATAAA

TGATCAAGAGAGAAAAAAATTCATGAATTTTCATTCTGAAATGCTTATTGCAGTAGTGTAGTGGTTGAAT

CAACTGAGTAGTCAAAATTATTATACTTTCTTCTCGGGACCCAAGTTTTATATTATACATAGGTAAAGTC

>Rhapidophyllum_hystrix_HQ720323.1

GGTTCAAGTAGAAATGTAAATGGAAGAATTACAAGGATATTTAGAAAGAGATAGATCTCTGCAACAACAC

TTTCTATATCCGCTTCTCTTTAAGGAGTATATTTACACATTTCTTCATGATCGTGGTTTAAATGGTTCGA

TTTTTTACGAATCCACGGAAATTTTTGGTTATGACAATAAATCTAGTTCAGTACTTGTGAAACGTTCAAT

TATTCGAATGTATCAACAGAATTATTTGATTTATTCGGTTAATGATTCTAACCAAAATCGATTCGTTGGG

CACAACAATTATTTTTATTTTCATTTTTATTCTCAGATGATATTGGAAGGTTTTGCAGTCATTGTGGAAA

TTCCATTCTTGCTGCGATTGGTATCTTCCCTCGAAGAAAAAAAAATACCAAAATCTCAGAATTTGAATTT

ACGATCTATTCATTCAATATTTCCCTTTTTGGAGGACAAATTATCGCATTTAAATTATGTGTCAGATATA

CTAATACCTTATCCCATCCATCTGAAAATCTTGGTTCAAATCCTTCAATTCTGGATCCAAGATGTTCCTT

CTTTACATTTATTGCGATTCTTTCTTCACGAATATCATAATTGGAATAGTCTTATTACTCCGAATAATTC

TATTTTTCTTTTTTCAAAAGAAAATAAAAGACTATTTCGGTTCCCATATAATTCTTATGTATCTGAATGT

GAATTTGTATTAGTTTTTCTTCGTAAACAATCTTCTTATTTACGATTAACATCTTCTGGAGCTTTTCTTG

AGCGAACGCATTTCTATGGAAAAATAGAATATTGTATAGTAGTGCGCCGTAATTATTTTCAGAAGACCCT

ATGGTTTTTCAAGGATCCCTTCATGCATTATGTTCGATATCAAGGAAAAGCAATTCTGGTTTCAAAGGGG

ACTCATCTTCTGATGAAGAAATGGAAATGTCACCTTGTCAATTTCTGGCAATATTATTTTCACTTTTGGT

CTCAACCGTACAGGATTCATATAAACCAATTATCAAACTGTTCTTTCTATTTTCTAGGTTATCTTTCAAG

TGTACTAATAAATCCTTCGGCGGTAAGGAATCAAATGCTAGAGAATTCATTTCTAATAGATACTGTTATT

AAAAAATTCGATACCAGAGTCCCAGTTATTCCTCTTATTAGATCATTGTCTAAAGCTAAATTTTGTACCG

TATCGGGGCATCCTATTAGTAAGCCGATCTGGACCGATTTATCAGATTGCGATATTATTGATCGATTTGG

TCGGATATGTAGAAATCTTTCTCATTATCACAGTGGATCCTCAAAAAAACAGAGTTTGTATCGAATAAAG

TATATACTTCGATTTTCGTGTGCTAGAACTTTGGCTCGTAAACATAAAAGTATGGTACGCGCTTTTTTGC

AAAGATTAGGTTCGGGATTATTAGAAGAATTTTTTATGGAAGAAGAAGAAGTTGTTTCTTTGATCTTCCC

AAAAACAACTTCTTTTTCTTTACATGAATCACATATGGAACGTATTTGGTATTTGGATATTATCCGTATC

AATGACCTGGTGAATTATTCATAATTGGTTTGTTGACGTGATGAGACTTATGAATAGAATAGAAATGATC

TATAAATGATCAAGAGAGAAAAAAATTCATGAATTTTCATTCTGAAATGCTCATTGCAGTAGTGTAGTGG

TTGAATCAACTGAGTAGTCAAAATTATTATACT

>Rhapidophyllum_hystrix_AM114571.1

GACCATATTGCACTATGTATCATTTGATAACCCAAAAATTGAAATAGGTCCCGCCTCTGGTTCAAGTAGA

AATGTAAATGGAAGAATTACAAGGATATTTAGAAAGAGATAGATCTCTGCAACAACACTTTCTATATCCG

CTTCTCTTTAAGGAGTATATTTACACATTTCTTCATGATCGTGGTTTAAATGGTTCGATTTTTTACGAAT

CCACGGAAATTTTTGGTTATGACAATAAATCTAGTTCAGTACTTGTGAAACGTTCAATTATTCGAATGTA

TCAACAGAATTATTTGATTTATTCGGTTAATGATTCTAACCAAAATCGATTCGTTGGGCACAACAATTAT

TTTTATTTTCATTTTTATTCTCAGATGATATTGGAAGGTTTTGCAGTCATTGTGGAAATTCCATTCTTGC

TGCGATTGGTATCTTCCCTCGAAGAAAAAAAAATACCAAAATCTCAGAATTTGAATTTACGATCTATTCA

TTCAATATTTCCCTTTTTGGAGGACAAATTATCGCATTTAAATTATGTGTCAGATATACTAATACCTTAT

CCCATCCATCTGAAAATCTTGGTTCAAATCCTTCAATTCTGGATCCAAGATGTTCCTTCTTTACATTTAT

TGCGATTCTTTCTTCACGAATATCATAATTGGAATAGTCTTATTACTCCGAATAATTCTATTTTTCTTTT

TTCAAAAGAAAATAAAAGACTATTTCGGTTCCCATATAATTCTTATGTATCTGAATGTGAATTTGTATTA

GTTTTTCTTCGTAAACAATCTTCTTATTTACGATTAACATCTTCTGGAGCTTTTCTTGAGCGAACGCATT

TCTATGGAAAAATAGAATATTGTATAGTAGTGCGCCGTAATTATTTTCAGAAGACCCTATGGTTTTTCAA

GGATCCCTTCATGCATTATGTTCGATATCAAGGAAAAGCAATTCTGGTTTCAAAGGGGACTCATCTTCTG

ATGAAGAAATGGAAATGTCACCTTGTCAATTTCTGGCAATATTATTTTCACTTTTGGTCTCAACCGTACA

GGATTCATATAAACCAATTATCAAACTGTTCTTTCTATTTTCTAGGTTATCTTTCAAGTGTACTAATAAA

TCCTTCGGCGGTAAGGAATCAAATGCTAGAGAATTCATTTCTAATAGATACTGTTATTAAAAAATTCGAT

ACCAGAGTCCCAGTTATTCCTCTTATTAGATCATTGTCTAAAGCTAAATTTTGTACCGTATCGGGGCATC

CTATTAGTAAGCCGATCTGGACCGATTTATCAGATTGCGATATTATTGATCGATTTGGTCGGATATGTAG

AAATCTTTCTCATTATCACAGTGGATCCTCAAAAAAACAGAGTTTGTATCGAATAAAGTATATACTTCGA

TTTTCGTGTGCTAGAACTTTGGCTCGTAAACATAAAAGTATGGTACGCGCTTTTTTGCAAAGATTAGGTT

CGGGATTATTAGAAGAATTTTTTATGGAAGAAGAAGAAGTTGTTTCTTTGATCTTCCCAAAAACAACTTC

TTTTTCTTTACATGAATCACATATGGAACGTATTTGGTATTTGGATATTATCCGTATCAATGACCTGGTG

AATTATTCATAATTGGTTTGTTGACGTGATGAGACTTATGAATAGAATAGAAATGATCTATAAATGATCA

AGAGAGAAAAAAATTCATGAATTTTCATTCTGAAATGCTCATTGCAGTAGTGTAGTGGTTGAATCAACTG

AGTAGTCAAAATTATTATACTTTCTTCTCGGGACCCAAGTTTTTTTTATATTATACATAGGTAAAGTC

>Sabal_palmetto_HQ720346.1

AAATGTAAATGTAAATGGAAGAATTACAAGGATATTTAGAAAAAGATAGATCTCGGCAACAACACTTTCT

ATATCCGCTTCTCTTTAAGGAGTATATTTACACATTTGCTCATGATCGTGGTTTAAATGGTTCGATTTTT

TACGAATCCACGGAAATTTTTGGTTATGACAATAAATCTAGTTCAGTACTTGTGAAACGTTCAATTATTC

GAATGTATCAACAGAATTATTTGATTTATTCGGTTAATGATTCTAACCAAAATCGATTCGTTGGGCACAA

CAATTATTTTGATTTTTATTTTTATTCTCAGATGATATTGGAAGGTTTTGCAGTCATTGTGGAAATTCCA

TTCTTGCTGCGATTAGTATCTCCCCTCGAAGAAAAAAAAATACCAAAATCTCAAAATTTGAATTTACGAT

CTATTCATTCAATATTTCCCTTTTTGGAGGACAAATTATCGCATTTAAATTATGTGTCAGATATACTAAT

ACCTTATCCCATCCATCTGAAAATCTTGGTTCAAATCCTTCAATTCTGGATCCAAGATGTTCCTTCTTTA

CATTTATTGCGATTCTTTCTTCACGAATATCATAATTGGAATAGTCTTATTACTCCGAATAATTCTATTT

TTCCTTTTTCACTTTTTTCAAAAGAAAATAAAAGACTATTTCGGTTCCCATATAATTCTTATGTATCTGA

ATGCGAATTTGTATTAGTTTTTCTTTGTAAACAATCTTCTTATTTACGATTAACATCTTCTGGAGCTTTT

CTTGAGCGAACACATTTCTATGGAAAAATAGAACATCTTATAGTAGTGCGCCGTAATTATTTTCAGAAGA

CCCTATGGTTCTTCAAGGATCCCTTCATGCATTATGCTCGATATCAAGGAAAAGCAATTCTGGTTTCAAA

GGGGACTCATCTTCTGATGAAGAAATGGAAATGTCACCTTGTCAATTTCTGGCAATATTATTTTCACTTT

TGGTCTCAACCGTACAGGATCCATATAAACCAATTATCAAACTGTTCTTTCTATTTTCTAGGTTATCTTT

CAAGTGTACTAATAAATCCTTCGGCGGTAAGGAATCAAATGCTAGAGAATTCATTTCTAATAGATACTGT

TATTAAAAAATTCGATACCAGAGTCCCAGTTATTCCTCTTATTGGATCATTGTCTAAAGCTAAATTTTGT

ACCGTATCGGGGCATCCTATTAGTAAGCCGATCTGGACCGATTTATCAGATTGCGATATTATTGATCGAT

TTGGTCGGATATGTAGAAATCTTTCTCATTATCACAGTGGATCCTCAAAAAAACAGAGTTTGTATCGAAT

AAAGTATATACTTCGATTTTCGTGTGCTAGAACTTTGGCTCGTAAACATAAAAGTATGGTACGCGCTTTT

TTGCAAAGATTAGGTTCAGGATTATTAGAAGAATTTTTTATGGAAGAAGAAGAAGTTCTTTCTTTGATCT

TCCCAAAAACAACTTCTTTTTCTTTACATGAATCACATATAGAACGTATTTGGTATTTGGATATTATCCG

TATCAATGACCTGGTGAATTATTCATAATTGGTTTGGTGACGTGATGAGACTTATGAATAGAAAATAGAA

TAGAAATGATCTATAAATGATCAAGAGAGAAAAAAATTCATGAATTTTCATTCTGAAATGCTCATTGCAG

TAGTGTAGT

>Sabal_minor_AM114554.1

GACCATATTGCACTATGTATCCTTTGATAACCCAAAAAATGAAATGGGTCCCGCCTCTGGTTCAAGTAGA

AATGTAAATGTAAATGGAAGAATTACAAGGATATTTAGAAAAAGATAGATCTCGGCAACAACACTTTCTA

TATCCGCTTCTCTTTAAGGAGTATATTTACACATTTGCTCATGATCGTGGTTTAAATGGTTCGATTTTTT

ACGAATCCACGGAAATTTTTGGTTATGACAATAAATCTAGTTCAGTACTTGTGAAACGTTCAATTATTCG

AATGTATCAACAGAATTATTTGATTTATTCGGTTAATGATTCTAACCAAAATCGATTCGTTGGGCACAAC

AATTATTTTGATTTTTATTTTTATTCTCAGATGATATTGGAAGGTTTTGCAGTCATTGTGGAAATTCCAT

TCTTGCTGCGATTAGTATCTCCCCTCGAAGAAAAAAAAATACCAAAATCTCAAAATTTGAATTTACGATC

TATTCATTCAATATTTCCCTTTTTGGAGGACAAATTATCGCATTTAAATTATGTGTCAGATATACTAATA

CCTTATCCCATCCATCTGAAAATCTTGGTTCAAATCCTTCAATTCTGGATCCAAGATGTTCCTTCTTTAC

ATTTATTGCGATTCTTTCTTCACGAATATCATAATTGGAATAGTCTTATTACTCCGAATAATTCTATTTT

TCCTTTTTCACTTTTTTCAAAAGAAAATAAAAGACTATTTCGGTTCCCATATAATTCTTATGTATCTGAA

TGCGAATTTGTATTAGTTTTTCTTTGTAAACAATCTTCTTATTTACGATTAACATCTTCTGGAGCTTTTC

TTGAGCGAACACATTTCTATGGAAAAATAGAACATCTTATAGTAGTGCGCCGTAATTATTTTCAGAAGAC

CCTATGGTTCTTCAAGGATCCCTTCATGCATTATGTTCGATATCAAGGAAAAGCAATTCTGGTTTCAAAG

GGGACTCATCTTCTGATGAAGAAATGGAAATGTCACCTTGTCAATTTCTGGCAATATTATTTTCACTTTT

GGTCTCAACCGTACAGGATCCATATAAACCAATTATCAAACTGTTCTTTCTATTTTCTAGGTTATCTTTC

AAGTGTACTAATAAATCCTTCGGCGGTAAGGAATCAAATGCTAGAGAATTCATTTCTAATAGATACTGTT

ATTAAAAAATTCGATACCAGAGTCCCAGTTATTCCTCTTATTGGATCATTGTCTAAAGCTAAATTTTGTA

CCGTATCGGGGCATCCTATTAGTAAGCCGATCTGGACCGATTTATCAGATTGCGATATTATTGATCGATT

TGGTCGGATATGTAGAAATCTTTCTCATTATCACAGTGGATCCTCAAAAAAACAGAGTTTGTATCGAATA

AAGTATATACTTCGATTTTCGTGTGCTAGAACTTTGGCTCGTAAACATAAAAGTATGGTACGCGCTTTTT

TGCAAAGATTAGGTTCAGGATTATTAGAAGAATTTTTTATGGAAGAAGAAGAAGTTCTTTCTTTGATCTT

CCCAAAAACAACTTCTTTTTCTTTACATGAATCACATATAGAACGTATTTGGTATTTGGATATTATCCGT

ATCAATGACCTGGTGAATTATTCATAATTGGTTTGGTGACGTGATGAGACTTATGAATAGAAAATAGAAT

AGAAATGATCTATAAATGATCAAGAGAGAAAAAAATTCATGAATTTTCATTCTGAAATGCTCATTGCAGT

AGTGTAGTGGTTGAATCAACTGAGTAGTCAAAATTATTATACTTTCTTCTCGGGACCCAAGTTTTATATT

ATACATAGGTAAAGTC

>Sabal_bermudana_AM114553.1

GACCATATTGCACTATGTATCATTTGATAACCCAAAAAATGAAATGGGTCCCGCCTCTGGTTCAAGTAGA

AATGTAAATGTAAATGGAAGAATTACAAGGATATTTAGAAAAAGATAGATCTCGGCAACAACACTTTCTA

TATCCGCTTCTCTTTAAGGAGTATATTTACACATTTGCTCATGATCGTGGTTTAAATGGTTCGATTTTTT

ACGAATCCACGGAAATTTTTGGTTATGACAATAAATCTAGTTCAGTACTTGTGAAACGTTCAATTATTCG

AATGTATCAACAGAATTATTTGATTTATTCGGTTAATGATTCTAACCAAAATCGATTCGTTGGGCACAAC

AATTATTTTGATTTTTATTTTTATTCTCAGATGATATTGGAAGGTTTTGCAGTCATTGTGGAAATTCCAT

TCTTGCTGCGATTAGTATCTCCCCTCGAAGAAAAAAAAATACCAAAATCTCAAAATTTGAATTTACGATC

TATTCATTCAATATTTCCCTTTTTGGAGGACAAATTATCGCATTTAAATTATGTGTCAGATATACTAATA

CCTTATCCCATCCATCTGAAAATCTTGGTTCAAATCCTTCAATTCTGGATCCAAGATGTTCCTTCTTTAC

ATTTATTGCGATTCTTTCTTCACGAATATCATAATTGGAATAGTCTTATTACTCCGAATAATTCTATTTT

TCCTTTTTCACTTTTTTCAAAAGAAAATAAAAGACTATTTCGGTTCCCATATAATTCTTATGTATCTGAA

TGCGAATTTGTATTAGTTTTTCTTTGTAAACAATCTTCTTATTTACGATTAACATCTTCTGGAGCTTTTC

TTGAGCGAACACATTTCTATGGAAAAATAGAACATCTTATAGTAGTGCGCCGTAATTATTTTCAGAAGAC

CCTATGGTTCTTCAAGGATCCCTTCATGCATTATGCTCGATATCAAGGAAAAGCAATTCTGGTTTCAAAG

GGGACTCATCTTCTGATGAAGAAATGGAAATGTCACCTTGTCAATTTCTGGCAATATTATTTTCACTTTT

GGTCTCAACCGTACAGGATCCATATAAACCAATTATCAAACTGTTCTTTCTATTTTCTAGGTTATCTTTC

AAGTGTACTAATAAATCCTTCGGCGGTAAGGAATCAAATGCTAGAGAATTCATTTCTAATAGATACTGTT

ATTAAAAAATTCGATACCAGAGTCCCAGTTATTCCTCTTATTGGATCATTGTCTAAAGCTAAATTTTGTA

CCGTATCGGGGCATCCTATTAGTAAGCCGATCTGGACCGATTTATCAGATTGCGATATTATTGATCGATT

TGGTCGGATATGTAGAAATCTTTCTCATTATCACAGTGGATCCTCAAAAAAACAGAGTTTGTATCGAATA

AAGTATATACTTCGATTTTCGTGTGCTAGAACTTTGGCTCGTAAACATAAAAGTATGGTACGCGCTTTTT

TGCAAAGATTAGGTTCAGGATTATTAGAAGAATTTTTTATGGAAGAAGAAGAAGTTCTTTCTTTGATCTT

CCCAAAAACAACTTCTTTTTCTTTACATGAATCACATATAGAACGTATTTGGTATTTGGATATTATCCGT

ATCAATGACCTGGTGAATTATTCATAATTGGTTTGGTGACGTGATGAGACTTATGAATAGAAAATAGAAT

AGAAATGATCTATAAATGATCAAGAGAGAAAAAAATTCATGAATTTTCATTCTGAAATGCTCATTGCAGT

AGTGTAGTGGTTGAATCAACTGAGTAGTCAAAATTATTATACTTTCTTCTCGGGACCCAAGTTTTATATT

ATACATAGGTAAAGTC

>Schippia_concolor_AM114555.1

GACCATATTGCACTATGTATCATTTGATAACCCCAAAAATGAAATAGGTCCCACCTCTGGTTCAAGTAGA

AATGGAAATGGAAGAATTACAAGGATATTTAGAAAAAGATAGATCTCGGCAACAACACTTTCTATATCCG

CTTCTCTTTAAGGAGTATATTTACACATTTGCTCATGATCGTGGTTTAAATGGTTCGATTTTTTACGAAT

CCACGGAAATTTTTGGTTATGACAATAAATCTAGTTCAGTACTTGTGAAACGTTCAATTATTCGAATGTA

TCAACAGAATTATTTGATTTATTCGGTTAATGATTCTAACCAAAATCGATTCGTTGGGCACAACAATTAT

TTTTATTTTCATTTTTATTCTCAGATGATATTGGAAGGTTTTGCAGTCATTGTGGAAATTCCATTCTTGC

TGCGATTAGTATCTTCCCTCGAAGAAAAAAAAATACCAAAATCTCAAAATTTGAATTTACGATCTATTCA

TTCAATATTTCCCTTTTTGGAGGACAAATTATCGCATTTAAATTATGTGTCAGATATACTAATACCTTAT

CCCATACATCTGAAAATCTTGGTTCAAATCCTTCAATTCTGGATCCAAGATGTTCCTTCTTTACATTTAT

TGCGATTCTTTCTTCACGAATATCATAATTGGAATAGTCTTATTACTCCGAATAATTCTATTTTTCCTTT

TTTACTTTTTTCAAAAGAAAATAAAAGACTATTTCGGTTCCCATATAATTCTTATGTATCTGAATGCGAA

TTTTTATTAGTTTTTCTTCGTAAACAATCTTCTTATTTACGATTAACATTTTCTGGAGCTTTTCTTGAGC

GAACACATTTTTATGGAAAAATAGAACATCTTATAGTAGTGCGCCGTAATTATTTTCAGAAGACCCTATG

GTTCTTCAAGGATCCCTTCATGCATTATGTTCGATATCAAGGAAAAGCAATTCTGGTTTCAAAGGGGACT

CATCTTCTGATGAAGAAATGGAAATGTCACCTTGTCAATTTCTGGCAATATTATTTTCACTTTTGGTCTC

AACCGTACAGGATCCATATAAAGCAATTATCAAACTGTTCTTTCTATTTTTTAGGTTATCTTTCAAGTGT

ACTAATAAATCCTTTGGCGGTAAGGAATCAAATGCTAGAGAATTCATTTCTAATAGATACTCTTATTAAA

AAATTCGATACCAGAGTCCCAGTTATTCCTCTTATTGGATCATTGTCTAAAGCTAAATTTTGTACCGTAT

CGGGGCATCCTATTAGTAAGCCCATCTGGACCGATTTATCAGATTGCGATATTATTGATCGATTTGGTCG

GATATGTAGAAATCTTTCTCATTATCACAGTGGATCCTCAAAAAAACAGAGTTTGTATCGAATAAAGTAT

ATACTTCGATTTTCGTGTGCTAGAACTTTGGCTCGTAAACATAAAAGTACGGTACGCGCTTTTTTGCAAA

GATTAGGTTCAGGATTATTAGAAGAATTTTTTATGGAAGAAGAAGAAGTTGTTTCTTTGATCTTCCCAAA

AACAACTTCTTTTTCTTTACATGAATCACATATAGAACGTATTTGGTATTTGGATATTATCCGTATCAAT

GACCTGGTGAATTATTCATAATTGGTTTGGTGACGTGATGAGACTTATGAATAGAAAATAGAATAGAAAT

GATCTATAAATGATCAAGAGAGAAAAAAATTCATGAATTTTCATTCTGAAATGCTCATTGCAGTAGTGTA

GTGGTTGAATCAACTGAGTAGTCAAAATTATTATACTTTCTTCTCGGGACCCAAGTTTTATATTATACAT

AGGTAAAGTC

>Serenoa_repens_HQ720325.1

AATAGGTCCCGCCTCTGGTTCAAGTAGAAATGTAAATGGAAGAATTACAAGGATATTTAGAAAGAGATAG

ATCTCTGCAACAACACTTTCTATATCCGCTTCTCTTTAAGGAGTATATTTACACATTTCTTCATGATCGT

GGTTTAAATGGTTCGATTTTTTACGAATCCACGGAAATTTTTGGTTATGACAATAAATCTAGTTCAGTAC

TTGTGAAACGTTCAATTATTCGAATGTATCGACAGAATTATTTGATTTATTCGGTTAATGATTCTAACCA

AAATCGATTCGTTGGGCACAACAATTATTTTGATTTTCATTTTTATTCTCAGATGATATTGGAAGGTTTT

GCAGTCATTGTGGAAATTCCATTCTTGCTGCGATTGGTATCTTCCCTCGAAGAAAAAAAAATACCAAAAT

CTCARAATTTGAATTTACGATCTATTCATTCAATATTTCCCTTTTTGGAGGACAAATTATCGCATTTAAA

TTATGTGTCATATATACTAATACCTTATCCCATCCATCTGAAAATCTTGGTTCAAATCCTTCAATTCTGG

ATCCAARATGTTCCTTCTTTACATTTATTGCGATTCTTTCTTCACGAATATCATAATTGGAATAGTCTTA

TTACTCCGAATAATTCTATTTTTCTTTTTTCAAAAGAAAATAAAAGACTATTTCGGTTCCCATATAATTC

TTATGTATCTGAATGCGAATTTGTATTAGTTTTTCTTCGTAAACAATCTTCTTATTTACGATTAACATCT

TCTGGAGCTTTTCTTGAGCGAACACATTTCTATGGAAAAATAGAATATCGTATAGTAGTGCGCCGTAATT

ATTTTCAGAAGACCCTATGGTTTTTCAAGGATCCCTTCATGCATTATGTTCGATATCAAGGAAAAGCAAT

TCTGGTTTCAAAGGGGACTCATCTTCTGATGAAGAAATGGAAATGTCACCTTGTCAATTTCTGGCAATAT

TATTTTCACTTTTGGTCTCAACCGTACAGGATTCATATAAACCAATTATCAAACTGTTCTTTCTATTTTC

TAGGTTATCTTTCAAGTGTACTAATAAATCCTTCGGCGGTAAGGAATCAAATGCTAGAGAATTCATTTCT

AATAGATACTGTTATTAAAAAATTCGATACCAGAGTCCCAGTTATTCCTCTTATTAGATCATTGTCTAAA

GCTAAATTTTGTACCGTATCGGGGCATCCTATTAGTAAGCCGATCTGGACCGATTTATCAGATTGCGATA

TTATTGATCGATTTGGTCGGATATGTAGAAATCTTTCTCATTATCACAGTGGATCCTCAAAAAAACAGAG

TTTGTATCGAATAAAGTATATACTTCGGTTTTCGTGTGCTAGAACTTTGGCTCGTAAACATAAAAGTATG

GTACGCGCTTTTTTGCAAAGATTAGGTTCGGGATTATTAGAAGAATTTTTTATGGAAGAAGAACAAGTTG

TTTCTTTGATCTTCCCAAAAACAACTTCTTTTTCTTTACATGAATCACATATAGAACGTATTTGGTATTT

GGATATTATCCGTATCAATGACCTGGTGAATTATTCATAATTGGTTTGTTGACGTGATGAGACTTATGAA

TAGAATAGAAATGATCTATAAATGATCAAGAGAGAAAAAAATTCATGAATTTTCATTCTGAAATGCTCAT

TGCAGTAGTGTAGTGGTTGAATCAACTGAGTAGTCAA

>Serenoa_repens_HQ720326.1

AAATGTAAATGGAAGAATTACAAGGATATTTAGAAAGAGATAGATCTCTGCAACAACACTTTCTATATCC

GCTTCTCTTTAAGGAGTATATTTACACATTTCTTCATGATCGTGGTTTAAATGGTTCGATTTTTTACGAA

TCCACGGAAATTTTTGGTTATGACAATAAATCTAGTTCAGTACTTGTGAAACGTTCAATTATTCGAATGT

ATCGACAGAATTATTTGATTTATTCGGTTAATGATTCTAACCAAAATCGATTCGTTGGGCACAACAATTA

TTTTGATTTTCATTTTTATTCTCAGATGATATTGGAAGGTTTTGCAGTCATTGTGGAAATTCCATTCTTG

CTGCGATTGGTATCTTCCCTCGAAGAAAAAAAAATACCAAAATCTCAGAATTTGAATTTACGATCTATTC

ATTCAATATTTCCCTTTTTGGAGGACAAATTATCGCATTTAAATTATGTGTCAGATATACTAATACCTTA

TCCCATCCATCTGAAAATCTTGGTTCAAATCCTTCAATTCTGGATCCAAGATGTTCCTTCTTTACATTTA

TTGCGATTCTTTCTTCACGAATATCATAATTGGAATAGTCTTATTACTCCGAATAATTCTATTTTTCTTT

TTTCAAAAGAAAATAAAAGACTATTTCGGTTCCCATATAATTCTTATGTATCTGAATGCGAATTTGTATT

AGTTTTTCTTCGTAAACAATCTTCTTATTTACGATTAACATCTTCTGGAGCTTTTCTTGAGCGAACACAT

TTCTATGGAAAAATAGAATATCGTATAGTAGTGCGCCGTAATTATTTTCAGAAGACCCTATGGTTTTTCA

AGGATCCCTTCATGCATTATGTTCGATATCAAGGAAAAGCAATTCTGGTTTCAAAGGGGACTCATCTTCT

GATGAAGAAATGGAAATGTCACCTTGTCAATTTCTGGCAATATTATTTTCACTTTTGGTCTCAACCGTAC

AGGATTCATATAAACCAATTATCAAACTGTTCTTTCTATTTTCTAGGTTATCTTTCAAGTGTACTAATAA

ATCCTTCGGCGGTAAGGAATCAAATGCTAGAGAATTCATTTCTAATAGATACTGTTATTAAAAAATTCGA

TACCAGAGTCCCAGTTATTCCTCTTATTAGATCATTGTCTAAAGCTAAATTTTGTACCGTATCGGGGCAT

CCTATTAGTAAGCCGATCTGGACCGATTTATCAGATTGCGATATTATTGATCGATTTGGTCGGATATGTA

GAAATCTTTCTCATTATCACAGTGGATCCTCAAAAAAACAGAGTTTGTATCGAATAAAGTATATACTTCG

GTTTTCGTGTGCTAGAACTTTGGCTCGTAAACATAAAAGTATGGTACGCGCTTTTTTGCAAAGATTAGGT

TCGGGATTATTAGAAGAATTTTTTATGGAAGAAGAACAAGTTGTTTCTTTGATCTTCCCAAAAACAACTT

CTTTTTCTTTACATGAATCACATATAGAACGTATTTGGTATTTGGATATTATCCGTATCAATGACCTGGT

GAATTATTCATAATTGGTTTGTTGACGTGATGAGACTTATGAATAGAATAGAAATGATCTATAAATGATC

AAGAGAGAAAAAAATTCATGAATTTTCATTCTGAAATGCTCATTGCAGTAGTGTAGT

>Serenoa_repens_AM114585.1

GACCATATTGCACTATGTATCATTTGATAACCCAAAAAATGAAATAGGTCCCGCCTCTGGTTCAAGTAGA

AATGTAAATGGAAGAATTACAAGGATATTTAGAAAGAGATAGATCTCTGCAACAACACTTTCTATATCCG

CTTCTCTTTAAGGAGTATATTTACACATTTCTTCATGATCGTGGTTTAAATGGTTCGATTTTTTACGAAT

CCACGGAAATTTTTGGTTATGACAATAAATCTAGTTCAGTACTTGTGAAACGTTCAATTATTCGAATGTA

TCGACAGAATTATTTGATTTATTCGGTTAATGATTCTAACCAAAATCGATTCGTTGGGCACAACAATTAT

TTTGATTTTCATTTTTATTCTCAGATGATATTGGAAGGTTTTGCAGTCATTGTGGAAATTCCATTCTTGC

TGCGATTGGTATCTTCCCTCGAAGAAAAAAAAATACCAAAATCTCAGAATTTGAATTTACGATCTATTCA

TTCAATATTTCCCTTTTTGGAGGACAAATTATCGCATTTAAATTATGTGTCAGATATACTAATACCTTAT

CCCATCCATCTGAAAATCTTGGTTCAAATCCTTCAATTCTGGATCCAAGATGTTCCTTCTTTACATTTAT

TGCGATTCTTTCTTCACGAATATCATAATTGGAATAGTCTTATTACTCCGAATAATTCTATTTTTCTTTT

TTCAAAAGAAAATAAAAGACTATTTCGGTTCCCATATAATTCTTATGTATCTGAATGCGAATTTGTATTA

GTTTTTCTTCGTAAACAATCTTCTTATTTACGATTAACATCTTCTGGAGCTTTTCTTGAGCGAACACATT

TCTATGGAAAAATAGAATATCGTATAGTAGTGCGCCGTAATTATTTTCAGAAGACCCTATGGTTTTTCAA

GGATCCCTTCATGCATTATGTTCGATATCAAGGAAAAGCAATTCTGGTTTCAAAGGGGACTCATCTTCTG

ATGAAGAAATGGAAATGTCACCTTGTCAATTTCTGGCAATATTATTTTCACTTTTGGTCTCAACCGTACA

GGATTCATATAAACCAATTATCAAACTGTTCTTTCTATTTTCTAGGTTATCTTTCAAGTGTACTAATAAA

TCCTTCGGCGGTAAGGAATCAAATGCTAGAGAATTCATTTCTAATAGATACTGTTATTAAAAAATTCGAT

ACCAGAGTCCCAGTTATTCCTCTTATTAGATCATTGTCTAAAGCTAAATTTTGTACCGTATCGGGGCATC

CTATTAGTAAGCCGATCTGGACCGATTTATCAGATTGCGATATTATTGATCGATTTGGTCGGATATGTAG

AAATCTTTCTCATTATCACAGTGGATCCTCAAAAAAACAGAGTTTGTATCGAATAAAGTATATACTTCGG

TTTTCGTGTGCTAGAACTTTGGCTCGTAAACATAAAAGTATGGTACGCGCTTTTTTGCAAAGATTAGGTT

CGGGATTATTAGAAGAATTTTTTATGGAAGAAGAACAAGTTGTTTCTTTGATCTTCCCAAAAACAACTTC

TTTTTCTTTACATGAATCACATATAGAACGTATTTGGTATTTGGATATTATCCGTATCAATGACCTGGTG

AATTATTCATAATTGGTTTGTTGACGTGATGAGACTTATGAATAGAATAGAAATGATCTATAAATGATCA

AGAGAGAAAAAAATTCATGAATTTTCATTCTGAAATGCTCATTGCAGTAGTGTAGTGGTTGAATCAACTG

AGTAGTCAAAATTATTATACTTTCTTCTCGGGACCCAAGTTTTATATTATACATAGGTAAAGTC

>Socratea_exorrhiza_GQ982095.1

ATGTTCCTTCTTTACATTTATTGCGATTCTTTCTTCACGAATATCATAACTGGAATAGTCTTATTACTCC

GAATAATTCCATTTTTTCAAAAGAAAATAAAAGACTATTTCGGTTCCCATATAATTCTTATGTATCTGAA

TGCGAATTTGTATTAGTTTTTCTTCGTAAACAATCTTCTTATTTACGATTAACATCTTCTGGAGCTTTTC

TTGAGCGAAAACATTTCTATGGAAAAATCGAACATCTTATAGTAGTGCGCCGTAATTATTTTCAGAAGAC

CCTATGGTTCTTCAGGGATCCCTTCATGCATTATGTTCGATATCAAGGAAAAGCAATTCTGGTTTCAAAG

GGGACTCATCTTCTGATGAAGAAATGGAAATGTCACCTTGTCGATTTTTGGCAATATTATTTTCACTTTT

GGTCTCAACCGTACAGGATCCATATAAACCAATTATCAAACTGTTCTTTCTATTTTCTAGGTTATCTTTC

AAGTGTATTAATAAATCCTTCGGCGGTAAGGAATCAAATGCTAGAGAATTCATTTCTAATAGATACTGTT

ATTAAAAAATTCGATACCAGAGTCCCAGTTATTCCTCTTATTGGATCATTGTCTAAAGCTAAATTTTGTA

CCGTATCGGGGCATCCTAT

>Socratea_exorrhiza_AM114618.1

GACCATATTGCACTATGTATCATTTGATAACCAAAAAAATGAAATGGGTCCTGCCTCTGGTTCAAGTAGA

AATGTAAATGGAAGAATTACAAGGATATTTAGAAAAAGATAGATCTCGGCAACAACACTTTCTATATCCG

CTTCTCTTTAAGGAGTATATTTACACATTTGCTCATGATCGTGGTTTAAATTTAAATGGTTCGATTTTTT

ACGAATCCACGGAAATTTTTGGTTATGACAATAAATCTAGTTCAGTACTTGTGAAACGTTCGATTATTCG

AATGTATCAACAGAATTTTTTGATTTATTCGGTTAATGATTCTAACCAAAATCGATTCGTTGGGCACAAC

AATTATTTTTATTTTCATTTTTATTCTCAGATGATATTGGAAGGTTTTGCAGTCATTGTGGAAATTCCAT

TCTTGCTGCGATTAGTATCTTCCCTCGAAGAAAAAAAAATACCAAAATCTCAGAATTTGAATTTACGATC

TATTCATTCAATATTTCCCTTTTTGGAGGACAAATTATCGCATTTAAATTATGTGTCAGATATACTAATA

CCTTATCCCATCCATCTGAAAATCTTGGTTCAAATCCTTCAATGCTGGATCCAAGATGTTCCTTCTTTAC

ATTTATTGCGATTCTTTCTTCACGAATATCATAATTGGAATAGTCTTATTACTCCGAATAATTCTATTTT

TTCAAAAGAAAATAAAAGACTATTTCGGTTCCCATATAATTCTTATGTATCTGAATGCGAATTTGTATTA

GTTTTTCTTCGTAAACAATCTTCTTATTTACGATTAACATCTTCTGGAGCTTTTCTTGAGCGAAAACATT

TCTATGGAAAAATCGAACATCTTATAGTAGTGCGCCGTAATTATTTTCAGAAGACCCTATGGTTCTTCAA

GGATCCCTTCATGCATTATGTTCGATATCAAGGAAAAGCAATTCTGGTTTCAAAGGGGACTCATCTTCTG

ATGAAGAAATGGAAATGTCACCTTGTCAATTTCTGGCAATATTATTTTCACTTTTGGTCTCAACCGTACA

GGATTCATATAAACCAATTATCAAACTGTTCTTTCTATTTTCTAGGTTATCTTTCAAGTGTACTAATAAA

TCCTTCGGCGGTAAGGAATCAAATGCTAGAGAATTCATTTCTAATAGATACTGTTATTAAAAAATTCGAT

ACCAGAGTCCCAGTTATTCCTCTTATTGGATCATTGTCTAAAGCTAAATTTTGTACCGTATCGGGGCATC

CTATTAGTAAGCCGATCTGGACCGATTTATCAGATTGCGATATTATTGATCGATTTGGTCGGATATGTAG

AAATCTTTCTCATTATCACAGTGGATCCTCAAAAAAACAGAGTTTGTATCGAATAAAGTATATACTTCGA

TTTTCGTGTGCTAGAACTTTGGCTCGTAAACATAAAAGTATGGTACGCGCTTTTTTGCAAAGATTAGGTT

CGGGATTATTAGAAGAATTCTTTATGGAAGAAGAACAAGCTGTTTCTTTGATCTTCCCAAAAACAACTTC

TTTTTCTTTACATGAATCACATATAGAACGTATTTGGTATTTGGATATTATCCGTATCAATGACCTGGTG

AATTATTCATAATGGGTTTGGTGACGTGATGAGACTTATGAATAGAATAGAAATGATCTATAAATGATCA

AGAGAGAAAAAAATTCATGAATTTTCATTCTGAAATGCTCATTGCAGTAGTGTAGTGGTTGAATCAACTG

AGTAGTCAAAATTATTATACTTTCTTCTCGGGACCCAAGTTTTATATTATACATAGGTAAAGTC

>Syagrus_smithii_HQ265572.1

GATAGAAATGTAAATGGAAGAATTACAAGGATATTTAGAAAAAGATAGATCTCGGCAACAACACTTTCTA

TATCCGCTTCTCTTTAAGGAGTATATTTACACATTTGCTCATGATCGTGGTTTAAATGGTTCGATTTTTT

ACGAATCCACGGAAATTTTTGGTTATGACAATAAATCTAGTTCAGTACTTGTGAAACGTTCAATTATTCG

AATGTATCAACAGAATTATTTGATTTATTCGGTTAATGATTCTAACCAAAATCGATTCGTTGGGCGCAAC

AATTATTTTGATTTTCATTTTTATTCTCAGATGATATTGGAAGGTTTTGCAGTCATTGTGGAAATTCCAT

TCTTGCTGCGATTAGTATCTTCCCCCGAAGAAAAAAAAATACCAAAATCTCAGAATTTGAATTTACGATC

TATTCATTCAATATTTCCCTTTTTGGAGGACAAATTATCGCATTTAAATTATGTGTCAGATATACTAATA

CCTTATCCCATCCATCTGAAAATCTTGGTTCAAATCCTTCAATGCTGGATCCAAGATGTTCCTTCTTTAC

ATTTATTGCGATTCTTTCTTCACGAATATCATAATTGGAATAGTCTTATTACTCCGAATAATTCTATTTT

TTTTTCAAAAGAAAATAAAAGACTATTTCGGTTCCCATATAATTCTTATGTATCTGAATGCGAATTTGTA

TTAGTTTTTCTTCGTAAACAATCTTCTTATTTACGATTAACATCTTCTGGAGCTTTTCTTGAGCGAACAC

ATTTCTATGGAAAAATAGAACATCTTATAGTAGTGCGCCGTAATTATTTTCAGAAGACCCTATGGTTCTT

CAAGGATCCCTTCATGCATTATGTTCGATATCAAGGAAAAGCAATTCTGGTTTCAAAGGGGACTCATCTT

CTGATGAAGAAATGGAAATGTCATCTTGTCAATTTCTGGCAATATTATTTTCACTTTTGGTCTCAACCGT

ACAGGATCCATATAAACCAATTATCAAGCTGTTCTTTCTATTTTCTAGGTTATCTTTCAAGTGTACTAAT

AAATCCTTCGGCGGTAAGGAATCAAATGCTAGAGAATTCATTTCTAATAGATACTGTTATTAAAAAATTC

GATACCAGAGTCCCAGTTATTCCTCTTATTGGATCATTGTCTAAAGCTAAATTTTGTACCGTATCGGGGC

ATCCTATTAGTAAGCCGATCTGGACCAATTTATCAGATTGCGATATTATTGATCGATTTGGTCGGATATG

TAGAAATCTTTCTCATTATCACAGTGGATCCTCAAAAAAACAGAGTTTGTATCGAATAAAGTATATACTT

CGATTTTCGTGTGCTAGAACTTTGGCTCGTAAACATAAAAGYATGGKWCGCGCTTTTTTGCAAAGATTAG

GTTCGGGATTATTAGAAGAATTCTTTACGGAAGAAGAAGAAGTTGTTTCTTTGATCTTCCCAAAAACAAT

CTCTTTTTCTTTACATGAATCACATATAGAACGTATTTGGTATTTGGATATTATCCGTATCAATGACCTG

GTGAATTATTCATAATGGGTTTGGTGACGTGATGAGACTTATGAATAGAATAGAAATGATCTATAAATGA

TCAAGAGAGAAAAAAATTCATGAATTTTCATTCTGAAATGCTCATTGCAGTAGTGTAGTGGTTGAATCAA

CTGAGTAGTCAAAATTATTATACTTTCTTCTCGGGACCCAAGTTTTATATTATACATAGGTAAAGTCGTG

TGCAATGAAAAATGCAAGCACGGTTTGGGGAGGGATCTTTTTCCTCTATTCCAACAAAGAAAAGTTATCT

ACTCCATCCNAACTAGTTAA

>Syagrus_smithii_AM114638.1

GACCATATTGCACTATGTATCATTTGATAACCCAAAAAATGAAATGGGTCCTGTCTCTGGTTCAAGTAGA

AATGTAAATGGAAGAATTACAAGGATATTTAGAAAAAGATAGATCTCGGCAACAACACTTTCTATATCCG

CTTCTCTTTAAGGAGTATATTTACACATTTGCTCATGATCGTGGTTTAAATGGTTCGATTTTTTACGAAT

CCACGGAAATTTTTGGTTATGACAATAAATCTAGTTCAGTACTTGTGAAACGTTCAATTATTCGAATGTA

TCAACAGAATTATTTGATTTATTCGGTTAATGATTCTAACCAAAATCGATTCGTTGGGCGCAACAATTAT

TTTGATTTTCATTTTTATTCTCAGATGATATTGGAAGGTTTTGCAGTCATTGTGGAAATTCCATTCTTGC

TGCGATTAGTATCTTCCCCCGAAGAAAAAAAAATACCAAAATCTCAGAATTTGAATTTACGATCTATTCA

TTCAATATTTCCCTTTTTGGAGGACAAATTATCGCATTTAAATTATGTGTCAGATATACTAATACCTTAT

CCCATCCATCTGAAAATCTTGGTTCAAATCCTTCAATGCTGGATCCAAGATGTTCCTTCTTTACATTTAT

TGCGATTCTTTCTTCACGAATATCATAATTGGAATAGTCTTATTACTCCGAATAATTCTATTTTTTTTTC

AAAAGAAAATAAAAGACTATTTCGGTTCCCATATAATTCTTATGTATCTGAATGCGAATTTGTATTAGTT

TTTCTTCGTAAACAATCTTCTTATTTACGATTAACATCTTCTGGAGCTTTTCTTGAGCGAACACATTTCT

ATGGAAAAATAGAACATCTTATAGTAGTGCGCCGTAATTATTTTCAGAAGACCCTATGGTTCTTCAAGGA

TCCCTTCATGCATTATGTTCGATATCAAGGAAAAGCAATTCTGGTTTCAAAGGGGACTCATCTTCTGATG

AAGAAATGGAAATGTCATCTTGTCAATTTCTGGCAATATTATTTTCACTTTTGGTCTCAACCGTACAGGA

TCCATATAAACCAATTATCAAGCTGTTCTTTCTATTTTCTAGGTTATCTTTCAAGTGTACTAATAAATCC

TTCGGCGGTAAGGAATCAAATGCTAGAGAATTCATTTCTAATAGATACTGTTATTAAAAAATTCGATACC

AGAGTCCCAGTTATTCCTCTTATTGGATCATTGTCTAAAGCTAAATTTTGTACCGTATCGGGGCATCCTA

TTAGTAAGCCGATCTGGACCAATTTATCAGATTGCGATATTATTGATCGATTTGGTCGGATATGTAGAAA

TCTTTCTCATTATCACAGTGGATCCTCAAAAAAACAGAGTTTGTATCGAATAAAGTATATACTTCGATTT

TCGTGTGCTAGAACTTTGGCTCGTAAACATAAAAGTATGGTACGCGCTTTTTTGCAAAGATTAGGTTCGG

GATTATTAGAAGAATTCTTTACGGAAGAAGAAGAAGTTGTTTCTTTGATCTTCCCAAAAACAATCTCTTT

TTCTTTACATGAATCACATATAGAACGTATTTGGTATTTGGATATTATCCGTATCAATGACCTGGTGAAT

TATTCATAATGGGTTTGGTGACGTGATGAGACTTATGAATAGAATAGAAATGATCTATAAATGATCAAGA

GAGAAAAAAATTCATGAATTTTCATTCTGAAATGCTCATTGCAGTAGTGTAGTGGTTGAATCAACTGAGT

AGTCAAAATTATTATACTTTCTTCTCGGGACCCAAGTTTTATATTATACATAGGTAAAGTC

>Synechanthus_fibrosus_DQ178700.1

CGTTCTGACCATATTGCACTATGTATCATTTGATAACCCCAAAAATGAAATGGGTCCTGCCTCTGGTTCA

AGTAGAAATGTAAATGGAAGAATTACAAGGATATTTAGAAAAAGATAGATCTCGGCAACAACACTTTCTA

TATCCGCTTCTCTTTAAGGAGTATATTTACACATTTGCTCATGATCGTGGTTTAAATGGTTCGATTTTTT

ACGAATCCACGGAAATTTTTGGTTATGACAATAAATCTAGTTCAGTACTTGTGAAACGTTCAATTATTCG

AATGTATCAACAGAATTATTTGATTTATTCGGTTAATGATTCTAACCAAAATCGATTCGTTGGGCACAAC

AATTTTTTTTATTTTCATTTTTATTCTCAGATGATATTGGAAGGTTTTGCAGTCATTGTGGAAATTCCAT

TCTTACTGCGATTAGTATCTTCTCTCGAAGAAAAAAAAATACCAAAATCTCAGAATTTGAATTTACGATC

TATTCATTCAATATTTCCCTTTTTGGAGGACAAATTATCGCATTTAAATTATGTGTCAGATATACTAATA

CCTTATCCCATCCATCTGAAAATCTTGGTTCAAATCCTTCAATGCTGGATCCAAGATGTTCCTTCTTTAC

ATTTATTGCGATTCTTTCTTCACGAATATCATAATTGGAATAGTCTTATTATTCCGAATAATTCTATTTT

TTTTTTTTCAAAAGAAAATAAAAGACTATTTCGGTTCCCATATAATTCTTATGTATCTGAATGCGAATTT

GTATTAGTTTTTCTTCGTAAACAATCTTCTTATTTACGATTAACATCTTCTGGAGCTTTTCTTGAGCGAA

CACATTTCTATGGAAAAATAGAACATCTTATAGTAGTGCGCCGTAATTATTTTCAGAGGACCCTATGGTT

CTTCAAGGATCCCTTCATGCATTATGTTCGATATCAAGGAAAAGCAATTCTGGTTTCAAAGGGGACTCAT

CTTCTGATGAAGAAATGGAAATGTCACCTTGTCAATTTCTGGCAATATTATTTTAACTTTTGGTCTCAAC

CGTACAGGATCCATATAAACCAATTATCAAGCTGTTCTTTCCATTTTCTAGGTTATCTTTCAAGTGTACT

AATAAATCCTTCGGCGGTAAGGAATCAAATGCTAGAGAATTCATTTCTAATAGATACTGTTATTAAAAAA

TTCGATACCAGAGTCCCAGTTATTCCTCTTATTGGATCATTGTCTAAAGCTAAATTTTGTACCGTATCGG

GGCATCCTATTAGTAAGCCGATCTGGACCAATTTATCAGATTGTGATATTATTGATCGATTTGGTCGGAT

ATGTAGAAATCTTTCTCATTATCACAGTGGATCCTCGAAAAAACAGAGTTTGTATCGAATAAAGTATATA

CTTCGATTTTCGTGTGCTAGAACTTTGGCTCGTAAACATAAAAGTACGGTACGCGCTTTTTTGCAAAGAT

TAGGTTCGGGATTATTAGAAGAATTCTTTACGGAAGAAGAACAAGTTGTTTCTTTGATCTTCCCCAAAAC

AACTTCTTTTTCTTTACATGAATCACATATAGAACGTATTTGGTATTTGGATATTATCCGTATCAATGAC

CTGGTGAATTATTCATAATGGGTTTGTTTGGTTACGTGATGAGACTTATGAATAGTCTGGAAATGATCTA

TAAATGATCAAGAGAGAAAAAAATTCATGAATTTTCATTCTGAAATGCTCATTGCAGTAGTGTAGTGGTT

GAATCAACTGAGTAGTCAAAATTATTATACTTTCTTCTCGGGACCCAAGTTTTATATTATACATAGGTAA

AGTCGTGTGCAATG

>Synechanthus_warscewiczianus_AM114622.1

GACCATATTGCACTATGTATCATTTGATAACCCCAAAAATTAAATGGGTCCTGCCTCTGGTTCAAGTAGA

AATGTAAATGGAAGAATTACAAGGATATTTAGAAAAAGATAGATCTCGGCAACAACACTTTCTATATCCG

CTTCTCTTTAAGGAGTATATTTACACATTTGCTCATGATCGTGGTTTAAATGGTTCGATTTTTTACGAAT

CCACGGAAATTTTTGGTTATGACAATAAATCTAGTTCAGTACTTGTGAAACGTTCAATTATTCGAATGTA

TCAACAGAATTATTTGATTTATTCGGTTAATGATTCTAACCAAAATCGATTCGTTGGGCACAACAATTAT

TTTTATTTTCATTTTTATTCTCAGATGATATTGGAAGGTTTTGCAGTCATTGTGGAAATTCCATTCTTGC

TGCGATTAGTATCTTCTCTCGAAGAAAAAAAAATACCAAAATCTCAGAATTTGAATTTACGATCTATTCA

TTCAATATTTCCCTTTTTGGAGGACAAATTATCGCATTTAAATTATGTGTCAGATATACTAATACCTTAT

CCCATCCATCTGAAAATCTTGGTTCAAATCCTTCAATGCTGGATCCAAGATGTTCCTTCTTTACATTTAT

TGCGATTCTTTCTTCACGAATATCATAATTGGAATAGTCTTATTATTCCGAATAATTCTATTTTTTTTTT

TTCAAAAGAAAATAAAAGACTATTTAGGTTCCCATATAATTCTTATGTATCTGAATGCGAATTTGTATTA

GTTTTTCTTCGTAAACAATCTTCTTATTTACGATTAACATCTTCTGGAGCTTTTCTTGAGCGAACACATT

TCTATGGAAAAATAGAACATCTTATAGTAGTGCGCCGTAATTATTTTCAGAGGACCCTATGGTTCTTCAA

GGATCCCTTCATGCATTATGTTCGATATCAAGGAAAAGCAATTCTGGTTTCAAAGGGGACTCATCTTCTG

ATGAAGAAATGGAAATGTCACCTTGTCAATTTCTGGCAATATTATTTTCACTTTTGGTCTCAACCGTACA

GGATCCATATAAACCAATTATCAAGCTGTTCTTTCCATTTTCTAGGTTATCTTTCAAGTGTACTAATAAA

TCCTTCGGCGGTAAGGAATCAAATGCTAGAGAATTCATTTCTAATAGATACTGTTATTAAAAAATTCGAT

ACCAGAGTCCCAGTTATTCCTCTTATTGGATCATTGTCTAAAGCTAAATTTTGTACCGTATCGGGGCATC

CTATTAGTAAGCCGATCTGGACCAATTTATCAGATTGTGATATTATTGATCGATTTGGTCGGATATGTAG

AAATCTTTCTCATTATCACAGTGGATCCTCAAAAAAACAGAGTTTGTATCGAATAAAGTATATACTTCGA

TTTTCGTGTGCTAGAACTTTGGCTCGTAAACATAAAAGTACGGTACGCGCTTTTTTGCAAAGATTAGGTT

CGGGATTATTAGAAGAATTCTTTACGGAAGAAGAACAAGTTGTTTCTTTGATCTTCCCCAAAACAACTTC

TTTTTCTTTACATGAATCACATATAGAACGTATTTGGTATTTGGATATTATCCGTATCAATGACCTGGTG

AATTATTCATAATGGGTTTGTTTGGTGGCGTGATGAGACTTATGAATAGTCTGGAAATGATCTATAAATG

ATCAAGAGAGAAAAAAATTCATGAATTTTCATTCTGAAATGCTCATTGCAGTAGTGTAGTGGTTGAATCA

ACTGAGTAGTCAAAATTATTATACTTTCTTCTCGGGACCCAAGTTTTATATTATACATAGGTAAAGTC

>Thrinax_morrisii_AM114560.1

GACCATATTGCACTATGTATCATTTGATAACCCCAAAAATGAAATAGGTCCCGCCTCTGGTTCAAGTAGA

AATGGAAATGGAAGAATTACAAGGATATTTAGAAAAAGATAGATCTCGGCAACAACACTTTCTATATCCG

CTTCTCTTTAAGGAGTATATTTACACATTTGCTCATGATCGTGGTTTAAATGGTTCGATTTTTTACGAAT

CCACGGAAATTTTTGGTTATGACAATAAATCTAGTTCAGTACTTGTGAAACGTTCAATTATTCGAATGTA

TCAACAGAATTATTTGATTTATTCGGTTAATGATTCTAACCAAAATCGATTCGTTGGGCACAACAATTAT

TTTTATTTTCATTTTTATTCTCAGATGATATTGAAAGGTTTTGCAGTCATTGTGGAAATTCCATTCTTGC

TGCGATTAGTATCTTCCCTCGAAGAAAAAAAAATACCAAAATCTCAAAATTTGAATTTACGATCTATTCA

TTCAATATTTCCCTTTTTGGAGGACAAATTATCACATTTAAATTATGTGTCAGATATACTAATACCTTAT

CCCATACATCTGAAAATCTTGGTTCAAATCCTTCAATTCTGGATCCAAGATGTTCCTTCTTTACATTTAT

TGCGATTCTTTCTTCACGAATATCATAATTGGAATAGTCTTATTACTCCGAATAATTCTATTTTTCCTTT

TTTACTTTTTTCAAAAGAAAATAAAAGACTATTTCGGTTCCCATATAATTCTTATGTATCTGAATGCGAA

TTTTTATTAGTTTTTCTTCGTAAACAATCTTCTTATTTACGATTAACATCTTCTGGAGCTTTTCTTGAGC

GAACACATTTCTATGGAAAAATAGAACATCTTATAGTAGTGCGCCGTAATTATTTTCAGAAGACCCTATG

GTTCTTCAAGGATCCCTTCATGCATTATGTTCGATATCAAGGAAAAGCAATTCTGGTTTCAAAGGGGACT

CATCTTCTGATGAAGAAATGGAAATGTCACCTTGTCAATTTCTGGCAATATTATTTTCACTTTTGGTCTC

AACCGTACAGGATTCATATAAAGCAATTATCAAACTGTTCTTTCTATTTTTTAGGTTATCTTTCAAGTGT

ACTAATAAATCCTTCGGCGGTAAGGAATCAAATGCTAGAGAATTCATTTCTAATAGATACTCTTATTAAA

AAATTCGATACCAGAGTCCCAGTTATTCCTCTTATTGGATCATTGTCTAAAGCTAAATTTTGTACCGTAT

CGGGGCATCCTATTAGTAAGCCGATCTGGACCGATTTATCAGATTGCGATATTATTGATCGATTTGGTCG

GATATGTAGAAATCTTTCTCATTATCACAGTGGATCCTCAAAAAAACAGAGTTTGTATCGAATAAAGTAT

ATACTTCGATTTTCGTGTGCTAGAACTTTGGCTCGTAAACATAAAAGTACGGTACGCGCTTTTTTGCAAA

GATTAGGTTCAGGATTATTAGAAGAATTTTTTATGGAAGAAGAAGAAGTTGTTTCTTTGATCTTCCCAAA

AACAACTTCTTTTTCTTTACATGAATCACATATAGAACGTATTTGGTATTTGGATATTATCTGTATCAAT

GACCTGGTGAATTATTCATAATTGGTTTGGTGACGTGATGAGACTTATGAATAGAAAATAGAATAGAAAT

GATCTATAAATGATCAAGAGAGAAAAAAATTCATGAATTTTCATTCTGAAATGCTCATTGCAGTAGTGTA

GTGGTTGAATCAACTGAGTAGTCAAAATTATTATACTTTCTTCTCGGGACCCAAGTTTTATATTATACAT

AGGTAAAGTC

>Thrinax_radiata_AM114561.1

GACCATATTGCACTATGTATCATTTGATAACCCCAAAAATGAAATAGGTCCCGCCTCTGGTTCAAGTAGA

AATGGAAATGGAAGAATTACAAGGATATTTAGAAAAAGATAGATCTCGGCAACAACACTTTCTATATCCG

CTTCTCTTTAAGGAGTATATTTACACATTTGCTCATGATCGTGGTTTAAATGGTTCGATTTTTTACGAAT

CCACGGAAATTTTTGGTTATGACAATAAATCTAGTTCAGTACTTGTGAAACGTTCAATTATTCGAATGTA

TCAACAGAATTATTTGATTTATTCGGTTAATGATTCTAACCAAAATCGATTCGTTGGGCACAACAATTAT

TTTGATTTTCATTTTTATTCTCAGATGATATTGGAAGGTTTTGCAGTCATTGTGGAAATTCCATTCTTGC

TGCGATTAGTATCTTCCCTCGAAGAAAAAAAAATACCAAAATCTCAAAATTTGAATTTACGATCTATTCA

TTCAATATTTCCCTTTTTGGAGGACAAATTATCGCATTTAAATTATGTGTCAGATATACTAATACCTTAT

CCCATACATCTGAAAATCTTGGTTCAAATCCTTCAATTCTGGATCCAAGATGTTCCTTCTTTACATTTAT

TGCGATTCTTTCTTCACGAATATCATAATTGGAATAGTCTTATTACTCCGAATAATTCTATTTTTCCTTT

TTTACTTTTTTCAAAAGAAAATAAAAGACTATTTCGGTTCCCATATAATTCTTATGTATCTGAATGCGAA

TTTTTATTAGTTTTTCTTCGTAAACAATCTTCTTATTTACGATTAACATCTTCTGGAGCTTTTCTTGAGC

GAACACATTTCTATGGAAAAATAGAACATCTTATAGTAGTGCGCCGTAATTATTTTCAGAAGACCCTATG

GTTCTTCAAGGATCCCTTCATGCATTATGTTCGATATCAAGGAAAAGCAATTCTGGTTTCAAAGGGGACT

CATCTTCTGATGAAGAAATGGAAATGTCACCTTGTCAATTTCTGGCAATATTATTTTCACTTTTGGTCTC

AACCGTACAGGATCCATATAAAGCAATTATCAAACCGTTCTTTCTATTTTTTAGGTTATCTTTCAAGTGT

ACTAATAAATCCTTCGGCGGTAAGGAATCAAATGCTAGAGAATTCATTTCTAATAGATACTCTTATTAAA

AAATTCGATACCAGAGTCCCAGTTATTCCTCTTATTGGATCATTGTCTAAAGCTAAATTTTGTACCGTAT

CGGGGCATCCTATTAGTAAGCCGATCTGGACCGATTTATCAGATTGCGATATTATTGATCGATTTGGTCG

GATATGTAGAAATCTTTCTCATTATCACAGTGGATCCTCAAAAAAACAGAGTTTGTATCGAATAAAGTAT

ATACTTCGATTTTCGTGTGCTAGAACTTTGGCTCGTAAACATAAAAGTACGGTACGCGCTTTTTTGCAAA

GATTAGGTTCAGGATTATTAGAAGAATTTTTTATGGAAGAAGAAGAAGTTGTTTCTTTGATCTTCCCAAA

AACAACTTCTTTTTCTTTACATGAATCACATATAGAACGTATTTGGTATTTGGATATTATCCGTATCAAT

GACCTGGTGAATTATTCATAATTGGTTTGGTGACGTGATGAGACTTATGAATAGAAAATAGAATAGAAAT

GATCTATAAATGATCAAGAGAGAAAAAAATTCATGAATTTTCATTCTGAAATGCTCATTGCAGTAGTGTA

GTGGTTGAATCAACTGAGTAGTCAAAATTATTATACTTTCTTCTCGGGACCCAAGTTTTATATTATACAT

AGGTAAAGTC

>Trithrinax_campestris_AM114556.1

GACCATATTGCACTATGTATCATTTGATAACTCCAAAAATGAAATAGGTCCCGCCTCTGGTTCAAGTAGA

AATGGAAATGGAAATGGAAGAATTACAAGGATATTTAGAAAAAGATAGATCTCGGCAACAACACTTTCTA

TATCCGCTTCTCTTTAAGGAGTATATTTACACATTTGCTCATGATCGTGGTTTAAATGGTTCGACTTTTT

ACGAATCCACGGAAATTTTTGGTTATGACAATAAATCTAGTTCAGTACTTGTGAAACGTTCAATTATTCG

AATGTATCAACAGAATTATTTGATTTATTCGGTTAATGATTCTAACCAAAATCGATTCGTTGGGCACAAC

AATTATTTTTATTTTCATTTTTATTCTCAGATGATATTGGAAGGTTTTGCAGTCATTGTGGAAATTCCAT

TCTTGCTGCGATTAGTATCTTCCCTCGAAGAAAAAAAAATACCAAAATCTCAAAATTTGAATTTACGATC

TATTCATTCAATATTTCCCTTTTTGGAGGACAAATTATCGCATTTAAATTATGTGTCAGATATACTAATA

CCTTATCCCATACATCTGAAAATCTTGGTTCAAATCCTTCAATTCTGGATCCAAGATGTTCCTTCTTTAC

ATTTATTGCGATTCTTTCTTCACGAATATCATAATTGGAATAGTCTTATTACTCCGAATAATTCTATTTT

TCCTTTTTTACTTTTTTCAAAAGAAAATAAAAGACTATTTCGGTTCCCATATAATTCTTATGTATCTGAA

TGCGAATTTTTATTAGTTTTTCTTCGTAAACAATCTTCTTATTTACGATTAACATCTTCTGGAGCTTTTC

TTGAGCGAACACATTTCTATGGAAAAATAGAACATCTTATAGTAGTGCACCGTAATTATTTTCAGAAGAC

CCTATGGTTCTTCAAGGATCCCTTCATGCATTATGTTCGATATCAAGGAAAAGCAATTCTGGTTTCAAAG

GGGACTCATCTTCTGATGAAGAAATGGAAATGTCACCTTGTCAATTTCTGGCAATATTATTTTCACTTTT

GGTCTCAACCGTACAGGATCCATATAAAGCAATTATCAAACTGTTCTTTCTATTTTTTAGGTTATCTTTC

AAGTGTACTAATAAATCCTTCGGCGGTAAGGAATCAAATGCTAGAGAATTCATTTCTAATAGATACTCTT

ATTAAAAAATTCGATACCAGAGTCCCAGTTATTCCTCTTATTGGATCATTGTCTAAAGCTAAATTTTGTA

CCGTATCGGGGCATTCCATTAGTAAGCCGATCTGGACCGATTTATCAGATTGCGATATTATTGATCGATT

TGGTCGGATATGTAGAAATCTTTCTCATTATCACAGTGGATCCTCAAAAAAACAGAGTTTGTATCGAATA

AAGTATATACTTCGATTTTCGTGTGCTAGAACTTTGGCTCGTAAACATAAAAGTACGGTACGCGCTTTTT

TGCAAAGATTAGGTTCAGGATTATTAGAAGAATTTTTTATGGAAGAAGAAGAAGTTGTTTCTTTGATCTT

CCCAAAAACAACTTCTTTTTCTTTACATGAATCACATATAGAACGTATTTGGTATTTGGATATTATCCGT

ATCAATGACCTGGTGAATTATTCATAATTGCTTTGGTGACGTGATGAGACTTATGAATAGAAAATAGAAT

AGAAATAATCTATAAATGATCAAGAGAGAAAAAAATTCATGAATTTTCATTCTGAAATGCTCATTGCAGT

AGTGTAGTGGTTGAATCAACTGAGTAGTCAAAATTATTATACTTTCTTCTCGGGACCCAAGTTTTATATT

ATACATAGGTAAAGTC

>Washingtonia_robusta_HQ720329.1

AAATGTAAATGGAAGAATTACAAGGATATTTAGAAAGAGATAGATCTCTGCAACAACACTTTCTATATCC

GCTTCTCTTTAAGGAGTATATTTACACATTTCTTCATGATCGTGGTTTAAATGGTTCGATTTTTTACGAA

TCCACGGAAATTTTTGGTTATGACAATAAATCTAGTTCAGTACTTGTGAAACGTTCAATTATTCGAATGT

ATCAACAGAATTATTTGATTTATTCGGTTAATGATTCTAACCAAAATCGATTCGTTGGGCACAACAATTA

TTTTTATTTTCATTTTTATTCTCAGATGATATTGGAAGGTTTTGCAGTCATTGTGGAAATTCCATTCTTG

CTGCGATTAGTATCTTCCCTCGAAGAAAAAAAAATACCAAAATCTCAGAATTTGAATTTACGATCTATTC

ATTCAATATTTCCCTTTTTGGAGGACAAATTATCGCATTTAAATTATGTGTCAGATATACTAATACCTTA

TCCCATCCATCTGAAAATCTTGGTTCAAATCCTTCAATTCTGGATCCAAGATGTTCCTTCTTTACATTTA

TTGCGATTCTTTCTTCACGAATATCATAATTGGAATAGTCTTATTACTCCGAATAATTCTATTTTTCTTT

TTTCAAAAGAAAATAAAAGACTATTTCGGTTCCCATATAATTCTTATGTATCTGAATGCGAATTTGTATT

AGTTTTTCTTCGTAAACAATCTTCTTATTTACGATTAACATCTTCTGGAGCTTTTCTTGAGCGAACACAT

TTCTATGGAAAAATAGAATATCGTATAGTAGTGCGCCGTAATTATTTTCAGAAGACCCTATGGTTTTTCA

AGGATCCCTTCATGCATTATGTTCGATATCAAGGAAAAGCAATTCTGGTTTCAAAGGGGACTCATCTTCT

GATGAAGAAATGGAAATGTCACCTTGTCAATTTCTGGCAATATTATTTTCACTTTTGGTCTCAACCGTAC

AGGATTCATATAAACCAATTATCAAACTGTTCTTTCTATTTTCTAGGTTATCTTTCAAGTGTACTAATAA

ATCCTTCGGCGGTAAGGAATCAAATGCTAGAGAATTCATTTCTAATAGATACTGTTATTAAAAAATTCGA

TACCAGAGTCCCAGTTATTCCTCTTATTAGATCATTGTCTAAAGCTAAATTTTGTACCGTATCGGGGCAT

CCTATTAGTAAGCCAATCTGGACCGATTTATCAGATTGCGATATTATTGATCGATTTGGTCGGATATGTA

GAAATCTTTCTCATTATCACAGTGGATCCTCAAAAAAACAGAGTTTGTATCGAATAAAGTATATACTTCG

ATTTTCGTGTGCTAGAACTTTGGCTCGTAAACATAAAAGTATGGTACGCGCTTTTTTGCAAAGATTAGGT

TCGGGATTATTAGAAGAATTTTTTATGGAAGAAGAACAAGTTGTTTCTTTGATCTTCCCAAAAACAACTT

CTTTTTCTTTACATGAATCACATATAGAACGTATTTGGTATTTGGATATTATCCGTATCAATGACCTGGT

GAATTATTCATAATTGGTTTGTTGACGTGATGAGACTTATGAATAGAATAGAAATGATCTATAAATGATC

AAGAGAGAAAAAAATTCATGAATTTTCATTCTGAAATGCTCATTGCAGTAGTGTAGTGGTTGAATCAACT

GAGTAGTCAAAATTA

>Washingtonia_filifera_HQ720327.1

AGAATTACAAGGATATTTAGAAAGAGATAGATCTCTGCAACAACACTTTCTATATCCGCTTCTCTTTAAG

GAGTATATTTACACATTTCTTCATGATCGTGGTTTAAATGGTTCGATTTTTTACGAATCCACGGAAATTT

TTGGTTATGACAATAAATCTAGTTCAGTACTTGTGAAACGTTCAATTATTCGAATGTATCAACAGAATTA

TTTGATTTATTCGGTTAATGATTCTAACCAAAATCGATTCGTTGGGCACAACAATTATTTTTATTTTCAT

TTTTATTCTCAGATGATATTGGAAGGTTTTGCAGTCATTGTGGAAATTCCATTCTTGCTGCGATTAGTAT

CTTCCCTCGAAGAAAAAAAAATACCAAAATCTCAGAATTTGAATTTACGATCTATTCATTCAATATTTCC

CTTTTTGGAGGACAAATTATCGCATTTAAATTATGTGTCAGATATACTAATACCTTATCCCATCCATCTG

AAAATCTTGGTTCAAATCCTTCAATTCTGGATCCAAGATGTTCCTTCTTTACATTTATTGCGATTCTTTC

TTCACGAATATCATAATTGGAATAGTCTTATTACTCCGAATAATTCTATTTTTCTTTTTTCAAAAGAAAA

TAAAAGACTATTTCGGTTCCCATATAATTCTTATGTATCTGAATGCGAATTTGTATTAGTTTTTCTTCGT

AAACAATCTTCTTATTTACGATTAACATCTTCTGGAGCTTTTCTTGAGCGAACACATTTCTATGGAAAAA

TAGAATATCGTATAGTAGTGCGCCGTAATTATTTTCAGAGGACCCTATGGTTTTTCAAGGATCCCTTCAT

GCATTATGTTCGATATCAAGGAAAAGCAATTCTGGTTTCAAAGGGGACTCATCTTCTGATGAAGAAATGG

AAATGTCACCTTGTCAATTTCTGGCAATATTATTTTCACTTTTGGTCTCAACCGTACAGGATTCATATAA

ACCAATTATCAAACTGTTCTTTCTATTTTCTAGGTTATCTTTCAAGTGTACTAATAAATCCTTCGGCGGT

AAGGAATCAAATGCTAGAGAATTCATTTCTAATAGATACTGTTATTAAAAAATTCGATACCAGAGTCCCA

GTTATTCCTCTTATTAGATCATTGTCTAAAGCTAAATTTTGTACCGTATCGGGGCATCCTATTAGTAAGC

CAATCTGGACCGATTTATCAGATTGCGATATTATTGATCGATTTGGTCGGATATGTAGAAATCTTTCTCA

TTATCACAGTGGATCCTCAAAAAAACAGAGTTTGTATCGAATAAAGTATATACTTCGATTTTCGTGTGCT

AGAACTTTGGCTCGTAAACATAAAAGTATGGTACGCGCTTTTTTGCAAAGATTAGGTTCGGGATTATTAG

AAGAATTTTTTATGGAAGAAGAACAAGTTGTTTCTTTGATCTTCCCAAAAACAACTTCTTTTTCTTTACA

TGAATCACATATAGAACGTATTTGGTATTTGGATATTATCCGTATCAATGACCTGGTGAATTATTCATAA

TTGGTTTGTTGACGTGATGAGACTTATGAATAGAATAGAAATGATCTATAAATGAAATGATCAAGAGAGA

AAAAAATTCATGAATTTTCATTCTGAAATGCTCATTGCAGTAGTGTAGTGGTTGAATCAACTGAGTAGTC

AAAATTATTATACTT

>Washingtonia_filifera_HQ720328.1

GTTCAAGTAGAAATGTAAATGGAAGAATTACAAGGATATTTAGAAAGAGATAGATCTCTGCAACAACACT

TTCTATATCCGCTTCTCTTTAAGGAGTATATTTACACATTTCTTCATGATCGTGGTTTAAATGGTTCGAT

TTTTTACGAATCCACGGAAATTTTTGGTTATGACAATAAATCTAGTTCAGTACTTGTGAAACGTTCAATT

ATTCGAATGTATCAACAGAATTATTTGATTTATTCGGTTAATGATTCTAACCAAAATCGATTCGTTGGGC

ACAACAATTATTTTTATTTTCATTTTTATTCTCAGATGATATTGGAAGGTTTTGCAGTCATTGTGGAAAT

TCCATTCTTGCTGCGATTAGTATCTTCCCTCGAAGAAAAAAAAATACCAAAATCTCAGAATTTGAATTTA

CGATCTATTCATTCAATATTTCCCTTTTTGGAGGACAAATTATCGCATTTAAATTATGTGTCAGATATAC

TAATACCTTATCCCATCCATCTGAAAATCTTGGTTCAAATCCTTCAATTCTGGATCCAAGATGTTCCTTC

TTTACATTTATTGCGATTCTTTCTTCACGAATATCATAATTGGAATAGTCTTATTACTCCGAATAATTCT

ATTTTTCTTTTTTCAAAAGAAAATAAAAGACTATTTCGGTTCCCATATAATTCTTATGTATCTGAATGCG

AATTTGTATTAGTTTTTCTTCGTAAACAATCTTCTTATTTACGATTAACATCTTCTGGAGCTTTTCTTGA

GCGAACACATTTCTATGGAAAAATAGAATATCGTATAGTAGTGCGCCGTAATTATTTTCAGAAGACCCTA

TGGTTTTTCAAGGATCCCTTCATGCATTATGTTCGATATCAAGGAAAAGCAATTCTGGTTTCAAAGGGGA

CTCATCTTCTGATGAAGAAATGGAAATGTCACCTTGTCAATTTCTGGCAATATTATTTTCACTTTTGGTC

TCAACCGTACAGGATTCATATAAACCAATTATCAAACTGTTCTTTCTATTTTCTAGGTTATCTTTCAAGT

GTACTAATAAATCCTTCGGCGGTAAGGAATCAAATGCTAGAGAATTCATTTCTAATAGATACTGTTATTA

AAAAATTCGATACCAGAGTCCCAGTTATTCCTCTTATTAGATCATTGTCTAAAGCTAAATTTTGTACCGT

ATCGGGGCATCCTATTAGTAAGCCAATCTGGACCGATTTATCAGATTGCGATATTATTGATCGATTTGGT

CGGATATGTAGAAATCTTTCTCATTATCACAGTGGATCCTCAAAAAAACAGAGTTTGTATCGAATAAAGT

ATATACTTCGATTTTCGTGTGCTAGAACTTTGGCTCGTAAACATAAAAGTATGGTACGCGCTTTTTTGCA

AAGATTAGGTTCGGGATTATTAGAAGAATTTTTTATGGAAGAAGAACAAGTTGTTTCTTTGATCTTCCCA

AAAACAACTTCTTTTTCTTTACATGAATCACATATAGAACGTATTTGGTATTTGGATATTATCCGTATCA

ATGACCTGGTGAATTATTCATAATTGGTTTGTTGACGTGATGAGACTTATGAATAGAATAGAAATGATCT

ATAAATGATCAAGAGAGAAAAAAATTCATGAATTTTCATTCTGAAATGCTCATTGCAGTAGTGTAGTGGT

TGAATCAACTGAGTAGTCAAAATTATTATACTTTCTTCTC

>Washingtonia_robusta_GU135111.1

ATTTAAATTATGTGTCAGATATACTAATACCTTATCCCATCCATCTGAAAATCTTGGTTCAAATCCTTCA

ATTCTGGATCCAAGATGTTCCTTCTTTACATTTATTGCGATTCTTTCTTCACGAATATCATAATTGGAAT

AGTCTTATTACTCCGAATAATTCTATTTTTCTTTTTTCAAAAGAAAATAAAAGACTATTTCGGTTCCCAT

ATAATTCTTATGTATCTGAATGCGAATTTGTATTAGTTTTTCTTCGTAAACAATCTTCTTATTTACGATT

AACATCTTCTGGAGCTTTTCTTGAGCGAACACATTTCTATGGAAAAATAGAATATCGTATAGTAGTGCGC

CGTAATTATTTTCAGAAGACCCTATGGTTTTTCAAGGATCCCTTCATGCATTATGTTCGATATCAAGGAA

AAGCAATTCTGGTTTCAAAGGGGACTCATCTTCTGATGAAGAAATGGAAATGTCACCTTGTCAATTTCTG

GCAATATTATTTTCACTTTTGGTCTCAACCGTACAGGATTCATATAAACCAATTATCAAACTGTTCTTTC

TATTTTCTAGGTTATCTTTCAAGTGTACTAATAAATCCTTCGGCGGTAAGGAATCAAATGCTAGAGAATT

CATTTCTAATAGATACTGTTATTAAAAAATTCGATACCAGAGTCCCAGTTATTCCTCTTATTAGATCATT

GTCTAAAGCTAAATTTTGTACCGTATCGGGGCATCCTATTAGTAAGCCAATCTGGACCGATTTATCAGAT

TGCGATATTATTGATCGATTTGGTCGGATATGTAGAAATCTTTCTCATTAT

>Washingtonia_robusta_AM114586.1

GACCATATTGCACTATGTATCATTTGATAACCCCAAAAATGAAATAGGTCCCGCCTCTGGTTCAAGTAGA

AATGTAAATGGAAGAATTACAAGGATATTTAGAAAGAGATAGATCTCTGCAACAACACTTTCTATATCCG

CTTCTCTTTAAGGAGTATATTTACACATTTCTTCATGATCGTGGTTTAAATGGTTCGATTTTTTACGAAT

CCACGGAAATTTTTGGTTATGACAATAAATCTAGTTCAGTACTTGTGAAACGTTCAATTATTCGAATGTA

TCAACAGAATTATTTGATTTATTCGGTTAATGATTCTAACCAAAATCGATTCGTTGGGCACAACAATTAT

TTTTATTTTCATTTTTATTCTCAGATGATATTGGAAGGTTTTGCAGTCATTGTGGAAATTCCATTCTTGC

TGCGATTAGTATCTTCCCTCGAAGAAAAAAAAATACCAAAATCTCAGAATTTGAATTTACGATCTATTCA

TTCAATATTTCCCTTTTTGGAGGACAAATTATCGCATTTAAATTATGTGTCAGATATACTAATACCTTAT

CCCATCCATCTGAAAATCTTGGTTCAAATCCTTCAATTCTGGATCCAAGATGTTCCTTCTTTACATTTAT

TGCGATTCTTTCTTCACGAATATCATAATTGGAATAGTCTTATTACTCCGAATAATTCTATTTTTCTTTT

TTCAAAAGAAAATAAAAGACTATTTCGGTTCCCATATAATTCTTATGTATCTGAATGCGAATTTGTATTA

GTTTTTCTTCGTAAACAATCTTCTTATTTACGATTAACATCTTCTGGAGCTTTTCTTGAGCGAACACATT

TCTATGGAAAAATAGAATATCGTATAGTAGTGCGCCGTAATTATTTTCAGAAGACCCTATGGTTTTTCAA

GGATCCCTTCATGCATTATGTTCGATATCAAGGAAAAGCAATTCTGGTTTCAAAGGGGACTCATCTTCTG

ATGAAGAAATGGAAATGTCACCTTGTCAATTTCTGGCAATATTATTTTCACTTTTGGTCTCAACCGTACA

GGATTCATATAAACCAATTATCAAACTGTTCTTTCTATTTTCTAGGTTATCTTTCAAGTGTACTAATAAA

TCCTTCGGCGGTAAGGAATCAAATGCTAGAGAATTCATTTCTAATAGATACTGTTATTAAAAAATTCGAT

ACCAGAGTCCCAGTTATTCCTCTTATTAGATCATTGTCTAAAGCTAAATTTTGTACCGTATCGGGGCATC

CTATTAGTAAGCCAATCTGGACCGATTTATCAGATTGCGATATTATTGATCGATTTGGTCGGATATGTAG

AAATCTTTCTCATTATCACAGTGGATCCTCAAAAAAACAGAGTTTGTATCGAATAAAGTATATACTTCGA

TTTTCGTGTGCTAGAACTTTGGCTCGTAAACATAAAAGTATGGTACGCGCTTTTTTGCAAAGATTAGGTT

CGGGATTATTAGAAGAATTTTTTATGGAAGAAGAACAAGTTGTTTCTTTGATCTTCCCAAAAACAACTTC

TTTTTCTTTACATGAATCACATATAGAACGTATTTGGTATTTGGATATTATCCGTATCAATGACCTGGTG

AATTATTCATAATTGGTTTGTTGACGTGATGAGACTTATGAATAGAATAGAAATGATCTATAAATGATCA

AGAGAGAAAAAAATTCATGAATTTTCATTCTGAAATGCTCATTGCAGTAGTGTAGTGGTTGAATCAACTG

AGTAGTCAAAATTATTATACTTTCTTCTCGGGACCCAAGTTTTATATTATACATAGGTAAAGTC

>Welfia_regia_AM114650.1

GACCATATTGCACTATGTATCATTTGATAACCCAAAAAATGAAATGGGTCCTGCCTCTGGTTCAAGTAGA

AATGTAAATGGAAGAATTACAAGGATATTTAGAAAAAGATAGATCTCGGCAACAACACTTTCTATATCCG

CTTCTCTTTAAGGAGTATATTTACACATTTGCTCATGATCGTGGTTTAAATGGTTCGATTTTTTACGAAT

CCACGGAAATTTTTGGTTATGACAATAAATCTAGTTCAGTACTTGTGAAACGTTCAATTATTCGAATGTA

TCAACAGAATTATTTGATTTATTCGGTTAACGATTCTAACCAAAATCGATTCGTTGGGCACAATTTTTAT

TTTCATTTTTATTCTCAGATGATATTGGAAGGTTTTGCAGTCATTGTGGAAATTCCATTCTTGCTGCGAT

TAGTATCTTCCCTCGAAGAAAAAAAAATACCAAAATCTCAGAATTTGAATTTACGATCTATTCATTCAAT

ATTTCCCTTTTTGGAGGACAAATTATCGCATTTAAATTATGTGTCAGATATACTAATACCTTATCCCATC

CATCTGAAAATCTTGGTTCAAATCCTTCAATGCTGGATCCAAGATGTTCCTTCTTTACATTTATTGCGAT

TCTTTCTTCACGAATATCATAATTGGAATAGTCTTATTACTCCGAATAATTCTATTTTCTTTTTTTCAAA

AGAAAATAAAAGACTATTTTGGTTCCCATATAATTCTTATGTATCTGAATGCGAATTTGTATTAGTTTTT

CTTCGTAAACAATCTTCTTATTTACGATTAACATCTTCTGGAGCTTTTCTTGAGCGAACACATTTCTATG

GAAAAATAGAACGTCTTATAGTAGTGCGCCATAATTATTTTCAGAAGACCCTATGGTTCTTCAAGGATCC

CTTCATGCATTATGTTCGATATCAAGGAAAAGCAATTCTGGTTTCAAAGGGGACTCATCTTCTGATGAAG

AAATGGAAATGTCACCTTGTCAATTTCTGGCAATATTATTTTCACTTTTGGTCTCAACCGTACAGGATCC

ATATAAACCAATTATCAAGCTGTTCTTTCTATTTTCTAGGTTATCTTTCAAGTGTACTAATAAATCCTTC

GGCGGTAAGGAATCAAATGCTAGAGAATTCATTTCTAATAGATACTGTTATTAAAAAATTCGATACCAGA

GTCCCAGTTATTCCTCTTATTGGATCATTGTCTAAAGCTAAATTTTGTACCGTATCGGGGCATCCTATTA

GTAAGCCGATCTGGACCGATTTATCAGATTGCGATATTATTGATCGATTTGGTCGGATATGTAGAAATCT

TTCTCATTATCACAGTGGATCCTCAAAAAAACAGAGTTTGTATCGAATAAAGTATATACTTCGATTTTCG

TGTGCTAGAACTTTGGCTCGTAAACATAAAAGTATGGTACGCGCTTTTTTGCAAAGATTAGGTTCGGGAT

TATTAGAAGAATTCTTTACGGAAGAAGAACAAGTTGTTTCTTTGATCTTCCCAAAAACAACTTCTTTTTC

TTTACATGAATCACATATAGAACGTATTTGGTATTTGGATATTATCCGTATCAATGACCTGGTGAATTAT

TCATAATAGGTTTGGTGACGTGATGAGACTTATGAATAGAATAGAAATGATCTATAAATGATCAAGAGAG

AAAAAAATTCATGAATTTCCATTCTGAAATGCTCATTGCAGTAGTGTAGTGGTTGAATCAACTGAGTAGT

CAAAATTATTATACTTTCTTCTCGGGACCCAAGTTTTATATTATACATAGGAAAAGTC

>Wendlandiella_gracilis_var.polyclada_DQ178701.1

CGTTCTGACCATATTGCACTATGTATCATTCGATAACCCCAAAAATGAAATGGGTCCTGCCTCTGGTTCA

AGTAGAAATGTAAATGGAAGAATTACAAGGATTTTTAGAAAAAGATAGATCTCGGCAACAACACTTTCTA

TATCCGCTTCTCTTTAAGGAGTATATTTACACATTTGCTCATGATCGTGGTTTAAATGGTTCGATTTTTT

ACGAATCCACGGAAATTTTTGGTTATGACAATAAATCTAGTTCAGTACTTGTGAAACGTTCAATTATTCG

AATGTATCAACAGAATTATTTGATTTATTCGGTTAATGATTCTAACCAAAATCGATTCGTTGGGCACAAC

AATTATTTTCATTTTTATTCTCAGATGATATTGGAAGGTTTTGCAGTCATTGTGGAAATTCCATTCTTGC

TGCGATTAGTATCTTCTCTCGAAGAAAAAAAAATACCAAAATCTCATAATTTGAATTTACGATCTATTCA

TTCAATATTTCCCTTTTTGGAGGACAAATTATCGCATTTAAATTATGTGTCAGATATACTAATACCTTAT

CCCATCCATCTGAAAATCTTGGTTCAAATCCTTCAATGCTGGATCCAAGATGTTCCTTCTTTACATTTAT

TGCGATTCTTTCTTCATGAATATCATAATTGGAATAGTCTTATTACTCCGAATAATTCTATTTTTTTTTT

TTCAAAAGAAAATAAAAGACTATTTCGGTTCCCATATAATTCTTATGTATCTGAATGCGAATTTGTATTA

GTTTTTCTTCGTAAACAATCTTCTTATTTACGATTAACATCTTCTGGAGCTTTTCTTGAGCGAACACATT

TCTATGGAAAAATAGAACATCTTATAGTAGTGCGCCGTAATTATTTTCAGAAGACCCTATGGTTCTTCAA

GGATCCCTTCATGCATTATGTTCGATATCAAGGAAAAGCAATTCTGGTTTCAAAGGGGACTCATCTTCTG

ATGAAGAAATGGAAATGTCACCTTGTCAATTTCTGGCAATATTATTTTCACTTTTGGTCTCAACCGTACA

GGATCCATATAAACCAATTATCAAGCTGTTCTTTCCATTTTCTAGGTTATCTTTCAAGTGTACTAATAAA

TCCTTKGGCGGTAAGGAATCAAATGCTAGAGAATTCATTTCTAATAGATACTGTTATTAAAAAATTCGAT

ACCAGAGTCCCAGTTATTCCTCTTATTGGATCATTGTCTAAAGCTAAATTTTGTACCGTATCGGGGCATC

CTATTAGTAAGCCGATCTGGACCAATTTATCAGATTGCGATATTATTGATCGATTTGGTCGGATATGTAG

AAATCTTTCTCATTATCACAGTGGATCCTCAAAAAAACAGAGTTTGTATCGAATAAAGTATATACTTCGA

TTTTCGTGTGCTAGAACTTTGGCTCGTAAACATAAAAGTACGGTACGCGCTTTTTTGCAAAGATTAGGTT

CGGGATTATTAGAAGAATTTTTTACGGAAGAAGAACAAGTTGTTTCTTTGATCTTCCCCAAAACAACTTC

TTTTTCTTTACATGAATCACATATAGAACGTATTTGGTATTTGGATATTATCCGTATCAATGACCTGGTG

AATTATTCATAATGGGTTTGTTTGGTGACGTGATGAGACTTATGAATAGTATGGAAATGATCTATAAATG

ATCAAGAGAGAAAAAAAAATTCATGAATTTTCATTCTGAAATGCTCATTGCAGTAGTGTAGTGGTTGAAT

CAACTGAGTAGTAAAAATTATTATACTTTCTTCTCGGGACCCAAGTTTTATATTATACATAGGTAAAGTC

GTGTGCAATG
[truncated: 5,672 more chars]
