# Supplementary material for: Adjacency and Area Explain Species Bioregional Shifts in Neotropical Palms
Source: Front Plant Sci. 2019 Feb 5;10:55. doi: 10.3389/fpls.2019.00055 (PMC6370682; doi:10.3389/fpls.2019.00055)
Supplement: Supplementary file 3 [file Data_Sheet_3.docx]

**Adjacency and area explain species bioregional shifts in Neotropical palms**

Cintia G. Freitas, Christine D. Bacon, Advaldo C. Souza-Neto, Rosane G. Collevatti

**Appendix S3**. **Figures**

**Figure S1**. Bayesian phylogenetic hypothesis for the American palms, based on the chloroplast *matK* *orf* and *RPB2* nuclear gene. Numbers above the branches are posterior probabilities.

**Figure S1**. (cont.)

**Figure S1**. (cont.)

**Figure S2**. Annual mean temperature and annual precipitation for each bioregion. Bioregion abbreviations are defined in Table S2 in Appendix S4.

**Figure S3**. Annual mean temperature and annual precipitation for each tropical rain forest bioregions (TRF) and non tropical rain forest bioregions (non-TRF).

**Figure S4**. Relationship between species richness and the bioregion area (km^2^). Bioregion abbreviations are defined in Table S2 in Appendix S4.
