# Supplementary material for: Adjacency and Area Explain Species Bioregional Shifts in Neotropical Palms
Source: Front Plant Sci. 2019 Feb 5;10:55. doi: 10.3389/fpls.2019.00055 (PMC6370682; doi:10.3389/fpls.2019.00055)
Supplement: Supplementary file 4 [file Data_Sheet_4.docx]

**Adjacency and area explain species bioregional shifts in Neotropical palms**

Cintia G. Freitas, Christine D. Bacon, Advaldo C. Souza-Neto, Rosane G. Collevatti

**Appendix S4**. **Tables**

**Table S1**. Species and outgroup taxa used to construct the phylogeny. Species indicated with an * were unsampled in the dataset and therefore inserted *a posteriori*.

| **Genus** | **Species** | **Insertion point** |
| --- | --- | --- |
| *Acoelorraphe* | *Acoelorrhaphe wrightii* | - |
| *Acrocomia* | *Acrocomia aculeata* | - |
| *Acrocomia* | *Acrocomia crispa** | - |
| *Acrocomia* | *Acrocomia hassleri* | Crown node of Acrocomia |
| *Aiphanes* | *Aiphanes acaulis* | Crown node of *Aiphanes* |
| *Aiphanes* | *Aiphanes aculeata** | - |
| *Aiphanes* | *Aiphanes chiribogensis* | Crown node of *Aiphanes* |
| *Aiphanes* | *Aiphanes deltoidea* | Crown node of *Aiphanes* |
| *Aiphanes* | *Aiphanes eggersii* | Crown node of *Aiphanes* |
| *Aiphanes* | *Aiphanes erinacea* | - |
| *Aiphanes* | *Aiphanes gelatinosa* | Crown node of *Aiphanes* |
| *Aiphanes* | *Aiphanes grandis* | - |
| *Aiphanes* | *Aiphanes hirsuta* | - |
| *Aiphanes* | *Aiphanes horrida** | - |
| *Aiphanes* | *Aiphanes killipii** | - |
| *Aiphanes* | *Aiphanes leiostachys* | Crown node of *Aiphanes* |
| *Aiphanes* | *Aiphanes lindeniana* | Crown node of *Aiphanes* |
| *Aiphanes* | *Aiphanes linearis* | Crown node of *Aiphanes* |
| *Aiphanes* | *Aiphanes macroloba* | Crown node of *Aiphanes* |
| *Aiphanes* | *Aiphanes minima* | - |
| *Aiphanes* | *Aiphanes parvifolia* | Crown node of *Aiphanes* |
| *Aiphanes* | *Aiphanes simplex* | Crown node of *Aiphanes* |
| *Aiphanes* | *Aiphanes spicata* | - |
| *Aiphanes* | *Aiphanes tricuspidata* | Crown node of *Aiphanes* |
| *Aiphanes* | *Aiphanes ulei* | - |
| *Aiphanes* | *Aiphanes verrucosa* | Crown node of *Aiphanes* |
| *Aiphanes* | *Aiphanes weberbaueri* | Crown node of *Aiphanes* |
| *Allagoptera* | *Allagoptera arenaria* | - |
| *Allagoptera* | *Allagoptera brevicalyx* | Steam node of *Allagoptera* |
| *Allagoptera* | *Allagoptera campestris* | Steam node of *Allagoptera* |
| *Allagoptera* | *Allagoptera caudescens* | - |
| *Allagoptera* | *Allagoptera leucocalyx* | Steam node of *Allagoptera* |
| *Ammandra* | *Ammandra decasperma* | - |
| *Aphandra* | *Aphandra natalia* | - |
| *Asterogyne* | *Asterogyne guianensis* | - |
| *Asterogyne* | *Asterogyne martiana* | - |
| *Asterogyne* | *Asterogyne ramosa* | Crown node of *Asterogyne* |
| *Astrocaryum* | *Astrocaryum acaule* | Crown node of *Astrocaryum* |
| *Astrocaryum* | *Astrocaryum aculeatissimum* | Crown node of *Astrocaryum* |
| *Astrocaryum* | *Astrocaryum aculeatum* | - |
| *Astrocaryum* | *Astrocaryum alatum* | - |
| *Astrocaryum* | *Astrocaryum campestre* | - |
| *Astrocaryum* | *Astrocaryum carnosum** | - |
| *Astrocaryum* | *Astrocaryum chambira* | - |
| *Astrocaryum* | *Astrocaryum chonta** | - |
| *Astrocaryum* | *Astrocaryum confertum* | Crown node of *Astrocaryum* |
| *Astrocaryum* | *Astrocaryum faranae** | - |
| *Astrocaryum* | *Astrocaryum farinosum** | - |
| *Astrocaryum* | *Astrocaryum ferrugineum** | - |
| *Astrocaryum* | *Astrocaryum gratum** | - |
| *Astrocaryum* | *Astrocaryum gynacanthum** | - |
| *Astrocaryum* | *Astrocaryum huaimi* | Crown node of *Astrocaryum* |
| *Astrocaryum* | *Astrocaryum huicungo** | - |
| *Astrocaryum* | *Astrocaryum jauari* | Crown node of *Astrocaryum* |
| *Astrocaryum* | *Astrocaryum javarense** | - |
| *Astrocaryum* | *Astrocaryum macrocalyx** | - |
| *Astrocaryum* | *Astrocaryum malybo* | - |
| *Astrocaryum* | *Astrocaryum mexicanum* | - |
| *Astrocaryum* | *Astrocaryum minus** | - |
| *Astrocaryum* | *Astrocaryum murumuru* | - |
| *Astrocaryum* | *Astrocaryum paramaca* | - |
| *Astrocaryum* | *Astrocaryum perangustatum** | - |
| *Astrocaryum* | *Astrocaryum rodriguesii** | - |
| *Astrocaryum* | *Astrocaryum sciophilum* | - |
| *Astrocaryum* | *Astrocaryum scopatum** | - |
| *Astrocaryum* | *Astrocaryum sociale** | - |
| *Astrocaryum* | *Astrocaryum standleyanum* | - |
| *Astrocaryum* | *Astrocaryum triandrum* | Crown node of *Astrocaryum* |
| *Astrocaryum* | *Astrocaryum ulei** | - |
| *Astrocaryum* | *Astrocaryum urostachys** | - |
| *Astrocaryum* | *Astrocaryum vulgare* | - |
| *Attalea* | *Attalea allenii* | - |
| *Attalea* | *Attalea amygdalina* | Crown node of *Attalea* |
| *Attalea* | *Attalea attaleoides* | Crown node of *Attalea* |
| *Attalea* | *Attalea butyracea* | - |
| *Attalea* | *Attalea cohune* | Crown node of *Attalea* |
| *Attalea* | *Attalea colenda* | Crown node of *Attalea* |
| *Attalea* | *Attalea crassispatha* | Crown node of *Attalea* |
| *Attalea* | *Attalea cuatrecasana* | Crown node of *Attalea* |
| *Attalea* | *Attalea dahlgreniana* | Crown node of *Attalea* |
| *Attalea* | *Attalea dubia* | Crown node of *Attalea* |
| *Attalea* | *Attalea eichleri* | Crown node of *Attalea* |
| *Attalea* | *Attalea exigua* | Crown node of *Attalea* |
| *Attalea* | *Attalea funifera* | Crown node of *Attalea* |
| *Attalea* | *Attalea geraensis* | Crown node of *Attalea* |
| *Attalea* | *Attalea humilis* | Crown node of *Attalea* |
| *Attalea* | *Attalea iguadummat* | Crown node of *Attalea* |
| *Attalea* | *Attalea insignis* | Crown node of *Attalea* |
| *Attalea* | *Attalea luetzelburgii* | Crown node of *Attalea* |
| *Attalea* | *Attalea maripa* | Crown node of *Attalea* |
| *Attalea* | *Attalea microcarpa* | Crown node of *Attalea* |
| *Attalea* | *Attalea nucifera* | Crown node of *Attalea* |
| *Attalea* | *Attalea oleifera* | Crown node of *Attalea* |
| *Attalea* | *Attalea phalerata* | - |
| *Attalea* | *Attalea pindobassu* | Crown node of *Attalea* |
| *Attalea* | *Attalea racemosa* | Crown node of *Attalea* |
| *Attalea* | *Attalea rostrata** | - |
| *Attalea* | *Attalea speciosa* | Crown node of *Attalea* |
| *Attalea* | *Attalea spectabilis* | Crown node of *Attalea* |
| *Attalea* | *Attalea tessmannii* | Crown node of *Attalea* |
| *Bactris* | *Bactris acanthocarpa* | - |
| *Bactris* | *Bactris acanthocarpoides* | Crown node of *Bactris* |
| *Bactris* | *Bactris aubletiana* | Crown node of *Bactris* |
| *Bactris* | *Bactris bahiensis* | Crown node of *Bactris* |
| *Bactris* | *Bactris balanophora* | Crown node of *Bactris* |
| *Bactris* | *Bactris barronis* | - |
| *Bactris* | *Bactris bidentula* | Crown node of *Bactris* |
| *Bactris* | *Bactris bifida* | - |
| *Bactris* | *Bactris brongniartii* | Crown node of *Bactris* |
| *Bactris* | *Bactris campestris* | - |
| *Bactris* | *Bactris caryotifolia* | Crown node of *Bactris* |
| *Bactris* | *Bactris caudata* | Crown node of *Bactris* |
| *Bactris* | *Bactris charnleyae* | Crown node of *Bactris* |
| *Bactris* | *Bactris coloniata* | Crown node of *Bactris* |
| *Bactris* | *Bactris coloradonis* | Crown node of *Bactris* |
| *Bactris* | *Bactris concinna* | Crown node of *Bactris* |
| *Bactris* | *Bactris constanciae* | - |
| *Bactris* | *Bactris corossilla* | Crown node of *Bactris* |
| *Bactris* | *Bactris cuspidata* | Crown node of *Bactris* |
| *Bactris* | *Bactris dianeura* | Crown node of *Bactris* |
| *Bactris* | *Bactris elegans* | Crown node of *Bactris* |
| *Bactris* | *Bactris ferruginea* | - |
| *Bactris* | *Bactris fissifrons* | Crown node of *Bactris* |
| *Bactris* | *Bactris gasipaes* | - |
| *Bactris* | *Bactris gastoniana* | Crown node of *Bactris* |
| *Bactris* | *Bactris glandulosa* | Crown node of *Bactris* |
| *Bactris* | *Bactris glassmanii* | Crown node of *Bactris* |
| *Bactris* | *Bactris glaucescens* | Crown node of *Bactris* |
| *Bactris* | *Bactris gracilior* | Crown node of *Bactris* |
| *Bactris* | *Bactris grayumii* | Crown node of *Bactris* |
| *Bactris* | *Bactris guineensis* | - |
| *Bactris* | *Bactris hatschbachii* | Crown node of *Bactris* |
| *Bactris* | *Bactris hirta* | Crown node of *Bactris* |
| *Bactris* | *Bactris hondurensis* | Crown node of *Bactris* |
| *Bactris* | *Bactris horridispatha* | Crown node of *Bactris* |
| *Bactris* | *Bactris killipii* | - |
| *Bactris* | *Bactris kunorum* | Crown node of *Bactris* |
| *Bactris* | *Bactris longiseta* | Crown node of *Bactris* |
| *Bactris* | *Bactris macroacantha* | Crown node of *Bactris* |
| *Bactris* | *Bactris major* | - |
| *Bactris* | *Bactris maraja** | - |
| *Bactris* | *Bactris mexicana* | Crown node of *Bactris* |
| *Bactris* | *Bactris militaris* | Crown node of *Bactris* |
| *Bactris* | *Bactris oligocarpa* | Crown node of *Bactris* |
| *Bactris* | *Bactris oligoclada* | Crown node of *Bactris* |
| *Bactris* | *Bactris panamensis* | Crown node of *Bactris* |
| *Bactris* | *Bactris pickelii* | Crown node of *Bactris* |
| *Bactris* | *Bactris pilosa* | Crown node of *Bactris* |
| *Bactris* | *Bactris pliniana* | - |
| *Bactris* | *Bactris plumeriana* | Crown node of *Bactris* |
| *Bactris* | *Bactris ptariana* | Crown node of *Bactris* |
| *Bactris* | *Bactris rhaphidacantha* | Crown node of *Bactris* |
| *Bactris* | *Bactris riparia* | - |
| *Bactris* | *Bactris setosa* | Crown node of *Bactris* |
| *Bactris* | *Bactris setulosa* | - |
| *Bactris* | *Bactris simplicifrons* | - |
| *Bactris* | *Bactris soeiroana* | Crown node of *Bactris* |
| *Bactris* | *Bactris syagroides* | Crown node of *Bactris* |
| *Bactris* | *Bactris tefensis* | Crown node of *Bactris* |
| *Bactris* | *Bactris tomentosa* | Crown node of *Bactris* |
| *Bactris* | *Bactris turbinocarpa* | Crown node of *Bactris* |
| *Bactris* | *Bactris vulgaris* | Crown node of *Bactris* |
| *Barcella* | *Barcella odora* | - |
| *Brahea* | *Brahea aculeata* | - |
| *Brahea* | *Brahea armata* | - |
| *Brahea* | *Brahea berlandieri** | - |
| *Brahea* | *Brahea brandegeei* | - |
| *Brahea* | *Brahea calcarea* | - |
| *Brahea* | *Brahea decumbens* | Steam node of *Brahea* |
| *Brahea* | *Brahea dulcis* | - |
| *Brahea* | *Brahea edulis* | Steam node of *Brahea* |
| *Brahea* | *Brahea moorei* | Steam node of *Brahea* |
| *Brahea* | *Brahea pimo* | Steam node of *Brahea* |
| *Butia* | *Butia archeri* | Crown node of *Butia* |
| *Butia* | *Butia campicola* | Crown node of *Butia* |
| *Butia* | *Butia capitata* | - |
| *Butia* | *Butia eriospatha* | Crown node of *Butia* |
| *Butia* | *Butia leptospatha* | Crown node of *Butia* |
| *Butia* | *Butia microspadix* | Crown node of *Butia* |
| *Butia* | *Butia paraguayensis* | Crown node of *Butia* |
| *Butia* | *Butia purpurascens* | Crown node of *Butia* |
| *Butia* | *Butia yatay* | - |
| *Calyptrogyne* | *Calyptrogyne allenii* | - |
| *Calyptrogyne* | *Calyptrogyne anomala* | - |
| *Calyptrogyne* | *Calyptrogyne brachystachys** | - |
| *Calyptrogyne* | *Calyptrogyne condensata* | Crown node of *Calyptrogyne* |
| *Calyptrogyne* | *Calyptrogyne costatifrons* | - |
| *Calyptrogyne* | *Calyptrogyne fortunensis** | - |
| *Calyptrogyne* | *Calyptrogyne ghiesbreghtiana* | - |
| *Calyptrogyne* | *Calyptrogyne kunorum* | Crown node of *Calyptrogyne* |
| *Calyptrogyne* | *Calyptrogyne osensis** | - |
| *Calyptrogyne* | *Calyptrogyne panamensis** | - |
| *Calyptrogyne* | *Calyptrogyne pubescens* | Crown node of *Calyptrogyne* |
| *Calyptrogyne* | *Calyptrogyne trichostachys* | - |
| *Calyptrogyne* | *Calyptrogyne tutensis** | - |
| *Calyptronoma* | *Calyptronoma occidentalis* | - |
| *Calyptronoma* | *Calyptronoma plumeriana* | - |
| *Calyptronoma* | *Calyptronoma rivalis* | - |
| *Ceroxylon* | *Ceroxylon alpinum* | - |
| *Ceroxylon* | *Ceroxylon amazonicum* | - |
| *Ceroxylon* | *Ceroxylon ceriferum* | Crown node of *Ceroxylon* |
| *Ceroxylon* | *Ceroxylon echinulatum* | - |
| *Ceroxylon* | *Ceroxylon parvifrons* | - |
| *Ceroxylon* | *Ceroxylon parvum* | - |
| *Ceroxylon* | *Ceroxylon quindiuense* | - |
| *Ceroxylon* | *Ceroxylon sasaimae* | Crown node of *Ceroxylon* |
| *Ceroxylon* | *Ceroxylon ventricosum* | - |
| *Ceroxylon* | *Ceroxylon vogelianum* | - |
| *Ceroxylon* | *Ceroxylon weberbaueri* | Crown node of *Ceroxylon* |
| *Chamaedorea* | *Chamaedorea adscendens* | - |
| *Chamaedorea* | *Chamaedorea allenii* | - |
| *Chamaedorea* | *Chamaedorea alternans** | - |
| *Chamaedorea* | *Chamaedorea amabilis* | - |
| *Chamaedorea* | *Chamaedorea anemophila** | - |
| *Chamaedorea* | *Chamaedorea angustisecta* | Crown node of *Chamaedorea* |
| *Chamaedorea* | *Chamaedorea arenbergiana* | - |
| *Chamaedorea* | *Chamaedorea brachyclada* | - |
| *Chamaedorea* | *Chamaedorea brachypoda* | - |
| *Chamaedorea* | *Chamaedorea carchensis* | Crown node of *Chamaedorea* |
| *Chamaedorea* | *Chamaedorea cataractarum* | - |
| *Chamaedorea* | *Chamaedorea coralliformis** | - |
| *Chamaedorea* | *Chamaedorea correae* | Crown node of *Chamaedorea* |
| *Chamaedorea* | *Chamaedorea costaricana* | - |
| *Chamaedorea* | *Chamaedorea crucensis** | - |
| *Chamaedorea* | *Chamaedorea dammeriana* | - |
| *Chamaedorea* | *Chamaedorea deckeriana* | Crown node of *Chamaedorea* |
| *Chamaedorea* | *Chamaedorea deneversiana* | Crown node of *Chamaedorea* |
| *Chamaedorea* | *Chamaedorea elatior* | - |
| *Chamaedorea* | *Chamaedorea elegans* | - |
| *Chamaedorea* | *Chamaedorea ernesti augustii* | - |
| *Chamaedorea* | *Chamaedorea fragrans* | - |
| *Chamaedorea* | *Chamaedorea frondosa** | - |
| *Chamaedorea* | *Chamaedorea geonomiformis* | - |
| *Chamaedorea* | *Chamaedorea glaucifolia* | - |
| *Chamaedorea* | *Chamaedorea graminifolia* | - |
| *Chamaedorea* | *Chamaedorea guntheriana* | Crown node of *Chamaedorea* |
| *Chamaedorea* | *Chamaedorea hooperiana* | - |
| *Chamaedorea* | *Chamaedorea ibarrae** | - |
| *Chamaedorea* | *Chamaedorea klotzschiana* | - |
| *Chamaedorea* | *Chamaedorea lehmannii* | Crown node of *Chamaedorea* |
| *Chamaedorea* | *Chamaedorea liebmannii* | Crown node of *Chamaedorea* |
| *Chamaedorea* | *Chamaedorea linearis* | - |
| *Chamaedorea* | *Chamaedorea lucidifrons* | Crown node of *Chamaedorea* |
| *Chamaedorea* | *Chamaedorea macrospadix* | - |
| *Chamaedorea* | *Chamaedorea metallica* | - |
| *Chamaedorea* | *Chamaedorea microphylla* | - |
| *Chamaedorea* | *Chamaedorea microspadix* | - |
| *Chamaedorea* | *Chamaedorea murriensis* | Crown node of *Chamaedorea* |
| *Chamaedorea* | *Chamaedorea nationsiana** | - |
| *Chamaedorea* | *Chamaedorea neurochlamys** | - |
| *Chamaedorea* | *Chamaedorea nubium* | - |
| *Chamaedorea* | *Chamaedorea oblongata* | - |
| *Chamaedorea* | *Chamaedorea oreophila* | Crown node of *Chamaedorea* |
| *Chamaedorea* | *Chamaedorea palmeriana* | Crown node of *Chamaedorea* |
| *Chamaedorea* | *Chamaedorea parvifolia* | - |
| *Chamaedorea* | *Chamaedorea parvisecta* | - |
| *Chamaedorea* | *Chamaedorea pauciflora* | - |
| *Chamaedorea* | *Chamaedorea pedunculata** | - |
| *Chamaedorea* | *Chamaedorea pinnatifrons* | - |
| *Chamaedorea* | *Chamaedorea pittieri* | Crown node of *Chamaedorea* |
| *Chamaedorea* | *Chamaedorea plumosa* | - |
| *Chamaedorea* | *Chamaedorea pochutlensis* | - |
| *Chamaedorea* | *Chamaedorea pumila* | - |
| *Chamaedorea* | *Chamaedorea pygmaea* | Crown node of *Chamaedorea* |
| *Chamaedorea* | *Chamaedorea queroana* | Crown node of *Chamaedorea* |
| *Chamaedorea* | *Chamaedorea quezalteca** | - |
| *Chamaedorea* | *Chamaedorea radicalis* | - |
| *Chamaedorea* | *Chamaedorea rigida* | Crown node of *Chamaedorea* |
| *Chamaedorea* | *Chamaedorea robertii* | Crown node of *Chamaedorea* |
| *Chamaedorea* | *Chamaedorea rojasiana* | - |
| *Chamaedorea* | *Chamaedorea rossteniorum** | - |
| *Chamaedorea* | *Chamaedorea sartorii* | - |
| *Chamaedorea* | *Chamaedorea scheryi* | Crown node of *Chamaedorea* |
| *Chamaedorea* | *Chamaedorea schiedeana* | - |
| *Chamaedorea* | *Chamaedorea schippii** | - |
| *Chamaedorea* | *Chamaedorea seifrizii* | - |
| *Chamaedorea* | *Chamaedorea selvae* | Crown node of *Chamaedorea* |
| *Chamaedorea* | *Chamaedorea serpens** | - |
| *Chamaedorea* | *Chamaedorea simplex* | Crown node of *Chamaedorea* |
| *Chamaedorea* | *Chamaedorea stenocarpa** | - |
| *Chamaedorea* | *Chamaedorea stolonifera** | - |
| *Chamaedorea* | *Chamaedorea stricta* | - |
| *Chamaedorea* | *Chamaedorea tenella** | - |
| *Chamaedorea* | *Chamaedorea tenerrima* | Crown node of *Chamaedorea* |
| *Chamaedorea* | *Chamaedorea tepejilote* | - |
| *Chamaedorea* | *Chamaedorea tuerckheimii* | - |
| *Chamaedorea* | *Chamaedorea undulatifolia* | Crown node of *Chamaedorea* |
| *Chamaedorea* | *Chamaedorea verecunda* | Crown node of *Chamaedorea* |
| *Chamaedorea* | *Chamaedorea vulgata* | - |
| *Chamaedorea* | *Chamaedorea warscewiczii* | - |
| *Chamaedorea* | *Chamaedorea whitelockiana* | - |
| *Chamaedorea* | *Chamaedorea woodsoniana* | - |
| *Chelyocarpus* | *Chelyocarpus chuco* | - |
| *Chelyocarpus* | *Chelyocarpus dianeurus* | - |
| *Chelyocarpus* | *Chelyocarpus repens* | - |
| *Chelyocarpus* | *Chelyocarpus ulei* | - |
| *Coccothrinax* | *Coccothrinax argentata* | - |
| *Coccothrinax* | *Coccothrinax argentea* | Crown node of *Coccothrinax* |
| *Coccothrinax* | *Coccothrinax barbadensis* | - |
| *Coccothrinax* | *Coccothrinax borhidiana** | - |
| *Coccothrinax* | *Coccothrinax camagueyana** | - |
| *Coccothrinax* | *Coccothrinax crinita* | - |
| *Coccothrinax* | *Coccothrinax ekmanii* | Crown node of *Coccothrinax* |
| *Coccothrinax* | *Coccothrinax gracilis* | Crown node of *Coccothrinax* |
| *Coccothrinax* | *Coccothrinax gundlachii* | Crown node of *Coccothrinax* |
| *Coccothrinax* | *Coccothrinax inaguensis** | - |
| *Coccothrinax* | *Coccothrinax litoralis** | - |
| *Coccothrinax* | *Coccothrinax miraguama* | - |
| *Coccothrinax* | *Coccothrinax pauciramosa* | Crown node of *Coccothrinax* |
| *Coccothrinax* | *Coccothrinax salvatoris* | - |
| *Coccothrinax* | *Coccothrinax spissa* | - |
| *Cocos* | *Cocos nucifera** | - |
| *Colpothrinax* | *Colpothrinax aphanopetala** | - |
| *Colpothrinax* | *Colpothrinax cookii* | - |
| *Colpothrinax* | *Colpothrinax wrightii* | - |
| *Copernicia* | *Copernicia alba* | - |
| *Copernicia* | *Copernicia baileyana* | - |
| *Copernicia* | *Copernicia berteroana* | - |
| *Copernicia* | *Copernicia cowellii* | Crown node of *Copernicia* |
| *Copernicia* | *Copernicia curtissii** | - |
| *Copernicia* | *Copernicia ekmanii* | - |
| *Copernicia* | *Copernicia fallaensis** | - |
| *Copernicia* | *Copernicia gigas* | Crown node of *Copernicia* |
| *Copernicia* | *Copernicia glabrescens* | - |
| *Copernicia* | *Copernicia hospita* | - |
| *Copernicia* | *Copernicia macroglossa* | - |
| *Copernicia* | *Copernicia prunifera* | - |
| *Copernicia* | *Copernicia rigida* | - |
| *Copernicia* | *Copernicia tectorum* | - |
| *Copernicia* | *Copernicia yarey** | - |
| *Cryosophila* | *Cryosophila cookii* | Crown node of *Cryosophila* |
| *Cryosophila* | *Cryosophila grayumii* | Crown node of *Cryosophila* |
| *Cryosophila* | *Cryosophila guagara* | Crown node of *Cryosophila* |
| *Cryosophila* | *Cryosophila kalbreyeri* | - |
| *Cryosophila* | *Cryosophila macrocarpa* | Crown node of *Cryosophila* |
| *Cryosophila* | *Cryosophila nana* | - |
| *Cryosophila* | *Cryosophila stauracantha* | - |
| *Cryosophila* | *Cryosophila warscewiczii* | - |
| *Cryosophila* | *Cryosophila williamsii* | - |
| *Desmoncus* | *Desmoncus cirrhifera* | - |
| *Desmoncus* | *Desmoncus giganteus* | Crown node of *Desmoncus* |
| *Desmoncus* | *Desmoncus mitis* | - |
| *Desmoncus* | *Desmoncus orthacanthos* | - |
| *Desmoncus* | *Desmoncus phoenicocarpus* | Crown node of *Desmoncus* |
| *Desmoncus* | *Desmoncus polyacanthos* | - |
| *Desmoncus* | *Desmoncus schippi** | Crown node of *Desmoncus* |
| *Desmoncus* | *Desmoncus stans* | Crown node of *Desmoncus* |
| *Dictyocaryum* | *Dictyocaryum lamarckianum* | - |
| *Dictyocaryum* | *Dictyocaryum ptarianum* | - |
| *Elaeis* | *Elaeis guineensis** | - |
| *Elaeis* | *Elaeis oleifera* | - |
| *Euterpe* | *Euterpe broadwayi* | Crown node of *Euterpe* |
| *Euterpe* | *Euterpe catinga* | Crown node of *Euterpe* |
| *Euterpe* | *Euterpe edulis* | Crown node of *Euterpe* |
| *Euterpe* | *Euterpe longibracteata* | Crown node of *Euterpe* |
| *Euterpe* | *Euterpe luminosa* | Crown node of *Euterpe* |
| *Euterpe* | *Euterpe oleracea* | - |
| *Euterpe* | *Euterpe precatoria* | - |
| *Gaussia* | *Gaussia attenuata* | - |
| *Gaussia* | *Gaussia gomez pompae* | - |
| *Gaussia* | *Gaussia maya* | - |
| *Gaussia* | *Gaussia princeps* | - |
| *Gaussia* | *Gaussia spirituana** | - |
| *Geonoma* | *Geonoma appuniana* | Crown node of *Geonoma* |
| *Geonoma* | *Geonoma arundinacea* | - |
| *Geonoma* | *Geonoma aspidiifolia* | Crown node of *Geonoma* |
| *Geonoma* | *Geonoma atrovirens** | - |
| *Geonoma* | *Geonoma baculifera* | - |
| *Geonoma* | *Geonoma bernalii** | - |
| *Geonoma* | *Geonoma brenesii** | - |
| *Geonoma* | *Geonoma brevispatha* | - |
| *Geonoma* | *Geonoma brongniartii* | - |
| *Geonoma* | *Geonoma camana* | - |
| *Geonoma* | *Geonoma chlamydostachys* | Crown node of *Geonoma* |
| *Geonoma* | *Geonoma chococola* | Crown node of *Geonoma* |
| *Geonoma* | *Geonoma concinna* | - |
| *Geonoma* | *Geonoma congesta* | - |
| *Geonoma* | *Geonoma cuneata* | - |
| *Geonoma* | *Geonoma densa* | Crown node of *Geonoma* |
| *Geonoma* | *Geonoma deversa* | - |
| *Geonoma* | *Geonoma divisa* | - |
| *Geonoma* | *Geonoma edulis** | - |
| *Geonoma* | *Geonoma elegans** | - |
| *Geonoma* | *Geonoma epetiolata* | - |
| *Geonoma* | *Geonoma ferruginea* | - |
| *Geonoma* | *Geonoma frontinensis** | - |
| *Geonoma* | *Geonoma gamiova* | Crown node of *Geonoma* |
| *Geonoma* | *Geonoma hollinensis** | - |
| *Geonoma* | *Geonoma hugonis** | - |
| *Geonoma* | *Geonoma interrupta* | - |
| *Geonoma* | *Geonoma jussieuana* | - |
| *Geonoma* | *Geonoma laxiflora* | - |
| *Geonoma* | *Geonoma leptospadix* | - |
| *Geonoma* | *Geonoma linearis* | Crown node of *Geonoma* |
| *Geonoma* | *Geonoma longipedunculata* | - |
| *Geonoma* | *Geonoma longivaginata* | - |
| *Geonoma* | *Geonoma macrostachys* | - |
| *Geonoma* | *Geonoma maxima* | - |
| *Geonoma* | *Geonoma monospatha** | - |
| *Geonoma* | *Geonoma oldemanii* | - |
| *Geonoma* | *Geonoma orbignyana* | - |
| *Geonoma* | *Geonoma paradoxa* | Crown node of *Geonoma* |
| *Geonoma* | *Geonoma pauciflora* | - |
| *Geonoma* | *Geonoma poeppigiana* | - |
| *Geonoma* | *Geonoma pohliana* | - |
| *Geonoma* | *Geonoma polyandra* | - |
| *Geonoma* | *Geonoma rubescens* | Crown node of *Geonoma* |
| *Geonoma* | *Geonoma schottiana* | - |
| *Geonoma* | *Geonoma scoparia* | - |
| *Geonoma* | *Geonoma simplicifrons* | - |
| *Geonoma* | *Geonoma spinescens* | Crown node of *Geonoma* |
| *Geonoma* | *Geonoma stricta* | - |
| *Geonoma* | *Geonoma supracostata** | - |
| *Geonoma* | *Geonoma tenuissima* | - |
| *Geonoma* | *Geonoma triandra* | Crown node of *Geonoma* |
| *Geonoma* | *Geonoma triglochin* | - |
| *Geonoma* | *Geonoma trigona* | Crown node of *Geonoma* |
| *Geonoma* | *Geonoma umbraculiformis* | - |
| *Geonoma* | *Geonoma undata* | - |
| *Geonoma* | *Geonoma weberbaueri* | - |
| *Hemithrinax* | *Hemithrinax compacta* | - |
| *Hemithrinax* | *Hemithrinax ekmaniana** | - |
| *Hemithrinax* | *Hemithrinax rivularis* | - |
| *Hyospathe* | *Hyospathe elegans* | Steam node of *Hyospathe* |
| *Hyospathe* | *Hyospathe macrorachis* | - |
| *Iriartea* | *Iriartea deltoidea* | - |
| *Iriartella* | *Iriartella setigera* | - |
| *Iriartella* | *Iriartella stenocarpa* | - |
| *Itaya* | *Itaya amicorum* | - |
| *Juania* | *Juania australis* | - |
| *Jubaea* | *Jubaea chilensis* | - |
| *Leopoldinia* | *Leopoldinia piassaba* | Steam node of *Leopoldinia* |
| *Leopoldinia* | *Leopoldinia pulchra* | - |
| *Lepidocaryum* | *Lepidocaryum tenue* | Crown node of *Mauritiinae* |
| *Leucothrinax* | *Leucothrinax morrisii* | - |
| *Lytocaryum* | *Lytocaryum hoehnei* | Steam node of *Lytocaryum* |
| *Lytocaryum* | *Lytocaryum weddellianum* | - |
| *Manicaria* | *Manicaria saccifera* | - |
| *Mauritia* | *Mauritia carana* | Steam node of *Mauritia* |
| *Mauritia* | *Mauritia flexuosa* | - |
| *Mauritiella* | *Mauritiella aculeata* | - |
| *Mauritiella* | *Mauritiella armata* | Steam node of *Mauritiella* |
| *Mauritiella* | *Mauritiella macroclada* | Steam node of *Mauritiella* |
| *Neonicholsonia* | *Neonicholsonia watsonii* | - |
| *Oenocarpus* | *Oenocarpus bacaba* | - |
| *Oenocarpus* | *Oenocarpus balickii* | Steam node of *Oenocarpus* |
| *Oenocarpus* | *Oenocarpus bataua* | Steam node of *Oenocarpus* |
| *Oenocarpus* | *Oenocarpus circumtextus* | Steam node of *Oenocarpus* |
| *Oenocarpus* | *Oenocarpus distichus* | Steam node of *Oenocarpus* |
| *Oenocarpus* | *Oenocarpus mapora* | Steam node of *Oenocarpus* |
| *Oenocarpus* | *Oenocarpus minor* | Steam node of *Oenocarpus* |
| *Oenocarpus* | *Oenocarpus simplex* | Steam node of *Oenocarpus* |
| *Parajubaea* | *Parajubaea cocoides* | Steam node of *Cocos* |
| *Parajubaea* | *Parajubaea torallyi* | Steam node of *Cocos* |
| *Pholidostachys* | *Pholidostachys dactyloides* | - |
| *Pholidostachys* | *Pholidostachys kalbreyeri* | - |
| *Pholidostachys* | *Pholidostachys pulchra* | - |
| *Pholidostachys* | *Pholidostachys synanthera* | - |
| *Phytelephas* | *Phytelephas aequatorialis* | - |
| *Phytelephas* | *Phytelephas macrocarpa* | - |
| *Phytelephas* | *Phytelephas schottii** | - |
| *Phytelephas* | *Phytelephas seemannii* | - |
| *Phytelephas* | *Phytelephas tenuicaulis* | - |
| *Phytelephas* | *Phytelephas tumacana* | - |
| *Prestoea* | *Prestoea acuminata* | Crown node of *Prestoea* |
| *Prestoea* | *Prestoea carderi* | Crown node of *Prestoea* |
| *Prestoea* | *Prestoea decurrens* | - |
| *Prestoea* | *Prestoea ensiformis* | Crown node of *Prestoea* |
| *Prestoea* | *Prestoea longipetiolata* | Crown node of *Prestoea* |
| *Prestoea* | *Prestoea pubens* | - |
| *Prestoea* | *Prestoea pubigera* | Crown node of *Prestoea* |
| *Prestoea* | *Prestoea schultzeana* | Crown node of *Prestoea* |
| *Prestoea* | *Prestoea simplicifolia* | Crown node of *Prestoea* |
| *Prestoea* | *Prestoea tenuiramosa* | Crown node of *Prestoea* |
| *Pseudophoenix* | *Pseudophoenix ekmanii** | - |
| *Pseudophoenix* | *Pseudophoenix lediniana** | - |
| *Pseudophoenix* | *Pseudophoenix sargentii* | - |
| *Pseudophoenix* | *Pseudophoenix vinifera* | - |
| *Raphia* | *Raphia taedigera* | Crown node of Lepidocaryeae |
| *Reinhardtia* | *Reinhardtia elegans* | Crown node of *Reinhardtia* |
| *Reinhardtia* | *Reinhardtia gracilis* | - |
| *Reinhardtia* | *Reinhardtia koschnyana* | Crown node of *Reinhardtia* |
| *Reinhardtia* | *Reinhardtia latisecta* | Crown node of *Reinhardtia* |
| *Reinhardtia* | *Reinhardtia paiewonskiana* | Crown node of *Reinhardtia* |
| *Reinhardtia* | *Reinhardtia simplex* | - |
| *Rhapidophyllum* | *Rhapidophyllum hystrix* | - |
| *Roystonea* | *Roystonea altissima* | Crown node of *Roystonea* |
| *Roystonea* | *Roystonea borinquena* | - |
| *Roystonea* | *Roystonea dunlapiana* | Crown node of *Roystonea* |
| *Roystonea* | *Roystonea lenis* | Crown node of *Roystonea* |
| *Roystonea* | *Roystonea oleracea* | - |
| *Roystonea* | *Roystonea princeps* | Crown node of *Roystonea* |
| *Roystonea* | *Roystonea regia* | - |
| *Roystonea* | *Roystonea violacea* | Crown node of *Roystonea* |
| *Sabal* | *Sabal bermudana** | - |
| *Sabal* | *Sabal causiarum* | Crown node of *Sabal* |
| *Sabal* | *Sabal domingensis* | Crown node of *Sabal* |
| *Sabal* | *Sabal etonia* | - |
| *Sabal* | *Sabal gretherae* | Crown node of *Sabal* |
| *Sabal* | *Sabal maritima* | Crown node of *Sabal* |
| *Sabal* | *Sabal mauritiiformis* | Crown node of *Sabal* |
| *Sabal* | *Sabal mexicana* | Crown node of *Sabal* |
| *Sabal* | *Sabal minor* | - |
| *Sabal* | *Sabal palmetto* | - |
| *Sabal* | *Sabal pumos* | Crown node of *Sabal* |
| *Sabal* | *Sabal rosei* | Crown node of *Sabal* |
| *Sabal* | *Sabal uresana* | Crown node of *Sabal* |
| *Sabal* | *Sabal yapa* | Crown node of *Sabal* |
| *Schippia* | *Schippia concolor* | - |
| *Serenoa* | *Serenoa repens* | - |
| *Socratea* | *Socratea exorrhiza* | - |
| *Socratea* | *Socratea hecatonandra* | - |
| *Socratea* | *Socratea karstenii** | - |
| *Socratea* | *Socratea montana* | Crown node of *Socratea* |
| *Socratea* | *Socratea rostrata* | - |
| *Socratea* | *Socratea salazarii* | - |
| *Syagrus* | *Syagrus amara* | Crown node of *Syagrus* |
| *Syagrus* | *Syagrus botryophora* | Crown node of *Syagrus* |
| *Syagrus* | *Syagrus campylospatha* | Crown node of *Syagrus* |
| *Syagrus* | *Syagrus cardenasii* | Crown node of *Syagrus* |
| *Syagrus* | *Syagrus cocoides* | Crown node of *Syagrus* |
| *Syagrus* | *Syagrus comosa* | Crown node of *Syagrus* |
| *Syagrus* | *Syagrus coronata* | Crown node of *Syagrus* |
| *Syagrus* | *Syagrus duartei* | Crown node of *Syagrus* |
| *Syagrus* | *Syagrus flexuosa* | Crown node of *Syagrus* |
| *Syagrus* | *Syagrus glaucescens* | Crown node of *Syagrus* |
| *Syagrus* | *Syagrus graminifolia* | Crown node of *Syagrus* |
| *Syagrus* | *Syagrus harleyi* | Crown node of *Syagrus* |
| *Syagrus* | *Syagrus inajai* | Crown node of *Syagrus* |
| *Syagrus* | *Syagrus macrocarpa* | Crown node of *Syagrus* |
| *Syagrus* | *Syagrus microphylla* | Crown node of *Syagrus* |
| *Syagrus* | *Syagrus oleracea* | Crown node of *Syagrus* |
| *Syagrus* | *Syagrus orinocensis* | Crown node of *Syagrus* |
| *Syagrus* | *Syagrus petraea* | Crown node of *Syagrus* |
| *Syagrus* | *Syagrus picrophylla* | Crown node of *Syagrus* |
| *Syagrus* | *Syagrus pleioclada* | Crown node of *Syagrus* |
| *Syagrus* | *Syagrus pseudococos* | Crown node of *Syagrus* |
| *Syagrus* | *Syagrus romanzoffiana* | - |
| *Syagrus* | *Syagrus ruschiana* | Crown node of *Syagrus* |
| *Syagrus* | *Syagrus sancona* | Crown node of *Syagrus* |
| *Syagrus* | *Syagrus schizophylla* | Crown node of *Syagrus* |
| *Syagrus* | *Syagrus smithii* | - |
| *Syagrus* | *Syagrus stratincola* | Crown node of *Syagrus* |
| *Syagrus* | *Syagrus vagans* | Crown node of *Syagrus* |
| *Syagrus* | *Syagrus werdermannii* | Crown node of *Syagrus* |
| *Synechanthus* | *Synechanthus fibrosus* | - |
| *Synechanthus* | *Synechanthus warscewiczianus* | - |
| *Thrinax* | *Thrinax ekmaniana** | - |
| *Thrinax* | *Thrinax excelsa** | - |
| *Thrinax* | *Thrinax morrisii** | - |
| *Thrinax* | *Thrinax parviflora* | - |
| *Thrinax* | *Thrinax radiata* | - |
| *Trithrinax* | *Trithrinax acanthocoma** | - |
| *Trithrinax* | *Trithrinax biflabellata** | - |
| *Trithrinax* | *Trithrinax brasiliensis* | - |
| *Trithrinax* | *Trithrinax campestris* | - |
| *Trithrinax* | *Trithrinax schizophylla* | - |
| *Washingtonia* | *Washingtonia filifera* | - |
| *Washingtonia* | *Washingtonia robusta* | - |
| *Welfia* | *Welfia regia* | - |
| *Wendlandiella* | *Wendlandiella gracilis* | - |
| *Wettinia* | *Wettinia aequalis* | Crown node of *Wettinia* |
| *Wettinia* | *Wettinia aequatorialis* | Crown node of *Wettinia* |
| *Wettinia* | *Wettinia anomala* | - |
| *Wettinia* | *Wettinia augusta* | - |
| *Wettinia* | *Wettinia castanea* | - |
| *Wettinia* | *Wettinia disticha* | - |
| *Wettinia* | *Wettinia drudei* | - |
| *Wettinia* | *Wettinia equalis** | - |
| *Wettinia* | *Wettinia fascicularis* | - |
| *Wettinia* | *Wettinia hirsuta* | - |
| *Wettinia* | *Wettinia kalbreyeri* | - |
| *Wettinia* | *Wettinia lanata* | - |
| *Wettinia* | *Wettinia longipetala* | - |
| *Wettinia* | *Wettinia maynensis* | - |
| *Wettinia* | *Wettinia microcarpa* | - |
| *Wettinia* | *Wettinia minima* | Crown node of *Wettinia* |
| *Wettinia* | *Wettinia oxycarpa* | Crown node of *Wettinia* |
| *Wettinia* | *Wettinia praemorsa* | - |
| *Wettinia* | *Wettinia quinaria* | - |
| *Wettinia* | *Wettinia radiata* | - |
| *Wettinia* | *Wettinia verruculosa* | Crown node of *Wettinia* |
| *Zombia* | *Zombia antillarum* | - |

**Table S2**. Correspondence between the original WWF classification of biome and the present study re-classification of bioregions.

| **Biome** | **Bioregion** | **Code** |
| --- | --- | --- |
| Tropical & Subtropical Moist Broadleaf Forests | Atlantic Coastal forest | ACF |
| Tropical & Subtropical Moist Broadleaf Forests | Amazon | AMA |
| Tropical & Subtropical Coniferous Forests | Central American moist forest | CMF |
| Tropical & Subtropical Dry Broadleaf Forests | Central American moist forest | CMF |
| Tropical & Subtropical Moist Broadleaf Forests | Central American moist forest | CMF |
| Deserts & Xeric Shrublands | Caatinga | CAA |
| Deserts & Xeric Shrublands | Caribbean | CAR |
| Flooded Grasslands & Savannas | Caribbean | CAR |
| Tropical & Subtropical Coniferous Forests | Caribbean | CAR |
| Tropical & Subtropical Moist Broadleaf Forests | Caribbean | CAR |
| Deserts & Xeric Shrublands | Caribbean dry frorest | CDF |
| Tropical & Subtropical Moist Broadleaf Forests | Caribbean dry frorest | CDF |
| Deserts & Xeric Shrublands | Central Andean | CAN |
| Montane Grasslands & Shrublands | Central Andean | CAN |
| Temperate Grasslands, Savannas & Shrublands | Central Andean | CAN |
| Tropical & Subtropical Dry Broadleaf Forests | Central Andean | CAN |
| Tropical & Subtropical Moist Broadleaf Forests | Central Andean | CAN |
| Deserts & Xeric Shrublands | Cerrado | CER |
| Tropical & Subtropical Grasslands, Savannas & Shrublands | Cerrado | CER |
| Tropical & Subtropical Moist Broadleaf Forests | Cerrado | CER |
| Flooded Grasslands & Savannas | Chaco and espinal | CHE |
| Montane Grasslands & Shrublands | Chaco and espinal | CHE |
| Temperate Grasslands, Savannas & Shrublands | Chaco and espinal | CHE |
| Tropical & Subtropical Grasslands, Savannas & Shrublands | Chaco and espinal | CHE |
| Tropical & Subtropical Moist Broadleaf Forests | Chaco and espinal | CHE |
| Flooded Grasslands & Savannas | Choco | CHO |
| Tropical & Subtropical Dry Broadleaf Forests | Choco | CHO |
| Tropical & Subtropical Moist Broadleaf Forests | Choco | CHO |
| Tropical & Subtropical Grasslands, Savannas & Shrublands | Grassland and Pampa | GRP |
| Flooded Grasslands & Savannas | Guiana Shield | GSH |
| Tropical & Subtropical Grasslands, Savannas & Shrublands | Guiana Shield | GSH |
| Tropical & Subtropical Moist Broadleaf Forests | Guiana Shield | GSH |
| Tropical & Subtropical Dry Broadleaf Forests | Inter-Andean forests | IAF |
| Tropical & Subtropical Moist Broadleaf Forests | Inter-Andean forests | IAF |
| Deserts & Xeric Shrublands | Llanos | LLA |
| Tropical & Subtropical Dry Broadleaf Forests | Llanos | LLA |
| Tropical & Subtropical Grasslands, Savannas & Shrublands | Llanos | LLA |
| Tropical & Subtropical Moist Broadleaf Forests | Llanos | LLA |
| Montane Grasslands & Shrublands | Northern Andes | NAN |
| Tropical & Subtropical Dry Broadleaf Forests | Northern Andes | NAN |
| Tropical & Subtropical Moist Broadleaf Forests | Northern Andes | NAN |
| Flooded Grasslands & Savannas | Pantanal | PAN |
| Flooded Grasslands & Savannas | Southeaster United States | SEU |
| Temperate Conifer Forests | Southeaster United States | SEU |
| Tropical & Subtropical Grasslands, Savannas & Shrublands | Southeaster United States | SEU |
| Tropical & Subtropical Moist Broadleaf Forests | Southeaster United States | SEU |
| Deserts & Xeric Shrublands | Tropical Central American dry forests | TAM |
| Tropical & Subtropical Coniferous Forests | Tropical Central American dry forests | TAM |
| Tropical & Subtropical Dry Broadleaf Forests | Tropical Central American dry forests | TAM |
| Tropical & Subtropical Moist Broadleaf Forests | Tropical Central American dry forests | TAM |
| Tropical & Subtropical Dry Broadleaf Forests | Western Amazon | WAM |
| Tropical & Subtropical Moist Broadleaf Forests | Western Amazon | WAM |
| Deserts & Xeric Shrublands | Xeric Mesoamerica | XMA |
| Mediterranean Forests, Woodlands & Scrub | Xeric Mesoamerica | XMA |
| Temperate Conifer Forests | Xeric Mesoamerica | XMA |
| Tropical & Subtropical Coniferous Forests | Xeric Mesoamerica | XMA |
| Tropical & Subtropical Dry Broadleaf Forests | Xeric Mesoamerica | XMA |
| Tropical & Subtropical Grasslands, Savannas & Shrublands | Xeric Mesoamerica | XMA |

**Table S3**. Bioregion matrix as a result of a cross match between the species list for the American palms generated by Gödel et al. (2015) and the biome map available on the WWF website. ACF, Atlantic Coastal forest; AMA, Amazon, CMF, Central America moist forest; CAA, Caatinga; CAR, Caribbean CDF, Caribbean dry forest; CAN, Cental Andes; CER, Cerrado; CHE, Chaco and Espinal; CHO, Choco; GRP, Grassland and Pampa; GSH, Guiana Shield; IAF, Inter-Andean forest; LLA, Llanos; NAN, Northern Andes; PAN, Pantanal; SEU, Southeastern America; TAM, Tropical Central American dry forests; WAM, Western Amazon; XMA, Xeric Mesoamerica.

| **Species** | **ACF** | **AMA** | **CMF** | **CAA** | **CAR** | **CDF** | **CAN** | **CER** | **CHE** | **CHO** | **GRP** | **GSH** | **IAF** | **LLA** | **NAN** | **PAN** | **SEU** | **TAM** | **WAM** | **XMA** |
| --- | --- | --- | --- | --- | --- | --- | --- | --- | --- | --- | --- | --- | --- | --- | --- | --- | --- | --- | --- | --- |
| *Acoelorrhaphe wrightii* | 0 | 0 | 1 | 0 | 1 | 0 | 0 | 0 | 0 | 0 | 0 | 0 | 0 | 0 | 0 | 0 | 1 | 1 | 0 | 0 |
| *Acrocomia aculeata* | 1 | 1 | 1 | 1 | 1 | 1 | 1 | 1 | 1 | 1 | 0 | 1 | 1 | 1 | 1 | 1 | 1 | 1 | 1 | 0 |
| *Acrocomia hassleri* | 1 | 0 | 0 | 1 | 0 | 0 | 0 | 1 | 0 | 0 | 0 | 0 | 0 | 0 | 0 | 0 | 0 | 0 | 0 | 0 |
| *Aiphanes acaulis* | 0 | 0 | 0 | 0 | 0 | 0 | 0 | 0 | 0 | 1 | 0 | 0 | 0 | 0 | 0 | 0 | 0 | 0 | 0 | 0 |
| *Aiphanes chiribogensis* | 0 | 0 | 0 | 0 | 0 | 0 | 0 | 0 | 0 | 1 | 0 | 0 | 0 | 0 | 1 | 0 | 0 | 0 | 0 | 0 |
| *Aiphanes deltoidea* | 0 | 0 | 0 | 0 | 0 | 0 | 0 | 0 | 0 | 0 | 0 | 0 | 0 | 0 | 0 | 0 | 0 | 0 | 1 | 0 |
| *Aiphanes eggersii* | 0 | 0 | 0 | 0 | 0 | 0 | 0 | 0 | 0 | 1 | 0 | 0 | 0 | 0 | 1 | 0 | 0 | 0 | 0 | 0 |
| *Aiphanes erinacea* | 0 | 0 | 0 | 0 | 0 | 0 | 0 | 0 | 0 | 0 | 0 | 0 | 0 | 0 | 1 | 0 | 0 | 0 | 0 | 0 |
| *Aiphanes gelatinosa* | 0 | 0 | 0 | 0 | 0 | 0 | 0 | 0 | 0 | 0 | 0 | 0 | 0 | 0 | 1 | 0 | 0 | 0 | 0 | 0 |
| *Aiphanes grandis* | 0 | 0 | 0 | 0 | 0 | 0 | 0 | 0 | 0 | 0 | 0 | 0 | 0 | 0 | 1 | 0 | 0 | 0 | 0 | 0 |
| *Aiphanes hirsuta* | 0 | 0 | 1 | 0 | 0 | 0 | 0 | 0 | 0 | 1 | 0 | 0 | 0 | 0 | 1 | 0 | 0 | 0 | 0 | 0 |
| *Aiphanes leiostachys* | 0 | 0 | 0 | 0 | 0 | 0 | 0 | 0 | 0 | 0 | 0 | 0 | 0 | 0 | 1 | 0 | 0 | 0 | 0 | 0 |
| *Aiphanes lindeniana* | 0 | 0 | 0 | 0 | 0 | 0 | 0 | 0 | 0 | 0 | 0 | 0 | 0 | 0 | 1 | 0 | 0 | 0 | 0 | 0 |
| *Aiphanes linearis* | 0 | 0 | 0 | 0 | 0 | 0 | 0 | 0 | 0 | 0 | 0 | 0 | 0 | 1 | 1 | 0 | 0 | 0 | 0 | 0 |
| *Aiphanes macroloba* | 0 | 0 | 0 | 0 | 0 | 0 | 0 | 0 | 0 | 1 | 0 | 0 | 0 | 0 | 1 | 0 | 0 | 0 | 0 | 0 |
| *Aiphanes minima* | 0 | 0 | 0 | 0 | 1 | 0 | 0 | 0 | 0 | 0 | 0 | 0 | 0 | 0 | 0 | 0 | 0 | 0 | 0 | 0 |
| *Aiphanes parvifolia* | 0 | 0 | 0 | 0 | 0 | 0 | 0 | 0 | 0 | 0 | 0 | 0 | 0 | 0 | 1 | 0 | 0 | 0 | 0 | 0 |
| *Aiphanes simplex* | 0 | 0 | 0 | 0 | 0 | 0 | 0 | 0 | 0 | 0 | 0 | 0 | 0 | 0 | 1 | 0 | 0 | 0 | 0 | 0 |
| *Aiphanes spicata* | 0 | 0 | 0 | 0 | 0 | 0 | 1 | 0 | 0 | 0 | 0 | 0 | 0 | 0 | 0 | 0 | 0 | 0 | 1 | 0 |
| *Aiphanes tricuspidata* | 0 | 0 | 0 | 0 | 0 | 0 | 1 | 0 | 0 | 1 | 0 | 0 | 0 | 0 | 1 | 0 | 0 | 0 | 0 | 0 |
| *Aiphanes ulei* | 0 | 0 | 0 | 0 | 0 | 0 | 0 | 0 | 0 | 0 | 0 | 0 | 0 | 0 | 1 | 0 | 0 | 0 | 1 | 0 |
| *Aiphanes verrucosa* | 0 | 0 | 0 | 0 | 0 | 0 | 0 | 0 | 0 | 0 | 0 | 0 | 0 | 0 | 1 | 0 | 0 | 0 | 0 | 0 |
| *Aiphanes weberbaueri* | 0 | 0 | 0 | 0 | 0 | 0 | 1 | 0 | 0 | 1 | 0 | 0 | 0 | 0 | 1 | 0 | 0 | 0 | 1 | 0 |
| *Allagoptera arenaria* | 1 | 0 | 0 | 1 | 0 | 0 | 0 | 1 | 0 | 0 | 0 | 0 | 0 | 0 | 0 | 0 | 0 | 0 | 0 | 0 |
| *Allagoptera brevicalyx* | 1 | 0 | 0 | 1 | 0 | 0 | 0 | 1 | 0 | 0 | 0 | 0 | 0 | 0 | 0 | 0 | 0 | 0 | 0 | 0 |
| *Allagoptera campestris* | 1 | 0 | 0 | 1 | 0 | 0 | 0 | 1 | 1 | 0 | 0 | 0 | 0 | 0 | 0 | 0 | 0 | 0 | 0 | 0 |
| *Allagoptera caudescens* | 1 | 0 | 0 | 1 | 0 | 0 | 0 | 0 | 0 | 0 | 0 | 0 | 0 | 0 | 0 | 0 | 0 | 0 | 0 | 0 |
| *Allagoptera leucocalyx* | 1 | 1 | 0 | 1 | 0 | 0 | 1 | 1 | 1 | 0 | 0 | 0 | 0 | 0 | 0 | 1 | 0 | 0 | 1 | 0 |
| *Ammandra decasperma* | 0 | 0 | 0 | 0 | 0 | 0 | 0 | 0 | 0 | 1 | 0 | 0 | 0 | 0 | 1 | 0 | 0 | 0 | 1 | 0 |
| *Aphandra natalia* | 0 | 0 | 0 | 0 | 0 | 0 | 0 | 0 | 0 | 0 | 0 | 0 | 0 | 0 | 1 | 0 | 0 | 0 | 1 | 0 |
| *Asterogyne guianensis* | 0 | 0 | 0 | 0 | 0 | 0 | 0 | 0 | 0 | 0 | 0 | 1 | 0 | 0 | 0 | 0 | 0 | 0 | 0 | 0 |
| *Asterogyne martiana* | 0 | 0 | 1 | 0 | 0 | 0 | 0 | 0 | 0 | 1 | 0 | 0 | 0 | 0 | 1 | 0 | 0 | 1 | 0 | 0 |
| *Asterogyne ramosa* | 0 | 0 | 0 | 0 | 0 | 0 | 0 | 0 | 0 | 0 | 0 | 0 | 0 | 1 | 0 | 0 | 0 | 0 | 0 | 0 |
| *Astrocaryum acaule* | 0 | 1 | 0 | 0 | 0 | 0 | 0 | 0 | 0 | 0 | 0 | 1 | 0 | 1 | 1 | 0 | 0 | 0 | 1 | 0 |
| *Astrocaryum aculeatissimum* | 1 | 0 | 0 | 0 | 0 | 0 | 0 | 1 | 0 | 0 | 0 | 0 | 0 | 0 | 0 | 0 | 0 | 0 | 0 | 0 |
| *Astrocaryum aculeatum* | 0 | 1 | 0 | 0 | 0 | 0 | 0 | 1 | 0 | 0 | 0 | 1 | 0 | 1 | 0 | 0 | 0 | 0 | 1 | 0 |
| *Astrocaryum alatum* | 0 | 0 | 1 | 0 | 0 | 0 | 0 | 0 | 0 | 0 | 0 | 0 | 0 | 0 | 0 | 0 | 0 | 1 | 0 | 0 |
| *Astrocaryum campestre* | 0 | 1 | 0 | 1 | 0 | 0 | 0 | 1 | 0 | 0 | 0 | 0 | 0 | 0 | 0 | 0 | 0 | 0 | 0 | 0 |
| *Astrocaryum chambira* | 0 | 1 | 0 | 0 | 0 | 0 | 0 | 0 | 0 | 0 | 0 | 0 | 0 | 0 | 1 | 0 | 0 | 0 | 1 | 0 |
| *Astrocaryum confertum* | 0 | 0 | 1 | 0 | 0 | 0 | 0 | 0 | 0 | 1 | 0 | 0 | 0 | 0 | 0 | 0 | 0 | 0 | 0 | 0 |
| *Astrocaryum huaimi* | 0 | 1 | 0 | 0 | 0 | 0 | 0 | 1 | 0 | 0 | 0 | 0 | 0 | 0 | 0 | 0 | 0 | 0 | 1 | 0 |
| *Astrocaryum jauari* | 0 | 1 | 0 | 0 | 0 | 0 | 0 | 1 | 0 | 0 | 0 | 1 | 0 | 1 | 0 | 0 | 0 | 0 | 1 | 0 |
| *Astrocaryum malybo* | 0 | 0 | 0 | 0 | 0 | 1 | 0 | 0 | 0 | 1 | 0 | 0 | 1 | 0 | 1 | 0 | 0 | 0 | 0 | 0 |
| *Astrocaryum mexicanum* | 0 | 0 | 1 | 0 | 0 | 0 | 0 | 0 | 0 | 0 | 0 | 0 | 0 | 0 | 0 | 0 | 0 | 1 | 0 | 0 |
| *Astrocaryum murumuru* | 0 | 1 | 0 | 0 | 0 | 0 | 1 | 1 | 0 | 0 | 0 | 1 | 0 | 0 | 0 | 0 | 0 | 0 | 1 | 0 |
| *Astrocaryum paramaca* | 0 | 1 | 0 | 0 | 0 | 0 | 0 | 1 | 0 | 0 | 0 | 1 | 0 | 0 | 0 | 0 | 0 | 0 | 0 | 0 |
| *Astrocaryum sciophilum* | 0 | 1 | 0 | 0 | 0 | 0 | 0 | 0 | 0 | 0 | 0 | 1 | 0 | 0 | 0 | 0 | 0 | 0 | 0 | 0 |
| *Astrocaryum standleyanum* | 0 | 0 | 1 | 0 | 0 | 0 | 0 | 0 | 0 | 1 | 0 | 0 | 0 | 1 | 1 | 0 | 0 | 0 | 0 | 0 |
| *Astrocaryum triandrum* | 0 | 0 | 0 | 0 | 0 | 0 | 0 | 0 | 0 | 0 | 0 | 0 | 0 | 0 | 1 | 0 | 0 | 0 | 0 | 0 |
| *Astrocaryum vulgare* | 1 | 1 | 0 | 1 | 0 | 0 | 0 | 1 | 0 | 0 | 0 | 1 | 0 | 0 | 0 | 0 | 0 | 0 | 0 | 0 |
| *Attalea allenii* | 0 | 0 | 1 | 0 | 0 | 0 | 0 | 0 | 0 | 1 | 0 | 0 | 0 | 0 | 1 | 0 | 0 | 0 | 0 | 0 |
| *Attalea amygdalina* | 0 | 0 | 0 | 0 | 0 | 0 | 0 | 0 | 0 | 0 | 0 | 0 | 1 | 0 | 1 | 0 | 0 | 0 | 0 | 0 |
| *Attalea attaleoides* | 0 | 1 | 0 | 0 | 0 | 0 | 0 | 0 | 0 | 0 | 0 | 1 | 0 | 0 | 0 | 0 | 0 | 0 | 1 | 0 |
| *Attalea butyracea* | 0 | 1 | 1 | 0 | 0 | 1 | 1 | 1 | 0 | 1 | 0 | 0 | 1 | 1 | 1 | 0 | 0 | 1 | 1 | 0 |
| *Attalea cohune* | 0 | 0 | 1 | 0 | 0 | 0 | 0 | 0 | 0 | 1 | 0 | 0 | 0 | 0 | 1 | 0 | 0 | 1 | 0 | 0 |
| *Attalea colenda* | 0 | 0 | 0 | 0 | 0 | 0 | 0 | 0 | 0 | 1 | 0 | 0 | 0 | 0 | 0 | 0 | 0 | 0 | 1 | 0 |
| *Attalea crassispatha* | 0 | 0 | 0 | 0 | 1 | 0 | 0 | 0 | 0 | 0 | 0 | 0 | 0 | 0 | 0 | 0 | 0 | 0 | 0 | 0 |
| *Attalea cuatrecasana* | 0 | 0 | 0 | 0 | 0 | 0 | 0 | 0 | 0 | 1 | 0 | 0 | 0 | 0 | 1 | 0 | 0 | 0 | 0 | 0 |
| *Attalea dahlgreniana* | 0 | 1 | 0 | 0 | 0 | 0 | 0 | 0 | 0 | 0 | 0 | 1 | 0 | 0 | 0 | 0 | 0 | 0 | 0 | 0 |
| *Attalea dubia* | 1 | 0 | 0 | 0 | 0 | 0 | 0 | 0 | 0 | 0 | 0 | 0 | 0 | 0 | 0 | 0 | 0 | 0 | 0 | 0 |
| *Attalea eichleri* | 1 | 1 | 0 | 0 | 0 | 0 | 0 | 1 | 0 | 0 | 0 | 0 | 0 | 0 | 0 | 1 | 0 | 0 | 0 | 0 |
| *Attalea exigua* | 1 | 0 | 0 | 0 | 0 | 0 | 0 | 1 | 0 | 0 | 0 | 0 | 0 | 0 | 0 | 0 | 0 | 0 | 0 | 0 |
| *Attalea funifera* | 1 | 0 | 0 | 1 | 0 | 0 | 0 | 0 | 0 | 0 | 0 | 0 | 0 | 0 | 0 | 0 | 0 | 0 | 0 | 0 |
| *Attalea geraensis* | 1 | 0 | 0 | 0 | 0 | 0 | 0 | 1 | 1 | 0 | 0 | 0 | 0 | 0 | 0 | 0 | 0 | 0 | 0 | 0 |
| *Attalea humilis* | 1 | 0 | 0 | 0 | 0 | 0 | 0 | 0 | 0 | 0 | 0 | 0 | 0 | 0 | 0 | 0 | 0 | 0 | 0 | 0 |
| *Attalea iguadummat* | 0 | 0 | 1 | 0 | 0 | 0 | 0 | 0 | 0 | 1 | 0 | 0 | 0 | 0 | 0 | 0 | 0 | 0 | 0 | 0 |
| *Attalea insignis* | 0 | 1 | 0 | 0 | 0 | 0 | 0 | 0 | 0 | 0 | 0 | 0 | 0 | 1 | 0 | 0 | 0 | 0 | 1 | 0 |
| *Attalea luetzelburgii* | 0 | 1 | 0 | 0 | 0 | 0 | 0 | 0 | 0 | 0 | 0 | 0 | 0 | 0 | 0 | 0 | 0 | 0 | 1 | 0 |
| *Attalea maripa* | 0 | 1 | 0 | 0 | 0 | 0 | 0 | 1 | 0 | 0 | 0 | 1 | 0 | 1 | 0 | 0 | 0 | 0 | 1 | 0 |
| *Attalea microcarpa* | 0 | 1 | 0 | 0 | 0 | 0 | 0 | 0 | 0 | 0 | 0 | 1 | 0 | 1 | 0 | 0 | 0 | 0 | 1 | 0 |
| *Attalea nucifera* | 0 | 0 | 0 | 0 | 0 | 0 | 0 | 0 | 0 | 1 | 0 | 0 | 1 | 0 | 1 | 0 | 0 | 0 | 0 | 0 |
| *Attalea oleifera* | 1 | 0 | 0 | 1 | 0 | 0 | 0 | 1 | 0 | 0 | 0 | 0 | 0 | 0 | 0 | 0 | 0 | 0 | 0 | 0 |
| *Attalea phalerata* | 1 | 1 | 0 | 0 | 0 | 0 | 1 | 1 | 1 | 0 | 0 | 0 | 0 | 0 | 0 | 1 | 0 | 0 | 1 | 0 |
| *Attalea pindobassu* | 1 | 0 | 0 | 1 | 0 | 0 | 0 | 1 | 0 | 0 | 0 | 0 | 0 | 0 | 0 | 0 | 0 | 0 | 0 | 0 |
| *Attalea racemosa* | 0 | 1 | 0 | 0 | 0 | 0 | 0 | 0 | 0 | 0 | 0 | 1 | 0 | 1 | 1 | 0 | 0 | 0 | 1 | 0 |
| *Attalea speciosa* | 0 | 1 | 0 | 1 | 0 | 0 | 0 | 1 | 0 | 0 | 0 | 1 | 0 | 0 | 0 | 1 | 0 | 0 | 1 | 0 |
| *Attalea spectabilis* | 0 | 1 | 0 | 0 | 0 | 0 | 0 | 1 | 0 | 0 | 0 | 0 | 0 | 0 | 0 | 0 | 0 | 0 | 1 | 0 |
| *Attalea tessmannii* | 0 | 0 | 1 | 0 | 0 | 0 | 0 | 0 | 0 | 0 | 0 | 0 | 0 | 0 | 0 | 0 | 0 | 0 | 1 | 0 |
| *Bactris acanthocarpa* | 1 | 1 | 0 | 0 | 0 | 0 | 1 | 1 | 0 | 0 | 0 | 1 | 0 | 1 | 0 | 0 | 0 | 0 | 1 | 0 |
| *Bactris acanthocarpoides* | 0 | 1 | 0 | 0 | 0 | 0 | 0 | 1 | 0 | 0 | 0 | 1 | 0 | 0 | 0 | 0 | 0 | 0 | 1 | 0 |
| *Bactris aubletiana* | 0 | 1 | 0 | 0 | 0 | 0 | 0 | 0 | 0 | 0 | 0 | 1 | 0 | 0 | 0 | 0 | 0 | 0 | 0 | 0 |
| *Bactris bahiensis* | 1 | 0 | 0 | 0 | 0 | 0 | 0 | 0 | 0 | 0 | 0 | 0 | 0 | 0 | 0 | 0 | 0 | 0 | 0 | 0 |
| *Bactris balanophora* | 0 | 1 | 0 | 0 | 0 | 0 | 0 | 1 | 0 | 0 | 0 | 1 | 0 | 0 | 0 | 0 | 0 | 0 | 1 | 0 |
| *Bactris barronis* | 0 | 0 | 1 | 0 | 0 | 0 | 0 | 0 | 0 | 1 | 0 | 0 | 0 | 0 | 1 | 0 | 0 | 0 | 0 | 0 |
| *Bactris bidentula* | 0 | 1 | 0 | 0 | 0 | 0 | 0 | 0 | 0 | 0 | 0 | 0 | 0 | 1 | 0 | 0 | 0 | 0 | 1 | 0 |
| *Bactris bifida* | 0 | 1 | 0 | 0 | 0 | 0 | 0 | 1 | 0 | 0 | 0 | 0 | 0 | 0 | 0 | 0 | 0 | 0 | 1 | 0 |
| *Bactris brongniartii* | 1 | 1 | 0 | 0 | 0 | 0 | 0 | 1 | 0 | 1 | 0 | 1 | 0 | 0 | 0 | 0 | 0 | 0 | 1 | 0 |
| *Bactris campestris* | 0 | 1 | 0 | 0 | 0 | 0 | 0 | 0 | 0 | 0 | 0 | 1 | 0 | 0 | 0 | 0 | 0 | 0 | 1 | 0 |
| *Bactris caryotifolia* | 1 | 0 | 0 | 0 | 0 | 0 | 0 | 1 | 0 | 0 | 0 | 0 | 0 | 0 | 0 | 0 | 0 | 0 | 0 | 0 |
| *Bactris caudata* | 0 | 0 | 1 | 0 | 0 | 0 | 0 | 0 | 0 | 0 | 0 | 0 | 0 | 0 | 0 | 0 | 0 | 0 | 0 | 0 |
| *Bactris charnleyae* | 0 | 0 | 1 | 0 | 0 | 0 | 0 | 0 | 0 | 1 | 0 | 0 | 0 | 0 | 0 | 0 | 0 | 0 | 0 | 0 |
| *Bactris coloniata* | 0 | 0 | 1 | 0 | 0 | 0 | 0 | 0 | 0 | 1 | 0 | 0 | 0 | 0 | 0 | 0 | 0 | 0 | 1 | 0 |
| *Bactris coloradonis* | 0 | 0 | 1 | 0 | 0 | 0 | 0 | 0 | 0 | 1 | 0 | 0 | 0 | 0 | 1 | 0 | 0 | 0 | 0 | 0 |
| *Bactris concinna* | 0 | 1 | 0 | 0 | 0 | 0 | 1 | 1 | 0 | 0 | 0 | 0 | 0 | 0 | 0 | 0 | 0 | 0 | 1 | 0 |
| *Bactris constanciae* | 0 | 1 | 0 | 0 | 0 | 0 | 0 | 0 | 0 | 0 | 0 | 1 | 0 | 0 | 0 | 0 | 0 | 0 | 0 | 0 |
| *Bactris corossilla* | 0 | 1 | 0 | 0 | 0 | 0 | 1 | 0 | 0 | 0 | 0 | 1 | 0 | 1 | 1 | 0 | 0 | 0 | 1 | 0 |
| *Bactris cuspidata* | 0 | 1 | 0 | 0 | 0 | 0 | 0 | 1 | 0 | 1 | 0 | 1 | 0 | 0 | 0 | 0 | 0 | 0 | 0 | 0 |
| *Bactris dianeura* | 0 | 0 | 1 | 0 | 0 | 0 | 0 | 0 | 0 | 0 | 0 | 0 | 0 | 0 | 0 | 0 | 0 | 1 | 0 | 0 |
| *Bactris elegans* | 0 | 1 | 0 | 0 | 0 | 0 | 0 | 0 | 0 | 0 | 0 | 1 | 0 | 0 | 0 | 0 | 0 | 0 | 1 | 0 |
| *Bactris ferruginea* | 1 | 0 | 0 | 1 | 0 | 0 | 0 | 0 | 0 | 0 | 0 | 0 | 0 | 0 | 0 | 0 | 0 | 0 | 0 | 0 |
| *Bactris fissifrons* | 0 | 1 | 0 | 0 | 0 | 0 | 1 | 1 | 0 | 0 | 0 | 0 | 0 | 0 | 0 | 0 | 0 | 0 | 1 | 0 |
| *Bactris gasipaes* | 1 | 1 | 1 | 0 | 0 | 1 | 1 | 1 | 0 | 1 | 0 | 1 | 1 | 1 | 1 | 0 | 0 | 0 | 1 | 0 |
| *Bactris gastoniana* | 0 | 1 | 0 | 0 | 0 | 0 | 0 | 0 | 0 | 0 | 0 | 1 | 0 | 0 | 0 | 0 | 0 | 0 | 1 | 0 |
| *Bactris glandulosa* | 0 | 0 | 1 | 0 | 0 | 0 | 0 | 0 | 0 | 1 | 0 | 0 | 0 | 0 | 1 | 0 | 0 | 1 | 0 | 0 |
| *Bactris glassmanii* | 1 | 0 | 0 | 0 | 0 | 0 | 0 | 1 | 0 | 0 | 0 | 0 | 0 | 0 | 0 | 0 | 0 | 0 | 0 | 0 |
| *Bactris glaucescens* | 1 | 1 | 0 | 0 | 0 | 0 | 0 | 1 | 1 | 0 | 0 | 0 | 0 | 0 | 0 | 1 | 0 | 0 | 1 | 0 |
| *Bactris gracilior* | 0 | 0 | 1 | 0 | 0 | 0 | 0 | 0 | 0 | 1 | 0 | 0 | 0 | 0 | 0 | 0 | 0 | 0 | 0 | 0 |
| *Bactris grayumii* | 0 | 0 | 1 | 0 | 0 | 0 | 0 | 0 | 0 | 0 | 0 | 0 | 0 | 0 | 0 | 0 | 0 | 0 | 0 | 0 |
| *Bactris guineensis* | 0 | 0 | 1 | 0 | 0 | 1 | 0 | 0 | 0 | 1 | 0 | 0 | 1 | 1 | 1 | 0 | 0 | 1 | 0 | 0 |
| *Bactris hatschbachii* | 1 | 0 | 0 | 0 | 0 | 0 | 0 | 0 | 0 | 0 | 0 | 0 | 0 | 0 | 0 | 0 | 0 | 0 | 0 | 0 |
| *Bactris hirta* | 1 | 1 | 0 | 1 | 0 | 0 | 1 | 1 | 0 | 0 | 0 | 1 | 0 | 0 | 0 | 0 | 0 | 0 | 1 | 0 |
| *Bactris hondurensis* | 0 | 0 | 1 | 0 | 0 | 0 | 0 | 0 | 0 | 1 | 0 | 0 | 0 | 0 | 1 | 0 | 0 | 1 | 0 | 0 |
| *Bactris horridispatha* | 1 | 0 | 0 | 1 | 0 | 0 | 0 | 0 | 0 | 0 | 0 | 0 | 0 | 0 | 0 | 0 | 0 | 0 | 0 | 0 |
| *Bactris killipii* | 0 | 1 | 0 | 0 | 0 | 0 | 1 | 0 | 0 | 0 | 0 | 0 | 0 | 0 | 0 | 0 | 0 | 0 | 1 | 0 |
| *Bactris kunorum* | 0 | 0 | 1 | 0 | 0 | 0 | 0 | 0 | 0 | 1 | 0 | 0 | 0 | 0 | 0 | 0 | 0 | 0 | 0 | 0 |
| *Bactris longiseta* | 0 | 0 | 1 | 0 | 0 | 0 | 0 | 0 | 0 | 0 | 0 | 0 | 0 | 0 | 0 | 0 | 0 | 0 | 0 | 0 |
| *Bactris macroacantha* | 0 | 1 | 0 | 0 | 0 | 0 | 0 | 0 | 0 | 0 | 0 | 0 | 0 | 0 | 1 | 0 | 0 | 0 | 1 | 0 |
| *Bactris major* | 0 | 1 | 1 | 1 | 0 | 1 | 1 | 1 | 1 | 1 | 0 | 1 | 1 | 1 | 1 | 1 | 0 | 1 | 1 | 0 |
| *Bactris mexicana* | 0 | 0 | 1 | 0 | 0 | 0 | 0 | 0 | 0 | 0 | 0 | 0 | 0 | 0 | 0 | 0 | 0 | 1 | 0 | 0 |
| *Bactris militaris* | 0 | 0 | 1 | 0 | 0 | 0 | 0 | 0 | 0 | 0 | 0 | 0 | 0 | 0 | 0 | 0 | 0 | 0 | 0 | 0 |
| *Bactris oligocarpa* | 0 | 1 | 0 | 0 | 0 | 0 | 0 | 0 | 0 | 0 | 0 | 1 | 0 | 0 | 0 | 0 | 0 | 0 | 1 | 0 |
| *Bactris oligoclada* | 0 | 1 | 0 | 0 | 0 | 0 | 0 | 0 | 0 | 0 | 0 | 1 | 0 | 0 | 0 | 0 | 0 | 0 | 0 | 0 |
| *Bactris panamensis* | 0 | 0 | 1 | 0 | 0 | 0 | 0 | 0 | 0 | 1 | 0 | 0 | 0 | 0 | 0 | 0 | 0 | 0 | 0 | 0 |
| *Bactris pickelii* | 1 | 0 | 0 | 1 | 0 | 0 | 0 | 0 | 0 | 0 | 0 | 0 | 0 | 0 | 0 | 0 | 0 | 0 | 0 | 0 |
| *Bactris pilosa* | 0 | 0 | 0 | 0 | 0 | 1 | 0 | 0 | 0 | 1 | 0 | 0 | 1 | 1 | 1 | 0 | 0 | 0 | 0 | 0 |
| *Bactris pliniana* | 0 | 1 | 0 | 0 | 0 | 0 | 0 | 0 | 0 | 0 | 0 | 1 | 0 | 0 | 0 | 0 | 0 | 0 | 1 | 0 |
| *Bactris plumeriana* | 0 | 0 | 0 | 0 | 1 | 0 | 0 | 0 | 0 | 0 | 0 | 0 | 0 | 0 | 0 | 0 | 1 | 0 | 0 | 0 |
| *Bactris ptariana* | 0 | 0 | 0 | 0 | 0 | 0 | 0 | 0 | 0 | 0 | 0 | 1 | 0 | 0 | 0 | 0 | 0 | 0 | 0 | 0 |
| *Bactris rhaphidacantha* | 0 | 1 | 0 | 0 | 0 | 0 | 0 | 0 | 0 | 0 | 0 | 1 | 0 | 0 | 0 | 0 | 0 | 0 | 0 | 0 |
| *Bactris riparia* | 0 | 1 | 0 | 0 | 0 | 0 | 0 | 1 | 0 | 0 | 0 | 0 | 0 | 0 | 0 | 0 | 0 | 0 | 1 | 0 |
| *Bactris setosa* | 1 | 0 | 0 | 1 | 0 | 0 | 0 | 1 | 0 | 0 | 1 | 0 | 0 | 0 | 0 | 1 | 0 | 0 | 0 | 0 |
| *Bactris setulosa* | 0 | 0 | 0 | 0 | 0 | 0 | 1 | 0 | 0 | 1 | 0 | 0 | 0 | 0 | 1 | 0 | 0 | 0 | 1 | 0 |
| *Bactris simplicifrons* | 0 | 1 | 0 | 0 | 0 | 0 | 1 | 1 | 0 | 0 | 0 | 1 | 0 | 1 | 1 | 0 | 0 | 0 | 1 | 0 |
| *Bactris soeiroana* | 1 | 0 | 0 | 0 | 0 | 0 | 0 | 0 | 0 | 0 | 0 | 0 | 0 | 0 | 0 | 0 | 0 | 0 | 0 | 0 |
| *Bactris syagroides* | 0 | 1 | 0 | 0 | 0 | 0 | 0 | 1 | 0 | 0 | 0 | 0 | 0 | 0 | 0 | 0 | 0 | 0 | 0 | 0 |
| *Bactris tefensis* | 0 | 1 | 0 | 0 | 0 | 0 | 0 | 0 | 0 | 0 | 0 | 0 | 0 | 0 | 0 | 0 | 0 | 0 | 0 | 0 |
| *Bactris tomentosa* | 0 | 1 | 0 | 0 | 0 | 0 | 0 | 1 | 0 | 0 | 0 | 1 | 0 | 0 | 0 | 0 | 0 | 0 | 1 | 0 |
| *Bactris turbinocarpa* | 0 | 1 | 0 | 0 | 0 | 0 | 0 | 0 | 0 | 0 | 0 | 0 | 0 | 0 | 0 | 0 | 0 | 0 | 0 | 0 |
| *Bactris vulgaris* | 1 | 0 | 0 | 0 | 0 | 0 | 0 | 1 | 0 | 0 | 0 | 0 | 0 | 0 | 0 | 0 | 0 | 0 | 0 | 0 |
| *Barcella odora* | 0 | 1 | 0 | 0 | 0 | 0 | 0 | 1 | 0 | 0 | 0 | 1 | 0 | 0 | 0 | 0 | 0 | 0 | 0 | 0 |
| *Brahea aculeata* | 0 | 0 | 0 | 0 | 0 | 0 | 0 | 0 | 0 | 0 | 0 | 0 | 0 | 0 | 0 | 0 | 0 | 1 | 0 | 0 |
| *Brahea armata* | 0 | 0 | 0 | 0 | 0 | 0 | 0 | 0 | 0 | 0 | 0 | 0 | 0 | 0 | 0 | 0 | 0 | 1 | 0 | 1 |
| *Brahea brandegeei* | 0 | 0 | 0 | 0 | 0 | 0 | 0 | 0 | 0 | 0 | 0 | 0 | 0 | 0 | 0 | 0 | 0 | 1 | 0 | 1 |
| *Brahea calcarea* | 0 | 0 | 1 | 0 | 0 | 0 | 0 | 0 | 0 | 0 | 0 | 0 | 0 | 0 | 0 | 0 | 0 | 1 | 0 | 1 |
| *Brahea decumbens* | 0 | 0 | 1 | 0 | 0 | 0 | 0 | 0 | 0 | 0 | 0 | 0 | 0 | 0 | 0 | 0 | 0 | 1 | 0 | 0 |
| *Brahea dulcis* | 0 | 0 | 1 | 0 | 0 | 0 | 0 | 0 | 0 | 0 | 0 | 0 | 0 | 0 | 0 | 0 | 0 | 1 | 0 | 1 |
| *Brahea edulis* | 0 | 0 | 0 | 0 | 0 | 0 | 0 | 0 | 0 | 0 | 0 | 0 | 0 | 0 | 0 | 0 | 0 | 0 | 0 | 1 |
| *Brahea moorei* | 0 | 0 | 1 | 0 | 0 | 0 | 0 | 0 | 0 | 0 | 0 | 0 | 0 | 0 | 0 | 0 | 0 | 1 | 0 | 0 |
| *Brahea pimo* | 0 | 0 | 0 | 0 | 0 | 0 | 0 | 0 | 0 | 0 | 0 | 0 | 0 | 0 | 0 | 0 | 0 | 1 | 0 | 0 |
| *Butia archeri* | 1 | 0 | 0 | 0 | 0 | 0 | 0 | 1 | 0 | 0 | 1 | 0 | 0 | 0 | 0 | 0 | 0 | 0 | 0 | 0 |
| *Butia campicola* | 1 | 0 | 0 | 0 | 0 | 0 | 0 | 1 | 1 | 0 | 0 | 0 | 0 | 0 | 0 | 0 | 0 | 0 | 0 | 0 |
| *Butia capitata* | 1 | 0 | 0 | 0 | 0 | 0 | 0 | 1 | 0 | 0 | 1 | 0 | 0 | 0 | 0 | 0 | 1 | 0 | 0 | 0 |
| *Butia eriospatha* | 1 | 0 | 0 | 0 | 0 | 0 | 0 | 1 | 0 | 0 | 1 | 0 | 0 | 0 | 0 | 0 | 0 | 0 | 0 | 0 |
| *Butia leptospatha* | 1 | 0 | 0 | 0 | 0 | 0 | 0 | 1 | 0 | 0 | 0 | 0 | 0 | 0 | 0 | 0 | 0 | 0 | 0 | 0 |
| *Butia microspadix* | 1 | 0 | 0 | 0 | 0 | 0 | 0 | 1 | 0 | 0 | 0 | 0 | 0 | 0 | 0 | 0 | 0 | 0 | 0 | 0 |
| *Butia paraguayensis* | 1 | 0 | 0 | 0 | 0 | 0 | 0 | 1 | 1 | 0 | 0 | 0 | 0 | 0 | 0 | 0 | 0 | 0 | 0 | 0 |
| *Butia purpurascens* | 1 | 0 | 0 | 0 | 0 | 0 | 0 | 1 | 0 | 0 | 0 | 0 | 0 | 0 | 0 | 0 | 0 | 0 | 0 | 0 |
| *Butia yatay* | 1 | 0 | 0 | 0 | 0 | 0 | 0 | 0 | 1 | 0 | 1 | 0 | 0 | 0 | 0 | 0 | 0 | 0 | 0 | 0 |
| *Calyptrogyne allenii* | 0 | 0 | 1 | 0 | 0 | 0 | 0 | 0 | 0 | 0 | 0 | 0 | 0 | 0 | 1 | 0 | 0 | 0 | 0 | 0 |
| *Calyptrogyne anomala* | 0 | 0 | 1 | 0 | 0 | 0 | 0 | 0 | 0 | 1 | 0 | 0 | 0 | 0 | 0 | 0 | 0 | 0 | 0 | 0 |
| *Calyptrogyne condensata* | 0 | 0 | 1 | 0 | 0 | 0 | 0 | 0 | 0 | 0 | 0 | 0 | 0 | 0 | 0 | 0 | 0 | 0 | 0 | 0 |
| *Calyptrogyne costatifrons* | 0 | 0 | 1 | 0 | 0 | 0 | 0 | 0 | 0 | 1 | 0 | 0 | 0 | 0 | 0 | 0 | 0 | 0 | 0 | 0 |
| *Calyptrogyne ghiesbreghtiana* | 0 | 0 | 1 | 0 | 0 | 0 | 0 | 0 | 0 | 1 | 0 | 0 | 0 | 0 | 0 | 0 | 0 | 1 | 0 | 0 |
| *Calyptrogyne kunorum* | 0 | 0 | 1 | 0 | 0 | 0 | 0 | 0 | 0 | 1 | 0 | 0 | 0 | 0 | 0 | 0 | 0 | 0 | 0 | 0 |
| *Calyptrogyne pubescens* | 0 | 0 | 1 | 0 | 0 | 0 | 0 | 0 | 0 | 0 | 0 | 0 | 0 | 0 | 0 | 0 | 0 | 0 | 0 | 0 |
| *Calyptrogyne trichostachys* | 0 | 0 | 1 | 0 | 0 | 0 | 0 | 0 | 0 | 0 | 0 | 0 | 0 | 0 | 0 | 0 | 0 | 1 | 0 | 0 |
| *Calyptronoma occidentalis* | 0 | 0 | 0 | 0 | 1 | 0 | 0 | 0 | 0 | 0 | 0 | 0 | 0 | 0 | 0 | 0 | 0 | 0 | 0 | 0 |
| *Calyptronoma plumeriana* | 0 | 0 | 0 | 0 | 1 | 0 | 0 | 0 | 0 | 0 | 0 | 0 | 0 | 0 | 0 | 0 | 0 | 0 | 0 | 0 |
| *Calyptronoma rivalis* | 0 | 0 | 0 | 0 | 1 | 0 | 0 | 0 | 0 | 0 | 0 | 0 | 0 | 0 | 0 | 0 | 0 | 0 | 0 | 0 |
| *Ceroxylon alpinum* | 0 | 0 | 0 | 0 | 0 | 0 | 0 | 0 | 0 | 0 | 0 | 0 | 0 | 0 | 1 | 0 | 0 | 0 | 0 | 0 |
| *Ceroxylon amazonicum* | 0 | 0 | 0 | 0 | 0 | 0 | 0 | 0 | 0 | 0 | 0 | 0 | 0 | 0 | 1 | 0 | 0 | 0 | 0 | 0 |
| *Ceroxylon ceriferum* | 0 | 0 | 0 | 0 | 0 | 0 | 0 | 0 | 0 | 0 | 0 | 0 | 0 | 0 | 1 | 0 | 0 | 0 | 0 | 0 |
| *Ceroxylon echinulatum* | 0 | 0 | 0 | 0 | 0 | 0 | 1 | 0 | 0 | 0 | 0 | 0 | 0 | 0 | 1 | 0 | 0 | 0 | 0 | 0 |
| *Ceroxylon parvifrons* | 0 | 0 | 0 | 0 | 0 | 0 | 1 | 0 | 0 | 0 | 0 | 0 | 0 | 0 | 1 | 0 | 0 | 0 | 1 | 0 |
| *Ceroxylon parvum* | 0 | 0 | 0 | 0 | 0 | 0 | 1 | 0 | 1 | 0 | 0 | 0 | 0 | 0 | 1 | 0 | 0 | 0 | 0 | 0 |
| *Ceroxylon quindiuense* | 0 | 0 | 0 | 0 | 0 | 0 | 1 | 0 | 0 | 0 | 0 | 0 | 0 | 0 | 1 | 0 | 0 | 0 | 0 | 0 |
| *Ceroxylon sasaimae* | 0 | 0 | 0 | 0 | 0 | 0 | 0 | 0 | 0 | 0 | 0 | 0 | 0 | 0 | 1 | 0 | 0 | 0 | 0 | 0 |
| *Ceroxylon ventricosum* | 0 | 0 | 0 | 0 | 0 | 0 | 0 | 0 | 0 | 0 | 0 | 0 | 0 | 0 | 1 | 0 | 0 | 0 | 0 | 0 |
| *Ceroxylon vogelianum* | 0 | 0 | 0 | 0 | 0 | 0 | 1 | 0 | 0 | 0 | 0 | 0 | 0 | 0 | 1 | 0 | 0 | 0 | 1 | 0 |
| *Ceroxylon weberbaueri* | 0 | 0 | 0 | 0 | 0 | 0 | 1 | 0 | 0 | 0 | 0 | 0 | 0 | 0 | 0 | 0 | 0 | 0 | 0 | 0 |
| *Chamaedorea adscendens* | 0 | 0 | 1 | 0 | 0 | 0 | 0 | 0 | 0 | 0 | 0 | 0 | 0 | 0 | 1 | 0 | 0 | 0 | 0 | 0 |
| *Chamaedorea allenii* | 0 | 0 | 1 | 0 | 0 | 0 | 0 | 0 | 0 | 1 | 0 | 0 | 0 | 0 | 1 | 0 | 0 | 0 | 1 | 0 |
| *Chamaedorea amabilis* | 0 | 0 | 1 | 0 | 0 | 0 | 0 | 0 | 0 | 0 | 0 | 0 | 0 | 0 | 0 | 0 | 0 | 0 | 0 | 0 |
| *Chamaedorea angustisecta* | 0 | 0 | 1 | 0 | 0 | 0 | 1 | 1 | 0 | 0 | 0 | 0 | 0 | 0 | 0 | 0 | 0 | 0 | 1 | 0 |
| *Chamaedorea arenbergiana* | 0 | 0 | 1 | 0 | 0 | 0 | 0 | 0 | 0 | 0 | 0 | 0 | 0 | 0 | 0 | 0 | 0 | 1 | 0 | 0 |
| *Chamaedorea brachyclada* | 0 | 0 | 1 | 0 | 0 | 0 | 0 | 0 | 0 | 0 | 0 | 0 | 0 | 0 | 0 | 0 | 0 | 0 | 0 | 0 |
| *Chamaedorea brachypoda* | 0 | 0 | 1 | 0 | 0 | 0 | 0 | 0 | 0 | 0 | 0 | 0 | 0 | 0 | 0 | 0 | 0 | 0 | 0 | 0 |
| *Chamaedorea carchensis* | 0 | 0 | 1 | 0 | 0 | 0 | 0 | 0 | 0 | 0 | 0 | 0 | 0 | 0 | 0 | 0 | 0 | 0 | 0 | 0 |
| *Chamaedorea cataractarum* | 0 | 0 | 1 | 0 | 0 | 0 | 0 | 0 | 0 | 0 | 0 | 0 | 0 | 0 | 0 | 0 | 0 | 1 | 0 | 1 |
| *Chamaedorea correae* | 0 | 0 | 1 | 0 | 0 | 0 | 0 | 0 | 0 | 1 | 0 | 0 | 0 | 0 | 0 | 0 | 0 | 0 | 0 | 0 |
| *Chamaedorea costaricana* | 0 | 0 | 1 | 0 | 0 | 0 | 1 | 0 | 0 | 1 | 0 | 0 | 0 | 0 | 1 | 0 | 0 | 1 | 0 | 0 |
| *Chamaedorea dammeriana* | 0 | 0 | 1 | 0 | 0 | 0 | 0 | 0 | 0 | 0 | 0 | 0 | 0 | 0 | 0 | 0 | 0 | 1 | 0 | 0 |
| *Chamaedorea deckeriana* | 0 | 0 | 1 | 0 | 0 | 0 | 0 | 0 | 0 | 1 | 0 | 0 | 0 | 0 | 0 | 0 | 0 | 0 | 0 | 0 |
| *Chamaedorea deneversiana* | 0 | 0 | 1 | 0 | 0 | 0 | 0 | 0 | 0 | 1 | 0 | 0 | 0 | 0 | 1 | 0 | 0 | 0 | 0 | 0 |
| *Chamaedorea elatior* | 0 | 0 | 1 | 0 | 0 | 0 | 0 | 0 | 0 | 0 | 0 | 0 | 0 | 0 | 0 | 0 | 0 | 1 | 0 | 1 |
| *Chamaedorea elegans* | 0 | 0 | 1 | 0 | 0 | 0 | 0 | 0 | 0 | 0 | 0 | 0 | 0 | 0 | 0 | 0 | 0 | 1 | 0 | 0 |
| *Chamaedorea ernesti augustii* | 0 | 0 | 1 | 0 | 0 | 0 | 0 | 0 | 0 | 0 | 0 | 0 | 0 | 0 | 0 | 0 | 0 | 0 | 0 | 0 |
| *Chamaedorea fragrans* | 1 | 0 | 0 | 0 | 0 | 0 | 1 | 0 | 0 | 0 | 0 | 0 | 0 | 0 | 1 | 0 | 0 | 0 | 1 | 0 |
| *Chamaedorea geonomiformis* | 0 | 0 | 1 | 0 | 0 | 0 | 0 | 0 | 0 | 1 | 0 | 0 | 0 | 0 | 1 | 0 | 0 | 1 | 0 | 0 |
| *Chamaedorea glaucifolia* | 0 | 0 | 1 | 0 | 0 | 0 | 0 | 0 | 0 | 0 | 0 | 0 | 0 | 0 | 0 | 0 | 0 | 1 | 0 | 0 |
| *Chamaedorea graminifolia* | 0 | 0 | 1 | 0 | 0 | 0 | 0 | 0 | 0 | 0 | 0 | 0 | 0 | 0 | 0 | 0 | 0 | 1 | 0 | 0 |
| *Chamaedorea guntheriana* | 0 | 0 | 1 | 0 | 0 | 0 | 0 | 0 | 0 | 0 | 0 | 0 | 0 | 0 | 0 | 0 | 0 | 0 | 0 | 0 |
| *Chamaedorea hooperiana* | 0 | 0 | 1 | 0 | 0 | 0 | 0 | 0 | 0 | 0 | 0 | 0 | 0 | 0 | 0 | 0 | 0 | 0 | 0 | 0 |
| *Chamaedorea klotzschiana* | 0 | 0 | 1 | 0 | 0 | 0 | 0 | 0 | 0 | 0 | 0 | 0 | 0 | 0 | 0 | 0 | 0 | 0 | 1 | 0 |
| *Chamaedorea lehmannii* | 0 | 0 | 0 | 0 | 0 | 0 | 0 | 0 | 0 | 0 | 0 | 0 | 0 | 0 | 0 | 0 | 0 | 1 | 0 | 0 |
| *Chamaedorea liebmannii* | 0 | 0 | 1 | 0 | 0 | 0 | 0 | 0 | 0 | 0 | 0 | 0 | 0 | 0 | 1 | 0 | 0 | 1 | 0 | 1 |
| *Chamaedorea linearis* | 0 | 0 | 1 | 0 | 0 | 1 | 1 | 0 | 1 | 1 | 0 | 0 | 1 | 0 | 1 | 0 | 0 | 0 | 1 | 0 |
| *Chamaedorea lucidifrons* | 0 | 0 | 1 | 0 | 0 | 0 | 0 | 0 | 0 | 1 | 0 | 0 | 0 | 0 | 0 | 0 | 0 | 0 | 0 | 0 |
| *Chamaedorea macrospadix* | 0 | 0 | 1 | 0 | 0 | 0 | 0 | 0 | 0 | 1 | 0 | 0 | 0 | 0 | 0 | 0 | 0 | 0 | 0 | 0 |
| *Chamaedorea metallica* | 0 | 0 | 1 | 0 | 0 | 0 | 0 | 0 | 0 | 0 | 0 | 0 | 0 | 0 | 0 | 0 | 0 | 0 | 0 | 0 |
| *Chamaedorea microphylla* | 0 | 0 | 1 | 0 | 0 | 0 | 0 | 0 | 0 | 0 | 0 | 0 | 0 | 0 | 0 | 0 | 0 | 0 | 0 | 0 |
| *Chamaedorea microspadix* | 0 | 0 | 1 | 0 | 0 | 0 | 0 | 0 | 0 | 0 | 0 | 0 | 0 | 0 | 0 | 0 | 1 | 1 | 0 | 1 |
| *Chamaedorea murriensis* | 0 | 0 | 0 | 0 | 0 | 0 | 0 | 0 | 0 | 0 | 0 | 0 | 0 | 0 | 1 | 0 | 0 | 0 | 0 | 0 |
| *Chamaedorea nubium* | 0 | 0 | 1 | 0 | 0 | 0 | 0 | 0 | 0 | 0 | 0 | 0 | 0 | 0 | 0 | 0 | 0 | 1 | 0 | 0 |
| *Chamaedorea oblongata* | 0 | 0 | 1 | 0 | 0 | 0 | 0 | 0 | 0 | 0 | 0 | 0 | 0 | 0 | 0 | 0 | 0 | 1 | 0 | 0 |
| *Chamaedorea oreophila* | 0 | 0 | 1 | 0 | 0 | 0 | 0 | 0 | 0 | 0 | 0 | 0 | 0 | 0 | 0 | 0 | 0 | 1 | 0 | 0 |
| *Chamaedorea palmeriana* | 0 | 0 | 1 | 0 | 0 | 0 | 0 | 0 | 0 | 0 | 0 | 0 | 0 | 0 | 0 | 0 | 0 | 0 | 0 | 0 |
| *Chamaedorea parvifolia* | 0 | 0 | 1 | 0 | 0 | 0 | 0 | 0 | 0 | 0 | 0 | 0 | 0 | 0 | 0 | 0 | 0 | 0 | 1 | 0 |
| *Chamaedorea parvisecta* | 0 | 0 | 1 | 0 | 0 | 0 | 0 | 0 | 0 | 0 | 0 | 0 | 0 | 0 | 0 | 0 | 0 | 0 | 0 | 0 |
| *Chamaedorea pauciflora* | 0 | 1 | 0 | 0 | 0 | 0 | 1 | 1 | 0 | 1 | 0 | 0 | 0 | 1 | 1 | 0 | 0 | 0 | 1 | 0 |
| *Chamaedorea pinnatifrons* | 0 | 1 | 1 | 0 | 0 | 1 | 1 | 1 | 1 | 1 | 0 | 0 | 1 | 1 | 1 | 0 | 0 | 1 | 1 | 0 |
| *Chamaedorea pittieri* | 0 | 0 | 1 | 0 | 0 | 0 | 0 | 0 | 0 | 0 | 0 | 0 | 0 | 0 | 0 | 0 | 0 | 0 | 0 | 0 |
| *Chamaedorea plumosa* | 0 | 0 | 0 | 0 | 0 | 0 | 0 | 0 | 0 | 0 | 0 | 0 | 0 | 0 | 0 | 0 | 0 | 1 | 0 | 0 |
| *Chamaedorea pochutlensis* | 0 | 0 | 1 | 0 | 0 | 0 | 0 | 0 | 0 | 0 | 0 | 0 | 0 | 0 | 0 | 0 | 0 | 1 | 0 | 1 |
| *Chamaedorea pumila* | 0 | 0 | 1 | 0 | 0 | 0 | 0 | 0 | 0 | 0 | 0 | 0 | 0 | 0 | 0 | 0 | 0 | 0 | 0 | 0 |
| *Chamaedorea pygmaea* | 0 | 0 | 1 | 0 | 0 | 0 | 0 | 0 | 0 | 1 | 0 | 0 | 0 | 0 | 0 | 0 | 0 | 1 | 0 | 0 |
| *Chamaedorea queroana* | 0 | 0 | 0 | 0 | 0 | 0 | 0 | 0 | 0 | 0 | 0 | 0 | 0 | 0 | 0 | 0 | 0 | 1 | 0 | 0 |
| *Chamaedorea radicalis* | 0 | 0 | 1 | 0 | 0 | 0 | 0 | 0 | 0 | 0 | 0 | 0 | 0 | 0 | 0 | 0 | 0 | 1 | 0 | 1 |
| *Chamaedorea rigida* | 0 | 0 | 1 | 0 | 0 | 0 | 0 | 0 | 0 | 0 | 0 | 0 | 0 | 0 | 0 | 0 | 0 | 1 | 0 | 0 |
| *Chamaedorea robertii* | 0 | 0 | 1 | 0 | 0 | 0 | 0 | 0 | 0 | 0 | 0 | 0 | 0 | 0 | 0 | 0 | 0 | 0 | 0 | 0 |
| *Chamaedorea rojasiana* | 0 | 0 | 1 | 0 | 0 | 0 | 0 | 0 | 0 | 0 | 0 | 0 | 0 | 0 | 0 | 0 | 0 | 1 | 0 | 0 |
| *Chamaedorea sartorii* | 0 | 0 | 1 | 0 | 0 | 0 | 0 | 0 | 0 | 0 | 0 | 0 | 0 | 0 | 0 | 0 | 0 | 1 | 0 | 0 |
| *Chamaedorea scheryi* | 0 | 0 | 1 | 0 | 0 | 0 | 0 | 0 | 0 | 0 | 0 | 0 | 0 | 0 | 0 | 0 | 0 | 0 | 0 | 0 |
| *Chamaedorea schiedeana* | 0 | 0 | 1 | 0 | 0 | 0 | 0 | 0 | 0 | 0 | 0 | 0 | 0 | 0 | 0 | 0 | 0 | 1 | 0 | 0 |
| *Chamaedorea seifrizii* | 0 | 0 | 1 | 0 | 0 | 0 | 0 | 0 | 0 | 0 | 0 | 0 | 0 | 0 | 0 | 0 | 0 | 1 | 0 | 0 |
| *Chamaedorea selvae* | 0 | 0 | 1 | 0 | 0 | 0 | 0 | 0 | 0 | 0 | 0 | 0 | 0 | 0 | 0 | 0 | 0 | 0 | 0 | 0 |
| *Chamaedorea simplex* | 0 | 0 | 1 | 0 | 0 | 0 | 0 | 0 | 0 | 0 | 0 | 0 | 0 | 0 | 0 | 0 | 0 | 0 | 0 | 0 |
| *Chamaedorea stricta* | 0 | 0 | 1 | 0 | 0 | 0 | 0 | 0 | 0 | 0 | 0 | 0 | 0 | 0 | 0 | 0 | 0 | 0 | 0 | 0 |
| *Chamaedorea tenerrima* | 0 | 0 | 1 | 0 | 0 | 0 | 0 | 0 | 0 | 0 | 0 | 0 | 0 | 0 | 0 | 0 | 0 | 0 | 0 | 0 |
| *Chamaedorea tepejilote* | 0 | 0 | 1 | 0 | 0 | 0 | 0 | 0 | 0 | 1 | 0 | 0 | 0 | 0 | 1 | 0 | 0 | 1 | 0 | 0 |
| *Chamaedorea tuerckheimii* | 0 | 0 | 1 | 0 | 0 | 0 | 0 | 0 | 0 | 0 | 0 | 0 | 0 | 0 | 0 | 0 | 0 | 1 | 0 | 0 |
| *Chamaedorea undulatifolia* | 0 | 0 | 1 | 0 | 0 | 0 | 0 | 0 | 0 | 0 | 0 | 0 | 0 | 0 | 0 | 0 | 0 | 0 | 0 | 0 |
| *Chamaedorea verecunda* | 0 | 0 | 1 | 0 | 0 | 0 | 0 | 0 | 0 | 0 | 0 | 0 | 0 | 0 | 0 | 0 | 0 | 0 | 0 | 0 |
| *Chamaedorea vulgata* | 0 | 0 | 0 | 0 | 0 | 0 | 0 | 0 | 0 | 0 | 0 | 0 | 0 | 0 | 0 | 0 | 0 | 1 | 0 | 0 |
| *Chamaedorea warscewiczii* | 0 | 0 | 1 | 0 | 0 | 0 | 0 | 0 | 0 | 1 | 0 | 0 | 0 | 0 | 0 | 0 | 0 | 0 | 0 | 0 |
| *Chamaedorea whitelockiana* | 0 | 0 | 0 | 0 | 0 | 0 | 0 | 0 | 0 | 0 | 0 | 0 | 0 | 0 | 0 | 0 | 0 | 1 | 0 | 0 |
| *Chamaedorea woodsoniana* | 0 | 0 | 1 | 0 | 0 | 0 | 0 | 0 | 0 | 1 | 0 | 0 | 0 | 0 | 0 | 0 | 0 | 1 | 0 | 0 |
| *Chelyocarpus chuco* | 0 | 1 | 0 | 0 | 0 | 0 | 0 | 0 | 0 | 0 | 0 | 0 | 0 | 0 | 0 | 0 | 0 | 0 | 1 | 0 |
| *Chelyocarpus dianeurus* | 0 | 0 | 0 | 0 | 0 | 0 | 0 | 0 | 0 | 1 | 0 | 0 | 0 | 1 | 0 | 0 | 0 | 0 | 0 | 0 |
| *Chelyocarpus repens* | 0 | 0 | 0 | 0 | 0 | 0 | 0 | 0 | 0 | 0 | 0 | 0 | 0 | 0 | 0 | 0 | 0 | 0 | 1 | 0 |
| *Chelyocarpus ulei* | 0 | 0 | 0 | 0 | 0 | 0 | 0 | 0 | 0 | 0 | 0 | 0 | 0 | 0 | 1 | 0 | 0 | 0 | 1 | 0 |
| *Coccothrinax argentata* | 0 | 0 | 0 | 0 | 1 | 0 | 0 | 0 | 0 | 0 | 0 | 0 | 0 | 0 | 0 | 0 | 1 | 0 | 0 | 0 |
| *Coccothrinax argentea* | 0 | 0 | 0 | 0 | 1 | 0 | 0 | 0 | 0 | 0 | 0 | 0 | 0 | 0 | 0 | 0 | 1 | 0 | 0 | 0 |
| *Coccothrinax barbadensis* | 1 | 0 | 0 | 0 | 1 | 0 | 0 | 0 | 0 | 0 | 0 | 0 | 0 | 0 | 0 | 0 | 1 | 0 | 1 | 0 |
| *Coccothrinax crinita* | 0 | 0 | 0 | 0 | 1 | 0 | 0 | 0 | 0 | 0 | 0 | 0 | 0 | 0 | 0 | 0 | 0 | 0 | 0 | 0 |
| *Coccothrinax ekmanii* | 0 | 0 | 0 | 0 | 1 | 0 | 0 | 0 | 0 | 0 | 0 | 0 | 0 | 0 | 0 | 0 | 0 | 0 | 0 | 0 |
| *Coccothrinax gracilis* | 0 | 0 | 0 | 0 | 1 | 0 | 0 | 0 | 0 | 0 | 0 | 0 | 0 | 0 | 0 | 0 | 0 | 0 | 0 | 0 |
| *Coccothrinax gundlachii* | 0 | 0 | 0 | 0 | 1 | 0 | 0 | 0 | 0 | 0 | 0 | 0 | 0 | 0 | 0 | 0 | 0 | 0 | 0 | 0 |
| *Coccothrinax miraguama* | 0 | 0 | 0 | 0 | 1 | 0 | 0 | 0 | 0 | 0 | 0 | 0 | 0 | 0 | 0 | 0 | 1 | 0 | 0 | 0 |
| *Coccothrinax pauciramosa* | 0 | 0 | 0 | 0 | 1 | 0 | 0 | 0 | 0 | 0 | 0 | 0 | 0 | 0 | 0 | 0 | 0 | 0 | 0 | 0 |
| *Coccothrinax salvatoris* | 0 | 0 | 0 | 0 | 1 | 0 | 0 | 0 | 0 | 0 | 0 | 0 | 0 | 0 | 0 | 0 | 0 | 0 | 0 | 0 |
| *Coccothrinax spissa* | 0 | 0 | 0 | 0 | 1 | 0 | 0 | 0 | 0 | 0 | 0 | 0 | 0 | 0 | 0 | 0 | 0 | 0 | 0 | 0 |
| *Colpothrinax cookii* | 0 | 0 | 1 | 0 | 0 | 0 | 0 | 0 | 0 | 1 | 0 | 0 | 0 | 0 | 0 | 0 | 0 | 0 | 0 | 0 |
| *Colpothrinax wrightii* | 0 | 0 | 0 | 0 | 1 | 0 | 0 | 0 | 0 | 0 | 0 | 0 | 0 | 0 | 0 | 0 | 0 | 0 | 0 | 0 |
| *Copernicia alba* | 1 | 0 | 0 | 1 | 0 | 0 | 1 | 1 | 1 | 0 | 0 | 0 | 0 | 0 | 0 | 1 | 0 | 0 | 0 | 0 |
| *Copernicia baileyana* | 0 | 0 | 0 | 0 | 1 | 0 | 0 | 0 | 0 | 0 | 0 | 0 | 0 | 0 | 0 | 0 | 1 | 0 | 0 | 0 |
| *Copernicia berteroana* | 0 | 0 | 0 | 0 | 1 | 0 | 0 | 0 | 0 | 0 | 0 | 0 | 0 | 0 | 0 | 0 | 0 | 0 | 0 | 0 |
| *Copernicia cowellii* | 0 | 0 | 0 | 0 | 1 | 0 | 0 | 0 | 0 | 0 | 0 | 0 | 0 | 0 | 0 | 0 | 0 | 0 | 0 | 0 |
| *Copernicia ekmanii* | 0 | 0 | 0 | 0 | 1 | 0 | 0 | 0 | 0 | 0 | 0 | 0 | 0 | 0 | 0 | 0 | 0 | 0 | 0 | 0 |
| *Copernicia gigas* | 0 | 0 | 0 | 0 | 1 | 0 | 0 | 0 | 0 | 0 | 0 | 0 | 0 | 0 | 0 | 0 | 0 | 0 | 0 | 0 |
| *Copernicia glabrescens* | 0 | 0 | 0 | 0 | 1 | 0 | 0 | 0 | 0 | 0 | 0 | 0 | 0 | 0 | 0 | 0 | 0 | 0 | 0 | 0 |
| *Copernicia hospita* | 0 | 0 | 0 | 0 | 1 | 0 | 0 | 0 | 0 | 0 | 0 | 0 | 0 | 0 | 0 | 0 | 0 | 0 | 0 | 0 |
| *Copernicia macroglossa* | 0 | 0 | 0 | 0 | 1 | 0 | 0 | 0 | 0 | 0 | 0 | 0 | 0 | 0 | 0 | 0 | 0 | 0 | 0 | 0 |
| *Copernicia prunifera* | 1 | 1 | 0 | 1 | 0 | 0 | 0 | 1 | 0 | 0 | 0 | 0 | 0 | 0 | 0 | 0 | 0 | 0 | 0 | 0 |
| *Copernicia rigida* | 0 | 0 | 0 | 0 | 1 | 0 | 0 | 0 | 0 | 0 | 0 | 0 | 0 | 0 | 0 | 0 | 0 | 0 | 0 | 0 |
| *Copernicia tectorum* | 0 | 0 | 0 | 0 | 0 | 1 | 0 | 0 | 0 | 1 | 0 | 0 | 1 | 1 | 1 | 0 | 0 | 0 | 0 | 0 |
| *Cryosophila cookii* | 0 | 0 | 1 | 0 | 0 | 0 | 0 | 0 | 0 | 0 | 0 | 0 | 0 | 0 | 0 | 0 | 0 | 0 | 0 | 0 |
| *Cryosophila grayumii* | 0 | 0 | 1 | 0 | 0 | 0 | 0 | 0 | 0 | 0 | 0 | 0 | 0 | 0 | 0 | 0 | 0 | 0 | 0 | 0 |
| *Cryosophila guagara* | 0 | 0 | 1 | 0 | 0 | 0 | 0 | 0 | 0 | 0 | 0 | 0 | 0 | 0 | 0 | 0 | 0 | 1 | 0 | 0 |
| *Cryosophila kalbreyeri* | 0 | 0 | 1 | 0 | 0 | 1 | 0 | 0 | 0 | 1 | 0 | 0 | 0 | 0 | 1 | 0 | 0 | 0 | 0 | 0 |
| *Cryosophila macrocarpa* | 0 | 0 | 0 | 0 | 0 | 0 | 0 | 0 | 0 | 1 | 0 | 0 | 0 | 0 | 0 | 0 | 0 | 0 | 0 | 0 |
| *Cryosophila nana* | 0 | 0 | 0 | 0 | 0 | 0 | 0 | 0 | 0 | 0 | 0 | 0 | 0 | 0 | 0 | 0 | 0 | 1 | 0 | 1 |
| *Cryosophila stauracantha* | 0 | 0 | 1 | 0 | 1 | 0 | 0 | 0 | 0 | 0 | 0 | 0 | 0 | 0 | 0 | 0 | 0 | 1 | 0 | 0 |
| *Cryosophila warscewiczii* | 0 | 0 | 1 | 0 | 0 | 0 | 0 | 0 | 0 | 1 | 0 | 0 | 0 | 0 | 0 | 0 | 0 | 0 | 0 | 0 |
| *Cryosophila williamsii* | 0 | 0 | 1 | 0 | 0 | 0 | 0 | 0 | 0 | 0 | 0 | 0 | 0 | 0 | 0 | 0 | 0 | 1 | 0 | 0 |
| *Desmoncus cirrhifera* | 0 | 0 | 0 | 0 | 0 | 0 | 0 | 0 | 0 | 1 | 0 | 0 | 1 | 0 | 1 | 0 | 0 | 0 | 0 | 0 |
| *Desmoncus giganteus* | 0 | 1 | 0 | 0 | 0 | 0 | 0 | 0 | 0 | 1 | 0 | 0 | 0 | 0 | 1 | 0 | 0 | 0 | 1 | 0 |
| *Desmoncus mitis* | 0 | 1 | 0 | 0 | 0 | 0 | 1 | 1 | 0 | 1 | 0 | 0 | 0 | 0 | 0 | 0 | 0 | 0 | 1 | 0 |
| *Desmoncus orthacanthos* | 1 | 1 | 1 | 1 | 0 | 1 | 0 | 1 | 0 | 1 | 0 | 1 | 0 | 1 | 1 | 1 | 0 | 1 | 1 | 0 |
| *Desmoncus phoenicocarpus* | 0 | 1 | 0 | 0 | 0 | 0 | 0 | 1 | 0 | 0 | 0 | 1 | 0 | 1 | 0 | 0 | 0 | 0 | 1 | 0 |
| *Desmoncus polyacanthos* | 1 | 1 | 1 | 1 | 0 | 0 | 1 | 1 | 0 | 0 | 0 | 1 | 0 | 1 | 1 | 0 | 0 | 0 | 1 | 0 |
| *Desmoncus stans* | 0 | 0 | 1 | 0 | 0 | 0 | 0 | 0 | 0 | 0 | 0 | 0 | 0 | 0 | 0 | 0 | 0 | 0 | 0 | 0 |
| *Dictyocaryum lamarckianum* | 0 | 0 | 1 | 0 | 0 | 0 | 1 | 0 | 0 | 1 | 0 | 0 | 0 | 0 | 1 | 0 | 0 | 0 | 1 | 0 |
| *Dictyocaryum ptarianum* | 0 | 0 | 0 | 0 | 0 | 0 | 0 | 0 | 0 | 0 | 0 | 1 | 0 | 0 | 0 | 0 | 0 | 0 | 1 | 0 |
| *Elaeis oleifera* | 0 | 1 | 1 | 0 | 0 | 0 | 1 | 0 | 0 | 1 | 0 | 0 | 0 | 1 | 1 | 0 | 0 | 1 | 1 | 0 |
| *Euterpe broadwayi* | 0 | 0 | 0 | 0 | 1 | 0 | 0 | 0 | 0 | 0 | 0 | 0 | 0 | 0 | 0 | 0 | 0 | 0 | 0 | 0 |
| *Euterpe catinga* | 0 | 1 | 0 | 0 | 0 | 0 | 0 | 0 | 0 | 0 | 0 | 1 | 0 | 1 | 1 | 0 | 0 | 0 | 1 | 0 |
| *Euterpe edulis* | 1 | 0 | 0 | 1 | 0 | 0 | 0 | 1 | 0 | 0 | 1 | 0 | 0 | 0 | 0 | 0 | 0 | 0 | 0 | 0 |
| *Euterpe longibracteata* | 0 | 1 | 0 | 0 | 0 | 0 | 0 | 0 | 0 | 0 | 0 | 1 | 0 | 0 | 0 | 0 | 0 | 0 | 0 | 0 |
| *Euterpe luminosa* | 0 | 0 | 0 | 0 | 0 | 0 | 1 | 0 | 0 | 0 | 0 | 0 | 0 | 0 | 0 | 0 | 0 | 0 | 0 | 0 |
| *Euterpe oleracea* | 1 | 1 | 1 | 0 | 0 | 1 | 0 | 1 | 0 | 1 | 0 | 1 | 0 | 0 | 1 | 0 | 0 | 0 | 1 | 0 |
| *Euterpe precatoria* | 0 | 1 | 1 | 0 | 0 | 1 | 1 | 1 | 0 | 1 | 0 | 1 | 1 | 1 | 1 | 0 | 0 | 1 | 1 | 0 |
| *Gaussia attenuata* | 0 | 0 | 0 | 0 | 1 | 0 | 0 | 0 | 0 | 0 | 0 | 0 | 0 | 0 | 0 | 0 | 0 | 0 | 0 | 0 |
| *Gaussia gomez pompae* | 0 | 0 | 1 | 0 | 0 | 0 | 0 | 0 | 0 | 0 | 0 | 0 | 0 | 0 | 0 | 0 | 0 | 0 | 0 | 0 |
| *Gaussia maya* | 0 | 0 | 1 | 0 | 0 | 0 | 0 | 0 | 0 | 0 | 0 | 0 | 0 | 0 | 0 | 0 | 0 | 0 | 0 | 0 |
| *Gaussia princeps* | 0 | 0 | 0 | 0 | 1 | 0 | 0 | 0 | 0 | 0 | 0 | 0 | 0 | 0 | 0 | 0 | 0 | 0 | 0 | 0 |
| *Geonoma appuniana* | 0 | 0 | 0 | 0 | 0 | 0 | 0 | 0 | 0 | 0 | 0 | 1 | 0 | 0 | 1 | 0 | 0 | 0 | 0 | 0 |
| *Geonoma arundinacea* | 0 | 1 | 0 | 0 | 0 | 0 | 1 | 0 | 0 | 0 | 0 | 1 | 0 | 0 | 1 | 0 | 0 | 0 | 1 | 0 |
| *Geonoma aspidiifolia* | 0 | 1 | 0 | 0 | 0 | 0 | 0 | 0 | 0 | 0 | 0 | 1 | 0 | 0 | 1 | 0 | 0 | 0 | 1 | 0 |
| *Geonoma baculifera* | 0 | 1 | 0 | 0 | 0 | 0 | 0 | 0 | 0 | 0 | 0 | 1 | 0 | 0 | 0 | 0 | 0 | 0 | 1 | 0 |
| *Geonoma brevispatha* | 1 | 1 | 0 | 1 | 0 | 0 | 1 | 1 | 1 | 0 | 0 | 0 | 0 | 0 | 0 | 0 | 0 | 0 | 1 | 0 |
| *Geonoma brongniartii* | 0 | 1 | 0 | 0 | 0 | 0 | 1 | 1 | 0 | 0 | 0 | 0 | 0 | 1 | 1 | 0 | 0 | 0 | 1 | 0 |
| *Geonoma camana* | 0 | 1 | 0 | 0 | 0 | 0 | 0 | 0 | 0 | 0 | 0 | 0 | 0 | 0 | 1 | 0 | 0 | 0 | 1 | 0 |
| *Geonoma chlamydostachys* | 0 | 0 | 0 | 0 | 0 | 0 | 0 | 0 | 0 | 1 | 0 | 0 | 0 | 0 | 1 | 0 | 0 | 0 | 0 | 0 |
| *Geonoma chococola* | 0 | 0 | 1 | 0 | 0 | 0 | 0 | 0 | 0 | 1 | 0 | 0 | 0 | 0 | 1 | 0 | 0 | 0 | 0 | 0 |
| *Geonoma concinna* | 0 | 0 | 1 | 0 | 0 | 0 | 0 | 0 | 0 | 1 | 0 | 0 | 0 | 0 | 1 | 0 | 0 | 0 | 0 | 0 |
| *Geonoma congesta* | 0 | 0 | 1 | 0 | 0 | 0 | 0 | 0 | 0 | 1 | 0 | 0 | 0 | 0 | 1 | 0 | 0 | 1 | 0 | 0 |
| *Geonoma cuneata* | 0 | 0 | 1 | 0 | 0 | 0 | 1 | 1 | 0 | 1 | 0 | 0 | 0 | 0 | 1 | 0 | 0 | 1 | 1 | 0 |
| *Geonoma densa* | 0 | 0 | 0 | 0 | 0 | 0 | 1 | 0 | 0 | 0 | 0 | 1 | 0 | 0 | 1 | 0 | 0 | 0 | 1 | 0 |
| *Geonoma deversa* | 0 | 1 | 1 | 0 | 0 | 1 | 1 | 1 | 0 | 1 | 0 | 1 | 0 | 1 | 1 | 0 | 0 | 1 | 1 | 0 |
| *Geonoma divisa* | 0 | 0 | 0 | 0 | 0 | 0 | 0 | 0 | 0 | 1 | 0 | 0 | 0 | 0 | 0 | 0 | 0 | 0 | 0 | 0 |
| *Geonoma epetiolata* | 0 | 0 | 1 | 0 | 0 | 0 | 0 | 0 | 0 | 0 | 0 | 0 | 0 | 0 | 0 | 0 | 0 | 0 | 0 | 0 |
| *Geonoma ferruginea* | 0 | 0 | 1 | 0 | 0 | 0 | 0 | 0 | 0 | 0 | 0 | 0 | 0 | 0 | 1 | 0 | 0 | 1 | 1 | 0 |
| *Geonoma gamiova* | 1 | 0 | 0 | 0 | 0 | 0 | 0 | 0 | 0 | 0 | 1 | 0 | 0 | 0 | 0 | 0 | 0 | 0 | 0 | 0 |
| *Geonoma interrupta* | 0 | 1 | 1 | 0 | 1 | 1 | 1 | 1 | 0 | 1 | 0 | 1 | 1 | 1 | 1 | 0 | 0 | 1 | 1 | 0 |
| *Geonoma jussieuana* | 0 | 0 | 1 | 0 | 0 | 0 | 1 | 0 | 0 | 1 | 0 | 0 | 0 | 0 | 1 | 0 | 0 | 0 | 1 | 0 |
| *Geonoma laxiflora* | 0 | 1 | 0 | 0 | 0 | 0 | 0 | 0 | 0 | 0 | 0 | 0 | 0 | 0 | 0 | 0 | 0 | 0 | 1 | 0 |
| *Geonoma leptospadix* | 0 | 1 | 0 | 0 | 0 | 0 | 1 | 1 | 0 | 1 | 0 | 1 | 0 | 0 | 1 | 0 | 0 | 0 | 1 | 0 |
| *Geonoma linearis* | 0 | 0 | 0 | 0 | 0 | 0 | 0 | 0 | 0 | 1 | 0 | 0 | 0 | 0 | 0 | 0 | 0 | 0 | 0 | 0 |
| *Geonoma longipedunculata* | 0 | 0 | 0 | 0 | 0 | 0 | 0 | 0 | 0 | 0 | 0 | 0 | 0 | 0 | 0 | 0 | 0 | 0 | 1 | 0 |
| *Geonoma longivaginata* | 0 | 0 | 1 | 0 | 0 | 0 | 0 | 0 | 0 | 1 | 0 | 0 | 0 | 0 | 0 | 0 | 0 | 0 | 0 | 0 |
| *Geonoma macrostachys* | 0 | 1 | 1 | 0 | 0 | 1 | 1 | 1 | 0 | 1 | 0 | 1 | 0 | 0 | 1 | 0 | 0 | 0 | 1 | 0 |
| *Geonoma maxima* | 0 | 1 | 0 | 0 | 0 | 0 | 1 | 1 | 0 | 0 | 0 | 1 | 0 | 0 | 1 | 0 | 0 | 0 | 1 | 0 |
| *Geonoma oldemanii* | 0 | 1 | 0 | 0 | 0 | 0 | 0 | 0 | 0 | 0 | 0 | 1 | 0 | 0 | 0 | 0 | 0 | 0 | 0 | 0 |
| *Geonoma orbignyana* | 0 | 0 | 1 | 0 | 0 | 0 | 1 | 0 | 0 | 1 | 0 | 0 | 0 | 0 | 1 | 0 | 0 | 0 | 1 | 0 |
| *Geonoma paradoxa* | 0 | 0 | 0 | 0 | 0 | 0 | 0 | 0 | 0 | 1 | 0 | 0 | 0 | 0 | 1 | 0 | 0 | 0 | 1 | 0 |
| *Geonoma pauciflora* | 1 | 0 | 0 | 1 | 0 | 0 | 1 | 1 | 0 | 0 | 1 | 0 | 0 | 0 | 1 | 0 | 0 | 0 | 0 | 0 |
| *Geonoma poeppigiana* | 0 | 0 | 0 | 0 | 0 | 0 | 1 | 0 | 0 | 0 | 0 | 0 | 0 | 1 | 1 | 0 | 0 | 0 | 1 | 0 |
| *Geonoma pohliana* | 1 | 1 | 0 | 1 | 0 | 0 | 0 | 1 | 0 | 0 | 0 | 0 | 0 | 0 | 0 | 0 | 0 | 0 | 0 | 0 |
| *Geonoma polyandra* | 0 | 0 | 0 | 0 | 0 | 0 | 0 | 0 | 0 | 0 | 0 | 0 | 0 | 0 | 1 | 0 | 0 | 0 | 1 | 0 |
| *Geonoma rubescens* | 1 | 0 | 0 | 0 | 0 | 0 | 0 | 0 | 0 | 0 | 0 | 0 | 0 | 0 | 0 | 0 | 0 | 0 | 0 | 0 |
| *Geonoma schottiana* | 1 | 0 | 0 | 1 | 0 | 0 | 0 | 1 | 0 | 0 | 1 | 0 | 0 | 0 | 1 | 0 | 0 | 0 | 0 | 0 |
| *Geonoma scoparia* | 0 | 0 | 1 | 0 | 0 | 0 | 0 | 0 | 0 | 0 | 0 | 0 | 0 | 0 | 0 | 0 | 0 | 0 | 0 | 0 |
| *Geonoma simplicifrons* | 0 | 0 | 0 | 0 | 0 | 0 | 0 | 1 | 0 | 0 | 0 | 0 | 0 | 1 | 1 | 0 | 0 | 0 | 0 | 0 |
| *Geonoma spinescens* | 0 | 0 | 0 | 0 | 0 | 0 | 0 | 0 | 0 | 0 | 0 | 0 | 0 | 1 | 1 | 0 | 0 | 0 | 0 | 0 |
| *Geonoma stricta* | 0 | 1 | 0 | 0 | 0 | 0 | 1 | 1 | 0 | 1 | 0 | 1 | 0 | 1 | 1 | 0 | 0 | 0 | 1 | 0 |
| *Geonoma tenuissima* | 0 | 0 | 0 | 0 | 0 | 0 | 0 | 0 | 0 | 1 | 0 | 0 | 0 | 0 | 1 | 0 | 0 | 0 | 0 | 0 |
| *Geonoma triandra* | 0 | 0 | 0 | 0 | 0 | 0 | 0 | 0 | 0 | 1 | 0 | 0 | 0 | 0 | 1 | 0 | 0 | 0 | 0 | 0 |
| *Geonoma triglochin* | 0 | 0 | 0 | 0 | 0 | 0 | 1 | 0 | 0 | 0 | 0 | 1 | 0 | 0 | 1 | 0 | 0 | 0 | 1 | 0 |
| *Geonoma trigona* | 0 | 0 | 0 | 0 | 0 | 0 | 1 | 0 | 0 | 0 | 0 | 0 | 0 | 0 | 1 | 0 | 0 | 0 | 1 | 0 |
| *Geonoma umbraculiformis* | 0 | 1 | 0 | 0 | 0 | 0 | 0 | 0 | 0 | 0 | 0 | 1 | 0 | 0 | 0 | 0 | 0 | 0 | 0 | 0 |
| *Geonoma undata* | 0 | 0 | 1 | 0 | 1 | 0 | 1 | 0 | 0 | 1 | 0 | 1 | 1 | 1 | 1 | 0 | 0 | 0 | 1 | 0 |
| *Geonoma weberbaueri* | 0 | 0 | 0 | 0 | 0 | 0 | 1 | 0 | 0 | 0 | 0 | 0 | 0 | 0 | 1 | 0 | 0 | 0 | 1 | 0 |
| *Hemithrinax compacta* | 0 | 0 | 0 | 0 | 1 | 0 | 0 | 0 | 0 | 0 | 0 | 0 | 0 | 0 | 0 | 0 | 0 | 0 | 0 | 0 |
| *Hemithrinax rivularis* | 0 | 0 | 0 | 0 | 1 | 0 | 0 | 0 | 0 | 0 | 0 | 0 | 0 | 0 | 0 | 0 | 0 | 0 | 0 | 0 |
| *Hyospathe elegans* | 0 | 1 | 1 | 0 | 0 | 1 | 1 | 1 | 0 | 1 | 0 | 1 | 0 | 0 | 1 | 0 | 0 | 0 | 1 | 0 |
| *Hyospathe macrorhachis* | 0 | 0 | 0 | 0 | 0 | 0 | 1 | 0 | 0 | 0 | 0 | 0 | 0 | 0 | 1 | 0 | 0 | 0 | 0 | 0 |
| *Iriartea deltoidea* | 0 | 1 | 1 | 0 | 0 | 0 | 1 | 0 | 0 | 1 | 0 | 1 | 0 | 0 | 1 | 0 | 0 | 0 | 1 | 0 |
| *Iriartella setigera* | 0 | 1 | 0 | 0 | 0 | 0 | 1 | 1 | 0 | 0 | 0 | 1 | 0 | 0 | 0 | 0 | 0 | 0 | 1 | 0 |
| *Iriartella stenocarpa* | 0 | 0 | 0 | 0 | 0 | 0 | 1 | 0 | 0 | 0 | 0 | 0 | 0 | 0 | 0 | 0 | 0 | 0 | 1 | 0 |
| *Itaya amicorum* | 0 | 0 | 0 | 0 | 0 | 0 | 0 | 0 | 0 | 0 | 0 | 0 | 0 | 0 | 0 | 0 | 0 | 0 | 1 | 0 |
| *Juania australis* | 0 | 0 | 0 | 0 | 0 | 0 | 1 | 0 | 0 | 0 | 0 | 0 | 0 | 0 | 0 | 0 | 0 | 0 | 0 | 0 |
| *Jubaea chilensis* | 0 | 0 | 0 | 0 | 0 | 0 | 1 | 0 | 0 | 0 | 0 | 0 | 0 | 0 | 0 | 0 | 0 | 0 | 0 | 0 |
| *Leopoldinia piassaba* | 0 | 1 | 0 | 0 | 0 | 0 | 0 | 0 | 0 | 0 | 0 | 0 | 0 | 1 | 0 | 0 | 0 | 0 | 0 | 0 |
| *Leopoldinia pulchra* | 0 | 1 | 0 | 0 | 0 | 0 | 0 | 0 | 0 | 0 | 0 | 1 | 0 | 1 | 0 | 0 | 0 | 0 | 1 | 0 |
| *Lepidocaryum tenue* | 0 | 1 | 0 | 0 | 0 | 0 | 0 | 0 | 0 | 0 | 0 | 1 | 0 | 0 | 0 | 0 | 0 | 0 | 1 | 0 |
| *Leucothrinax morrisii* | 0 | 0 | 0 | 0 | 1 | 0 | 0 | 0 | 0 | 0 | 0 | 0 | 0 | 0 | 0 | 0 | 1 | 0 | 0 | 0 |
| *Lytocaryum hoehnei* | 1 | 0 | 0 | 0 | 0 | 0 | 0 | 1 | 0 | 0 | 0 | 0 | 0 | 0 | 0 | 0 | 0 | 0 | 0 | 0 |
| *Lytocaryum weddellianum* | 1 | 0 | 0 | 0 | 0 | 0 | 0 | 0 | 0 | 0 | 0 | 0 | 0 | 0 | 0 | 0 | 0 | 0 | 0 | 0 |
| *Manicaria saccifera* | 0 | 1 | 1 | 0 | 0 | 0 | 0 | 0 | 0 | 1 | 0 | 1 | 0 | 0 | 0 | 0 | 0 | 0 | 1 | 0 |
| *Mauritia carana* | 0 | 1 | 0 | 0 | 0 | 0 | 0 | 0 | 0 | 0 | 0 | 0 | 0 | 0 | 0 | 0 | 0 | 0 | 1 | 0 |
| *Mauritia flexuosa* | 1 | 1 | 1 | 0 | 0 | 0 | 1 | 1 | 0 | 0 | 0 | 1 | 0 | 1 | 1 | 0 | 0 | 0 | 1 | 0 |
| *Mauritiella aculeata* | 0 | 1 | 0 | 0 | 0 | 0 | 0 | 1 | 0 | 0 | 0 | 1 | 0 | 1 | 0 | 0 | 0 | 0 | 1 | 0 |
| *Mauritiella armata* | 0 | 1 | 0 | 0 | 0 | 0 | 0 | 1 | 0 | 0 | 0 | 1 | 0 | 0 | 0 | 0 | 0 | 0 | 1 | 0 |
| *Mauritiella macroclada* | 0 | 0 | 0 | 0 | 0 | 0 | 0 | 0 | 0 | 1 | 0 | 0 | 0 | 0 | 1 | 0 | 0 | 0 | 0 | 0 |
| *Neonicholsonia watsonii* | 0 | 0 | 1 | 0 | 0 | 0 | 0 | 0 | 0 | 0 | 0 | 0 | 0 | 0 | 0 | 0 | 0 | 0 | 0 | 0 |
| *Oenocarpus bacaba* | 0 | 1 | 0 | 0 | 0 | 0 | 0 | 0 | 0 | 0 | 0 | 1 | 0 | 1 | 0 | 0 | 0 | 0 | 1 | 0 |
| *Oenocarpus balickii* | 0 | 1 | 0 | 0 | 0 | 0 | 0 | 0 | 0 | 0 | 0 | 1 | 0 | 0 | 1 | 0 | 0 | 0 | 1 | 0 |
| *Oenocarpus bataua* | 0 | 1 | 0 | 0 | 0 | 1 | 1 | 1 | 0 | 1 | 0 | 1 | 0 | 1 | 1 | 0 | 0 | 0 | 1 | 0 |
| *Oenocarpus circumtextus* | 0 | 0 | 0 | 0 | 0 | 0 | 0 | 0 | 0 | 0 | 0 | 0 | 0 | 0 | 0 | 0 | 0 | 0 | 1 | 0 |
| *Oenocarpus distichus* | 0 | 1 | 0 | 0 | 0 | 0 | 1 | 1 | 0 | 0 | 0 | 0 | 0 | 0 | 0 | 0 | 0 | 0 | 1 | 0 |
| *Oenocarpus mapora* | 0 | 1 | 1 | 0 | 0 | 1 | 1 | 0 | 0 | 1 | 0 | 0 | 1 | 1 | 1 | 0 | 0 | 0 | 1 | 0 |
| *Oenocarpus minor* | 0 | 1 | 0 | 0 | 0 | 0 | 1 | 0 | 0 | 1 | 0 | 0 | 1 | 1 | 1 | 0 | 0 | 0 | 1 | 0 |
| *Oenocarpus simplex* | 0 | 0 | 0 | 0 | 0 | 0 | 0 | 0 | 0 | 0 | 0 | 0 | 0 | 0 | 1 | 0 | 0 | 0 | 0 | 0 |
| *Parajubaea cocoides* | 0 | 0 | 0 | 0 | 0 | 0 | 0 | 0 | 0 | 1 | 0 | 0 | 0 | 0 | 1 | 0 | 0 | 0 | 0 | 0 |
| *Parajubaea torallyi* | 0 | 0 | 0 | 0 | 0 | 0 | 1 | 0 | 0 | 0 | 0 | 0 | 0 | 0 | 0 | 0 | 0 | 0 | 0 | 0 |
| *Pholidostachys dactyloides* | 0 | 0 | 1 | 0 | 0 | 0 | 0 | 0 | 0 | 1 | 0 | 0 | 0 | 0 | 1 | 0 | 0 | 0 | 0 | 0 |
| *Pholidostachys kalbreyeri* | 0 | 0 | 1 | 0 | 0 | 0 | 0 | 0 | 0 | 1 | 0 | 0 | 0 | 0 | 0 | 0 | 0 | 0 | 0 | 0 |
| *Pholidostachys pulchra* | 0 | 0 | 1 | 0 | 0 | 0 | 0 | 0 | 0 | 1 | 0 | 0 | 0 | 0 | 0 | 0 | 0 | 0 | 0 | 0 |
| *Pholidostachys synanthera* | 0 | 0 | 0 | 0 | 0 | 0 | 1 | 1 | 0 | 1 | 0 | 0 | 0 | 0 | 1 | 0 | 0 | 0 | 1 | 0 |
| *Phytelephas aequatorialis* | 0 | 0 | 0 | 0 | 0 | 0 | 0 | 0 | 0 | 1 | 0 | 0 | 0 | 0 | 1 | 0 | 0 | 0 | 0 | 0 |
| *Phytelephas macrocarpa* | 0 | 0 | 0 | 0 | 0 | 0 | 1 | 0 | 0 | 1 | 0 | 0 | 0 | 0 | 1 | 0 | 0 | 0 | 1 | 0 |
| *Phytelephas seemannii* | 0 | 0 | 1 | 0 | 0 | 0 | 0 | 0 | 0 | 1 | 0 | 0 | 0 | 0 | 1 | 0 | 0 | 0 | 0 | 0 |
| *Phytelephas tenuicaulis* | 0 | 0 | 0 | 0 | 0 | 0 | 1 | 0 | 0 | 0 | 0 | 0 | 0 | 0 | 1 | 0 | 0 | 0 | 1 | 0 |
| *Phytelephas tumacana* | 0 | 0 | 0 | 0 | 0 | 0 | 0 | 0 | 0 | 1 | 0 | 0 | 0 | 0 | 0 | 0 | 0 | 0 | 0 | 0 |
| *Prestoea acuminata* | 0 | 0 | 1 | 0 | 1 | 0 | 1 | 0 | 0 | 1 | 0 | 0 | 0 | 0 | 1 | 0 | 0 | 1 | 1 | 0 |
| *Prestoea carderi* | 0 | 0 | 0 | 0 | 0 | 0 | 1 | 0 | 0 | 0 | 0 | 0 | 0 | 0 | 1 | 0 | 0 | 0 | 1 | 0 |
| *Prestoea decurrens* | 0 | 0 | 1 | 0 | 0 | 0 | 0 | 0 | 0 | 1 | 0 | 0 | 0 | 0 | 1 | 0 | 0 | 1 | 0 | 0 |
| *Prestoea ensiformis* | 0 | 0 | 1 | 0 | 0 | 0 | 1 | 0 | 0 | 1 | 0 | 0 | 0 | 0 | 1 | 0 | 0 | 0 | 1 | 0 |
| *Prestoea longipetiolata* | 0 | 0 | 1 | 0 | 0 | 0 | 0 | 0 | 0 | 0 | 0 | 0 | 0 | 0 | 1 | 0 | 0 | 0 | 0 | 0 |
| *Prestoea pubens* | 0 | 0 | 1 | 0 | 0 | 0 | 0 | 0 | 0 | 1 | 0 | 0 | 0 | 0 | 0 | 0 | 0 | 0 | 0 | 0 |
| *Prestoea pubigera* | 0 | 0 | 0 | 0 | 0 | 0 | 0 | 0 | 0 | 0 | 0 | 0 | 0 | 1 | 0 | 0 | 0 | 0 | 0 | 0 |
| *Prestoea schultzeana* | 0 | 1 | 1 | 0 | 0 | 0 | 1 | 0 | 0 | 0 | 0 | 0 | 0 | 0 | 1 | 0 | 0 | 0 | 1 | 0 |
| *Prestoea simplicifolia* | 0 | 0 | 0 | 0 | 0 | 0 | 0 | 0 | 0 | 1 | 0 | 0 | 0 | 0 | 1 | 0 | 0 | 0 | 0 | 0 |
| *Prestoea tenuiramosa* | 0 | 0 | 0 | 0 | 0 | 0 | 0 | 0 | 0 | 0 | 0 | 1 | 0 | 0 | 0 | 0 | 0 | 0 | 0 | 0 |
| *Pseudophoenix sargentii* | 0 | 0 | 1 | 0 | 1 | 0 | 0 | 0 | 0 | 0 | 0 | 0 | 0 | 0 | 0 | 0 | 1 | 1 | 0 | 0 |
| *Pseudophoenix vinifera* | 0 | 0 | 0 | 0 | 1 | 0 | 0 | 0 | 0 | 0 | 0 | 0 | 0 | 0 | 0 | 0 | 0 | 0 | 0 | 0 |
| *Raphia taedigera* | 0 | 0 | 1 | 0 | 0 | 0 | 0 | 0 | 0 | 1 | 0 | 0 | 0 | 0 | 0 | 0 | 0 | 0 | 0 | 0 |
| *Reinhardtia elegans* | 0 | 0 | 0 | 0 | 0 | 0 | 0 | 0 | 0 | 0 | 0 | 0 | 0 | 0 | 0 | 0 | 0 | 1 | 0 | 0 |
| *Reinhardtia gracilis* | 0 | 0 | 1 | 0 | 0 | 0 | 0 | 0 | 0 | 1 | 0 | 0 | 0 | 0 | 0 | 0 | 0 | 1 | 0 | 0 |
| *Reinhardtia koschnyana* | 0 | 0 | 1 | 0 | 0 | 0 | 0 | 0 | 0 | 1 | 0 | 0 | 0 | 0 | 1 | 0 | 0 | 0 | 0 | 0 |
| *Reinhardtia latisecta* | 0 | 0 | 1 | 0 | 0 | 0 | 0 | 0 | 0 | 0 | 0 | 0 | 0 | 0 | 0 | 0 | 0 | 1 | 0 | 0 |
| *Reinhardtia paiewonskiana* | 0 | 0 | 0 | 0 | 1 | 0 | 0 | 0 | 0 | 0 | 0 | 0 | 0 | 0 | 0 | 0 | 0 | 0 | 0 | 0 |
| *Reinhardtia simplex* | 0 | 0 | 1 | 0 | 0 | 0 | 0 | 0 | 0 | 1 | 0 | 0 | 0 | 0 | 0 | 0 | 0 | 1 | 0 | 0 |
| *Rhapidophyllum hystrix* | 0 | 0 | 0 | 0 | 0 | 0 | 0 | 0 | 0 | 0 | 0 | 0 | 0 | 0 | 0 | 0 | 1 | 0 | 0 | 0 |
| *Roystonea altissima* | 0 | 0 | 0 | 0 | 1 | 0 | 0 | 0 | 0 | 0 | 0 | 0 | 0 | 0 | 0 | 0 | 0 | 0 | 0 | 0 |
| *Roystonea borinquena* | 0 | 0 | 0 | 0 | 1 | 0 | 0 | 0 | 0 | 0 | 0 | 0 | 0 | 0 | 0 | 0 | 0 | 0 | 0 | 0 |
| *Roystonea dunlapiana* | 0 | 0 | 1 | 0 | 0 | 0 | 0 | 0 | 0 | 0 | 0 | 0 | 0 | 0 | 0 | 0 | 0 | 0 | 0 | 0 |
| *Roystonea lenis* | 0 | 0 | 0 | 0 | 1 | 0 | 0 | 0 | 0 | 0 | 0 | 0 | 0 | 0 | 0 | 0 | 0 | 0 | 0 | 0 |
| *Roystonea oleracea* | 1 | 0 | 1 | 0 | 1 | 0 | 0 | 0 | 0 | 0 | 0 | 1 | 0 | 1 | 0 | 0 | 0 | 0 | 0 | 0 |
| *Roystonea princeps* | 0 | 0 | 0 | 0 | 1 | 0 | 0 | 0 | 0 | 0 | 0 | 0 | 0 | 0 | 0 | 0 | 0 | 0 | 0 | 0 |
| *Roystonea regia* | 1 | 0 | 1 | 0 | 1 | 0 | 0 | 1 | 0 | 0 | 0 | 0 | 1 | 0 | 1 | 0 | 1 | 1 | 0 | 0 |
| *Roystonea violacea* | 0 | 0 | 0 | 0 | 1 | 0 | 0 | 0 | 0 | 0 | 0 | 0 | 0 | 0 | 0 | 0 | 0 | 0 | 0 | 0 |
| *Sabal causiarum* | 0 | 0 | 0 | 0 | 1 | 0 | 0 | 0 | 0 | 0 | 0 | 0 | 0 | 0 | 0 | 0 | 1 | 0 | 0 | 0 |
| *Sabal domingensis* | 0 | 0 | 1 | 0 | 1 | 0 | 0 | 0 | 0 | 0 | 0 | 0 | 0 | 0 | 0 | 0 | 0 | 0 | 0 | 0 |
| *Sabal etonia* | 0 | 0 | 0 | 0 | 0 | 0 | 0 | 0 | 0 | 0 | 0 | 0 | 0 | 0 | 0 | 0 | 1 | 0 | 0 | 0 |
| *Sabal gretherae* | 0 | 0 | 1 | 0 | 0 | 0 | 0 | 0 | 0 | 0 | 0 | 0 | 0 | 0 | 0 | 0 | 0 | 1 | 0 | 0 |
| *Sabal maritima* | 0 | 0 | 0 | 0 | 1 | 0 | 0 | 0 | 0 | 0 | 0 | 0 | 0 | 0 | 0 | 0 | 0 | 0 | 0 | 0 |
| *Sabal mauritiiformis* | 0 | 0 | 1 | 0 | 1 | 1 | 0 | 0 | 0 | 1 | 0 | 0 | 1 | 1 | 1 | 0 | 0 | 0 | 0 | 0 |
| *Sabal mexicana* | 0 | 0 | 1 | 0 | 0 | 0 | 0 | 0 | 0 | 0 | 0 | 0 | 0 | 0 | 0 | 0 | 0 | 1 | 0 | 1 |
| *Sabal minor* | 1 | 0 | 1 | 0 | 0 | 0 | 0 | 0 | 0 | 0 | 0 | 0 | 0 | 0 | 0 | 0 | 1 | 0 | 0 | 1 |
| *Sabal palmetto* | 1 | 0 | 1 | 1 | 1 | 0 | 0 | 0 | 0 | 0 | 0 | 0 | 0 | 0 | 0 | 0 | 1 | 0 | 0 | 0 |
| *Sabal pumos* | 0 | 0 | 0 | 0 | 0 | 0 | 0 | 0 | 0 | 0 | 0 | 0 | 0 | 0 | 0 | 0 | 0 | 1 | 0 | 1 |
| *Sabal rosei* | 0 | 0 | 0 | 0 | 0 | 0 | 0 | 0 | 0 | 0 | 0 | 0 | 0 | 0 | 0 | 0 | 0 | 1 | 0 | 1 |
| *Sabal uresana* | 0 | 0 | 0 | 0 | 0 | 0 | 0 | 0 | 0 | 0 | 0 | 0 | 0 | 0 | 0 | 0 | 0 | 1 | 0 | 1 |
| *Sabal yapa* | 0 | 0 | 1 | 0 | 0 | 0 | 0 | 0 | 0 | 0 | 0 | 0 | 0 | 0 | 0 | 0 | 0 | 1 | 0 | 0 |
| *Schippia concolor* | 0 | 0 | 1 | 0 | 0 | 0 | 0 | 0 | 0 | 0 | 0 | 0 | 0 | 0 | 0 | 0 | 0 | 0 | 0 | 0 |
| *Serenoa repens* | 0 | 0 | 1 | 0 | 0 | 0 | 0 | 0 | 0 | 0 | 0 | 0 | 0 | 0 | 0 | 0 | 1 | 0 | 0 | 0 |
| *Socratea exorrhiza* | 0 | 1 | 1 | 1 | 0 | 1 | 1 | 1 | 0 | 1 | 0 | 1 | 0 | 1 | 1 | 0 | 0 | 0 | 1 | 0 |
| *Socratea hecatonandra* | 0 | 0 | 0 | 0 | 0 | 0 | 0 | 0 | 0 | 1 | 0 | 0 | 0 | 0 | 1 | 0 | 0 | 0 | 0 | 0 |
| *Socratea montana* | 0 | 0 | 0 | 0 | 0 | 0 | 0 | 0 | 0 | 0 | 0 | 0 | 0 | 0 | 1 | 0 | 0 | 0 | 0 | 0 |
| *Socratea rostrata* | 0 | 0 | 0 | 0 | 0 | 0 | 0 | 0 | 0 | 1 | 0 | 0 | 0 | 0 | 1 | 0 | 0 | 0 | 1 | 0 |
| *Socratea salazarii* | 0 | 0 | 0 | 0 | 0 | 0 | 1 | 0 | 0 | 0 | 0 | 0 | 0 | 0 | 1 | 0 | 0 | 0 | 1 | 0 |
| *Syagrus amara* | 0 | 0 | 0 | 0 | 1 | 0 | 0 | 0 | 0 | 0 | 0 | 0 | 0 | 0 | 0 | 0 | 0 | 0 | 0 | 0 |
| *Syagrus botryophora* | 1 | 0 | 0 | 0 | 0 | 0 | 0 | 0 | 0 | 0 | 0 | 0 | 0 | 0 | 0 | 0 | 0 | 0 | 0 | 0 |
| *Syagrus campylospatha* | 1 | 0 | 0 | 0 | 0 | 0 | 0 | 1 | 1 | 0 | 0 | 0 | 0 | 0 | 0 | 0 | 0 | 0 | 0 | 0 |
| *Syagrus cardenasii* | 0 | 0 | 0 | 0 | 0 | 0 | 1 | 1 | 1 | 0 | 0 | 0 | 0 | 0 | 0 | 1 | 0 | 0 | 0 | 0 |
| *Syagrus cocoides* | 0 | 1 | 0 | 1 | 0 | 0 | 0 | 1 | 0 | 0 | 0 | 1 | 0 | 0 | 0 | 0 | 0 | 0 | 0 | 0 |
| *Syagrus comosa* | 1 | 1 | 0 | 1 | 0 | 0 | 0 | 1 | 0 | 0 | 0 | 0 | 0 | 0 | 0 | 0 | 0 | 0 | 0 | 0 |
| *Syagrus coronata* | 1 | 0 | 0 | 1 | 0 | 0 | 0 | 1 | 0 | 0 | 0 | 0 | 0 | 0 | 0 | 0 | 0 | 0 | 0 | 0 |
| *Syagrus duartei* | 0 | 0 | 0 | 0 | 0 | 0 | 0 | 1 | 0 | 0 | 0 | 0 | 0 | 0 | 0 | 0 | 0 | 0 | 0 | 0 |
| *Syagrus flexuosa* | 1 | 1 | 0 | 1 | 0 | 0 | 0 | 1 | 0 | 0 | 0 | 0 | 0 | 0 | 0 | 1 | 0 | 0 | 0 | 0 |
| *Syagrus glaucescens* | 1 | 0 | 0 | 0 | 0 | 0 | 0 | 1 | 0 | 0 | 0 | 0 | 0 | 0 | 0 | 0 | 0 | 0 | 0 | 0 |
| *Syagrus graminifolia* | 1 | 0 | 0 | 0 | 0 | 0 | 0 | 1 | 0 | 0 | 0 | 0 | 0 | 0 | 0 | 0 | 0 | 0 | 0 | 0 |
| *Syagrus harleyi* | 1 | 0 | 0 | 1 | 0 | 0 | 0 | 1 | 0 | 0 | 0 | 0 | 0 | 0 | 0 | 0 | 0 | 0 | 0 | 0 |
| *Syagrus inajai* | 0 | 1 | 0 | 0 | 0 | 0 | 0 | 1 | 0 | 0 | 0 | 1 | 0 | 1 | 0 | 0 | 0 | 0 | 1 | 0 |
| *Syagrus macrocarpa* | 1 | 0 | 0 | 1 | 0 | 0 | 0 | 1 | 0 | 0 | 0 | 0 | 0 | 0 | 0 | 0 | 0 | 0 | 0 | 0 |
| *Syagrus microphylla* | 0 | 0 | 0 | 1 | 0 | 0 | 0 | 1 | 0 | 0 | 0 | 0 | 0 | 0 | 0 | 0 | 0 | 0 | 0 | 0 |
| *Syagrus oleracea* | 1 | 1 | 0 | 1 | 0 | 0 | 0 | 1 | 1 | 0 | 0 | 0 | 0 | 0 | 0 | 0 | 0 | 0 | 0 | 0 |
| *Syagrus orinocensis* | 0 | 1 | 0 | 0 | 0 | 0 | 0 | 0 | 0 | 0 | 0 | 1 | 0 | 1 | 1 | 0 | 1 | 0 | 1 | 0 |
| *Syagrus petraea* | 1 | 1 | 0 | 1 | 0 | 0 | 0 | 1 | 1 | 0 | 0 | 0 | 0 | 0 | 0 | 0 | 0 | 0 | 0 | 0 |
| *Syagrus picrophylla* | 1 | 0 | 0 | 0 | 0 | 0 | 0 | 0 | 0 | 0 | 0 | 0 | 0 | 0 | 0 | 0 | 0 | 0 | 0 | 0 |
| *Syagrus pleioclada* | 1 | 0 | 0 | 0 | 0 | 0 | 0 | 1 | 0 | 0 | 0 | 0 | 0 | 0 | 0 | 0 | 0 | 0 | 0 | 0 |
| *Syagrus pseudococos* | 1 | 0 | 0 | 1 | 0 | 0 | 0 | 0 | 0 | 0 | 0 | 0 | 0 | 0 | 0 | 0 | 0 | 0 | 0 | 0 |
| *Syagrus romanzoffiana* | 1 | 0 | 0 | 0 | 0 | 0 | 1 | 1 | 1 | 0 | 1 | 0 | 0 | 0 | 1 | 0 | 1 | 1 | 1 | 0 |
| *Syagrus ruschiana* | 1 | 0 | 0 | 1 | 0 | 0 | 0 | 0 | 0 | 0 | 0 | 0 | 0 | 0 | 0 | 0 | 0 | 0 | 0 | 0 |
| *Syagrus sancona* | 0 | 1 | 0 | 0 | 0 | 0 | 1 | 1 | 0 | 1 | 0 | 0 | 1 | 1 | 1 | 0 | 0 | 0 | 1 | 0 |
| *Syagrus schizophylla* | 1 | 0 | 0 | 1 | 0 | 0 | 0 | 0 | 0 | 0 | 0 | 0 | 0 | 0 | 0 | 0 | 1 | 0 | 0 | 0 |
| *Syagrus smithii* | 0 | 1 | 0 | 0 | 0 | 0 | 0 | 0 | 0 | 0 | 0 | 0 | 0 | 0 | 1 | 0 | 0 | 0 | 1 | 0 |
| *Syagrus stratincola* | 0 | 0 | 0 | 0 | 0 | 0 | 0 | 0 | 0 | 0 | 0 | 1 | 0 | 0 | 0 | 0 | 0 | 0 | 0 | 0 |
| *Syagrus vagans* | 1 | 0 | 0 | 1 | 0 | 0 | 0 | 1 | 0 | 0 | 0 | 0 | 0 | 0 | 0 | 0 | 0 | 0 | 0 | 0 |
| *Syagrus werdermannii* | 0 | 0 | 0 | 1 | 0 | 0 | 0 | 1 | 0 | 0 | 0 | 0 | 0 | 0 | 0 | 0 | 0 | 0 | 0 | 0 |
| *Synechanthus fibrosus* | 0 | 0 | 1 | 0 | 0 | 0 | 0 | 0 | 0 | 0 | 0 | 0 | 0 | 0 | 0 | 0 | 0 | 1 | 0 | 0 |
| *Synechanthus warscewiczianus* | 0 | 0 | 1 | 0 | 0 | 0 | 0 | 0 | 0 | 1 | 0 | 0 | 0 | 0 | 1 | 0 | 0 | 1 | 0 | 0 |
| *Thrinax parviflora* | 0 | 0 | 0 | 0 | 1 | 0 | 0 | 0 | 0 | 0 | 0 | 0 | 0 | 0 | 0 | 0 | 1 | 0 | 0 | 0 |
| *Thrinax radiata* | 0 | 0 | 1 | 0 | 1 | 0 | 0 | 0 | 0 | 0 | 0 | 0 | 0 | 0 | 0 | 0 | 1 | 1 | 0 | 0 |
| *Trithrinax brasiliensis* | 1 | 0 | 0 | 0 | 0 | 0 | 0 | 1 | 0 | 0 | 1 | 0 | 0 | 0 | 0 | 0 | 0 | 0 | 0 | 0 |
| *Trithrinax campestris* | 0 | 0 | 0 | 0 | 0 | 0 | 1 | 0 | 1 | 0 | 1 | 0 | 0 | 0 | 0 | 0 | 0 | 0 | 0 | 0 |
| *Trithrinax schizophylla* | 0 | 0 | 0 | 0 | 0 | 0 | 1 | 1 | 1 | 0 | 0 | 0 | 0 | 0 | 0 | 0 | 0 | 0 | 0 | 0 |
| *Washingtonia filifera* | 0 | 0 | 0 | 0 | 0 | 0 | 0 | 0 | 0 | 1 | 0 | 0 | 0 | 0 | 0 | 0 | 0 | 0 | 0 | 1 |
| *Washingtonia robusta* | 0 | 0 | 0 | 0 | 1 | 0 | 0 | 0 | 0 | 0 | 0 | 0 | 0 | 0 | 0 | 0 | 0 | 1 | 0 | 1 |
| *Welfia regia* | 0 | 0 | 1 | 0 | 0 | 0 | 0 | 0 | 0 | 1 | 0 | 0 | 0 | 0 | 1 | 0 | 0 | 1 | 0 | 0 |
| *Wendlandiella gracilis* | 0 | 0 | 0 | 0 | 0 | 0 | 1 | 0 | 0 | 0 | 0 | 0 | 0 | 0 | 0 | 0 | 0 | 0 | 1 | 0 |
| *Wettinia aequalis* | 0 | 0 | 0 | 0 | 0 | 0 | 0 | 0 | 0 | 1 | 0 | 0 | 0 | 0 | 1 | 0 | 0 | 0 | 1 | 0 |
| *Wettinia aequatorialis* | 0 | 0 | 0 | 0 | 0 | 0 | 0 | 0 | 0 | 0 | 0 | 0 | 0 | 0 | 1 | 0 | 0 | 0 | 0 | 0 |
| *Wettinia anomala* | 0 | 0 | 0 | 0 | 0 | 0 | 0 | 0 | 0 | 0 | 0 | 0 | 0 | 0 | 1 | 0 | 0 | 0 | 1 | 0 |
| *Wettinia augusta* | 0 | 0 | 0 | 0 | 0 | 0 | 1 | 0 | 0 | 0 | 0 | 0 | 0 | 0 | 0 | 0 | 0 | 0 | 1 | 0 |
| *Wettinia castanea* | 0 | 0 | 0 | 0 | 0 | 0 | 0 | 0 | 0 | 0 | 0 | 0 | 0 | 0 | 1 | 0 | 0 | 0 | 0 | 0 |
| *Wettinia disticha* | 0 | 0 | 0 | 0 | 0 | 0 | 0 | 0 | 0 | 0 | 0 | 0 | 0 | 1 | 1 | 0 | 0 | 0 | 0 | 0 |
| *Wettinia drudei* | 0 | 1 | 0 | 0 | 0 | 0 | 0 | 0 | 0 | 0 | 0 | 0 | 0 | 0 | 1 | 0 | 0 | 0 | 1 | 0 |
| *Wettinia fascicularis* | 0 | 0 | 0 | 0 | 0 | 0 | 0 | 0 | 0 | 0 | 0 | 0 | 0 | 0 | 1 | 0 | 0 | 0 | 0 | 0 |
| *Wettinia hirsuta* | 0 | 0 | 0 | 0 | 0 | 0 | 0 | 0 | 0 | 1 | 0 | 0 | 0 | 0 | 1 | 0 | 0 | 0 | 1 | 0 |
| *Wettinia kalbreyeri* | 0 | 0 | 0 | 0 | 0 | 0 | 0 | 0 | 0 | 1 | 0 | 0 | 1 | 1 | 1 | 0 | 0 | 0 | 0 | 0 |
| *Wettinia lanata* | 0 | 0 | 0 | 0 | 0 | 0 | 0 | 0 | 0 | 0 | 0 | 0 | 0 | 0 | 1 | 0 | 0 | 0 | 0 | 0 |
| *Wettinia longipetala* | 0 | 0 | 0 | 0 | 0 | 0 | 1 | 0 | 0 | 0 | 0 | 0 | 0 | 0 | 1 | 0 | 0 | 0 | 0 | 0 |
| *Wettinia maynensis* | 0 | 0 | 0 | 0 | 0 | 0 | 0 | 0 | 0 | 0 | 0 | 0 | 0 | 0 | 1 | 0 | 0 | 0 | 1 | 0 |
| *Wettinia microcarpa* | 0 | 0 | 0 | 0 | 0 | 0 | 0 | 0 | 0 | 0 | 0 | 0 | 0 | 0 | 1 | 0 | 0 | 0 | 0 | 0 |
| *Wettinia minima* | 0 | 0 | 0 | 0 | 0 | 0 | 0 | 0 | 0 | 0 | 0 | 0 | 0 | 0 | 1 | 0 | 0 | 0 | 0 | 0 |
| *Wettinia oxycarpa* | 0 | 0 | 0 | 0 | 0 | 0 | 0 | 0 | 0 | 0 | 0 | 0 | 0 | 0 | 1 | 0 | 0 | 0 | 0 | 0 |
| *Wettinia praemorsa* | 0 | 0 | 0 | 0 | 0 | 0 | 0 | 0 | 0 | 0 | 0 | 0 | 0 | 1 | 1 | 0 | 0 | 0 | 0 | 0 |
| *Wettinia quinaria* | 0 | 0 | 0 | 0 | 0 | 0 | 0 | 0 | 0 | 1 | 0 | 0 | 0 | 0 | 1 | 0 | 0 | 0 | 0 | 0 |
| *Wettinia radiata* | 0 | 0 | 0 | 0 | 0 | 0 | 0 | 0 | 0 | 1 | 0 | 0 | 0 | 0 | 1 | 0 | 0 | 0 | 0 | 0 |
| *Wettinia verruculosa* | 0 | 0 | 0 | 0 | 0 | 0 | 0 | 0 | 0 | 0 | 0 | 0 | 0 | 0 | 1 | 0 | 0 | 0 | 0 | 0 |
| *Zombia antillarum* | 0 | 0 | 0 | 0 | 1 | 0 | 0 | 0 | 0 | 0 | 0 | 0 | 0 | 0 | 0 | 0 | 1 | 0 | 0 | 0 |
| **Total** | **82** | **115** | **172** | **44** | **59** | **22** | **86** | **111** | **22** | **126** | **12** | **79** | **22** | **56** | **169** | **12** | **25** | **86** | **143** | **19** |

**Table S4**. Geodesic distance between bioregions (kilometres).

| **Bioregion** | **ACF** | **AMA** | **CMF** | **CAA** | **CAR** | **CDF** | **CAN** | **CER** | **CHE** | **CHO** | **GRP** | **GSH** | **IAF** | **LLA** | **NAN** | **PAN** | **SEU** | **TAM** | **WAM** | **XMA** |
| --- | --- | --- | --- | --- | --- | --- | --- | --- | --- | --- | --- | --- | --- | --- | --- | --- | --- | --- | --- | --- |
| Atlantic Coastal forest (ACF) | 0 |  |  |  |  |  |  |  |  |  |  |  |  |  |  |  |  |  |  |  |
| Amazon (AMA) | 550 | 0 |  |  |  |  |  |  |  |  |  |  |  |  |  |  |  |  |  |  |
| Central American moist forest (CMF) | 4,049 | 883 | 0 |  |  |  |  |  |  |  |  |  |  |  |  |  |  |  |  |  |
| Caatinga (CAA) | 0 | 0 | 4,230 | 0 |  |  |  |  |  |  |  |  |  |  |  |  |  |  |  |  |
| Caribbean (CAR) | 3,296 | 1,058 | 292 | 2,873 | 0 |  |  |  |  |  |  |  |  |  |  |  |  |  |  |  |
| Caribbean dry frorest (CDF) | 3,579 | 489 | 317 | 3,401 | 5,777 | 0 |  |  |  |  |  |  |  |  |  |  |  |  |  |  |
| Central Andes (CAN) | 733 | 614 | 1,186 | 2,280 | 2,398 | 1,480 | 0 |  |  |  |  |  |  |  |  |  |  |  |  |  |
| Cerrado (CER) | 0 | 0 | 2,743 | 0 | 2,135 | 2,283 | 0 | 0 |  |  |  |  |  |  |  |  |  |  |  |  |
| Chaco and Espinal (CHE) | 0 | 273 | 3,353 | 1,726 | 3,366 | 3,092 | 0 | 0 | 0 |  |  |  |  |  |  |  |  |  |  |  |
| Choco (CHO) | 3,401 | 396 | 0 | 3,678 | 1,574 | 0 | 0 | 1,872 | 2,542 | 0 |  |  |  |  |  |  |  |  |  |  |
| Grassland and Pampa (GRP) | 0 | 1,645 | 4,768 | 1,774 | 4,559 | 4,498 | 1,059 | 534 | 0 | 3,986 | 0 |  |  |  |  |  |  |  |  |  |
| Guiana Shield (GSH) | 1,896 | 0 | 1,201 | 1,210 | 334 | 663 | 1,487 | 745 | 2,048 | 698 | 3,193 | 0 |  |  |  |  |  |  |  |  |
| Inter-Andean forest (IAF) | 3,494 | 322 | 476 | 3,688 | 1,661 | 398 | 764 | 1,940 | 2,661 | 0 | 4,070 | 778 | 0 |  |  |  |  |  |  |  |
| Llanos (LLA) | 3,009 | 0 | 678 | 2,587 | 277 | 0 | 990 | 1,826 | 2,524 | 164 | 3,940 | 0 | 115 | 0 |  |  |  |  |  |  |
| Northern Andes (NAN) | 3,300 | 141 | 206 | 3,511 | 286 | 0 | 0 | 1,581 | 2,219 | 0 | 3,641 | 505 | 0 | 0 | 0 |  |  |  |  |  |
| Pantanal (PAN) | 209 | 187 | 3,258 | 1,321 | 3,059 | 2,928 | 397 | 0 | 0 | 2,560 | 796 | 1,781 | 2,602 | 2,390 | 2,319 | 0 |  |  |  |  |
| Southeastern United States (SEU) | 5,915 | 2,612 | 826 | 5,484 | 302 | 1,777 | 3,222 | 4,355 | 5,260 | 1,798 | 6,581 | 2,683 | 2,317 | 2,199 | 1,751 | 5,213 | 0 |  |  |  |
| Tropical Central American dry forests (TAM) | 5,042 | 1,758 | 0 | 5,045 | 367 | 1,098 | 1,662 | 3,545 | 4,040 | 746 | 5,434 | 2,076 | 1,213 | 1,539 | 1,013 | 4,031 | 894 | 0 |  |  |
| Western Amazon (WAM) | 994 | 0 | 871 | 0 | 1,424 | 605 | 0 | 0 | 218 | 199 | 1,561 | 250 | 77 | 0 | 0 | 290 | 2,607 | 1,441 | 0 |  |
| Xeric Mesoamerica (XMA) | 4,821 | 1,648 | 0 | 5,028 | 955 | 1,050 | 1,566 | 3,244 | 3,960 | 701 | 5,387 | 2,026 | 1,256 | 1,443 | 984 | 3,921 | 0 | 0 | 1,435 | 0 |

**Table S5**. Bioregion area in kilometres squared.

| **Bioregion** | **Area** |
| --- | --- |
| Atlantic Coastal forest (ACF) | 1,398,362.56 |
| Amazon (AMA) | 3,466,241.53 |
| Central American moist forest (CMF) | 643,306.84 |
| Caatinga (CAA) | 775,976.65 |
| Caribbean (CAR) | 224,580.54 |
| Caribean dry frorest (CDF) | 155,131.21 |
| Central Andes (CAN) | 1,041,826.12 |
| Cerrado (CER) | 3,104,631.45 |
| Chaco and Espinal (CHE) | 1,418,150.50 |
| Choco (CHO) | 220,276.49 |
| Grassland and Pampa (GRP) | 428,148.09 |
| Guiana Shield (GSH) | 1,105,856.43 |
| Inter-Andean forest (IAF) | 294,923.38 |
| Llanos (LLA) | 539,964.57 |
| Northern Andes (NAN) | 499,157.84 |
| Pantanal (PAN) | 188,724.18 |
| Southeastern America (SEU) | 463,364.57 |
| Tropical Central American dry forests (TAM) | 982,264.65 |
| Western Amazon (WAM) | 1,619,802.28 |
| Xeric Mesoamerica (XMA) | 1,627,523.31 |

**Table S6**. Phylogenetic beta diversity showing the dissimilarity in lineage composition between bioregions. P values were calculated after randomizations and significant values shown in bold font. Below diagonal - Phylosor index of phylogenetic beta diversity. Upper diagonal - proportion of phylogenetic beta diversity explained due phylogenetic turnover. Values higher than 60% are shown in bold.

| **Bioregion** | **ACF** | **AMA** | **CMF** | **CAA** | **CAR** | **CDF** | **CAN** | **CER** | **CHE** | **CHO** | **GRP** | **GSH** | **IAF** | **LLA** | **NAN** | **PAN** | **SEU** | **TAM** | **WAM** | **XMA** |
| --- | --- | --- | --- | --- | --- | --- | --- | --- | --- | --- | --- | --- | --- | --- | --- | --- | --- | --- | --- | --- |
| Atlantic Coastal forest (ACF) | 0.000 | **89.43** | **78.87** | 29.04 | **94.65** | **61.58** | **96.83** | **67.71** | 33.07 | **86.87** | 0.97 | **99.70** | 54.40 | **92.96** | **80.04** | 6.32 | **65.60** | **96.52** | **85.38** | **72.03** |
| Amazon (AMA) | **0.564** | 0.000 | **84.38** | 50.19 | **91.14** | 29.25 | **86.34** | **96.80** | 51.13 | **90.80** | 54.70 | 50.89 | 40.47 | 55.83 | **83.91** | 10.74 | **76.97** | **97.36** | **66.21** | **76.20** |
| Central American moist forest (CMF) | **0.695** | **0.635** | 0.000 | 54.43 | **63.27** | 10.91 | **68.66** | **83.09** | 51.01 | **70.93** | 56.17 | **72.89** | 23.18 | 56.20 | **90.22** | 36.50 | 42.78 | 30.78 | **89.19** | 23.57 |
| Caatinga (CAA) | **0.371** | **0.573** | **0.741** | 0.000 | **83.11** | **94.38** | **61.46** | 25.48 | **90.06** | 54.44 | **65.40** | **75.32** | **95.06** | **71.42** | **48.22** | 16.08 | **97.68** | **73.84** | 59.12 | **90.46** |
| Caribbean (CAR) | **0.616** | **0.735** | **0.639** | **0.627** | 0.000 | **74.55** | **93.57** | **90.41** | **73.53** | **78.75** | **63.51** | **97.22** | **68.79** | **99.16** | **74.53** | **48.62** | **42.12** | **84.37** | **86.48** | **70.66** |
| Caribean dry frorest (CDF) | **0.558** | **0.516** | **0.617** | **0.395** | **0.573** | 0.000 | 36.16 | 29.83 | **94.19** | 0.00 | **78.78** | **65.05** | **98.65** | 33.10 | 0.00 | **51.71** | **99.79** | **51.57** | 33.43 | **82.45** |
| Central Andes (CAN) | **0.559** | **0.395** | **0.566** | **0.561** | **0.674** | **0.469** | 0.000 | **88.26** | 35.05 | **72.99** | 46.44 | **95.32** | 42.60 | **82.83** | 49.61 | 21.47 | **78.17** | **98.38** | 48.24 | **77.64** |
| Cerrado (CER) | **0.268** | **0.318** | **0.635** | **0.460** | **0.701** | **0.506** | **0.381** | 0.000 | 27.43 | **91.21** | 13.46 | **82.89** | 35.07 | **71.80** | **84.77** | 0.00 | **69.76** | **97.76** | **84.22** | **76.32** |
| Chaco and Espinal (CHE) | **0.466** | **0.643** | **0.769** | **0.476** | **0.616** | **0.463** | **0.508** | **0.537** | 0.000 | 48.45 | **74.38** | **83.22** | **93.45** | **68.96** | 40.40 | 36.02 | **96.08** | **60.06** | 42.79 | **90.22** |
| Choco (CHO) | **0.669** | **0.524** | **0.328** | **0.665** | **0.651** | **0.502** | **0.436** | **0.576** | **0.692** | 0.000 | 53.31 | **81.02** | 15.25 | 58.96 | **84.94** | 24.19 | **66.85** | **82.89** | **99.56** | 51.89 |
| Grassland and Pampa (GRP) | **0.549** | **0.787** | **0.867** | **0.553** | **0.724** | **0.633** | **0.715** | **0.650** | **0.528** | **0.819** | 0.000 | **68.85** | **77.80** | **62.43** | 45.71 | **93.25** | **83.73** | 57.49 | 52.91 | **99.89** |
| Guiana Shield (GSH) | **0.630** | **0.226** | **0.645** | **0.624** | **0.742** | **0.576** | **0.494** | **0.421** | **0.774** | **0.590** | **0.792** | 0.000 | **67.29** | **89.41** | **70.86** | 49.06 | **89.02** | **96.95** | 47.04 | **97.88** |
| Inter Andean forest (IAF) | **0.512** | **0.556** | **0.650** | **0.403** | **0.518** | **0.187** | **0.493** | **0.523** | **0.450** | **0.541** | **0.625** | **0.589** | 0.000 | 18.20 | 0.00 | 44.51 | **99.41** | 49.13 | 41.21 | **85.73** |
| Llhanos (LLA) | **0.506** | **0.341** | **0.588** | **0.514** | **0.587** | **0.351** | **0.408** | **0.424** | **0.590** | **0.478** | **0.725** | **0.386** | **0.303** | 0.000 | 36.41 | 31.56 | **78.77** | **88.47** | 51.11 | **74.31** |
| Northern Andes (NAN) | **0.635** | **0.508** | **0.449** | **0.671** | **0.660** | **0.539** | **0.361** | **0.549** | **0.692** | **0.262** | **0.815** | **0.550** | **0.537** | **0.425** | 0.000 | 23.12 | 57.52 | **78.72** | **91.29** | 56.00 |
| Pantanal (PAN) | **0.632** | **0.710** | **0.852** | **0.436** | **0.717** | **0.541** | **0.694** | **0.678** | **0.425** | **0.783** | 0.636 | **0.757** | **0.510** | **0.662** | **0.800** | 0.000 | **64.41** | 36.13 | 28.16 | **97.68** |
| Southeastern United States (SEU) | **0.588** | **0.768** | **0.717** | **0.634** | **0.374** | **0.606** | **0.724** | **0.706** | **0.543** | **0.755** | **0.688** | **0.814** | **0.521** | **0.633** | **0.736** | **0.613** | 0.000 | 53.60 | **72.97** | **81.65** |
| Tropical Central American dry forests (TAM) | **0.669** | **0.687** | **0.343** | **0.677** | **0.519** | **0.563** | **0.633** | **0.652** | **0.648** | **0.500** | **0.773** | **0.714** | **0.548** | **0.570** | **0.533** | **0.749** | **0.576** | 0.000 | **92.43** | 7.79 |
| Western Amazon (WAM) | **0.640** | **0.223** | **0.575** | **0.687** | **0.743** | **0.600** | **0.282** | **0.421** | **0.667** | **0.462** | **0.816** | **0.336** | **0.627** | **0.431** | **0.399** | **0.790** | **0.788** | **0.687** | 0.000 | **80.01** |
| Xeric Mesoamerica (XMA) | **0.808** | **0.873** | **0.786** | **0.813** | **0.761** | **0.668** | **0.855** | **0.869** | **0.737** | **0.812** | **0.871** | **0.982** | **0.715** | **0.790** | **0.842** | **0.846** | **0.654** | **0.606** | **0.911** | 0.000 |

**Table S7**. Taxonomic beta diversity showing the dissimilarity in species composition between bioregions. Below diagonal - Sorensen index of taxonomic beta diversity. Upper diagonal – proportion of beta diversity explained due to taxonomic turnover. Values higher than 60% are shown in bold.

| **Bioregion** | **ACF** | **AMA** | **CMF** | **CAA** | **CAR** | **CDF** | **CAN** | **CER** | **CHE** | **CHO** | **GRP** | **GSH** | **IAF** | **LLA** | **NAN** | **PAN** | **SEU** | **TAM** | **WAM** | **XMA** |
| --- | --- | --- | --- | --- | --- | --- | --- | --- | --- | --- | --- | --- | --- | --- | --- | --- | --- | --- | --- | --- |
| **Atlantic Coastal forest (ACF)** | 0.000 | **94.55** | **95.31** | 38.55 | **98.51** | **88.64** | **99.66** | **73.36** | 44.72 | **98.64** | 10.88 | **99.70** | **91.65** | **97.38** | **94.90** | 30.92 | **79.96** | **99.88** | **93.83** | **96.65** |
| **Amazon (AMA)** | 0.787 | 0.000 | **95.99** | **76.39** | **98.88** | 40.74 | **88.24** | **97.56** | **68.03** | **98.62** | **100.00** | 43.85 | **63.87** | 55.82 | **90.14** | 29.13 | **94.71** | **98.34** | **72.91** | **100.00** |
| **Central American moist forest (CMF)** | 0.921 | 0.861 | 0.000 | **91.44** | **87.85** | 22.32 | **86.39** | **96.00** | **85.34** | **79.95** | **100.00** | **90.78** | 47.24 | **79.29** | **99.53** | **77.53** | **70.44** | 40.68 | **97.10** | 52.91 |
| **Caatinga (CAA)** | 0.413 | 0.774 | 0.944 | 0.000 | **99.31** | **93.10** | **92.44** | 37.30 | **84.00** | **95.40** | **77.78** | **93.18** | **96.77** | **98.48** | **90.01** | 46.67 | **96.38** | **97.69** | **88.02** | **100.00** |
| **Caribbean (CAR)** | 0.929 | 0.977 | 0.887 | 0.961 | 0.000 | **93.27** | **98.70** | **98.39** | **97.87** | **96.76** | **100.00** | **98.96** | **88.16** | **99.74** | **94.82** | **94.32** | 58.15 | **96.76** | **96.29** | **97.23** |
| **Caribean dry frorest (CDF)** | 0.923 | 0.781 | 0.814 | 0.879 | 0.926 | 0.000 | 49.25 | 46.06 | **100.00** | 0.00 | **100.00** | 59.62 | **100.00** | 46.25 | 0.00 | **91.07** | **99.70** | **70.91** | 33.83 | **100.00** |
| **Central Andes (CAN)** | 0.844 | 0.600 | 0.790 | 0.860 | 0.944 | 0.738 | 0.000 | 89.45 | 54.04 | **88.03** | **79.95** | **98.24** | 58.60 | **86.64** | 55.75 | 57.06 | **95.47** | **99.90** | 47.69 | **100.00** |
| **Cerrado (CER)** | 0.399 | 0.425 | 0.873 | 0.548 | 0.965 | 0.789 | 0.592 | 0.000 | 24.93 | **98.37** | 29.29 | **85.90** | **68.34** | **77.80** | **92.87** | 0.00 | **89.25** | **98.17** | **88.99** | **100.00** |
| **Chaco and Espinal (CHE)** | 0.712 | 0.869 | 0.959 | 0.758 | 0.975 | 0.818 | 0.757 | 0.729 | 0.000 | **86.49** | **91.07** | **94.66** | **100.00** | **93.56** | **77.60** | **70.83** | **99.37** | **88.36** | **66.33** | **100.00** |
| **Choco (CHO)** | 0.952 | 0.776 | 0.477 | 0.953 | 0.946 | 0.703 | 0.668 | 0.806 | 0.946 | 0.000 | **100.00** | **93.23** | 12.46 | **76.33** | **75.46** | **78.41** | **97.29** | **91.65** | **96.49** | **96.06** |
| **Grassland and Pampa (GRP)** | 0.766 | 1.000 | 1.000 | 0.857 | 1.000 | 1.000 | 0.938 | 0.854 | 0.824 | 1.000 | 0.000 | **100.00** | **100.00** | **100.00** | **77.57** | **100.00** | **93.43** | **93.58** | **92.86** | **100.00** |
| **Guiana Shield (GSH)** | 0.863 | 0.289 | 0.865 | 0.854 | 0.942 | 0.762 | 0.683 | 0.589 | 0.960 | 0.815 | 1.000 | 0.000 | **82.53** | **83.57** | **81.01** | **73.09** | **95.68** | **99.65** | 54.04 | **100.00** |
| **Inter Andean forest (IAF)** | 0.942 | 0.854 | 0.866 | 0.939 | 0.877 | 0.364 | 0.776 | 0.865 | 0.818 | 0.730 | 1.000 | 0.881 | 0.000 | 46.25 | 0.00 | **94.44** | **99.37** | **74.70** | 53.19 | **100.00** |
| **Llanos (LLA)** | 0.899 | 0.544 | 0.833 | 0.900 | 0.913 | 0.590 | 0.660 | 0.689 | 0.923 | 0.725 | 1.000 | 0.556 | 0.590 | 0.000 | 46.47 | **82.26** | **96.78** | **95.61** | 47.78 | **100.00** |
| **Northern Andes (NAN)** | 0.912 | 0.704 | 0.654 | 0.934 | 0.947 | 0.770 | 0.528 | 0.786 | 0.937 | 0.410 | 0.967 | 0.750 | 0.770 | 0.653 | 0.000 | **77.57** | **87.61** | **87.04** | **90.67** | **95.76** |
| **Pantanal (PAN)** | 0.809 | 0.858 | 0.967 | 0.714 | 0.972 | 0.824 | 0.876 | 0.805 | 0.588 | 0.957 | 0.917 | 0.912 | 0.882 | 0.912 | 0.967 | 0.000 | **96.90** | **79.89** | 45.80 | **100.00** |
| **Southeastern United States (SEU)** | 0.850 | 0.971 | 0.909 | 0.913 | 0.619 | 0.957 | 0.964 | 0.941 | 0.915 | 0.987 | 0.892 | 0.962 | 0.915 | 0.951 | 0.959 | 0.946 | 0.000 | **82.39** | **88.20** | **98.42** |
| **Tropical Central American dry forests (TAM)** | 0.952 | 0.910 | 0.457 | 0.954 | 0.876 | 0.833 | 0.860 | 0.888 | 0.926 | 0.736 | 0.980 | 0.927 | 0.852 | 0.859 | 0.788 | 0.939 | 0.874 | 0.000 | **95.76** | 22.71 |
| **Western Amazon (WAM)** | 0.858 | 0.310 | 0.778 | 0.904 | 0.950 | 0.806 | 0.395 | 0.567 | 0.891 | 0.658 | 0.987 | 0.468 | 0.855 | 0.598 | 0.494 | 0.910 | 0.952 | 0.886 | 0.000 | 100.00 |
| **Xeric Mesoamerica (XMA)** | 0.980 | 1.000 | 0.895 | 1.000 | 0.974 | 1.000 | 1.000 | 1.000 | 1.000 | 0.986 | 1.000 | 1.000 | 1.000 | 1.000 | 0.989 | 1.000 | 0.909 | 0.695 | 1.000 | 0.000 |
